# Synergistic Effect of Erastin Combined with Nutlin-3 on Vestibular Schwannoma Cells as p53 Modulates Erastin-Induced Ferroptosis Response

**DOI:** 10.1155/2022/7507857

**Published:** 2022-03-21

**Authors:** Weiwei He, Wenying Shu, Lu Xue, Yaoxuan Wang, Yongchuan Chai, Hao Wu, Zhaoyan Wang

**Affiliations:** ^1^Department of Otolaryngology Head and Neck Surgery, Shanghai Ninth People's Hospital, Shanghai JiaoTong University School of Medicine, Shanghai 200011, China; ^2^Ear Institute, Shanghai JiaoTong University School of Medicine, Shanghai 200125, China; ^3^Shanghai Key Laboratory of Translational Medicine on Ear and Nose Diseases, Shanghai 200125, China

## Abstract

Vestibular schwannoma (VS) is a rare neurotology neoplasm that results in partial neurological defects. As we know, a comprehensive understanding of basic mechanisms and targeted therapy is vital for disease management. In VS, p53 has been proved to suppress tumor progression via a cooperative with the key protein, merlin, as well as regulation of the cell cycle. However, there are more potential mechanisms of p53 in VS needed to exploit. First, via genome-wide RNA expression analysis, we identified differentially expressed genes in VS compared with normal nerves, and then, bioinformatics analyses were used to analyze these differential expression data and suggested a high level of enrichment of cysteine and glutathione metabolism pathways in VS. Meanwhile, we observed a downregulation of SLC7A11/xCT, a component of the cystine/glutamate antiporter (also known as system x_c_^−^) involved in cystine uptake. Next, for a deeper study, our group extracted tumor cells from vestibular schwannoma tissues and established two immortalized cell lines named JEI-001 and JEI-002. Secondly, in our established cells, we demonstrated that ferroptosis participated in erastin-induced growth inhibition. As a novel cell death process, ferroptosis driven by iron-mediated lipid reactive oxygen species (lipid ROS), as well as cysteine and glutathione metabolism. Furthermore, ferroptosis contributes to the inhibitory effects of tumor suppressor p53. Here, we show that p53 sensitizes schwannoma cells to ferroptosis by repressing expression of SLC7A11/xCT. Finally, erastin combined with Nutlin-3, which s to p53 activation, triggered antitumor effects of ferroptosis on the growth of schwannoma cells in vitro. These findings present potential mechanism of p53 in schwannomas and raise the possibility of treatment strategies directed against this pathogenesis.

## 1. Introduction

Vestibular schwannomas (VSs) are often slow-growing tumors which usually invade the vestibular nerve in the cranial nerve system, resulting in hearing loss and neurological defects [1]. VSs are tumors which develop from transformed schwann cells, the cells which produce myelin in the nervous system. Although the molecular basis of vestibular schwannoma is largely unknown, our previous study showed that the classical suppressor p53 performs an essential role in VS. Downregulation of p53 is one of the mechanisms of VS tumorigenesis, acting by inhibiting the cell cycle and regulating the stability of merlin [2]. The experiment further demonstrated that treatment with Nutlin-3 could active p53 through inhibiting p53–MDM2 interaction and block the proliferation of schwannoma cells [2]. In our current study, we show that p53 regulates the sensitivity of erastin-induced ferroptosis in cancer therapy.

Ferroptosis is an iron-mediated, caspase-independent cell death process that requires the accumulation of lipid reactive oxygen species (lipid ROS). Ferroptosis was first defined in 2012 [3], and later research proved that glutathione peroxidase 4 (GPX4) and the cystine/glutamate antiporter (known as system x_c_^−^) are the primary participants in ferroptosis signalling pathways [4]. GPX4 is a glutathione-regulated lipid repair enzyme which scavenges lipid peroxides [4] while system x_c_^−^ is an amino acid transporter system which exchanges intracellular glutamate and extracellular cystine [5]. The resulting intracellular cystine acts as the limiting substrate for glutathione (GSH) synthesis and also as cofactor in the activation of GPX4. In recent years, ferroptosis has been considered to be one of critical pathways in tumor suppression by p53 [6]. p53 regulates ferroptosis partly through suppressing a component of system x_c_^−^, SLC7A11/xCT, leading to reduced cystine import and finally decreased GSH production and accumulated lipid ROS [6]. However, the role of p53 in modulating ferroptosis can vary with context, but whether and how p53 might contribute to ferroptosis in schwannoma cells is not completely clear.

In the current study, we began with gene set enrichment analysis (GSEA) of VS which showed that cysteine and GSH metabolism are downregulated, and at the same time, low expression of SLC7A11/xCT protein was shown in most VS sample. Based on the data from schwannoma tissues, we verified the occurrence of erastin-induced ferroptosis in two immortalized VS cell lines established by our group. With the aim of gaining insight into the unconventional molecular mechanisms of p53 in cancer therapy, we further investigated whether p53-mediated regulation critically contributes to ferroptosis by cystine/glutamate transporters in schwannoma cells. Meanwhile, we showed that, *in vitro*, combination therapy targeted at p53 and ferroptosis works in a coordinated manner to reduce the growth of schwannomas.

## 2. Materials and Methods

### 2.1. Ethics Statement

The studies involving human participants were reviewed and approved by the Research Ethics Review Committee of Shanghai JiaoTong University (document serial number: SH9H-2019-T95-2,version date: 2019-07-31 (v2.1)). The patients/participants provided their written informed consent to participate in this study. All procedures performed in studies involving human participants were in accordance with the 1964 Helsinki Declaration and its later amendments or comparable ethical standards.

### 2.2. Clinical Sample Collection

The VS tissues were collected immediately for primary culture or maintained at -80°C until needed. The nontumoral great auricular nerves (GANs) were regarded as normal nerve controls. All patients who donated tissue obtained informed written consent. This research was approved by the Research Ethics Review Committee of Shanghai JiaoTong University.

### 2.3. Cell Line and Cell Culture

#### 2.3.1. Cell Culture

All cells were grown in 90% DMEM with 10% fetal bovine serum (FBS) and 100 U/ml of penicillin and streptomycin and cultured in 37°C incubator with humidified atmosphere of 5% CO_2_.

#### 2.3.2. Cell Line

**Figure 1:**
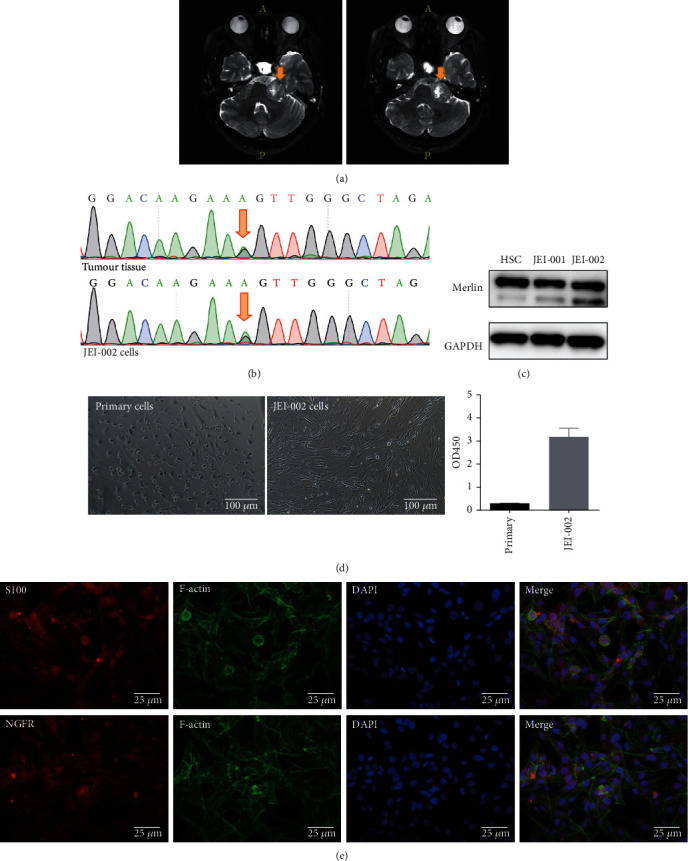
Characteristics of JEI-002 cells. (a) MRI of a 44-year-old vestibular schwannoma patient. (b) Cells were confirmed to contain a specific NF2 heterozygous mutation (c.240G > A) at Exon 2 using Sanger sequencing. (c) Expression of merlin was noted in HSC, JEI-001, and JEI-002. (d) The cells had become immortalized: primary cells, JEI-002. The CCK-8 results showed the significantly different growth speed of primary cells and JEI-002 cells (*p* < 0.05). (e) Immunocytochemical staining of JEI-002 cells for S100 and NGFR, produced positive results.

Human schwann cells (HSCs) were purchased from ScienCell Research Laboratories. The HEI-193, an HPV E6-E7 immortalized schwannoma cell line, was a kind gift from House Ear Institute. The JEI-001, a hHERT (human telomerase reverse transcriptase) immortalized schwannoma cell line [7], as well as the JEI-002, a SV40 (Simian Virus 40) immortalized schwannoma cell line (Figure 1), were established by Ear Institute Shanghai JiaoTong University School of Medicine.

#### 2.3.3. Infection

Except for a nonsense shRNA, lentiviral shRNA vectors were constructed against p53 using target sequences (P53.NM_000546). Also, p53 was ectopically expressed in cells using the lentiviral vector.

### 2.4. Primary Vestibular Schwannoma Cell Culture

The tissue was taken to the laboratory at 4°C after being placed in DMEM. Tumor tissue was minced and digested at 37°C for 2 hours with 0.125% enzymes-EDTA+0.1% Collagenase type II. Then, the cells were passed through a 200 mesh filter screen and finally cultured in DMEM/high-glucose medium with 10% FBS.

### 2.5. Cell Viability Assay

Reagents used are as follows: erastin (#E7781, Sigma Aldrich), ferrostatin-1 (Fer-1) (#RM02804, ABclonal), liproxstatin-1 (Lip-1) (#RM02805, ABclonal), desferrioxamine mesylate (DFO) (#RM02807, ABclonal), necrostatin-1 (#RM02815, ABclonal), chloroquine (#RM02814, ABclonal), and Z-VAD-FMK (#IZ0050, solarbio).

Cell viability was evaluated using the Cell Counting Kit-8 (CCK-8) (LJ621, Dojindo, Japan). 5000 cells per well were seeded in a 96-well plate; upon a 24-hour treatment, each well was replaced with fresh medium containing CCK-8 reagent. After incubation for 2 h at 37°C, then, cell viability was measured on a microplate reader (Synergy HT, Bio-Tek, USA) at 450 nm.

### 2.6. Western Blotting

Proteins were extracted and analyzed by western blotting according to standard protocols using primary antibodies specific for p53 (#A18803, ABclonal), SLC7A11/xCT (#A2413, ABclonal), and GAPDH (#AC033, ABclonal).

### 2.7. Flow Cytometry Analysis for Lipid Peroxidation

#### 2.7.1. Assessment of Lipid Peroxidation

To induce ferroptosis, 150,000 cells/well were seeded in 6-well dishes and then treated with erastin (50 *μ*M) for 24 h. Cells were incubated with C11-BODIPY (581/591) (#A2413, ABclonal) for 30 min according to standard protocols. Subsequently, cells were resuspended and analyzed by Flow Cytometer (Beckman Coulter, USA) using the 488 nm laser.

### 2.8. Assessment of Cell Death

#### 2.8.1. Flow Cytometry Analysis

Pharmingen Annexin V-FITC/PI staining kit (Becton Dickinson, USA) was used following description in the manufacturer's instructions.150,000 cells/well were seeded in 6-well dishes; then, cells were treated with erastin (50 *μ*M) for 24 h and then analyzed by Cytometer (Beckman Coulter, USA).

#### 2.8.2. Immunofluorescence Analysis

Cell death was also analyzed by staining for SYTOX Green (#C1070S, Beyotime), at the same time, living cell staining by CM-Dil (#C7000, Invitrogen). Next, images were acquired to use confocal microscope (Leica, Germany).

### 2.9. Immunofluorescence Analysis

Treated cells were fixed with 4% paraformaldehydees, permeabilized in Triton X-100, and then blocked with bovine serum albumin. Immunocytochemical analysis was performed using antibodies specific for xCT (#ab37185, Abcam) and S100 (#ab868, Abcam). Nuclear counterstaining was conducted using DAPI (#C1002, Beyotime). Images were acquired using a confocal microscope (Leica, Germany).

### 2.10. Transmission Electron Microscopy

Cell (with or without treatment) pellets were fixed in 2.5% glutaraldehyde in 0.1 M sodium cacodylate buffer pH 7.4, postfixed in 2% aqueous osmium tetraoxide, dehydrated in gradual ethanol (50–100%) and propylene oxide, and embedded and cured for 48 h at 60°C. Ultrathin sections were cut, stained with uranyl acetate and lead citrate, and then examined with a transmission electron microscope.

### 2.11. Tumor Xenograft Model and Treatments

To establish xenograft models, HEI-193 (1 × 10^6^ in 0.1 ml PBS) were injected into 4-week-old BALB/c nude mice subcutaneously. Tumors were allowed to grow for one week; then, mice were randomly divided into four groups (*n* = 4) and subsequently treated with different methods once a day as follows: control (i.p., solvent), erastin (i.p. 40 mg/kg), Nutlin-3 (i.p. 40 mg/kg), and Nutlin-3 (i.p. 40 mg/kg) and erastin (i.p. 40 mg/kg). Nutlin-3 and erastin were dissolved in the solvent which is 1: 1: 1: 7 solution of DMSO: tw80: propanediol: phosphate-buffered saline. Tumors were measured every two days until the endpoint, and the volume was calculated according to the equation *v* = length × width^2^ × 1/2. After continuous administration for two weeks, the mice were euthanized; then, the tumors were removed and weighed. All the experimental procedures were conducted in accordance with the guidelines of the Committee of Laboratory Animals of the Ninth People's Hospital, Shanghai Jiao Tong University School of Medicine.

### 2.12. Bioinformatics Methods

Differential levels of gene expression between the normal nerve controls and the VSs were measured by a moderated *t*-test; then, *p* value was corrected using the Benjamini-Hochberg algorithm (false discovery rate (FDR)). Difference was confirmed when the absolute fold-change was ≥2 and adjusted *p* value was <0.05. The obtained integrated differentially expressed genes (DEGs) were analyzed using the DAVID database for the Gene Ontology (GO) functional annotation and Kyoto Encyclopedia of Genes and Genomes (KEGG) pathway analyses. Statistically significant *p* values were set at 0.05 or less. The differentially expressed gene set was analyzed by Gene Set Enrichment Analysis (GSEA) software using GSEA software version 2.2.2.0 [8, 9]. A pathway was considered a significant enrichment if normalized enrichment scores (NES) > 1 or<1, normal *p*‐val < 0.05, and FDR *q*‐val < 0.25.

### 2.13. Statistics

All values in the present study were expressed as mean ± standard deviation (SD) from at least three independent experiments. Statistical analysis was performed using one-way analysis of variance (ANOVA) or Student's *t*-test to investigate if the differences were significant among the mean values of different groups.

## 3. Results

### 3.1. Bioinformatics Analysis of VSs Compared with Normal Nerves

**Figure 2:**
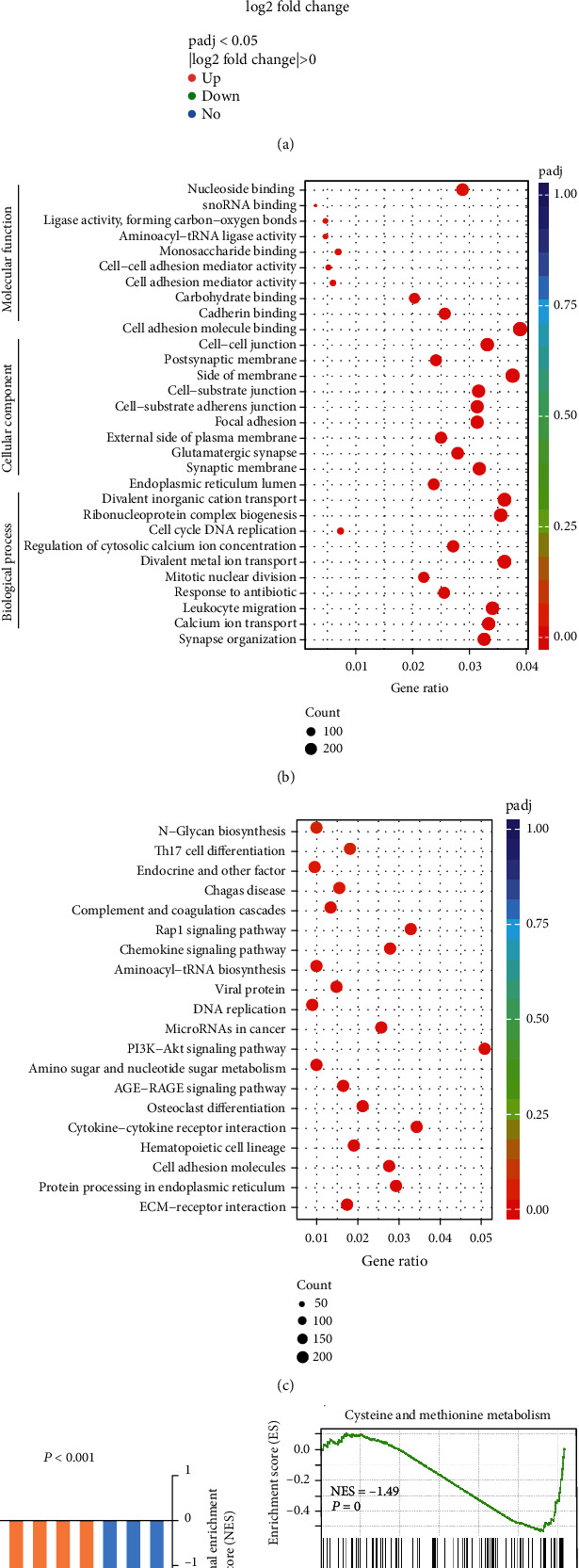
Proteomic analysis between vestibular schwannomas (VSs) and nerve controls (NCs). (a) Volcano plot exhibiting relationship between magnitude (log2 of fold-change) and statistical significance (-log10 of *p*adj) of DEGs in a comparison of VSs and NCs. (b) The bubble chart revealed the relative frequent enrichment pathways in VSs by KEGG. For each dot, the color denoted the *p* value and the size denoted the proteins number. (c) The bubble chart revealed the relative frequent enrichment pathways in VSs by GO. For each dot, the color denoted the *p* value and the size denoted the proteins number.(d) NES indicated the distribution in VSs as compared to NCs. GSEA analysis of the proteomic database in VSs, cysteine, and methionine metabolism (right panel) and glutathione metabolism (left panel). VS, *n* = 3 tumors; NC, *n* = 3.

We compared the expression profile of VS with that of normal nervous tissue. As shown in Figure 2(a), we found that 10,982 genes were differentially expressed, of which 6,491 genes were significantly upregulated and 4,491 were downregulated. The complete list of differentially expressed genes (DEGs) (*P* value <0.05) is shown as Supplemental Table 1. Furthermore, we then conducted GO functional annotation and KEGG pathway analyses of DEGs in VS. The main enriched GO terms were categorized on the basis of biological process (BP), cellular component (CC), and molecular function (MF) to determine which were enriched in the DEGs found in VS. Top 10 generally changed GO terms in the VS group classified by BP, CC, and MF and ranked by enrichment score were listed in Figure 2(b). KEGG pathway analysis was performed, and the most obvious pathways were selected and ranked by enrichment score (Figure 2(c)).

Moreover, we used GSEA, a pathway enrichment method that evaluates microarray data at the level of gene sets, to analyze DEGs in VS compared with normal nerves. The GSEA approach requires large-scale knowledge-based databases, and gene expression data are then compared with predefined gene sets from the Molecular Signatures Database (MSigDB v5.0) [9]. An overview of the involved downregulated gene sets is presented in Figure 2(d) and Supplemental Table 2. GSEA confirmed that downregulated genes were enriched in cysteine and methionine metabolism and glutathione metabolism (Figure 2(d)). Next, we analyzed and explored the possible mechanisms and impacts involved in the attenuation of regulatory ability of cysteine and glutathione.

Cysteine can be taken up by system x_c_^−^, in the form of cystine [5], and supports the cellular antioxidant system and promotes ROS detoxification as a limiting substrate for GSH synthesis. The attenuation of the regulatory ability of cysteine metabolism, together with glutathione metabolism, may contribute to some cancer-specific characteristics, for example ferroptosis. Based on the above findings, reasonable speculation and verification were performed in subsequent experiments.

### 3.2. Introduction of Two Schwannoma Cell Lines

The ideal method of studying the molecular biology of VS *in vitro* is to use a primary cell culture that best takes on its characteristics. However, the limited incidence and lifespan of benign tumors leads to difficulty in obtaining such cells and the growth of primary cells is so slow that they cannot be used in some experiments. Different stable immortalized cell lines are necessary tools to explore the molecular basis and targeted therapy of VS [10].

In our previous studies, primary schwannoma cells (JEI-001) were obtained from a 41-year-old vestibular schwannoma patient, cultured, and immortalized using lentivirus-mediated infection, and JEI-001 contains an Exon 5 mutation of the NF2 gene (c.515delG) [7]. Another schwannoma cell line was established from cells obtained from a 44-year-old vestibular schwannoma patient using the same technique and named JEI-002 (Figure 1(a)). The JEI-002 cell line contains a specific Exon 2 mutation of the NF2 gene (c.240G > A). A common mutations in the NF2 gene (c.240G > A) was detected in the primary tumor tissue and in JEI-002, indicating that the immortalized cell line retained most of its original tumor characteristics (Figure 1(b)). At the same time, the schwannoma cell origin of JEI-002 was confirmed using STR (short tandem repeats) techniques (Table 1). In addition, we detected the expression of key protein merlin, and compared with HSC, there was no significantly change in expression of merlin was noted in JEI-001 and JEI-002 (Figure 1(c), Figure S2). As show in Figure 1(d), the JEI-002 cells appeared to be of an irregular polygonal shape and grew in a disorderly manner compared to the primary tumor cells. And CCK-8 assay results show the growth rate of the primary cells and JEI-002 cells, a large increase in the growth rate was observed by measuring the optical density value at 450 nm (OD_450_). Then, immunocytochemical staining was used to evaluate the presence of certain biomarkers of JEI-002 cells (Figure 1(e)), such as S100 and NGFR, which used to discriminate schwann cells [11, 12]. JEI-002 cells did not undergo tumorigenic transformation in animals (data not shown), which means the JEI-002 cell line in the log phase was implanted subcutaneously in nude mice but no tumor was found.

In conclusion, we successfully obtained two schwannoma cell lines using lentivirus-mediated infection.

### 3.3. Erastin Triggers Growth Inhibition in Schwannoma Cells

Based on the results of bioinformatics analysis of VS, we have focused on the possible role of glutathione and cysteine which are critical participants of ferroptosis pathway, but exactly how cysteine and glutathione metabolism influence VS cells remains unclear.

**Figure 3:**
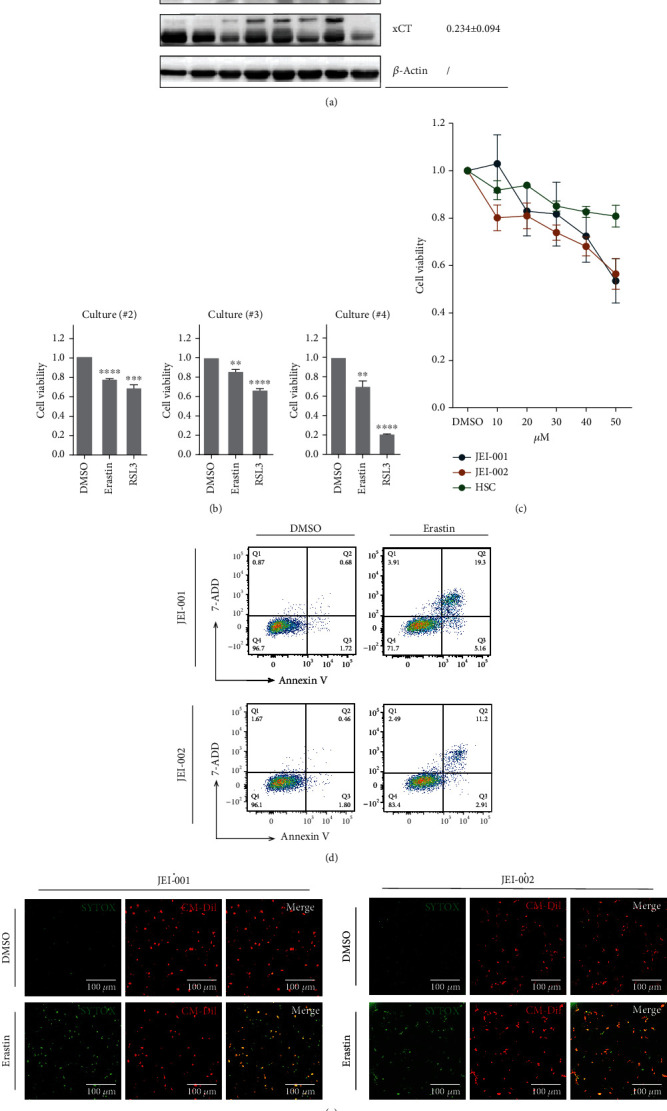
Erastin induced growth inhibition in schwannoma cells. (a) Three proteins in different individuals were evaluated by western blot (left panel). The relative average gray scale values were presented (right panel). (b) The viabilities of schwannoma primary cultures were incubated with erastin at 50 *μ*M and RSL3 at 20 *μ*m for 24 h. ^∗∗^*p* < 0.005. (c) The cell viability was measured by CCK-8. The cell viability of DMSO (solvent for erastin) was set as the reference. Triplicate in each group (*p* < 0.01). (d) After treatment with 50 *μ*M erastin for 24 h. Cells were sampled for staining with annexin-V and 7-AAD and then examined via flow cytometry. (e) Different schwannoma cell lines were cocultured with erastin (50 *μ*M) for 24 h. Dead cells were labeled by SYTOX Green and living cells were labeled by CM Dil (scale bar = 100 *μ*m).

System x_c_^−^, which plays a key role in regulating the balance of cellular cysteine and glutathione [13] and is used as an attack target of erastin [14], consists of the regulatory protein SLC3A2 coupled to the transporter protein SLC7A11/xCT [15]. In tumor tissues, as shown in Figure 3(a), the expression of SLC7A11/xCT decreased in most samples. In addition, decreased levels of merlin and p53 which were found to be a feature of VS in our previous study were further confirmed in most tumors tissues by western blot analysis (Figure 3(a), Figure S1).

Next, three samples of primary schwannoma cells were extracted from tumors and subjected to treatment with the ferroptosis inducers, erastin, and RSL3. Following 24-hour treatment, the viability of primary cultured schwannoma cells was decreased (Figure 3(b)). To further confirm the effect of erastin, two schwannoma cell lines were incubated for 24 hours with different concentrations of erastin, and then, the cell viability and cell death were assessed. Erastin leads to depletion of GSH, inactivation of GPX4, and accumulation of lipid ROS. Another possible mechanism of erastin-induced cell death is associated with the p62-Keap1-NRF2 pathway [16] which was not explored in this study. As shown in Figure 3(c), cell viability decreased in schwannoma cells but the effect of erastin was not obvious in Human schwann cells (HSCs), and the nontumor cells are tested. In JEI cells, treatment with erastin would increase the annexin V-positive cell fraction and the annexin-V/7-AAD-positive cell (Figure 3(d)). In Figure 3(e), to labeled death cell by SYTOX Green and labeled control living cell by CM-Dil, fluorescent microscopy images of cells demonstrated erastin would prominently triggered schwannoma cell death.

In summary, our results support the hypothesis that erastin is capable of selectively inducing growth inhibition of vestibular schwannoma cells.

### 3.4. Ferroptosis Contributes to Erastin-Induced Growth Inhibition in Schwannoma Cells

**Figure 4:**
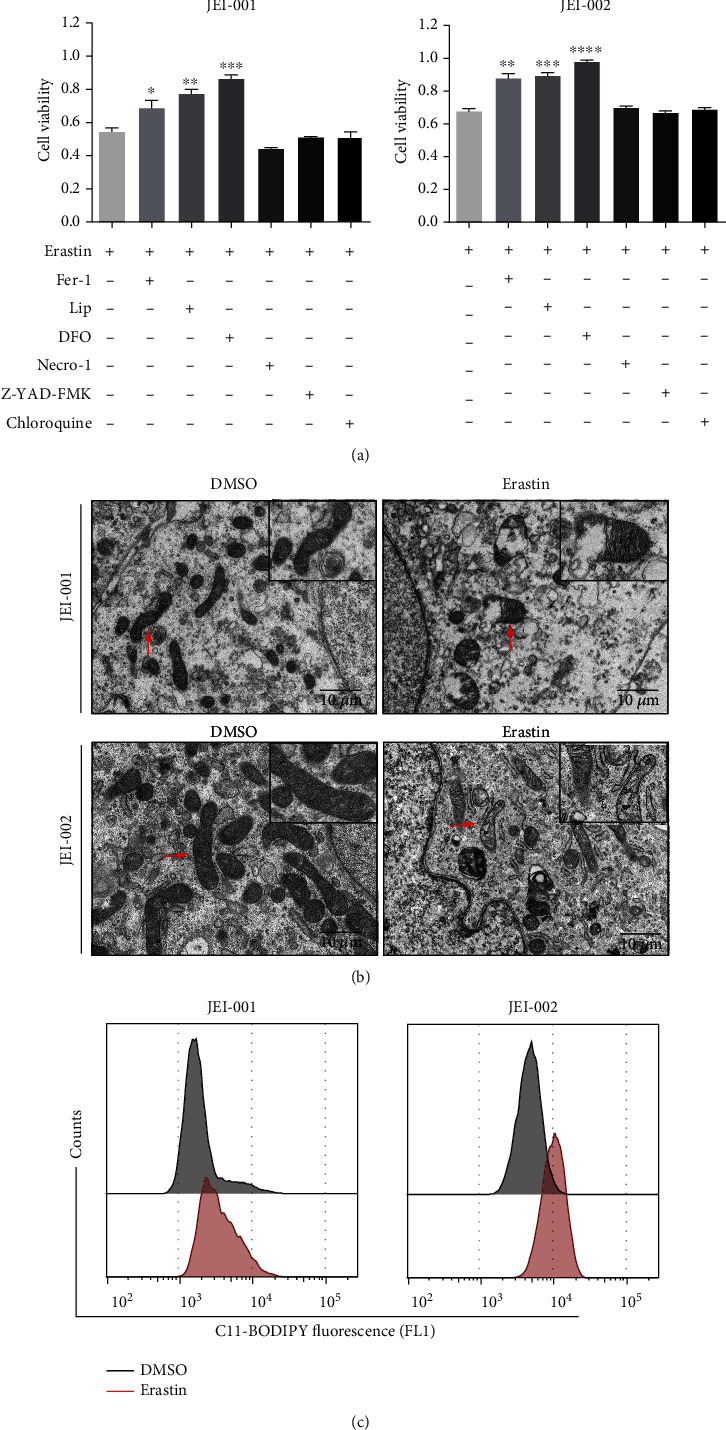
Ferroptosis participates in erastin-induced response in schwannoma cells. (a) Schwannoma cells were cultured with erastin for 24 hours with or without the specific inhibitors; then, the cell viability was measured. Triplicate in each group. ^∗^*p* < 0.05 versus the erastin treatment group. (b) Ultrastructural analysis revealed the erastin-treated (50 *μ*M for 24 h) schwannoma cells and the outer membrane rupture (red arrows) (scale bars: 10 *μ*m). (c) Erastin (50 *μ*M for 24 h)-induced lipid peroxidation in schwannoma cells as determined by BODIPY 581/591 C11. Triplicate in each group.

The results of previous studies on ferroptosis progress showed that erastin triggers morphological changes or biochemical processes difference with cell death or cytostasis induced by proapoptotic or pronecrotic agents [17–19]. To identify whether ferroptosis is involved in the erastin-induced cell death of schwannoma cells, we used the combination of erastin and ferroptosis inhibitors to confirm the specificity. In our study, liproxstatin-1 (Lip-1), ferrostatin-1 (Fer-1), and desferrioxamine mesylate (DFO) were used as ferroptosis inhibitors. The synthetic antioxidants Fer-1 and Lip-1 were demonstrated to suppress ferroptosis by scavenging lipid peroxide, while erastin-induced growth suppression could effectively be inhibited by the iron chelator DFO [3, 18, 20]. As shown in Figure 4(a), in two schwannoma cell lines, all three ferroptosis inhibitors rescued erastin-induced growth inhibition, while at the same time, a necroptosis inhibitor (necrostatin-1), an autophagy inhibitor (chloroquine), and a general caspase inhibitor (Z-VAD-FMK) could not weaken erastin toxicity [3, 4, 17, 19, 21].

Furthermore, imaging by electron microscopy showed changes in mitochondrial morphology. Morphological examination of cells following treatment with erastin for 24 hours mainly revealed that mitochondria in erastin-treated cells appeared smaller, with shrunken or obliterated cristae, and rupture of the outer mitochondrial membrane (Figure 4(b)), consistent with past research about ferroptosis [3, 18, 22]. In these ferroptotic cells, the remaining structure was integrated without chromatin margination. In terms of biochemical mechanism, since lipid peroxidation is one hallmark of ferroptosis, we estimated the levels of lipid peroxidation by BODIPY 581/591 C11 [23, 24] following 24-hour treatment with erastin. As shown in Figure 4(c), erastin resulted in a more intense fluorescence, which implied an increase in lipid ROS.

Taken together, all the above data indicate that ferroptosis might partly contribute to erastin-induced growth suppression in schwannoma cells. Furthermore, results suggest that erastin promotes characteristic morphological changes and lipid ROS accumulation associated with ferroptosis.

### 3.5. p53 Performs as a Regulator in Erastin-Induced Ferroptosis in VS Cells

As an evolutionarily conserved protein, the tumor suppressor p53 plays essential roles in tumorigenesis. Generally, p53 activity focuses on the response to cellular stress in cell cycle arrest, apoptosis, and/or senescence. Moreover, other unconventional activities of p53 are also considered to reflect its crucial role in tumor suppressor function [25–29]. Recently, some studies have suggested that p53 inhibits cystine uptake and sensitizes cells to ferroptosis by repressing expression of SLC7A11/xCT [6]. SLC7A11, a component of system x_c_^−^ [30–32], has been demonstrated to be a novel p53 target gene in some malignant cells. In our previous research, a decrease of p53 was found in most vestibular schwannomas and p53 was shown to slow tumor growth by inhibiting the cell cycle and coexpression with merlin [2]. We next aimed to find potential functions of p53 and to figure out whether p53 could affect cell sensitivity to ferroptosis by regulating the expression of SLC7A11/xCT in VS, as described below.

**Figure 5:**
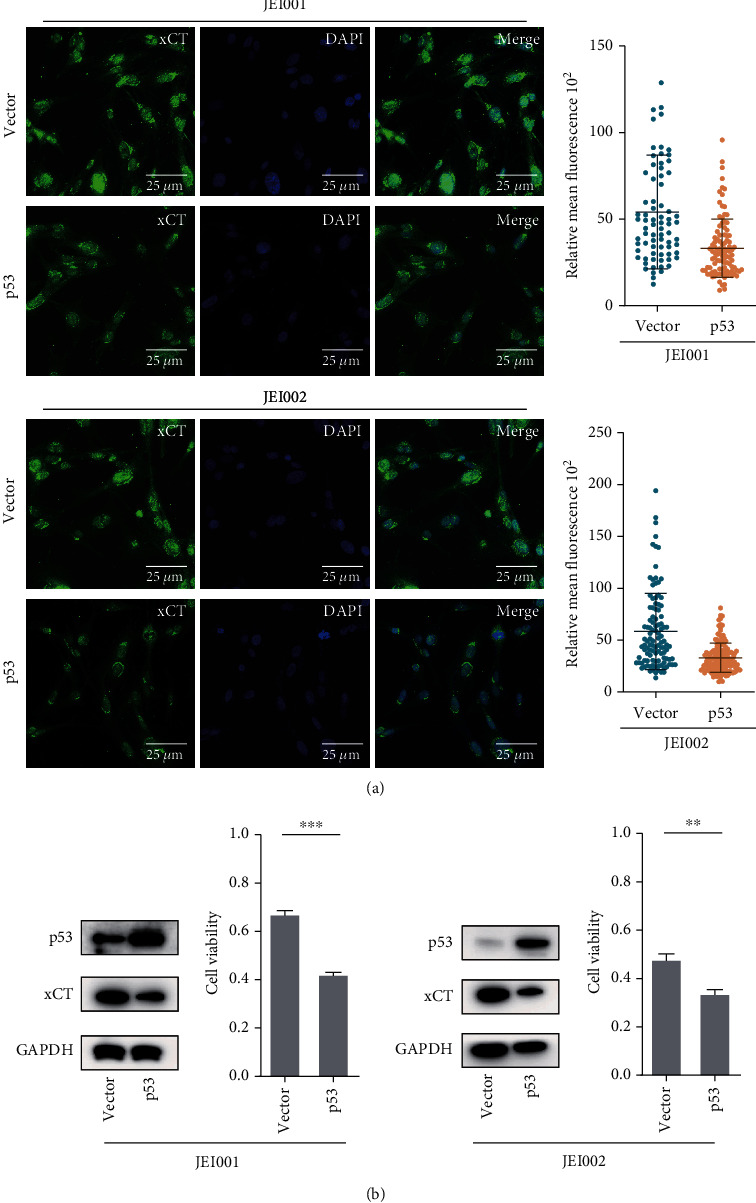
p53 regulated erastin-induced ferroptosis by SLC7A11/xCT in schwannoma cells. (a) Fluorescence analysis of SLC7A11/xCT between schwannoma cells and schwannoma cells transfected with p53. Relative expression of SLC7A11/xCT decreased significantly in the control and p53-overexpression cells (scale bar: 20 *μ*M). (b) Expression of proteins between the control and overexpression-p53 schwannoma cells. The cell viability was measured with CCK-8 following 24 h erastin treatment in different schwannoma cells.

Western blot analysis revealed that p53 activation reduced SLC7A11/xCT protein levels, and schwannoma cells infected with a vector expressing p53 exhibited decreased SLC7A11/xCT levels (Figure 5(b), Figure S3). The changes in SLC7A11/xCT in response to activated p53 were further evidenced by fluorescence analyses (Figure 5(a)). To confirm this effect of p53 in erastin-induced ferroptosis, we analyzed the cell viability of p53-overexpressing schwannoma cells after 24-hour treatment with erastin (Figure 5(b)). Compared with control cells, erastin triggered more prominent cell death in p53-overexpressing schwannoma cells. An explanation for this phenomenon is that p53 recognizes and binds to the SLC7A11/xCT gene and downregulates protein levels, and the decline of SLC7A11/xCT blocks system x_c_^−^-mediated cystine import and enhances inducer efficacy, sensitizing the iron-dependent accumulation of lipid ROS.

### 3.6. Nutlin-3 Contributes to Growth Inhibition in Ferroptosis Progress of Schwannoma

The research compound Nutlin-3 is an MDM2 antagonist [33] and is able to bind MDM2 and disrupt the p53–MDM2 interaction, leading to p53 stabilization and activation [34]. In our previous studies, Nutlin-3 restored normal levels and normal biological activity of p53 and efficiently reduced the growth of schwannoma *in vitro* [2].

**Figure 6:**
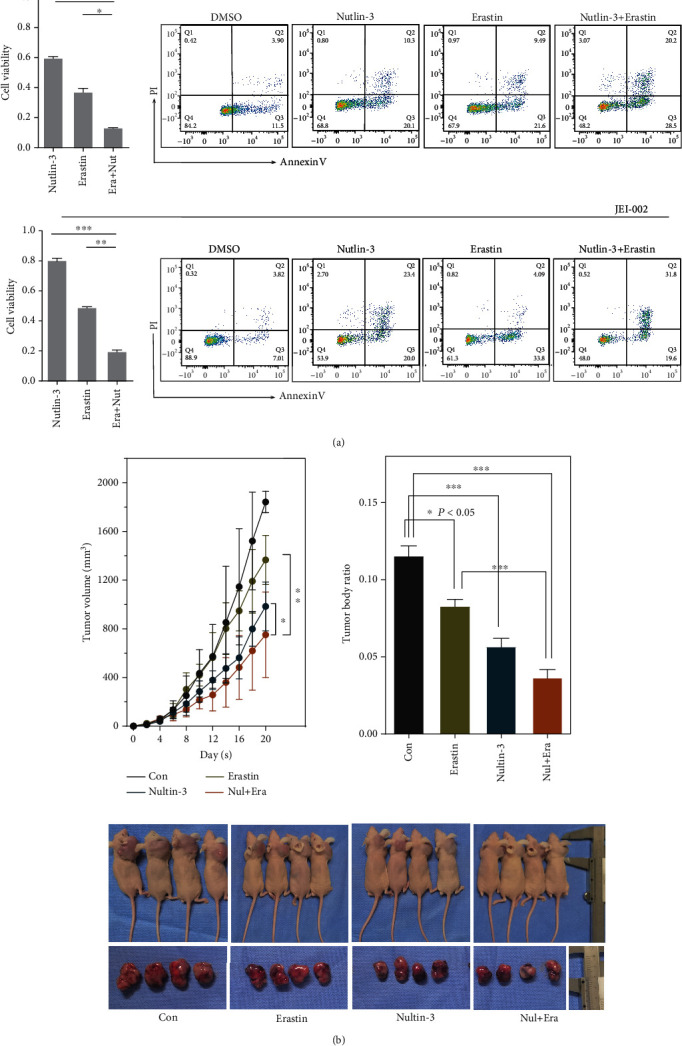
The inhibition effects of Nutlin-3 and/or erastin schwannoma in vitro. (a) The cell was treat with Nutlin-3 (20 *μ*M), erastin (50 *μ*M), and Nutlin-3 (20 *μ*M) and erastin (50 *μ*M) for 24 hrs. The cell viabilities in the Nutlin-3 group, erastin group, and Nutlin-3 and erastin group (^∗∗^*p* < 0.005). The death cells in different groups were evaluated by Annexin V-FITC and PI. (b) The dynamic changes in the HEI-193 schwannomas (left panel) and the tumor-to-body weight ratios (right panel) in different groups. The images of mice and resected tumors (bottom panel) in the endpoint. *n* = 4.^∗^*p* < 0.05.

As shown in Figure 6(a), the sensitivity of erastin markedly decreased when p53 was activated by Nutlin-3 treatment. The VS cells were subjected to different treatments: DMSO control, erastin alone, Nutlin-3 alone, and erastin combined with Nutlin-3. In all treated groups of two schwannoma cells, significant decreases in viability were observed, but by contrast, we found more remarkable decreases in the erastin+Nutlin-3 groups. Collectively, the cell death rates in the erastin+Nutlin-3 group were increased compared with other groups when analyzed by flow cytometry (Figure 6(a)). The figure shows between-group differences due to the sensitization of p53 in ferroptosis.

Because no tumorigenic transformations were detected in our established human schwannoma cells, JEI-001 and JEI-002, we used HEI-193 to establish heterologous subcutaneous cancer models in nude mice for further drug experiments. No mice died during the experiments and there were no significant differences in the weight of nontumor-bearing mice. After two weeks of treatment, we recorded and measured the tumor volumes every two days and found that the growth of xenogeneic tumors was affected by Nutlin-3 and erastin, whereas the volumes and weights of the tumors in the erastin+Nutlin-3 groups were significantly lower (Figure 6(b), Figure S4). These findings indicate that activation of p53 by Nutlin-3 alleviates tumor growth, since in the erastin+Nutlin-3 group, the tumors were markedly smaller than those in the control group or the erastin group.

Because no tumorigenic transformations were detected in our established human schwannoma cells, JEI-001 and JEI-002, we used HEI-193 to establish heterologous subcutaneous cancer models in nude mice for further drug experiments. No mice died during the experiments, and there were no significant differences in the weight of nontumor-bearing mice. After two weeks of treatment, we recorded and measured the tumor volumes every two days and found that the growth of xenogeneic tumors was affected by Nutlin-3 and erastin, whereas the volumes and weights of the tumors in the erastin+Nutlin-3 groups were significantly lower (Figure 6(b), Figure S4). These findings indicate that activation of p53 by Nutlin-3 alleviates tumor growth, since in the erastin+Nutlin-3 group, the tumors were markedly smaller than those in the control group or the erastin group.

## 4. Discussion and Conclusion

Ferroptosis is an iron-mediated, caspase-independent process of cell death that requires the accumulation of lipid peroxides and ROS. Ferroptosis occurs due to dysregulation of reduction capacity that protects cells from oxidative stress instead of activation of cell death mechanisms related to necroptosis, pyroptosis, or apoptosis. So far, few studies have explored the natural function of ferroptosis, but large numbers of studies have shown that it is triggered by degenerative processes or may be instigated by therapeutics in some cancers. This ferroptosis pathway was first discovered in 2012 [3] and proved to be dominated by the GSH-regulated lipid repair enzyme, GPX4 [4]. As a central regulator in ferroptosis, GPX4 uses GSH as a cosubstrate for protecting cells by neutralising lipid peroxides, which are by-products of cellular metabolism.

The direct inhibition or indirect inhibition of GPX4 through depletion of its substrate GSH or building blocks of GSH can trigger then reduce ferroptosis. Two major ways to induce ferroptosis have been reported: type I inhibitors affect GPX4 by directly inhibiting the enzyme or by reducing expression levels [35] and include inhibitors such as RSL3, altretamine, ML162, and FIN56. Meanwhile, type II inhibitors result in depletion of GSH by blocking system x_c_^−^ and include inhibitors such as erastin, IKE [36], sorafenib [37], and sulfasalazine. System x_c_^−^ is the amino acid antiporter by which cancer cells take up extracellular cysteine, in the form of cystine, while exporting intracellular glutamate [5]. Cysteine participates in ferroptosis-related.

In this study, based on previous GSEA and the expression of specific proteins in tumor tissues, the system x_c_^−^ inhibitor, erastin, was generally selected as an antitumor drug and used as a ferroptosis inducer. The results show that, compared with HSC, schwannoma cells are more sensitive to inducers. On the one hand, this difference may be due to the functional decline of cysteine and glutathione metabolic regulation according to GSEA. As is well known, cysteine acts as the limiting substrate for GSH which contributes to the function of GPX4 in ferroptosis. The downregulation of cysteine and glutathione metabolism might enhance the effect of system x_c_^−^ inhibitor by reducing GPX4 pathway so as to make schwannoma cells more sensitive to erastin. On the other hand, the relatively low expression of SLC7A11/xCT, a regulatory component of the system x_c_^−^, might be one reason for the sensitivity to ferroptosis. It is hard to determine which factor is more critical in increasing sensitivity to erastin in VS cells. Another possibility is that lower expression of SLC7A11/xCT in VS might partly contribute to the downregulation of cysteine and glutathione metabolism and then work together with the downregulation of function to enhance the response to erastin. In any case, it can be inferred that the key functional proteins, SLC7A11/xCT, and key amino acid metabolic pathways, cysteine and glutathione metabolism, may be one reason for the varied susceptibility to system x_c_^−^ inhibitors in schwannoma cells.

Cysteine supports the cellular antioxidant system and promotes lipid ROS detoxification. Cellular cysteine can be synthesized through the cellular trans-sulphuration pathway in some cells, as well as taken up from extracellular matrix by the antiporter. To maintain an adequate supply of cysteine, in some cells, intracellular excess methionine is converted into cysteine through trans-sulphuration in various cancer cell lines [38]. However, it is less clear whether cysteine produced by the trans-sulphuration pathway can support GSH synthesis and maintain cytoactivity in schwannoma cells when extracellular sources of cysteine become limited. Nevertheless, in this study, given the decrease of both CBS and SLC7A11/xCT in VS, whether or not trans-sulphuration complements the source of cysteine has limited impact on GSH synthesis.

It has been increasingly recognized that some oncogenic pathways are related to ferroptosis, rendering cancer cells extremely vulnerable to ferroptotic death. Ferroptosis was implicated as a critical component of tumor suppression by p53 in 2015 [6], because it inhibits the expression of SLC7A11/xCT in the cystine/glutamate antiporter. The p53 tumor suppressor is best known as a transcription factor that either activates or inhibits mRNA synthesis by recognizing and binding to the promoters of target genes. The p53 protein could use its apoptosis and senescence pathways, as well as ferroptosis pathway, to supress tumor development. Previous study has proved that p53 downregulation is one of the mechanisms involved in tumorigenesis and development of schwannomas, acting by enhancing merlin stability [2] and activating p21 tumor suppressors [39]. However, revealing the mechanism underlying how p53 suppresses tumor initiation and progression has remained one of the key questions in VS.

Oncogenic alterations in the p53 network may modulate ferroptosis sensitivity through diverse mechanisms, contributing to either tumor suppression or oncogenesis. First, p53 is capable of reducing the expression of SLC7A11/xCT transcriptionally via binding to a specific gene sequence, resulting in reduced cystine import and increased lipid ROS [6]. The acetylation-defective mutant of p53 which cannot induce apoptosis retains almost complete ability to induce ferroptosis [6, 40, 41]. Meanwhile, mutant p53 which has lost its DNA-binding domain can still reduce SLC7A11/xCT levels by affecting other transcription factors [42], hinting that there might be an integrated transcription factor network which controls the expression of SLC7A11/xCT. Secondly, the importance of p53 as a metabolic gene regulator in ferroptosis has been reported, as several metabolism-related genes, such as SAT1, FDXR88, and GLS2, responded to p53-mediated ferroptosis in a variety of conditions [43, 44]. In addition, in human colorectal cancer cells, p53 directly binds the dipeptidyl peptidase DPP4 to inhibit NOX-mediated lipid peroxidation, and DPP4 inhibitors limit the anticancer activity of ferroptosis activators [45]. In fibrosarcoma cells, p53 also limits ferroptosis by inducing CDKN1A expression [46]. Thirdly, MDM2 and MDMX bind p53 and regulate its stability, thus promoting ferroptosis in a p53-independent manner [47], and implying that the function of p53 in ferroptosis might not depend on the MDM family.

In schwannoma cells, based on the above results and previous studies [6], we speculate that SLC7A11/xCT protein levels are downregulated by p53, thus enhancing the sensitivity of p53-overexpressing schwannoma cells to ferroptosis as the response to erastin is more intense. As shown in several studies, p53 recognizes and binds to the SLC7A11/xCT gene and downregulates its level transcriptionally, as the 5′ flanking region of the SLC7A11/xCT gene at chromosome 4q28-31 [30] contains one site that matches the consensus p53-binding sequence. SLC7A11/xCT is a key part of the system x_c_^−^ cystine/glutamate antiporter, and the decrease of SLC7A11/xCT reduces cystine uptake levels [6]. In some malignant cells, SLC7A11 is highly expressed and its overexpression reduces ferroptosis, which is perhaps the adaptive response to increased sensitivity of systemic cysteine depletion [41]. However, in our studies, compared with normal nerves, the expression of SLC7A11/xCT decreased in schwannoma tissues, as well as the metabolic regulation of cystine and GSH. These biological characteristics might explain why schwannoma cells are more sensitive to ferroptosis inducers than normal schwann cells.

However, different from predictions, there was little change in the expression of SLC7A11/xCT when p53 was inhibited in schwannoma cells; therefore, sensitivity to ferroptosis did not weaken in p53-null tumor cells (data not shown). There is one potential explanation for this outcome. In schwannomas, p53 is expressed at a low level and rarely binds to the regulatory region under nonstress conditions, so it has a lesser effect on SLC7A11/xCT when p53 is further inhibited. During establishing VS cell lines, JEI-001 was immortalized by a hHERT and JEI-002 was immortalized by SV40. As the hHERT gene has been found to stabilize telomere length and the SV40 gene has been found to bind to a variety of proteins, these immortalizations which prolong cell cycle and promote the expression of cell immortalization related genes partly disrupt p53-dependent pathway. And maybe this is one reason that why p53 silencing is fail to further improve xCT expression. The role of p53 in regulating ferroptosis can vary with context.

In a study by Chen et al., a “co-expression” relationship between p53 and NF2 in vestibular schwannomas was proved, as well as the overexpression of merlin accompanied by the translocation of p53 into the nucleus [2]. Based on this, we hypothesized that merlin could also promote ferroptosis through its interaction with p53; however, overexpression of NF2 did not change the sensitivity to erastin. One possible explanation might be the conflicting roles of p53 and NF2 in ferroptosis. In some epithelial and mesenchymal cancer cells, antagonizing NF2 allows the protooncogenic transcriptional coactivator YAP to promote ferroptosis via several modulators [48]. Therefore, we speculate that although the overexpression of merlin could inhibit ferroptosis; at the same time, merlin promotes the translocation of p53 into the nucleus, which may decrease expression of SLC7A11/xCT, promote ferroptosis, and finally offset the inhibition of merlin.

To date, precise information on the relevance of tumor suppression by p53 to p53-mediated ferroptosis has not yet been elucidated. However a firmer understanding of these potential functions and mechanisms may give us a deeper insight into the evolution of p53 in schwannoma, including not only its action via the p21/cyclinD1 axis but also p53 blockage of the proliferation of schwannoma cells via synergism of p53 and merlin [2]. More importantly, a better exploration of how p53 mediates tumor suppression in VS would support the design and testing of novel therapy strategies. More and more discoveries will continue to promote the search for the key activities of p53 and its anticancer effects.

## Figures and Tables

**Table 1:** STR results for tumor tissue and JEI-002.

Marker	Tissue		Cell line	
	Allele 1	Allele 2	Allele 1	Allele 2
01-D5S818	11	13	11	13
02-D13S317	8	13	8	13
03-D7S820	10	11	10	11
04-D16S539	11	13	11	13
05-VWA	16	18	16	18
06-TH01	7	9.3	7	9.3
07-AMEL	X	X	X	X
08-TPOX	8	12	8	12
09-CSF1PO	10	10	10	10
10-D12S391	19	21	19	21
11-FGA	23	24	23	24
12-D2S1338	18	25	18	25
13-D21S11	30	32.2	30	32.2
14-D18S51	13	18	13	18
15-D8S1179	13	16	13	16
16-D3S1358	15	18	15	18
17-D6S1043	11	22	11	22
18-PENTAE	14	21	14	21
19-D19S433	13.2	14	13.2	14
20-PENTAD	9	12	9	12

## Data Availability

All original datasets generated for this study are included in the article and supplementary material; further requests are available from the corresponding authors.

## Supplementary Materials

Supplementary 1 Supplementary Figure: (S1) original WB data for Figure 3(a). (S2) Original WB data for Figure 1(c). (S3) Original WB data for Figure 5(b). (S4) Original data for bottom panel of Figure 6(b).

Supplementary 2 Supplementary Table 1: complete list of differentially expressed genes (DEGs) of vestibular schwannomas compared with human normal nerves.

Supplementary 3 Supplementary Table 2: top deregulated gene sets from GSEA analysis.
