# Supplementary figures and images for: Synergistic Effect of Erastin Combined with Nutlin-3 on Vestibular Schwannoma Cells as p53 Modulates Erastin-Induced Ferroptosis Response
Source: J Oncol. 2022 Mar 21;2022:7507857. doi: 10.1155/2022/7507857 (PMC8961447; doi:10.1155/2022/7507857)

**Figure S1**

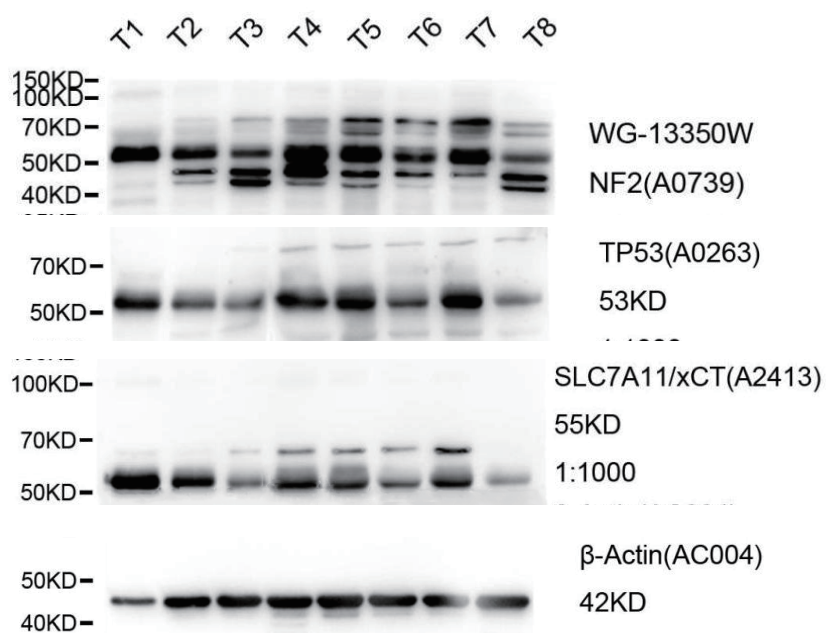

**Figure S2**

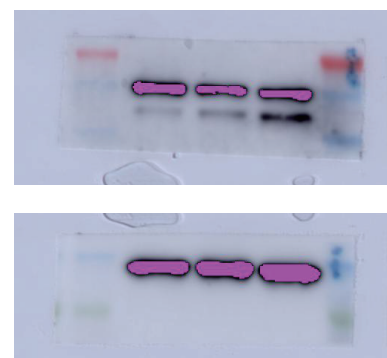

**Figure S3**

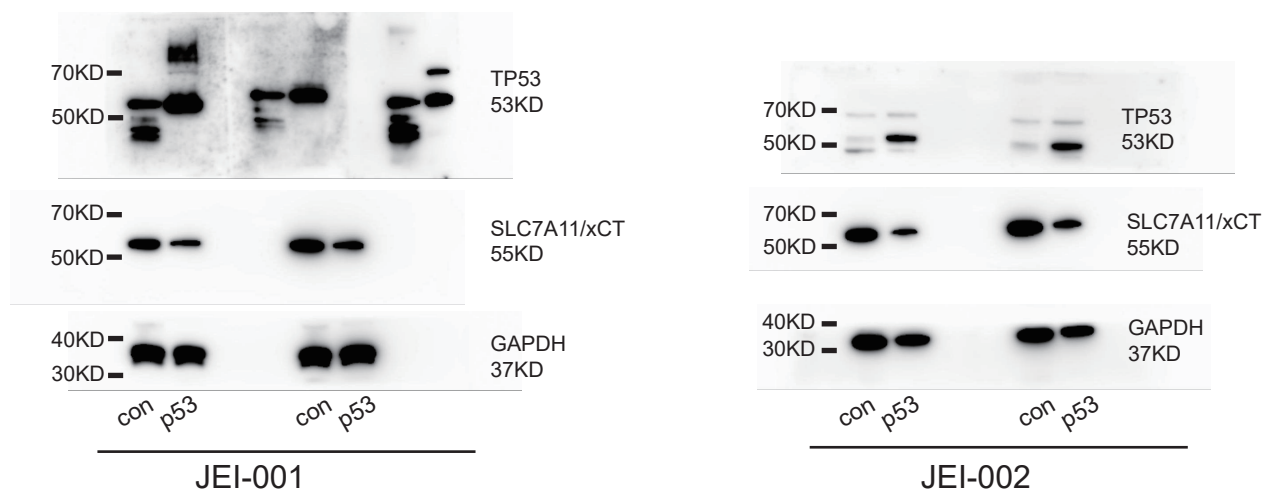

**Figure S4**

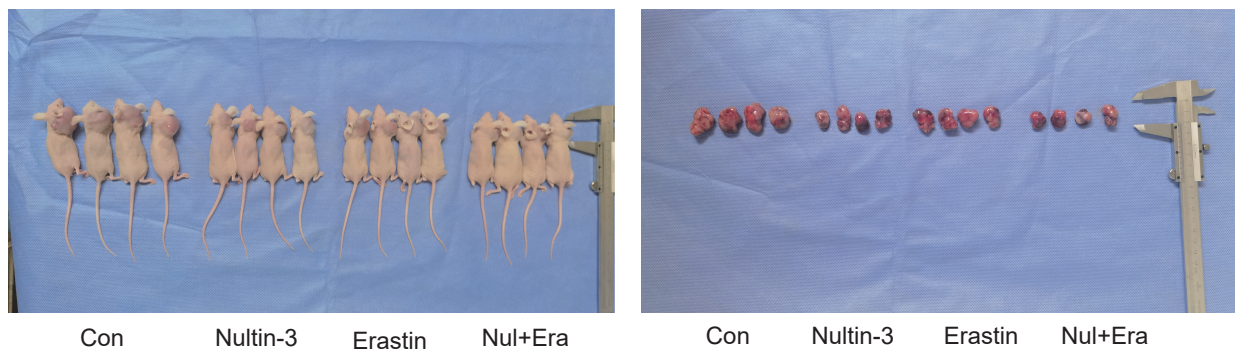

Supplement: Supplementary 1 — Supplementary Figure: (S1) original WB data for Figure 3(a). (S2) Original WB data for Figure 1(c). (S3) Original WB data for Figure 5(b). (S4) Original data for bottom panel of Figure 6(b). [file 7507857.f1.pdf]
