# Supplementary material for: Synergistic Effect of Erastin Combined with Nutlin-3 on Vestibular Schwannoma Cells as p53 Modulates Erastin-Induced Ferroptosis Response
Source: J Oncol. 2022 Mar 21;2022:7507857. doi: 10.1155/2022/7507857 (PMC8961447; doi:10.1155/2022/7507857)
Supplement: Supplementary 2 — Supplementary Table 1: complete list of differentially expressed genes (DEGs) of vestibular schwannomas compared with human normal nerves. [file 7507857.f2.pdf]

gene_id	T_VS	Ctrl	log2FoldChange	pvalue	padj	gene_name
ENSG00000106366	232.0903255	140456.8487	-9.241025527	3.30E-276	8.57E-272	SERPINE1
ENSG00000165092	26023.08633	26.69177742	9.924282371	2.21E-198	2.87E-194	ALDH1A1
ENSG00000019582	74272.78678	11.96639716	12.58706607	6.57E-164	5.69E-160	CD74
ENSG00000158887	64355.59062	77.13439585	9.704404015	1.20E-123	7.77E-120	MPZ
ENSG00000171766	13548.00568	5.391691008	11.29699516	3.93E-119	2.04E-115	GATM
ENSG00000101938	3679.234125	7.134247879	9.003621468	1.01E-111	4.39E-108	CHRDL1
ENSG00000106565	4852.638789	11.04787628	8.77290322	1.29E-108	4.77E-105	TMEM176B
ENSG00000091409	14400.859	396.6089791	5.183055506	7.42E-103	2.41E-99	ITGA6
ENSG00000145335	4533.77252	15.28607785	8.222349348	2.44E-98	7.05E-95	SNCA
ENSG00000197971	27790.00794	218.4320041	6.991323482	1.54E-95	4.01E-92	MBP
ENSG00000150630	85.49704281	2727.230107	-4.995791116	2.30E-95	5.43E-92	VEGFC
ENSG00000109099	35872.97618	389.0448636	6.526112735	2.13E-94	4.62E-91	PMP22
ENSG00000012171	48787.79257	195.5072197	7.961856235	4.81E-91	9.61E-88	SEMA3B
ENSG00000118971	5822.763817	477.9939056	3.607038252	1.72E-90	3.19E-87	CCND2
ENSG00000157368	4448.03694	4.259554931	10.01507423	5.39E-89	9.34E-86	IL34
ENSG00000105767	15198.43065	208.7681723	6.18742897	1.07E-87	1.74E-84	CADM4
ENSG00000007237	13773.80616	38.94633396	8.460068502	4.88E-87	7.46E-84	AC005747.1
ENSG00000130558	3569.682069	12.95329767	8.108392514	3.81E-85	5.50E-82	OLFM1
ENSG00000110876	1422.249339	43.6577647	5.029024473	4.61E-85	6.31E-82	SELPLG
ENSG00000169679	19.42885831	1441.701928	-6.215418237	7.26E-82	9.44E-79	BUB1
ENSG00000137642	4251.839482	6.266477676	9.431606908	1.10E-80	1.36E-77	SORL1
ENSG00000165795	13415.81388	18.76045071	9.491767203	1.30E-78	1.54E-75	NDRG2
ENSG00000174607	2234.303166	5.982042707	8.523688402	4.95E-77	5.59E-74	UGT8
ENSG00000123485	12.048375	1252.42273	-6.695713567	8.28E-77	8.97E-74	HJURP
ENSG00000141526	321.1446943	18732.55191	-5.866050698	1.21E-76	1.26E-73	SLC16A3
ENSG00000170458	4924.094571	26.63614645	7.536893736	9.51E-73	9.51E-70	CD14
ENSG00000138160	43.55458478	1443.509011	-5.050378341	3.88E-72	3.74E-69	KIF11
ENSG00000180354	5970.646715	298.4158867	4.323148999	5.64E-72	5.23E-69	MTURN
ENSG00000153902	18701.67573	2.476859868	12.86783933	7.94E-70	7.12E-67	LGI4
ENSG00000185818	1431.429345	18.15015831	6.309607038	1.20E-69	1.04E-66	NAT8L
ENSG00000010327	3252.035123	9.640633454	8.394231196	9.56E-69	8.01E-66	STAB1
ENSG00000137285	2696.559878	84.30176545	4.998113245	1.20E-68	9.71E-66	TUBB2B
ENSG00000005513	3275.408571	5.410604229	9.246249661	1.43E-68	1.13E-65	SOX8
ENSG00000105825	255.9612035	24715.55377	-6.593422548	1.95E-68	1.49E-65	TFPI2
ENSG00000065361	8181.601325	6.124838129	10.38645242	1.42E-67	1.05E-64	ERBB3
ENSG00000168071	1091.456602	14.51048079	6.241753077	1.96E-67	1.42E-64	CCDC88B
ENSG00000178573	3932.815685	2.833398881	10.42285637	2.75E-67	1.93E-64	MAF
ENSG00000130208	1739.002059	28.39188434	5.930590763	1.17E-66	7.89E-64	APOC1
ENSG00000180155	1676.805059	39.8480778	5.395334298	1.18E-66	7.89E-64	LYNX1
ENSG00000181409	16619.43715	11.87178388	10.4652797	2.46E-66	1.60E-63	AATK
ENSG00000070731	1966.463477	6.074087452	8.334434263	5.10E-66	3.23E-63	ST6GALNAC2
ENSG00000215018	5196.511387	8.711500088	9.227502403	1.28E-65	7.90E-63	COL28A1
ENSG00000102879	2985.588018	112.3250856	4.732221457	8.79E-65	5.31E-62	CORO1A
ENSG00000021300	8067.206321	1.702418679	12.14202587	3.68E-64	2.17E-61	PLEKHB1
ENSG00000196338	1680.43853	18.6910436	6.479423791	5.60E-64	3.23E-61	NLGN3
ENSG00000187260	4221.680509	7.309009153	9.151468719	6.39E-64	3.61E-61	WDR86
ENSG00000089041	891.375745	13.05364315	6.102608315	8.90E-64	4.92E-61	P2RX7
ENSG00000100644	1264.852174	26765.7696	-4.403356636	1.44E-63	7.78E-61	HIF1A
ENSG00000184661	13.33222965	676.7925174	-5.666556546	2.14E-63	1.13E-60	CDCA2
ENSG00000276043	58.85507827	1835.992571	-4.96244413	2.75E-62	1.40E-59	UHRF1

ENSG00000185274	2350.916399	7.624254098	8.239679326	2.75E-62	1.40E-59	GALNT17
ENSG00000235098	1000.244627	11.77986753	6.403010446	4.71E-62	2.35E-59	ANKRD65
ENSG00000079819	14171.27006	1575.473817	3.168993027	1.21E-61	5.95E-59	EPB41L2
ENSG00000106780	5097.385642	234.2180584	4.443167263	1.27E-61	6.13E-59	MEGF9
ENSG00000171992	5373.211401	225.4842246	4.573602519	2.34E-61	1.10E-58	SYNPO
ENSG00000162654	1129.309432	10.14125216	6.776330232	3.24E-61	1.50E-58	GBP4
ENSG00000161888	21.11014955	687.0561919	-5.021723161	6.43E-61	2.93E-58	SPC24
ENSG00000100307	1678.88165	90.45471662	4.215587161	1.06E-60	4.73E-58	CBX7
ENSG00000123975	77.69111537	1645.344261	-4.404204767	4.22E-60	1.86E-57	CKS2
ENSG00000086730	1204.62443	27.15799012	5.47614534	4.36E-60	1.89E-57	LAT2
ENSG00000100146	10798.70008	3.300895859	11.69454765	7.12E-60	3.03E-57	SOX10
ENSG00000143248	2179.105406	39.47403823	5.779853399	1.23E-59	5.16E-57	RGS5
ENSG00000018625	9343.698174	2.180528143	12.07890759	1.31E-59	5.39E-57	ATP1A2
ENSG00000198915	997.9921688	26.13925216	5.253246974	1.60E-59	6.48E-57	RASGEF1A
ENSG00000168209	538.7169342	6171.472038	-3.517839017	1.99E-59	7.96E-57	DDIT4
ENSG00000125848	2057.398324	23.86783515	6.426015737	2.85E-59	1.12E-56	FLRT3
ENSG00000077157	2230.03683	221.9489553	3.328964777	6.97E-59	2.70E-56	PPP1R12B
ENSG00000164109	52.85422581	1456.729595	-4.783891489	1.19E-58	4.55E-56	MAD2L1
ENSG00000134333	3183.081898	36297.19864	-3.511375359	1.21E-58	4.55E-56	LDHA
ENSG00000099282	2891.754644	43.60585815	6.052601207	2.05E-58	7.62E-56	TSPAN15
ENSG00000179403	12319.04335	187.9820612	6.034754434	3.32E-58	1.21E-55	VWA1
ENSG00000170075	1864.37526	4.299693123	8.75811749	4.29E-58	1.55E-55	GPR37L1
ENSG00000068489	58.62981954	1068.413768	-4.18723363	5.64E-58	1.99E-55	PRR11
ENSG00000180448	1827.652236	63.06291381	4.858478204	5.68E-58	1.99E-55	AC004151.1
ENSG00000176619	1082.311774	7942.656582	-2.875536384	1.54E-57	5.35E-55	LMNB2
ENSG00000134057	56.36301552	3379.853528	-5.905186195	1.92E-57	6.57E-55	CCNB1
ENSG00000134121	10700.13205	6.358522421	10.74157054	3.11E-57	1.05E-54	CHL1
ENSG00000139549	2480.906075	2.649180997	9.937143008	3.62E-57	1.20E-54	DHH
ENSG00000089820	1931.483561	15.15633506	6.983274408	1.99E-56	6.54E-54	ARHGAP4
ENSG00000139734	24.52101176	1006.21859	-5.359035621	2.48E-56	8.05E-54	DIAPH3
ENSG00000053702	2400.013173	60.70865188	5.308744254	1.20E-55	3.85E-53	NRIP2
ENSG00000161638	558.2114648	35854.45201	-6.005257816	2.03E-55	6.44E-53	ITGA5
ENSG00000176533	842.1853338	8.75395003	6.603527295	4.86E-55	1.52E-52	GNG7
ENSG00000160255	3556.942564	35.76901634	6.640875278	1.01E-54	3.13E-52	ITGB2
ENSG00000128268	1896.804894	79.61583203	4.576662503	1.41E-54	4.32E-52	MGAT3
ENSG00000109458	2310.383266	178.7942495	3.691037819	1.45E-54	4.37E-52	GAB1
ENSG00000170271	1706.138302	104.612126	4.029127552	2.48E-54	7.39E-52	FAXDC2
ENSG00000101057	11.19010703	1092.599559	-6.608836882	2.62E-54	7.73E-52	MYBL2
ENSG00000187955	16314.7266	12.86138132	10.29783761	3.42E-54	9.98E-52	COL14A1
ENSG00000131747	78.64842229	6080.0661	-6.271441654	1.05E-52	3.02E-50	TOP2A
ENSG00000137807	66.05439437	1502.374003	-4.507891115	1.15E-52	3.29E-50	AC027237.1
ENSG00000186417	4132.749682	5.208757394	9.606883327	1.53E-52	4.32E-50	GLDN
ENSG00000260807	1652.227708	6.135450615	8.078227231	8.25E-52	2.31E-49	AC009041.2
ENSG00000105559	17048.29383	528.9979236	5.00981992	9.10E-52	2.52E-49	PLEKHA4
ENSG00000150471	1433.985974	4.963176346	8.162504	1.02E-51	2.80E-49	ADGRL3
ENSG00000133401	6050.748667	80.76778042	6.229904872	2.88E-51	7.78E-49	PDZD2
ENSG00000158008	1812.054468	31.36910613	5.854066866	6.72E-51	1.80E-48	EXTL1
ENSG00000064300	3127.796297	4.013973884	9.620340886	1.08E-50	2.86E-48	NGFR
ENSG00000162944	962.0923887	29.53347703	5.021066854	1.39E-50	3.65E-48	RFTN2
ENSG00000166682	1573.405752	6.490833752	7.925039537	2.19E-50	5.70E-48	TMPRSS5
ENSG00000138778	34.33435124	972.5985088	-4.825886546	2.36E-50	6.08E-48	CENPE

ENSG00000160307	49226.27692	0	17.92087751	4.96E-50	1.26E-47	S100B
ENSG00000151892	3674.717287	1.702418679	11.00982326	6.01E-50	1.52E-47	GFRA1
ENSG00000171791	1928.618386	16.37589238	6.875532216	8.33E-50	2.08E-47	BCL2
ENSG00000117399	29.04563079	2504.180769	-6.4298037	1.89E-49	4.68E-47	CDC20
ENSG00000109805	23.12508347	1171.673512	-5.659469641	2.01E-49	4.92E-47	NCAPG
ENSG00000147588	50215.51433	0	17.94958079	2.33E-49	5.65E-47	PMP2
ENSG00000130294	4154.078023	1.518200796	11.49235791	2.63E-49	6.33E-47	KIF1A
ENSG00000073111	52.05376822	962.8561644	-4.209385852	7.98E-49	1.90E-46	MCM2
ENSG00000111679	932.8324823	5.289033778	7.444303021	1.74E-48	4.11E-46	PTPN6
ENSG00000114166	2561.707296	162.459604	3.979198499	4.12E-48	9.64E-46	KAT2B
ENSG00000002933	3899.020262	9.979543515	8.588553991	9.15E-48	2.12E-45	TMEM176A
ENSG00000137841	2183.206374	28.55950076	6.250138682	2.41E-47	5.55E-45	PLCB2
ENSG00000072571	12.96341352	920.4908317	-6.147469473	3.04E-47	6.93E-45	HMMR
ENSG00000058085	22.1158784	459.7594897	-4.377803044	3.12E-47	7.05E-45	LAMC2
ENSG00000092421	747.1773534	16.67093983	5.492703392	3.81E-47	8.53E-45	SEMA6A
ENSG00000160781	2275.553102	36.22004024	5.976312074	4.01E-47	8.91E-45	PAQR6
ENSG00000135929	2107.012095	239.5524961	3.136970536	5.66E-47	1.25E-44	CYP27A1
ENSG00000137491	2410.739806	2.231278821	10.11931316	6.48E-47	1.42E-44	SLCO2B1
ENSG00000128050	585.9756064	5481.556913	-3.22583213	6.74E-47	1.46E-44	PAICS
ENSG00000142945	30.47282937	1389.415345	-5.509726325	8.17E-47	1.75E-44	KIF2C
ENSG00000102109	888.270913	12.18946897	6.186692691	8.27E-47	1.76E-44	PCSK1N
ENSG00000204682	3638.511147	137.5830014	4.726947327	1.28E-46	2.71E-44	MIR1915HG
ENSG00000010610	1597.056475	8.438833479	7.549768459	1.38E-46	2.89E-44	CD4
ENSG00000166432	1936.201908	5.676254372	8.404762878	4.75E-46	9.87E-44	ZMAT1
ENSG00000071539	19.20331485	1182.507752	-5.94040963	4.83E-46	9.97E-44	TRIP13
ENSG00000178695	10222.68496	607.8649399	4.071410072	5.20E-46	1.06E-43	KCTD12
ENSG00000153993	474.722875	11.69959115	5.331355046	6.10E-46	1.24E-43	SEMA3D
ENSG00000142347	1315.525939	62.0713898	4.405381358	6.40E-46	1.29E-43	MYO1F
ENSG00000175063	21.37323155	1593.500517	-6.218152998	7.03E-46	1.40E-43	UBE2C
ENSG00000154678	990.7279934	6.888666832	7.177003256	8.56E-46	1.70E-43	PDE1C
ENSG00000126562	20.40177849	580.2272867	-4.83319801	9.05E-46	1.78E-43	WNK4
ENSG00000079931	4453.85741	89.640698	5.631740676	1.52E-45	2.96E-43	MOXD1
ENSG00000135378	621.7240065	8.835510684	6.122717659	1.77E-45	3.43E-43	PRRG4
ENSG00000021645	1867.536208	3.210006989	9.177853068	1.90E-45	3.65E-43	NRXN3
ENSG00000112742	14.59086878	616.6902236	-5.404328154	2.00E-45	3.83E-43	TTK
ENSG00000024526	7.474649858	980.4536347	-7.026347066	2.08E-45	3.94E-43	DEPDC1
ENSG00000143110	735.9232644	17.0594447	5.424213999	2.61E-45	4.91E-43	C1orf162
ENSG00000149503	142.2730373	1009.918882	-2.827473186	3.13E-45	5.85E-43	INCENP
ENSG00000105894	1819.812806	76.46710699	4.574182535	4.31E-45	7.99E-43	PTN
ENSG00000102934	2669.269396	34.0690378	6.294800461	4.57E-45	8.41E-43	PLLP
ENSG00000115290	1106.328151	21.56573655	5.67331957	7.17E-45	1.31E-42	GRB14
ENSG00000143226	1407.968033	3.475657132	8.628782508	8.07E-45	1.47E-42	FCGR2A
ENSG00000154856	1524.105337	5.952517001	7.980568175	1.15E-44	2.08E-42	APCDD1
ENSG00000090889	19.64527023	916.0373923	-5.542541679	1.95E-44	3.50E-42	KIF4A
ENSG00000235162	163.6420371	2446.875312	-3.902956437	2.03E-44	3.61E-42	C12orf75
ENSG00000156970	20.14607618	954.2386696	-5.562660921	2.12E-44	3.74E-42	BUB1B
ENSG00000135451	9.610504235	817.0986617	-6.404437485	2.15E-44	3.77E-42	TROAP
ENSG00000155849	1631.057862	5.463795051	8.181101191	2.40E-44	4.19E-42	ELMO1
ENSG00000162512	10506.22661	711.9604536	3.882828457	2.52E-44	4.36E-42	SDC3
ENSG00000203760	21.52688941	506.4665931	-4.557393643	3.06E-44	5.27E-42	CENPW
ENSG00000163535	46.32728061	660.1769514	-3.833375825	3.99E-44	6.82E-42	SGO2

ENSG00000130592	2106.142208	6.931245169	8.223517461	4.87E-44	8.27E-42	LSP1
ENSG00000089685	22.78649746	1962.890472	-6.425540003	5.44E-44	9.18E-42	BIRC5
ENSG00000182253	1543.606858	121.1780969	3.672275538	6.49E-44	1.09E-41	SYNM
ENSG00000158869	1335.865205	9.438786619	7.127617301	1.02E-43	1.70E-41	FCER1G
ENSG00000102547	3790.06061	176.4111853	4.426316834	1.06E-43	1.75E-41	CAB39L
ENSG00000113083	309.1008482	10442.94567	-5.078593313	1.31E-43	2.15E-41	LOX
ENSG00000174348	1277.627811	6.593619377	7.571201438	1.33E-43	2.18E-41	PODN
ENSG00000105011	38.67117881	649.015339	-4.068277164	1.47E-43	2.38E-41	ASF1B
ENSG00000171812	1985.821511	72.6367087	4.768765183	1.76E-43	2.84E-41	COL8A2
ENSG00000076382	82.9883979	1386.553577	-4.061863214	2.31E-43	3.70E-41	SPAG5
ENSG00000169607	13.48058818	913.5854565	-6.076697636	4.72E-43	7.52E-41	CKAP2L
ENSG00000095713	959.3876305	2.751966622	8.39466529	6.30E-43	9.98E-41	CRTAC1
ENSG00000149633	5125.346751	114.88911	5.480076783	6.54E-43	1.03E-40	KIAA1755
ENSG00000139597	485.1920466	8.775303396	5.768143957	7.64E-43	1.20E-40	N4BP2L1
ENSG00000127589	505.0482156	4010.324104	-2.989167073	1.44E-42	2.24E-40	TUBBP1
ENSG00000146938	1971.976285	2.842855491	9.427524327	1.58E-42	2.45E-40	NLGN4X
ENSG00000121621	11.16614516	456.6625422	-5.351878978	1.77E-42	2.72E-40	KIF18A
ENSG00000135077	732.9971249	3.822739535	7.547377998	2.09E-42	3.20E-40	HAVCR2
ENSG00000196218	1115.293878	3.148643826	8.440107988	2.53E-42	3.85E-40	RYR1
ENSG00000121039	4408.881693	107.7648794	5.352917559	2.56E-42	3.86E-40	RDH10
ENSG00000145416	1133.796869	61.70680684	4.1955765	2.77E-42	4.16E-40	1-Mar
ENSG00000137309	792.5087202	19621.62803	-4.629826955	3.86E-42	5.77E-40	HMGA1
ENSG00000156475	1805.7548	1.702418679	9.984239139	4.94E-42	7.33E-40	PPP2R2B
ENSG00000161921	1474.318047	15.40076023	6.568457764	6.50E-42	9.60E-40	CXCL16
ENSG00000175445	6133.246658	1.702418679	11.7597983	6.69E-42	9.81E-40	LPL
ENSG00000143126	974.8856477	118.9364624	3.034442028	7.31E-42	1.07E-39	CELSR2
ENSG00000117650	5.718965719	576.7469845	-6.651167123	7.59E-42	1.10E-39	NEK2
ENSG00000151150	4078.073006	59.92971558	6.089463567	8.83E-42	1.27E-39	ANK3
ENSG00000101447	3.928036872	723.2676528	-7.522997664	1.08E-41	1.55E-39	FAM83D
ENSG00000165480	8.397210272	509.3220685	-5.915164199	1.47E-41	2.10E-39	SKA3
ENSG00000171860	1120.339684	2.079026788	9.02913498	1.77E-41	2.51E-39	C3AR1
novel.86	3.32769377	511.940427	-7.264070359	2.38E-41	3.36E-39	-
ENSG00000206052	893.1434125	6.796622087	7.034964292	2.52E-41	3.53E-39	DOK6
ENSG00000169213	5.834871573	1938.827409	-8.385112357	2.77E-41	3.87E-39	RAB3B
ENSG00000176887	3.038213869	627.5222076	-7.694172545	2.90E-41	4.04E-39	SOX11
ENSG00000114948	10458.58623	439.0705476	4.574529972	3.37E-41	4.66E-39	ADAM23
ENSG00000261371	1406.130148	33.89453332	5.372315396	3.52E-41	4.84E-39	PECAM1
ENSG00000198554	61.46680781	587.3840907	-3.256742747	8.46E-41	1.16E-38	WDHD1
ENSG00000184060	477.1340627	10.57806756	5.479794979	9.01E-41	1.23E-38	ADAP2
ENSG00000136999	2987.911019	69.98799442	5.418640209	9.98E-41	1.35E-38	CCN3
ENSG00000145703	540.1630641	7.338534859	6.176955088	1.06E-40	1.42E-38	IQGAP2
ENSG00000182578	5049.700368	56.40061084	6.488434939	1.48E-40	1.98E-38	CSF1R
ENSG00000196376	2316.254304	1.742556871	10.34126599	1.68E-40	2.24E-38	SLC35F1
ENSG00000171208	73.71551076	906.6000527	-3.621290491	1.70E-40	2.26E-38	NETO2
ENSG00000255197	518.3287443	8.876804751	5.858174114	1.76E-40	2.33E-38	AC090559.1
ENSG00000129675	3061.477847	234.904004	3.705263816	1.93E-40	2.53E-38	ARHGEF6
ENSG00000162511	4386.464497	32.51411926	7.0820515	4.24E-40	5.54E-38	LAPTM5
ENSG00000128604	712.5722388	2.954969332	7.948411626	5.66E-40	7.35E-38	IRF5
ENSG00000133246	427.4723928	3.984448178	6.756509849	6.78E-40	8.76E-38	PRAM1
ENSG00000067840	3098.217138	23.39648536	7.059010423	1.27E-39	1.64E-37	PDZD4
ENSG00000132561	9602.780888	58.04492405	7.366390911	1.28E-39	1.64E-37	MATN2

ENSG00000093009	11.38381073	960.7137329	-6.393404328	1.43E-39	1.82E-37	CDC45
ENSG00000011201	591.8726477	16.47970548	5.165390795	1.79E-39	2.27E-37	ANOS1
ENSG00000143228	28.40753568	788.8997623	-4.795010044	2.38E-39	3.01E-37	NUF2
ENSG00000166428	1775.962366	5.53217468	8.345957171	2.79E-39	3.50E-37	PLD4
ENSG00000184574	462.7384364	3.627909165	7.008098869	3.19E-39	3.98E-37	LPAR5
ENSG00000166073	456.3282669	2728.528107	-2.579745581	3.43E-39	4.26E-37	GPR176
ENSG00000219438	5044.334657	1.10029862	12.190148	4.08E-39	5.04E-37	TAFA5
ENSG00000069188	574.2421997	8.752794155	6.049056978	4.68E-39	5.76E-37	SDK2
ENSG00000132182	429.6468534	5.819049794	6.220274745	4.75E-39	5.83E-37	NUP210
ENSG00000087586	41.76770161	1553.018738	-5.218386151	5.38E-39	6.56E-37	AURKA
ENSG00000164647	4.277976998	1020.215269	-7.905175056	5.84E-39	7.09E-37	STEAP1
ENSG00000164045	11.16865246	402.1208404	-5.168404918	6.12E-39	7.40E-37	CDC25A
ENSG00000165868	6334.832548	266.4353795	4.571594136	1.06E-38	1.27E-36	HSPA12A
ENSG00000179583	2021.20795	53.69263522	5.236811393	2.08E-38	2.49E-36	CIITA
ENSG00000197892	1490.345779	199.1284848	2.903808334	2.28E-38	2.72E-36	KIF13B
ENSG00000188487	1317.935884	3.566546002	8.523452668	2.31E-38	2.74E-36	INSC
ENSG00000140678	1424.677539	15.16206725	6.568014493	2.36E-38	2.79E-36	ITGAX
ENSG00000163219	501.2340317	4.566499142	6.755246461	3.17E-38	3.72E-36	ARHGAP25
ENSG00000172020	1344.474295	4.280779903	8.290093008	3.87E-38	4.53E-36	GAP43
ENSG00000148053	5377.066161	1.029478846	12.29423592	4.07E-38	4.74E-36	NTRK2
ENSG00000146122	2656.217031	344.9390159	2.944338033	4.86E-38	5.63E-36	DAAM2
ENSG00000169258	11.20013622	283.2107744	-4.66054853	5.77E-38	6.66E-36	GPRIN1
ENSG00000133477	1311.974646	1.844058227	9.508482216	6.54E-38	7.52E-36	FAM83F
ENSG00000076344	2271.884352	23.00122082	6.626550934	6.58E-38	7.53E-36	RGS11
ENSG00000113578	5391.059667	57.55234929	6.547254765	7.58E-38	8.63E-36	FGF1
ENSG00000107902	1637.177723	180.0948539	3.185025258	8.83E-38	1.00E-35	LHPP
ENSG00000159217	2.158491326	1251.130084	-9.19820343	1.14E-37	1.29E-35	IGF2BP1
ENSG00000163431	532.0991137	23.04264328	4.521926006	1.33E-37	1.50E-35	LMOD1
ENSG00000197943	457.8242301	15.05354943	4.925882035	1.48E-37	1.66E-35	PLCG2
ENSG00000174125	478.0997892	3.781445468	6.932766335	1.54E-37	1.72E-35	TLR1
ENSG00000129244	938.3443825	1.762625967	9.03249133	2.20E-37	2.44E-35	ATP1B2
ENSG00000088325	67.76960574	3382.619035	-5.640734765	2.79E-37	3.09E-35	TPX2
ENSG00000064225	1782.842398	80.7184424	4.46691082	3.00E-37	3.30E-35	ST3GAL6
ENSG00000099889	3527.338892	83.09996548	5.406753973	3.68E-37	4.04E-35	ARVCF
ENSG00000162645	3336.472802	72.73636502	5.52225319	3.78E-37	4.12E-35	GBP2
ENSG00000069535	525.3623096	3.108505634	7.355094447	4.12E-37	4.48E-35	MAOB
ENSG00000186469	8739.546095	300.7035325	4.86026136	4.88E-37	5.28E-35	GNG2
ENSG00000148655	439.6550508	7.114178783	5.941337298	5.22E-37	5.63E-35	LRMDA
ENSG00000144810	1070.875279	14455.16496	-3.754819409	5.28E-37	5.67E-35	COL8A1
ENSG00000124406	816.9720957	12.1481749	6.068911923	5.58E-37	5.96E-35	ATP8A1
ENSG00000125430	29.94415806	2881.324935	-6.588054393	6.09E-37	6.49E-35	HS3ST3B1
ENSG00000126787	6.870545809	1314.432174	-7.572030823	6.27E-37	6.65E-35	DLGAP5
ENSG00000141480	1969.496935	150.2180188	3.712903335	7.06E-37	7.45E-35	ARRB2
ENSG00000081237	1605.314915	1.783850939	9.806924371	8.30E-37	8.73E-35	PTPRC
ENSG00000196611	3.948166524	1930.836835	-8.935985055	8.98E-37	9.41E-35	MMP1
ENSG00000102996	6175.286369	516.9813112	3.578876889	1.03E-36	1.07E-34	MMP15
ENSG00000170779	120.3686984	1130.150183	-3.231132362	1.43E-36	1.49E-34	CDCA4
ENSG00000049246	1478.34683	34.39515202	5.421930217	1.79E-36	1.85E-34	PER3
ENSG00000077152	31.64224528	713.7517537	-4.497698751	1.88E-36	1.94E-34	UBE2T
ENSG00000071991	10454.59395	0	15.6855921	2.08E-36	2.14E-34	CDH19
ENSG00000133056	404.1241741	8.112976048	5.622790529	2.13E-36	2.18E-34	PIK3C2B

ENSG00000169508	457.1528278	5.492036488	6.399167808	2.61E-36	2.66E-34	GPR183
ENSG00000225217	1652.559041	6.582878497	7.983864301	2.80E-36	2.84E-34	HSPA7
ENSG00000074800	8132.948608	56209.79752	-2.788982076	3.64E-36	3.68E-34	ENO1
ENSG00000188906	995.2559865	37.75155045	4.714431371	3.90E-36	3.92E-34	LRRK2
ENSG00000123243	2722.419181	3.444975551	9.594107205	3.91E-36	3.92E-34	ITIH5
ENSG00000187479	613.912779	29.03285833	4.401423571	4.58E-36	4.57E-34	C11orf96
novel.828	360.1023044	28.72822587	3.648392744	5.95E-36	5.92E-34	-
ENSG00000187699	3115.627247	21.54695172	7.166915988	6.83E-36	6.77E-34	C2orf88
ENSG00000165804	2120.735121	247.1076815	3.102486541	7.03E-36	6.94E-34	ZNF219
ENSG00000153044	54.84415768	577.25462	-3.396478471	8.26E-36	8.13E-34	CENPH
ENSG00000108106	624.9699002	4928.528207	-2.979410941	8.50E-36	8.34E-34	UBE2S
ENSG00000146670	34.1343793	1138.234849	-5.061458875	1.05E-35	1.03E-33	CDCA5
ENSG00000123338	1265.858172	1.467450118	9.781642334	1.10E-35	1.07E-33	NCKAP1L
ENSG00000132507	2824.392379	15141.3466	-2.422459143	1.53E-35	1.48E-33	EIF5A
ENSG00000128872	11123.10925	335.4736919	5.051706274	1.53E-35	1.48E-33	TMOD2
ENSG00000134690	28.73351394	957.9914801	-5.059154525	1.69E-35	1.62E-33	CDCA8
ENSG00000170915	1860.532011	160.4485716	3.537320063	2.33E-35	2.23E-33	PAQR8
ENSG00000139044	1345.459628	1.426156051	9.87277146	2.53E-35	2.41E-33	B4GALNT3
ENSG00000153208	363.1529119	5.411760104	6.074156958	2.53E-35	2.41E-33	MERTK
ENSG00000172005	7227.18198	0	15.15296541	2.62E-35	2.48E-33	MAL
ENSG00000140534	21.6878557	382.114937	-4.136168025	3.50E-35	3.31E-33	TICRR
ENSG00000111640	9238.239738	63818.13877	-2.788273912	3.90E-35	3.67E-33	GAPDH
ENSG00000148219	1357.637741	101.6634496	3.73715926	4.41E-35	4.14E-33	ASTN2
ENSG00000106772	757.1986256	46.03325161	4.037604193	5.81E-35	5.43E-33	PRUNE2
ENSG00000148773	55.9414036	3805.863089	-6.087341704	5.93E-35	5.52E-33	MKI67
ENSG00000100055	1434.26942	14.8869605	6.60585418	6.27E-35	5.81E-33	CYTH4
ENSG00000214357	965.9988572	79.90758744	3.595210563	8.61E-35	7.96E-33	NEURL1B
ENSG00000162946	758.4183463	63.31551133	3.585283039	9.02E-35	8.31E-33	DISC1
ENSG00000126882	472.2006092	11.79048002	5.320116311	1.01E-34	9.25E-33	FAM78A
ENSG00000090661	1308.422645	200.1812435	2.709190497	1.12E-34	1.02E-32	CERS4
ENSG00000183018	443.7489267	7.65493568	5.830420438	1.38E-34	1.26E-32	AC118754.1
ENSG00000145242	346.0350211	11.15999012	4.957481059	1.43E-34	1.30E-32	EPHA5
ENSG00000100167	550.5473874	5.594693718	6.656261007	1.57E-34	1.42E-32	SEPT3
ENSG00000072274	415.8127505	4085.45097	-3.296392704	1.58E-34	1.42E-32	TFRC
ENSG00000015285	514.20045	9.008987688	5.837997267	1.74E-34	1.56E-32	WAS
ENSG00000239697	1409.769553	177.597073	2.98802291	2.25E-34	2.01E-32	TNFSF12
ENSG00000266714	3489.127205	55.40019098	5.974166673	2.37E-34	2.12E-32	MYO15B
ENSG00000100505	424.3699585	10.28404758	5.357551493	2.40E-34	2.13E-32	TRIM9
ENSG00000163739	21.99767872	12699.58024	-9.174453438	2.88E-34	2.56E-32	CXCL1
ENSG00000104812	471.3037785	3227.574884	-2.775656654	3.67E-34	3.24E-32	GYS1
ENSG00000011426	43.68628987	3435.439052	-6.295492269	4.00E-34	3.53E-32	ANLN
ENSG00000186517	871.4462888	6.186201292	7.148896246	4.11E-34	3.61E-32	ARHGAP30
ENSG00000168078	11.06284707	1112.137674	-6.645867421	4.57E-34	4.00E-32	PBK
ENSG00000187957	2.431602522	3259.953608	-10.39165219	4.73E-34	4.13E-32	DNER
ENSG00000235092	446.0327691	6.685664122	6.042035968	4.94E-34	4.29E-32	ID2-AS1
ENSG00000164007	1605.889631	1.762625967	9.810578565	4.96E-34	4.29E-32	CLDN19
ENSG00000162063	44.03930676	598.9106032	-3.767049308	5.64E-34	4.87E-32	CCNF
ENSG00000197329	1022.862948	116.8646276	3.127840063	5.99E-34	5.16E-32	PELI1
ENSG00000170571	802.5133956	3.465044647	7.820777851	7.53E-34	6.46E-32	EMB
ENSG00000094804	31.55127021	946.6564591	-4.904912017	7.77E-34	6.64E-32	CDC6
ENSG00000005108	1212.849159	5.565296406	7.74550406	7.99E-34	6.81E-32	THSD7A

ENSG00000124785	2817.336533	75.28820144	5.223557934	8.04E-34	6.83E-32	NRN1
ENSG00000168961	1199.99019	28.64106141	5.379576405	8.97E-34	7.59E-32	LGALS9
ENSG00000064787	5033.380514	0	14.63105	1.31E-33	1.11E-31	BCAS1
ENSG00000134285	185.7801965	1803.919424	-3.27993129	1.37E-33	1.15E-31	FKBP11
ENSG00000123096	1620.874409	141.7349844	3.513339233	1.65E-33	1.38E-31	SSPN
ENSG00000185811	613.0566193	3.718798035	7.400964296	2.12E-33	1.77E-31	IKZF1
ENSG00000075218	17.9938531	1026.711431	-5.831016291	2.25E-33	1.87E-31	GTSE1
ENSG00000152583	7518.043118	3.52640781	11.04773391	2.27E-33	1.88E-31	SPARCL1
ENSG00000165891	11.53997588	356.9121258	-4.952603307	3.37E-33	2.79E-31	E2F7
ENSG00000163507	36.65248394	574.6877229	-3.971392846	3.48E-33	2.87E-31	CIP2A
ENSG00000143476	17.71572731	497.6492589	-4.80787285	4.02E-33	3.31E-31	DTL
ENSG00000112531	12134.28317	2210.757213	2.456411231	6.24E-33	5.11E-31	QKI
ENSG00000118640	653.7300099	9.061022635	6.152665339	8.11E-33	6.62E-31	VAMP8
ENSG00000118985	348.1072237	4451.545921	-3.676625591	8.83E-33	7.19E-31	ELL2
ENSG00000165863	791.0003199	1.793307549	8.783961294	1.02E-32	8.29E-31	C10orf82
ENSG00000091651	62.14781206	682.6203946	-3.458012745	1.03E-32	8.33E-31	ORC6
ENSG00000183287	1.535511275	782.0824851	-9.009561631	1.10E-32	8.85E-31	CCBE1
ENSG00000170345	32204.14636	133.7649322	7.910882558	1.10E-32	8.88E-31	FOS
ENSG00000116701	398.474642	11.78102341	5.075809104	1.18E-32	9.50E-31	NCF2
ENSG00000171320	8.730781693	308.1872089	-5.137284531	1.22E-32	9.75E-31	ESCO2
ENSG00000071073	1163.49807	1.782695063	9.341793438	1.49E-32	1.18E-30	MGAT4A
ENSG00000167972	1392.71205	28.22315924	5.615756037	1.63E-32	1.29E-30	ABCA3
ENSG00000111665	29.30230201	835.1601269	-4.832574288	1.89E-32	1.50E-30	CDCA3
ENSG00000165304	50.59995828	1075.458681	-4.408646372	1.96E-32	1.55E-30	MELK
ENSG00000133985	820.8040791	1.742556871	8.842938736	2.14E-32	1.69E-30	TTC9
ENSG00000220785	399.3897677	30.44942937	3.719318386	2.16E-32	1.69E-30	MTMR9LP
ENSG00000013810	206.1022115	2972.282493	-3.850334025	2.30E-32	1.80E-30	TACC3
ENSG00000187764	640.0677006	51.85589743	3.621616719	2.53E-32	1.98E-30	SEMA4D
ENSG00000113368	110.6624179	1873.724672	-4.08201675	2.89E-32	2.25E-30	LMNB1
ENSG00000188130	49.75848594	1512.565831	-4.927553301	3.12E-32	2.42E-30	MAPK12
ENSG00000054356	8.553446699	766.4151763	-6.490297054	3.55E-32	2.75E-30	PTPRN
ENSG00000173372	5058.324158	0	14.63817124	4.74E-32	3.65E-30	C1QA
ENSG00000169129	4384.111665	0	14.43182065	4.74E-32	3.65E-30	AFAP1L2
ENSG00000245848	735.5988538	6.613688473	6.774373031	5.70E-32	4.37E-30	CEBPA
ENSG00000054690	450.6212407	11.86245566	5.24882578	5.85E-32	4.47E-30	PLEKHH1
ENSG00000146013	5663.337782	0	14.80118702	7.69E-32	5.86E-30	GFRA3
ENSG00000040275	165.3701844	1479.394123	-3.160849265	8.53E-32	6.48E-30	SPDL1
ENSG00000114346	94.09096263	1285.086837	-3.772264876	1.04E-31	7.85E-30	ECT2
ENSG00000196814	1074.311915	150.3680875	2.836800521	1.15E-31	8.72E-30	MVB12B
ENSG00000118507	712.295125	83.51542751	3.091893989	1.17E-31	8.78E-30	AKAP7
ENSG00000168268	571.6353259	4641.250642	-3.021320667	1.25E-31	9.37E-30	NT5DC2
ENSG00000131401	1710.483766	1.487519214	10.2085411	1.27E-31	9.53E-30	NAPSB
ENSG00000179915	6731.325919	0	15.05042015	1.38E-31	1.03E-29	NRXN1
ENSG00000029153	34.69181326	500.2107151	-3.851741028	1.38E-31	1.03E-29	ARNTL2
ENSG00000136546	17595.2769	0.336469917	15.4748403	1.50E-31	1.12E-29	SCN7A
ENSG00000130203	22942.50806	1042.402552	4.46030943	1.69E-31	1.25E-29	APOE
ENSG00000011422	372.3189103	4021.149871	-3.432994671	1.77E-31	1.31E-29	PLAUR
ENSG00000143195	408.5456657	2.812173909	7.157614873	1.93E-31	1.42E-29	ILDR2
ENSG00000198075	474.1717809	12.14933078	5.283854683	2.24E-31	1.64E-29	SULT1C4
ENSG00000173369	3675.342436	0	14.17739472	2.24E-31	1.64E-29	C1QB
ENSG00000138180	19.15922333	1277.673783	-6.055986345	2.44E-31	1.78E-29	CEP55

ENSG00000213390	1968.228515	368.8336457	2.415196614	3.06E-31	2.23E-29	ARHGAP19
ENSG00000169403	580.7845406	3.964379082	7.198774275	3.16E-31	2.29E-29	PTAFR
ENSG00000167995	1128.66889	81.37850518	3.793718692	3.26E-31	2.36E-29	BEST1
ENSG00000013573	91.29735386	891.9355696	-3.287296911	3.30E-31	2.38E-29	DDX11
ENSG00000136026	1076.320847	12520.98963	-3.540270304	3.44E-31	2.48E-29	CKAP4
ENSG00000159189	3948.615864	0	14.28087156	3.81E-31	2.73E-29	C1QC
ENSG00000150753	1144.553096	6993.124933	-2.611240209	4.19E-31	3.00E-29	CCT5
ENSG00000126016	413.5561182	19.3614149	4.427726641	4.98E-31	3.56E-29	AMOT
ENSG00000248441	704.158819	5.820334064	6.88250721	5.24E-31	3.73E-29	LINC01197
ENSG00000186684	298.6170472	10.50596351	4.836787667	5.38E-31	3.82E-29	CYP27C1
ENSG00000065328	15.1370199	443.4383417	-4.873132092	7.05E-31	4.99E-29	MCM10
ENSG00000163873	6153.14547	0	14.92085542	8.32E-31	5.87E-29	GRIK3
ENSG00000204136	974.9403327	2.120320855	8.828550342	8.40E-31	5.92E-29	GGTA1P
ENSG00000122877	2702.213816	11.70776349	7.857167753	9.56E-31	6.72E-29	EGR2
ENSG00000051180	13.2377071	445.8842632	-5.070863229	9.85E-31	6.89E-29	RAD51
ENSG00000188747	388.4330546	9.121101528	5.425684661	1.13E-30	7.88E-29	NOXA1
ENSG00000104738	346.4750814	3069.649301	-3.147232775	1.17E-30	8.12E-29	MCM4
ENSG00000138356	94.05217009	3208.595479	-5.093121789	1.22E-30	8.45E-29	AOX1
ENSG00000081189	1270.852924	108.8201596	3.544529199	1.22E-30	8.48E-29	MEF2C
ENSG00000260230	550.6736941	8.834226414	5.979558917	1.25E-30	8.62E-29	FRRS1L
ENSG00000156510	412.7898937	10.1494245	5.353177484	1.27E-30	8.78E-29	HKDC1
ENSG00000175592	97.65609561	5195.54101	-5.733274748	1.28E-30	8.82E-29	FOSL1
ENSG00000001617	127.1871322	1311.85506	-3.36617495	1.38E-30	9.47E-29	SEMA3F
ENSG00000115414	14651.28771	591146.1238	-5.334419805	1.71E-30	1.17E-28	FN1
ENSG00000154330	468.9013228	2.079026788	7.77303661	1.83E-30	1.25E-28	PGM5
ENSG00000143119	1533.722198	1.161661783	10.46586748	1.84E-30	1.25E-28	CD53
ENSG00000196502	616.3962522	11.83421423	5.684799709	2.06E-30	1.40E-28	SULT1A1
ENSG00000168496	205.5880148	1478.54703	-2.846101841	2.22E-30	1.50E-28	FEN1
ENSG00000141576	493.6744782	10.24146924	5.604172933	3.22E-30	2.18E-28	RNF157
ENSG00000118322	786.041825	3.718798035	7.755001891	3.44E-30	2.31E-28	ATP10B
ENSG00000177133	1316.142459	1.365948763	9.852649167	3.59E-30	2.41E-28	PRDM16-DT
ENSG00000135678	249.0325195	2672.915974	-3.424402249	4.03E-30	2.70E-28	CPM
ENSG00000100077	526.6542553	74.03521843	2.833118657	4.22E-30	2.82E-28	GRK3
ENSG00000204323	672.950268	14.08440627	5.568186881	4.24E-30	2.82E-28	SMIM5
ENSG00000100162	16.84603396	484.1135379	-4.842092594	4.49E-30	2.98E-28	CENPM
ENSG00000184261	2910.182821	5.420060839	9.071473676	5.15E-30	3.42E-28	KCNK12
ENSG00000145569	602.4776393	22.81944308	4.71955501	5.18E-30	3.42E-28	OTULINL
ENSG00000142611	919.4578207	4.668000497	7.622630911	5.20E-30	3.43E-28	PRDM16
ENSG00000149554	57.92868564	692.9561729	-3.579791645	5.90E-30	3.88E-28	CHEK1
ENSG00000100196	309.2713883	1969.751604	-2.671295584	6.73E-30	4.41E-28	KDELR3
ENSG00000051341	13.21757745	392.9654141	-4.88962864	7.74E-30	5.06E-28	POLQ
ENSG00000145476	1178.552812	126.9703179	3.213851477	7.86E-30	5.13E-28	CYP4V2
ENSG00000011600	3163.815252	0	13.96117041	1.03E-29	6.70E-28	TYROBP
ENSG00000116117	1381.073408	145.4367015	3.248289986	1.24E-29	8.08E-28	PARD3B
ENSG00000106066	956.9105796	3.108505634	8.231440545	1.37E-29	8.85E-28	CPVL
ENSG00000099204	2665.203818	95.9556146	4.793663275	1.40E-29	9.04E-28	ABLIM1
ENSG00000175600	26.37623305	271.8650254	-3.366376667	1.47E-29	9.49E-28	SUGCT
ENSG00000104490	768.8954542	35.64184208	4.425396161	2.01E-29	1.30E-27	NCALD
ENSG00000100234	25628.34261	453.3050135	5.82057724	2.71E-29	1.74E-27	TIMP3
ENSG00000105357	924.38849	5.685582588	7.382397251	3.11E-29	1.99E-27	MYH14
ENSG00000149564	315.4329505	14.84810658	4.415368208	3.69E-29	2.36E-27	ESAM

ENSG00000151883	1017.358243	198.4025297	2.357424016	3.81E-29	2.43E-27	PARP8
ENSG00000158528	1603.955899	5.474407537	8.16678191	4.40E-29	2.79E-27	PPP1R9A
ENSG00000214160	293.0515975	1332.89468	-2.185232916	4.68E-29	2.96E-27	ALG3
ENSG00000170954	284.7649224	23.47000207	3.594473494	5.29E-29	3.35E-27	ZNF415
ENSG00000169860	424.9426052	3.943154111	6.750066805	5.47E-29	3.45E-27	P2RY1
ENSG00000121152	29.38769302	778.5623143	-4.724772386	6.13E-29	3.85E-27	NCAPH
ENSG00000131473	1771.442127	6391.338113	-1.851232717	6.48E-29	4.07E-27	ACLY
ENSG00000237515	689.9978801	9.192049696	6.22080748	6.81E-29	4.26E-27	SHISA9
ENSG00000100526	29.95683709	490.1561731	-4.032648867	6.88E-29	4.29E-27	CDKN3
ENSG00000135476	28.79995767	668.4363189	-4.533767549	6.92E-29	4.31E-27	ESPL1
ENSG00000188488	1439.101304	1.069617038	10.38500673	7.27E-29	4.52E-27	SERPINA5
ENSG00000078596	660.9632183	1.365948763	8.853252299	7.54E-29	4.67E-27	ITM2A
ENSG00000165168	2767.009595	0	13.76783079	7.76E-29	4.80E-27	CYBB
ENSG00000203747	2516.715092	0	13.63106411	9.57E-29	5.91E-27	FCGR3A
ENSG00000187741	58.16139499	718.7900566	-3.626235477	1.24E-28	7.66E-27	FANCA
ENSG00000117266	979.3411517	11.12815267	6.459496114	1.64E-28	1.01E-26	CDK18
ENSG00000069493	728.4235308	60.48570846	3.591297132	1.78E-28	1.09E-26	CLEC2D
novel.601	0	2111.068866	-13.62502343	2.04E-28	1.24E-26	-
ENSG00000146859	810.3500978	138.5857798	2.548679631	2.06E-28	1.26E-26	TMEM140
ENSG00000174371	6.860445348	405.5324846	-5.875183287	2.26E-28	1.37E-26	EXO1
ENSG00000118193	13.91118945	585.1635527	-5.39409505	2.38E-28	1.44E-26	KIF14
ENSG00000148841	430.1279723	4035.89395	-3.230043888	2.80E-28	1.69E-26	ITPRIP
novel.984	5206.291349	0	14.67977424	2.94E-28	1.78E-26	-
ENSG00000132854	2393.434687	0.672939833	11.70685118	3.13E-28	1.89E-26	KANK4
ENSG00000117308	182.3683657	1369.682786	-2.909192344	3.36E-28	2.02E-26	GALE
ENSG00000106263	1835.35108	6751.894858	-1.879226508	3.85E-28	2.31E-26	EIF3B
ENSG00000100321	1323.275328	249.2259945	2.408991158	4.05E-28	2.43E-26	SYNGR1
ENSG00000075884	4572.029642	0.336469917	13.5305761	4.30E-28	2.57E-26	ARHGAP15
ENSG00000206579	2445.902724	0	13.58991327	4.74E-28	2.82E-26	XKR4
ENSG00000179409	212.0816097	959.9837366	-2.178297088	5.60E-28	3.33E-26	GEMIN4
ENSG00000123473	41.33437958	460.840922	-3.477718137	6.61E-28	3.92E-26	STIL
ENSG00000024422	2051.58594	9104.40724	-2.149844126	6.88E-28	4.07E-26	EHD2
ENSG00000164087	37.37854862	429.8014449	-3.522036977	7.12E-28	4.20E-26	POC1A
ENSG00000173269	345.0743367	8.836666559	5.275433501	7.17E-28	4.22E-26	MMRN2
ENSG00000144619	479.800269	3.881790948	6.933029716	9.60E-28	5.64E-26	CNTN4
ENSG00000126785	4924.245765	11.28412911	8.756229426	9.94E-28	5.83E-26	RHOJ
ENSG00000110799	1485.086143	6.15680398	7.887856949	1.02E-27	5.98E-26	VWF
ENSG00000167900	48.11506142	2438.536388	-5.661706345	1.02E-27	5.98E-26	TK1
ENSG00000157470	458.6030048	12.96403855	5.13007447	1.06E-27	6.19E-26	FAM81A
ENSG00000161800	122.2581245	1670.708686	-3.772856012	1.07E-27	6.20E-26	RACGAP1
ENSG00000106089	58.46111789	830.7767117	-3.82790772	1.11E-27	6.41E-26	STX1A
ENSG00000172264	584.0295665	4.515748464	6.988195713	1.12E-27	6.46E-26	MACROD2
ENSG00000198431	1153.898793	7253.019657	-2.652124778	1.35E-27	7.78E-26	TXNRD1
ENSG00000166689	1032.624361	12.4953857	6.354110974	1.44E-27	8.27E-26	PLEKHA7
ENSG00000158402	2.081662396	273.6278269	-7.016970943	1.58E-27	9.09E-26	CDC25C
ENSG00000151503	311.1652596	1252.162496	-2.008719714	1.80E-27	1.03E-25	NCAPD3
ENSG00000160298	79.7488159	476.4843907	-2.578062182	1.81E-27	1.04E-25	C21orf58
ENSG00000197757	4.616563014	443.7682619	-6.599866278	2.00E-27	1.14E-25	HOXC6
ENSG00000111348	2994.359226	119.8424914	4.641263238	2.30E-27	1.31E-25	ARHGDIB
ENSG00000279118	496.9031758	24.03198393	4.362849017	2.35E-27	1.33E-25	AC093535.2
ENSG00000025772	336.6370531	1772.94411	-2.397157354	2.54E-27	1.44E-25	TOMM34

ENSG00000163412	791.6616089	62.19655627	3.668986487	2.85E-27	1.61E-25	EIF4E3
ENSG00000096063	173.2456324	987.7393729	-2.511174137	3.23E-27	1.82E-25	SRPK1
ENSG00000167553	741.4270892	7638.034943	-3.364790678	3.26E-27	1.84E-25	TUBA1C
ENSG00000140968	1217.773425	1.069617038	10.14405789	3.45E-27	1.94E-25	IRF8
ENSG00000125844	2812.678627	11153.20149	-1.987438433	3.47E-27	1.95E-25	RRBP1
ENSG00000141540	1346.518844	96.89248795	3.797388716	3.51E-27	1.97E-25	TTYH2
ENSG00000198948	792.2821224	49.12844783	4.015431396	3.53E-27	1.97E-25	MFAP3L
ENSG00000169554	9745.066093	744.1057263	3.711216283	3.65E-27	2.04E-25	ZEB2
ENSG00000173457	851.0674928	4052.07912	-2.251418576	4.04E-27	2.25E-25	PPP1R14B
ENSG00000123219	39.79378244	632.1166695	-3.988710796	4.07E-27	2.26E-25	CENPK
ENSG00000170312	51.75286329	2072.299544	-5.322661152	4.07E-27	2.26E-25	CDK1
ENSG00000122122	560.0967299	2.058957692	8.037151458	4.82E-27	2.67E-25	SASH3
ENSG00000166091	2031.979101	0	13.32244634	4.97E-27	2.74E-25	CMTM5
ENSG00000143412	497.5720712	29.64674675	4.06708359	5.22E-27	2.88E-25	ANXA9
ENSG00000144843	418.9488915	42.52935304	3.294423267	5.43E-27	2.98E-25	ADPRH
ENSG00000120539	132.0467189	676.6862641	-2.357070994	6.05E-27	3.32E-25	MASTL
ENSG00000137877	787.5116602	45.1360841	4.122037503	6.35E-27	3.46E-25	SPTBN5
ENSG00000187140	2380.082967	0	13.55056614	6.35E-27	3.46E-25	FOXD3
ENSG00000111247	54.47784885	381.4294581	-2.808035355	6.53E-27	3.56E-25	RAD51AP1
ENSG00000186185	12.15049072	961.6388032	-6.307748047	7.12E-27	3.87E-25	KIF18B
ENSG00000149948	139.2191823	10552.88731	-6.243621827	7.59E-27	4.12E-25	HMGA2
ENSG00000103089	912.3641875	1.060160428	9.728856116	7.64E-27	4.14E-25	FA2H
ENSG00000065308	1212.835331	7134.820328	-2.556565821	8.15E-27	4.40E-25	TRAM2
ENSG00000090104	1653.483736	0	13.0250571	8.30E-27	4.47E-25	RGS1
ENSG00000105516	401.7226157	27.98433787	3.84993049	9.33E-27	5.02E-25	DBP
ENSG00000113643	752.0478659	2889.286055	-1.941750147	9.68E-27	5.19E-25	RARS
ENSG00000149599	805.6000314	1.396630344	9.138939925	9.71E-27	5.20E-25	DUSP15
ENSG00000169302	702.47565	17.06877291	5.368178849	1.20E-26	6.41E-25	STK32A
ENSG00000154930	1269.769518	108.1448268	3.552098344	1.45E-26	7.73E-25	ACSS1
ENSG00000235505	1208.584747	1.742556871	9.40766019	1.53E-26	8.14E-25	CASP17P
ENSG00000103710	503.3780661	2.527610546	7.644528623	1.73E-26	9.18E-25	RASL12
ENSG00000132122	1051.932673	108.5222868	3.277990192	1.88E-26	9.96E-25	SPATA6
ENSG00000100784	593.0393306	20.12408772	4.889449003	1.91E-26	1.01E-24	RPS6KA5
ENSG00000013441	5873.125571	834.0617164	2.815968376	2.06E-26	1.09E-24	CLK1
ENSG00000171877	636.4665292	21.22682649	4.90719526	2.09E-26	1.10E-24	FRMD5
ENSG00000067225	6441.713867	43512.24109	-2.755906828	2.14E-26	1.13E-24	PKM
ENSG00000095970	1538.347755	0	12.92090848	2.16E-26	1.13E-24	TREM2
ENSG00000187123	320.0404963	34.75973498	3.207734489	2.30E-26	1.20E-24	LYPD6
ENSG00000182481	478.8141007	5642.791767	-3.558940048	2.33E-26	1.22E-24	KPNA2
ENSG00000129538	2175.678385	0	13.42102293	2.39E-26	1.24E-24	RNASE1
ENSG00000066336	1555.074104	0	12.93647518	2.58E-26	1.35E-24	SPI1
ENSG00000100399	1457.183194	5.279577167	8.097653918	2.88E-26	1.49E-24	CHADL
ENSG00000148218	3507.629236	645.3293116	2.442132509	3.11E-26	1.61E-24	ALAD
ENSG00000110077	1935.932466	0	13.25252417	3.16E-26	1.63E-24	MS4A6A
ENSG00000123329	482.7499428	1.753169357	8.077012112	3.30E-26	1.70E-24	ARHGAP9
ENSG00000130755	526.0064425	11.04543614	5.587916008	4.04E-26	2.08E-24	GMFG
ENSG00000108018	4227.003082	0.356539013	13.41737866	4.32E-26	2.22E-24	SORCS1
ENSG00000126878	1599.906086	1.345879667	10.14698061	4.46E-26	2.29E-24	AIF1L
ENSG00000198794	425.9544761	43.5242975	3.295616124	4.67E-26	2.39E-24	SCAMP5
ENSG00000242808	1089.863042	1.049547942	9.987636577	4.69E-26	2.40E-24	SOX2-OT
ENSG00000076864	758.6498892	25.00801517	4.931723493	4.79E-26	2.45E-24	RAP1GAP

ENSG00000168329	3535.490281	0.356539013	13.15962052	5.14E-26	2.62E-24	CX3CR1
ENSG00000109610	2344.827851	59.71756023	5.292276624	5.30E-26	2.70E-24	SOD3
ENSG00000147799	2284.194722	124.4035966	4.198525919	5.68E-26	2.88E-24	ARHGAP39
ENSG00000107099	1131.772687	4.057708096	8.083111983	5.75E-26	2.91E-24	DOCK8
ENSG00000131153	29.96443026	593.4893993	-4.308241337	5.93E-26	3.00E-24	GINS2
ENSG00000128228	277.94599	1315.144504	-2.242293338	6.07E-26	3.06E-24	SDF2L1
ENSG00000004700	342.3857504	1336.200315	-1.964525051	6.55E-26	3.30E-24	RECQL
ENSG00000105227	13575.54922	112.4334751	6.916108195	6.70E-26	3.37E-24	PRX
ENSG00000112984	24.22372523	1865.811	-6.264922985	6.71E-26	3.37E-24	KIF20A
ENSG00000183578	69.38327051	459.4700117	-2.727751092	7.06E-26	3.54E-24	TNFAIP8L3
ENSG00000106538	7705.524759	59.37664239	7.01672709	7.30E-26	3.65E-24	RARRES2
ENSG00000173320	638.06793	3.52640781	7.48456905	7.58E-26	3.78E-24	STOX2
ENSG00000177875	1582.141763	60.8125462	4.704801444	8.33E-26	4.15E-24	CCDC184
ENSG00000117724	105.8612553	2461.957042	-4.53874324	8.40E-26	4.17E-24	CENPF
ENSG00000198771	841.9045116	37.66279776	4.484864657	9.83E-26	4.88E-24	RCSD1
ENSG00000186462	380.9791751	5.14739423	6.17167443	1.05E-25	5.21E-24	NAP1L2
ENSG00000087250	691.2182294	4.849778236	7.182846327	1.10E-25	5.45E-24	MT3
ENSG00000139946	550.8647873	55.56035856	3.311269322	1.13E-25	5.57E-24	PELI2
ENSG00000105695	1410.458979	1.386017859	9.953221042	1.23E-25	6.04E-24	MAG
ENSG00000125148	3466.512865	47783.36142	-3.784924062	1.26E-25	6.19E-24	MT2A
ENSG00000164466	510.1588174	2354.516844	-2.206270639	1.36E-25	6.65E-24	SFXN1
ENSG00000197461	1340.274636	159.5854249	3.072101513	1.36E-25	6.65E-24	PDGFA
ENSG00000158457	559.107085	13.36174324	5.390332989	1.57E-25	7.67E-24	TSPAN33
ENSG00000121207	1331.868967	1.161661783	10.25403094	1.70E-25	8.29E-24	LRAT
ENSG00000042088	132.1261976	712.5523683	-2.430545359	1.92E-25	9.34E-24	TDP1
ENSG00000144485	407.411538	7.633710709	5.713861695	1.93E-25	9.37E-24	HES6
ENSG00000120057	2458.568498	0.356539013	12.63553367	1.96E-25	9.52E-24	SFRP5
ENSG00000088320	786.9235003	1.386017859	9.107597067	1.99E-25	9.63E-24	REM1
ENSG00000244682	793.8425799	1.753169357	8.798012333	2.36E-25	1.14E-23	FCGR2C
ENSG00000198133	309.8212576	4.179278547	6.183125889	2.78E-25	1.34E-23	TMEM229B
ENSG00000130508	935.8482265	13952.2101	-3.898159356	2.85E-25	1.37E-23	PXDN
ENSG00000171793	232.7615849	1906.042793	-3.033913246	2.85E-25	1.37E-23	CTPS1
ENSG00000106278	2377.973429	0.356539013	12.58746794	3.03E-25	1.45E-23	PTPRZ1
ENSG00000156298	369.3565134	2.241891306	7.411101492	3.10E-25	1.48E-23	TSPAN7
ENSG00000161381	478.8820373	4.963176346	6.582448421	3.41E-25	1.63E-23	PLXDC1
ENSG00000175161	2236.907118	0	13.46105936	3.59E-25	1.71E-23	CADM2
ENSG00000137460	689.3778646	16.7522437	5.378112612	3.65E-25	1.74E-23	FHDC1
ENSG00000084676	1392.882702	261.5450776	2.411849649	4.08E-25	1.94E-23	NCOA1
ENSG00000205269	450.9520323	20.779527	4.430502081	4.15E-25	1.97E-23	TMEM170B
ENSG00000230798	1492.366558	0	12.87716707	4.17E-25	1.97E-23	FOXD3-AS1
ENSG00000185518	491.9431509	2.762579107	7.438589902	4.52E-25	2.14E-23	SV2B
ENSG00000100522	165.6031785	1047.598166	-2.661239502	5.02E-25	2.37E-23	GNPNAT1
ENSG00000108381	2830.113412	7.839153564	8.477323268	5.45E-25	2.56E-23	ASPA
ENSG00000111206	33.82162171	1196.36668	-5.142372819	5.53E-25	2.60E-23	FOXM1
ENSG00000106078	3013.785503	0.336469917	12.92932666	5.62E-25	2.64E-23	COBL
ENSG00000142583	788.8981036	21.38139027	5.209074628	6.41E-25	3.00E-23	SLC2A5
ENSG00000143479	73.45472259	753.5386058	-3.358023311	6.69E-25	3.13E-23	DYRK3
ENSG00000182752	168.4949435	5565.824417	-5.045385606	7.45E-25	3.48E-23	PAPPA
ENSG00000042980	1432.708908	20.97153204	6.105614903	8.31E-25	3.87E-23	ADAM28
ENSG00000117115	690.7348744	16.5882233	5.390809767	8.79E-25	4.09E-23	PADI2
ENSG00000114805	561.046363	1.773238453	8.29214533	9.34E-25	4.33E-23	PLCH1

ENSG00000204767	5.167800002	728.4531139	-7.14128441	9.61E-25	4.45E-23	INSYN2B
ENSG00000131773	1755.957677	129.538499	3.758805959	9.73E-25	4.50E-23	KHDRBS3
ENSG00000108830	208.8269836	9.099876557	4.532524031	9.81E-25	4.52E-23	RND2
ENSG00000165025	1261.440355	0	12.63455189	9.89E-25	4.56E-23	SYK
ENSG00000163918	121.2194444	659.1443092	-2.443498602	1.07E-24	4.91E-23	RFC4
ENSG00000082996	2097.797203	454.8897364	2.204779408	1.08E-24	4.94E-23	RNF13
ENSG00000159713	762.7169368	6.489677877	6.878856677	1.08E-24	4.96E-23	TPPP3
ENSG00000166851	53.32702421	1921.399378	-5.169890823	1.11E-24	5.10E-23	PLK1
ENSG00000112029	86.80618425	597.5017249	-2.783967979	1.14E-24	5.23E-23	FBXO5
ENSG00000117385	980.5015997	3981.189066	-2.021534136	1.17E-24	5.35E-23	P3H1
ENSG00000177301	1185.500713	0	12.54507317	1.22E-24	5.56E-23	KCNA2
ENSG00000164683	708.5901246	74.31610456	3.254668705	1.29E-24	5.88E-23	HEY1
ENSG00000150625	1127.175313	0	12.47220425	1.39E-24	6.32E-23	GPM6A
ENSG00000102384	25.57932295	358.2254914	-3.805620059	1.48E-24	6.69E-23	CENPI
ENSG00000179218	6276.518852	36774.24612	-2.550661743	1.57E-24	7.09E-23	CALR
ENSG00000115956	1026.852514	0	12.33773795	1.82E-24	8.22E-23	PLEK
ENSG00000198892	1543.441861	358.4177535	2.106860573	1.89E-24	8.49E-23	SHISA4
ENSG00000168528	17.69957207	8197.92547	-8.856403101	1.90E-24	8.56E-23	SERINC2
ENSG00000165959	1115.064933	47.24669155	4.561969749	2.00E-24	8.96E-23	CLMN
ENSG00000151322	176.0508522	5.084746798	5.126509278	2.04E-24	9.15E-23	NPAS3
ENSG00000244055	416.2807637	17.95661221	4.53554419	2.11E-24	9.46E-23	AC007566.1
ENSG00000085514	391.3322386	10.69950961	5.209747302	2.24E-24	1.00E-22	PILRA
ENSG00000116285	402.2264828	7884.534458	-4.293128592	2.35E-24	1.05E-22	ERRFI1
ENSG00000165490	10.89554126	227.8324504	-4.386574002	2.38E-24	1.06E-22	DDIAS
ENSG00000018236	2995.610375	13.75482442	7.775329403	2.43E-24	1.08E-22	CNTN1
ENSG00000095370	241.9635119	5.430673325	5.485206979	2.45E-24	1.09E-22	SH2D3C
ENSG00000136810	1315.574762	6187.613439	-2.233759577	2.47E-24	1.09E-22	TXN
ENSG00000060558	283.9599485	1.803920035	7.305194923	2.58E-24	1.14E-22	GNA15
ENSG00000153976	0.908699006	769.0072193	-9.725336917	2.99E-24	1.32E-22	HS3ST3A1
ENSG00000119535	1329.591688	0.672939833	10.85944308	3.50E-24	1.54E-22	CSF3R
ENSG00000128242	1893.653213	0.387220594	12.25892679	3.65E-24	1.60E-22	GAL3ST1
ENSG00000260423	229.2371816	2.904218654	6.315837534	3.81E-24	1.67E-22	LINC02367
ENSG00000146147	2246.26931	2.475703993	9.814229659	4.01E-24	1.76E-22	MLIP
ENSG00000185477	574.9543154	5.685582588	6.696985958	4.62E-24	2.02E-22	GPRIN3
ENSG00000109956	1565.332886	0	12.94595949	4.87E-24	2.13E-22	B3GAT1
ENSG00000179776	362.8645987	2.191140629	7.391414658	5.36E-24	2.34E-22	CDH5
ENSG00000179715	224.6911793	13.68644479	4.039818529	5.43E-24	2.36E-22	PCED1B
ENSG00000037965	1.233423615	333.9256271	-8.104189241	5.56E-24	2.42E-22	HOXC8
ENSG00000165071	289.4858947	13.62379736	4.425994919	5.57E-24	2.42E-22	TMEM71
ENSG00000197415	5.994584213	290.8107558	-5.592276893	5.68E-24	2.46E-22	VEPH1
ENSG00000169291	312.1849642	1.733100261	7.449449706	6.43E-24	2.78E-22	SHE
ENSG00000137812	16.12226311	667.4561777	-5.366862271	6.70E-24	2.89E-22	KNL1
ENSG00000171819	4058.678753	0.356539013	13.35872887	6.89E-24	2.97E-22	ANGPTL7
ENSG00000145824	2016.963652	0.774441189	11.4440781	7.33E-24	3.15E-22	CXCL14
ENSG00000082074	1124.390592	0	12.46861469	7.40E-24	3.18E-22	FYB1
ENSG00000154065	407.6476801	60.06665041	2.763804668	7.54E-24	3.23E-22	ANKRD29
ENSG00000171867	15019.72173	2508.920199	2.581760824	7.56E-24	3.24E-22	PRNP
ENSG00000111602	196.4990869	1249.377608	-2.668874883	7.93E-24	3.39E-22	TIMELESS
ENSG00000085840	12.67393362	271.3972372	-4.419237853	7.96E-24	3.40E-22	ORC1
ENSG00000253636	394.3585178	9.641789329	5.351369867	8.00E-24	3.41E-22	AC022893.1
ENSG00000127616	1233.005014	3900.213564	-1.661370466	8.18E-24	3.48E-22	SMARCA4

ENSG00000152952	548.8259903	9313.729305	-4.085059843	9.27E-24	3.94E-22	PLOD2
ENSG00000198286	470.9843529	16.73602741	4.800861608	1.02E-23	4.34E-22	CARD11
ENSG00000086062	696.8572718	5662.272436	-3.022515514	1.04E-23	4.40E-22	B4GALT1
ENSG00000239445	646.3854331	7.735212064	6.373301535	1.54E-23	6.52E-22	ST3GAL6-AS1
ENSG00000145386	93.7619076	2053.901904	-4.452922356	1.69E-23	7.13E-22	CCNA2
ENSG00000130309	2058.880108	9362.416821	-2.184983653	1.72E-23	7.26E-22	COLGALT1
ENSG00000110446	3413.589754	244.6243531	3.803750113	1.87E-23	7.86E-22	SLC15A3
ENSG00000171848	36.38019946	3407.505383	-6.547743673	1.90E-23	7.96E-22	RRM2
ENSG00000189056	7522.300117	158.9428752	5.563298252	1.94E-23	8.15E-22	RELN
ENSG00000125851	1109.671026	0	12.44961944	1.96E-23	8.21E-22	PCSK2
ENSG00000038945	1643.304738	0.336469917	12.05430582	2.14E-23	8.96E-22	MSR1
ENSG00000095585	537.2225208	1.110911105	8.959363771	2.39E-23	9.97E-22	BLNK
ENSG00000139910	214.0251562	23.74498042	3.173699433	2.40E-23	9.97E-22	NOVA1
ENSG00000100116	111.8506389	638.4716778	-2.513453924	2.50E-23	1.04E-21	GCAT
ENSG00000100364	3173.911658	878.0703823	1.853808435	2.63E-23	1.09E-21	KIAA0930
ENSG00000177303	2006.756947	339.7093375	2.561631993	3.00E-23	1.24E-21	CASKIN2
ENSG00000110880	1522.729815	5349.439108	-1.812789572	3.10E-23	1.28E-21	CORO1C
ENSG00000140451	26.2001517	387.8367706	-3.890659743	3.21E-23	1.32E-21	PIF1
ENSG00000146592	683.9741244	28.24652038	4.600679895	3.21E-23	1.32E-21	CREB5
ENSG00000145362	1531.703296	76.46903324	4.320822232	3.22E-23	1.33E-21	ANK2
ENSG00000169313	1089.791172	0	12.42361728	3.31E-23	1.36E-21	P2RY12
ENSG00000074047	49.39977027	632.4970236	-3.680251728	3.37E-23	1.38E-21	GLI2
ENSG00000172243	1136.60307	0	12.48426253	3.40E-23	1.39E-21	CLEC7A
ENSG00000110693	776.7379103	4.198191768	7.513466401	3.44E-23	1.41E-21	SOX6
ENSG00000158106	653.6086673	20.38400567	4.99529195	3.59E-23	1.47E-21	RHPN1
ENSG00000215386	1184.736954	19.02049705	5.950280379	3.73E-23	1.52E-21	MIR99AHG
ENSG00000169896	916.3379394	0	12.1734235	4.01E-23	1.63E-21	ITGAM
ENSG00000184602	2483.603484	255.4359078	3.280330428	4.07E-23	1.65E-21	SNN
ENSG00000104884	308.2372528	1611.830793	-2.386815372	4.10E-23	1.66E-21	ERCC2
ENSG00000144554	87.8201901	605.924856	-2.786748018	4.14E-23	1.68E-21	FANCD2
ENSG00000235501	304.111083	21.61533135	3.807473834	4.28E-23	1.73E-21	AC105942.1
ENSG00000188404	443.6787492	1.345879667	8.284811798	4.79E-23	1.93E-21	SELL
ENSG00000034152	578.8999753	2503.813492	-2.112586794	4.98E-23	2.01E-21	MAP2K3
ENSG00000181631	926.1892752	0	12.18891898	5.42E-23	2.18E-21	P2RY13
ENSG00000198597	1090.751143	0	12.42490186	5.60E-23	2.25E-21	ZNF536
ENSG00000154920	23.81987786	222.0034304	-3.218239598	5.79E-23	2.32E-21	EME1
ENSG00000134910	1588.139091	5545.80514	-1.804040064	6.06E-23	2.43E-21	STT3A
ENSG00000159167	160.784891	21057.28663	-7.032998004	6.43E-23	2.58E-21	STC1
ENSG00000102003	360.9300301	17.41713958	4.368315563	6.46E-23	2.58E-21	SYP
ENSG00000168615	390.8136247	5688.683134	-3.863776365	7.13E-23	2.84E-21	ADAM9
ENSG00000242173	179.2815157	4.637318915	5.266563421	7.68E-23	3.06E-21	ARHGDIG
ENSG00000155659	774.9418768	0	11.93164907	7.77E-23	3.09E-21	VSIG4
ENSG00000108829	1023.367784	5506.354342	-2.427885813	8.19E-23	3.25E-21	LRRC59
ENSG00000181449	1571.484334	0	12.95167178	8.19E-23	3.25E-21	SOX2
ENSG00000177602	2.44546393	163.0570193	-6.07059128	8.99E-23	3.56E-21	HASPIN
ENSG00000109846	24447.46982	399.6075924	5.934404026	9.63E-23	3.80E-21	CRYAB
ENSG00000128594	719.984738	12.57681796	5.82778633	9.63E-23	3.80E-21	LRRC4
ENSG00000185164	117.1274354	1098.394567	-3.229105368	9.86E-23	3.89E-21	NOMO2
ENSG00000067057	542.584166	6412.19483	-3.563001881	9.92E-23	3.91E-21	PFKP
ENSG00000114867	3921.296534	13347.68139	-1.767188547	1.05E-22	4.14E-21	EIF4G1
ENSG00000172349	879.215204	7.102282028	6.97223988	1.10E-22	4.32E-21	IL16

ENSG00000005486	1854.562344	546.5011061	1.762629868	1.15E-22	4.50E-21	RHBDD2
ENSG00000145390	5152.381998	370.597988	3.796540662	1.22E-22	4.79E-21	USP53
ENSG00000177807	1083.375843	0	12.41506728	1.32E-22	5.15E-21	KCNJ10
ENSG00000177575	781.9226137	9.038385	6.457280042	1.37E-22	5.34E-21	CD163
ENSG00000155926	555.9432497	16.32732506	5.094371472	1.51E-22	5.90E-21	SLA
ENSG00000262223	174.7018074	4.168666062	5.35410742	1.66E-22	6.46E-21	AC110285.1
ENSG00000101144	1413.688164	0.774441189	10.93299681	1.72E-22	6.69E-21	BMP7
ENSG00000184838	16.53774932	585.6892621	-5.144166511	1.82E-22	7.06E-21	PRR16
ENSG00000135424	3513.702973	96.16709362	5.189301419	1.85E-22	7.14E-21	ITGA7
ENSG00000280010	1289.303188	3.953766596	8.349821765	1.87E-22	7.22E-21	AP001350.2
ENSG00000120708	4269.982144	120233.0199	-4.815476419	1.92E-22	7.41E-21	TGFBI
ENSG00000122952	103.0903826	1252.875997	-3.602914938	1.92E-22	7.41E-21	ZWINT
ENSG00000134013	487.3620596	17983.91543	-5.205647868	1.95E-22	7.52E-21	LOXL2
ENSG00000101265	1485.879556	1.682349583	9.730422813	2.17E-22	8.34E-21	RASSF2
ENSG00000115677	5268.369521	22485.08825	-2.093542437	2.23E-22	8.55E-21	HDLBP
ENSG00000187796	481.159742	39.17886238	3.612494026	2.26E-22	8.65E-21	CARD9
ENSG00000138964	787.2074615	0	11.95434228	2.28E-22	8.72E-21	PARVG
novel.613	213.6952748	23.21612028	3.199201673	2.40E-22	9.17E-21	-
ENSG00000124374	374.854994	38.41716985	3.290624334	2.58E-22	9.83E-21	PAIP2B
ENSG00000183230	1061.023273	0	12.38492651	2.73E-22	1.04E-20	CTNNA3
ENSG00000211448	1430.398301	12.63818112	6.815562309	2.76E-22	1.05E-20	DIO2
ENSG00000131095	471.6352403	4.800183434	6.639610065	2.80E-22	1.06E-20	GFAP
ENSG00000164764	4435.311527	37.97731919	6.863398215	2.83E-22	1.07E-20	SBSPON
ENSG00000223802	579.5415192	22.91722002	4.671096548	2.91E-22	1.10E-20	CERS1
ENSG00000179750	6.261355894	335.3873578	-5.733756888	2.97E-22	1.12E-20	APOBEC3B
ENSG00000162062	19.13429254	283.9203508	-3.893261985	3.03E-22	1.14E-20	TEDC2
ENSG00000072163	2921.874762	42.08808972	6.112387469	3.23E-22	1.22E-20	LIMS2
ENSG00000043462	766.4756435	0	11.91579012	3.27E-22	1.23E-20	LCP2
ENSG00000100304	504.9070939	2158.619299	-2.096045166	3.82E-22	1.44E-20	TTLL12
ENSG00000168675	358.4760152	2.120320855	7.384954973	3.84E-22	1.44E-20	LDLRAD4
ENSG00000117593	205.0547562	882.7893428	-2.106137034	3.86E-22	1.45E-20	DARS2
ENSG00000033327	861.3540297	206.5686494	2.05936575	3.96E-22	1.48E-20	GAB2
ENSG00000157927	542.888536	35.59109141	3.925160073	4.44E-22	1.66E-20	RADIL
ENSG00000087253	2472.543391	267.0190059	3.211975042	4.51E-22	1.68E-20	LPCAT2
ENSG00000039068	2412.97522	0.356539013	12.608517	4.52E-22	1.68E-20	CDH1
ENSG00000169242	238.4708774	17.66156476	3.744650805	4.66E-22	1.73E-20	EFNA1
ENSG00000188522	38.85040819	942.8337882	-4.602992731	4.83E-22	1.79E-20	FAM83G
ENSG00000181754	378.9927748	50.48519677	2.907030111	4.85E-22	1.80E-20	AMIGO1
ENSG00000121211	9.611757884	206.2143409	-4.418038976	5.03E-22	1.87E-20	MND1
ENSG00000066294	639.3571508	0	11.65424328	5.55E-22	2.05E-20	CD84
ENSG00000166927	796.199288	0	11.9707349	5.58E-22	2.06E-20	MS4A7
ENSG00000112902	5429.503323	213.4131028	4.670219854	5.73E-22	2.12E-20	SEMA5A
ENSG00000185862	796.5584571	21.73104121	5.188699056	6.62E-22	2.44E-20	EVI2B
ENSG00000126860	472.3315158	14.33803126	5.053050959	6.62E-22	2.44E-20	EVI2A
ENSG00000162711	403.6857781	1.487519214	8.127000546	7.11E-22	2.61E-20	NLRP3
ENSG00000167363	931.3786469	91.95973639	3.342718672	7.54E-22	2.77E-20	FN3K
ENSG00000214222	158.7600708	854.2944632	-2.427540294	7.56E-22	2.77E-20	TUBBP2
ENSG00000084710	513.7131787	24.64240473	4.374336365	7.60E-22	2.78E-20	EFR3B
ENSG00000187164	1281.292549	76.56912193	4.062774903	8.01E-22	2.93E-20	SHTN1
ENSG00000133574	921.2140495	0	12.18106993	8.62E-22	3.15E-20	GIMAP4
ENSG00000107937	543.3562333	2276.182215	-2.066759708	8.73E-22	3.18E-20	GTPBP4

ENSG00000167723	593.7495262	20.59646499	4.845555302	8.85E-22	3.22E-20	TRPV3
ENSG00000188783	1826.895098	2.92428775	9.298188349	9.73E-22	3.53E-20	PRELP
ENSG00000109062	1089.031567	160.7918953	2.75806422	9.95E-22	3.61E-20	SLC9A3R1
ENSG00000259417	1068.856373	0.693008929	10.54384565	1.04E-21	3.78E-20	CTXND1
ENSG00000174672	387.9621235	5.565296406	6.101059858	1.15E-21	4.15E-20	BRSK2
ENSG00000124225	2390.749985	406.6736321	2.556010995	1.17E-21	4.21E-20	PMEPA1
ENSG00000144230	225.6733739	1.733100261	6.980580166	1.21E-21	4.38E-20	GPR17
ENSG00000167106	4191.622464	406.0003071	3.368400684	1.23E-21	4.42E-20	FAM102A
ENSG00000089154	1440.684907	5013.552891	-1.799042174	1.27E-21	4.55E-20	GCN1
ENSG00000213186	31.36411949	502.5584866	-4.004237107	1.41E-21	5.06E-20	TRIM59
ENSG00000073792	578.6450838	2525.970863	-2.126220615	1.50E-21	5.39E-20	IGF2BP2
ENSG00000197629	1140.995536	0	12.4897796	1.65E-21	5.90E-20	MPEG1
ENSG00000073849	1546.620567	153.2586928	3.336903194	1.67E-21	5.98E-20	ST6GAL1
ENSG00000230490	187.1701847	2.211209725	6.43659482	1.68E-21	6.00E-20	AL139383.1
ENSG00000205744	216.8911919	3.250145181	6.066280519	1.76E-21	6.26E-20	DENND1C
ENSG00000090382	1069.145347	0	12.39594019	1.76E-21	6.26E-20	LYZ
ENSG00000116991	3298.598651	247.0948041	3.738291415	1.91E-21	6.79E-20	SIPA1L2
ENSG00000163644	2233.832153	450.5197715	2.309994578	1.93E-21	6.85E-20	PPM1K
ENSG00000078687	786.4743623	81.72353262	3.264548317	1.94E-21	6.87E-20	TNRC6C
ENSG00000160862	881.846854	5.298361994	7.408000183	2.03E-21	7.21E-20	AZGP1
ENSG00000142731	38.77419217	429.3149403	-3.466786625	2.10E-21	7.43E-20	PLK4
ENSG00000138182	130.6439094	874.815917	-2.743850245	2.20E-21	7.78E-20	KIF20B
ENSG00000133107	11.67071206	285.5745745	-4.606375398	2.52E-21	8.88E-20	TRPC4
ENSG00000198363	1979.419532	9040.69068	-2.191348088	2.58E-21	9.08E-20	ASPH
ENSG00000166803	10.49273408	332.9816217	-4.983463946	2.59E-21	9.13E-20	PCLAF
ENSG00000136731	1096.526447	3613.060109	-1.720275681	2.80E-21	9.86E-20	UGGT1
ENSG00000145819	522.8570255	66.22602293	2.976922044	2.86E-21	1.00E-19	ARHGAP26
ENSG00000151490	249.7498799	11.83061821	4.398764273	2.88E-21	1.01E-19	PTPRO
ENSG00000171943	1685.169632	329.3612301	2.355643266	2.99E-21	1.05E-19	SRGAP2C
ENSG00000183307	193.2771986	3.912472529	5.616703192	3.31E-21	1.16E-19	TMEM121B
ENSG00000124207	866.5250921	3757.596637	-2.116614342	3.33E-21	1.16E-19	CSE1L
ENSG00000146918	219.4457678	1628.733665	-2.891389792	3.33E-21	1.16E-19	NCAPG2
ENSG00000152078	320.3922328	8.875648876	5.166712351	3.39E-21	1.18E-19	TMEM56
ENSG00000150722	545.4448176	8.032699664	6.071140742	3.42E-21	1.19E-19	PPP1R1C
ENSG00000273079	613.2281445	0	11.59410129	3.63E-21	1.26E-19	GRIN2B
ENSG00000163154	295.2376256	1.386017859	7.689622688	3.67E-21	1.27E-19	TNFAIP8L2
ENSG00000086300	241.9063423	3.485113743	6.087772941	4.00E-21	1.39E-19	SNX10
ENSG00000168216	1797.276954	495.6565791	1.85882892	4.07E-21	1.41E-19	LMBRD1
ENSG00000080986	56.08427682	946.644224	-4.078426897	4.15E-21	1.43E-19	NDC80
ENSG00000104324	1525.527371	440.8628405	1.790645401	4.26E-21	1.47E-19	CPQ
ENSG00000010278	10459.43113	874.6719916	3.579616589	4.81E-21	1.66E-19	CD9
ENSG00000137563	150.9652399	1603.647078	-3.409504203	4.89E-21	1.68E-19	GGH
ENSG00000105967	521.2139688	1.080229524	8.91844577	5.38E-21	1.85E-19	TFEC
ENSG00000180353	1774.790516	270.6239863	2.713528401	5.43E-21	1.86E-19	HCLS1
ENSG00000147257	3551.221151	0.672939833	12.29417942	5.68E-21	1.95E-19	GPC3
ENSG00000123901	1094.077138	0.336469917	11.46748225	6.06E-21	2.08E-19	GPR83
ENSG00000240583	4213.08646	6.218167144	9.392359486	6.51E-21	2.23E-19	AQP1
ENSG00000189067	4465.88227	498.7176517	3.162094041	6.63E-21	2.27E-19	LITAF
ENSG00000137692	293.3076558	1321.521905	-2.172044396	7.15E-21	2.44E-19	DCUN1D5
ENSG00000163563	810.3306751	0	11.99603835	7.45E-21	2.54E-19	MNDA
ENSG00000274956	738.7796328	0	11.86282763	8.08E-21	2.75E-19	NKAIN3-IT1

ENSG00000114013	625.4191908	0	11.62234507	8.14E-21	2.76E-19	CD86
ENSG00000139354	4475.638444	322.0292918	3.796264706	8.73E-21	2.96E-19	GAS2L3
ENSG00000183111	1824.410306	36.32308265	5.647675318	9.17E-21	3.11E-19	ARHGEF37
ENSG00000065911	340.7820001	1867.81988	-2.454790092	9.29E-21	3.14E-19	MTHFD2
ENSG00000110934	516.8112551	0	11.34716912	9.59E-21	3.24E-19	BIN2
ENSG00000079616	311.5044585	1411.932164	-2.180436725	9.65E-21	3.26E-19	KIF22
ENSG00000066279	18.69087005	1433.953474	-6.25749572	1.03E-20	3.46E-19	ASPM
ENSG00000157193	70.67317994	373.8257399	-2.402064013	1.05E-20	3.54E-19	LRP8
ENSG00000196405	1916.534144	407.505678	2.234215469	1.12E-20	3.76E-19	EVL
ENSG00000114853	2326.715399	605.4979168	1.942215763	1.14E-20	3.83E-19	ZBTB47
ENSG00000123636	844.1888987	222.2301795	1.925181359	1.28E-20	4.29E-19	BAZ2B
ENSG00000185624	4871.541973	26227.67474	-2.428647664	1.29E-20	4.32E-19	P4HB
ENSG00000095752	31.819509	1454.296159	-5.514276544	1.33E-20	4.44E-19	IL11
ENSG00000157064	41.54466544	284.4800549	-2.776734537	1.42E-20	4.74E-19	NMNAT2
ENSG00000064999	1829.734385	514.7743051	1.829395915	1.45E-20	4.85E-19	ANKS1A
ENSG00000143772	1337.248397	136.4245501	3.292397889	1.49E-20	4.96E-19	ITPKB
ENSG00000131462	391.214696	1709.016521	-2.127230456	1.58E-20	5.27E-19	TUBG1
ENSG00000156802	212.4431883	1291.735641	-2.60457365	1.76E-20	5.83E-19	ATAD2
ENSG00000148344	75.20366742	5948.218943	-6.306206239	1.89E-20	6.28E-19	PTGES
ENSG00000161929	180.3967397	3.923085015	5.51628567	1.91E-20	6.34E-19	SCIMP
ENSG00000145675	3640.943806	507.1452648	2.84344563	2.06E-20	6.81E-19	PIK3R1
ENSG00000145623	707.3139504	5599.18218	-2.984673814	2.12E-20	6.99E-19	OSMR
ENSG00000189159	322.1773474	2990.605945	-3.214739807	2.29E-20	7.55E-19	JPT1
ENSG00000072041	0.293240848	864.1158216	-11.37423918	2.40E-20	7.91E-19	SLC6A15
ENSG00000156261	1557.824065	5256.503681	-1.75460665	2.56E-20	8.42E-19	CCT8
ENSG00000127564	20.885931	850.5707765	-5.344517669	2.66E-20	8.74E-19	PKMYT1
ENSG00000121966	1374.245803	0.387220594	11.79632873	2.69E-20	8.84E-19	CXCR4
ENSG00000134504	1181.500363	231.9425318	2.349140705	2.69E-20	8.84E-19	KCTD1
ENSG00000069122	1407.529403	1.426156051	9.939803877	2.83E-20	9.26E-19	ADGRF5
ENSG00000240038	552.6199336	39.91876918	3.79463041	3.00E-20	9.82E-19	AMY2B
ENSG00000071246	1644.109311	279.4585507	2.556753978	3.28E-20	1.07E-18	VASH1
ENSG00000196664	628.8891025	0	11.6303112	3.31E-20	1.08E-18	TLR7
ENSG00000235314	200.5896288	9.448243229	4.389111775	3.32E-20	1.08E-18	LINC00957
ENSG00000196305	1118.792266	4352.066199	-1.959809235	3.53E-20	1.15E-18	IARS
ENSG00000111058	769.4844269	205.6040013	1.903605325	3.75E-20	1.22E-18	ACSS3
ENSG00000120963	2440.741344	715.1891046	1.770992301	3.85E-20	1.25E-18	ZNF706
ENSG00000146090	974.7125915	0.713078025	10.40784863	3.90E-20	1.27E-18	RASGEF1C
ENSG00000143375	166.4020543	3.210006989	5.688810826	4.03E-20	1.30E-18	CGN
ENSG00000186466	444.4554628	2.649180997	7.437473086	4.28E-20	1.39E-18	AQP7P1
ENSG00000182287	5703.165969	477.8022733	3.577731921	4.34E-20	1.40E-18	AP1S2
ENSG00000044090	542.7837555	1974.15184	-1.862625098	4.39E-20	1.42E-18	CUL7
ENSG00000272168	167.559831	6.807234573	4.619499728	4.52E-20	1.46E-18	CASC15
ENSG00000124882	1.50778846	2095.954765	-10.4381398	5.02E-20	1.62E-18	EREG
ENSG00000167912	371.6465992	2.782648203	7.033393051	5.06E-20	1.63E-18	AC090152.1
ENSG00000155792	277.048503	15.14341082	4.182563566	5.08E-20	1.63E-18	DEPTOR
ENSG00000118137	162.6583048	3.973835692	5.361796118	5.33E-20	1.71E-18	APOA1
ENSG00000136811	481.0258582	1596.979136	-1.731223035	5.37E-20	1.72E-18	ODF2
ENSG00000127586	253.7982395	994.6719283	-1.970355573	6.04E-20	1.93E-18	CHTF18
ENSG00000197959	271.3253494	12.72662985	4.421677354	6.12E-20	1.96E-18	DNM3
ENSG00000144893	391.9911924	1.029478846	8.514975594	6.30E-20	2.01E-18	MED12L
ENSG00000189319	1977.383832	366.7702401	2.430042008	7.20E-20	2.29E-18	FAM53B

ENSG00000183207	821.5442495	3744.541189	-2.188424304	7.43E-20	2.37E-18	RUVBL2
ENSG00000112799	609.0168922	0	11.58398695	7.50E-20	2.39E-18	LY86
ENSG00000153303	1037.255009	0	12.35234965	8.14E-20	2.58E-18	FRMD1
ENSG00000101605	176.0324031	17.9967504	3.291809582	8.17E-20	2.59E-18	MYOM1
ENSG00000139514	407.9997288	2705.818384	-2.729396791	8.30E-20	2.63E-18	SLC7A1
ENSG00000105997	1.509042109	195.5791482	-7.015188695	8.62E-20	2.73E-18	HOXA3
ENSG00000167895	621.0183979	0	11.61218062	9.04E-20	2.86E-18	TMC8
ENSG00000198223	727.9429641	0.672939833	9.992492837	9.07E-20	2.86E-18	CSF2RA
ENSG00000111424	13.79395868	215.8816275	-3.962357219	9.28E-20	2.93E-18	VDR
ENSG00000146192	501.9369884	0	11.30499919	9.57E-20	3.01E-18	FGD2
ENSG00000140650	64.18398675	379.7076939	-2.564629296	1.04E-19	3.28E-18	PMM2
ENSG00000089057	1769.201735	528.914248	1.74190498	1.07E-19	3.37E-18	SLC23A2
ENSG00000242125	189.2786014	1101.983613	-2.541576075	1.08E-19	3.38E-18	SNHG3
ENSG00000107719	1735.578272	18.26496909	6.561467663	1.12E-19	3.51E-18	PALD1
ENSG00000129810	10.83633469	166.9601636	-3.941993966	1.13E-19	3.53E-18	SGO1
ENSG00000008394	64.20622425	5720.935078	-6.478180383	1.18E-19	3.68E-18	MGST1
ENSG00000150275	949.3351173	0.356539013	11.26273835	1.18E-19	3.69E-18	PCDH15
ENSG00000062822	311.1000695	1273.504472	-2.033331019	1.22E-19	3.81E-18	POLD1
ENSG00000159388	6061.764344	226.4716859	4.741732135	1.22E-19	3.81E-18	BTG2
ENSG00000166825	111.2248227	19966.6042	-7.488302758	1.34E-19	4.16E-18	ANPEP
novel.383	669.6261526	1.426156051	8.867504713	1.35E-19	4.21E-18	-
ENSG00000110848	454.966499	0	11.16328687	1.38E-19	4.28E-18	CD69
ENSG00000261455	251.7339426	30.56051573	3.042815967	1.38E-19	4.28E-18	LINC01003
ENSG00000177570	248.859088	4.93365064	5.641982566	1.40E-19	4.33E-18	SAMD12
ENSG00000174059	968.4786782	5.514545729	7.436694817	1.41E-19	4.37E-18	CD34
ENSG00000100060	327.6170046	1.060160428	8.251279748	1.45E-19	4.49E-18	MFNG
ENSG00000225697	256.7034947	1646.32609	-2.6808608	1.56E-19	4.82E-18	SLC26A6
ENSG00000128815	447.7433204	0	11.14015187	1.57E-19	4.85E-18	WDFY4
ENSG00000138593	6525.330186	912.6601757	2.837835647	1.62E-19	4.98E-18	SECISBP2L
ENSG00000175928	420.6785056	0	11.05038628	1.77E-19	5.44E-18	LRRN1
ENSG00000010292	627.2128133	3128.445576	-2.318583055	1.77E-19	5.44E-18	NCAPD2
ENSG00000186994	311.847758	7.398742147	5.38520207	1.84E-19	5.66E-18	KANK3
ENSG00000143333	483.6347115	13.31112096	5.173029766	1.87E-19	5.73E-18	RGS16
ENSG00000138675	0.616711807	529.1539813	-9.770083959	1.93E-19	5.92E-18	FGF5
ENSG00000245526	681.6086816	0	11.74662613	1.96E-19	5.99E-18	LINC00461
ENSG00000167325	437.0103555	2587.483147	-2.566076959	2.27E-19	6.92E-18	RRM1
ENSG00000198846	1021.596593	6.473204801	7.279512202	2.37E-19	7.23E-18	TOX
ENSG00000157570	396.9963271	1.753169357	7.798881083	2.44E-19	7.43E-18	TSPAN18
ENSG00000158164	5.117368968	115.0263016	-4.485135394	2.48E-19	7.55E-18	TMSB15A
ENSG00000143409	1022.204626	105.0260938	3.280186203	2.49E-19	7.56E-18	MINDY1
ENSG00000115271	504.312905	76.64109758	2.714915496	2.58E-19	7.82E-18	GCA
ENSG00000125319	25.05964099	223.3451533	-3.153674785	2.63E-19	7.97E-18	C17orf53
ENSG00000163468	2220.893536	8231.629684	-1.890089099	2.65E-19	8.01E-18	CCT3
ENSG00000188501	250.3800255	16.49849031	3.935634784	2.67E-19	8.06E-18	LCTL
ENSG00000261115	299.3462599	1.049547942	8.121691891	2.74E-19	8.28E-18	TMEM178B
ENSG00000180198	208.3456947	1502.306873	-2.850572451	2.78E-19	8.40E-18	RCC1
ENSG00000205517	341.2888211	3.903015919	6.44131235	3.03E-19	9.14E-18	RGL3
ENSG00000121964	1411.668183	240.4503872	2.554599824	3.12E-19	9.40E-18	GTDC1
ENSG00000171659	763.5833094	0.387220594	10.9485185	3.16E-19	9.50E-18	GPR34
ENSG00000198517	3199.432531	756.2153044	2.08136509	3.28E-19	9.84E-18	MAFK
ENSG00000070087	1261.745507	6038.422489	-2.258736749	3.39E-19	1.02E-17	PFN2

ENSG00000235823	709.442739	72.81309257	3.287469822	3.46E-19	1.04E-17	OLMALINC
ENSG00000136492	22.48817074	310.9834237	-3.785797858	3.53E-19	1.06E-17	BRIP1
ENSG00000197046	5.500046503	982.3836265	-7.484272687	3.60E-19	1.08E-17	SIGLEC15
ENSG00000196169	695.0382596	0	11.7746153	3.67E-19	1.09E-17	KIF19
ENSG00000092758	4494.21772	93.46741874	5.585322644	3.70E-19	1.10E-17	COL9A3
ENSG00000154263	955.1593097	5.218214004	7.500526619	3.71E-19	1.11E-17	ABCA10
ENSG00000128918	279.0240738	3.230076085	6.432313293	3.88E-19	1.15E-17	ALDH1A2
ENSG00000113269	3366.716538	660.4089794	2.349553689	3.95E-19	1.17E-17	RNF130
ENSG00000088538	337.360097	11.40313103	4.899672446	4.09E-19	1.21E-17	DOCK3
ENSG00000156535	86.58747815	3092.732375	-5.159191075	4.16E-19	1.23E-17	CD109
ENSG00000121898	1031.732949	0	12.34454892	4.45E-19	1.32E-17	CPXM2
ENSG00000164691	416.578204	0	11.03605787	4.53E-19	1.34E-17	TAGAP
ENSG00000188976	885.6755124	3201.235998	-1.853778307	4.64E-19	1.37E-17	NOC2L
ENSG00000185985	610.2116001	0.693008929	9.734871659	4.99E-19	1.47E-17	SLITRK2
ENSG00000132326	259.1232445	38.41961	2.754068627	5.09E-19	1.50E-17	PER2
ENSG00000167123	989.5104759	5618.127611	-2.505382447	5.18E-19	1.52E-17	CERCAM
ENSG00000164626	538.6203052	0	11.40695556	5.21E-19	1.53E-17	KCNK5
ENSG00000044574	5969.09225	41054.43689	-2.78196234	5.25E-19	1.54E-17	HSPA5
ENSG00000133561	518.4432101	0	11.35168014	5.45E-19	1.60E-17	GIMAP6
ENSG00000057019	1328.188935	10845.20139	-3.029489403	5.71E-19	1.67E-17	DCBLD2
ENSG00000105851	451.6254702	0	11.15260136	5.74E-19	1.68E-17	PIK3CG
ENSG00000165424	6633.058322	1149.812004	2.528017337	5.79E-19	1.69E-17	ZCCHC24
ENSG00000115306	31138.56704	5951.688871	2.38728621	5.96E-19	1.74E-17	SPTBN1
ENSG00000095203	5292.248493	109.7729924	5.592722882	5.97E-19	1.74E-17	EPB41L4B
ENSG00000135636	183.7035487	8.304210397	4.471464755	6.14E-19	1.79E-17	DYSF
ENSG00000254986	254.5349025	1193.83169	-2.229766759	6.15E-19	1.79E-17	DPP3
ENSG00000137558	554.0946812	0	11.44763923	6.24E-19	1.81E-17	PI15
ENSG00000148948	645.8708489	0.713078025	9.813963528	6.36E-19	1.85E-17	LRRC4C
ENSG00000075702	44.94646738	492.4785164	-3.453401993	6.55E-19	1.90E-17	WDR62
ENSG00000221955	12.09622747	145.7047105	-3.589610669	6.59E-19	1.91E-17	SLC12A8
novel.342	370.3538376	0	10.86645927	6.76E-19	1.96E-17	-
ENSG00000145912	544.5638285	1850.659529	-1.765057916	6.94E-19	2.01E-17	NHP2
ENSG00000120915	157.1171723	7.715142968	4.328284244	7.06E-19	2.04E-17	EPHX2
ENSG00000113569	344.6847936	1125.0599	-1.706589377	7.28E-19	2.10E-17	NUP155
ENSG00000105963	642.9906813	27.91937868	4.531777317	7.32E-19	2.11E-17	ADAP1
ENSG00000163328	2936.665571	225.4293515	3.704560354	7.52E-19	2.17E-17	GPR155
ENSG00000171307	363.4164928	1197.329167	-1.71997988	8.14E-19	2.34E-17	ZDHHC16
ENSG00000235859	259.5130887	11.22849815	4.535838083	8.45E-19	2.43E-17	AC006978.1
ENSG00000244734	566.0093555	0	11.47833503	8.92E-19	2.56E-17	HBB
ENSG00000167851	389.6976182	0	10.93983026	9.98E-19	2.86E-17	CD300A
ENSG00000181163	4224.657823	17110.94877	-2.018047516	1.02E-18	2.91E-17	NPM1
ENSG00000122641	65.06037527	4740.193742	-6.187480507	1.03E-18	2.94E-17	INHBA
ENSG00000135439	175.1595183	3.647978261	5.601143626	1.11E-18	3.17E-17	AGAP2
ENSG00000113719	2411.707307	7223.910245	-1.582702268	1.17E-18	3.34E-17	ERGIC1
ENSG00000198805	273.6055441	1106.488013	-2.015999755	1.23E-18	3.51E-17	PNP
ENSG00000188643	1510.225381	5934.131067	-1.974222194	1.25E-18	3.55E-17	S100A16
ENSG00000156711	81.32752105	520.7867598	-2.678966854	1.25E-18	3.56E-17	MAPK13
ENSG00000114480	403.2418295	2759.187296	-2.774446887	1.27E-18	3.62E-17	GBE1
ENSG00000125355	697.0405109	14.54116237	5.587295496	1.33E-18	3.77E-17	TMEM255A
ENSG00000144115	1186.440682	22.12142547	5.754338015	1.38E-18	3.91E-17	THNSL2
ENSG00000068305	2145.604491	630.6723925	1.766627433	1.38E-18	3.92E-17	MEF2A

ENSG00000171115	482.0756191	0	11.24674255	1.38E-18	3.92E-17	GIMAP8
ENSG00000164318	1143.964236	154.1366559	2.891015523	1.40E-18	3.96E-17	EGFLAM
ENSG00000082438	1000.429912	96.44446495	3.371936404	1.42E-18	4.01E-17	COBLL1
ENSG00000266094	426.6886158	25.66217018	4.057986484	1.48E-18	4.17E-17	RASSF5
ENSG00000104903	536.5599931	60.03468456	3.161742805	1.51E-18	4.26E-17	LYL1
ENSG00000180777	1189.176791	0	12.54952724	1.54E-18	4.35E-17	ANKRD30B
ENSG00000127399	262.789169	11.92394722	4.448747002	1.56E-18	4.40E-17	LRRC61
ENSG00000116396	238.643882	44.13600256	2.43479973	1.64E-18	4.59E-17	KCNC4
ENSG00000164684	735.4422773	14.60393819	5.643762903	1.66E-18	4.65E-17	ZNF704
ENSG00000138363	565.1513184	2475.576187	-2.131268557	1.66E-18	4.66E-17	ATIC
ENSG00000103494	140.5424261	533.22404	-1.923695728	1.86E-18	5.22E-17	RPGRIP1L
ENSG00000130540	3.3831394	194.9672207	-5.861565955	1.89E-18	5.30E-17	SULT4A1
ENSG00000110324	902.8940182	0.387220594	11.19030386	1.91E-18	5.33E-17	IL10RA
ENSG00000117984	8773.236899	2256.186364	1.959234103	1.91E-18	5.33E-17	CTSD
ENSG00000242689	922.8993135	7.327922374	6.965104503	1.93E-18	5.38E-17	CNTF
ENSG00000112312	96.020007	732.4908167	-2.931878671	1.94E-18	5.39E-17	GMNN
ENSG00000106537	1576.044506	199.5444135	2.982867069	1.95E-18	5.43E-17	TSPAN13
ENSG00000135643	2545.370142	106.8016784	4.576511779	2.05E-18	5.70E-17	KCNMB4
ENSG00000141753	17521.89691	99528.77993	-2.505957838	2.06E-18	5.72E-17	IGFBP4
ENSG00000101336	671.779893	0.356539013	10.76372848	2.23E-18	6.18E-17	HCK
ENSG00000154153	686.7980464	16.93201366	5.330474496	2.29E-18	6.34E-17	RETREG1
ENSG00000167670	207.6255144	988.9455652	-2.2516479	2.33E-18	6.45E-17	CHAF1A
ENSG00000162267	281.8359037	2.406040094	6.830703246	2.43E-18	6.71E-17	ITIH3
ENSG00000227502	732.4580579	0.336469917	10.88863296	2.46E-18	6.78E-17	LINC01268
ENSG00000009790	282.9961873	4.902969058	5.833055453	2.53E-18	6.97E-17	TRAF3IP3
ENSG00000253729	1612.168143	5903.906775	-1.872698842	2.75E-18	7.57E-17	PRKDC
ENSG00000108424	2665.147456	7974.001564	-1.581134101	2.78E-18	7.64E-17	KPNB1
ENSG00000265415	6.404984563	140.2014193	-4.459108407	2.88E-18	7.91E-17	AC099850.3
ENSG00000185046	158.3696854	2.415496705	5.989950698	2.90E-18	7.97E-17	ANKS1B
ENSG00000074695	1530.416132	7271.759077	-2.248366638	2.95E-18	8.09E-17	LMAN1
ENSG00000129195	17.76365104	695.659281	-5.290124894	2.99E-18	8.18E-17	PIMREG
ENSG00000273771	252.5461972	3.749479616	6.110846728	3.03E-18	8.29E-17	AC024337.2
ENSG00000166510	14.04722496	803.6181039	-5.840582256	3.08E-18	8.42E-17	CCDC68
ENSG00000157456	51.10674774	1205.289882	-4.558224961	3.18E-18	8.69E-17	CCNB2
ENSG00000141968	645.3071286	0.387220594	10.70571483	3.38E-18	9.22E-17	VAV1
ENSG00000141524	1267.352514	24.14666631	5.706017305	3.51E-18	9.57E-17	TMC6
ENSG00000139438	530.5195971	24.817166	4.409911121	3.59E-18	9.77E-17	FAM222A
ENSG00000125912	998.3633479	3669.615726	-1.878089828	3.77E-18	1.02E-16	NCLN
ENSG00000175216	1201.150019	3850.851571	-1.680797415	4.09E-18	1.11E-16	CKAP5
ENSG00000142185	601.7312753	0.356539013	10.60487509	4.13E-18	1.12E-16	TRPM2
ENSG00000070159	37.10543742	310.0985725	-3.061194519	4.15E-18	1.12E-16	PTPN3
ENSG00000099901	901.0166494	3825.581311	-2.086131634	4.34E-18	1.18E-16	RANBP1
ENSG00000158292	788.5011062	123.9617726	2.670567255	4.40E-18	1.19E-16	GPR153
ENSG00000113810	304.2530579	1845.79269	-2.601110958	4.52E-18	1.22E-16	SMC4
ENSG00000123836	549.0891755	112.6026325	2.284941292	4.62E-18	1.25E-16	PFKFB2
ENSG00000150337	802.7781504	0.336469917	11.02087133	4.64E-18	1.25E-16	FCGR1A
ENSG00000108179	360.0709903	3136.0461	-3.122642578	4.71E-18	1.27E-16	PPIF
ENSG00000128595	4080.754535	19071.0617	-2.224496797	4.76E-18	1.28E-16	CALU
ENSG00000239713	601.1367735	98.83937898	2.603264433	4.78E-18	1.29E-16	APOBEC3G
ENSG00000087269	404.3632511	1254.536454	-1.63350061	4.79E-18	1.29E-16	NOP14
ENSG00000185480	33.37647485	221.6908352	-2.733151154	4.97E-18	1.33E-16	PARPBP

ENSG00000112149	1365.9313	66.41700049	4.362285224	5.03E-18	1.35E-16	CD83
ENSG00000265531	312.9703071	0	10.62377318	5.18E-18	1.39E-16	FCGR1CP
ENSG00000110079	308.9744577	0	10.60496561	5.22E-18	1.40E-16	MS4A4A
ENSG00000170498	1.481319294	298.7050521	-7.634372207	5.25E-18	1.40E-16	KISS1
ENSG00000102230	174.7626671	3.088436538	5.777372962	5.28E-18	1.41E-16	PCYT1B
ENSG00000120526	197.1909037	944.9626666	-2.26121793	5.29E-18	1.41E-16	NUDCD1
ENSG00000163673	1034.852125	0	12.34890917	5.47E-18	1.46E-16	DCLK3
ENSG00000138347	0.616711807	488.4342552	-9.653026333	5.52E-18	1.47E-16	MYPN
ENSG00000147872	281.5122189	4029.12697	-3.839344196	5.54E-18	1.47E-16	PLIN2
ENSG00000115504	6434.89431	1114.62813	2.52946814	5.76E-18	1.53E-16	EHBP1
ENSG00000155629	656.2394812	0.356539013	10.72993409	5.83E-18	1.55E-16	PIK3AP1
ENSG00000111669	4162.980873	18916.39261	-2.183946916	5.84E-18	1.55E-16	TPI1
ENSG00000120899	559.8496338	117.3036264	2.253869257	5.88E-18	1.56E-16	PTK2B
ENSG00000128965	13.27051578	244.453971	-4.201968186	5.89E-18	1.56E-16	CHAC1
ENSG00000109674	2.654282684	211.4634933	-6.293057919	5.90E-18	1.56E-16	NEIL3
ENSG00000184292	392.997003	7.306569008	5.758786825	6.12E-18	1.62E-16	TACSTD2
ENSG00000163517	1507.574748	301.4755683	2.32296253	6.16E-18	1.62E-16	HDAC11
ENSG00000253626	81.25054957	413.543784	-2.347944693	6.32E-18	1.66E-16	EIF5AL1
ENSG00000211445	3308.677339	171.2794591	4.27090422	6.34E-18	1.67E-16	GPX3
ENSG00000111186	81.58580157	1376.552586	-4.077491499	6.37E-18	1.67E-16	WNT5B
ENSG00000107551	5309.58033	189.5264828	4.809172934	6.51E-18	1.71E-16	RASSF4
ENSG00000161835	908.2803883	79.4569959	3.511703604	6.76E-18	1.77E-16	GRASP
ENSG00000114554	1081.366719	3212.674308	-1.570875812	6.87E-18	1.80E-16	PLXNA1
ENSG00000048740	3621.367098	42.14482938	6.421509259	6.95E-18	1.82E-16	CELF2
ENSG00000235568	249.7468024	1.060160428	7.859710295	7.08E-18	1.85E-16	NFAM1
ENSG00000074706	595.7649332	1.060160428	9.117660316	7.11E-18	1.86E-16	IPCEF1
ENSG00000143171	565.7004489	0.713078025	9.622793042	7.27E-18	1.90E-16	RXRG
ENSG00000140525	147.5470421	1008.172357	-2.772029727	7.94E-18	2.07E-16	FANCI
ENSG00000188229	2430.164205	15829.50636	-2.70347279	8.31E-18	2.16E-16	TUBB4B
ENSG00000180537	3.049567979	113.4632579	-5.225727702	8.33E-18	2.17E-16	RNF182
ENSG00000144847	712.7518144	0.336469917	10.84925344	8.93E-18	2.32E-16	IGSF11
ENSG00000182103	647.2479012	0.387220594	10.71017738	9.07E-18	2.36E-16	FAM181B
ENSG00000131504	655.779093	2415.293772	-1.881106714	9.64E-18	2.50E-16	DIAPH1
ENSG00000175061	1286.673969	5399.748004	-2.069324861	9.74E-18	2.52E-16	SNHG29
ENSG00000106688	62.8442161	699.1025743	-3.477020183	9.84E-18	2.55E-16	SLC1A1
ENSG00000182568	1529.924348	142.2342248	3.429096253	1.01E-17	2.61E-16	SATB1
ENSG00000256673	207.0679658	7.267715086	4.806447788	1.03E-17	2.66E-16	AC141557.1
ENSG00000137801	5699.598087	41552.89013	-2.866002058	1.04E-17	2.68E-16	THBS1
ENSG00000151690	805.4891714	117.8447684	2.770604482	1.04E-17	2.68E-16	MFSD6
ENSG00000188536	328.6024769	0	10.69404939	1.04E-17	2.68E-16	HBA2
ENSG00000130024	2549.49416	744.6806796	1.775753394	1.08E-17	2.78E-16	PHF10
ENSG00000259668	139.3628384	11.2603356	3.639242086	1.11E-17	2.86E-16	AC066613.1
ENSG00000150510	195.6858519	5.737617535	5.091715614	1.11E-17	2.86E-16	FAM124A
ENSG00000140280	658.4230628	131.235605	2.327241334	1.12E-17	2.88E-16	LYSMD2
ENSG00000157388	220.632094	1.762625967	6.947288181	1.12E-17	2.88E-16	CACNA1D
ENSG00000213853	2729.167032	700.2363673	1.962792556	1.25E-17	3.19E-16	EMP2
ENSG00000012048	131.556911	681.4686899	-2.372681107	1.26E-17	3.24E-16	BRCA1
ENSG00000110104	246.6562635	794.1742368	-1.687096058	1.28E-17	3.27E-16	CCDC86
ENSG00000166130	335.0917536	2866.309912	-3.096864584	1.39E-17	3.56E-16	IKBIP
ENSG00000010319	256.2481768	1.00940975	7.904133698	1.43E-17	3.65E-16	SEMA3G
ENSG00000175857	550.0283133	0	11.43701595	1.44E-17	3.66E-16	GAPT

ENSG00000006747	681.3466312	0.723690511	9.88932043	1.48E-17	3.76E-16	SCIN
ENSG00000180806	0.922560414	187.4892765	-7.688191086	1.54E-17	3.92E-16	HOXC9
ENSG00000104894	1032.302993	48.84871757	4.403737756	1.60E-17	4.06E-16	CD37
ENSG00000169750	80.4869467	640.8538303	-2.993538315	1.60E-17	4.07E-16	RAC3
ENSG00000077585	1226.898184	234.0674761	2.39017736	1.61E-17	4.08E-16	GPR137B
ENSG00000114757	322.1046212	5.788368213	5.80323081	1.65E-17	4.19E-16	PEX5L
ENSG00000243836	294.7314209	0	10.53697965	1.69E-17	4.27E-16	WDR86-AS1
ENSG00000197471	355.1024065	0	10.80570304	1.71E-17	4.33E-16	SPN
ENSG00000119922	3006.206502	218.4522956	3.781460289	1.71E-17	4.33E-16	IFIT2
ENSG00000009335	1166.181718	3154.236444	-1.435492013	1.73E-17	4.37E-16	UBE3C
ENSG00000265681	99.82049917	564.3313957	-2.498695432	1.79E-17	4.51E-16	RPL17
ENSG00000065413	909.920063	89.75764495	3.338650937	1.81E-17	4.55E-16	ANKRD44
ENSG00000109794	332.5327214	38.55667322	3.103058363	1.83E-17	4.61E-16	FAM149A
ENSG00000011105	1936.507332	669.7472321	1.531904976	1.95E-17	4.90E-16	TSPAN9
ENSG00000160877	828.2388843	3341.964856	-2.012523205	1.95E-17	4.91E-16	NACC1
ENSG00000164024	397.2327004	1246.834013	-1.650334158	1.99E-17	4.98E-16	METAP1
ENSG00000156395	433.4249113	0	11.09349256	1.99E-17	4.98E-16	SORCS3
ENSG00000171302	547.4692974	2098.567672	-1.938539788	2.08E-17	5.20E-16	CANT1
ENSG00000119326	7846.597713	1003.20436	2.96725369	2.12E-17	5.30E-16	CTNNAL1
ENSG00000198576	237.6862351	6.481377142	5.199216598	2.26E-17	5.66E-16	ARC
ENSG00000197930	425.418738	3569.675883	-3.068800971	2.30E-17	5.75E-16	ERO1A
ENSG00000198925	241.3701212	1004.768008	-2.057392244	2.32E-17	5.80E-16	ATG9A
ENSG00000142230	936.1357574	3606.881354	-1.946096135	2.34E-17	5.82E-16	SAE1
ENSG00000080823	203.2977475	1002.4125	-2.30172155	2.39E-17	5.95E-16	MOK
ENSG00000148935	207.0647034	6.931245169	4.875821849	2.39E-17	5.96E-16	GAS2
ENSG00000140450	2508.262525	390.5804581	2.683760025	2.41E-17	5.98E-16	ARRDC4
ENSG00000105383	291.571132	0	10.52128331	2.42E-17	6.02E-16	CD33
ENSG00000143870	1040.442779	7663.475132	-2.880856586	2.43E-17	6.02E-16	PDIA6
ENSG00000123352	205.4396841	934.2704646	-2.185565051	2.49E-17	6.18E-16	SPATS2
ENSG00000166816	283.5117408	14.08556214	4.322048389	2.59E-17	6.41E-16	LDHD
ENSG00000184486	410.506408	0.774441189	9.153117499	2.59E-17	6.41E-16	POU3F2
ENSG00000100100	2091.489028	196.4405314	3.413427875	2.66E-17	6.58E-16	PIK3IP1
ENSG00000123095	1638.305641	198.2859551	3.047787444	2.67E-17	6.60E-16	BHLHE41
ENSG00000183549	330.0724431	0	10.70054011	2.69E-17	6.64E-16	ACSM5
novel.215	192.6714139	20.36136804	3.250241215	2.70E-17	6.65E-16	-
ENSG00000238018	240.4881921	10.82236433	4.477159551	2.70E-17	6.66E-16	AC093110.1
ENSG00000275367	133.3963334	3.913628404	5.082486074	2.71E-17	6.68E-16	AC092111.1
ENSG00000163938	854.3289722	2562.46104	-1.584690174	2.73E-17	6.71E-16	GNL3
ENSG00000179967	218.2424595	774.002434	-1.826173226	2.99E-17	7.35E-16	PPP1R14BP3
ENSG00000167513	38.38406401	837.2607514	-4.445662549	3.07E-17	7.54E-16	CDT1
ENSG00000214491	313.5574173	2.772035718	6.791767185	3.11E-17	7.62E-16	SEC14L6
ENSG00000175792	453.8654928	2207.790446	-2.282502824	3.13E-17	7.65E-16	RUVBL1
ENSG00000163814	38.56949037	7674.886246	-7.636696084	3.60E-17	8.80E-16	CDCP1
ENSG00000072110	918.8639755	12957.34702	-3.817852325	3.62E-17	8.86E-16	ACTN1
ENSG00000259495	228.3231405	25.26330961	3.171387874	3.65E-17	8.92E-16	AC016705.2
ENSG00000135205	335.1689548	36.14562445	3.219335199	3.66E-17	8.94E-16	CCDC146
ENSG00000108883	949.2181413	2711.504602	-1.514268235	3.70E-17	9.02E-16	EFTUD2
ENSG00000119777	1137.644699	3585.594607	-1.65614172	3.79E-17	9.23E-16	TMEM214
ENSG00000185272	275.9304874	1.080229524	8.001111752	3.82E-17	9.29E-16	RBM11
ENSG00000179144	400.8677649	0	10.98059629	3.94E-17	9.59E-16	GIMAP7
ENSG00000174123	244.2023087	0	10.26562566	4.13E-17	1.00E-15	TLR10

ENSG00000059573	712.9938083	3025.124547	-2.085137799	4.35E-17	1.05E-15	ALDH18A1
ENSG00000165478	461.4937021	0.336469917	10.22218934	4.35E-17	1.05E-15	HEPACAM
ENSG00000145632	2936.015402	625.1649572	2.231336069	4.37E-17	1.06E-15	PLK2
ENSG00000174791	111.3545625	823.3402587	-2.886558083	4.38E-17	1.06E-15	RIN1
ENSG00000213203	305.2147231	0	10.58725467	5.22E-17	1.26E-15	GIMAP1
ENSG00000145050	571.9461026	2777.662232	-2.280094614	5.42E-17	1.31E-15	MANF
ENSG00000282608	240.9231516	0	10.24627727	5.43E-17	1.31E-15	ADORA3
ENSG00000187912	258.7850427	0	10.34919942	5.77E-17	1.39E-15	CLEC17A
ENSG00000144583	2.668144092	259.8863588	-6.589857345	5.80E-17	1.40E-15	4-Mar
ENSG00000169413	429.2316784	0	11.07925162	5.90E-17	1.42E-15	RNASE6
ENSG00000262877	307.0327347	40.77233088	2.918146781	6.15E-17	1.48E-15	AC110285.2
ENSG00000132470	7718.589639	130.640771	5.883384963	6.34E-17	1.52E-15	ITGB4
ENSG00000103855	941.5121623	3356.47261	-1.833962688	6.74E-17	1.62E-15	CD276
novel.999	744.1073336	0	11.87305967	6.98E-17	1.67E-15	-
ENSG00000178104	837.71586	2874.622923	-1.778948262	7.06E-17	1.69E-15	PDE4DIP
ENSG00000281404	145.211714	3.168712922	5.498015029	7.38E-17	1.77E-15	LINC01176
ENSG00000169432	1789.18439	26.99897841	6.044882635	7.62E-17	1.82E-15	SCN9A
ENSG00000179388	1463.355318	7.754125285	7.55415763	7.68E-17	1.83E-15	EGR3
ENSG00000127084	290.6354935	0	10.51662619	7.87E-17	1.88E-15	FGD3
ENSG00000196872	222.9106634	1.487519214	7.268535853	8.31E-17	1.98E-15	KIAA1211L
ENSG00000197992	655.1596842	0	11.68937926	8.43E-17	2.01E-15	CLEC9A
ENSG00000276231	141.2526915	2.261960402	6.020770845	8.57E-17	2.04E-15	PIK3R6
ENSG00000106105	774.8632091	3982.574118	-2.361850281	8.57E-17	2.04E-15	GARS
ENSG00000160691	1354.085782	11005.27564	-3.02284164	8.81E-17	2.09E-15	SHC1
ENSG00000232774	3.883945353	104.181428	-4.732486437	8.90E-17	2.11E-15	AL355916.1
ENSG00000151650	261.3782237	0	10.36362831	8.99E-17	2.13E-15	VENTX
ENSG00000140030	507.212631	0.356539013	10.35827673	9.20E-17	2.18E-15	GPR65
ENSG00000142512	249.1735845	17.39707049	3.835063777	9.24E-17	2.19E-15	SIGLEC10
ENSG00000158321	790.3080756	156.5191593	2.335073122	9.62E-17	2.27E-15	AUTS2
ENSG00000149636	170.1459894	623.8312314	-1.87476121	9.80E-17	2.31E-15	DSN1
ENSG00000165688	808.8885366	2384.727932	-1.559852411	9.84E-17	2.32E-15	PMPCA
ENSG00000010671	437.1155201	0.356539013	10.14371811	9.99E-17	2.36E-15	BTK
ENSG00000171817	145.164431	7.367932171	4.321302214	1.04E-16	2.45E-15	ZNF540
ENSG00000184730	340.8075869	0.743759607	8.8887286	1.08E-16	2.54E-15	APOBR
ENSG00000175489	289.4113154	0	10.51057629	1.09E-16	2.57E-15	LRRC25
ENSG00000163584	341.1336635	3037.969185	-3.154884367	1.14E-16	2.67E-15	RPL22L1
ENSG00000105697	321.807052	0	10.66399017	1.17E-16	2.74E-15	HAMP
ENSG00000100462	310.9806161	1200.025562	-1.948568874	1.20E-16	2.82E-15	PRMT5
ENSG00000136404	151.8600778	10.54854185	3.832447096	1.26E-16	2.94E-15	TM6SF1
ENSG00000259134	326.5369816	0	10.68469074	1.27E-16	2.98E-15	LINC00924
ENSG00000160791	441.852488	0	11.12105966	1.33E-16	3.10E-15	CCR5
ENSG00000189091	1427.897943	4777.668006	-1.742512552	1.35E-16	3.16E-15	SF3B3
ENSG00000164649	68.62654879	467.8068643	-2.767687822	1.40E-16	3.26E-15	CDCA7L
ENSG00000183401	485.4319653	86.44331128	2.488450307	1.41E-16	3.29E-15	CCDC159
ENSG00000181789	1609.094754	6618.664185	-2.040288724	1.43E-16	3.34E-15	COPG1
ENSG00000135931	136.2244032	1485.763919	-3.447123644	1.46E-16	3.40E-15	ARMC9
ENSG00000124749	563.8628381	13.32057757	5.39563521	1.46E-16	3.40E-15	COL21A1
ENSG00000196460	13.69846721	678.2339057	-5.630874061	1.53E-16	3.57E-15	RFX8
ENSG00000163734	4.548509626	438.5802249	-6.591820324	1.54E-16	3.58E-15	CXCL3
ENSG00000170891	181.8318878	3.048298346	5.844280202	1.55E-16	3.60E-15	CYTL1
ENSG00000198142	1167.08439	327.8869731	1.831970981	1.55E-16	3.60E-15	SOWAHC

ENSG00000047365	581.4338799	7.839153564	6.191045062	1.56E-16	3.61E-15	ARAP2
ENSG00000245105	115.3395124	5.289033778	4.429310498	1.56E-16	3.61E-15	A2M-AS1
ENSG00000163590	231.9625951	39.64263494	2.55082244	1.58E-16	3.66E-15	PPM1L
ENSG00000144366	1403.45596	216.2471564	2.697765421	1.65E-16	3.81E-15	GULP1
ENSG00000140945	47.15510501	1710.774478	-5.180244953	1.67E-16	3.85E-15	CDH13
ENSG00000138759	882.3867823	152.1118815	2.536547405	1.70E-16	3.92E-15	FRAS1
ENSG00000229132	72.42288057	332.5236157	-2.199708136	1.71E-16	3.93E-15	EIF4A1P10
ENSG00000168779	7.688839213	460.8976148	-5.911648067	1.72E-16	3.97E-15	SHOX2
novel.74	380.0290222	0	10.90386946	1.73E-16	3.98E-15	-
ENSG00000223891	337.3627621	46.12542475	2.866668379	1.75E-16	4.01E-15	OSER1-DT
ENSG00000162706	1416.885441	29.11043778	5.61092053	1.75E-16	4.02E-15	CADM3
ENSG00000198353	30.13828904	327.0998176	-3.441234033	1.79E-16	4.11E-15	HOXC4
ENSG00000286220	323.3084585	1.518200796	7.793205396	1.91E-16	4.37E-15	AC007546.3
ENSG00000260549	277.8762995	2002.052708	-2.848658246	1.91E-16	4.38E-15	MT1L
ENSG00000247774	337.2756464	0.693008929	8.878656064	1.92E-16	4.38E-15	PCED1B-AS1
ENSG00000089597	4615.611963	15514.50436	-1.749007714	1.94E-16	4.44E-15	GANAB
ENSG00000085999	40.91674207	292.9486712	-2.839969492	1.97E-16	4.51E-15	RAD54L
ENSG00000227039	250.0504281	7.692633727	5.034457114	2.06E-16	4.70E-15	ITGB2-AS1
ENSG00000103811	2036.292389	153.1135044	3.734135553	2.10E-16	4.78E-15	CTSH
ENSG00000175463	242.2663821	1.386017859	7.409092583	2.14E-16	4.87E-15	TBC1D10C
ENSG00000231160	137.3451838	6.765940506	4.336714611	2.18E-16	4.95E-15	KLF3-AS1
ENSG00000074842	1638.630949	6110.688914	-1.898824009	2.22E-16	5.04E-15	MYDGF
ENSG00000169436	18.43322991	215.1490755	-3.543085543	2.24E-16	5.08E-15	COL22A1
ENSG00000149257	4236.951363	26925.96867	-2.667922928	2.30E-16	5.21E-15	SERPINH1
ENSG00000073350	137.1160219	6.339737595	4.419529448	2.47E-16	5.61E-15	LLGL2
ENSG00000171291	328.4451301	29.87914677	3.464182025	2.53E-16	5.73E-15	ZNF439
ENSG00000196911	459.762478	136.5706848	1.750890386	2.55E-16	5.77E-15	KPNA5
ENSG00000130520	853.0062812	3237.57873	-1.924431016	2.58E-16	5.83E-15	LSM4
ENSG00000155265	137.349016	4.483782613	4.974505168	2.58E-16	5.84E-15	GOLGA7B
ENSG00000097021	599.7319278	1953.123188	-1.703419956	2.75E-16	6.21E-15	ACOT7
ENSG00000151117	346.0902804	42.34128235	3.036577098	2.76E-16	6.22E-15	TMEM86A
ENSG00000073282	189.4299647	3.108505634	5.893981356	2.77E-16	6.24E-15	TP63
ENSG00000074410	8.000884792	2106.273411	-8.043784552	2.79E-16	6.29E-15	CA12
ENSG00000117069	507.4817441	0.672939833	9.476911376	2.81E-16	6.33E-15	ST6GALNAC5
ENSG00000104635	255.945219	4161.403514	-4.023295638	2.82E-16	6.33E-15	SLC39A14
ENSG00000272848	280.4753504	11.92394722	4.544604381	2.87E-16	6.44E-15	AL135910.1
ENSG00000090097	5723.640682	1142.737194	2.324371904	2.90E-16	6.50E-15	PCBP4
ENSG00000168280	197.1444474	4.401194479	5.50189146	2.92E-16	6.55E-15	KIF5C
ENSG00000135624	2878.622757	9746.305975	-1.759514633	2.96E-16	6.63E-15	CCT7
ENSG00000235750	257.992035	1.682349583	7.194234925	2.96E-16	6.63E-15	KIAA0040
ENSG00000105486	247.6518206	889.052366	-1.844272365	3.01E-16	6.72E-15	LIG1
ENSG00000173786	18336.32792	2874.032915	2.673530508	3.07E-16	6.85E-15	CNP
ENSG00000135218	109.7947337	3.546476906	4.939928747	3.22E-16	7.18E-15	CD36
ENSG00000188859	140.5454754	3.302051734	5.434109445	3.31E-16	7.37E-15	FAM78B
ENSG00000103184	513.0551243	0.336469917	10.37481381	3.32E-16	7.40E-15	SEC14L5
ENSG00000160219	314.9401364	48.24446134	2.704888309	3.33E-16	7.40E-15	GAB3
ENSG00000148400	1969.480507	360.594557	2.449124895	3.35E-16	7.46E-15	NOTCH1
ENSG00000187840	326.9746073	1803.24187	-2.463560658	3.41E-16	7.57E-15	EIF4EBP1
ENSG00000100296	287.7024877	938.3578271	-1.705379739	3.48E-16	7.73E-15	THOC5
ENSG00000173757	1895.25934	721.7662849	1.392892417	3.48E-16	7.73E-15	STAT5B
ENSG00000283154	29.33127847	164.4229681	-2.486558447	3.61E-16	8.01E-15	IQCJ-SCHIP1

ENSG00000005844	554.2351608	0.387220594	10.48619927	3.71E-16	8.21E-15	ITGAL
ENSG00000092201	1488.446065	3955.768155	-1.41022527	3.76E-16	8.33E-15	SUPT16H
ENSG00000160712	932.918044	171.1152163	2.448120109	3.78E-16	8.37E-15	IL6R
ENSG00000108239	657.1140632	212.7369649	1.626965511	3.89E-16	8.60E-15	TBC1D12
ENSG00000181773	14.46611612	178.8971166	-3.624842874	3.93E-16	8.68E-15	GPR3
ENSG00000163083	153.3046849	5.554683921	4.759077074	3.94E-16	8.68E-15	INHBB
ENSG00000188580	314.1829457	0	10.62920874	4.01E-16	8.84E-15	NKAIN2
ENSG00000106799	2780.235271	909.4087465	1.612309384	4.12E-16	9.08E-15	TGFBR1
ENSG00000149054	10.37460566	126.6939269	-3.615637951	4.13E-16	9.08E-15	ZNF215
ENSG00000175785	209.4844967	0	10.04462661	4.13E-16	9.08E-15	PRIMA1
ENSG00000224318	233.0623198	0	10.19848645	4.14E-16	9.09E-15	CHL1-AS2
ENSG00000196878	20.54157494	612.0017094	-4.899570446	4.15E-16	9.11E-15	LAMB3
ENSG00000148429	1344.832108	275.9182855	2.284773423	4.31E-16	9.46E-15	USP6NL
ENSG00000091127	134.1980443	468.9889803	-1.805298097	4.35E-16	9.53E-15	PUS7
ENSG00000188015	5.42697852	125.479845	-4.526995488	4.37E-16	9.57E-15	S100A3
ENSG00000249353	205.4860695	820.1367199	-1.997375799	4.44E-16	9.71E-15	NPM1P27
ENSG00000117154	222.592464	0	10.13186222	4.52E-16	9.87E-15	IGSF21
ENSG00000185306	384.5823052	2.058957692	7.506967652	4.64E-16	1.01E-14	C12orf56
ENSG00000163803	203.7868705	6.786009602	4.904422086	4.69E-16	1.02E-14	PLB1
ENSG00000041353	10.44634872	698.98126	-6.06838862	4.73E-16	1.03E-14	RAB27B
ENSG00000136514	206.9271295	31.05167783	2.733240464	4.74E-16	1.03E-14	RTP4
ENSG00000224243	301.2488799	0	10.5686701	4.91E-16	1.07E-14	SOX1-OT
ENSG00000184445	206.9771767	1006.184544	-2.280799649	4.93E-16	1.07E-14	KNTC1
ENSG00000176890	63.04397423	810.1544919	-3.684731068	5.04E-16	1.09E-14	TYMS
ENSG00000143847	19.69312269	931.4305469	-5.563626521	5.09E-16	1.10E-14	PPFIA4
ENSG00000253552	4.870726937	130.5394324	-4.749306573	5.09E-16	1.10E-14	HOXA-AS2
ENSG00000109519	482.9783231	1531.972847	-1.665224778	5.17E-16	1.12E-14	GRPEL1
ENSG00000178965	235.5879467	0	10.21409992	5.21E-16	1.13E-14	ERICH3
ENSG00000134042	175.788268	1.436768536	6.935526214	5.27E-16	1.14E-14	MRO
ENSG00000183856	54.21523756	1435.708536	-4.725405905	5.29E-16	1.14E-14	IQGAP3
ENSG00000165312	807.4797025	119.5769228	2.757462982	5.66E-16	1.22E-14	OTUD1
ENSG00000120327	140.3770859	6.919348414	4.35387135	5.72E-16	1.23E-14	PCDHB14
ENSG00000129450	109.6589116	4.646775526	4.556719365	6.00E-16	1.29E-14	SIGLEC9
ENSG00000164124	762.0667505	39.9912584	4.247908611	6.08E-16	1.31E-14	TMEM144
ENSG00000149781	761.1632025	71.00557651	3.425313474	6.37E-16	1.37E-14	FERMT3
novel.327	349.8533223	0	10.78445392	6.42E-16	1.38E-14	-
ENSG00000137203	209.2734991	1.4981317	7.175403964	6.75E-16	1.45E-14	TFAP2A
ENSG00000185010	293.1635995	51.92068103	2.498975019	6.96E-16	1.49E-14	F8
ENSG00000065000	1964.78453	5587.845393	-1.507878088	7.09E-16	1.52E-14	AP3D1
ENSG00000169429	137.7632913	31148.08059	-7.82071225	7.44E-16	1.59E-14	CXCL8
ENSG00000076555	642.5354336	92.94257416	2.788030062	7.48E-16	1.60E-14	ACACB
ENSG00000105122	413.5349039	0.356539013	10.06368657	7.51E-16	1.61E-14	RASAL3
ENSG00000101000	115.4442064	1206.166065	-3.38553097	7.80E-16	1.67E-14	PROCR
ENSG00000237438	423.3702002	30.49329198	3.792032888	7.96E-16	1.70E-14	CECR7
ENSG00000184500	5523.307443	427.2513376	3.691753773	8.21E-16	1.75E-14	PROS1
ENSG00000139679	2605.112283	134.6070767	4.272887095	8.26E-16	1.76E-14	LPAR6
ENSG00000284526	246.0547105	0	10.27677256	8.35E-16	1.78E-14	AC015802.6
ENSG00000147576	218.7700628	12.90010685	4.09835961	8.53E-16	1.82E-14	ADHFE1
ENSG00000197256	8236.265697	3144.630345	1.389106571	8.55E-16	1.82E-14	KANK2
ENSG00000120280	187.3364503	0	9.883259828	8.61E-16	1.83E-14	CXorf21
ENSG00000180233	462.6283997	76.89022746	2.586768164	8.71E-16	1.85E-14	ZNRF2

ENSG00000140538	1093.410125	0.336469917	11.46652427	8.80E-16	1.87E-14	NTRK3
ENSG00000128944	168.4747277	1197.46441	-2.829611219	8.97E-16	1.90E-14	KNSTRN
ENSG00000053747	1109.500078	107.1464147	3.370248881	8.98E-16	1.90E-14	LAMA3
ENSG00000164611	115.499724	1732.26295	-3.906834796	9.36E-16	1.98E-14	PTTG1
ENSG00000226415	54.83538214	304.3431976	-2.471948117	1.01E-15	2.14E-14	TPI1P1
ENSG00000265808	1388.650809	4421.85308	-1.671041029	1.02E-15	2.16E-14	SEC22B
ENSG00000166928	245.3972242	0	10.27255746	1.04E-15	2.19E-14	MS4A14
ENSG00000107317	1721.799668	6.471920532	8.056222941	1.04E-15	2.19E-14	PTGDS
ENSG00000162882	353.5502417	12.8424681	4.76898965	1.04E-15	2.19E-14	HAAO
ENSG00000018280	281.1020636	12.28981445	4.519180256	1.06E-15	2.24E-14	SLC11A1
ENSG00000136250	487.8792917	0.387220594	10.30221189	1.07E-15	2.26E-14	AOAH
ENSG00000132744	183.6200246	0	9.854387396	1.13E-15	2.37E-14	ACY3
ENSG00000174442	166.0186644	819.7930707	-2.30431735	1.14E-15	2.40E-14	ZWILCH
ENSG00000099194	13638.78854	3728.220421	1.871086696	1.14E-15	2.40E-14	SCD
ENSG00000162738	225.3703177	24.98704699	3.170304399	1.22E-15	2.57E-14	VANGL2
ENSG00000104043	407.4362394	60.83415635	2.744264196	1.23E-15	2.57E-14	ATP8B4
ENSG00000133048	16.66243072	721.4346647	-5.436609242	1.25E-15	2.61E-14	CHI3L1
ENSG00000082126	9.002567969	129.2972375	-3.838699193	1.26E-15	2.63E-14	MPP4
ENSG00000196411	402.0846223	1292.1027	-1.684139617	1.28E-15	2.69E-14	EPHB4
ENSG00000126453	176.7517723	674.5271837	-1.93235743	1.29E-15	2.69E-14	BCL2L12
ENSG00000180398	1509.805589	7529.614481	-2.318299063	1.31E-15	2.74E-14	MCFD2
ENSG00000160593	547.7405229	0	11.4310213	1.32E-15	2.75E-14	JAML
ENSG00000259673	186.1620198	30.66445723	2.598413852	1.32E-15	2.76E-14	IQCH-AS1
ENSG00000123360	170.5530713	4.555886656	5.207464543	1.35E-15	2.81E-14	PDE1B
ENSG00000130244	545.7833197	166.9729595	1.708284994	1.38E-15	2.87E-14	FAM98C
ENSG00000115935	3284.377108	1078.825526	1.606391407	1.44E-15	3.00E-14	WIPF1
ENSG00000136295	990.2281954	5849.072125	-2.562297741	1.45E-15	3.00E-14	TTYH3
ENSG00000111879	135.1885159	8.122432658	4.044437477	1.45E-15	3.01E-14	FAM184A
ENSG00000165661	357.5718055	1172.071063	-1.712552326	1.46E-15	3.03E-14	QSOX2
ENSG00000141756	2246.654202	17751.03001	-2.982083858	1.51E-15	3.13E-14	FKBP10
ENSG00000070190	210.1472226	0	10.04881335	1.53E-15	3.17E-14	DAPP1
ENSG00000113532	364.9999549	38.33676508	3.256265403	1.54E-15	3.18E-14	ST8SIA4
ENSG00000101444	738.0244005	3615.62789	-2.292634141	1.56E-15	3.24E-14	AHCY
ENSG00000124875	35.31264202	1931.949767	-5.775005589	1.59E-15	3.28E-14	CXCL6
ENSG00000272888	1210.585723	377.5869538	1.68084966	1.63E-15	3.36E-14	LINC01578
novel.367	1.774560141	141.7469155	-6.294644145	1.68E-15	3.46E-14	-
ENSG00000245522	3.959520633	92.43583808	-4.55105107	1.70E-15	3.51E-14	AC026250.1
ENSG00000224616	204.1264261	34.20147753	2.573489884	1.71E-15	3.53E-14	RTCA-AS1
ENSG00000105852	118.6870511	1.762625967	6.0510619	1.83E-15	3.76E-14	PON3
ENSG00000163823	515.8636054	8.325306974	5.974628213	1.84E-15	3.78E-14	CCR1
ENSG00000011485	746.4651488	2334.110821	-1.64480252	1.84E-15	3.78E-14	PPP5C
ENSG00000144354	26.3133368	628.1349183	-4.577193376	1.85E-15	3.81E-14	CDCA7
ENSG00000160310	6610.475146	1837.463964	1.847050113	1.87E-15	3.83E-14	PRMT2
ENSG00000138092	76.07759279	456.9475854	-2.586216261	1.92E-15	3.94E-14	CENPO
ENSG00000103260	9923.675306	1964.464904	2.336868844	1.93E-15	3.95E-14	METRN
ENSG00000214787	207.1194088	0	10.02817675	1.95E-15	3.98E-14	MS4A4E
ENSG00000065150	2576.900285	6350.350053	-1.301199022	1.96E-15	4.02E-14	IPO5
ENSG00000225492	189.1054549	2.058957692	6.475801097	1.98E-15	4.04E-14	GBP1P1
ENSG00000182580	300.9714662	18.43472168	4.032064855	1.98E-15	4.04E-14	EPHB3
ENSG00000157214	32.51430339	977.5597429	-4.910376006	2.02E-15	4.12E-14	STEAP2
ENSG00000138670	264.9995147	0.693008929	8.530501306	2.04E-15	4.16E-14	RASGEF1B

ENSG00000197081	1103.056096	6782.700608	-2.620374816	2.05E-15	4.17E-14	IGF2R
ENSG00000101003	58.56323361	600.1336621	-3.356884699	2.11E-15	4.30E-14	GINS1
ENSG00000286546	342.6770401	0	10.75450882	2.13E-15	4.33E-14	AL079338.1
novel.302	297.0114904	0	10.54826129	2.15E-15	4.37E-14	-
ENSG00000100401	1488.815124	5701.629377	-1.937281543	2.17E-15	4.41E-14	RANGAP1
ENSG00000187902	193.6187473	1.467450118	7.068736001	2.18E-15	4.43E-14	SHISA7
ENSG00000187583	2.465593581	150.6592822	-5.948976427	2.22E-15	4.49E-14	PLEKHN1
ENSG00000167613	328.6785209	0	10.69416357	2.22E-15	4.50E-14	LAIR1
ENSG00000138615	578.9922994	4.515748464	6.985941189	2.23E-15	4.51E-14	CILP
ENSG00000176014	2470.783459	10030.94179	-2.021406791	2.27E-15	4.59E-14	TUBB6
ENSG00000144642	1413.152945	369.140928	1.936011771	2.29E-15	4.63E-14	RBMS3
ENSG00000173207	179.6363561	1162.638865	-2.694835889	2.30E-15	4.64E-14	CKS1B
ENSG00000172893	253.3992211	978.5135511	-1.949591304	2.38E-15	4.80E-14	DHCR7
ENSG00000187554	233.2722622	2.50754145	6.538313624	2.41E-15	4.85E-14	TLR5
ENSG00000227036	169.8045401	27.44383776	2.625142344	2.49E-15	5.01E-14	LINC00511
ENSG00000187961	181.2312599	732.5929691	-2.014727044	2.51E-15	5.06E-14	KLHL17
ENSG00000185634	1517.063503	121.0385591	3.645832749	2.53E-15	5.10E-14	SHC4
ENSG00000107611	562.53278	29.48833016	4.259888082	2.54E-15	5.11E-14	CUBN
ENSG00000162722	311.1673532	0	10.61518395	2.57E-15	5.16E-14	TRIM58
ENSG00000100934	973.8275615	4711.444972	-2.274474528	2.60E-15	5.22E-14	SEC23A
ENSG00000165801	5711.681646	1452.185344	1.975902241	2.62E-15	5.25E-14	ARHGEF40
ENSG00000112182	274.4155052	9.527235343	4.858642808	2.62E-15	5.25E-14	BACH2
ENSG00000114993	2237.18572	353.4163996	2.66275482	2.63E-15	5.26E-14	RTKN
ENSG00000164347	253.0576293	797.7740332	-1.656643087	2.71E-15	5.43E-14	GFM2
ENSG00000164032	1056.583322	5869.117112	-2.473834137	2.84E-15	5.69E-14	H2AFZ
ENSG00000004487	850.6770955	2351.304269	-1.466688973	2.89E-15	5.77E-14	KDM1A
ENSG00000183044	1008.853777	90.43649255	3.476886386	2.90E-15	5.80E-14	ABAT
ENSG00000160949	75.10076912	449.5686081	-2.581370647	2.92E-15	5.82E-14	TONSL
ENSG00000114270	528.3656337	13315.31245	-4.655402843	2.97E-15	5.92E-14	COL7A1
ENSG00000167772	227.9574721	3956.445821	-4.117461604	2.97E-15	5.92E-14	ANGPTL4
ENSG00000134917	500.2656035	0.713078025	9.44593683	3.04E-15	6.05E-14	ADAMTS8
ENSG00000105355	681.613675	3993.388745	-2.550670004	3.05E-15	6.06E-14	PLIN3
ENSG00000145907	1706.400029	4215.660828	-1.304807878	3.12E-15	6.20E-14	G3BP1
ENSG00000106560	352.7950431	4.106018629	6.449174171	3.12E-15	6.20E-14	GIMAP2
ENSG00000127824	107.6172516	1174.6296	-3.448643409	3.13E-15	6.22E-14	TUBA4A
ENSG00000267594	297.9089066	0	10.55260519	3.14E-15	6.22E-14	CYP4F24P
ENSG00000244617	99.24475868	5.676254372	4.116495362	3.21E-15	6.37E-14	ASPRV1
ENSG00000132718	3915.990701	901.0479596	2.120020722	3.23E-15	6.39E-14	SYT11
ENSG00000102172	1253.192121	4407.7476	-1.814314968	3.25E-15	6.44E-14	SMS
ENSG00000166582	95.6536269	582.3050549	-2.606310159	3.28E-15	6.49E-14	CENPV
ENSG00000198019	228.5785024	0	10.17054621	3.42E-15	6.76E-14	FCGR1B
ENSG00000143851	187.8707766	0	9.887138039	3.43E-15	6.77E-14	PTPN7
ENSG00000274536	222.3771201	0	10.13038479	3.44E-15	6.79E-14	AL034397.3
ENSG00000146555	436.6655877	17.65339242	4.61686101	3.46E-15	6.82E-14	SDK1
ENSG00000134627	222.096859	20.52423256	3.445983356	3.48E-15	6.85E-14	PIWIL4
ENSG00000163735	1.536764924	8663.877295	-12.46689481	3.54E-15	6.97E-14	CXCL5
ENSG00000127463	864.9454476	3536.154321	-2.031487437	3.58E-15	7.03E-14	EMC1
ENSG00000130726	2403.300791	8053.42134	-1.744630956	3.62E-15	7.11E-14	TRIM28
ENSG00000133019	228.5634156	0	10.17034182	3.63E-15	7.13E-14	CHRM3
ENSG00000170390	480.1801983	110.6780411	2.117663343	3.66E-15	7.18E-14	DCLK2
ENSG00000136628	1543.577437	4605.300329	-1.577110396	3.67E-15	7.19E-14	EPRS

ENSG00000138434	9744.460444	2394.799274	2.024541044	3.73E-15	7.30E-14	ITPRID2
ENSG00000257337	633.7273089	69.23431148	3.190555241	3.73E-15	7.31E-14	AC068888.1
ENSG00000183691	4.612802068	264.7635484	-5.851397903	3.86E-15	7.55E-14	NOG
ENSG00000070778	1718.721502	491.1552959	1.807239509	4.07E-15	7.96E-14	AL162171.1
ENSG00000117143	372.7252212	2087.789397	-2.486086752	4.18E-15	8.17E-14	UAP1
ENSG00000118785	2205.432227	71.31521766	4.951009182	4.19E-15	8.18E-14	SPP1
ENSG00000100368	197.0029693	0	9.955649881	4.30E-15	8.39E-14	CSF2RB
ENSG00000161847	177.7992275	751.4306606	-2.0796434	4.31E-15	8.40E-14	RAVER1
ENSG00000155034	108.6853073	479.2620303	-2.141082987	4.38E-15	8.53E-14	FBXL18
ENSG00000164010	504.617258	137.4738413	1.876175682	4.40E-15	8.56E-14	ERMAP
ENSG00000115484	1133.860374	5282.086956	-2.219978194	4.40E-15	8.56E-14	CCT4
ENSG00000090520	632.283299	1759.649415	-1.476733608	4.41E-15	8.57E-14	DNAJB11
ENSG00000127418	114.0995637	1930.36854	-4.081144863	4.65E-15	9.03E-14	FGFRL1
ENSG00000088448	4252.809661	863.2734463	2.30074107	4.71E-15	9.15E-14	ANKRD10
ENSG00000160179	343.1949387	11.2108692	4.9256924	4.73E-15	9.16E-14	ABCG1
ENSG00000176204	245.9482213	0	10.27607755	4.84E-15	9.39E-14	LRRTM4
ENSG00000134222	193.4425669	848.0242271	-2.132543916	5.10E-15	9.88E-14	PSRC1
ENSG00000286619	3.298717306	101.1823393	-4.928563222	5.36E-15	1.04E-13	AC119673.2
ENSG00000066032	207.568246	0	10.03144253	5.71E-15	1.10E-13	CTNNA2
ENSG00000102053	110.7064385	7.806031838	3.8163124	5.79E-15	1.12E-13	ZC3H12B
ENSG00000146250	496.7284716	10.94637493	5.498179966	5.82E-15	1.12E-13	PRSS35
ENSG00000144677	2482.673018	566.3340933	2.131604395	5.91E-15	1.14E-13	CTDSPL
ENSG00000131016	2929.375142	15086.37604	-2.364594611	5.96E-15	1.15E-13	AKAP12
ENSG00000108984	193.5244104	26.61980177	2.85606589	6.05E-15	1.16E-13	MAP2K6
ENSG00000214517	578.8181318	1575.197264	-1.444265698	6.08E-15	1.17E-13	PPME1
ENSG00000123374	213.2282465	1281.573251	-2.58759094	6.43E-15	1.24E-13	CDK2
ENSG00000159131	383.1024496	1579.866689	-2.044208843	6.50E-15	1.25E-13	GART
ENSG00000213088	185.602177	4.330374705	5.425118939	6.52E-15	1.25E-13	ACKR1
ENSG00000225889	315.0235343	0.356539013	9.67142224	6.62E-15	1.27E-13	AC012368.1
ENSG00000105366	168.0130414	0	9.726288064	6.84E-15	1.31E-13	SIGLEC8
ENSG00000171246	12.2852013	432.373967	-5.141428247	6.88E-15	1.32E-13	NPTX1
ENSG00000170624	553.213591	7.246490114	6.238315591	7.07E-15	1.35E-13	SGCD
ENSG00000118777	374.2989688	12.83172722	4.863824986	7.23E-15	1.38E-13	ABCG2
ENSG00000130150	1912.327774	410.1403118	2.220720886	7.37E-15	1.41E-13	MOSPD2
ENSG00000139645	990.9812719	3576.122474	-1.851394722	7.68E-15	1.47E-13	ANKRD52
ENSG00000141497	273.2730831	0.693008929	8.576164693	7.73E-15	1.48E-13	ZMYND15
ENSG00000163106	187.3146683	0	9.883019011	7.83E-15	1.49E-13	HPGDS
ENSG00000058262	3565.957588	13071.62293	-1.874099841	7.84E-15	1.49E-13	SEC61A1
ENSG00000027847	325.9988669	1337.42159	-2.036351748	7.88E-15	1.50E-13	B4GALT7
ENSG00000114646	160.8141095	4.494523493	5.130827049	8.13E-15	1.55E-13	CSPG5
ENSG00000164128	235.3551668	0	10.21262706	8.14E-15	1.55E-13	NPY1R
ENSG00000072694	265.8561007	0	10.38816501	8.24E-15	1.57E-13	FCGR2B
ENSG00000178947	104.8896875	3.923085015	4.73384778	8.28E-15	1.57E-13	SMIM10L2A
ENSG00000103740	165.1814957	2.782648203	5.862814321	8.30E-15	1.58E-13	ACSBG1
ENSG00000137501	486.1114152	77.34369151	2.648242762	8.35E-15	1.58E-13	SYTL2
ENSG00000213047	342.6178472	71.14546507	2.2647625	8.43E-15	1.60E-13	DENND1B
ENSG00000068438	520.9132655	1406.261419	-1.432851142	8.47E-15	1.60E-13	FTSJ1
ENSG00000165474	794.5650349	11.50591665	6.11028575	8.59E-15	1.62E-13	GJB2
ENSG00000087589	140.3169817	4.963176346	4.810782159	8.69E-15	1.64E-13	CASS4
ENSG00000107829	1600.318899	505.5382644	1.662469175	8.82E-15	1.67E-13	FBXW4
ENSG00000176809	231.0704505	31.22528323	2.882728044	8.84E-15	1.67E-13	LRRC37A3

ENSG00000185668	281.5775949	0.387220594	9.509277894	8.92E-15	1.68E-13	POU3F1
ENSG00000155886	489.2234569	4.401194479	6.807427626	8.92E-15	1.68E-13	SLC24A2
ENSG00000234323	164.0522953	0	9.691549086	9.30E-15	1.75E-13	LINC01505
ENSG00000046889	608.8710543	0.336469917	10.62186525	9.40E-15	1.77E-13	PREX2
ENSG00000120251	219.9146752	0	10.11435536	9.40E-15	1.77E-13	GRIA2
ENSG00000182199	691.9481639	3661.627449	-2.403921452	9.47E-15	1.78E-13	SHMT2
ENSG00000134061	231.8986146	0	10.19089042	9.54E-15	1.79E-13	CD180
ENSG00000149212	3032.183678	223.8725661	3.758714224	1.01E-14	1.90E-13	SESN3
ENSG00000227195	170.637992	0	9.748848881	1.02E-14	1.91E-13	MIR663AHG
ENSG00000197249	312.1572261	15.24465539	4.369693055	1.02E-14	1.91E-13	SERPINA1
ENSG00000162998	1939.540167	16.46922139	6.875888601	1.02E-14	1.91E-13	FRZB
ENSG00000132128	1182.705863	2925.544773	-1.306613989	1.04E-14	1.94E-13	LRRC41
ENSG00000135299	318.9993096	18.4278336	4.101941049	1.13E-14	2.11E-13	ANKRD6
ENSG00000104870	4715.883725	1147.262352	2.039385677	1.13E-14	2.11E-13	FCGRT
ENSG00000120457	179.7566355	0	9.823449019	1.15E-14	2.16E-13	KCNJ5
ENSG00000111860	677.567669	187.23432	1.854984771	1.16E-14	2.16E-13	CEP85L
ENSG00000227953	208.8551496	3.801514564	5.751227404	1.19E-14	2.21E-13	LINC01341
ENSG00000125753	973.4665679	2673.122056	-1.457324001	1.21E-14	2.25E-13	VASP
ENSG00000251429	226.3869177	3.912472529	5.847602152	1.22E-14	2.28E-13	AC098679.2
ENSG00000136828	195.8518606	19.66018677	3.31478944	1.26E-14	2.35E-13	RALGPS1
ENSG00000145936	158.2649198	0	9.639730965	1.26E-14	2.35E-13	KCNMB1
ENSG00000155660	1619.362158	16032.89051	-3.307543062	1.27E-14	2.36E-13	PDIA4
ENSG00000177406	256.8425357	51.08718843	2.330631858	1.28E-14	2.37E-13	AC021054.1
ENSG00000254726	32.71706771	412.987136	-3.657883722	1.32E-14	2.45E-13	MEX3A
ENSG00000166387	1165.63576	35.76598107	5.021888026	1.37E-14	2.53E-13	PPFIBP2
ENSG00000108010	735.6058493	2090.263594	-1.506806622	1.39E-14	2.58E-13	GLRX3
ENSG00000196419	2619.225564	8602.175814	-1.715585873	1.41E-14	2.62E-13	XRCC6
ENSG00000096968	765.6732495	177.9056395	2.10417354	1.42E-14	2.62E-13	JAK2
ENSG00000168843	322.1555508	2.200597239	7.21073274	1.45E-14	2.68E-13	FSTL5
ENSG00000114279	162.2534889	1.702418679	6.517753304	1.46E-14	2.70E-13	FGF12
ENSG00000144381	2649.336354	11226.88341	-2.083300432	1.55E-14	2.87E-13	HSPD1
ENSG00000136108	341.6653845	1569.431639	-2.199771364	1.56E-14	2.87E-13	CKAP2
ENSG00000125885	52.39110059	241.5350303	-2.205487798	1.59E-14	2.93E-13	MCM8
ENSG00000158517	176.0405375	0	9.793474073	1.65E-14	3.04E-13	NCF1
ENSG00000185615	162.1431393	4.768345977	5.104186225	1.65E-14	3.05E-13	PDIA2
ENSG00000147536	39.98644595	419.2624537	-3.390912127	1.70E-14	3.13E-13	GINS4
ENSG00000125384	17.11065434	396.4648775	-4.537131576	1.71E-14	3.14E-13	PTGER2
ENSG00000137726	389.4193328	0	10.93882288	1.71E-14	3.14E-13	FXYD6
ENSG00000141696	366.2493481	2249.197514	-2.618793826	1.72E-14	3.16E-13	P3H4
ENSG00000100883	706.0263188	2424.60447	-1.779871812	1.74E-14	3.18E-13	SRP54
ENSG00000166265	178.6187315	7.968896356	4.491799328	1.77E-14	3.24E-13	CYYR1
ENSG00000203952	176.3356457	1.069617038	7.356642046	1.78E-14	3.26E-13	CCDC160
novel.1068	376.3931979	54.67765634	2.778450099	1.79E-14	3.27E-13	-
ENSG00000248323	3.328947419	915.7787986	-8.103532812	1.89E-14	3.46E-13	LUCAT1
ENSG00000163577	115.0850638	364.9527067	-1.665263213	1.98E-14	3.63E-13	EIF5A2
ENSG00000177542	221.0073484	991.9066648	-2.166377226	2.01E-14	3.67E-13	SLC25A22
ENSG00000228878	136.5103082	8.245158985	4.049187283	2.02E-14	3.68E-13	SEPT7-AS1
ENSG00000079246	2877.464337	8364.612242	-1.53955088	2.07E-14	3.77E-13	XRCC5
ENSG00000198113	167.0321999	750.7843528	-2.168815369	2.19E-14	4.00E-13	TOR4A
ENSG00000241749	9.307162927	153.2883469	-4.036120851	2.20E-14	4.01E-13	RPSAP52
ENSG00000127743	588.2737355	2.169915658	8.087046377	2.23E-14	4.06E-13	IL17B

ENSG00000151746	1638.629773	312.0222304	2.392200338	2.39E-14	4.35E-13	BICD1
ENSG00000162881	109.4717609	5.309102873	4.353237925	2.43E-14	4.41E-13	OXER1
ENSG00000171777	139.0198786	0	9.452675984	2.45E-14	4.46E-13	RASGRP4
ENSG00000161940	111.4653115	1.406086955	6.282244549	2.61E-14	4.74E-13	BCL6B
ENSG00000196369	969.5177908	194.7966974	2.315746563	2.66E-14	4.83E-13	SRGAP2B
ENSG00000168411	339.2013014	1395.720711	-2.040715048	2.69E-14	4.89E-13	RFWD3
ENSG00000123689	46.6109192	1627.78851	-5.127076233	2.98E-14	5.40E-13	G0S2
ENSG00000138185	802.7585569	116.300638	2.78464888	3.23E-14	5.85E-13	ENTPD1
ENSG00000088930	825.6598491	2289.543183	-1.471533107	3.26E-14	5.90E-13	XRN2
ENSG00000107738	2279.989035	266.9776903	3.093216826	3.34E-14	6.03E-13	VSIR
ENSG00000109321	0.586481695	329.7214339	-9.095669149	3.36E-14	6.07E-13	AREG
ENSG00000251615	118.0102351	15.03116859	2.970446318	3.41E-14	6.16E-13	AC104825.1
ENSG00000140368	155.012331	4.025742245	5.282945058	3.48E-14	6.28E-13	PSTPIP1
ENSG00000187474	301.4101139	0.336469917	9.607355707	3.49E-14	6.29E-13	FPR3
ENSG00000101197	285.4508836	0	10.49074124	3.54E-14	6.37E-13	BIRC7
ENSG00000118308	260.5614256	0.774441189	8.493046468	3.54E-14	6.37E-13	LRMP
ENSG00000205339	1626.466867	4826.130697	-1.569163836	3.55E-14	6.38E-13	IPO7
ENSG00000173391	1805.778207	16.39138515	6.775634904	3.55E-14	6.38E-13	OLR1
ENSG00000160752	493.3630292	1315.11458	-1.414383224	3.55E-14	6.39E-13	FDPS
ENSG00000154102	13.11832503	252.3695356	-4.268002411	3.57E-14	6.42E-13	C16orf74
ENSG00000204161	203.6188092	0	10.00326755	3.61E-14	6.48E-13	TMEM273
ENSG00000231313	37.6343222	182.9873042	-2.282212014	3.62E-14	6.48E-13	CLIC1P1
ENSG00000171444	1302.795598	325.9144564	1.999127831	3.78E-14	6.77E-13	MCC
ENSG00000182557	162.6174319	0	9.678827415	3.80E-14	6.81E-13	SPNS3
ENSG00000107833	154.0893436	852.4568395	-2.46847123	3.94E-14	7.04E-13	NPM3
ENSG00000182700	1125.844063	218.9754111	2.363468682	3.98E-14	7.12E-13	IGIP
ENSG00000130762	670.6082584	32.30910877	4.371376543	4.16E-14	7.44E-13	ARHGEF16
ENSG00000157827	3359.747038	804.7477149	2.061710372	4.18E-14	7.47E-13	FMNL2
ENSG00000204628	9674.408938	23848.85983	-1.301671234	4.19E-14	7.47E-13	RACK1
ENSG00000246430	542.186108	6.553481185	6.354191009	4.26E-14	7.60E-13	LINC00968
ENSG00000143036	87.51824498	7.490786892	3.540799235	4.30E-14	7.67E-13	SLC44A3
ENSG00000092929	532.8539842	38.7114938	3.776958367	4.31E-14	7.68E-13	UNC13D
ENSG00000157303	265.4266401	14.95333235	4.136642697	4.33E-14	7.70E-13	SUSD3
ENSG00000177868	379.5775756	104.9051185	1.85544829	4.46E-14	7.92E-13	SVBP
ENSG00000114790	133.0519064	3.944309986	5.075527123	4.47E-14	7.93E-13	ARHGEF26
ENSG00000172794	127.9731155	0	9.333286794	4.70E-14	8.34E-13	RAB37
ENSG00000106624	5511.923278	1971.674329	1.483058527	4.73E-14	8.39E-13	AEBP1
ENSG00000170266	586.0323766	2116.857158	-1.852881712	4.79E-14	8.49E-13	GLB1
ENSG00000167208	225.8136247	0	10.15253641	4.89E-14	8.67E-13	SNX20
ENSG00000160460	442.5188066	20.71662278	4.425194124	4.96E-14	8.78E-13	SPTBN4
ENSG00000183960	296.1062398	0	10.54386082	5.05E-14	8.95E-13	KCNH8
ENSG00000136231	16.93894686	892.8318467	-5.716688777	5.07E-14	8.96E-13	IGF2BP3
ENSG00000112561	858.6150131	115.0693123	2.89711459	5.08E-14	8.97E-13	TFEB
ENSG00000120438	2039.04072	6152.524574	-1.593353004	5.08E-14	8.97E-13	TCP1
ENSG00000161654	197.6784611	564.075926	-1.512478312	5.08E-14	8.97E-13	LSM12
ENSG00000198894	708.5016301	196.823946	1.848090999	5.09E-14	8.98E-13	CIPC
ENSG00000125835	1030.909321	3218.380355	-1.642465863	5.10E-14	8.98E-13	SNRPB
ENSG00000100365	181.6137511	0	9.838225253	5.13E-14	9.03E-13	NCF4
ENSG00000239332	16.31499789	295.0905898	-4.175465198	5.15E-14	9.07E-13	LINC01119
ENSG00000119203	416.1915242	1174.616714	-1.496981361	5.18E-14	9.12E-13	CPSF3
ENSG00000089159	993.1288361	2952.287357	-1.5716482	5.27E-14	9.27E-13	PXN

ENSG00000058056	274.3704006	796.4461674	-1.537798971	5.29E-14	9.30E-13	USP13
ENSG00000106853	295.4849084	1939.682435	-2.714862205	5.31E-14	9.32E-13	PTGR1
ENSG00000166508	1126.92859	4801.695607	-2.0911506	5.32E-14	9.34E-13	MCM7
ENSG00000227082	207.1473733	0.713078025	8.1730302	5.34E-14	9.37E-13	AC244021.1
ENSG00000100353	1857.346022	5156.993161	-1.473280908	5.52E-14	9.68E-13	EIF3D
ENSG00000092445	1032.793263	385.4461293	1.422085759	5.67E-14	9.93E-13	TYRO3
ENSG00000061918	167.8429001	16.66276749	3.325347635	5.76E-14	1.01E-12	GUCY1B1
ENSG00000167721	460.457229	1605.261912	-1.801972878	6.05E-14	1.06E-12	TSR1
novel.379	261.823898	17.07822952	3.94312418	6.11E-14	1.07E-12	-
ENSG00000162496	6428.029976	1102.86879	2.543128329	6.13E-14	1.07E-12	DHRS3
ENSG00000053918	155.0937472	0	9.610453384	6.24E-14	1.09E-12	KCNQ1
ENSG00000105605	1.50778846	559.1363298	-8.531663955	6.35E-14	1.11E-12	CACNG7
ENSG00000109089	303.8373864	1475.418066	-2.279803698	6.42E-14	1.12E-12	CDR2L
ENSG00000147113	149.2374924	0	9.554912775	6.42E-14	1.12E-12	DIPK2B
ENSG00000116062	571.6162375	1703.879747	-1.575637982	6.89E-14	1.20E-12	MSH6
ENSG00000157551	2.461832635	358.8171024	-7.19719653	7.01E-14	1.22E-12	KCNJ15
ENSG00000255389	186.3786174	9.161368115	4.336007094	7.06E-14	1.23E-12	Z97989.1
ENSG00000105185	789.308899	2338.581294	-1.566946643	7.12E-14	1.24E-12	PDCD5
ENSG00000110328	588.8856345	23.68934946	4.642351905	7.24E-14	1.26E-12	GALNT18
ENSG00000277196	162.2052523	1.029478846	7.244523625	7.27E-14	1.26E-12	AC007325.2
ENSG00000258227	419.4900759	0	11.04633454	7.30E-14	1.27E-12	CLEC5A
ENSG00000256663	9.930142978	178.3149029	-4.163792119	7.31E-14	1.27E-12	AC112777.1
ENSG00000102900	498.0255065	1571.640444	-1.658253382	7.49E-14	1.30E-12	NUP93
ENSG00000073008	347.4334136	2003.214334	-2.52770425	7.57E-14	1.31E-12	PVR
ENSG00000162873	401.4253992	1.00940975	8.569855505	7.64E-14	1.32E-12	KLHDC8A
ENSG00000118407	221.4593181	1.130980201	7.669122745	7.94E-14	1.37E-12	FILIP1
ENSG00000077063	171.4382201	2.406040094	6.115863625	8.07E-14	1.39E-12	CTTNBP2
ENSG00000180828	165.6681121	1.130980201	7.254443893	8.59E-14	1.48E-12	BHLHE22
ENSG00000178685	2746.225488	664.1682196	2.04744185	8.69E-14	1.50E-12	PARP10
ENSG00000109685	972.1808074	3323.98632	-1.773482243	8.85E-14	1.52E-12	NSD2
ENSG00000197785	341.5301322	1759.691646	-2.36547062	8.89E-14	1.53E-12	ATAD3A
ENSG00000084623	1762.22504	5042.310196	-1.516706818	8.90E-14	1.53E-12	EIF3I
ENSG00000196407	93.73070823	7.235749235	3.702242031	9.04E-14	1.55E-12	THEM5
ENSG00000196924	12883.02463	43088.92507	-1.741842595	9.13E-14	1.57E-12	FLNA
ENSG00000148848	54.51219557	1299.919039	-4.576682926	9.34E-14	1.60E-12	ADAM12
ENSG00000170606	1320.967094	4073.587041	-1.624803746	9.37E-14	1.61E-12	HSPA4
ENSG00000231999	195.419721	20.16666606	3.278355925	9.80E-14	1.68E-12	LRRC8C-DT
ENSG00000134668	33.03731936	1634.551377	-5.626998982	9.81E-14	1.68E-12	SPOCD1
ENSG00000182919	480.0800479	147.2346325	1.706555882	9.85E-14	1.69E-12	C11orf54
ENSG00000151849	157.6475676	531.259824	-1.752224822	9.87E-14	1.69E-12	CENPJ
ENSG00000123562	3086.06038	7686.61539	-1.31662496	1.04E-13	1.77E-12	MORF4L2
ENSG00000166794	2095.692573	6226.051183	-1.57089979	1.05E-13	1.79E-12	PPIB
ENSG00000242247	617.3222186	1615.35553	-1.387808097	1.07E-13	1.83E-12	ARFGAP3
ENSG00000232024	71.16033795	290.4224734	-2.028906395	1.08E-13	1.85E-12	LSM12P1
ENSG00000245025	118.8873081	11.92266295	3.322534489	1.09E-13	1.86E-12	AC107959.1
ENSG00000166451	78.55099299	457.2001014	-2.542061077	1.09E-13	1.86E-12	CENPN
ENSG00000092853	20.02515573	652.2276915	-5.023194347	1.09E-13	1.86E-12	CLSPN
ENSG00000286473	117.9926127	0	9.216116659	1.11E-13	1.89E-12	AC133485.7
ENSG00000113407	1019.942164	3047.60576	-1.579315175	1.11E-13	1.89E-12	TARS
ENSG00000165280	3692.042149	11218.27701	-1.60338608	1.12E-13	1.91E-12	VCP
novel.259	178.9757659	0	9.817123174	1.12E-13	1.91E-12	-

ENSG00000144040	1040.647857	297.9699999	1.804523226	1.13E-13	1.92E-12	SFXN5
ENSG00000168393	358.1933893	1722.488524	-2.265863992	1.15E-13	1.94E-12	DTYMK
ENSG00000149970	216.2854945	1.049547942	7.657594054	1.15E-13	1.95E-12	CNKSR2
novel.494	0	129.22316	-9.593740696	1.16E-13	1.96E-12	-
ENSG00000239264	32.20218654	348.2916767	-3.435876693	1.17E-13	1.98E-12	TXNDC5
ENSG00000116221	722.4019198	2252.446866	-1.640771943	1.18E-13	2.00E-12	MRPL37
ENSG00000206053	817.7716672	2588.708344	-1.662377095	1.21E-13	2.04E-12	JPT2
ENSG00000198740	721.9338947	234.8889436	1.620945084	1.24E-13	2.09E-12	ZNF652
ENSG00000171314	2215.20176	10915.89276	-2.300955699	1.25E-13	2.11E-12	PGAM1
ENSG00000167775	337.4149168	1677.778562	-2.314031559	1.25E-13	2.11E-12	CD320
ENSG00000009950	403.3557857	5.359725157	6.264716786	1.25E-13	2.11E-12	MLXIPL
novel.777	5.196776466	269.8245742	-5.703460296	1.25E-13	2.11E-12	-
ENSG00000132334	1543.746312	299.6062567	2.366381024	1.27E-13	2.13E-12	PTPRE
ENSG00000168439	1661.504347	5969.848359	-1.84527535	1.27E-13	2.13E-12	STIP1
ENSG00000130032	261.3273787	0	10.36331338	1.28E-13	2.15E-12	PRRG3
ENSG00000203685	162.6995049	0	9.680099938	1.30E-13	2.18E-12	STUM
ENSG00000101846	1504.435412	264.9053288	2.504787292	1.31E-13	2.19E-12	STS
ENSG00000196396	686.2236294	2187.674548	-1.67263061	1.31E-13	2.20E-12	PTPN1
ENSG00000258733	82.15018831	3.159256312	4.676821949	1.33E-13	2.24E-12	LINC02328
ENSG00000148843	526.3733381	2009.2358	-1.932547252	1.33E-13	2.24E-12	PDCD11
ENSG00000166949	950.7528965	2675.644191	-1.492755323	1.34E-13	2.24E-12	SMAD3
ENSG00000101811	171.2938085	566.7433439	-1.726607944	1.34E-13	2.25E-12	CSTF2
ENSG00000164694	296.9917551	3.658590747	6.355232551	1.38E-13	2.31E-12	FNDC1
ENSG00000029993	216.5499133	1367.113637	-2.658822427	1.39E-13	2.32E-12	HMGB3
ENSG00000166189	291.3728132	915.1644857	-1.65145443	1.39E-13	2.33E-12	HPS6
ENSG00000263465	1778.814248	626.2038927	1.50589242	1.40E-13	2.34E-12	SRSF8
ENSG00000088827	492.2061454	0.693008929	9.431904249	1.40E-13	2.34E-12	SIGLEC1
ENSG00000197062	467.1953302	136.7203683	1.774226279	1.42E-13	2.37E-12	ZSCAN26
ENSG00000000938	126.0811108	2.629111901	5.628054477	1.44E-13	2.40E-12	FGR
ENSG00000075388	255.26202	0	10.32942659	1.44E-13	2.40E-12	FGF4
ENSG00000183763	41.06245076	214.1193056	-2.381554349	1.44E-13	2.40E-12	TRAIP
ENSG00000135454	792.3715889	66.50758539	3.57772601	1.46E-13	2.44E-12	B4GALNT1
ENSG00000102383	117.4158755	3.404837359	5.05976736	1.48E-13	2.47E-12	ZDHHC15
ENSG00000221963	1018.233477	314.4865515	1.694676448	1.49E-13	2.48E-12	APOL6
ENSG00000072657	22.757877	367.1974921	-4.01226378	1.49E-13	2.48E-12	TRHDE
ENSG00000169245	362.1041005	12.34056513	4.878760555	1.50E-13	2.49E-12	CXCL10
ENSG00000117091	216.4319264	0	10.09132155	1.51E-13	2.51E-12	CD48
ENSG00000175287	338.6616045	31.65623803	3.413584644	1.52E-13	2.53E-12	PHYHD1
ENSG00000110660	61.20304198	617.9856912	-3.336640011	1.54E-13	2.55E-12	SLC35F2
ENSG00000141655	215.4974156	14.41830765	3.915258431	1.55E-13	2.56E-12	TNFRSF11A
ENSG00000164934	395.1928357	1195.096522	-1.596827619	1.58E-13	2.61E-12	DCAF13
ENSG00000163378	183.0723347	564.8369762	-1.625497301	1.58E-13	2.62E-12	EOGT
ENSG00000213585	2476.845051	7782.254262	-1.651677156	1.59E-13	2.63E-12	VDAC1
ENSG00000263528	102.0686407	547.0637987	-2.421825602	1.59E-13	2.63E-12	IKBKE
ENSG00000113240	1321.317553	306.4348103	2.108387346	1.61E-13	2.65E-12	CLK4
ENSG00000179256	113.9181552	0	9.165786371	1.63E-13	2.68E-12	SMCO3
ENSG00000170802	886.9441815	264.4193382	1.746020845	1.64E-13	2.71E-12	FOXN2
ENSG00000168495	441.600564	1219.510671	-1.465602376	1.70E-13	2.80E-12	POLR3D
ENSG00000104833	292.8479518	0.356539013	9.566140572	1.70E-13	2.80E-12	TUBB4A
ENSG00000135363	1076.572888	41.14949974	4.704758817	1.87E-13	3.07E-12	LMO2
ENSG00000167747	300.5974918	1463.390896	-2.283542963	1.88E-13	3.10E-12	C19orf48

ENSG00000115602	3.240764379	367.2715608	-6.810209108	1.89E-13	3.11E-12	IL1RL1
ENSG00000135097	125.8573198	1.00940975	6.879114931	1.90E-13	3.12E-12	MSI1
ENSG00000126217	380.642341	6.789605622	5.784627471	1.90E-13	3.12E-12	MCF2L
ENSG00000114850	1912.226417	8607.970768	-2.1704705	1.95E-13	3.20E-12	SSR3
ENSG00000182389	292.3383418	4.077777192	6.13101521	1.95E-13	3.20E-12	CACNB4
novel.390	139.4076416	0	9.456655914	1.95E-13	3.20E-12	-
ENSG00000184432	1765.716518	6797.13303	-1.944692881	2.00E-13	3.27E-12	COPB2
ENSG00000070814	754.3933657	2059.139658	-1.448509953	2.00E-13	3.28E-12	TCOF1
ENSG00000152953	112.0743593	5.278421292	4.391621052	2.10E-13	3.44E-12	STK32B
ENSG00000108924	372.5590529	1.161661783	8.399747559	2.11E-13	3.46E-12	HLF
ENSG00000136848	1280.630311	432.8885843	1.565371127	2.13E-13	3.48E-12	DAB2IP
ENSG00000111452	777.5833157	2.395427609	8.315544911	2.13E-13	3.48E-12	ADGRD1
ENSG00000162174	102.184689	9.599339387	3.404283946	2.19E-13	3.57E-12	ASRGL1
novel.154	111.2266187	2.272572888	5.670006241	2.19E-13	3.57E-12	-
ENSG00000204789	188.0493941	8.325435369	4.502018492	2.23E-13	3.63E-12	ZNF204P
ENSG00000078269	122.0337631	938.873609	-2.943667395	2.23E-13	3.63E-12	SYNJ2
ENSG00000152154	265.6192049	33.62747051	2.988075076	2.26E-13	3.67E-12	TMEM178A
ENSG00000121858	410.6366134	8.010318817	5.667687621	2.26E-13	3.67E-12	TNFSF10
ENSG00000169247	787.7551695	118.8832715	2.728369039	2.28E-13	3.71E-12	SH3TC2
ENSG00000163902	3318.810012	9001.609846	-1.439505634	2.29E-13	3.71E-12	RPN1
ENSG00000131981	5054.169358	1184.473449	2.093145705	2.32E-13	3.76E-12	LGALS3
ENSG00000120659	0	112.1048392	-9.390992124	2.34E-13	3.79E-12	TNFSF11
ENSG00000138039	280.5603107	0	10.46578288	2.36E-13	3.82E-12	LHCGR
ENSG00000089220	11062.85313	4473.140917	1.306330904	2.36E-13	3.82E-12	PEBP1
ENSG00000172817	1688.684777	136.5314925	3.626999983	2.38E-13	3.85E-12	CYP7B1
ENSG00000157600	259.9308958	862.8796756	-1.731011809	2.41E-13	3.90E-12	TMEM164
ENSG00000173706	543.2134859	3079.049396	-2.503083242	2.42E-13	3.92E-12	HEG1
ENSG00000214960	152.7723243	31.10358438	2.293802902	2.43E-13	3.92E-12	CRPPA
ENSG00000158825	14.06484731	350.2727992	-4.640665994	2.48E-13	4.00E-12	CDA
ENSG00000129003	2775.189054	644.1487234	2.107095894	2.48E-13	4.00E-12	VPS13C
ENSG00000136542	224.459823	1241.843926	-2.468423156	2.51E-13	4.05E-12	GALNT5
ENSG00000230438	182.1338329	16.41962659	3.458031243	2.52E-13	4.06E-12	SERPINB9P1
ENSG00000156787	62.24190734	268.113538	-2.106810026	2.52E-13	4.06E-12	TBC1D31
ENSG00000176896	120.9842278	17.78429108	2.759894964	2.59E-13	4.16E-12	TCEANC
ENSG00000203727	1005.641607	18.1846927	5.781084538	2.62E-13	4.22E-12	SAMD5
ENSG00000117480	212.2856711	20.91158154	3.345953355	2.65E-13	4.27E-12	FAAH
ENSG00000138166	168.6001631	2454.588375	-3.86398528	2.66E-13	4.28E-12	DUSP5
ENSG00000131044	116.499113	1.396630344	6.349530749	2.75E-13	4.41E-12	TTLL9
ENSG00000167261	160.9693485	1.682349583	6.514719229	2.75E-13	4.41E-12	DPEP2
ENSG00000113739	229.4141439	16235.37087	-6.145175982	2.83E-13	4.54E-12	STC2
ENSG00000163931	3134.139211	9008.937687	-1.523294118	2.86E-13	4.58E-12	TKT
ENSG00000186638	22.25037553	164.6498927	-2.885133527	3.08E-13	4.93E-12	KIF24
ENSG00000234743	39.56232673	219.05856	-2.467835137	3.12E-13	5.00E-12	EIF5AP4
ENSG00000081818	133.9691393	0	9.399878496	3.13E-13	5.01E-12	PCDHB4
ENSG00000270419	186.0471823	22.24684872	3.063851585	3.25E-13	5.19E-12	CAHM
ENSG00000180881	176.2699849	22.87032215	2.936379031	3.28E-13	5.24E-12	CAPS2
ENSG00000102144	3028.32615	16589.24652	-2.453651613	3.32E-13	5.31E-12	PGK1
ENSG00000112280	266.4769906	0	10.39175517	3.52E-13	5.61E-12	COL9A1
ENSG00000223768	322.1009179	953.5702249	-1.56610875	3.53E-13	5.63E-12	LINC00205
ENSG00000148335	218.3427087	929.7914464	-2.090848664	3.57E-13	5.69E-12	NTMT1
ENSG00000228305	97.93909345	367.5455332	-1.907928847	3.57E-13	5.69E-12	AC016734.1

ENSG00000140553	884.2932596	2358.517368	-1.415167892	3.62E-13	5.76E-12	UNC45A
ENSG00000100036	89.65757557	367.560932	-2.034224751	3.63E-13	5.78E-12	SLC35E4
ENSG00000172578	218.5336197	0.336469917	9.143498401	3.65E-13	5.81E-12	KLHL6
ENSG00000198380	834.1785493	2871.979085	-1.783632873	3.69E-13	5.86E-12	GFPT1
ENSG00000099785	1179.571532	378.7809194	1.638751026	3.87E-13	6.14E-12	2-Mar
ENSG00000167074	717.4486095	119.9676922	2.582432383	3.95E-13	6.27E-12	TEF
ENSG00000187391	471.7858795	61.80959247	2.928880125	4.01E-13	6.36E-12	MAGI2
ENSG00000157916	1314.248255	3342.337432	-1.346655145	4.05E-13	6.42E-12	RER1
ENSG00000108846	74.04815671	2538.252787	-5.098688523	4.10E-13	6.49E-12	ABCC3
ENSG00000225733	6159.126857	2357.179822	1.385675018	4.13E-13	6.54E-12	FGD5-AS1
ENSG00000100345	8075.164319	40933.25205	-2.341707978	4.15E-13	6.56E-12	MYH9
ENSG00000130816	1288.864704	3659.77226	-1.505601555	4.21E-13	6.65E-12	DNMT1
ENSG00000165283	934.9583927	2931.233815	-1.648666402	4.33E-13	6.85E-12	STOML2
ENSG00000197776	351.2769723	41.40384825	3.090453555	4.35E-13	6.87E-12	KLHDC1
ENSG00000026559	59.71448553	1831.546702	-4.939495731	4.46E-13	7.03E-12	KCNG1
ENSG00000154319	0.308355904	263.2401633	-9.659027988	4.49E-13	7.08E-12	FAM167A
ENSG00000259969	86.60710995	1.874739808	5.581731603	4.50E-13	7.09E-12	AL049838.1
ENSG00000064042	3588.4982	36.12264848	6.631284743	4.50E-13	7.09E-12	LIMCH1
ENSG00000188677	156.2259528	822.9693012	-2.397626911	4.53E-13	7.13E-12	PARVB
ENSG00000118705	3030.096011	7631.758583	-1.332665927	4.55E-13	7.16E-12	RPN2
ENSG00000133321	483.2385255	43.1115324	3.481624501	4.57E-13	7.19E-12	PLAAT4
ENSG00000197299	26.35345356	235.1556681	-3.158305645	4.60E-13	7.23E-12	BLM
ENSG00000064102	389.2705366	1124.327315	-1.530017961	4.65E-13	7.30E-12	INTS13
ENSG00000104321	2.140868972	210.1872178	-6.624276798	4.68E-13	7.34E-12	TRPA1
ENSG00000184144	374.0031402	0.387220594	9.919005899	4.69E-13	7.36E-12	CNTN2
ENSG00000157502	2.152223082	153.913327	-6.173697648	4.85E-13	7.61E-12	PWWP3B
ENSG00000279675	112.7336242	0	9.150832083	5.15E-13	8.07E-12	AC080100.1
ENSG00000160886	15.68478068	1217.004136	-6.280083676	5.18E-13	8.12E-12	LY6K
ENSG00000122140	353.0996975	892.7540361	-1.338024227	5.34E-13	8.36E-12	MRPS2
ENSG00000129295	182.7322103	36.77983875	2.310520658	5.54E-13	8.67E-12	LRRC6
ENSG00000161647	49.17533827	263.3858781	-2.419708597	5.63E-13	8.80E-12	MPP3
ENSG00000164588	281.3056074	0	10.4696176	5.84E-13	9.13E-12	HCN1
ENSG00000204282	173.6456472	0.713078025	7.91871968	5.93E-13	9.26E-12	TNRC6C-AS1
ENSG00000184454	1979.358773	0	13.28451785	6.15E-13	9.59E-12	NCMAP
ENSG00000128342	103.2943998	1871.912429	-4.17960111	6.20E-13	9.66E-12	LIF
ENSG00000166676	178.2816716	6.205114513	4.854896918	6.22E-13	9.69E-12	TVP23A
ENSG00000164938	1342.015033	402.6276108	1.736811402	6.37E-13	9.92E-12	TP53INP1
ENSG00000033100	1078.468201	4296.934243	-1.994256497	6.49E-13	1.01E-11	CHPF2
ENSG00000186174	327.2109347	2901.919215	-3.148795267	6.50E-13	1.01E-11	BCL9L
ENSG00000179364	3473.831654	1148.500938	1.596951616	6.54E-13	1.02E-11	PACS2
ENSG00000145147	4111.520005	107.3678639	5.257551009	6.80E-13	1.06E-11	SLIT2
ENSG00000120278	300.4894483	17.45971792	4.095752911	6.94E-13	1.08E-11	PLEKHG1
ENSG00000105509	2.113146158	520.817796	-7.943851178	6.96E-13	1.08E-11	HAS1
ENSG00000128059	166.0645794	588.7286861	-1.82647965	7.03E-13	1.09E-11	PPAT
ENSG00000149380	17.20474962	230.9505882	-3.746210318	7.05E-13	1.09E-11	P4HA3
ENSG00000151835	469.4215186	1709.784591	-1.864682624	7.08E-13	1.10E-11	SACS
ENSG00000122679	130.8087785	0	9.364854564	7.21E-13	1.12E-11	RAMP3
ENSG00000174004	237.5699138	31.31707118	2.928665172	7.23E-13	1.12E-11	NRROS
ENSG00000082397	3731.565038	1349.005809	1.468058141	7.27E-13	1.12E-11	EPB41L3
ENSG00000161911	154.6278459	0	9.606745874	7.28E-13	1.12E-11	TREML1
ENSG00000123124	3213.347503	636.5141955	2.336115307	7.34E-13	1.13E-11	WWP1

ENSG00000174899	2.137108026	81.92730603	-5.268625902	7.35E-13	1.14E-11	PQLC2L
ENSG00000150995	1377.5207	210.772141	2.708992899	7.63E-13	1.18E-11	ITPR1
ENSG00000188352	391.5218692	1009.554428	-1.366445057	7.70E-13	1.19E-11	FOCAD
ENSG00000283994	119.0047246	0	9.229023041	7.90E-13	1.22E-11	AC092652.3
ENSG00000260257	240.6544146	29.85090533	3.012266719	7.93E-13	1.22E-11	AL035071.1
ENSG00000108342	2.082916045	27408.47356	-13.67990202	7.95E-13	1.22E-11	CSF3
novel.194	228.1018161	0	10.16749746	8.05E-13	1.24E-11	-
ENSG00000164104	1051.172742	3405.910012	-1.696103761	8.12E-13	1.25E-11	HMGB2
ENSG00000287115	100.5457375	3.892403433	4.677990432	8.28E-13	1.27E-11	AC009951.3
ENSG00000012174	301.1576035	1024.423887	-1.7666499	8.32E-13	1.28E-11	MBTPS2
ENSG00000106628	937.2548577	2788.42236	-1.573003479	8.40E-13	1.29E-11	POLD2
ENSG00000260855	176.2581047	10.33479826	4.088970582	8.53E-13	1.31E-11	AL591848.4
ENSG00000178882	493.1675838	18.45581825	4.746872061	8.59E-13	1.32E-11	RFLNA
ENSG00000197841	434.5294768	125.0376825	1.795929838	8.62E-13	1.32E-11	ZNF181
ENSG00000051596	217.5073346	589.1462503	-1.437608242	8.89E-13	1.36E-11	THOC3
ENSG00000172322	279.1373424	0	10.45844872	8.90E-13	1.36E-11	CLEC12A
ENSG00000106367	685.5563722	2774.19667	-2.016696633	9.03E-13	1.38E-11	AP1S1
ENSG00000165816	118.0142533	2.211209725	5.764985908	9.18E-13	1.40E-11	VWA2
ENSG00000107815	164.3649538	482.2885557	-1.553339736	9.22E-13	1.41E-11	TWNK
ENSG00000137474	179.8791384	0.336469917	8.863032274	9.38E-13	1.43E-11	MYO7A
ENSG00000163531	1164.022179	11.66634103	6.649966522	9.39E-13	1.43E-11	NFASC
ENSG00000152213	108.9447709	1.049547942	6.664381219	9.42E-13	1.44E-11	ARL11
ENSG00000154277	2044.814971	6984.656673	-1.772274092	9.44E-13	1.44E-11	UCHL1
ENSG00000078579	105.1641949	1.487519214	6.185156256	9.48E-13	1.44E-11	FGF20
ENSG00000183833	129.1484161	3.302051734	5.309121119	9.49E-13	1.44E-11	MAATS1
ENSG00000164116	168.6873349	2.038888596	6.320577979	9.55E-13	1.45E-11	GUCY1A1
ENSG00000164576	1174.755066	375.4784825	1.645643988	9.67E-13	1.47E-11	SAP30L
ENSG00000197536	218.6315479	52.06360485	2.070259914	9.96E-13	1.51E-11	C5orf56
novel.41	165.5768516	0.387220594	8.74332178	1.05E-12	1.59E-11	-
ENSG00000273015	506.2492575	171.6643211	1.56151125	1.07E-12	1.63E-11	AC008124.1
ENSG00000197859	139.2580301	2.71182843	5.632453125	1.09E-12	1.65E-11	ADAMTSL2
ENSG00000163909	291.2952556	11.34305213	4.677616239	1.12E-12	1.70E-11	HEYL
ENSG00000184271	382.6337403	97.62195783	1.971819809	1.15E-12	1.74E-11	AC139768.1
ENSG00000121774	2027.384968	4555.332738	-1.167946142	1.16E-12	1.75E-11	KHDRBS1
ENSG00000169169	70.7418028	589.6676491	-3.059901267	1.16E-12	1.76E-11	CPT1C
ENSG00000164418	1403.87446	65.71179084	4.419406081	1.21E-12	1.82E-11	GRIK2
ENSG00000251141	115.1315913	9.081091731	3.645175501	1.22E-12	1.84E-11	MRPS30-DT
ENSG00000091831	87.04598822	3.139187216	4.764847531	1.22E-12	1.84E-11	ESR1
ENSG00000134313	3324.745051	1124.381789	1.563908478	1.26E-12	1.90E-11	KIDINS220
ENSG00000182132	256.5599495	0	10.336762	1.26E-12	1.90E-11	KCNIP1
ENSG00000114354	1266.821095	3068.447794	-1.276247557	1.27E-12	1.91E-11	TFG
ENSG00000229647	2.450478525	131.7163646	-5.75675078	1.28E-12	1.93E-11	MYOSLID
ENSG00000203880	769.4370298	241.158037	1.673811812	1.32E-12	1.99E-11	PCMTD2
ENSG00000175166	2403.454566	10262.82604	-2.094218473	1.34E-12	2.02E-11	PSMD2
ENSG00000164129	94.99679833	0	8.903621162	1.35E-12	2.03E-11	NPY5R
ENSG00000132305	988.8127344	2393.443271	-1.275345215	1.37E-12	2.05E-11	IMMT
ENSG00000172009	605.3236774	2160.707972	-1.835664981	1.39E-12	2.08E-11	AC006538.1
ENSG00000115594	409.5723764	4299.054121	-3.391944032	1.43E-12	2.15E-11	IL1R1
ENSG00000077238	499.4591152	2510.636917	-2.329601294	1.44E-12	2.16E-11	IL4R
ENSG00000184113	567.4328096	26.20048693	4.439486117	1.49E-12	2.23E-11	CLDN5
ENSG00000183484	197.5024503	0.387220594	8.997534838	1.50E-12	2.25E-11	GPR132

ENSG00000049239	1581.898965	5038.36163	-1.671241278	1.52E-12	2.28E-11	H6PD
ENSG00000110665	159.4627424	1.00940975	7.228219769	1.56E-12	2.33E-11	C11orf21
ENSG00000115165	166.4884417	2.292641984	6.236923322	1.56E-12	2.34E-11	CYTIP
ENSG00000071564	921.6666514	2276.344176	-1.304263823	1.59E-12	2.38E-11	TCF3
novel.102	88.68207683	6.440083075	3.781019302	1.60E-12	2.39E-11	-
ENSG00000158042	537.7805955	2338.159549	-2.1203925	1.60E-12	2.40E-11	MRPL17
ENSG00000081138	307.8550867	0	10.59975154	1.61E-12	2.41E-11	CDH7
ENSG00000108561	949.1986086	3180.857317	-1.744773783	1.64E-12	2.44E-11	C1QBP
ENSG00000108370	700.4653454	47.81911033	3.87619621	1.65E-12	2.46E-11	RGS9
ENSG00000170373	430.9795594	7.919429948	5.750843639	1.65E-12	2.47E-11	CST1
ENSG00000168283	1596.796236	583.3037109	1.452577922	1.67E-12	2.48E-11	BMI1
ENSG00000272316	183.883933	32.68070819	2.497907346	1.69E-12	2.51E-11	AL021368.2
ENSG00000100342	709.0919266	127.1945928	2.477951378	1.83E-12	2.73E-11	APOL1
ENSG00000140090	310.0467488	2.160459047	7.167604228	1.86E-12	2.76E-11	SLC24A4
ENSG00000188897	100.6483948	2.486316478	5.328945681	1.87E-12	2.78E-11	AC099489.1
ENSG00000157020	1503.239982	4547.720113	-1.597067659	1.89E-12	2.81E-11	SEC13
ENSG00000121807	179.2582934	0	9.81942311	1.89E-12	2.81E-11	CCR2
ENSG00000140988	15112.74876	43512.62632	-1.525676423	1.93E-12	2.86E-11	RPS2
ENSG00000177283	1433.057246	180.0671732	2.991876963	1.93E-12	2.87E-11	FZD8
ENSG00000183682	217.6349505	35.00672869	2.630207951	1.97E-12	2.92E-11	BMP8A
ENSG00000183971	3.969621095	71.96107193	-4.188269082	1.98E-12	2.94E-11	NPW
ENSG00000180592	265.273682	24.35527283	3.449717999	1.99E-12	2.95E-11	SKIDA1
ENSG00000078114	1546.807661	57.92694961	4.735640843	2.01E-12	2.98E-11	NEBL
ENSG00000149136	1434.73108	3786.691691	-1.400210328	2.02E-12	2.98E-11	SSRP1
ENSG00000115241	1289.153199	3503.793302	-1.44254689	2.03E-12	3.00E-11	PPM1G
ENSG00000067208	1742.161367	504.1472247	1.789372793	2.07E-12	3.06E-11	EVI5
ENSG00000105855	18400.72612	283.8029836	6.018280861	2.07E-12	3.06E-11	ITGB8
ENSG00000136531	828.4199026	29.61978958	4.801373335	2.10E-12	3.10E-11	SCN2A
ENSG00000214870	217.2881734	0.356539013	9.135537594	2.11E-12	3.11E-11	AC004540.1
ENSG00000114541	666.936818	18.97804711	5.127087839	2.11E-12	3.11E-11	FRMD4B
ENSG00000251442	199.126217	13.29793993	3.917388199	2.19E-12	3.22E-11	LINC01094
ENSG00000230910	241.5507913	3.792057953	5.968497317	2.25E-12	3.32E-11	AL391807.1
ENSG00000259207	20.6837365	1116.014937	-5.754170742	2.27E-12	3.33E-11	ITGB3
ENSG00000177084	460.3238589	1376.8985	-1.580488836	2.29E-12	3.37E-11	POLE
ENSG00000184005	94.1074026	3.495726228	4.725865454	2.31E-12	3.40E-11	ST6GALNAC3
ENSG00000178773	21.23253711	1655.250139	-6.286231494	2.35E-12	3.45E-11	CPNE7
ENSG00000132432	853.315021	3427.531387	-2.006047853	2.42E-12	3.56E-11	SEC61G
ENSG00000115107	321.6438753	3085.749376	-3.26208383	2.43E-12	3.56E-11	STEAP3
ENSG00000283196	90.3451328	313.5874578	-1.795239812	2.45E-12	3.60E-11	AC006453.2
ENSG00000035115	757.8258251	241.9849795	1.646386339	2.48E-12	3.63E-11	SH3YL1
ENSG00000188613	259.3675378	18.31315122	3.82456044	2.56E-12	3.75E-11	NANOS1
ENSG00000065485	176.952128	1715.103667	-3.277102569	2.57E-12	3.76E-11	PDIA5
ENSG00000170537	160.7377944	14.15394177	3.511448938	2.62E-12	3.83E-11	TMC7
ENSG00000067704	1024.830442	2682.425474	-1.388255896	2.64E-12	3.86E-11	IARS2
ENSG00000168913	159.7137441	2.864080462	5.799500502	2.65E-12	3.88E-11	ENHO
ENSG00000100433	96.45472522	0	8.925150488	2.69E-12	3.92E-11	KCNK10
ENSG00000112624	596.6058591	201.6665794	1.565276062	2.79E-12	4.08E-11	BICRAL
ENSG00000228058	155.9637264	0	9.618759654	2.80E-12	4.09E-11	LINC01736
ENSG00000198081	443.8617836	146.3000237	1.600204987	2.88E-12	4.20E-11	ZBTB14
ENSG00000186088	1366.206611	109.7246819	3.640064198	2.96E-12	4.31E-11	GSAP
ENSG00000164182	275.3281344	811.6126488	-1.559677906	3.07E-12	4.48E-11	NDUFAF2

ENSG00000177614	211.2559926	9.803497972	4.43476743	3.13E-12	4.56E-11	PGBD5
ENSG00000149573	229.951734	0.774441189	8.305895088	3.14E-12	4.57E-11	MPZL2
ENSG00000167695	391.215052	1139.258395	-1.541828533	3.14E-12	4.57E-11	FAM57A
ENSG00000188732	268.4945024	16.01922497	4.072335905	3.19E-12	4.63E-11	FAM221A
ENSG00000180383	121.5494409	1.518200796	6.3848515	3.20E-12	4.65E-11	DEFB124
ENSG00000116260	2625.190342	11559.59451	-2.138575343	3.23E-12	4.68E-11	QSOX1
ENSG00000103222	73.02405005	364.7682317	-2.320290386	3.28E-12	4.75E-11	ABCC1
ENSG00000066056	325.0452347	17.02735045	4.264046866	3.29E-12	4.78E-11	TIE1
ENSG00000135547	95.72243574	1.365948763	6.072171611	3.33E-12	4.83E-11	HEY2
ENSG00000147724	214.4488366	0	10.07845505	3.34E-12	4.84E-11	FAM135B
ENSG00000130985	4123.373192	11702.0155	-1.504829972	3.35E-12	4.84E-11	UBA1
ENSG00000105519	1487.939296	103.0135204	3.852873422	3.36E-12	4.86E-11	CAPS
ENSG00000178921	333.1668852	1267.717494	-1.927990976	3.37E-12	4.87E-11	PFAS
ENSG00000108559	296.6860375	884.5716658	-1.576181612	3.37E-12	4.87E-11	NUP88
ENSG00000077312	604.6069139	1539.035808	-1.347955277	3.40E-12	4.90E-11	SNRPA
ENSG00000162702	226.7346196	1092.715637	-2.268704072	3.41E-12	4.93E-11	ZNF281
ENSG00000120262	138.4339385	15.50110571	3.148347399	3.43E-12	4.95E-11	CCDC170
ENSG00000013375	634.2099359	1862.132029	-1.553882577	3.54E-12	5.10E-11	PGM3
ENSG00000230590	1686.921998	461.3633608	1.870564657	3.56E-12	5.13E-11	FTX
ENSG00000125869	100.2331499	0	8.980571806	3.61E-12	5.20E-11	LAMP5
ENSG00000027001	162.0378317	451.5283293	-1.478836916	3.65E-12	5.26E-11	MIPEP
ENSG00000152689	242.5596397	25.56884116	3.252317785	3.70E-12	5.33E-11	RASGRP3
ENSG00000178977	105.2775931	4.340987191	4.605967224	3.71E-12	5.33E-11	LINC00324
ENSG00000134531	2779.110386	11441.81075	-2.041632677	3.75E-12	5.39E-11	EMP1
ENSG00000182968	127.2003814	0	9.324772028	3.76E-12	5.40E-11	SOX1
ENSG00000115275	1041.014478	2646.084108	-1.345748886	3.77E-12	5.42E-11	MOGS
ENSG00000165732	921.0958111	3465.378158	-1.911707697	3.85E-12	5.52E-11	DDX21
ENSG00000107331	8291.984196	1094.994888	2.920942985	3.93E-12	5.64E-11	ABCA2
ENSG00000072195	48.79971191	1225.132973	-4.650948634	4.08E-12	5.85E-11	SPEG
ENSG00000239887	159.2198189	11.27223236	3.81008861	4.16E-12	5.95E-11	C1orf226
ENSG00000277829	143.5877628	5.238154706	4.805009702	4.17E-12	5.96E-11	AL031651.2
ENSG00000106004	2.12199297	248.5417312	-6.873253397	4.27E-12	6.11E-11	HOXA5
ENSG00000065427	1315.232814	4136.271789	-1.653108616	4.28E-12	6.12E-11	KARS
ENSG00000135838	445.3838489	45.7205752	3.280221454	4.31E-12	6.16E-11	NPL
ENSG00000181790	304.6922067	7.406914488	5.375084842	4.33E-12	6.18E-11	ADGRB1
ENSG00000089356	81.88717757	0	8.68952898	4.33E-12	6.18E-11	FXYD3
ENSG00000112303	117.2101335	1.161661783	6.748606547	4.35E-12	6.20E-11	VNN2
ENSG00000150681	219.6554247	0.693008929	8.264637179	4.35E-12	6.20E-11	RGS18
ENSG00000143515	1173.630155	2658.634293	-1.179680208	4.36E-12	6.21E-11	ATP8B2
ENSG00000060762	845.1378295	182.1794501	2.212227305	4.36E-12	6.21E-11	MPC1
ENSG00000247746	156.5985302	30.06233717	2.37919345	4.37E-12	6.23E-11	USP51
ENSG00000084774	742.4563371	2131.135499	-1.521384929	4.41E-12	6.27E-11	CAD
ENSG00000102981	135.8439486	10.21322781	3.722686504	4.52E-12	6.43E-11	PARD6A
ENSG00000205364	284.6372512	16.86821035	4.070045134	4.53E-12	6.44E-11	MT1M
ENSG00000285564	82.67531213	0	8.703099803	4.66E-12	6.62E-11	AL031121.2
ENSG00000165733	715.0818971	1699.518387	-1.248968231	4.67E-12	6.63E-11	BMS1
ENSG00000129167	77.81063963	2.10970837	5.181923173	4.67E-12	6.63E-11	TPH1
ENSG00000178764	448.6973802	134.7607286	1.736363579	4.80E-12	6.81E-11	ZHX2
ENSG00000163864	78.38053915	5.381078522	3.864806058	4.82E-12	6.84E-11	NMNAT3
ENSG00000164236	1190.352469	73.63216673	4.012909181	4.87E-12	6.90E-11	ANKRD33B
ENSG00000010030	124.0158174	19.63652165	2.666970784	4.88E-12	6.91E-11	ETV7

ENSG00000254343	78.83775213	2.884149558	4.777699693	4.93E-12	6.98E-11	AC091563.1
ENSG00000196208	138.1756139	1.080229524	7.002606683	5.00E-12	7.07E-11	GREB1
ENSG00000131653	708.1474854	2245.878519	-1.665074001	5.03E-12	7.12E-11	TRAF7
ENSG00000129083	1706.773331	4388.265749	-1.362399474	5.08E-12	7.18E-11	COPB1
ENSG00000267272	167.0534135	15.84574797	3.400066145	5.33E-12	7.53E-11	LINC01140
ENSG00000189057	14.024232	247.4906294	-4.137123425	5.41E-12	7.63E-11	FAM111B
ENSG00000166035	176.4902568	0.336469917	8.835128051	5.41E-12	7.63E-11	LIPC
ENSG00000142065	325.7165536	61.4171064	2.410330403	5.46E-12	7.69E-11	ZFP14
novel.427	4.529633624	132.3806185	-4.866085158	5.47E-12	7.70E-11	-
ENSG00000152926	1044.851184	279.4319319	1.90374595	5.60E-12	7.89E-11	ZNF117
ENSG00000115596	191.0474189	2.742510011	6.088419618	5.63E-12	7.93E-11	WNT6
ENSG00000134901	131.9078475	765.9438383	-2.538315127	5.65E-12	7.94E-11	POGLUT2
ENSG00000176171	745.0780527	6754.785614	-3.180426523	5.65E-12	7.95E-11	BNIP3
ENSG00000271533	338.5020626	64.32402199	2.394734219	5.67E-12	7.97E-11	Z83843.1
ENSG00000134716	323.0953219	22.24569285	3.860201886	5.75E-12	8.08E-11	CYP2J2
ENSG00000147437	246.3559714	45.69562582	2.42899366	5.80E-12	8.13E-11	GNRH1
ENSG00000011478	139.613384	642.9353657	-2.202545839	5.82E-12	8.16E-11	QPCTL
ENSG00000058404	474.6537512	17.31191382	4.783075355	5.95E-12	8.34E-11	CAMK2B
ENSG00000083097	635.3174123	127.8985522	2.311100645	5.99E-12	8.40E-11	DOP1A
ENSG00000186235	168.2872644	9.608667603	4.145736641	6.01E-12	8.42E-11	LINC02610
ENSG00000146143	118.676053	473.8371491	-1.998253244	6.02E-12	8.43E-11	PRIM2
ENSG00000173540	265.2443333	1005.636202	-1.922949223	6.05E-12	8.47E-11	GMPPB
ENSG00000112249	606.8670502	2054.889125	-1.759817228	6.08E-12	8.50E-11	ASCC3
ENSG00000204899	346.944859	1089.00784	-1.650629825	6.12E-12	8.55E-11	MZT1
ENSG00000101412	106.9411632	646.2304262	-2.594520616	6.12E-12	8.55E-11	E2F1
ENSG00000145014	148.4167629	812.5282375	-2.452886641	6.18E-12	8.62E-11	TMEM44
ENSG00000160226	564.527927	165.6128713	1.76959309	6.19E-12	8.64E-11	CFAP410
ENSG00000164136	189.8261483	52.6066735	1.849965328	6.20E-12	8.65E-11	IL15
ENSG00000123179	203.3125344	1015.458565	-2.320793843	6.25E-12	8.71E-11	EBPL
ENSG00000072210	2359.026859	850.2495041	1.472352706	6.35E-12	8.84E-11	ALDH3A2
ENSG00000175302	109.1238302	0	9.103879156	6.40E-12	8.91E-11	ANKRD30BP1
ENSG00000260260	139.2249364	417.7371767	-1.585346721	6.46E-12	8.99E-11	SNHG19
ENSG00000129173	1.7871679	96.9674645	-5.747408594	6.60E-12	9.18E-11	E2F8
ENSG00000163638	1781.638722	95.13965692	4.229065935	6.69E-12	9.30E-11	ADAMTS9
ENSG00000184840	2222.300615	7264.159632	-1.708757395	6.78E-12	9.41E-11	TMED9
ENSG00000166813	248.5493793	861.8725086	-1.794262619	6.91E-12	9.59E-11	KIF7
ENSG00000140950	193.3207484	1100.984894	-2.509758586	7.09E-12	9.84E-11	MEAK7
ENSG00000165457	100.0455719	0	8.977859517	7.14E-12	9.91E-11	FOLR2
ENSG00000165178	136.5326879	0.713078025	7.571749762	7.27E-12	1.01E-10	NCF1C
ENSG00000229520	133.740476	0	9.397233469	7.30E-12	1.01E-10	LINC00404
ENSG00000155307	372.2564417	25.69973983	3.865577004	7.34E-12	1.02E-10	SAMSN1
ENSG00000073150	17.91723763	121.2807541	-2.759783123	7.42E-12	1.03E-10	PANX2
ENSG00000107771	1866.469584	621.0166511	1.587386197	7.49E-12	1.04E-10	CCSER2
ENSG00000166405	99.65638485	0	8.973094935	7.50E-12	1.04E-10	RIC3
ENSG00000143669	1286.990663	323.406307	1.993284935	7.53E-12	1.04E-10	LYST
novel.830	0.879722543	136.4724411	-7.242832246	7.75E-12	1.07E-10	-
ENSG00000186335	176.4197956	0	9.796433111	7.76E-12	1.07E-10	SLC36A2
ENSG00000153563	272.5341388	1.029478846	8.003791525	7.78E-12	1.07E-10	CD8A
ENSG00000100485	1179.977401	456.6831252	1.369013235	7.87E-12	1.09E-10	SOS2
ENSG00000115361	125.0122997	0	9.299376237	7.88E-12	1.09E-10	ACADL
ENSG00000035403	2850.306142	6680.475645	-1.228852115	8.07E-12	1.11E-10	VCL

ENSG00000143621	1369.507243	4153.11597	-1.600645312	8.09E-12	1.11E-10	ILF2
ENSG00000197157	1975.631929	7040.124386	-1.833293641	8.10E-12	1.11E-10	SND1
novel.439	145.9159253	2.221822211	6.065270886	8.31E-12	1.14E-10	-
ENSG00000106348	559.2321666	1879.494741	-1.748845156	8.48E-12	1.17E-10	IMPDH1
ENSG00000130204	831.8616149	2338.729963	-1.49141885	8.51E-12	1.17E-10	TOMM40
ENSG00000253877	75.90269382	0	8.5799621	8.52E-12	1.17E-10	LINC01608
ENSG00000134684	744.3684708	2547.56967	-1.775195381	8.67E-12	1.19E-10	YARS
ENSG00000173406	220.8903325	0.743759607	8.256717553	8.74E-12	1.20E-10	DAB1
ENSG00000128510	7.08717119	424.2322613	-5.908953579	8.79E-12	1.20E-10	CPA4
ENSG00000174437	2430.559965	7623.991402	-1.649274643	8.81E-12	1.21E-10	ATP2A2
ENSG00000197558	152.1113075	10.81303612	3.798875666	8.86E-12	1.21E-10	SSPO
ENSG00000169989	101.102602	2.802717299	5.151011942	8.87E-12	1.21E-10	TIGD4
ENSG00000196547	2960.621485	430.3984619	2.782135988	9.10E-12	1.25E-10	MAN2A2
ENSG00000138468	765.6287623	162.8434853	2.233166399	9.16E-12	1.25E-10	SENP7
ENSG00000115085	149.1599076	0	9.554218255	9.26E-12	1.27E-10	ZAP70
ENSG00000013725	138.1223477	0	9.44326803	9.35E-12	1.28E-10	CD6
ENSG00000138757	1154.056169	3698.759884	-1.680410862	9.45E-12	1.29E-10	G3BP2
ENSG00000187122	153.2365613	2.058957692	6.17827526	9.48E-12	1.29E-10	SLIT1
ENSG00000168546	301.7052359	3.404837359	6.43942648	9.60E-12	1.31E-10	GFRA2
ENSG00000145794	311.2927771	35.49904666	3.126970999	9.67E-12	1.32E-10	MEGF10
ENSG00000196368	28.09813959	223.1829779	-2.992517626	1.00E-11	1.36E-10	NUDT11
ENSG00000205189	569.2729599	228.3890384	1.317802271	1.01E-11	1.37E-10	ZBTB10
ENSG00000213190	260.9481214	751.2270159	-1.525580531	1.03E-11	1.41E-10	MLLT11
ENSG00000079950	2565.495489	1063.409962	1.270747125	1.08E-11	1.46E-10	STX7
ENSG00000186193	9.925128383	287.5482725	-4.855094923	1.08E-11	1.47E-10	SAPCD2
ENSG00000196262	11261.83224	23871.67553	-1.083857675	1.08E-11	1.47E-10	PPIA
ENSG00000242372	733.902063	2045.507795	-1.478913436	1.08E-11	1.47E-10	EIF6
ENSG00000129353	7941.59419	2507.807725	1.663006524	1.08E-11	1.47E-10	SLC44A2
ENSG00000101955	295.9303692	3817.349695	-3.689329442	1.09E-11	1.48E-10	SRPX
ENSG00000100075	743.8965422	2215.456722	-1.574261911	1.09E-11	1.48E-10	SLC25A1
ENSG00000049449	1176.588103	4315.894395	-1.875097328	1.13E-11	1.53E-10	RCN1
ENSG00000179115	785.994581	2305.348237	-1.552533514	1.13E-11	1.53E-10	FARSA
ENSG00000133116	133.60934	0.387220594	8.433577942	1.13E-11	1.53E-10	KL
ENSG00000149925	77.00001066	435.1172477	-2.497425571	1.16E-11	1.56E-10	ALDOA
ENSG00000214652	106.0684206	1.00940975	6.63611353	1.17E-11	1.59E-10	ZNF727
ENSG00000075188	209.3868539	657.5380449	-1.651228921	1.20E-11	1.62E-10	NUP37
ENSG00000186871	10.75198386	262.0370916	-4.602750131	1.23E-11	1.66E-10	ERCC6L
ENSG00000167701	121.5833607	6.980711577	4.13850025	1.23E-11	1.66E-10	GPT
ENSG00000164442	986.8660391	7004.832534	-2.827467127	1.25E-11	1.69E-10	CITED2
ENSG00000184916	275.3510561	36.99918614	2.901556822	1.27E-11	1.72E-10	JAG2
ENSG00000109944	168.7362706	23.53367698	2.83906968	1.30E-11	1.75E-10	JHY
ENSG00000128849	455.7982234	4.892356573	6.530733818	1.30E-11	1.75E-10	CGNL1
ENSG00000105483	1182.071512	419.151774	1.495957459	1.31E-11	1.77E-10	CARD8
ENSG00000132640	1603.050814	407.0848686	1.977042655	1.32E-11	1.77E-10	BTBD3
ENSG00000154783	354.8196489	4.474454397	6.289261902	1.32E-11	1.78E-10	FGD5
ENSG00000150672	83.29564234	7.326638104	3.523612695	1.34E-11	1.80E-10	DLG2
ENSG00000082269	502.1721508	113.6318074	2.145376347	1.35E-11	1.81E-10	FAM135A
ENSG00000102032	888.0096456	83.87590087	3.407181833	1.36E-11	1.83E-10	RENBP
ENSG00000287642	135.0959308	0.356539013	8.450084474	1.37E-11	1.84E-10	AC093599.2
ENSG00000173890	95.97258181	6.510902848	3.887026149	1.37E-11	1.84E-10	GPR160
ENSG00000176788	453.9188417	4614.234918	-3.345743205	1.38E-11	1.84E-10	BASP1

ENSG00000122378	1722.584286	336.7206516	2.35463753	1.39E-11	1.86E-10	PRXL2A
ENSG00000082684	85.25946106	0	8.747124455	1.41E-11	1.89E-10	SEMA5B
ENSG00000136270	685.4677348	1940.26532	-1.501187008	1.43E-11	1.91E-10	TBRG4
ENSG00000188486	686.8852598	2752.798839	-2.002875686	1.43E-11	1.91E-10	H2AFX
ENSG00000135472	152.0688538	6.03394926	4.648822724	1.43E-11	1.91E-10	FAIM2
ENSG00000165704	324.0262726	942.6909585	-1.540901535	1.44E-11	1.93E-10	HPRT1
ENSG00000089177	689.388207	193.9886678	1.828111013	1.45E-11	1.93E-10	KIF16B
ENSG00000182050	97.92293856	1.161661783	6.491261461	1.45E-11	1.94E-10	MGAT4C
ENSG00000029639	166.8093063	517.0933092	-1.632861226	1.46E-11	1.94E-10	TFB1M
ENSG00000136868	462.6866366	1576.008842	-1.768391592	1.46E-11	1.94E-10	SLC31A1
ENSG00000181908	92.01843178	0	8.857233938	1.51E-11	2.02E-10	AP003774.1
ENSG00000131781	93.93959825	11.03610792	3.083260535	1.51E-11	2.02E-10	FMO5
ENSG00000231312	497.8854966	182.6593918	1.446532928	1.52E-11	2.02E-10	MAP4K3-DT
ENSG00000135298	371.7248654	9.683211791	5.250349317	1.52E-11	2.02E-10	ADGRB3
ENSG00000050820	151.4769021	451.8464339	-1.576098191	1.53E-11	2.03E-10	BCAR1
ENSG00000175895	500.9955252	167.3426323	1.581580333	1.54E-11	2.04E-10	PLEKHF2
ENSG00000138380	393.3931466	104.3348359	1.91557537	1.54E-11	2.05E-10	CARF
ENSG00000105737	353.760003	9.225171423	5.244075635	1.55E-11	2.06E-10	GRIK5
ENSG00000269378	532.2286238	2279.977719	-2.098791207	1.56E-11	2.08E-10	AC022149.1
ENSG00000112640	335.4173483	962.7416322	-1.521142748	1.57E-11	2.08E-10	PPP2R5D
ENSG00000243069	204.0213914	0.356539013	9.044808776	1.59E-11	2.11E-10	ARHGEF26-AS1
ENSG00000279821	13.95144876	106.3186543	-2.932641951	1.60E-11	2.12E-10	AC145098.2
ENSG00000164309	336.2241743	21.38164706	3.968499288	1.60E-11	2.12E-10	CMYA5
ENSG00000138119	1933.829338	7988.503651	-2.046526561	1.63E-11	2.16E-10	MYOF
ENSG00000006634	86.74447038	396.8967658	-2.194853713	1.64E-11	2.17E-10	DBF4
ENSG00000126822	2185.185033	294.2965712	2.891661974	1.66E-11	2.20E-10	PLEKHG3
ENSG00000119969	92.44746649	605.465497	-2.710952409	1.68E-11	2.23E-10	HELLS
ENSG00000280852	160.0523019	20.36021216	2.980657127	1.68E-11	2.23E-10	AC025048.6
ENSG00000125968	527.6309913	6868.088082	-3.702414028	1.69E-11	2.23E-10	ID1
ENSG00000183091	149.458065	21.09451516	2.831652756	1.70E-11	2.25E-10	NEB
ENSG00000130147	344.0762994	3278.326911	-3.252383378	1.72E-11	2.27E-10	SH3BP4
ENSG00000156515	1965.169715	6786.990877	-1.788061722	1.74E-11	2.30E-10	HK1
ENSG00000166598	7195.103641	45458.2656	-2.659458644	1.75E-11	2.31E-10	HSP90B1
ENSG00000169562	210.6073263	0	10.05201006	1.75E-11	2.31E-10	GJB1
ENSG00000110811	487.1347077	3822.582549	-2.972272034	1.79E-11	2.36E-10	P3H3
ENSG00000277954	72.24526084	0	8.508749737	1.79E-11	2.36E-10	AC092376.2
ENSG00000165810	80.94713734	0	8.672837252	1.80E-11	2.37E-10	BTNL9
ENSG00000086598	2505.891721	6213.974028	-1.310231893	1.83E-11	2.41E-10	TMED2
ENSG00000240476	0.293240848	329.7829598	-9.984413603	1.85E-11	2.43E-10	LINC00973
ENSG00000146410	10.60487898	130.8465522	-3.62668866	1.85E-11	2.43E-10	MTFR2
ENSG00000126500	230.6284673	23.42742373	3.301013551	1.87E-11	2.45E-10	FLRT1
ENSG00000131788	655.2200219	1528.744265	-1.222173654	1.87E-11	2.46E-10	PIAS3
ENSG00000138606	217.5284335	20.84777824	3.390090305	1.88E-11	2.46E-10	SHF
ENSG00000181997	87.10595024	0	8.77893996	1.89E-11	2.48E-10	AQP7P2
ENSG00000287706	97.92942062	0	8.947492849	1.89E-11	2.48E-10	AC092924.2
ENSG00000213626	695.6070518	111.2018111	2.64266691	1.90E-11	2.48E-10	LBH
ENSG00000143321	3014.220776	9662.25141	-1.680551496	1.91E-11	2.50E-10	HDGF
ENSG00000172590	584.9041184	1444.813341	-1.304669222	1.92E-11	2.51E-10	MRPL52
ENSG00000279561	83.90329388	9.233343763	3.175930576	1.93E-11	2.53E-10	AL845472.2
ENSG00000272106	257.8446183	76.90186743	1.744315672	1.95E-11	2.55E-10	AL691432.2
ENSG00000198900	1074.105744	3207.534938	-1.578389824	1.99E-11	2.60E-10	TOP1

ENSG00000150093	7550.633346	38427.11254	-2.347452675	2.00E-11	2.61E-10	ITGB1
ENSG00000107651	592.8817533	1556.090779	-1.392147652	2.02E-11	2.64E-10	SEC23IP
ENSG00000131475	527.6446827	1868.056086	-1.824096952	2.06E-11	2.69E-10	VPS25
ENSG00000171903	131.0087233	1.069617038	6.928523367	2.06E-11	2.69E-10	CYP4F11
ENSG00000120889	1402.088162	4224.09116	-1.591035862	2.07E-11	2.70E-10	TNFRSF10B
ENSG00000119285	385.6285465	1191.596219	-1.627705801	2.12E-11	2.76E-10	HEATR1
ENSG00000145817	739.0684946	2424.60181	-1.714162265	2.16E-11	2.82E-10	YIPF5
ENSG00000185291	217.8307777	30.45199791	2.834113708	2.17E-11	2.82E-10	IL3RA
ENSG00000186047	112.6029162	0	9.148674827	2.17E-11	2.82E-10	DLEU7
ENSG00000138032	642.1798786	251.4232525	1.352997875	2.17E-11	2.82E-10	PPM1B
ENSG00000166226	1463.700223	4652.962958	-1.668610959	2.20E-11	2.85E-10	CCT2
ENSG00000131203	106.9287971	6.777708867	3.975468766	2.21E-11	2.87E-10	IDO1
ENSG00000136286	148.987445	1.762625967	6.38388376	2.23E-11	2.90E-10	MYO1G
ENSG00000186074	106.3242651	0	9.065838055	2.24E-11	2.91E-10	CD300LF
ENSG00000165140	99.1971906	0	8.965618194	2.24E-11	2.91E-10	FBP1
ENSG00000111481	1362.237552	3184.540746	-1.225111744	2.32E-11	3.00E-10	COPZ1
ENSG00000136875	380.8949227	1202.353381	-1.658753835	2.34E-11	3.03E-10	PRPF4
ENSG00000055732	218.3839224	6.032793384	5.171901182	2.35E-11	3.04E-10	MCOLN3
ENSG00000128923	1950.972284	518.9878009	1.910544922	2.35E-11	3.04E-10	MINDY2
ENSG00000250722	771.8530473	21.64973735	5.148841281	2.38E-11	3.07E-10	SELENOP
ENSG00000272523	152.6684567	17.62027069	3.10631603	2.38E-11	3.07E-10	LINC01023
ENSG00000173714	116.1558127	0	9.194078919	2.38E-11	3.08E-10	WFIKKN2
ENSG00000115233	694.3082094	2060.757318	-1.569683717	2.38E-11	3.08E-10	PSMD14
ENSG00000100351	278.9975195	6.460023776	5.4571653	2.40E-11	3.10E-10	GRAP2
ENSG00000185386	155.5931573	561.1546863	-1.851341527	2.43E-11	3.14E-10	MAPK11
ENSG00000148180	27720.42689	4213.060264	2.718013089	2.46E-11	3.17E-10	GSN
ENSG00000100344	0.61420451	160.0256136	-8.041796567	2.47E-11	3.19E-10	PNPLA3
ENSG00000120992	434.641507	1516.063912	-1.802490163	2.49E-11	3.21E-10	LYPLA1
ENSG00000197977	250.489006	5.075290187	5.630163572	2.49E-11	3.21E-10	ELOVL2
ENSG00000254470	241.2446847	695.6396058	-1.527735361	2.50E-11	3.22E-10	AP5B1
ENSG00000179271	996.1621619	2500.710512	-1.327952615	2.57E-11	3.31E-10	GADD45GIP1
ENSG00000128039	199.3236096	655.6863626	-1.718079596	2.60E-11	3.35E-10	SRD5A3
ENSG00000146094	334.6888911	87.3260135	1.941100362	2.61E-11	3.35E-10	DOK3
ENSG00000175602	1247.730834	4172.111024	-1.74147347	2.65E-11	3.41E-10	CCDC85B
ENSG00000111275	2111.617979	509.3973365	2.051494683	2.65E-11	3.41E-10	ALDH2
ENSG00000163235	512.4242643	9.189609551	5.807538063	2.65E-11	3.41E-10	TGFA
ENSG00000036672	147.3828565	0.356539013	8.575758047	2.66E-11	3.41E-10	USP2
ENSG00000138379	83.84820425	1.161661783	6.269775224	2.68E-11	3.43E-10	MSTN
ENSG00000151422	1760.190655	388.199975	2.180748238	2.69E-11	3.44E-10	FER
ENSG00000100412	771.2885333	1684.307205	-1.126817727	2.69E-11	3.45E-10	ACO2
ENSG00000153898	104.2358362	7.275887426	3.849921983	2.69E-11	3.45E-10	MCOLN2
ENSG00000062485	1390.626497	3545.764466	-1.350460879	2.70E-11	3.45E-10	CS
ENSG00000107863	3731.895804	1440.735807	1.373054761	2.71E-11	3.46E-10	ARHGAP21
ENSG00000196182	1291.174145	521.7059355	1.306917219	2.75E-11	3.52E-10	STK40
ENSG00000223749	93.44950567	498.6807803	-2.414767898	2.78E-11	3.55E-10	MIR503HG
ENSG00000025039	305.1093735	15.99684412	4.256967288	2.81E-11	3.59E-10	RRAGD
ENSG00000152315	119.6048817	0.356539013	8.27383586	2.83E-11	3.61E-10	KCNK13
ENSG00000173402	7997.271614	2361.074588	1.759921832	2.83E-11	3.61E-10	DAG1
ENSG00000116985	3756.86541	2.852312101	10.36055808	2.84E-11	3.62E-10	BMP8B
ENSG00000134207	75.84062394	0	8.579142127	2.85E-11	3.63E-10	SYT6
ENSG00000280061	88.02238496	0	8.79397486	2.87E-11	3.66E-10	AC011504.1

novel.911	2503.485033	0	13.62341675	2.88E-11	3.67E-10	-
ENSG00000117425	169.6028445	12.78097654	3.725177226	2.89E-11	3.68E-10	PTCH2
ENSG00000139618	59.9442603	298.5443921	-2.317753616	2.90E-11	3.69E-10	BRCA2
ENSG00000123064	713.620308	1967.835258	-1.463569166	2.90E-11	3.69E-10	DDX54
ENSG00000154760	342.7236678	74.090721	2.210503163	2.92E-11	3.72E-10	SLFN13
ENSG00000187210	134.1319287	578.4397239	-2.108297179	2.94E-11	3.74E-10	GCNT1
ENSG00000182487	83.89208196	1.436768536	5.868424439	2.97E-11	3.78E-10	NCF1B
ENSG00000112294	484.8323037	40.31827171	3.583735245	3.00E-11	3.81E-10	ALDH5A1
ENSG00000158445	222.640159	0.672939833	8.294394447	3.02E-11	3.83E-10	KCNB1
ENSG00000198399	1206.733076	469.6477117	1.361537504	3.03E-11	3.84E-10	ITSN2
ENSG00000152818	4369.281594	934.6988024	2.22457817	3.06E-11	3.88E-10	UTRN
ENSG00000236404	122.2938674	5.371621912	4.50751206	3.09E-11	3.92E-10	VLDLR-AS1
ENSG00000168309	267.4249463	2.069570178	6.985863269	3.10E-11	3.93E-10	FAM107A
ENSG00000214826	46.60637532	247.9787906	-2.410090476	3.15E-11	3.99E-10	DDX12P
ENSG00000121417	492.5317717	173.7504928	1.504252958	3.16E-11	4.00E-10	ZNF211
ENSG00000273203	97.004752	4.748276881	4.366699594	3.18E-11	4.02E-10	AC006946.2
ENSG00000181513	496.7408411	162.6448493	1.611492521	3.20E-11	4.05E-10	ACBD4
ENSG00000185721	508.2575241	1343.080065	-1.402051892	3.26E-11	4.11E-10	DRG1
ENSG00000099341	1605.463107	4359.395336	-1.44114416	3.35E-11	4.23E-10	PSMD8
ENSG00000168621	2.962638588	514.2027817	-7.430170977	3.37E-11	4.25E-10	GDNF
ENSG00000261087	279.6604894	14.25531473	4.308446688	3.37E-11	4.25E-10	AP003469.4
ENSG00000110955	5862.692758	16618.84082	-1.503207321	3.38E-11	4.26E-10	ATP5F1B
novel.25	69.49136974	1.386017859	5.604629204	3.38E-11	4.26E-10	-
ENSG00000171224	112.375834	10.54854185	3.400153941	3.38E-11	4.26E-10	FAM241B
ENSG00000136895	175.8218325	27.84025817	2.667598376	3.39E-11	4.27E-10	GARNL3
ENSG00000214212	138.0247484	6.247564456	4.480137626	3.47E-11	4.37E-10	C19orf38
ENSG00000149557	3348.520451	1026.255699	1.706474631	3.51E-11	4.42E-10	FEZ1
ENSG00000155962	244.7164612	4.136700211	5.911487455	3.51E-11	4.42E-10	CLIC2
ENSG00000143344	1524.980416	310.3535286	2.296589161	3.61E-11	4.53E-10	RGL1
ENSG00000279207	299.4466675	56.82968626	2.395045858	3.64E-11	4.57E-10	AC015813.6
ENSG00000175318	124.746612	10.48833456	3.557358825	3.65E-11	4.58E-10	GRAMD2A
ENSG00000168824	465.3568876	3.52640781	7.035896557	3.66E-11	4.59E-10	NSG1
ENSG00000160883	83.04835957	0	8.709994949	3.89E-11	4.88E-10	HK3
ENSG00000122042	1946.626759	565.4722052	1.783723225	3.90E-11	4.89E-10	UBL3
ENSG00000122729	726.2942406	3765.735615	-2.374277578	3.98E-11	4.98E-10	ACO1
ENSG00000187808	81.71639555	1.049547942	6.249763379	4.03E-11	5.04E-10	SOWAHD
ENSG00000171889	0.615458159	182.3697257	-8.229062297	4.06E-11	5.08E-10	MIR31HG
ENSG00000149418	96.29254838	2.089639274	5.496994099	4.06E-11	5.08E-10	ST14
ENSG00000090006	11715.47717	1845.41239	2.666551842	4.14E-11	5.17E-10	LTBP4
ENSG00000131746	0.600343103	106.5302961	-7.45879814	4.20E-11	5.24E-10	TNS4
ENSG00000100605	601.9118748	45.66627569	3.716263631	4.29E-11	5.36E-10	ITPK1
ENSG00000101255	113.9733435	921.0084252	-3.01511642	4.45E-11	5.55E-10	TRIB3
ENSG00000122884	694.3317287	4011.940876	-2.530654201	4.48E-11	5.59E-10	P4HA1
ENSG00000101407	243.5714504	656.6312796	-1.430778097	4.52E-11	5.64E-10	TTI1
ENSG00000283787	100.8940245	1.130980201	6.537202839	4.57E-11	5.69E-10	PRR33
ENSG00000132819	467.2291787	174.7068873	1.419215081	4.59E-11	5.72E-10	RBM38
ENSG00000196365	1338.856695	3453.165037	-1.366866097	4.60E-11	5.73E-10	LONP1
ENSG00000155016	1381.916562	265.676174	2.378322915	4.63E-11	5.76E-10	CYP2U1
ENSG00000180901	1318.642254	524.3808366	1.330064267	4.66E-11	5.80E-10	KCTD2
ENSG00000166741	250.2197705	5412.209608	-4.43512356	4.68E-11	5.82E-10	NNMT
ENSG00000072954	132.3144869	11.36312123	3.53526012	4.73E-11	5.88E-10	TMEM38A

ENSG00000183010	152.884212	1363.172024	-3.156988241	4.76E-11	5.92E-10	PYCR1
ENSG00000152766	125.7954638	6.297159258	4.340250185	4.93E-11	6.12E-10	ANKRD22
ENSG00000188133	129.646015	0	9.351912482	4.93E-11	6.12E-10	TMEM215
ENSG00000164880	1495.185205	4015.1442	-1.425079446	4.94E-11	6.12E-10	INTS1
ENSG00000165028	126.2435445	16.17404554	2.95308424	4.95E-11	6.13E-10	NIPSNAP3B
ENSG00000085563	174.4309752	3.11911812	5.781415977	4.95E-11	6.13E-10	ABCB1
ENSG00000286169	38.7471101	758.3416708	-4.291255917	4.95E-11	6.13E-10	AHRR
ENSG00000129757	156.3178971	10.22037267	3.925340726	5.00E-11	6.19E-10	CDKN1C
ENSG00000163655	784.0815924	1864.651587	-1.24989474	5.02E-11	6.21E-10	GMPS
ENSG00000278730	529.7651102	104.3637665	2.340941464	5.15E-11	6.37E-10	AC005332.6
ENSG00000112319	2.763920295	206.8287301	-6.233624686	5.17E-11	6.39E-10	EYA4
ENSG00000184481	1082.925268	158.4375393	2.774591711	5.21E-11	6.44E-10	FOXO4
ENSG00000172348	262.4413914	9.438786619	4.787294721	5.23E-11	6.46E-10	RCAN2
ENSG00000105974	606.4142779	11103.92141	-4.194720987	5.40E-11	6.67E-10	CAV1
ENSG00000125651	1129.908335	2630.578379	-1.219106852	5.41E-11	6.67E-10	GTF2F1
ENSG00000003400	227.6403125	20.93421918	3.433142078	5.49E-11	6.77E-10	CASP10
ENSG00000130635	6901.286633	21637.07342	-1.648571088	5.55E-11	6.84E-10	COL5A1
ENSG00000184669	118.1688645	0	9.218167222	5.60E-11	6.90E-10	OR7E14P
ENSG00000110917	2144.718897	5503.143239	-1.359478786	5.61E-11	6.91E-10	MLEC
ENSG00000083520	613.5498484	1351.519048	-1.139194758	5.64E-11	6.94E-10	DIS3
ENSG00000256268	0.616711807	99.18487314	-7.352181622	5.72E-11	7.04E-10	LINC02454
ENSG00000149782	525.5821258	1463.254364	-1.476974771	5.81E-11	7.15E-10	PLCB3
ENSG00000116871	2689.33521	5812.910085	-1.112012885	5.83E-11	7.17E-10	MAP7D1
ENSG00000185418	857.5655922	254.1960118	1.75525115	5.85E-11	7.19E-10	TARSL2
ENSG00000169710	2222.083	5768.722478	-1.376326312	5.97E-11	7.34E-10	FASN
ENSG00000164463	745.751534	155.1844401	2.266041179	5.98E-11	7.34E-10	CREBRF
ENSG00000166501	139.587583	0	9.458527829	6.01E-11	7.38E-10	PRKCB
ENSG00000154655	84.85275073	1.548882377	5.861340868	6.13E-11	7.52E-10	L3MBTL4
ENSG00000129158	344.2730249	95.21720201	1.854435416	6.17E-11	7.56E-10	SERGEF
ENSG00000173456	344.2801904	1194.410881	-1.794899954	6.21E-11	7.61E-10	RNF26
ENSG00000264456	68.25411391	4.391737868	3.976511077	6.23E-11	7.63E-10	AC138207.4
ENSG00000280339	101.7680914	11.07740199	3.196344904	6.28E-11	7.69E-10	AP001528.3
ENSG00000183742	76.39841391	2.731897526	4.757086132	6.29E-11	7.69E-10	MACC1
ENSG00000204147	40.29988772	178.6420787	-2.148898961	6.30E-11	7.71E-10	ASAH2B
ENSG00000113749	128.3688986	1.365948763	6.503486949	6.32E-11	7.73E-10	HRH2
ENSG00000146006	80.62101655	4.992702053	4.00770548	6.44E-11	7.87E-10	LRRTM2
ENSG00000215861	21.90168905	199.6161796	-3.184520179	6.48E-11	7.92E-10	AC245297.1
ENSG00000096060	596.4093463	4793.669855	-3.006855592	6.49E-11	7.93E-10	FKBP5
ENSG00000113361	1413.917196	24.15496705	5.866891166	6.50E-11	7.93E-10	CDH6
ENSG00000178033	1328.925185	313.7376205	2.08190366	6.63E-11	8.09E-10	CALHM5
ENSG00000286699	107.9747571	10.97705651	3.290253732	6.71E-11	8.18E-10	AC084198.2
ENSG00000278058	63.41243471	0	8.320737283	6.72E-11	8.19E-10	AC009159.3
ENSG00000186908	1540.712018	542.4339755	1.506205293	6.74E-11	8.21E-10	ZDHHC17
ENSG00000237928	104.5872007	0	9.041987676	6.76E-11	8.23E-10	NFIA-AS2
ENSG00000122012	144.8407024	0	9.511838806	6.81E-11	8.29E-10	SV2C
ENSG00000173200	116.3501133	5.606590473	4.359440865	6.88E-11	8.36E-10	PARP15
ENSG00000123560	28153.74409	34.40961732	9.675030326	6.91E-11	8.40E-10	PLP1
ENSG00000243335	653.4212434	243.6458473	1.422948684	6.96E-11	8.46E-10	KCTD7
ENSG00000064419	759.1379716	1642.217493	-1.113204064	7.09E-11	8.62E-10	TNPO3
ENSG00000110422	2163.404785	604.9643172	1.837873217	7.22E-11	8.76E-10	HIPK3
ENSG00000176907	669.0391836	103.9613915	2.688069629	7.28E-11	8.83E-10	TCIM

ENSG00000121989	487.3413908	110.5935264	2.13790486	7.32E-11	8.87E-10	ACVR2A
ENSG00000178235	113.0061225	0.336469917	8.19215257	7.37E-11	8.93E-10	SLITRK1
ENSG00000178722	87.99598706	0	8.793481373	7.43E-11	9.00E-10	C5orf64
ENSG00000163701	267.7409562	43.42305293	2.622163499	7.43E-11	9.00E-10	IL17RE
ENSG00000214706	356.4829791	1381.622812	-1.954711685	7.55E-11	9.14E-10	IFRD2
ENSG00000233429	66.9655294	337.5903952	-2.334707985	7.56E-11	9.15E-10	HOTAIRM1
ENSG00000198851	162.1555169	0	9.674782145	7.57E-11	9.16E-10	CD3E
ENSG00000100029	994.1790125	2944.107701	-1.566272775	7.57E-11	9.16E-10	PES1
ENSG00000125648	1365.687747	417.8025555	1.70942476	7.62E-11	9.21E-10	SLC25A23
ENSG00000266964	99.79199343	0	8.975052968	7.69E-11	9.28E-10	FXYD1
ENSG00000231817	67.48381551	0	8.410662024	7.72E-11	9.32E-10	LINC01198
ENSG00000196466	143.5391983	36.15880547	1.987706177	7.75E-11	9.36E-10	ZNF799
ENSG00000176454	329.8419125	936.1433254	-1.504595311	7.76E-11	9.36E-10	LPCAT4
ENSG00000169442	140.2406506	0	9.465267315	7.98E-11	9.62E-10	CD52
ENSG00000162851	125.5918727	397.6114664	-1.663441383	8.02E-11	9.67E-10	TFB2M
ENSG00000158769	287.7859843	81.16946631	1.827825815	8.05E-11	9.70E-10	F11R
ENSG00000166394	2825.476692	145.3566947	4.279662487	8.23E-11	9.90E-10	CYB5R2
ENSG00000106290	314.5677457	796.5267009	-1.340179417	8.31E-11	1.00E-09	TAF6
ENSG00000248429	324.4827458	16.25676207	4.308786001	8.34E-11	1.00E-09	FAM198B-AS1
ENSG00000169087	393.4759156	130.3609467	1.593265625	8.34E-11	1.00E-09	HSPBAP1
ENSG00000187513	117.761228	0.336469917	8.251338161	8.35E-11	1.00E-09	GJA4
ENSG00000205632	111.5678271	0	9.135774295	8.36E-11	1.00E-09	LINC01310
novel.83	0	175.469676	-10.03568556	8.38E-11	1.01E-09	-
ENSG00000152256	101.9619376	778.1542033	-2.931897554	8.39E-11	1.01E-09	PDK1
ENSG00000165072	277.0283997	26.26197848	3.399458291	8.44E-11	1.01E-09	MAMDC2
ENSG00000266709	3.928036872	77.99784652	-4.312115163	8.48E-11	1.02E-09	AC005224.3
ENSG00000139793	2775.215191	747.4672743	1.892235721	8.65E-11	1.04E-09	MBNL2
ENSG00000126264	118.7140185	0.336469917	8.262956698	8.77E-11	1.05E-09	HCST
ENSG00000280304	89.06586582	1.029478846	6.380607403	8.82E-11	1.06E-09	AC104260.2
ENSG00000175538	163.4502709	15.41967345	3.395177479	8.82E-11	1.06E-09	KCNE3
ENSG00000280237	451.0357183	18.72976913	4.594942397	8.84E-11	1.06E-09	MIR4697HG
ENSG00000172269	387.7516784	986.0501144	-1.34658158	9.01E-11	1.08E-09	DPAGT1
ENSG00000146005	70.8744768	1.365948763	5.638262421	9.05E-11	1.08E-09	PSD2
ENSG00000136824	258.4839949	1082.409049	-2.066567855	9.11E-11	1.09E-09	SMC2
ENSG00000152767	237.9458245	1127.380339	-2.244220287	9.12E-11	1.09E-09	FARP1
ENSG00000112562	1725.016163	0	13.08608351	9.15E-11	1.09E-09	SMOC2
ENSG00000136156	15791.56171	4304.129817	1.875319293	9.21E-11	1.10E-09	ITM2B
ENSG00000168004	129.3908269	0.336469917	8.387830621	9.27E-11	1.11E-09	PLAAT5
ENSG00000146386	128.8118945	623.7485152	-2.276178418	9.31E-11	1.11E-09	ABRACL
ENSG00000244119	91.63948736	1.029478846	6.422280013	9.56E-11	1.14E-09	PDCL3P4
ENSG00000116824	150.7600673	0	9.569641781	9.66E-11	1.15E-09	CD2
ENSG00000124006	4675.181423	1428.079415	1.71091867	9.69E-11	1.15E-09	OBSL1
ENSG00000260244	132.7579098	0.336469917	8.424258665	9.83E-11	1.17E-09	AC104083.1
ENSG00000106819	480.8806846	2.476859868	7.593852061	9.89E-11	1.18E-09	ASPN
ENSG00000136518	444.4220812	1095.095015	-1.301276683	9.90E-11	1.18E-09	ACTL6A
ENSG00000151725	86.69067784	633.6938277	-2.869008559	9.92E-11	1.18E-09	CENPU
ENSG00000106462	133.5993543	615.5980973	-2.203604445	9.94E-11	1.18E-09	EZH2
ENSG00000244694	198.9356324	29.65376321	2.74860316	1.03E-10	1.22E-09	PTCHD4
ENSG00000228232	50.15746126	314.7887442	-2.650244962	1.04E-10	1.24E-09	GAPDHP1
ENSG00000007255	267.7895482	65.4193591	2.033770196	1.06E-10	1.26E-09	TRAPPC6A
ENSG00000072682	589.3912698	6750.314646	-3.517637654	1.09E-10	1.29E-09	P4HA2

ENSG00000164877	6896.423995	916.8595024	2.9110284	1.11E-10	1.32E-09	MICALL2
ENSG00000071626	1839.106157	4220.410416	-1.198363396	1.12E-10	1.33E-09	DAZAP1
ENSG00000180425	145.1577358	27.30091394	2.411129411	1.13E-10	1.33E-09	C11orf71
ENSG00000074527	2883.986409	226.400609	3.670254755	1.13E-10	1.33E-09	NTN4
ENSG00000164823	1017.972991	365.4595712	1.477564488	1.13E-10	1.33E-09	OSGIN2
ENSG00000163069	6101.048648	2274.143989	1.423671077	1.13E-10	1.34E-09	SGCB
ENSG00000188283	346.5384052	116.2672123	1.5747103	1.14E-10	1.34E-09	ZNF383
novel.629	161.7526548	27.10967959	2.574743237	1.15E-10	1.36E-09	-
ENSG00000224307	69.47933145	0	8.451765819	1.17E-10	1.38E-09	AL161785.1
ENSG00000127903	71.88167277	1.029478846	6.067992122	1.19E-10	1.41E-09	ZNF835
ENSG00000108518	5895.447165	19556.341	-1.729964124	1.21E-10	1.42E-09	PFN1
ENSG00000103035	376.2001631	1194.753036	-1.667383827	1.22E-10	1.44E-09	PSMD7
ENSG00000145685	2286.323115	993.8012853	1.201826699	1.23E-10	1.45E-09	LHFPL2
ENSG00000101439	22137.53724	5314.003729	2.058567513	1.27E-10	1.50E-09	CST3
ENSG00000106952	133.413556	0.774441189	7.522469073	1.27E-10	1.50E-09	TNFSF8
ENSG00000267121	193.9680895	47.7851367	2.021523115	1.29E-10	1.51E-09	AC008105.3
ENSG00000134697	772.9884161	1866.070212	-1.271550812	1.30E-10	1.53E-09	GNL2
ENSG00000272894	75.75000487	3.52640781	4.409749552	1.32E-10	1.55E-09	AC004982.2
ENSG00000148798	1.200686205	133.4609292	-6.787613258	1.33E-10	1.56E-09	INA
ENSG00000154473	1080.683065	2917.224757	-1.432774995	1.33E-10	1.56E-09	BUB3
ENSG00000187942	163.8487915	14.17760689	3.525793188	1.33E-10	1.57E-09	LDLRAD2
ENSG00000164035	483.7074319	7.603029127	5.979906586	1.34E-10	1.58E-09	EMCN
ENSG00000166923	0	54.70551259	-8.353571899	1.34E-10	1.58E-09	GREM1
ENSG00000130517	1594.736399	456.6737033	1.804826925	1.35E-10	1.58E-09	PGPEP1
ENSG00000196323	1510.885336	344.4265819	2.13357533	1.36E-10	1.59E-09	ZBTB44
ENSG00000102385	8181.323734	226.7189583	5.173805465	1.38E-10	1.62E-09	DRP2
ENSG00000125166	804.3656691	2146.884345	-1.416306771	1.39E-10	1.62E-09	GOT2
ENSG00000148677	9.774262555	7454.368267	-9.575579668	1.39E-10	1.63E-09	ANKRD1
ENSG00000172508	170.5032379	28.1861847	2.604849672	1.40E-10	1.64E-09	CARNS1
ENSG00000101084	206.4853163	656.2961408	-1.668971097	1.40E-10	1.64E-09	RAB5IF
ENSG00000140848	1277.84058	390.5959637	1.709993894	1.41E-10	1.65E-09	CPNE2
ENSG00000235257	293.6223234	48.76044442	2.586411226	1.42E-10	1.66E-09	ITGA9-AS1
ENSG00000178700	283.9494211	99.57838669	1.511084182	1.43E-10	1.67E-09	DHFR2
ENSG00000113494	152.5716129	8.264072205	4.207274346	1.44E-10	1.69E-09	PRLR
ENSG00000074657	2182.165987	900.159572	1.277708256	1.45E-10	1.69E-09	ZNF532
ENSG00000213430	13.60150863	87.8210284	-2.690703352	1.47E-10	1.72E-09	HSPD1P1
ENSG00000257702	347.1155419	48.92459321	2.822785111	1.48E-10	1.73E-09	LBX2-AS1
ENSG00000286214	195.8760631	8.286581446	4.545609327	1.50E-10	1.74E-09	AUXG01000058.1
ENSG00000129993	87.75299092	0	8.788719575	1.51E-10	1.76E-09	CBFA2T3
ENSG00000101347	13286.06473	2166.371717	2.616578457	1.52E-10	1.76E-09	SAMHD1
ENSG00000072501	1003.957163	2686.916234	-1.420230849	1.52E-10	1.76E-09	SMC1A
ENSG00000236283	160.0135253	0.672939833	7.816418394	1.52E-10	1.77E-09	AC019197.1
ENSG00000236345	2.40137241	70.59242623	-4.86852109	1.55E-10	1.80E-09	SCAT8
ENSG00000196961	1593.576865	4870.503623	-1.611824124	1.59E-10	1.85E-09	AP2A1
ENSG00000174684	1906.996596	881.7485626	1.113020176	1.66E-10	1.93E-09	B4GAT1
ENSG00000131620	96.0709084	2.11916498	5.486991517	1.68E-10	1.95E-09	ANO1
ENSG00000163406	130.0415572	7.277171696	4.138943788	1.70E-10	1.97E-09	SLC15A2
ENSG00000119812	762.2699811	2169.251876	-1.508972982	1.71E-10	1.98E-09	FAM98A
ENSG00000099985	250.6300595	1.161661783	7.82122668	1.71E-10	1.99E-09	OSM
ENSG00000153446	614.3627508	5.900610448	6.691664316	1.72E-10	1.99E-09	C16orf89
ENSG00000153132	7.222993233	138.2470797	-4.254424826	1.73E-10	2.01E-09	CLGN

ENSG00000134755	109.6169992	0.336469917	8.147899795	1.74E-10	2.01E-09	DSC2
ENSG00000186847	163.8474791	1.702418679	6.544067047	1.76E-10	2.03E-09	KRT14
ENSG00000127377	173.5846606	0.336469917	8.81160165	1.76E-10	2.04E-09	CRYGN
ENSG00000229809	492.3924735	163.0414453	1.594741769	1.76E-10	2.04E-09	ZNF688
ENSG00000102265	5139.388931	36577.0923	-2.831284323	1.77E-10	2.04E-09	TIMP1
ENSG00000127423	7.59306301	68.95548031	-3.1870153	1.78E-10	2.05E-09	AUNIP
ENSG00000260001	3.620934617	97.1692173	-4.742115125	1.78E-10	2.06E-09	TGFBR3L
ENSG00000161955	203.0501362	30.21831362	2.740842393	1.79E-10	2.07E-09	TNFSF13
ENSG00000261121	127.7810374	0	9.331573585	1.80E-10	2.07E-09	LINC02473
ENSG00000167994	1144.537333	119.1145629	3.264271333	1.81E-10	2.09E-09	RAB3IL1
ENSG00000235927	3.969621095	63.03834961	-3.9967375	1.82E-10	2.10E-09	NEXN-AS1
ENSG00000158290	1370.610103	5052.74307	-1.88218044	1.83E-10	2.11E-09	CUL4B
ENSG00000267221	99.81018525	5.371621912	4.21510032	1.84E-10	2.12E-09	C17orf113
ENSG00000169230	589.4456749	1843.100119	-1.644931145	1.84E-10	2.12E-09	PRELID1
ENSG00000096384	10503.58477	27887.31693	-1.408738535	1.85E-10	2.13E-09	HSP90AB1
ENSG00000173545	434.6779059	1048.774375	-1.270897212	1.87E-10	2.15E-09	ZNF622
ENSG00000145832	95.83983653	0	8.915932077	1.88E-10	2.16E-09	SLC25A48
ENSG00000181019	474.9695427	3426.037907	-2.850788781	1.89E-10	2.17E-09	NQO1
ENSG00000153989	834.0185244	1861.320416	-1.158110391	1.89E-10	2.17E-09	NUS1
novel.120	120.6279203	0.693008929	7.399312688	1.92E-10	2.20E-09	-
ENSG00000139131	172.016041	458.1124921	-1.413498771	1.92E-10	2.20E-09	YARS2
ENSG00000100156	125.3894369	13.21663607	3.24373925	1.93E-10	2.22E-09	SLC16A8
ENSG00000166197	1126.024019	3062.450155	-1.443592609	1.95E-10	2.23E-09	NOLC1
ENSG00000279688	113.849461	0.774441189	7.296778733	1.95E-10	2.23E-09	AQP7P3
ENSG00000147853	2754.412198	1288.063532	1.096489432	1.96E-10	2.24E-09	AK3
ENSG00000138777	491.8854274	1282.539354	-1.382786833	1.96E-10	2.24E-09	PPA2
ENSG00000140254	79.37065476	0	8.644802517	1.96E-10	2.25E-09	DUOXA1
ENSG00000222041	104.7885403	694.8026119	-2.729291516	2.00E-10	2.28E-09	CYTOR
ENSG00000106638	565.1017137	1444.174375	-1.353668285	2.00E-10	2.29E-09	TBL2
ENSG00000126749	370.7082945	1190.830747	-1.683762167	2.08E-10	2.38E-09	EMG1
ENSG00000265743	57.65850901	0	8.182947969	2.09E-10	2.39E-09	AC138207.5
ENSG00000205795	389.4119962	19.7285664	4.306781078	2.10E-10	2.40E-09	CYS1
ENSG00000117682	391.2600412	877.6362167	-1.165537814	2.10E-10	2.40E-09	DHDDS
ENSG00000167680	264.369511	38.05502704	2.791157789	2.12E-10	2.41E-09	SEMA6B
ENSG00000176170	1054.819876	3419.934406	-1.696931616	2.12E-10	2.42E-09	SPHK1
ENSG00000076356	181.6277989	2029.648049	-3.482272219	2.18E-10	2.49E-09	PLXNA2
ENSG00000096996	109.8083378	1.426156051	6.258824396	2.19E-10	2.49E-09	IL12RB1
ENSG00000179818	565.516717	135.9206394	2.055236858	2.21E-10	2.51E-09	PCBP1-AS1
ENSG00000144120	73.56428893	231.7808703	-1.655918561	2.22E-10	2.53E-09	TMEM177
ENSG00000265798	56.66148443	0	8.157945613	2.23E-10	2.53E-09	AC138207.6
ENSG00000150938	1144.264593	12873.88367	-3.492019185	2.23E-10	2.54E-09	CRIM1
ENSG00000278982	54.79254427	0	8.10966624	2.26E-10	2.57E-09	AL139125.1
ENSG00000198739	71.43283624	0	8.49176052	2.27E-10	2.58E-09	LRRTM3
ENSG00000197245	60.72931775	0	8.25779181	2.28E-10	2.59E-09	FAM110D
ENSG00000113212	140.734963	1.753169357	6.307225788	2.32E-10	2.64E-09	PCDHB7
ENSG00000163749	80.912363	2.191140629	5.224596349	2.34E-10	2.65E-09	CCDC158
ENSG00000179222	2500.306605	10008.06297	-2.001015822	2.35E-10	2.67E-09	MAGED1
ENSG00000107862	1047.829862	3056.090354	-1.544160429	2.36E-10	2.67E-09	GBF1
ENSG00000090530	271.9668053	862.2226137	-1.664755704	2.38E-10	2.69E-09	P3H2
ENSG00000183684	622.809115	2120.886106	-1.768000879	2.38E-10	2.70E-09	ALYREF
ENSG00000198796	0.922560414	94.55808517	-6.697957999	2.41E-10	2.73E-09	ALPK2

ENSG00000123977	0.308355904	79.33499309	-7.929246364	2.43E-10	2.75E-09	DAW1
ENSG00000108387	739.7769015	22.40855737	5.045899323	2.46E-10	2.78E-09	SEPT4
ENSG00000107736	295.1579758	24.98563432	3.568160588	2.48E-10	2.81E-09	CDH23
ENSG00000150637	99.33014934	3.781445468	4.681815897	2.48E-10	2.81E-09	CD226
ENSG00000189410	1.188078446	61.94609494	-5.681416128	2.51E-10	2.83E-09	SH2D5
ENSG00000270127	116.288016	3.250145181	5.165598539	2.51E-10	2.84E-09	AC027020.2
ENSG00000113248	156.7081953	4.340987191	5.178231659	2.52E-10	2.84E-09	PCDHB15
novel.134	152.6330142	0	9.587936022	2.56E-10	2.89E-09	-
ENSG00000223552	69.27935951	0	8.447588705	2.57E-10	2.90E-09	AC098613.1
ENSG00000158352	196.1696328	45.65779938	2.100872327	2.60E-10	2.93E-09	SHROOM4
ENSG00000175170	67.82025024	2.435565801	4.767588318	2.63E-10	2.97E-09	FAM182B
ENSG00000270231	197.363837	537.1039047	-1.443857589	2.63E-10	2.97E-09	NBPF8
ENSG00000198879	737.8506571	119.6817162	2.626398312	2.66E-10	3.00E-09	SFMBT2
ENSG00000169372	204.0000631	1164.062722	-2.512759805	2.66E-10	3.00E-09	CRADD
ENSG00000113140	112574.4543	40908.94548	1.460382542	2.67E-10	3.01E-09	SPARC
ENSG00000102302	76.01763076	348.0211126	-2.195039429	2.70E-10	3.03E-09	FGD1
ENSG00000104177	766.1946443	193.9795495	1.982641147	2.70E-10	3.03E-09	MYEF2
ENSG00000198498	192.9729596	517.8475057	-1.424703436	2.72E-10	3.06E-09	TMA16
ENSG00000023734	1143.013381	3554.025211	-1.636744446	2.78E-10	3.12E-09	STRAP
ENSG00000100479	32.77842558	213.3617698	-2.701076151	2.79E-10	3.13E-09	POLE2
ENSG00000076003	283.4686022	1592.556733	-2.490283222	2.80E-10	3.14E-09	MCM6
ENSG00000167528	795.3404641	84.03436432	3.2397176	2.81E-10	3.15E-09	ZNF641
ENSG00000144136	625.1134732	4073.095307	-2.703964235	2.81E-10	3.15E-09	SLC20A1
ENSG00000168502	280.9307239	750.0846182	-1.417324398	2.83E-10	3.17E-09	MTCL1
ENSG00000180644	76.38533543	0	8.588516176	2.85E-10	3.20E-09	PRF1
novel.687	2.738704777	110.3580099	-5.337494206	2.87E-10	3.21E-09	-
ENSG00000131171	3180.104029	1267.866557	1.326712495	2.93E-10	3.28E-09	SH3BGRL
ENSG00000161405	155.8196259	1.060160428	7.184009893	2.98E-10	3.33E-09	IKZF3
ENSG00000124541	467.2062136	1238.229421	-1.406431545	3.01E-10	3.36E-09	RRP36
ENSG00000212864	162.5580835	31.58156545	2.368594331	3.11E-10	3.48E-09	RNF208
ENSG00000183715	100.6663454	0	8.987625393	3.13E-10	3.49E-09	OPCML
ENSG00000125864	6.704493655	88.0995556	-3.719855222	3.15E-10	3.52E-09	BFSP1
ENSG00000224209	60.17758256	0	8.24543957	3.16E-10	3.53E-09	LINC00466
ENSG00000176402	181.8003178	0.336469917	8.877982053	3.16E-10	3.53E-09	GJC3
ENSG00000176124	277.2453981	35.71394613	2.950421723	3.17E-10	3.54E-09	DLEU1
ENSG00000148826	121.4337058	0	9.257807738	3.22E-10	3.59E-09	NKX6-2
ENSG00000172172	322.3433996	897.8765753	-1.478167769	3.23E-10	3.60E-09	MRPL13
ENSG00000241644	743.6587947	8.519109863	6.445116349	3.23E-10	3.60E-09	INMT
novel.1011	1144.044515	0	12.49361882	3.24E-10	3.60E-09	-
ENSG00000146731	2131.853286	6613.417722	-1.633342116	3.24E-10	3.61E-09	CCT6A
ENSG00000153107	354.1863013	920.2496989	-1.377661232	3.25E-10	3.61E-09	ANAPC1
ENSG00000116815	361.6318318	138.5268568	1.384316545	3.25E-10	3.61E-09	CD58
ENSG00000137075	980.053092	384.9307882	1.348038374	3.26E-10	3.62E-09	RNF38
ENSG00000130518	421.6011848	14.80796839	4.833129549	3.26E-10	3.62E-09	IQCN
ENSG00000260314	73.29418323	0	8.528912504	3.27E-10	3.63E-09	MRC1
ENSG00000169282	220.6751297	43.99436301	2.325047796	3.33E-10	3.69E-09	KCNAB1
ENSG00000264707	54.99656189	0	8.114611614	3.34E-10	3.71E-09	L3MBTL4-AS1
ENSG00000143842	951.5908665	303.5336269	1.649134035	3.36E-10	3.72E-09	SOX13
ENSG00000245293	76.76744385	2.618499415	4.912640898	3.38E-10	3.75E-09	AC096564.1
ENSG00000078804	2481.501258	497.9536693	2.31756441	3.40E-10	3.77E-09	TP53INP2
ENSG00000225285	108.9809567	1.069617038	6.66316818	3.43E-10	3.80E-09	LINC01770

ENSG00000178752	2.373649595	70.73072655	-4.878458752	3.43E-10	3.80E-09	ERFE
ENSG00000278175	370.2329769	46.94103161	2.979485735	3.44E-10	3.81E-09	GLIDR
ENSG00000236849	88.70525576	0	8.805159461	3.48E-10	3.85E-09	LINC01474
ENSG00000239922	102.3641315	0	9.010994099	3.49E-10	3.86E-09	AC092958.1
ENSG00000117906	1318.86432	2786.665616	-1.079291682	3.55E-10	3.92E-09	RCN2
ENSG00000197093	699.1407014	88.06010656	2.986185263	3.55E-10	3.92E-09	GAL3ST4
ENSG00000204843	1951.288868	4404.765801	-1.174650626	3.55E-10	3.92E-09	DCTN1
ENSG00000134109	526.0889865	2036.04989	-1.952195669	3.55E-10	3.92E-09	EDEM1
ENSG00000182054	677.8001204	2090.015372	-1.624611814	3.59E-10	3.96E-09	IDH2
ENSG00000149294	7904.323766	270.1148316	4.871534252	3.64E-10	4.01E-09	NCAM1
ENSG00000125246	113.5055323	25.2938628	2.17007126	3.68E-10	4.05E-09	CLYBL
ENSG00000072958	1102.218915	2739.744723	-1.313532625	3.73E-10	4.11E-09	AP1M1
ENSG00000182866	129.901361	0	9.354780709	3.73E-10	4.12E-09	LCK
ENSG00000286646	64.18280437	1.029478846	5.904768569	3.74E-10	4.12E-09	AL121933.1
ENSG00000120324	59.30949887	2.058957692	4.799022686	3.75E-10	4.13E-09	PCDHB10
ENSG00000165626	319.6578629	23.6165219	3.75094916	3.77E-10	4.15E-09	BEND7
ENSG00000137055	455.1978132	1013.883951	-1.155530067	3.78E-10	4.16E-09	PLAA
ENSG00000236901	67.08874368	5.889869568	3.531621535	3.78E-10	4.16E-09	MIR600HG
ENSG00000138650	428.8315272	19.45256056	4.459247282	3.83E-10	4.21E-09	PCDH10
ENSG00000272720	95.92874719	10.45521283	3.20115046	3.92E-10	4.30E-09	AL022322.1
ENSG00000178498	983.6664049	384.2681228	1.356011956	3.93E-10	4.32E-09	DTX3
ENSG00000095139	2408.082351	6317.422976	-1.391464846	3.95E-10	4.33E-09	ARCN1
ENSG00000135604	202.5358252	11.70203129	4.097464109	3.97E-10	4.36E-09	STX11
ENSG00000042753	1109.405991	4279.66865	-1.947741601	4.01E-10	4.39E-09	AP2S1
ENSG00000114654	73.19582846	0	8.527059024	4.01E-10	4.40E-09	EFCC1
ENSG00000255366	77.15213013	1.00940975	6.178105877	4.04E-10	4.42E-09	AC120036.4
ENSG00000118729	92.72916759	0.672939833	7.021234855	4.05E-10	4.43E-09	CASQ2
ENSG00000071127	3901.272669	11076.27351	-1.505490818	4.07E-10	4.45E-09	WDR1
ENSG00000113048	596.196839	1653.06978	-1.471560845	4.09E-10	4.47E-09	MRPS27
ENSG00000165359	592.1462605	146.8819463	2.011214369	4.10E-10	4.49E-09	INTS6L
novel.759	61.00235767	0	8.26434275	4.13E-10	4.52E-09	-
ENSG00000232445	0	45.44289991	-8.087607544	4.19E-10	4.58E-09	AC006329.1
ENSG00000253368	398.7896218	2234.184572	-2.486301334	4.20E-10	4.59E-09	TRNP1
ENSG00000267374	1.83125942	53.8216545	-4.88775646	4.24E-10	4.63E-09	MIR924HG
ENSG00000287342	55.13335285	0	8.119102331	4.36E-10	4.76E-09	AC188616.1
ENSG00000131467	1100.012113	3252.056671	-1.563967533	4.40E-10	4.80E-09	PSME3
ENSG00000247809	392.3019283	64.03573421	2.616659434	4.43E-10	4.83E-09	NR2F2-AS1
ENSG00000234741	1511.641966	3427.661321	-1.181154658	4.44E-10	4.84E-09	GAS5
ENSG00000254416	70.07094245	0	8.465097051	4.44E-10	4.84E-09	AP000924.1
ENSG00000162614	98.91816716	1147.653985	-3.537013728	4.47E-10	4.87E-09	NEXN
ENSG00000163923	78.18210559	340.1509991	-2.122246467	4.55E-10	4.95E-09	RPL39L
ENSG00000100220	747.9202684	2106.620316	-1.494146451	4.57E-10	4.97E-09	RTCB
ENSG00000061273	1899.72202	4586.732771	-1.271602617	4.61E-10	5.02E-09	HDAC7
ENSG00000166171	152.6953528	415.5638406	-1.444474234	4.62E-10	5.02E-09	DPCD
ENSG00000144591	575.794491	1525.023622	-1.405085849	4.68E-10	5.09E-09	GMPPA
ENSG00000171094	124.0468464	3.200550379	5.270698465	4.70E-10	5.11E-09	ALK
ENSG00000231574	0	84.17680891	-8.975159056	4.71E-10	5.11E-09	LINC02015
ENSG00000092969	97.85441411	1331.370129	-3.766723829	4.71E-10	5.11E-09	TGFB2
ENSG00000134871	9205.68849	27996.61107	-1.604659517	4.73E-10	5.14E-09	COL4A2
ENSG00000080573	3248.441549	492.1233991	2.723090755	4.77E-10	5.17E-09	COL5A3
ENSG00000184845	70.82236485	0	8.480345966	4.78E-10	5.19E-09	DRD1

ENSG00000164344	121.5138402	0.387220594	8.297357943	4.80E-10	5.20E-09	KLKB1
novel.254	0.921306765	61.68519669	-6.081817977	4.80E-10	5.21E-09	-
ENSG00000178980	5330.03131	2210.049975	1.270049976	4.81E-10	5.21E-09	SELENOW
ENSG00000101294	1820.381706	4694.250517	-1.366618873	4.82E-10	5.22E-09	HM13
ENSG00000149571	118.6244118	9.835463823	3.58101028	4.83E-10	5.22E-09	KIRREL3
novel.869	80.92428658	2.679862578	4.975115022	4.85E-10	5.25E-09	-
ENSG00000108344	1539.250215	3684.475948	-1.259173512	4.93E-10	5.33E-09	PSMD3
ENSG00000184117	378.4594445	1358.974449	-1.844504154	4.98E-10	5.38E-09	NIPSNAP1
ENSG00000185345	84.23729257	11.96395702	2.822758198	5.00E-10	5.40E-09	PRKN
ENSG00000132906	401.8748784	142.2538272	1.499239797	5.00E-10	5.40E-09	CASP9
ENSG00000120738	15877.76892	467.1769008	5.086812414	5.01E-10	5.40E-09	EGR1
ENSG00000147874	377.2754277	899.0505871	-1.252572582	5.08E-10	5.49E-09	HAUS6
ENSG00000189001	0	113.8761857	-9.411606644	5.18E-10	5.59E-09	SBSN
ENSG00000139508	476.0235238	87.70300679	2.43978965	5.24E-10	5.65E-09	SLC46A3
ENSG00000175662	5594.555075	1105.176526	2.33974966	5.25E-10	5.66E-09	TOM1L2
ENSG00000150967	58.64235602	223.9489428	-1.932551723	5.27E-10	5.68E-09	ABCB9
ENSG00000105255	61.5742663	629.5718246	-3.352522881	5.28E-10	5.68E-09	FSD1
ENSG00000140749	49.95573712	0	7.976318216	5.29E-10	5.69E-09	IGSF6
ENSG00000145649	153.6640692	0	9.597199364	5.32E-10	5.73E-09	GZMA
ENSG00000151612	64.68919439	436.5396791	-2.755316469	5.33E-10	5.73E-09	ZNF827
ENSG00000136014	166.4827032	2.415496705	6.079294621	5.43E-10	5.84E-09	USP44
ENSG00000250366	62.98530964	0	8.311329664	5.49E-10	5.89E-09	TUNAR
ENSG00000265298	142.928114	29.91941336	2.258626424	5.52E-10	5.93E-09	AC132812.1
ENSG00000031003	766.356647	229.6347822	1.738529836	5.59E-10	6.00E-09	FAM13B
ENSG00000155085	171.9485292	30.47437876	2.491713665	5.62E-10	6.03E-09	AK9
ENSG00000152804	333.6301088	31.88362937	3.394166024	5.64E-10	6.05E-09	HHEX
ENSG00000170485	68.29690869	1125.473296	-4.043199906	5.66E-10	6.07E-09	NPAS2
ENSG00000136169	486.413831	184.2439644	1.399491656	5.81E-10	6.22E-09	SETDB2
ENSG00000180818	0	127.7472933	-9.579027156	5.85E-10	6.27E-09	HOXC10
ENSG00000197632	0.61420451	8180.39495	-13.70812804	5.87E-10	6.28E-09	SERPINB2
ENSG00000147140	3111.407246	8068.845747	-1.374842588	5.90E-10	6.32E-09	NONO
ENSG00000171316	1482.702877	211.2621472	2.811555253	5.92E-10	6.33E-09	CHD7
ENSG00000184343	101.1611956	14.17516674	2.843098878	5.97E-10	6.39E-09	SRPK3
ENSG00000141101	343.7187111	941.0353568	-1.453216501	6.00E-10	6.42E-09	NOB1
ENSG00000172197	265.310066	36.72091573	2.848986495	6.02E-10	6.43E-09	MBOAT1
ENSG00000004142	1182.097427	2774.439875	-1.230871485	6.02E-10	6.43E-09	POLDIP2
ENSG00000157017	79.00616869	2.537067156	4.969415952	6.08E-10	6.49E-09	GHRL
novel.170	146.301066	1.049547942	7.096496538	6.08E-10	6.49E-09	-
ENSG00000131828	810.3088262	1753.61815	-1.113726775	6.16E-10	6.57E-09	PDHA1
ENSG00000164465	242.5531413	1422.815794	-2.552540795	6.16E-10	6.57E-09	DCBLD1
novel.137	55.21637875	0	8.121084793	6.16E-10	6.57E-09	-
ENSG00000262370	50.01250767	0	7.977817729	6.18E-10	6.59E-09	AC108134.3
ENSG00000253671	98.16160359	0	8.951280731	6.39E-10	6.80E-09	AC027117.1
ENSG00000168970	166.5657848	40.20346094	2.05117228	6.41E-10	6.83E-09	JMJD7-PLA2G4B
ENSG00000118526	5.541630725	194.24213	-5.138680332	6.43E-10	6.84E-09	TCF21
ENSG00000185614	310.3649449	75.30770978	2.046437556	6.48E-10	6.89E-09	INKA1
ENSG00000172086	1166.049799	502.489371	1.214076832	6.48E-10	6.89E-09	KRCC1
ENSG00000086288	57.68727166	0	8.183689742	6.48E-10	6.89E-09	NME8
novel.1114	67.98211417	2.954969332	4.551502949	6.49E-10	6.90E-09	-
ENSG00000136710	1024.655472	388.0278297	1.401075474	6.50E-10	6.91E-09	CCDC115
ENSG00000109047	51.60597187	0	8.022913656	6.52E-10	6.92E-09	RCVRN

ENSG00000286248	86.26880867	0.774441189	6.898797901	6.67E-10	7.08E-09	AC069209.2
ENSG00000111711	808.0188429	1871.071528	-1.211335167	6.69E-10	7.10E-09	GOLT1B
ENSG00000121895	101.8631559	767.2563957	-2.912421361	6.85E-10	7.27E-09	TMEM156
ENSG00000197136	740.5671397	1829.032579	-1.304265502	6.89E-10	7.31E-09	PCNX3
ENSG00000112852	93.38244903	2.0188195	5.472723805	6.91E-10	7.33E-09	PCDHB2
ENSG00000152684	411.22616	1250.105769	-1.604144914	7.03E-10	7.45E-09	PELO
ENSG00000174851	1039.302984	2556.575559	-1.298518273	7.12E-10	7.54E-09	YIF1A
ENSG00000178026	428.4801746	116.1664816	1.884241639	7.13E-10	7.55E-09	LRRC75B
ENSG00000103005	587.6632097	1841.167501	-1.647334638	7.13E-10	7.55E-09	USB1
ENSG00000100625	21.46406408	386.1282686	-4.171354206	7.18E-10	7.59E-09	SIX4
novel.644	69.60315864	285.6236214	-2.036018452	7.20E-10	7.61E-09	-
ENSG00000197724	1843.164305	674.1721258	1.451186346	7.20E-10	7.61E-09	PHF2
ENSG00000132361	817.7052506	1819.835885	-1.154283506	7.41E-10	7.82E-09	CLUH
novel.316	321.1748538	70.88186989	2.180732838	7.43E-10	7.84E-09	-
ENSG00000270885	18.82593664	318.1408012	-4.080067859	7.54E-10	7.95E-09	RASL10B
ENSG00000150048	151.1287413	0.356539013	8.611325072	7.56E-10	7.97E-09	CLEC1A
ENSG00000272674	70.55942538	1.467450118	5.612720474	7.58E-10	8.00E-09	PCDHB16
ENSG00000253837	8.194659761	97.9969777	-3.57950637	7.60E-10	8.01E-09	AC090197.1
ENSG00000163171	680.3321142	2417.041322	-1.828998684	7.72E-10	8.13E-09	CDC42EP3
novel.685	4.207416313	59.6109218	-3.818400047	7.78E-10	8.19E-09	-
ENSG00000242861	137.3088992	23.32823412	2.557691211	7.79E-10	8.20E-09	AL591895.1
ENSG00000275024	71.74395669	0	8.498931628	7.80E-10	8.21E-09	AC005383.1
ENSG00000203797	82.11423159	3.169868797	4.680119299	7.89E-10	8.30E-09	DDO
ENSG00000154217	186.9647987	962.0181392	-2.363297768	7.99E-10	8.41E-09	PITPNC1
ENSG00000231062	66.15300604	0	8.382117596	8.08E-10	8.50E-09	AC103563.2
ENSG00000198604	434.0944304	1078.211813	-1.312630625	8.17E-10	8.59E-09	BAZ1A
ENSG00000286282	80.56597036	0	8.666372189	8.18E-10	8.59E-09	AC021733.3
ENSG00000224812	91.33335402	1.049547942	6.414492187	8.22E-10	8.63E-09	TMEM72-AS1
ENSG00000091513	80.99491854	0.387220594	7.71190714	8.25E-10	8.66E-09	TF
ENSG00000023608	126.9927447	506.1140349	-1.99542103	8.30E-10	8.70E-09	SNAPC1
ENSG00000124571	471.5441526	1179.329205	-1.322668152	8.30E-10	8.70E-09	XPO5
ENSG00000131899	233.6744721	926.8794028	-1.987749579	8.48E-10	8.88E-09	LLGL1
ENSG00000101916	80.23557446	0	8.659497091	8.58E-10	8.99E-09	TLR8
ENSG00000226791	81.47540886	0	8.681627013	8.64E-10	9.04E-09	LINC02611
ENSG00000123131	808.6815677	2587.200965	-1.677873583	8.65E-10	9.06E-09	PRDX4
ENSG00000103342	1397.94548	3541.644225	-1.341214938	8.71E-10	9.11E-09	GSPT1
ENSG00000108639	1483.754839	522.8993407	1.504971271	8.99E-10	9.41E-09	SYNGR2
ENSG00000141542	873.4941672	215.3100607	2.021243269	9.01E-10	9.42E-09	RAB40B
ENSG00000092470	103.4895001	433.8601804	-2.067547345	9.02E-10	9.43E-09	WDR76
ENSG00000055118	67.59359532	1.161661783	5.955164565	9.03E-10	9.43E-09	KCNH2
ENSG00000177426	359.2049154	1322.050261	-1.87995278	9.25E-10	9.66E-09	TGIF1
ENSG00000179348	1264.063178	47.40318158	4.734149261	9.32E-10	9.73E-09	GATA2
ENSG00000014138	76.58237315	228.3869022	-1.576969672	9.36E-10	9.76E-09	POLA2
ENSG00000005812	1301.554593	546.4398714	1.251921711	9.39E-10	9.79E-09	FBXL3
ENSG00000049192	27.9318027	307.5152369	-3.460588594	9.46E-10	9.86E-09	ADAMTS6
ENSG00000162817	274.0318706	18.45967106	3.883818303	9.49E-10	9.89E-09	C1orf115
ENSG00000132170	56.12049044	251.0392896	-2.162693113	9.53E-10	9.93E-09	PPARG
ENSG00000213740	65.42410588	296.3877047	-2.181237696	9.57E-10	9.96E-09	SERBP1P1
ENSG00000171962	122.6173106	22.72380231	2.434572853	9.57E-10	9.96E-09	DRC3
ENSG00000129128	1598.960738	3427.351196	-1.099925163	9.60E-10	9.99E-09	SPCS3
ENSG00000115138	79.04782419	4.881744087	3.99480714	9.80E-10	1.02E-08	POMC

ENSG00000140263	180.5094269	648.8757416	-1.845291469	9.85E-10	1.02E-08	SORD
ENSG00000169994	49.73047839	0	7.969625972	9.97E-10	1.04E-08	MYO7B
ENSG00000236120	46.62804335	0	7.876857961	1.01E-09	1.05E-08	AC110995.1
novel.904	111.7837397	0	9.13844512	1.02E-09	1.06E-08	-
ENSG00000285967	452.3064622	140.9611384	1.682502427	1.02E-09	1.06E-08	NIPBL-DT
ENSG00000185905	61.02284333	0	8.264520469	1.02E-09	1.06E-08	C16orf54
ENSG00000250343	108.2576131	0.387220594	8.130677611	1.03E-09	1.07E-08	STK32A-AS1
ENSG00000244038	2412.893372	6778.320469	-1.490168115	1.04E-09	1.08E-08	DDOST
ENSG00000130598	60.67666415	0	8.256351484	1.04E-09	1.08E-08	TNNI2
ENSG00000204856	200.1164032	570.4766075	-1.51116783	1.05E-09	1.08E-08	FAM216A
ENSG00000157399	361.1305002	89.9991164	2.004634641	1.06E-09	1.10E-08	ARSE
ENSG00000173641	8.576083648	209.1816862	-4.612767875	1.07E-09	1.10E-08	HSPB7
ENSG00000122126	447.8406788	1072.760732	-1.260497017	1.07E-09	1.11E-08	OCRL
ENSG00000261236	518.3092713	1729.535899	-1.738672122	1.08E-09	1.12E-08	BOP1
ENSG00000139116	755.3134919	127.5560461	2.565550402	1.09E-09	1.13E-08	KIF21A
ENSG00000115053	6372.65889	16361.65188	-1.360375472	1.09E-09	1.13E-08	NCL
ENSG00000065371	50.49841203	0	7.991416397	1.09E-09	1.13E-08	ROPN1
ENSG00000134516	728.5098425	105.1301981	2.794288319	1.10E-09	1.14E-08	DOCK2
ENSG00000099250	945.7834261	4902.271875	-2.373934776	1.11E-09	1.14E-08	NRP1
ENSG00000161643	58.49775844	2.079026788	4.776587196	1.12E-09	1.15E-08	SIGLEC16
ENSG00000163273	59.82598934	0	8.235904219	1.13E-09	1.16E-08	NPPC
ENSG00000137857	260.1273088	17.04040308	3.935441684	1.14E-09	1.17E-08	DUOX1
ENSG00000078098	70.9915216	1856.44982	-4.709360438	1.14E-09	1.18E-08	FAP
ENSG00000087365	2983.735325	6608.528995	-1.147174925	1.15E-09	1.18E-08	SF3B2
ENSG00000105173	76.28941703	338.4319193	-2.14932038	1.15E-09	1.18E-08	CCNE1
ENSG00000250971	59.52655152	0	8.228656226	1.15E-09	1.19E-08	AC110772.2
ENSG00000078399	3.690241654	238.7334001	-6.022618168	1.16E-09	1.20E-08	HOXA9
ENSG00000163239	119.8169353	1.426156051	6.385714219	1.20E-09	1.24E-08	TDRD10
ENSG00000236609	544.1119875	45.42082303	3.57791695	1.21E-09	1.24E-08	ZNF853
ENSG00000168303	324.0848666	817.8904622	-1.335656987	1.21E-09	1.25E-08	MPLKIP
ENSG00000100216	558.5361192	1405.713565	-1.331781352	1.21E-09	1.25E-08	TOMM22
ENSG00000278530	104.9882004	0	9.04818949	1.22E-09	1.25E-08	CHMP1B2P
ENSG00000002834	4339.093898	9940.139403	-1.195902577	1.23E-09	1.26E-08	LASP1
ENSG00000151962	70.96509587	3.82158366	4.187086085	1.23E-09	1.27E-08	RBM46
ENSG00000170500	94.12587916	0	8.889946764	1.25E-09	1.28E-08	LONRF2
ENSG00000111667	1279.296694	2799.805503	-1.130048105	1.25E-09	1.28E-08	USP5
ENSG00000131242	118.7636944	5.911222933	4.309461354	1.26E-09	1.29E-08	RAB11FIP4
ENSG00000198618	697.3173484	1580.454927	-1.180405094	1.27E-09	1.30E-08	PPIAP22
novel.384	64.32049297	0	8.341601829	1.30E-09	1.33E-08	-
ENSG00000138095	1128.23438	2757.747987	-1.289521048	1.31E-09	1.34E-08	LRPPRC
ENSG00000229950	50.37477082	0	7.988739116	1.31E-09	1.34E-08	TFAP2A-AS1
ENSG00000008838	887.1162717	1992.737346	-1.16760697	1.31E-09	1.34E-08	MED24
ENSG00000275880	3.972128392	50.94439301	-3.689198713	1.32E-09	1.35E-08	AL139385.1
ENSG00000006210	214.7424728	18.42282492	3.550920453	1.32E-09	1.35E-08	CX3CL1
ENSG00000103056	139.4754819	33.28282825	2.070866417	1.32E-09	1.35E-08	SMPD3
ENSG00000285051	50.24897797	2.191140629	4.539029044	1.32E-09	1.35E-08	SLC7A14-AS1
ENSG00000149100	1216.689945	2814.451296	-1.209970066	1.33E-09	1.36E-08	EIF3M
ENSG00000118946	106.6564845	0.713078025	7.216516873	1.34E-09	1.36E-08	PCDH17
ENSG00000176209	741.8234415	258.8754895	1.518014543	1.36E-09	1.39E-08	SMIM19
ENSG00000010256	1968.296169	5012.017822	-1.348383812	1.37E-09	1.39E-08	UQCRC1
ENSG00000164134	478.3688825	1422.957916	-1.572992164	1.37E-09	1.40E-08	NAA15

ENSG00000277476	141.9511481	21.09451516	2.756734379	1.37E-09	1.40E-08	AC005332.5
ENSG00000166839	474.9007672	115.1841574	2.045330354	1.38E-09	1.40E-08	ANKDD1A
ENSG00000134321	628.4573637	97.0371284	2.692891636	1.40E-09	1.42E-08	RSAD2
ENSG00000015171	3201.512329	1206.424291	1.40792033	1.41E-09	1.43E-08	ZMYND11
ENSG00000119888	75.91856433	2.486316478	4.923123568	1.41E-09	1.43E-08	EPCAM
ENSG00000136997	590.8977367	3319.523948	-2.489798763	1.41E-09	1.43E-08	MYC
ENSG00000009780	321.1712351	104.3880267	1.621539299	1.41E-09	1.44E-08	FAM76A
ENSG00000135924	4295.576173	1322.610736	1.699718213	1.42E-09	1.44E-08	DNAJB2
ENSG00000167771	8.914669664	227.8268341	-4.67980836	1.42E-09	1.44E-08	RCOR2
ENSG00000109790	1340.023276	2981.736833	-1.153930842	1.42E-09	1.45E-08	KLHL5
ENSG00000127526	614.304561	1806.97018	-1.556409059	1.43E-09	1.45E-08	SLC35E1
ENSG00000105552	451.6689651	1121.010939	-1.311376726	1.43E-09	1.45E-08	BCAT2
ENSG00000100591	1088.837351	3066.959008	-1.494144755	1.43E-09	1.45E-08	AHSA1
ENSG00000166971	820.282776	289.6543723	1.501114804	1.44E-09	1.46E-08	AKTIP
ENSG00000198931	955.7094848	2444.907723	-1.355128097	1.46E-09	1.48E-08	APRT
ENSG00000073737	193.3017153	0.713078025	8.074524189	1.48E-09	1.50E-08	DHRS9
ENSG00000067369	520.3287498	1368.100066	-1.394604868	1.50E-09	1.52E-08	TP53BP1
ENSG00000198300	81.3935375	5.574753017	3.846507697	1.51E-09	1.53E-08	PEG3
ENSG00000081665	513.8694307	211.0771246	1.283303047	1.51E-09	1.53E-08	ZNF506
ENSG00000061676	1785.408035	3901.963624	-1.127966779	1.52E-09	1.54E-08	NCKAP1
ENSG00000143977	685.5988548	1534.546247	-1.162416218	1.54E-09	1.55E-08	SNRPG
ENSG00000144445	508.0803747	201.5816795	1.333999716	1.55E-09	1.57E-08	KANSL1L
novel.92	0	79.99650289	-8.901820797	1.56E-09	1.58E-08	-
ENSG00000120885	20237.69635	893.2776255	4.501605815	1.57E-09	1.58E-08	CLU
ENSG00000265107	76.15456391	0.356539013	7.622572045	1.57E-09	1.59E-08	GJA5
ENSG00000141543	986.1076219	2355.106964	-1.256122001	1.58E-09	1.59E-08	EIF4A3
ENSG00000143569	1814.54074	3936.727388	-1.117320916	1.58E-09	1.59E-08	UBAP2L
ENSG00000187595	106.7569192	0.387220594	8.110625173	1.59E-09	1.60E-08	ZNF385C
ENSG00000153291	551.5046516	104.1021319	2.406721999	1.60E-09	1.61E-08	SLC25A27
ENSG00000168374	2262.19309	8224.133495	-1.862184437	1.60E-09	1.61E-08	ARF4
ENSG00000086205	126.3478679	0	9.314814072	1.60E-09	1.61E-08	FOLH1
ENSG00000131477	274.3193118	13.43256301	4.355315458	1.65E-09	1.66E-08	RAMP2
ENSG00000174080	2140.239411	765.7137507	1.483242768	1.65E-09	1.66E-08	CTSF
ENSG00000020256	201.8481963	519.1772506	-1.36292387	1.65E-09	1.66E-08	ZFP64
ENSG00000105063	1126.311719	2770.259328	-1.298322141	1.66E-09	1.67E-08	PPP6R1
ENSG00000169507	87.16655301	0.356539013	7.818145708	1.67E-09	1.68E-08	SLC38A11
ENSG00000198435	127.1991271	11.87422403	3.422669077	1.67E-09	1.68E-08	NRARP
ENSG00000123395	303.3145564	762.652669	-1.33062411	1.70E-09	1.71E-08	ATG101
ENSG00000156966	147.9117409	5.654901006	4.734551606	1.71E-09	1.72E-08	B3GNT7
ENSG00000287419	91.54278568	0	8.850582113	1.72E-09	1.73E-08	AL121929.3
ENSG00000144711	2230.809999	624.9061823	1.836031162	1.72E-09	1.73E-08	IQSEC1
novel.20	68.56930752	2.954969332	4.561759519	1.73E-09	1.73E-08	-
ENSG00000173198	94.88905476	0.387220594	7.939710663	1.73E-09	1.74E-08	CYSLTR1
ENSG00000171502	429.4751626	57.12442974	2.91358111	1.75E-09	1.75E-08	COL24A1
ENSG00000287778	44.39397675	0	7.806361934	1.77E-09	1.77E-08	AC006230.1
ENSG00000277147	172.6884832	46.44169718	1.892484054	1.77E-09	1.78E-08	LINC00869
ENSG00000166925	5735.481027	2034.458306	1.495387035	1.77E-09	1.78E-08	TSC22D4
ENSG00000103353	896.155579	2490.840894	-1.47488188	1.78E-09	1.78E-08	UBFD1
ENSG00000179639	53.33658269	0	8.070268929	1.79E-09	1.79E-08	FCER1A
ENSG00000166275	593.4044411	198.3351775	1.580354729	1.80E-09	1.80E-08	BORCS7
ENSG00000110713	1047.47619	2174.357321	-1.053740541	1.80E-09	1.80E-08	NUP98

ENSG00000090539	182.1661278	9.915740207	4.193748102	1.80E-09	1.80E-08	CHRD
ENSG00000107159	3.590704505	319.2596244	-6.469564154	1.81E-09	1.81E-08	CA9
ENSG00000167085	868.5461796	2269.234625	-1.385694829	1.81E-09	1.81E-08	PHB
ENSG00000172331	920.2027257	321.3687155	1.516911267	1.81E-09	1.81E-08	BPGM
ENSG00000239911	59.18399075	0	8.220410166	1.83E-09	1.83E-08	PRKAG2-AS1
ENSG00000139832	972.592389	226.4931552	2.103648647	1.87E-09	1.86E-08	RAB20
ENSG00000116337	560.7037418	2280.776482	-2.024156947	1.87E-09	1.87E-08	AMPD2
ENSG00000244462	863.2777039	1822.726464	-1.07823363	1.89E-09	1.88E-08	RBM12
ENSG00000177694	330.2783117	57.65513491	2.51394789	1.89E-09	1.88E-08	NAALADL2
ENSG00000128564	1.789675198	58.40180139	-5.012065411	1.93E-09	1.92E-08	VGF
ENSG00000134532	333.8061074	39.07936881	3.098172669	1.94E-09	1.93E-08	SOX5
ENSG00000266028	3205.130259	1211.901255	1.403226424	1.94E-09	1.93E-08	SRGAP2
ENSG00000118518	1491.976184	662.0297302	1.172510805	1.96E-09	1.95E-08	RNF146
ENSG00000253988	47.63216256	0	7.9080497	1.96E-09	1.95E-08	AC079015.1
ENSG00000053524	27.98606596	153.0024649	-2.453482611	1.98E-09	1.97E-08	MCF2L2
ENSG00000006453	47.33795279	258.488958	-2.45039979	1.99E-09	1.98E-08	BAIAP2L1
ENSG00000120162	191.5766597	8.437549209	4.513419377	2.00E-09	1.98E-08	MOB3B
ENSG00000138696	374.3322357	32.33046213	3.527498485	2.00E-09	1.99E-08	BMPR1B
ENSG00000272143	168.7205864	34.85550414	2.272013514	2.01E-09	2.00E-08	FGF14-AS2
ENSG00000004897	1030.668833	2110.928656	-1.034311562	2.06E-09	2.04E-08	CDC27
ENSG00000139626	85.42354755	0.672939833	6.905686176	2.06E-09	2.04E-08	ITGB7
ENSG00000137441	903.6763244	0	12.15335141	2.08E-09	2.06E-08	FGFBP2
ENSG00000175879	3.083559037	168.5821775	-5.782263584	2.08E-09	2.06E-08	HOXD8
ENSG00000183527	236.5140821	886.0644973	-1.90570464	2.10E-09	2.07E-08	PSMG1
ENSG00000151320	150.8387788	13.01478923	3.526659771	2.13E-09	2.11E-08	AKAP6
ENSG00000121577	79.0376528	5.573468747	3.85121634	2.13E-09	2.11E-08	POPDC2
ENSG00000070808	110.5432931	5.258223802	4.422133212	2.13E-09	2.11E-08	CAMK2A
ENSG00000158079	1411.880423	353.3613766	1.998602981	2.13E-09	2.11E-08	PTPDC1
ENSG00000196268	388.10833	85.50968281	2.181076285	2.15E-09	2.12E-08	ZNF493
ENSG00000116883	51.80712619	306.4163169	-2.563539149	2.16E-09	2.14E-08	AL591845.1
ENSG00000178971	608.2948777	216.4355655	1.49128477	2.16E-09	2.14E-08	CTC1
ENSG00000006071	69.48477332	0	8.451910342	2.17E-09	2.15E-08	ABCC8
ENSG00000111786	1062.270775	2869.176733	-1.433477046	2.20E-09	2.17E-08	SRSF9
ENSG00000170871	1619.793206	654.8428051	1.306681138	2.20E-09	2.17E-08	KIAA0232
ENSG00000105364	418.9795489	988.058287	-1.237924683	2.23E-09	2.20E-08	MRPL4
ENSG00000117139	482.2551496	2215.488603	-2.19971903	2.25E-09	2.21E-08	KDM5B
ENSG00000198844	84.11422048	0.387220594	7.765769753	2.26E-09	2.22E-08	ARHGEF15
ENSG00000176049	797.325129	19.16342087	5.372264959	2.27E-09	2.24E-08	JAKMIP2
ENSG00000131042	55.62824622	0	8.131214265	2.29E-09	2.26E-08	LILRB2
ENSG00000196227	442.0358235	163.0029294	1.438254918	2.30E-09	2.26E-08	FAM217B
ENSG00000115363	178.3072992	855.4242282	-2.262315133	2.30E-09	2.26E-08	EVA1A
ENSG00000002587	84.76016601	0.336469917	7.777752819	2.31E-09	2.27E-08	HS3ST1
ENSG00000233117	15.15714956	115.235854	-2.928795267	2.31E-09	2.27E-08	LINC00702
ENSG00000237797	49.66034498	0	7.96720671	2.32E-09	2.28E-08	MACORIS
ENSG00000136237	1072.325234	30.39367001	5.14491402	2.33E-09	2.29E-08	RAPGEF5
ENSG00000012779	90.22019415	0	8.828787012	2.34E-09	2.30E-08	ALOX5
ENSG00000066923	224.9434495	43.88083651	2.361247354	2.34E-09	2.30E-08	STAG3
ENSG00000134323	58.35853146	0	8.200063741	2.35E-09	2.31E-08	MYCN
ENSG00000123416	4460.924113	15617.75642	-1.807796964	2.37E-09	2.32E-08	TUBA1B
ENSG00000174837	62.0029294	0	8.287801289	2.37E-09	2.32E-08	ADGRE1
ENSG00000075240	789.026027	258.9879413	1.60626398	2.37E-09	2.33E-08	GRAMD4

ENSG00000160208	513.4866816	1189.125396	-1.211783863	2.38E-09	2.33E-08	RRP1B
ENSG00000173947	83.31645618	0.743759607	6.853282482	2.38E-09	2.33E-08	PIFO
ENSG00000254087	968.9117741	125.3034954	2.952726246	2.40E-09	2.35E-08	LYN
ENSG00000102349	144.3087137	8.602982267	4.048804371	2.42E-09	2.37E-08	KLF8
ENSG00000182934	2059.527433	4308.109591	-1.064763084	2.43E-09	2.38E-08	SRPRA
ENSG00000176115	57.37264787	0	8.176199972	2.45E-09	2.39E-08	AQP7P4
ENSG00000116017	170.65822	1023.18886	-2.583986006	2.46E-09	2.41E-08	ARID3A
ENSG00000085733	4109.295324	8436.225536	-1.037673495	2.49E-09	2.43E-08	CTTN
ENSG00000168952	98.23283285	14.11624373	2.789098666	2.49E-09	2.43E-08	STXBP6
ENSG00000161179	144.6924589	636.6565158	-2.138238776	2.50E-09	2.44E-08	YDJC
ENSG00000165121	100.2436498	18.27301303	2.455191329	2.51E-09	2.45E-08	AL353743.1
ENSG00000182957	2427.155988	242.51768	3.323877006	2.51E-09	2.45E-08	SPATA13
ENSG00000101266	1315.917979	3199.725751	-1.281799365	2.52E-09	2.46E-08	CSNK2A1
ENSG00000135372	614.7052172	1523.048386	-1.309250756	2.53E-09	2.47E-08	NAT10
ENSG00000118473	57.92563635	750.3205077	-3.696086812	2.54E-09	2.47E-08	SGIP1
ENSG00000177853	1246.082116	337.6326568	1.884191015	2.56E-09	2.50E-08	ZNF518A
ENSG00000089335	972.7493653	346.4727094	1.488655256	2.58E-09	2.51E-08	ZNF302
ENSG00000146425	704.7676362	1943.119555	-1.463144651	2.58E-09	2.51E-08	DYNLT1
ENSG00000099260	169.2453383	6.421169854	4.717372396	2.62E-09	2.55E-08	PALMD
ENSG00000283632	113.6628082	5.983198582	4.236490639	2.62E-09	2.55E-08	EXOC3L2
ENSG00000255240	51.54189289	0	8.02083906	2.63E-09	2.55E-08	AP001636.3
ENSG00000141076	129.0782393	420.1669288	-1.703374139	2.63E-09	2.56E-08	UTP4
ENSG00000135842	1981.997277	416.3489627	2.251605438	2.66E-09	2.58E-08	FAM129A
ENSG00000180769	229.6459292	49.69081488	2.204814131	2.70E-09	2.62E-08	WDFY3-AS2
ENSG00000101445	109.8325132	0.336469917	8.150765836	2.71E-09	2.63E-08	PPP1R16B
ENSG00000107581	3946.282435	7812.208964	-0.985215087	2.72E-09	2.64E-08	EIF3A
ENSG00000149575	159.5422486	1.069617038	7.213333713	2.72E-09	2.64E-08	SCN2B
ENSG00000165685	120.6594323	5.127325134	4.528124865	2.73E-09	2.65E-08	TMEM52B
ENSG00000227619	0.293240848	70.92414425	-7.765862269	2.74E-09	2.65E-08	AL391056.1
ENSG00000198056	42.18408547	153.6229374	-1.865215476	2.74E-09	2.65E-08	PRIM1
ENSG00000267127	52.39464807	2.51699806	4.382218509	2.74E-09	2.66E-08	AC090360.1
ENSG00000171914	1042.097467	124.0727774	3.068715097	2.76E-09	2.67E-08	TLN2
ENSG00000183454	49.26191165	0	7.956835549	2.76E-09	2.67E-08	GRIN2A
ENSG00000113263	51.55958652	0	8.021316002	2.78E-09	2.69E-08	ITK
ENSG00000159164	190.0486428	790.8844888	-2.057173	2.79E-09	2.70E-08	SV2A
ENSG00000132622	146.1496304	6.389332397	4.509632949	2.81E-09	2.72E-08	HSPA12B
ENSG00000184408	0	57.83506762	-8.433542517	2.81E-09	2.72E-08	KCND2
ENSG00000285976	38.45476689	175.9541721	-2.194809747	2.82E-09	2.73E-08	AL135905.2
ENSG00000121933	108.0693944	0.387220594	8.12823837	2.87E-09	2.78E-08	TMIGD3
ENSG00000099814	543.3307761	1731.939794	-1.672249647	2.90E-09	2.80E-08	CEP170B
ENSG00000107566	804.1180588	1925.828634	-1.260173273	2.91E-09	2.81E-08	ERLIN1
ENSG00000187068	234.8590038	21.26007661	3.457363796	2.94E-09	2.84E-08	C3orf70
ENSG00000126457	1698.328683	4434.746289	-1.38466247	2.98E-09	2.88E-08	PRMT1
ENSG00000122223	64.57608057	0	8.346173743	2.99E-09	2.88E-08	CD244
ENSG00000265354	649.5453174	1703.761718	-1.391436552	3.04E-09	2.93E-08	TIMM23
ENSG00000177181	46.27588066	0	7.865374589	3.07E-09	2.96E-08	RIMKLA
ENSG00000150787	263.4027168	748.83515	-1.507856881	3.09E-09	2.97E-08	PTS
ENSG00000167614	72.37850432	0.336469917	7.549835204	3.10E-09	2.98E-08	TTYH1
ENSG00000182263	885.2370106	96.44788539	3.197382093	3.11E-09	2.99E-08	FIGN
novel.928	102.8907228	0	9.018441688	3.13E-09	3.01E-08	-
novel.112	73.27743104	0	8.529615282	3.14E-09	3.02E-08	-

ENSG00000100842	2016.6296	498.4436755	2.016940374	3.20E-09	3.07E-08	EFS
ENSG00000078967	358.1944295	122.4616331	1.54725349	3.23E-09	3.10E-08	UBE2D4
ENSG00000176177	56.13567642	0	8.144115778	3.24E-09	3.11E-08	ENTHD1
ENSG00000146701	1471.545468	3790.181172	-1.365002899	3.25E-09	3.12E-08	MDH2
ENSG00000134779	1054.455519	2332.454633	-1.145218159	3.26E-09	3.13E-08	TPGS2
ENSG00000104413	117.0877029	1.161661783	6.730468737	3.28E-09	3.15E-08	ESRP1
ENSG00000125388	359.5918965	125.2888674	1.521935145	3.29E-09	3.16E-08	GRK4
ENSG00000167625	165.9106368	510.041123	-1.619707059	3.37E-09	3.24E-08	ZNF526
ENSG00000100450	85.60001273	0	8.752938344	3.38E-09	3.24E-08	GZMH
ENSG00000159788	1068.993137	420.4595705	1.346675033	3.38E-09	3.24E-08	RGS12
ENSG00000256667	147.0345969	27.28084484	2.4306533	3.40E-09	3.25E-08	KLRA1P
ENSG00000157103	2442.755801	0	13.58795988	3.48E-09	3.34E-08	SLC6A1
ENSG00000165105	96.7172812	1.080229524	6.488381106	3.52E-09	3.37E-08	RASEF
ENSG00000205593	468.3697485	93.57511901	2.323534637	3.53E-09	3.37E-08	DENND6B
ENSG00000060709	80.56323399	0	8.66545646	3.54E-09	3.38E-08	RIMBP2
ENSG00000143546	50.10130362	0	7.98123964	3.63E-09	3.47E-08	S100A8
ENSG00000103202	949.1261227	4009.394532	-2.078753923	3.63E-09	3.47E-08	NME4
ENSG00000171604	2308.257555	1024.568143	1.171834289	3.64E-09	3.47E-08	CXXC5
ENSG00000158470	720.9362572	1527.153702	-1.083022664	3.64E-09	3.48E-08	B4GALT5
ENSG00000186188	48.34060489	0	7.929619432	3.70E-09	3.53E-08	FFAR4
ENSG00000160191	945.0794796	34.72717401	4.762246409	3.71E-09	3.54E-08	PDE9A
ENSG00000229417	70.37639161	0.774441189	6.604932778	3.73E-09	3.55E-08	NPM1P25
ENSG00000072071	463.6152949	101.5553514	2.193230551	3.76E-09	3.59E-08	ADGRL1
ENSG00000164161	42.21940145	1837.954087	-5.444272731	3.77E-09	3.59E-08	HHIP
novel.343	53.73115632	0	8.081998055	3.82E-09	3.64E-08	-
ENSG00000171357	119.5342501	21.2563522	2.49320997	3.85E-09	3.66E-08	LURAP1
ENSG00000286156	210.3537197	72.81463363	1.530342105	3.90E-09	3.71E-08	AC026273.1
ENSG00000103942	223.4036628	39.71332632	2.495976343	3.91E-09	3.73E-08	HOMER2
ENSG00000277734	351.191791	6.705604823	5.722313586	3.93E-09	3.74E-08	TRAC
ENSG00000115648	84.54370995	2379.148809	-4.815066158	3.95E-09	3.75E-08	MLPH
ENSG00000064201	88.67616424	7.693789602	3.539545497	3.98E-09	3.78E-08	TSPAN32
ENSG00000135930	820.519701	1828.467704	-1.156135645	4.00E-09	3.80E-08	EIF4E2
ENSG00000197451	1941.424587	5379.282594	-1.470349636	4.04E-09	3.84E-08	HNRNPAB
ENSG00000100804	1453.357084	3901.156478	-1.424604139	4.05E-09	3.84E-08	PSMB5
ENSG00000143839	0	99.12617255	-9.2132319	4.06E-09	3.86E-08	REN
ENSG00000119711	881.9188427	311.8546143	1.500136548	4.10E-09	3.89E-08	ALDH6A1
ENSG00000115514	263.37743	809.1285151	-1.619800735	4.14E-09	3.93E-08	TXNDC9
ENSG00000250634	0.293240848	59.51181341	-7.51381603	4.14E-09	3.93E-08	LINC01182
ENSG00000135441	511.239865	135.8161031	1.912210297	4.16E-09	3.94E-08	BLOC1S1
ENSG00000142408	11.51984623	455.7838649	-5.306141049	4.16E-09	3.94E-08	CACNG8
ENSG00000163539	2176.600142	728.6137045	1.578703381	4.17E-09	3.95E-08	CLASP2
ENSG00000147168	79.75998402	0	8.650959904	4.18E-09	3.96E-08	IL2RG
ENSG00000092096	3918.672391	880.7320299	2.153905254	4.32E-09	4.09E-08	SLC22A17
ENSG00000100219	763.5708442	2309.607371	-1.597019944	4.34E-09	4.11E-08	XBP1
ENSG00000183751	534.7281368	1407.634701	-1.39666325	4.34E-09	4.11E-08	TBL3
ENSG00000211584	658.8441317	239.2396913	1.461314274	4.37E-09	4.13E-08	SLC48A1
ENSG00000259038	65.204119	0	8.36114845	4.40E-09	4.16E-08	AL121820.2
ENSG00000157540	1497.459177	698.1906375	1.100646695	4.40E-09	4.16E-08	DYRK1A
ENSG00000280255	62.65940231	0	8.303429165	4.40E-09	4.16E-08	AC004947.2
ENSG00000132341	2805.310936	9311.897268	-1.730961859	4.48E-09	4.23E-08	RAN
ENSG00000172927	9.476007113	125.6141641	-3.73148343	4.48E-09	4.23E-08	MYEOV

ENSG00000198515	67.0363039	1.00940975	5.977483194	4.52E-09	4.26E-08	CNGA1
ENSG00000117834	101.3501417	3.12857473	4.994784125	4.57E-09	4.31E-08	SLC5A9
ENSG00000080166	46.30471493	0	7.866865968	4.60E-09	4.34E-08	DCT
novel.113	78.42950308	0.336469917	7.66579174	4.63E-09	4.36E-08	-
ENSG00000197937	774.7220369	148.3068181	2.385265026	4.67E-09	4.40E-08	ZNF347
ENSG00000012124	502.0905743	47.73986143	3.398579759	4.68E-09	4.41E-08	CD22
ENSG00000146223	1822.32407	4012.48916	-1.138814152	4.71E-09	4.43E-08	RPL7L1
ENSG00000134375	617.1236704	1657.993474	-1.426015652	4.72E-09	4.44E-08	TIMM17A
novel.311	73.79169895	0.713078025	6.684825978	4.74E-09	4.46E-08	-
ENSG00000064547	239.0721182	53.57820963	2.159941986	4.82E-09	4.53E-08	LPAR2
ENSG00000108592	694.2934232	1734.587019	-1.321162177	4.82E-09	4.53E-08	FTSJ3
ENSG00000184867	750.4569215	2039.43554	-1.442479644	4.86E-09	4.57E-08	ARMCX2
ENSG00000253320	151.0440782	32.16618495	2.225183483	4.88E-09	4.58E-08	AZIN1-AS1
ENSG00000163811	420.7818753	1149.786927	-1.450307001	4.95E-09	4.65E-08	WDR43
ENSG00000219626	261.2413347	57.25820092	2.187806549	4.97E-09	4.67E-08	FAM228B
ENSG00000103326	627.9042194	1632.264427	-1.378030277	5.00E-09	4.69E-08	CAPN15
ENSG00000248187	0.308355904	56.58868153	-7.441159532	5.01E-09	4.69E-08	AC078850.1
ENSG00000213512	77.37535193	0	8.607935627	5.04E-09	4.72E-08	GBP7
ENSG00000121579	1178.166468	2689.159483	-1.190730102	5.05E-09	4.74E-08	NAA50
ENSG00000261701	88.82721605	1.029478846	6.383008882	5.06E-09	4.74E-08	HPR
ENSG00000111728	160.7211531	2.792104814	5.829159029	5.09E-09	4.77E-08	ST8SIA1
ENSG00000186340	1416.638488	3471.878489	-1.293161219	5.10E-09	4.77E-08	THBS2
ENSG00000058335	177.2351853	6.389204003	4.8141109	5.14E-09	4.81E-08	RASGRF1
novel.313	62.46042711	0	8.298111161	5.15E-09	4.82E-08	-
ENSG00000104341	683.2042325	2761.585368	-2.015256776	5.26E-09	4.92E-08	LAPTM4B
ENSG00000160223	67.06646239	1.803920035	5.221378004	5.29E-09	4.94E-08	ICOSLG
ENSG00000197566	207.2245307	62.52112944	1.729452167	5.30E-09	4.96E-08	ZNF624
ENSG00000271971	69.6627925	11.44455349	2.603061289	5.38E-09	5.03E-08	AC120053.1
ENSG00000125457	442.0312641	103.5889401	2.091884422	5.40E-09	5.05E-08	MIF4GD
ENSG00000189343	594.0346851	2045.012417	-1.783623245	5.43E-09	5.07E-08	RPS2P46
ENSG00000128739	227.8582631	71.35065113	1.674820974	5.45E-09	5.09E-08	SNRPN
ENSG00000230109	40.34397924	0	7.667783812	5.46E-09	5.09E-08	LINC02643
ENSG00000136856	643.663863	211.4661434	1.60674138	5.53E-09	5.16E-08	SLC2A8
ENSG00000150627	172.5068612	21.55139965	3.007024417	5.55E-09	5.18E-08	WDR17
ENSG00000239305	1102.649462	361.0560309	1.610229408	5.56E-09	5.19E-08	RNF103
ENSG00000127249	79.6528975	1.426156051	5.796296097	5.57E-09	5.19E-08	ATP13A4
ENSG00000156265	160.8920217	39.81868049	2.011102074	5.57E-09	5.19E-08	MAP3K7CL
ENSG00000151135	914.2053763	2537.214118	-1.472703152	5.58E-09	5.20E-08	TMEM263
ENSG00000273373	183.6235721	48.69188922	1.914313026	5.63E-09	5.24E-08	AL355488.1
ENSG00000066382	42.87017559	0	7.755202918	5.66E-09	5.27E-08	MPPED2
ENSG00000121957	218.4686159	556.1070765	-1.348020855	5.70E-09	5.30E-08	GPSM2
ENSG00000213281	777.9667716	1907.28968	-1.293912164	5.78E-09	5.38E-08	NRAS
ENSG00000165194	117.4990002	2.537067156	5.539671329	5.80E-09	5.39E-08	PCDH19
ENSG00000109762	235.4527932	670.309984	-1.509993688	5.81E-09	5.39E-08	SNX25
ENSG00000133706	1484.013961	3335.741299	-1.168494127	5.84E-09	5.42E-08	LARS
ENSG00000188985	36.65624488	229.3605873	-2.646547868	5.85E-09	5.43E-08	DHFRP1
ENSG00000001561	496.1770527	26.295357	4.232387135	5.86E-09	5.43E-08	ENPP4
ENSG00000135446	700.9698652	1807.173992	-1.366484722	5.86E-09	5.44E-08	CDK4
ENSG00000100320	116.772908	678.8145902	-2.539179444	5.93E-09	5.49E-08	RBFOX2
ENSG00000123496	0.308355904	66.64336435	-7.675980562	5.96E-09	5.53E-08	IL13RA2
ENSG00000178665	99.29566008	12.35130601	2.997873772	6.02E-09	5.58E-08	ZNF713

ENSG00000051825	182.3262829	458.0468906	-1.329550902	6.04E-09	5.59E-08	MPHOSPH9
ENSG00000131351	93.81609923	303.2077351	-1.6930647	6.04E-09	5.60E-08	HAUS8
ENSG00000269713	141.9111023	463.7099171	-1.707290627	6.20E-09	5.74E-08	NBPF9
ENSG00000185278	822.6415834	205.218403	2.00267842	6.22E-09	5.75E-08	ZBTB37
ENSG00000162383	46.95764036	0	7.887505117	6.22E-09	5.75E-08	SLC1A7
ENSG00000130449	1860.123488	326.606729	2.510163344	6.28E-09	5.80E-08	ZSWIM6
ENSG00000170445	1093.187229	2480.745436	-1.182284728	6.30E-09	5.82E-08	HARS
ENSG00000156509	3.634796025	44.5160783	-3.614725243	6.30E-09	5.82E-08	FBXO43
ENSG00000078487	212.3669014	65.19410394	1.701391098	6.34E-09	5.85E-08	ZCWPW1
ENSG00000229671	42.93432549	0	7.757222036	6.39E-09	5.90E-08	LINC01150
novel.445	11.25558185	67.67669601	-2.590557483	6.39E-09	5.90E-08	-
ENSG00000134115	56.55686176	0	8.155712259	6.50E-09	6.00E-08	CNTN6
ENSG00000261888	210.6185257	19.75364418	3.404348096	6.55E-09	6.04E-08	AC144831.1
ENSG00000183734	86.81538672	2.587817834	5.091079104	6.62E-09	6.11E-08	ASCL2
novel.492	565.8769015	2473.257449	-2.127718447	6.64E-09	6.12E-08	-
ENSG00000197043	2933.653759	9279.58211	-1.661375031	6.68E-09	6.15E-08	ANXA6
ENSG00000132837	83.25072445	11.27223236	2.872544499	6.92E-09	6.38E-08	DMGDH
ENSG00000275395	116.4861919	1.029478846	6.77767408	6.93E-09	6.38E-08	FCGBP
ENSG00000101310	597.3001671	1627.512824	-1.446263752	6.95E-09	6.40E-08	SEC23B
ENSG00000163617	294.6773443	69.3388481	2.089133144	6.97E-09	6.41E-08	CCDC191
ENSG00000197170	835.3158132	1759.216452	-1.074689704	6.97E-09	6.41E-08	PSMD12
ENSG00000147642	219.9074693	12.54729225	4.122719466	6.98E-09	6.42E-08	SYBU
ENSG00000103507	639.621443	1802.812911	-1.494834462	7.00E-09	6.43E-08	BCKDK
ENSG00000100311	1274.655701	174.4612122	2.870501261	7.04E-09	6.46E-08	PDGFB
ENSG00000129422	1175.881586	27.39925164	5.419265587	7.16E-09	6.58E-08	MTUS1
ENSG00000170054	0	53.63584837	-8.324979676	7.19E-09	6.61E-08	SERPINA9
ENSG00000161640	45.74993046	0	7.848823598	7.23E-09	6.63E-08	SIGLEC11
novel.552	16.56074228	270.5989897	-4.032843863	7.29E-09	6.69E-08	-
ENSG00000116750	401.0098002	929.634524	-1.21336907	7.34E-09	6.73E-08	UCHL5
ENSG00000095637	7656.580476	230.4885791	5.053389765	7.38E-09	6.76E-08	SORBS1
ENSG00000164172	1964.270906	751.2868037	1.386554541	7.42E-09	6.80E-08	MOCS2
ENSG00000182180	663.5939382	1729.529148	-1.382229671	7.47E-09	6.84E-08	MRPS16
ENSG00000065717	873.3458863	96.01723456	3.18335881	7.48E-09	6.85E-08	TLE2
ENSG00000138829	150.2739788	4618.615676	-4.94198347	7.53E-09	6.90E-08	FBN2
ENSG00000233005	66.70137973	0	8.393998642	7.58E-09	6.94E-08	AC018742.1
ENSG00000203710	93.29415162	0	8.877189392	7.59E-09	6.94E-08	CR1
ENSG00000176593	267.7537061	68.62602686	1.963337251	7.79E-09	7.13E-08	AC008969.1
ENSG00000041515	149.1302202	27.26894809	2.45617734	7.83E-09	7.16E-08	MYO16
ENSG00000169031	580.3770748	60.21239956	3.266011243	7.87E-09	7.19E-08	COL4A3
ENSG00000131471	179.7670488	9.914455938	4.188479762	7.87E-09	7.19E-08	AOC3
ENSG00000112175	104.9716305	0	9.04737689	7.89E-09	7.21E-08	BMP5
ENSG00000135245	133.0819224	1264.457081	-3.248109024	8.05E-09	7.35E-08	HILPDA
ENSG00000085788	2344.097833	715.6519652	1.711987007	8.10E-09	7.40E-08	DDHD2
ENSG00000104885	570.4364517	2105.248746	-1.883604048	8.16E-09	7.45E-08	DOT1L
ENSG00000172534	899.5023076	1760.836608	-0.969108555	8.19E-09	7.47E-08	HCFC1
ENSG00000140022	104.0675188	0.336469917	8.073001626	8.22E-09	7.50E-08	STON2
ENSG00000011021	1270.094301	429.365049	1.564772057	8.23E-09	7.50E-08	CLCN6
novel.85	73.99439201	0.356539013	7.581743849	8.26E-09	7.52E-08	-
ENSG00000173432	0.921306765	3156.821482	-11.74817171	8.29E-09	7.55E-08	SAA1
ENSG00000064652	1096.422398	318.2912298	1.78463057	8.37E-09	7.61E-08	SNX24
ENSG00000080608	354.8540136	1119.346277	-1.657712257	8.37E-09	7.61E-08	PUM3

ENSG00000128298	126.7452481	24.25377147	2.389880798	8.38E-09	7.62E-08	BAIAP2L2
ENSG00000259275	382.6712945	31.94324154	3.57703398	8.39E-09	7.63E-08	AC087477.2
ENSG00000163874	141.3083937	2403.840472	-4.088343401	8.41E-09	7.64E-08	ZC3H12A
ENSG00000063180	405.8130982	33.5140724	3.601887066	8.41E-09	7.64E-08	CA11
ENSG00000089157	12913.15747	35035.35772	-1.439979476	8.46E-09	7.68E-08	RPLP0
ENSG00000128708	541.1800052	1326.744695	-1.293999531	8.46E-09	7.68E-08	HAT1
ENSG00000119041	446.8290095	986.6730264	-1.143029785	8.48E-09	7.70E-08	GTF3C3
ENSG00000115884	59.18678278	708.3565823	-3.58213022	8.49E-09	7.70E-08	SDC1
ENSG00000144649	121.7422028	4.514592589	4.733619034	8.52E-09	7.73E-08	GASK1A
ENSG00000149968	2.157237677	4109.664974	-10.89938543	8.61E-09	7.81E-08	MMP3
ENSG00000104976	286.7979057	842.7664235	-1.554970425	8.64E-09	7.83E-08	SNAPC2
ENSG00000138346	57.59017089	204.9486904	-1.830192046	8.65E-09	7.84E-08	DNA2
ENSG00000171241	194.7678076	1292.141895	-2.72963921	8.81E-09	7.99E-08	SHCBP1
ENSG00000168679	632.6593255	171.0146609	1.886281294	8.94E-09	8.09E-08	SLC16A4
ENSG00000154258	1643.153526	42.59585328	5.266926592	9.10E-09	8.24E-08	ABCA9
ENSG00000164300	1339.784666	235.007654	2.510214842	9.18E-09	8.31E-08	SERINC5
ENSG00000166979	401.127145	95.66159463	2.065280114	9.19E-09	8.31E-08	EVA1C
ENSG00000278970	161.3751619	38.75479565	2.061168298	9.23E-09	8.35E-08	HEIH
ENSG00000131196	448.5088337	152.944441	1.550535078	9.24E-09	8.36E-08	NFATC1
ENSG00000163170	133.4986622	453.2425635	-1.764275195	9.26E-09	8.37E-08	BOLA3
ENSG00000186106	412.5129496	135.5637152	1.604631767	9.29E-09	8.39E-08	ANKRD46
ENSG00000249565	31.64099163	133.5840064	-2.080484144	9.35E-09	8.44E-08	SERBP1P5
ENSG00000148158	619.8810529	226.0547984	1.456266311	9.40E-09	8.49E-08	SNX30
ENSG00000166396	0	872.2668778	-12.34972082	9.50E-09	8.58E-08	SERPINB7
ENSG00000158813	90.00948136	6.584162767	3.751070772	9.51E-09	8.58E-08	EDA
ENSG00000133121	4867.189953	902.4655487	2.43099234	9.57E-09	8.63E-08	STARD13
ENSG00000159618	87.77394694	0.356539013	7.827386983	9.64E-09	8.69E-08	ADGRG5
ENSG00000178562	62.94200106	1.00940975	5.886927696	9.68E-09	8.73E-08	CD28
ENSG00000071967	5449.949344	1854.856573	1.554953934	9.72E-09	8.76E-08	CYBRD1
ENSG00000281692	63.73498019	0	8.328389092	9.89E-09	8.90E-08	PACRG-AS1
ENSG00000109927	71.11470805	8.386798532	3.095667397	9.89E-09	8.90E-08	TECTA
ENSG00000103876	307.9239966	804.2306237	-1.384747917	9.95E-09	8.95E-08	FAH
ENSG00000116473	2633.735641	1162.501648	1.179983945	1.00E-08	9.01E-08	RAP1A
ENSG00000164946	144.7094973	0.723690511	7.652593874	1.00E-08	9.02E-08	FREM1
ENSG00000154134	1153.604403	378.0100274	1.610283733	1.01E-08	9.10E-08	ROBO3
ENSG00000235636	9.689840462	80.02286494	-3.043849673	1.02E-08	9.16E-08	NUS1P1
ENSG00000164398	99.79531114	1.069617038	6.53587888	1.02E-08	9.18E-08	ACSL6
novel.3	2.153476731	56.99340268	-4.738387141	1.03E-08	9.22E-08	-
ENSG00000126767	512.0586563	1291.633263	-1.334797751	1.03E-08	9.25E-08	ELK1
ENSG00000056972	102.8001472	654.590311	-2.670484606	1.04E-08	9.29E-08	TRAF3IP2
ENSG00000170837	90.9265008	1.702418679	5.69310928	1.04E-08	9.29E-08	GPR27
ENSG00000122218	3268.17947	8377.975858	-1.35807508	1.04E-08	9.32E-08	COPA
ENSG00000163629	1586.135116	558.912268	1.504659455	1.05E-08	9.37E-08	PTPN13
ENSG00000213516	861.733675	316.4687817	1.445095768	1.05E-08	9.37E-08	RBMXL1
ENSG00000111664	38.19057378	138.0911501	-1.854062604	1.05E-08	9.37E-08	GNB3
ENSG00000105568	2726.863723	7291.136761	-1.418916348	1.05E-08	9.38E-08	PPP2R1A
ENSG00000139973	0.308355904	58.96789285	-7.503006093	1.05E-08	9.42E-08	SYT16
ENSG00000137098	75.72124187	12.44335075	2.599451627	1.05E-08	9.42E-08	SPAG8
ENSG00000163762	188.4375128	12.14586315	3.953458291	1.05E-08	9.42E-08	TM4SF18
ENSG00000286710	46.98070424	0	7.887189216	1.07E-08	9.55E-08	AC188617.1
ENSG00000257167	15.79656958	87.39366962	-2.470634162	1.08E-08	9.65E-08	TMPO-AS1

ENSG00000107864	162.1047027	19.16098073	3.07012908	1.08E-08	9.69E-08	CPEB3
ENSG00000169684	3.064683035	46.68483808	-3.939930399	1.09E-08	9.70E-08	CHRNA5
ENSG00000168743	62.02585143	0.336469917	7.326282609	1.09E-08	9.72E-08	NPNT
ENSG00000172939	872.8907592	2833.013009	-1.698313156	1.09E-08	9.76E-08	OXSR1
ENSG00000243279	201.8307165	474.242033	-1.232462964	1.10E-08	9.79E-08	PRAF2
ENSG00000176986	473.2343612	1301.832911	-1.459842341	1.10E-08	9.81E-08	SEC24C
ENSG00000186076	30.1936634	183.2113222	-2.602811856	1.10E-08	9.84E-08	AC012085.1
ENSG00000106823	974.6204186	24.82906276	5.290681582	1.12E-08	9.96E-08	ECM2
ENSG00000153234	1856.993653	298.1349663	2.638664376	1.12E-08	9.96E-08	NR4A2
ENSG00000135249	175.9465139	417.2505094	-1.246275341	1.12E-08	1.00E-07	RINT1
ENSG00000105991	9.097090523	74.89943979	-3.04253011	1.15E-08	1.02E-07	HOXA1
ENSG00000188511	41.1908931	0	7.697956744	1.16E-08	1.04E-07	C22orf34
ENSG00000135750	60.18567428	321.2600224	-2.415896587	1.17E-08	1.04E-07	KCNK1
ENSG00000196381	73.3995617	6.961926751	3.37716161	1.17E-08	1.04E-07	ZNF781
ENSG00000147027	1213.598182	310.2893869	1.966998889	1.19E-08	1.05E-07	TMEM47
ENSG00000135426	69.61801644	0	8.454726058	1.19E-08	1.06E-07	TESPA1
ENSG00000168040	123.4711334	355.3395912	-1.525861492	1.19E-08	1.06E-07	FADD
ENSG00000102001	45.27871388	1.41669944	4.983081633	1.19E-08	1.06E-07	CACNA1F
ENSG00000174989	226.1059126	525.7052685	-1.217242433	1.20E-08	1.06E-07	FBXW8
ENSG00000178531	34.65141141	170.9821815	-2.303965593	1.22E-08	1.08E-07	CTXN1
ENSG00000174332	6.776307989	169.4586337	-4.650578356	1.22E-08	1.08E-07	GLIS1
ENSG00000165655	2122.460758	394.7524418	2.426491448	1.22E-08	1.08E-07	ZNF503
ENSG00000225964	68.22792983	1.130980201	5.969648023	1.22E-08	1.08E-07	NRIR
ENSG00000135631	418.3650159	1954.236869	-2.223733753	1.23E-08	1.09E-07	RAB11FIP5
ENSG00000132199	281.5137291	1019.963454	-1.857263348	1.24E-08	1.10E-07	ENOSF1
ENSG00000105438	1831.834592	5684.555254	-1.633758444	1.24E-08	1.10E-07	KDELR1
ENSG00000135047	1996.049107	7349.446237	-1.880482766	1.27E-08	1.13E-07	CTSL
ENSG00000148200	87.94061235	12.94152931	2.765897446	1.29E-08	1.14E-07	NR6A1
ENSG00000196313	433.3230636	1024.083295	-1.240535152	1.29E-08	1.14E-07	POM121
ENSG00000130226	87.53546789	0.387220594	7.823310789	1.29E-08	1.14E-07	DPP6
ENSG00000271936	4.193554905	55.35162365	-3.715793608	1.30E-08	1.15E-07	AC012073.1
ENSG00000136274	704.8408074	152.3175815	2.211932375	1.31E-08	1.16E-07	NACAD
ENSG00000111358	465.8643347	980.9320359	-1.074378251	1.31E-08	1.16E-07	GTF2H3
ENSG00000159082	441.2329737	162.8105392	1.437407032	1.33E-08	1.18E-07	SYNJ1
ENSG00000026751	104.2148368	0	9.036925357	1.33E-08	1.18E-07	SLAMF7
ENSG00000158864	1095.423786	2550.726551	-1.219441243	1.35E-08	1.19E-07	NDUFS2
ENSG00000105374	124.482802	3.637365776	5.104125097	1.36E-08	1.20E-07	NKG7
ENSG00000132840	506.2167885	75.46424699	2.743154231	1.36E-08	1.20E-07	BHMT2
ENSG00000175899	8982.261704	617.82039	3.861515567	1.36E-08	1.20E-07	A2M
ENSG00000075415	4271.99766	9406.241728	-1.138736549	1.38E-08	1.21E-07	SLC25A3
novel.160	99.42041275	14.7774152	2.739696426	1.38E-08	1.22E-07	-
ENSG00000175352	19.52449197	205.6099434	-3.397818156	1.38E-08	1.22E-07	NRIP3
ENSG00000220205	1728.735381	683.3768432	1.33940176	1.38E-08	1.22E-07	VAMP2
ENSG00000196074	197.149948	20.73682027	3.253283134	1.38E-08	1.22E-07	SYCP2
novel.223	38.30243395	0	7.593538326	1.39E-08	1.22E-07	-
ENSG00000227517	0	46.65214872	-8.127136773	1.39E-08	1.23E-07	LINC01483
ENSG00000224660	521.1616197	103.0752688	2.337911668	1.39E-08	1.23E-07	SH3BP5-AS1
ENSG00000100749	160.9977127	520.2172604	-1.692700024	1.41E-08	1.24E-07	VRK1
ENSG00000046653	21808.50135	535.8954298	5.346582828	1.42E-08	1.25E-07	GPM6B
ENSG00000228577	120.5235546	0.356539013	8.285508278	1.43E-08	1.26E-07	AC010731.2
ENSG00000273344	216.8743253	76.57356985	1.502668799	1.44E-08	1.27E-07	PAXIP1-AS1

ENSG00000117569	961.6912509	240.9358171	1.997343464	1.45E-08	1.27E-07	PTBP2
ENSG00000146215	36.77820552	0	7.535048402	1.45E-08	1.27E-07	CRIP3
ENSG00000178966	90.23048059	279.0709106	-1.629923243	1.45E-08	1.28E-07	RMI1
ENSG00000260805	73.42205645	8.040872005	3.203475632	1.46E-08	1.28E-07	AC092803.2
ENSG00000266524	106.0896888	0	9.062653277	1.46E-08	1.28E-07	GDF10
ENSG00000112214	51.91976965	0	8.031482728	1.46E-08	1.28E-07	FHL5
ENSG00000237181	47.61983953	2.527610546	4.241965486	1.46E-08	1.28E-07	AC147651.4
ENSG00000163884	419.7435241	45.6014449	3.198049347	1.47E-08	1.29E-07	KLF15
ENSG00000198753	4226.122105	304.0808738	3.797251828	1.49E-08	1.30E-07	PLXNB3
ENSG00000168487	1440.24369	3452.927426	-1.261464452	1.50E-08	1.31E-07	BMP1
ENSG00000243444	14.74674921	82.77855597	-2.484478603	1.52E-08	1.33E-07	PALM2
ENSG00000148606	401.0014672	1086.117116	-1.43713544	1.53E-08	1.33E-07	POLR3A
ENSG00000189190	163.0602575	39.93909506	2.027512645	1.53E-08	1.34E-07	ZNF600
ENSG00000285756	75.34937681	287.6708447	-1.933409809	1.54E-08	1.35E-07	BX890604.2
ENSG00000137601	1295.268675	413.1438485	1.64801487	1.54E-08	1.35E-07	NEK1
ENSG00000168685	94.87644665	1154.485267	-3.605509251	1.54E-08	1.35E-07	IL7R
ENSG00000237484	38.34806385	0	7.594257673	1.54E-08	1.35E-07	LINC01684
ENSG00000160224	69.6610403	0.774441189	6.586331442	1.54E-08	1.35E-07	AIRE
ENSG00000287792	39.4538715	0	7.636364877	1.55E-08	1.36E-07	AC140118.2
ENSG00000260442	8.47529285	59.50710869	-2.811799259	1.56E-08	1.36E-07	ATP2A1-AS1
ENSG00000173681	453.8607076	169.1900298	1.423996541	1.56E-08	1.36E-07	BCLAF3
ENSG00000142156	9842.677741	78531.89974	-2.996157455	1.56E-08	1.36E-07	COL6A1
ENSG00000196584	27.44596961	226.3130937	-3.043161514	1.56E-08	1.36E-07	XRCC2
ENSG00000113273	346.018466	863.5994786	-1.319865898	1.59E-08	1.38E-07	ARSB
ENSG00000169855	191.9787976	1324.17484	-2.786266143	1.59E-08	1.39E-07	ROBO1
ENSG00000167984	135.9882886	18.1703558	2.900424713	1.60E-08	1.39E-07	NLRC3
ENSG00000162367	124.5907041	0.743759607	7.426625711	1.61E-08	1.40E-07	TAL1
ENSG00000143179	372.5297657	1142.870241	-1.617602748	1.61E-08	1.40E-07	UCK2
ENSG00000185986	306.3808925	34.38941983	3.149005088	1.61E-08	1.40E-07	SDHAP3
ENSG00000215218	83.95486385	1.854670712	5.528845156	1.63E-08	1.42E-07	UBE2QL1
ENSG00000104154	525.6355355	176.5591178	1.574375241	1.65E-08	1.43E-07	SLC30A4
ENSG00000145284	1247.018404	307.8607439	2.018984649	1.65E-08	1.43E-07	SCD5
ENSG00000137124	202.0217273	758.0873726	-1.908443659	1.65E-08	1.43E-07	ALDH1B1
ENSG00000148634	1059.588858	2374.706636	-1.164128197	1.65E-08	1.44E-07	HERC4
ENSG00000175395	658.9966951	235.6072399	1.484058075	1.67E-08	1.45E-07	ZNF25
ENSG00000167766	1603.12797	598.5767233	1.421669483	1.67E-08	1.45E-07	ZNF83
ENSG00000117519	9640.094038	1995.06818	2.272472603	1.68E-08	1.46E-07	CNN3
ENSG00000237517	37.32979851	0	7.555724887	1.69E-08	1.47E-07	DGCR5
ENSG00000107789	235.7175562	675.1603234	-1.518747561	1.69E-08	1.47E-07	MINPP1
ENSG00000143761	5876.43528	12553.9141	-1.095115835	1.71E-08	1.48E-07	ARF1
ENSG00000169385	45.59605914	0	7.844766671	1.71E-08	1.48E-07	RNASE2
ENSG00000136205	15551.53987	2695.536197	2.528510892	1.72E-08	1.49E-07	TNS3
ENSG00000225746	64.88748576	286.5938601	-2.142064173	1.72E-08	1.49E-07	MEG8
ENSG00000015532	552.2843927	2179.121718	-1.980245608	1.72E-08	1.49E-07	XYLT2
ENSG00000167703	75.07193485	4.1272436	4.217549563	1.72E-08	1.49E-07	SLC43A2
ENSG00000084453	39.17115839	0	7.625170869	1.74E-08	1.50E-07	SLCO1A2
novel.322	81.22644586	0	8.677758532	1.75E-08	1.51E-07	-
ENSG00000251144	0	67.0138079	-8.646361395	1.75E-08	1.51E-07	AC113346.1
ENSG00000095380	463.4749572	1209.129537	-1.383356271	1.76E-08	1.52E-07	NANS
ENSG00000173727	54.19979433	231.5485046	-2.093948153	1.76E-08	1.52E-07	AP000769.1
ENSG00000102445	435.8322505	14.02034617	4.968244575	1.77E-08	1.52E-07	RUBCNL

ENSG00000088881	441.8284879	34.49254378	3.685292445	1.77E-08	1.52E-07	EBF4
ENSG00000198785	119.3797933	0.336469917	8.27109864	1.77E-08	1.53E-07	GRIN3A
ENSG00000180787	265.8567147	45.46206992	2.54383368	1.77E-08	1.53E-07	ZFP3
ENSG00000172915	383.0312916	89.42775914	2.099546799	1.78E-08	1.53E-07	NBEA
ENSG00000279881	0.925067711	54.35483417	-5.895715765	1.78E-08	1.54E-07	AC041040.1
ENSG00000026297	1928.418252	329.3710032	2.548836814	1.78E-08	1.54E-07	RNASET2
ENSG00000108602	62.83437255	2.618499415	4.621587688	1.79E-08	1.54E-07	ALDH3A1
ENSG00000179091	1537.359378	3285.581905	-1.095654301	1.79E-08	1.54E-07	CYC1
ENSG00000118263	319.5648342	1230.014075	-1.944380355	1.79E-08	1.54E-07	KLF7
ENSG00000121310	1133.319657	277.7267003	2.028012192	1.80E-08	1.55E-07	ECHDC2
ENSG00000126602	603.5924964	1594.303992	-1.401158469	1.81E-08	1.56E-07	TRAP1
novel.1004	1.536764924	61.47851674	-5.335668634	1.83E-08	1.57E-07	-
ENSG00000105738	250.3985455	1452.6695	-2.536311024	1.83E-08	1.58E-07	SIPA1L3
ENSG00000049089	474.0656741	70.4754321	2.752571874	1.84E-08	1.58E-07	COL9A2
novel.1017	68.12742341	1.682349583	5.2779404	1.85E-08	1.59E-07	-
ENSG00000163947	411.8058315	81.9325715	2.326931439	1.90E-08	1.63E-07	ARHGEF3
ENSG00000134452	830.2999189	1641.960306	-0.983660817	1.90E-08	1.63E-07	FBH1
ENSG00000186231	68.66522627	0.336469917	7.47292775	1.94E-08	1.67E-07	KLHL32
ENSG00000088387	1198.796123	215.8016548	2.472917195	1.96E-08	1.68E-07	DOCK9
ENSG00000196220	215.2967313	27.73631667	2.964274304	1.99E-08	1.71E-07	SRGAP3
ENSG00000068120	672.7938286	1659.516308	-1.302451638	2.02E-08	1.73E-07	COASY
ENSG00000175550	2494.862652	6061.645353	-1.280728031	2.02E-08	1.73E-07	DRAP1
ENSG00000143862	2074.999938	730.0084905	1.507488786	2.02E-08	1.73E-07	ARL8A
ENSG00000120306	739.5549131	315.9705219	1.226436434	2.04E-08	1.75E-07	CYSTM1
ENSG00000109654	1151.688008	342.8502757	1.747894373	2.06E-08	1.76E-07	TRIM2
ENSG00000100033	69.88411991	0	8.461147126	2.06E-08	1.77E-07	PRODH
ENSG00000186409	99.02659456	18.06872605	2.458032084	2.10E-08	1.80E-07	CCDC30
novel.1099	37.64413792	0	7.568654135	2.13E-08	1.82E-07	-
ENSG00000132530	635.6814395	259.0619248	1.294652242	2.13E-08	1.83E-07	XAF1
ENSG00000142303	674.4119389	288.6619836	1.225025003	2.13E-08	1.83E-07	ADAMTS10
ENSG00000279660	33.66344745	0	7.406614481	2.14E-08	1.83E-07	AC005703.6
ENSG00000121316	515.8358381	22.14791591	4.536313015	2.14E-08	1.83E-07	PLBD1
ENSG00000180767	39.92515935	0	7.652370878	2.14E-08	1.83E-07	CHST13
ENSG00000188641	817.4797349	290.347976	1.492336665	2.15E-08	1.84E-07	DPYD
ENSG00000259834	49.88323861	0	7.97363655	2.16E-08	1.85E-07	AL365361.1
ENSG00000110975	102.1210533	0	9.008159302	2.19E-08	1.87E-07	SYT10
novel.210	0	533.4220434	-11.64020923	2.24E-08	1.92E-07	-
ENSG00000151623	196.2828888	8.8591758	4.451893139	2.25E-08	1.92E-07	NR3C2
ENSG00000206172	38.0747392	0	7.584698633	2.26E-08	1.92E-07	HBA1
ENSG00000103152	468.3546453	1251.551339	-1.417787564	2.27E-08	1.93E-07	MPG
ENSG00000154146	40.25572493	397.4140894	-3.302873226	2.31E-08	1.97E-07	NRGN
ENSG00000111897	8645.540432	3800.233396	1.185851252	2.31E-08	1.97E-07	SERINC1
ENSG00000170011	50.80057096	2.079026788	4.574589485	2.31E-08	1.97E-07	MYRIP
ENSG00000261799	301.6470988	106.6911528	1.500768559	2.32E-08	1.97E-07	AC007406.5
ENSG00000280241	0.308355904	66.35520497	-7.669812153	2.32E-08	1.97E-07	AC079298.3
ENSG00000113712	1071.454626	2288.811854	-1.095029322	2.33E-08	1.98E-07	CSNK1A1
ENSG00000104611	19.32577368	324.75157	-4.073263727	2.34E-08	1.99E-07	SH2D4A
ENSG00000136244	24.70433026	5438.066619	-7.781535176	2.37E-08	2.01E-07	IL6
ENSG00000147676	156.0671941	0.387220594	8.657801781	2.37E-08	2.02E-07	MAL2
ENSG00000167815	1120.930284	2739.76778	-1.289341833	2.39E-08	2.03E-07	PRDX2
ENSG00000019549	131.5725514	3056.473252	-4.538228478	2.40E-08	2.04E-07	SNAI2

ENSG00000161653	17.1446454	241.6214115	-3.818649255	2.42E-08	2.05E-07	NAGS
ENSG00000115523	41.37073539	0	7.704488675	2.43E-08	2.06E-07	GNLY
ENSG00000152520	1287.140761	441.8265767	1.543136181	2.43E-08	2.06E-07	PAN3
ENSG00000233672	47.36671579	4.01512976	3.576718255	2.43E-08	2.06E-07	RNASEH2B-AS1
ENSG00000141744	61.29585578	0	8.27207973	2.43E-08	2.06E-07	PNMT
ENSG00000147255	63.3611773	0.774441189	6.449964458	2.44E-08	2.07E-07	IGSF1
ENSG00000171631	146.044723	23.91730155	2.613412391	2.47E-08	2.09E-07	P2RY6
ENSG00000196378	232.4015881	58.41267066	1.993881539	2.47E-08	2.09E-07	ZNF34
ENSG00000173327	5013.390571	1675.041018	1.581747065	2.48E-08	2.10E-07	MAP3K11
ENSG00000232389	23.08503764	95.58319763	-2.051191153	2.52E-08	2.13E-07	AL583856.1
ENSG00000117450	1834.620994	5054.317654	-1.462122767	2.52E-08	2.13E-07	PRDX1
ENSG00000068878	673.4335059	1395.627309	-1.051239529	2.54E-08	2.15E-07	PSME4
novel.39	0	38.53557665	-7.85152724	2.55E-08	2.15E-07	-
ENSG00000184293	45.8670187	0	7.852497395	2.55E-08	2.15E-07	CLECL1
ENSG00000021826	83.04008223	330.747248	-1.993810356	2.55E-08	2.15E-07	CPS1
ENSG00000273038	36.15669258	165.1792196	-2.191908962	2.57E-08	2.17E-07	AL365203.2
ENSG00000225614	245.4745951	834.9550177	-1.766267558	2.58E-08	2.18E-07	ZNF469
ENSG00000215483	245.3928544	11.03841967	4.472725203	2.59E-08	2.19E-07	LINC00598
ENSG00000156453	1007.030055	67.17334601	3.903416927	2.60E-08	2.19E-07	PCDH1
ENSG00000008311	2306.906244	447.9077274	2.364698534	2.60E-08	2.19E-07	AASS
ENSG00000185085	281.9705861	773.5611235	-1.456190761	2.64E-08	2.23E-07	INTS5
ENSG00000155561	756.9628703	1791.26411	-1.242475419	2.65E-08	2.24E-07	NUP205
ENSG00000165934	782.5300399	1602.062423	-1.033808121	2.66E-08	2.24E-07	CPSF2
ENSG00000116171	3329.217325	1317.719667	1.336905727	2.69E-08	2.26E-07	SCP2
ENSG00000065268	588.7946601	1402.389869	-1.252076997	2.69E-08	2.26E-07	WDR18
ENSG00000164062	789.6384793	1972.443424	-1.320797785	2.69E-08	2.26E-07	APEH
ENSG00000075151	1578.629777	3555.102499	-1.171151063	2.69E-08	2.26E-07	EIF4G3
ENSG00000124243	76.96909636	282.9902243	-1.878845021	2.71E-08	2.28E-07	BCAS4
ENSG00000271913	96.65190513	25.54890046	1.921026396	2.71E-08	2.28E-07	AL035530.2
ENSG00000177640	111.364592	27.2927416	2.022331327	2.72E-08	2.29E-07	CASC2
ENSG00000186716	504.2983034	1237.794554	-1.295151027	2.73E-08	2.30E-07	BCR
ENSG00000167088	617.5607259	1494.38568	-1.275080855	2.74E-08	2.31E-07	SNRPD1
ENSG00000143256	752.0290455	1715.080823	-1.189447653	2.77E-08	2.32E-07	PFDN2
ENSG00000048140	619.8081828	1483.794466	-1.259605008	2.77E-08	2.33E-07	TSPAN17
ENSG00000205208	150.1036107	402.2738159	-1.422268113	2.77E-08	2.33E-07	C4orf46
ENSG00000276248	14.7328878	89.26707797	-2.594873055	2.78E-08	2.33E-07	AL442125.1
ENSG00000102007	727.7335372	2107.160653	-1.53376121	2.81E-08	2.36E-07	PLP2
ENSG00000112599	85.49898064	13.45147623	2.676248204	2.81E-08	2.36E-07	GUCA1B
ENSG00000246898	100.6544493	9.509606392	3.394880516	2.82E-08	2.37E-07	LINC00920
ENSG00000196689	311.7183635	83.83144315	1.897203509	2.82E-08	2.37E-07	TRPV1
ENSG00000164414	344.6872296	137.0736152	1.32969729	2.87E-08	2.40E-07	SLC35A1
ENSG00000138430	781.532644	1829.818558	-1.227511984	2.88E-08	2.42E-07	OLA1
ENSG00000164125	6771.680852	259.1510411	4.707112504	2.89E-08	2.42E-07	GASK1B
ENSG00000145708	110.1979674	1.426156051	6.265044136	2.96E-08	2.48E-07	CRHBP
ENSG00000177453	411.0830618	33.34992361	3.628355473	2.96E-08	2.48E-07	NIM1K
ENSG00000150403	1256.01023	3006.237076	-1.259234756	2.96E-08	2.48E-07	TMCO3
ENSG00000174130	277.3406327	42.33709121	2.706489727	2.97E-08	2.48E-07	TLR6
ENSG00000105556	246.6665061	727.4695783	-1.559968072	3.02E-08	2.53E-07	MIER2
ENSG00000108231	71.68972127	0.693008929	6.649163144	3.03E-08	2.54E-07	LGI1
ENSG00000136159	176.4995031	634.4301513	-1.846325481	3.04E-08	2.54E-07	NUDT15
ENSG00000156709	421.1803361	1035.814352	-1.298430387	3.05E-08	2.54E-07	AIFM1

ENSG00000037474	942.2330146	1990.342829	-1.078963949	3.05E-08	2.55E-07	NSUN2
novel.630	8.732035341	83.75900077	-3.260908405	3.05E-08	2.55E-07	-
ENSG00000238273	10.05740294	82.73307077	-3.0451812	3.05E-08	2.55E-07	AC108058.1
ENSG00000167264	136.84244	401.6995181	-1.554241192	3.05E-08	2.55E-07	DUS2
ENSG00000118707	737.9499258	279.2793887	1.401495123	3.06E-08	2.55E-07	TGIF2
ENSG00000286593	34.86796587	0	7.457405847	3.06E-08	2.55E-07	AP006261.1
ENSG00000007908	57.38156525	0	8.175940582	3.07E-08	2.56E-07	SELE
ENSG00000111530	1500.48323	3062.942409	-1.029472471	3.14E-08	2.61E-07	CAND1
ENSG00000138442	333.912993	1048.736	-1.651496563	3.14E-08	2.61E-07	WDR12
ENSG00000100147	20.21907289	105.6974926	-2.385850322	3.19E-08	2.66E-07	CCDC134
ENSG00000075420	1206.76171	3458.419401	-1.519003456	3.21E-08	2.67E-07	FNDC3B
ENSG00000167460	10772.10161	28442.20601	-1.400730862	3.23E-08	2.69E-07	TPM4
ENSG00000115183	2375.724328	529.2307644	2.166639803	3.25E-08	2.70E-07	TANC1
ENSG00000198018	166.337192	437.9863834	-1.397162746	3.25E-08	2.70E-07	ENTPD7
ENSG00000152402	47.35690007	5.064677702	3.231690031	3.26E-08	2.71E-07	GUCY1A2
ENSG00000113391	918.6481124	361.307635	1.345932464	3.27E-08	2.72E-07	FAM172A
ENSG00000027697	2449.801419	1070.176511	1.194964654	3.31E-08	2.75E-07	IFNGR1
ENSG00000107560	788.9447978	333.838591	1.240785676	3.31E-08	2.75E-07	RAB11FIP2
ENSG00000049541	332.4412478	810.8629471	-1.286504823	3.35E-08	2.78E-07	RFC2
ENSG00000182179	2275.571403	827.8974893	1.458410635	3.36E-08	2.79E-07	UBA7
ENSG00000213553	583.1743607	1846.331235	-1.662885943	3.37E-08	2.80E-07	RPLP0P6
ENSG00000168273	97.90044381	249.3052434	-1.348697167	3.38E-08	2.80E-07	SMIM4
ENSG00000138674	2517.435455	6440.200975	-1.35517343	3.42E-08	2.83E-07	SEC31A
ENSG00000196295	1136.979783	346.4552432	1.714758993	3.42E-08	2.83E-07	GARS-DT
ENSG00000116741	628.7014399	99.2341768	2.661311099	3.43E-08	2.84E-07	RGS2
ENSG00000136297	37.78429038	0	7.572866183	3.46E-08	2.87E-07	MMD2
ENSG00000176472	115.581824	30.78718356	1.907731208	3.49E-08	2.89E-07	ZNF575
ENSG00000169083	45.82299845	260.1008602	-2.506626278	3.49E-08	2.89E-07	AR
ENSG00000186575	274.0056739	1007.665971	-1.878716136	3.53E-08	2.92E-07	NF2
ENSG00000130725	1142.953664	2481.086654	-1.118074274	3.54E-08	2.93E-07	UBE2M
ENSG00000077235	1267.020696	2559.816735	-1.01451056	3.54E-08	2.93E-07	GTF3C1
ENSG00000124256	50.54028099	0	7.992538801	3.56E-08	2.95E-07	ZBP1
ENSG00000149489	217.0903378	70.13464265	1.630532453	3.58E-08	2.96E-07	ROM1
ENSG00000197376	35.78383077	0	7.495688858	3.59E-08	2.96E-07	AC089987.1
ENSG00000146433	823.441127	1624.363139	-0.980022529	3.60E-08	2.97E-07	TMEM181
ENSG00000137996	534.1654031	1266.435884	-1.245698892	3.61E-08	2.98E-07	RTCA
ENSG00000279875	44.82436456	0	7.820352938	3.62E-08	2.99E-07	AC074029.4
ENSG00000180113	82.90719511	3.067083173	4.804042508	3.62E-08	2.99E-07	TDRD6
ENSG00000111404	46.82364144	0	7.882300069	3.68E-08	3.03E-07	RERGL
ENSG00000130826	563.3779131	1552.495387	-1.462597483	3.73E-08	3.08E-07	DKC1
ENSG00000121742	1035.168375	0	12.34934098	3.75E-08	3.09E-07	GJB6
ENSG00000249249	58.63462067	5.330327845	3.4526918	3.75E-08	3.09E-07	AC010226.1
ENSG00000162241	211.2934314	46.56198336	2.183280088	3.75E-08	3.09E-07	SLC25A45
ENSG00000020426	224.5485477	531.7369532	-1.244168332	3.75E-08	3.09E-07	MNAT1
ENSG00000135480	2.14337627	2497.00532	-10.18863784	3.76E-08	3.10E-07	KRT7
ENSG00000144668	129.4179357	9.680771646	3.740013765	3.80E-08	3.13E-07	ITGA9
ENSG00000183773	52.57889205	2.894762044	4.190562167	3.80E-08	3.13E-07	AIFM3
ENSG00000102796	193.301959	33.59922907	2.526594308	3.82E-08	3.14E-07	DHRS12
ENSG00000131876	493.1595532	1258.913402	-1.352077429	3.83E-08	3.15E-07	SNRPA1
ENSG00000170545	56.28181238	421.2231896	-2.904900817	3.86E-08	3.17E-07	SMAGP
ENSG00000174175	41.6402991	0	7.713366876	3.86E-08	3.17E-07	SELP

ENSG00000238142	120.1653927	17.27537164	2.799347637	3.89E-08	3.20E-07	BX284668.5
ENSG00000187037	34.09021651	0	7.424414599	3.90E-08	3.20E-07	GPR141
ENSG00000167840	197.5040329	58.71961487	1.751629446	3.91E-08	3.21E-07	ZNF232
novel.405	108.5149953	1.060160428	6.663429298	3.93E-08	3.22E-07	-
ENSG00000076043	646.7791033	1611.414492	-1.317059411	3.96E-08	3.25E-07	REXO2
ENSG00000055163	767.6161108	161.8948908	2.243881573	3.96E-08	3.25E-07	CYFIP2
ENSG00000109917	545.4469563	1311.645764	-1.26561995	4.00E-08	3.28E-07	ZPR1
ENSG00000164088	882.0917597	382.3639857	1.205763735	4.01E-08	3.28E-07	PPM1M
ENSG00000220008	59.2912636	1.793307549	5.045470832	4.03E-08	3.31E-07	LINGO3
ENSG00000138080	1509.564171	8099.538749	-2.423747292	4.13E-08	3.38E-07	EMILIN1
ENSG00000237149	139.3115098	41.68280781	1.737402634	4.14E-08	3.39E-07	ZNF503-AS2
ENSG00000254838	99.58767511	9.487097151	3.399712647	4.15E-08	3.40E-07	GVINP1
ENSG00000151929	597.1718948	2158.402534	-1.853962796	4.16E-08	3.40E-07	BAG3
ENSG00000164303	158.9223457	0.713078025	7.792383889	4.16E-08	3.40E-07	ENPP6
ENSG00000132465	170.1787532	0.356539013	8.782701266	4.16E-08	3.40E-07	JCHAIN
ENSG00000213551	209.1780352	634.9495548	-1.602380137	4.17E-08	3.41E-07	DNAJC9
ENSG00000143653	3080.573145	1011.869442	1.605891321	4.17E-08	3.41E-07	SCCPDH
ENSG00000100983	673.2856883	1682.566202	-1.321415467	4.19E-08	3.42E-07	GSS
ENSG00000109686	4663.242252	1250.615098	1.898460128	4.22E-08	3.45E-07	SH3D19
ENSG00000128165	6.398645048	137.2219801	-4.425749753	4.23E-08	3.46E-07	ADM2
ENSG00000174996	425.416061	1060.212717	-1.317098146	4.23E-08	3.46E-07	KLC2
ENSG00000185666	50.78502863	1.00940975	5.57697744	4.25E-08	3.47E-07	SYN3
ENSG00000234420	925.9513406	298.5756688	1.633100288	4.27E-08	3.48E-07	ZNF37BP
ENSG00000138449	1157.90479	70.15411663	4.043231384	4.28E-08	3.49E-07	SLC40A1
ENSG00000156968	58.44313953	2.527610546	4.537689468	4.29E-08	3.50E-07	MPV17L
ENSG00000165917	68.04976811	5.411760104	3.655684347	4.30E-08	3.51E-07	RAPSN
ENSG00000073905	39.72859235	139.5408901	-1.813123741	4.39E-08	3.57E-07	VDAC1P1
ENSG00000149177	2880.26772	765.1454405	1.912229084	4.39E-08	3.57E-07	PTPRJ
ENSG00000179598	187.4897094	44.5656731	2.073135393	4.39E-08	3.58E-07	PLD6
ENSG00000005448	253.7311551	948.9867616	-1.902583909	4.41E-08	3.59E-07	WDR54
ENSG00000167004	4645.694103	14259.89888	-1.617986716	4.42E-08	3.59E-07	PDIA3
ENSG00000077232	2136.508799	4268.961885	-0.998572914	4.42E-08	3.59E-07	DNAJC10
ENSG00000101871	1431.151877	401.5813677	1.832956494	4.46E-08	3.63E-07	MID1
ENSG00000160256	211.8276592	1009.858131	-2.253520526	4.46E-08	3.63E-07	FAM207A
ENSG00000230897	1231.681241	3039.864434	-1.303417269	4.47E-08	3.63E-07	RPS18P12
ENSG00000256269	279.2587499	772.2010137	-1.467765284	4.47E-08	3.64E-07	HMBS
ENSG00000164181	249.2653564	13.9295857	4.166488158	4.52E-08	3.68E-07	ELOVL7
ENSG00000148834	1142.224636	3710.706635	-1.699752022	4.53E-08	3.68E-07	GSTO1
ENSG00000123570	67.95886431	8.692586867	2.972712888	4.56E-08	3.70E-07	RAB9B
ENSG00000232977	91.2394722	11.57802069	2.966643576	4.57E-08	3.71E-07	LINC00327
ENSG00000143434	489.0591797	129.1199889	1.921759682	4.60E-08	3.73E-07	SEMA6C
ENSG00000179195	2842.689606	1110.493673	1.356055992	4.67E-08	3.79E-07	ZNF664
ENSG00000171951	146.8718632	14.3901946	3.342972774	4.71E-08	3.82E-07	SCG2
ENSG00000039123	707.8110079	1563.236328	-1.14323924	4.72E-08	3.83E-07	MTREX
ENSG00000196781	2864.826614	667.3654738	2.102312222	4.73E-08	3.83E-07	TLE1
ENSG00000112425	314.0197718	83.98523624	1.902914745	4.76E-08	3.86E-07	EPM2A
ENSG00000118564	2105.194611	1110.409801	0.922868408	4.79E-08	3.88E-07	FBXL5
ENSG00000261308	60.41175938	0.356539013	7.289239968	4.85E-08	3.93E-07	FIGNL2
ENSG00000167601	1954.114734	4864.27206	-1.3156224	4.86E-08	3.94E-07	AXL
ENSG00000196126	53.50338994	0	8.075362432	4.88E-08	3.95E-07	HLA-DRB1
ENSG00000174944	359.9280566	0	10.82524481	4.92E-08	3.98E-07	P2RY14

ENSG00000124491	1208.479079	0	12.572647	4.96E-08	4.01E-07	F13A1
ENSG00000103175	79.6077227	3.52640781	4.484100749	4.97E-08	4.02E-07	WFDC1
ENSG00000114812	54.76002033	0.743759607	6.246946142	4.99E-08	4.03E-07	VIPR1
ENSG00000165113	86.63637115	15.23661144	2.499777909	5.03E-08	4.06E-07	GKAP1
ENSG00000064545	435.8466814	857.2398819	-0.975724739	5.03E-08	4.06E-07	TMEM161A
ENSG00000197013	275.8579881	99.03043775	1.479880032	5.06E-08	4.08E-07	ZNF429
ENSG00000142453	551.8557445	1302.067054	-1.238423388	5.06E-08	4.09E-07	CARM1
ENSG00000146067	1710.274548	677.6927202	1.33562981	5.10E-08	4.12E-07	FAM193B
ENSG00000115009	7.083410244	5661.071308	-9.643721643	5.12E-08	4.13E-07	CCL20
ENSG00000257621	1259.910136	445.2182458	1.500648858	5.14E-08	4.14E-07	PSMA3-AS1
ENSG00000108622	69.95019203	2.038888596	5.053243336	5.16E-08	4.16E-07	ICAM2
ENSG00000165775	1570.427055	699.6859099	1.166273304	5.21E-08	4.20E-07	FUNDC2
ENSG00000257226	50.38673784	0	7.989417976	5.27E-08	4.24E-07	AC079584.2
ENSG00000170027	2616.211963	5691.250097	-1.121329981	5.29E-08	4.26E-07	YWHAG
ENSG00000174718	703.547603	204.9793248	1.779402948	5.32E-08	4.28E-07	RESF1
ENSG00000134245	247.7261144	70.51967989	1.812248691	5.33E-08	4.29E-07	WNT2B
ENSG00000157227	7218.250598	62409.48861	-3.112046708	5.35E-08	4.30E-07	MMP14
ENSG00000171617	241.3321832	807.479788	-1.742863861	5.35E-08	4.31E-07	ENC1
ENSG00000101680	120.5599935	1420.768864	-3.559347299	5.37E-08	4.31E-07	LAMA1
ENSG00000153721	1486.959041	202.0305329	2.878732404	5.38E-08	4.33E-07	CNKSR3
ENSG00000171643	30.23872383	0	7.252242939	5.40E-08	4.34E-07	S100Z
ENSG00000132514	63.51880922	0	8.322459559	5.42E-08	4.35E-07	CLEC10A
ENSG00000179564	55.49045886	3.189937893	4.110874226	5.49E-08	4.41E-07	LSMEM2
ENSG00000142669	1623.717847	5537.510048	-1.769910764	5.53E-08	4.44E-07	SH3BGRL3
ENSG00000130529	246.9102841	1005.881338	-2.02651967	5.54E-08	4.45E-07	TRPM4
ENSG00000134324	2256.288786	526.7228034	2.098846978	5.56E-08	4.46E-07	LPIN1
ENSG00000185559	77.380038	0	8.607578294	5.57E-08	4.47E-07	DLK1
ENSG00000007312	42.6548035	0	7.74797647	5.61E-08	4.50E-07	CD79B
ENSG00000136758	1785.447138	3967.267698	-1.151934986	5.62E-08	4.51E-07	YME1L1
ENSG00000168785	745.6095744	1566.699365	-1.071114118	5.66E-08	4.54E-07	TSPAN5
ENSG00000248019	174.2563484	26.3752482	2.726416949	5.69E-08	4.56E-07	FAM13A-AS1
ENSG00000167100	29.92298823	346.1009223	-3.532612261	5.69E-08	4.56E-07	SAMD14
ENSG00000121060	945.5855234	2222.424265	-1.232868748	5.72E-08	4.58E-07	TRIM25
ENSG00000137713	331.5773245	907.6790111	-1.452941343	5.73E-08	4.59E-07	PPP2R1B
ENSG00000173530	418.677432	2189.55267	-2.386676049	5.74E-08	4.59E-07	TNFRSF10D
ENSG00000135144	191.5029082	6.298315133	4.938050665	5.74E-08	4.59E-07	DTX1
ENSG00000164106	102.2792401	1.080229524	6.567933389	5.78E-08	4.62E-07	SCRG1
ENSG00000133816	279.2355967	5893.261229	-4.399656666	5.85E-08	4.68E-07	MICAL2
ENSG00000186642	112.5542798	6.520359459	4.113303139	5.89E-08	4.70E-07	PDE2A
ENSG00000158560	142.5579577	6.960770875	4.342829111	5.92E-08	4.73E-07	DYNC1I1
ENSG00000263535	5.803387812	63.992	-3.467001693	5.93E-08	4.73E-07	AK4P1
ENSG00000054654	1263.0947	356.7251763	1.823641545	5.95E-08	4.75E-07	SYNE2
ENSG00000132388	461.3570095	1213.499651	-1.395264558	5.95E-08	4.75E-07	UBE2G1
ENSG00000234616	442.3580412	1035.261556	-1.22674228	5.98E-08	4.77E-07	JRK
ENSG00000260135	38.00048848	0	7.581356333	6.01E-08	4.79E-07	MMP2-AS1
ENSG00000105640	7528.935185	17443.11549	-1.212145197	6.07E-08	4.84E-07	RPL18A
ENSG00000130787	135.9113888	484.0306674	-1.832510288	6.09E-08	4.85E-07	HIP1R
ENSG00000264985	35.12177379	0	7.46885712	6.09E-08	4.85E-07	AC124804.1
ENSG00000137809	150.1405628	4029.581429	-4.746486359	6.12E-08	4.87E-07	ITGA11
ENSG00000279453	465.3081982	123.2247726	1.919123524	6.20E-08	4.94E-07	Z99129.4
ENSG00000143537	1154.898143	2856.160783	-1.306355233	6.27E-08	4.99E-07	ADAM15

ENSG00000185736	82.60356837	0.336469917	7.739857926	6.28E-08	5.00E-07	ADARB2
ENSG00000152061	1027.384772	327.5365987	1.649120294	6.41E-08	5.10E-07	RABGAP1L
ENSG00000167962	478.6142865	1205.303748	-1.332225202	6.42E-08	5.11E-07	ZNF598
ENSG00000048828	1937.677844	3687.998206	-0.928544797	6.42E-08	5.11E-07	FAM120A
ENSG00000178467	1431.229274	513.6920678	1.478097382	6.43E-08	5.11E-07	P4HTM
ENSG00000132467	542.3163274	1246.339396	-1.20076866	6.46E-08	5.13E-07	UTP3
novel.761	41.57690466	0	7.712243557	6.48E-08	5.15E-07	-
ENSG00000260077	135.7414331	20.44524044	2.730717734	6.49E-08	5.16E-07	AC104794.2
ENSG00000198856	924.8604565	3528.958753	-1.932063877	6.50E-08	5.16E-07	OSTC
ENSG00000119729	3667.208398	1732.192195	1.082265302	6.52E-08	5.17E-07	RHOQ
ENSG00000206195	54.53991838	0	8.10253094	6.53E-08	5.18E-07	DUXAP8
ENSG00000182197	656.6340979	2268.761084	-1.788687752	6.53E-08	5.18E-07	EXT1
ENSG00000136367	129.810501	17.76550626	2.857773136	6.54E-08	5.18E-07	ZFHX2
ENSG00000153790	126.6678775	37.29795801	1.768036241	6.54E-08	5.18E-07	C7orf31
ENSG00000102805	411.7754473	120.4182494	1.772818699	6.59E-08	5.22E-07	CLN5
ENSG00000134851	1311.83751	3154.512426	-1.265711398	6.60E-08	5.23E-07	TMEM165
ENSG00000168661	158.2743799	50.59731061	1.645533157	6.61E-08	5.24E-07	ZNF30
ENSG00000244041	70.17899824	12.96275428	2.438297299	6.63E-08	5.25E-07	LINC01011
ENSG00000100991	1585.339328	3125.689124	-0.979336272	6.63E-08	5.25E-07	TRPC4AP
ENSG00000129472	671.1280522	335.253925	1.001378146	6.64E-08	5.25E-07	RAB2B
ENSG00000162461	107.0056691	15.41021684	2.784864219	6.65E-08	5.26E-07	SLC25A34
ENSG00000171243	105.7970188	0.774441189	7.174075173	6.65E-08	5.26E-07	SOSTDC1
ENSG00000177842	117.4947848	16.11383825	2.85636877	6.67E-08	5.27E-07	ZNF620
ENSG00000161509	172.5249257	5.421216714	4.995026313	6.69E-08	5.29E-07	GRIN2C
ENSG00000111252	1164.459126	5305.985647	-2.187921586	6.71E-08	5.30E-07	SH2B3
ENSG00000198523	75.60876844	1.060160428	6.140487336	6.73E-08	5.32E-07	PLN
ENSG00000142173	10476.62797	117857.4646	-3.49179959	6.78E-08	5.36E-07	COL6A2
ENSG00000167447	196.2398371	467.9956241	-1.254410459	6.79E-08	5.36E-07	SMG8
ENSG00000110448	54.29467254	0	8.095987312	6.82E-08	5.38E-07	CD5
ENSG00000168995	42.74047925	0	7.75076728	6.82E-08	5.38E-07	SIGLEC7
ENSG00000113194	769.6204349	1706.931197	-1.149004392	6.85E-08	5.40E-07	FAF2
ENSG00000135773	51.7959859	1.518200796	5.150851603	6.90E-08	5.44E-07	CAPN9
ENSG00000184164	789.5772803	2751.12645	-1.800718761	6.96E-08	5.49E-07	CRELD2
ENSG00000115339	107.5798994	12.5649212	3.098264004	6.98E-08	5.50E-07	GALNT3
ENSG00000182117	697.1612107	1713.880671	-1.297852486	6.99E-08	5.50E-07	NOP10
ENSG00000224411	26.08041363	101.1451548	-1.956984426	6.99E-08	5.51E-07	HSP90AA2P
ENSG00000070371	165.4456172	406.0902625	-1.295155751	7.02E-08	5.53E-07	CLTCL1
ENSG00000198939	82.3226509	14.15638192	2.531873737	7.06E-08	5.56E-07	ZFP2
ENSG00000124120	263.3565609	812.3517371	-1.624948715	7.07E-08	5.56E-07	TTPAL
ENSG00000146416	821.5599347	394.329227	1.058595958	7.12E-08	5.60E-07	AIG1
ENSG00000226752	745.8267961	244.8576647	1.605881152	7.13E-08	5.60E-07	CUTALP
ENSG00000155980	631.8796265	49.39739002	3.680276631	7.13E-08	5.61E-07	KIF5A
ENSG00000196155	795.4364582	230.1152506	1.789286581	7.14E-08	5.61E-07	PLEKHG4
ENSG00000076351	199.5720035	45.57636712	2.128047199	7.14E-08	5.61E-07	SLC46A1
ENSG00000184995	0.307102255	57.25430093	-7.457162777	7.19E-08	5.64E-07	IFNE
ENSG00000267078	49.19740575	0	7.954946072	7.22E-08	5.67E-07	AC015802.1
ENSG00000136240	2730.2731	8009.210619	-1.55263847	7.24E-08	5.69E-07	KDELR2
ENSG00000269918	59.07309984	5.493192363	3.4420543	7.28E-08	5.71E-07	AF131215.6
ENSG00000161692	149.5684577	445.7499785	-1.57531122	7.32E-08	5.74E-07	DBF4B
novel.255	0	31.29024241	-7.551171501	7.36E-08	5.78E-07	-
ENSG00000189157	43.380726	1.447381022	4.915773104	7.38E-08	5.79E-07	FAM47E

ENSG00000122971	469.9138337	165.3683649	1.506724032	7.40E-08	5.80E-07	ACADS
ENSG00000180801	81.38855074	691.910991	-3.088559274	7.43E-08	5.82E-07	ARSJ
ENSG00000162909	4235.557098	9240.765518	-1.125454433	7.44E-08	5.83E-07	CAPN2
ENSG00000168028	8958.56841	22799.2306	-1.347659903	7.48E-08	5.86E-07	RPSA
ENSG00000159202	1595.406916	3173.315364	-0.992107456	7.48E-08	5.86E-07	UBE2Z
ENSG00000258867	31.65485304	0	7.317474605	7.50E-08	5.87E-07	LINC01146
ENSG00000100298	82.32319253	0.713078025	6.843147407	7.51E-08	5.88E-07	APOBEC3H
ENSG00000144580	641.5732391	1238.621929	-0.948986106	7.53E-08	5.89E-07	CNOT9
ENSG00000147138	72.83030291	0	8.519882224	7.54E-08	5.90E-07	GPR174
ENSG00000074219	216.733374	722.5679876	-1.737495401	7.57E-08	5.92E-07	TEAD2
ENSG00000080819	369.971659	937.8799617	-1.341732234	7.65E-08	5.97E-07	CPOX
ENSG00000256751	65.49488003	1.049547942	5.935427085	7.69E-08	6.00E-07	PLBD1-AS1
ENSG00000232628	59.83691618	1.813376645	5.053132749	7.69E-08	6.00E-07	AC092809.2
novel.123	2.125753916	69.78481614	-5.037667228	7.70E-08	6.01E-07	-
ENSG00000102225	1258.523038	2920.327947	-1.214253209	7.75E-08	6.05E-07	CDK16
ENSG00000180574	29.11744512	110.9130097	-1.930193502	7.75E-08	6.05E-07	EIF2S3B
ENSG00000175315	52.14034161	3.882946823	3.73375951	7.77E-08	6.06E-07	CST6
ENSG00000100592	845.7508394	241.9203587	1.806501949	7.81E-08	6.09E-07	DAAM1
ENSG00000237807	235.2356693	66.26384937	1.824401483	7.81E-08	6.09E-07	AC022034.1
ENSG00000237238	778.3091223	38.47352433	4.34172599	7.83E-08	6.10E-07	BMS1P10
ENSG00000117616	2242.766861	1228.956346	0.867863867	7.84E-08	6.11E-07	RSRP1
ENSG00000138735	1424.393861	236.5106188	2.589626434	7.87E-08	6.13E-07	PDE5A
ENSG00000250934	51.19263695	1.885352294	4.81248238	7.90E-08	6.15E-07	AC016924.1
ENSG00000172465	531.5044965	218.0145343	1.284400588	7.96E-08	6.20E-07	TCEAL1
ENSG00000160211	549.0566345	2082.006529	-1.923145922	8.04E-08	6.26E-07	G6PD
ENSG00000244300	112.6941913	6.176873076	4.167642322	8.07E-08	6.28E-07	GATA2-AS1
ENSG00000204118	29.29603377	0	7.206716377	8.09E-08	6.29E-07	NAP1L6
ENSG00000010932	94.67238629	0	8.898397825	8.12E-08	6.31E-07	FMO1
novel.860	1229.789102	166.2224274	2.888231214	8.19E-08	6.36E-07	-
ENSG00000035499	33.21948332	282.4908083	-3.089277147	8.19E-08	6.36E-07	DEPDC1B
ENSG00000117594	5.238289417	124.1924216	-4.574690622	8.27E-08	6.43E-07	HSD11B1
ENSG00000224594	10.29519816	99.18932107	-3.266699289	8.34E-08	6.47E-07	RPL29P19
ENSG00000134138	115.5378035	393.3463889	-1.767645889	8.34E-08	6.48E-07	MEIS2
ENSG00000119917	3338.075731	1152.950023	1.533655794	8.40E-08	6.52E-07	IFIT3
ENSG00000163347	874.5025554	172.1694818	2.343462969	8.50E-08	6.59E-07	CLDN1
ENSG00000050767	34.15694498	0	7.42715952	8.50E-08	6.59E-07	COL23A1
ENSG00000141367	4269.528031	9654.682679	-1.177163807	8.51E-08	6.60E-07	CLTC
ENSG00000143194	32.96474958	0	7.376704	8.55E-08	6.63E-07	MAEL
ENSG00000180539	43.94485548	4.677457107	3.233948931	8.56E-08	6.64E-07	C9orf139
ENSG00000274712	182.9684241	30.21317654	2.605629985	8.59E-08	6.66E-07	AC005332.4
novel.503	225.6732313	552.1962741	-1.290705186	8.60E-08	6.66E-07	-
ENSG00000138795	234.4995598	23.8854641	3.296926475	8.64E-08	6.69E-07	LEF1
ENSG00000183049	833.4617451	112.4546657	2.887773142	8.64E-08	6.69E-07	CAMK1D
ENSG00000140285	1401.307194	3562.121882	-1.34593798	8.65E-08	6.69E-07	FGF7
ENSG00000260317	48.52198556	3.586615098	3.757527419	8.66E-08	6.69E-07	AC009812.4
ENSG00000186654	238.7701296	36.43763664	2.706693131	8.68E-08	6.71E-07	PRR5
ENSG00000163694	243.1370448	29.38567293	3.053262231	8.79E-08	6.79E-07	RBM47
ENSG00000113580	2533.211808	891.5751174	1.506540379	8.79E-08	6.79E-07	NR3C1
ENSG00000177707	368.1986528	1323.565143	-1.84550756	8.81E-08	6.81E-07	NECTIN3
ENSG00000216285	38.70044001	178.1950363	-2.203986873	8.87E-08	6.85E-07	AC078819.1
ENSG00000172432	872.8757867	2091.090357	-1.260228048	8.89E-08	6.86E-07	GTPBP2

ENSG00000272391	673.6705457	1310.615784	-0.960185939	8.89E-08	6.86E-07	POM121C
ENSG00000145040	8.437540845	145.9486565	-4.109871617	8.90E-08	6.87E-07	UCN2
ENSG00000088247	2463.542156	4644.508123	-0.91480642	8.95E-08	6.90E-07	KHSRP
ENSG00000143374	422.876102	841.2365821	-0.992472091	8.96E-08	6.91E-07	TARS2
ENSG00000273156	70.82118247	7.592288247	3.227117598	8.97E-08	6.91E-07	AC124016.2
ENSG00000164112	484.8120631	15.69837622	4.940917469	8.98E-08	6.92E-07	TMEM155
ENSG00000082781	1570.420733	9943.732565	-2.662690483	9.00E-08	6.93E-07	ITGB5
ENSG00000215915	62.62519779	0.336469917	7.341202976	9.02E-08	6.94E-07	ATAD3C
ENSG00000213139	130.9918839	39.01111757	1.746688582	9.05E-08	6.97E-07	CRYGS
ENSG00000173085	162.9892977	506.8113634	-1.637029699	9.07E-08	6.98E-07	COQ2
ENSG00000278989	82.57868173	4.809640044	4.120912163	9.21E-08	7.08E-07	AP001148.1
ENSG00000136937	681.7735465	1395.109344	-1.033224344	9.23E-08	7.10E-07	NCBP1
ENSG00000095015	687.2396569	103.7761117	2.725156909	9.26E-08	7.12E-07	MAP3K1
ENSG00000139970	191.1244613	11.09991123	4.09542845	9.27E-08	7.13E-07	RTN1
ENSG00000113211	62.68759584	0	8.30436877	9.28E-08	7.13E-07	PCDHB6
ENSG00000242147	0	48.57322564	-8.181433386	9.37E-08	7.20E-07	LINC02657
ENSG00000207736	57.37666572	0	8.176723432	9.42E-08	7.23E-07	MIR657
ENSG00000066735	61.64833068	2.058957692	4.864152102	9.42E-08	7.23E-07	KIF26A
ENSG00000129657	1872.310605	4556.062892	-1.283016861	9.42E-08	7.23E-07	SEC14L1
ENSG00000246228	0.907445357	37.07617048	-5.352662723	9.44E-08	7.25E-07	CASC8
ENSG00000175832	129.0091023	1220.865644	-3.242739807	9.48E-08	7.27E-07	ETV4
ENSG00000116990	124.2762061	10.35242721	3.597067327	9.50E-08	7.28E-07	MYCL
ENSG00000178789	33.23313091	0	7.387604181	9.59E-08	7.36E-07	CD300LB
ENSG00000151014	48.89971977	202.8539953	-2.051158403	9.60E-08	7.36E-07	NOCT
ENSG00000140403	498.0944579	182.2961403	1.448563742	9.60E-08	7.36E-07	DNAJA4
ENSG00000230445	43.48653139	1.762625967	4.605559062	9.64E-08	7.39E-07	LRRC37A6P
ENSG00000170667	173.5160934	36.6439314	2.24655927	9.65E-08	7.39E-07	RASA4B
ENSG00000143398	823.7776757	1735.289506	-1.074717598	9.66E-08	7.39E-07	PIP5K1A
ENSG00000236453	0	341.6934692	-10.99760213	9.68E-08	7.41E-07	AC003092.1
ENSG00000100122	29.14642159	0	7.198611792	9.69E-08	7.41E-07	CRYBB1
ENSG00000284753	11.92139977	93.34935027	-2.972448209	9.71E-08	7.43E-07	EEF1AKMT4
ENSG00000172757	8683.975572	17303.44461	-0.994634361	9.71E-08	7.43E-07	CFL1
ENSG00000258943	29.54518309	0	7.219141187	9.87E-08	7.55E-07	AL049871.1
ENSG00000117411	962.6443797	2047.608689	-1.088983829	9.88E-08	7.55E-07	B4GALT2
ENSG00000196917	136.8486369	14.20841687	3.254901383	9.91E-08	7.58E-07	HCAR1
ENSG00000171497	366.1953856	727.2060768	-0.989555801	1.00E-07	7.64E-07	PPID
ENSG00000167286	65.74535393	0	8.372191303	1.00E-07	7.66E-07	CD3D
ENSG00000270071	29.30613423	0	7.207149444	1.00E-07	7.67E-07	AP001172.1
ENSG00000174780	1153.978744	2589.068045	-1.165970112	1.01E-07	7.71E-07	SRP72
ENSG00000159259	65.5602561	418.2887345	-2.672676333	1.01E-07	7.73E-07	CHAF1B
ENSG00000154080	52.63713006	0	8.051566563	1.01E-07	7.73E-07	CHST9
ENSG00000231609	211.177514	20.55388666	3.362418227	1.02E-07	7.77E-07	AC007098.1
ENSG00000185298	357.4146711	1030.129146	-1.527507855	1.02E-07	7.77E-07	CCDC137
ENSG00000259863	29.5035276	156.3724639	-2.404212629	1.02E-07	7.78E-07	SH3RF3-AS1
ENSG00000091483	887.918454	2306.159678	-1.377115909	1.03E-07	7.82E-07	FH
ENSG00000233101	6.100389606	54.52856796	-3.164147413	1.03E-07	7.83E-07	HOXB-AS3
ENSG00000049323	884.8762585	6234.092876	-2.816720524	1.03E-07	7.83E-07	LTBP1
ENSG00000279833	53.8708815	4.778958462	3.514187807	1.03E-07	7.83E-07	AL031846.2
ENSG00000205837	35.76377239	0	7.494773976	1.03E-07	7.84E-07	LINC00487
ENSG00000170421	33.95550557	538.3983827	-3.988080575	1.03E-07	7.85E-07	KRT8
ENSG00000163251	78.851471	18.54811979	2.084417268	1.03E-07	7.86E-07	FZD5

ENSG00000103356	275.2355063	641.1037774	-1.220383253	1.04E-07	7.89E-07	EARS2
ENSG00000134817	60.49954262	0	8.252183444	1.05E-07	8.01E-07	APLNR
ENSG00000129235	304.2248651	825.4179325	-1.440480323	1.06E-07	8.03E-07	TXNDC17
ENSG00000130054	48.7113154	0.387220594	6.977546466	1.06E-07	8.04E-07	FAM155B
ENSG00000078401	80.23786864	379.1989844	-2.240770386	1.07E-07	8.12E-07	EDN1
ENSG00000173991	88.59654363	9.070479245	3.273236641	1.07E-07	8.15E-07	TCAP
ENSG00000114670	215.1750557	38.53557665	2.475427606	1.08E-07	8.16E-07	NEK11
ENSG00000196591	1333.146917	3064.245189	-1.200769444	1.08E-07	8.19E-07	HDAC2
ENSG00000069956	579.2363822	1393.293408	-1.265992085	1.09E-07	8.28E-07	MAPK6
novel.522	60.76073024	2.587817834	4.577170222	1.10E-07	8.33E-07	-
ENSG00000162599	1165.794293	313.9163158	1.892051256	1.11E-07	8.39E-07	NFIA
novel.994	46.16037426	0	7.863107898	1.12E-07	8.48E-07	-
ENSG00000176208	69.91283947	241.4711326	-1.787025746	1.14E-07	8.63E-07	ATAD5
ENSG00000101443	90.53372315	0.743759607	6.966166252	1.14E-07	8.65E-07	WFDC2
ENSG00000186810	50.61710992	0	7.994777863	1.15E-07	8.71E-07	CXCR3
ENSG00000070785	298.2903851	833.9078206	-1.483652333	1.16E-07	8.79E-07	EIF2B3
ENSG00000130304	2320.093769	498.9349192	2.217755943	1.16E-07	8.80E-07	SLC27A1
ENSG00000167658	26053.59977	65144.84763	-1.322167911	1.17E-07	8.82E-07	EEF2
ENSG00000140564	1565.70983	3698.871574	-1.24030226	1.17E-07	8.82E-07	FURIN
ENSG00000090615	1926.592403	3670.98781	-0.93005554	1.18E-07	8.89E-07	GOLGA3
ENSG00000243323	29.14266064	0	7.198462601	1.18E-07	8.95E-07	PTPRVP
ENSG00000130638	1209.591946	2544.808757	-1.073068086	1.19E-07	8.98E-07	ATXN10
ENSG00000174428	711.6133975	218.4320041	1.704160766	1.19E-07	9.00E-07	GTF2IRD2B
ENSG00000159173	46.99664602	0.336469917	6.926994442	1.20E-07	9.07E-07	TNNI1
ENSG00000261801	19.70336535	503.6824722	-4.677821868	1.20E-07	9.08E-07	LOXL1-AS1
ENSG00000205336	640.5069647	84.36603548	2.926320347	1.21E-07	9.11E-07	ADGRG1
ENSG00000112208	129.6428666	993.0171217	-2.937859406	1.22E-07	9.18E-07	BAG2
ENSG00000119401	229.457853	562.9810556	-1.294608298	1.23E-07	9.25E-07	TRIM32
ENSG00000198821	75.73993189	0.336469917	7.614496261	1.23E-07	9.28E-07	CD247
ENSG00000132164	91.39204608	0	8.847506286	1.23E-07	9.28E-07	SLC6A11
ENSG00000132938	0.909952655	45.26369071	-5.640871131	1.24E-07	9.31E-07	MTUS2
ENSG00000239521	320.0737048	78.98200291	2.019736388	1.24E-07	9.35E-07	CASTOR3
ENSG00000189423	128.6089171	19.83849688	2.698087532	1.24E-07	9.36E-07	USP32P3
ENSG00000141499	176.2938755	415.5008549	-1.237492615	1.25E-07	9.39E-07	WRAP53
ENSG00000050730	3.076037145	190.2929616	-5.956839283	1.25E-07	9.44E-07	TNIP3
ENSG00000066697	240.1103713	906.6617768	-1.916483501	1.26E-07	9.47E-07	MSANTD3
ENSG00000197815	358.3314937	34.34897767	3.38411098	1.27E-07	9.53E-07	AC122129.1
ENSG00000205363	176.5475252	12.69954429	3.791843154	1.27E-07	9.56E-07	INSYN1
ENSG00000154864	301.2964191	16.14580411	4.21327605	1.28E-07	9.62E-07	PIEZO2
ENSG00000181045	601.564726	275.9590188	1.124949588	1.28E-07	9.62E-07	SLC26A11
ENSG00000159593	649.9868187	1438.118336	-1.145573164	1.29E-07	9.67E-07	NAE1
ENSG00000147246	50.04706855	0	7.978644966	1.29E-07	9.68E-07	HTR2C
ENSG00000182858	133.7083077	320.5289299	-1.261592362	1.30E-07	9.74E-07	ALG12
ENSG00000078618	1868.701541	3867.489191	-1.049288368	1.30E-07	9.74E-07	NRDC
ENSG00000174307	4498.462035	1756.788361	1.356306641	1.30E-07	9.76E-07	PHLDA3
ENSG00000287677	84.8238021	0	8.739900498	1.30E-07	9.77E-07	AC025839.1
ENSG00000232611	50.53469692	7.938214774	2.674722575	1.30E-07	9.79E-07	AL683813.1
ENSG00000167674	1419.325963	2966.066156	-1.063343574	1.31E-07	9.82E-07	HDGFL2
ENSG00000109881	104.507366	403.6409206	-1.950474631	1.31E-07	9.83E-07	CCDC34
ENSG00000168490	189.6671473	47.42859769	1.999587744	1.32E-07	9.87E-07	PHYHIP
ENSG00000158435	521.2354112	1070.223245	-1.038028867	1.32E-07	9.88E-07	CNOT11

ENSG00000153253	34.78827364	0	7.455054933	1.32E-07	9.88E-07	SCN3A
ENSG00000126561	764.5617149	147.4979365	2.372168158	1.32E-07	9.89E-07	STAT5A
ENSG00000162892	1.233423615	10324.07308	-13.03501347	1.32E-07	9.92E-07	IL24
ENSG00000164440	87.21017346	6.685664122	3.694152242	1.35E-07	1.01E-06	TXLNB
ENSG00000105137	1211.199915	2839.597155	-1.229130388	1.35E-07	1.01E-06	SYDE1
ENSG00000065183	331.0267557	1029.708148	-1.637663411	1.35E-07	1.01E-06	WDR3
ENSG00000095587	128.0969152	3.168712922	5.326272895	1.36E-07	1.01E-06	TLL2
ENSG00000065882	677.8698547	1902.130257	-1.488534013	1.36E-07	1.02E-06	TBC1D1
ENSG00000263618	73.35487288	0	8.53101977	1.36E-07	1.02E-06	AP005121.1
ENSG00000183508	76.00928214	3.210006989	4.560487809	1.37E-07	1.02E-06	TENT5C
novel.590	9.182766265	104.9388354	-3.517704322	1.38E-07	1.03E-06	-
ENSG00000250072	45.0144495	5.319715359	3.070907962	1.39E-07	1.03E-06	SH3TC2-DT
ENSG00000121552	131.0821035	9.632461113	3.749766826	1.39E-07	1.04E-06	CSTA
ENSG00000090621	2281.355954	5116.52927	-1.165332545	1.40E-07	1.04E-06	PABPC4
ENSG00000106809	4498.013734	3.761376372	10.2165979	1.41E-07	1.05E-06	OGN
novel.998	36.67734345	0	7.53106767	1.41E-07	1.05E-06	-
ENSG00000159899	518.9106546	1013.441998	-0.965508765	1.41E-07	1.05E-06	NPR2
ENSG00000234362	38.97577377	1.742556871	4.451308309	1.41E-07	1.05E-06	LINC01914
ENSG00000163395	2.459325337	53.72241771	-4.461129295	1.42E-07	1.05E-06	IGFN1
ENSG00000139155	108.836486	1.426156051	6.247348976	1.42E-07	1.06E-06	SLCO1C1
ENSG00000173692	1241.444319	3433.502494	-1.467782779	1.43E-07	1.06E-06	PSMD1
ENSG00000092199	6169.619746	13306.61319	-1.108916719	1.43E-07	1.06E-06	HNRNPC
ENSG00000133687	174.8559197	2416.251791	-3.788834298	1.43E-07	1.07E-06	TMTC1
ENSG00000178952	2306.086181	5255.883038	-1.188533215	1.43E-07	1.07E-06	TUFM
ENSG00000072818	371.7666332	106.3334107	1.807499681	1.44E-07	1.07E-06	ACAP1
ENSG00000048991	384.2393137	777.3031539	-1.016266714	1.44E-07	1.07E-06	R3HDM1
ENSG00000173465	234.8414242	604.6289223	-1.364536914	1.46E-07	1.08E-06	ZNRD2
ENSG00000076604	441.0848561	1212.930049	-1.459710146	1.48E-07	1.10E-06	TRAF4
ENSG00000242114	3.958266985	38.78560562	-3.297381409	1.48E-07	1.10E-06	MTFP1
ENSG00000165322	1778.297586	529.1210438	1.749345794	1.49E-07	1.11E-06	ARHGAP12
ENSG00000109738	423.3633903	63.35736615	2.73740807	1.51E-07	1.12E-06	GLRB
ENSG00000164935	0.616711807	52.64120789	-6.439078991	1.51E-07	1.12E-06	DCSTAMP
ENSG00000259030	92.20415848	8.396255142	3.465998891	1.51E-07	1.12E-06	FPGT-TNNI3K
ENSG00000188803	77.1035504	0	8.602204081	1.51E-07	1.12E-06	SHISA6
ENSG00000185825	2216.984757	4837.175652	-1.125597712	1.52E-07	1.13E-06	BCAP31
ENSG00000115525	649.6891317	208.6576935	1.637221361	1.52E-07	1.13E-06	ST3GAL5
ENSG00000169783	47.37201512	5.656185276	3.052942031	1.54E-07	1.14E-06	LINGO1
ENSG00000224126	5.484931447	67.66651588	-3.630585579	1.54E-07	1.14E-06	UBE2SP2
ENSG00000109943	49.70590361	0	7.968571871	1.55E-07	1.15E-06	CRTAM
ENSG00000188993	107.5262487	10.52731688	3.342802154	1.55E-07	1.15E-06	LRRC66
ENSG00000255690	443.0639016	8.888701506	5.629599711	1.56E-07	1.15E-06	TRIL
ENSG00000198722	1382.049618	618.6645385	1.15939363	1.57E-07	1.17E-06	UNC13B
ENSG00000166669	461.046649	70.16930544	2.714160963	1.57E-07	1.17E-06	ATF7IP2
ENSG00000136541	48.23153674	0.774441189	6.056489828	1.58E-07	1.17E-06	ERMN
ENSG00000007923	356.3283248	920.912925	-1.369817282	1.59E-07	1.17E-06	DNAJC11
ENSG00000124766	859.749938	356.1575438	1.271327333	1.59E-07	1.18E-06	SOX4
ENSG00000139874	5.196776466	345.1964378	-6.056175827	1.59E-07	1.18E-06	SSTR1
ENSG00000158856	112.5149459	6.05286248	4.211951231	1.59E-07	1.18E-06	DMTN
ENSG00000144406	38.25006509	0	7.590448363	1.60E-07	1.18E-06	UNC80
ENSG00000187017	121.5474043	4.748276881	4.68659888	1.60E-07	1.18E-06	ESPN
ENSG00000154654	1868.789459	70.91948639	4.718182026	1.61E-07	1.19E-06	NCAM2

ENSG00000167258	515.6235052	1077.342548	-1.063121065	1.61E-07	1.19E-06	CDK12
ENSG00000162244	9427.807773	18342.40738	-0.960194364	1.64E-07	1.21E-06	RPL29
ENSG00000143106	930.3890685	2307.206293	-1.310413582	1.67E-07	1.23E-06	PSMA5
ENSG00000198959	1347.80591	30635.5379	-4.506545454	1.67E-07	1.23E-06	TGM2
ENSG00000259877	132.6029704	37.28618965	1.834261284	1.67E-07	1.23E-06	AC009113.1
ENSG00000106636	1942.647665	4016.574179	-1.047978215	1.68E-07	1.24E-06	YKT6
ENSG00000080298	431.9430927	100.753953	2.101477227	1.70E-07	1.25E-06	RFX3
novel.781	63.43765022	204.4915963	-1.688074412	1.71E-07	1.26E-06	-
ENSG00000067715	8.539585292	75.82780246	-3.155777667	1.71E-07	1.26E-06	SYT1
ENSG00000128591	624.9002598	9285.164306	-3.893301202	1.72E-07	1.27E-06	FLNC
ENSG00000125637	424.4354879	97.56213573	2.119934647	1.73E-07	1.27E-06	PSD4
ENSG00000257176	98.92516303	10.65949981	3.209819369	1.73E-07	1.27E-06	AC009318.1
ENSG00000172116	64.50601843	0	8.344758645	1.73E-07	1.28E-06	CD8B
ENSG00000204556	41.83665264	2.435565801	4.07154383	1.74E-07	1.28E-06	AL450124.1
ENSG00000160013	50.33228861	399.0557056	-2.987428733	1.74E-07	1.28E-06	PTGIR
ENSG00000163006	28.99018516	109.8543903	-1.922456595	1.76E-07	1.29E-06	CCDC138
ENSG00000170522	410.443056	1087.823353	-1.40580886	1.77E-07	1.30E-06	ELOVL6
ENSG00000179057	62.90786746	4.688069593	3.749415956	1.78E-07	1.31E-06	IGSF22
ENSG00000138135	463.2141123	17.46100219	4.72254513	1.79E-07	1.32E-06	CH25H
ENSG00000114251	283.8432138	9455.912553	-5.058109138	1.80E-07	1.32E-06	WNT5A
ENSG00000169762	750.0771233	239.7872548	1.644105517	1.81E-07	1.33E-06	TAPT1
ENSG00000147364	642.5348771	277.3759751	1.212183717	1.81E-07	1.33E-06	FBXO25
ENSG00000083845	4768.857758	12924.62667	-1.438422602	1.82E-07	1.34E-06	RPS5
ENSG00000245573	118.8597275	36.80936446	1.688707919	1.82E-07	1.34E-06	BDNF-AS
ENSG00000161551	402.8859928	125.8581225	1.678635501	1.83E-07	1.35E-06	ZNF577
ENSG00000130741	1968.015006	4738.257285	-1.267599026	1.84E-07	1.35E-06	EIF2S3
ENSG00000260400	131.4846263	22.41343766	2.543572237	1.84E-07	1.35E-06	AL513534.2
ENSG00000264575	78.13167456	18.17151168	2.099828819	1.84E-07	1.35E-06	LINC00526
ENSG00000164983	999.5477659	381.4126342	1.390543981	1.84E-07	1.35E-06	TMEM65
ENSG00000188785	257.341305	108.5744501	1.244633523	1.85E-07	1.36E-06	ZNF548
ENSG00000167178	329.9413754	5.492036488	5.914059683	1.86E-07	1.36E-06	ISLR2
ENSG00000116729	1418.096569	3702.387991	-1.384477642	1.86E-07	1.36E-06	WLS
ENSG00000061794	534.7904636	1176.193289	-1.137331425	1.87E-07	1.37E-06	MRPS35
ENSG00000162878	1547.4192	206.8523265	2.902148797	1.88E-07	1.37E-06	PKDCC
ENSG00000091137	284.9462434	10.89562425	4.704043628	1.88E-07	1.38E-06	SLC26A4
ENSG00000181072	0	31.94396505	-7.576621002	1.89E-07	1.38E-06	CHRM2
ENSG00000102543	338.9326635	136.3982352	1.313433375	1.89E-07	1.38E-06	CDADC1
ENSG00000223812	83.31515874	1.733100261	5.559702052	1.90E-07	1.38E-06	AC073365.1
ENSG00000058804	280.6139485	934.9673045	-1.736791224	1.91E-07	1.39E-06	NDC1
ENSG00000236963	57.66177176	0.387220594	7.221953667	1.91E-07	1.39E-06	LINC01141
ENSG00000234807	43.79237999	3.698728939	3.592623918	1.92E-07	1.40E-06	LINC01135
ENSG00000140396	888.8476818	330.7334715	1.425389971	1.93E-07	1.41E-06	NCOA2
ENSG00000167207	101.5423623	3.067083173	5.088448849	1.94E-07	1.42E-06	NOD2
ENSG00000135063	59.9528933	3.444975551	4.089704605	1.95E-07	1.42E-06	FAM189A2
ENSG00000122545	6148.256868	3310.081162	0.893257516	1.95E-07	1.42E-06	SEPT7
ENSG00000168488	1419.84022	3040.947238	-1.098697666	1.96E-07	1.43E-06	ATXN2L
ENSG00000197324	3099.61945	5702.079515	-0.879380082	1.97E-07	1.43E-06	LRP10
ENSG00000277494	84.26217992	1.069617038	6.292189537	1.97E-07	1.43E-06	GPIHBP1
ENSG00000144229	118.6241107	0.672939833	7.396905304	1.98E-07	1.44E-06	THSD7B
ENSG00000198842	36.2611734	0	7.5142306	1.98E-07	1.44E-06	DUSP27
ENSG00000168754	45.1628793	0.387220594	6.869606521	2.00E-07	1.46E-06	FAM178B

ENSG00000180875	26.69615653	410.673385	-3.94452591	2.01E-07	1.46E-06	GREM2
ENSG00000128536	270.3011592	31.27718978	3.109883615	2.01E-07	1.46E-06	CDHR3
ENSG00000172059	301.4768149	104.3098865	1.533098299	2.02E-07	1.47E-06	KLF11
ENSG00000231453	94.03239609	0.387220594	7.926731377	2.03E-07	1.47E-06	LINC01305
ENSG00000181458	180.2534239	724.7828873	-2.008048804	2.03E-07	1.48E-06	TMEM45A
ENSG00000175756	1378.840307	3041.878431	-1.141576915	2.04E-07	1.48E-06	AURKAIP1
ENSG00000166965	219.0388284	720.6960731	-1.718282243	2.05E-07	1.49E-06	RCCD1
ENSG00000025800	1019.291219	1890.105347	-0.890921664	2.07E-07	1.50E-06	KPNA6
ENSG00000178035	1568.62178	4718.280468	-1.588821349	2.08E-07	1.51E-06	IMPDH2
ENSG00000061455	67.92416124	8.652448675	2.976467319	2.09E-07	1.51E-06	PRDM6
ENSG00000225683	37.00661228	0	7.542795424	2.10E-07	1.52E-06	PACRG-AS3
ENSG00000106006	0	38.40814561	-7.846776071	2.10E-07	1.52E-06	HOXA6
ENSG00000166148	29.71012379	0	7.226480935	2.11E-07	1.53E-06	AVPR1A
ENSG00000188820	152.28498	13.25664586	3.529803198	2.11E-07	1.53E-06	CALHM6
ENSG00000119147	73.17146749	0	8.526661761	2.11E-07	1.53E-06	C2orf40
ENSG00000167986	3165.490769	6738.96934	-1.090059314	2.12E-07	1.54E-06	DDB1
novel.773	49.5333701	0	7.9639341	2.15E-07	1.56E-06	-
ENSG00000105612	900.4053788	455.042408	0.984813167	2.15E-07	1.56E-06	DNASE2
ENSG00000055044	476.0448525	1345.332696	-1.49892317	2.17E-07	1.57E-06	NOP58
ENSG00000188321	473.3128161	162.2483005	1.544823408	2.18E-07	1.58E-06	ZNF559
ENSG00000013306	1326.907188	2835.276433	-1.095357178	2.19E-07	1.58E-06	SLC25A39
ENSG00000164244	911.6786821	2217.561888	-1.282344008	2.19E-07	1.58E-06	PRRC1
ENSG00000125977	1338.864087	3526.107212	-1.397177132	2.19E-07	1.59E-06	EIF2S2
ENSG00000163220	57.39951613	0.356539013	7.215618284	2.21E-07	1.60E-06	S100A9
ENSG00000147065	8242.145945	15177.42233	-0.880824356	2.21E-07	1.60E-06	MSN
ENSG00000251448	29.45191419	0	7.214816876	2.22E-07	1.60E-06	AC063919.1
ENSG00000081760	342.2864705	833.9862735	-1.284346498	2.24E-07	1.62E-06	AACS
ENSG00000121690	881.4548409	94.0578048	3.228802002	2.24E-07	1.62E-06	DEPDC7
ENSG00000100714	649.3217552	2028.153246	-1.643275902	2.26E-07	1.63E-06	MTHFD1
ENSG00000214106	233.2915099	99.89119149	1.224022887	2.27E-07	1.64E-06	PAXIP1-AS2
ENSG00000149295	64.59320507	0.387220594	7.38538762	2.28E-07	1.65E-06	DRD2
ENSG00000186314	24.30592476	98.60551911	-2.021457436	2.30E-07	1.66E-06	PRELID2
ENSG00000176714	126.5790103	9.623004503	3.700606419	2.30E-07	1.66E-06	CCDC121
novel.279	32.09240674	0	7.338719552	2.30E-07	1.66E-06	-
ENSG00000280734	76.90627138	14.37945372	2.419535951	2.32E-07	1.67E-06	LINC01232
ENSG00000166897	48.16175934	692.6639945	-3.846917871	2.33E-07	1.68E-06	ELFN2
ENSG00000069702	781.0395286	125.3725299	2.637500419	2.33E-07	1.68E-06	TGFBR3
ENSG00000114841	614.2081593	199.2863406	1.624620032	2.33E-07	1.68E-06	DNAH1
ENSG00000173660	1303.617276	3296.917645	-1.338628849	2.33E-07	1.68E-06	UQCRH
ENSG00000224165	66.41701352	9.591038651	2.785376619	2.38E-07	1.71E-06	DNAJC27-AS1
ENSG00000125741	389.893801	786.0314311	-1.011737795	2.39E-07	1.72E-06	OPA3
ENSG00000250903	160.7731378	53.17811198	1.593724536	2.40E-07	1.73E-06	GMDS-DT
ENSG00000173950	239.5575518	908.4894466	-1.923583111	2.40E-07	1.73E-06	XXYLT1
ENSG00000003096	397.7783262	61.74468047	2.684815444	2.41E-07	1.73E-06	KLHL13
ENSG00000092964	4047.380047	2116.217844	0.935409698	2.41E-07	1.73E-06	DPYSL2
ENSG00000047457	626.5978175	1.130980201	9.138683586	2.41E-07	1.74E-06	CP
ENSG00000170889	0.308355904	46.93589453	-7.169552641	2.42E-07	1.74E-06	RPS9
ENSG00000169764	1187.573964	3549.333332	-1.579613413	2.43E-07	1.74E-06	UGP2
ENSG00000225101	30.61101617	0	7.270559012	2.43E-07	1.74E-06	OR52K3P
ENSG00000138768	1543.817202	3264.986821	-1.080460471	2.44E-07	1.75E-06	USO1
ENSG00000231889	156.3238097	42.68263256	1.872862849	2.45E-07	1.76E-06	TRAF3IP2-AS1

ENSG00000061656	44.2344063	435.7651442	-3.300689272	2.47E-07	1.77E-06	SPAG4
ENSG00000166557	860.7681718	2969.606815	-1.786651243	2.47E-07	1.77E-06	TMED3
ENSG00000010438	0	41.04915426	-7.942561068	2.47E-07	1.77E-06	PRSS3
ENSG00000149328	164.8654312	33.25330255	2.311509088	2.48E-07	1.77E-06	GLB1L2
ENSG00000147434	34.68615792	0	7.45093034	2.48E-07	1.78E-06	CHRNA6
ENSG00000108094	565.1933021	1078.828154	-0.93275305	2.49E-07	1.79E-06	CUL2
ENSG00000211893	94.52449742	0	8.896147821	2.49E-07	1.79E-06	IGHG2
ENSG00000107959	924.5603824	2012.31812	-1.122086593	2.51E-07	1.80E-06	PITRM1
ENSG00000171714	42.66497559	0	7.748749746	2.51E-07	1.80E-06	ANO5
ENSG00000130175	3297.498265	7554.712458	-1.195984806	2.52E-07	1.80E-06	PRKCSH
novel.184	376.897666	22.6095523	4.052253694	2.52E-07	1.80E-06	-
ENSG00000131831	69.5977446	5.961973611	3.530120361	2.55E-07	1.82E-06	RAI2
ENSG00000163527	1721.60916	5304.052941	-1.623417831	2.56E-07	1.83E-06	STT3B
ENSG00000188573	56.90673027	3.944309986	3.850383044	2.57E-07	1.84E-06	FBLL1
ENSG00000163781	475.6245937	939.9747033	-0.982878163	2.64E-07	1.89E-06	TOPBP1
ENSG00000144579	3407.043867	1843.82348	0.885890757	2.64E-07	1.89E-06	CTDSP1
ENSG00000114268	186.8557034	1515.2699	-3.019362631	2.65E-07	1.89E-06	PFKFB4
ENSG00000185842	21.05749595	123.9666528	-2.559956024	2.66E-07	1.90E-06	DNAH14
ENSG00000125107	1101.52818	2774.948926	-1.332937119	2.70E-07	1.93E-06	CNOT1
ENSG00000147955	1079.841976	3082.730095	-1.513530248	2.70E-07	1.93E-06	SIGMAR1
ENSG00000133422	553.5080166	1146.391953	-1.050143372	2.71E-07	1.93E-06	MORC2
ENSG00000113296	163.3728885	12.2178388	3.741746993	2.71E-07	1.93E-06	THBS4
ENSG00000073067	56.50328269	0	8.15450367	2.71E-07	1.94E-06	CYP2W1
ENSG00000141564	517.2273261	1311.906778	-1.342538072	2.72E-07	1.94E-06	RPTOR
ENSG00000188112	11.96925224	170.4470412	-3.835824708	2.72E-07	1.94E-06	C6orf132
ENSG00000285517	22.53484083	128.3973259	-2.507472613	2.73E-07	1.94E-06	AC010198.2
ENSG00000231680	30.83057613	0	7.279286815	2.73E-07	1.95E-06	AP003774.3
ENSG00000251247	167.9987531	42.3933173	1.98999745	2.73E-07	1.95E-06	ZNF345
ENSG00000282057	10.29519816	87.3197674	-3.086008515	2.75E-07	1.95E-06	AC092807.3
novel.769	31.16099951	0	7.29633954	2.75E-07	1.95E-06	-
ENSG00000130733	454.5495456	1150.197334	-1.339382517	2.75E-07	1.96E-06	YIPF2
ENSG00000173473	951.8424196	2255.36147	-1.24465266	2.76E-07	1.96E-06	SMARCC1
novel.31	28.94129251	148.9398293	-2.365268458	2.76E-07	1.96E-06	-
ENSG00000175643	68.15904972	274.8472902	-2.011716417	2.76E-07	1.97E-06	RMI2
ENSG00000181191	1021.856166	435.7169873	1.229358448	2.77E-07	1.97E-06	PJA1
ENSG00000273356	42.86989085	3.546476906	3.585212126	2.78E-07	1.98E-06	LINC02019
ENSG00000105197	737.0301137	1860.705004	-1.336177667	2.78E-07	1.98E-06	TIMM50
ENSG00000134905	1089.088497	2444.955054	-1.166591765	2.78E-07	1.98E-06	CARS2
ENSG00000162595	220.5198344	27.40973573	3.000902216	2.80E-07	1.98E-06	DIRAS3
ENSG00000160299	576.4177718	1394.15506	-1.274000586	2.80E-07	1.99E-06	PCNT
ENSG00000141385	504.4129835	1019.730565	-1.015709041	2.81E-07	1.99E-06	AFG3L2
ENSG00000130528	82.47938517	0.387220594	7.737597856	2.81E-07	2.00E-06	HRC
ENSG00000101082	35.49875255	0	7.482722319	2.82E-07	2.00E-06	SLA2
ENSG00000185158	241.1405324	82.35004131	1.552011453	2.82E-07	2.00E-06	LRRC37B
ENSG00000131373	185.7619334	408.0457926	-1.134846871	2.83E-07	2.00E-06	HACL1
ENSG00000234661	42.69700099	0	7.750451662	2.83E-07	2.01E-06	CHL1-AS1
ENSG00000176485	3648.945029	199.0646599	4.19542459	2.84E-07	2.01E-06	PLAAT3
ENSG00000260572	45.56199681	2.598430319	4.163012238	2.84E-07	2.01E-06	AC069224.1
ENSG00000097046	112.0985346	307.8947772	-1.45866374	2.84E-07	2.01E-06	CDC7
ENSG00000138385	1004.250075	3323.596016	-1.726756682	2.85E-07	2.02E-06	SSB
ENSG00000106397	2430.28515	5070.156829	-1.06086456	2.85E-07	2.02E-06	PLOD3

ENSG00000101198	94.68832841	0.693008929	7.057223714	2.86E-07	2.02E-06	NKAIN4
ENSG00000130669	607.8872259	1268.96067	-1.061672405	2.87E-07	2.03E-06	PAK4
ENSG00000111269	2112.002995	645.924531	1.709365669	2.92E-07	2.07E-06	CREBL2
ENSG00000183317	27.99742007	0	7.140392758	2.93E-07	2.07E-06	EPHA10
ENSG00000198157	201.6558176	76.90071155	1.389381318	2.94E-07	2.07E-06	HMGN5
ENSG00000130589	1154.281476	3070.184635	-1.411199262	2.94E-07	2.08E-06	HELZ2
ENSG00000156299	496.4612455	76.72749134	2.692216955	2.94E-07	2.08E-06	TIAM1
ENSG00000135722	40.61583679	130.4412703	-1.684891807	2.94E-07	2.08E-06	FBXL8
ENSG00000204116	447.1646613	164.154959	1.446251259	2.96E-07	2.09E-06	CHIC1
ENSG00000262801	33.61015311	0	7.405224602	2.97E-07	2.09E-06	U91319.1
ENSG00000179058	66.82677313	10.76215704	2.635075425	2.98E-07	2.10E-06	C9orf50
ENSG00000166822	590.7225941	219.1274532	1.430726378	2.98E-07	2.10E-06	TMEM170A
ENSG00000183723	302.3038839	720.6214814	-1.253499237	2.98E-07	2.10E-06	CMTM4
ENSG00000116353	305.6111636	640.6410324	-1.068184458	3.02E-07	2.13E-06	MECR
ENSG00000228956	28.87087436	0	7.184898554	3.03E-07	2.13E-06	SATB1-AS1
ENSG00000120616	794.3091808	249.2707559	1.671193044	3.04E-07	2.15E-06	EPC1
ENSG00000139433	2861.307053	1032.729161	1.470485516	3.09E-07	2.18E-06	GLTP
ENSG00000228486	119.5177388	40.65204469	1.557193927	3.09E-07	2.18E-06	C2orf92
ENSG00000270164	32.45230478	0	7.354587437	3.11E-07	2.19E-06	LINC01480
ENSG00000184156	454.8140524	35.93761305	3.666413081	3.12E-07	2.19E-06	KCNQ3
ENSG00000174807	733.1665045	7725.53489	-3.397480594	3.19E-07	2.25E-06	CD248
ENSG00000150893	314.2280805	0	10.62941059	3.21E-07	2.26E-06	FREM2
ENSG00000281103	47.53953439	1.00940975	5.485581086	3.22E-07	2.26E-06	TRG-AS1
ENSG00000186889	211.3259991	79.37744303	1.413284173	3.22E-07	2.26E-06	TMEM17
ENSG00000182985	2705.116959	116.5927189	4.537139948	3.24E-07	2.28E-06	CADM1
ENSG00000187514	10131.05133	17686.57598	-0.803878803	3.25E-07	2.28E-06	PTMA
ENSG00000224189	2.421502061	39.11261893	-4.013551944	3.25E-07	2.28E-06	HAGLR
ENSG00000125910	38.60369489	0	7.603757405	3.26E-07	2.29E-06	S1PR4
ENSG00000177627	63.14223025	0.356539013	7.353153759	3.26E-07	2.29E-06	C12orf54
ENSG00000128487	836.0143255	375.5725819	1.153777331	3.29E-07	2.31E-06	SPECC1
ENSG00000198176	1395.658661	2749.973981	-0.978586961	3.31E-07	2.32E-06	TFDP1
ENSG00000118004	651.8807078	2.10970837	8.264250755	3.31E-07	2.32E-06	COLEC11
ENSG00000226125	26.20362792	0	7.046093633	3.31E-07	2.32E-06	LINC01907
ENSG00000136161	810.7208789	161.3698835	2.327226268	3.32E-07	2.33E-06	RCBTB2
ENSG00000179151	416.6421842	837.5152498	-1.00703964	3.35E-07	2.35E-06	EDC3
ENSG00000231346	41.77089309	1.029478846	5.291553043	3.35E-07	2.35E-06	LINC01160
ENSG00000071242	633.5542727	2413.017076	-1.929482019	3.35E-07	2.35E-06	RPS6KA2
ENSG00000083312	1822.073181	4193.282574	-1.202572345	3.37E-07	2.36E-06	TNPO1
ENSG00000128512	1006.434367	261.9825225	1.94269973	3.38E-07	2.36E-06	DOCK4
ENSG00000127578	46.20725781	2.486316478	4.207234847	3.39E-07	2.37E-06	WFIKKN1
ENSG00000138722	27.37025179	0	7.109025022	3.39E-07	2.37E-06	MMRN1
ENSG00000103495	325.1367667	731.761368	-1.17076351	3.40E-07	2.38E-06	MAZ
ENSG00000172236	29.37529872	0	7.210245384	3.40E-07	2.38E-06	TPSAB1
ENSG00000125656	720.1547035	1447.830187	-1.007665082	3.40E-07	2.38E-06	CLPP
ENSG00000080822	2515.819177	1221.809782	1.041946987	3.43E-07	2.40E-06	CLDND1
ENSG00000088992	36.79235166	1.753169357	4.365993575	3.45E-07	2.41E-06	TESC
ENSG00000254554	0.894837599	49.40710342	-5.770403495	3.47E-07	2.42E-06	AC080023.1
ENSG00000160505	27.35388309	0	7.108224858	3.51E-07	2.45E-06	NLRP4
ENSG00000227486	45.86275955	5.401147618	3.089239262	3.51E-07	2.45E-06	AL050309.1
ENSG00000165609	641.7712022	1259.105219	-0.972173365	3.53E-07	2.46E-06	NUDT5
ENSG00000177000	1170.988626	400.4281695	1.548135067	3.53E-07	2.47E-06	MTHFR

ENSG00000008226	41.12291098	2.893606169	3.835941261	3.54E-07	2.47E-06	DLEC1
ENSG00000246731	75.01230135	9.762203905	2.94664213	3.58E-07	2.49E-06	AC100786.1
ENSG00000259865	406.074033	126.967621	1.678298732	3.58E-07	2.50E-06	AL390728.6
ENSG00000129595	60.85575134	0.713078025	6.406351287	3.59E-07	2.50E-06	EPB41L4A
ENSG00000213445	4339.762146	1466.341761	1.565619495	3.59E-07	2.50E-06	SIPA1
ENSG00000135318	601.4015595	11228.06608	-4.222699647	3.63E-07	2.53E-06	NT5E
ENSG00000154945	1761.823676	771.2389554	1.191739583	3.63E-07	2.53E-06	ANKRD40
ENSG00000205045	28.30061883	0	7.155975029	3.63E-07	2.53E-06	SLFN12L
ENSG00000106682	5696.893777	10032.2042	-0.816378251	3.64E-07	2.53E-06	EIF4H
ENSG00000109670	928.1570433	335.587488	1.467289733	3.64E-07	2.53E-06	FBXW7
ENSG00000139629	84.37812886	588.0397149	-2.800787035	3.65E-07	2.54E-06	GALNT6
ENSG00000160606	31.86994004	153.8820506	-2.273495405	3.66E-07	2.54E-06	TLCD1
ENSG00000011304	3264.398465	7081.497784	-1.117283028	3.66E-07	2.54E-06	PTBP1
ENSG00000178741	865.4302822	1884.653193	-1.122753025	3.69E-07	2.57E-06	COX5A
ENSG00000108604	880.0686322	2197.007727	-1.320039054	3.74E-07	2.60E-06	SMARCD2
ENSG00000179331	28.01002782	0	7.141029438	3.74E-07	2.60E-06	RAB39A
ENSG00000107262	1435.243225	559.7860063	1.35785781	3.76E-07	2.61E-06	BAG1
ENSG00000242071	959.7330698	1937.870475	-1.013840451	3.76E-07	2.61E-06	RPL7AP6
ENSG00000258708	64.62537266	9.233343763	2.801423703	3.77E-07	2.62E-06	SLC25A21-AS1
ENSG00000160801	87.33360121	4.861674991	4.14854604	3.78E-07	2.62E-06	PTH1R
ENSG00000099822	21.47165725	318.8995741	-3.894733544	3.79E-07	2.63E-06	HCN2
ENSG00000250657	0	77.75204304	-8.861220173	3.80E-07	2.63E-06	AC097451.1
ENSG00000113396	137.7700004	2.476859868	5.789957366	3.81E-07	2.64E-06	SLC27A6
ENSG00000103064	143.5236559	370.4005422	-1.368036015	3.82E-07	2.65E-06	SLC7A6
ENSG00000109079	779.8473506	1439.169519	-0.884058113	3.82E-07	2.65E-06	TNFAIP1
ENSG00000136717	3955.200038	1318.084482	1.585413452	3.83E-07	2.65E-06	BIN1
ENSG00000197747	8421.409347	3346.338122	1.331479187	3.86E-07	2.67E-06	S100A10
ENSG00000185942	26.1972884	0	7.045747286	3.86E-07	2.68E-06	NKAIN3
ENSG00000273271	63.93469523	11.50591665	2.476121748	3.86E-07	2.68E-06	AP000254.1
ENSG00000169692	439.0706064	1048.99329	-1.256333384	3.88E-07	2.69E-06	AGPAT2
ENSG00000089847	142.3846127	34.40807626	2.045271026	3.89E-07	2.69E-06	ANKRD24
ENSG00000159055	138.7651452	347.2739454	-1.323785486	3.91E-07	2.71E-06	MIS18A
ENSG00000184216	1564.290977	3341.055624	-1.094910213	3.91E-07	2.71E-06	IRAK1
ENSG00000102886	115.0473118	38.23783226	1.587033176	3.92E-07	2.71E-06	GDPD3
ENSG00000182004	510.0960485	1249.619033	-1.292835984	3.92E-07	2.71E-06	SNRPE
ENSG00000233850	26.58372688	0	7.066470466	3.92E-07	2.71E-06	AC103563.7
ENSG00000133110	227.7779128	3919.135582	-4.105021788	3.93E-07	2.71E-06	POSTN
ENSG00000268460	37.26737263	0	7.554460003	3.93E-07	2.71E-06	AC006262.1
ENSG00000099804	699.8674784	1466.274015	-1.066809484	3.94E-07	2.72E-06	CDC34
ENSG00000267680	347.4751552	127.2299319	1.449972102	4.01E-07	2.77E-06	ZNF224
ENSG00000125743	1499.597265	3492.774867	-1.219803198	4.01E-07	2.77E-06	SNRPD2
ENSG00000131097	37.65445185	1.4981317	4.698232068	4.01E-07	2.77E-06	HIGD1B
ENSG00000177239	1649.715707	3236.788779	-0.972322837	4.02E-07	2.77E-06	MAN1B1
ENSG00000139343	514.4940204	1195.061068	-1.216096085	4.06E-07	2.80E-06	SNRPF
ENSG00000103528	107.8464138	13.58250329	2.99772589	4.08E-07	2.81E-06	SYT17
ENSG00000169299	299.0121472	783.9121374	-1.390949464	4.08E-07	2.81E-06	PGM2
ENSG00000163728	1146.243392	598.1292139	0.938360146	4.08E-07	2.81E-06	TTC14
ENSG00000106080	386.6652446	1128.926212	-1.545988259	4.10E-07	2.83E-06	FKBP14
ENSG00000138756	442.9266314	135.5740237	1.709168472	4.12E-07	2.84E-06	BMP2K
ENSG00000168288	814.1283912	2047.30764	-1.330516242	4.13E-07	2.85E-06	MMADHC
ENSG00000181035	468.5126339	220.0763989	1.090430286	4.14E-07	2.85E-06	SLC25A42

ENSG00000159917	131.9381214	32.83437289	2.00486399	4.15E-07	2.86E-06	ZNF235
ENSG00000116704	268.5397882	623.7426068	-1.216363148	4.15E-07	2.86E-06	SLC35D1
ENSG00000172007	486.2743906	206.6521838	1.234291525	4.23E-07	2.91E-06	RAB33B
ENSG00000265206	214.1952972	8.050200221	4.752135726	4.23E-07	2.91E-06	AC004687.1
ENSG00000182378	226.632191	1146.09046	-2.33791339	4.24E-07	2.92E-06	PLCXD1
ENSG00000197208	67.57604423	726.8694751	-3.426915305	4.27E-07	2.94E-06	SLC22A4
ENSG00000119523	524.1327175	1098.433211	-1.067193205	4.28E-07	2.94E-06	ALG2
ENSG00000119714	22.31237413	626.4404807	-4.811896391	4.31E-07	2.96E-06	GPR68
ENSG00000150961	623.6441443	3291.233868	-2.399939148	4.33E-07	2.98E-06	SEC24D
ENSG00000069974	170.0937627	610.1539393	-1.843323517	4.35E-07	2.99E-06	RAB27A
ENSG00000146966	941.5467131	143.4734661	2.715799703	4.36E-07	3.00E-06	DENND2A
ENSG00000122574	55.74791302	1.069617038	5.695710749	4.37E-07	3.00E-06	WIPF3
ENSG00000175087	249.620939	69.45849198	1.843635779	4.37E-07	3.00E-06	PDIK1L
ENSG00000128833	37.71623699	1.854670712	4.379730143	4.39E-07	3.01E-06	MYO5C
ENSG00000196705	644.3215899	226.6623123	1.506936172	4.40E-07	3.02E-06	ZNF431
ENSG00000250548	0.879722543	35.59566773	-5.302150694	4.43E-07	3.04E-06	LINC01303
ENSG00000140157	435.0906279	1141.276935	-1.391636182	4.43E-07	3.04E-06	NIPA2
ENSG00000101251	30.53669454	0	7.267152525	4.43E-07	3.04E-06	SEL1L2
novel.950	0	28.07065041	-7.39307003	4.44E-07	3.04E-06	-
ENSG00000119383	1978.508779	4738.505468	-1.260100354	4.48E-07	3.07E-06	PTPA
ENSG00000170004	2527.226691	4560.501609	-0.851615359	4.50E-07	3.08E-06	CHD3
ENSG00000261098	66.6118996	15.5812537	2.102250017	4.52E-07	3.10E-06	AP000766.1
ENSG00000064666	1120.50663	5059.108198	-2.174789172	4.54E-07	3.11E-06	CNN2
ENSG00000141639	59.92803344	0	8.238549673	4.56E-07	3.12E-06	MAPK4
ENSG00000140575	2708.932744	5668.391697	-1.065166155	4.56E-07	3.12E-06	IQGAP1
ENSG00000068796	253.3299854	570.7175176	-1.172203773	4.61E-07	3.15E-06	KIF2A
ENSG00000159588	60.20691503	13.58494344	2.145360324	4.61E-07	3.15E-06	CCDC17
ENSG00000087116	1111.939838	3146.220899	-1.500601164	4.61E-07	3.15E-06	ADAMTS2
ENSG00000232682	36.67086174	0	7.531182151	4.62E-07	3.15E-06	AL592430.1
ENSG00000177311	1348.284411	3307.052829	-1.294314836	4.62E-07	3.15E-06	ZBTB38
ENSG00000114416	1755.010539	3247.015526	-0.887599616	4.63E-07	3.16E-06	FXR1
ENSG00000145901	1329.01445	10351.81742	-2.961439403	4.64E-07	3.17E-06	TNIP1
ENSG00000162444	57.1907252	2.91483114	4.30556805	4.66E-07	3.18E-06	RBP7
ENSG00000152455	95.32814719	289.9143715	-1.605734732	4.67E-07	3.19E-06	SUV39H2
ENSG00000152270	112.5755209	28.91000361	1.96392065	4.69E-07	3.20E-06	PDE3B
ENSG00000159307	61.49782086	0	8.275859232	4.70E-07	3.21E-06	SCUBE1
ENSG00000227051	152.272416	574.7485	-1.915799796	4.71E-07	3.21E-06	C14orf132
ENSG00000118515	3608.447613	868.8496585	2.054176513	4.72E-07	3.22E-06	SGK1
novel.905	5597.02658	31.40145716	7.476392402	4.72E-07	3.22E-06	-
ENSG00000272808	128.01084	8.253331325	3.971835717	4.75E-07	3.24E-06	AC015712.6
ENSG00000086504	893.7930151	1873.749854	-1.068041265	4.75E-07	3.24E-06	MRPL28
ENSG00000156219	62.61150675	1.426156051	5.448863926	4.77E-07	3.25E-06	ART3
ENSG00000121797	43.87318333	0.387220594	6.826660176	4.77E-07	3.25E-06	CCRL2
ENSG00000167595	127.2119208	406.5562184	-1.675360874	4.82E-07	3.28E-06	PROSER3
ENSG00000171587	40.78482316	0	7.683117791	4.89E-07	3.33E-06	DSCAM
ENSG00000158163	124.5320944	339.2984989	-1.446998568	4.90E-07	3.34E-06	DZIP1L
ENSG00000140526	873.0385316	2541.668101	-1.541579936	4.94E-07	3.36E-06	ABHD2
ENSG00000260118	43.20561357	0.387220594	6.805913806	4.94E-07	3.36E-06	AL157700.1
ENSG00000284730	36.39072685	2.51699806	3.855281466	4.96E-07	3.37E-06	C12orf81
ENSG00000154309	322.3953697	98.68237505	1.707213748	4.98E-07	3.39E-06	DISP1
ENSG00000223382	23.92826182	0	6.914325212	4.99E-07	3.39E-06	LINC01778

ENSG00000198822	65.42119949	0	8.365140778	4.99E-07	3.39E-06	GRM3
ENSG00000167642	527.9584245	21.20230947	4.63242821	5.00E-07	3.39E-06	SPINT2
ENSG00000131055	23.93954466	0	6.914936257	5.02E-07	3.41E-06	COX4I2
ENSG00000225194	49.73627627	0	7.969527711	5.07E-07	3.44E-06	LINC00092
ENSG00000173110	406.8461802	55.47635776	2.876427527	5.08E-07	3.44E-06	HSPA6
novel.292	80.67270123	19.93413764	2.012495642	5.09E-07	3.45E-06	-
ENSG00000143324	412.4636731	827.7483749	-1.004898269	5.09E-07	3.45E-06	XPR1
ENSG00000088298	382.0347927	827.7952391	-1.115360926	5.09E-07	3.45E-06	EDEM2
ENSG00000063978	846.2510718	1771.191639	-1.065496918	5.12E-07	3.47E-06	RNF4
ENSG00000173272	619.4490837	1271.968364	-1.038034334	5.15E-07	3.49E-06	MZT2A
ENSG00000113205	46.56528929	0.713078025	6.019940927	5.15E-07	3.49E-06	PCDHB3
ENSG00000101182	2580.96001	5345.525499	-1.050478025	5.15E-07	3.49E-06	PSMA7
novel.784	32.12062775	0	7.338420513	5.17E-07	3.50E-06	-
ENSG00000177363	332.925083	14.4102637	4.526155018	5.19E-07	3.51E-06	LRRN4CL
novel.30	143.7598415	32.40560144	2.154467818	5.20E-07	3.52E-06	-
ENSG00000234155	0.921306765	54.44431038	-5.896884602	5.21E-07	3.52E-06	LINC02535
ENSG00000120328	40.22710447	1.386017859	4.820784538	5.22E-07	3.53E-06	PCDHB12
ENSG00000179761	40.51658437	2.567748738	3.999389349	5.23E-07	3.53E-06	PIPOX
ENSG00000185565	336.1106923	25.8498085	3.704901551	5.23E-07	3.54E-06	LSAMP
ENSG00000198715	517.938161	1211.388016	-1.225657001	5.23E-07	3.54E-06	GLMP
ENSG00000153561	1109.218902	417.9560571	1.407543592	5.25E-07	3.55E-06	RMND5A
ENSG00000232859	197.6639868	47.91171583	2.039672306	5.25E-07	3.55E-06	LYRM9
ENSG00000145287	60.15780892	6.338453325	3.271571547	5.26E-07	3.55E-06	PLAC8
ENSG00000152207	93.47009043	2.375358513	5.262116862	5.28E-07	3.57E-06	CYSLTR2
ENSG00000138772	1730.778752	112.3051106	3.944624036	5.31E-07	3.59E-06	ANXA3
ENSG00000234571	48.33175807	0.672939833	6.088986449	5.37E-07	3.62E-06	AC239798.2
ENSG00000100764	624.4155434	1272.975495	-1.027879621	5.38E-07	3.63E-06	PSMC1
ENSG00000177508	74.25582022	921.3586491	-3.633834321	5.38E-07	3.63E-06	IRX3
ENSG00000090273	1714.386782	3822.295125	-1.156805741	5.39E-07	3.63E-06	NUDC
ENSG00000196470	410.4086224	160.3848155	1.357083968	5.41E-07	3.65E-06	SIAH1
ENSG00000138688	2342.334569	950.3552304	1.301500458	5.43E-07	3.66E-06	KIAA1109
ENSG00000101150	2280.153445	4745.304111	-1.057415783	5.44E-07	3.67E-06	TPD52L2
ENSG00000173598	3446.3375	1037.211649	1.732118067	5.45E-07	3.67E-06	NUDT4
ENSG00000151468	179.8359004	12.76103584	3.805909354	5.46E-07	3.68E-06	CCDC3
ENSG00000235954	451.4446634	109.7878901	2.040402678	5.49E-07	3.69E-06	TTC28-AS1
ENSG00000283041	1861.270807	4836.20133	-1.377636399	5.50E-07	3.70E-06	AC008038.1
ENSG00000251867	60.77068851	10.18126196	2.586399528	5.50E-07	3.70E-06	AC009812.1
ENSG00000105401	3011.873273	6209.808595	-1.04383965	5.50E-07	3.70E-06	CDC37
ENSG00000239672	408.6230641	1137.133921	-1.476875648	5.50E-07	3.70E-06	NME1
ENSG00000064393	2466.591587	6870.222576	-1.477794634	5.59E-07	3.76E-06	HIPK2
ENSG00000197265	356.1337075	1036.784231	-1.541938485	5.59E-07	3.76E-06	GTF2E2
ENSG00000134698	865.4299711	353.4582073	1.292372079	5.59E-07	3.76E-06	AGO4
ENSG00000111087	249.1344774	16.45732464	3.919223321	5.61E-07	3.77E-06	GLI1
ENSG00000105664	0.909952655	157.2812498	-7.435115301	5.62E-07	3.77E-06	COMP
ENSG00000197283	10.23348429	63.18127343	-2.623938352	5.62E-07	3.77E-06	SYNGAP1
ENSG00000162951	297.0521648	0	10.54831585	5.64E-07	3.78E-06	LRRTM1
ENSG00000116819	85.51165967	16.00976836	2.424665274	5.66E-07	3.80E-06	TFAP2E
ENSG00000180318	0	36.69314102	-7.776236338	5.71E-07	3.83E-06	ALX1
ENSG00000065978	11003.06991	20888.35617	-0.924806371	5.74E-07	3.85E-06	YBX1
ENSG00000272031	60.89920212	162.5917057	-1.416973213	5.75E-07	3.85E-06	ANKRD34A
ENSG00000279360	39.10289154	0	7.623258485	5.83E-07	3.91E-06	AC007546.2

ENSG00000198612	1071.027883	2362.530001	-1.141482081	5.84E-07	3.91E-06	COPS8
ENSG00000035664	137.9500273	10.99596973	3.644750516	5.84E-07	3.91E-06	DAPK2
ENSG00000254054	23.94086958	0	6.915017422	5.88E-07	3.94E-06	AC087273.2
ENSG00000168005	352.5499276	790.2665036	-1.164172616	5.91E-07	3.96E-06	SPINDOC
ENSG00000152779	1811.098674	98.2560906	4.202910298	5.93E-07	3.97E-06	SLC16A12
ENSG00000205502	47.21473886	0.336469917	6.933138179	5.94E-07	3.98E-06	C2CD4B
ENSG00000152672	23.83624656	0	6.909290562	5.94E-07	3.98E-06	CLEC4F
ENSG00000229644	63.05017156	1128.446135	-4.16162243	5.97E-07	4.00E-06	NAMPTP1
ENSG00000136930	1589.118209	3256.002147	-1.034809935	5.98E-07	4.00E-06	PSMB7
ENSG00000245904	37.04658685	2.241891306	4.08629274	6.03E-07	4.03E-06	AC025164.1
ENSG00000228203	2.775203133	93.55847036	-5.084525501	6.04E-07	4.04E-06	RNF144A-AS1
ENSG00000178568	55.30158413	1.00940975	5.709264372	6.05E-07	4.05E-06	ERBB4
ENSG00000093144	2416.514466	1216.473897	0.990044912	6.05E-07	4.05E-06	ECHDC1
ENSG00000226711	94.82405066	26.1817021	1.856865045	6.07E-07	4.05E-06	FAM66C
ENSG00000077348	234.4332742	500.8711158	-1.095224757	6.08E-07	4.06E-06	EXOSC5
ENSG00000082516	262.6217482	618.8576991	-1.23720366	6.12E-07	4.09E-06	GEMIN5
ENSG00000284650	42.8365127	0	7.755217923	6.16E-07	4.11E-06	AL031430.1
ENSG00000275713	3.965860148	61.78772553	-3.965105872	6.17E-07	4.12E-06	HIST1H2BH
ENSG00000250303	208.885677	36.40323064	2.519103868	6.17E-07	4.12E-06	AP002884.1
ENSG00000185129	1276.849037	574.3365181	1.15279136	6.19E-07	4.13E-06	PURA
ENSG00000205581	2716.336233	5317.379878	-0.969113023	6.19E-07	4.13E-06	HMGN1
novel.1045	2.420248412	78.95962239	-5.025826687	6.22E-07	4.15E-06	-
ENSG00000102898	1298.3183	2578.884871	-0.990137911	6.22E-07	4.15E-06	NUTF2
ENSG00000261218	23.82113151	0	6.908454659	6.28E-07	4.19E-06	AC099524.1
ENSG00000114491	305.0471763	790.1452153	-1.373517436	6.29E-07	4.19E-06	UMPS
ENSG00000130653	404.2991885	95.63253565	2.08124533	6.31E-07	4.20E-06	PNPLA7
ENSG00000167850	57.11375373	1.426156051	5.316113916	6.32E-07	4.21E-06	CD300C
ENSG00000169136	287.8280953	815.3290411	-1.502109912	6.32E-07	4.21E-06	ATF5
ENSG00000160193	106.9546965	411.6218549	-1.945088148	6.32E-07	4.21E-06	WDR4
ENSG00000152642	935.595863	206.0349218	2.181707214	6.33E-07	4.21E-06	GPD1L
ENSG00000115761	277.9532985	646.2319201	-1.217564099	6.38E-07	4.24E-06	NOL10
ENSG00000092529	89.48593935	10.5661708	3.089375802	6.39E-07	4.25E-06	CAPN3
ENSG00000115468	731.1813293	20.40420316	5.159584179	6.40E-07	4.26E-06	EFHD1
ENSG00000131148	401.7557807	826.1776044	-1.040471464	6.43E-07	4.27E-06	EMC8
ENSG00000146021	319.8206798	100.1706178	1.677480003	6.44E-07	4.28E-06	KLHL3
ENSG00000267023	116.5311384	39.46085721	1.562140149	6.46E-07	4.29E-06	LRRC37A16P
ENSG00000196449	214.6034753	495.9534375	-1.208519704	6.47E-07	4.30E-06	YRDC
ENSG00000186111	858.2335603	1971.989117	-1.200012056	6.54E-07	4.34E-06	PIP5K1C
ENSG00000100346	74.70813367	2.323323566	5.060830304	6.55E-07	4.35E-06	CACNA1I
ENSG00000164695	90.76846943	5.104815894	4.160433646	6.56E-07	4.35E-06	CHMP4C
ENSG00000123213	349.3049754	1025.291555	-1.553730596	6.56E-07	4.35E-06	NLN
ENSG00000174173	253.2680577	570.0390211	-1.171010868	6.56E-07	4.35E-06	TRMT10C
ENSG00000177465	66.00632809	8.681974381	2.931053548	6.61E-07	4.38E-06	ACOT4
ENSG00000163013	108.7039698	389.0807853	-1.838983308	6.62E-07	4.39E-06	FBXO41
ENSG00000146233	127.2457544	8.528438079	3.910728161	6.73E-07	4.46E-06	CYP39A1
ENSG00000171100	374.9755581	181.7632517	1.044901103	6.77E-07	4.49E-06	MTM1
ENSG00000120068	3.694002601	51.32087272	-3.806558662	6.79E-07	4.50E-06	HOXB8
ENSG00000267369	32.14082867	0	7.339328775	6.80E-07	4.50E-06	AC015911.7
ENSG00000120875	220.3326131	3227.913835	-3.87276134	6.90E-07	4.57E-06	DUSP4
ENSG00000198252	269.1479383	592.2783738	-1.138278241	6.91E-07	4.57E-06	STYX
ENSG00000150347	531.5334024	1280.033367	-1.268106108	6.94E-07	4.60E-06	ARID5B

ENSG00000175567	713.6906823	91.84758819	2.955653093	6.95E-07	4.60E-06	UCP2
ENSG00000100347	541.3862176	1146.163689	-1.082113169	6.96E-07	4.61E-06	SAMM50
ENSG00000107372	4892.589375	2196.462416	1.155408657	7.01E-07	4.64E-06	ZFAND5
ENSG00000165512	616.3304655	291.6198254	1.07948873	7.02E-07	4.64E-06	ZNF22
ENSG00000203778	565.9542833	257.6344032	1.135022001	7.05E-07	4.66E-06	FAM229B
ENSG00000177606	21466.58488	2730.45985	2.974920488	7.11E-07	4.70E-06	JUN
novel.420	22.73384386	203.9851517	-3.164753361	7.11E-07	4.70E-06	-
ENSG00000160321	24.03908181	0	6.920409575	7.13E-07	4.71E-06	ZNF208
ENSG00000075914	336.8458006	768.3442065	-1.190000094	7.16E-07	4.73E-06	EXOSC7
ENSG00000118900	555.1595325	1181.481398	-1.089725248	7.18E-07	4.74E-06	UBN1
ENSG00000272990	67.15142647	9.835463823	2.759447164	7.19E-07	4.75E-06	AC084036.1
ENSG00000073536	209.1343709	487.4677062	-1.22139369	7.22E-07	4.77E-06	NLE1
ENSG00000065135	1305.211397	2668.902699	-1.032021918	7.23E-07	4.77E-06	GNAI3
ENSG00000203875	914.8799715	3249.350745	-1.828607804	7.27E-07	4.80E-06	SNHG5
ENSG00000144837	29.00551368	0	7.191294733	7.28E-07	4.80E-06	PLA1A
ENSG00000145861	93.48174453	8.846123169	3.395523052	7.29E-07	4.80E-06	C1QTNF2
ENSG00000230587	44.82479148	1.00940975	5.402024658	7.29E-07	4.80E-06	LINC02580
ENSG00000197599	60.51240763	7.481330282	3.012135031	7.30E-07	4.81E-06	CCDC154
ENSG00000273923	22.65701493	0	6.835946904	7.31E-07	4.82E-06	AC104260.1
ENSG00000170166	2.152223082	50.50428556	-4.565735477	7.35E-07	4.84E-06	HOXD4
ENSG00000215021	1750.162958	4431.624969	-1.340411928	7.37E-07	4.85E-06	PHB2
ENSG00000212719	256.418559	586.1395371	-1.192732905	7.39E-07	4.86E-06	C17orf51
ENSG00000198093	235.0233034	97.17016324	1.272677267	7.40E-07	4.87E-06	ZNF649
ENSG00000134001	961.7308803	2304.742486	-1.261056497	7.41E-07	4.88E-06	EIF2S1
ENSG00000103994	3142.219705	1431.623541	1.134143738	7.48E-07	4.92E-06	ZNF106
ENSG00000128408	4.499403511	39.02044579	-3.107870471	7.50E-07	4.93E-06	RIBC2
novel.344	103.5799057	20.35088395	2.345805814	7.51E-07	4.94E-06	-
ENSG00000163359	3332.865548	65504.85935	-4.296778839	7.57E-07	4.98E-06	COL6A3
ENSG00000205116	36.93569559	1.813376645	4.358338223	7.58E-07	4.98E-06	TMEM88B
ENSG00000168398	40.44372985	445.6629986	-3.462726914	7.59E-07	4.98E-06	BDKRB2
ENSG00000152377	58.27202934	695.0383755	-3.577110841	7.60E-07	4.99E-06	SPOCK1
ENSG00000070950	122.1364486	424.2467609	-1.797325192	7.62E-07	5.00E-06	RAD18
ENSG00000019186	1.22840902	98.80513574	-6.338657421	7.67E-07	5.03E-06	CYP24A1
ENSG00000139641	1546.977857	3055.505413	-0.982013294	7.72E-07	5.07E-06	ESYT1
ENSG00000213080	31.95282375	108.1696006	-1.758472416	7.73E-07	5.07E-06	AL354714.2
ENSG00000104147	4.874559155	61.02916229	-3.651085585	7.74E-07	5.07E-06	OIP5
ENSG00000171657	49.40213503	1.060160428	5.526076333	7.75E-07	5.08E-06	GPR82
ENSG00000183208	64.64363576	13.8399811	2.216053235	7.78E-07	5.10E-06	GDPGP1
ENSG00000177990	420.8893779	90.93770638	2.21042124	7.78E-07	5.10E-06	DPY19L2
ENSG00000115486	647.2154468	1243.698841	-0.942317289	7.83E-07	5.13E-06	GGCX
ENSG00000232119	658.0843332	1271.759791	-0.95061896	7.88E-07	5.16E-06	MCTS1
ENSG00000114698	2213.293026	232.0760587	3.252717692	7.91E-07	5.18E-06	PLSCR4
ENSG00000132635	1138.537543	550.6765791	1.047862993	7.95E-07	5.20E-06	PCED1A
ENSG00000277135	49.94424082	4.300848999	3.537408038	7.98E-07	5.22E-06	AC012409.3
ENSG00000187486	31.18560212	0	7.295866487	8.06E-07	5.28E-06	KCNJ11
ENSG00000005100	364.3575019	726.7963467	-0.996236868	8.17E-07	5.35E-06	DHX33
ENSG00000265296	25.66506996	0	7.015763808	8.18E-07	5.35E-06	FEM1AP2
ENSG00000068654	570.7200193	1720.493831	-1.591891457	8.18E-07	5.35E-06	POLR1A
ENSG00000255571	48.48354902	0	7.932752905	8.28E-07	5.42E-06	MIR9-3HG
ENSG00000140961	290.062675	76.4710882	1.921079402	8.29E-07	5.42E-06	OSGIN1
ENSG00000225670	23.41610176	0	6.882595784	8.29E-07	5.42E-06	CADM3-AS1

ENSG00000105048	0.923814062	85.76107759	-6.54927994	8.30E-07	5.42E-06	TNNT1
ENSG00000090339	373.1238123	1957.287069	-2.391031248	8.31E-07	5.43E-06	ICAM1
ENSG00000272789	22.43433477	0	6.821185524	8.35E-07	5.45E-06	AC010976.2
ENSG00000100226	741.4298809	1772.755393	-1.257364972	8.37E-07	5.46E-06	GTPBP1
ENSG00000179083	26.85594044	0	7.080156446	8.39E-07	5.47E-06	FAM133A
ENSG00000131355	24.37390688	0	6.940265032	8.43E-07	5.50E-06	ADGRE3
ENSG00000278921	28.11576195	0	7.146244918	8.44E-07	5.50E-06	EPB41L4A-DT
ENSG00000164818	455.8538588	985.0292396	-1.111759699	8.46E-07	5.52E-06	DNAAF5
ENSG00000286190	270.3845526	83.92014866	1.689428391	8.47E-07	5.52E-06	AC055839.2
ENSG00000134308	4377.606984	8671.683784	-0.986201425	8.49E-07	5.53E-06	YWHAQ
ENSG00000101224	881.3984212	2567.460771	-1.542394604	8.49E-07	5.53E-06	CDC25B
ENSG00000277672	30.19058663	0	7.250718103	8.53E-07	5.56E-06	AC011603.4
ENSG00000100385	134.8727362	4.799027558	4.823765647	8.57E-07	5.58E-06	IL2RB
ENSG00000035687	602.5698419	1226.750671	-1.025813898	8.62E-07	5.61E-06	ADSS
ENSG00000166974	2164.058656	560.2246192	1.949244179	8.63E-07	5.61E-06	MAPRE2
ENSG00000226200	131.4162169	24.62580326	2.409548926	8.63E-07	5.61E-06	SGMS1-AS1
ENSG00000054219	172.947191	9.601779531	4.157548367	8.63E-07	5.62E-06	LY75
ENSG00000144867	498.8441559	1376.369164	-1.464506131	8.67E-07	5.64E-06	SRPRB
ENSG00000266088	4.622831258	109.1605638	-4.568056735	8.68E-07	5.65E-06	AC004585.1
ENSG00000162739	40.20725955	0	7.662549172	8.71E-07	5.66E-06	SLAMF6
ENSG00000183378	42.15167624	0	7.732026927	8.71E-07	5.66E-06	OVCH2
ENSG00000139146	16.18676901	105.0693485	-2.701218571	8.74E-07	5.68E-06	SINHCAF
ENSG00000106948	883.7309355	261.6292883	1.756175089	8.79E-07	5.71E-06	AKNA
ENSG00000261959	26.2364366	0	7.047633857	8.80E-07	5.71E-06	AC015909.3
ENSG00000116489	2037.521122	3992.919504	-0.970708124	8.81E-07	5.72E-06	CAPZA1
ENSG00000229152	396.0157046	101.3691257	1.966867225	8.83E-07	5.73E-06	ANKRD10-IT1
ENSG00000162736	1764.48146	3501.202103	-0.988537398	8.86E-07	5.75E-06	NCSTN
ENSG00000185904	360.9999456	12.63000878	4.828469899	8.86E-07	5.75E-06	LINC00839
ENSG00000182183	47.51847927	1.803920035	4.725224932	8.90E-07	5.77E-06	SHISAL2A
ENSG00000182141	381.6868776	94.45473879	2.014977964	8.96E-07	5.81E-06	ZNF708
novel.744	0.308355904	41.85307398	-7.008298257	8.96E-07	5.81E-06	-
ENSG00000211592	1235.109381	0	12.60407159	8.97E-07	5.81E-06	IGKC
ENSG00000135045	69.97791484	359.7791728	-2.363422127	8.97E-07	5.81E-06	C9orf40
ENSG00000105698	3957.651668	1735.053451	1.18983681	8.97E-07	5.81E-06	USF2
ENSG00000163596	210.9334346	73.8868192	1.510580129	8.99E-07	5.82E-06	ICA1L
ENSG00000113209	70.67786636	2.375358513	4.855357618	9.07E-07	5.87E-06	PCDHB5
ENSG00000121653	942.864067	185.279561	2.347796088	9.09E-07	5.88E-06	MAPK8IP1
ENSG00000186407	39.00782735	0	7.61914435	9.10E-07	5.89E-06	CD300E
ENSG00000165669	887.6625379	408.0209372	1.120673282	9.11E-07	5.90E-06	FAM204A
ENSG00000161203	5801.432416	13251.60499	-1.191711683	9.14E-07	5.91E-06	AP2M1
ENSG00000187372	51.93975676	2.965581817	4.15503938	9.14E-07	5.91E-06	PCDHB13
ENSG00000122733	53.30069724	4.02458637	3.741132975	9.15E-07	5.91E-06	PHF24
novel.602	0.293240848	382.1351436	-10.19720379	9.20E-07	5.94E-06	-
ENSG00000047617	61.25605123	1.00940975	5.86034532	9.28E-07	5.99E-06	ANO2
ENSG00000179933	647.784433	1450.86277	-1.163411382	9.29E-07	6.00E-06	C14orf119
ENSG00000057704	558.3238521	46.52488043	3.588728585	9.41E-07	6.08E-06	TMCC3
ENSG00000148700	7284.841636	2159.908802	1.753815357	9.49E-07	6.13E-06	ADD3
ENSG00000137073	529.4618127	1226.953661	-1.212287687	9.50E-07	6.13E-06	UBAP2
ENSG00000110427	196.7590487	479.8451899	-1.286274803	9.52E-07	6.14E-06	KIAA1549L
ENSG00000139318	4017.692254	1039.190421	1.95106514	9.59E-07	6.18E-06	DUSP6
ENSG00000250802	69.82132276	6.604231863	3.384081946	9.60E-07	6.20E-06	ZBED3-AS1

ENSG00000165475	1158.101487	275.3064052	2.073306326	9.70E-07	6.25E-06	CRYL1
ENSG00000227544	38.37138498	1.41669944	4.746768886	9.85E-07	6.35E-06	AC018647.1
ENSG00000163794	91.06819164	15.97091444	2.506960368	9.86E-07	6.36E-06	UCN
ENSG00000106153	2797.075801	6197.396475	-1.147740226	9.89E-07	6.37E-06	CHCHD2
ENSG00000229422	38.53263601	0.743759607	5.740037885	9.98E-07	6.43E-06	AL512625.2
ENSG00000175564	66.94776485	12.08681174	2.464531479	1.02E-06	6.55E-06	UCP3
ENSG00000155970	319.664985	139.8266905	1.193482601	1.02E-06	6.56E-06	MICU3
ENSG00000163430	9185.520017	26274.47872	-1.516245236	1.02E-06	6.58E-06	FSTL1
ENSG00000187105	61.68927451	7.489631017	3.038526461	1.02E-06	6.59E-06	HEATR4
ENSG00000074370	242.5403906	29.48002942	3.044999126	1.03E-06	6.63E-06	ATP2A3
ENSG00000005981	45.02175793	0.672939833	5.987409509	1.03E-06	6.64E-06	ASB4
ENSG00000132646	888.5096917	2853.692775	-1.683510922	1.04E-06	6.66E-06	PCNA
ENSG00000087301	279.0543328	99.22210447	1.489545201	1.04E-06	6.67E-06	TXNDC16
ENSG00000183066	51.61320903	7.683177117	2.761809994	1.04E-06	6.68E-06	WBP2NL
ENSG00000233369	1001.51928	307.5607349	1.70423878	1.04E-06	6.71E-06	GTF2IP4
ENSG00000277443	15763.56907	4070.092326	1.953389135	1.05E-06	6.72E-06	MARCKS
ENSG00000085265	26.54464996	0	7.064430112	1.05E-06	6.73E-06	FCN1
ENSG00000182872	983.6216574	1730.416062	-0.81490751	1.05E-06	6.74E-06	RBM10
ENSG00000285894	48.52284011	1.080229524	5.492173325	1.05E-06	6.74E-06	AL136372.2
ENSG00000121671	670.9598477	269.0749755	1.31856262	1.05E-06	6.76E-06	CRY2
ENSG00000108798	440.8666211	31.28138092	3.822241375	1.06E-06	6.82E-06	ABI3
ENSG00000154310	104.813428	13.42207892	2.958396572	1.06E-06	6.82E-06	TNIK
ENSG00000115649	1542.930541	667.5219083	1.208812786	1.07E-06	6.84E-06	CNPPD1
ENSG00000162458	233.5048714	1853.573596	-2.988880172	1.07E-06	6.84E-06	FBLIM1
ENSG00000116120	654.804065	1845.477591	-1.495038631	1.07E-06	6.88E-06	FARSB
ENSG00000244486	708.2593731	2092.283431	-1.562937331	1.08E-06	6.89E-06	SCARF2
ENSG00000228252	26.56269959	0	7.064302078	1.08E-06	6.92E-06	COL6A4P2
ENSG00000198768	2.46684723	671.6856215	-8.093367156	1.08E-06	6.93E-06	APCDD1L
ENSG00000182685	237.1009914	85.39479082	1.473400643	1.08E-06	6.93E-06	BRICD5
ENSG00000006125	2643.376315	5527.179126	-1.064211617	1.08E-06	6.93E-06	AP2B1
ENSG00000172137	34.48904929	0.387220594	6.480111711	1.09E-06	6.95E-06	CALB2
ENSG00000272343	43.98615496	3.342189926	3.746586029	1.09E-06	6.99E-06	AC107952.2
ENSG00000178075	28.25910588	105.0007121	-1.894244478	1.09E-06	7.01E-06	GRAMD1C
ENSG00000196177	759.2181505	273.3719714	1.473398308	1.10E-06	7.01E-06	ACADSB
ENSG00000131873	701.6548438	1652.167917	-1.235637502	1.10E-06	7.06E-06	CHSY1
ENSG00000000419	467.4819027	1039.582758	-1.153159045	1.11E-06	7.12E-06	DPM1
ENSG00000177839	40.24820304	0.713078025	5.810117829	1.11E-06	7.13E-06	PCDHB9
ENSG00000140279	46.97623163	0.387220594	6.926502831	1.12E-06	7.16E-06	DUOX2
ENSG00000148926	707.5042259	8808.18654	-3.638096885	1.13E-06	7.19E-06	ADM
ENSG00000119862	419.8425464	33.37183774	3.648337193	1.13E-06	7.19E-06	LGALSL
ENSG00000204673	992.3476807	1995.682357	-1.007986945	1.13E-06	7.20E-06	AKT1S1
ENSG00000272010	55.94405309	10.02900992	2.478941503	1.13E-06	7.21E-06	AC100814.1
ENSG00000259803	10.57590252	153.1149514	-3.854370911	1.13E-06	7.21E-06	SLC22A31
ENSG00000184378	18.9343206	94.08403845	-2.316598468	1.13E-06	7.23E-06	ACTRT3
ENSG00000185359	1125.538473	2654.129368	-1.237497324	1.13E-06	7.24E-06	HGS
ENSG00000185551	5550.059759	2601.046677	1.093297077	1.14E-06	7.26E-06	NR2F2
ENSG00000110042	1462.198538	168.1449641	3.121405699	1.14E-06	7.27E-06	DTX4
ENSG00000237248	36.15640784	0.387220594	6.548666329	1.15E-06	7.31E-06	LINC00987
ENSG00000225342	35.78293312	3.607840069	3.314833028	1.15E-06	7.36E-06	AC079630.1
ENSG00000115415	2675.160861	7682.77029	-1.522051279	1.16E-06	7.36E-06	STAT1
ENSG00000182168	88.96370596	1.702418679	5.670291906	1.16E-06	7.42E-06	UNC5C

ENSG00000139547	37.71874429	2.92428775	3.704869905	1.17E-06	7.42E-06	RDH16
ENSG00000157856	46.28046833	0.743759607	6.001475912	1.17E-06	7.44E-06	DRC1
ENSG00000136928	8.237497632	53.52378171	-2.703140353	1.17E-06	7.44E-06	GABBR2
ENSG00000136840	222.5710376	778.6102612	-1.806949916	1.17E-06	7.44E-06	ST6GALNAC4
ENSG00000221995	118.3608594	37.40202791	1.65908585	1.18E-06	7.48E-06	TIAF1
ENSG00000158125	0	25.93355251	-7.274908319	1.18E-06	7.50E-06	XDH
ENSG00000011332	11.94027577	103.8919844	-3.1243746	1.18E-06	7.50E-06	DPF1
ENSG00000108788	583.9271953	1173.056448	-1.006426354	1.19E-06	7.55E-06	MLX
ENSG00000081041	57.05655591	929.582555	-4.026091171	1.20E-06	7.62E-06	CXCL2
ENSG00000100697	2554.974602	798.0718113	1.678682491	1.20E-06	7.62E-06	DICER1
ENSG00000054277	89.94490418	231.6411101	-1.364167817	1.20E-06	7.62E-06	OPN3
ENSG00000108039	806.2493683	1642.219954	-1.026290114	1.21E-06	7.68E-06	XPNPEP1
ENSG00000226891	37.48156198	1.773238453	4.390920139	1.21E-06	7.68E-06	LINC01359
novel.377	7.974202162	140.1726642	-4.128769922	1.21E-06	7.70E-06	-
novel.21	33.69554412	0	7.409071321	1.22E-06	7.72E-06	-
ENSG00000162896	21.84659943	0	6.782776169	1.22E-06	7.72E-06	PIGR
ENSG00000011376	304.6213327	865.1080257	-1.506367158	1.22E-06	7.72E-06	LARS2
ENSG00000166225	887.8792943	291.2046542	1.608265996	1.22E-06	7.74E-06	FRS2
ENSG00000023892	695.0937647	137.9398912	2.331438667	1.22E-06	7.75E-06	DEF6
ENSG00000277587	22.47584772	0	6.823630956	1.22E-06	7.75E-06	AC008759.3
ENSG00000167881	1269.795642	2413.615263	-0.926714991	1.23E-06	7.80E-06	SRP68
ENSG00000145740	685.7970036	1240.187751	-0.854588237	1.24E-06	7.83E-06	SLC30A5
ENSG00000170689	0.307102255	30.89916901	-6.56690444	1.24E-06	7.84E-06	HOXB9
ENSG00000175262	26.47834877	0	7.060018282	1.25E-06	7.91E-06	C1orf127
ENSG00000231969	43.92025287	0.387220594	6.829649033	1.25E-06	7.92E-06	AC007364.1
ENSG00000072858	45.476748	1.702418679	4.690549233	1.25E-06	7.94E-06	SIDT1
ENSG00000177951	650.857464	1495.854518	-1.200686345	1.26E-06	7.94E-06	BET1L
ENSG00000164163	952.1639846	2296.063195	-1.269972228	1.26E-06	7.96E-06	ABCE1
ENSG00000113282	924.0698741	1862.421647	-1.011187509	1.26E-06	7.97E-06	CLINT1
novel.887	360.2406031	0	10.82650823	1.26E-06	7.98E-06	-
ENSG00000158683	71.15206096	6.317228354	3.512023686	1.26E-06	7.98E-06	PKD1L1
ENSG00000144959	148.1113847	473.2594174	-1.676454675	1.27E-06	8.00E-06	NCEH1
ENSG00000196812	68.14078662	15.41954506	2.144057733	1.27E-06	8.01E-06	ZSCAN16
ENSG00000126353	18.23311543	191.3102053	-3.391250647	1.27E-06	8.01E-06	CCR7
ENSG00000186866	785.2782764	1969.53591	-1.326368061	1.27E-06	8.02E-06	POFUT2
ENSG00000237061	26.12429169	0	7.041817299	1.28E-06	8.06E-06	AC100823.1
ENSG00000287306	51.09707456	0.713078025	6.155225398	1.28E-06	8.08E-06	AC016821.1
ENSG00000212232	3.915429114	35.95464689	-3.19649458	1.30E-06	8.19E-06	SNORD17
ENSG00000259236	59.66130555	5.361009426	3.474603509	1.30E-06	8.19E-06	GOLGA8VP
ENSG00000138115	30.30824433	0	7.254635428	1.31E-06	8.25E-06	CYP2C8
ENSG00000148488	98.14292509	5.290189653	4.205351192	1.31E-06	8.28E-06	ST8SIA6
ENSG00000233608	72.34543838	631.743748	-3.126930664	1.31E-06	8.29E-06	TWIST2
ENSG00000244306	57.84644232	0	8.187555445	1.32E-06	8.29E-06	DUXAP10
ENSG00000169908	1070.197432	5977.80922	-2.48181112	1.32E-06	8.33E-06	TM4SF1
ENSG00000163071	762.2542511	226.9613881	1.746872911	1.32E-06	8.33E-06	SPATA18
ENSG00000138111	165.8043329	63.82571503	1.377279671	1.33E-06	8.35E-06	MFSD13A
ENSG00000184898	571.4422637	158.8160393	1.845465144	1.33E-06	8.35E-06	RBM43
ENSG00000069011	0.925067711	34.83286651	-5.253061842	1.33E-06	8.35E-06	PITX1
ENSG00000143641	2387.352891	4425.212317	-0.890324139	1.33E-06	8.39E-06	GALNT2
ENSG00000172115	1539.141129	3796.085167	-1.302489606	1.34E-06	8.41E-06	CYCS
ENSG00000099364	421.5266304	1047.54084	-1.312983443	1.35E-06	8.46E-06	FBXL19

ENSG00000127472	46.23834212	0.387220594	6.902308332	1.36E-06	8.53E-06	PLA2G5
ENSG00000047315	1048.579561	2050.401046	-0.967367723	1.36E-06	8.54E-06	POLR2B
ENSG00000132376	892.091922	375.9575851	1.245891405	1.36E-06	8.55E-06	INPP5K
ENSG00000166473	54.62305935	0.713078025	6.251904694	1.36E-06	8.56E-06	PKD1L2
ENSG00000077420	1000.316883	63.62176638	3.977120525	1.38E-06	8.67E-06	APBB1IP
ENSG00000166670	0	24.52083428	-7.199647678	1.38E-06	8.67E-06	MMP10
ENSG00000157837	1200.099914	628.4008942	0.933613191	1.39E-06	8.70E-06	SPPL3
ENSG00000101695	264.4566111	37.49137572	2.822120742	1.39E-06	8.74E-06	RNF125
novel.155	130.9722959	27.39180281	2.259319783	1.39E-06	8.75E-06	-
ENSG00000120051	10.68546886	67.69535244	-2.665662169	1.40E-06	8.77E-06	CFAP58
ENSG00000130202	1536.087085	4159.192983	-1.437077368	1.40E-06	8.77E-06	NECTIN2
novel.893	128.7781455	4.485066883	4.827024215	1.40E-06	8.79E-06	-
ENSG00000149923	1261.694123	3028.225173	-1.263009144	1.40E-06	8.79E-06	PPP4C
ENSG00000165929	25.32286519	0	6.995374661	1.40E-06	8.80E-06	TC2N
ENSG00000188290	380.5246395	152.9280491	1.313388682	1.40E-06	8.80E-06	HES4
ENSG00000017427	21.89946649	0	6.785955663	1.41E-06	8.83E-06	IGF1
ENSG00000070526	24.06938319	0	6.922201325	1.43E-06	8.97E-06	ST6GALNAC1
ENSG00000107130	805.6131964	2666.345649	-1.72664777	1.43E-06	8.97E-06	NCS1
ENSG00000234186	22.85447958	0	6.848952886	1.44E-06	8.99E-06	C16orf82
ENSG00000159433	3231.757815	402.8687655	3.004215948	1.44E-06	9.00E-06	STARD9
novel.555	2.71348926	137.3149708	-5.660157722	1.44E-06	9.00E-06	-
ENSG00000164961	640.7602728	1185.155482	-0.887247091	1.44E-06	9.00E-06	WASHC5
ENSG00000256771	166.1042692	63.78326509	1.380270474	1.45E-06	9.06E-06	ZNF253
ENSG00000106479	718.2849689	245.6741234	1.547767504	1.45E-06	9.07E-06	ZNF862
ENSG00000213366	671.9143511	187.2061601	1.844891165	1.46E-06	9.14E-06	GSTM2
ENSG00000160789	7719.325209	19393.24448	-1.328999086	1.46E-06	9.15E-06	LMNA
ENSG00000189050	266.1909272	99.5525854	1.416719527	1.46E-06	9.15E-06	RNFT1
novel.265	141.8589472	30.16871882	2.226370417	1.47E-06	9.17E-06	-
ENSG00000174953	330.7839181	668.0684782	-1.014477629	1.47E-06	9.21E-06	DHX36
ENSG00000155897	0	56.41682713	-8.398153134	1.48E-06	9.25E-06	ADCY8
ENSG00000171847	36.20189555	1.10029862	5.064429134	1.48E-06	9.25E-06	FAM90A1
ENSG00000076248	311.5987958	779.99642	-1.324120354	1.49E-06	9.30E-06	UNG
ENSG00000102524	339.2113428	59.33033964	2.512357247	1.49E-06	9.32E-06	TNFSF13B
ENSG00000001626	22.00881936	0	6.794290475	1.51E-06	9.42E-06	CFTR
ENSG00000124942	72461.71733	24279.62386	1.577466961	1.51E-06	9.42E-06	AHNAK
ENSG00000131089	557.3473997	288.1983394	0.951661423	1.51E-06	9.43E-06	ARHGEF9
ENSG00000130988	53.89319028	1.844058227	4.890685457	1.52E-06	9.49E-06	RGN
ENSG00000163950	639.556538	1543.891682	-1.271682632	1.52E-06	9.49E-06	SLBP
ENSG00000197345	546.5368225	1049.350355	-0.941179247	1.54E-06	9.59E-06	MRPL21
ENSG00000077458	827.5324121	275.6689329	1.586104026	1.55E-06	9.63E-06	FAM76B
ENSG00000213468	44.26449422	6.24640858	2.842799786	1.55E-06	9.65E-06	FIRRE
ENSG00000158717	878.1787517	383.4364752	1.194786165	1.56E-06	9.72E-06	RNF166
ENSG00000250748	2.124500267	36.26488315	-4.092816967	1.56E-06	9.72E-06	AC025419.1
ENSG00000278126	53.11745001	9.906283597	2.412442256	1.56E-06	9.72E-06	AC139768.2
ENSG00000164144	1521.424124	715.5176118	1.088398941	1.57E-06	9.78E-06	ARFIP1
ENSG00000157224	409.6902221	1193.063713	-1.541857047	1.58E-06	9.80E-06	CLDN12
ENSG00000182575	92.00582367	10.27215083	3.171848472	1.58E-06	9.85E-06	NXPH3
ENSG00000018699	196.8004195	505.6421716	-1.362024441	1.59E-06	9.88E-06	TTC27
ENSG00000181291	50.90408252	0.387220594	7.041080257	1.59E-06	9.88E-06	TMEM132E
ENSG00000180447	85.03746508	694.9054819	-3.03090225	1.59E-06	9.90E-06	GAS1
ENSG00000214402	126.1468978	2.893606169	5.449226034	1.60E-06	9.96E-06	LCNL1

ENSG00000272927	35.98791966	0.774441189	5.632746444	1.61E-06	9.97E-06	AC107464.3
ENSG00000156990	379.7659521	778.8601746	-1.036062546	1.61E-06	1.00E-05	RPUSD3
ENSG00000101331	41.78281667	0.672939833	5.878235053	1.61E-06	1.00E-05	CCM2L
ENSG00000237489	95.37968933	22.6247411	2.068496482	1.62E-06	1.00E-05	C10orf143
ENSG00000261253	0.586481695	47.01501504	-6.287748617	1.62E-06	1.01E-05	AC137932.2
novel.1029	0	30.12272004	-7.496328762	1.63E-06	1.01E-05	-
novel.115	30.25473653	0	7.253515057	1.63E-06	1.01E-05	-
ENSG00000137872	692.0148144	41.25604414	4.07125954	1.63E-06	1.01E-05	SEMA6D
ENSG00000197406	55.97012281	0	8.139989426	1.64E-06	1.01E-05	DIO3
ENSG00000099800	792.4544692	1936.93957	-1.289540174	1.64E-06	1.02E-05	TIMM13
ENSG00000136936	376.6788025	175.5284831	1.102046531	1.64E-06	1.02E-05	XPA
ENSG00000122642	2043.500759	4930.833147	-1.270861108	1.65E-06	1.02E-05	FKBP9
ENSG00000128829	1332.802422	2624.671753	-0.977540792	1.66E-06	1.03E-05	EIF2AK4
ENSG00000133657	1432.342771	6437.361051	-2.168056864	1.66E-06	1.03E-05	ATP13A3
ENSG00000156052	2057.034189	1032.597024	0.994086266	1.67E-06	1.03E-05	GNAQ
ENSG00000180479	112.3600782	30.28540898	1.893479319	1.67E-06	1.03E-05	ZNF571
ENSG00000100138	1411.90663	2883.839248	-1.030356434	1.67E-06	1.03E-05	SNU13
ENSG00000100266	1011.180867	2032.381928	-1.007093564	1.67E-06	1.03E-05	PACSIN2
ENSG00000134339	0.308355904	614.8916006	-10.88347714	1.67E-06	1.04E-05	SAA2
ENSG00000119771	1084.484672	276.0619672	1.974794398	1.68E-06	1.04E-05	KLHL29
ENSG00000220563	40.91806699	1.049547942	5.256980157	1.69E-06	1.04E-05	PKMP3
ENSG00000178301	140.4169618	22.74990757	2.618841028	1.69E-06	1.04E-05	AQP11
ENSG00000100767	1226.636482	178.370662	2.780655672	1.70E-06	1.05E-05	PAPLN
ENSG00000111339	832.8814274	1.813376645	8.846011632	1.71E-06	1.05E-05	ART4
ENSG00000198467	4136.997201	14886.17065	-1.847321842	1.71E-06	1.05E-05	TPM2
ENSG00000136160	2926.682342	171.9877509	4.088163321	1.72E-06	1.06E-05	EDNRB
ENSG00000142961	624.5587875	274.9259912	1.184131076	1.73E-06	1.07E-05	MOB3C
ENSG00000131094	86.84547499	10.54854185	3.032153745	1.73E-06	1.07E-05	C1QL1
ENSG00000236675	5.659759143	54.01147618	-3.244857453	1.74E-06	1.07E-05	MTX1P1
ENSG00000261390	23.69917087	0	6.900131488	1.74E-06	1.08E-05	MAFTRR
ENSG00000158710	4949.623086	11894.44528	-1.264893841	1.76E-06	1.09E-05	TAGLN2
ENSG00000224592	35.33617661	0.387220594	6.514383648	1.76E-06	1.09E-05	AL139158.2
ENSG00000146263	121.6142315	373.4542345	-1.618894207	1.77E-06	1.09E-05	MMS22L
ENSG00000130649	50.51101979	7.938214774	2.67255184	1.78E-06	1.09E-05	CYP2E1
ENSG00000135999	598.1017087	275.9278705	1.115328644	1.78E-06	1.10E-05	EPC2
novel.670	604.4221337	0	11.57309835	1.78E-06	1.10E-05	-
ENSG00000126218	256.8169916	58.50728395	2.132707431	1.79E-06	1.10E-05	F10
ENSG00000148396	1420.075634	2954.202967	-1.056707107	1.79E-06	1.10E-05	SEC16A
ENSG00000112118	795.2208404	2026.462373	-1.349715957	1.79E-06	1.10E-05	MCM3
ENSG00000099821	1078.641449	1936.064196	-0.843961749	1.79E-06	1.10E-05	POLRMT
ENSG00000181722	87.39041555	12.12579406	2.846877654	1.80E-06	1.11E-05	ZBTB20
ENSG00000235944	72.83585949	16.33691006	2.150372629	1.80E-06	1.11E-05	ZNF815P
ENSG00000173175	133.9032918	7.14370449	4.226372919	1.80E-06	1.11E-05	ADCY5
ENSG00000155957	601.9108885	182.0082377	1.724389219	1.81E-06	1.11E-05	TMBIM4
ENSG00000120645	142.5037646	2.893606169	5.624942615	1.81E-06	1.11E-05	IQSEC3
ENSG00000130307	50.82919142	7.04335901	2.838738595	1.81E-06	1.11E-05	USHBP1
ENSG00000140931	2633.447628	1304.711998	1.013409501	1.81E-06	1.11E-05	CMTM3
ENSG00000234511	51.69634965	0	8.026318346	1.81E-06	1.11E-05	C5orf58
ENSG00000223519	69.74600438	2.762579107	4.633896705	1.82E-06	1.12E-05	KIF28P
ENSG00000231113	104.4495987	27.88052476	1.909580669	1.82E-06	1.12E-05	AL035587.1
ENSG00000075292	2818.547254	1390.12172	1.019817083	1.82E-06	1.12E-05	ZNF638

ENSG00000145687	859.1820334	252.8803938	1.763391572	1.84E-06	1.13E-05	SSBP2
ENSG00000269514	47.75795541	2.11916498	4.480539436	1.84E-06	1.13E-05	AC024257.3
ENSG00000286293	42.9369475	0	7.758619651	1.84E-06	1.13E-05	AP005901.5
ENSG00000144895	978.5041452	1823.093453	-0.897708579	1.84E-06	1.13E-05	EIF2A
ENSG00000128016	6548.995085	1074.643614	2.607280846	1.85E-06	1.13E-05	ZFP36
ENSG00000168763	714.6711998	255.1975532	1.485837734	1.85E-06	1.14E-05	CNNM3
ENSG00000168350	30.25383889	0.356539013	6.291106905	1.86E-06	1.14E-05	DEGS2
ENSG00000095464	37.34455756	3.251301057	3.52871057	1.86E-06	1.14E-05	PDE6C
ENSG00000105835	1023.5123	11353.12691	-3.471467813	1.86E-06	1.14E-05	NAMPT
novel.727	34.48278104	0.356539013	6.479853957	1.87E-06	1.14E-05	-
ENSG00000003147	93.04558703	7.26655921	3.66250299	1.87E-06	1.15E-05	ICA1
ENSG00000175029	671.3774863	1245.791319	-0.892055731	1.88E-06	1.15E-05	CTBP2
novel.45	34.1951677	0	7.430357242	1.88E-06	1.15E-05	-
ENSG00000055070	2045.561539	3934.7161	-0.943727851	1.89E-06	1.16E-05	SZRD1
ENSG00000178802	578.7926312	1252.766834	-1.113715277	1.89E-06	1.16E-05	MPI
ENSG00000214174	450.1285536	105.5173843	2.090417104	1.91E-06	1.17E-05	AMZ2P1
ENSG00000245750	57.95082439	1.345879667	5.372979648	1.91E-06	1.17E-05	DRAIC
ENSG00000183258	1177.810632	2774.380922	-1.23592785	1.91E-06	1.17E-05	DDX41
ENSG00000225434	101.2288218	6.337425844	3.988994157	1.91E-06	1.17E-05	LINC01504
ENSG00000185271	55.28317849	5.247611316	3.425196003	1.92E-06	1.17E-05	KLHL33
ENSG00000164040	1185.662573	2499.794093	-1.076259602	1.93E-06	1.18E-05	PGRMC2
ENSG00000196116	562.4745851	160.4156723	1.80833202	1.93E-06	1.18E-05	TDRD7
ENSG00000134049	428.0157798	998.6523144	-1.222409962	1.94E-06	1.18E-05	IER3IP1
ENSG00000184470	2499.343449	830.5298464	1.589432977	1.94E-06	1.18E-05	TXNRD2
ENSG00000257501	29.09878258	0	7.195852015	1.94E-06	1.19E-05	AC007424.1
ENSG00000169418	39.09196436	2.140389951	4.184794282	1.95E-06	1.19E-05	NPR1
ENSG00000179133	65.75083959	8.022087178	3.019649401	1.95E-06	1.19E-05	C10orf67
ENSG00000177352	362.5577972	714.4571133	-0.978560429	1.96E-06	1.19E-05	CCDC71
ENSG00000161944	38.47643493	0.356539013	6.637156761	1.97E-06	1.20E-05	ASGR2
ENSG00000127415	629.0517096	332.2374641	0.921154827	1.97E-06	1.20E-05	IDUA
ENSG00000267296	102.8073409	12.9320727	2.990704182	1.98E-06	1.20E-05	CEBPA-DT
ENSG00000221946	40.92613052	0.356539013	6.72783342	1.99E-06	1.21E-05	FXYD7
ENSG00000181847	45.48441208	0	7.840599901	1.99E-06	1.21E-05	TIGIT
ENSG00000104972	38.37460394	0	7.595270527	1.99E-06	1.21E-05	LILRB1
ENSG00000142864	3498.929805	9142.749516	-1.385763758	2.01E-06	1.23E-05	SERBP1
ENSG00000125868	2313.46881	6752.456039	-1.545406827	2.02E-06	1.23E-05	DSTN
ENSG00000087008	206.1447646	473.8695689	-1.200289361	2.05E-06	1.25E-05	ACOX3
ENSG00000171840	83.22948299	3.068367442	4.731903723	2.06E-06	1.26E-05	NINJ2
ENSG00000115091	2670.232755	5753.261139	-1.107462675	2.07E-06	1.26E-05	ACTR3
ENSG00000225756	76.5432253	10.00662908	2.933374777	2.07E-06	1.26E-05	DBH-AS1
ENSG00000138755	215.5182409	0	10.08533103	2.08E-06	1.26E-05	CXCL9
ENSG00000164949	440.2741712	127.7369251	1.787260698	2.08E-06	1.26E-05	GEM
ENSG00000170382	26.92009034	0	7.083556673	2.08E-06	1.27E-05	LRRN2
ENSG00000102393	395.6469438	790.122822	-0.997701057	2.09E-06	1.27E-05	GLA
ENSG00000188603	31.30462818	1.080229524	4.861705849	2.09E-06	1.27E-05	CLN3
ENSG00000115896	90.04082258	13.646435	2.710794248	2.10E-06	1.28E-05	PLCL1
ENSG00000159399	649.9708179	2638.347814	-2.021166759	2.10E-06	1.28E-05	HK2
ENSG00000136830	7193.468676	16805.90537	-1.224200937	2.11E-06	1.28E-05	FAM129B
ENSG00000206561	75.46199208	19.8620336	1.929797708	2.11E-06	1.28E-05	COLQ
novel.191	22.20022923	0	6.805727801	2.12E-06	1.28E-05	-
ENSG00000179454	373.6035917	186.0332908	1.00658782	2.12E-06	1.29E-05	KLHL28

ENSG00000122882	450.4304556	879.8317237	-0.966233142	2.12E-06	1.29E-05	ECD
ENSG00000118402	86.23481761	231.7749154	-1.426744592	2.13E-06	1.29E-05	ELOVL4
ENSG00000186496	72.01713881	12.28164211	2.540681189	2.14E-06	1.30E-05	ZNF396
ENSG00000111057	43.59896068	4115.926008	-6.561084035	2.14E-06	1.30E-05	KRT18
ENSG00000272335	243.4301434	87.75619761	1.470153025	2.14E-06	1.30E-05	AC093297.2
ENSG00000130595	56.42941581	0.693008929	6.308532821	2.15E-06	1.30E-05	TNNT3
ENSG00000177674	547.9076768	1525.410655	-1.47706038	2.15E-06	1.31E-05	AGTRAP
ENSG00000250451	0	18.25294394	-6.771658998	2.16E-06	1.31E-05	HOXC-AS1
ENSG00000179772	211.2344237	5.84040316	5.163972475	2.16E-06	1.31E-05	FOXS1
ENSG00000162928	361.68742	662.4576498	-0.873226126	2.17E-06	1.31E-05	PEX13
ENSG00000167196	514.2878512	1014.022298	-0.979744502	2.17E-06	1.31E-05	FBXO22
ENSG00000158987	375.4171857	145.1595869	1.370038609	2.17E-06	1.31E-05	RAPGEF6
ENSG00000086827	237.6577412	526.0119216	-1.146880598	2.18E-06	1.32E-05	ZW10
ENSG00000006695	140.9466295	298.7223642	-1.084395109	2.19E-06	1.32E-05	COX10
ENSG00000163166	736.217404	1349.143959	-0.874028443	2.21E-06	1.33E-05	IWS1
ENSG00000119689	1489.891428	2850.863598	-0.936302835	2.22E-06	1.34E-05	DLST
ENSG00000248371	1.201939854	40.17825476	-5.053109828	2.22E-06	1.34E-05	LINC02056
ENSG00000169715	1199.488123	4689.745354	-1.966998731	2.22E-06	1.34E-05	MT1E
ENSG00000113615	494.2575387	1601.283261	-1.695763075	2.23E-06	1.35E-05	SEC24A
ENSG00000111052	319.3441201	52.12655625	2.610717589	2.24E-06	1.35E-05	LIN7A
ENSG00000105220	1748.943866	4470.966167	-1.354092242	2.24E-06	1.35E-05	GPI
ENSG00000181982	830.1872371	222.6461554	1.899314178	2.24E-06	1.35E-05	CCDC149
novel.1095	67.58560271	0.743759607	6.541040987	2.24E-06	1.35E-05	-
ENSG00000049247	19.91831015	0	6.650228068	2.25E-06	1.35E-05	UTS2
ENSG00000266865	94.31314424	17.50931272	2.419129827	2.25E-06	1.36E-05	AC138207.8
ENSG00000254415	37.13351624	0.672939833	5.706710624	2.26E-06	1.36E-05	SIGLEC14
ENSG00000114770	1555.531437	717.1683335	1.117055902	2.26E-06	1.36E-05	ABCC5
ENSG00000226476	43.80082771	0	7.786271477	2.26E-06	1.36E-05	LINC01748
ENSG00000105246	154.53922	26.34199808	2.561188349	2.26E-06	1.36E-05	EBI3
ENSG00000054179	200.499023	0	9.981122113	2.27E-06	1.37E-05	ENTPD2
ENSG00000260401	55.79146325	1.080229524	5.693236634	2.30E-06	1.38E-05	AP002761.4
ENSG00000110721	861.8459502	243.1643518	1.826330361	2.30E-06	1.39E-05	CHKA
ENSG00000188315	265.1046515	85.31361535	1.634024703	2.31E-06	1.39E-05	C3orf62
ENSG00000151388	107.8806177	1838.674138	-4.091446058	2.31E-06	1.39E-05	ADAMTS12
ENSG00000137331	12.83106769	129.4652863	-3.331828148	2.31E-06	1.39E-05	IER3
ENSG00000031081	1176.519868	595.703841	0.981424458	2.33E-06	1.40E-05	ARHGAP31
ENSG00000115310	5128.743199	10516.7549	-1.036046841	2.34E-06	1.41E-05	RTN4
ENSG00000260597	0.308355904	39.51669778	-6.925060573	2.34E-06	1.41E-05	AC012531.1
ENSG00000106404	342.6671114	99.62353357	1.780662039	2.35E-06	1.41E-05	CLDN15
ENSG00000226751	24.13485801	0	6.925944069	2.36E-06	1.42E-05	AF127936.1
ENSG00000180992	466.0829971	1077.049921	-1.20850777	2.36E-06	1.42E-05	MRPL14
ENSG00000284727	38.21675821	0.672939833	5.750290886	2.36E-06	1.42E-05	AC116562.4
ENSG00000151348	1628.51461	3278.532132	-1.00949182	2.38E-06	1.43E-05	EXT2
ENSG00000166477	315.4390762	737.9892489	-1.226672893	2.38E-06	1.43E-05	LEO1
ENSG00000102452	436.346606	37.68098747	3.529794773	2.39E-06	1.44E-05	NALCN
ENSG00000128340	404.2944256	3300.179475	-3.029116595	2.41E-06	1.45E-05	RAC2
ENSG00000007202	1609.687577	2870.385304	-0.834520295	2.41E-06	1.45E-05	KIAA0100
ENSG00000004779	661.5439306	1532.400042	-1.212103111	2.45E-06	1.47E-05	NDUFAB1
ENSG00000104356	115.9559395	348.0528813	-1.586567764	2.46E-06	1.48E-05	POP1
ENSG00000124181	1323.585123	2549.5035	-0.945624034	2.46E-06	1.48E-05	PLCG1
ENSG00000263874	156.2292155	41.76526755	1.90436395	2.46E-06	1.48E-05	LINC00672

ENSG00000256940	0.308355904	29.85334548	-6.520413171	2.46E-06	1.48E-05	AP001453.2
ENSG00000102572	380.9607528	1150.699947	-1.594881419	2.47E-06	1.48E-05	STK24
ENSG00000114686	781.8269254	1778.258795	-1.18575091	2.47E-06	1.48E-05	MRPL3
ENSG00000170873	318.8512629	886.3787235	-1.475223024	2.48E-06	1.48E-05	MTSS1
ENSG00000184009	30900.71404	78259.62203	-1.340631626	2.48E-06	1.49E-05	ACTG1
ENSG00000151465	680.4103831	1479.774807	-1.121154864	2.49E-06	1.49E-05	CDC123
ENSG00000117528	1832.962759	910.0408718	1.010174104	2.49E-06	1.49E-05	ABCD3
ENSG00000238105	72.55801843	12.28981445	2.564574398	2.51E-06	1.50E-05	GOLGA2P5
ENSG00000155755	225.7338338	755.0362614	-1.742414938	2.51E-06	1.50E-05	TMEM237
ENSG00000054983	1239.35639	500.6728175	1.307428209	2.53E-06	1.51E-05	GALC
ENSG00000101337	1044.150433	2253.039689	-1.109424633	2.53E-06	1.51E-05	TM9SF4
ENSG00000106244	2587.765939	4719.853848	-0.867017169	2.53E-06	1.51E-05	PDAP1
ENSG00000164830	1575.236564	592.9578506	1.40964006	2.56E-06	1.53E-05	OXR1
ENSG00000147421	483.9786097	231.8774442	1.062435872	2.57E-06	1.53E-05	HMBOX1
ENSG00000172974	206.5721035	478.5855076	-1.212387597	2.58E-06	1.54E-05	AC007318.1
ENSG00000005073	5.534108833	122.1708708	-4.470014516	2.60E-06	1.55E-05	HOXA11
ENSG00000224982	6.606281425	106.3361892	-4.004800014	2.61E-06	1.56E-05	TMEM233
ENSG00000117602	569.1306999	161.2136374	1.821041278	2.61E-06	1.56E-05	RCAN3
ENSG00000277534	138.0373287	23.06399664	2.573793737	2.61E-06	1.56E-05	AC007996.1
ENSG00000240219	21.64780986	0	6.769078184	2.61E-06	1.56E-05	AL512306.2
ENSG00000113088	67.63979469	0.356539013	7.451387407	2.63E-06	1.57E-05	GZMK
ENSG00000272491	41.45376164	2.058957692	4.29274427	2.63E-06	1.57E-05	AL109659.2
ENSG00000268205	773.0582217	353.7033691	1.128569331	2.66E-06	1.59E-05	AC005261.2
ENSG00000261997	62.93724372	1.161661783	5.827016402	2.67E-06	1.59E-05	AC007336.1
ENSG00000135870	944.6029188	444.5296848	1.087515055	2.68E-06	1.60E-05	RC3H1
ENSG00000159147	229.1738584	524.7651031	-1.195310141	2.68E-06	1.60E-05	DONSON
ENSG00000172270	6482.298299	15215.48868	-1.230951915	2.69E-06	1.60E-05	BSG
ENSG00000160613	391.6609971	984.0508026	-1.328970703	2.69E-06	1.60E-05	PCSK7
ENSG00000197121	575.3950888	263.4032971	1.126463482	2.69E-06	1.60E-05	PGAP1
novel.186	31.11851764	0.387220594	6.331918381	2.69E-06	1.60E-05	-
ENSG00000278817	50.69594795	8.836666559	2.509596232	2.70E-06	1.60E-05	AC007325.4
ENSG00000122483	102.143789	287.5071755	-1.492767839	2.70E-06	1.61E-05	CCDC18
ENSG00000011009	698.1716419	1480.669056	-1.084505634	2.71E-06	1.61E-05	LYPLA2
ENSG00000155465	424.5340283	97.60145638	2.123128208	2.71E-06	1.61E-05	SLC7A7
ENSG00000164066	593.023547	199.7612052	1.569753586	2.72E-06	1.62E-05	INTU
ENSG00000109814	597.3503416	1676.847125	-1.489344856	2.75E-06	1.63E-05	UGDH
ENSG00000078369	5546.970858	10437.42476	-0.912000226	2.75E-06	1.63E-05	GNB1
ENSG00000197479	26.31480391	0	7.050743747	2.75E-06	1.63E-05	PCDHB11
ENSG00000134294	5040.586032	17286.07983	-1.777961499	2.75E-06	1.64E-05	SLC38A2
ENSG00000176165	0	22.10148476	-7.044441799	2.75E-06	1.64E-05	FOXG1
ENSG00000151093	114.8280366	278.6185555	-1.277669662	2.76E-06	1.64E-05	OXSM
ENSG00000164400	0.308355904	2337.391224	-12.8099698	2.77E-06	1.64E-05	CSF2
ENSG00000185955	60.43718836	13.57548683	2.15207345	2.78E-06	1.65E-05	C7orf61
ENSG00000175573	712.9279768	2142.956456	-1.587731337	2.78E-06	1.65E-05	C11orf68
ENSG00000147202	242.0760566	605.8329272	-1.322859612	2.79E-06	1.65E-05	DIAPH2
ENSG00000216490	34.01561014	0.693008929	5.574258615	2.79E-06	1.66E-05	IFI30
ENSG00000232295	47.60200372	8.234546499	2.530459069	2.79E-06	1.66E-05	AL589935.1
ENSG00000134780	261.5254437	99.55844599	1.392304305	2.80E-06	1.66E-05	DAGLA
ENSG00000156931	823.7964815	391.2666729	1.074256087	2.83E-06	1.68E-05	VPS8
ENSG00000164690	862.6283073	2.0188195	8.721038075	2.85E-06	1.69E-05	SHH
ENSG00000100297	681.3863957	2195.195651	-1.687943084	2.85E-06	1.69E-05	MCM5

ENSG00000176946	642.8741748	1188.651769	-0.886586477	2.87E-06	1.70E-05	THAP4
ENSG00000232160	75.09213577	18.11832086	2.056243552	2.88E-06	1.70E-05	RAP2C-AS1
ENSG00000268355	26.03750449	0	7.03625632	2.88E-06	1.70E-05	AC243960.3
ENSG00000163564	32.52726715	0	7.356648947	2.88E-06	1.71E-05	PYHIN1
ENSG00000179104	212.6584186	59.85200774	1.827434213	2.90E-06	1.72E-05	TMTC2
ENSG00000198015	681.1485577	1294.016563	-0.925970109	2.90E-06	1.72E-05	MRPL42
ENSG00000183722	5062.21762	1009.268327	2.326227644	2.91E-06	1.72E-05	LHFPL6
ENSG00000240184	222.5729747	958.2372541	-2.106252449	2.91E-06	1.72E-05	PCDHGC3
ENSG00000169021	882.9600263	1987.132728	-1.170392284	2.91E-06	1.72E-05	UQCRFS1
ENSG00000136122	48.0475775	152.4496829	-1.665920177	2.93E-06	1.73E-05	BORA
ENSG00000167767	4.266622888	49.26949226	-3.536222181	2.93E-06	1.73E-05	KRT80
ENSG00000095574	583.7530524	229.9291877	1.34526883	2.94E-06	1.74E-05	IKZF5
ENSG00000186350	2073.963619	1090.272661	0.927835827	2.94E-06	1.74E-05	RXRA
ENSG00000140836	898.9623966	386.3060992	1.218618361	2.96E-06	1.75E-05	ZFHX3
ENSG00000145604	206.2997474	586.7599066	-1.508592114	2.96E-06	1.75E-05	SKP2
novel.843	72.41682579	11.77028253	2.63307482	2.96E-06	1.75E-05	-
ENSG00000197635	88.44230069	1881.819795	-4.41151853	2.96E-06	1.75E-05	DPP4
ENSG00000167191	571.3028622	47.14929979	3.596198026	2.97E-06	1.75E-05	GPRC5B
ENSG00000182195	471.9289811	1092.526355	-1.210727445	2.97E-06	1.75E-05	LDOC1
ENSG00000108771	723.0022629	285.7944483	1.337914305	2.98E-06	1.76E-05	DHX58
ENSG00000151376	708.9137144	178.5201702	1.989598685	2.99E-06	1.76E-05	ME3
ENSG00000110455	823.6904015	302.3010169	1.44650831	3.00E-06	1.77E-05	ACCS
ENSG00000109758	29.9757131	1.365948763	4.401239226	3.01E-06	1.77E-05	HGFAC
ENSG00000123358	4546.410076	605.5175791	2.908389829	3.02E-06	1.78E-05	NR4A1
ENSG00000288062	22.28938118	0	6.812561031	3.03E-06	1.78E-05	AL136981.3
ENSG00000077984	79.94003978	1.447381022	5.793652601	3.03E-06	1.79E-05	CST7
ENSG00000174343	0	60.30910216	-8.494566613	3.05E-06	1.80E-05	CHRNA9
ENSG00000077044	326.1313987	2139.632205	-2.713699098	3.07E-06	1.81E-05	DGKD
ENSG00000154589	121.2665415	15.17293653	2.992708716	3.07E-06	1.81E-05	LY96
ENSG00000267731	29.53132169	0.387220594	6.256781999	3.08E-06	1.81E-05	AC005332.2
ENSG00000166483	229.9017028	775.0057382	-1.753747976	3.08E-06	1.81E-05	WEE1
ENSG00000130522	5540.473972	1440.030936	1.943955103	3.08E-06	1.81E-05	JUND
ENSG00000102100	381.2714745	786.4320554	-1.044894455	3.09E-06	1.82E-05	SLC35A2
ENSG00000119977	604.0484672	1548.001939	-1.357938349	3.09E-06	1.82E-05	TCTN3
ENSG00000279811	21.60468726	0	6.768008561	3.11E-06	1.83E-05	AC093330.2
ENSG00000087111	663.7704041	1489.224788	-1.165970112	3.11E-06	1.83E-05	PIGS
ENSG00000164929	285.5163014	24.46481813	3.551632497	3.11E-06	1.83E-05	BAALC
ENSG00000166704	256.3685274	113.6805503	1.172266548	3.12E-06	1.83E-05	ZNF606
ENSG00000111796	54.65582424	0.356539013	7.14377446	3.12E-06	1.83E-05	KLRB1
ENSG00000180867	296.3296431	739.915626	-1.3200122	3.13E-06	1.84E-05	PDIA3P1
ENSG00000138069	2308.19642	4809.190045	-1.059044468	3.14E-06	1.85E-05	RAB1A
ENSG00000166012	916.7714217	1820.152003	-0.989391776	3.15E-06	1.85E-05	TAF1D
ENSG00000273837	20.37907027	0	6.682173363	3.16E-06	1.86E-05	AC018755.4
novel.1079	70.98127859	1.487519214	5.60265478	3.17E-06	1.86E-05	-
ENSG00000231721	169.2845021	46.49805166	1.869693778	3.22E-06	1.89E-05	LINC-PINT
ENSG00000110245	0	34.43948135	-7.685230803	3.22E-06	1.89E-05	APOC3
novel.135	37.04816868	0	7.545894879	3.22E-06	1.89E-05	-
ENSG00000115295	1141.657658	542.5274801	1.073246831	3.22E-06	1.89E-05	CLIP4
ENSG00000196639	58.59410411	457.0524944	-2.964433217	3.23E-06	1.89E-05	HRH1
novel.722	82.99369723	189.5880684	-1.191522744	3.24E-06	1.90E-05	-
ENSG00000132254	780.6525815	1610.711521	-1.044691492	3.24E-06	1.90E-05	ARFIP2

ENSG00000277945	0	29.58024651	-7.465181417	3.25E-06	1.91E-05	AC107308.1
ENSG00000250786	231.432456	783.7134423	-1.759520667	3.26E-06	1.91E-05	SNHG18
ENSG00000230982	62.65076931	12.48348894	2.324293031	3.27E-06	1.92E-05	DSTNP1
ENSG00000271009	102.7565542	12.83301149	2.988693142	3.27E-06	1.92E-05	AC116667.1
ENSG00000128973	198.8888205	538.7383415	-1.438020114	3.31E-06	1.94E-05	CLN6
ENSG00000205129	14.60723749	101.0204551	-2.789977368	3.33E-06	1.95E-05	C4orf47
novel.771	33.45477089	0	7.397252016	3.34E-06	1.95E-05	-
ENSG00000214108	23.13393029	0	6.866408162	3.35E-06	1.96E-05	TPT1P5
ENSG00000101199	1930.601347	4166.751015	-1.109770795	3.35E-06	1.96E-05	ARFGAP1
ENSG00000122481	114.7361638	39.34758749	1.542921509	3.37E-06	1.97E-05	RWDD3
ENSG00000203485	8942.084528	3530.112123	1.340894101	3.37E-06	1.97E-05	INF2
ENSG00000139880	75.15191181	330.5044922	-2.137779925	3.38E-06	1.97E-05	CDH24
ENSG00000007047	464.9108597	1302.096765	-1.485676617	3.39E-06	1.98E-05	MARK4
ENSG00000181104	446.6704334	3791.027349	-3.085408491	3.40E-06	1.98E-05	F2R
ENSG00000138193	725.798138	149.4684455	2.278093566	3.41E-06	1.99E-05	PLCE1
ENSG00000140464	967.9303505	1911.273439	-0.981667461	3.42E-06	1.99E-05	PML
ENSG00000133119	149.393274	519.027708	-1.796743794	3.42E-06	2.00E-05	RFC3
ENSG00000198947	5894.018318	385.302203	3.934848536	3.43E-06	2.00E-05	DMD
ENSG00000147601	1077.361186	461.3933188	1.223323755	3.44E-06	2.01E-05	TERF1
ENSG00000182397	37.85295668	2.864080462	3.723452963	3.44E-06	2.01E-05	DNM1P46
ENSG00000171155	401.7124009	867.1400481	-1.110249339	3.44E-06	2.01E-05	C1GALT1C1
ENSG00000151458	2225.380182	660.5908721	1.752007016	3.44E-06	2.01E-05	ANKRD50
ENSG00000233966	16.13139465	84.14609297	-2.386671216	3.45E-06	2.01E-05	UBE2SP1
ENSG00000120705	1972.58694	3701.961783	-0.908266842	3.45E-06	2.01E-05	ETF1
ENSG00000153827	2410.759658	4160.091805	-0.787165002	3.47E-06	2.02E-05	TRIP12
ENSG00000247077	335.2986501	836.8441335	-1.319922604	3.47E-06	2.02E-05	PGAM5
ENSG00000178162	62.3227823	8.947624524	2.79869596	3.47E-06	2.02E-05	FAR2P2
ENSG00000129038	243.9958381	2797.477561	-3.519373484	3.48E-06	2.03E-05	LOXL1
ENSG00000136478	1361.069089	581.3034191	1.226946464	3.50E-06	2.03E-05	TEX2
ENSG00000090013	949.6397532	526.0577451	0.852418451	3.51E-06	2.04E-05	BLVRB
ENSG00000126067	1435.437683	3186.928869	-1.15079592	3.51E-06	2.04E-05	PSMB2
ENSG00000174939	96.22019275	930.0584811	-3.272698526	3.52E-06	2.05E-05	ASPHD1
ENSG00000128284	577.6697978	199.2857927	1.534471325	3.52E-06	2.05E-05	APOL3
ENSG00000109919	130.743118	745.8048526	-2.512594299	3.54E-06	2.06E-05	MTCH2
ENSG00000095261	481.4322125	989.7182423	-1.039851985	3.57E-06	2.07E-05	PSMD5
ENSG00000153283	80.30753133	3.036401591	4.755916032	3.59E-06	2.08E-05	CD96
ENSG00000259070	30.94404595	0	7.284711548	3.59E-06	2.09E-05	LINC00639
novel.1033	35.6405581	1.345879667	4.662014364	3.59E-06	2.09E-05	-
ENSG00000139192	464.5057305	179.0288328	1.375719279	3.59E-06	2.09E-05	TAPBPL
ENSG00000246263	50.33214641	10.42453125	2.27213203	3.60E-06	2.09E-05	UBR5-AS1
ENSG00000109906	10.75721192	197.1724879	-4.199289136	3.60E-06	2.09E-05	ZBTB16
ENSG00000187984	47.30625557	5.983198582	2.970407238	3.60E-06	2.09E-05	ANKRD19P
ENSG00000070961	647.1379608	2526.103985	-1.964720108	3.60E-06	2.09E-05	ATP2B1
ENSG00000164068	382.9834663	785.4694835	-1.036005157	3.62E-06	2.10E-05	RNF123
ENSG00000141522	2473.464294	5030.255936	-1.02411126	3.63E-06	2.10E-05	ARHGDIA
ENSG00000212802	715.1010846	1596.806674	-1.159065864	3.64E-06	2.11E-05	RPL15P3
ENSG00000130303	2640.195599	358.6319639	2.880191294	3.65E-06	2.12E-05	BST2
ENSG00000082258	1495.972507	529.1371914	1.499451823	3.65E-06	2.12E-05	CCNT2
ENSG00000205611	34.55674667	1.396630344	4.602180505	3.66E-06	2.12E-05	LINC01597
ENSG00000180481	74.96293798	10.55799846	2.817867432	3.66E-06	2.12E-05	GLIPR1L2
ENSG00000075399	619.2317466	281.6182862	1.136313789	3.67E-06	2.12E-05	VPS9D1

ENSG00000185305	324.3258808	139.7782516	1.214308208	3.67E-06	2.12E-05	ARL15
ENSG00000070423	694.9542418	1384.765861	-0.994497772	3.67E-06	2.13E-05	AC004156.1
ENSG00000153066	539.5318939	998.8531203	-0.88839648	3.69E-06	2.14E-05	TXNDC11
ENSG00000248445	33.57401076	1.885352294	4.202932757	3.70E-06	2.14E-05	SEMA6A-AS1
ENSG00000138780	126.0750563	335.3529519	-1.412363796	3.70E-06	2.14E-05	GSTCD
ENSG00000166535	27.85790835	0	7.13305577	3.70E-06	2.14E-05	A2ML1
ENSG00000100335	556.2462232	1036.156682	-0.897224137	3.73E-06	2.15E-05	MIEF1
ENSG00000258791	0.308355904	34.09483909	-6.708105981	3.73E-06	2.15E-05	LINC00520
ENSG00000100276	34.54413891	1.396630344	4.601720026	3.73E-06	2.15E-05	RASL10A
ENSG00000115137	229.4758752	76.54751177	1.58457792	3.73E-06	2.16E-05	DNAJC27
ENSG00000259891	90.22901348	19.72052245	2.185941995	3.75E-06	2.17E-05	AC107375.1
ENSG00000164850	280.952523	23.93176685	3.54836372	3.77E-06	2.18E-05	GPER1
ENSG00000285278	46.27502646	0.672939833	6.03252228	3.78E-06	2.18E-05	AL138885.3
ENSG00000228716	320.2535742	998.4186416	-1.640791244	3.80E-06	2.19E-05	DHFR
ENSG00000233954	73.93435871	174.5922049	-1.23937192	3.81E-06	2.20E-05	UQCRHL
ENSG00000119938	62.430197	461.2047599	-2.886180741	3.82E-06	2.20E-05	PPP1R3C
ENSG00000091129	1556.903189	375.7999136	2.05132412	3.83E-06	2.21E-05	NRCAM
ENSG00000169223	1860.205487	4266.172509	-1.197533834	3.83E-06	2.21E-05	LMAN2
novel.238	33.92338142	0	7.418707131	3.84E-06	2.21E-05	-
ENSG00000170734	261.5867581	747.8970389	-1.515953393	3.84E-06	2.22E-05	POLH
ENSG00000166788	165.170426	379.2841539	-1.199699591	3.85E-06	2.22E-05	SAAL1
ENSG00000251364	100.303042	4.088389678	4.593607525	3.87E-06	2.23E-05	AC107884.1
ENSG00000276975	116.0187374	3.801514564	4.915334251	3.87E-06	2.23E-05	HYDIN2
ENSG00000107186	1470.423649	589.1146096	1.319854121	3.88E-06	2.24E-05	MPDZ
novel.168	11.1535374	78.00276117	-2.80222203	3.90E-06	2.24E-05	-
ENSG00000163291	354.6662931	791.1073507	-1.157252765	3.90E-06	2.24E-05	PAQR3
ENSG00000131459	348.4036662	5221.538933	-3.905721041	3.90E-06	2.24E-05	GFPT2
ENSG00000102743	171.6579382	511.5444986	-1.575495379	3.90E-06	2.25E-05	SLC25A15
ENSG00000270689	52.99058949	1.742556871	4.90330536	3.91E-06	2.25E-05	BUD13P1
ENSG00000135094	61.87978638	3.618452555	4.101251798	3.91E-06	2.25E-05	SDS
ENSG00000286796	21.54458304	0	6.762758658	3.91E-06	2.25E-05	AC010883.2
ENSG00000213463	1186.018541	573.7887103	1.047161524	3.93E-06	2.26E-05	SYNJ2BP
ENSG00000261884	57.4908472	14.73599274	1.965078983	3.94E-06	2.26E-05	AC040162.1
ENSG00000149403	25.930944	0	7.02979965	3.95E-06	2.27E-05	GRIK4
ENSG00000141569	448.6707536	980.8643195	-1.128482107	3.95E-06	2.27E-05	TRIM65
ENSG00000145247	83.5292059	468.2687238	-2.487657227	3.96E-06	2.27E-05	OCIAD2
ENSG00000115816	377.7614315	769.7228096	-1.027056051	3.96E-06	2.27E-05	CEBPZ
ENSG00000111726	568.8876885	1237.691254	-1.121583205	3.99E-06	2.29E-05	CMAS
ENSG00000204832	12.29530177	131.9852724	-3.427913128	3.99E-06	2.29E-05	ST8SIA6-AS1
ENSG00000168672	71.49566157	14.33815966	2.316692824	4.00E-06	2.29E-05	LRATD2
novel.471	38.56300831	1.029478846	5.179916134	4.00E-06	2.29E-05	-
ENSG00000172780	27.00046675	114.1993931	-2.079473423	4.03E-06	2.31E-05	RAB43
ENSG00000196656	535.4838465	1358.492228	-1.34336061	4.04E-06	2.32E-05	AC004057.1
ENSG00000037241	360.1022606	849.7816344	-1.239140575	4.05E-06	2.32E-05	RPL26L1
ENSG00000275835	57.43178317	172.7248667	-1.588343406	4.07E-06	2.33E-05	TUBGCP5
ENSG00000254995	39.41522185	5.656185276	2.788562612	4.07E-06	2.34E-05	STX16-NPEPL1
ENSG00000170515	1364.257188	3111.185946	-1.189446227	4.09E-06	2.34E-05	PA2G4
ENSG00000187642	2.653029035	51.96300258	-4.274261439	4.10E-06	2.35E-05	PERM1
ENSG00000133138	129.3583304	670.3401611	-2.373653289	4.10E-06	2.35E-05	TBC1D8B
novel.116	182.4666927	33.98315762	2.419732309	4.15E-06	2.38E-05	-
ENSG00000112578	240.842989	725.7135743	-1.591657518	4.15E-06	2.38E-05	BYSL

ENSG00000157107	727.7489485	351.4597608	1.050129707	4.16E-06	2.38E-05	FCHO2
ENSG00000132780	1480.189056	3405.86255	-1.202161499	4.16E-06	2.38E-05	NASP
ENSG00000115112	30.08374105	0.387220594	6.283622987	4.16E-06	2.38E-05	TFCP2L1
novel.671	123.9632503	1.00940975	6.894796087	4.17E-06	2.39E-05	-
ENSG00000101346	1311.222009	2809.066222	-1.099303094	4.20E-06	2.40E-05	POFUT1
ENSG00000178814	419.733522	128.6803138	1.703590583	4.20E-06	2.40E-05	OPLAH
ENSG00000147883	518.2438392	144.0007976	1.845815354	4.21E-06	2.41E-05	CDKN2B
ENSG00000165023	93.65906461	2.0188195	5.497547137	4.21E-06	2.41E-05	DIRAS2
ENSG00000142227	1425.680232	3105.562836	-1.123286786	4.21E-06	2.41E-05	EMP3
ENSG00000172724	51.66574792	0	8.024546054	4.22E-06	2.41E-05	CCL19
ENSG00000136938	2019.104274	4204.310859	-1.058244587	4.22E-06	2.41E-05	ANP32B
ENSG00000174652	792.2619075	351.1722437	1.174517563	4.22E-06	2.41E-05	ZNF266
ENSG00000136908	534.6240832	1061.045357	-0.988962802	4.22E-06	2.41E-05	DPM2
ENSG00000233030	34.59073773	0.713078025	5.59238564	4.23E-06	2.41E-05	AC243772.2
ENSG00000197816	79.93102294	12.60737115	2.666659638	4.24E-06	2.42E-05	CCDC180
ENSG00000121691	2222.366298	1078.318708	1.043438288	4.26E-06	2.43E-05	CAT
ENSG00000237975	178.1170157	15.8148096	3.504226583	4.30E-06	2.45E-05	FLG-AS1
ENSG00000198625	1659.401767	560.1731669	1.566825183	4.32E-06	2.46E-05	MDM4
ENSG00000075643	24.76285266	170.2064216	-2.783692358	4.34E-06	2.47E-05	MOCOS
ENSG00000163126	39.74976219	3.678659843	3.453847244	4.34E-06	2.47E-05	ANKRD23
ENSG00000076944	236.0621966	81.71523189	1.527942627	4.34E-06	2.47E-05	STXBP2
ENSG00000163568	38.70810444	2.099095884	4.181224308	4.36E-06	2.48E-05	AIM2
ENSG00000126243	214.3430431	459.2422695	-1.09861536	4.36E-06	2.49E-05	LRFN3
ENSG00000110435	358.0481079	720.5014523	-1.008815467	4.38E-06	2.49E-05	PDHX
ENSG00000237440	243.9408085	90.76089014	1.424612671	4.40E-06	2.50E-05	AC008554.1
ENSG00000198513	423.5903578	65.63965244	2.686954597	4.40E-06	2.51E-05	ATL1
ENSG00000176697	124.7784952	710.7048334	-2.509543362	4.40E-06	2.51E-05	BDNF
ENSG00000254854	22.46044793	0	6.824084267	4.41E-06	2.51E-05	AP003390.1
ENSG00000287720	19.57213097	0	6.625157833	4.41E-06	2.51E-05	AC023480.1
ENSG00000167550	225.8781195	52.41055886	2.109256954	4.41E-06	2.51E-05	RHEBL1
ENSG00000039560	3273.448861	5823.616909	-0.831074938	4.41E-06	2.51E-05	RAI14
ENSG00000104763	3777.445286	1115.885437	1.759029052	4.41E-06	2.51E-05	ASAH1
ENSG00000103489	6.363400341	48.01432588	-2.916468914	4.47E-06	2.54E-05	XYLT1
ENSG00000120658	96.04661871	26.04592314	1.885932793	4.48E-06	2.54E-05	ENOX1
ENSG00000183354	717.9012633	274.4981528	1.387978141	4.48E-06	2.55E-05	KIAA2026
ENSG00000239405	32.00583335	0.387220594	6.372482371	4.49E-06	2.55E-05	TMED10P2
ENSG00000004846	29.61155556	0	7.222489788	4.49E-06	2.55E-05	ABCB5
ENSG00000258289	922.9274978	457.3227677	1.012801952	4.50E-06	2.55E-05	CHURC1
ENSG00000225398	21.99774999	0	6.792140296	4.51E-06	2.56E-05	PGM5P4
ENSG00000203865	62.31880754	17.51864094	1.828506244	4.55E-06	2.58E-05	ATP1A1-AS1
ENSG00000181195	677.9482878	25.45125191	4.732010716	4.57E-06	2.59E-05	PENK
ENSG00000226491	18.8121465	0	6.567037149	4.58E-06	2.60E-05	FTOP1
ENSG00000116141	254.5417135	58.17338257	2.126660412	4.59E-06	2.60E-05	MARK1
ENSG00000164039	1017.811253	424.2658497	1.261774092	4.63E-06	2.62E-05	BDH2
ENSG00000183798	278.2154658	22.64725034	3.61424832	4.63E-06	2.62E-05	EMILIN3
ENSG00000108797	447.5875425	1745.651677	-1.963256279	4.65E-06	2.63E-05	CNTNAP1
ENSG00000249992	115.1455518	1442.876319	-3.647602881	4.65E-06	2.63E-05	TMEM158
ENSG00000141736	4712.753553	1538.321809	1.615118095	4.66E-06	2.64E-05	ERBB2
ENSG00000110717	990.8993249	2189.761842	-1.143939894	4.67E-06	2.65E-05	NDUFS8
ENSG00000182667	4039.354732	807.8849852	2.3221877	4.68E-06	2.65E-05	NTM
ENSG00000259004	24.80067593	0	6.965199094	4.69E-06	2.66E-05	LINC02285

ENSG00000233270	42.8046295	117.0438368	-1.452180486	4.70E-06	2.66E-05	SNRPEP4
ENSG00000189221	162.7119552	494.5754161	-1.604347082	4.70E-06	2.66E-05	MAOA
ENSG00000268388	3.675126598	102.1684222	-4.800438025	4.72E-06	2.67E-05	FENDRR
ENSG00000235478	18.99073514	0	6.581544245	4.73E-06	2.67E-05	LINC01664
ENSG00000163466	3325.010789	7451.671541	-1.164241975	4.73E-06	2.68E-05	ARPC2
ENSG00000255545	19.54949403	0	6.623623614	4.74E-06	2.68E-05	AP004608.1
ENSG00000172458	150.7662094	34.5297751	2.121760075	4.76E-06	2.69E-05	IL17D
ENSG00000234719	44.8897681	3.801514564	3.534497383	4.76E-06	2.69E-05	NPIPB2
ENSG00000111790	1129.386175	626.85479	0.849354734	4.77E-06	2.69E-05	FGFR1OP2
ENSG00000188064	0	276.1954219	-10.69058206	4.77E-06	2.69E-05	WNT7B
ENSG00000112218	7.882542911	61.27371586	-2.961730117	4.77E-06	2.69E-05	GPR63
ENSG00000168256	611.3277046	1271.450197	-1.056647107	4.77E-06	2.70E-05	NKIRAS2
ENSG00000204291	3752.035522	378.1768981	3.310101892	4.81E-06	2.71E-05	COL15A1
novel.266	21.04112724	0	6.728122221	4.82E-06	2.72E-05	-
ENSG00000105926	933.8133425	277.3691342	1.751213913	4.83E-06	2.73E-05	MPP6
ENSG00000005421	19.06004218	0	6.586316526	4.86E-06	2.74E-05	PON1
ENSG00000116830	235.0167504	572.99155	-1.28617204	4.86E-06	2.74E-05	TTF2
ENSG00000254369	0	27.15007457	-7.346122885	4.87E-06	2.75E-05	HOXA-AS3
ENSG00000204054	994.9869349	401.3597089	1.30953531	4.93E-06	2.78E-05	LINC00963
ENSG00000159374	42.53527925	6.185045417	2.791033828	4.95E-06	2.79E-05	M1AP
ENSG00000141854	94.17190851	11.85055891	3.001746157	4.95E-06	2.79E-05	MISP3
ENSG00000135486	11916.27361	23276.30132	-0.965944274	4.96E-06	2.79E-05	HNRNPA1
ENSG00000242779	123.6190216	33.65126402	1.875777117	4.96E-06	2.79E-05	ZNF702P
ENSG00000166342	5.853747575	251.0402824	-5.426207503	5.03E-06	2.83E-05	NETO1
ENSG00000205959	67.99862507	8.083450341	3.062421154	5.03E-06	2.83E-05	AC105345.1
ENSG00000111644	58.40978886	8.661905285	2.756082954	5.05E-06	2.84E-05	ACRBP
ENSG00000105281	905.2874153	3197.439486	-1.820595605	5.06E-06	2.85E-05	SLC1A5
novel.595	18.47348922	0	6.541084023	5.08E-06	2.86E-05	-
ENSG00000125257	208.8393782	462.145028	-1.145574948	5.09E-06	2.86E-05	ABCC4
ENSG00000198189	889.1051352	292.8641694	1.601097039	5.10E-06	2.87E-05	HSD17B11
ENSG00000152128	20.78800351	0	6.711946848	5.11E-06	2.88E-05	TMEM163
ENSG00000184949	26.21114981	93.33642604	-1.829088247	5.13E-06	2.88E-05	FAM227A
ENSG00000112339	504.1644752	1011.17455	-1.003831428	5.13E-06	2.88E-05	HBS1L
ENSG00000162520	70.17287219	275.3930558	-1.973105737	5.13E-06	2.88E-05	SYNC
ENSG00000211751	53.77875153	0.356539013	7.120444126	5.14E-06	2.89E-05	TRBC1
ENSG00000186187	181.7790916	423.2608879	-1.220149453	5.15E-06	2.90E-05	ZNRF1
ENSG00000165156	1195.31221	413.989589	1.529055917	5.22E-06	2.93E-05	ZHX1
ENSG00000197548	335.1683134	1106.872871	-1.723240857	5.27E-06	2.96E-05	ATG7
ENSG00000116983	58.7288147	0.336469917	7.247533821	5.27E-06	2.96E-05	HPCAL4
ENSG00000269335	120.296884	272.0946469	-1.178497228	5.28E-06	2.96E-05	IKBKG
ENSG00000113758	1785.985402	7989.048093	-2.161334486	5.28E-06	2.97E-05	DBN1
ENSG00000187193	40.59062127	153.5618314	-1.918143159	5.29E-06	2.97E-05	MT1X
ENSG00000250120	29.57193699	0.356539013	6.257302776	5.31E-06	2.98E-05	PCDHA10
ENSG00000117598	39.78586145	0	7.64758887	5.32E-06	2.98E-05	PLPPR5
ENSG00000086570	29.95175123	2.884149558	3.38100473	5.33E-06	2.99E-05	FAT2
ENSG00000136010	408.0432491	1176.40347	-1.527817574	5.34E-06	2.99E-05	ALDH1L2
ENSG00000100379	348.0831197	769.2125119	-1.143598423	5.34E-06	2.99E-05	KCTD17
ENSG00000174574	1004.835275	1811.642153	-0.850483504	5.36E-06	3.00E-05	AKIRIN1
ENSG00000250397	21.09628814	84.34665554	-1.998763183	5.38E-06	3.01E-05	AP006623.1
ENSG00000110013	310.5760846	894.0583525	-1.525421738	5.38E-06	3.01E-05	SIAE
ENSG00000146678	0	23.90224114	-7.163138783	5.40E-06	3.02E-05	IGFBP1

ENSG00000205436	31.71774929	0.356539013	6.358461489	5.40E-06	3.02E-05	EXOC3L4
ENSG00000162572	268.5272239	90.33874997	1.574643697	5.44E-06	3.04E-05	SCNN1D
ENSG00000185189	4435.62909	1046.842287	2.083125259	5.44E-06	3.05E-05	NRBP2
ENSG00000183379	19.24239177	0	6.600819833	5.45E-06	3.05E-05	SYNDIG1L
ENSG00000148057	189.3403152	33.74831745	2.481807256	5.46E-06	3.05E-05	IDNK
ENSG00000088832	3775.820069	8604.048152	-1.188183472	5.47E-06	3.06E-05	FKBP1A
ENSG00000230333	34.30627242	0.713078025	5.580440736	5.49E-06	3.07E-05	AC004160.1
ENSG00000274561	47.85999986	7.898076582	2.600382183	5.49E-06	3.07E-05	AC005332.3
ENSG00000131409	128.3145221	6.011440019	4.431015272	5.49E-06	3.07E-05	LRRC4B
ENSG00000188707	114.0732805	12.04911369	3.229687118	5.52E-06	3.09E-05	ZBED6CL
novel.690	3.060922088	111.7290958	-5.194991225	5.53E-06	3.09E-05	-
ENSG00000174514	65.79987444	8.030387913	3.020291378	5.53E-06	3.09E-05	MFSD4A
ENSG00000140577	925.0539234	459.0682786	1.011095896	5.53E-06	3.09E-05	CRTC3
ENSG00000132382	869.2491682	1973.038073	-1.182603715	5.54E-06	3.09E-05	MYBBP1A
ENSG00000086289	1710.044556	760.9180964	1.168635436	5.55E-06	3.10E-05	EPDR1
ENSG00000213199	110.0977193	13.80099878	2.991933492	5.58E-06	3.12E-05	ASIC3
ENSG00000167554	78.06494609	25.55835707	1.612728442	5.59E-06	3.12E-05	ZNF610
ENSG00000173531	289.0314031	130.1906806	1.151285379	5.61E-06	3.13E-05	MST1
ENSG00000100994	1537.939759	4963.893232	-1.69054209	5.61E-06	3.13E-05	PYGB
ENSG00000040633	648.0576589	1334.353445	-1.041835214	5.61E-06	3.13E-05	PHF23
ENSG00000123610	469.181157	126.2410236	1.892637044	5.62E-06	3.13E-05	TNFAIP6
ENSG00000100336	112.0276447	1.00940975	6.747891321	5.65E-06	3.15E-05	APOL4
ENSG00000235513	95.16715307	18.52561055	2.366171927	5.68E-06	3.17E-05	AL035681.1
ENSG00000268043	430.3039834	165.884382	1.375717969	5.68E-06	3.17E-05	NBPF12
ENSG00000146070	46.6926918	1.548882377	4.978616534	5.69E-06	3.17E-05	PLA2G7
ENSG00000101935	129.2238054	345.8212985	-1.420824205	5.72E-06	3.19E-05	AMMECR1
ENSG00000164975	733.2938423	1509.686733	-1.041612683	5.73E-06	3.19E-05	SNAPC3
ENSG00000278068	21.5845576	0	6.766618596	5.73E-06	3.19E-05	AL136317.3
ENSG00000063244	2447.219593	5126.534992	-1.066909028	5.76E-06	3.21E-05	U2AF2
ENSG00000067365	227.2353978	554.4930597	-1.286381589	5.78E-06	3.22E-05	METTL22
ENSG00000076770	302.3262632	53.47470049	2.496397746	5.80E-06	3.23E-05	MBNL3
novel.593	33.1790102	2.864080462	3.531495206	5.81E-06	3.23E-05	-
ENSG00000019485	496.7288897	108.5597749	2.192245625	5.87E-06	3.26E-05	PRDM11
ENSG00000182827	731.3770341	1744.899222	-1.254495482	5.88E-06	3.27E-05	ACBD3
ENSG00000119705	441.9909338	1100.803411	-1.316695281	5.90E-06	3.28E-05	SLIRP
ENSG00000141378	294.9014482	571.5913239	-0.954971347	5.94E-06	3.30E-05	PTRH2
ENSG00000171421	298.7362439	647.4907984	-1.116152789	5.95E-06	3.30E-05	MRPL36
ENSG00000106789	26.32852277	175.1920132	-2.736130127	5.96E-06	3.31E-05	CORO2A
ENSG00000187134	157.0525517	557.4825637	-1.827996377	5.96E-06	3.31E-05	AKR1C1
ENSG00000279713	5.104761209	43.53503838	-3.086881806	6.00E-06	3.33E-05	AC080038.4
ENSG00000250673	18.36392288	0	6.533302971	6.00E-06	3.33E-05	REELD1
ENSG00000131015	11.94403672	133.6454511	-3.486080371	6.00E-06	3.33E-05	ULBP2
ENSG00000141068	1043.420726	239.4348254	2.12282203	6.00E-06	3.33E-05	KSR1
ENSG00000196189	113.4489039	25.30319102	2.171864087	6.03E-06	3.34E-05	SEMA4A
ENSG00000183828	306.0572081	127.4550586	1.265830466	6.11E-06	3.39E-05	NUDT14
ENSG00000217236	51.40753796	0	8.01731464	6.18E-06	3.43E-05	SP9
ENSG00000138382	390.1150141	780.6097136	-1.001052985	6.20E-06	3.44E-05	METTL5
ENSG00000241170	61.66022678	10.56861094	2.535079051	6.20E-06	3.44E-05	AP001992.1
ENSG00000084693	369.616277	660.037016	-0.836335331	6.20E-06	3.44E-05	AGBL5
ENSG00000105996	11.00643252	60.02420047	-2.451719289	6.23E-06	3.45E-05	HOXA2
ENSG00000173171	185.5122146	458.1926744	-1.303581927	6.23E-06	3.45E-05	MTX1

ENSG00000068912	902.0966116	2218.213792	-1.298079636	6.24E-06	3.45E-05	ERLEC1
ENSG00000197147	609.9068324	90.11919265	2.756661788	6.25E-06	3.46E-05	LRRC8B
ENSG00000109971	6728.847089	28268.65129	-2.070788171	6.26E-06	3.46E-05	HSPA8
ENSG00000164867	56.70682959	10.69963801	2.401748195	6.28E-06	3.48E-05	NOS3
ENSG00000107185	483.6486567	958.1073954	-0.986073767	6.32E-06	3.50E-05	RGP1
ENSG00000151470	220.9264029	92.34148158	1.258092176	6.33E-06	3.50E-05	C4orf33
ENSG00000172575	59.47096335	4.319762219	3.783838652	6.35E-06	3.51E-05	RASGRP1
ENSG00000087303	1348.8478	7863.364649	-2.543435053	6.36E-06	3.52E-05	NID2
ENSG00000108854	657.2874951	1630.249807	-1.310303895	6.38E-06	3.53E-05	SMURF2
ENSG00000186792	35.37274624	133.1468058	-1.913935832	6.39E-06	3.53E-05	HYAL3
ENSG00000233593	89.60513544	12.52234287	2.848646997	6.44E-06	3.56E-05	LINC02609
ENSG00000196660	28.83333582	0	7.182715789	6.45E-06	3.56E-05	SLC30A10
novel.1085	62.92449341	5.421216714	3.541496843	6.45E-06	3.56E-05	-
ENSG00000287024	36.4446341	1.345879667	4.697254404	6.48E-06	3.58E-05	AL022162.1
ENSG00000139496	789.813039	1673.761159	-1.083274588	6.49E-06	3.58E-05	NUP58
novel.182	46.66271859	1.345879667	5.060032252	6.54E-06	3.61E-05	-
ENSG00000213316	18.89399003	0	6.57279462	6.55E-06	3.61E-05	LTC4S
ENSG00000137871	620.8344846	327.7630097	0.921862491	6.56E-06	3.62E-05	ZNF280D
ENSG00000280433	192.2282327	62.51655311	1.6177185	6.59E-06	3.63E-05	FP565260.6
ENSG00000274021	104.8647723	9.834179554	3.420273456	6.59E-06	3.64E-05	AC024909.1
ENSG00000118596	46.93195413	343.7785099	-2.874118626	6.67E-06	3.68E-05	SLC16A7
ENSG00000067596	665.0889058	1177.19275	-0.823818017	6.67E-06	3.68E-05	DHX8
ENSG00000225140	37.84403859	1.00940975	5.163096098	6.71E-06	3.70E-05	AL358216.1
ENSG00000132963	1382.191518	2978.960962	-1.107971723	6.72E-06	3.70E-05	POMP
ENSG00000198246	205.7716455	70.55138895	1.544722496	6.76E-06	3.73E-05	SLC29A3
ENSG00000256069	31.69378742	0.387220594	6.357483711	6.77E-06	3.73E-05	A2MP1
ENSG00000130956	885.3203724	439.3160131	1.010913676	6.79E-06	3.74E-05	HABP4
ENSG00000135900	319.3964889	631.0025354	-0.982175516	6.83E-06	3.76E-05	MRPL44
ENSG00000237978	30.04752743	0.672939833	5.40090302	6.84E-06	3.76E-05	KCNMB2-AS1
ENSG00000188706	487.1697104	1544.436485	-1.66465194	6.85E-06	3.77E-05	ZDHHC9
ENSG00000163508	47.38888203	0	7.899852432	6.87E-06	3.78E-05	EOMES
ENSG00000251209	52.16333491	1.762625967	4.872787321	6.87E-06	3.78E-05	LINC00923
ENSG00000162976	680.9526301	217.0669201	1.648253161	6.90E-06	3.80E-05	PQLC3
ENSG00000134107	4591.473812	1174.399207	1.96719753	6.91E-06	3.80E-05	BHLHE40
ENSG00000223485	9.77927715	96.68982357	-3.306994356	6.91E-06	3.80E-05	LINC01615
ENSG00000134909	576.0020724	175.2483205	1.717827048	6.94E-06	3.82E-05	ARHGAP32
ENSG00000182601	30.16490075	0	7.248234302	6.97E-06	3.83E-05	HS3ST4
ENSG00000100823	1887.927278	3862.432821	-1.032793685	6.99E-06	3.84E-05	APEX1
ENSG00000189306	466.0288489	1986.858237	-2.092112045	7.00E-06	3.85E-05	RRP7A
ENSG00000184110	473.9361359	1053.533196	-1.152702758	7.02E-06	3.85E-05	EIF3C
ENSG00000116898	684.4672343	1389.504612	-1.02161624	7.02E-06	3.85E-05	MRPS15
ENSG00000008083	291.1171821	2391.805296	-3.038363977	7.03E-06	3.86E-05	JARID2
ENSG00000287077	42.92401156	8.661905285	2.313487736	7.06E-06	3.87E-05	AL157893.2
ENSG00000079462	115.2612164	287.9647495	-1.321235505	7.06E-06	3.87E-05	PAFAH1B3
ENSG00000174171	49.01737747	2.079026788	4.531688636	7.06E-06	3.87E-05	AC020659.1
ENSG00000187609	599.5724171	173.3463667	1.791362725	7.07E-06	3.88E-05	EXD3
ENSG00000070182	357.0114488	23.67976445	3.919462287	7.09E-06	3.89E-05	SPTB
ENSG00000198055	497.6238101	1091.946629	-1.13382827	7.09E-06	3.89E-05	GRK6
ENSG00000136261	520.6716228	1271.92617	-1.288832149	7.09E-06	3.89E-05	BZW2
novel.729	20.75010896	0	6.709452216	7.10E-06	3.89E-05	-
ENSG00000224546	26.3963627	82.70658032	-1.647290545	7.12E-06	3.90E-05	EIF4BP3

ENSG00000128482	106.4742771	27.25016326	1.966427546	7.12E-06	3.90E-05	RNF112
ENSG00000149115	1728.820901	4084.83377	-1.240410947	7.12E-06	3.90E-05	TNKS1BP1
ENSG00000132535	410.1045966	1450.936527	-1.82280629	7.13E-06	3.90E-05	DLG4
ENSG00000233098	61.40064846	1.742556871	5.116873744	7.15E-06	3.91E-05	CCDC144NL-AS1
ENSG00000280798	682.4900687	230.708124	1.565677017	7.15E-06	3.91E-05	LINC00294
ENSG00000197253	18.36141558	0	6.533089666	7.16E-06	3.92E-05	TPSB2
novel.882	8.175783758	44.55865663	-2.447723442	7.20E-06	3.94E-05	-
ENSG00000134250	1870.878676	4365.268698	-1.222392117	7.21E-06	3.94E-05	NOTCH2
ENSG00000173338	56.7227001	7.094109687	2.993675102	7.25E-06	3.96E-05	KCNK7
ENSG00000288018	88.1212101	10.82352021	3.028244373	7.31E-06	4.00E-05	AP003120.1
ENSG00000186827	40.1952647	2.038888596	4.255730853	7.35E-06	4.02E-05	TNFRSF4
ENSG00000188681	41.55358318	0.336469917	6.74840479	7.36E-06	4.02E-05	TEKT4P2
ENSG00000165238	64.41400317	1.345879667	5.531208826	7.37E-06	4.03E-05	WNK2
ENSG00000066084	1792.340143	668.5181579	1.42297405	7.39E-06	4.03E-05	DIP2B
ENSG00000169641	704.9926818	1417.759767	-1.00788941	7.40E-06	4.04E-05	LUZP1
ENSG00000099954	65.16918651	1.060160428	5.929131774	7.41E-06	4.05E-05	CECR2
ENSG00000085760	416.5776067	784.244802	-0.91270726	7.42E-06	4.05E-05	MTIF2
ENSG00000183918	56.31121612	0	8.148814588	7.44E-06	4.06E-05	SH2D1A
ENSG00000256235	370.4365628	1341.133371	-1.856335902	7.45E-06	4.06E-05	SMIM3
ENSG00000147383	341.054328	690.2926084	-1.01725237	7.45E-06	4.06E-05	NSDHL
ENSG00000188322	36.57920249	0.774441189	5.648931351	7.46E-06	4.07E-05	SBK1
ENSG00000188738	47.18952299	3.606684194	3.712053621	7.47E-06	4.08E-05	FSIP2
ENSG00000075340	31.91027061	1.161661783	4.860400388	7.48E-06	4.08E-05	ADD2
ENSG00000234284	108.5543576	31.06473046	1.798466486	7.49E-06	4.09E-05	ZNF879
ENSG00000286194	17.76357977	0	6.48534746	7.51E-06	4.09E-05	AC138207.9
ENSG00000245552	108.8196622	12.52491141	3.108794263	7.52E-06	4.10E-05	AP000787.1
ENSG00000135437	45.59208438	8.95823701	2.34701781	7.56E-06	4.12E-05	RDH5
ENSG00000105953	1707.090334	3125.83772	-0.872683105	7.57E-06	4.13E-05	OGDH
ENSG00000100129	1279.803566	2778.497112	-1.118407391	7.60E-06	4.14E-05	EIF3L
ENSG00000171490	1496.027189	3131.187491	-1.065704449	7.62E-06	4.15E-05	RSL1D1
ENSG00000107984	109.9109524	1532.553098	-3.801803363	7.67E-06	4.17E-05	DKK1
ENSG00000143401	985.8505784	2196.915095	-1.156190226	7.68E-06	4.18E-05	ANP32E
ENSG00000285758	17.82905459	0	6.490149332	7.69E-06	4.18E-05	AC036214.4
ENSG00000246705	5085.58423	1603.566549	1.665066544	7.72E-06	4.20E-05	H2AFJ
ENSG00000183281	43.73979767	7.429423729	2.546809343	7.75E-06	4.22E-05	PLGLB1
ENSG00000171219	67.02849727	8.640680314	2.95700007	7.76E-06	4.22E-05	CDC42BPG
ENSG00000181804	329.1953812	78.99137831	2.059019506	7.80E-06	4.24E-05	SLC9A9
ENSG00000163050	578.8407959	260.6031487	1.151733259	7.81E-06	4.24E-05	COQ8A
ENSG00000105419	325.831106	961.0652251	-1.560116515	7.83E-06	4.26E-05	MEIS3
ENSG00000214846	29.20931784	1.436768536	4.345505141	7.87E-06	4.27E-05	AC114744.1
ENSG00000112293	49.33185943	4.485066883	3.433433231	7.88E-06	4.28E-05	GPLD1
ENSG00000143185	25.12923276	0	6.98423869	7.88E-06	4.28E-05	XCL2
ENSG00000268758	34.57318629	0	7.445253687	7.89E-06	4.28E-05	ADGRE4P
ENSG00000101146	345.3004656	665.5043259	-0.946526639	7.89E-06	4.28E-05	RAE1
ENSG00000144407	19.21871464	0	6.597327841	7.95E-06	4.31E-05	PTH2R
ENSG00000132842	773.4050688	1497.219263	-0.953091466	8.00E-06	4.34E-05	AP3B1
ENSG00000124422	1892.240827	3376.737052	-0.835551703	8.01E-06	4.34E-05	USP22
ENSG00000218336	1006.033869	469.1653642	1.101003121	8.13E-06	4.41E-05	TENM3
ENSG00000182175	498.9387595	20.6403276	4.59110472	8.16E-06	4.43E-05	RGMA
ENSG00000233682	22.01154012	0	6.793105306	8.16E-06	4.43E-05	AL356417.2
ENSG00000167157	25.00935215	536.2924113	-4.423179093	8.17E-06	4.43E-05	PRRX2

ENSG00000179314	76.51578722	6.860425395	3.459159021	8.23E-06	4.46E-05	WSCD1
ENSG00000132463	1155.17007	2450.403788	-1.08503515	8.24E-06	4.47E-05	GRSF1
ENSG00000197405	435.6197132	99.15979536	2.137413688	8.32E-06	4.51E-05	C5AR1
ENSG00000168899	1516.64991	632.8997848	1.261163131	8.33E-06	4.51E-05	VAMP5
ENSG00000122335	390.2641718	91.22984696	2.094613001	8.34E-06	4.51E-05	SERAC1
ENSG00000123353	286.0212677	581.6713885	-1.024625546	8.34E-06	4.52E-05	ORMDL2
ENSG00000006459	453.8096912	148.9864704	1.608178806	8.35E-06	4.52E-05	KDM7A
ENSG00000246223	33.3295913	0.713078025	5.539049529	8.36E-06	4.52E-05	LINC01550
ENSG00000259081	25.7166121	0.387220594	6.056672613	8.37E-06	4.53E-05	AF111169.3
ENSG00000164221	347.4820642	168.5361316	1.043864355	8.39E-06	4.54E-05	CCDC112
ENSG00000090061	105.680515	254.1990939	-1.265839371	8.39E-06	4.54E-05	CCNK
ENSG00000266017	137.4902521	24.12030426	2.516212683	8.40E-06	4.54E-05	MIR4477B
ENSG00000101400	1239.41047	432.0782776	1.521039802	8.41E-06	4.55E-05	SNTA1
ENSG00000197619	236.9002362	95.78260432	1.308512904	8.41E-06	4.55E-05	ZNF615
ENSG00000117395	678.3213848	1844.628666	-1.443479439	8.47E-06	4.58E-05	EBNA1BP2
ENSG00000080824	11941.75879	27321.25509	-1.194025005	8.47E-06	4.58E-05	HSP90AA1
ENSG00000196167	25.7597347	0	7.019994433	8.48E-06	4.58E-05	COLCA1
novel.816	95.41360912	467.4083902	-2.292174827	8.51E-06	4.60E-05	-
ENSG00000240032	0	17.64953961	-6.719521985	8.52E-06	4.60E-05	LNCSRLR
ENSG00000248540	88.30375754	0.336469917	7.836149964	8.53E-06	4.60E-05	AC010931.2
ENSG00000188157	2702.076548	9281.782594	-1.780328052	8.54E-06	4.61E-05	AGRN
ENSG00000124608	399.9616329	781.1796922	-0.965883515	8.56E-06	4.62E-05	AARS2
ENSG00000134830	67.3195152	349.8365786	-2.377599536	8.61E-06	4.64E-05	C5AR2
ENSG00000155744	436.8617991	194.312937	1.169008638	8.62E-06	4.65E-05	FAM126B
ENSG00000158850	401.3231704	834.7246177	-1.056845907	8.64E-06	4.66E-05	B4GALT3
ENSG00000118246	450.8473807	878.4693709	-0.962553939	8.65E-06	4.67E-05	FASTKD2
ENSG00000131370	610.3532736	198.98256	1.618009548	8.67E-06	4.67E-05	SH3BP5
ENSG00000107562	4133.849998	190.2690306	4.440867764	8.67E-06	4.67E-05	CXCL12
ENSG00000144228	534.1069089	259.8316141	1.039407803	8.71E-06	4.69E-05	SPOPL
ENSG00000132004	84.05766375	254.5990291	-1.599766854	8.72E-06	4.70E-05	FBXW9
ENSG00000283317	16.90147958	0	6.413272589	8.74E-06	4.71E-05	AL831711.1
ENSG00000271895	124.4601813	18.02987213	2.783535317	8.76E-06	4.72E-05	AL109811.2
ENSG00000003056	802.0587201	1402.403058	-0.80606239	8.80E-06	4.74E-05	M6PR
ENSG00000091972	728.1955075	62.44868706	3.541409952	8.82E-06	4.75E-05	CD200
ENSG00000250295	57.05563078	2.079026788	4.75438319	8.84E-06	4.76E-05	RDH10-AS1
ENSG00000254166	1.830005771	74.36868744	-5.34781517	8.87E-06	4.77E-05	CASC19
ENSG00000166938	735.1219978	239.2976337	1.618080843	8.87E-06	4.77E-05	DIS3L
ENSG00000117226	1125.181433	420.2929472	1.42037445	8.92E-06	4.80E-05	GBP3
ENSG00000124440	29.33643526	376.1210666	-3.680974532	8.98E-06	4.83E-05	HIF3A
ENSG00000181481	732.9627341	316.6612191	1.210421647	8.98E-06	4.83E-05	RNF135
ENSG00000158458	43.67905271	5.828506405	2.916429509	9.01E-06	4.84E-05	NRG2
ENSG00000101194	259.4384541	1399.271736	-2.430859394	9.02E-06	4.85E-05	SLC17A9
ENSG00000258498	248.153179	0.387220594	9.326960292	9.03E-06	4.85E-05	DIO3OS
ENSG00000090020	302.5672503	838.4967565	-1.470616551	9.04E-06	4.86E-05	SLC9A1
ENSG00000111445	219.7196619	438.3046768	-0.996296861	9.05E-06	4.86E-05	RFC5
ENSG00000185946	486.6561861	190.0234711	1.357689838	9.06E-06	4.87E-05	RNPC3
ENSG00000164733	4904.619897	23537.78538	-2.262764955	9.13E-06	4.90E-05	CTSB
ENSG00000106803	1171.171284	2283.055912	-0.962969537	9.14E-06	4.91E-05	SEC61B
novel.927	30.85077705	0.356539013	6.318410744	9.25E-06	4.96E-05	-
ENSG00000171681	1474.387645	827.0581916	0.834310345	9.27E-06	4.97E-05	ATF7IP
ENSG00000164509	0.586481695	42.44748842	-6.143128342	9.29E-06	4.98E-05	IL31RA

ENSG00000105829	401.2834096	926.7846739	-1.207478629	9.29E-06	4.98E-05	BET1
ENSG00000111490	64.81978837	13.63684999	2.249072483	9.29E-06	4.98E-05	TBC1D30
ENSG00000230487	553.3457688	146.2653266	1.917832353	9.30E-06	4.99E-05	PSMG3-AS1
ENSG00000108823	45.18307987	2.079026788	4.414193956	9.36E-06	5.02E-05	SGCA
ENSG00000163659	348.2659237	1917.925645	-2.46125134	9.37E-06	5.02E-05	TIPARP
ENSG00000250125	19.26509999	0	6.602282317	9.38E-06	5.03E-05	LINC02232
ENSG00000164944	806.5798358	1585.874448	-0.975172808	9.40E-06	5.03E-05	VIRMA
ENSG00000116771	21.12784317	79.00340346	-1.902920843	9.41E-06	5.04E-05	AGMAT
ENSG00000100027	75.34386367	13.67454804	2.475024088	9.41E-06	5.04E-05	YPEL1
ENSG00000174373	714.2532344	273.7320592	1.383530032	9.43E-06	5.05E-05	RALGAPA1
ENSG00000100528	1092.071218	2490.832598	-1.189708695	9.43E-06	5.05E-05	CNIH1
ENSG00000198700	1233.387965	2365.964676	-0.939711208	9.45E-06	5.06E-05	IPO9
ENSG00000224689	32.7411005	0.336469917	6.404175215	9.47E-06	5.06E-05	ZNF812P
ENSG00000265018	37.23026136	5.411760104	2.786671002	9.47E-06	5.06E-05	AGAP12P
ENSG00000232453	46.36621499	3.718798035	3.663700296	9.47E-06	5.06E-05	AC105277.1
ENSG00000134574	1149.715457	491.4997285	1.226226256	9.48E-06	5.07E-05	DDB2
ENSG00000125810	684.7412703	75.02271405	3.192262445	9.50E-06	5.08E-05	CD93
ENSG00000139631	737.2134474	335.6834671	1.135268783	9.56E-06	5.11E-05	CSAD
novel.278	29.78716653	0.336469917	6.268199741	9.58E-06	5.12E-05	-
ENSG00000238243	23.90207739	0	6.911968123	9.59E-06	5.12E-05	OR2W3
ENSG00000167371	207.3096088	46.24429827	2.167486433	9.60E-06	5.13E-05	PRRT2
ENSG00000285219	59.38897764	10.85433018	2.438135893	9.61E-06	5.13E-05	HULC
ENSG00000134539	38.17198251	1.733100261	4.430516431	9.62E-06	5.13E-05	KLRD1
ENSG00000114737	155.5309449	47.42055374	1.709455157	9.65E-06	5.15E-05	CISH
ENSG00000177989	390.6935744	123.946023	1.658492986	9.67E-06	5.16E-05	ODF3B
ENSG00000117010	140.4086844	49.9077693	1.492885719	9.68E-06	5.16E-05	ZNF684
ENSG00000241878	497.4383566	1380.228493	-1.472460475	9.69E-06	5.17E-05	PISD
novel.667	125.6291978	269.1202036	-1.098368441	9.72E-06	5.18E-05	-
ENSG00000119227	236.7307512	65.35110787	1.855438541	9.75E-06	5.20E-05	PIGZ
ENSG00000160963	35.65122803	0	7.48911618	9.79E-06	5.22E-05	COL26A1
ENSG00000052795	669.9577672	275.6838649	1.281636877	9.81E-06	5.23E-05	FNIP2
ENSG00000244968	42.43832066	8.193252432	2.367020821	9.81E-06	5.23E-05	LIFR-AS1
ENSG00000257732	0.308355904	22.96925497	-6.138399364	9.82E-06	5.23E-05	AC089983.1
ENSG00000162337	637.8930381	1733.201523	-1.442158252	9.82E-06	5.23E-05	LRP5
ENSG00000091592	723.2773098	222.7863823	1.699672827	9.87E-06	5.26E-05	NLRP1
ENSG00000185090	51.07583345	6.584162767	2.934412207	9.91E-06	5.27E-05	MANEAL
ENSG00000172765	586.4073623	312.8922655	0.906773086	9.92E-06	5.28E-05	TMCC1
ENSG00000214279	110.1679515	36.73610454	1.585438166	9.93E-06	5.28E-05	SCART1
ENSG00000174946	28.53884133	0	7.167929265	9.93E-06	5.28E-05	GPR171
ENSG00000156017	218.3447178	479.9630488	-1.136699319	9.93E-06	5.28E-05	CARNMT1
ENSG00000264522	1190.562122	614.0363254	0.955694649	1.00E-05	5.32E-05	OTUD7B
ENSG00000180096	152.9907725	30.64425974	2.320478665	1.00E-05	5.33E-05	SEPT1
ENSG00000101213	53.4299663	4.199347643	3.655162812	1.01E-05	5.35E-05	PTK6
ENSG00000132692	41.36406805	0.336469917	6.741716694	1.01E-05	5.37E-05	BCAN
ENSG00000168477	294.4595032	11.80237677	4.635437151	1.01E-05	5.38E-05	TNXB
ENSG00000269893	428.1381677	1025.734536	-1.260798521	1.01E-05	5.38E-05	SNHG8
ENSG00000165689	609.5743432	1223.491455	-1.005337161	1.02E-05	5.42E-05	ENTR1
ENSG00000112651	461.9849058	813.8900889	-0.816777535	1.02E-05	5.42E-05	MRPL2
ENSG00000139173	273.6509605	77.24933501	1.82189594	1.02E-05	5.43E-05	TMEM117
ENSG00000130707	187.4859043	1119.343415	-2.578050069	1.02E-05	5.43E-05	ASS1
novel.159	63.75986753	17.90586153	1.831348543	1.03E-05	5.47E-05	-

ENSG00000166402	478.2197133	229.7551156	1.057556504	1.03E-05	5.49E-05	TUB
ENSG00000107554	343.8526225	1210.703333	-1.81629106	1.04E-05	5.49E-05	DNMBP
ENSG00000196482	47.83019702	2.844011366	4.06593931	1.04E-05	5.50E-05	ESRRG
ENSG00000114529	3.385646697	40.22121828	-3.581077353	1.04E-05	5.50E-05	C3orf52
ENSG00000118503	623.8740932	3916.065959	-2.650034511	1.04E-05	5.52E-05	TNFAIP3
ENSG00000286548	19.90577367	0	6.649232979	1.05E-05	5.57E-05	AL596214.1
ENSG00000168300	1308.904758	687.1478855	0.929814912	1.05E-05	5.59E-05	PCMTD1
ENSG00000167656	35.03326258	0	7.463884963	1.06E-05	5.59E-05	LY6D
ENSG00000196329	16.37420447	0	6.366863532	1.06E-05	5.59E-05	GIMAP5
ENSG00000280077	224.1438465	74.86272204	1.583927269	1.06E-05	5.59E-05	AL353763.2
ENSG00000111880	249.4357257	528.4885587	-1.083743068	1.06E-05	5.60E-05	RNGTT
ENSG00000231863	30.15562666	0.672939833	5.408929565	1.06E-05	5.60E-05	AL139393.1
ENSG00000005022	1699.214512	4563.288462	-1.42529421	1.06E-05	5.61E-05	SLC25A5
ENSG00000135898	34.85417539	0.387220594	6.494580463	1.06E-05	5.61E-05	GPR55
ENSG00000173221	603.1947717	1745.391159	-1.53305817	1.06E-05	5.63E-05	GLRX
ENSG00000182632	19.85061277	0	6.644008134	1.07E-05	5.67E-05	CCNYL2
ENSG00000260876	20.1346508	0	6.666055016	1.07E-05	5.67E-05	LINC01229
ENSG00000128692	101.7395422	298.5488872	-1.554156345	1.07E-05	5.68E-05	EIF2S2P4
ENSG00000078814	157.1723766	36.23805437	2.114599579	1.08E-05	5.69E-05	MYH7B
ENSG00000008513	446.1915816	3236.71541	-2.858807633	1.08E-05	5.69E-05	ST3GAL1
ENSG00000108671	1122.412588	2050.096161	-0.869172517	1.08E-05	5.70E-05	PSMD11
ENSG00000170703	32.37952153	0.713078025	5.496550024	1.08E-05	5.71E-05	TTLL6
ENSG00000222022	0	18.81004551	-6.811630967	1.08E-05	5.71E-05	AC112721.1
ENSG00000089876	371.8303561	742.7325043	-0.997970813	1.09E-05	5.73E-05	DHX32
ENSG00000136950	646.66703	1427.366782	-1.142532741	1.09E-05	5.75E-05	ARPC5L
ENSG00000272732	33.25150872	0.693008929	5.544457101	1.09E-05	5.76E-05	AC004982.1
ENSG00000164111	10608.63107	18480.71105	-0.800789634	1.09E-05	5.76E-05	ANXA5
ENSG00000180346	74.44798623	225.7857278	-1.600330811	1.09E-05	5.76E-05	TIGD2
ENSG00000136813	1751.898205	3145.458726	-0.844347518	1.10E-05	5.78E-05	ECPAS
ENSG00000168404	226.2212765	579.9830371	-1.358530327	1.10E-05	5.80E-05	MLKL
ENSG00000222032	0	19.8076869	-6.887063147	1.10E-05	5.81E-05	AC112721.2
ENSG00000150768	574.4928993	1172.115114	-1.029037273	1.10E-05	5.81E-05	DLAT
ENSG00000278107	29.85729994	2.171071533	3.790380564	1.11E-05	5.83E-05	AC027575.2
ENSG00000245694	133.7110719	398.8685934	-1.576126369	1.11E-05	5.84E-05	CRNDE
ENSG00000196839	48.24811891	272.5175453	-2.498246915	1.11E-05	5.85E-05	ADA
novel.835	27.15007893	2.10970837	3.666172691	1.11E-05	5.87E-05	-
ENSG00000276547	35.17868653	139.6819685	-1.990399681	1.11E-05	5.87E-05	PCDHGB5
ENSG00000111181	179.3398511	0	9.820208645	1.11E-05	5.87E-05	SLC6A12
ENSG00000142552	1348.698774	6198.445399	-2.200377636	1.12E-05	5.92E-05	RCN3
ENSG00000172403	1072.586506	57.11352611	4.229351562	1.13E-05	5.93E-05	SYNPO2
ENSG00000197576	1.843867178	34.78584024	-4.250440252	1.13E-05	5.94E-05	HOXA4
ENSG00000164305	374.5882929	763.7287074	-1.028206666	1.13E-05	5.95E-05	CASP3
ENSG00000211459	22796.05949	6788.431158	1.747645525	1.13E-05	5.96E-05	MT-RNR1
ENSG00000198824	326.4753553	674.3426615	-1.046766258	1.14E-05	6.01E-05	CHAMP1
ENSG00000151967	128.2301152	27.01647897	2.244541287	1.14E-05	6.02E-05	SCHIP1
ENSG00000287232	28.08650075	1.834601616	3.959132624	1.15E-05	6.04E-05	AC079848.2
ENSG00000164142	89.33270877	17.36394876	2.366543779	1.15E-05	6.04E-05	FAM160A1
ENSG00000126266	17.36607192	0	6.451143048	1.15E-05	6.07E-05	FFAR1
ENSG00000011523	593.6736916	308.9078173	0.942909412	1.16E-05	6.07E-05	CEP68
ENSG00000166147	2162.777819	8645.580044	-1.99912551	1.16E-05	6.09E-05	FBN1
ENSG00000185436	68.89493013	11.43265673	2.603978593	1.16E-05	6.09E-05	IFNLR1

ENSG00000144028	2471.483577	4500.961996	-0.864808824	1.16E-05	6.10E-05	SNRNP200
novel.291	22.26541931	0	6.810946105	1.16E-05	6.10E-05	-
ENSG00000259439	44.94460047	0	7.823443254	1.16E-05	6.10E-05	LINC01833
ENSG00000143727	1010.68888	1742.052702	-0.785382334	1.17E-05	6.12E-05	ACP1
ENSG00000227496	2.431602522	26.42728315	-3.446497214	1.17E-05	6.12E-05	AC099066.2
ENSG00000120332	34.97502457	0	7.461544937	1.17E-05	6.12E-05	TNN
ENSG00000196684	47.08525598	1.426156051	5.038052415	1.17E-05	6.13E-05	HSH2D
ENSG00000119915	1.539272221	27.29132894	-4.161326009	1.17E-05	6.15E-05	ELOVL3
ENSG00000155846	126.9786695	30.50518873	2.050431195	1.17E-05	6.15E-05	PPARGC1B
ENSG00000065054	1679.423849	822.1944405	1.030140241	1.17E-05	6.15E-05	SLC9A3R2
ENSG00000276128	25.28329007	0	6.994917529	1.17E-05	6.16E-05	AL591441.1
ENSG00000092621	476.1566971	1743.419429	-1.872629226	1.18E-05	6.16E-05	PHGDH
ENSG00000119421	667.2752625	1260.894416	-0.918077528	1.18E-05	6.17E-05	NDUFA8
ENSG00000059377	888.4104114	363.1205796	1.291401064	1.18E-05	6.19E-05	TBXAS1
ENSG00000030110	375.6074559	747.1369693	-0.992580554	1.19E-05	6.21E-05	BAK1
novel.207	34.22826111	0.672939833	5.595449425	1.19E-05	6.22E-05	-
ENSG00000164916	525.2866338	1398.496809	-1.412779255	1.19E-05	6.24E-05	FOXK1
ENSG00000137103	678.8553555	358.2609252	0.922085276	1.20E-05	6.27E-05	TMEM8B
ENSG00000089195	223.9949178	513.7312257	-1.19776637	1.20E-05	6.28E-05	TRMT6
ENSG00000167083	54.27676545	4.106018629	3.746947736	1.21E-05	6.31E-05	GNGT2
novel.100	16.35156752	0	6.365076395	1.21E-05	6.32E-05	-
ENSG00000141556	513.0574476	1448.161849	-1.497161368	1.21E-05	6.33E-05	TBCD
ENSG00000115694	1913.664934	3476.583436	-0.861246548	1.21E-05	6.34E-05	STK25
ENSG00000174721	169.0162202	31.49568526	2.418554817	1.22E-05	6.37E-05	FGFBP3
ENSG00000273486	75.52524434	14.72409598	2.369666958	1.23E-05	6.41E-05	AC096992.2
ENSG00000228412	67.19028993	5.207601518	3.673176612	1.23E-05	6.45E-05	AL022068.1
ENSG00000107742	313.6787609	26.48188663	3.560984594	1.24E-05	6.47E-05	SPOCK2
ENSG00000092140	343.1839576	744.0645731	-1.116586587	1.24E-05	6.49E-05	G2E3
ENSG00000067248	626.3382486	1103.087144	-0.816706003	1.24E-05	6.50E-05	DHX29
ENSG00000105088	171.4407271	31.03019607	2.469181755	1.25E-05	6.50E-05	OLFM2
ENSG00000154640	441.9611893	867.2353037	-0.972186155	1.25E-05	6.51E-05	BTG3
ENSG00000138386	1762.000052	827.4792103	1.090621949	1.25E-05	6.52E-05	NAB1
ENSG00000172399	46.61697398	4.82970914	3.292166791	1.25E-05	6.52E-05	MYOZ2
ENSG00000244716	788.9836443	1883.111922	-1.25508548	1.25E-05	6.52E-05	BX679664.3
ENSG00000275620	31.37568741	0	7.30507074	1.25E-05	6.53E-05	AL121827.2
ENSG00000186205	397.0276152	21.4785721	4.202785912	1.25E-05	6.54E-05	MARC1
ENSG00000062524	77.33393808	18.28490979	2.070897683	1.26E-05	6.54E-05	LTK
ENSG00000197769	65.12341407	3.761376372	4.087302467	1.26E-05	6.55E-05	MAP1LC3C
ENSG00000279092	0	33.47136567	-7.644261068	1.27E-05	6.60E-05	AC025678.3
novel.874	42.58062441	184.2210703	-2.112870124	1.27E-05	6.60E-05	-
novel.657	47.62750362	0.774441189	6.018807585	1.27E-05	6.61E-05	-
ENSG00000034510	16103.64608	31582.54693	-0.971749275	1.27E-05	6.62E-05	TMSB10
ENSG00000197050	162.9250049	63.05255812	1.367099286	1.27E-05	6.63E-05	ZNF420
ENSG00000140374	1037.717425	1973.640879	-0.927398426	1.28E-05	6.64E-05	ETFA
ENSG00000168781	348.0857413	123.2459161	1.496133306	1.28E-05	6.66E-05	PPIP5K1
ENSG00000275580	49.86107238	5.513261459	3.19214916	1.28E-05	6.68E-05	AC022306.2
ENSG00000169895	792.4006336	1796.549677	-1.180897051	1.28E-05	6.68E-05	SYAP1
ENSG00000143363	432.149373	191.9007795	1.171459126	1.29E-05	6.69E-05	PRUNE1
ENSG00000250208	71.59777659	1.702418679	5.359674116	1.29E-05	6.70E-05	FZD10-AS1
ENSG00000184939	896.3856548	514.1669196	0.802124833	1.29E-05	6.70E-05	ZFP90
ENSG00000038358	543.831369	1135.288715	-1.061682773	1.29E-05	6.71E-05	EDC4

ENSG00000166920	9.995689068	320.4250918	-5.0023399	1.29E-05	6.72E-05	C15orf48
ENSG00000274737	1.509042109	21.96112949	-3.859291061	1.29E-05	6.73E-05	AC004466.2
ENSG00000106305	159.906992	530.2045695	-1.729869226	1.30E-05	6.75E-05	AIMP2
ENSG00000106003	540.2682382	69.1653711	2.965711045	1.30E-05	6.76E-05	LFNG
ENSG00000074964	1164.613172	372.7589828	1.644342643	1.31E-05	6.78E-05	ARHGEF10L
ENSG00000064932	82.44962613	264.9576081	-1.682965916	1.31E-05	6.80E-05	SBNO2
ENSG00000175591	40.13433411	1.467450118	4.792011782	1.31E-05	6.80E-05	P2RY2
ENSG00000182021	18.91043	0	6.574094073	1.31E-05	6.81E-05	AL591379.1
ENSG00000005194	446.0472145	1053.214705	-1.239799394	1.31E-05	6.81E-05	CIAPIN1
ENSG00000159840	76.98664745	289.4181198	-1.910782567	1.32E-05	6.85E-05	ZYX
ENSG00000004468	113.5709633	2.71054416	5.422452726	1.32E-05	6.86E-05	CD38
ENSG00000159199	909.3849538	1852.262994	-1.026465583	1.33E-05	6.87E-05	ATP5MC1
ENSG00000175137	625.3070191	1335.8222	-1.094868971	1.33E-05	6.88E-05	SH3BP5L
ENSG00000148604	27.22962897	0	7.10088119	1.33E-05	6.89E-05	RGR
ENSG00000101361	1238.137883	3333.108496	-1.428805407	1.33E-05	6.90E-05	NOP56
ENSG00000178826	32.10243593	0.713078025	5.48505844	1.33E-05	6.91E-05	TMEM139
ENSG00000067113	1004.180783	323.4215214	1.633601226	1.33E-05	6.91E-05	PLPP1
ENSG00000104549	393.0803745	1038.92022	-1.4022614	1.34E-05	6.93E-05	SQLE
ENSG00000157593	1340.093185	2697.231962	-1.009134568	1.34E-05	6.93E-05	SLC35B2
ENSG00000154269	27.92553446	0.672939833	5.296371335	1.34E-05	6.96E-05	ENPP3
ENSG00000153975	142.7599105	285.4443178	-0.999539604	1.34E-05	6.96E-05	ZUP1
ENSG00000261083	40.75645996	0.672939833	5.851603663	1.36E-05	7.03E-05	LINC02516
ENSG00000142208	2144.093726	3783.115463	-0.819137167	1.36E-05	7.04E-05	AKT1
ENSG00000125814	423.3497553	189.0934859	1.162702782	1.36E-05	7.04E-05	NAPB
ENSG00000170085	121.8866586	250.9051333	-1.042161605	1.36E-05	7.05E-05	SIMC1
ENSG00000011638	43.8881562	186.961093	-2.089465943	1.37E-05	7.07E-05	TMEM159
ENSG00000181638	100.5156496	215.2028271	-1.097536088	1.37E-05	7.10E-05	ZFP41
ENSG00000165684	385.2958018	790.7679396	-1.037050497	1.38E-05	7.12E-05	SNAPC4
ENSG00000169085	18.3286069	0	6.530587423	1.38E-05	7.15E-05	VXN
novel.794	9.733931982	93.87731165	-3.269374181	1.38E-05	7.15E-05	-
novel.162	19.53974957	0	6.621289037	1.39E-05	7.17E-05	-
ENSG00000137078	52.14244946	1.161661783	5.551913541	1.39E-05	7.17E-05	SIT1
ENSG00000196876	65.96076945	254.8509715	-1.948686337	1.39E-05	7.18E-05	SCN8A
ENSG00000168090	1697.441111	2907.641947	-0.77647994	1.40E-05	7.21E-05	COPS6
ENSG00000135127	49.97260402	3.210006989	3.955570711	1.40E-05	7.22E-05	BICDL1
ENSG00000163898	55.9776447	0.356539013	7.178350545	1.40E-05	7.23E-05	LIPH
ENSG00000196275	374.2099774	63.61355968	2.554066305	1.41E-05	7.27E-05	GTF2IRD2
ENSG00000175264	42.43832066	9.04912588	2.235369342	1.41E-05	7.28E-05	CHST1
ENSG00000243244	662.4712796	286.8976882	1.208420219	1.41E-05	7.30E-05	STON1
ENSG00000282851	100.0308841	12.15634724	3.048688809	1.42E-05	7.32E-05	BISPR
ENSG00000172543	90.20250088	8.600413728	3.405325444	1.42E-05	7.32E-05	CTSW
ENSG00000156042	108.800102	29.37061251	1.883733213	1.42E-05	7.33E-05	CFAP70
ENSG00000110841	1093.543504	2731.33659	-1.320693074	1.43E-05	7.36E-05	PPFIBP1
ENSG00000111145	846.5213747	3117.04854	-1.880535944	1.43E-05	7.37E-05	ELK3
ENSG00000083290	1059.766763	468.4136078	1.178074822	1.43E-05	7.38E-05	ULK2
ENSG00000147443	74.03063276	1.345879667	5.736777684	1.43E-05	7.38E-05	DOK2
ENSG00000082196	101.5426901	14.10678712	2.841936622	1.43E-05	7.39E-05	C1QTNF3
ENSG00000144476	1180.354004	166.1550065	2.827735293	1.44E-05	7.40E-05	ACKR3
ENSG00000186812	1055.736456	437.5909908	1.270885274	1.44E-05	7.42E-05	ZNF397
ENSG00000249958	25.73047351	1.049547942	4.586447062	1.44E-05	7.42E-05	CCT7P2
novel.98	15.7235016	0	6.308780175	1.44E-05	7.43E-05	-

ENSG00000164294	909.0356496	3582.425335	-1.978637108	1.44E-05	7.43E-05	GPX8
ENSG00000243696	36.34140727	2.221822211	4.058809689	1.45E-05	7.44E-05	AC006254.1
ENSG00000127324	565.7420396	1.682349583	8.373639037	1.46E-05	7.49E-05	TSPAN8
novel.683	0	28.44049885	-7.413331425	1.46E-05	7.51E-05	-
ENSG00000137135	40.27069779	148.4678032	-1.881559628	1.46E-05	7.53E-05	ARHGEF39
novel.474	29.69633366	3.200550379	3.205958377	1.46E-05	7.53E-05	-
ENSG00000285700	28.2164102	0	7.151596645	1.47E-05	7.55E-05	AC104260.3
ENSG00000273247	103.5710589	28.01399197	1.887181914	1.48E-05	7.59E-05	AC097376.2
ENSG00000078808	3091.705648	5847.75693	-0.919487486	1.49E-05	7.63E-05	SDF4
ENSG00000226496	24.82470907	0	6.966730601	1.49E-05	7.64E-05	LINC00323
ENSG00000179270	15.76633947	0	6.312305155	1.49E-05	7.67E-05	PCARE
ENSG00000244586	6.693139545	156.3692874	-4.546883015	1.49E-05	7.67E-05	WNT5A-AS1
ENSG00000119715	15.44913675	0	6.283110155	1.49E-05	7.67E-05	ESRRB
novel.205	2.773949485	51.94049334	-4.23573968	1.50E-05	7.68E-05	-
ENSG00000132383	973.9561623	1896.009917	-0.961243876	1.51E-05	7.73E-05	RPA1
ENSG00000134201	82.79325422	2.129777466	5.274021162	1.51E-05	7.74E-05	GSTM5
ENSG00000094914	822.9254194	1417.089212	-0.783994039	1.51E-05	7.74E-05	AAAS
ENSG00000231252	25.02983815	0	6.978848948	1.51E-05	7.76E-05	AC099792.1
ENSG00000185101	37.92645159	1.854670712	4.380941282	1.52E-05	7.81E-05	ANO9
ENSG00000164054	2836.081805	5293.042703	-0.900243392	1.53E-05	7.83E-05	SHISA5
ENSG00000186301	90.55811195	18.15144258	2.316482013	1.53E-05	7.85E-05	MST1P2
ENSG00000184363	13.68216978	58.62988188	-2.100984433	1.53E-05	7.86E-05	PKP3
ENSG00000066855	286.4831671	684.9333025	-1.257813421	1.54E-05	7.87E-05	MTFR1
ENSG00000122565	2027.831722	3847.616244	-0.924095458	1.54E-05	7.88E-05	CBX3
ENSG00000026508	4400.380227	11010.11612	-1.323095957	1.54E-05	7.90E-05	CD44
ENSG00000177706	1637.236017	5616.489239	-1.778364699	1.54E-05	7.91E-05	FAM20C
ENSG00000138035	484.9401052	1027.070864	-1.08282667	1.55E-05	7.92E-05	PNPT1
ENSG00000136448	1601.914273	2750.544298	-0.780007157	1.55E-05	7.93E-05	NMT1
ENSG00000145545	346.7696753	624.7214637	-0.849408466	1.55E-05	7.95E-05	SRD5A1
ENSG00000132967	490.5784373	1041.672876	-1.086724578	1.55E-05	7.95E-05	HMGB1P5
ENSG00000165443	21.23329256	0	6.742961804	1.56E-05	7.96E-05	PHYHIPL
ENSG00000186017	267.2245645	123.2207099	1.116618684	1.56E-05	7.96E-05	ZNF566
novel.144	16.12380149	0	6.344196626	1.56E-05	7.97E-05	-
ENSG00000284707	74.50702244	6.74587141	3.461062375	1.56E-05	7.98E-05	AC079781.5
ENSG00000116649	1144.469201	2988.865093	-1.385033876	1.56E-05	7.98E-05	SRM
ENSG00000180855	79.74032509	26.43673976	1.590157175	1.57E-05	8.04E-05	ZNF443
ENSG00000068831	293.3324708	29.34481122	3.316510235	1.59E-05	8.10E-05	RASGRP2
ENSG00000074181	973.6808108	2932.123782	-1.590465695	1.59E-05	8.10E-05	NOTCH3
ENSG00000232533	69.17688778	215.0030353	-1.635852761	1.59E-05	8.11E-05	AC093673.1
ENSG00000184588	274.1585607	1775.682992	-2.695308283	1.60E-05	8.15E-05	PDE4B
ENSG00000135040	251.7435449	492.0518683	-0.966974584	1.60E-05	8.16E-05	NAA35
ENSG00000237187	1341.88249	417.9239285	1.682377523	1.60E-05	8.17E-05	NR2F1-AS1
ENSG00000233328	47.84642318	135.4993983	-1.500503488	1.60E-05	8.18E-05	PFN1P1
ENSG00000107954	108.7050534	12.51648228	3.118178532	1.60E-05	8.18E-05	NEURL1
ENSG00000286558	18.32867817	0	6.53056623	1.60E-05	8.18E-05	AC098679.5
ENSG00000130429	182.4844572	82.22975513	1.150515868	1.60E-05	8.18E-05	ARPC1B
ENSG00000214783	48.6340592	5.911222933	3.021323532	1.61E-05	8.19E-05	POLR2J4
ENSG00000177103	21.44525935	0	6.755427878	1.61E-05	8.21E-05	DSCAML1
ENSG00000189058	58205.07668	685.2277239	6.408319816	1.61E-05	8.22E-05	APOD
ENSG00000013619	421.785853	123.2544267	1.776317296	1.61E-05	8.22E-05	MAMLD1
ENSG00000173805	41.0465806	3.32212083	3.646845973	1.62E-05	8.23E-05	HAP1

ENSG00000156471	749.516809	1778.033176	-1.24646785	1.62E-05	8.26E-05	PTDSS1
ENSG00000147852	1077.912715	233.8881257	2.205036536	1.62E-05	8.27E-05	VLDLR
ENSG00000228692	23.08879858	0.387220594	5.900547146	1.63E-05	8.31E-05	AL445307.1
ENSG00000152556	1101.236219	2630.152036	-1.256031039	1.64E-05	8.33E-05	PFKM
novel.608	26.62656475	0.693008929	5.221014453	1.64E-05	8.33E-05	-
ENSG00000114209	533.6059604	1005.278015	-0.913752288	1.65E-05	8.40E-05	PDCD10
novel.422	189.4266748	77.78367056	1.28527443	1.65E-05	8.40E-05	-
ENSG00000137561	29.47594733	1.436768536	4.360000418	1.65E-05	8.42E-05	TTPA
ENSG00000105341	571.0192277	1038.627121	-0.862766584	1.65E-05	8.42E-05	DMAC2
ENSG00000244879	485.3407058	1316.157531	-1.439009994	1.66E-05	8.42E-05	GABPB1-AS1
ENSG00000103254	750.1778143	412.5713016	0.862500325	1.66E-05	8.43E-05	FAM173A
ENSG00000197045	3675.793442	1642.841141	1.161743082	1.66E-05	8.43E-05	GMFB
ENSG00000253954	13.72242908	67.62034153	-2.305633746	1.66E-05	8.44E-05	HMGN1P38
ENSG00000148734	28.88487796	0	7.185334076	1.66E-05	8.44E-05	NPFFR1
ENSG00000034053	5.240796714	118.0043753	-4.497785102	1.66E-05	8.45E-05	APBA2
ENSG00000241684	199.8798341	12.79732123	3.974092598	1.67E-05	8.47E-05	ADAMTS9-AS2
ENSG00000233006	105.7392946	19.3956925	2.447636939	1.67E-05	8.51E-05	MIR3936HG
ENSG00000258441	459.7119204	161.8765255	1.506023017	1.68E-05	8.54E-05	LINC00641
ENSG00000116685	1380.468089	2790.321566	-1.015191528	1.70E-05	8.62E-05	KIAA2013
ENSG00000184613	34.91115975	0.336469917	6.496924811	1.70E-05	8.63E-05	NELL2
ENSG00000225231	47.09940212	0	7.891051121	1.71E-05	8.66E-05	LINC02470
ENSG00000162981	132.7503875	23.11333466	2.523123854	1.71E-05	8.67E-05	LRATD1
ENSG00000111727	731.7566389	243.7434143	1.585349235	1.71E-05	8.67E-05	HCFC2
ENSG00000106771	2763.135319	1328.863205	1.055971966	1.71E-05	8.68E-05	TMEM245
ENSG00000269044	189.394835	67.65418677	1.487468435	1.72E-05	8.73E-05	AC024075.2
ENSG00000147862	1227.11195	214.7529716	2.513559966	1.72E-05	8.74E-05	NFIB
ENSG00000133739	538.8780003	185.8430023	1.534497687	1.73E-05	8.75E-05	LRRCC1
ENSG00000138030	131.5200848	32.19686653	2.026302575	1.73E-05	8.75E-05	KHK
ENSG00000108578	468.3433903	1085.56617	-1.213192804	1.73E-05	8.75E-05	BLMH
ENSG00000183780	99.22653903	5.501493098	4.180714894	1.73E-05	8.76E-05	SLC35F3
ENSG00000267481	52.66421179	14.76667432	1.836932047	1.73E-05	8.76E-05	AC011477.2
ENSG00000132702	33.30766636	0.336469917	6.429161756	1.74E-05	8.79E-05	HAPLN2
ENSG00000123178	282.0691977	542.3261943	-0.943187757	1.74E-05	8.79E-05	SPRYD7
ENSG00000264364	3087.229043	1704.038995	0.857267973	1.74E-05	8.79E-05	DYNLL2
ENSG00000148308	785.9857494	1343.434536	-0.773254285	1.74E-05	8.82E-05	GTF3C5
ENSG00000196465	469.6930045	1175.0387	-1.322832262	1.74E-05	8.82E-05	MYL6B
ENSG00000181036	37.30612138	1.00940975	5.144765823	1.74E-05	8.83E-05	FCRL6
ENSG00000169813	2791.067547	4926.226651	-0.819711829	1.75E-05	8.83E-05	HNRNPF
novel.626	91.54087603	12.67703504	2.855487222	1.75E-05	8.84E-05	-
ENSG00000226149	40.61304475	5.869800472	2.806715197	1.75E-05	8.86E-05	AL356124.1
ENSG00000277531	29.3123312	0.672939833	5.369313551	1.76E-05	8.90E-05	PNMA8C
ENSG00000169032	899.0061721	2143.668194	-1.2536819	1.77E-05	8.96E-05	MAP2K1
ENSG00000203709	175.9352029	42.17995889	2.057926414	1.78E-05	8.97E-05	MIR29B2CHG
ENSG00000176623	1781.288093	816.9055348	1.124362414	1.78E-05	8.98E-05	RMDN1
ENSG00000171223	11662.61089	2257.642743	2.36901539	1.78E-05	8.99E-05	JUNB
ENSG00000130164	522.0047733	2021.611474	-1.953244734	1.78E-05	9.00E-05	LDLR
ENSG00000174444	17868.94947	37030.14701	-1.051250931	1.79E-05	9.04E-05	RPL4
ENSG00000096717	700.3973603	342.5069177	1.031471836	1.80E-05	9.07E-05	SIRT1
ENSG00000132481	354.1722261	170.3697529	1.054411673	1.80E-05	9.07E-05	AC087289.1
ENSG00000256223	275.2514202	98.59174297	1.479325259	1.80E-05	9.10E-05	ZNF10
ENSG00000140873	1.835020366	707.4478401	-8.592904021	1.81E-05	9.13E-05	ADAMTS18

ENSG00000008735	57.54664884	12.49410143	2.199625873	1.81E-05	9.15E-05	MAPK8IP2
ENSG00000179066	15.45798357	0	6.283860266	1.81E-05	9.15E-05	AC020907.1
ENSG00000100284	615.71948	1285.068738	-1.061226631	1.82E-05	9.15E-05	TOM1
ENSG00000186153	245.1888059	82.15667079	1.575887968	1.82E-05	9.18E-05	WWOX
ENSG00000205300	0	29.11989439	-7.443358461	1.83E-05	9.23E-05	AL356414.1
ENSG00000160654	36.61021553	0	7.527454176	1.83E-05	9.23E-05	CD3G
ENSG00000284543	1.203193503	89.79499217	-6.217337581	1.83E-05	9.24E-05	LINC01226
ENSG00000122986	202.9664258	61.52605659	1.719800031	1.84E-05	9.25E-05	HVCN1
ENSG00000154553	465.3001482	121.2193909	1.941040769	1.84E-05	9.25E-05	PDLIM3
ENSG00000245164	34.66305026	0.387220594	6.486542	1.84E-05	9.25E-05	LINC00861
ENSG00000173114	118.3562717	19.06063525	2.624591041	1.84E-05	9.27E-05	LRRN3
ENSG00000072310	4394.954432	1636.385194	1.425340555	1.84E-05	9.28E-05	SREBF1
ENSG00000282849	0	15.06069429	-6.494118819	1.85E-05	9.29E-05	AL359834.1
ENSG00000232949	4.539734085	169.3117161	-5.220379503	1.85E-05	9.31E-05	AC002480.2
novel.197	115.6041488	7.012677428	4.036275177	1.85E-05	9.31E-05	-
ENSG00000164841	58.12056622	4.717595299	3.629355327	1.86E-05	9.35E-05	TMEM74
ENSG00000254944	29.21669754	0.713078025	5.348798527	1.86E-05	9.35E-05	ATP5PBP5
ENSG00000141012	340.1663715	761.6934367	-1.162526545	1.86E-05	9.35E-05	GALNS
ENSG00000188811	720.7529547	246.8013449	1.545125727	1.86E-05	9.37E-05	NHLRC3
ENSG00000180822	218.9616152	533.3860658	-1.28432818	1.87E-05	9.37E-05	PSMG4
ENSG00000136827	682.3176169	1365.547126	-1.001178275	1.87E-05	9.40E-05	TOR1A
ENSG00000099622	7774.844397	3875.699935	1.004387064	1.87E-05	9.40E-05	CIRBP
ENSG00000244480	46.27531119	7.601744858	2.612076088	1.88E-05	9.42E-05	AC005154.2
ENSG00000197134	27.67889243	1.406086955	4.276793577	1.88E-05	9.43E-05	ZNF257
novel.428	16.11878689	80.83492296	-2.325827123	1.90E-05	9.54E-05	-
ENSG00000107949	777.3813579	1370.92421	-0.818622981	1.90E-05	9.54E-05	BCCIP
ENSG00000162889	1450.168862	2721.93701	-0.908292546	1.91E-05	9.59E-05	MAPKAPK2
ENSG00000243660	99.41421577	38.16457234	1.383144648	1.91E-05	9.60E-05	ZNF487
ENSG00000141314	22.972964	0.387220594	5.894043852	1.91E-05	9.61E-05	RHBDL3
ENSG00000177963	1564.987566	3001.514417	-0.939644382	1.92E-05	9.61E-05	RIC8A
ENSG00000178209	9100.898035	21738.15906	-1.256151556	1.92E-05	9.61E-05	PLEC
ENSG00000235109	185.7860653	17.33583572	3.415125885	1.92E-05	9.64E-05	ZSCAN31
ENSG00000122705	1790.669158	3822.217871	-1.093994682	1.92E-05	9.64E-05	CLTA
ENSG00000170634	313.1822301	135.6638038	1.20726087	1.92E-05	9.64E-05	ACYP2
ENSG00000185658	1489.581292	791.1234383	0.913247516	1.93E-05	9.66E-05	BRWD1
ENSG00000239779	416.6632828	217.2880185	0.939174365	1.93E-05	9.69E-05	WBP1
ENSG00000223947	3.312578714	28.41054078	-3.09308079	1.94E-05	9.71E-05	AC016738.1
ENSG00000104112	24.31817652	0.356539013	5.976952506	1.94E-05	9.71E-05	SCG3
ENSG00000184515	27.78484036	0.387220594	6.167420558	1.94E-05	9.72E-05	BEX5
ENSG00000272512	33.40538004	3.159256312	3.383937044	1.94E-05	9.73E-05	AL645608.7
ENSG00000004455	1839.417688	2971.432215	-0.691906718	1.95E-05	9.77E-05	AK2
ENSG00000248994	30.12088015	0	7.245839619	1.95E-05	9.77E-05	AC124852.1
ENSG00000137414	1112.12236	541.6761014	1.037287968	1.96E-05	9.82E-05	FAM8A1
ENSG00000175294	6.669248947	40.99540268	-2.617177229	1.96E-05	9.82E-05	CATSPER1
ENSG00000168826	125.4518628	50.45451519	1.313052919	1.96E-05	9.82E-05	ZBTB49
ENSG00000042493	1132.640574	2487.161547	-1.134642868	1.97E-05	9.83E-05	CAPG
ENSG00000144596	33.27715116	1.793307549	4.212070044	1.97E-05	9.83E-05	GRIP2
ENSG00000119431	289.6256202	100.6883175	1.522380619	1.97E-05	9.84E-05	HDHD3
ENSG00000255717	581.5459529	1133.279027	-0.962658072	1.97E-05	9.84E-05	SNHG1
ENSG00000287837	50.26925016	10.47528193	2.266399878	1.98E-05	9.87E-05	AL590064.1
ENSG00000198825	1672.91393	785.3750802	1.09129943	2.00E-05	1.00E-04	INPP5F

ENSG00000001167	366.7287864	660.3684772	-0.84821169	2.02E-05	0.000100798	NFYA
ENSG00000107625	750.6731631	1467.677311	-0.967159557	2.02E-05	0.000100906	DDX50
ENSG00000159423	989.4422386	412.4469527	1.261944462	2.03E-05	0.000101147	ALDH4A1
ENSG00000137547	429.9656661	1031.184636	-1.262389095	2.03E-05	0.000101154	MRPL15
ENSG00000143157	1822.374349	862.1146092	1.079785754	2.03E-05	0.000101367	POGK
ENSG00000064205	35.56233298	2.954969332	3.61180364	2.04E-05	0.000101883	CCN5
ENSG00000160948	304.2043645	69.55361917	2.129817355	2.04E-05	0.000101946	VPS28
ENSG00000179889	110.7658586	288.6610501	-1.383110268	2.05E-05	0.000102214	PDXDC1
ENSG00000170113	567.7242999	268.8454008	1.077970074	2.07E-05	0.000103033	NIPA1
ENSG00000128578	34.20472617	226.632179	-2.728605332	2.07E-05	0.00010328	STRIP2
ENSG00000130713	388.7379343	906.3695453	-1.221313856	2.09E-05	0.000103943	EXOSC2
ENSG00000166869	16.7593893	0	6.399881297	2.09E-05	0.000103977	CHP2
ENSG00000273253	52.67180495	14.67462957	1.841877728	2.09E-05	0.000104074	AL022328.4
ENSG00000255189	15.50959698	0	6.288214244	2.09E-05	0.000104218	GLYATL1P1
ENSG00000165915	1506.579117	2925.668948	-0.957412355	2.10E-05	0.000104824	SLC39A13
ENSG00000234232	16.79943514	0	6.405152189	2.11E-05	0.00010503	AC243772.3
ENSG00000108479	396.3882219	872.1143637	-1.137826864	2.12E-05	0.000105534	GALK1
ENSG00000186132	173.1158651	65.19654409	1.405286988	2.12E-05	0.000105677	C2orf76
ENSG00000103549	813.2490124	1554.766312	-0.93501774	2.12E-05	0.000105677	RNF40
ENSG00000274272	40.92419269	7.002064943	2.529751186	2.13E-05	0.000105775	AC069281.2
ENSG00000169181	39.40164483	0	7.633510598	2.13E-05	0.00010601	GSG1L
novel.5	28.2575675	0.672939833	5.317072096	2.13E-05	0.000106053	-
ENSG00000141934	38.37439048	486.4689051	-3.664243761	2.14E-05	0.000106261	PLPP2
ENSG00000104435	11.05686356	343.8021192	-4.96041822	2.14E-05	0.000106285	STMN2
ENSG00000148600	36.54961312	2.587817834	3.842038754	2.14E-05	0.000106292	CDHR1
ENSG00000118898	7.95937184	48.30338435	-2.606500889	2.14E-05	0.000106395	PPL
ENSG00000269837	99.24257956	24.02137145	2.041584144	2.14E-05	0.000106534	IPO5P1
ENSG00000197165	27.38823015	1.722487775	3.950308942	2.15E-05	0.000106802	SULT1A2
ENSG00000182326	10943.27502	2147.762693	2.349137944	2.15E-05	0.000106865	C1S
ENSG00000111581	579.6578082	1093.630484	-0.91612373	2.16E-05	0.000107052	NUP107
ENSG00000272692	36.04594386	6.53212782	2.472593761	2.16E-05	0.000107119	AC010997.4
ENSG00000105707	24.53988776	0.743759607	5.087307479	2.16E-05	0.000107379	HPN
ENSG00000038210	361.8389704	738.4197714	-1.028933533	2.17E-05	0.000107677	PI4K2B
ENSG00000259683	120.7447242	15.50914966	2.968706257	2.17E-05	0.000107712	AC243562.2
ENSG00000099338	45.60566106	3.068367442	3.860218111	2.17E-05	0.000107823	CATSPERG
ENSG00000174370	114.5747981	13.73475533	3.069364115	2.19E-05	0.000108406	C11orf45
ENSG00000034971	29.5040258	0	7.21598068	2.19E-05	0.000108619	MYOC
ENSG00000188001	28.24148353	3.169868797	3.13811735	2.19E-05	0.000108732	TPRG1
ENSG00000057608	3479.171592	6534.687513	-0.909423469	2.20E-05	0.000108871	GDI2
novel.363	58.3366934	0.774441189	6.304699557	2.20E-05	0.000108964	-
ENSG00000142405	16.4510334	0	6.373098165	2.21E-05	0.000109371	NLRP12
ENSG00000197728	1329.712578	3413.804503	-1.360356099	2.21E-05	0.000109371	RPS26
ENSG00000081923	119.2247111	657.4677509	-2.463709361	2.21E-05	0.000109583	ATP8B1
ENSG00000131236	2738.85382	4911.090317	-0.842453404	2.22E-05	0.000110199	CAP1
ENSG00000178338	261.234311	123.312883	1.082122509	2.23E-05	0.0001103	ZNF354B
ENSG00000034677	946.3987765	466.1407778	1.02147001	2.23E-05	0.000110324	RNF19A
ENSG00000255274	16.11495468	0	6.343531637	2.23E-05	0.000110324	SMIM35
ENSG00000164093	0	14.86933155	-6.473811011	2.23E-05	0.000110341	PITX2
ENSG00000226026	32.94315282	0.713078025	5.522555664	2.23E-05	0.000110392	AC092802.1
ENSG00000113108	737.0381925	338.6410862	1.122169552	2.23E-05	0.000110392	APBB3
ENSG00000236816	17.36954813	0	6.453399488	2.23E-05	0.000110539	ANKRD20A7P

ENSG00000198914	40.29390386	0	7.665851673	2.24E-05	0.000110949	POU3F3
ENSG00000166780	50.14442623	150.2766379	-1.581414355	2.24E-05	0.000110954	BMERB1
ENSG00000187773	23.62638762	0	6.895279538	2.25E-05	0.000111421	DIPK1C
ENSG00000214193	123.5158378	480.5619327	-1.959414092	2.27E-05	0.000112131	SH3D21
ENSG00000117228	1684.033677	745.5383765	1.175132335	2.27E-05	0.000112384	GBP1
ENSG00000181333	5.775664997	81.53246103	-3.819366316	2.28E-05	0.000112665	HEPHL1
ENSG00000227487	74.81552088	4.044655466	4.220394481	2.28E-05	0.000112665	NCAM1-AS1
ENSG00000272398	37.15142333	0.387220594	6.586649158	2.28E-05	0.000112777	CD24
ENSG00000138698	2440.592346	1254.609877	0.960053738	2.29E-05	0.000112931	RAP1GDS1
ENSG00000167103	12.93436579	69.10028352	-2.413574977	2.29E-05	0.000113041	PIP5KL1
ENSG00000011007	588.841971	1187.279891	-1.011969677	2.29E-05	0.000113063	ELOA
ENSG00000261026	35.17783233	0	7.471033478	2.29E-05	0.000113063	AC105046.1
ENSG00000255400	0	27.12242825	-7.34093992	2.29E-05	0.000113148	AC124276.2
ENSG00000229140	14.81480259	0	6.222912631	2.31E-05	0.000113739	CCDC26
ENSG00000163933	302.6542235	632.4294275	-1.063603352	2.31E-05	0.000113811	RFT1
ENSG00000115919	143.6850495	848.8370736	-2.562307273	2.31E-05	0.000114011	KYNU
ENSG00000016391	59.06489377	4.248942446	3.788947031	2.31E-05	0.000114126	CHDH
ENSG00000242715	9.098344172	43.68142982	-2.264794027	2.32E-05	0.00011421	CCDC169
ENSG00000092200	61.16708491	6.705604823	3.207514176	2.32E-05	0.0001144	RPGRIP1
ENSG00000227066	25.60543611	0	7.012966343	2.33E-05	0.000114652	Z98257.1
ENSG00000070495	516.6911336	1156.379271	-1.161885312	2.33E-05	0.000114659	JMJD6
ENSG00000155363	905.1030889	1853.296005	-1.03371885	2.33E-05	0.000114784	MOV10
ENSG00000177700	1546.309146	4043.829452	-1.386997513	2.34E-05	0.000115126	POLR2L
ENSG00000287023	0	15.21294633	-6.507214713	2.34E-05	0.000115239	AC018978.1
ENSG00000284734	15.06645922	0	6.247682951	2.35E-05	0.000115673	AC099063.4
ENSG00000087085	49.72958075	7.022134039	2.8106531	2.36E-05	0.000115949	ACHE
ENSG00000127863	2357.486114	532.0868862	2.147049456	2.36E-05	0.000116235	TNFRSF19
ENSG00000152127	998.951595	2282.960934	-1.192488585	2.36E-05	0.000116285	MGAT5
novel.995	125.3550892	2.292641984	5.79996319	2.37E-05	0.000116381	-
ENSG00000102034	185.8314105	611.5938243	-1.71891636	2.37E-05	0.000116381	ELF4
ENSG00000204253	76.12189777	183.2931397	-1.268239965	2.37E-05	0.00011649	HNRNPCP2
ENSG00000285928	64.81742327	13.73847974	2.228418541	2.37E-05	0.00011657	AC103591.4
ENSG00000231752	15.39494477	0	6.278524757	2.38E-05	0.000117034	EMBP1
ENSG00000122592	1.22840902	45.54838247	-5.224387496	2.38E-05	0.000117068	HOXA7
ENSG00000283236	45.79728473	6.186201292	2.897360698	2.38E-05	0.000117126	AC074141.1
ENSG00000125484	429.6495467	779.8107422	-0.860299087	2.40E-05	0.00011786	GTF3C4
ENSG00000117394	1104.36841	5400.770354	-2.289987594	2.40E-05	0.000117875	SLC2A1
ENSG00000169594	5.544138023	78.76903001	-3.833823129	2.40E-05	0.00011791	BNC1
ENSG00000135622	159.1480477	338.3722728	-1.087503077	2.41E-05	0.000118111	SEMA4F
ENSG00000139329	8460.301241	609.757074	3.794465975	2.41E-05	0.000118414	LUM
ENSG00000135744	94.50673358	7.186282827	3.700171873	2.42E-05	0.000118774	AGT
ENSG00000161395	620.4887754	265.805964	1.222017896	2.42E-05	0.000118861	PGAP3
ENSG00000216866	17.89710798	58.89450454	-1.7208743	2.42E-05	0.000118892	RPS2P55
ENSG00000198732	223.7644439	1249.635142	-2.481689082	2.42E-05	0.00011891	SMOC1
ENSG00000242498	1261.821769	710.6187526	0.828529557	2.43E-05	0.000118956	ARPIN
ENSG00000250579	23.46284312	0	6.885671104	2.43E-05	0.000119015	AC022424.1
ENSG00000126001	621.2770532	1926.327156	-1.632358523	2.44E-05	0.000119592	CEP250
ENSG00000142694	693.7931137	1724.122898	-1.313398594	2.44E-05	0.000119693	EVA1B
ENSG00000139209	80.05232758	4.872287477	4.0250159	2.44E-05	0.000119728	SLC38A4
ENSG00000177150	187.0139765	487.0208606	-1.380671778	2.45E-05	0.000120029	FAM210A
ENSG00000172167	75.84599454	178.4974854	-1.233257929	2.45E-05	0.000120044	MTBP

ENSG00000248587	3.576843098	84.1730845	-4.549533006	2.45E-05	0.000120146	GDNF-AS1
ENSG00000165511	110.5272091	33.71031543	1.713364321	2.46E-05	0.000120252	C10orf25
ENSG00000165030	239.7546768	957.2478669	-1.996841624	2.46E-05	0.00012039	NFIL3
ENSG00000143499	438.361409	842.6472453	-0.943073221	2.46E-05	0.000120585	SMYD2
ENSG00000233029	27.18887112	0.336469917	6.138055458	2.47E-05	0.000120677	AC244453.2
ENSG00000186310	341.2164332	24.89402194	3.771331136	2.47E-05	0.000120741	NAP1L3
ENSG00000167645	992.5043448	1880.075559	-0.921709617	2.47E-05	0.000121013	YIF1B
ENSG00000187650	177.0264931	80.33597371	1.141004742	2.48E-05	0.000121106	VMAC
ENSG00000170919	746.6675459	272.7892784	1.453001671	2.50E-05	0.000122122	TPT1-AS1
ENSG00000196843	717.4138229	183.4256138	1.96877279	2.50E-05	0.000122192	ARID5A
ENSG00000210194	55.40513913	12.82111474	2.108080829	2.51E-05	0.000122799	MT-TE
ENSG00000215022	46.50837655	11.83177408	1.974729529	2.51E-05	0.000122799	AL008729.1
ENSG00000275557	61.99959608	10.55787006	2.560582388	2.51E-05	0.000122834	AC242842.1
ENSG00000225125	23.62617416	0.743759607	5.032754141	2.52E-05	0.0001232	RANP4
ENSG00000136842	48.41939912	1.762625967	4.765479751	2.52E-05	0.000123347	TMOD1
ENSG00000183199	23.04728563	73.6692228	-1.67589809	2.53E-05	0.000123347	HSP90AB3P
ENSG00000216937	115.7100529	33.26417182	1.794209357	2.53E-05	0.000123407	CCDC7
ENSG00000270091	43.97945944	4.382281258	3.339395482	2.54E-05	0.000123993	AC015726.1
ENSG00000101773	312.7059001	651.2360866	-1.058415556	2.54E-05	0.000124031	RBBP8
ENSG00000285587	22.29948164	0.356539013	5.851542486	2.54E-05	0.000124073	AC012470.1
ENSG00000141431	66.00923518	2.567748738	4.696585886	2.54E-05	0.000124073	ASXL3
novel.95	38.69850253	0	7.607591093	2.55E-05	0.00012458	-
ENSG00000183423	30.87821513	2.598430319	3.599368615	2.56E-05	0.00012478	LRIT3
novel.136	0	15.38642333	-6.52207153	2.56E-05	0.000124943	-
ENSG00000177054	167.4191804	324.9215669	-0.956352958	2.57E-05	0.000125214	ZDHHC13
ENSG00000143458	653.864823	285.3928908	1.195211455	2.57E-05	0.000125214	GABPB2
ENSG00000099968	749.3096589	1292.955457	-0.787029133	2.58E-05	0.000125515	BCL2L13
ENSG00000187815	174.0611338	79.25600097	1.133159092	2.58E-05	0.000125515	ZFP69
ENSG00000184220	263.950592	556.7112175	-1.077093692	2.58E-05	0.000125654	CMSS1
ENSG00000271303	41.10557336	146.2889789	-1.831959254	2.58E-05	0.000125661	SRXN1
ENSG00000120053	357.6768554	802.5921149	-1.166502958	2.59E-05	0.000125914	GOT1
ENSG00000171703	1003.188062	491.8789867	1.028882554	2.60E-05	0.000126421	TCEA2
ENSG00000264278	77.12601697	26.76144131	1.524098565	2.60E-05	0.000126584	ZNF236-DT
ENSG00000271605	278.5299923	82.70503926	1.754458574	2.62E-05	0.000127531	MILR1
ENSG00000223547	185.3305767	55.67876535	1.730821332	2.63E-05	0.000127781	ZNF844
ENSG00000127561	9.107119713	69.53555786	-2.931492541	2.63E-05	0.000128192	SYNGR3
ENSG00000004660	110.1062376	231.5048173	-1.07284477	2.64E-05	0.0001283	CAMKK1
ENSG00000177369	17.35568672	0	6.452282986	2.64E-05	0.000128628	AC004797.1
ENSG00000223458	45.45207447	1.345879667	5.02677859	2.65E-05	0.000129019	LMO7DN-IT1
ENSG00000213347	169.9154035	497.3838787	-1.549312401	2.65E-05	0.000129085	MXD3
ENSG00000139269	2.772695836	35.5968236	-3.6938157	2.66E-05	0.000129179	INHBE
ENSG00000258451	47.84846011	1.10029862	5.459715178	2.66E-05	0.000129447	AL163636.1
ENSG00000101298	76.18543476	367.0196143	-2.26882355	2.67E-05	0.000129659	SNPH
ENSG00000184925	31.01675793	3.984448178	2.967352327	2.67E-05	0.000129773	LCN12
ENSG00000169241	341.4304091	765.2475848	-1.164541425	2.67E-05	0.000129906	SLC50A1
ENSG00000164930	199.2485607	780.0697611	-1.96949822	2.67E-05	0.000129906	FZD6
ENSG00000133740	269.1095459	105.8295	1.347083519	2.68E-05	0.000130249	E2F5
novel.774	17.99657386	0	6.502678928	2.69E-05	0.000130568	-
ENSG00000167552	18740.33377	7517.435103	1.317839187	2.70E-05	0.000131136	TUBA1A
ENSG00000268182	32.21458083	4.62670643	2.793922787	2.71E-05	0.000131597	SMIM17
ENSG00000076258	115.7557538	21.6874354	2.40878598	2.71E-05	0.000131648	FMO4

ENSG00000179300	0	15.08179087	-6.492521846	2.72E-05	0.000131865	RTL3
ENSG00000172531	1645.857703	3002.65812	-0.867419706	2.73E-05	0.000132266	PPP1CA
ENSG00000110619	75.94442023	278.7226598	-1.875393454	2.73E-05	0.000132347	CARS
novel.628	84.02934363	9.192049696	3.187478187	2.73E-05	0.000132548	-
ENSG00000143156	138.620006	355.6936432	-1.359553898	2.74E-05	0.000132706	NME7
ENSG00000215769	81.7086602	13.20473931	2.637274861	2.74E-05	0.000132871	ARHGAP27P1-BPTFP1-KPNA2P3
ENSG00000280422	27.45725245	1.518200796	4.231812264	2.76E-05	0.000133677	AC115284.2
ENSG00000171873	14.75706313	249.0657201	-4.078590701	2.76E-05	0.000133962	ADRA1D
ENSG00000285884	14.24594325	0	6.166043528	2.77E-05	0.000134042	AL022345.4
ENSG00000164099	9.411714671	61.29262908	-2.706774173	2.79E-05	0.000135048	PRSS12
ENSG00000084764	585.8474485	243.9317765	1.265049027	2.79E-05	0.000135193	MAPRE3
ENSG00000150175	14.58452927	0	6.199585902	2.79E-05	0.000135194	FRMPD2B
ENSG00000141030	690.4619176	1388.237387	-1.007830533	2.79E-05	0.000135282	COPS3
ENSG00000115942	327.0972243	619.5925503	-0.921206096	2.80E-05	0.000135642	ORC2
ENSG00000171453	245.2264863	479.9077218	-0.969209869	2.81E-05	0.000135736	POLR1C
ENSG00000249307	260.4952225	8.552231589	4.920287195	2.81E-05	0.000135867	LINC01088
ENSG00000151689	849.4417062	334.6153568	1.343144536	2.81E-05	0.000136011	INPP1
ENSG00000099817	2087.425403	3840.340045	-0.879520016	2.82E-05	0.000136526	POLR2E
ENSG00000011260	485.0384875	892.003902	-0.879190807	2.83E-05	0.000136745	UTP18
ENSG00000144158	34.11765459	94.47100226	-1.467283781	2.83E-05	0.000136938	AL078621.1
ENSG00000153395	9.224279216	93.37476606	-3.344146178	2.84E-05	0.00013723	LPCAT1
ENSG00000234665	113.4386769	3.761376372	4.896835314	2.84E-05	0.000137365	AL512625.3
ENSG00000119688	1211.583393	551.8414857	1.134789662	2.85E-05	0.000137937	ABCD4
ENSG00000129680	388.1848304	803.8693922	-1.050488987	2.86E-05	0.000137946	MAP7D3
ENSG00000011258	518.6591123	222.1041142	1.224892393	2.86E-05	0.000137946	MBTD1
ENSG00000170369	59.566127	2.129777466	4.798239672	2.86E-05	0.000138136	CST2
ENSG00000128709	0.307102255	23.46067385	-6.174405056	2.86E-05	0.000138136	HOXD9
ENSG00000108821	19391.43127	136942.3757	-2.820083018	2.86E-05	0.000138196	COL1A1
ENSG00000117625	1105.798326	498.9054056	1.148105116	2.87E-05	0.00013849	RCOR3
ENSG00000125740	5372.015183	75.63331042	6.150576528	2.87E-05	0.000138559	FOSB
ENSG00000247775	14.56439962	0	6.197797898	2.87E-05	0.000138579	SNCA-AS1
ENSG00000158169	165.6084779	361.3491518	-1.126388443	2.89E-05	0.000139238	FANCC
ENSG00000171621	163.2828938	464.4228664	-1.508489811	2.90E-05	0.000139658	SPSB1
ENSG00000164692	30715.21541	110566.4812	-1.847893309	2.90E-05	0.000139977	COL1A2
ENSG00000225210	15.86079075	0	6.320239386	2.91E-05	0.000140306	DUXAP9
ENSG00000168062	166.3677069	41.4310622	2.009947831	2.92E-05	0.000140621	BATF2
ENSG00000140398	643.2062119	157.7298551	2.02841518	2.93E-05	0.000141044	NEIL1
ENSG00000069966	124.2849101	714.2026583	-2.522986701	2.93E-05	0.000141055	GNB5
ENSG00000162129	271.2174636	541.3002983	-0.996635585	2.95E-05	0.000141998	CLPB
ENSG00000286694	15.96881871	0	6.331495661	2.96E-05	0.000142678	AL161935.3
ENSG00000178226	45.57091455	8.947624524	2.346212781	2.97E-05	0.000142851	PRSS36
ENSG00000166348	295.7662973	116.1550984	1.347471098	2.97E-05	0.000143131	USP54
ENSG00000130475	191.2252243	52.46233702	1.868633605	2.98E-05	0.000143432	FCHO1
ENSG00000213930	431.9024897	228.0090098	0.921162112	2.98E-05	0.00014347	GALT
ENSG00000119927	299.3025956	1683.253271	-2.491517726	2.99E-05	0.000143711	GPAM
ENSG00000148346	0.616711807	80.72810895	-7.045588553	2.99E-05	0.000143768	LCN2
ENSG00000093217	40.91004655	121.9159959	-1.573200042	3.00E-05	0.000144243	XYLB
ENSG00000163867	473.0740795	250.3244478	0.918183788	3.00E-05	0.000144333	ZMYM6
ENSG00000215866	23.40474765	1.029478846	4.455817358	3.00E-05	0.0001444	LINC01356
ENSG00000108309	35.99425918	5.196860638	2.816599922	3.00E-05	0.0001444	RUNDC3A
ENSG00000270872	210.5291172	65.28725738	1.689999764	3.01E-05	0.000144596	SRGAP2D

ENSG00000175334	1731.474413	3113.211919	-0.846481216	3.05E-05	0.000146802	BANF1
ENSG00000110057	1601.541907	777.3294691	1.042983749	3.06E-05	0.000146826	UNC93B1
ENSG00000238197	175.3835393	41.1622484	2.089251385	3.06E-05	0.000146826	PAXBP1-AS1
ENSG00000280254	75.42821449	16.24717707	2.208355905	3.06E-05	0.000146969	AC233723.2
ENSG00000111554	321.1392806	163.8351849	0.969716433	3.06E-05	0.000147091	MDM1
ENSG00000285791	27.85218174	1.029478846	4.712125752	3.06E-05	0.000147112	AC009879.3
ENSG00000121988	77.38150581	172.6140372	-1.157788384	3.07E-05	0.000147287	ZRANB3
ENSG00000101856	4425.667859	2203.714453	1.00580384	3.10E-05	0.0001486	PGRMC1
ENSG00000182732	40.70835059	0.743759607	5.809247496	3.10E-05	0.000148627	RGS6
ENSG00000132623	8.246273173	62.58377687	-2.927706705	3.10E-05	0.000148902	ANKEF1
ENSG00000091428	131.3150969	10.71957871	3.621739927	3.10E-05	0.000148902	RAPGEF4
ENSG00000169683	324.5617816	572.9845682	-0.819916338	3.10E-05	0.000148902	LRRC45
ENSG00000205086	17.11280564	0	6.431597402	3.11E-05	0.000148902	C2orf91
ENSG00000276953	0	14.09501875	-6.401070653	3.11E-05	0.000149024	TRBV12-4
ENSG00000100422	999.3745888	2040.3112	-1.02972634	3.11E-05	0.000149265	CERK
ENSG00000168522	1888.433129	1039.878015	0.860789639	3.12E-05	0.000149294	FNTA
ENSG00000128266	172.1416917	35.36874311	2.288147025	3.12E-05	0.000149582	GNAZ
ENSG00000269892	35.03867662	1.702418679	4.320074375	3.13E-05	0.000149869	AC125494.2
ENSG00000277851	15.22269565	0	6.261176471	3.13E-05	0.000149963	LINC02391
ENSG00000072133	11.79170378	51.16990496	-2.117408694	3.13E-05	0.000150017	RPS6KA6
ENSG00000178295	137.7891479	402.9296404	-1.548095358	3.14E-05	0.000150147	GEN1
ENSG00000248144	452.3590675	3.781445468	6.8937543	3.14E-05	0.000150148	ADH1C
ENSG00000131771	111.902066	3.741307276	4.882478979	3.14E-05	0.000150492	PPP1R1B
ENSG00000186577	1327.397142	522.6915864	1.345081502	3.15E-05	0.000150779	SMIM29
ENSG00000172869	1060.919896	511.0340122	1.054057537	3.16E-05	0.000151329	DMXL1
ENSG00000166448	203.6583412	8.959392885	4.506397264	3.17E-05	0.000151509	TMEM130
ENSG00000108582	1183.903053	3532.329967	-1.577129301	3.17E-05	0.000151514	CPD
ENSG00000004948	28.51703075	1.762625967	3.998536591	3.17E-05	0.0001517	CALCR
ENSG00000103642	481.6165559	912.036155	-0.921263185	3.18E-05	0.000152053	LACTB
ENSG00000285576	19.19077836	0	6.596861395	3.18E-05	0.000152131	AL138686.2
ENSG00000185652	70.78513922	3.801514564	4.200325709	3.19E-05	0.000152273	NTF3
ENSG00000148341	2801.007045	1103.433105	1.344077924	3.19E-05	0.000152284	SH3GLB2
ENSG00000213344	1.186824798	32.26023748	-4.744966976	3.20E-05	0.000152732	PCNPP3
ENSG00000260625	21.74706228	1.080229524	4.335665253	3.20E-05	0.000152784	AC026471.2
ENSG00000139266	550.3469882	132.8521779	2.052179745	3.20E-05	0.000152833	9-Mar
ENSG00000187510	37.2102739	1.548882377	4.649426039	3.20E-05	0.000152846	C12orf74
ENSG00000159086	1702.925042	503.5146459	1.758035187	3.20E-05	0.000152846	PAXBP1
ENSG00000122862	2247.841312	9255.129844	-2.041754419	3.21E-05	0.000153032	SRGN
ENSG00000144824	2175.403012	586.5007934	1.890955699	3.21E-05	0.000153137	PHLDB2
ENSG00000137265	55.33682849	1.365948763	5.303705113	3.22E-05	0.000153414	IRF4
ENSG00000248485	43.26697143	2.842855491	3.921674367	3.23E-05	0.000154012	PCP4L1
ENSG00000172789	0	13.39126895	-6.322935351	3.23E-05	0.000154083	HOXC5
ENSG00000163879	438.106549	189.35554	1.210567314	3.23E-05	0.000154175	DNALI1
ENSG00000108262	2596.960978	1098.161106	1.241919473	3.24E-05	0.000154559	GIT1
ENSG00000263711	0	26.05452785	-7.286889064	3.24E-05	0.000154574	AC079062.1
ENSG00000274922	50.299409	9.07163512	2.455061988	3.25E-05	0.000155068	AL139384.1
ENSG00000160072	606.3235649	1264.724874	-1.060793265	3.26E-05	0.000155581	ATAD3B
ENSG00000283689	0.308355904	18.25294394	-5.809927819	3.27E-05	0.000155857	AC018553.2
ENSG00000135269	269.5971583	912.4772677	-1.759430277	3.28E-05	0.000156411	TES
ENSG00000131187	0.307102255	18.42410919	-5.82169024	3.28E-05	0.000156411	F12
ENSG00000145555	511.5608385	2719.341968	-2.41042385	3.29E-05	0.000156601	MYO10

ENSG00000134352	5649.596226	3204.67565	0.818023285	3.29E-05	0.000156794	IL6ST
ENSG00000125245	37.01280926	2.171071533	4.097659588	3.30E-05	0.00015718	GPR18
novel.203	0	235.5159062	-10.46071776	3.31E-05	0.000157706	-
ENSG00000287988	14.55931375	0	6.197351304	3.33E-05	0.000158553	FO082814.1
ENSG00000028310	753.4302888	1283.335453	-0.768254903	3.34E-05	0.000158769	BRD9
ENSG00000229656	8.183305651	102.6763142	-3.648405815	3.35E-05	0.00015921	ITGB1-DT
ENSG00000065802	674.5690733	1280.297485	-0.924200449	3.36E-05	0.000159766	ASB1
ENSG00000161016	16814.52023	33295.70283	-0.985635937	3.36E-05	0.000159766	RPL8
ENSG00000176769	49.93381218	1.426156051	5.123360021	3.36E-05	0.000159786	TCERG1L
ENSG00000163093	660.8883306	186.7934891	1.82197059	3.38E-05	0.000160416	BBS5
ENSG00000146476	379.1085381	796.621057	-1.071731914	3.38E-05	0.000160416	ARMT1
ENSG00000284882	14.60089797	0	6.201077727	3.38E-05	0.00016059	AL359762.1
ENSG00000157087	91.64676796	3.108505634	4.865321133	3.38E-05	0.00016078	ATP2B2
ENSG00000197497	223.8820334	46.0191715	2.281754635	3.39E-05	0.00016089	ZNF665
ENSG00000168758	1795.609611	866.9057562	1.050897193	3.39E-05	0.000161196	SEMA4C
ENSG00000116670	453.9748182	895.8388083	-0.980711567	3.40E-05	0.000161433	MAD2L2
ENSG00000001630	22.51743194	78.33876436	-1.801951822	3.40E-05	0.000161491	CYP51A1
ENSG00000101745	1185.593168	482.907812	1.296028988	3.45E-05	0.000163663	ANKRD12
ENSG00000198848	4.280484296	128.0399221	-4.904876647	3.45E-05	0.000163737	CES1
novel.131	14.74800285	0	6.216974801	3.48E-05	0.000165226	-
ENSG00000122477	63.31039026	13.8398527	2.201412753	3.48E-05	0.000165226	LRRC39
ENSG00000105329	1595.832671	3329.232618	-1.06095341	3.49E-05	0.000165747	TGFB1
ENSG00000227110	33.82441374	6.45069556	2.389884967	3.51E-05	0.000166462	LMCD1-AS1
ENSG00000078589	41.26320563	0	7.700164139	3.54E-05	0.000167899	P2RY10
ENSG00000154582	819.6805221	1596.872126	-0.962180808	3.55E-05	0.000168328	ELOC
ENSG00000273017	26.32963423	2.537067156	3.384103039	3.56E-05	0.000168765	AP000240.1
ENSG00000163606	34.30182729	0.672939833	5.602074977	3.56E-05	0.000168831	CD200R1
ENSG00000091844	42.93808643	161.652893	-1.912890483	3.58E-05	0.000169416	RGS17
ENSG00000174738	1110.116175	510.5249172	1.120963604	3.58E-05	0.000169604	NR1D2
novel.385	27.09065889	0	7.09416979	3.59E-05	0.000170045	-
ENSG00000140545	3941.051025	6419.770125	-0.703915473	3.62E-05	0.000171403	MFGE8
ENSG00000067334	726.5883088	1239.828271	-0.771140935	3.63E-05	0.000171993	DNTTIP2
ENSG00000273329	137.9200979	45.01181671	1.619278452	3.65E-05	0.00017271	AC078846.1
ENSG00000085491	5.101000263	52.82879904	-3.368422713	3.66E-05	0.00017325	SLC25A24
ENSG00000185187	990.6272128	327.0044812	1.599888137	3.68E-05	0.000174197	SIGIRR
ENSG00000125834	323.1426466	683.3642098	-1.080041813	3.69E-05	0.000174434	STK35
ENSG00000175893	750.8467965	320.8693811	1.226050129	3.70E-05	0.000174878	ZDHHC21
ENSG00000136715	347.4838164	611.2084487	-0.814468641	3.70E-05	0.000175204	SAP130
ENSG00000144134	331.5578787	149.1992809	1.152179576	3.71E-05	0.000175644	RABL2A
ENSG00000157637	2002.592865	3720.699353	-0.893775257	3.73E-05	0.000176247	SLC38A10
ENSG00000171798	77.59529572	6.928805024	3.490764905	3.73E-05	0.000176247	KNDC1
ENSG00000073910	253.088528	59.71165246	2.081133464	3.73E-05	0.000176417	FRY
ENSG00000112893	1690.874775	760.7705269	1.151829125	3.73E-05	0.000176417	MAN2A1
ENSG00000143942	23.73831907	117.0930933	-2.305072998	3.74E-05	0.000176561	CHAC2
ENSG00000236892	0.89358395	36.0965904	-5.324801758	3.75E-05	0.000176969	AL357833.1
ENSG00000163462	95.4137951	332.3261228	-1.799040294	3.75E-05	0.000177081	TRIM46
ENSG00000112242	233.3596342	484.0611862	-1.052768365	3.76E-05	0.000177329	E2F3
ENSG00000047346	571.3427696	317.2974412	0.848437675	3.77E-05	0.000178026	FAM214A
novel.818	6.347031636	39.75030086	-2.649116024	3.78E-05	0.000178388	-
ENSG00000128262	22.01787964	232.9663381	-3.405091641	3.80E-05	0.000179108	POM121L9P
ENSG00000120279	70.60555383	4.394178013	3.97924231	3.80E-05	0.00017921	MYCT1

ENSG00000144747	708.8382775	1332.415132	-0.910305834	3.81E-05	0.000179858	TMF1
ENSG00000135124	490.4809092	239.3489327	1.03632992	3.82E-05	0.000180357	P2RX4
ENSG00000167964	5.186676005	36.07596055	-2.800330744	3.83E-05	0.000180409	RAB26
ENSG00000122224	22.40940399	0	6.818975358	3.84E-05	0.000180918	LY9
ENSG00000140386	396.3411252	194.0089468	1.032077531	3.84E-05	0.000181065	SCAPER
ENSG00000211677	340.7272908	0	10.74612372	3.85E-05	0.000181296	IGLC2
ENSG00000100426	320.5995837	728.3044361	-1.183260467	3.85E-05	0.000181395	ZBED4
ENSG00000179743	155.1928575	61.14328392	1.345906474	3.85E-05	0.000181601	AL450998.2
ENSG00000164023	220.2013349	468.2497627	-1.08915601	3.87E-05	0.000182064	SGMS2
ENSG00000167434	17.99908116	0	6.502928828	3.89E-05	0.000182951	CA4
novel.752	16.03053258	0	6.336593917	3.89E-05	0.000182951	-
ENSG00000007384	5041.311868	1476.99406	1.771170536	3.89E-05	0.000183018	RHBDF1
ENSG00000198881	13.96405651	0	6.13700023	3.89E-05	0.000183107	ASB12
ENSG00000175048	274.4648797	92.10206509	1.573058985	3.90E-05	0.000183705	ZDHHC14
ENSG00000132603	348.699147	874.6756692	-1.327181959	3.91E-05	0.000183764	NIP7
ENSG00000226252	31.00447835	0	7.288961943	3.93E-05	0.000184752	AL135960.1
ENSG00000113845	1036.828385	1968.983854	-0.925377433	3.93E-05	0.000184828	TIMMDC1
ENSG00000127995	569.2960523	275.5348706	1.046180817	3.93E-05	0.000184957	CASD1
novel.91	0.308355904	210.9929357	-9.340268927	3.94E-05	0.000185142	-
ENSG00000112530	20.54136148	0.387220594	5.732742175	3.94E-05	0.000185197	PACRG
ENSG00000273328	24.15498766	0.336469917	5.965216335	3.95E-05	0.000185633	AC099329.2
ENSG00000234614	36.34663533	6.817847059	2.414270661	3.96E-05	0.000185847	C2CD4D-AS1
ENSG00000054118	2180.431487	3494.404829	-0.68042176	3.98E-05	0.000186791	THRAP3
ENSG00000165895	609.6691516	170.0862514	1.843077481	3.98E-05	0.000186974	ARHGAP42
ENSG00000137752	822.5481834	361.1382335	1.186652273	3.98E-05	0.000186974	CASP1
ENSG00000089902	349.8313807	736.7156357	-1.074770803	3.99E-05	0.000187099	RCOR1
ENSG00000250846	19.90570239	0.336469917	5.687670905	3.99E-05	0.000187099	EPHA5-AS1
ENSG00000075539	1864.855152	839.4427821	1.151821217	3.99E-05	0.000187231	FRYL
ENSG00000162613	1365.359006	2511.69526	-0.879533372	4.01E-05	0.000188084	FUBP1
ENSG00000159216	743.0916662	1683.45092	-1.179845752	4.01E-05	0.000188349	RUNX1
ENSG00000182782	24.04026419	0.336469917	5.958813721	4.01E-05	0.000188349	HCAR2
ENSG00000086232	1441.758503	2642.476804	-0.873935259	4.02E-05	0.000188493	EIF2AK1
ENSG00000136490	1424.134022	680.6288578	1.065455673	4.05E-05	0.000189817	LIMD2
ENSG00000151687	88.64335591	28.11562172	1.652999627	4.05E-05	0.000190075	ANKAR
ENSG00000267107	29.53174861	0	7.217372081	4.07E-05	0.000190779	PCAT19
ENSG00000067177	36.88193054	341.6366261	-3.212686913	4.07E-05	0.000190971	PHKA1
ENSG00000156127	35.5641561	1.060160428	5.053588442	4.08E-05	0.000191152	BATF
ENSG00000110080	2418.319357	957.2130886	1.337423925	4.09E-05	0.000191446	ST3GAL4
ENSG00000223799	33.7412453	5.717548439	2.558887619	4.09E-05	0.000191604	IL10RB-DT
ENSG00000227954	3.975889338	41.91991255	-3.402436486	4.10E-05	0.000192123	TARID
ENSG00000055609	1593.327594	664.5012643	1.262033151	4.10E-05	0.000192198	KMT2C
ENSG00000125971	584.5784683	280.8229244	1.058039029	4.12E-05	0.000192797	DYNLRB1
ENSG00000166986	1461.27157	3147.19091	-1.106945134	4.12E-05	0.000192797	MARS
ENSG00000129055	645.0626101	1349.622244	-1.065122474	4.12E-05	0.00019297	ANAPC13
ENSG00000266274	21.33569301	0.336469917	5.786257249	4.14E-05	0.000193537	RN7SL138P
ENSG00000174243	1408.017855	2448.621735	-0.798382147	4.16E-05	0.000194461	DDX23
ENSG00000254254	31.61889632	0.336469917	6.35547594	4.18E-05	0.000195355	AC012349.1
ENSG00000062282	22.42967618	389.0424325	-4.117386573	4.19E-05	0.000195915	DGAT2
ENSG00000142186	1802.073189	3146.978551	-0.80421596	4.19E-05	0.000195915	SCYL1
ENSG00000279722	60.37325192	11.16127439	2.421664431	4.21E-05	0.000196634	AC007342.7
ENSG00000280434	2.990361403	27.3423364	-3.185388208	4.21E-05	0.000196879	AL031595.3

ENSG00000100632	1346.899263	2532.353217	-0.910987835	4.21E-05	0.000196964	ERH
ENSG00000010379	528.5689748	1.00940975	9.007682501	4.23E-05	0.00019751	SLC6A13
ENSG00000198829	27.7456209	1.386017859	4.288563302	4.25E-05	0.000198438	SUCNR1
ENSG00000177646	595.8375966	1125.580858	-0.917766035	4.25E-05	0.000198438	ACAD9
ENSG00000156738	37.86752974	0	7.57623699	4.25E-05	0.000198441	MS4A1
ENSG00000273576	76.83026883	26.28333185	1.542377219	4.25E-05	0.000198549	AC009283.1
ENSG00000286757	25.29360399	1.110911105	4.54287217	4.26E-05	0.000198905	AL137139.1
ENSG00000181074	22.40027244	0.743759607	4.955313368	4.26E-05	0.000199033	OR52N4
ENSG00000231890	3.593211803	34.42313667	-3.250338227	4.26E-05	0.000199033	DARS-AS1
ENSG00000123146	750.1637394	1276.326453	-0.766570362	4.27E-05	0.000199378	ADGRE5
ENSG00000286403	19.93439412	0	6.652153243	4.28E-05	0.000199532	AC010378.2
ENSG00000257219	8.565983187	77.25668981	-3.17499783	4.28E-05	0.000199532	LNCOG
ENSG00000214050	10.02466553	52.09390125	-2.375943739	4.28E-05	0.00019973	FBXO16
ENSG00000196605	221.9069162	85.57664977	1.376099471	4.29E-05	0.000200148	ZNF846
ENSG00000078674	2365.220145	1345.069473	0.814143052	4.31E-05	0.000201056	PCM1
ENSG00000087258	127.6296845	4.523920805	4.833131576	4.32E-05	0.000201333	GNAO1
ENSG00000256083	0.293240848	28.05883487	-6.428792593	4.32E-05	0.000201534	AC090673.1
ENSG00000170037	574.3126026	1061.425819	-0.885881869	4.34E-05	0.000202021	CNTROB
ENSG00000075461	48.93170138	0.356539013	6.984252326	4.38E-05	0.000204061	CACNG4
ENSG00000066044	1219.005215	2169.006788	-0.831441613	4.39E-05	0.000204274	ELAVL1
ENSG00000081148	26.13940675	1.773238453	3.871543602	4.39E-05	0.000204319	IMPG2
ENSG00000105808	43.86029084	7.643038925	2.529442217	4.39E-05	0.000204626	RASA4
ENSG00000251136	235.7709652	108.2662217	1.123375285	4.40E-05	0.000204941	AF117829.1
ENSG00000173418	889.1057767	1534.769622	-0.787803612	4.41E-05	0.00020508	NAA20
ENSG00000273409	19.63524104	0	6.629275649	4.42E-05	0.000205486	AP003481.1
ENSG00000130311	851.2463098	1645.881675	-0.95105377	4.43E-05	0.000206203	DDA1
ENSG00000124783	2317.052239	4249.906858	-0.875207765	4.43E-05	0.000206257	SSR1
ENSG00000287226	14.82608543	0	6.223878432	4.45E-05	0.000206882	AC084740.1
ENSG00000185880	53.43332781	14.24727079	1.902022213	4.45E-05	0.00020693	TRIM69
ENSG00000102312	188.6132104	466.5434787	-1.30612511	4.46E-05	0.000207387	PORCN
ENSG00000013364	3543.541315	7546.072689	-1.090493165	4.48E-05	0.000208254	MVP
ENSG00000272108	13.62672415	0	6.102080383	4.48E-05	0.000208403	AC244517.1
ENSG00000165527	2137.091673	3625.896967	-0.762670312	4.50E-05	0.000208963	ARF6
ENSG00000125746	633.4480845	1402.384518	-1.146505403	4.50E-05	0.000208963	EML2
ENSG00000153317	943.1452302	2056.00258	-1.124349639	4.51E-05	0.000209362	ASAP1
ENSG00000188051	30.42386545	3.566546002	3.086594976	4.51E-05	0.000209377	TMEM221
ENSG00000149273	11813.37732	27508.76753	-1.21947706	4.51E-05	0.000209518	RPS3
ENSG00000179051	48.8092429	275.5450972	-2.497753127	4.51E-05	0.00020954	RCC2
ENSG00000161956	354.1654162	678.2533362	-0.937813008	4.55E-05	0.000211159	SENP3
ENSG00000214021	1027.373968	335.7871859	1.613677364	4.56E-05	0.000211406	TTLL3
ENSG00000248487	179.5317765	69.6506726	1.365037368	4.57E-05	0.000212254	ABHD14A
ENSG00000138867	1123.157244	1915.010145	-0.769643996	4.58E-05	0.000212533	GUCD1
novel.812	21.96186454	0	6.791822253	4.60E-05	0.000213579	-
ENSG00000158467	595.8937379	208.0675518	1.51809263	4.62E-05	0.00021424	AHCYL2
ENSG00000100314	28.37243317	2.180528143	3.71138891	4.64E-05	0.000215237	CABP7
ENSG00000169972	128.1934309	318.4939885	-1.313786182	4.66E-05	0.000216043	PUSL1
ENSG00000068366	880.2947893	2357.876107	-1.42134811	4.66E-05	0.000216175	ACSL4
ENSG00000176083	30.40924894	0	7.259706911	4.67E-05	0.000216206	ZNF683
ENSG00000211689	15.88099168	0	6.322050975	4.69E-05	0.00021711	TRGC1
ENSG00000287733	31.63089117	4.963176346	2.661510616	4.70E-05	0.000217623	AC083862.3
ENSG00000233621	35.10694347	138.6910872	-1.980243308	4.71E-05	0.000218366	LINC01137

ENSG00000196335	29.51961157	0.356539013	6.256479837	4.72E-05	0.00021861	STK31
ENSG00000154518	1625.776174	3577.391556	-1.137873737	4.73E-05	0.000219003	ATP5MC3
ENSG00000163121	20.85215341	0.387220594	5.75431249	4.74E-05	0.000219264	NEURL3
ENSG00000287397	19.31825179	0	6.60471403	4.74E-05	0.000219611	AP002528.1
ENSG00000268754	0	20.17471001	-6.912871693	4.77E-05	0.000220821	LINC01081
ENSG00000119487	1119.532005	1905.795924	-0.767465048	4.79E-05	0.000221772	MAPKAP1
ENSG00000182083	14.95209175	0	6.235082251	4.80E-05	0.000222076	OR6B2
ENSG00000231711	196.4620904	70.82320366	1.469243446	4.80E-05	0.000222103	LINC00899
ENSG00000164051	181.8618328	374.6928337	-1.043461565	4.81E-05	0.000222231	CCDC51
ENSG00000008869	1004.764675	467.771816	1.103141386	4.81E-05	0.000222307	HEATR5B
ENSG00000113851	900.2267348	449.2616514	1.003405735	4.82E-05	0.000222608	CRBN
ENSG00000172888	1282.927532	644.631084	0.992542305	4.82E-05	0.000222962	ZNF621
ENSG00000197077	540.9384191	166.3426792	1.701157861	4.84E-05	0.000223476	KIAA1671
ENSG00000162433	182.7542191	1131.14877	-2.630123349	4.84E-05	0.000223663	AK4
ENSG00000150457	2915.715262	998.4156151	1.546115562	4.88E-05	0.000225212	LATS2
ENSG00000197258	135.5365178	372.8203116	-1.460050039	4.90E-05	0.000226404	EIF4BP6
ENSG00000125804	46.11843403	0.336469917	6.899829934	4.90E-05	0.000226514	FAM182A
ENSG00000122025	27.95841406	0	7.13840198	4.92E-05	0.000227084	FLT3
ENSG00000154174	930.7115865	1612.443922	-0.793037536	4.92E-05	0.000227329	TOMM70
ENSG00000141985	1838.649939	3043.351635	-0.726997501	4.93E-05	0.000227486	SH3GL1
ENSG00000267886	0.894837599	19.65787502	-4.442191805	4.93E-05	0.000227683	AC074135.1
ENSG00000238227	657.5399621	1268.701685	-0.948358273	4.94E-05	0.000228117	TMEM250
ENSG00000123983	811.3358939	1697.60901	-1.065358041	4.95E-05	0.000228471	ACSL3
ENSG00000104689	70.19397075	190.0233427	-1.436682864	4.96E-05	0.000228915	TNFRSF10A
ENSG00000099308	523.0142468	239.2607407	1.128926962	4.97E-05	0.000229072	MAST3
ENSG00000161981	208.4187627	636.0189279	-1.610177125	4.97E-05	0.000229104	SNRNP25
ENSG00000250138	14.12878375	0	6.155249002	4.98E-05	0.000229591	AC139495.3
ENSG00000231672	22.63813893	0.743759607	4.970379659	4.98E-05	0.000229604	DIRC3
ENSG00000183876	581.2799926	147.2124616	1.982985661	4.98E-05	0.000229604	ARSI
novel.183	14.70265769	0	6.212840774	4.99E-05	0.000229848	-
ENSG00000235217	215.138913	100.5096562	1.099861221	5.00E-05	0.000230363	TSPY26P
ENSG00000205250	993.2278309	1727.150018	-0.798169133	5.01E-05	0.00023058	E2F4
ENSG00000120832	268.507664	128.378845	1.06318317	5.01E-05	0.00023078	MTERF2
ENSG00000159352	1935.952034	3262.524586	-0.752977918	5.02E-05	0.000231161	PSMD4
ENSG00000169251	564.5313717	1328.230322	-1.234619593	5.04E-05	0.000231871	NMD3
ENSG00000237949	32.81589285	0.713078025	5.517361016	5.04E-05	0.0002321	LINC00844
ENSG00000185669	65.47306946	12.08552747	2.445847933	5.05E-05	0.000232473	SNAI3
ENSG00000123119	247.8291114	62.6967898	1.98409134	5.05E-05	0.000232473	NECAB1
ENSG00000153214	432.6847826	885.826327	-1.033568662	5.06E-05	0.00023292	TMEM87B
novel.350	25.96805526	0.387220594	6.071731772	5.09E-05	0.000234085	-
ENSG00000187166	18.46818989	0	6.541975839	5.11E-05	0.000235113	H1FNT
ENSG00000135392	208.8636245	389.1803729	-0.89795082	5.12E-05	0.000235272	DNAJC14
ENSG00000155975	447.5349724	813.7081827	-0.862586766	5.12E-05	0.000235481	VPS37A
ENSG00000258810	21.73208941	0	6.774866532	5.12E-05	0.000235481	AL133371.2
ENSG00000229224	17.37588764	0	6.453673857	5.15E-05	0.000236529	AC105398.1
ENSG00000161048	39.65050977	7.378673051	2.411745582	5.16E-05	0.000236936	NAPEPLD
ENSG00000163817	445.8256107	0.713078025	9.283935313	5.16E-05	0.000237245	SLC6A20
ENSG00000225096	52.89408567	1.029478846	5.647488299	5.18E-05	0.000237834	AL445250.1
ENSG00000115216	1763.438022	3025.159518	-0.77868812	5.18E-05	0.000238008	NRBP1
ENSG00000280560	14.64373584	0	6.205025297	5.20E-05	0.00023865	LINC01374
ENSG00000232884	15.83063191	0	6.317818026	5.21E-05	0.000238962	AF127936.2

ENSG00000272425	35.90711632	0.672939833	5.672307625	5.21E-05	0.000239225	AC009902.3
ENSG00000184207	379.0017363	826.9653206	-1.125982006	5.23E-05	0.00024	PGP
ENSG00000266313	19.32681386	0.387220594	5.644881862	5.24E-05	0.000240614	AC026254.2
ENSG00000137463	137.3150093	10.14125216	3.752929553	5.25E-05	0.000240761	MGARP
novel.172	19.04206382	0	6.586127797	5.27E-05	0.000241569	-
ENSG00000174456	529.9946722	223.2058727	1.248191691	5.27E-05	0.000241739	C12orf76
ENSG00000264247	300.8387202	153.3634518	0.971017843	5.29E-05	0.000242724	LINC00909
ENSG00000168792	407.1021694	138.6598573	1.551775605	5.30E-05	0.000242724	ABHD15
ENSG00000278156	54.34654321	11.14937764	2.287494942	5.32E-05	0.000243892	TSC22D1-AS1
novel.886	1541.320403	0.774441189	10.98423274	5.33E-05	0.000244034	-
ENSG00000185633	645.5473303	73.83662928	3.126149625	5.36E-05	0.000245562	NDUFA4L2
ENSG00000114450	976.1713814	1819.249278	-0.89827387	5.37E-05	0.000246013	GNB4
ENSG00000149231	1311.300764	646.3726009	1.020892602	5.38E-05	0.000246177	CCDC82
ENSG00000169570	137.3055652	63.09256791	1.121167975	5.40E-05	0.00024711	DTWD2
ENSG00000247416	32.67158	1.782695063	4.18944839	5.40E-05	0.000247284	AP000802.1
ENSG00000103226	6.423860566	45.03792197	-2.815178054	5.40E-05	0.000247382	NOMO3
ENSG00000111752	729.484334	368.6133864	0.985203793	5.41E-05	0.000247429	PHC1
ENSG00000170396	18.450781	82.14344258	-2.153372954	5.41E-05	0.000247496	ZNF804A
ENSG00000136153	573.8669701	5116.007587	-3.156285165	5.43E-05	0.000248227	LMO7
ENSG00000165494	1299.665724	676.4327204	0.94206473	5.44E-05	0.00024877	PCF11
ENSG00000189350	30.59013107	1.386017859	4.431358048	5.45E-05	0.000249264	TOGARAM2
ENSG00000184441	98.97086455	23.37081246	2.078601511	5.46E-05	0.000249543	AP001062.1
ENSG00000229337	32.18846767	3.372871508	3.28669109	5.47E-05	0.000249876	AC079305.3
ENSG00000104064	174.6882308	334.8301622	-0.939097172	5.47E-05	0.000249969	GABPB1
ENSG00000132681	18.58277082	0	6.550391642	5.47E-05	0.0002502	ATP1A4
ENSG00000106415	298.9745537	97.85487143	1.610854179	5.48E-05	0.000250362	GLCCI1
ENSG00000124795	1643.790673	3159.781034	-0.942889231	5.49E-05	0.000250614	DEK
ENSG00000100453	13.31202873	0	6.068498791	5.50E-05	0.000251137	GZMB
ENSG00000286431	23.63376733	76.53317487	-1.693636678	5.51E-05	0.000251733	BX842570.1
ENSG00000286662	16.21323818	0	6.351831927	5.52E-05	0.000251826	AC025160.1
ENSG00000012232	1056.638968	1764.625699	-0.739794076	5.52E-05	0.000251897	EXTL3
ENSG00000184381	435.7903259	146.6348242	1.572236047	5.52E-05	0.000252149	PLA2G6
ENSG00000185482	137.7255675	29.99524181	2.19236917	5.53E-05	0.000252385	STAC3
ENSG00000176293	241.8399851	80.09090624	1.592303483	5.55E-05	0.000253311	ZNF135
ENSG00000060688	420.0009623	784.4060439	-0.901568231	5.56E-05	0.000253819	SNRNP40
ENSG00000205937	1802.145403	3279.654944	-0.863811052	5.57E-05	0.000253864	RNPS1
ENSG00000148154	1159.695679	3694.071135	-1.671354638	5.57E-05	0.000253954	UGCG
ENSG00000127191	299.6040429	603.3659222	-1.009735091	5.58E-05	0.000254244	TRAF2
ENSG00000138107	2662.68265	4463.010008	-0.745111503	5.61E-05	0.000255627	ACTR1A
ENSG00000104918	24.86253235	0	6.969035962	5.61E-05	0.000255673	RETN
ENSG00000114771	0	14.33114319	-6.425543032	5.62E-05	0.000256082	AADAC
novel.293	14.4195173	0	6.184651618	5.62E-05	0.000256166	-
ENSG00000171425	231.8317161	608.183465	-1.391111193	5.63E-05	0.000256463	ZNF581
ENSG00000152503	88.8289248	12.60377513	2.827027085	5.68E-05	0.000258732	TRIM36
ENSG00000236698	96.29165108	215.7732381	-1.163133983	5.70E-05	0.000259401	EIF1AXP1
ENSG00000260633	28.38955732	1.110911105	4.707728583	5.71E-05	0.000259834	AC010207.1
ENSG00000228824	220.5694832	13.57176241	4.029991025	5.73E-05	0.000260876	MIR4500HG
ENSG00000105928	385.1611469	1045.246225	-1.440131897	5.74E-05	0.000261323	GSDME
ENSG00000105880	20.23064047	0	6.671423842	5.75E-05	0.000261722	DLX5
ENSG00000099203	155.2070036	399.8308488	-1.365474221	5.75E-05	0.00026183	TMED1
ENSG00000106852	35.50369588	1.834601616	4.291453881	5.77E-05	0.000262428	LHX6

ENSG00000223704	26.19603475	2.079026788	3.624265706	5.79E-05	0.000263328	LINC01422
ENSG00000139546	343.9820775	740.2864804	-1.106092799	5.80E-05	0.000263736	TARBP2
ENSG00000181027	533.8890733	946.773008	-0.826657837	5.82E-05	0.000264849	FKRP
ENSG00000168421	52.33873173	4.799027558	3.460667207	5.85E-05	0.000266028	RHOH
ENSG00000143319	426.5741503	856.4312228	-1.00599032	5.86E-05	0.000266171	ISG20L2
ENSG00000233830	52.63809863	140.2160945	-1.414197408	5.89E-05	0.000267858	EIF4HP1
ENSG00000070366	314.3520451	678.8048212	-1.110687664	5.90E-05	0.000267858	SMG6
ENSG00000152582	425.3328369	119.3489707	1.835016712	5.92E-05	0.000269047	SPEF2
ENSG00000100281	360.2201481	743.5624009	-1.045115898	5.92E-05	0.000269069	HMGXB4
ENSG00000242299	2366.736945	4509.089598	-0.929941879	5.94E-05	0.000269907	AC073861.1
ENSG00000124143	0	11.90259386	-6.153504865	5.95E-05	0.000270185	ARHGAP40
ENSG00000122696	129.0771278	268.9324369	-1.059560244	5.96E-05	0.000270789	SLC25A51
ENSG00000237451	19.83263441	0.387220594	5.682797915	5.99E-05	0.000271999	CDK2AP2P2
ENSG00000005302	870.3433214	508.830684	0.774765892	6.00E-05	0.000272116	MSL3
ENSG00000245498	101.8293074	27.34349228	1.893850915	6.01E-05	0.000272476	AP000866.1
ENSG00000088205	1114.5871	1823.016387	-0.709761553	6.01E-05	0.000272755	DDX18
ENSG00000151360	34.1697662	0	7.427958544	6.03E-05	0.000273511	ALLC
ENSG00000231231	0	16.25175339	-6.600940262	6.04E-05	0.000273981	LINC01423
ENSG00000182379	12.4839196	71.85152663	-2.528021697	6.04E-05	0.000274082	NXPH4
ENSG00000236411	15.32445536	0	6.272312418	6.05E-05	0.000274144	NDUFAF4P3
ENSG00000267074	15.57381815	0	6.293905662	6.08E-05	0.000275375	AC015911.3
ENSG00000167186	435.4185165	230.6879733	0.915689918	6.08E-05	0.00027555	COQ7
ENSG00000267414	30.94508579	0.743759607	5.414916923	6.10E-05	0.000276225	AC120049.1
ENSG00000129351	4480.700998	8058.110055	-0.846706279	6.10E-05	0.000276237	ILF3
ENSG00000235387	62.75492161	8.102363562	2.94456573	6.10E-05	0.000276323	SPAAR
ENSG00000281005	71.86684245	10.01968171	2.827512247	6.11E-05	0.000276909	LINC00921
ENSG00000180549	13.70606037	0	6.109684322	6.14E-05	0.000277993	FUT7
ENSG00000254952	13.70606037	0	6.109684322	6.14E-05	0.000277993	AP001257.1
ENSG00000140832	36.45161435	1.783850939	4.348204543	6.14E-05	0.000278089	MARVELD3
ENSG00000175866	1111.961569	411.1076228	1.435058122	6.14E-05	0.000278089	BAIAP2
ENSG00000288075	63.32013471	11.7314286	2.429128151	6.15E-05	0.000278122	AC108519.1
ENSG00000003756	3315.970472	1547.357763	1.099701269	6.19E-05	0.000280173	RBM5
ENSG00000237596	2.447971227	34.17627135	-3.806990138	6.19E-05	0.000280189	AL138828.1
ENSG00000259956	983.7638183	1695.758612	-0.785688413	6.20E-05	0.000280318	RBM15B
ENSG00000163815	498.273154	20.71345912	4.585204889	6.22E-05	0.000281147	CLEC3B
ENSG00000170439	79.23632766	5.199300783	3.916555338	6.25E-05	0.000282594	METTL7B
ENSG00000105784	44.42115792	2.231278821	4.337677274	6.25E-05	0.000282699	RUNDC3B
ENSG00000186493	36.87587611	0.387220594	6.576008644	6.26E-05	0.000282824	C5orf38
ENSG00000178307	388.0067403	703.762788	-0.859177161	6.26E-05	0.000282942	TMEM11
ENSG00000077514	266.0651347	502.9506562	-0.91859293	6.27E-05	0.000283287	POLD3
novel.567	26.75912405	0	7.075097045	6.28E-05	0.000283746	-
ENSG00000129255	769.8501947	1401.456497	-0.864378897	6.30E-05	0.000284431	MPDU1
ENSG00000166523	23.02367977	0	6.858105634	6.31E-05	0.000284982	CLEC4E
ENSG00000079102	175.3893921	30.67635399	2.511996191	6.32E-05	0.000285135	RUNX1T1
ENSG00000160185	39.90649681	0.774441189	5.759034121	6.33E-05	0.0002856	UBASH3A
ENSG00000103266	893.9405747	1624.348491	-0.861692945	6.33E-05	0.0002856	STUB1
ENSG00000117707	30.09057876	0.356539013	6.282427071	6.33E-05	0.000285695	PROX1
ENSG00000183784	14.32785805	0	6.173666801	6.35E-05	0.000286323	C9orf66
ENSG00000128652	0.308355904	22.9223571	-6.141221258	6.35E-05	0.000286323	HOXD3
ENSG00000114796	1332.285049	393.0316794	1.761828921	6.35E-05	0.000286323	KLHL24
ENSG00000124217	132.7427669	260.9427007	-0.9754164	6.36E-05	0.00028695	MOCS3

ENSG00000009694	13.98927203	0	6.139428256	6.38E-05	0.000287457	TENM1
ENSG00000149547	1347.782348	2574.921681	-0.934079989	6.39E-05	0.000287805	EI24
ENSG00000006638	295.7350426	121.1272178	1.289289995	6.39E-05	0.000288179	TBXA2R
ENSG00000169118	251.3404526	460.6279831	-0.873638333	6.40E-05	0.0002884	CSNK1G1
ENSG00000124562	866.7033964	1733.207097	-1.000015588	6.41E-05	0.000288721	SNRPC
ENSG00000007174	13.04024245	0	6.038398409	6.42E-05	0.000289184	DNAH9
ENSG00000278376	40.35540462	8.856735655	2.178919237	6.43E-05	0.000289365	AP004609.3
ENSG00000184545	75.08304766	2.975038428	4.673787659	6.43E-05	0.000289527	DUSP8
ENSG00000204403	23.5152829	1.365948763	4.056332267	6.44E-05	0.000289839	CASP12
ENSG00000110429	941.7255287	482.1787064	0.965650859	6.45E-05	0.000290436	FBXO3
ENSG00000144909	570.9041761	331.1246264	0.785451791	6.45E-05	0.000290436	OSBPL11
ENSG00000286904	14.02827769	0	6.143099656	6.46E-05	0.000290828	AC093675.1
ENSG00000140015	25.16613056	0	6.988092937	6.48E-05	0.00029139	KCNH5
ENSG00000286125	14.95717761	0	6.235611115	6.48E-05	0.000291476	AC006115.2
ENSG00000197044	258.2873288	101.0579904	1.351741248	6.49E-05	0.000291701	ZNF441
ENSG00000154719	238.3890064	557.030782	-1.22420566	6.49E-05	0.000291701	MRPL39
ENSG00000215612	20.8814146	0	6.717063045	6.53E-05	0.000293676	HMX1
ENSG00000229847	47.68964477	0.693008929	6.07171329	6.55E-05	0.000294267	EMX2OS
ENSG00000126062	784.2837584	1374.280993	-0.809305039	6.55E-05	0.000294417	TMEM115
novel.712	26.85433078	75.66797321	-1.49213452	6.57E-05	0.00029542	-
ENSG00000213866	90.1826994	202.3163677	-1.166164709	6.58E-05	0.000295516	YBX1P10
ENSG00000129103	1372.549803	2486.695478	-0.857491836	6.58E-05	0.000295582	SUMF2
ENSG00000163072	32.38614578	3.271370153	3.315954572	6.63E-05	0.00029758	NOSTRIN
ENSG00000127990	2409.147986	1118.94883	1.106215309	6.63E-05	0.00029758	SGCE
ENSG00000251127	0	14.85743479	-6.470383653	6.65E-05	0.000298598	AC091173.1
ENSG00000228522	39.6524476	1.783850939	4.471407218	6.65E-05	0.000298598	AL845321.1
ENSG00000258311	47.79445376	10.38439306	2.202158339	6.66E-05	0.000298974	AC009779.3
ENSG00000036530	40.8660263	6.195657903	2.731850901	6.67E-05	0.000299108	CYP46A1
ENSG00000254639	0.925067711	22.38031593	-4.616186576	6.67E-05	0.000299279	AC116021.1
ENSG00000225792	13.03390294	0	6.037765798	6.72E-05	0.000301329	AC004540.2
ENSG00000171130	861.0930277	190.608617	2.174343547	6.73E-05	0.000301628	ATP6V0E2
ENSG00000165495	354.8436556	62.18290853	2.509662866	6.75E-05	0.00030286	PKNOX2
novel.1001	1217.570154	0	12.58338386	6.78E-05	0.000303942	-
ENSG00000279302	16.18906285	0	6.351604095	6.79E-05	0.000304295	AC013643.3
ENSG00000287287	1.81990531	25.44727071	-3.805218914	6.80E-05	0.000304632	AL133476.1
ENSG00000271653	12.71927879	0	6.002645096	6.80E-05	0.000304655	AC097359.3
ENSG00000142149	21.55210493	0.743759607	4.898361897	6.82E-05	0.000305744	HUNK
novel.248	16.13633798	0	6.345455894	6.85E-05	0.000306704	-
ENSG00000279955	17.19214186	0	6.4377256	6.85E-05	0.000306965	AC129778.1
ENSG00000100442	660.2838953	1360.488437	-1.04320117	6.87E-05	0.000307668	FKBP3
ENSG00000220804	42.2127772	8.836666559	2.247346368	6.90E-05	0.000308706	LINC01881
ENSG00000152749	383.8766236	719.5723661	-0.906164515	6.91E-05	0.000309433	GPR180
ENSG00000133624	561.6703109	252.4481079	1.153974826	6.92E-05	0.000309798	ZNF767P
ENSG00000198944	17.77020402	0	6.484212825	6.93E-05	0.000310169	SOWAHA
ENSG00000119899	405.5372109	860.9075312	-1.085882771	6.94E-05	0.000310367	SLC17A5
ENSG00000037042	812.9639787	412.5030848	0.979499788	6.96E-05	0.000311281	TUBG2
novel.17	42.30701502	6.084699937	2.797299347	6.96E-05	0.000311459	-
novel.625	3.342808826	40.39418171	-3.593638447	6.96E-05	0.000311459	-
ENSG00000188033	100.2752758	40.68401055	1.298821462	6.98E-05	0.000311944	ZNF490
ENSG00000121064	1701.983887	714.3531718	1.252478129	6.98E-05	0.000312034	SCPEP1
ENSG00000065357	418.395076	974.1622985	-1.218965606	7.00E-05	0.00031279	DGKA

ENSG00000137207	1448.437452	2722.909202	-0.910576593	7.01E-05	0.000313051	YIPF3
ENSG00000168917	115.3216766	371.7350044	-1.689305158	7.02E-05	0.000313446	SLC35G2
ENSG00000185304	6.425185486	63.72634986	-3.313055252	7.02E-05	0.000313802	RGPD2
ENSG00000158246	38.1581211	134.7609041	-1.822408646	7.05E-05	0.000314744	TENT5B
ENSG00000228476	12.98228952	0	6.03261447	7.07E-05	0.000315527	CSTP1
ENSG00000134369	896.1950238	2866.397588	-1.677481791	7.08E-05	0.00031597	NAV1
ENSG00000188647	2125.489936	886.5688489	1.261530548	7.08E-05	0.00031597	PTAR1
ENSG00000215105	112.0852861	36.05383649	1.634981033	7.09E-05	0.000316339	TTC3P1
ENSG00000231826	13.05410386	0	6.039789003	7.11E-05	0.000317086	LINC01819
ENSG00000183431	1069.352592	2075.659925	-0.956983256	7.11E-05	0.000317209	SF3A3
ENSG00000118156	29.35907256	3.515795324	3.045414228	7.12E-05	0.000317444	ZNF541
ENSG00000265817	171.4095009	72.46767962	1.240424428	7.12E-05	0.00031759	FSBP
ENSG00000287725	137.4317575	10.59672399	3.708635953	7.15E-05	0.000318666	AP003071.5
ENSG00000102699	1105.874225	1809.836456	-0.710708227	7.15E-05	0.000318907	PARP4
ENSG00000225151	8.27148869	53.30554302	-2.692672823	7.16E-05	0.000319209	GOLGA2P7
ENSG00000140474	1284.692735	494.494335	1.377068269	7.16E-05	0.000319231	ULK3
ENSG00000266200	20.57431235	0	6.695683108	7.17E-05	0.000319324	PNLIPRP2
ENSG00000182993	170.3511341	37.18828432	2.195427087	7.18E-05	0.000319814	C12orf60
ENSG00000228626	0	11.87306815	-6.150297528	7.18E-05	0.000319861	AC245100.3
ENSG00000168876	294.3882476	130.0953438	1.176820557	7.19E-05	0.000320403	ANKRD49
ENSG00000007944	457.2816102	116.8863662	1.9664074	7.20E-05	0.000320453	MYLIP
ENSG00000100629	94.77934623	210.259206	-1.150747766	7.23E-05	0.000321875	CEP128
ENSG00000018189	1551.83215	771.1343248	1.009066864	7.23E-05	0.000321877	RUFY3
ENSG00000185651	1664.744288	2748.438396	-0.723283491	7.27E-05	0.000323376	UBE2L3
ENSG00000253955	25.75038969	1.130980201	4.558969182	7.27E-05	0.000323417	AC008663.2
ENSG00000169660	641.602899	270.3481904	1.24788603	7.29E-05	0.000324216	HEXD
ENSG00000121851	515.7042651	257.3311834	1.002253858	7.30E-05	0.000324628	POLR3GL
ENSG00000137747	13.08684127	0	6.043056974	7.30E-05	0.000324628	TMPRSS13
ENSG00000250990	20.26950393	0.693008929	4.827615995	7.31E-05	0.000324953	AC098851.1
ENSG00000260871	17.89836163	0.336469917	5.53336428	7.33E-05	0.00032579	AC093510.2
ENSG00000100109	294.772436	563.7272682	-0.935367792	7.34E-05	0.000326132	TFIP11
ENSG00000179603	34.72440813	2.567748738	3.7724375	7.36E-05	0.000327178	GRM8
ENSG00000147180	226.7209877	58.26388625	1.960921536	7.37E-05	0.000327391	ZNF711
ENSG00000225905	22.57384649	1.753169357	3.665061997	7.39E-05	0.000328376	AL391244.1
ENSG00000007129	18.64853037	0	6.554051686	7.39E-05	0.000328424	CEACAM21
ENSG00000143545	581.2991252	1241.493223	-1.094446534	7.40E-05	0.000328786	RAB13
ENSG00000006327	2393.475157	6283.114758	-1.392336458	7.42E-05	0.000329452	TNFRSF12A
ENSG00000240376	385.5465173	702.8227378	-0.866652658	7.42E-05	0.000329463	AC010343.1
ENSG00000272686	104.1155569	39.96977664	1.378492959	7.46E-05	0.00033101	AC006333.2
ENSG00000183092	113.391307	17.65339242	2.673995758	7.46E-05	0.000331128	BEGAIN
ENSG00000151778	195.4978032	86.65217458	1.174713927	7.46E-05	0.000331135	SERP2
ENSG00000265763	100.2389478	10.59813665	3.236571007	7.46E-05	0.000331135	ZNF488
ENSG00000109787	1045.578415	591.0689151	0.822475386	7.48E-05	0.000331803	KLF3
ENSG00000242193	288.5057808	103.7828714	1.472755131	7.50E-05	0.000332575	CRYZL2P
novel.300	25.83781728	0	7.024535348	7.55E-05	0.000335014	-
ENSG00000137261	23.86869924	0.356539013	5.950223118	7.57E-05	0.000335488	KIAA0319
ENSG00000160014	4373.437916	8056.81502	-0.881475246	7.57E-05	0.000335585	CALM3
ENSG00000106483	7334.442203	51.35352773	7.157413132	7.57E-05	0.00033571	SFRP4
ENSG00000149480	1445.332099	2445.023737	-0.758479989	7.58E-05	0.000336075	MTA2
ENSG00000198754	56.67875112	7.194455167	2.979459767	7.59E-05	0.000336144	OXCT2
ENSG00000285847	20.92278536	1.029478846	4.295391841	7.59E-05	0.000336248	AL713852.1

ENSG00000182327	51.9859283	8.752794155	2.57771894	7.61E-05	0.000337198	GLTPD2
ENSG00000126522	231.3510836	524.1500251	-1.179644959	7.63E-05	0.000337899	ASL
ENSG00000134278	832.5364667	375.0716592	1.150354313	7.63E-05	0.000337948	SPIRE1
ENSG00000134686	3399.222768	7538.168659	-1.148960359	7.67E-05	0.000339424	PHC2
ENSG00000103241	36.97978711	387.9018204	-3.390614975	7.69E-05	0.000340428	FOXF1
ENSG00000251034	82.51216672	18.25063219	2.176194847	7.69E-05	0.000340565	AC087854.1
ENSG00000157350	632.5555408	1433.872964	-1.18053061	7.70E-05	0.000340915	ST3GAL2
ENSG00000287356	62.4243282	18.43587755	1.764096409	7.71E-05	0.000341148	AL590822.3
ENSG00000073331	658.4861322	249.3696887	1.400508227	7.71E-05	0.000341201	ALPK1
ENSG00000160753	737.7468326	1236.799703	-0.745506927	7.71E-05	0.000341201	RUSC1
ENSG00000177981	556.2593291	288.8553669	0.944460245	7.73E-05	0.000341998	ASB8
ENSG00000147155	134.3422859	286.1549217	-1.09072254	7.74E-05	0.000342183	EBP
ENSG00000129474	541.9833265	1165.359503	-1.104298743	7.74E-05	0.000342253	AJUBA
ENSG00000090238	1619.353626	592.357435	1.451383572	7.75E-05	0.000342688	YPEL3
ENSG00000273186	5.700089716	39.31220119	-2.782770711	7.76E-05	0.000343107	AL359091.5
ENSG00000221829	240.6438716	615.1876443	-1.354391922	7.78E-05	0.000343952	FANCG
ENSG00000143390	1198.351779	746.3416415	0.682984076	7.80E-05	0.000344393	RFX5
ENSG00000270681	21.99370431	1.060160428	4.359357876	7.81E-05	0.000345046	AC095055.1
ENSG00000115266	609.2600655	143.0671223	2.090860319	7.85E-05	0.000346618	APC2
ENSG00000165197	18.75168628	0.387220594	5.601047049	7.86E-05	0.000346869	VEGFD
ENSG00000175426	11.47074012	708.694502	-5.948986773	7.87E-05	0.000347311	PCSK1
ENSG00000197958	10427.68364	16700.17534	-0.679442906	7.87E-05	0.000347473	RPL12
ENSG00000171551	24.24797184	1.069617038	4.494891509	7.87E-05	0.00034751	ECEL1
ENSG00000213398	692.1300473	295.066646	1.229311207	7.89E-05	0.000348098	LCAT
ENSG00000286962	17.78524781	0	6.485397833	7.90E-05	0.000348332	AC027128.1
ENSG00000115128	888.8379496	1820.387242	-1.034317109	7.90E-05	0.000348476	SF3B6
ENSG00000203650	12.4436603	0	5.9707059	7.91E-05	0.00034873	LINC01285
ENSG00000174482	12.4436603	0	5.9707059	7.91E-05	0.00034873	LINGO2
ENSG00000167112	436.1711491	783.9564326	-0.846255822	7.93E-05	0.000349752	TRUB2
ENSG00000238078	26.73717128	1.161661783	4.598978377	7.94E-05	0.000350106	LINC01352
ENSG00000163479	1851.013077	3716.366094	-1.005603511	7.97E-05	0.000351393	SSR2
ENSG00000162430	2134.526529	3636.766087	-0.768679743	7.97E-05	0.000351393	SELENON
ENSG00000125378	224.5971389	18.41722112	3.603615906	7.98E-05	0.000351803	BMP4
ENSG00000235023	18.13873542	0.336469917	5.553181735	7.99E-05	0.000352109	AP001626.1
ENSG00000185420	170.30291	317.150793	-0.896634089	8.00E-05	0.000352125	SMYD3
ENSG00000174839	803.4223539	391.8806429	1.036533047	8.01E-05	0.000352667	DENND6A
ENSG00000164941	402.7111502	725.8877491	-0.849937168	8.01E-05	0.000352667	INTS8
ENSG00000230707	23.96483145	0.774441189	5.036658354	8.04E-05	0.000353942	AL589987.1
ENSG00000005156	281.2672021	546.5022145	-0.958603811	8.04E-05	0.000353942	LIG3
ENSG00000147650	405.6229301	906.3356147	-1.159576313	8.05E-05	0.000354088	LRP12
ENSG00000142867	259.6031654	531.8833787	-1.034870133	8.06E-05	0.000354738	BCL10
ENSG00000124787	57.01525677	152.936397	-1.425473637	8.07E-05	0.000354923	RPP40
ENSG00000173581	668.2645948	378.959919	0.818532105	8.10E-05	0.000356287	CCDC106
ENSG00000102054	1710.940674	2828.966935	-0.725537479	8.10E-05	0.000356302	RBBP7
ENSG00000053372	633.9382365	1574.809405	-1.312883084	8.11E-05	0.000356497	MRTO4
ENSG00000135018	1734.273805	3132.317392	-0.852976128	8.11E-05	0.000356649	UBQLN1
ENSG00000146856	114.3747983	20.48910305	2.472856646	8.13E-05	0.00035742	AGBL3
ENSG00000135315	312.1633678	125.6423711	1.31151412	8.14E-05	0.000357763	CEP162
ENSG00000137070	434.7084215	222.5684475	0.966572928	8.15E-05	0.000358084	IL11RA
ENSG00000281162	0.601596751	28.67246651	-5.565506079	8.15E-05	0.000358084	LINC01127
ENSG00000103510	932.9072326	526.5685911	0.825519126	8.16E-05	0.000358633	KAT8

ENSG00000134970	1366.987173	2317.462528	-0.761545589	8.17E-05	0.000358929	TMED7
ENSG00000272288	58.06479241	17.92477476	1.695145833	8.20E-05	0.000360271	AL451165.2
ENSG00000251192	109.1415948	42.02745007	1.377765395	8.23E-05	0.000361102	ZNF674
ENSG00000130222	53.0291957	7.836585025	2.777304447	8.23E-05	0.000361112	GADD45G
ENSG00000197540	69.42431275	3.465044647	4.308124398	8.24E-05	0.000361414	GZMM
ENSG00000228408	15.61038778	0	6.297141476	8.25E-05	0.00036189	AL031056.1
ENSG00000171017	0.293240848	16.38637647	-5.651106541	8.25E-05	0.00036189	LRRC8E
ENSG00000245146	69.74205745	23.82769695	1.544407343	8.26E-05	0.000362074	MALINC1
ENSG00000156599	1422.193438	2645.111616	-0.895289739	8.26E-05	0.000362074	ZDHHC5
ENSG00000227392	23.28264482	0.336469917	5.913242617	8.28E-05	0.000363115	HPN-AS1
ENSG00000100258	1807.299712	3612.777319	-0.999275787	8.29E-05	0.000363546	LMF2
ENSG00000170043	874.2201992	1643.669532	-0.910755189	8.33E-05	0.000365355	TRAPPC1
ENSG00000187244	662.9400011	314.5866402	1.075337282	8.35E-05	0.000365992	BCAM
ENSG00000127951	2857.116311	38.46732541	6.21373624	8.37E-05	0.000366571	FGL2
ENSG00000089692	53.39077467	10.04907902	2.408901532	8.37E-05	0.000366571	LAG3
ENSG00000135324	46.14550049	0.356539013	6.899695986	8.39E-05	0.000367574	MRAP2
ENSG00000237190	331.7813696	776.6099138	-1.227435166	8.40E-05	0.00036787	CDKN2AIPNL
ENSG00000287527	13.79395868	0	6.120861367	8.41E-05	0.000368206	AC005753.3
novel.306	24.2206763	0	6.931375534	8.43E-05	0.000369032	-
ENSG00000125755	1347.37299	2386.40332	-0.824544414	8.44E-05	0.000369496	SYMPK
ENSG00000112367	393.8022502	218.5279017	0.84904365	8.45E-05	0.000369914	FIG4
ENSG00000162772	1096.636756	200.1176502	2.454768901	8.46E-05	0.000370398	ATF3
ENSG00000082153	2698.786463	6579.008716	-1.2856123	8.48E-05	0.000370909	BZW1
ENSG00000128951	929.0239127	2013.430307	-1.11605833	8.49E-05	0.000371268	DUT
ENSG00000165644	356.5039076	154.0833367	1.210268844	8.49E-05	0.000371297	COMTD1
novel.351	20.77754704	0	6.711794165	8.50E-05	0.000371591	-
ENSG00000230457	17.85301646	62.27225634	-1.804838786	8.50E-05	0.000371612	PA2G4P4
ENSG00000272541	21.87529115	0.336469917	5.824187182	8.53E-05	0.000372736	AL021368.3
ENSG00000010165	500.5195349	972.6200384	-0.958747268	8.55E-05	0.000373608	EEF1AKNMT
ENSG00000177042	597.8284268	347.161352	0.784257949	8.56E-05	0.000373948	TMEM80
ENSG00000119844	679.1806499	337.9470026	1.007079601	8.57E-05	0.000374293	AFTPH
ENSG00000125863	465.8219954	230.2525706	1.015335171	8.57E-05	0.000374304	MKKS
novel.1081	58.67398233	5.800264968	3.315816783	8.57E-05	0.0003744	-
ENSG00000140259	431.4136348	966.6245922	-1.164031609	8.59E-05	0.000375324	MFAP1
ENSG00000169967	1311.795954	778.6411521	0.752512514	8.61E-05	0.000375994	MAP3K2
ENSG00000286271	23.0926308	1.722487775	3.704989669	8.63E-05	0.000376908	AC008945.2
ENSG00000135905	1135.099241	457.917837	1.309102742	8.64E-05	0.000377109	DOCK10
ENSG00000276728	56.31509178	15.36879438	1.872303174	8.67E-05	0.000378526	AC142472.1
ENSG00000100316	25587.30968	42913.77445	-0.746013711	8.68E-05	0.000378637	RPL3
ENSG00000145016	350.7875205	130.0047929	1.430902529	8.68E-05	0.000378637	RUBCN
ENSG00000111816	47.72983315	5.533330555	3.122303982	8.69E-05	0.000378868	FRK
ENSG00000234520	0	14.1030627	-6.395192165	8.69E-05	0.000379194	AC018464.1
ENSG00000132716	1469.243197	714.3468789	1.040573543	8.72E-05	0.000380314	DCAF8
ENSG00000255491	34.02968501	5.502648973	2.64321135	8.74E-05	0.000381193	AC100858.3
ENSG00000129116	7453.928958	2672.320861	1.47982351	8.75E-05	0.000381218	PALLD
ENSG00000181396	453.3149675	1018.222066	-1.167504668	8.75E-05	0.000381268	OGFOD3
ENSG00000109618	326.1658879	171.6931233	0.924667402	8.77E-05	0.0003822	SEPSECS
ENSG00000175556	47.57220053	5.349241065	3.148313737	8.78E-05	0.000382409	LONRF3
ENSG00000099840	280.7117769	75.44446904	1.898610515	8.78E-05	0.00038243	IZUMO4
ENSG00000070756	9758.704586	16653.65244	-0.771084735	8.80E-05	0.000383319	PABPC1
ENSG00000225828	204.8859555	66.83960739	1.617664948	8.81E-05	0.000383848	FAM229A

ENSG00000101290	1312.998407	680.0160309	0.949700712	8.83E-05	0.000384424	CDS2
ENSG00000157873	152.3595158	26.60533647	2.522836636	8.87E-05	0.000385974	TNFRSF14
ENSG00000122691	131.5250553	1020.070957	-2.955602458	8.90E-05	0.000387218	TWIST1
ENSG00000173638	209.1887485	667.1175504	-1.673397776	8.93E-05	0.000388575	SLC19A1
ENSG00000230510	40.2397835	7.245205845	2.478189464	8.95E-05	0.000389271	PPP5D1
ENSG00000104331	1738.78113	3339.820985	-0.941656642	8.95E-05	0.000389496	IMPAD1
ENSG00000152990	387.4904202	870.2427619	-1.167018265	8.96E-05	0.00038986	ADGRA3
ENSG00000077274	131.0070546	0	9.367145775	8.98E-05	0.00039038	CAPN6
ENSG00000115368	642.0614376	1149.009356	-0.839730925	9.00E-05	0.000391146	WDR75
ENSG00000132141	56.55693268	17.52925343	1.688404849	9.08E-05	0.000394769	CCT6B
novel.1014	6.684364003	52.31337704	-2.967035299	9.10E-05	0.00039553	-
ENSG00000176438	1209.645672	604.7807763	1.000381362	9.10E-05	0.000395684	SYNE3
ENSG00000102468	19.99144941	0.356539013	5.693205877	9.14E-05	0.000397255	HTR2A
ENSG00000284652	18.94281141	0.387220594	5.616447867	9.15E-05	0.000397618	BX323043.1
ENSG00000260947	24.71750749	0.713078025	5.106397091	9.16E-05	0.00039778	AL356489.2
ENSG00000108557	563.1275807	1212.872731	-1.106683093	9.18E-05	0.00039855	RAI1
ENSG00000206432	27.20692075	0.743759607	5.2306882	9.18E-05	0.000398815	TMEM200C
ENSG00000166257	33.17016339	5.676254372	2.537540084	9.21E-05	0.000399725	SCN3B
ENSG00000261210	12.09121287	0	5.929869721	9.25E-05	0.000401523	CLEC19A
ENSG00000249577	12.09121287	0	5.929869721	9.25E-05	0.000401523	AC010424.1
ENSG00000063046	4310.657848	9000.838696	-1.062145198	9.35E-05	0.000405653	EIF4B
ENSG00000286747	19.07766454	1.080229524	4.14615005	9.38E-05	0.000407012	AL627095.2
ENSG00000143549	5564.708879	10502.47808	-0.916389098	9.40E-05	0.000407733	TPM3
ENSG00000127920	1129.808421	4049.168464	-1.841588881	9.42E-05	0.000408419	GNG11
ENSG00000107242	23.15559832	0.774441189	4.986607743	9.44E-05	0.000409249	PIP5K1B
ENSG00000205683	18.2520627	59.70895553	-1.71160708	9.44E-05	0.000409263	DPF3
ENSG00000187951	12.68020186	45.97047578	-1.854492221	9.44E-05	0.000409263	AC091057.1
ENSG00000129473	2501.424145	1214.743164	1.042276151	9.47E-05	0.000410373	BCL2L2
ENSG00000150551	114.1871494	11.74101361	3.272292234	9.47E-05	0.000410516	LYPD1
ENSG00000163631	24.33837744	0.713078025	5.085737848	9.48E-05	0.000410933	ALB
ENSG00000106701	279.0886528	109.9095889	1.344674452	9.50E-05	0.000411425	FSD1L
ENSG00000246922	233.5220839	66.17141945	1.819972948	9.54E-05	0.000413044	UBAP1L
ENSG00000165637	1716.237114	3254.485127	-0.923234992	9.54E-05	0.000413202	VDAC2
ENSG00000267577	1.218308559	32.61780397	-4.744316518	9.56E-05	0.000414081	AC010327.4
ENSG00000227582	54.68692451	2.099095884	4.688607508	9.57E-05	0.000414348	ADGRF5P1
ENSG00000152253	0	22.44831038	-7.066783778	9.58E-05	0.000414875	SPC25
ENSG00000116731	962.1623507	564.8485218	0.768501027	9.60E-05	0.000415466	PRDM2
ENSG00000124641	216.9199552	439.3642421	-1.018920635	9.61E-05	0.000416022	MED20
ENSG00000132669	897.6848665	1551.135415	-0.789196574	9.67E-05	0.000418578	RIN2
ENSG00000143816	105.4491577	8.509653252	3.627649232	9.68E-05	0.000418826	WNT9A
ENSG00000127952	280.6419561	685.6808212	-1.288734843	9.71E-05	0.000420038	STYXL1
ENSG00000277449	104.7189489	16.55424967	2.650970485	9.75E-05	0.000421836	CEBPB-AS1
ENSG00000260398	47.49619832	5.574624622	3.109748818	9.83E-05	0.000424926	AC068700.1
ENSG00000214192	23.24349663	70.71181333	-1.601828946	9.83E-05	0.000424926	UBE2V1P2
ENSG00000205763	208.8832563	88.01097848	1.247456807	9.83E-05	0.000425027	RP9P
ENSG00000164430	329.5795425	145.365077	1.179467006	9.85E-05	0.000425587	CGAS
ENSG00000152465	499.1297045	834.3675048	-0.741221114	9.89E-05	0.000427388	NMT2
ENSG00000285748	20.79581013	0.336469917	5.749118136	9.90E-05	0.000427516	AC090337.2
ENSG00000171385	27.9556933	2.466247382	3.487204032	9.90E-05	0.000427516	KCND3
ENSG00000168259	1062.076732	1760.677657	-0.729256353	9.91E-05	0.000427789	DNAJC7
novel.42	13.77884362	0	6.119358083	9.92E-05	0.000428532	-

ENSG00000259820	168.5318261	52.3147897	1.691815955	9.94E-05	0.00042903	AC083843.3
ENSG00000161277	401.487115	183.7117654	1.129328308	9.96E-05	0.000429797	THAP8
ENSG00000079739	731.2169383	1906.289746	-1.382259129	9.97E-05	0.000430412	PGM1
ENSG00000168491	5.102253911	31.38699187	-2.61672166	1.00E-04	0.00043146	CCDC110
ENSG00000126953	82.54310849	213.6441498	-1.373199654	0.000100091	0.000431828	TIMM8A
ENSG00000071462	938.4598614	1724.753448	-0.878045248	0.00010044	0.000433263	BUD23
ENSG00000103591	416.2733956	875.9032104	-1.073663743	0.000100558	0.000433699	AAGAB
ENSG00000117560	27.94720249	0.336469917	6.17578918	0.000100607	0.000433839	FASLG
ENSG00000166716	1176.000044	707.4502493	0.733411051	0.00010064	0.000433909	ZNF592
ENSG00000275963	23.42333892	0.774441189	5.004062156	0.000100848	0.000434667	AC008115.4
ENSG00000142224	0.308355904	37.72017972	-6.85501481	0.000100849	0.000434667	IL19
ENSG00000100065	720.5289211	313.1754159	1.201821976	0.000100915	0.000434877	CARD10
ENSG00000137817	1376.360843	563.8109181	1.28783219	0.000101045	0.000435366	PARP6
ENSG00000082898	2015.301645	3633.557003	-0.850487656	0.00010115	0.000435746	XPO1
ENSG00000285852	17.95882186	0.356539013	5.537775798	0.000101687	0.000437985	AL353147.1
ENSG00000228395	72.33448407	12.16007166	2.560946124	0.00010175	0.000438183	AL356481.1
ENSG00000233038	19.5421856	0.356539013	5.659448319	0.000102065	0.00043947	AC011899.2
ENSG00000144278	12.14791215	0	5.935919463	0.000102258	0.000440228	GALNT13
ENSG00000065675	32.6605819	3.862877727	3.063614462	0.000102305	0.000440357	PRKCQ
ENSG00000136826	914.0504079	109.6284117	3.05866278	0.000103021	0.000443363	KLF4
ENSG00000124203	29.86030544	0	7.23341639	0.000103131	0.000443765	ZNF831
ENSG00000174600	517.7679015	35.80740354	3.851367899	0.000103539	0.000445391	CMKLR1
ENSG00000108405	55.91543228	4.821536799	3.516093473	0.000103544	0.000445391	P2RX1
novel.1118	14.68406642	0	6.208879024	0.000103566	0.000445415	-
ENSG00000139687	1947.963623	1009.594823	0.948076208	0.000103678	0.000445822	RB1
ENSG00000227191	27.99387258	0	7.140269509	0.000103704	0.000445858	TRGC2
ENSG00000228797	7.90517986	40.08176209	-2.343236884	0.000103752	0.000445949	FAM207BP
ENSG00000215568	17.84681949	0	6.491398653	0.000103769	0.000445949	GAB4
ENSG00000278867	60.39915162	7.417526974	3.039439086	0.000103776	0.000445949	AC090616.6
ENSG00000196263	398.5775295	157.5125155	1.3408062	0.000103815	0.000446043	ZNF471
ENSG00000158669	1150.862134	1833.74449	-0.672088471	0.00010384	0.000446075	GPAT4
ENSG00000131116	1433.201283	725.2297204	0.982770012	0.000103936	0.000446416	ZNF428
ENSG00000110958	2777.168159	5842.507431	-1.073012306	0.000104141	0.000447223	PTGES3
ENSG00000112276	700.8961999	287.8832572	1.284731428	0.000104329	0.000447956	BVES
ENSG00000101557	1155.035888	2037.71931	-0.819135945	0.000104554	0.000448846	USP14
ENSG00000117009	49.75228897	6.1460631	3.020500371	0.000104614	0.000449031	KMO
ENSG00000135333	287.4555429	18.30151126	3.970928082	0.00010481	0.000449796	EPHA7
ENSG00000145949	61.17607426	21.41220025	1.511275943	0.000105295	0.000451805	MYLK4
ENSG00000015676	1396.336845	2181.686739	-0.64371822	0.00010547	0.000452414	NUDCD3
ENSG00000117133	372.9352227	792.1859364	-1.08726888	0.000105472	0.000452414	RPF1
ENSG00000142657	1198.675218	2737.777835	-1.191656917	0.00010568	0.000453194	PGD
ENSG00000110583	384.3334365	716.5413115	-0.898611168	0.000105689	0.000453194	NAA40
ENSG00000215845	243.709155	30.48524803	2.994996883	0.000105795	0.000453575	TSTD1
ENSG00000229739	58.77531476	8.397539412	2.796654832	0.000106083	0.000454734	AL096803.2
ENSG00000238129	19.00932641	0	6.583561088	0.000106314	0.00045565	AL121904.1
ENSG00000114767	299.3628146	712.3560693	-1.251051889	0.000106337	0.000455671	RRP9
ENSG00000145220	285.7096491	709.1475562	-1.312089706	0.00010646	0.000456126	LYAR
ENSG00000198721	552.9934639	1034.457779	-0.903223758	0.000106696	0.000457062	ECI2
ENSG00000259583	126.4537594	54.35727432	1.221792336	0.000107179	0.000459052	AC015712.2
ENSG00000047936	0	12.35130601	-6.210242022	0.000107367	0.000459781	ROS1
ENSG00000259660	58.12586555	9.955878399	2.542093451	0.000107386	0.000459789	DNM1P47

ENSG00000025434	1152.084254	262.2295418	2.134659775	0.000107972	0.000462221	NR1H3
ENSG00000162545	219.4613807	790.5237806	-1.848485874	0.00010801	0.000462306	CAMK2N1
ENSG00000155093	486.8435893	154.2227117	1.657933765	0.00010808	0.000462531	PTPRN2
ENSG00000184903	341.3911184	146.9680833	1.215843299	0.000108117	0.000462613	IMMP2L
ENSG00000089009	12148.73493	23345.87881	-0.942366695	0.000108247	0.000463095	AC004086.1
ENSG00000223482	502.5743447	271.0179662	0.890215925	0.000108371	0.000463548	NUTM2A-AS1
ENSG00000226757	42.94991126	2.038888596	4.362055624	0.000108403	0.000463606	AC079341.1
ENSG00000059691	1339.933476	516.2668802	1.375877434	0.000108533	0.000464055	GATB
novel.580	59.28317224	18.39458348	1.691219774	0.000108546	0.000464055	-
ENSG00000156804	210.2102607	981.9807157	-2.223866223	0.000108566	0.000464055	FBXO32
ENSG00000120322	19.9141932	0	6.650508659	0.000108579	0.000464055	PCDHB8
ENSG00000189108	20.34980907	0.356539013	5.720130443	0.000108665	0.000464346	IL1RAPL2
ENSG00000172382	60.7852341	12.168244	2.320463878	0.000109088	0.000466079	PRSS27
ENSG00000168769	801.1418476	307.0083377	1.384166223	0.000109108	0.000466087	TET2
ENSG00000119397	531.4607332	291.0464604	0.868083842	0.000109172	0.000466285	CNTRL
ENSG00000270578	33.95987978	1.742556871	4.262788716	0.000109861	0.000469147	AP000787.2
ENSG00000272555	12.91548979	0	6.025786015	0.000109941	0.000469414	AC009974.1
ENSG00000064199	38.8752677	106.5807084	-1.457571515	0.000110588	0.000472098	SPA17
ENSG00000288066	339.8192235	167.0045401	1.025413463	0.000110638	0.000472235	AC097448.1
ENSG00000204272	463.3008846	853.0960371	-0.880611999	0.00011085	0.000473061	NBDY
ENSG00000273442	15.80506039	0	6.317357716	0.000110969	0.000473492	AC006946.3
ENSG00000188282	18.08851785	0	6.509903092	0.000111031	0.000473678	RUFY4
novel.376	0	11.8506873	-6.148090121	0.000111057	0.00047371	-
ENSG00000048162	213.409955	558.8489233	-1.389020439	0.000111368	0.000474959	NOP16
ENSG00000165272	56.36644829	3.404837359	4.022893838	0.00011161	0.000475915	AQP3
ENSG00000234742	32.56097348	96.30110877	-1.565874437	0.00011178	0.000476561	AC144530.1
ENSG00000120049	203.6333124	56.39372277	1.854281851	0.000112255	0.000478505	KCNIP2
ENSG00000237493	112.6737334	298.1315333	-1.402907936	0.000112476	0.00047937	AC034102.2
ENSG00000105053	348.3782546	607.2283673	-0.801557959	0.000112675	0.000480138	VRK3
ENSG00000198755	7535.296957	15164.72186	-1.009000849	0.000112704	0.000480186	RPL10A
ENSG00000108591	403.7668112	666.0029708	-0.72213414	0.000112873	0.000480825	DRG2
ENSG00000132016	75.33326501	17.32625072	2.115595596	0.000112969	0.000481155	C19orf57
ENSG00000169188	313.2708838	532.9548882	-0.766696528	0.00011308	0.00048155	APEX2
ENSG00000146648	551.6136708	3624.935109	-2.716301193	0.00011324	0.000482152	EGFR
ENSG00000163599	26.08947391	0.356539013	6.07650097	0.000113743	0.000484212	CTLA4
ENSG00000237380	0.879722543	21.38267454	-4.573009261	0.000114619	0.000487862	HOXD-AS2
ENSG00000249436	11.78536427	0	5.892949665	0.000114794	0.000488528	AC008780.1
ENSG00000262814	192.0704579	470.798145	-1.293897724	0.000114813	0.000488528	MRPL12
ENSG00000032219	553.1091404	256.3577079	1.110189292	0.00011499	0.000489201	ARID4A
ENSG00000174840	465.5820207	761.7858791	-0.710471222	0.000115125	0.000489698	PDE12
ENSG00000168481	70.11702746	1.854670712	5.257620704	0.000115458	0.000491032	LGI3
ENSG00000279406	22.09191654	1.00940975	4.384935973	0.0001156	0.000491557	AL359183.1
ENSG00000076706	12655.9255	3236.226517	1.967502981	0.000115969	0.000493046	AP002956.1
ENSG00000278727	48.27820683	7.278327571	2.711909448	0.000116789	0.00049645	AC000403.1
novel.899	92.81387372	6.899279318	3.753074438	0.000116978	0.000497169	-
ENSG00000263956	270.6621097	129.9096661	1.060328261	0.000117327	0.000498572	NBPF11
ENSG00000004961	292.955564	616.4474685	-1.073536669	0.000117854	0.000500732	HCCS
novel.44	25.46907243	0.356539013	6.041764671	0.000117908	0.000500876	-
ENSG00000147123	1047.148443	1917.961823	-0.873061667	0.000118074	0.000501502	NDUFB11
ENSG00000109832	26.51248237	0	7.062115356	0.000118101	0.000501535	DDX25
ENSG00000210164	26.25148038	77.04723139	-1.554108169	0.000118279	0.000502208	MT-TG

ENSG00000131127	394.1943875	199.4632852	0.984055847	0.000118513	0.000503117	ZNF141
ENSG00000124171	74.29759077	9.027772514	3.057148305	0.000118596	0.000503391	PARD6B
ENSG00000271259	29.36756337	2.822786395	3.367054303	0.00011873	0.000503874	AC010201.1
ENSG00000265982	45.56192554	11.98402612	1.934484812	0.000118926	0.000504626	AC103810.3
ENSG00000135829	2193.894011	4010.771065	-0.870459044	0.000119536	0.000507131	DHX9
ENSG00000121413	789.6354616	334.4566834	1.238846352	0.000119622	0.000507413	ZSCAN18
ENSG00000230498	16.95789413	0	6.418921488	0.000120142	0.000509535	AL035409.1
ENSG00000198585	1791.441158	900.6879779	0.991740967	0.000120558	0.000511217	NUDT16
ENSG00000162065	181.7921267	391.8918162	-1.108394021	0.000120889	0.000512536	TBC1D24
ENSG00000114861	696.4675861	368.076016	0.920525661	0.000121141	0.000513519	FOXP1
ENSG00000172336	433.3923707	828.0958691	-0.934119289	0.000121425	0.000514639	POP7
ENSG00000255833	19.02884316	0	6.583046351	0.000121724	0.000515824	TIFAB
ENSG00000248719	0	11.16829086	-6.061948099	0.000121793	0.000515958	AC021127.1
ENSG00000158258	858.9009461	119.9480898	2.841300069	0.000121795	0.000515958	CLSTN2
ENSG00000104814	32.41010765	1.345879667	4.536597853	0.000121879	0.000516226	MAP4K1
ENSG00000115866	889.3663069	1840.219339	-1.049009138	0.000122086	0.000517022	DARS
ENSG00000197217	1123.783387	2106.587353	-0.906353236	0.000122691	0.000519499	ENTPD4
ENSG00000198909	1340.04198	776.7357438	0.786595025	0.000122714	0.00051951	MAP3K3
ENSG00000272254	16.11850216	0	6.345532893	0.000123594	0.000523134	AC022893.3
ENSG00000013588	35.98827532	2552.05824	-6.14830231	0.00012361	0.000523134	GPRC5A
ENSG00000259678	11.79546473	0	5.894053831	0.000123839	0.000524018	AC066613.2
ENSG00000250155	59.92782033	8.038560254	2.904909496	0.000123877	0.000524094	AC008957.1
ENSG00000248528	26.23790371	1.161661783	4.568201257	0.000124335	0.000525942	LINC02058
ENSG00000260101	24.42523556	2.934900236	3.076554913	0.000124355	0.000525942	AC008074.2
ENSG00000168589	12.05972911	0	5.926460255	0.000124448	0.00052625	DYNLRB2
ENSG00000186198	23.31788953	0.713078025	5.023127235	0.000125034	0.000528642	SLC51B
ENSG00000248671	56.01085283	17.02046238	1.7119073	0.000125209	0.000529298	AP003498.1
ENSG00000273654	6.685617652	32.33735021	-2.278845836	0.000125611	0.000530908	AC020904.2
ENSG00000135698	290.8503395	701.8206707	-1.270357543	0.000125676	0.000531098	MPHOSPH6
ENSG00000259826	43.5192688	11.71966024	1.886333626	0.000125981	0.000532302	AC072061.1
ENSG00000116406	872.5445919	1679.162259	-0.944490593	0.00012605	0.000532505	EDEM3
ENSG00000081014	317.1650722	608.4744959	-0.940336917	0.00012636	0.000533729	AP4E1
ENSG00000176658	299.5897383	1518.454058	-2.341567222	0.00012649	0.000534191	MYO1D
ENSG00000163214	373.4761179	622.073567	-0.736118936	0.000126654	0.000534798	DHX57
ENSG00000180139	35.2051557	4.901813183	2.827730048	0.000126994	0.000536145	ACTA2-AS1
ENSG00000141552	1011.57705	1800.852572	-0.832169105	0.000127126	0.000536614	ANAPC11
ENSG00000182108	116.5083589	43.74891036	1.410020573	0.000127204	0.000536856	DEXI
ENSG00000067182	2493.339893	4681.985786	-0.909104431	0.000127818	0.000539361	TNFRSF1A
ENSG00000124762	5415.885285	13220.0639	-1.287461661	0.00012794	0.000539788	CDKN1A
ENSG00000167635	1100.190505	1926.526776	-0.808123303	0.000128374	0.000541531	ZNF146
ENSG00000126970	371.8874118	204.6535146	0.861652978	0.000129013	0.000544138	ZC4H2
ENSG00000224081	40.05360169	5.361009426	2.898189941	0.000129316	0.000545328	SLC44A3-AS1
ENSG00000113812	342.9174706	599.6279064	-0.806387617	0.000129598	0.000546426	ACTR8
ENSG00000132003	62.84447301	262.7390307	-2.062484128	0.000129687	0.000546716	ZSWIM4
ENSG00000169857	203.7299581	442.245228	-1.118975788	0.000130187	0.000548734	AVEN
ENSG00000167653	3.590704505	25.6610143	-2.827229828	0.000130387	0.000549485	PSCA
ENSG00000273796	42.10238414	3.168712922	3.719494776	0.000130448	0.000549656	BX322562.1
ENSG00000229980	40.54409372	10.42568713	1.960710519	0.000130549	0.000549991	TOB1-AS1
ENSG00000224578	432.5854592	863.9524816	-0.998405506	0.00013069	0.000550496	HNRNPA1P48
ENSG00000052749	284.7849812	798.6423895	-1.487963439	0.00013112	0.000552219	RRP12
ENSG00000146197	274.2700201	2700.054941	-3.299482805	0.000131163	0.000552308	SCUBE3

ENSG00000256427	21.43641254	0.387220594	5.792922167	0.000131452	0.000553437	AC010175.1
ENSG00000237649	0	16.63337018	-6.63998455	0.00013175	0.000554599	KIFC1
ENSG00000175854	292.232363	647.8919707	-1.148838014	0.000132012	0.000555615	SWI5
ENSG00000236333	25.75708522	117.0756271	-2.184187041	0.000132226	0.000556425	TRHDE-AS1
ENSG00000131263	918.6280815	1450.162055	-0.658633971	0.000132625	0.000558013	RLIM
ENSG00000276148	31.7499885	3.302051734	3.282247669	0.000132994	0.000559474	AC084824.5
ENSG00000169093	626.032514	1723.855643	-1.461148005	0.000133092	0.000559798	ASMTL
ENSG00000253276	411.6343534	1556.042791	-1.918260988	0.000133173	0.000560048	CCDC71L
ENSG00000281398	107.0263408	462.8589364	-2.11215752	0.000133493	0.000561301	SNHG4
novel.512	132.365033	37.2628285	1.824034357	0.000133904	0.00056294	-
ENSG00000100532	350.3906808	152.2857096	1.200564686	0.00013466	0.000565979	CGRRF1
ENSG00000062716	2545.666489	5185.813101	-1.026497759	0.000134671	0.000565979	VMP1
novel.267	15.49670449	0	6.288972439	0.000134856	0.000566664	-
ENSG00000168356	27.18797347	2.120320855	3.668863134	0.000134883	0.000566687	SCN11A
ENSG00000112062	765.9241503	1428.204826	-0.899112977	0.000135409	0.000568804	MAPK14
ENSG00000099849	26.13962021	83.57409374	-1.676295589	0.000135575	0.00056941	RASSF7
ENSG00000234636	42.31600402	9.711453227	2.124934284	0.000135995	0.000571081	MED14OS
ENSG00000076554	277.3788683	27.99777568	3.304547593	0.000136526	0.000573218	TPD52
ENSG00000143858	28.17189176	1.813376645	3.966211897	0.000136627	0.000573549	SYT2
ENSG00000137815	781.1204644	1265.650338	-0.696207879	0.000136656	0.00057358	RTF1
ENSG00000137074	397.0619897	739.8971576	-0.898328373	0.000136876	0.000574412	APTX
ENSG00000138152	25.40804273	0.743759607	5.132690028	0.000137344	0.000576282	BTBD16
ENSG00000106399	223.3972523	562.6243326	-1.333180506	0.00013769	0.000577638	RPA3
ENSG00000175324	318.2728451	630.9714339	-0.987867261	0.000138095	0.000579247	LSM1
ENSG00000100372	301.2422828	594.3652006	-0.980768975	0.000138232	0.000579725	SLC25A17
ENSG00000238098	25.77080408	2.038888596	3.613749619	0.000138474	0.00058065	ABCA17P
ENSG00000172215	54.34377866	4.230029225	3.674592468	0.000138876	0.000582239	CXCR6
ENSG00000231233	2.430348874	22.42161	-3.210031262	0.000139071	0.000582962	CFAP58-DT
ENSG00000163349	1405.424744	724.27776	0.956124933	0.00013926	0.000583597	HIPK1
ENSG00000062038	20.97439878	1.365948763	3.890409937	0.000139267	0.000583597	CDH3
ENSG00000205143	43.32466746	7.449492825	2.532503918	0.000139505	0.00058441	ARID3C
ENSG00000166685	431.2347057	771.4103647	-0.838706515	0.000139506	0.00058441	COG1
ENSG00000104205	631.6827981	278.4664788	1.180840159	0.000139791	0.000585509	SGK3
ENSG00000169567	1624.977683	3449.893953	-1.086229009	0.000139931	0.000586002	HINT1
ENSG00000286369	27.32616027	3.169868797	3.094502243	0.000139998	0.000586188	AP002813.1
ENSG00000137574	263.4811985	496.8693767	-0.915212679	0.0001402	0.000586938	TGS1
ENSG00000086696	0.307102255	17.96362868	-5.783791474	0.000140262	0.000587102	HSD17B2
ENSG00000101463	279.9070456	24.97429832	3.481520518	0.000140294	0.000587141	SYNDIG1
ENSG00000277978	35.58128026	5.952517001	2.564316223	0.000140453	0.000587649	AC010542.5
ENSG00000138821	105.0328458	487.5228104	-2.215189437	0.00014046	0.000587649	SLC39A8
ENSG00000153294	0.308355904	21.32691518	-6.030335644	0.00014054	0.000587889	ADGRF4
ENSG00000160087	881.3844291	1497.062212	-0.764075654	0.000140657	0.000588283	UBE2J2
ENSG00000198720	531.5811992	236.1618201	1.17099846	0.000141157	0.000590279	ANKRD13B
ENSG00000075131	71.15378497	212.4119722	-1.578306958	0.00014138	0.000591117	TIPIN
ENSG00000180061	25.96110284	1.456837632	4.166925585	0.000141531	0.000591653	TMEM150B
ENSG00000144182	141.0220626	41.60612745	1.757096394	0.000141577	0.00059175	LIPT1
ENSG00000188986	1365.91379	2343.806046	-0.778971795	0.000141705	0.00059219	NELFB
ENSG00000167968	46.54870713	7.278327571	2.659477068	0.00014183	0.000592618	DNASE1L2
ENSG00000106683	707.8968252	1785.319998	-1.334473756	0.000141999	0.000593227	LIMK1
ENSG00000197808	162.5150318	80.15201261	1.018445365	0.000142131	0.000593681	ZNF461
ENSG00000144730	333.5908723	47.56792548	2.810182778	0.000142291	0.000594257	IL17RD

ENSG00000259354	1.803536605	54.39710854	-4.90997673	0.000142563	0.000595295	AC025580.2
ENSG00000106554	626.2271717	1206.199015	-0.945991336	0.000142699	0.000595769	CHCHD3
ENSG00000187498	8993.432558	24604.7973	-1.452004765	0.000142843	0.000596275	COL4A1
ENSG00000008294	4672.457317	2538.206318	0.880434281	0.000143312	0.000598135	SPAG9
ENSG00000112679	898.3688756	277.2811175	1.69499906	0.000143386	0.000598349	DUSP22
novel.90	0	23.46602086	-7.131416111	0.000143579	0.000598964	-
ENSG00000197860	274.3503419	526.8035937	-0.941024964	0.00014358	0.000598964	SGTB
ENSG00000153094	175.4891146	34.86393327	2.328252126	0.000143824	0.000599887	BCL2L11
ENSG00000103335	2269.067539	4223.509696	-0.896296065	0.000144328	0.000601856	PIEZO1
ENSG00000241935	30.4777727	5.471967392	2.490041546	0.000144343	0.000601856	HOGA1
ENSG00000040608	60.49954297	10.96644402	2.457126065	0.000144571	0.000602713	RTN4R
ENSG00000102780	239.9805328	507.8866995	-1.081751776	0.000144755	0.00060338	DGKH
ENSG00000287263	261.5501447	88.01483129	1.569475106	0.00014481	0.000603516	AC008875.3
ENSG00000198901	3.297463658	24.18308009	-2.869234776	0.000145082	0.000604506	PRC1
novel.678	55.51588784	13.30983669	2.062072096	0.000145094	0.000604506	-
ENSG00000124767	1827.375336	3247.332628	-0.829564364	0.000145229	0.000604969	GLO1
ENSG00000160563	212.4640734	399.9474574	-0.912758415	0.000145744	0.000607017	MED27
ENSG00000143799	1653.725943	3037.516089	-0.877304249	0.000145784	0.000607085	PARP1
ENSG00000174579	643.3818904	310.2738473	1.052506705	0.000146075	0.000608199	MSL2
ENSG00000207359	11.50222388	0	5.857615499	0.000146328	0.000609158	RNU6-925P
ENSG00000269113	234.3411595	19.58016717	3.57577751	0.000146489	0.000609731	TRABD2B
ENSG00000226025	14.06234001	0	6.146545136	0.00014696	0.00061159	AC005515.1
novel.1116	37.85295668	5.767143241	2.717632122	0.000147077	0.000611981	-
ENSG00000228427	14.0748765	0	6.1477407	0.00014713	0.000612105	AL590764.1
ENSG00000134884	5234.937007	2633.087178	0.991486375	0.000147693	0.000614345	ARGLU1
ENSG00000035141	439.9445596	863.8304788	-0.97377272	0.000147746	0.000614467	FAM136A
ENSG00000116954	554.0761921	275.5395412	1.006948989	0.000147798	0.000614587	RRAGC
ENSG00000070019	11.54756905	0	5.862711969	0.00014826	0.000616335	GUCY2C
ENSG00000063438	1.84261353	37.73348914	-4.363310474	0.00014827	0.000616335	AHRR
ENSG00000159640	72.644022	15.45852737	2.232942814	0.000148289	0.000616335	ACE
ENSG00000154096	1635.028823	360.21726	2.182549904	0.000148512	0.000617161	THY1
ENSG00000181234	210.1087839	0	10.04863702	0.000148598	0.000617421	TMEM132C
ENSG00000141682	151.8029231	725.1779978	-2.255854524	0.000148814	0.000618217	PMAIP1
ENSG00000167193	85.64930483	180.4470262	-1.075069669	0.000148921	0.000618562	CRK
ENSG00000149761	317.8644535	669.2002638	-1.073716186	0.000149826	0.000622224	NUDT22
ENSG00000182134	159.0531691	65.75608581	1.272009394	0.000149857	0.000622252	TDRKH
ENSG00000116667	655.8675445	250.8365781	1.386599286	0.000150014	0.000622806	C1orf21
novel.682	18.12738131	166.8438243	-3.202767259	0.000150434	0.000624449	-
ENSG00000224116	0	12.0643025	-6.171406171	0.000150466	0.000624455	INHBA-AS1
ENSG00000261103	56.32121783	4.167381792	3.781145058	0.000150484	0.000624455	AC009063.3
ENSG00000132436	180.2077665	404.8220907	-1.167326399	0.000150645	0.000625024	FIGNL1
ENSG00000249825	17.98751358	0.356539013	5.542120541	0.000151381	0.000627979	AC012636.1
ENSG00000283638	14.04060071	0	6.146603382	0.000151512	0.000628421	AC002407.1
ENSG00000221914	764.2294134	1343.424707	-0.813831007	0.000151641	0.000628857	PPP2R2A
ENSG00000169100	6696.421934	14203.43838	-1.084787402	0.000152045	0.000630433	SLC25A6
ENSG00000235706	121.6492624	37.58239298	1.699281412	0.000152239	0.000631134	DICER1-AS1
ENSG00000095794	571.7451342	306.5107671	0.898733269	0.000152392	0.00063167	CREM
ENSG00000241058	253.6147076	131.0681642	0.951270241	0.000152654	0.000632603	NSUN6
ENSG00000166145	206.9628286	36.6982781	2.495676846	0.000152666	0.000632603	SPINT1
ENSG00000126461	1744.098063	3102.181762	-0.830716439	0.000152796	0.000633041	SCAF1
ENSG00000004478	1180.979208	2996.33724	-1.343309407	0.000153147	0.000634395	FKBP4

ENSG00000158417	2330.353217	4015.392158	-0.785016953	0.000153204	0.000634527	EIF5B
ENSG00000100300	2374.39425	4089.373251	-0.784293499	0.000153309	0.00063486	TSPO
ENSG00000204851	101.6857775	17.81510106	2.505492855	0.000153966	0.000637482	PNMA8B
ENSG00000116212	371.7365731	924.8383221	-1.315220296	0.000154369	0.000639046	LRRC42
ENSG00000115661	390.5524252	648.5666612	-0.731585112	0.00015441	0.000639115	STK16
ENSG00000140563	577.1332931	79.52948513	2.85753247	0.000154624	0.000639899	MCTP2
ENSG00000178105	239.6049061	623.9439526	-1.381336311	0.000154906	0.000640963	DDX10
ENSG00000225556	33.70621405	4.597180723	2.864559045	0.000154937	0.00064099	C2CD4D
ENSG00000167280	1421.854173	609.0900888	1.223453158	0.000155202	0.000641984	ENGASE
ENSG00000127419	810.0338352	350.0969073	1.20971907	0.000155426	0.000642807	TMEM175
ENSG00000158773	1095.418361	628.4664485	0.801976812	0.000156215	0.000645877	USF1
ENSG00000177302	25.11884757	66.16209123	-1.397968245	0.000156218	0.000645877	TOP3A
ENSG00000277117	32.41017892	3.139187216	3.347423704	0.00015632	0.000646199	FP565260.3
ENSG00000069248	719.6878611	1155.738113	-0.683406461	0.000157009	0.000648933	NUP133
ENSG00000230426	52.3197848	6.940701779	2.898351012	0.000157052	0.000648933	ERVMER61-1
novel.1060	40.59040781	6.676207511	2.592933148	0.000157057	0.000648933	-
ENSG00000198934	80.91217737	26.57837931	1.6063262	0.000157451	0.000650459	MAGEE1
ENSG00000158806	40.63123623	0.356539013	6.716049437	0.000157941	0.000652379	NPM2
ENSG00000181991	356.092238	624.980611	-0.812005905	0.000158167	0.000653208	MRPS11
ENSG00000101203	350.1332536	1.161661783	8.261816754	0.000158323	0.00065375	COL20A1
ENSG00000259869	17.49834648	0	6.461987299	0.000158521	0.000654415	AL022344.1
ENSG00000153157	22.58165311	1.110911105	4.375908428	0.000158535	0.000654415	SYCP2L
ENSG00000166377	561.4335552	272.7165793	1.041471202	0.000158741	0.000655164	ATP9B
ENSG00000160961	582.6828608	264.8585125	1.137439378	0.00015878	0.000655218	ZNF333
ENSG00000248479	20.59143651	0.356539013	5.737243912	0.000159752	0.000659124	AC104137.1
ENSG00000114439	807.4247246	1617.467978	-1.002336372	0.000159834	0.000659359	BBX
ENSG00000171815	13.69101659	0	6.108399562	0.000159933	0.000659664	PCDHB1
ENSG00000237773	23.23590346	1.733100261	3.714512356	0.000160704	0.000662736	AC073332.1
ENSG00000143067	179.8062839	553.5515847	-1.62169611	0.000160921	0.000663529	ZNF697
ENSG00000150556	18.12258018	0	6.512579424	0.00016104	0.000663905	LYPD6B
ENSG00000261373	50.06140032	176.2613391	-1.816351508	0.000161064	0.000663905	VPS9D1-AS1
ENSG00000067141	474.0800048	1501.325723	-1.663130072	0.000161615	0.000666072	NEO1
ENSG00000267508	86.40706674	35.61804857	1.279489612	0.000161951	0.00066735	ZNF285
ENSG00000088179	265.337816	139.359322	0.928358908	0.000162023	0.00066754	PTPN4
ENSG00000142197	342.5834719	181.4090245	0.917592492	0.000162581	0.000669737	DOP1B
ENSG00000101311	18.45865889	188.0923425	-3.351309087	0.000163219	0.000672256	FERMT1
ENSG00000221821	166.6744531	61.43529611	1.436028988	0.000163257	0.000672306	C6orf226
ENSG00000079277	1018.490525	399.1236782	1.351447158	0.000163961	0.000675097	MKNK1
ENSG00000165046	39.71340602	95.69068797	-1.268770957	0.00016408	0.000675483	LETM2
ENSG00000178623	51.066089	9.082247606	2.478240203	0.000164571	0.000677397	GPR35
ENSG00000124019	32.44424125	2.486316478	3.697339287	0.000164941	0.00067881	FAM124B
ENSG00000232721	11.45938601	0	5.852771887	0.000165036	0.000679094	AC239800.2
ENSG00000259887	14.09353904	0	6.151629571	0.000165348	0.000680269	AC068987.1
ENSG00000015479	183.5442358	67.98834493	1.434503958	0.00016543	0.000680501	MATR3
ENSG00000254996	60.61076241	18.8845897	1.679451352	0.000165929	0.000682446	ANKHD1-EIF4EBP3
ENSG00000100614	1393.061093	781.675499	0.833863666	0.000166133	0.000683175	PPM1A
ENSG00000234290	70.25740899	17.72292792	1.982981993	0.000166289	0.000683709	AC116366.1
ENSG00000151276	1324.570187	360.7507086	1.876050313	0.000167135	0.000687078	MAGI1
ENSG00000164188	220.8975509	1.722487775	6.989147616	0.000167472	0.000688354	RANBP3L
ENSG00000174109	143.0147865	317.264799	-1.150487478	0.000167707	0.000689213	C16orf91
ENSG00000273179	67.68288946	16.21405534	2.062932489	0.000167802	0.000689495	AC092535.4

ENSG00000253159	49.4289602	301.318868	-2.608993156	0.000168292	0.000691399	PCDHGA12
novel.340	21.00588254	1.487519214	3.853087035	0.000168606	0.000692577	-
ENSG00000241360	64.07721244	174.4053713	-1.444061686	0.00016871	0.000692897	PDXP
ENSG00000111276	1645.13001	673.4819078	1.288264788	0.000168743	0.000692922	CDKN1B
ENSG00000233452	17.83664776	103.4840055	-2.534595205	0.000169115	0.000694339	STXBP5-AS1
ENSG00000148334	713.1542044	1378.16192	-0.950484671	0.000169353	0.000695102	PTGES2
ENSG00000100567	1195.380864	1944.942076	-0.702283438	0.000169354	0.000695102	PSMA3
ENSG00000137225	11.53872224	0	5.861731999	0.000169429	0.000695297	CAPN11
ENSG00000265688	41.40494026	108.7207132	-1.392236279	0.000169607	0.00069592	MAFG-DT
ENSG00000183298	45.08751748	105.6655268	-1.229600518	0.000169913	0.000697063	RPSAP19
ENSG00000132872	13.14354055	0	6.048983266	0.000170261	0.000698384	SYT4
ENSG00000259768	33.23660713	104.2302993	-1.650228055	0.000170293	0.000698404	AC004943.2
ENSG00000228707	0.894837599	17.8632832	-4.301173206	0.000170476	0.000699045	AL691426.1
ENSG00000124602	45.76565843	6.673767367	2.794277809	0.000170669	0.000699724	UNC5CL
ENSG00000276259	24.13457328	2.782648203	3.090964174	0.00017122	0.000701872	AC009118.3
ENSG00000154639	69.04905835	10.8236486	2.66398592	0.000171541	0.000703077	CXADR
ENSG00000164742	156.8302265	14.07494966	3.474383498	0.000172202	0.000705676	ADCY1
ENSG00000168140	8.257627283	49.36572814	-2.58260742	0.000172805	0.000708034	VASN
ENSG00000052850	51.43393586	0.693008929	6.184176971	0.000173038	0.000708878	ALX4
ENSG00000111729	12.49402006	0	5.976123832	0.000173116	0.000709086	CLEC4A
ENSG00000229334	6.080259955	31.20495734	-2.359682457	0.000173221	0.000709391	AC046143.1
ENSG00000143318	26.27411733	1.426156051	4.196872236	0.000173245	0.000709391	CASQ1
ENSG00000178999	187.2485371	848.6404744	-2.180536478	0.000173349	0.000709705	AURKB
ENSG00000119125	0.922560414	17.07005718	-4.227772327	0.000173447	0.000709994	GDA
ENSG00000142207	734.9373388	1410.692186	-0.940958315	0.000173495	0.000710076	URB1
ENSG00000020577	2363.839573	846.1519233	1.482429558	0.00017464	0.000714652	SAMD4A
novel.193	59.48754517	0.387220594	7.266220958	0.000175501	0.000718063	-
ENSG00000037280	95.56104217	11.75735829	3.028936933	0.000176135	0.000720543	FLT4
ENSG00000107957	9399.150444	4679.793634	1.006127854	0.000176642	0.000722501	SH3PXD2A
ENSG00000083454	7.990855601	64.83897759	-3.026213239	0.000177015	0.000723915	P2RX5
ENSG00000139915	19.66192367	0	6.630321623	0.00017705	0.000723945	MDGA2
ENSG00000115561	1408.69299	884.3241925	0.671558849	0.000177233	0.000724576	CHMP3
ENSG00000281128	11.24297409	0	5.824152951	0.000177285	0.000724678	PTENP1-AS
ENSG00000100181	123.0182074	5.900610448	4.37308488	0.00017746	0.000725278	TPTEP1
ENSG00000159403	7182.39218	2415.255549	1.572280183	0.000177591	0.0007257	C1R
ENSG00000260027	38.38498949	308.3969245	-3.007026447	0.000177642	0.000725757	HOXB7
ENSG00000135314	26.99287359	82.01040774	-1.605546999	0.000177661	0.000725757	KHDC1
novel.691	0.293240848	47.55201316	-7.19176998	0.000177728	0.000725915	-
ENSG00000112406	715.1703211	382.7518826	0.902161189	0.000177928	0.00072662	HECA
ENSG00000163661	687.5691419	9310.772695	-3.759331899	0.000178804	0.000730079	PTX3
ENSG00000169139	925.8907062	1650.78148	-0.83425191	0.000179211	0.000731527	UBE2V2
ENSG00000112210	117.5316666	505.7803432	-2.106184908	0.000179214	0.000731527	RAB23
ENSG00000163946	1357.959733	835.5736243	0.700841841	0.000179382	0.000732095	TASOR
ENSG00000225313	72.63768284	194.0609346	-1.416133402	0.000179575	0.000732753	AL513327.1
ENSG00000161281	525.9781213	107.8480283	2.284289509	0.000179599	0.000732753	COX7A1
ENSG00000144891	10.3997499	65.42218443	-2.657933822	0.000180244	0.000735267	AGTR1
ENSG00000225083	17.89710798	0.723690511	4.637899351	0.000180297	0.000735369	GRTP1-AS1
ENSG00000120696	202.7161657	89.00759239	1.186150085	0.000180375	0.000735571	KBTBD7
ENSG00000014641	1523.085094	3039.560779	-0.996955655	0.000180876	0.000737497	MDH1
ENSG00000170242	1919.684264	1162.287903	0.72380026	0.000181204	0.000738716	USP47
ENSG00000165995	74.3219083	17.68163385	2.065893736	0.000181232	0.000738716	CACNB2

ENSG00000272823	23.55059888	2.200597239	3.43822212	0.000181688	0.000740459	AL445423.1
ENSG00000197054	38.49008287	9.313620147	2.045381615	0.000182408	0.000743278	ZNF763
ENSG00000135549	116.9779933	14.76551844	2.986230214	0.000182525	0.000743638	PKIB
ENSG00000146038	23.98399219	0	6.917174011	0.000182628	0.000743941	DCDC2
ENSG00000281376	6.996480853	36.97351325	-2.40599732	0.000182875	0.000744832	ABALON
ENSG00000163625	1826.423164	927.8828697	0.977095437	0.000183249	0.000746235	WDFY3
ENSG00000279267	119.1453752	34.98267839	1.77454243	0.000183641	0.000747715	AL078621.3
ENSG00000237548	0.909952655	16.72413066	-4.20378738	0.00018395	0.000748857	TTLL11-IT1
ENSG00000125375	529.5206354	202.7086442	1.384443196	0.000184182	0.000749686	DMAC2L
ENSG00000177551	18.12634112	0	6.512894488	0.000184469	0.000750698	NHLH2
ENSG00000131507	3711.535291	2178.052095	0.768826051	0.000184489	0.000750698	NDFIP1
ENSG00000130723	1658.225789	3084.640078	-0.89549644	0.000185356	0.00075411	PRRC2B
ENSG00000158055	29.09502164	1.00940975	4.791266593	0.000185422	0.000754258	GRHL3
ENSG00000134595	16.49240415	0	6.37814035	0.000186194	0.000757282	SOX3
ENSG00000169131	287.4335651	140.5784941	1.033148068	0.000186231	0.000757311	ZNF354A
ENSG00000142798	13323.79735	7034.203378	0.921555167	0.000186559	0.000758529	HSPG2
ENSG00000177462	12.62858845	0	5.993156479	0.000186759	0.000759222	OR2T8
ENSG00000227295	3.016830569	23.53953757	-2.959545272	0.000186867	0.00075954	ELL2P1
ENSG00000151287	74.48602297	193.5747471	-1.378483384	0.000188158	0.00076467	TEX30
ENSG00000278558	30.32712033	2.211209725	3.796066809	0.000188608	0.00076638	TMEM191B
ENSG00000215548	26.57975247	1.029478846	4.650051947	0.000189198	0.000768654	FRG1JP
ENSG00000164434	12.57815742	0	5.987847593	0.000189644	0.000770348	FABP7
ENSG00000204442	44.1226174	219.4410755	-2.3158044	0.000189918	0.000771339	FAM155A
ENSG00000160888	3936.039625	777.5012079	2.339794945	0.000190041	0.00077172	IER2
ENSG00000184007	2694.848318	4361.742391	-0.69473797	0.000190118	0.00077191	PTP4A2
ENSG00000065457	242.947644	501.4799609	-1.046272456	0.000190754	0.000774373	ADAT1
ENSG00000102081	1028.155103	607.8007045	0.758222882	0.000191607	0.000777713	FMR1
ENSG00000069482	2.152223082	33.02462694	-3.951410497	0.000191845	0.000778556	GAL
ENSG00000170412	141.5938554	25.23622405	2.483604294	0.000191936	0.000778805	GPRC5C
novel.389	0	329.5321133	-10.94531833	0.000193028	0.000783115	-
ENSG00000132356	1050.227517	1819.298475	-0.792574556	0.00019325	0.000783892	PRKAA1
novel.599	2.135854377	43.03159434	-4.334572307	0.000193929	0.000786522	-
ENSG00000091947	452.6400896	847.6702097	-0.905351842	0.000194734	0.000789666	TMEM101
ENSG00000134283	701.4706871	1174.559074	-0.743826495	0.000195497	0.000792634	PPHLN1
ENSG00000173852	548.0236393	1178.093607	-1.104391187	0.000195546	0.00079271	DPY19L1
ENSG00000272008	54.11605642	8.315978758	2.706339293	0.000195813	0.000793667	AL139274.2
ENSG00000117090	21.22501521	0	6.740731781	0.00019596	0.000794143	SLAMF1
ENSG00000121594	11.1560447	0	5.814089766	0.00019693	0.000797946	CD80
ENSG00000204065	19.0514801	0	6.58478602	0.000197021	0.000798193	TCEAL5
ENSG00000261324	88.45423988	23.78396274	1.896701147	0.000197216	0.000798819	AC010168.2
ENSG00000254645	14.0317539	0	6.145661566	0.000197237	0.000798819	AC087379.1
ENSG00000073584	238.9208967	122.0284949	0.967966206	0.000197905	0.000801397	SMARCE1
ENSG00000169189	769.3031453	1254.535252	-0.705525469	0.000198206	0.000802491	NSMCE1
novel.1058	25.02077753	1.365948763	4.150409258	0.00019824	0.000802505	-
ENSG00000154545	48.71117321	186.1417614	-1.933603215	0.000198611	0.000803862	MAGED4
ENSG00000088340	82.06808718	747.6927016	-3.187895153	0.000198637	0.000803862	FER1L4
ENSG00000242259	648.1738502	346.4944955	0.903135836	0.000198718	0.000804063	C22orf39
ENSG00000269927	4.163324793	31.81653401	-2.922519835	0.00019893	0.000804797	AC004817.3
ENSG00000138758	1804.867958	4008.589038	-1.1512762	0.000199406	0.000806596	SEPT11
ENSG00000173065	237.3364924	462.3120025	-0.961669769	0.000199663	0.00080751	FAM222B
ENSG00000274031	18.1298886	1.10029862	4.065701671	0.00020048	0.000810687	AC092140.2

ENSG00000007171	28.80851975	0.356539013	6.221207374	0.000201241	0.00081364	NOS2
ENSG00000108511	23.99652867	220.0575669	-3.198499044	0.000201683	0.000815299	HOXB6
ENSG00000131115	210.7777676	97.76599035	1.110355896	0.000201766	0.000815508	ZNF227
ENSG00000188002	135.9831322	30.25259122	2.161860562	0.000201876	0.000815824	AC026412.1
ENSG00000228655	22.17480025	0.713078025	4.951845123	0.000201931	0.000815921	AC079793.1
ENSG00000104897	600.4615831	1087.197405	-0.856229165	0.000202146	0.000816663	SF3A2
ENSG00000257261	22.16498452	79.88551056	-1.85286564	0.000202237	0.000816904	AC008014.1
ENSG00000172466	2266.065322	1246.792179	0.862181962	0.000202413	0.000817488	ZNF24
ENSG00000096092	697.2150345	283.6579112	1.296530287	0.00020267	0.000818397	TMEM14A
ENSG00000251179	0.894837599	17.67333312	-4.291528959	0.00020291	0.000819241	TMEM92-AS1
ENSG00000198146	248.3390222	547.877817	-1.141107464	0.000203132	0.000819975	ZNF770
ENSG00000106976	127.5032513	1087.613643	-3.092642289	0.000203166	0.000819975	DNM1
ENSG00000144410	19.09403324	0.774441189	4.709982748	0.000203187	0.000819975	CPO
ENSG00000280011	12.12896488	0	5.933933311	0.000203293	0.000820133	AL031595.2
ENSG00000285530	11.87730825	0	5.903127388	0.000203316	0.000820133	AL445928.2
ENSG00000214182	124.3598453	238.8607243	-0.941728238	0.000203321	0.000820133	PTMAP5
ENSG00000143994	59.54965919	6.33729745	3.247167096	0.000203951	0.000822549	ABHD1
ENSG00000243829	3675.611213	7199.494239	-0.969915547	0.000204055	0.000822838	AC011495.1
ENSG00000004534	2794.513956	1557.501922	0.843425222	0.000204216	0.000823362	RBM6
ENSG00000117533	957.4705249	363.9553096	1.396279685	0.000204833	0.00082572	VAMP4
ENSG00000198492	1003.438065	1565.756807	-0.641990078	0.000205673	0.000828976	YTHDF2
ENSG00000141298	753.6463172	410.6080187	0.876343163	0.00020634	0.000831539	SSH2
ENSG00000145916	727.8403672	340.8994634	1.093390893	0.000206682	0.000832786	RMND5B
novel.1086	39.0709805	4.300848999	3.183979046	0.000207378	0.000835462	-
ENSG00000229563	0	15.54813197	-6.537451544	0.000207649	0.000836423	LINC01204
ENSG00000170161	153.6810389	30.40124723	2.335564556	0.000208587	0.000840073	AL512625.1
ENSG00000134198	12.49283768	0	5.976044747	0.000209154	0.000842226	TSPAN2
ENSG00000123739	618.5694602	361.0248698	0.776725936	0.000209211	0.000842324	PLA2G12A
ENSG00000172500	809.6688913	1608.276169	-0.990232344	0.000209402	0.000842962	FIBP
ENSG00000169174	1.233423615	52.51518951	-5.423339382	0.000209555	0.000843449	PCSK9
ENSG00000213024	826.906209	1359.676846	-0.717621515	0.000209665	0.000843759	NUP62
ENSG00000174306	659.3980331	1421.023059	-1.107702059	0.000210337	0.000846332	ZHX3
ENSG00000157741	740.7581664	325.3221313	1.187852209	0.000210684	0.000847597	UBN2
ENSG00000130052	781.074426	110.9350866	2.816137404	0.000210747	0.000847718	STARD8
ENSG00000286872	17.41517803	0.356539013	5.493113343	0.000211022	0.000848696	AC024270.5
ENSG00000160190	236.920678	45.10095459	2.389218231	0.000211626	0.00085099	SLC37A1
ENSG00000273507	15.09919663	0.387220594	5.288783218	0.000212056	0.000852589	AL354809.1
ENSG00000217027	22.73391513	1.783850939	3.666939894	0.000212589	0.0008546	TPT1P4
ENSG00000133937	17.10431483	0.356539013	5.467124499	0.000212832	0.000855445	GSC
ENSG00000073050	434.3047597	868.9971124	-1.000244649	0.000213337	0.00085734	XRCC1
ENSG00000173020	2822.828032	1686.738194	0.742880865	0.00021367	0.000858546	GRK2
ENSG00000167394	57.19280523	150.6586399	-1.398478787	0.000213713	0.000858585	ZNF668
ENSG00000185414	390.6427165	677.2592909	-0.794289597	0.00021393	0.000859324	MRPL30
ENSG00000086758	2695.278469	4939.657167	-0.873917949	0.000214321	0.000860762	HUWE1
ENSG00000165379	216.5869262	62.80453692	1.786154476	0.000214412	0.000860997	LRFN5
ENSG00000072422	263.0830777	561.7086583	-1.094799787	0.000214628	0.00086163	RHOBTB1
ENSG00000171885	14.60981606	0	6.202164408	0.000214636	0.00086163	AQP4
ENSG00000100416	559.2864896	1160.31707	-1.052685514	0.00021472	0.000861834	TRMU
ENSG00000111886	23.25986533	2.261960402	3.403972975	0.000215292	0.000863995	GABRR2
ENSG00000269343	103.5698761	213.9991006	-1.046618437	0.000216078	0.000867017	ZNF587B
ENSG00000175309	1193.356331	604.7052043	0.980747707	0.00021652	0.000868655	PHYKPL

ENSG00000074071	980.8457976	1862.572761	-0.925376164	0.000217112	0.000870893	MRPS34
ENSG00000155961	40.42241782	5.095359283	2.994616581	0.000217496	0.000872301	RAB39B
ENSG00000185989	192.1438106	385.9776051	-1.006821501	0.000217631	0.000872707	RASA3
ENSG00000261671	18.1954347	0.723690511	4.661654698	0.000217737	0.000872996	AL158211.1
ENSG00000177889	1264.204833	2113.083422	-0.741249364	0.000217882	0.000873445	UBE2N
ENSG00000161912	163.0685626	75.72689623	1.109559349	0.000219401	0.000879398	ADCY10P1
ENSG00000119906	1339.397907	667.8263517	1.004052102	0.000219603	0.000880072	SLF2
ENSG00000130299	268.478189	486.3121961	-0.856516341	0.000220027	0.000881637	GTPBP3
ENSG00000152518	3678.435416	1119.491654	1.716042345	0.000220145	0.00088197	ZFP36L2
ENSG00000119318	2428.607443	4360.566932	-0.844455541	0.000221033	0.000885303	RAD23B
ENSG00000182674	14.16159243	0	6.158076437	0.000221045	0.000885303	KCNB2
ENSG00000196642	1689.686627	2689.346716	-0.670478736	0.000221802	0.0008882	RABL6
ENSG00000250241	26.53476331	1.130980201	4.596169826	0.000222181	0.000889581	AC105383.1
ENSG00000280604	1.847628125	26.71642283	-3.8638303	0.000222497	0.000890708	AJ239328.1
ENSG00000185432	2197.100615	304.5256898	2.850453412	0.000222785	0.000891722	METTL7A
ENSG00000133104	2593.576364	1520.182285	0.770502548	0.000222926	0.000892152	SPART
ENSG00000104415	71.13708739	1036.442714	-3.865254272	0.000223104	0.000892725	CCN4
novel.127	10.85904291	0	5.775112086	0.000223445	0.000893951	-
ENSG00000084092	284.4410517	584.2635602	-1.038609211	0.000223561	0.000894204	NOA1
ENSG00000177576	186.1663502	87.69830208	1.084024539	0.000223577	0.000894204	C18orf32
ENSG00000178726	299.1817454	3366.198858	-3.49203629	0.00022364	0.000894319	THBD
ENSG00000182048	25.47623831	1.161661783	4.523310492	0.000224509	0.000897658	TRPC2
ENSG00000130720	3.082305388	278.12438	-6.498591258	0.000224619	0.000897959	FIBCD1
ENSG00000253620	15.20980315	0	6.261832841	0.000224657	0.000897972	AC144568.1
ENSG00000269404	15.19497283	0.387220594	5.296849073	0.000225059	0.000899371	SPIB
ENSG00000165672	1545.857189	3108.762671	-1.008032149	0.000225076	0.000899371	PRDX3
ENSG00000215045	24.85938431	0.693008929	5.12910243	0.000225633	0.000901421	GRID2IP
ENSG00000198440	171.1334551	59.80729322	1.512728226	0.000225659	0.000901421	ZNF583
ENSG00000037749	129.8298752	320.2956649	-1.303494938	0.000226151	0.000903248	MFAP3
ENSG00000183426	4.294345703	29.48375383	-2.786998634	0.000226499	0.000904499	NPIPA1
ENSG00000285269	20.38763235	1.049547942	4.254749819	0.000226557	0.000904593	AL160269.1
ENSG00000268555	22.02615698	0.387220594	5.834306404	0.000228917	0.000913873	AC123912.4
ENSG00000129596	332.8906226	36.54042226	3.184958942	0.00022907	0.000914344	CDO1
ENSG00000147606	26.73989204	1.161661783	4.591369184	0.000229428	0.000915632	SLC26A7
ENSG00000229694	24.1971848	1.080229524	4.489736468	0.000229507	0.000915807	AL158071.1
ENSG00000196267	107.6089468	43.55497908	1.30924315	0.00022965	0.000916239	ZNF836
ENSG00000237870	0	10.11874292	-5.919019792	0.000229975	0.000917394	AC073130.1
ENSG00000135407	237.9631781	63.22744778	1.910408176	0.000230921	0.000921025	AVIL
ENSG00000123685	75.10294824	18.120761	2.047033801	0.000232028	0.000925299	BATF3
ENSG00000231764	14.68524879	0	6.209182892	0.000232296	0.000926226	DLX6-AS1
ENSG00000152413	88.30645082	208.0062698	-1.23604184	0.000232499	0.000926894	HOMER1
ENSG00000168852	22.98299319	3.647978261	2.669379216	0.000234503	0.000934737	TPTE2P5
ENSG00000143013	2109.100792	1022.516785	1.044783356	0.00023468	0.000935299	LMO4
ENSG00000143147	265.1604817	583.0348843	-1.136594381	0.000234836	0.000935778	GPR161
ENSG00000124191	97.59566321	530.5279834	-2.442692598	0.00023492	0.000935969	TOX2
ENSG00000101470	15.84692935	0.336469917	5.35711916	0.000235064	0.000936401	TNNC2
ENSG00000006744	956.6586301	1840.574177	-0.944175521	0.00023538	0.000937513	ELAC2
ENSG00000114302	586.3819767	1031.362278	-0.814542467	0.000236244	0.000940731	PRKAR2A
ENSG00000088766	106.7405784	217.39559	-1.025825561	0.00023626	0.000940731	CRLS1
ENSG00000139055	29.59708124	2.751966622	3.395947646	0.000236856	0.000942961	ERP27
ENSG00000147509	21.64390637	182.2642903	-3.074165302	0.000237492	0.000945347	RGS20

ENSG00000167700	227.5405184	583.9404816	-1.359589034	0.000237743	0.0009462	MFSD3
ENSG00000213753	352.5104516	695.4449042	-0.980030157	0.000238292	0.00094824	CENPBD1P1
ENSG00000115008	30.34542687	523.9921998	-4.109479129	0.000238846	0.000950299	IL1A
ENSG00000059804	563.4799823	1994.865055	-1.823942019	0.000238915	0.00095043	SLC2A3
ENSG00000204934	125.4519337	21.74178209	2.520675036	0.00023896	0.000950462	ATP6V0E2-AS1
ENSG00000234072	82.0031547	175.2830776	-1.096255386	0.000239363	0.000951919	AC074117.1
ENSG00000115042	470.0834333	791.0351783	-0.750619665	0.000239896	0.000953893	FAHD2A
ENSG00000242574	21.86526196	0	6.784733813	0.000240179	0.000954874	HLA-DMB
ENSG00000177125	363.5768896	184.348501	0.980830405	0.000240634	0.000956534	ZBTB34
ENSG00000167291	1326.969114	2684.586373	-1.016596364	0.000240768	0.000956921	TBC1D16
ENSG00000254102	11.99292937	0	5.919006554	0.000240947	0.000957488	AC090136.3
ENSG00000145934	463.8052211	30.83921851	3.909856779	0.000241203	0.000958356	TENM2
novel.253	11.4291559	0	5.849248237	0.000241275	0.000958495	-
ENSG00000187634	68.72575741	846.9900123	-3.623892211	0.000241836	0.000960579	SAMD11
ENSG00000134802	451.1574607	1257.615431	-1.47871722	0.000242133	0.000961611	SLC43A3
ENSG00000172183	380.6903519	98.61073773	1.950849463	0.000242985	0.000964848	ISG20
ENSG00000167522	2211.247521	4369.852161	-0.98263649	0.000243215	0.000965611	ANKRD11
ENSG00000229119	159.3658554	320.8607423	-1.010307538	0.00024414	0.000969138	AC026403.1
ENSG00000087088	1677.623973	3274.828327	-0.964986488	0.000245617	0.000974852	BAX
ENSG00000174021	172.3232142	331.6329511	-0.944771099	0.000245806	0.000975452	GNG5
ENSG00000178734	83.31520288	5.423656859	3.923540864	0.000246356	0.000977484	LMO7DN
ENSG00000250616	25.78849771	3.537020296	2.854961429	0.000247216	0.000980746	AC012645.1
ENSG00000119138	1024.3794	2723.730523	-1.410721767	0.000247338	0.000981084	KLF9
ENSG00000032389	323.8318135	565.4390151	-0.804525226	0.000247507	0.000981602	EIPR1
ENSG00000158423	35.69063313	5.992655192	2.56460898	0.000248949	0.000987172	RIBC1
ENSG00000087510	53.76786814	454.1590729	-3.077986545	0.000250074	0.000991479	TFAP2C
ENSG00000043591	25.3367266	2.884149558	3.137514979	0.000250329	0.000992339	ADRB1
ENSG00000284713	37.7962574	3.749479616	3.358833813	0.000250403	0.00099248	SMIM38
ENSG00000261040	19.08916084	199.3100529	-3.385960191	0.000251083	0.000994954	WFDC21P
ENSG00000273987	21.72693262	1.742556871	3.61389197	0.000251103	0.000994954	AC121761.2
ENSG00000076716	1173.758217	219.8838927	2.415548807	0.000251396	0.000995962	GPC4
ENSG00000276644	30.09817193	1.049547942	4.818864729	0.000252198	0.000998986	DACH1
ENSG00000258743	81.97963571	1.722487775	5.553914594	0.000252655	0.001000646	LINC02301
ENSG00000160294	866.2410422	1576.072632	-0.863391052	0.000254777	0.001008896	MCM3AP
ENSG00000146282	536.7457915	888.7390348	-0.727621139	0.00025492	0.001009229	RARS2
ENSG00000149131	6993.909738	2670.224806	1.389037131	0.000254939	0.001009229	SERPING1
ENSG00000152661	1069.855189	3826.749776	-1.838795253	0.000256012	0.001013199	GJA1
ENSG00000133878	25.97252822	1.10029862	4.579111495	0.00025602	0.001013199	DUSP26
ENSG00000011028	2620.254116	13183.16861	-2.330941674	0.000256109	0.001013398	AC080038.1
ENSG00000120837	862.8870619	448.8088893	0.942463551	0.000256289	0.001013954	NFYB
ENSG00000137693	1659.371213	3141.059488	-0.920628954	0.000256477	0.001014544	YAP1
ENSG00000136045	820.820665	1467.588245	-0.83831884	0.000256606	0.001014902	PWP1
ENSG00000273013	13.70577564	0	6.111898507	0.000257111	0.001016743	AC117490.2
ENSG00000113522	30.12031068	88.51296267	-1.553862208	0.000257443	0.001017831	RAD50
ENSG00000134460	22.75160841	0	6.841064858	0.000257465	0.001017831	IL2RA
ENSG00000227218	27.14123212	3.515795324	2.933662981	0.000257673	0.0010185	AL157935.1
ENSG00000160401	17.31320486	64.19068318	-1.892591202	0.000257993	0.001019612	CFAP157
ENSG00000212743	24.9966022	3.647978261	2.790160357	0.000258356	0.001020887	LINC02656
ENSG00000109113	1705.789027	3073.379027	-0.849370069	0.000258635	0.001021836	RAB34
ENSG00000162975	26.01773084	1.456837632	4.169220779	0.000259412	0.001024751	KCNF1
ENSG00000255435	24.70593992	3.251301057	2.93212995	0.000260012	0.001026966	AP001267.3

ENSG00000108932	80.47336933	2800.863098	-5.121357651	0.000260126	0.001027257	SLC16A6
ENSG00000160410	666.9707099	1459.843187	-1.12995274	0.000260288	0.001027742	SHKBP1
ENSG00000012061	1119.464138	2135.19993	-0.931419927	0.000261215	0.001031247	ERCC1
ENSG00000069869	390.9509458	1431.191556	-1.871904024	0.000261446	0.001031844	NEDD4
ENSG00000165501	91.18230221	220.3204857	-1.274358954	0.000261485	0.001031844	LRR1
ENSG00000102024	1852.900324	3862.629543	-1.059904714	0.000261486	0.001031844	PLS3
ENSG00000101236	813.2264036	1281.094238	-0.655678519	0.000261562	0.001031988	RNF24
ENSG00000272599	3.637303322	24.67282952	-2.760499189	0.000262058	0.001033787	AC016394.2
ENSG00000186376	379.3454513	211.2176083	0.844847887	0.000263183	0.00103807	ZNF75D
ENSG00000115364	590.5096421	1033.314421	-0.80736051	0.000263267	0.001038243	MRPL19
ENSG00000286449	21.82675451	0	6.781165102	0.000263606	0.001039302	AC016590.4
ENSG00000103018	1021.107222	1691.8598	-0.728639207	0.000263616	0.001039302	CYB5B
ENSG00000102409	1382.686159	532.8116008	1.37635203	0.000264182	0.001041378	BEX4
ENSG00000160446	367.0354614	704.5898249	-0.941062463	0.000264222	0.001041378	ZDHHC12
ENSG00000124802	112.8747452	273.3225993	-1.276550713	0.000264282	0.001041454	EEF1E1
ENSG00000143771	627.3085905	1122.978121	-0.840292528	0.000264534	0.001042216	CNIH4
ENSG00000087502	791.9133052	1324.991338	-0.742681593	0.000264555	0.001042216	ERGIC2
ENSG00000163092	22.43469077	0	6.820837759	0.000265018	0.00104388	XIRP2
ENSG00000272320	25.80207438	1.874739808	3.818208167	0.000265269	0.00104471	AL445309.1
ENSG00000185567	307.0657547	1758.735355	-2.517976528	0.00026595	0.001047235	AHNAK2
ENSG00000152234	4717.313429	9270.402068	-0.974682741	0.000266299	0.001048448	ATP5F1A
ENSG00000229212	3.968367446	26.26082261	-2.731313507	0.000266422	0.001048773	AC044860.1
ENSG00000101608	3654.80161	7944.83868	-1.120248249	0.000267445	0.00105264	MYL12A
ENSG00000244754	2043.463171	1164.933827	0.810934034	0.000267821	0.001053922	N4BP2L2
ENSG00000114859	241.5620296	106.4902863	1.183841126	0.000267851	0.001053922	CLCN2
ENSG00000232457	4.546002329	59.59615253	-3.71120793	0.000268077	0.001054649	SLC16A6P1
ENSG00000232098	102.3881372	201.1352791	-0.973160302	0.000269051	0.001058322	AC012313.1
ENSG00000178567	850.9241797	442.075929	0.94456726	0.000269852	0.001061311	EPM2AIP1
ENSG00000112701	2227.739042	1266.545755	0.814666977	0.000270286	0.001062858	SENP6
ENSG00000108700	99.13412397	12.16580385	3.032953882	0.000270596	0.001063914	CCL8
ENSG00000122435	467.8624452	224.3235903	1.059794838	0.00027074	0.001064197	TRMT13
ENSG00000287125	2.460578986	25.1202574	-3.360530675	0.000270749	0.001064197	AC018695.9
ENSG00000186814	938.3129592	394.6437356	1.249856847	0.000272812	0.001072142	ZSCAN30
ENSG00000160145	100.7464917	13.08432474	2.94742602	0.000274315	0.001077887	KALRN
ENSG00000102962	13.74631968	0	6.113925108	0.000274497	0.001078437	CCL22
ENSG00000175544	16.2066852	0.336469917	5.391608954	0.00027467	0.001078955	CABP4
ENSG00000174502	0	10.60746487	-5.985433638	0.00027495	0.00107989	SLC26A9
novel.499	38.72752208	5.798980698	2.745984947	0.000275597	0.001082267	-
ENSG00000109339	375.770601	84.23749542	2.155011813	0.000275654	0.001082329	MAPK10
ENSG00000130037	10.64263099	0	5.74489476	0.000275869	0.001083007	KCNA5
ENSG00000158714	18.20539261	0.356539013	5.55801653	0.000277546	0.001089427	SLAMF8
ENSG00000154803	806.6121032	408.4653174	0.982343185	0.000277628	0.001089584	FLCN
ENSG00000136699	895.225829	1827.69652	-1.029614929	0.000278014	0.001090846	SMPD4
novel.545	0.308355904	12.92261609	-5.311113724	0.000278033	0.001090846	-
ENSG00000112039	131.4711643	356.6612106	-1.439674153	0.000278395	0.0010921	FANCE
ENSG00000156642	1131.992162	1939.538171	-0.776950122	0.000278594	0.001092718	NPTN
ENSG00000109381	668.6586977	392.4171271	0.768263705	0.00027911	0.001094575	ELF2
ENSG00000187837	215.7083853	101.9867385	1.080304288	0.000279647	0.001096515	HIST1H1C
ENSG00000250334	12.8125477	0	6.012473327	0.00027982	0.001097029	LINC00989
ENSG00000267586	16.03819702	0.356539013	5.375455927	0.000280161	0.001098201	LINC00907
ENSG00000144908	63.34899612	2.793260689	4.491423046	0.000280355	0.001098796	ALDH1L1

ENSG00000171346	0	10.63814645	-5.989192028	0.000280516	0.001099261	KRT15
ENSG00000143786	77.8428354	629.8208011	-3.016355525	0.000280713	0.001099864	CNIH3
ENSG00000213976	48.90660093	6.827303669	2.842075496	0.000281684	0.001103503	AC010615.1
ENSG00000089693	2726.494744	4590.0956	-0.751395174	0.000281996	0.001104559	MLF2
ENSG00000130270	34.65398998	106.6034276	-1.620295668	0.000282091	0.001104765	ATP8B3
ENSG00000066739	1214.593002	552.0732434	1.137669324	0.000282448	0.001105996	ATG2B
ENSG00000272515	11.70728169	0	5.884088626	0.000282693	0.00110679	AL355578.1
ENSG00000018408	3494.503265	1442.511652	1.276299025	0.000282865	0.001107253	WWTR1
ENSG00000120647	139.5658872	317.5639089	-1.187035551	0.000282897	0.001107253	CCDC77
ENSG00000166743	13.16743114	0	6.051568262	0.000283173	0.001108166	ACSM1
ENSG00000115828	82.86492671	587.4500399	-2.825740398	0.000283824	0.001110547	QPCT
ENSG00000232934	20.81064045	2.567748738	3.038624469	0.000284966	0.001114731	AL157786.1
ENSG00000196139	444.9428413	56.70109934	2.971034268	0.000284979	0.001114731	AKR1C3
ENSG00000165476	1026.025544	1754.56784	-0.774002732	0.000285869	0.001118045	REEP3
ENSG00000126012	1403.118339	3769.054594	-1.425487577	0.000286113	0.001118829	KDM5C
ENSG00000282221	10.31916003	0	5.700617842	0.000286446	0.001119961	AC119427.1
ENSG00000273032	14.21947409	0.336469917	5.201866063	0.000286664	0.001120648	DGCR9
ENSG00000230294	10.57840982	0	5.737039326	0.0002871	0.001122181	LINC02370
ENSG00000172318	23.49425561	2.129777466	3.453175006	0.000287675	0.001124259	B3GALT1
ENSG00000229891	25.33554422	2.058957692	3.584686526	0.000287766	0.001124449	LINC01315
ENSG00000123411	296.9647049	146.0812715	1.024646035	0.000288605	0.001127556	IKZF4
ENSG00000129347	424.6729709	804.6333968	-0.922389838	0.000289391	0.001130455	KRI1
ENSG00000235961	74.65311496	29.57348683	1.337768193	0.00028961	0.001131143	PNMA6A
ENSG00000100593	0	11.88124049	-6.147807793	0.0002902	0.001133275	ISM2
ENSG00000119640	94.91133605	191.2408111	-1.01111386	0.000290511	0.001134321	ACYP1
ENSG00000049860	1434.979739	3363.762997	-1.229095848	0.000291451	0.001137819	HEXB
ENSG00000153814	597.050504	238.9824703	1.320689116	0.000292059	0.001140021	JAZF1
ENSG00000149124	11.28079737	0	5.828655318	0.000292238	0.001140551	GLYAT
ENSG00000197771	1161.62533	1995.748377	-0.780956396	0.000292357	0.001140841	MCMBP
ENSG00000215414	13.39394352	51.80715453	-1.951180908	0.000292834	0.00114253	PSMA6P1
ENSG00000197852	643.6908429	197.3551523	1.706676987	0.000293589	0.001145307	INKA2
ENSG00000168653	1758.307657	3745.350964	-1.090876939	0.000294892	0.001150217	NDUFS5
ENSG00000158186	3428.420238	1582.757166	1.115000993	0.000295951	0.001154175	MRAS
ENSG00000160113	723.4312827	1303.084847	-0.848882329	0.000297003	0.001158102	NR2F6
ENSG00000283399	11.48334788	0	5.85544056	0.000297057	0.001158138	AC004381.2
ENSG00000163512	278.1341372	516.0789719	-0.892222493	0.00029722	0.001158601	AZI2
novel.1064	20.46105633	0.723690511	4.82966273	0.000297937	0.001161221	-
ENSG00000217702	19.17093344	0.693008929	4.749977628	0.00029802	0.00116137	AC073263.1
ENSG00000115520	487.0476232	841.2420918	-0.788257914	0.000298124	0.001161461	COQ10B
ENSG00000135740	110.0544107	278.9049511	-1.34049908	0.000298133	0.001161461	SLC9A5
ENSG00000259319	43.58356124	7.653779805	2.493560156	0.000299334	0.001165968	AF111167.2
ENSG00000111344	10.97620241	0	5.789117744	0.00029962	0.001166907	RASAL1
ENSG00000205838	24.14245118	2.129777466	3.492506495	0.000299765	0.001167297	TTC23L
ENSG00000277299	10.28642262	0	5.696517743	0.000299872	0.001167537	AC084876.1
ENSG00000153187	6287.476662	10799.76161	-0.780472424	0.000300373	0.001169314	HNRNPU
ENSG00000132825	213.3512035	100.0128903	1.092382989	0.000300439	0.001169393	PPP1R3D
ENSG00000260455	10.63636274	0	5.74414658	0.000300774	0.001170416	NBAT1
ENSG00000152822	10.25995345	0	5.693189977	0.000300792	0.001170416	GRM1
ENSG00000137806	301.6545063	550.6818445	-0.868750147	0.000301336	0.00117236	NDUFAF1
ENSG00000235194	263.2289728	143.8843986	0.871555018	0.000303083	0.001178977	PPP1R3E
ENSG00000186603	210.8924636	79.90057097	1.399555722	0.000303164	0.001179119	HPDL

ENSG00000137955	957.7197612	1567.939788	-0.711284789	0.000303533	0.001180377	RABGGTB
ENSG00000281392	74.40311143	12.8424681	2.524358859	0.000304294	0.001183047	AC107204.1
ENSG00000103647	1147.251593	251.8373616	2.186954734	0.000304311	0.001183047	CORO2B
ENSG00000150656	23.26272863	1.049547942	4.444898874	0.000305287	0.001186662	CNDP1
novel.610	42.79743578	3.199394504	3.7370291	0.000305568	0.00118758	-
ENSG00000162734	9984.185854	4188.643467	1.25311793	0.000305645	0.0011877	PEA15
ENSG00000112081	3550.045947	6606.943491	-0.896193364	0.000305841	0.001188284	SRSF3
ENSG00000172586	412.718007	856.591695	-1.053677881	0.000306243	0.001189669	CHCHD1
ENSG00000258168	20.70859601	1.793307549	3.529871836	0.000307559	0.0011946	AC025569.1
ENSG00000118257	5000.793725	1835.826441	1.44587658	0.000310708	0.001206652	NRP2
ENSG00000216819	25.63985444	3.302051734	2.974364509	0.000311279	0.001208688	TUBB2BP1
ENSG00000262823	11.92014612	0	5.908013622	0.000311439	0.001209129	AC127521.1
ENSG00000158966	42.24594154	200.3973801	-2.247040951	0.000311572	0.001209467	CACHD1
ENSG00000154380	605.9320932	2697.100606	-2.154310102	0.000312855	0.001214264	ENAH
ENSG00000081870	287.4362142	524.197599	-0.867208404	0.000313177	0.001215334	HSPB11
ENSG00000185627	1705.271329	2793.508892	-0.712081947	0.000314177	0.001219031	PSMD13
ENSG00000287151	33.23793205	80.95456684	-1.281913924	0.000314928	0.001221762	C2orf27A
ENSG00000266904	94.42543169	37.22829411	1.345634172	0.000315153	0.001222453	LINC00663
ENSG00000275409	14.46987706	0.336469917	5.227626913	0.000315226	0.001222555	AC026367.2
ENSG00000131969	39.28885953	6.123682254	2.684189361	0.000315709	0.001224245	ABHD12B
ENSG00000283486	20.12308323	0	6.665505379	0.000316214	0.001225901	FAM95C
ENSG00000117758	1575.307525	1020.027317	0.627148607	0.000316231	0.001225901	STX12
ENSG00000169689	508.7828335	895.4386634	-0.815707295	0.000316618	0.001227221	CENPX
novel.70	19.36589079	0.774441189	4.726765422	0.000317167	0.001229162	-
ENSG00000182648	213.7779447	102.2839222	1.066124898	0.000317912	0.001231867	LINC01006
novel.381	15.1634178	0.336469917	5.294221314	0.000318009	0.001232059	-
ENSG00000137054	273.9405113	562.7971542	-1.039418199	0.000318117	0.001232294	POLR1E
ENSG00000168701	593.2791895	1018.762532	-0.780039443	0.000318592	0.001233949	TMEM208
ENSG00000102043	16.19686947	0.336469917	5.388433248	0.000319284	0.001236446	MTMR8
ENSG00000125827	1382.496343	722.6974857	0.935367471	0.000319706	0.001237895	TMX4
ENSG00000173611	251.3657394	138.7195038	0.856705908	0.000319939	0.001238615	SCAI
ENSG00000049769	268.8067024	133.7664264	1.008160248	0.000320422	0.001240297	PPP1R3F
ENSG00000127129	16.28122029	0	6.35797415	0.000320582	0.001240733	EDN2
ENSG00000186204	12.21714792	0	5.943649204	0.000320949	0.001241968	CYP4F12
ENSG00000171227	56.97757568	7.633710709	2.886260107	0.000321645	0.001244476	TMEM37
ENSG00000168306	418.7952062	74.10459118	2.496196042	0.000322519	0.001247514	ACOX2
ENSG00000176463	353.6773602	156.899783	1.173946879	0.000322526	0.001247514	SLCO3A1
ENSG00000104267	55.71113028	9.784713146	2.499002842	0.00032275	0.001248195	CA2
ENSG00000287544	0	10.98407298	-6.035955518	0.000324439	0.001254541	AC097518.2
ENSG00000255112	813.7951476	1606.09028	-0.981005057	0.00032469	0.001255322	CHMP1B
ENSG00000005884	2102.116131	8257.278793	-1.973809258	0.000326503	0.001262145	ITGA3
ENSG00000116604	1034.948905	2192.443883	-1.0828198	0.00032748	0.001265734	MEF2D
ENSG00000164451	19.62979917	0.356539013	5.668360427	0.000327903	0.001267179	CALHM4
ENSG00000197498	410.4557194	887.1828573	-1.112439897	0.000328222	0.001268223	RPF2
ENSG00000151704	12.76329904	0	6.007305688	0.00032848	0.001269032	KCNJ1
ENSG00000144231	440.4674327	750.8375436	-0.769781893	0.000329766	0.00127381	POLR2D
ENSG00000232386	40.07957266	4.106018629	3.307651574	0.000330651	0.001277041	AC015712.1
ENSG00000233967	94.794932	25.54543283	1.892707964	0.000330738	0.001277186	AL359715.1
ENSG00000143643	548.5656335	254.7135831	1.106826379	0.000330887	0.001277572	TTC13
ENSG00000198185	435.7842284	81.15034315	2.422970344	0.00033133	0.001279091	ZNF334
ENSG00000271730	18.90158319	1.10029862	4.122944272	0.000332249	0.00128245	AL390208.1

ENSG00000286488	19.1230097	2.191140629	3.14137987	0.000332422	0.001282926	AC103858.3
ENSG00000277013	18.88897543	0.713078025	4.718448072	0.000332503	0.001283047	AC008556.1
ENSG00000206567	46.51973066	108.4123563	-1.218596655	0.000332619	0.001283306	AC022007.1
ENSG00000132581	514.407802	1003.929404	-0.964850778	0.000333056	0.001284799	SDF2
ENSG00000055332	1786.444449	3296.111827	-0.883660869	0.000333693	0.001287067	EIF2AK2
ENSG00000167664	13.7879039	0	6.118079296	0.000334053	0.001288133	TMIGD2
ENSG00000128606	103.4012732	365.3984651	-1.821829755	0.000334069	0.001288133	LRRC17
ENSG00000140470	24.11695092	1.345879667	4.10875314	0.000334406	0.001289241	ADAMTS17
ENSG00000258352	18.64580961	0.713078025	4.701465914	0.000334738	0.001290329	AC119044.1
ENSG00000132329	153.4893562	29.13106763	2.391767358	0.000334965	0.001291015	RAMP1
ENSG00000114982	1688.540548	931.7602262	0.857899294	0.000335297	0.001291913	KANSL3
ENSG00000175305	31.59912268	88.62165607	-1.487226834	0.000335298	0.001291913	CCNE2
ENSG00000273148	52.57602875	18.42526507	1.517007243	0.000335465	0.001292366	LINC00653
ENSG00000183696	1334.038741	584.0415759	1.191533753	0.000336549	0.001296351	UPP1
ENSG00000287787	3.646150135	24.77574354	-2.769596123	0.000336962	0.001297749	AC092275.1
ENSG00000104142	594.9653532	997.479783	-0.745581123	0.000337054	0.001297912	VPS18
ENSG00000159231	119.2483173	43.80210118	1.440303507	0.000337936	0.001301115	CBR3
ENSG00000198218	1056.807899	1732.727793	-0.713213439	0.000338137	0.001301693	QRICH1
ENSG00000215146	113.9088814	10.21451208	3.468076634	0.000338228	0.001301852	BX322639.1
ENSG00000100731	902.9710237	1907.649772	-1.078868852	0.000338419	0.001302393	PCNX1
ENSG00000179820	1079.501508	3011.051953	-1.479875884	0.00033866	0.001303129	MYADM
ENSG00000249852	1.848881773	29.0397464	-3.982553877	0.000338767	0.001303179	AC145676.1
novel.585	5690.220059	473.4451173	3.587286026	0.000338773	0.001303179	-
ENSG00000205038	10.55695525	0	5.734374224	0.000340484	0.001309477	PKHD1L1
ENSG00000060982	822.5983929	2908.670532	-1.821958663	0.000340511	0.001309477	BCAT1
ENSG00000159289	27.52956534	0	7.116382037	0.000341374	0.0013126	GOLGA6A
ENSG00000079156	54.12303667	162.7956887	-1.587974707	0.000341761	0.001313895	OSBPL6
ENSG00000220201	45.16065674	14.35938463	1.653042604	0.000342575	0.001316827	ZGLP1
ENSG00000080546	1095.438614	314.5486979	1.799442399	0.000343753	0.001321161	SESN1
ENSG00000256597	10.52547149	0	5.730481504	0.000344035	0.001322049	LINC02393
ENSG00000117791	266.8219711	81.52900622	1.707692565	0.000344165	0.001322354	MARC2
ENSG00000123159	1732.881767	2853.378204	-0.719564979	0.000344345	0.001322837	GIPC1
ENSG00000198752	1399.633597	2602.476129	-0.894734601	0.000344393	0.001322837	CDC42BPB
ENSG00000153179	902.3277083	256.9541901	1.812066437	0.000344958	0.001324812	RASSF3
ENSG00000171365	512.5478675	221.6099637	1.208559773	0.000346765	0.001331556	CLCN5
ENSG00000126091	32.52927625	92.88565892	-1.511818156	0.000347211	0.001333072	ST3GAL3
ENSG00000261008	2.654282684	32.62306944	-3.606068832	0.000347598	0.001334358	LINC01572
ENSG00000102753	867.3128554	1534.919083	-0.823664104	0.000349263	0.001340553	KPNA3
ENSG00000259884	199.7058039	1.682349583	6.871196561	0.00034968	0.001341809	AC025259.3
ENSG00000162434	2053.915948	5294.553088	-1.366121323	0.000349727	0.001341809	JAK1
ENSG00000135093	297.5699482	157.5162871	0.91820231	0.000349745	0.001341809	USP30
ENSG00000170145	722.6899044	1184.454428	-0.712799267	0.000350014	0.001342641	SIK2
ENSG00000167770	1279.972869	2144.641289	-0.744654056	0.000350219	0.001343229	OTUB1
ENSG00000112655	370.2876182	2911.457783	-2.975121728	0.000351513	0.001347992	PTK7
ENSG00000272572	36.47522021	9.069194976	2.013718916	0.000352969	0.001353379	AL138762.1
ENSG00000104365	1523.177857	867.4056982	0.81245897	0.000353241	0.001354222	IKBKB
ENSG00000063854	1143.504444	581.9297654	0.974196751	0.000353644	0.001355567	HAGH
ENSG00000139083	552.7710956	310.2105235	0.832965156	0.00035377	0.001355847	ETV6
ENSG00000102098	9.141182043	38.15280398	-2.064122491	0.000354878	0.001359893	SCML2
ENSG00000173578	19.99291652	0	6.654485026	0.000355363	0.001361552	XCR1
ENSG00000099864	1606.755336	214.9554264	2.901344175	0.000356037	0.001363932	PALM

ENSG00000142675	64.09447914	15.76547158	2.023405916	0.000356309	0.001364772	CNKSR1
ENSG00000164197	107.6366693	33.99462201	1.667666072	0.000356913	0.001366884	RNF180
ENSG00000237854	77.36381219	30.48969596	1.343310402	0.000357914	0.001370519	LINC00674
ENSG00000279499	11.71731088	0	5.885155401	0.000358795	0.001373688	AL157770.1
ENSG00000102038	791.3721798	1989.927735	-1.330345525	0.000359317	0.001375483	SMARCA1
ENSG00000259763	17.88296184	0.356539013	5.533960724	0.00035944	0.001375696	LINC02157
ENSG00000198910	14234.71165	314.3416762	5.501046731	0.000359478	0.001375696	L1CAM
ENSG00000139324	456.25232	971.0457392	-1.090102856	0.000360225	0.001378352	TMTC3
novel.73	235.2863576	55.68017802	2.075799676	0.000360983	0.001381048	-
ENSG00000253660	10.67160745	0	5.748510942	0.000361375	0.001382343	AC008464.1
ENSG00000231290	8.421100869	62.61646623	-2.891360366	0.000362647	0.001387008	APCDD1L-DT
ENSG00000146556	600.6928257	287.601438	1.063468717	0.000362788	0.001387342	WASH2P
ENSG00000151640	38.37202537	268.4344683	-2.807301195	0.000363867	0.001391263	DPYSL4
novel.606	29.83773976	0.356539013	6.270459428	0.000364682	0.001394173	-
ENSG00000188211	20.42566908	95.4905918	-2.227194189	0.000372786	0.001424947	NCR3LG1
ENSG00000183283	4095.46563	2422.577679	0.757521656	0.000374294	0.001430501	DAZAP2
ENSG00000183060	351.1788874	154.602098	1.182386945	0.000374922	0.001432688	LYSMD4
ENSG00000136463	328.2105104	613.4495013	-0.902611667	0.000377221	0.001441262	TACO1
ENSG00000108785	55.41774689	13.66406395	2.021089428	0.000378135	0.00144454	HSD17B1P1
ENSG00000197712	1728.765057	3323.564657	-0.942881346	0.000378266	0.001444828	FAM114A1
ENSG00000164885	171.2112805	426.1591991	-1.315997045	0.000378583	0.001445828	CDK5
ENSG00000279806	0.293240848	13.33820652	-5.356225559	0.00037939	0.001448698	AC018629.1
ENSG00000275734	8.484139662	31.22515483	-1.880396921	0.00038014	0.001451347	AC010538.1
ENSG00000205609	317.0787557	642.7006452	-1.019587277	0.000382264	0.001459242	EIF3CL
ENSG00000132964	244.0262987	429.3788252	-0.815031249	0.000382905	0.001461474	CDK8
novel.652	27.47731083	80.10917748	-1.541314867	0.000383034	0.001461753	-
ENSG00000167034	19.95355486	64.02542569	-1.683877169	0.000383677	0.001463989	NKX3-1
ENSG00000254598	20.49379374	85.54943581	-2.063613065	0.000384086	0.001465337	CSNK2A3
ENSG00000198466	309.9285179	696.5235923	-1.167754207	0.000384527	0.001466802	ZNF587
ENSG00000116857	1113.898715	1892.998556	-0.764944997	0.000385447	0.001470098	TMEM9
ENSG00000139405	266.6624281	550.7678531	-1.046885406	0.000386094	0.001472349	RITA1
ENSG00000143952	774.3051073	474.507636	0.70679568	0.000386633	0.001474186	VPS54
ENSG00000169976	1032.219942	1969.15362	-0.931935518	0.000387367	0.001476769	SF3B5
ENSG00000167977	591.0985038	998.9115426	-0.757001318	0.000387475	0.001476965	KCTD5
ENSG00000183137	186.5398253	336.4207239	-0.850997198	0.000387731	0.001477722	CEP57L1
ENSG00000091986	583.9905108	6640.827068	-3.507408015	0.000388519	0.001480508	CCDC80
ENSG00000103375	10.2637144	0	5.693626866	0.00038892	0.00148182	AQP8
ENSG00000165271	670.0068879	1356.779959	-1.018137306	0.000388977	0.00148182	NOL6
ENSG00000145506	72.89639099	5.390406738	3.779735817	0.000390024	0.00148559	NKD2
ENSG00000115604	33.92158578	129.7211286	-1.936992088	0.000390791	0.001488093	IL18R1
ENSG00000135903	37.50268838	1.130980201	5.086001324	0.000390795	0.001488093	PAX3
ENSG00000198453	195.8090937	99.68472116	0.973952945	0.000391443	0.001490193	ZNF568
ENSG00000197358	2.751312536	28.66545004	-3.384259153	0.000391523	0.001490193	BNIP3P1
ENSG00000136451	1595.281255	869.1155529	0.876448191	0.000391524	0.001490193	VEZF1
ENSG00000007264	56.49962049	7.520184204	2.926523044	0.000391576	0.001490193	MATK
ENSG00000180891	1849.890082	920.3149277	1.007002979	0.000391828	0.001490931	CUEDC1
ENSG00000196923	1710.595524	5439.998915	-1.669153607	0.000392022	0.001491453	PDLIM7
ENSG00000087152	1133.821799	1789.060782	-0.657839899	0.000392287	0.001492243	ATXN7L3
ENSG00000257894	62.18606192	2.038888596	4.904941064	0.000393006	0.001494759	AC027288.3
ENSG00000262873	30.81259777	4.1272436	2.928738746	0.000393245	0.001495299	AC127496.5
ENSG00000123810	53.85862975	110.1763949	-1.032336932	0.000393263	0.001495299	B9D2

ENSG00000236859	157.1533147	80.4599843	0.966156367	0.000393423	0.001495686	NIFK-AS1
ENSG00000101152	1618.626394	2862.527165	-0.822426386	0.000394148	0.001498224	DNAJC5
ENSG00000105971	628.9998356	2346.942527	-1.899789159	0.000394843	0.001500648	CAV2
novel.750	13.80309023	0	6.119630247	0.000394951	0.001500836	-
ENSG00000233354	11.09934542	0	5.807241152	0.000395347	0.001502123	LINC00028
ENSG00000244005	286.0449445	510.8934133	-0.836894528	0.000395759	0.001503467	NFS1
ENSG00000109572	1843.800359	1182.83545	0.640382868	0.00039595	0.001503975	CLCN3
ENSG00000166401	335.1053462	583.5187388	-0.800285948	0.000396416	0.001505423	SERPINB8
ENSG00000160991	898.8438246	1599.1321	-0.831181779	0.000396448	0.001505423	ORAI2
novel.75	14.42077095	0.387220594	5.223178801	0.000398348	0.001512419	-
ENSG00000170955	2483.880687	1410.574294	0.816333034	0.000398601	0.001513156	CAVIN3
ENSG00000277767	22.30198894	2.873537073	2.957470144	0.000398776	0.001513601	AL442128.2
ENSG00000102678	13.80685117	0	6.120004776	0.00039959	0.00151647	FGF9
ENSG00000286873	43.72210404	14.04298381	1.639862719	0.00040036	0.001519168	AC012306.3
ENSG00000069329	1524.066238	2412.056337	-0.662356328	0.000402008	0.001525201	VPS35
ENSG00000268947	34.86420493	4.178122672	3.042802451	0.000402641	0.00152738	AC002128.1
ENSG00000260816	11.88998728	0	5.904697101	0.000403387	0.001529985	AC027279.1
ENSG00000105447	507.3939842	1172.73336	-1.20902525	0.000403948	0.001531888	GRWD1
ENSG00000213730	13.32463649	0.336469917	5.107923492	0.000404817	0.001534932	POLD2P1
ENSG00000197261	6.109236418	30.28784912	-2.31636512	0.000404869	0.001534932	C6orf141
ENSG00000253476	49.9429159	12.57437781	1.990906173	0.000406349	0.001540177	AC091185.1
ENSG00000139160	98.92594631	44.48539671	1.153087059	0.00040637	0.001540177	ETFBKMT
ENSG00000136816	484.2796138	998.8444937	-1.044812959	0.000406481	0.001540369	TOR1B
ENSG00000179240	159.5910432	76.70703706	1.05678459	0.000406988	0.001542068	GVQW3
ENSG00000179041	257.5778035	618.3841447	-1.264039394	0.000407072	0.001542135	RRS1
ENSG00000109220	221.2410545	459.8431053	-1.055508502	0.000407125	0.001542135	CHIC2
ENSG00000172159	804.3075604	347.8010417	1.20955564	0.00040756	0.00154356	FRMD3
ENSG00000172315	331.0061991	585.5300282	-0.823278289	0.000407667	0.001543741	TP53RK
ENSG00000123454	24.75629968	4.993857928	2.305512994	0.000408564	0.001546911	DBH
ENSG00000235001	5.099746614	26.80389126	-2.388970758	0.000409833	0.001551488	EIF4A1P2
ENSG00000168944	689.068611	410.5297501	0.747698221	0.000410259	0.001552847	CEP120
ENSG00000104881	183.3967312	523.4790674	-1.512938895	0.000410311	0.001552847	PPP1R13L
ENSG00000248727	29.65349578	2.0188195	3.832097391	0.000410777	0.001554384	LINC01948
ENSG00000130347	78.64265224	165.9295761	-1.078101731	0.000411092	0.00155535	RTN4IP1
ENSG00000285417	14.02208071	0	6.142853954	0.000412083	0.001558873	BX571818.1
ENSG00000185352	1.188078446	21.91842275	-4.186506943	0.000412525	0.001560315	HS6ST3
ENSG00000100243	4747.381855	8907.192695	-0.907845704	0.000413545	0.001563945	CYB5R3
novel.688	2.991615051	27.24443107	-3.183224794	0.000416461	0.001574745	-
ENSG00000106526	33.92026121	4.565343267	2.88025482	0.000416586	0.001574989	ACTR3C
ENSG00000205707	450.4485049	179.2967132	1.327382827	0.000416742	0.00157535	ETFRF1
ENSG00000140990	1032.435686	1673.445683	-0.696763735	0.000416905	0.001575735	NDUFB10
ENSG00000141404	128.8316685	48.52085236	1.405136443	0.000418267	0.001580653	GNAL
ENSG00000187189	1155.568818	633.2588442	0.867287395	0.000418414	0.001580978	TSPYL4
ENSG00000186197	11.62565163	0	5.871859965	0.000418914	0.00158264	EDARADD
ENSG00000125686	569.4711362	1041.696712	-0.871228623	0.000419144	0.001583278	MED1
ENSG00000142002	1152.735776	2206.025034	-0.936235362	0.000419806	0.001585546	DPP9
ENSG00000268903	21.21567021	1.060160428	4.310163304	0.000420574	0.001588023	AL627309.6
ENSG00000125445	530.3346381	1182.189086	-1.156805594	0.000420584	0.001588023	MRPS7
ENSG00000274253	21.15717564	0.356539013	5.774214149	0.000420656	0.001588066	AC138649.1
ENSG00000260296	27.70765543	4.758889366	2.55623423	0.000421491	0.001590985	AC095057.3
ENSG00000136874	713.4619359	430.5068985	0.728768176	0.00042191	0.001592336	STX17

ENSG00000133169	163.1073548	1814.258879	-3.475402056	0.000422215	0.001593256	BEX1
ENSG00000108448	89.34635637	331.0258098	-1.888352664	0.000423533	0.001597997	TRIM16L
ENSG00000283602	19.03475539	0.774441189	4.700359108	0.00042385	0.001598961	AC116353.5
ENSG00000233535	10.66408556	0	5.747629044	0.000424321	0.001600263	AC003035.2
ENSG00000205784	13.29566002	0.336469917	5.10517112	0.000424335	0.001600263	ARRDC5
ENSG00000228903	66.401685	11.44699363	2.525143865	0.000424425	0.001600263	RASA4CP
ENSG00000254545	0	16.52673175	-6.625203777	0.000424441	0.001600263	AL354919.2
ENSG00000286481	24.60514912	3.291439249	2.917576449	0.000424764	0.001601245	AC011246.1
ENSG00000136982	90.87803542	274.4294477	-1.594597237	0.000424946	0.001601701	DSCC1
ENSG00000014216	2001.747963	3603.974118	-0.848248571	0.000426069	0.001605702	CAPN1
ENSG00000181524	203.0049336	356.5615886	-0.813409143	0.000426217	0.001606024	RPL24P4
ENSG00000111196	223.0161566	452.6216583	-1.021795996	0.000426696	0.001607596	MAGOHB
ENSG00000084207	5385.197243	9635.756667	-0.83941882	0.000426853	0.001607955	GSTP1
ENSG00000149809	182.7668421	76.33659351	1.25618849	0.000428293	0.001613147	TM7SF2
ENSG00000267056	15.15310388	0	6.256516622	0.000428553	0.001613889	AC005336.1
ENSG00000152208	13.22517061	0	6.059562066	0.00043045	0.0016208	GRID2
ENSG00000082014	1155.996189	545.7692089	1.082426006	0.000430762	0.001621646	SMARCD3
ENSG00000125991	2199.257376	3894.256836	-0.824343066	0.000430799	0.001621646	ERGIC3
ENSG00000166845	139.0753676	319.2932847	-1.19888919	0.000431884	0.001625295	C18orf54
ENSG00000186818	14.65014663	0	6.20602658	0.000431894	0.001625295	LILRB4
ENSG00000178297	21.14922647	60.42229034	-1.515764132	0.000431992	0.001625338	TMPRSS9
ENSG00000099769	23.11372936	3.189937893	2.848817598	0.000432031	0.001625338	IGFALS
ENSG00000048707	2191.287873	957.6030392	1.194427813	0.000432786	0.001627943	VPS13D
ENSG00000155876	1572.434422	2572.762401	-0.71029229	0.000434123	0.001632738	RRAGA
ENSG00000158481	20.2280619	0	6.67153396	0.000434319	0.00163308	CD1C
ENSG00000100519	1037.656396	1619.683349	-0.64251908	0.00043434	0.00163308	PSMC6
ENSG00000224861	96.92501633	194.6549766	-1.00594353	0.000435692	0.001637927	YBX1P1
novel.225	14.31274299	0.387220594	5.210320698	0.000436177	0.001639512	-
ENSG00000124688	229.4414843	407.3553994	-0.828385839	0.000436285	0.001639681	MAD2L1BP
ENSG00000116996	0	137.050708	-9.679561345	0.000437361	0.001643489	ZP4
ENSG00000007392	1856.776626	1029.613814	0.850932302	0.000438244	0.001646566	LUC7L
ENSG00000113013	2900.105809	5296.897	-0.869110926	0.000438421	0.001646996	HSPA9
ENSG00000054116	961.9885628	1556.311647	-0.693961128	0.000438741	0.001647959	TRAPPC3
ENSG00000253541	28.02066992	1.069617038	4.705709367	0.000440356	0.001653703	SEPT10P1
ENSG00000205426	4.27672335	33.17803485	-2.963200762	0.000440398	0.001653703	KRT81
ENSG00000197472	0.601596751	14.52237754	-4.588989569	0.000440497	0.001653836	ZNF695
ENSG00000227456	22.18525671	1.396630344	3.965932125	0.00044086	0.00165496	LINC00310
novel.79	11.23169125	0	5.8229486	0.000443326	0.001663977	-
ENSG00000088854	1448.715326	751.380377	0.947566825	0.000444008	0.001666295	C20orf194
ENSG00000198168	982.1753643	200.6174295	2.290766753	0.000444385	0.001667469	SVIP
ENSG00000133805	276.9993531	1357.559634	-2.292988454	0.000445193	0.001670261	AMPD3
ENSG00000105875	520.1326684	231.5924613	1.166729746	0.000445427	0.001670898	WDR91
ENSG00000203883	232.3754593	31.36247485	2.885154885	0.000446095	0.001673161	SOX18
ENSG00000270412	13.59649403	0.336469917	5.137445342	0.000446604	0.001674829	AL136084.3
ENSG00000135164	2449.252016	1110.291672	1.141598413	0.000447643	0.001678483	DMTF1
ENSG00000125450	639.9695322	1097.469231	-0.778216081	0.000448284	0.001680643	NUP85
ENSG00000181800	14.48757069	0.356539013	5.229129862	0.000448437	0.001680976	CELF2-AS1
ENSG00000151532	252.0277964	407.1795635	-0.691898347	0.000449075	0.001683123	VTI1A
ENSG00000048028	262.6491866	490.5905699	-0.900941908	0.000450738	0.00168895	USP28
ENSG00000116161	1010.386707	1796.326468	-0.830279873	0.000450759	0.00168895	CACYBP
ENSG00000112796	46.92826445	3.444975551	3.74801584	0.000452738	0.00169612	ENPP5

ENSG00000119559	427.8618372	735.4568056	-0.781522235	0.000453024	0.001696947	C19orf25
ENSG00000250133	0.293240848	18.55654892	-5.835392549	0.000453387	0.001698061	HOXC-AS2
ENSG00000232593	468.7523124	160.1888293	1.549880045	0.000455165	0.001704475	KANTR
ENSG00000260473	16.12351675	0.387220594	5.384574813	0.00045636	0.001708702	AC068987.3
ENSG00000160285	81.80722843	216.8967223	-1.406538772	0.000457121	0.001711253	LSS
ENSG00000285533	68.88135345	20.83832163	1.731647819	0.000457173	0.001711253	RELA-DT
ENSG00000275016	9.985588607	0	5.653659883	0.000457797	0.001713341	AC015574.1
ENSG00000160199	275.902906	490.0508877	-0.828247541	0.000458032	0.001713975	PKNOX1
ENSG00000167657	1124.435885	1936.438866	-0.784039915	0.000458957	0.00171719	DAPK3
ENSG00000216901	9.67096446	0	5.607667608	0.000459218	0.001717917	ZNF603P
ENSG00000183560	15.57123958	0	6.294030771	0.000460957	0.001724176	IZUMO1R
ENSG00000105723	772.4012503	1260.436877	-0.706262813	0.00046138	0.001725511	GSK3A
ENSG00000258086	0.307102255	16.33215817	-5.6479781	0.000461727	0.00172656	AC079313.1
novel.600	0	11.58606464	-6.111070036	0.000461832	0.001726703	-
ENSG00000111696	500.6724106	185.7770629	1.42890162	0.000462433	0.001728598	NT5DC3
ENSG00000172053	1858.848285	3024.632478	-0.702323525	0.000462472	0.001728598	QARS
ENSG00000253658	0	8.916942943	-5.738861425	0.000462904	0.001729798	LINC01592
ENSG00000176102	474.5225192	787.44882	-0.730554978	0.000462926	0.001729798	CSTF3
ENSG00000119682	756.7197598	1175.37329	-0.635249299	0.000463333	0.001731068	AREL1
ENSG00000261526	53.27214805	19.84196451	1.427924596	0.000464234	0.001734186	AC012615.1
novel.822	12.29125609	55.31850193	-2.167336906	0.000464693	0.001735649	-
ENSG00000141568	722.8290608	1230.692143	-0.76762577	0.00046485	0.001735987	FOXK2
ENSG00000184277	863.7387925	515.7920628	0.74355925	0.000465337	0.001737557	TM2D3
ENSG00000150045	18.65744846	0.743759607	4.685202281	0.000465759	0.001738882	KLRF1
ENSG00000033627	1136.762699	694.6852794	0.710857397	0.000467077	0.001743552	ATP6V0A1
ENSG00000198225	121.3224578	290.2240001	-1.257235041	0.000467591	0.001745221	FKBP1C
ENSG00000261126	41.16650431	4.545274171	3.166888435	0.000467875	0.001745877	RBFADN
ENSG00000214290	30.48328549	0.387220594	6.301360474	0.000467901	0.001745877	COLCA2
ENSG00000285886	19.68678318	1.702418679	3.484448643	0.000468534	0.001747921	AC211476.6
ENSG00000169249	529.8984535	224.6788794	1.238810837	0.000468626	0.001747921	ZRSR2
ENSG00000272040	48.56392579	15.23661144	1.664977668	0.000468651	0.001747921	AC010245.2
ENSG00000162924	132.4268178	43.35693787	1.610410131	0.000470828	0.00175579	REL
ENSG00000267201	0	10.50724778	-5.97771281	0.000473397	0.001765115	LINC01775
ENSG00000060138	2968.442024	4688.790226	-0.659555397	0.000474013	0.001767159	YBX3
ENSG00000236841	3.694002601	30.34908389	-3.046557613	0.000474328	0.001768079	AC007750.1
novel.46	79.15994161	6.533412089	3.58682761	0.000474688	0.001769168	-
ENSG00000156026	260.5786207	592.4406904	-1.185475632	0.000477352	0.001778842	MCU
ENSG00000273855	15.28817047	0.336469917	5.305087115	0.000477746	0.001780044	AC020658.5
ENSG00000011451	1016.530962	1603.009199	-0.65713142	0.000477812	0.001780044	WIZ
ENSG00000164219	558.2744762	334.0984278	0.740202878	0.000477916	0.001780177	PGGT1B
ENSG00000177556	596.9770244	1153.537199	-0.950134811	0.000478906	0.00178361	ATOX1
ENSG00000169193	182.5453443	88.0442286	1.050041633	0.0004801	0.001787801	CCDC126
ENSG00000261644	21.70289949	1.844058227	3.57960964	0.000481238	0.001791782	AC007728.2
ENSG00000182836	26.18761487	0	7.044100195	0.000481757	0.001793455	PLCXD3
ENSG00000137825	6.34327069	32.11872633	-2.335861683	0.000482273	0.001795118	ITPKA
ENSG00000231663	41.40737629	12.39260008	1.732596899	0.000483017	0.00179763	COA6-AS1
ENSG00000119280	5349.736182	1851.464891	1.530665616	0.000483339	0.001798574	C1orf198
ENSG00000121578	327.97941	697.2330528	-1.088498999	0.000483586	0.001799234	B4GALT4
ENSG00000196421	30.52186422	6.696276607	2.176666873	0.000483814	0.001799824	C20orf204
ENSG00000119655	3767.558844	1975.548278	0.931256738	0.000484199	0.001800998	NPC2
ENSG00000106245	501.4080052	826.9168689	-0.722108026	0.000484492	0.001801829	BUD31

ENSG00000258636	124.8905257	33.39763903	1.898670348	0.000484993	0.001803435	AL121821.1
ENSG00000112096	2093.867101	9899.243702	-2.241129686	0.000485096	0.001803562	SOD2
ENSG00000225259	46.36893611	111.8492879	-1.270759453	0.000485526	0.001804782	ST13P6
ENSG00000101624	89.87462823	181.3679872	-1.014093366	0.000485587	0.001804782	CEP76
ENSG00000243927	1315.851776	477.6159917	1.46163315	0.000485633	0.001804782	MRPS6
ENSG00000130706	1056.784934	1794.779701	-0.764183335	0.000486068	0.001806139	ADRM1
ENSG00000166579	815.6347451	1596.946212	-0.969098895	0.000486607	0.001807727	NDEL1
ENSG00000125249	2259.379188	977.5741554	1.208342364	0.000486634	0.001807727	RAP2A
ENSG00000198208	203.2942434	41.16956884	2.299716573	0.000486876	0.001808366	RPS6KL1
ENSG00000254876	41.33180101	10.94521905	1.908230518	0.000487388	0.001810011	SUGT1P4-STRA6LP
ENSG00000286724	47.99352806	13.77977381	1.792848781	0.000488117	0.001812459	AL732509.1
ENSG00000083544	485.2158663	243.6599746	0.993366745	0.00048911	0.001815886	TDRD3
novel.684	0	106.7399426	-9.31923398	0.000489726	0.001817913	-
ENSG00000173511	1793.845688	935.0221876	0.940243104	0.000490607	0.001820924	VEGFB
ENSG00000172748	202.5488042	69.09609238	1.549938463	0.000491007	0.001822031	ZNF596
ENSG00000287978	42.87497672	3.434234671	3.672175805	0.000491046	0.001822031	AC245407.2
novel.470	6.009699269	36.79507474	-2.611135904	0.000491349	0.001822896	-
ENSG00000273521	4.189793959	38.44309954	-3.191125015	0.000492566	0.001827151	AL162274.1
ENSG00000143452	25.09280568	1.110911105	4.523633768	0.000493202	0.001829246	HORMAD1
ENSG00000172667	2608.09011	1111.645681	1.230057737	0.000493395	0.001829704	ZMAT3
ENSG00000169067	0	12.06918279	-6.178396074	0.000493481	0.00182976	ACTBL2
ENSG00000226465	24.5134186	4.33153058	2.505337915	0.000493756	0.001830422	AL390198.1
ENSG00000260877	0.925067711	22.63150078	-4.624726654	0.0004938	0.001830422	AP005233.2
ENSG00000106546	5763.115768	1822.709231	1.660865945	0.000494109	0.001831305	AHR
ENSG00000266053	65.13942712	21.24676719	1.624841189	0.000494409	0.001831968	NDUFV2-AS1
ENSG00000225031	57.77581071	203.1263239	-1.814414689	0.000494429	0.001831968	EIF4BP7
ENSG00000133142	2963.115487	1628.002924	0.863975848	0.000494577	0.001832256	TCEAL4
ENSG00000140718	719.8445241	1194.254284	-0.73023439	0.000495381	0.001834972	FTO
ENSG00000100478	211.6608242	97.97070969	1.109263304	0.000495974	0.001836908	AP4S1
ENSG00000221953	30.82144458	5.422372589	2.512214368	0.000496339	0.001837998	C1orf229
ENSG00000182512	572.1004012	1254.170715	-1.132647683	0.000497081	0.001840482	GLRX5
ENSG00000129951	3.053328925	30.48969596	-3.324573522	0.000497797	0.00184287	PLPPR3
ENSG00000260838	15.20729586	0	6.261385573	0.000498298	0.001844311	AC022893.2
ENSG00000159714	406.931387	221.244178	0.879091217	0.000498328	0.001844311	ZDHHC1
ENSG00000211897	85.28746791	0	8.747882132	0.000499167	0.001847153	IGHG3
ENSG00000217801	6.696971762	50.82572943	-2.923106042	0.000499378	0.001847671	AL390719.1
ENSG00000259642	45.08404127	116.5396221	-1.37098209	0.00050011	0.001850114	ST20-AS1
ENSG00000250490	19.46507193	0.774441189	4.731455878	0.000500549	0.001851476	LINC02145
ENSG00000117152	145.3903294	1117.012777	-2.941963306	0.00050131	0.001854026	RGS4
ENSG00000126895	13.3712353	0.387220594	5.112391749	0.000502686	0.001858851	AVPR2
ENSG00000186481	38.06102033	1.00940975	5.190765493	0.00050466	0.001865884	ANKRD20A5P
ENSG00000115806	1214.198949	2035.461225	-0.745382534	0.000508544	0.001879977	GORASP2
ENSG00000116031	9.409207373	0	5.567425889	0.000508696	0.001880274	CD207
ENSG00000099139	356.3522154	156.9231913	1.185070948	0.000509194	0.001881846	PCSK5
ENSG00000088812	1110.574839	2091.667981	-0.91330225	0.000509482	0.001882641	ATRN
ENSG00000105708	146.9940807	74.36248852	0.981561402	0.000511064	0.001888218	ZNF14
ENSG00000097007	2123.606709	4206.265829	-0.985993663	0.00051289	0.001894695	ABL1
ENSG00000206559	47.94846763	16.52930029	1.536576801	0.000513138	0.001895344	ZCWPW2
ENSG00000003393	1385.670681	698.2982091	0.988628435	0.000513755	0.001897351	ALS2
ENSG00000146909	433.5125795	763.5508165	-0.81679927	0.000514271	0.001898988	NOM1
ENSG00000197083	586.5031738	52.07837411	3.491510737	0.000515078	0.001901698	ZNF300P1

ENSG00000143742	2469.823339	4217.293833	-0.771964318	0.000515927	0.001904562	SRP9
ENSG00000156253	224.1564104	495.7147914	-1.145712205	0.000516275	0.001905575	RWDD2B
ENSG00000206077	12.73564749	0.356539013	5.042461485	0.000517401	0.001909458	ZDHHC11B
ENSG00000182983	285.029701	81.66948989	1.801139794	0.000518128	0.001911869	ZNF662
novel.84	9.392838668	0	5.565181875	0.000518483	0.001912567	-
ENSG00000154127	188.7197709	390.6197568	-1.050246172	0.000518517	0.001912567	UBASH3B
ENSG00000092108	903.5277734	1387.22739	-0.618419803	0.000518537	0.001912567	SCFD1
ENSG00000166311	1080.37824	1993.912633	-0.884216455	0.000518843	0.001913421	SMPD1
ENSG00000269220	15.90996814	0.356539013	5.362671479	0.000520203	0.001918133	LINC00528
ENSG00000256612	9.410461022	0	5.567599849	0.000520268	0.001918133	CYP2B7P
ENSG00000187151	38.10539623	2.873537073	3.728523567	0.000520348	0.001918154	ANGPTL5
ENSG00000044524	1092.986008	27.87620523	5.291650158	0.000521085	0.001920602	EPHA3
ENSG00000105819	879.2283586	1354.031821	-0.623036985	0.000521952	0.001923522	PMPCB
ENSG00000101474	1836.357062	3484.17413	-0.924085279	0.000522447	0.001925073	APMAP
novel.1073	311.4769491	133.9161568	1.215775182	0.000524228	0.001931204	-
ENSG00000166446	102.9842486	260.9692255	-1.34186487	0.000524259	0.001931204	CDYL2
ENSG00000102924	11.95643102	0	5.914678148	0.000524971	0.001933552	CBLN1
ENSG00000165300	1971.930382	279.5121958	2.819102346	0.000525724	0.001936049	SLITRK5
ENSG00000108576	29.13653459	1.161661783	4.706719573	0.000526468	0.001938515	SLC6A4
ENSG00000164638	47.85165124	215.3265681	-2.169720155	0.000527081	0.001940499	SLC29A4
ENSG00000226413	22.52731894	0.672939833	5.00183293	0.000527256	0.001940867	OR8T1P
ENSG00000172878	418.6498253	165.3102123	1.33934026	0.000527711	0.001942267	METAP1D
ENSG00000102786	485.2896459	888.8725232	-0.872891802	0.000531606	0.001956324	INTS6
ENSG00000118523	3334.946567	24864.72242	-2.898381203	0.000531815	0.001956817	CCN2
ENSG00000154040	41.04643806	105.7578283	-1.368134819	0.000532042	0.001957375	CABYR
ENSG00000181610	526.3186747	974.0649921	-0.888442639	0.000533581	0.001962759	MRPS23
ENSG00000164989	98.9030959	27.99880316	1.814343964	0.000533856	0.001963493	CCDC171
ENSG00000158526	1222.18056	757.2345688	0.690557258	0.000538102	0.00197883	TSR2
ENSG00000092841	10244.68098	17482.41669	-0.771044629	0.0005394	0.00198332	MYL6
ENSG00000187626	212.2607678	113.5846528	0.903100253	0.000539745	0.001984308	ZKSCAN4
ENSG00000160678	152.8652643	30.84841833	2.310413045	0.000540517	0.001986866	S100A1
ENSG00000149548	34.24358963	96.24488268	-1.493012304	0.000540838	0.001987766	CCDC15
ENSG00000260259	0.908699006	13.38065646	-3.878696443	0.000541136	0.001988579	LINC02166
novel.788	150.780213	381.7562278	-1.340185332	0.000542141	0.001991991	-
ENSG00000128654	377.6723502	745.5347337	-0.981568692	0.000543174	0.001995504	MTX2
ENSG00000205362	31.12353224	77.38802084	-1.311784814	0.000543434	0.001996094	MT1A
ENSG00000279631	75.65110846	9.998456736	2.908113113	0.000543488	0.001996094	AL158211.5
ENSG00000134759	1134.387468	1736.124236	-0.613837033	0.000543805	0.001996975	ELP2
ENSG00000197857	282.5213683	132.7920646	1.087648793	0.000544575	0.001999352	ZNF44
ENSG00000261416	9.9717272	0	5.651837176	0.000544606	0.001999352	AC012645.3
ENSG00000105576	1697.737273	2595.806571	-0.612511369	0.000545826	0.00200345	TNPO2
ENSG00000115155	14.00313344	0.356539013	5.178852452	0.000545877	0.00200345	OTOF
ENSG00000135951	97.46733558	26.72014725	1.865225152	0.000546496	0.002005441	TSGA10
ENSG00000163040	122.3403241	308.7765675	-1.335761175	0.000546876	0.00200655	CCDC74A
ENSG00000160710	3923.122204	6462.005275	-0.719989467	0.000547675	0.0020092	ADAR
ENSG00000100968	22.50231689	71.95953087	-1.676704745	0.0005489	0.002013408	NFATC4
ENSG00000135052	1225.372355	3661.03091	-1.579126292	0.000549169	0.002013914	GOLM1
ENSG00000285094	14.95306066	0.356539013	5.275815354	0.000549193	0.002013914	LINC01488
ENSG00000132676	1177.118657	1877.957828	-0.674001551	0.000549383	0.002014328	DAP3
ENSG00000226622	27.25735143	1.456837632	4.235293407	0.000551277	0.002020952	AC092155.1
ENSG00000077327	18.00359756	0	6.505115337	0.000551355	0.002020952	SPAG6

ENSG00000008130	758.2954862	1369.799685	-0.853266415	0.000551423	0.002020952	NADK
ENSG00000277156	28.32354051	0.336469917	6.195301838	0.000553117	0.002026873	AL589743.5
ENSG00000023902	2518.366098	1281.729583	0.974661286	0.000555282	0.002034509	PLEKHO1
novel.627	16.05832667	1.467450118	3.473917272	0.000555357	0.002034509	-
ENSG00000136167	1546.487758	204.8211997	2.917155035	0.000555491	0.002034711	LCP1
ENSG00000134440	1891.55146	3243.749458	-0.77820998	0.000555706	0.002035212	NARS
ENSG00000130640	1213.974007	2137.498843	-0.816052499	0.000555925	0.002035728	TUBGCP2
ENSG00000158711	1321.028681	808.8667393	0.707863275	0.00055616	0.002036301	ELK4
ENSG00000172244	36.83232623	105.5737388	-1.520007214	0.000556446	0.002037062	C5orf34
ENSG00000140854	424.6519867	808.4632218	-0.928617459	0.000557995	0.002042388	KATNB1
ENSG00000069998	477.2855262	1022.273948	-1.098803045	0.000558058	0.002042388	HDHD5
ENSG00000187243	54.63917115	155.5062823	-1.508257852	0.000558551	0.002043905	MAGED4B
ENSG00000274286	10.04479518	0	5.661412299	0.000559692	0.002047644	ADRA2B
ENSG00000069399	381.4586684	762.3005995	-0.998388178	0.000559731	0.002047644	BCL3
ENSG00000197713	531.5420911	1062.654541	-0.999703462	0.000559875	0.002047883	RPE
ENSG00000125170	433.1971999	774.8020466	-0.839149584	0.000560001	0.002048056	DOK4
ENSG00000145833	1009.802977	1756.250622	-0.798322051	0.00056086	0.00205091	DDX46
ENSG00000177096	66.58446152	159.0352583	-1.258104093	0.00056165	0.002053508	PHETA2
novel.970	64.60356244	1.793307549	5.170483167	0.000562243	0.002055388	-
ENSG00000169740	528.7540817	302.5824638	0.8051828	0.000562462	0.002055611	ZNF32
ENSG00000141401	149.1867057	298.808724	-1.002979605	0.000562463	0.002055611	IMPA2
ENSG00000132424	5336.49027	2501.582126	1.093151845	0.00056262	0.002055897	PNISR
ENSG00000188610	50.25496148	129.9396241	-1.370095203	0.000563309	0.002058126	FAM72B
ENSG00000109929	938.7256781	421.2701811	1.155388313	0.000565682	0.002066505	SC5D
ENSG00000268089	48.29916321	7.673720506	2.66229564	0.000567027	0.002071127	GABRQ
ENSG00000224699	40.08953058	11.87191227	1.757937625	0.000569031	0.00207814	LAMTOR5-AS1
ENSG00000156463	3.691495303	54.78882424	-3.895445057	0.000569107	0.00207814	SH3RF2
ENSG00000215244	36.10765773	8.029103644	2.177942114	0.000570011	0.002081149	LINC02649
ENSG00000135316	2553.058855	4272.390053	-0.74285604	0.000570453	0.002082469	SYNCRIP
ENSG00000249274	18.8886907	0.713078025	4.720601761	0.00057109	0.002084425	PDLIM1P4
ENSG00000277363	10.92827867	0	5.783436007	0.000571149	0.002084425	SRCIN1
ENSG00000183615	67.59785446	12.45511911	2.437138248	0.000571867	0.002086752	FAM167B
ENSG00000099251	95.76860763	20.93280651	2.195777048	0.000572238	0.002087812	HSD17B7P2
ENSG00000168765	447.4102632	204.4301044	1.128582509	0.000572658	0.002089053	GSTM4
ENSG00000183474	12.57941107	54.29120644	-2.105627659	0.000573352	0.002091291	GTF2H2C
ENSG00000285887	22.46832583	2.772035718	2.99072282	0.000573963	0.002093225	AL009176.1
ENSG00000205629	416.333757	704.0763978	-0.757669152	0.00057471	0.002095654	LCMT1
ENSG00000139737	53.44922614	4.606637334	3.530882667	0.000574809	0.00209572	SLAIN1
ENSG00000078902	1857.111874	1112.883275	0.738982191	0.000574927	0.002095859	TOLLIP
ENSG00000088826	661.8712773	1243.734861	-0.910316428	0.000575124	0.002096281	SMOX
ENSG00000130813	941.8204351	581.1765835	0.696695984	0.000576825	0.002102185	C19orf66
ENSG00000125409	29.14516794	2.0188195	3.808226142	0.000577201	0.002103262	TEKT3
ENSG00000142192	26515.60827	14942.0468	0.827487796	0.00057988	0.002112727	APP
ENSG00000171150	606.380818	1060.836271	-0.807068808	0.000580079	0.002113155	SOCS5
ENSG00000261150	19.94721535	1.386017859	3.815782584	0.000580202	0.002113308	EPPK1
ENSG00000221883	48.22623742	14.90014152	1.689281968	0.000580687	0.002114777	ARIH2OS
ENSG00000166573	19.65698034	0	6.630151834	0.000581515	0.002117497	GALR1
ENSG00000104381	336.0093157	105.3745889	1.670860995	0.00058261	0.002121189	GDAP1
ENSG00000196428	791.5053551	451.8105809	0.808695573	0.00058307	0.002122565	TSC22D2
ENSG00000156795	93.632852	193.823877	-1.050223824	0.000583224	0.002122828	WDYHV1
ENSG00000185133	65.00246614	11.89313725	2.452736442	0.000583405	0.002123188	INPP5J

ENSG00000147614	0	13.17508521	-6.297047594	0.000584942	0.002128483	ATP6V0D2
ENSG00000213442	1256.046628	2472.677574	-0.977199709	0.000587021	0.002135736	RPL18AP3
ENSG00000167925	748.2749807	133.2653066	2.487957194	0.000587099	0.002135736	GHDC
ENSG00000172409	162.4236573	328.7382703	-1.017672402	0.000589189	0.002143038	CLP1
ENSG00000065154	1505.015382	2819.757719	-0.905778545	0.000589822	0.00214504	OAT
ENSG00000100221	985.5923736	1816.542296	-0.882089847	0.000591643	0.002151362	JOSD1
ENSG00000107338	144.6297049	354.1305051	-1.291975279	0.00059209	0.002152684	SHB
ENSG00000183580	721.4879778	378.5520682	0.930227037	0.000593847	0.002158474	FBXL7
ENSG00000197180	86.09459388	24.54090337	1.80384447	0.000593848	0.002158474	AC244090.1
ENSG00000235106	360.6906089	654.3302428	-0.858930623	0.00059438	0.002159828	BRD3OS
ENSG00000138071	4765.363346	8502.807045	-0.835386928	0.000594387	0.002159828	ACTR2
ENSG00000106268	312.9788254	575.559265	-0.879326962	0.000595791	0.002164627	NUDT1
ENSG00000069849	1942.259144	3945.754583	-1.022628177	0.000597431	0.00217028	ATP1B3
ENSG00000231106	20.95817261	0.387220594	5.761198873	0.000598002	0.002172054	LINC01436
ENSG00000267532	21.16838721	0.743759607	4.864626041	0.00059894	0.002175156	MIR497HG
ENSG00000101665	382.4441803	862.8359539	-1.17415437	0.00059999	0.002178663	SMAD7
ENSG00000171596	21.51080544	0.356539013	5.798110918	0.000600343	0.00217964	NMUR1
ENSG00000075618	3338.031962	8732.630498	-1.387440102	0.000600741	0.002180782	FSCN1
ENSG00000123892	111.5402464	23.3646479	2.262008463	0.000601828	0.002184422	RAB38
ENSG00000148459	56.24084142	164.4955045	-1.54831973	0.000602942	0.002188158	PDSS1
ENSG00000105376	9.861120675	738.059796	-6.226741937	0.000603549	0.002190058	ICAM5
ENSG00000096696	120.8631919	876.9445726	-2.859475333	0.000603701	0.002190304	DSP
ENSG00000121104	277.9228542	112.3351845	1.308581056	0.000604736	0.002193751	FAM117A
novel.334	21.4887814	1.823989131	3.573300175	0.000605158	0.002194974	-
ENSG00000168887	756.2718369	425.6765946	0.82935856	0.000605812	0.00219685	C2orf68
ENSG00000233695	303.2051482	113.8335259	1.415024087	0.000605844	0.00219685	GAS6-AS1
ENSG00000102595	560.5619956	1092.963847	-0.962931029	0.000605983	0.002197047	UGGT2
ENSG00000147044	840.0495955	432.5070616	0.957664943	0.000606794	0.002199682	CASK
ENSG00000248932	125.8985481	48.47442121	1.38119463	0.000607051	0.002200307	AC046134.2
ENSG00000150636	143.6583234	22.79017416	2.650046278	0.00060864	0.002205759	CCDC102B
ENSG00000108395	376.0772054	622.8690104	-0.727933362	0.000609352	0.002208028	TRIM37
ENSG00000120093	114.363899	501.4982196	-2.133001184	0.000609892	0.00220968	HOXB3
ENSG00000172216	1483.369285	4211.448089	-1.505367016	0.000610656	0.002212137	CEBPB
ENSG00000271344	37.9269223	6.144907225	2.630721125	0.000611204	0.002213816	AC018638.6
ENSG00000163798	519.253215	819.4968546	-0.658581814	0.000612018	0.002216456	SLC4A1AP
ENSG00000132394	1.509042109	20.81696826	-3.77939124	0.000612143	0.002216597	EEFSEC
ENSG00000259104	11.50856339	0	5.858346176	0.000612848	0.00221884	PTCSC3
ENSG00000235978	18.46589605	1.10029862	4.089161675	0.000614274	0.002223693	AC018816.1
ENSG00000131018	1598.234399	674.6792939	1.244005525	0.000614761	0.002225147	SYNE1
ENSG00000273387	47.75913779	8.680818506	2.463442154	0.000617763	0.002235701	AC005005.3
ENSG00000152217	189.4490107	33.65383256	2.48993305	0.000617997	0.002236235	SETBP1
ENSG00000175283	310.2122556	581.9636575	-0.908188767	0.000618189	0.002236618	DOLK
ENSG00000162374	10.28133675	0	5.695837032	0.000618491	0.0022374	ELAVL4
ENSG00000198242	3847.750409	7647.04561	-0.990902521	0.000620159	0.002243121	RPL23A
ENSG00000236830	49.34139007	10.516576	2.234567941	0.000621016	0.00224591	CBR3-AS1
ENSG00000164669	57.00992961	3.250145181	4.135289155	0.000621577	0.002247628	INTS4P1
ENSG00000271755	29.97822039	5.12488499	2.560166825	0.00062287	0.002251987	AL031118.1
ENSG00000169955	210.4620323	97.94349573	1.101245082	0.000623172	0.002252766	ZNF747
novel.856	12.0269917	107.8571345	-3.16239053	0.000623296	0.002252899	-
ENSG00000233765	10.61992277	0	5.74218591	0.00062468	0.002257334	AL591479.1
ENSG00000154556	1471.059761	558.9875141	1.395503798	0.000624696	0.002257334	SORBS2

ENSG00000147454	1089.423892	3625.086178	-1.734367559	0.000626991	0.002265292	SLC25A37
ENSG00000086159	12.02817408	0	5.922691173	0.000627073	0.002265292	AQP6
ENSG00000163812	467.4592223	996.2394324	-1.092018053	0.000627814	0.002267655	ZDHHC3
ENSG00000224431	3.945659226	37.37335411	-3.242510433	0.000628319	0.002269162	AC063976.2
ENSG00000226784	4.909803862	34.2780295	-2.808153929	0.000628723	0.002270306	PGAM4
ENSG00000164405	1540.724321	2707.328967	-0.813318885	0.000629967	0.002274483	UQCRQ
ENSG00000145692	13.27302307	0.336469917	5.102926883	0.000630153	0.002274837	BHMT
ENSG00000165802	1136.412084	2501.657308	-1.138485397	0.000630386	0.002275363	NSMF
ENSG00000104497	221.0104259	108.8613253	1.021155196	0.000633498	0.002286086	SNX16
ENSG00000130433	0.921306765	17.20108424	-4.235850023	0.000633533	0.002286086	CACNG6
ENSG00000226903	9.616772479	0	5.600304377	0.000635205	0.002291481	LINC00354
ENSG00000257346	9.616772479	0	5.600304377	0.000635205	0.002291481	AC011603.1
ENSG00000185920	1005.986837	197.0622066	2.351965471	0.00063638	0.002295402	PTCH1
ENSG00000232104	50.94578146	7.377517176	2.779594722	0.000636861	0.00229682	RFX3-AS1
ENSG00000267498	15.08658887	0.693008929	4.40463191	0.00063757	0.002299058	AC007786.1
ENSG00000273259	10.18563182	0	5.683493555	0.000640424	0.002309029	AL049839.2
ENSG00000230415	33.65585429	8.76340664	1.951812125	0.000640784	0.002310006	LINC01786
ENSG00000119411	13.79055374	0	6.118509242	0.000641818	0.00231341	BSPRY
ENSG00000220614	9.058013599	0	5.513351539	0.000642306	0.00231485	AL583834.1
ENSG00000137504	2034.154699	1030.992626	0.980629799	0.000646798	0.002330716	CREBZF
ENSG00000168032	3.94189828	22.1737172	-2.493959906	0.000647149	0.002331656	ENTPD3
ENSG00000183813	15.68213084	0	6.303931195	0.000649629	0.002340269	CCR4
ENSG00000287461	12.36933866	0.387220594	5.001271111	0.000649836	0.002340691	AL080317.4
ENSG00000267801	9.074382304	0	5.515676734	0.000650426	0.002342488	AC087289.6
ENSG00000074935	426.0090953	240.98045	0.821035953	0.000650642	0.002342944	TUBE1
ENSG00000115750	193.5714083	326.1821615	-0.752337888	0.000650867	0.002343428	TAF1B
ENSG00000047249	529.5809212	1050.181151	-0.98792543	0.000651898	0.002346695	ATP6V1H
ENSG00000253174	0	8.6512928	-5.693427824	0.000651955	0.002346695	AC009630.2
ENSG00000161682	58.01625577	171.6831059	-1.565991426	0.000652436	0.0023481	FAM171A2
ENSG00000268996	97.17087508	26.65292349	1.860772888	0.000652871	0.002349339	MAN1B1-DT
ENSG00000140688	565.1036946	998.250183	-0.820560778	0.000653943	0.002352872	C16orf58
ENSG00000133619	691.6584041	289.9483795	1.254978787	0.000654324	0.002353916	KRBA1
ENSG00000070614	5502.713997	3028.994728	0.861404406	0.000654659	0.002354798	NDST1
ENSG00000186648	9.338646688	0	5.557669183	0.000655225	0.002356505	CARMIL3
ENSG00000151025	14.37542578	0.356539013	5.218836914	0.000655738	0.002358026	GPR158
ENSG00000154262	899.4884849	84.25666543	3.415204366	0.000656149	0.002359176	ABCA6
ENSG00000134508	477.3600049	1233.299817	-1.369174994	0.000656474	0.002360017	CABLES1
ENSG00000238072	49.09746915	105.325204	-1.100383661	0.000656675	0.002360413	AC009244.1
ENSG00000198406	30.68938348	82.8756094	-1.435072267	0.00065691	0.002360931	BZW1P2
ENSG00000037897	111.8330166	270.8448876	-1.27717651	0.000658383	0.002365899	METTL1
ENSG00000163520	5020.392226	455.5436596	3.461935418	0.000659345	0.002369028	FBLN2
ENSG00000134602	61.05063742	189.9781959	-1.638065932	0.000659819	0.002370402	STK26
ENSG00000278740	9.399106912	0	5.566054845	0.000660403	0.002372174	AC005332.7
novel.760	10.05991024	0	5.663433334	0.000661811	0.002376901	-
novel.1115	27.60345969	3.658590747	2.927912264	0.000663595	0.002382979	-
ENSG00000241231	11.07670847	0	5.804356538	0.00066481	0.002387013	AC068308.1
ENSG00000138131	468.7789078	268.9394533	0.801966468	0.000665326	0.002388535	LOXL4
ENSG00000131368	702.8565858	1104.857138	-0.652726373	0.000666305	0.00239172	MRPS25
ENSG00000108963	202.4405194	378.3009178	-0.901241143	0.000666424	0.002391818	DPH1
ENSG00000153310	1252.069399	646.3013613	0.953738669	0.000666775	0.002392748	FAM49B
ENSG00000160049	781.4856187	1393.82476	-0.834999506	0.000666906	0.002392884	DFFA

ENSG00000155506	2776.242034	4535.840595	-0.708206683	0.000670668	0.002406053	LARP1
ENSG00000240291	51.88367039	14.30978983	1.857569835	0.000672574	0.002412555	AL450384.2
ENSG00000271538	22.04037439	1.854670712	3.597433734	0.000674869	0.002420456	LINC02427
ENSG00000133433	67.89840409	182.1039596	-1.424281193	0.000676087	0.00242449	GSTT2B
ENSG00000165275	327.6790905	175.3396889	0.902271867	0.000678162	0.002431595	TRMT10B
ENSG00000105323	3440.083439	5362.364443	-0.640419485	0.000679418	0.002435761	HNRNPUL1
ENSG00000163689	15.21392011	61.96847578	-2.026642047	0.000680117	0.002437929	C3orf67
ENSG00000158552	563.203342	340.5946085	0.726010773	0.000681545	0.002442713	ZFAND2B
ENSG00000175348	1579.152564	893.8267953	0.820743215	0.000683697	0.002450085	TMEM9B
ENSG00000180089	49.85752489	18.37451439	1.442833792	0.000684474	0.002452535	TMEM86B
ENSG00000068001	790.9481909	1375.114838	-0.797980413	0.000684821	0.002453437	HYAL2
ENSG00000116774	4784.780221	833.5464391	2.52094402	0.000685463	0.0024554	OLFML3
ENSG00000107140	790.8194798	476.488933	0.730989071	0.000685766	0.002456147	TESK1
ENSG00000205403	279.8694775	26.70624271	3.385645599	0.000686979	0.002460151	CFI
ENSG00000283667	12.36808501	0.336469917	5.00112856	0.0006879	0.002462916	AC009802.1
ENSG00000143878	10750.01405	4285.764396	1.32670327	0.000687941	0.002462916	RHOB
ENSG00000272121	7.868681504	38.69086394	-2.293929659	0.000688568	0.002464779	AC006058.3
ENSG00000115461	11848.81809	1749.857853	2.759465777	0.000688746	0.002464779	IGFBP5
ENSG00000177943	291.2122463	147.6258815	0.978723381	0.000688835	0.002464779	MAMDC4
ENSG00000284128	19.22254685	1.365948763	3.76941677	0.00068884	0.002464779	AP000356.3
ENSG00000179428	0	9.171852206	-5.775031146	0.000689692	0.002467488	AC073072.1
ENSG00000160209	1475.382883	3186.076294	-1.110705566	0.000689971	0.002468144	PDXK
ENSG00000226259	1.774560141	34.86799601	-4.281941322	0.000691545	0.002473436	GTF2H2B
ENSG00000164620	41.53936611	113.6149492	-1.448682914	0.000691662	0.002473514	RELL2
ENSG00000117016	64.1318317	12.17641634	2.405247834	0.000693849	0.002480994	RIMS3
ENSG00000118514	57.218278	3.81084278	3.931125527	0.000694085	0.002481495	ALDH8A1
ENSG00000126814	297.9466854	507.9648872	-0.769672536	0.000695565	0.002486445	TRMT5
ENSG00000101138	411.549746	650.5434631	-0.660734228	0.00069613	0.00248812	CSTF1
ENSG00000175518	19.59107825	2.476859868	2.972846659	0.000696359	0.002488597	UBQLNL
ENSG00000173401	22.28060564	2.038888596	3.40818854	0.000697035	0.002490671	GLIPR1L1
ENSG00000164543	851.0390304	1505.322984	-0.822978184	0.000697535	0.002492115	STK17A
ENSG00000267279	43.06004707	144.2609599	-1.742876335	0.00069871	0.002495968	AC090409.1
ENSG00000124713	28.79264924	7.194455167	2.002005816	0.000699284	0.002497678	GNMT
ENSG00000169519	259.1796594	479.0291163	-0.885997899	0.000699925	0.002499624	METTL15
ENSG00000135390	2181.455119	3701.073898	-0.762670568	0.00070039	0.002500939	ATP5MC2
ENSG00000084754	2894.992075	4393.76109	-0.601884184	0.000701163	0.002503349	HADHA
ENSG00000228204	11.91484679	0	5.909870888	0.000701258	0.002503349	AC004830.1
ENSG00000168938	173.3611384	475.3841865	-1.45582305	0.000701556	0.00250407	PPIC
ENSG00000211679	104.4345104	0	9.040082157	0.000702468	0.002506981	IGLC3
ENSG00000179958	232.9960743	657.7268991	-1.497719063	0.00070265	0.002507284	DCTPP1
ENSG00000240230	610.6495804	328.2459523	0.895897025	0.000703515	0.002510027	COX19
ENSG00000186105	9.044152191	0	5.511359691	0.000704065	0.002511644	LRRC70
ENSG00000117640	1028.89701	635.1928116	0.695996952	0.00070423	0.002511889	MTFR1L
ENSG00000197748	42.56716245	6.124838129	2.80005058	0.000704353	0.002511984	CFAP43
ENSG00000175967	10.17302406	0	5.681819003	0.000705018	0.002514009	FO393415.1
ENSG00000283010	9.613011533	0	5.599762207	0.000705314	0.002514721	AL589826.2
ENSG00000161798	14.34895661	0.336469917	5.216368997	0.000708751	0.002526515	AQP5
ENSG00000145741	4947.150634	7843.944205	-0.66499304	0.000708817	0.002526515	BTF3
ENSG00000170049	202.9226353	69.04953284	1.557382385	0.000709323	0.002527973	KCNAB3
ENSG00000263958	0	13.17778214	-6.303938003	0.000710159	0.002530604	AC091138.1
ENSG00000166592	52.58724032	1561.360577	-4.891883299	0.000711393	0.002534653	RRAD

ENSG00000096401	731.2511425	1191.11198	-0.703999515	0.00071171	0.002535089	CDC5L
ENSG00000073861	24.85744648	1.049547942	4.543346879	0.00071171	0.002535089	TBX21
ENSG00000136141	735.3189921	392.8948046	0.904906713	0.000712907	0.002539005	LRCH1
ENSG00000091527	2937.007643	4623.690717	-0.654708753	0.000714021	0.002542622	CDV3
ENSG00000170627	9.764162094	0	5.620237458	0.000715217	0.002546185	GTSF1
ENSG00000260997	9.764162094	0	5.620237458	0.000715217	0.002546185	AC004847.1
ENSG00000163600	16.92189397	0	6.413796347	0.000715337	0.002546261	ICOS
ENSG00000224596	15.83933618	132.9567145	-3.069374258	0.000716574	0.002550092	ZMIZ1-AS1
ENSG00000262003	0	10.79296702	-6.016592905	0.000716609	0.002550092	AC087392.1
ENSG00000236107	17.95860839	0.356539013	5.539414788	0.000717367	0.002552439	SCN1A-AS1
ENSG00000167543	1605.11038	897.3318841	0.838922122	0.000719214	0.002558661	TP53I13
ENSG00000134259	72.48774213	9.691384131	2.902433403	0.000719619	0.00255975	NGF
ENSG00000142279	1254.59104	583.2264011	1.104563859	0.000719804	0.00256006	WTIP
ENSG00000187257	591.2482158	348.8366039	0.761013607	0.000721018	0.002564027	RSBN1L
ENSG00000089012	17.54236672	0	6.465781164	0.00072128	0.002564604	SIRPG
ENSG00000267745	73.52618092	26.59973267	1.462839688	0.000723585	0.00257245	AC060766.7
ENSG00000198677	1719.296059	2560.316595	-0.574542106	0.000724768	0.002576304	TTC37
ENSG00000170315	1647.83281	5019.370546	-1.60698907	0.000725554	0.002578744	UBB
ENSG00000079785	1711.720602	2893.64884	-0.757574582	0.000729381	0.00259199	DDX1
ENSG00000286378	9.740271497	0	5.617021627	0.000730092	0.002594162	AL713852.2
ENSG00000227155	12.14791215	0.356539013	4.974033555	0.000731108	0.002597418	AL161725.1
ENSG00000229638	273.3419635	475.6130377	-0.799218205	0.000731659	0.00259902	RPL4P4
ENSG00000214456	10.98128828	0	5.789959904	0.000733144	0.002603939	PLIN5
ENSG00000173621	592.7585825	1330.354149	-1.166530384	0.000734544	0.002608554	LRFN4
ENSG00000118217	898.3687059	1362.288572	-0.600805248	0.000734712	0.002608796	ATF6
ENSG00000237476	29.60940426	7.114178783	2.051966352	0.000736432	0.002614478	LINC01637
ENSG00000284194	39.91087067	11.52598575	1.794234974	0.000736514	0.002614478	SCO2
ENSG00000170961	89.06875695	873.0813287	-3.293236896	0.00073976	0.002625643	HAS2
ENSG00000234444	368.2626602	173.2174415	1.087537227	0.000740156	0.002626688	ZNF736
ENSG00000111639	1322.119671	2466.758096	-0.89989291	0.000742841	0.002635859	MRPL51
ENSG00000109501	658.3133362	1770.330719	-1.427138424	0.000743234	0.002636893	WFS1
ENSG00000280152	0.601596751	13.26969849	-4.456168326	0.000743727	0.00263828	AC009078.3
ENSG00000131149	705.5087172	1067.638929	-0.597752608	0.000743929	0.002638636	GSE1
ENSG00000276855	176.9556043	69.84319122	1.339487654	0.000746585	0.002647343	AC015922.3
ENSG00000263126	27.45091294	6.817847059	2.010275949	0.00074665	0.002647343	AC040162.3
ENSG00000179766	28.70292782	4.035198856	2.845359564	0.000746689	0.002647343	ATP8B5P
ENSG00000139154	979.0343719	514.9406716	0.9274214	0.000748145	0.002652143	AEBP2
ENSG00000272602	202.4689976	86.10807845	1.233685172	0.000748996	0.002654797	ZNF595
ENSG00000173960	1212.780773	635.5311734	0.931940887	0.000749559	0.002656429	UBXN2A
ENSG00000279846	9.385245505	0	5.564133398	0.000751121	0.002661605	AC015660.4
ENSG00000231856	32.88297732	5.931292029	2.454947234	0.000751379	0.002662153	AL162377.1
novel.645	8.514369774	35.25462149	-2.055458739	0.000752009	0.002663714	-
ENSG00000159166	11.65086714	0	5.874970969	0.000752024	0.002663714	LAD1
ENSG00000170615	12.06223641	0.387220594	4.965079535	0.000752173	0.002663791	SLC26A5
ENSG00000180846	0.308355904	11.16829086	-5.100039304	0.000752251	0.002663791	CSNK1G2-AS1
ENSG00000103042	363.9932735	666.2212092	-0.872125811	0.000753358	0.002667347	SLC38A7
ENSG00000041357	1793.115591	3090.152323	-0.785308435	0.000754215	0.002670019	PSMA4
ENSG00000064490	574.974873	948.0220917	-0.721293137	0.000755003	0.002672167	RFXANK
ENSG00000130751	26.95143191	163.7068204	-2.603604011	0.000755028	0.002672167	NPAS1
ENSG00000138629	491.2623045	874.8518647	-0.832903511	0.000757349	0.002679751	UBL7
ENSG00000171806	163.8288319	91.93175175	0.833712597	0.00075747	0.002679751	METTL18

ENSG00000126088	1371.665365	2287.949343	-0.738034449	0.00075748	0.002679751	UROD
ENSG00000229246	114.2368104	0	9.169521717	0.000757856	0.002680716	LINC00377
ENSG00000273214	9.988167176	0	5.653996479	0.000758541	0.002682772	AL031587.4
ENSG00000134571	22.19145368	1.548882377	3.900905765	0.000758729	0.002683075	MYBPC3
ENSG00000249743	9.128574284	0	5.523410284	0.000759982	0.002686406	AC116345.1
ENSG00000167755	9.128574284	0	5.523410284	0.000759982	0.002686406	KLK6
ENSG00000259485	9.128574284	0	5.523410284	0.000759982	0.002686406	LINC02253
ENSG00000103550	163.3407759	337.3894815	-1.047498233	0.000763392	0.002698093	KNOP1
ENSG00000188186	1801.673953	1037.885395	0.795587767	0.000765079	0.002703689	LAMTOR4
ENSG00000116044	2758.496412	1447.696312	0.929888175	0.000766687	0.002709002	NFE2L2
ENSG00000001631	262.43457	147.6882249	0.829424053	0.000768007	0.002713239	KRIT1
ENSG00000256481	16.39404938	0.356539013	5.408432498	0.000768095	0.002713239	AP006333.2
ENSG00000104327	16.25077672	0.672939833	4.524800894	0.00076927	0.002717022	CALB1
ENSG00000284052	41.12548955	11.61931476	1.81336034	0.000769506	0.002717487	AC006460.2
ENSG00000234215	36.55330244	0.672939833	5.71191166	0.000771941	0.002725715	CT66
ENSG00000260742	21.38952899	3.159256312	2.74260539	0.000772967	0.002728965	AC009962.1
ENSG00000161298	151.4716028	72.18311625	1.068501364	0.000773886	0.002731838	ZNF382
ENSG00000286746	32.84092274	0.713078025	5.518361186	0.000774698	0.002734334	AL590807.1
ENSG00000267612	15.11055074	0.672939833	4.417230842	0.00077696	0.002741944	AC008759.2
ENSG00000279838	30.33887389	2.527610546	3.590568179	0.000777553	0.00274361	AL356273.3
ENSG00000166763	9.602911072	0	5.598349495	0.000777643	0.00274361	STRCP1
ENSG00000198520	58.14982741	15.73479	1.885486205	0.000778907	0.002747695	ARMH1
ENSG00000163354	23.35940248	3.974991568	2.560091855	0.000780761	0.002753863	DCST2
ENSG00000231298	0.616711807	14.554215	-4.581696026	0.000781223	0.002755116	MANCR
novel.307	13.50447878	0.356539013	5.12849608	0.000781901	0.002757136	-
ENSG00000166762	120.2958876	36.98412573	1.700173562	0.000782072	0.002757365	CATSPER2
ENSG00000274659	21.84276721	3.11911812	2.780838598	0.000782338	0.002757927	LINC02371
ENSG00000263001	516.8671002	854.4958773	-0.725600438	0.000784022	0.002763489	GTF2I
ENSG00000285906	11.75262686	45.7766729	-1.956530101	0.000785386	0.002767693	AC083855.2
ENSG00000082175	88.71609502	0	8.804745135	0.000785428	0.002767693	PGR
ENSG00000135709	394.8148443	172.3263101	1.194669605	0.000785672	0.002768179	KIAA0513
ENSG00000162576	7664.268957	4080.959695	0.909183664	0.000785923	0.002768688	MXRA8
ENSG00000120063	1399.63633	834.1238843	0.746885325	0.000786848	0.002771569	GNA13
ENSG00000272381	25.1985398	0.693008929	5.151340206	0.000789925	0.002782032	LINC02664
ENSG00000184178	262.3076222	554.0863436	-1.079480861	0.000790144	0.002782424	SCFD2
ENSG00000196337	0.307102255	11.8707564	-5.188470396	0.000791649	0.002787329	CGB7
ENSG00000260464	22.75655208	3.709341424	2.641339352	0.000791751	0.002787329	AL049796.1
ENSG00000275464	155.4248826	395.717475	-1.349135812	0.000792218	0.002788594	FP565260.1
ENSG00000254040	10.50025597	0	5.727073867	0.000793265	0.002791903	AC103719.1
ENSG00000171045	297.1986679	132.1500163	1.171300872	0.000793487	0.002792305	TSNARE1
ENSG00000128641	816.474713	3262.349449	-1.998462643	0.000794054	0.002793921	MYO1B
ENSG00000287684	10.70817708	0	5.753297413	0.000794163	0.002793928	AL392172.2
ENSG00000171759	10.74313705	0	5.760576689	0.000794336	0.002794157	PAH
ENSG00000117118	862.1686616	1487.490871	-0.786836829	0.000796304	0.002800701	SDHB
ENSG00000286758	11.79671838	0.356539013	4.93242812	0.000796912	0.002802459	AL445438.1
ENSG00000196951	40.79066448	12.42199739	1.725847967	0.00079852	0.002807734	SCOC-AS1
ENSG00000011454	1508.57227	947.7057589	0.670944733	0.000798741	0.002808133	RABGAP1
ENSG00000168036	4034.839175	8186.314759	-1.020738431	0.000798962	0.002808529	CTNNB1
ENSG00000127241	34.6668112	3.913628404	3.141522605	0.000802823	0.002821719	MASP1
ENSG00000247982	94.57887574	39.86583514	1.246383545	0.000803291	0.002822981	LINC00926
ENSG00000287405	13.18985463	0.356539013	5.094696912	0.00080393	0.002824844	AC017028.2

ENSG00000275216	0	10.4966353	-5.975963381	0.00080518	0.002828854	AL161431.1
ENSG00000247596	738.9899851	1408.149701	-0.930043588	0.000805454	0.002829434	TWF2
ENSG00000178078	31.60066106	87.4682138	-1.471618387	0.000806013	0.002831015	STAP2
ENSG00000137522	294.7023304	506.7705704	-0.78211972	0.000806151	0.002831117	RNF121
ENSG00000139289	3298.207187	7132.806945	-1.112770883	0.000809691	0.002842995	PHLDA1
ENSG00000119718	337.3218892	694.1050048	-1.041569034	0.000809752	0.002842995	EIF2B2
ENSG00000259462	13.90735724	0.693008929	4.286467228	0.000810039	0.002843617	CPEB1-AS1
ENSG00000146285	23.49801655	1.345879667	4.074705747	0.000811105	0.002846977	SCML4
ENSG00000124831	938.6361945	2069.032478	-1.140350584	0.000811235	0.002846989	LRRFIP1
ENSG00000247498	8.495493772	48.66981234	-2.521818448	0.000811328	0.002846989	GPRC5D-AS1
ENSG00000287281	18.98342672	1.029478846	4.161887564	0.000812087	0.002849267	AC012101.2
ENSG00000186222	273.6381386	482.388516	-0.818249666	0.000813178	0.002852711	BLOC1S4
ENSG00000278897	22.49827121	2.200597239	3.370541951	0.000813859	0.002854715	AC020951.1
ENSG00000167588	18.50873392	1.487519214	3.667944135	0.000815679	0.002860711	GPD1
ENSG00000135766	193.4525957	459.8661965	-1.248953672	0.000816084	0.002861694	EGLN1
ENSG00000174903	1485.940539	2565.08812	-0.787577018	0.000816179	0.002861694	RAB1B
ENSG00000204311	81.21684325	31.34800955	1.369879234	0.00081672	0.002863203	PJVK
ENSG00000009954	1667.215694	2631.771898	-0.658582508	0.000817421	0.002865276	BAZ1B
ENSG00000248890	35.85983333	267.109325	-2.897722636	0.000817868	0.002866454	HHIP-AS1
ENSG00000051620	830.8983832	1530.852642	-0.881373332	0.000818163	0.002867102	HEBP2
ENSG00000244953	10.01832602	0	5.657985473	0.000819045	0.002869806	AC087521.1
ENSG00000133872	4962.052387	2629.294201	0.91628493	0.000819227	0.002870057	SARAF
ENSG00000112234	896.2078379	473.0178862	0.922264766	0.000819589	0.002870937	FBXL4
ENSG00000173557	12.13537566	0	5.93471965	0.000820252	0.002872873	C2orf70
ENSG00000129515	1378.402281	2419.588328	-0.811922598	0.000821816	0.002877962	SNX6
ENSG00000152223	714.3297348	1222.178367	-0.774585963	0.000824255	0.002886116	EPG5
ENSG00000005189	50.06132905	171.6067764	-1.778097175	0.000825676	0.002890701	REXO5
ENSG00000197021	338.1407246	552.8445682	-0.709598017	0.000825974	0.002891353	CXorf40B
ENSG00000126581	627.4987467	982.9141717	-0.647498832	0.000826511	0.002892626	BECN1
ENSG00000100564	337.4711179	155.3753021	1.117757454	0.00082656	0.002892626	PIGH
ENSG00000196597	97.88769385	35.62981694	1.459224735	0.000828144	0.00289778	ZNF782
ENSG00000021488	18.34128593	0.774441189	4.642984927	0.000829165	0.002900962	SLC7A9
ENSG00000100427	12.99238999	0.356539013	5.071833155	0.000829351	0.002901221	MLC1
ENSG00000196159	172.8623416	444.0239284	-1.36168398	0.000831312	0.00290769	FAT4
ENSG00000179262	1342.225375	2128.283466	-0.664954109	0.000832833	0.002912618	RAD23A
ENSG00000165698	188.4075118	105.0917765	0.841160519	0.000835717	0.002922309	SPACA9
ENSG00000272995	10.21084734	0	5.686677373	0.000836229	0.002923707	AC097505.1
novel.487	5.201791061	41.60325494	-3.001134696	0.000837822	0.002928881	-
ENSG00000188662	26.01250279	2.751966622	3.212291674	0.000838194	0.00292979	HILS1
ENSG00000137288	595.2236344	981.2056488	-0.721235219	0.000838567	0.0029307	UQCC2
ENSG00000233927	3098.270342	7127.74379	-1.202007759	0.000841179	0.00293943	RPS28
ENSG00000175773	15.87687472	0.723690511	4.466209503	0.000841997	0.002941785	AP002986.1
ENSG00000179523	283.755433	147.300876	0.946234041	0.000842079	0.002941785	EIF3J-DT
ENSG00000177409	1351.183478	586.9781321	1.20248424	0.000844251	0.002948977	SAMD9L
novel.104	54.60364171	16.53045616	1.72505475	0.000848014	0.002961722	-
ENSG00000242220	16.66117707	1.456837632	3.52861636	0.000848431	0.002962694	TCP10L
ENSG00000165591	11.80173297	0.356539013	4.93295761	0.00084852	0.002962694	FAAH2
ENSG00000144395	33.45907382	124.0432048	-1.887775925	0.000849286	0.002964971	CCDC150
ENSG00000126838	15.18487237	0.713078025	4.403881008	0.000852226	0.002974833	PZP
ENSG00000111731	915.499605	501.8292266	0.866812237	0.000852711	0.002976128	C2CD5
ENSG00000225098	0.307102255	15.54368405	-5.581364393	0.000852842	0.002976184	BCRP1

ENSG00000256292	0	92.68222418	-9.11511946	0.000853918	0.00297954	LINC02376
ENSG00000197291	73.78141216	7.133092004	3.368371217	0.000854817	0.002982274	RAMP2-AS1
ENSG00000127720	112.3404468	51.28903527	1.132875629	0.000856633	0.002988209	METTL25
ENSG00000170745	406.4563932	87.38463255	2.219258344	0.000857965	0.002992454	KCNS3
ENSG00000145536	45.27698987	4.178122672	3.424066792	0.000858679	0.002994542	ADAMTS16
ENSG00000198888	40414.70941	21451.41499	0.913813157	0.000861312	0.003003323	MT-ND1
ENSG00000145375	134.7281827	299.2982503	-1.152474304	0.000862605	0.003007428	SPATA5
ENSG00000170832	1990.608321	1068.636652	0.897566536	0.000864893	0.003014998	USP32
ENSG00000125037	688.0299862	1178.005031	-0.775976601	0.000867845	0.003024882	EMC3
ENSG00000185596	666.1981343	372.2556797	0.839846031	0.000869283	0.003029489	WASH3P
ENSG00000229191	16.79979114	0.743759607	4.533237686	0.000869985	0.00303153	AL358473.1
ENSG00000211829	15.99807991	0	6.332772438	0.000870567	0.003033151	TRDC
ENSG00000233384	2.138361675	44.720832	-4.388316697	0.000871921	0.003037459	AC096537.1
ENSG00000183807	74.54042807	8.990202861	3.041246298	0.000872317	0.003038301	FAM162B
ENSG00000107798	2494.50653	1389.586883	0.843953255	0.000872396	0.003038301	LIPA
ENSG00000123643	463.5741109	208.4878125	1.151553613	0.000876856	0.003053423	SLC36A1
ENSG00000100554	822.1244748	1580.934719	-0.943493154	0.00087847	0.003058634	ATP6V1D
ENSG00000184678	377.0259788	147.6082869	1.354375051	0.000879225	0.003060854	HIST2H2BE
ENSG00000127955	527.0564777	1016.099129	-0.947062906	0.000883645	0.003075829	GNAI1
ENSG00000181929	949.605078	1493.922666	-0.653712351	0.00088401	0.003076687	PRKAG1
ENSG00000234997	1.17296339	28.64178492	-4.587728739	0.000885502	0.003081468	AC016745.1
ENSG00000256660	15.37753588	0	6.275663607	0.000887493	0.003087981	CLEC12B
ENSG00000156564	17.54738132	0	6.466218479	0.000887955	0.003089174	LRFN2
ENSG00000188649	26.70840828	3.53586442	2.906839862	0.000889943	0.003095676	CC2D2B
ENSG00000197928	271.6253563	164.6648247	0.722535535	0.000890225	0.003096244	ZNF677
ENSG00000138769	44.76440253	5.919395274	2.930675691	0.000890382	0.003096375	CDKL2
ENSG00000101489	17.63026503	1.130980201	4.007796191	0.000892666	0.003103902	CELF4
ENSG00000244625	32.11539969	80.38428424	-1.322262079	0.000894444	0.003109668	MIATNB
ENSG00000113648	1876.683578	2957.36329	-0.656055356	0.00089506	0.003111392	H2AFY
ENSG00000074696	1171.676631	2702.462376	-1.205815185	0.000895325	0.003111897	HACD3
ENSG00000260005	54.60084932	13.28976759	2.039405894	0.000896489	0.003115529	AC027601.2
ENSG00000184828	112.1661162	0	9.143120216	0.000898004	0.003120376	ZBTB7C
novel.707	0	16.02842479	-6.580531531	0.000900525	0.003128715	-
ENSG00000132639	32.5046302	140.6461502	-2.115141581	0.000901026	0.003130039	SNAP25
ENSG00000105671	583.6209476	1147.79509	-0.97603545	0.000901555	0.003131457	DDX49
ENSG00000171443	388.6511037	223.2214939	0.800053247	0.000903363	0.003137319	ZNF524
ENSG00000137101	69.26603974	17.70144616	1.977099928	0.000904581	0.003141127	CD72
ENSG00000204977	564.5436825	351.7750529	0.682453311	0.000906287	0.003146632	TRIM13
ENSG00000272871	45.04439488	10.52847275	2.087845092	0.000906935	0.003148461	AL159169.2
ENSG00000271254	291.9115853	124.5713755	1.228867526	0.000908739	0.003154301	AC240274.1
ENSG00000178988	1663.001666	1071.727974	0.633772475	0.000909687	0.003157169	MRFAP1L1
ENSG00000265800	1.203193503	15.81866241	-3.714735635	0.000910136	0.003158306	AC022211.3
ENSG00000070061	966.1770047	1479.151565	-0.614285441	0.000913021	0.003167895	ELP1
ENSG00000169918	9.945329305	0	5.648284982	0.000914832	0.003173754	OTUD7A
ENSG00000112414	840.5512659	96.49115288	3.121792017	0.000917807	0.003183651	ADGRG6
ENSG00000165244	90.79124858	242.9406505	-1.419976894	0.000918214	0.003184636	ZNF367
ENSG00000151062	41.32900898	8.500196642	2.276687038	0.000921531	0.003195716	CACNA2D4
ENSG00000245648	47.87596911	4.921882279	3.272889356	0.000922409	0.003198334	AC022075.1
ENSG00000131469	7052.006675	12917.71711	-0.873256532	0.000922537	0.003198351	RPL27
ENSG00000179698	100.0778546	30.81670927	1.699661012	0.000923635	0.003201728	WDR97
ENSG00000116001	2529.814194	1435.226129	0.817826565	0.000926158	0.003210048	TIA1

ENSG00000198393	467.7968713	216.137226	1.113762041	0.000927575	0.003214529	ZNF26
ENSG00000108219	1416.936926	3281.427834	-1.211479073	0.000927731	0.003214642	TSPAN14
ENSG00000207611	15.21990361	0	6.262387084	0.000928795	0.003217899	MIR149
ENSG00000158623	373.8462152	713.2751253	-0.9323783	0.000929526	0.003219599	COPG2
ENSG00000225422	31.42325479	94.79583221	-1.594738659	0.000929534	0.003219599	RBMS1P1
ENSG00000163016	18.01147545	1.854670712	3.309689285	0.000929874	0.003220348	ALMS1P1
ENSG00000116459	1707.787593	2824.846558	-0.726163221	0.000931667	0.003226129	ATP5PB
ENSG00000243224	4.602701607	27.58650479	-2.58890218	0.00093256	0.003228792	AC006252.1
ENSG00000102245	10.09898716	0	5.668694131	0.000932822	0.003229267	CD40LG
ENSG00000102158	1280.081467	1919.956706	-0.584723889	0.000933488	0.003230749	MAGT1
ENSG00000013503	137.2100737	294.3644372	-1.102418124	0.000933499	0.003230749	POLR3B
ENSG00000267128	20.92250063	0.774441189	4.828021371	0.000933953	0.003231891	RNF157-AS1
ENSG00000170653	709.9658958	454.0742923	0.645193559	0.000934506	0.003233374	ATF7
ENSG00000230291	18.02186065	66.23530397	-1.880133896	0.000938472	0.003246662	AC078817.1
ENSG00000183628	15.52471204	0.672939833	4.457316756	0.000938618	0.003246737	DGCR6
ENSG00000260992	17.86938517	2.884149558	2.635287929	0.000941882	0.003257592	DOCK9-DT
ENSG00000204241	74.92075681	2.71054416	4.814609481	0.000942826	0.003260423	AP000911.1
ENSG00000205903	1506.758324	773.2659816	0.962616745	0.000947463	0.003276022	ZNF316
ENSG00000163145	51.26996408	5.463795051	3.211245234	0.000948328	0.003278578	C1QTNF7
ENSG00000166855	721.6129154	1167.080895	-0.693701	0.000950146	0.003284425	CLPX
ENSG00000171105	858.5512345	382.5913642	1.166838252	0.000950896	0.003286581	INSR
ENSG00000184985	535.391218	212.9796863	1.328867201	0.00095144	0.003288025	SORCS2
ENSG00000135720	3541.691507	2082.798671	0.765979156	0.000952468	0.00329114	DYNC1LI2
ENSG00000197323	1046.667728	579.4258412	0.853412367	0.000952659	0.003291362	TRIM33
ENSG00000188938	1249.522748	623.6992706	1.001935665	0.000952805	0.003291428	FAM120AOS
ENSG00000171561	11.84213482	0	5.899360103	0.000953995	0.003295101	OR2AT4
ENSG00000182621	359.4323264	61.61635034	2.541843296	0.000958974	0.003311858	PLCB1
ENSG00000200714	18.1085053	2.496928964	2.853941793	0.000961948	0.003321685	RF00019
ENSG00000090863	4045.23304	7478.401407	-0.88646179	0.000962409	0.003322837	GLG1
ENSG00000166104	0.293240848	19.20659433	-5.879960061	0.000964545	0.003329769	AC126323.1
ENSG00000196504	1594.524615	2510.977756	-0.655228798	0.000965515	0.003332676	PRPF40A
ENSG00000196867	354.5948507	170.6836664	1.054965243	0.000967313	0.003338438	ZFP28
ENSG00000008018	2316.571426	3397.140275	-0.552313856	0.000968196	0.00334104	PSMB1
ENSG00000174928	48.69716926	18.06872605	1.434467226	0.000973802	0.00335994	C3orf33
ENSG00000101040	1016.891616	2048.758998	-1.010420011	0.000975041	0.003363767	ZMYND8
ENSG00000182022	543.1967167	185.416881	1.550757927	0.000976618	0.003368763	CHST15
ENSG00000249776	26.73620236	3.801514564	2.789311853	0.000976867	0.003369174	AC124854.1
ENSG00000172602	22.22788077	1.753169357	3.646312015	0.000977309	0.00337025	RND1
ENSG00000179859	164.0087451	62.1066477	1.402989722	0.000977869	0.003371734	RNF227
ENSG00000243431	26.66535694	4.045811341	2.737561709	0.000980055	0.003378821	RPL5P30
ENSG00000278266	0	10.60630899	-5.986182064	0.000980709	0.003380628	AC079949.2
ENSG00000173262	1.17296339	20.19721925	-4.079509682	0.000982541	0.003386495	SLC2A14
ENSG00000100294	215.4999225	364.3719872	-0.758087423	0.000984604	0.003393156	MCAT
ENSG00000266378	0.601596751	14.9166146	-4.623573588	0.000987912	0.003404105	AC005224.2
ENSG00000070718	680.1740435	388.6975115	0.807571322	0.000988734	0.003406484	AP3M2
ENSG00000143507	113.5207182	248.1453327	-1.127850567	0.000990081	0.003410674	DUSP10
ENSG00000115593	9.708787736	0	5.612786716	0.000991096	0.003413716	SMYD1
novel.235	9.72508517	0	5.615003882	0.000991322	0.003414023	-
ENSG00000248698	0.586481695	19.11968666	-5.000512516	0.000991448	0.003414023	LINC01085
ENSG00000101353	62.95697392	23.31890591	1.425984525	0.000991675	0.003414352	MROH8
ENSG00000139239	392.611451	639.5367866	-0.703765805	0.000991965	0.0034149	RPL14P1

ENSG00000223745	629.5280281	325.0146731	0.954204986	0.000992643	0.003416782	CCDC18-AS1
ENSG00000132958	11.46063966	0.356539013	4.891250379	0.000993143	0.003418048	TPTE2
ENSG00000236287	1243.177457	705.3811583	0.817336521	0.00099328	0.003418067	ZBED5
ENSG00000242686	8.761011805	0	5.465145995	0.000994309	0.003421155	AC107464.1
ENSG00000104723	413.7269382	2175.94135	-2.39502904	0.00099762	0.003432094	TUSC3
ENSG00000104907	772.5065296	1306.15157	-0.757690759	0.000997893	0.003432578	TRMT1
ENSG00000157613	220.9584391	1695.057337	-2.939649577	0.000998689	0.003434861	CREB3L1
ENSG00000070540	644.2785375	1534.290469	-1.251578296	0.000999729	0.003437981	WIPI1
ENSG00000126778	83.77613231	974.6565243	-3.540651365	0.001000064	0.00343868	SIX1
ENSG00000182359	142.7539983	59.38284131	1.268897924	0.00100033	0.003439138	KBTBD3
ENSG00000279141	12.40709067	0.336469917	5.005105605	0.00100183	0.003443841	AL590226.2
ENSG00000168564	495.1008056	295.8065056	0.742401053	0.001002695	0.003446359	CDKN2AIP
ENSG00000161980	140.8474483	326.324199	-1.213216691	0.001004873	0.003453387	POLR3K
ENSG00000277559	2.463086283	21.89159398	-3.164915396	0.001009778	0.003469786	AC018553.1
ENSG00000104973	529.5388547	858.4094252	-0.696705127	0.001011652	0.003475763	MED25
ENSG00000214975	31.82591979	72.98708315	-1.198684333	0.001014419	0.00348481	PPIAP29
ENSG00000104866	527.5069388	915.8336415	-0.795570944	0.001015319	0.003487439	PPP1R37
ENSG00000214114	145.5777659	331.5035935	-1.188018026	0.001015936	0.003489099	MYCBP
ENSG00000179593	3.699017196	57.83378335	-3.971187565	0.001016938	0.003492078	ALOX15B
ENSG00000133193	206.7461757	391.6690011	-0.922145809	0.001020111	0.003502513	FAM104A
ENSG00000185442	91.01804534	26.32591019	1.783935703	0.001020471	0.003503284	FAM174B
ENSG00000285964	0	11.00902236	-6.045849001	0.001021336	0.003505791	AC002074.2
ENSG00000196236	264.713697	460.7743405	-0.799348362	0.001023033	0.003511153	XPNPEP3
ENSG00000138614	481.3230021	740.9152877	-0.622273832	0.001023279	0.003511533	INTS14
ENSG00000163558	605.3943368	950.9625819	-0.651545638	0.001024071	0.003513786	PRKCI
novel.272	8.738303585	0	5.461763357	0.00102583	0.003519357	-
ENSG00000224914	115.5346123	37.91223162	1.602356166	0.001027247	0.003523752	LINC00863
ENSG00000168476	561.2614601	1061.137402	-0.918882137	0.001031203	0.003536856	REEP4
ENSG00000088038	22.51868559	75.50587938	-1.745013714	0.001036556	0.003554748	CNOT3
ENSG00000101977	20.48870788	1.069617038	4.252046321	0.001039695	0.003564753	MCF2
ENSG00000006704	289.5604735	527.3465811	-0.864294299	0.001039748	0.003564753	GTF2IRD1
ENSG00000157353	538.7168198	312.7049311	0.784440499	0.001040183	0.003565773	FCSK
ENSG00000214694	43.69187393	8.633663848	2.3223104	0.001044427	0.003579848	ARHGEF33
ENSG00000286861	14.7517638	0.693008929	4.374107979	0.001044939	0.00358113	AL049779.4
ENSG00000174325	9.136096177	0	5.524558838	0.001045477	0.003582502	DIRC1
ENSG00000257365	416.6794502	163.7629525	1.347027502	0.001045629	0.003582552	FNTB
ENSG00000141627	489.3455119	906.3645925	-0.889093839	0.001045857	0.003582861	DYM
ENSG00000085832	1502.42176	957.2810608	0.650275582	0.001046382	0.003584185	EPS15
ENSG00000120802	867.8907197	1834.98587	-1.08036302	0.001049181	0.003593301	TMPO
ENSG00000236682	85.58092327	18.54940406	2.199295746	0.001049691	0.003594573	MAP3K2-DT
ENSG00000135447	12.57976707	0	5.985826957	0.001052333	0.003603144	PPP1R1A
ENSG00000272462	90.39270053	26.14413245	1.782199354	0.001052955	0.003604799	U91328.1
ENSG00000007376	380.6178093	659.6032487	-0.793259772	0.001054395	0.003609254	RPUSD1
ENSG00000236756	15.74363125	2.160459047	2.868447067	0.001056118	0.003614675	DNAJC9-AS1
ENSG00000198028	0	7.827256809	-5.551164459	0.001056644	0.003615999	ZNF560
ENSG00000159450	14.49376766	0.387220594	5.229574858	0.001060184	0.003627636	TCHH
ENSG00000226396	37.91767605	5.909938664	2.694199943	0.001061187	0.003630591	AL031727.1
ENSG00000167110	2169.122338	3446.682768	-0.668014612	0.00106225	0.003633747	GOLGA2
ENSG00000025708	1580.357179	601.7181626	1.393478891	0.00106323	0.003636622	TYMP
ENSG00000005187	52.71102407	10.80473538	2.275168252	0.001063487	0.003637024	ACSM3
ENSG00000136732	704.1816361	1990.286878	-1.498984777	0.00106537	0.003642984	GYPC

ENSG00000155906	260.6013293	502.9982904	-0.949264724	0.001065623	0.003643367	RMND1
ENSG00000165682	16.00434815	0	6.333360787	0.001070284	0.003658822	CLEC1B
ENSG00000062194	1894.329785	1242.136393	0.609013038	0.001071889	0.003663827	GPBP1
ENSG00000168282	17.07283107	53.97506241	-1.664994684	0.00107349	0.003668816	MGAT2
ENSG00000205710	3.36043118	23.39789802	-2.804655257	0.001074768	0.0036727	C17orf107
ENSG00000284719	21.95720595	1.161661783	4.302790994	0.001075262	0.003673907	AL033527.5
ENSG00000271367	15.77135407	1.396630344	3.471420105	0.001078112	0.003683158	AL034374.1
ENSG00000109046	11739.4165	3516.584178	1.739154328	0.001079902	0.003688789	WSB1
ENSG00000138738	354.7179656	147.6340066	1.264757648	0.001082439	0.003696969	PRDM5
ENSG00000181016	61.26404384	23.0319024	1.413268058	0.001084153	0.003702336	LSMEM1
ENSG00000103150	311.2803112	177.6433286	0.808227548	0.001090266	0.003722722	MLYCD
ENSG00000101158	1008.247734	1694.525057	-0.748877959	0.001090532	0.003723143	NELFCD
ENSG00000184640	4940.702639	7735.115074	-0.646691007	0.001090715	0.003723277	SEPT9
ENSG00000104408	3115.177477	5652.881708	-0.859686815	0.001090879	0.003723349	EIF3E
ENSG00000163686	338.3968107	147.5238194	1.198074376	0.001093326	0.003731211	ABHD6
novel.765	54.67051272	241.7055189	-2.144828037	0.00109402	0.003733089	-
ENSG00000240654	9.876022268	0	5.638941626	0.001094458	0.003734094	C1QTNF9
novel.1066	62.93802665	23.34945909	1.431235339	0.001094756	0.00373462	-
ENSG00000140416	2284.065749	8174.947859	-1.83963065	0.00109962	0.003750718	TPM1
ENSG00000089639	540.8261609	236.0983551	1.196832679	0.001105495	0.003770264	GMIP
ENSG00000136379	110.0090218	414.1726857	-1.913105225	0.001107777	0.003777549	ABHD17C
ENSG00000010244	1837.037328	2905.786998	-0.661631969	0.001108301	0.00377884	ZNF207
ENSG00000107371	278.2094281	504.5439279	-0.859344866	0.001109431	0.003782137	EXOSC3
ENSG00000223821	11.56017681	0.356539013	4.902235717	0.001109609	0.003782137	AL603910.1
ENSG00000163743	405.8668796	235.1314894	0.787025585	0.001109704	0.003782137	RCHY1
ENSG00000148572	377.0095815	626.0215886	-0.731298113	0.001112177	0.003790067	NRBF2
novel.665	46.6230722	1.548882377	4.95111094	0.001113343	0.003793542	-
ENSG00000181450	193.4721563	85.69865258	1.172337396	0.001114133	0.003795735	ZNF678
ENSG00000078081	34.1269996	213.283116	-2.644913864	0.001115567	0.003800123	LAMP3
ENSG00000099365	22.75655208	118.2100281	-2.375834283	0.001119006	0.003811338	STX1B
ENSG00000130396	322.1281968	1055.161228	-1.711850699	0.001119604	0.003812875	AFDN
ENSG00000102878	141.6995902	55.10920626	1.365717303	0.001120154	0.00381421	HSF4
ENSG00000230091	93.10658889	20.73926041	2.166094101	0.001120289	0.00381421	TMEM254-AS1
ENSG00000197912	1985.160769	3502.580043	-0.819055071	0.001124173	0.003826932	SPG7
novel.792	231.047299	1069.197546	-2.210555233	0.00112472	0.003827691	-
ENSG00000253123	10.66652159	0	5.748132448	0.001124759	0.003827691	AC091182.1
ENSG00000259943	178.3200926	84.17403044	1.084633095	0.001124838	0.003827691	AL050341.2
ENSG00000139899	85.41269199	188.6203382	-1.141617883	0.001126676	0.003832639	CBLN3
ENSG00000130479	628.8258811	1216.786245	-0.952059915	0.001126693	0.003832639	MAP1S
ENSG00000272368	39.57360957	6.520359459	2.605330198	0.001126735	0.003832639	AC074032.1
ENSG00000163393	63.12965032	144.2788925	-1.193301141	0.001127935	0.003836219	SLC22A15
ENSG00000101189	390.2742285	720.9234176	-0.884834653	0.001129417	0.003840758	MRGBP
ENSG00000124596	619.0149353	349.4643156	0.824863813	0.001130286	0.003843209	OARD1
ENSG00000238266	3.620934617	27.37789827	-2.92110926	0.001130685	0.003844062	LINC00707
ENSG00000139579	502.9497847	860.2224291	-0.77463962	0.001132027	0.00384812	NABP2
ENSG00000176826	9.05675995	48.30150497	-2.417043652	0.001133667	0.003853192	FKBP9P1
ENSG00000196557	71.7949565	9.447087354	2.918743739	0.001134631	0.003855965	CACNA1H
ENSG00000175356	55.75091886	10.43629961	2.41771395	0.001134832	0.003856143	SCUBE2
ENSG00000268812	1.186824798	14.85871906	-3.626457735	0.001136226	0.003860374	AC004264.1
ENSG00000227934	1.801029308	16.25419353	-3.165726667	0.001137017	0.003862558	AL732414.1
ENSG00000101216	344.06841	586.8363177	-0.769846108	0.001139914	0.003871893	GMEB2

ENSG00000280194	23.18547243	2.261960402	3.392263034	0.001140358	0.003872515	AD000864.1
ENSG00000143368	840.0384378	1484.063083	-0.821192344	0.001140395	0.003872515	SF3B4
ENSG00000236204	35.98888858	7.652495535	2.242400337	0.001142484	0.0038791	LINC01376
ENSG00000151240	1601.602619	782.2560901	1.034010627	0.001143752	0.003882898	DIP2C
ENSG00000234175	26.0666235	5.513261459	2.257115707	0.001144606	0.003884362	AL355355.2
ENSG00000172824	90.26390218	36.1576496	1.319812975	0.001144612	0.003884362	CES4A
ENSG00000229956	35.77254793	8.856735655	2.008138501	0.001144632	0.003884362	ZRANB2-AS2
ENSG00000085117	871.2853109	3867.772114	-2.150252593	0.001152606	0.003910911	CD82
ENSG00000164105	231.5060663	400.3689896	-0.78993111	0.001154213	0.003915852	SAP30
ENSG00000134070	139.0072712	551.1130995	-1.986840543	0.001155502	0.003919715	IRAK2
ENSG00000224152	69.13454846	18.61192309	1.885884523	0.001155659	0.003919735	AC009506.1
ENSG00000160097	433.4634383	7.879291756	5.777626092	0.001156878	0.003923359	FNDC5
ENSG00000150990	310.7240596	627.4956141	-1.014356753	0.001158675	0.003928718	DHX37
ENSG00000252464	14.46736977	1.10029862	3.7391089	0.001158761	0.003928718	RN7SKP70
ENSG00000126003	205.7315722	362.0054773	-0.814508346	0.001160163	0.003932957	PLAGL2
ENSG00000033011	213.1631982	478.8485545	-1.168326132	0.001167016	0.003955675	ALG1
ENSG00000132950	409.9603431	192.4021689	1.092349783	0.001169343	0.003963046	ZMYM5
ENSG00000238113	301.0423268	103.3268388	1.542660271	0.001172417	0.003972944	LINC01410
ENSG00000259158	18.41804359	2.547679642	2.865966673	0.001173488	0.003976054	ADAM20P1
ENSG00000254319	0.307102255	11.27107648	-5.117158396	0.001173676	0.003976173	AC246817.2
ENSG00000156466	10.73074276	140.3937751	-3.711707052	0.001174105	0.003977108	GDF6
ENSG00000129646	232.3116098	103.5287328	1.16491562	0.001176341	0.003984163	QRICH2
ENSG00000143164	885.6073284	1572.54465	-0.828341267	0.001177002	0.003985882	DCAF6
ENSG00000198835	142.8685517	38.60639642	1.884496616	0.00117761	0.00398742	GJC2
ENSG00000135111	202.624391	944.6971792	-2.221297755	0.001179241	0.003992424	TBX3
ENSG00000121644	589.7166351	992.0308722	-0.750698592	0.001181039	0.003997992	DESI2
ENSG00000226308	11.59262948	0	5.8703452	0.001183021	0.004004179	AL122058.1
ENSG00000153714	161.2219031	78.54266616	1.038639425	0.001184283	0.004007808	LURAP1L
ENSG00000147677	2227.764307	3622.143458	-0.701260666	0.001184402	0.004007808	EIF3H
ENSG00000259146	17.56102926	51.59037568	-1.557904944	0.001184879	0.004008901	AC005476.2
ENSG00000198858	725.9445859	1193.552101	-0.717601499	0.001185616	0.004010871	R3HDM4
ENSG00000262001	37.70488288	100.7985863	-1.418007425	0.001188307	0.004019451	DLGAP1-AS2
ENSG00000154511	98.29991697	264.5984662	-1.429232605	0.00118898	0.004021203	DIPK1A
ENSG00000113555	122.168118	41.30833588	1.568263058	0.00119002	0.004024196	PCDH12
ENSG00000038427	988.0352765	4089.173111	-2.049247998	0.001190363	0.004024834	VCAN
ENSG00000034533	176.7775298	77.87242326	1.181309708	0.00119083	0.004025889	ASTE1
ENSG00000101343	400.8316385	686.1264634	-0.775891961	0.001194857	0.004038977	CRNKL1
ENSG00000197448	2879.916945	1625.892295	0.824624555	0.001196011	0.004041866	GSTK1
ENSG00000125843	188.6019984	361.7064615	-0.94025669	0.001196023	0.004041866	AP5S1
ENSG00000279283	1.21705491	12.62628437	-3.37837568	0.001196817	0.004044025	AC131009.4
ENSG00000143442	2600.165245	1193.974027	1.12301101	0.001197004	0.004044131	POGZ
ENSG00000253106	72.93092368	25.66815916	1.509569211	0.001198852	0.004049849	AC090198.1
ENSG00000253288	14.13666165	0	6.154273879	0.001200239	0.004054006	AC046195.1
ENSG00000111962	1103.955855	403.132665	1.453967944	0.001200877	0.004055633	UST
ENSG00000215193	741.9203292	1122.081104	-0.596641704	0.00120694	0.004075578	PEX26
ENSG00000233894	8.705566175	0	5.456829002	0.001207298	0.004076259	AC105271.1
ENSG00000213057	36.45348091	9.111773312	1.988235421	0.001208565	0.004080006	C1orf220
ENSG00000196363	867.3112621	1467.47438	-0.758835048	0.001208808	0.004080296	WDR5
ENSG00000130822	32.41706007	5.430673325	2.582693914	0.001209387	0.004081721	PNCK
ENSG00000287789	11.42783098	0	5.84875879	0.001210282	0.004084211	AL158136.1
ENSG00000162729	1396.929112	678.685212	1.041936341	0.001211261	0.004086746	IGSF8

ENSG00000130363	140.8939046	287.3045452	-1.027930697	0.001211348	0.004086746	RSPH3
ENSG00000168447	26.46072606	0.743759607	5.181330459	0.001214183	0.004095778	SCNN1B
ENSG00000229388	22.00881936	2.751966622	2.969424809	0.001216825	0.004104159	LINC01715
ENSG00000039523	1139.358514	1684.951005	-0.564464511	0.001217745	0.00410673	RIPOR1
ENSG00000154898	23.80358043	3.179325408	2.893939876	0.00121893	0.004110192	CCDC144CP
ENSG00000175387	1963.377277	1218.900413	0.688009453	0.001224759	0.004129308	SMAD2
ENSG00000243953	0	10.91312481	-6.024803339	0.001226053	0.004133137	AC073359.1
ENSG00000272459	17.61271395	2.853467977	2.619765511	0.001230131	0.004146347	AC139795.3
ENSG00000169933	7.362789684	39.28622433	-2.421413495	0.001234099	0.004159182	FRMPD4
ENSG00000239306	602.0798912	1031.00713	-0.776366527	0.001234762	0.004160875	RBM14
ENSG00000275022	0.909952655	13.57317508	-3.901698971	0.001235716	0.004163549	MIR6753
ENSG00000176390	469.8503527	265.9338617	0.820981841	0.001236416	0.004165369	CRLF3
ENSG00000156959	19.0823944	0.774441189	4.69593006	0.001237585	0.004168767	LHFPL4
ENSG00000156218	737.1950433	9.265309615	6.311252636	0.001245561	0.00419509	ADAMTSL3
ENSG00000248592	25.16670003	5.513261459	2.206754451	0.001246153	0.004196539	STIMATE-MUSTN1
ENSG00000261305	11.98916843	0.336469917	4.957079942	0.001247288	0.004199817	AC005586.2
ENSG00000084444	93.94880107	232.9770537	-1.309821809	0.001247549	0.004200111	FAM234B
ENSG00000126524	3019.587356	1622.094505	0.896307297	0.001247699	0.004200111	SBDS
ENSG00000105270	2135.617068	992.6716459	1.10559317	0.001248741	0.004203075	CLIP3
ENSG00000272356	31.13614	8.601697997	1.855520232	0.001250858	0.004209657	AL080317.3
ENSG00000245571	96.170189	37.1150244	1.374580611	0.001251399	0.00421093	FAM111A-DT
ENSG00000204876	9.30340198	0	5.552533472	0.001251613	0.004211104	AC021218.1
ENSG00000075336	400.0558273	754.7066327	-0.916166799	0.001253298	0.00421623	TIMM21
novel.643	111.5911757	52.53328518	1.090306805	0.001255884	0.004224381	-
novel.462	41.36364042	208.6492175	-2.33545661	0.001256275	0.004225036	-
ENSG00000229944	81.47344355	169.6858966	-1.058349095	0.001256404	0.004225036	EIF4EP2
ENSG00000273007	41.10403498	13.63453824	1.592064232	0.001257901	0.004229525	AC021205.3
ENSG00000125144	2.446717578	21.78294777	-3.163385433	0.001260287	0.004236998	MT1G
ENSG00000182165	186.6649336	82.62660791	1.173352293	0.001264791	0.004251589	TP53TG1
ENSG00000173275	432.7104569	169.9990866	1.347863225	0.001267438	0.004259472	ZNF449
ENSG00000273143	1.203193503	13.77733366	-3.509316418	0.001267464	0.004259472	AL355512.1
ENSG00000231170	36.50516559	3.72123818	3.266232829	0.001275594	0.004286241	AC002451.1
ENSG00000186715	51.21101475	8.314822883	2.626088325	0.001277905	0.004293451	MST1L
ENSG00000151948	12.909435	0	6.023120308	0.001278505	0.004294911	GLT1D1
ENSG00000141441	305.5720867	147.8929786	1.048943077	0.001283633	0.004311578	GAREM1
ENSG00000196535	1922.407755	929.3838146	1.048869104	0.00128391	0.004311953	MYO18A
ENSG00000210077	21.30768546	2.782648203	2.915317768	0.001285058	0.004315249	MT-TV
ENSG00000160094	929.8195677	423.1429132	1.135131275	0.001291128	0.004335072	ZNF362
ENSG00000197142	215.0470675	58.47133689	1.881203861	0.001291523	0.00433584	ACSL5
ENSG00000133256	149.9618052	19.68051265	2.924626951	0.001293759	0.004342785	PDE6B
ENSG00000254027	24.12948741	4.912425669	2.281192996	0.001295539	0.004347816	AC009902.2
ENSG00000065548	971.3000847	1622.759632	-0.740678482	0.001295612	0.004347816	ZC3H15
ENSG00000143436	381.2294634	645.8236504	-0.760770584	0.00129576	0.004347816	MRPL9
ENSG00000215186	17.73001599	0	6.481496376	0.001296856	0.004350933	GOLGA6B
ENSG00000137941	736.9703142	238.8829768	1.625558461	0.001298081	0.00435416	TTLL7
ENSG00000103194	994.1494944	1573.676515	-0.662455571	0.001298153	0.00435416	USP10
ENSG00000111885	699.5291883	1881.569384	-1.427462043	0.001299239	0.004357238	MAN1A1
ENSG00000181830	629.3779726	954.2995667	-0.600629782	0.001300489	0.004360868	SLC35C1
ENSG00000268062	14.61350573	1.110911105	3.748001064	0.001301604	0.004364043	SRP14P4
ENSG00000273487	20.25947474	1.345879667	3.860439671	0.001306249	0.004379053	AC104836.1
ENSG00000240875	22.14610852	89.05636965	-2.006174359	0.001308267	0.004385253	LINC00886

ENSG00000135677	1980.910591	3772.311809	-0.929320578	0.001308608	0.004385829	GNS
ENSG00000183520	535.6495152	951.9254241	-0.829856708	0.001310847	0.004392765	UTP11
ENSG00000198001	682.0957791	359.5501933	0.923129924	0.001313629	0.004401522	IRAK4
ENSG00000113716	1314.554904	2316.375804	-0.8171394	0.001326119	0.004442797	HMGXB3
ENSG00000152404	387.3718211	195.8076482	0.983752526	0.001328051	0.004448697	CWF19L2
ENSG00000156876	89.72312166	162.5281592	-0.857035583	0.001330917	0.004457724	SASS6
ENSG00000120549	689.4801255	247.0845768	1.480096347	0.001331714	0.004459652	KIAA1217
ENSG00000155438	396.2739973	701.5361323	-0.824537933	0.001331836	0.004459652	NIFK
ENSG00000163808	9.795645855	67.60926264	-2.789606694	0.001333736	0.004465437	KIF15
ENSG00000129480	60.68536877	147.2473468	-1.280801666	0.001334564	0.004467636	DTD2
novel.1096	17.56486148	1.161661783	3.986008097	0.001334806	0.004467869	-
ENSG00000148468	1274.460702	743.3458916	0.777676065	0.001335427	0.004469372	FAM171A1
ENSG00000114473	225.908804	127.640946	0.822477779	0.00133652	0.004472454	IQCG
ENSG00000176105	606.552197	1279.37357	-1.076788386	0.001337523	0.004475234	YES1
ENSG00000136383	242.4358277	80.05996787	1.59834554	0.001338868	0.004479156	ALPK3
ENSG00000278540	26.05401574	82.6513005	-1.666834648	0.001342487	0.004490684	ACACA
ENSG00000233223	169.3692089	94.31875023	0.845441728	0.001342991	0.004491795	AC016876.1
ENSG00000227542	0.615458159	11.86129979	-4.285809188	0.001343347	0.004492406	AC092614.1
ENSG00000108055	954.8763899	1395.501689	-0.547512832	0.001344863	0.004496896	SMC3
ENSG00000283696	14.54545234	0.713078025	4.342341432	0.001345826	0.004499537	AL592295.4
ENSG00000170962	1725.656529	52.50342115	5.037799828	0.001346147	0.004500032	PDGFD
ENSG00000254333	8.209774817	0	5.37045076	0.001349386	0.004510224	NDST1-AS1
ENSG00000145041	407.5510772	639.9669238	-0.650619794	0.001349543	0.004510224	DCAF1
ENSG00000284723	12.15138836	0	5.938294805	0.001355617	0.00452994	OR8S1
ENSG00000197584	9.175173102	0	5.530286002	0.001356666	0.004532863	KCNMB2
ENSG00000099624	1246.614285	2119.10411	-0.765495427	0.001357955	0.004536258	ATP5F1D
ENSG00000110048	1012.781541	1625.629758	-0.682498738	0.001358076	0.004536258	OSBP
ENSG00000196914	4421.832274	2589.452016	0.771990971	0.001358206	0.004536258	ARHGEF12
ENSG00000249797	15.69975319	0	6.30565327	0.001360833	0.004544447	LINC02147
ENSG00000070761	1013.100939	608.7749035	0.734553396	0.001363941	0.00455424	CFAP20
ENSG00000165269	12.50669909	0.387220594	5.015616975	0.001366868	0.004563429	AQP7
ENSG00000141562	542.1604046	1256.714633	-1.212711496	0.00136934	0.004571093	NARF
ENSG00000173811	15.10170393	1.773238453	3.079581236	0.00137136	0.004577249	CCDC13-AS1
ENSG00000131389	470.8731602	1498.439879	-1.67006	0.001371788	0.00457809	SLC6A6
ENSG00000186479	14.91559339	0.774441189	4.345873508	0.001372346	0.004579362	RGS7BP
ENSG00000179431	343.6831647	1317.29551	-1.938673849	0.001376022	0.00459104	FJX1
ENSG00000254486	0	9.65941828	-5.849338702	0.001383136	0.004614182	LINC02547
novel.680	85.73802951	23.75931732	1.845047683	0.001384839	0.004619246	-
ENSG00000197119	1909.018835	732.4496938	1.382173761	0.001385009	0.004619246	SLC25A29
ENSG00000000003	876.2071097	469.554781	0.899634076	0.001386247	0.004622575	TSPAN6
ENSG00000165169	1050.422985	634.4697503	0.727694432	0.001386363	0.004622575	DYNLT3
ENSG00000160856	12.28136909	0	5.95117489	0.001387476	0.00462569	FCRL3
ENSG00000140105	4030.959972	2161.02373	0.899301149	0.001388001	0.004626847	WARS
ENSG00000261123	4.792644359	24.79465676	-2.367631089	0.001388336	0.004627371	AC009065.5
ENSG00000114942	224.5101122	577.7560563	-1.364004275	0.001388755	0.004628173	EEF1B2
ENSG00000080947	314.4495898	146.1085326	1.106126416	0.001389469	0.004629961	CROCCP3
ENSG00000184083	379.5801976	215.5680049	0.816707753	0.001391264	0.004635345	FAM120C
ENSG00000249906	11.61909865	0	5.873385666	0.001393361	0.004641736	AC006487.1
ENSG00000123505	892.870638	1839.892586	-1.043268206	0.001396358	0.004650567	AMD1
ENSG00000254685	308.412223	163.0101215	0.918397723	0.001396369	0.004650567	FPGT
ENSG00000226539	8.222382576	0	5.372455067	0.001396856	0.004651591	AC012512.1

ENSG00000186777	2.405133356	17.96735309	-2.899982333	0.001397481	0.004653076	ZNF732
ENSG00000101342	22.06265534	1.161661783	4.30802011	0.001397979	0.004654139	TLDC2
ENSG00000102802	210.2412017	1215.49433	-2.531662854	0.001399655	0.004659121	MEDAG
ENSG00000198203	47.57868224	5.391691008	3.141726065	0.001401779	0.004665592	SULT1C2
ENSG00000004776	184.4049232	1281.771424	-2.797455013	0.001403583	0.00467092	HSPB6
ENSG00000183850	7.228007828	27.74834182	-1.936710112	0.001403739	0.00467092	ZNF730
ENSG00000241399	170.9538538	50.9645905	1.744673635	0.001406968	0.004681066	CD302
ENSG00000234899	16.3288593	1.844058227	3.170091172	0.001411374	0.004695123	SOX9-AS1
ENSG00000017483	84.38976805	476.2701714	-2.497044534	0.001414832	0.004706023	SLC38A5
ENSG00000196352	543.4088716	1021.741949	-0.911264482	0.00141757	0.004714528	CD55
ENSG00000241468	1130.864045	1995.650649	-0.819420653	0.001420683	0.004724277	ATP5MF
ENSG00000122188	11.36264089	0	5.838854745	0.001422857	0.004730817	LAX1
ENSG00000099994	105.9432681	978.2568725	-3.20721067	0.001423135	0.004730817	SUSD2
ENSG00000197780	488.1632313	879.2258795	-0.849060675	0.001423196	0.004730817	TAF13
ENSG00000253873	34.07850639	150.700028	-2.145344317	0.001426782	0.00474213	PCDHGA11
ENSG00000102218	610.6844253	364.0334623	0.745868819	0.001427639	0.004744369	RP2
ENSG00000146733	249.2525498	531.5147121	-1.093115402	0.001428461	0.004746496	PSPH
ENSG00000164778	24.90272038	0.672939833	5.154155747	0.001434054	0.00476447	EN2
ENSG00000213213	41.28394854	13.02296157	1.669457544	0.001438121	0.004777371	CCDC183
ENSG00000130255	4935.988015	9143.576921	-0.889437602	0.00143881	0.004779048	RPL36
ENSG00000229272	11.98311364	0	5.915625431	0.001443471	0.004793476	AL157388.1
ENSG00000174891	205.4467791	349.411206	-0.766101583	0.001443522	0.004793476	RSRC1
ENSG00000224043	75.49236474	27.61500301	1.450780176	0.001444562	0.004796317	CCNT2-AS1
ENSG00000105321	521.5083093	795.9343757	-0.60968148	0.001446337	0.004801594	CCDC9
ENSG00000111012	4.821620823	23.41783872	-2.272769925	0.001449787	0.004811967	CYP27B1
ENSG00000140932	8.223636224	0	5.372663198	0.001449832	0.004811967	CMTM2
ENSG00000284430	6.654133891	33.55421059	-2.330022888	0.001450949	0.004814445	AC020912.1
ENSG00000250067	113.6054688	39.04038649	1.545248648	0.001450949	0.004814445	YJEFN3
ENSG00000239474	30.65643261	5.22754222	2.574207272	0.001453304	0.004821303	KLHL41
ENSG00000280286	24.70056932	2.241891306	3.489398092	0.001453561	0.004821303	FRG1KP
ENSG00000158985	735.5081902	351.4888198	1.064819485	0.001453573	0.004821303	CDC42SE2
ENSG00000259920	11.91763883	0.356539013	4.945680292	0.001454981	0.004825358	AC007938.1
ENSG00000166987	1086.516414	654.4412476	0.731579752	0.001455801	0.004827463	MBD6
ENSG00000126464	653.3814537	998.1338305	-0.61115225	0.001458701	0.004836461	PRR12
ENSG00000135919	4767.508808	26026.76161	-2.448701872	0.001459559	0.004838688	SERPINE2
ENSG00000254612	101.4474128	222.5620262	-1.132871453	0.001463046	0.004849629	AP001000.1
ENSG00000117600	59.52934391	5.800264968	3.343584969	0.001463813	0.004851552	PLPPR4
ENSG00000246379	11.05177769	0	5.798817047	0.00146456	0.004853408	AC040168.1
ENSG00000167470	1056.211161	2251.077364	-1.091655093	0.001466642	0.004859689	MIDN
ENSG00000070444	395.0954933	660.7013039	-0.741314667	0.001468203	0.004864241	MNT
ENSG00000173726	3098.150069	4696.972969	-0.600327129	0.001469998	0.004869568	TOMM20
ENSG00000188266	126.8231172	54.21807491	1.226662931	0.001470502	0.004870615	HYKK
ENSG00000188933	9.829779456	135.4801339	-3.786258773	0.001471214	0.004872354	USP32P1
ENSG00000234585	129.5089831	51.2477412	1.338573919	0.00147236	0.004875526	CCT6P3
ENSG00000179456	310.000957	154.1095232	1.007518692	0.001472604	0.004875714	ZBTB18
ENSG00000165338	501.2972572	191.6683451	1.385746895	0.00147323	0.004877166	HECTD2
ENSG00000203721	1.539272221	29.69120447	-4.276306248	0.001475001	0.004882406	LINC00862
ENSG00000231646	9.094511955	0	5.518578921	0.001475467	0.004883327	FSIP2-AS1
ENSG00000079482	322.8580398	146.1298388	1.144085319	0.001488577	0.004926087	OPHN1
ENSG00000179528	52.46646241	13.31227683	1.970041897	0.001489217	0.004927578	LBX2
ENSG00000189292	39.01646	2.587817834	3.927192453	0.001489993	0.004929517	ALKAL2

ENSG00000196967	160.0790276	84.15511722	0.929810776	0.001492268	0.004936416	ZNF585A
ENSG00000139835	150.5426316	72.14644569	1.060398591	0.001493725	0.004940609	GRTP1
ENSG00000070729	0.293240848	16.04746641	-5.620759283	0.001499294	0.004958394	CNGB1
ENSG00000006831	28.52337026	88.61374084	-1.634283461	0.001503358	0.004971204	ADIPOR2
ENSG00000172638	2426.27929	5107.831851	-1.074017811	0.001505615	0.004978031	EFEMP2
novel.584	268.5815163	43.1330957	2.641628574	0.001508491	0.004986907	-
ENSG00000167232	412.3972728	215.1865165	0.939192176	0.001511214	0.004995275	ZNF91
ENSG00000241489	49.27040246	8.803544832	2.493138169	0.00151206	0.004997286	AC244197.3
ENSG00000263786	2.082916045	18.17151168	-3.114025236	0.001512208	0.004997286	AC022211.1
ENSG00000115590	0.616711807	15.43259769	-4.665200476	0.00151262	0.004998012	IL1R2
ENSG00000180667	396.6698684	195.2115171	1.022827075	0.001514513	0.005003631	YOD1
ENSG00000188687	53.2967941	8.633663848	2.613784798	0.001514773	0.005003855	SLC4A5
ENSG00000260912	72.95065389	19.21275888	1.921973091	0.001522315	0.005028129	AL158206.1
ENSG00000167393	45.460494	6.490833752	2.810807523	0.001524094	0.005033366	PPP2R3B
ENSG00000085872	399.4010502	688.367246	-0.785457565	0.001526373	0.005040251	CHERP
ENSG00000230712	3.355416585	27.14061796	-3.023064622	0.001528498	0.005046627	GGTLC4P
ENSG00000227827	4.119233273	51.63110899	-3.638241864	0.001529158	0.005048164	AC138969.2
ENSG00000108786	99.53241546	45.36963999	1.135163325	0.001531938	0.005056701	HSD17B1
ENSG00000163110	1067.61095	2412.47506	-1.176282537	0.001533758	0.005062065	PDLIM5
ENSG00000108515	154.2018164	75.23488223	1.033656632	0.001534766	0.005064746	ENO3
ENSG00000286288	23.24385263	2.578361223	3.188797719	0.001535273	0.005065778	AL109809.5
ENSG00000233610	11.56490667	0	5.866964317	0.001538528	0.005075872	LINC00462
ENSG00000167461	344.9613372	611.4735509	-0.826273155	0.001542369	0.005087409	RAB8A
ENSG00000162819	1131.699606	700.4996714	0.691857407	0.001542417	0.005087409	BROX
ENSG00000143748	256.3684561	419.6636132	-0.711324268	0.001549721	0.005110854	NVL
ENSG00000141574	166.7151668	818.1651113	-2.295204814	0.001556232	0.005131674	SECTM1
ENSG00000100418	1042.797273	1649.016123	-0.661023031	0.001556978	0.005133484	DESI1
ENSG00000178878	425.1630346	188.5425488	1.172305598	0.001557417	0.00513428	APOLD1
ENSG00000188338	79.70572042	4.880459818	4.040473704	0.001561399	0.005146753	SLC38A3
ENSG00000259250	83.24897261	21.11000793	1.97499612	0.001561905	0.005147769	AC018904.1
ENSG00000115268	7340.855268	11240.26638	-0.614671313	0.001564045	0.005154167	RPS15
ENSG00000172081	924.669477	2690.893019	-1.540983713	0.001564753	0.005155846	MOB3A
ENSG00000180773	249.9824901	471.9792891	-0.916214829	0.001566859	0.005162131	SLC36A4
ENSG00000286760	35.65309459	9.955878399	1.835357427	0.001572824	0.005181127	AL022069.3
ENSG00000152240	296.2977321	497.1201892	-0.747061204	0.001573643	0.005183126	HAUS1
ENSG00000182903	517.1066629	289.1893495	0.838193527	0.00157383	0.005183126	ZNF721
ENSG00000267787	60.53171091	21.58323711	1.495617099	0.001580564	0.005204641	AC027097.2
ENSG00000156170	371.4736496	181.4557596	1.032092494	0.001582254	0.005209549	NDUFAF6
ENSG00000286071	16.23942261	1.00940975	3.949188484	0.001584268	0.005215519	AL136171.1
ENSG00000167105	38.66491057	13.13635968	1.551582737	0.001584599	0.005215945	TMEM92
ENSG00000227345	312.6116898	533.5533651	-0.771376025	0.001586173	0.005220467	PARG
ENSG00000108691	3280.284206	1636.355586	1.003273777	0.001587542	0.005224309	CCL2
ENSG00000186020	368.5166819	184.0372373	1.001724298	0.001590906	0.005234719	ZNF529
ENSG00000258634	172.5203232	86.49637371	0.995362519	0.001593364	0.00524214	AL160006.1
ENSG00000164484	64.86075934	402.8884403	-2.635728478	0.001597805	0.005256087	TMEM200A
ENSG00000166135	884.5235047	1344.465813	-0.604041801	0.00159851	0.005257739	HIF1AN
ENSG00000198108	53.66918519	246.9859479	-2.203527592	0.001600864	0.005264815	CHSY3
ENSG00000242732	351.7843156	188.8566379	0.895975541	0.001602586	0.005269814	RTL5
ENSG00000285816	4.822874471	21.76629912	-2.16846504	0.00160317	0.005271065	AP000944.2
ENSG00000136267	17.45926955	1.10029862	4.005300257	0.001604317	0.005274168	DGKB
ENSG00000279673	11.04300215	0	5.797766681	0.001611636	0.005297561	AC092919.2

ENSG00000119703	53.42036404	16.60012006	1.688786682	0.00161383	0.005304101	ZC2HC1C
ENSG00000133424	548.946061	189.787685	1.533447664	0.001614691	0.005306258	LARGE1
ENSG00000159479	558.5683584	910.2670392	-0.704650663	0.001616432	0.005310869	MED8
ENSG00000162144	627.2883451	1139.280566	-0.860982176	0.001616503	0.005310869	CYB561A3
ENSG00000204869	25.7667584	3.005720009	3.128068169	0.001618843	0.005317886	IGFL4
ENSG00000174547	538.8081506	901.90254	-0.743493514	0.001621103	0.005324638	MRPL11
ENSG00000226862	8.67784336	0	5.452521459	0.001621488	0.005325227	AC104463.2
ENSG00000221817	149.5249357	59.98085144	1.315482246	0.001622939	0.00532932	PPP3CB-AS1
ENSG00000133027	228.2431632	368.8736555	-0.693027556	0.001626216	0.005339406	PEMT
ENSG00000109534	363.8480908	694.621921	-0.933383308	0.001628636	0.005346676	GAR1
ENSG00000249604	15.37990099	1.029478846	3.858999056	0.001629301	0.005348184	AC096564.2
ENSG00000284722	9.735256902	0	5.616481865	0.001633251	0.00536047	AP003175.1
novel.174	27.67491802	4.952563861	2.474438708	0.001634041	0.005362387	-
ENSG00000115232	448.291295	1154.280002	-1.364778534	0.00163707	0.005371648	ITGA4
ENSG00000141429	1038.589027	2514.501157	-1.275791036	0.001637991	0.005373993	GALNT1
ENSG00000137843	21.41510051	1.834601616	3.560541016	0.001641316	0.005384221	PAK6
ENSG00000186522	2726.465284	1675.893098	0.702132184	0.001647667	0.005403845	SEPT10
ENSG00000206341	43.06101563	1.110911105	5.294628675	0.001647714	0.005403845	HLA-H
ENSG00000168807	763.3387915	1199.697731	-0.652549446	0.001648266	0.005404974	SNTB2
ENSG00000147654	428.3198762	221.0476439	0.953048409	0.001649735	0.005409109	EBAG9
ENSG00000102755	347.1285053	125.0939555	1.470583185	0.001650546	0.005411084	FLT1
ENSG00000159263	35.88211427	11.88252476	1.597374622	0.001656741	0.005430708	SIM2
ENSG00000117477	88.21415048	35.06796346	1.32977296	0.001657542	0.005432648	CCDC181
ENSG00000184068	90.20727418	34.65121716	1.379761199	0.001662876	0.005449443	SREBF2-AS1
ENSG00000069431	83.32511665	9.793013881	3.082819262	0.001667449	0.005463741	ABCC9
ENSG00000232229	46.51535681	8.86734814	2.386036844	0.001677974	0.005497534	LINC00865
ENSG00000284946	11.84177881	82.25915244	-2.792010142	0.001684249	0.005517398	AC068831.7
ENSG00000145864	7.843465986	0	5.305759421	0.001685929	0.005522204	GABRB2
ENSG00000109743	279.1084814	81.00014608	1.782419574	0.001688057	0.005528478	BST1
ENSG00000171103	128.7922356	230.2910393	-0.838412846	0.001690649	0.005536268	TRMT61B
ENSG00000166831	70.10008894	16.00159602	2.128120568	0.001691141	0.005537181	RBPMS2
ENSG00000186675	7.912773023	0	5.317168069	0.001691545	0.005537807	MAGEE2
ENSG00000196235	2293.412893	3598.850598	-0.64994163	0.001692615	0.005540611	SUPT5H
ENSG00000087266	2915.928642	1106.015131	1.39868206	0.001700359	0.005565258	SH3BP2
ENSG00000105472	806.4045989	4298.836994	-2.414415284	0.001705186	0.005580353	CLEC11A
ENSG00000197496	408.1367868	1160.617682	-1.507851592	0.001712946	0.005605044	SLC2A10
ENSG00000047849	13821.65141	9429.261417	0.551731835	0.001714021	0.005607855	MAP4
ENSG00000055955	14.71275815	0.713078025	4.360245701	0.001716313	0.005614646	ITIH4
ENSG00000141293	34.99411438	3.689272328	3.258103488	0.001716829	0.005615627	SKAP1
ENSG00000273729	38.83117618	13.07486812	1.576808624	0.001717777	0.005618023	AC007686.3
ENSG00000110107	1353.70402	2305.238224	-0.768146583	0.001718619	0.00562007	PRPF19
ENSG00000142856	215.4579114	398.1737051	-0.886126226	0.001720119	0.005624264	ITGB3BP
ENSG00000204971	12.88672678	0	6.020758699	0.001722542	0.00563148	AP000812.1
ENSG00000123415	348.7409168	542.3463446	-0.636860765	0.00172397	0.00563544	SMUG1
ENSG00000280099	82.9594927	14.40440311	2.51610323	0.001726895	0.00564429	AL603750.1
novel.130	96.83188962	41.94357766	1.209046106	0.001727659	0.005646075	-
ENSG00000078140	1331.396733	1928.465602	-0.534486139	0.001728065	0.005646692	UBE2K
ENSG00000103932	424.0286235	755.3417205	-0.832650054	0.001732985	0.005662059	RPAP1
ENSG00000149256	206.3800797	66.17167624	1.639711899	0.001735219	0.005668643	TENM4
ENSG00000213096	280.0529392	141.4493464	0.983879325	0.001736996	0.005673736	ZNF254
ENSG00000015520	8.202252925	0	5.369306661	0.001737337	0.005674136	NPC1L1

ENSG00000270441	55.31820974	16.11011384	1.787964982	0.001742687	0.005690893	AC135506.1
ENSG00000251365	8.117830831	0	5.355786118	0.001743373	0.00569242	LINC02236
ENSG00000175164	8.233736686	0	5.374328036	0.001745559	0.005698251	ABO
ENSG00000249574	8.238751281	0	5.375123451	0.001745598	0.005698251	AC226118.1
ENSG00000225793	37.4352479	10.53908524	1.818030263	0.001747776	0.005704362	AL080250.1
ENSG00000104529	52.77370686	122.1308954	-1.210810148	0.001747909	0.005704362	EEF1D
ENSG00000134444	895.5363918	546.3576212	0.712773509	0.001749075	0.005707451	RELCH
novel.586	34.76996711	8.978306106	1.952542147	0.001750735	0.00571215	-
ENSG00000225963	36.99114122	10.18254623	1.849524002	0.001751456	0.005713784	AC009950.1
ENSG00000204778	85.80698054	28.8804779	1.573440982	0.001753719	0.005720447	CBWD4P
ENSG00000111142	1054.13471	1888.488333	-0.841373852	0.001756113	0.005727538	METAP2
ENSG00000100302	16.35901814	71.40957415	-2.125824429	0.001759651	0.005738357	RASD2
ENSG00000147649	1825.36291	3000.667939	-0.717210445	0.001760163	0.005739308	MTDH
ENSG00000276916	3.912921816	18.87397722	-2.26819173	0.001761468	0.005742841	AL442125.2
ENSG00000089248	1608.542747	2707.560827	-0.751249946	0.001763372	0.005748328	ERP29
ENSG00000083067	134.7798247	27.8888255	2.273859729	0.001764535	0.005751397	TRPM3
ENSG00000060642	193.7333004	383.9990394	-0.986569746	0.001766989	0.005758675	PIGV
ENSG00000112992	589.9971688	1116.528966	-0.920543546	0.001771123	0.005771421	NNT
ENSG00000196071	8.558461294	0	5.429920424	0.001772196	0.005774193	OR2L13
ENSG00000169962	27.74666108	5.685710982	2.281120139	0.00177943	0.005797038	TAS1R3
ENSG00000114923	862.8996734	344.2728704	1.32640099	0.001780612	0.00580016	SLC4A3
ENSG00000137710	3506.091937	2123.745498	0.723215569	0.001781605	0.005802445	RDX
ENSG00000172037	6914.287817	11336.93854	-0.713355436	0.00178176	0.005802445	LAMB2
ENSG00000184465	263.1292931	536.9162536	-1.028261981	0.001785148	0.005812751	WDR27
ENSG00000233381	1.232169966	13.57433095	-3.47767836	0.00178566	0.005813689	AK4P3
ENSG00000170209	14.98329077	0.672939833	4.412119338	0.001785967	0.005813958	ANKK1
ENSG00000180616	83.875456	10.7941229	2.951236755	0.001786901	0.005816272	SSTR2
ENSG00000163958	9.764233365	0	5.620502043	0.001789261	0.005823222	ZDHHC19
ENSG00000198648	371.8872967	776.7446522	-1.06303506	0.001790758	0.005827366	STK39
ENSG00000254332	10.29394451	39.99776129	-1.954668094	0.001792091	0.005830971	AF201337.1
ENSG00000269950	8.432454979	0	5.410354616	0.001796319	0.005843997	AP000962.1
ENSG00000222881	0	7.868550876	-5.5576467	0.001798261	0.005849584	RF00019
ENSG00000171530	1380.829225	2268.855214	-0.716465644	0.00180222	0.005861728	TBCA
ENSG00000142748	23.3105811	1.722487775	3.729346207	0.001802499	0.005861901	FCN3
ENSG00000066230	510.5932706	109.7094931	2.219347982	0.001803347	0.005863831	SLC9A3
ENSG00000272950	14.70140404	0.774441189	4.324770397	0.001803694	0.005863831	AC093799.1
ENSG00000287917	18.47599651	53.35847705	-1.528120431	0.00180377	0.005863831	AP000944.4
ENSG00000135046	15518.00598	6573.762089	1.239113989	0.001806259	0.00587119	ANXA1
ENSG00000000457	265.4536231	144.9552999	0.87265201	0.001806843	0.005872352	SCYL3
ENSG00000111231	410.0774161	656.9827758	-0.679854344	0.001808267	0.005876246	GPN3
ENSG00000091140	964.7062261	1697.390049	-0.815266858	0.001813911	0.005893851	DLD
ENSG00000107679	1236.808276	586.0584088	1.077644856	0.001816924	0.005902903	PLEKHA1
ENSG00000198356	1156.801913	1891.896066	-0.709744466	0.001817961	0.005905531	ASNA1
ENSG00000160688	423.1981771	714.8588932	-0.756708435	0.001821888	0.00591755	FLAD1
ENSG00000232472	462.5471246	916.7284757	-0.987130959	0.001822865	0.005919984	EEF1B2P3
ENSG00000214578	23.04993512	0.713078025	5.00716287	0.001827433	0.005934074	HMGN2P15
ENSG00000175147	21.0990089	1.447381022	3.871497214	0.001831872	0.005947746	TMEM51-AS1
ENSG00000225791	248.4870243	139.7738037	0.82886715	0.001832608	0.005949393	TRAM2-AS1
ENSG00000225630	3016.393104	1855.543384	0.701032537	0.001833076	0.00595017	MTND2P28
ENSG00000009724	97.83250513	27.97629392	1.803894634	0.001833842	0.005951911	MASP2
ENSG00000287901	10.74843638	0	5.758657993	0.001834929	0.005954694	AL137026.3

ENSG00000261616	35.11041968	8.703199352	2.019015851	0.001837425	0.00596205	AC036108.3
ENSG00000086200	274.2433114	570.6086614	-1.057497852	0.001838643	0.005964748	IPO11
ENSG00000272123	10.80234363	0.336469917	4.806632319	0.001838715	0.005964748	AC008966.2
ENSG00000241666	10.63127688	0.336469917	4.781647765	0.001842639	0.00597673	AL031733.2
ENSG00000277778	88.71098237	24.63294812	1.844231729	0.001845546	0.005985412	PGM5P2
ENSG00000141447	1554.726187	673.9480046	1.205553781	0.001849303	0.005996127	OSBPL1A
ENSG00000277159	26.00129087	3.149799701	3.029726642	0.001849312	0.005996127	AL139384.2
ENSG00000163866	524.1098237	863.7392048	-0.721099174	0.001851403	0.006001859	SMIM12
ENSG00000143429	54.03395599	163.5527577	-1.598063648	0.001851542	0.006001859	LSP1P4
ENSG00000105641	26.77431037	0.336469917	6.1142776	0.001853332	0.006006914	SLC5A5
ENSG00000124587	453.8366596	1154.240573	-1.346479793	0.001855647	0.006013666	PEX6
ENSG00000236266	10.53557195	0.336469917	4.770106263	0.001859429	0.00602517	Z98884.1
ENSG00000175104	390.7512151	235.1205729	0.733522198	0.001864525	0.006040859	TRAF6
ENSG00000284428	42.55616435	10.70079388	1.990181606	0.001864735	0.006040859	AC092329.4
ENSG00000198324	180.2990973	349.9781025	-0.956366687	0.001865773	0.006043466	PHETA1
ENSG00000144550	7.54897149	0	5.250424069	0.001866528	0.006044534	CPNE9
ENSG00000131885	10.2850977	0	5.696287976	0.001866568	0.006044534	KRT17P1
ENSG00000103472	14.23716771	0.672939833	4.335943119	0.001868134	0.006048854	RRN3P2
ENSG00000108799	1431.530449	802.8239288	0.834814158	0.001868661	0.006049804	EZH1
ENSG00000090659	9.190288158	0	5.532608345	0.001870506	0.006055024	CD209
ENSG00000254427	20.45568573	2.038888596	3.287947525	0.001871475	0.006057406	AC103736.1
ENSG00000240342	1808.701746	7893.403361	-2.125715945	0.001872321	0.00605939	RPS2P5
ENSG00000104889	0.601596751	11.61931476	-4.268735586	0.001873619	0.006062835	RNASEH2A
ENSG00000119787	449.9613469	692.1315633	-0.621379529	0.001877689	0.006075246	ATL2
ENSG00000177791	28.6354439	4.159209451	2.764026677	0.00187968	0.006080931	MYOZ1
ENSG00000188827	202.4530558	332.6378186	-0.715946221	0.001882853	0.006090438	SLX4
ENSG00000226833	59.71334624	17.88592083	1.731298876	0.001884679	0.006094918	AC092164.1
ENSG00000170561	18.96197214	1.00940975	4.176861583	0.001884707	0.006094918	IRX2
ENSG00000272842	22.13357203	3.53586442	2.635323225	0.001892827	0.006120417	AL391834.1
ENSG00000144029	892.7171966	1465.072343	-0.714505162	0.001893942	0.006123259	MRPS5
novel.855	38.28130756	2.140389951	4.15896486	0.001895764	0.006128387	-
ENSG00000120694	1043.446363	1971.742207	-0.918284085	0.001896803	0.006130722	HSPH1
ENSG00000204791	20.91415201	0.743759607	4.842944524	0.001896958	0.006130722	SMPD5
ENSG00000198301	461.9434638	703.4351664	-0.606986755	0.00189774	0.006132486	SDAD1
ENSG00000268751	18.24203351	1.90542139	3.305051984	0.001898653	0.006134671	SCGB1B2P
ENSG00000073614	693.1817461	1096.374544	-0.661365204	0.001903346	0.006149071	KDM5A
ENSG00000197008	125.5775131	65.02995515	0.948407469	0.001903786	0.006149727	ZNF138
ENSG00000253944	12.84618275	0.387220594	5.056633795	0.001904233	0.006150408	AC027117.2
ENSG00000144815	1590.972367	835.3590628	0.929581157	0.001906099	0.006155669	NXPE3
novel.93	17.07283107	1.161661783	3.942179078	0.001907359	0.006158972	-
ENSG00000063177	8439.113589	13673.55719	-0.696231175	0.001913338	0.006177512	RPL18
ENSG00000133661	23.37458881	2.792104814	3.046768463	0.001915027	0.006182098	SFTPD
ENSG00000121297	210.3800894	495.9432318	-1.236560481	0.001915235	0.006182098	TSHZ3
ENSG00000156140	13.55386963	116.1954122	-3.102227818	0.001917844	0.006189753	ADAMTS3
ENSG00000183888	8.860548954	0	5.480115935	0.001920573	0.006197791	SRARP
ENSG00000131470	85.81973084	222.0499428	-1.370936593	0.0019327	0.006236151	PSMC3IP
ENSG00000129925	1129.682206	1819.42967	-0.687720839	0.001935316	0.006243692	TMEM8A
ENSG00000141504	1271.09645	752.2337291	0.757273293	0.001935518	0.006243692	SAT2
ENSG00000254676	17.27788888	3.220619475	2.420979357	0.001937223	0.006248417	AP000873.4
ENSG00000230838	0.293240848	9.293551051	-4.836553113	0.001944569	0.00627133	LINC01614
ENSG00000123388	0	17.18473956	-6.685906138	0.001948741	0.006284008	HOXC11

ENSG00000261857	16.32481362	0.336469917	5.402235357	0.001949703	0.006286327	MIA
ENSG00000287439	9.139857123	0	5.525245682	0.001951265	0.006290585	AC092078.3
ENSG00000110066	1022.16626	661.5832827	0.628012002	0.001952934	0.006294544	KMT5B
ENSG00000137494	171.768501	331.2478195	-0.947522977	0.001952978	0.006294544	ANKRD42
ENSG00000170425	109.5467229	452.773757	-2.047351235	0.001953661	0.006295964	ADORA2B
ENSG00000083857	3103.502172	5327.160037	-0.779508498	0.001954512	0.006297925	FAT1
ENSG00000174842	126.0524194	243.7219798	-0.952454133	0.001955601	0.006299872	GLMN
ENSG00000137266	235.9434984	111.3922279	1.083772529	0.001955601	0.006299872	SLC22A23
ENSG00000274225	29.80031593	4.402350354	2.772005209	0.001956394	0.006301647	AP001065.1
ENSG00000166796	0.308355904	14.75978625	-5.506623807	0.001964467	0.006326866	LDHC
ENSG00000064687	486.240711	193.427037	1.328685654	0.001965413	0.006329125	ABCA7
ENSG00000159720	900.3563985	1697.522639	-0.914912005	0.001966167	0.006330772	ATP6V0D1
ENSG00000160932	155.8342428	415.4350781	-1.415054884	0.001968285	0.006336805	LY6E
ENSG00000285881	3.033199274	28.13286548	-3.210463943	0.001968657	0.006337217	AC105206.3
ENSG00000175224	1019.422481	1820.730797	-0.836753733	0.001970749	0.006343164	ATG13
ENSG00000179119	371.6127897	676.6525817	-0.864814367	0.001971622	0.006345191	SPTY2D1
ENSG00000165410	1661.810255	2626.158412	-0.660210732	0.001972735	0.006347985	CFL2
ENSG00000081386	272.3541421	160.0239098	0.768131111	0.001975895	0.006357364	ZNF510
ENSG00000116191	345.6608622	685.1436472	-0.986671278	0.001977551	0.006361472	RALGPS2
ENSG00000183621	263.9820479	116.3031125	1.184672192	0.001977661	0.006361472	ZNF438
ENSG00000267058	3.665026137	20.87961569	-2.511580855	0.001982321	0.006375674	AC006213.2
ENSG00000257681	30.96765182	6.65498254	2.20448829	0.001982732	0.006376206	AC025265.1
ENSG00000156050	204.739377	84.80854872	1.268593084	0.001985348	0.006383829	FAM161B
ENSG00000272476	5.515161559	31.99270795	-2.543775832	0.001987628	0.006390367	AL024507.2
ENSG00000260565	409.0253175	179.7912486	1.185049399	0.001988655	0.006392877	ERVK13-1
ENSG00000179111	4.597687012	28.86858114	-2.654053955	0.00199119	0.006400237	HES7
novel.315	9.931396626	0	5.646267288	0.00199424	0.006409246	-
ENSG00000186364	104.4348396	51.5795064	1.017154639	0.001996088	0.006414393	NUDT17
ENSG00000183087	2241.371465	4862.266953	-1.117266325	0.001999711	0.006425239	GAS6
ENSG00000146232	523.5993445	262.0120141	0.999838884	0.002002362	0.006432961	NFKBIE
ENSG00000286451	9.260564109	0	5.546209091	0.002002775	0.006433494	AL499602.1
ENSG00000163516	821.8183463	2046.361316	-1.315980903	0.002006309	0.006444051	ANKZF1
ENSG00000177888	538.0887543	315.7327621	0.769796011	0.002006958	0.006445336	ZBTB41
ENSG00000232034	0	8.834354808	-5.726248452	0.002007773	0.006447158	AC092168.2
ENSG00000110651	15596.8314	9671.78348	0.689424816	0.002008362	0.006448253	CD81
ENSG00000229425	14.74201934	0.356539013	5.252688238	0.002011729	0.006458265	AJ009632.2
ENSG00000169239	198.9227685	89.1123858	1.159805186	0.002012438	0.006459741	CA5B
ENSG00000140682	979.7461784	2145.004771	-1.130377008	0.002016165	0.006470906	TGFB1I1
ENSG00000272325	308.4365409	158.515598	0.959082437	0.002019138	0.006479646	NUDT3
ENSG00000198429	115.7562523	47.81607507	1.271754719	0.002020574	0.006483455	ZNF69
ENSG00000261061	53.12977303	115.6758803	-1.12345497	0.002021146	0.00648449	AC092718.4
ENSG00000263004	39.30962992	7.765765251	2.352088649	0.002021486	0.006484779	AC007114.1
ENSG00000117122	124.2591529	755.5526862	-2.604467368	0.002022641	0.006487683	MFAP2
ENSG00000178252	1872.402215	3089.803126	-0.722604642	0.00202641	0.006498969	WDR6
ENSG00000286367	0.615458159	10.90379659	-4.161476904	0.002027992	0.00650324	AC000072.1
ENSG00000146085	630.7107912	976.8955996	-0.631475084	0.00203127	0.00651295	MMUT
ENSG00000284691	183.8500844	82.19637661	1.163971145	0.002033493	0.006519271	AC073111.4
ENSG00000165996	151.6690674	281.6305685	-0.892095204	0.002035173	0.006523854	HACD1
ENSG00000132466	1339.97249	2088.340876	-0.640156891	0.002037575	0.006530746	ANKRD17
ENSG00000229771	1.845120827	15.85636045	-3.114197121	0.002040358	0.006538861	AL035665.1
ENSG00000117475	462.2805392	715.0986821	-0.629609267	0.002040898	0.006539783	BLZF1

ENSG00000286315	18.95549044	1.834601616	3.386984075	0.002041316	0.006540318	AC087175.1
ENSG00000142687	1002.849404	1505.588514	-0.586028735	0.002042363	0.006541741	KIAA0319L
ENSG00000129696	94.73016884	191.7243144	-1.018770741	0.002042418	0.006541741	TTI2
ENSG00000272375	14.8500473	2.10970837	2.796678643	0.002042516	0.006541741	AC026979.3
ENSG00000100170	10.10901635	0	5.670294298	0.002043074	0.006542721	SLC5A1
ENSG00000163882	672.2904452	1118.428421	-0.734142629	0.002044051	0.006544455	POLR2H
ENSG00000167693	226.3400022	738.1170655	-1.705647172	0.002044119	0.006544455	NXN
ENSG00000276710	6.636511537	25.02949693	-1.913957724	0.002044476	0.006544791	CSPG4P10
ENSG00000135503	402.4152588	670.210572	-0.73605018	0.002044801	0.006545027	ACVR1B
ENSG00000183844	10.08519703	0	5.667099157	0.002047802	0.006553824	FAM3B
ENSG00000159921	565.2283051	900.588729	-0.67167485	0.002050535	0.006561762	GNE
ENSG00000253731	33.89873537	70.36973961	-1.054951685	0.002051013	0.006562484	PCDHGA6
ENSG00000083444	3794.598415	11150.90116	-1.555145467	0.002055282	0.006575208	PLOD1
ENSG00000133026	1238.267246	2931.262107	-1.243268183	0.002055496	0.006575208	MYH10
ENSG00000165282	551.526789	897.6058818	-0.7029416	0.002058127	0.006582147	PIGO
ENSG00000128917	198.2122896	23.06818778	3.107713971	0.002058172	0.006582147	DLL4
ENSG00000188761	32.21171753	4.566499142	2.809436905	0.002060895	0.006590045	BCL2L15
ENSG00000067066	1710.365687	2833.466849	-0.728197829	0.002061377	0.006590773	SP100
ENSG00000005238	606.4511089	1305.084376	-1.105439718	0.002061864	0.006590959	FAM214B
ENSG00000267554	8.144299997	0	5.360006513	0.002061942	0.006590959	AC015911.8
ENSG00000146469	8.13043859	0	5.357769907	0.002062421	0.006591678	VIP
ENSG00000237324	10.03978059	0.356539013	4.698732227	0.002063367	0.006593892	AC093158.1
ENSG00000184860	5.821010166	31.36221806	-2.437171214	0.002063923	0.006594357	SDR42E1
ENSG00000230479	1.803536605	20.82642487	-3.521445894	0.00206402	0.006594357	AP000695.1
ENSG00000185347	176.8974379	394.5818373	-1.158276735	0.002066407	0.006601169	TEDC1
ENSG00000198718	413.9144066	228.5101765	0.858217206	0.002074202	0.006625256	TOGARAM1
ENSG00000108669	1128.275992	647.6506148	0.801116152	0.002075142	0.006627085	CYTH1
ENSG00000258366	16.83600477	50.55473227	-1.582630151	0.002075285	0.006627085	RTEL1
ENSG00000121892	1540.734738	2196.55705	-0.511585206	0.002078862	0.006637694	PDS5A
ENSG00000011114	21.7911538	55.13898876	-1.338348251	0.002080546	0.006642255	BTBD7
ENSG00000158941	1315.309331	1917.451164	-0.543847305	0.002080831	0.006642348	CCAR2
ENSG00000129968	3169.578248	2171.418629	0.545644374	0.002081492	0.006643642	ABHD17A
novel.810	13.02387375	42.10990981	-1.690925881	0.002083405	0.006648931	-
ENSG00000084652	1526.38635	2309.498023	-0.597569796	0.002087235	0.006660334	TXLNA
ENSG00000153233	5.497539206	23.4486487	-2.096683892	0.002089833	0.006667808	PTPRR
ENSG00000176678	99.24066921	627.117747	-2.660244257	0.002092485	0.006675448	FOXL1
ENSG00000269694	10.78722857	0.387220594	4.804751754	0.002092918	0.006676009	AC005197.1
ENSG00000165821	293.0981528	152.5448913	0.942573969	0.002093887	0.006678029	SALL2
ENSG00000131019	29.65879511	104.1890868	-1.81315944	0.002094065	0.006678029	ULBP3
ENSG00000001497	863.4941317	1428.471854	-0.72618589	0.002094911	0.006679907	LAS1L
ENSG00000122694	943.7711732	407.1375334	1.21345794	0.002095964	0.006682445	GLIPR2
ENSG00000264176	18.94531871	2.833398881	2.732617768	0.002098927	0.006691072	MAGOH2P
ENSG00000169871	1484.75081	2629.502999	-0.824484409	0.002103043	0.00670337	TRIM56
ENSG00000257150	8.092615314	0	5.351644214	0.002104561	0.006706935	PGAM1P5
ENSG00000259846	8.090108017	0	5.35123988	0.002104678	0.006706935	AC092131.1
ENSG00000261504	10.04103424	0.387220594	4.698897639	0.002105592	0.006709026	LINC01686
ENSG00000143252	1396.921799	2177.488641	-0.640494905	0.002107166	0.006712803	SDHC
ENSG00000174938	276.6401674	1025.219842	-1.889748707	0.002107295	0.006712803	SEZ6L2
ENSG00000164619	32.98251447	172.882115	-2.389091738	0.002109726	0.006719726	BMPER
ENSG00000182809	2867.223354	1487.616379	0.946522299	0.00211571	0.006737957	CRIP2
ENSG00000269978	8.116577183	0	5.355523082	0.002116057	0.006738238	AL359881.1

novel.820	0.616711807	28.74534124	-5.553061849	0.002118925	0.006746544	-
ENSG00000180871	14.84628635	1.069617038	3.787679135	0.002121071	0.006752549	CXCR2
ENSG00000116984	2135.988738	1153.525862	0.888837729	0.002123158	0.006758363	MTR
ENSG00000111254	29.75554023	8.16257085	1.858413516	0.002124404	0.006761503	AKAP3
ENSG00000013583	385.5570715	939.4999337	-1.28506518	0.002127217	0.006769625	HEBP1
ENSG00000280639	9.687333165	0.336469917	4.648062394	0.002128905	0.006774167	LINC02204
ENSG00000266338	330.5494718	846.9269853	-1.357079244	0.002130536	0.006778527	NBPF15
ENSG00000171720	752.4142738	1370.892219	-0.865281759	0.002131689	0.006780864	HDAC3
ENSG00000139531	528.8194574	294.3581443	0.844521718	0.002131792	0.006780864	SUOX
ENSG00000197756	10563.91974	16663.65975	-0.657565943	0.002132309	0.006781676	RPL37A
ENSG00000127837	1544.441661	2302.414643	-0.576058514	0.002133347	0.006784147	AAMP
ENSG00000277999	13.65946156	1.386017859	3.26666475	0.002137748	0.00679731	AC009093.6
ENSG00000185453	585.6410824	320.320101	0.87131469	0.002139614	0.006802412	ZSWIM9
ENSG00000154016	7.925380782	0	5.319314929	0.002140307	0.006803783	GRAP
ENSG00000237212	7.880035613	0	5.311800829	0.002141512	0.006806781	AL449403.1
ENSG00000140694	35.10346726	120.4199532	-1.778702283	0.002144187	0.006814061	PARN
ENSG00000268442	15.94743541	1.783850939	3.156845128	0.002144327	0.006814061	AC073534.1
ENSG00000226581	8.382095215	0	5.40240565	0.002151711	0.006835191	AC092634.3
ENSG00000164674	57.35355805	209.0788753	-1.864932479	0.002151774	0.006835191	SYTL3
ENSG00000166289	347.7237028	100.7773613	1.787469953	0.002151973	0.006835191	PLEKHF1
novel.612	9.70370187	0.387220594	4.65017614	0.002152029	0.006835191	-
ENSG00000279443	3.020591515	22.20097834	-2.881148729	0.00215856	0.006854613	AL513497.1
ENSG00000260331	16.44071947	1.436768536	3.518487609	0.002158672	0.006854613	AC079148.1
ENSG00000286584	23.43343938	4.758889366	2.314215347	0.00216025	0.006858786	AC023494.1
ENSG00000167037	18.00667432	2.51699806	2.838715483	0.002160607	0.006859082	SGSM1
ENSG00000182240	1687.279376	765.6879488	1.139779278	0.002163476	0.006866652	BACE2
ENSG00000259781	655.0813213	1115.740364	-0.768529106	0.00216352	0.006866652	HMGB1P6
ENSG00000020633	103.6298656	16.76054443	2.634195518	0.00216979	0.006885712	RUNX3
novel.922	52.63949447	6.33729745	3.065345526	0.002181137	0.006920875	-
ENSG00000109684	9.813268209	0	5.627329148	0.002181476	0.006921106	CLNK
ENSG00000215067	118.5608313	45.22710136	1.387054808	0.002182483	0.006923454	ALOX12-AS1
ENSG00000087074	5027.107977	1927.255173	1.383270817	0.002187373	0.006938119	PPP1R15A
ENSG00000231507	9.252970946	0	5.545066832	0.002189505	0.006944034	LINC01353
ENSG00000271327	70.50920816	17.36407716	2.018673603	0.00219102	0.006947991	AC010201.2
ENSG00000270547	16.44594753	0.713078025	4.519697739	0.002192535	0.006951948	LINC01235
ENSG00000180626	211.5384366	99.25163017	1.09058684	0.002196945	0.006965081	ZNF594
ENSG00000250659	1.509042109	12.168244	-3.009408748	0.002198391	0.006968814	AP001363.1
ENSG00000103227	544.9536166	260.0286281	1.068497474	0.002199103	0.006970221	LMF1
ENSG00000198954	856.0362619	1305.12927	-0.608682054	0.002199923	0.006971968	KIF1BP
ENSG00000013523	711.1631499	405.8046248	0.810023638	0.002204454	0.006985477	ANGEL1
ENSG00000257027	68.33763836	22.80895899	1.575224651	0.002218574	0.007029363	AC010186.3
ENSG00000171533	443.4712835	126.4615612	1.81149235	0.002219438	0.007031244	MAP6
ENSG00000176919	19.51070183	2.435565801	2.978103966	0.002223221	0.007042369	C8G
ENSG00000277053	546.9845914	306.2489229	0.837437492	0.002224902	0.007046835	GTF2IP1
ENSG00000251532	9.653270835	0	5.605080273	0.002227606	0.007054538	AC091849.2
ENSG00000232310	11.63797465	0.387220594	4.914503163	0.002239186	0.007090346	AL078590.2
ENSG00000131558	802.660345	1208.947745	-0.590922776	0.002241112	0.00709558	EXOC4
ENSG00000169604	3393.71221	5803.118104	-0.774006835	0.002249964	0.007122739	ANTXR1
ENSG00000116455	301.264706	629.6628325	-1.063988253	0.002254653	0.007136713	WDR77
ENSG00000233718	7.284707106	0	5.198215878	0.002258798	0.007148962	MYCNOS
ENSG00000120798	701.673166	442.9760034	0.663774751	0.002260479	0.00715341	NR2C1

ENSG00000280159	9.71505598	0.387220594	4.651657318	0.002261205	0.007154146	AC016583.1
ENSG00000168778	129.2058984	234.8223618	-0.861908183	0.002261262	0.007154146	TCTN2
ENSG00000071794	638.3881612	1156.614368	-0.857236906	0.002271904	0.00718694	HLTF
ENSG00000257851	6.665488001	26.39313394	-1.987345866	0.00227828	0.007206231	HNRNPA3P10
ENSG00000175155	682.5317632	334.7059292	1.028757502	0.002278673	0.007206597	YPEL2
novel.356	15.699611	0.774441189	4.414102607	0.002279451	0.007208181	-
ENSG00000219545	225.4348667	128.8498096	0.805992615	0.002279916	0.007208775	UMAD1
ENSG00000067167	2948.597476	7083.28976	-1.264442786	0.002284309	0.007221786	TRAM1
ENSG00000138375	426.717352	663.0542948	-0.63572182	0.002284659	0.007222013	SMARCAL1
ENSG00000258056	184.6668669	87.13747608	1.082267472	0.002289393	0.007236097	AC009779.2
ENSG00000261786	879.2857041	300.9721583	1.547243707	0.002291627	0.007242276	AC006058.1
ENSG00000260668	3.914175465	21.20778487	-2.430643417	0.002294885	0.007251693	AC093536.1
ENSG00000151006	28.62262268	89.25988593	-1.638580653	0.002295737	0.007253502	PRSS53
ENSG00000171827	171.6888087	87.98384574	0.965332477	0.002298733	0.007262084	ZNF570
ENSG00000253846	64.05353531	201.7292612	-1.654963215	0.00230329	0.007275597	PCDHGA10
novel.204	0	8.633663848	-5.695590716	0.002304015	0.007277	-
ENSG00000162777	127.6075892	34.4363177	1.889253524	0.002306201	0.007283022	DENND2D
ENSG00000171658	11.57898154	62.03343497	-2.422727082	0.002307719	0.007286927	NMRAL2P
ENSG00000117543	294.5592712	567.7953788	-0.94744252	0.002314555	0.007307627	DPH5
ENSG00000183718	329.224204	162.4241705	1.019930247	0.002316599	0.007313191	TRIM52
ENSG00000111674	1079.207276	4369.815189	-2.017545829	0.002318145	0.007317181	ENO2
ENSG00000088899	521.390395	232.2933729	1.167532228	0.002322576	0.007329955	LZTS3
ENSG00000204209	35.10554728	3.047014077	3.553319539	0.002322756	0.007329955	DAXX
ENSG00000169314	0.925067711	16.51282721	-4.174413625	0.002324938	0.007335949	C22orf15
ENSG00000163482	395.3670665	721.0988205	-0.866673343	0.002325937	0.007338211	STK36
ENSG00000075568	779.9205054	1351.435454	-0.792818348	0.002329413	0.007348284	TMEM131
ENSG00000047410	1810.776034	2804.335301	-0.63096089	0.002335117	0.007365386	TPR
ENSG00000261051	7.66863829	43.0932143	-2.492451137	0.002337115	0.007370791	AC107021.2
ENSG00000105696	1094.870469	259.521275	2.076088859	0.002343693	0.007390639	AC003112.1
ENSG00000165899	59.05978007	8.212037258	2.857348056	0.002345287	0.007394768	OTOGL
ENSG00000204860	13.64907636	0.387220594	5.144432782	0.00234651	0.007397728	FAM201A
ENSG00000162745	1095.100538	350.4564216	1.643943354	0.002350614	0.007409769	OLFML2B
ENSG00000270959	76.05753405	34.6595179	1.131817825	0.002353118	0.007416761	LPP-AS2
ENSG00000234699	13.08558762	0.713078025	4.189216705	0.002353775	0.00741793	AL139339.1
ENSG00000237945	58.9618804	8.050328615	2.877500621	0.002361228	0.007440516	LINC00649
ENSG00000124818	9.845792156	0	5.634514958	0.0023625	0.007443623	OPN5
ENSG00000141527	45.1550014	10.87311501	2.05912811	0.002368279	0.007460926	CARD14
ENSG00000156011	404.5805166	1233.249378	-1.607856987	0.002370335	0.007466498	PSD3
ENSG00000171311	378.6317374	616.6943807	-0.704208334	0.002372299	0.007470951	EXOSC1
ENSG00000287467	16.26965272	1.702418679	3.213797146	0.002372324	0.007470951	AC037459.4
ENSG00000165138	369.9448338	558.4843616	-0.59452092	0.002378114	0.007488278	ANKS6
ENSG00000137513	204.5894088	349.0655834	-0.770940212	0.002379508	0.007491759	NARS2
ENSG00000183801	381.5703819	10.9275901	5.122395701	0.002386498	0.007512855	OLFML1
ENSG00000143418	1303.280971	1854.636083	-0.508977495	0.002397903	0.007547846	CERS2
ENSG00000242880	8.371994754	0	5.400757771	0.002398197	0.007547856	AC026341.1
ENSG00000203808	57.84078733	20.16769354	1.528423848	0.002411196	0.007587849	BVES-AS1
ENSG00000175946	0	7.795290958	-5.53917832	0.00242103	0.007617874	KLHL38
ENSG00000229700	7.823336335	0	5.302332975	0.002425053	0.007628683	AL008627.1
ENSG00000254607	7.823336335	0	5.302332975	0.002425053	0.007628683	AP001783.1
ENSG00000125967	324.8653799	543.0702451	-0.740665763	0.002425409	0.00762888	NECAB3
ENSG00000104679	800.8998601	485.2338588	0.722604961	0.002426562	0.007631582	R3HCC1

ENSG00000148362	1040.811414	499.6234239	1.058194809	0.002428312	0.007636162	PAXX
ENSG00000258667	8.26744301	62.44324602	-2.911280069	0.002429656	0.007639465	HIF1A-AS2
ENSG00000136891	472.0493759	820.5433204	-0.797448692	0.002430988	0.007642726	TEX10
ENSG00000168175	1203.779256	1892.73487	-0.653052932	0.002431333	0.007642888	MAPK1IP1L
ENSG00000171084	18.7163703	47.24797582	-1.335269953	0.002433221	0.007647324	FAM86JP
ENSG00000101457	441.2903843	722.8003876	-0.711643388	0.002433333	0.007647324	DNTTIP1
ENSG00000157869	468.6845274	302.6050543	0.631088218	0.002434096	0.007648255	RAB28
ENSG00000186952	29.80310796	8.591085512	1.794185949	0.002434218	0.007648255	TMEM232
ENSG00000135362	148.4019323	439.3253066	-1.566483282	0.002435078	0.007650033	PRR5L
novel.873	85.05136992	321.0261969	-1.915176756	0.002436334	0.007653052	-
ENSG00000143184	19.07168103	0	6.586644123	0.00243821	0.007658019	XCL1
ENSG00000213542	10.49273408	0.336469917	4.764740483	0.002439309	0.007660546	AC007000.1
ENSG00000279026	19.6692321	2.822786395	2.788481855	0.002442332	0.007669111	AC005225.4
ENSG00000157680	50.08048979	21.14655011	1.242516443	0.002444209	0.007674079	DGKI
ENSG00000180638	16.41704234	2.904218654	2.507415856	0.002446248	0.00767955	SLC47A2
ENSG00000218891	346.5303848	582.0348156	-0.748259458	0.002446621	0.007679794	ZNF579
ENSG00000167220	59.76030106	26.66926817	1.165615784	0.002447276	0.007680922	HDHD2
ENSG00000185324	924.5480869	1445.545846	-0.644700515	0.002449034	0.007685511	CDK10
ENSG00000286532	75.79062018	27.27498425	1.470783035	0.002452671	0.007694821	PARTICL
ENSG00000143869	46.07429872	2.894762044	3.995783797	0.002452763	0.007694821	GDF7
ENSG00000100865	429.0452288	732.9431121	-0.772950675	0.002452889	0.007694821	CINP
ENSG00000106123	57.77677962	8.195692577	2.803212557	0.00245582	0.007703084	EPHB6
ENSG00000259433	7.517487729	0	5.244951176	0.002460359	0.00771639	AC051619.3
ENSG00000279633	12.51672828	0.723690511	4.120094024	0.00246221	0.007721264	AL137918.1
ENSG00000091536	44.17728009	11.56740821	1.924220669	0.002464244	0.007726709	MYO15A
ENSG00000158428	7.241869235	0	5.190605702	0.002465159	0.007728647	CATIP
ENSG00000267361	7.272099347	0	5.195985368	0.002466398	0.007731597	SEC24AP1
ENSG00000163528	205.9615886	342.0385691	-0.732040211	0.002468278	0.007736559	CHCHD4
ENSG00000167555	327.3385663	187.1551995	0.806119741	0.002471905	0.007746835	ZNF528
ENSG00000172932	1399.314214	935.6019734	0.580858971	0.002472153	0.007746835	ANKRD13D
ENSG00000165171	28.42300674	102.655954	-1.85397619	0.002486846	0.007791937	METTL27
ENSG00000235831	23.55937442	5.63611618	2.051402609	0.002492794	0.007809631	BHLHE40-AS1
novel.604	0.61420451	12.02544858	-4.30789036	0.002495406	0.007816372	-
ENSG00000153201	1611.220467	2388.547596	-0.568022868	0.002495547	0.007816372	RANBP2
ENSG00000175193	242.0431767	145.1121755	0.737968448	0.002496017	0.007816902	PARL
ENSG00000198003	31.31368845	8.732725059	1.849178575	0.002498017	0.007822221	CCDC151
ENSG00000180190	281.4985869	141.5793463	0.993262315	0.002502859	0.00783644	TDRP
ENSG00000261156	0	6.908735928	-5.367453323	0.002503257	0.007836742	LINC01989
ENSG00000130764	845.9504234	1374.746111	-0.700674883	0.00250373	0.007837277	LRRC47
ENSG00000092094	810.555812	432.8521834	0.904694855	0.002504422	0.007838501	OSGEP
ENSG00000113460	385.3382402	821.2269452	-1.091992908	0.002510359	0.007856137	BRIX1
ENSG00000274825	0	7.430579604	-5.476613642	0.002511441	0.007858574	AL023803.2
ENSG00000112706	22.03528853	4.73882027	2.228607219	0.002514055	0.007865807	IMPG1
ENSG00000107485	17.16094284	1.41669944	3.586673802	0.0025149	0.007867504	GATA3
ENSG00000261490	167.619979	50.86210884	1.716924826	0.00251666	0.007872063	AC005674.2
ENSG00000250486	50.83309491	16.2554778	1.638578562	0.00251861	0.007877213	FAM218A
ENSG00000218350	0.308355904	8.723268448	-4.741492184	0.002521958	0.007886733	LYPLA1P3
ENSG00000132821	19.41137815	185.5542354	-3.258020501	0.002525982	0.007897909	VSTM2L
ENSG00000104237	4.513264919	25.85096438	-2.51081327	0.002526139	0.007897909	RP1
ENSG00000122873	218.501879	529.2720844	-1.276836342	0.002530254	0.007909823	CISD1
ENSG00000172250	50.94169233	12.51288626	2.03342122	0.002533153	0.00791793	SERHL

ENSG00000235385	0	10.15875272	-5.921871861	0.002534447	0.007921022	LINC02154
ENSG00000145088	59.23295467	18.19402092	1.693871715	0.002535998	0.007924918	EAF2
ENSG00000175806	67.52625429	15.06172177	2.170417298	0.002536594	0.007925827	MSRA
ENSG00000225178	632.6982022	1509.810633	-1.254765174	0.002537626	0.007928098	RPSAP58
ENSG00000136098	445.4629995	200.6403582	1.149790936	0.002539769	0.00793384	NEK3
ENSG00000188690	705.7113661	1180.799109	-0.742829302	0.002545022	0.007949294	UROS
ENSG00000061938	2059.517283	834.5969733	1.303496878	0.00255164	0.007969006	TNK2
ENSG00000066136	808.267494	477.587913	0.758466103	0.002555587	0.007980374	NFYC
ENSG00000058091	396.0994815	964.4112453	-1.284087068	0.002556457	0.00798213	CDK14
ENSG00000272275	12.93945165	0.672939833	4.19882816	0.002559177	0.007989664	AC092687.3
ENSG00000068615	16.47374161	2.496928964	2.715583477	0.00256172	0.007996641	REEP1
novel.598	14.7199953	0	6.214622339	0.002566208	0.008009687	-
ENSG00000187824	192.5728457	65.00556653	1.56369539	0.002567234	0.008011928	TMEM220
novel.851	8.535824346	0	5.426522729	0.002568445	0.008014744	-
ENSG00000286086	9.392838668	0.336469917	4.603359224	0.002571148	0.008022216	AC099661.1
ENSG00000109466	510.4338794	289.1866526	0.819547297	0.002574398	0.008030654	KLHL2
ENSG00000243566	11.09049861	0	5.805666111	0.00257447	0.008030654	UPK3B
ENSG00000232022	9.96169801	0.336469917	4.688823245	0.002575388	0.008031628	FAAHP1
ENSG00000145198	33.7367289	4.148596966	3.005922219	0.002575401	0.008031628	VWA5B2
ENSG00000241158	22.83907979	1.069617038	4.411563435	0.002586738	0.008066014	ADAMTS9-AS1
ENSG00000253187	0	9.632461113	-5.853004595	0.002588495	0.008070526	HOXA10-AS
ENSG00000137177	1244.075142	832.9059965	0.579020125	0.00259008	0.008074497	KIF13A
ENSG00000103544	834.7143305	1272.555316	-0.608250109	0.00259274	0.008081822	VPS35L
ENSG00000151914	7839.719627	4009.323386	0.967466275	0.002599384	0.008101559	DST
ENSG00000158815	19.2690744	1.844058227	3.403342451	0.002601094	0.008105917	FGF17
ENSG00000172425	14.36059546	1.110911105	3.720096939	0.002602665	0.008109839	TTC36
ENSG00000231204	11.9287082	0.387220594	4.949981691	0.002603859	0.008112587	AC011752.1
ENSG00000226051	8.355626049	0	5.39812295	0.002605031	0.008115265	ZNF503-AS1
ENSG00000144283	1789.818795	793.1469535	1.173818283	0.002608969	0.008126559	PKP4
ENSG00000286695	1.54052587	22.41660131	-3.870191524	0.002612767	0.008137412	AC026316.5
ENSG00000197102	6986.248384	11300.44761	-0.693772694	0.002613401	0.008138413	DYNC1H1
ENSG00000136872	10.03351234	0.356539013	4.698022159	0.002617015	0.008148691	ALDOB
ENSG00000198793	984.9401164	1569.794583	-0.672298831	0.00262168	0.008162236	MTOR
ENSG00000111911	688.4254426	379.6269039	0.858839015	0.002623213	0.00816603	HINT3
ENSG00000116962	2522.673603	8318.9693	-1.721489838	0.002624988	0.008170577	NID1
ENSG00000102010	9.721324223	0.336469917	4.652506381	0.002625478	0.008171124	BMX
ENSG00000092931	451.0279354	275.1983194	0.711834994	0.002626373	0.008172931	MFSD11
ENSG00000167676	10.30780592	35.63953033	-1.793691491	0.00262954	0.008181805	PLIN4
ENSG00000160124	153.4977482	284.6570468	-0.891024324	0.002633417	0.008192888	CCDC58
ENSG00000157985	1464.618494	758.1255933	0.950386771	0.002634687	0.008195857	AGAP1
ENSG00000197565	19.61016771	82.38354822	-2.07360286	0.002640098	0.008211706	COL4A6
ENSG00000256050	34.50882293	7.733927794	2.170761888	0.002647463	0.008232794	AL583722.1
novel.34	11.86978636	1.080229524	3.460103626	0.002647511	0.008232794	-
ENSG00000110025	39.74342267	9.020884443	2.125677122	0.002648688	0.008235467	SNX15
ENSG00000116539	1613.091646	955.1806544	0.756166889	0.002649484	0.008236957	ASH1L
ENSG00000146001	11.8357953	0.693008929	4.05543943	0.002650496	0.008239117	PCDHB18P
ENSG00000168237	78.24325	162.4869935	-1.053759371	0.002656226	0.008254978	GLYCTK
ENSG00000130066	8089.746753	2674.964413	1.596662656	0.002656234	0.008254978	SAT1
ENSG00000267072	8.577337297	0	5.43301529	0.002657221	0.008257057	NAGPA-AS1
ENSG00000088882	2578.398444	353.7489265	2.865397172	0.002658134	0.008258906	CPXM1
ENSG00000273893	37.65291346	8.926271159	2.092974375	0.002659485	0.008262117	AL133520.1

ENSG00000241456	8.944615042	0	5.496276181	0.002662143	0.008269385	AC005996.1
ENSG00000176915	1317.276274	2565.6154	-0.961599196	0.00266359	0.008272891	ANKLE2
ENSG00000250651	8.078753907	0	5.349278777	0.002664077	0.008273414	PABPC1P7
ENSG00000141750	4.311968057	51.79256084	-3.589476424	0.002665455	0.008276704	STAC2
ENSG00000233836	13.31077508	1.803920035	2.888693195	0.002668857	0.008286278	AC139769.1
ENSG00000100578	261.7450749	500.2655406	-0.935007919	0.002669191	0.008286325	KIAA0586
ENSG00000140350	1603.676594	2681.272067	-0.741572521	0.002672357	0.008295162	ANP32A
ENSG00000196526	1123.05776	2650.506075	-1.238957861	0.00267523	0.008303087	AFAP1
novel.778	10.02599045	0	5.659132651	0.002675986	0.008304444	-
ENSG00000116514	462.4943295	259.8728269	0.83131237	0.002676309	0.008304454	RNF19B
ENSG00000148840	619.2916092	1136.064839	-0.875366732	0.002677655	0.008307639	PPRC1
ENSG00000254681	39.24734658	174.1265526	-2.149326899	0.002678639	0.008309166	PKD1P5
ENSG00000179912	721.5882144	419.9135481	0.781771707	0.002678787	0.008309166	R3HDM2
ENSG00000146205	17.842916	57.05245409	-1.674179343	0.002685801	0.008329925	ANO7
ENSG00000224795	7.922873484	0	5.318966736	0.002687051	0.008332808	NTM-AS1
ENSG00000261402	7.503626321	0	5.242515191	0.002687905	0.008334463	AL591222.1
ENSG00000287670	16.45074866	1.447381022	3.515348891	0.002688927	0.008336637	AC108479.3
ENSG00000258301	19.57087733	3.903015919	2.318630628	0.002697714	0.008362682	VASH1-AS1
ENSG00000272631	44.63588892	16.45963639	1.437314775	0.002698128	0.008362682	AC067750.1
ENSG00000232815	163.2036449	45.90769996	1.833100486	0.002698549	0.008362682	DUX4L50
ENSG00000286177	28.25527366	74.19145166	-1.395331836	0.002698615	0.008362682	AC011462.5
ENSG00000100241	2058.219238	3506.082117	-0.768361472	0.002701048	0.008369222	SBF1
ENSG00000196693	294.7241844	184.7216887	0.674396378	0.002703771	0.00837666	ZNF33B
ENSG00000259321	10.2398238	0.387220594	4.728861738	0.002708856	0.008391414	AL136295.2
ENSG00000133466	286.6148878	539.5336568	-0.912334269	0.002712532	0.008401799	C1QTNF6
ENSG00000160746	554.8143649	1081.438842	-0.962645279	0.002716309	0.008412494	ANO10
ENSG00000131080	422.6350433	138.6118036	1.606798691	0.002717479	0.008415116	EDA2R
ENSG00000249158	10.07126435	0.356539013	4.702866379	0.00272624	0.00843992	PCDHA11
ENSG00000230623	3.632288727	24.58882872	-2.754232676	0.002726362	0.00843992	AC104461.1
ENSG00000164707	123.224847	34.07374252	1.857479412	0.002726463	0.00843992	SLC13A4
ENSG00000170275	4178.007641	6848.182304	-0.712894063	0.002727698	0.008442737	CRTAP
ENSG00000142507	1458.492065	2206.900257	-0.597489079	0.002728721	0.008444896	PSMB6
ENSG00000120729	17.01620306	1.069617038	3.984815145	0.002734744	0.008462527	MYOT
ENSG00000170509	14.78457248	2.140389951	2.783963431	0.002735646	0.00846431	HSD17B13
ENSG00000213923	1568.016329	3306.588233	-1.076289141	0.002736667	0.008466461	CSNK1E
ENSG00000215440	613.9143493	315.6993495	0.959997893	0.002737183	0.00846705	NPEPL1
ENSG00000164924	9166.416577	12572.44956	-0.455838523	0.0027376	0.008467332	YWHAZ
ENSG00000122548	9.825591233	0	5.631664586	0.002738995	0.008470639	KIAA0087
ENSG00000152578	45.00922144	8.4577467	2.405643348	0.00274074	0.008475027	GRIA4
ENSG00000131379	9.352508095	0.336469917	4.597948942	0.00274119	0.008475407	C3orf20
ENSG00000196233	814.9763778	451.3391368	0.852815886	0.002743342	0.008480974	LCOR
ENSG00000153982	80.6091208	22.26550516	1.863775316	0.002743643	0.008480974	GDPD1
ENSG00000117419	785.9232085	1275.916433	-0.698948357	0.002744835	0.00848365	ERI3
ENSG00000131165	1343.654823	2148.979168	-0.677447173	0.002747563	0.008491071	CHMP1A
ENSG00000235310	12.28025798	0	5.951230975	0.002750515	0.008499184	GXYLT1P6
ENSG00000141232	1375.504363	642.6135616	1.097551503	0.002755416	0.008513315	TOB1
ENSG00000204381	363.0644295	827.4828885	-1.188288745	0.002758249	0.008521053	LAYN
ENSG00000054967	143.9191547	277.7117558	-0.947625699	0.002760469	0.0085269	RELT
ENSG00000156103	111.1753334	235.5501619	-1.083465327	0.002765457	0.008541291	MMP16
ENSG00000149679	110.0594966	198.9574479	-0.854345075	0.002774509	0.008568231	CABLES2
ENSG00000214756	29.93921474	69.03922432	-1.206691204	0.002776242	0.008572564	CSKMT

ENSG00000091732	230.2490369	385.4369766	-0.743304391	0.002778053	0.008577135	ZC3HC1
ENSG00000166707	33.68490202	10.62997411	1.656245516	0.002780327	0.008583135	ZCCHC18
ENSG00000283051	6.976351202	0	5.135921797	0.002781172	0.008584726	LINC01668
ENSG00000166441	12442.57788	19526.18782	-0.650120929	0.002782258	0.008587058	RPL27A
ENSG00000089351	1242.120213	2725.976209	-1.133839626	0.002783091	0.008588272	GRAMD1A
ENSG00000061936	2289.066318	1024.42355	1.159977946	0.002783313	0.008588272	SFSWAP
ENSG00000273033	228.2101407	115.7377102	0.981395428	0.002783999	0.008589369	LINC02035
ENSG00000247828	504.0895281	266.4901926	0.919623531	0.002785701	0.0085936	TMEM161B-AS1
ENSG00000253669	8.976098803	40.42768862	-2.166421735	0.002787713	0.008598785	GASAL1
ENSG00000261798	12.42861651	0.387220594	5.007420541	0.002789847	0.008604348	AL033527.3
ENSG00000189077	781.1972376	442.8383582	0.818660225	0.002797032	0.008625484	TMEM120A
ENSG00000196150	279.7883192	136.3682772	1.038628823	0.002797921	0.008627201	ZNF250
ENSG00000272690	65.23141454	13.73835135	2.250275157	0.00280049	0.008634097	LINC02018
ENSG00000173156	103.5638492	389.952224	-1.913558137	0.002801419	0.008635937	RHOD
ENSG00000168710	1720.586636	2703.347921	-0.651784958	0.002802321	0.00863769	AHCYL1
ENSG00000114988	437.3374883	665.5274771	-0.606093282	0.002806723	0.008650235	LMAN2L
ENSG00000130045	4.180947147	26.91484922	-2.680165798	0.002812654	0.008667483	NXNL2
ENSG00000120594	4313.371094	120.4690941	5.161770434	0.002812994	0.008667504	PLXDC2
ENSG00000073670	51.9029024	15.75614337	1.710086777	0.002814292	0.008670475	ADAM11
ENSG00000026025	58188.36705	34783.37762	0.742333334	0.002818885	0.008683596	VIM
ENSG00000135119	113.2414813	30.506473	1.886125733	0.00282774	0.008709843	RNFT2
ENSG00000254827	12.50913512	0.693008929	4.135747043	0.002828316	0.008710584	SLC22A18AS
ENSG00000279332	18.52363552	2.211209725	3.087177786	0.00283018	0.008714675	AC090772.4
ENSG00000162441	955.5898754	561.20762	0.767343461	0.002830315	0.008714675	LZIC
ENSG00000176142	688.0460427	1053.129686	-0.61384604	0.002833092	0.008722189	TMEM39A
ENSG00000241839	1319.668522	811.7972391	0.701161069	0.002836162	0.008730606	PLEKHO2
ENSG00000188383	10.46626491	0.356539013	4.761366766	0.002841952	0.008747394	GPAT2P2
ENSG00000270562	22.2355452	4.555886656	2.270183969	0.002847035	0.008762002	AC097634.1
ENSG00000187398	32.67945755	0.356539013	6.401934086	0.002849118	0.008767372	LUZP2
novel.68	10.18563182	0.356539013	4.721991768	0.002850134	0.00876946	-
ENSG00000227115	7.273352996	0	5.196214655	0.002854209	0.008780959	LINC01630
ENSG00000287016	0	6.501446238	-5.280818642	0.00285587	0.008784388	AC010996.1
novel.515	42.75788814	112.2701438	-1.394552548	0.002856	0.008784388	-
ENSG00000287326	0	6.45069556	-5.270926031	0.002864352	0.008809035	AL589787.1
ENSG00000087470	1383.96017	2026.241237	-0.550046315	0.002865513	0.008811564	DNM1L
ENSG00000090554	24.71471546	4.921882279	2.3159157	0.002871732	0.008829641	FLT3LG
ENSG00000197506	0.293240848	8.76340664	-4.747438464	0.002874047	0.008834474	SLC28A3
ENSG00000235865	190.7563179	52.53097343	1.861791846	0.002874316	0.008834474	GSN-AS1
novel.484	306.1939275	509.1789223	-0.733287717	0.002874324	0.008834474	-
ENSG00000056277	77.09335083	144.2478257	-0.902729507	0.00287598	0.008838519	ZNF280C
ENSG00000137478	807.3587353	402.1771949	1.004914727	0.002877526	0.008842223	FCHSD2
ENSG00000267707	14.27491972	1.518200796	3.281382851	0.002878785	0.008845047	AC015961.2
ENSG00000196730	405.6431846	58.12417295	2.80136155	0.002887895	0.008871989	DAPK1
ENSG00000179869	105.5987991	10.85189004	3.283496516	0.002891231	0.008881187	ABCA13
novel.757	7.522502324	0	5.245765388	0.002893779	0.008887964	-
ENSG00000173436	179.2631383	95.90332287	0.900691297	0.002895303	0.008891592	MICOS10
ENSG00000157191	1111.757931	1871.172015	-0.751122981	0.002898987	0.008901855	NECAP2
ENSG00000136436	2255.208968	1384.795468	0.703637547	0.002901507	0.008908539	CALCOCO2
ENSG00000250305	10.43374096	0	5.715937401	0.002916379	0.008953112	TRMT9B
ENSG00000205542	51795.59397	27249.5313	0.926588427	0.002917039	0.008953112	TMSB4X
ENSG00000101049	23.58591486	3.300895859	2.849392436	0.002917058	0.008953112	SGK2

ENSG00000286000	12.77060747	0.356539013	5.048466063	0.002917903	0.008954648	AC234775.4
ENSG00000008277	93.74729004	22.40984164	2.062521914	0.002920492	0.008961532	ADAM22
ENSG00000269929	254.2243682	111.7429535	1.185964118	0.002920973	0.008961951	AL158152.1
ENSG00000164597	60.94175526	151.3709	-1.312353996	0.002925581	0.008975028	COG5
ENSG00000102910	1144.540033	1623.68511	-0.504462038	0.002926372	0.00897551	LONP2
ENSG00000110881	64.49742852	198.2837721	-1.619136193	0.002926429	0.00897551	ASIC1
ENSG00000086015	954.6409876	1450.148266	-0.603080607	0.002928581	0.00898105	MAST2
ENSG00000105254	3303.297049	1947.60898	0.762191633	0.002932583	0.008992262	TBCB
ENSG00000083750	397.2698551	223.8895062	0.82830572	0.002933293	0.008993377	RRAGB
ENSG00000165916	2166.851418	3541.596866	-0.708882418	0.002933859	0.008994053	PSMC3
novel.190	20.89004795	2.975038428	2.834602919	0.002935208	0.008997127	-
ENSG00000223561	9.073128655	0.336469917	4.553747552	0.002938106	0.00900478	AC005165.1
ENSG00000213433	28.73218902	8.937012039	1.682371071	0.002938773	0.00900478	RPLP1P6
ENSG00000131188	128.4736645	333.3543986	-1.375668471	0.002939036	0.00900478	PRR7
ENSG00000119772	705.6793296	431.3605886	0.710098265	0.002939091	0.00900478	DNMT3A
ENSG00000197520	25.30649648	2.0188195	3.60995996	0.002943111	0.009016031	FAM177B
ENSG00000177535	7.30232946	0	5.20139941	0.002943611	0.0090165	OR2B11
ENSG00000132256	354.4360907	657.8220944	-0.892715705	0.002945019	0.009019311	TRIM5
ENSG00000136942	9167.787973	14676.69613	-0.67889894	0.002945223	0.009019311	RPL35
ENSG00000182890	69.36557689	22.80895899	1.597626074	0.002950413	0.00903414	GLUD2
ENSG00000270055	215.959615	83.35654419	1.371146253	0.002950904	0.009034578	AC127502.2
ENSG00000109193	0.293240848	180.138729	-9.112336401	0.002952608	0.00903873	SULT1E1
ENSG00000240990	2.135854377	20.44536884	-3.265367986	0.002953021	0.009038929	HOXA11-AS
ENSG00000123815	660.0406304	412.4728824	0.677538127	0.00295649	0.009048481	COQ8B
ENSG00000273183	21.36689204	3.486269618	2.597899365	0.002957949	0.009051882	AC093726.2
ENSG00000198336	6.963743444	0	5.133591817	0.002961655	0.009062155	MYL4
ENSG00000174915	145.4664477	284.408208	-0.96696715	0.002965687	0.009073372	PTDSS2
ENSG00000166025	1086.123247	1835.647632	-0.757248354	0.002966019	0.009073372	AMOTL1
ENSG00000254413	12.46887581	1.049547942	3.54410836	0.002968933	0.009081216	CHKB-CPT1B
ENSG00000280231	2.140868972	13.96155155	-2.713874443	0.002972594	0.009091343	AL031719.2
ENSG00000135049	366.7623071	237.4985	0.626708679	0.00297459	0.009096378	AGTPBP1
ENSG00000266208	44.53363066	146.4835181	-1.718623852	0.002975325	0.009097154	AC080112.1
ENSG00000161021	79.57574075	12.01586357	2.730459555	0.002975544	0.009097154	MAML1
ENSG00000137496	179.5944593	87.42173548	1.040972557	0.002976886	0.009100185	IL18BP
ENSG00000140382	311.549262	491.0770273	-0.656193893	0.002978001	0.009102523	HMG20A
ENSG00000130158	759.0488899	474.0301685	0.679091364	0.002982439	0.009115017	DOCK6
ENSG00000162231	2022.201901	1144.592216	0.820997186	0.002989415	0.009135262	NXF1
novel.176	8.170769163	0	5.364263887	0.002994232	0.009148366	-
ENSG00000172000	17.59000573	3.200550379	2.451342853	0.002994408	0.009148366	ZNF556
ENSG00000243795	15.32821631	1.069617038	3.835062916	0.002994808	0.009148515	LINC02044
ENSG00000066455	786.9533974	1245.489824	-0.662199417	0.00299534	0.009149063	GOLGA5
ENSG00000133863	1.229662669	13.74793635	-3.498208811	0.002995816	0.009149442	TEX15
ENSG00000233818	0.894837599	29.4668484	-5.031212154	0.002997064	0.009152178	AP000695.2
ENSG00000139668	422.3773486	787.6115301	-0.898511983	0.003000672	0.009162117	WDFY2
ENSG00000245958	243.0989527	110.6157789	1.135622624	0.003001569	0.009163182	AC093752.1
ENSG00000129911	471.6308251	758.8037302	-0.68598088	0.003002056	0.009163182	KLF16
ENSG00000135763	144.2416571	327.3423759	-1.183176671	0.003002079	0.009163182	URB2
ENSG00000140057	2.447971227	18.76875145	-2.941510559	0.003005374	0.009172161	AK7
ENSG00000272416	10.89435888	0.336469917	4.817516182	0.00301382	0.009196858	AC025175.1
ENSG00000244128	0	6.726958189	-5.333292471	0.003018186	0.0092091	LINC01322
ENSG00000215399	12.38918358	0	5.966144217	0.003022692	0.009221765	HMGB3P7

ENSG00000227740	9.932721546	0.387220594	4.685040074	0.003024263	0.009225476	AL513329.1
ENSG00000272142	30.55850512	78.93857267	-1.370301215	0.003025911	0.009228592	AL359643.3
ENSG00000028839	216.2499053	439.8142729	-1.024590167	0.003025995	0.009228592	TBPL1
novel.479	757.5885958	1836.594293	-1.277610304	0.003037063	0.009261259	-
ENSG00000133895	536.5833581	866.1611919	-0.690628134	0.003042563	0.009276942	MEN1
ENSG00000278771	81.17279517	255.7975579	-1.655026238	0.003048589	0.009294226	RN7SL3
ENSG00000132849	257.3299075	139.6532607	0.880691908	0.00305168	0.009302557	PATJ
ENSG00000099284	209.531293	673.5444615	-1.684863271	0.003054353	0.009309612	H2AFY2
ENSG00000173653	295.8228975	494.4841892	-0.741520306	0.003059581	0.009324455	RCE1
novel.163	0.307102255	8.265228081	-4.666075981	0.00306231	0.009331676	-
ENSG00000198816	902.9805543	465.9195049	0.955250995	0.003062696	0.009331759	ZNF358
ENSG00000275764	187.3511381	95.96006253	0.964166926	0.003064528	0.009336246	AC092747.4
ENSG00000241859	35.39445771	0.672939833	5.674394506	0.003065792	0.009339003	ANOS2P
ENSG00000108848	4998.573573	3115.283257	0.682231414	0.003066773	0.009340894	LUC7L3
ENSG00000173258	23.02081646	4.872287477	2.220529819	0.003070558	0.009351327	ZNF483
ENSG00000166033	4733.42261	8261.825664	-0.803533378	0.003071347	0.009352633	HTRA1
ENSG00000163870	807.7181667	1456.35539	-0.850179959	0.003072972	0.009356484	TPRA1
ENSG00000092871	285.2424507	170.2289652	0.74515038	0.003082861	0.009385494	RFFL
ENSG00000078142	775.0454325	1267.550948	-0.70945132	0.003084717	0.009390044	PIK3C3
ENSG00000224321	0	15.20104957	-6.505972334	0.003086106	0.009393173	RPL12P14
ENSG00000153936	478.2483463	929.8363927	-0.959129669	0.003086881	0.009394432	HS2ST1
ENSG00000178385	301.6149743	166.4744769	0.858607661	0.003095861	0.009420656	PLEKHM3
ENSG00000003402	2645.154242	1599.255956	0.726144063	0.0030988	0.009428496	CFLAR
ENSG00000177599	94.14623788	33.58861659	1.489235416	0.003105368	0.009447373	ZNF491
ENSG00000136527	1978.172847	3075.716595	-0.636854701	0.003106737	0.009450432	TRA2B
ENSG00000176834	330.1013761	168.8399121	0.966237715	0.003108803	0.009455608	VSIG10
ENSG00000267169	90.31461795	44.87017716	1.01280773	0.003111036	0.009461292	AC022098.1
ENSG00000102287	1433.942784	557.2202053	1.363799084	0.003115836	0.009474783	GABRE
ENSG00000174720	1023.992462	713.3740581	0.521278382	0.003120926	0.009489149	LARP7
ENSG00000103021	76.71024602	172.8904754	-1.172329786	0.003125303	0.009501346	CCDC113
ENSG00000076242	548.5165389	992.5481523	-0.855983418	0.003130185	0.009514848	MLH1
ENSG00000235453	118.0379579	55.09774187	1.096415337	0.003130477	0.009514848	SMIM27
ENSG00000111859	322.2810015	1190.266362	-1.885109669	0.003132655	0.009520353	NEDD9
ENSG00000226091	18.9659469	0.672939833	4.760983476	0.003135534	0.009527989	LINC00937
ENSG00000175634	545.033451	914.7357958	-0.746756458	0.003136948	0.009531011	RPS6KB2
ENSG00000213862	66.34254935	139.410505	-1.073201403	0.003137262	0.009531011	AC044787.1
ENSG00000102854	14.69792782	0.672939833	4.386053474	0.003139735	0.009537409	MSLN
ENSG00000179840	8.566054458	0	5.431320065	0.003140392	0.00953829	PIK3CD-AS1
ENSG00000153012	38.31310388	3.741307276	3.334340352	0.003142669	0.009543078	LGI2
ENSG00000122779	488.4461742	285.8117733	0.772622271	0.003142703	0.009543078	TRIM24
ENSG00000276842	10.57339522	0.336469917	4.774604785	0.003143764	0.009545183	AC023510.2
ENSG00000101911	412.2047359	749.6671955	-0.8633414	0.0031474	0.009555106	PRPS2
ENSG00000267102	82.62083542	37.45033844	1.143319762	0.003154221	0.009574696	AC060766.1
ENSG00000070081	761.998506	1509.612832	-0.986271106	0.00315721	0.009582647	NUCB2
ENSG00000119537	2180.970192	1292.06221	0.755430284	0.003160593	0.009591796	KDSR
ENSG00000100201	14593.50024	7398.391215	0.980069826	0.003162812	0.00959741	DDX17
ENSG00000146757	244.1822069	111.826008	1.12537922	0.003164893	0.009601724	ZNF92
ENSG00000115738	1778.290666	387.9487814	2.196124126	0.003165111	0.009601724	ID2
novel.173	22.33132141	74.91218845	-1.747221593	0.003165343	0.009601724	-
ENSG00000255224	41.46944617	12.87186541	1.685118769	0.003170347	0.009615782	AC109322.1
ENSG00000028528	2893.601275	1933.687425	0.58144775	0.00317191	0.009619398	SNX1

ENSG00000133317	7.822082686	0	5.302048548	0.003174133	0.009625016	LGALS12
ENSG00000177464	73.57417558	334.6790408	-2.185054692	0.003174983	0.009626473	GPR4
ENSG00000137185	315.3565644	183.6933657	0.7802397	0.003176628	0.009629617	ZSCAN9
ENSG00000133703	1308.822133	836.0694908	0.646519244	0.003176762	0.009629617	KRAS
ENSG00000274461	11.72239674	0.743759607	4.01662653	0.003184521	0.00965201	AC023282.1
ENSG00000170323	108.1197375	6.15680398	4.125837237	0.003188279	0.009662273	FABP4
ENSG00000168275	283.3548046	503.1000145	-0.828788521	0.003190755	0.00966865	COA6
ENSG00000145354	422.5052771	652.6699014	-0.627708163	0.00319619	0.009683988	CISD2
ENSG00000286615	14.19809079	1.4981317	3.2821537	0.003199752	0.009693652	AC011416.4
ENSG00000255980	8.184630571	0	5.366522948	0.003203512	0.00970391	AP000439.3
ENSG00000114547	13.15231609	1.080229524	3.607374355	0.00321188	0.009728126	ROPN1B
ENSG00000129159	15.47560592	1.762625967	3.118471474	0.003214652	0.009735387	KCNC1
ENSG00000179094	600.5960402	1551.76168	-1.369332462	0.003217518	0.009742929	AC129492.1
novel.1043	63.61850487	0	8.325169918	0.003221969	0.009755271	-
ENSG00000180785	7.862341989	0	5.308831448	0.003230636	0.009779488	OR51E1
ENSG00000221340	25.92263882	3.964379082	2.713041919	0.00323072	0.009779488	RNU6ATAC18P
ENSG00000272791	19.90702731	3.139187216	2.645987276	0.003232817	0.009784695	AC073389.3
ENSG00000162676	34.54463711	7.796575227	2.140651143	0.003233771	0.009786442	GFI1
ENSG00000233264	9.231587646	0	5.541769197	0.003235114	0.009789366	AC006042.3
ENSG00000285410	531.8747096	302.342615	0.815110962	0.003236617	0.009792773	GABPB1-IT1
ENSG00000046651	686.357345	415.6396219	0.723733734	0.003241473	0.009806325	OFD1
ENSG00000271392	7.81699682	0	5.301176459	0.00324273	0.009808983	AC006237.1
ENSG00000180957	799.3528963	1400.441868	-0.809164599	0.003246985	0.009820713	PITPNB
ENSG00000132554	39.67747714	3.515795324	3.4876071	0.003248117	0.009822992	RGS22
ENSG00000169905	1027.887504	1506.350673	-0.551356262	0.003252042	0.009833719	TOR1AIP2
ENSG00000215251	217.7433776	375.0483453	-0.784637378	0.003256603	0.009845354	FASTKD5
ENSG00000272864	14.18290446	1.467450118	3.292433281	0.003256648	0.009845354	AC135803.1
ENSG00000197362	184.3451204	107.1280966	0.783575181	0.003271391	0.009888773	ZNF786
ENSG00000156256	1286.849471	866.5720297	0.570428666	0.003273465	0.009893893	USP16
ENSG00000151247	700.2806555	1154.444991	-0.721405146	0.003279577	0.009911212	EIF4E
ENSG00000105700	1054.137304	1547.935927	-0.554193172	0.003282959	0.009919415	KXD1
ENSG00000283294	8.952208206	0	5.497318557	0.003283055	0.009919415	AP005212.4
ENSG00000138668	3995.094289	5802.732119	-0.538501199	0.00328778	0.009932538	HNRNPD
ENSG00000273853	6.944867441	0	5.130080979	0.003290794	0.009940487	AC023158.2
ENSG00000187866	258.1305785	128.3847528	1.006113528	0.00329132	0.009940919	FAM122A
ENSG00000128567	556.6132986	257.8430566	1.109351587	0.003292503	0.009943337	PODXL
ENSG00000137818	17233.51608	27530.8712	-0.675835736	0.003296532	0.009954348	RPLP1
ENSG00000101751	888.2626988	418.2660712	1.087157826	0.003300812	0.009966114	POLI
ENSG00000115946	266.2669294	559.7790367	-1.072618842	0.003302681	0.009970597	PNO1
ENSG00000157992	41.50537506	11.14706589	1.897876819	0.003305249	0.00997719	KRTCAP3
ENSG00000180879	2097.628241	3388.978117	-0.692097469	0.003314634	0.010004356	SSR4
ENSG00000134077	513.7003727	778.3795435	-0.599847234	0.003319027	0.010016451	THUMPD3
ENSG00000198932	301.0434498	168.0447127	0.841602575	0.003320084	0.010018479	GPRASP1
ENSG00000287276	27.21122333	1.874739808	3.884336788	0.00332253	0.010024696	BX664718.2
ENSG00000180758	163.5623883	321.2797063	-0.972972287	0.003323748	0.010027205	GPR157
ENSG00000090316	1132.752391	1697.374462	-0.583407213	0.003325371	0.010030939	MAEA
ENSG00000006534	2096.626167	1093.973257	0.938758247	0.003327877	0.01003733	ALDH3B1
ENSG00000136944	10.44391269	0	5.717324957	0.003336454	0.010062032	LMX1B
ENSG00000225868	7.960625489	0	5.32534742	0.003337947	0.010065367	AC016582.1
ENSG00000133316	312.7372692	546.182616	-0.805069634	0.003339095	0.010067661	WDR74
ENSG00000116030	1439.451495	2217.308512	-0.623244974	0.003342187	0.010075812	SUMO1

ENSG00000261187	7.250716047	0	5.192152329	0.003343807	0.010079527	AC079322.1
ENSG00000259570	11.70853534	0.743759607	4.01485224	0.003350314	0.010097969	AC243562.1
ENSG00000095383	329.8157549	1064.944888	-1.691320731	0.003351576	0.010100602	TBC1D2
ENSG00000126790	432.0925033	631.1743426	-0.546670366	0.003352662	0.010102705	L3HYPDH
ENSG00000166575	476.9699209	292.3931107	0.705988607	0.003356715	0.010113744	TMEM135
ENSG00000149311	2154.576601	1229.787663	0.809199387	0.00336137	0.010126594	ATM
ENSG00000105186	440.8392546	746.8506768	-0.760275833	0.003364344	0.010134379	ANKRD27
ENSG00000288048	10.04730248	0.387220594	4.699871868	0.003366734	0.010140402	AC104088.3
ENSG00000177675	47.48004308	283.2757083	-2.576336843	0.003368827	0.010145531	CD163L1
ENSG00000168079	878.0606158	19.8764989	5.46366856	0.003374295	0.01016082	SCARA5
ENSG00000188313	1052.553624	1888.701071	-0.8436391	0.003391453	0.010211303	PLSCR1
ENSG00000203666	293.3721342	164.8454938	0.831577993	0.003397478	0.010228259	EFCAB2
ENSG00000279662	26.40868572	5.992655192	2.130801648	0.003402118	0.010241042	AC131649.2
ENSG00000143891	267.8562763	138.7899384	0.949011271	0.003405188	0.010249096	GALM
ENSG00000161204	991.0600662	1506.34653	-0.603840168	0.003407725	0.010255542	ABCF3
ENSG00000100982	1022.768584	606.7694281	0.753685682	0.003408638	0.010257103	PCIF1
novel.221	33.47572726	11.81170499	1.502305557	0.003410312	0.010260951	-
ENSG00000279645	16.44573407	1.069617038	3.937223045	0.003412344	0.01026508	AC016251.1
ENSG00000241506	107.7389988	199.4581138	-0.88988029	0.003412474	0.01026508	PSMC1P1
ENSG00000249364	16.79950641	0.713078025	4.552017267	0.003448754	0.010373013	AC112206.2
ENSG00000104983	219.4634176	350.016023	-0.673830997	0.003450761	0.010377847	CCDC61
ENSG00000156049	30.5789195	6.359806691	2.25668388	0.003451692	0.010379445	GNA14
ENSG00000177830	1172.492547	2027.026325	-0.789863027	0.003461659	0.010408213	CHID1
ENSG00000237419	16.92823349	0	6.414583545	0.003462483	0.010409486	AL954642.1
ENSG00000082701	821.1852057	1278.693147	-0.638768485	0.003466732	0.010420376	GSK3B
ENSG00000280073	8.258880932	0	5.378607557	0.003466908	0.010420376	AL157996.1
ENSG00000134490	155.0389858	269.4139668	-0.797131287	0.003471157	0.010431941	TMEM241
ENSG00000228288	24.22171613	107.7116418	-2.153404218	0.003472743	0.010435501	PCAT6
ENSG00000172995	20.71403788	1.702418679	3.569541677	0.003474728	0.010440256	ARPP21
ENSG00000248360	12.11134252	0.713078025	4.078444191	0.003480636	0.010456799	LINC00504
ENSG00000168724	1239.813179	799.5706325	0.632753347	0.003481145	0.010457121	DNAJC21
ENSG00000169026	198.9255323	64.50507622	1.621608799	0.003487742	0.010475725	SLC49A3
ENSG00000105948	112.5163856	225.0463005	-1.001364324	0.003489835	0.010480801	TTC26
ENSG00000198964	943.940847	403.494028	1.225491178	0.003494472	0.010493513	SGMS1
ENSG00000142541	25984.5452	46133.06931	-0.828149281	0.003494987	0.010493846	RPL13A
ENSG00000279926	38.35662593	9.804653847	1.973592152	0.003500911	0.01051042	AL138831.3
novel.1018	6.667995298	0	5.070819567	0.003502843	0.010515004	-
ENSG00000087053	676.8446532	1076.056037	-0.669132213	0.003514943	0.010550107	MTMR2
ENSG00000189180	698.9500195	395.7225525	0.82130627	0.003517494	0.010556545	ZNF33A
ENSG00000158122	423.9336303	268.6705924	0.658069828	0.003517961	0.010556727	PRXL2C
ENSG00000124194	10.15916265	0.336469917	4.718464603	0.003521271	0.01056544	GDAP1L1
ENSG00000238057	19.33433576	2.731897526	2.789606673	0.0035228	0.010568808	ZEB2-AS1
ENSG00000198373	909.1959087	590.2756079	0.622686055	0.003530131	0.010589578	WWP2
ENSG00000271046	7.614517581	0	5.261770399	0.003533922	0.010599727	AL512631.1
ENSG00000231246	61.51641178	6.614844348	3.2091155	0.003538087	0.010610995	AL445426.1
ENSG00000101425	37.23319558	2.395427609	3.937283818	0.00354228	0.010622342	BPI
ENSG00000105325	900.273148	1366.077373	-0.601391082	0.003544779	0.010628609	FZR1
ENSG00000153443	370.7629134	609.0572711	-0.715624644	0.003551152	0.010646491	UBALD1
ENSG00000172031	3.286109548	23.58913237	-2.835839996	0.003553751	0.010653052	EPHX4
ENSG00000148358	1517.169321	2307.820096	-0.605218451	0.003555212	0.010655831	GPR107
ENSG00000276663	18.74506203	1.793307549	3.386652791	0.003555498	0.010655831	AC009090.3

novel.287	0	14.70903557	-6.46180755	0.003558399	0.010663295	-
ENSG00000154229	2402.955751	1151.547575	1.061420304	0.003571516	0.010701367	PRKCA
ENSG00000258539	12.0458677	1.049547942	3.496339997	0.003588911	0.010752249	AC068896.1
ENSG00000189419	12.82383053	1.029478846	3.595170178	0.003595994	0.010772228	SPATA41
ENSG00000286107	8.667742899	0	5.450609915	0.003603805	0.01079438	AC007655.2
ENSG00000164011	283.4068887	170.955995	0.729937397	0.003619914	0.010841381	ZNF691
ENSG00000146151	28.91761537	1.874739808	3.969067012	0.003623082	0.010849619	HMGCLL1
ENSG00000220749	200.1085249	79.53033736	1.333915797	0.003626058	0.010856694	RPL21P28
ENSG00000260386	0	14.61069787	-6.448361578	0.00362628	0.010856694	LINC01225
ENSG00000265203	10.74600035	0	5.758567769	0.003629512	0.010865117	RBP3
ENSG00000182771	80.16390232	12.93078843	2.637277091	0.003630921	0.010868084	GRID1
ENSG00000239857	45.47492488	97.89128521	-1.104300253	0.003639005	0.010891026	GET4
novel.158	0	15.56922855	-6.539687772	0.003642156	0.010899201	-
ENSG00000126070	822.5215884	459.7456192	0.839626908	0.003644798	0.010905851	AGO3
ENSG00000182162	85.50484909	10.83053667	2.987712683	0.003646981	0.010911124	P2RY8
ENSG00000143127	678.0372415	2442.731238	-1.849181637	0.00364816	0.010913396	ITGA10
ENSG00000184523	12.16650342	0.387220594	4.978652274	0.003649087	0.010914913	PTGER4P2
ENSG00000232527	3.374292587	23.82068049	-2.828696169	0.003658057	0.010940437	AC245595.1
ENSG00000198205	82.70296368	40.94490879	1.014325783	0.003658463	0.010940437	ZXDA
ENSG00000270210	9.305909278	0.356539013	4.59149153	0.003662509	0.010951278	AC104695.3
ENSG00000133958	14.69506452	0.774441189	4.316716082	0.003666191	0.010961024	UNC79
ENSG00000186352	107.1192811	245.7070824	-1.198703882	0.003673808	0.010982534	ANKRD37
ENSG00000107147	14.83493224	1.702418679	3.07994101	0.003675298	0.010985723	KCNT1
novel.1087	11.5409448	0	5.86372544	0.003679151	0.010995976	-
ENSG00000267640	49.93868424	8.338487999	2.572043942	0.003683306	0.011006093	AC016582.3
ENSG00000139163	1509.638732	944.2738832	0.677144897	0.003683713	0.011006093	ETNK1
ENSG00000182208	789.6096621	446.4054996	0.823250023	0.003683807	0.011006093	MOB2
ENSG00000275026	8.870649415	0	5.481859854	0.003685642	0.01101031	GXYLT1P4
ENSG00000226194	17.19751246	1.049547942	4.01230792	0.003689272	0.011019887	LINC02519
ENSG00000137033	142.539195	33.44594956	2.090095873	0.003692733	0.011028957	IL33
ENSG00000182173	427.7173974	214.9157893	0.991850494	0.003695159	0.011033883	TSEN54
ENSG00000253369	7.542703246	0	5.249242858	0.003695232	0.011033883	AC022034.3
ENSG00000231881	3.308817768	34.23532277	-3.366854096	0.003696024	0.01103498	AL109615.2
ENSG00000133135	9.740271497	45.99614868	-2.23818764	0.003700015	0.011045627	RNF128
ENSG00000179532	471.5020145	204.1475832	1.208626195	0.003702189	0.011050524	DNHD1
ENSG00000148229	587.1737853	1079.168678	-0.878388989	0.003702506	0.011050524	POLE3
ENSG00000250159	28.39994252	8.846123169	1.674433728	0.003718077	0.011095723	AC106791.1
ENSG00000167600	48.2277758	149.9407631	-1.635350617	0.00371929	0.011098066	CYP2S1
ENSG00000196873	233.3726573	74.66703977	1.64362284	0.003721483	0.011103337	CBWD3
ENSG00000171700	614.370348	301.7172493	1.026609231	0.003736498	0.011146855	RGS19
ENSG00000088682	526.609879	830.1963774	-0.657009241	0.003741979	0.011161923	COQ9
ENSG00000248810	60.85761825	11.30175807	2.424905294	0.003743221	0.011164346	LINC02432
ENSG00000223797	109.9980675	47.26000097	1.214386847	0.003751367	0.011187357	ENTPD3-AS1
ENSG00000254991	0	7.428139459	-5.469806506	0.003758014	0.011205894	AC124276.1
ENSG00000276291	272.5953423	168.6127307	0.693200487	0.003758593	0.011206334	FRG1HP
ENSG00000078319	156.2555425	275.906436	-0.820558572	0.00376285	0.011217739	PMS2P1
ENSG00000111863	1.846374476	16.99666887	-3.2089648	0.003763777	0.011219216	ADTRP
ENSG00000183513	844.6219516	515.014959	0.714258914	0.003772218	0.011243089	COA5
ENSG00000162980	1033.262383	607.6949652	0.765753894	0.003777187	0.011256606	ARL5A
ENSG00000254139	159.3695591	1.069617038	7.216368815	0.003779097	0.011261007	AC104051.2
ENSG00000162728	8.379587918	0	5.401765897	0.003783642	0.011273259	KCNJ9

ENSG00000149541	1158.872933	2022.80696	-0.803800999	0.003787446	0.011282641	B3GAT3
ENSG00000159346	1632.534205	2546.897681	-0.641610195	0.00378766	0.011282641	ADIPOR1
ENSG00000176101	799.7740086	1264.61199	-0.661180789	0.00379123	0.011291982	SSNA1
ENSG00000158488	19.41639274	0	6.612499001	0.003794591	0.011300694	CD1E
ENSG00000114933	29.50359887	8.938167914	1.72195289	0.003796113	0.011303934	INO80D
ENSG00000180229	627.7414148	148.2196064	2.082394829	0.00380584	0.0113316	HERC2P3
ENSG00000185829	29.94032585	5.502648973	2.454371276	0.003806949	0.0113336	ARL17A
ENSG00000198792	1532.146834	2673.619606	-0.803127064	0.003809387	0.011339561	TMEM184B
ENSG00000275964	88.24341202	43.15012953	1.032644272	0.003810045	0.011340218	AL355001.2
ENSG00000083720	460.8321277	1014.559822	-1.138891853	0.003810807	0.01134119	OXCT1
ENSG00000182308	27.41029763	4.646775526	2.559807065	0.003813375	0.01134753	DCAF4L1
ENSG00000149150	132.0880458	308.6006975	-1.224538708	0.003814202	0.011348691	SLC43A1
ENSG00000055130	1159.688834	1721.564208	-0.569906841	0.003815946	0.011352582	CUL1
ENSG00000159314	40.73174229	2.659793482	3.958558737	0.003816422	0.011352697	ARHGAP27
ENSG00000273650	17.24912588	0	6.441680562	0.003826203	0.01138049	AC100793.4
ENSG00000134709	21.44343623	2.171071533	3.311061111	0.003827765	0.011382812	HOOK1
ENSG00000103043	563.6919396	1071.791611	-0.926690352	0.00382786	0.011382812	VAC14
novel.634	3.926783224	23.46846101	-2.572933651	0.003831794	0.011393207	-
ENSG00000080189	942.8361768	1478.165226	-0.648676104	0.003833323	0.011396449	SLC35C2
ENSG00000163875	1148.207211	770.5206935	0.575847567	0.003836397	0.011404004	MEAF6
ENSG00000134986	50.44805227	104.1852808	-1.048821795	0.003836742	0.011404004	NREP
ENSG00000115598	2.158491326	28.32466059	-3.723371502	0.003840374	0.011413492	IL1RL2
ENSG00000159063	416.2709317	656.7424599	-0.658290439	0.003844254	0.011423718	ALG8
ENSG00000147647	13.83575636	0.387220594	5.161194877	0.00384966	0.01143774	DPYS
ENSG00000266777	21.36187744	4.677457107	2.194015756	0.003849853	0.01143774	SH3GL1P1
ENSG00000086475	776.8405058	1272.749915	-0.712541271	0.003852009	0.011442837	SEPHS1
ENSG00000072135	1258.258429	840.8458938	0.58137096	0.003852892	0.011444151	PTPN18
ENSG00000248008	76.58641883	153.4248622	-1.00406368	0.00385815	0.011458459	NRAV
ENSG00000198131	217.9600743	351.2252246	-0.68850565	0.003862129	0.011468963	ZNF544
ENSG00000158545	814.3816894	1269.618759	-0.640602467	0.003866654	0.011481091	ZC3H18
ENSG00000178175	8.592452353	0	5.435544933	0.003867612	0.011481974	ZNF366
ENSG00000147224	471.4991631	902.7374578	-0.937435325	0.003867835	0.011481974	PRPS1
ENSG00000230067	10.72705308	0.336469917	4.793698139	0.003872299	0.01149391	HSPD1P6
ENSG00000112378	391.0479694	2254.747821	-2.52766031	0.003874184	0.011498193	PERP
ENSG00000253738	722.0346981	375.9179133	0.940962798	0.003874864	0.011498898	OTUD6B-AS1
ENSG00000254802	8.257698554	0	5.378464495	0.003876838	0.01150344	AC022182.2
ENSG00000250539	31.28442726	8.396255142	1.907198915	0.003897961	0.011563917	KRT8P33
ENSG00000160796	884.0647693	504.8692586	0.808315379	0.003898109	0.011563917	NBEAL2
ENSG00000287632	7.019189073	0	5.143960761	0.003900185	0.011568753	AC112253.1
ENSG00000130590	79.95945777	31.77896436	1.325860848	0.00390332	0.01157673	SAMD10
ENSG00000033050	340.9301173	607.3933464	-0.833519235	0.003907545	0.011587939	ABCF2
ENSG00000242540	0	6.706889093	-5.329229885	0.003914208	0.011606372	AC010729.2
ENSG00000223774	14.67876709	0.672939833	4.388294452	0.003922866	0.011630717	AL513217.1
ENSG00000204581	6.995227205	0	5.139483502	0.003926772	0.011640969	ACOXL-AS1
ENSG00000157851	11.38653149	0	5.842064414	0.003939548	0.011677103	DPYSL5
ENSG00000080839	174.8852685	323.8421077	-0.889173194	0.003939859	0.011677103	RBL1
ENSG00000276251	7.771651652	0	5.293391439	0.003940954	0.011679014	AC007786.2
ENSG00000093072	977.9933473	285.749909	1.774434415	0.003945088	0.011689933	ADA2
ENSG00000252498	18.11824976	1.386017859	3.684223749	0.003945679	0.01169035	RNU6-1016P
novel.766	6.903283219	0	5.122233594	0.003952134	0.011706898	-
ENSG00000145241	477.4502965	297.2858525	0.682645609	0.003952165	0.011706898	CENPC

ENSG00000099998	681.7439214	3486.562877	-2.354588595	0.003953596	0.011709801	GGT5
ENSG00000156697	259.8573297	565.0191176	-1.12105993	0.003955696	0.011714687	UTP14A
ENSG00000214851	11.92773929	0.693008929	4.067613067	0.00396356	0.011736637	LINC00612
ENSG00000273523	12.33151539	0.672939833	4.131860612	0.003967111	0.011745813	AL139082.1
ENSG00000124214	1651.174974	2353.849602	-0.511619541	0.003973646	0.011763821	STAU1
ENSG00000106477	231.6444235	409.1298746	-0.821321883	0.003975044	0.01176662	CEP41
ENSG00000211772	39.93060122	3.108505634	3.667977512	0.003976087	0.011768365	TRBC2
ENSG00000173705	138.5028746	439.123143	-1.665130337	0.003978854	0.01177435	SUSD5
ENSG00000152382	362.2776633	200.5073578	0.853439416	0.003979015	0.01177435	TADA1
ENSG00000109189	845.0523645	428.8154775	0.978425962	0.003979554	0.011774603	USP46
ENSG00000247516	127.2805437	71.16656164	0.837842859	0.003982085	0.011780751	MIR4458HG
ENSG00000136522	470.4300676	749.4682556	-0.672259572	0.003989935	0.011802632	MRPL47
ENSG00000168427	8.485393311	0.356539013	4.456738061	0.003995074	0.011816487	KLHL30
ENSG00000162733	2370.942025	3770.166221	-0.669186591	0.003997594	0.011822065	DDR2
ENSG00000197063	744.0938205	1360.502209	-0.870396379	0.00399787	0.011822065	MAFG
ENSG00000237638	39.72218156	9.245112124	2.099730649	0.003998918	0.011823818	LINC02245
ENSG00000167244	123.5426898	11.22148168	3.457479039	0.003999435	0.011824001	IGF2
novel.202	6.696971762	0	5.076447597	0.004000426	0.011825586	-
ENSG00000277566	2.111892509	16.46549698	-2.957544714	0.004007286	0.011844517	AC089999.2
ENSG00000249751	12.84897478	0.723690511	4.157328627	0.004009207	0.011848847	ECSCR
ENSG00000232591	8.127931293	0	5.357212825	0.004012613	0.011856216	LINC02642
ENSG00000196542	8.127931293	0	5.357212825	0.004012613	0.011856216	SPTSSB
ENSG00000078328	10.14809328	0	5.675801745	0.004014502	0.011859185	RBFOX1
ENSG00000112305	79.39923177	141.1395769	-0.830100674	0.00401481	0.011859185	SMAP1
ENSG00000162222	290.3989237	523.8433717	-0.851376722	0.004014987	0.011859185	TTC9C
ENSG00000215039	576.4511506	319.1392348	0.852895646	0.004015735	0.011860046	CD27-AS1
ENSG00000133063	6.625157428	0	5.062495157	0.004018527	0.01186694	CHIT1
ENSG00000159377	3025.204203	4330.878276	-0.517639965	0.004019456	0.011868334	PSMB4
ENSG00000162300	102.4476998	192.8292709	-0.911263225	0.004027775	0.011890552	ZFPL1
ENSG00000255153	44.38040007	14.63436299	1.608328619	0.004027895	0.011890552	TOLLIP-AS1
ENSG00000162753	7.017935424	0	5.143740634	0.004030412	0.011896629	SLC9C2
ENSG00000152904	431.2267844	267.9591841	0.687242975	0.004043602	0.011934208	GGPS1
ENSG00000271228	13.89600313	1.161661783	3.646538889	0.00404776	0.011945123	AL121655.1
ENSG00000129534	311.0881296	780.4112272	-1.327129345	0.004048302	0.011945365	MIS18BP1
ENSG00000205177	3.269740843	19.42290645	-2.559531289	0.00405924	0.011976074	C11orf91
ENSG00000229415	19.3562176	0	6.608101984	0.004059631	0.011976074	SFTA3
ENSG00000154734	897.5646014	2083.78885	-1.215253649	0.004063774	0.011986934	ADAMTS1
ENSG00000078295	33.51147017	2.375358513	3.792883877	0.004068388	0.01199918	ADCY2
ENSG00000174804	1042.866807	416.0450447	1.325170406	0.004070286	0.012003416	FZD4
ENSG00000182272	2603.129272	818.3733673	1.669662869	0.00407612	0.012019257	B4GALNT4
ENSG00000050030	10.3997499	0.356539013	4.749167411	0.004079505	0.012027874	NEXMIF
ENSG00000115084	1135.002	1734.451916	-0.611911512	0.004082191	0.012034428	SLC35F5
ENSG00000186056	40.30719615	10.49894705	1.931453973	0.004084308	0.012039303	MATN1-AS1
ENSG00000286681	48.06498639	15.55185639	1.622816948	0.004101397	0.012088304	AF235103.3
ENSG00000225400	11.50723847	1.41669944	3.009208737	0.004106365	0.012101572	RAB28P5
ENSG00000152683	424.794077	674.5917914	-0.667713105	0.004107043	0.012102198	SLC30A6
ENSG00000126391	1640.696289	917.6316919	0.838023786	0.004112212	0.012116057	FRMD8
ENSG00000156858	919.810149	573.1467907	0.682892395	0.004114127	0.012120324	PRR14
ENSG00000169826	1560.481147	837.6160274	0.897656494	0.004116125	0.012124835	CSGALNACT2
ENSG00000134317	27.9799399	2.099095884	3.722191808	0.004122286	0.012141607	GRHL1
ENSG00000187239	1093.442698	484.2680074	1.174691964	0.004124429	0.012146542	FNBP1

ENSG00000268069	0.586481695	11.22148168	-4.231195191	0.004127995	0.012155667	AC004466.1
ENSG00000099949	860.4990843	490.2319891	0.811871317	0.004128548	0.012155917	LZTR1
ENSG00000164904	1206.463956	1720.678166	-0.512124659	0.004136129	0.012176858	ALDH7A1
ENSG00000108784	1062.827897	741.3117553	0.519766497	0.004142423	0.012194007	NAGLU
ENSG00000160972	710.8644401	348.5074887	1.028860043	0.004145038	0.012200321	PPP1R16A
novel.650	40.73808216	90.94510803	-1.161284973	0.00414862	0.012209481	-
ENSG00000162704	3535.265401	6211.095339	-0.813093198	0.004152269	0.012218837	ARPC5
ENSG00000272777	21.76719193	5.697479343	1.928986057	0.004153564	0.012221263	AC019131.2
ENSG00000267547	82.44844375	31.57480577	1.378616926	0.004158054	0.012233088	AC060766.4
ENSG00000268673	8.899269874	0	5.489182746	0.004161561	0.01224202	AC004597.1
novel.468	446.7496737	1157.132475	-1.37320392	0.004171034	0.012268497	-
ENSG00000172733	61.9066119	20.25986668	1.613334871	0.004173625	0.012274729	PURG
ENSG00000136636	1819.983536	977.0710372	0.897375101	0.004174185	0.012274985	KCTD3
ENSG00000227725	12.0458677	1.467450118	3.058887749	0.004179668	0.012289719	GCOM2
ENSG00000130811	2242.376887	3447.352117	-0.620448414	0.004182161	0.012295657	EIF3G
ENSG00000115902	816.3279573	241.1085831	1.758624592	0.004190059	0.012317484	SLC1A4
ENSG00000224934	77.53022035	38.64512195	1.003187358	0.004191126	0.012319225	AL391684.1
ENSG00000272574	4.51201127	19.35324256	-2.097005264	0.004196388	0.012333299	AL596325.2
ENSG00000142327	1663.460405	1019.059446	0.707298227	0.004209484	0.012370387	RNPEPL1
ENSG00000146530	37.67673279	6.544024575	2.509961726	0.004217539	0.012392658	VWDE
ENSG00000280145	25.72699729	4.199347643	2.600655968	0.004222602	0.012406132	CU638689.4
ENSG00000087263	653.6364288	1139.981306	-0.802777624	0.004223172	0.012406404	OGFOD1
ENSG00000244699	8.72193488	0.356539013	4.49777384	0.004225515	0.012411883	AC139453.2
ENSG00000274220	38.61351062	11.9746979	1.679584812	0.004231198	0.01242717	AC009163.6
ENSG00000256594	37.19111316	12.30171121	1.585858016	0.004235365	0.012438004	AC010186.2
ENSG00000120725	804.0920433	1462.879289	-0.863494276	0.00424221	0.012456696	SIL1
ENSG00000272750	55.22704903	17.5800041	1.651153136	0.004244621	0.012462367	AL592148.3
ENSG00000178028	920.843245	546.7686013	0.751754559	0.004246998	0.012467938	DMAP1
ENSG00000143155	558.9313329	897.8972049	-0.684260414	0.004251932	0.012481013	TIPRL
ENSG00000163017	115.2006852	34.95957404	1.716205644	0.004253433	0.012484008	ACTG2
ENSG00000217716	34.64019985	82.29672209	-1.247235386	0.004258424	0.012497245	RPS10P3
ENSG00000184967	277.0937052	498.6673678	-0.848203934	0.004261644	0.012505283	NOC4L
ENSG00000114503	1440.038974	2211.663209	-0.619043726	0.004262565	0.012506574	NCBP2
ENSG00000113645	128.0523804	429.5616522	-1.745747688	0.004266807	0.012517605	WWC1
ENSG00000285954	7.628378988	0	5.264240944	0.004278187	0.012549574	AC119428.3
ENSG00000232377	14.40816319	1.069617038	3.746019904	0.004279172	0.012551045	AC016910.1
novel.544	11.93400753	0.693008929	4.068743478	0.004280405	0.012551882	-
ENSG00000242258	14.03050025	0.774441189	4.250366517	0.004280423	0.012551882	LINC00996
ENSG00000100902	69.61737606	139.6447372	-1.003207315	0.004291168	0.012581969	PSMA6
ENSG00000121775	249.9273289	389.7614436	-0.641369033	0.004291959	0.01258287	TMEM39B
ENSG00000080854	53.62151871	12.56363693	2.10274808	0.004299122	0.012602446	IGSF9B
ENSG00000188611	8.168190595	29.18613784	-1.833429001	0.004303307	0.012613291	ASAH2
ENSG00000129245	1268.674664	1781.035707	-0.489275509	0.004308311	0.012626534	FXR2
ENSG00000056558	699.0113071	277.982449	1.331152302	0.004310461	0.01263141	TRAF1
ENSG00000259658	100.627581	40.76886325	1.30695268	0.004316181	0.012646746	AC027559.1
ENSG00000268001	82.12951667	27.89126564	1.556428412	0.004318107	0.012650963	CARD8-AS1
ENSG00000247199	33.74500624	8.795244097	1.95050765	0.004319327	0.012653109	AC011346.1
ENSG00000091622	104.2915663	22.37556404	2.221118358	0.004321406	0.012657773	PITPNM3
ENSG00000287853	14.54545234	2.51699806	2.531995195	0.004326473	0.012671188	AL031668.2
ENSG00000239213	121.2008532	60.52340651	1.0041374	0.004330861	0.012682608	NCK1-DT
ENSG00000167965	618.009633	940.8335911	-0.606450301	0.004332706	0.01268658	MLST8

ENSG00000136866	107.4955482	38.97542731	1.468608684	0.004333892	0.012688623	ZFP37
ENSG00000154721	638.9615767	166.284176	1.943011744	0.004338844	0.01270169	JAM2
ENSG00000138814	588.3218896	889.1718215	-0.596011716	0.004339663	0.012702656	PPP3CA
ENSG00000113456	373.927889	618.2886667	-0.725945635	0.004351502	0.012735875	RAD1
ENSG00000111652	1232.645401	1877.840893	-0.607487283	0.004357753	0.012752735	COPS7A
ENSG00000159212	109.3205666	19.25862927	2.509035527	0.004380374	0.012817492	CLIC6
ENSG00000287825	35.56394264	12.20953807	1.542074966	0.004381618	0.012819686	AL035587.2
ENSG00000271270	45.21964985	119.8527187	-1.406259061	0.004382855	0.012821861	TMCC1-AS1
ENSG00000240661	5.087138855	26.07801739	-2.355321306	0.00438592	0.012829385	AC063952.1
ENSG00000263266	808.5903079	1349.651432	-0.739232022	0.00438684	0.01283063	RPS7P1
ENSG00000153029	629.7804231	197.0086903	1.67554029	0.004387669	0.01283161	MR1
ENSG00000102119	996.3955704	1577.634431	-0.662993627	0.004388651	0.012833039	EMD
ENSG00000249637	19.74480738	3.587770973	2.459984804	0.004389569	0.012834278	AC008438.1
ENSG00000205060	443.0827097	712.397817	-0.685563809	0.004391284	0.012837848	SLC35B4
ENSG00000105369	27.33919496	2.578361223	3.418748431	0.004391812	0.012837946	CD79A
ENSG00000281501	45.35436044	17.18088675	1.409175168	0.004395679	0.012847803	SEPSECS-AS1
ENSG00000230797	16.99342357	42.8539262	-1.336742964	0.004396666	0.012849243	YY2
ENSG00000137267	1579.242245	849.7891773	0.893703965	0.004416093	0.012904567	TUBB2A
ENSG00000120509	563.5508023	854.5365769	-0.600707796	0.004416656	0.012904761	PDZD11
ENSG00000156508	126271.643	192299.821	-0.606827371	0.004422959	0.012921724	EEF1A1
ENSG00000196646	198.300587	103.7693864	0.935406406	0.004425195	0.012926803	ZNF136
ENSG00000247903	26.46155279	7.092953812	1.893671106	0.004435108	0.012954305	AC024896.1
ENSG00000105426	3245.947927	1407.115478	1.206076047	0.004436335	0.012956431	PTPRS
ENSG00000136696	0	7.040918865	-5.392703494	0.004438011	0.012959871	IL36B
ENSG00000275450	11.98032161	0.743759607	4.046144879	0.004440208	0.012964829	AL845472.1
ENSG00000232938	21.05756722	2.731897526	2.915198747	0.004443162	0.012971997	RPL23AP87
ENSG00000287759	8.719427583	0.356539013	4.497384132	0.004444862	0.012975502	AL353151.2
ENSG00000184347	296.5539601	58.7392516	2.337662193	0.00445804	0.013012507	SLIT3
ENSG00000177731	183.116826	475.5350737	-1.376265919	0.004465054	0.013031519	FLII
ENSG00000175984	78.52968061	30.12614048	1.37806352	0.004468332	0.01303962	DENND2C
ENSG00000283633	25.5307863	1.396630344	4.176088931	0.004478171	0.013066864	AP000547.3
ENSG00000119778	326.1182489	154.7087364	1.074051526	0.004484502	0.013083869	ATAD2B
ENSG00000076650	295.2761765	186.4288121	0.664203556	0.004495092	0.013113293	GPATCH1
ENSG00000251664	7.973233248	0	5.327602607	0.004504735	0.013139948	PCDHA12
ENSG00000159256	746.5424335	454.0964504	0.717655632	0.004508073	0.01314821	MORC3
ENSG00000095564	1883.846043	1085.774725	0.795144429	0.004510084	0.013152598	BTAF1
ENSG00000273341	15.63803932	0.713078025	4.44699733	0.004512042	0.013156832	AC004921.1
novel.488	5.452194037	22.71781333	-2.058009773	0.004519276	0.013176446	-
ENSG00000254585	25.14810876	3.506338714	2.829274971	0.004520319	0.013177834	MAGEL2
ENSG00000232053	7.345167331	0	5.209256763	0.004520767	0.013177834	AC078845.1
ENSG00000122482	853.7859413	555.5499621	0.620251171	0.00452353	0.013184171	ZNF644
novel.565	6.374754451	0	5.005698951	0.004523955	0.013184171	-
ENSG00000226403	6.362146692	0	5.003146143	0.00452666	0.013190575	AL392089.1
ENSG00000158158	320.2914416	177.7622021	0.849775772	0.004529395	0.013196848	CNNM4
ENSG00000147119	113.5655214	349.5454439	-1.622399252	0.004530129	0.013196848	CHST7
ENSG00000112306	7279.77512	14374.89998	-0.981598526	0.004530337	0.013196848	RPS12
ENSG00000279187	1.524157165	11.14822176	-2.876173983	0.004538486	0.013219104	AC027601.5
ENSG00000286254	6.694464465	0	5.075998575	0.004542113	0.013227112	AC091889.1
ENSG00000274925	75.55407861	37.19774093	1.0212578	0.004542254	0.013227112	ZKSCAN2-DT
ENSG00000086544	373.2142465	590.0069571	-0.660666684	0.004547137	0.013239696	ITPKC
ENSG00000273509	6.679349408	0	5.073053403	0.004547594	0.013239696	CNTNAP3P1

ENSG00000181444	223.5317495	86.26302774	1.37638386	0.004558619	0.013270305	ZNF467
ENSG00000255135	53.706439	108.6441612	-1.018171758	0.004561154	0.013276197	AP002360.1
ENSG00000100292	809.7405342	427.8755564	0.92053798	0.004565601	0.013287654	HMOX1
ENSG00000077147	3356.652541	5018.151297	-0.580121158	0.004566249	0.01328805	TM9SF3
ENSG00000100612	1257.857217	815.3041386	0.625608764	0.004567022	0.013288811	DHRS7
ENSG00000255222	8.034662387	0	5.341702677	0.004569383	0.013294191	SETP17
ENSG00000271447	6.61129602	0	5.059739181	0.004571526	0.013296837	MMP28
ENSG00000270806	6.61129602	0	5.059739181	0.004571526	0.013296837	C17orf50
ENSG00000257800	36.11607727	10.3029608	1.805661475	0.004571827	0.013296837	FNBP1P1
ENSG00000144724	391.1015645	1231.172281	-1.654533974	0.004573022	0.013298822	PTPRG
novel.198	19.42320298	0	6.614075146	0.004575052	0.013303238	-
ENSG00000197965	1059.926407	4093.527432	-1.949446101	0.004576831	0.01330692	MPZL1
ENSG00000240445	88.21185699	153.8900002	-0.803720251	0.004579581	0.013313427	FOXO3B
ENSG00000272158	11.19888257	0.774441189	3.931348564	0.004581434	0.013316247	AL139022.2
ENSG00000132965	546.0628696	87.67698307	2.640100244	0.004581576	0.013316247	ALOX5AP
ENSG00000255395	9.520098632	0	5.583670621	0.004586685	0.013329604	AP001972.4
ENSG00000132423	111.594039	200.8722447	-0.848791698	0.004588312	0.013332841	COQ3
ENSG00000079385	25.97607535	5.064677702	2.361659772	0.004611504	0.013398734	CEACAM1
ENSG00000136378	751.973643	1073.429704	-0.513649611	0.004612282	0.013399496	ADAMTS7
ENSG00000215883	174.4548807	78.52670666	1.148559132	0.004613462	0.013401425	CYB5RL
ENSG00000138594	910.7467166	1837.402485	-1.012689047	0.004625497	0.013434884	TMOD3
novel.537	9.191613078	0	5.533131748	0.004633952	0.013456877	-
ENSG00000185519	0.615458159	12.41968564	-4.347238958	0.004634287	0.013456877	FAM131C
ENSG00000110756	1544.488621	810.3481664	0.930234922	0.004634906	0.013456877	HPS5
ENSG00000180488	461.1792911	704.2659961	-0.610792883	0.004635141	0.013456877	MIGA1
ENSG00000143198	1298.404446	1887.942928	-0.540112214	0.004638497	0.013465116	MGST3
ENSG00000007516	47.94692925	14.26489974	1.756020024	0.004639866	0.013467584	BAIAP3
ENSG00000013016	484.9128219	1109.574357	-1.194198005	0.004641357	0.013469851	EHD3
ENSG00000263006	79.96619708	10.80357951	2.882665821	0.004641684	0.013469851	ROCK1P1
ENSG00000237991	48.65963072	101.7223727	-1.065800264	0.004660027	0.013521573	RPL35P1
ENSG00000130939	801.2498158	1297.403442	-0.695016093	0.004660881	0.013522542	UBE4B
ENSG00000183779	918.6081473	457.706277	1.004829471	0.004662219	0.013524913	ZNF703
ENSG00000279191	5.099746614	21.05322109	-2.037515118	0.004669027	0.01354315	AC068491.4
ENSG00000234451	10.13018619	0.336469917	4.71449547	0.004678652	0.013569553	BX005266.3
ENSG00000100888	1161.425302	1770.852539	-0.608411606	0.004685942	0.01358918	CHD8
ENSG00000076662	16.86644834	1.386017859	3.577614746	0.004690971	0.013601126	ICAM3
ENSG00000186615	31.56792365	74.52321687	-1.242504909	0.004691109	0.013601126	KTN1-AS1
ENSG00000237525	0.600343103	8.947624524	-3.889387607	0.004696242	0.013614489	AC012668.3
novel.745	8.505522962	45.62279826	-2.426868925	0.004704997	0.013638349	-
ENSG00000119013	551.051353	1103.096905	-1.001474009	0.004711446	0.013655521	NDUFB3
ENSG00000222043	9.108373362	0.336469917	4.558701059	0.004714091	0.013661663	AC079305.1
ENSG00000211895	10.45010967	0	5.718245698	0.00471473	0.01366199	IGHA1
ENSG00000105221	2265.044663	3279.178838	-0.533703457	0.004716144	0.013664562	AKT2
ENSG00000085433	532.1219642	340.5138183	0.643721753	0.004717903	0.013666845	WDR47
ENSG00000196922	342.6018345	205.3301788	0.737908049	0.004717984	0.013666845	ZNF252P
ENSG00000181788	280.9891038	491.5731981	-0.807132761	0.004719456	0.013669585	SIAH2
ENSG00000101276	7.024203668	0	5.144973132	0.004730885	0.013701162	SLC52A3
ENSG00000272156	22.11204619	5.482579877	2.023583594	0.004734747	0.013710818	AC008280.3
ENSG00000142606	7.015428127	0	5.143331854	0.004735354	0.013711047	MMEL1
ENSG00000114739	263.3264021	115.9653583	1.185339046	0.004736665	0.013713316	ACVR2B
ENSG00000158473	135.2677089	384.3524744	-1.507138429	0.004737562	0.013714385	CD1D

ENSG00000246526	8.58360554	0	5.434228939	0.004740458	0.01372017	LINC002481
ENSG00000128596	62.47038538	161.9618921	-1.374247775	0.004740617	0.01372017	CCDC136
ENSG00000147854	831.0360287	1438.765628	-0.791941938	0.004747361	0.013738157	UHRF2
ENSG00000219881	6.62265013	0	5.061956073	0.004748235	0.013739158	GAPDHP42
ENSG00000204179	43.45554582	7.613641613	2.500153822	0.004749929	0.013741511	PTPN20
ENSG00000223403	132.0607941	238.8407024	-0.854644247	0.004750106	0.013741511	MEG9
ENSG00000261351	43.67898144	16.70406156	1.383085999	0.00475769	0.013761504	AC116913.1
ENSG00000120949	15.88851357	2.089639274	2.902999643	0.004758077	0.013761504	TNFRSF8
novel.388	12.66257951	1.365948763	3.169176944	0.004758874	0.013762277	-
ENSG00000254231	9.98308131	0.723690511	3.79549967	0.004761443	0.013768176	AC103760.1
ENSG00000198156	17.99009215	2.466247382	2.855372817	0.004765893	0.013779111	NPIPB6
ENSG00000178586	8.547178455	0	5.428468275	0.004766286	0.013779111	OR6B3
ENSG00000214552	1.818651661	11.93327544	-2.712890927	0.004767466	0.013780989	COPS8P2
ENSG00000116254	31.202655	3.932541625	2.986335009	0.004769404	0.013785059	CHD5
ENSG00000101204	16.63742866	0	6.389585806	0.004779329	0.013812208	CHRNA4
ENSG00000135597	469.7898643	673.0359741	-0.518912385	0.004789254	0.01383935	REPS1
ENSG00000163636	1183.676647	1919.627433	-0.697631412	0.004791109	0.013843171	PSMD6
ENSG00000174606	839.0704776	512.0645657	0.712717865	0.004793851	0.013849556	ANGEL2
ENSG00000185640	0	14.02034617	-6.388416321	0.004797126	0.013857475	KRT79
novel.56	9.896151919	33.01717811	-1.732017452	0.004799446	0.013862637	-
ENSG00000188177	495.7140571	234.4445038	1.081350794	0.004808302	0.01388667	ZC3H6
ENSG00000278925	9.469382864	0	5.578686812	0.004810073	0.013888698	AC005753.1
ENSG00000283355	9.469382864	0	5.578686812	0.004810073	0.013888698	AC074194.2
ENSG00000182606	1112.788491	736.9830028	0.594343332	0.004812949	0.013895457	TRAK1
ENSG00000279672	31.95637123	123.7724176	-1.951194513	0.004814857	0.013899423	AP006621.5
ENSG00000005007	1839.25018	2736.45856	-0.573294621	0.004818757	0.013909136	UPF1
ENSG00000230565	33.6190712	10.08921721	1.739930945	0.004822799	0.013919256	ZNF32-AS2
ENSG00000227615	158.0649482	266.570131	-0.754348953	0.004825071	0.013924267	AP001324.1
ENSG00000204634	244.0081774	411.9440566	-0.755394609	0.004830888	0.013939504	TBC1D8
ENSG00000268521	41.92559113	10.69963801	1.968793532	0.004831978	0.013941103	VN1R83P
ENSG00000197894	2096.642212	3498.856624	-0.738874847	0.004836844	0.013953593	ADH5
ENSG00000269293	179.3257498	98.26049167	0.870162986	0.004844065	0.013972873	ZSCAN16-AS1
ENSG00000164114	276.8231292	497.927401	-0.847334658	0.004846208	0.013977501	MAP9
ENSG00000241255	0	6.215726999	-5.214135285	0.004846908	0.013977969	AL136126.1
ENSG00000116852	360.2725441	184.0469038	0.970473076	0.004848234	0.013980242	KIF21B
ENSG00000239557	2.712235611	14.08427787	-2.371194531	0.004854023	0.013995379	AC092045.1
ENSG00000213793	144.0877854	80.05179552	0.845452483	0.004857961	0.014005179	ZNF888
ENSG00000123191	114.9786176	252.5428839	-1.133875119	0.004859784	0.014008881	ATP7B
novel.609	17.81143224	2.862924587	2.635011414	0.004866794	0.014027533	-
ENSG00000255136	6.344524339	0	4.999570623	0.004868524	0.014030962	AP001972.3
ENSG00000196616	3104.732028	50.30397979	5.947127924	0.004871044	0.014036669	ADH1B
novel.419	27.32784085	1.345879667	4.307566067	0.004878644	0.014057008	-
ENSG00000100105	882.5389163	552.1388918	0.676453217	0.004901746	0.014122007	PATZ1
ENSG00000112309	19.97375578	4.717595299	2.089254825	0.004903638	0.014125893	B3GAT2
ENSG00000095209	158.4444343	341.1867924	-1.106670496	0.004905638	0.014130086	TMEM38B
ENSG00000137218	223.9231034	116.3161651	0.947139157	0.004918889	0.014166685	FRS3
ENSG00000185009	625.4903223	1046.366104	-0.742599993	0.004923338	0.014177927	AP3M1
ENSG00000136718	814.83373	1296.884685	-0.670708828	0.004928545	0.014191349	IMP4
ENSG00000126351	1806.057055	1027.832249	0.813542934	0.004930593	0.01419567	THRA
ENSG00000269981	15.65419456	1.90542139	3.083677166	0.004932046	0.014198281	AL627309.7
ENSG00000154114	546.0001311	368.5851922	0.567159962	0.004933478	0.01420083	TBCEL

ENSG00000210176	64.45548829	153.0745218	-1.247786879	0.004935326	0.014204576	MT-TH
ENSG00000124155	1545.596501	2595.614276	-0.74789739	0.004936293	0.014205784	PIGT
ENSG00000273437	15.79280864	1.345879667	3.503146929	0.004942632	0.014222214	AC108673.3
ENSG00000251661	67.96302435	22.95864248	1.570625455	0.004943097	0.014222214	AC136475.1
novel.730	7.185169957	0	5.180096063	0.004946533	0.014230524	-
ENSG00000231125	21.96089563	5.737617535	1.937320387	0.004948642	0.014235017	AF129075.1
ENSG00000165125	7.180155362	0	5.179177744	0.004949574	0.01423612	TRPV6
ENSG00000159111	495.022723	742.0095161	-0.583955309	0.00495892	0.014261423	MRPL10
ENSG00000115963	2626.838851	1455.994706	0.851373232	0.004959571	0.014261717	RND3
ENSG00000254164	6.709579521	0	5.078974024	0.004964637	0.0142738	AC008663.3
ENSG00000135312	1.21705491	15.94981786	-3.719203853	0.004964872	0.0142738	HTR1B
ENSG00000214063	1486.473465	3240.43206	-1.124385233	0.004966223	0.014276105	TSPAN4
ENSG00000163554	6.333170229	0	4.997251745	0.004985689	0.014328892	SPTA1
ENSG00000261522	6.333170229	0	4.997251745	0.004985689	0.014328892	AL845331.3
ENSG00000132694	1138.829914	809.6350287	0.492029028	0.004990505	0.014341147	ARHGEF11
ENSG00000235016	43.7794875	13.51296779	1.693354464	0.004991259	0.014341728	SEMA3F-AS1
ENSG00000070010	697.3730665	1168.4505	-0.744482529	0.005000645	0.014367109	UFD1
ENSG00000171517	2.758834428	39.84037218	-3.85418368	0.005003878	0.014374806	LPAR3
ENSG00000100626	158.9063052	31.28335434	2.340319017	0.005006734	0.014381145	GALNT16
ENSG00000255559	16.111265	2.079026788	2.927329359	0.005007191	0.014381145	ZNF252P-AS1
ENSG00000108651	849.5219248	1295.375204	-0.608855829	0.005010172	0.014388116	UTP6
ENSG00000198855	148.3409029	248.4467543	-0.743585234	0.005013077	0.014394868	FICD
ENSG00000198382	605.5215533	407.5811209	0.570835175	0.005021782	0.014418269	UVRAG
ENSG00000106070	683.7754614	1337.171697	-0.967308035	0.005026345	0.014429776	GRB10
ENSG00000146828	1229.758625	748.6651406	0.715942724	0.005027561	0.014431671	SLC12A9
ENSG00000125966	20.63706641	48.8217604	-1.243322511	0.005050457	0.014495792	MMP24
ENSG00000105976	2003.401097	1086.766647	0.882472494	0.005053744	0.014501381	MET
ENSG00000007080	1225.170751	1791.249859	-0.54809898	0.005053953	0.014501381	CCDC124
ENSG00000237624	10.27632216	29.58525519	-1.523092246	0.005054078	0.014501381	OXCT2P1
ENSG00000162543	8.864238629	0	5.480958149	0.005058234	0.014511701	UBXN10
ENSG00000101017	349.9280976	182.7457859	0.938920894	0.005060774	0.014517385	CD40
ENSG00000204287	392.6460953	0	10.95062144	0.005065442	0.014529174	HLA-DRA
ENSG00000109332	4007.89787	5541.988543	-0.467551361	0.005067373	0.014533107	UBE2D3
ENSG00000229618	8.978677372	0	5.501150279	0.005074666	0.014552416	AC011287.1
ENSG00000106603	617.2297761	922.5331758	-0.579464484	0.005078861	0.014562839	COA1
ENSG00000100650	7210.711602	4292.290939	0.74845525	0.005083119	0.014573441	SRSF5
ENSG00000006118	1620.740977	5700.949885	-1.814524869	0.005090059	0.014591727	TMEM132A
ENSG00000161681	12.63632381	480.6369311	-5.249940383	0.005092428	0.014596908	SHANK1
ENSG00000226674	25.37265548	2.395427609	3.381057622	0.005097998	0.014611261	TEX41
ENSG00000286381	46.37967731	3.741307276	3.614626661	0.005107038	0.014635556	AL078622.1
ENSG00000230561	21.76343098	6.410557368	1.758526826	0.005123581	0.014681346	CCDC192
ENSG00000266235	1.802282956	14.02304311	-2.958902674	0.005124567	0.014682551	MIR3176
ENSG00000166924	125.6742582	278.2776721	-1.145569091	0.005130677	0.014698435	NYAP1
ENSG00000209082	112.1864329	40.58567285	1.472004258	0.005139318	0.014721567	MT-TL1
ENSG00000127774	20.65092782	48.90319266	-1.244611928	0.00514026	0.014722643	EMC6
ENSG00000167785	376.7664591	233.9483458	0.687697086	0.00514713	0.014740695	ZNF558
ENSG00000154079	134.1991554	216.5642808	-0.690162494	0.005152087	0.014753265	SDHAF4
ENSG00000262619	6.701986357	0	5.077497097	0.005158002	0.014768576	LINC00621
ENSG00000116761	46.34203966	104.8782897	-1.178209426	0.005159127	0.014770168	CTH
ENSG00000119698	0	12.93908917	-6.273532096	0.00516171	0.014775936	PPP4R4
ENSG00000120334	91.52081695	219.8594101	-1.265809125	0.005163357	0.014779022	CENPL

ENSG00000144048	315.7507657	514.7016875	-0.705091628	0.00517442	0.014809056	DUSP11
ENSG00000225472	8.704312526	0.336469917	4.495084439	0.0051821	0.014829403	AL136366.1
ENSG00000234663	9.805746316	0.336469917	4.664068844	0.005184858	0.01483566	LINC01934
ENSG00000164889	1642.489925	2510.712295	-0.612201953	0.005190955	0.014851472	SLC4A2
ENSG00000076685	348.4616361	629.3035705	-0.853121133	0.005194072	0.014858753	NT5C2
novel.672	35.46627205	1.936102971	4.227965715	0.005200276	0.014874862	-
ENSG00000248309	30.05039073	5.401147618	2.476391878	0.005212443	0.014908023	MEF2C-AS1
ENSG00000213881	9.323531631	30.40240311	-1.705434845	0.005230731	0.014958681	NPM1P6
ENSG00000101413	462.104501	681.5853332	-0.560654759	0.00523682	0.014974447	RPRD1B
ENSG00000267500	0.586481695	10.03731066	-4.066216264	0.005239338	0.014979999	ZNF887P
ENSG00000223459	0.894837599	18.38183482	-4.354391198	0.005251846	0.015014107	TCAF1P1
ENSG00000101210	3.692748952	34.53268197	-3.228164859	0.005254744	0.01502074	EEF1A2
ENSG00000103995	162.3720873	275.2692204	-0.761661029	0.005259037	0.015031356	CEP152
ENSG00000228630	0.586481695	23.99441428	-5.335260661	0.005270362	0.01506207	HOTAIR
ENSG00000204271	351.9760262	197.6873967	0.831548144	0.005274206	0.015071398	SPIN3
ENSG00000181588	613.9781005	1029.226079	-0.74501804	0.005288213	0.01510976	MEX3D
ENSG00000119616	573.3647116	986.5260998	-0.783292792	0.005291176	0.015116564	FCF1
ENSG00000188171	179.313142	83.84757789	1.093565302	0.005295476	0.015127186	ZNF626
ENSG00000112715	1569.401981	7923.876389	-2.335950421	0.005296487	0.015128412	VEGFA
ENSG00000270184	4.836735879	19.70734142	-2.021530719	0.005301766	0.015141825	AC018695.4
ENSG00000238228	12.34788409	1.41669944	3.113086305	0.005302402	0.015141978	OR7E7P
ENSG00000175886	6.944867441	23.57021915	-1.758672021	0.00530651	0.015152043	RPL7AP66
ENSG00000032444	1533.637253	2617.024289	-0.770828032	0.005320562	0.015190498	PNPLA6
ENSG00000125630	303.5109812	507.0473123	-0.741027265	0.005328411	0.015211234	POLR1B
ENSG00000145860	1455.95578	2296.751963	-0.657591746	0.00533069	0.015216069	RNF145
ENSG00000054965	2773.666488	1556.378434	0.833801051	0.005335484	0.015228078	FAM168A
ENSG00000243199	281.9072622	559.3618586	-0.988786666	0.005337664	0.015232628	AC115223.1
ENSG00000113448	332.8924056	982.3880928	-1.561477923	0.005340717	0.015239668	PDE4D
ENSG00000126216	294.9774938	480.0455425	-0.702452595	0.005353876	0.015275539	TUBGCP3
ENSG00000205639	6.915890978	23.90797334	-1.787265357	0.005359137	0.015288869	MFSD2B
ENSG00000169297	0	6.23695197	-5.218500055	0.005360475	0.015291009	NR0B1
ENSG00000167862	322.5008618	531.22222	-0.720533405	0.005361798	0.015293102	MRPL58
ENSG00000161202	2694.684221	1953.970592	0.463716992	0.005365142	0.015300962	DVL3
ENSG00000287884	26.77248726	2.495773089	3.419312394	0.005369245	0.015310983	AC005594.1
ENSG00000023191	133.6802998	284.1448356	-1.087613819	0.005388756	0.015364934	RNH1
ENSG00000160214	482.4761213	848.3907019	-0.814576758	0.005390127	0.015366701	RRP1
ENSG00000140479	72.89821375	10.52603261	2.793599275	0.005390559	0.015366701	PCSK6
ENSG00000183955	655.9174936	1054.091534	-0.684769106	0.005404272	0.015404101	KMT5A
ENSG00000152784	97.12569958	338.2381161	-1.800563322	0.005405887	0.015407017	PRDM8
ENSG00000140750	967.1540697	1406.866831	-0.540855034	0.005418508	0.015441291	ARHGAP17
ENSG00000112877	48.08805062	103.8506091	-1.109823561	0.005423636	0.015454211	CEP72
ENSG00000285669	29.24943495	7.866239125	1.89298126	0.005432857	0.015478786	AC026979.4
ENSG00000235652	100.9358656	38.52684355	1.394274952	0.00544584	0.015514076	AL356599.1
ENSG00000112499	22.19159588	0.356539013	5.843404875	0.005448736	0.015520623	SLC22A2
ENSG00000075426	2049.827551	5240.206126	-1.354058709	0.005451491	0.015526769	FOSL2
ENSG00000157259	1086.937326	780.6723482	0.477518752	0.005453419	0.015530557	GATAD1
ENSG00000239569	92.36515259	46.96097231	0.978204109	0.005461133	0.01555082	KMT2E-AS1
ENSG00000131400	23.10488255	5.361009426	2.106898133	0.005463039	0.015554545	NAPSA
ENSG00000162961	490.6058041	704.6877174	-0.522396879	0.005463915	0.015555333	DPY30
ENSG00000226744	10.85778926	0.672939833	3.947015162	0.005469919	0.015570719	AC005326.1
ENSG00000214575	18.97590482	1.426156051	3.727690443	0.005474109	0.01558094	CPEB1

ENSG00000198270	158.862044	87.90857804	0.851873307	0.005477804	0.01558975	TMEM116
ENSG00000284966	22.18393179	4.881744087	2.165796236	0.005480309	0.015595172	AL138689.2
ENSG00000280071	128.7903687	253.2630725	-0.976485056	0.005483315	0.015602014	GATD3B
ENSG00000205659	311.865468	189.1705986	0.720168563	0.005492546	0.015625699	LIN52
ENSG00000213977	196.6808949	106.1058098	0.88930542	0.005492841	0.015625699	TAX1BP3
ENSG00000179542	26.67219466	112.2086866	-2.073369031	0.005494689	0.015629243	SLITRK4
ENSG00000260459	7.030543183	0	5.14623546	0.005497269	0.01563145	FTLP14
ENSG00000255422	7.030543183	0	5.14623546	0.005497269	0.01563145	AP002954.1
ENSG00000159387	7.030543183	0	5.14623546	0.005497269	0.01563145	IRX6
ENSG00000188000	13.90832615	0.356539013	5.171215454	0.005500266	0.01563826	OR7D2
ENSG00000103671	369.0617063	543.4104862	-0.558465736	0.005510354	0.015665227	TRIP4
ENSG00000086189	606.1933551	930.5882688	-0.618618743	0.005515753	0.01567886	DIMT1
ENSG00000163257	549.5793824	920.2935831	-0.743391454	0.005527563	0.015710711	DCAF16
ENSG00000170903	278.2656291	438.0344843	-0.654666741	0.005530215	0.015716529	MSANTD4
novel.758	12.6249697	0	5.991214701	0.005531541	0.015717872	-
ENSG00000271856	6.982619446	0	5.137168614	0.005531897	0.015717872	LINC01215
ENSG00000287766	15.30808665	1.793307549	3.093842984	0.005533983	0.01572208	AC006130.3
ENSG00000210100	2.695866906	17.43964883	-2.6920034	0.005536001	0.015726091	MT-TI
ENSG00000113272	222.2995082	365.9479551	-0.719693588	0.005548474	0.015759801	THG1L
ENSG00000155545	535.1909776	347.0044424	0.624736049	0.005550448	0.015763685	MIER3
ENSG00000162631	814.5773072	215.7029194	1.917765131	0.005553035	0.015769308	NTNG1
ENSG00000287074	13.58388628	2.496928964	2.439348274	0.005554862	0.015772772	AC137561.1
ENSG00000119185	2750.238153	1666.451324	0.722838944	0.005568125	0.015808703	ITGB1BP1
ENSG00000164978	295.6858492	531.8904898	-0.847184878	0.00557526	0.015827233	NUDT2
ENSG00000134186	1648.43063	1120.611213	0.55671044	0.005581869	0.015844263	PRPF38B
ENSG00000007001	6.404984563	0	5.011892112	0.005587347	0.015854931	UPP2
ENSG00000123427	117.6086816	194.9688901	-0.728518559	0.005587791	0.015854931	EEF1AKMT3
ENSG00000100263	359.3657398	561.360386	-0.643984459	0.005588029	0.015854931	RHBDD3
ENSG00000234318	6.403730914	0	5.011638109	0.005588068	0.015854931	AC099794.1
ENSG00000272505	8.030901441	0	5.340952817	0.00559051	0.015858962	AC104964.4
ENSG00000279716	54.70686853	24.50063679	1.154384551	0.00559071	0.015858962	AC006128.1
ENSG00000084112	2519.736791	5205.757442	-1.046762008	0.005595195	0.015869951	SSH1
ENSG00000287726	6.891929109	0	5.119913797	0.005597437	0.015872846	AC005740.5
ENSG00000223511	6.891929109	0	5.119913797	0.005597437	0.015872846	AL683807.1
ENSG00000110719	1684.022694	3383.302668	-1.006416697	0.005599308	0.015876417	TCIRG1
ENSG00000142765	77.64633932	20.37912538	1.932106065	0.005605265	0.015891575	SYTL1
ENSG00000287919	6.067652196	0	4.934553442	0.005623966	0.015941317	AC092802.4
ENSG00000104626	15.68539359	79.69739269	-2.341428297	0.005624232	0.015941317	ERI1
ENSG00000272145	43.14794537	18.39458348	1.233478644	0.005624651	0.015941317	NFYC-AS1
ENSG00000152348	105.4783918	171.0425171	-0.697912878	0.005632719	0.015962442	ATG10
ENSG00000259917	36.99622709	74.09915013	-1.00326796	0.005636524	0.015971483	HNRNPLP2
ENSG00000121940	332.0986161	504.9049489	-0.604462414	0.005642788	0.01598749	CLCC1
ENSG00000142920	337.5956842	190.3372222	0.827683117	0.005643518	0.015987813	AZIN2
ENSG00000144152	72.21202489	22.9976248	1.655298079	0.005645679	0.015992192	FBLN7
ENSG00000176422	135.2214668	223.0162606	-0.722014945	0.005652023	0.016008417	SPRYD4
ENSG00000214765	347.915501	196.7522743	0.821761788	0.005658504	0.016025025	SEPT7P2
ENSG00000122861	1691.807825	8476.154434	-2.324843305	0.005662706	0.01603518	PLAU
ENSG00000257511	42.84181203	12.84002796	1.737076813	0.00566942	0.01605244	AC084824.1
ENSG00000167971	40.75863908	7.031462255	2.544740264	0.005676572	0.01607094	CASKIN1
ENSG00000156831	235.0580496	367.4061582	-0.644504634	0.005677673	0.01607116	NSMCE2
ENSG00000171368	60.60915275	14.75362169	2.045687673	0.005677887	0.01607116	TPPP

ENSG00000215068	140.4211496	61.24645473	1.194645666	0.005684161	0.016087167	AC025171.2
ENSG00000274184	12.0244844	0.693008929	4.083736625	0.005693401	0.016111562	AC011815.2
ENSG00000259775	38.97939252	8.163726725	2.252275065	0.005696831	0.016119513	AL138976.2
ENSG00000124193	3633.893603	2280.636202	0.672079145	0.005710648	0.01615685	SRSF6
ENSG00000165678	2439.488626	3684.422804	-0.594917738	0.005717885	0.016175563	GHITM
ENSG00000233834	8.416086274	0.336469917	4.446303124	0.005719319	0.016177859	AC005083.1
ENSG00000177192	357.1606928	640.4127551	-0.842744829	0.005724899	0.016191879	PUS1
ENSG00000183250	77.43673763	157.477386	-1.023498952	0.005726128	0.016193553	LINC01547
ENSG00000167578	63.76019571	14.70402689	2.122851021	0.005726737	0.016193553	RAB4B
novel.533	133.4998446	252.8477391	-0.92144111	0.005729557	0.016199763	-
ENSG00000167702	672.4006556	299.3323058	1.16832242	0.00573077	0.01620143	KIFC2
ENSG00000088305	49.20173616	95.36983923	-0.955656931	0.005735812	0.01621392	DNMT3B
ENSG00000029725	1334.382138	787.6522416	0.760374916	0.00574227	0.016228832	RABEP1
ENSG00000256915	1.495180701	16.41461791	-3.444630615	0.005742337	0.016228832	AC090023.2
ENSG00000224078	1812.55101	1096.15571	0.725651592	0.005767149	0.016297184	SNHG14
ENSG00000152894	1.802282956	14.56611175	-3.01515314	0.005768551	0.016298643	PTPRK
ENSG00000257605	19.27512918	45.47358149	-1.236612691	0.00576892	0.016298643	AC073611.2
ENSG00000115944	972.8617912	1899.64296	-0.965460502	0.005775198	0.016314603	COX7A2L
ENSG00000168038	33.62318815	84.91624899	-1.334446998	0.005791035	0.016357566	ULK4
ENSG00000184635	41.77278748	99.12761958	-1.248902779	0.005793152	0.016361767	ZNF93
ENSG00000077264	49.14735855	4.534661685	3.429257169	0.005805354	0.016394445	PAK3
novel.441	3.638556971	34.1321176	-3.232828022	0.005806019	0.016394542	-
ENSG00000094916	1938.783252	2881.158389	-0.571610814	0.005815484	0.016419485	CBX5
ENSG00000224950	16.99474849	3.9336975	2.107832558	0.005822272	0.016436864	AL390066.1
ENSG00000275630	0.909952655	10.28404758	-3.502625723	0.005823091	0.016437388	AC004816.2
ENSG00000168453	722.0840251	213.7116179	1.757273778	0.005823966	0.016438072	HR
ENSG00000078124	728.6574789	423.798468	0.781519847	0.005829836	0.016452854	ACER3
ENSG00000171552	1240.952971	2431.173276	-0.97033176	0.005831384	0.016455434	BCL2L1
ENSG00000188153	662.2437705	1076.915609	-0.701187177	0.005833349	0.016459192	COL4A5
ENSG00000108590	54.4050656	104.8001495	-0.946921166	0.005838702	0.016470793	MED31
ENSG00000079308	4598.922727	2267.606027	1.020229862	0.005838728	0.016470793	TNS1
ENSG00000081479	6.315547875	0	4.99359986	0.005852365	0.016507468	LRP2
novel.339	14.75845932	0	6.216697393	0.005864322	0.016539399	-
ENSG00000141759	1360.809127	2052.314936	-0.592824023	0.005874734	0.016566968	TXNL4A
ENSG00000167792	2160.113688	3068.279035	-0.506308442	0.005878307	0.016575244	NDUFV1
ENSG00000287629	11.40394038	1.049547942	3.418927603	0.005882711	0.016585862	AC006059.5
ENSG00000143797	1974.659577	883.7406124	1.159704953	0.005884439	0.016588934	MBOAT2
ENSG00000286833	10.80735822	0.743759607	3.89761244	0.005889156	0.016600428	AC097532.3
ENSG00000066777	667.7129587	1050.119715	-0.653063677	0.005897656	0.016622586	ARFGEF1
ENSG00000048544	605.5238468	1013.732319	-0.743788804	0.005899345	0.016625543	MRPS10
ENSG00000188921	397.3249129	132.1017998	1.587360341	0.005903073	0.016634246	HACD4
ENSG00000083535	261.9936822	436.0494757	-0.73570161	0.00590374	0.016634319	PIBF1
ENSG00000198807	29.44754033	126.414933	-2.104020513	0.005912969	0.016658517	PAX9
ENSG00000103047	201.0091607	400.6172804	-0.995723166	0.005922542	0.016683678	TANGO6
ENSG00000115524	8801.414045	5380.18869	0.710135183	0.005927152	0.016694853	SF3B1
ENSG00000196715	857.2244113	1261.439668	-0.557590418	0.005928278	0.016696216	VKORC1L1
ENSG00000125871	362.7073659	605.5368307	-0.739923525	0.005946493	0.016745699	MGME1
ENSG00000278965	6.393630453	0	5.009595616	0.005955098	0.016768114	AC122713.2
ENSG00000196290	335.5276698	503.6186345	-0.585943737	0.00595697	0.016771568	NIF3L1
ENSG00000274008	13.37499625	1.396630344	3.236833379	0.00596502	0.016792414	RF00017
ENSG00000109063	99.12932354	35.70320525	1.470767903	0.005966225	0.016793985	MYH3

ENSG00000170017	3518.398354	7519.546844	-1.095750552	0.00596972	0.016802004	ALCAM
ENSG00000188227	214.3327288	92.10232188	1.216137618	0.005985056	0.016843343	ZNF793
ENSG00000112144	606.8794883	321.6457485	0.915082264	0.005988617	0.016851539	ICK
ENSG00000157766	30.68311524	8.744621814	1.797409579	0.005993335	0.016862988	ACAN
ENSG00000167535	345.1799446	648.666913	-0.909564852	0.0059974	0.0168726	CACNB3
ENSG00000010295	1296.685133	805.8510966	0.686613639	0.006003394	0.016887634	IFFO1
ENSG00000112759	1160.931013	470.7065194	1.302125021	0.006007016	0.016895993	SLC29A1
ENSG00000111537	6.082767252	0	4.93777583	0.006008473	0.016898261	IFNG
ENSG00000204316	114.0056262	53.76833528	1.084681964	0.006009826	0.016898829	MRPL38
ENSG00000111110	104.9943811	29.2076196	1.844237486	0.006009976	0.016898829	PPM1H
ENSG00000272578	11.43166319	31.72333339	-1.468186856	0.006011754	0.016901999	AP000347.1
ENSG00000182896	6.302940116	0	4.990992324	0.006012628	0.016902627	TMEM95
ENSG00000261286	2.446717578	13.91195675	-2.51600212	0.006017762	0.016915231	ATP2C2-AS1
ENSG00000060069	293.4578096	434.7126206	-0.566382976	0.006019077	0.016917097	CTDP1
ENSG00000115904	1103.032409	696.1358578	0.664214827	0.006023441	0.016927531	SOS1
ENSG00000174788	8.830247571	0.336469917	4.513557513	0.006028861	0.016940929	PCP2
ENSG00000204923	102.6338816	53.91840396	0.926866278	0.006034575	0.016955153	FBXO48
ENSG00000232706	7.206624528	0	5.183969236	0.00604044	0.016969796	NUTM2HP
ENSG00000151148	808.2207961	1168.144429	-0.53122157	0.006041266	0.016970282	UBE3B
ENSG00000169397	6.983944366	0	5.13744102	0.006044766	0.016978277	RNASE3
ENSG00000236526	13.06384832	0.693008929	4.206103284	0.006053432	0.01700078	AL035448.1
ENSG00000162494	81.95225224	5.127325134	3.989011287	0.006062147	0.017023416	LRRC38
ENSG00000260593	7.354014143	0	5.211003876	0.006063131	0.017024338	AC009097.2
ENSG00000166133	161.049583	265.883415	-0.723635635	0.006068129	0.017036532	RPUSD2
ENSG00000130717	878.4721503	592.671421	0.567609329	0.006072571	0.017047161	UCK1
ENSG00000204745	32.58973648	70.19973023	-1.107387016	0.006086716	0.017085023	AC083899.1
ENSG00000170836	378.2427204	222.5612211	0.763729461	0.006089744	0.017091677	PPM1D
novel.577	8.237497632	0.387220594	4.412843163	0.006091603	0.017095047	-
ENSG00000259702	8.589660321	0	5.438097505	0.006095696	0.017104686	AC024337.1
ENSG00000115827	489.5277627	302.6943205	0.692575553	0.006098118	0.017109632	DCAF17
ENSG00000187653	1135.980401	652.3125105	0.800022147	0.00610584	0.01712945	TMSB4XP8
ENSG00000258461	35.95496879	9.04912588	1.99487994	0.006109179	0.017136967	AC012651.1
ENSG00000184313	15.72572416	0.672939833	4.493896258	0.006111672	0.017142109	MROH7
ENSG00000162757	38.03455081	90.82875619	-1.255467832	0.006115556	0.01715115	C1orf74
ENSG00000075711	1462.519083	996.1137736	0.554032785	0.006118938	0.017158783	DLG1
ENSG00000126261	1431.028057	2184.0566	-0.61005701	0.006124237	0.017171789	UBA2
ENSG00000184922	975.3681446	421.2305787	1.211898184	0.006127627	0.017179441	FMNL1
ENSG00000163710	18.44848716	120.2917518	-2.705877686	0.006130251	0.017184942	PCOLCE2
ENSG00000287672	2.418994764	12.81995886	-2.40739106	0.006141243	0.017213173	AC003086.1
ENSG00000211451	48.64806315	21.52315821	1.176684896	0.006141646	0.017213173	GNRHR2
ENSG00000162385	454.395133	676.9946682	-0.575601737	0.006143544	0.017213306	MAGOH
ENSG00000237886	53.07823054	16.40760144	1.69871912	0.006143613	0.017213306	NALT1
ENSG00000123130	939.2656176	1470.783774	-0.646829301	0.006143681	0.017213306	ACOT9
ENSG00000170265	543.2592038	817.1006226	-0.588933989	0.006145034	0.017215238	ZNF282
ENSG00000178149	357.8308414	521.1086489	-0.542066289	0.006147487	0.017220255	DALRD3
ENSG00000164483	30.62293975	5.941904515	2.35302744	0.006152827	0.017233354	SAMD3
ENSG00000169490	750.3234357	1255.848887	-0.743352887	0.006175391	0.017294689	TM2D2
ENSG00000251257	2.989107754	15.53050302	-2.367369801	0.006187232	0.017325984	AC010457.1
ENSG00000273765	7.154939844	0	5.174383888	0.006194643	0.017344868	AC024257.4
ENSG00000235552	802.881557	1353.913089	-0.753904764	0.006195926	0.017346589	RPL6P27
ENSG00000082512	416.7069467	237.9383163	0.807373044	0.006200216	0.017356731	TRAF5

novel.521	164.4037178	55.05383208	1.580319741	0.00620095	0.017356915	-
ENSG00000097096	116.2765475	36.65022435	1.661514636	0.00620342	0.017361959	SYDE2
ENSG00000197343	1432.533527	953.9516536	0.586785373	0.006211096	0.017381567	ZNF655
ENSG00000257277	6.582319557	0	5.053858923	0.006213899	0.01738754	AC092652.2
ENSG00000113504	170.1357026	43.22377463	1.973759885	0.006215757	0.017390867	SLC12A7
ENSG00000250863	7.458281153	0	5.234090221	0.006225624	0.017416597	AC114757.1
ENSG00000055208	2893.096167	1675.409043	0.788264568	0.006226516	0.017417216	TAB2
ENSG00000089682	295.9009088	173.6325996	0.769653412	0.006236393	0.017442966	RBM41
ENSG00000173253	11.96563383	0	5.913963516	0.006242604	0.017458459	DMRT2
ENSG00000070882	631.9236015	332.4530527	0.926289211	0.006244144	0.017460887	OSBPL3
ENSG00000185608	479.8320813	694.8577758	-0.534347154	0.006249022	0.017472647	MRPL40
ENSG00000101928	167.9359556	278.4884272	-0.730460342	0.006254635	0.017486459	MOSPD1
ENSG00000164379	21.77256253	204.0028959	-3.228395865	0.006263919	0.01751053	FOXQ1
ENSG00000234418	6.370993505	0	5.004978904	0.00626817	0.017518652	AC015983.2
ENSG00000227403	3.620934617	17.62831464	-2.276466223	0.006268292	0.017518652	LINC01806
ENSG00000279526	6.729709172	0	5.083013515	0.006268847	0.017518652	AC011239.2
ENSG00000157657	490.7270096	799.8708386	-0.704586964	0.006273258	0.017529095	ZNF618
ENSG00000198830	3421.919394	6768.599134	-0.984107588	0.006274893	0.017531778	HMGN2
ENSG00000213465	1520.53349	947.242689	0.682934613	0.006276319	0.017533874	ARL2
ENSG00000120075	23.69694831	132.3898652	-2.483313891	0.006281711	0.017547052	HOXB5
ENSG00000168939	36.73787494	102.9767555	-1.484607927	0.006287639	0.017559877	SPRY3
ENSG00000100079	31.42959431	9.345457604	1.749370599	0.006287654	0.017559877	LGALS2
ENSG00000116668	180.9459404	82.77380408	1.130312553	0.00628982	0.017564038	SWT1
ENSG00000272037	26.16963686	7.530925084	1.797734802	0.006292624	0.01756998	AP002907.1
ENSG00000138073	955.1210236	1393.088937	-0.544471603	0.006295062	0.0175749	PREB
ENSG00000204335	15.7161219	0	6.30738984	0.006295988	0.017575596	SP5
ENSG00000269743	70.90631657	34.73979428	1.028216863	0.006297568	0.017578117	SLC25A53
ENSG00000057657	249.4910285	985.8042475	-1.982201718	0.006298682	0.017579339	PRDM1
novel.1000	0.308355904	16.1818327	-5.632423432	0.006304521	0.017593745	-
novel.912	0	11.84135909	-6.148472713	0.006305206	0.017593766	-
ENSG00000254109	44.41514658	14.66517296	1.598537448	0.006308294	0.017600492	RBPMS-AS1
ENSG00000254837	84.44625352	42.35215162	0.99497798	0.00631355	0.017613265	AP001372.2
ENSG00000272872	7.644676422	0	5.267222365	0.006317641	0.017622786	AP000525.1
ENSG00000266554	6.304193765	0	4.991226013	0.006319331	0.017625607	LINC01443
ENSG00000132688	8845.006356	1551.866634	2.510773434	0.006324662	0.017636061	NES
ENSG00000151806	345.1031424	610.1589948	-0.822557405	0.006325004	0.017636061	GUF1
ENSG00000047188	837.5083116	544.0707586	0.622170931	0.006325115	0.017636061	YTHDC2
ENSG00000058668	1500.605548	3377.325697	-1.170247519	0.006327176	0.017639913	ATP2B4
ENSG00000115993	2062.763127	1234.200198	0.740802294	0.006333709	0.017656234	TRAK2
ENSG00000271784	8.202252925	0.336469917	4.407396672	0.006334788	0.017657347	AL031055.1
novel.410	49.21420138	12.60608688	1.975172345	0.006343431	0.017679541	-
ENSG00000166123	1268.71173	717.6037365	0.82222609	0.006345877	0.01768446	GPT2
ENSG00000162069	0	7.927473894	-5.563219271	0.006347713	0.017687679	BICDL2
ENSG00000171863	6310.341709	10165.40854	-0.687898425	0.006359007	0.017717249	RPS7
ENSG00000257322	9.915099193	0.387220594	4.682428489	0.006359762	0.017717453	AC138123.1
ENSG00000079335	132.9189473	59.59684169	1.156770223	0.006361835	0.017721328	CDC14A
ENSG00000179088	9.202824645	0	5.53489482	0.00636411	0.017725766	C12orf42
ENSG00000255622	6.598688262	0	5.057100995	0.00638357	0.017778062	PCDHB17P
ENSG00000270207	6.593673666	0	5.056106801	0.006387978	0.017786525	AC077690.1
ENSG00000255750	6.593673666	0	5.056106801	0.006387978	0.017786525	AC022509.1
ENSG00000119596	1037.324051	1482.780582	-0.515415234	0.006395941	0.017806787	YLPM1

ENSG00000272323	19.79892809	3.873490212	2.341340478	0.006402895	0.017824238	AC026801.2
ENSG00000233237	827.5997973	363.6411855	1.186205729	0.006403745	0.017824695	LINC00472
ENSG00000245975	17.3005971	3.302051734	2.404346479	0.006406949	0.017831703	AC090515.2
ENSG00000279569	41.51393713	6.938261635	2.586803522	0.0064142	0.017849972	AC020763.4
ENSG00000075624	58238.03705	99741.50426	-0.776235655	0.006428342	0.017887411	ACTB
ENSG00000132153	1593.867888	2435.764523	-0.611941979	0.006435054	0.017904169	DHX30
ENSG00000132661	232.8923924	369.5831847	-0.666763964	0.006445521	0.017931372	NXT1
ENSG00000258702	11.89367696	0.774441189	4.010881438	0.006446934	0.017933384	AL137786.1
ENSG00000237506	259.8517602	998.7734856	-1.942370327	0.006456846	0.01795903	RPSAP15
ENSG00000114391	6268.303736	9553.442563	-0.607972881	0.006465334	0.017980716	RPL24
ENSG00000077549	3463.886073	5438.790139	-0.650855954	0.006471242	0.017995218	CAPZB
ENSG00000267372	13.43044188	1.793307549	2.902433746	0.00647545	0.018004994	AC005330.1
ENSG00000227388	16.56289357	2.455634897	2.738189143	0.006478571	0.018011745	AL133410.1
ENSG00000140471	290.7298182	173.3046531	0.745575601	0.006479486	0.018011857	LINS1
ENSG00000135387	4025.410422	5952.682897	-0.564455083	0.006479998	0.018011857	CAPRIN1
ENSG00000196810	382.2086515	248.1292448	0.623738174	0.006484042	0.018020873	CTBP1-DT
ENSG00000108666	237.254578	380.3648973	-0.680973312	0.006484629	0.018020873	C17orf75
novel.875	77.29332278	26.49005897	1.537998714	0.006488005	0.018028327	-
novel.829	23.39582957	2.0188195	3.500202114	0.006490922	0.018034503	-
ENSG00000133328	10.8426742	0.713078025	3.919826508	0.006497376	0.018050506	PLAAT2
ENSG00000285972	8.364472862	0	5.399108762	0.006517174	0.01810357	CERNA2
ENSG00000003249	177.1682274	56.18558298	1.660508681	0.006526997	0.01812855	DBNDD1
ENSG00000197753	15.70587925	42.50311939	-1.432970063	0.006527562	0.01812855	LHFPL5
ENSG00000269553	11.28706561	0.774441189	3.93700973	0.006540979	0.018163871	U62631.1
ENSG00000262089	41.95735963	15.03348034	1.480321514	0.006541778	0.018164147	AC040977.1
ENSG00000266588	10.13047093	0.356539013	4.711118185	0.006565505	0.018228081	AC006441.4
ENSG00000016864	1367.094958	2019.453734	-0.562914147	0.006570704	0.018239053	GLT8D1
ENSG00000010310	7.340152735	59.35275484	-3.015707927	0.006570861	0.018239053	GIPR
ENSG00000115365	1007.799523	1494.569508	-0.568468869	0.006572727	0.018242283	LANCL1
ENSG00000204120	1069.797296	1541.56198	-0.526946725	0.006585082	0.018274622	GIGYF2
ENSG00000139517	283.2140117	151.8005365	0.898660085	0.006592614	0.018293572	LNX2
ENSG00000108468	985.5191003	1886.967633	-0.93725157	0.006600477	0.018313433	CBX1
novel.11	14.31281426	1.080229524	3.729937301	0.006602438	0.018316918	-
ENSG00000058453	1189.64371	678.4428883	0.810514306	0.0066034	0.018317632	CROCC
ENSG00000158406	43.78765013	18.0168195	1.282140792	0.006610338	0.018334921	HIST1H4H
ENSG00000157131	0	11.93083529	-6.156005853	0.006612966	0.018339009	C8A
novel.641	31.55023002	8.864907996	1.844531539	0.006613224	0.018339009	-
ENSG00000249242	111.9873861	37.85878401	1.564877879	0.006620343	0.018356792	TMEM150C
ENSG00000173267	252.0187198	82.96449049	1.601223467	0.006632565	0.018388716	SNCG
ENSG00000090581	1868.747444	1222.737246	0.612147274	0.006640089	0.018407613	GNPTG
ENSG00000120533	944.9044496	1425.933807	-0.593630008	0.006643027	0.018413794	ENY2
ENSG00000168497	645.6666374	91.48544538	2.818098735	0.006658008	0.018453351	CAVIN2
ENSG00000177483	22.63179941	5.186248153	2.143891931	0.006661713	0.01846165	RBM44
ENSG00000162542	651.2434644	318.1606819	1.032761809	0.006667485	0.018475674	TMCO4
ENSG00000286067	49.63678256	140.1879343	-1.496237917	0.006672925	0.018488777	AC004263.2
ENSG00000236268	9.537649715	0	5.586333708	0.006676551	0.018496851	LINC01361
ENSG00000137161	1860.074736	1124.989946	0.725469747	0.006679774	0.018503495	CNPY3
ENSG00000112237	490.5815853	821.1375377	-0.743570271	0.006680374	0.018503495	CCNC
ENSG00000228570	14.08719953	2.180528143	2.70289702	0.006682523	0.018507475	NUTM2E
ENSG00000272908	26.29836393	3.048298346	3.081185512	0.006683957	0.018509475	AC006033.2
ENSG00000125826	1727.801953	2470.011216	-0.515609863	0.006692009	0.018529797	RBCK1

ENSG00000148082	388.3751726	1072.709546	-1.465464188	0.006714989	0.018590061	SHC3
ENSG00000257093	30.38869201	8.847279044	1.772885501	0.006715709	0.018590061	KIAA1147
ENSG00000140521	1048.629752	1645.646145	-0.649972008	0.00671592	0.018590061	POLG
ENSG00000183762	225.5241883	602.2959284	-1.417009985	0.006722517	0.018606341	KREMEN1
ENSG00000285106	20.35238764	63.63905701	-1.6413699	0.006729562	0.018623855	AC016831.7
ENSG00000250423	14.47893734	0	6.189053616	0.006740112	0.018649496	KIAA1210
ENSG00000015475	460.1888751	918.4493159	-0.99688706	0.006740263	0.018649496	BID
ENSG00000267213	4.598940661	28.77452862	-2.653131525	0.006744415	0.018658997	AC007773.1
ENSG00000108510	1262.453979	829.4192129	0.606014981	0.006776171	0.018744858	MED13
ENSG00000244357	6.036168435	0	4.927832434	0.006777497	0.018746529	RN7SL145P
ENSG00000116754	6281.659164	3465.458292	0.85813596	0.00678033	0.01875237	SRSF11
ENSG00000124574	526.0825764	798.3873606	-0.601687958	0.006793423	0.018786447	ABCC10
ENSG00000250326	22.08439464	6.24640858	1.836920921	0.006794098	0.018786447	AC104596.1
ENSG00000167641	1415.438682	64.19959185	4.461885139	0.006799794	0.018800198	PPP1R14A
ENSG00000225465	41.89836652	6.809674718	2.604588414	0.006803997	0.018809149	RFPL1S
ENSG00000235033	43.69388303	11.3843462	1.93854779	0.00680448	0.018809149	AL590999.1
ENSG00000238178	9.33613939	0.693008929	3.715391583	0.006828538	0.018873644	AC078993.1
ENSG00000166801	1354.960158	810.5625867	0.741116843	0.006832871	0.018883612	FAM111A
ENSG00000105866	174.5030179	90.85481427	0.943886368	0.006838554	0.018897305	SP4
ENSG00000197375	219.2410659	124.359734	0.819182884	0.006847122	0.018918971	SLC22A5
ENSG00000130224	213.2176478	112.5753714	0.922044999	0.006848398	0.018920484	LRCH2
ENSG00000273058	26.98130601	7.072884716	1.925432308	0.006853594	0.018932827	AL359921.2
ENSG00000227355	7.59306301	0.336469917	4.296009743	0.006875637	0.0189917	AL359644.1
ENSG00000123453	178.5476444	92.17057312	0.951680406	0.006877816	0.018994425	SARDH
ENSG00000138246	1217.375794	1776.282981	-0.54517561	0.006878086	0.018994425	DNAJC13
ENSG00000108528	1028.498758	1649.068825	-0.681305089	0.00688365	0.019007771	SLC25A11
ENSG00000103249	841.8245659	1305.808897	-0.633158501	0.006889474	0.01902183	CLCN7
ENSG00000221866	43.31678921	6.612404203	2.717973733	0.006895174	0.019035546	PLXNA4
ENSG00000146278	2433.964695	1272.010506	0.936459228	0.006905376	0.019061685	PNRC1
ENSG00000242267	10.85528196	0.672939833	3.949045363	0.006917719	0.019093728	SKINT1L
ENSG00000169057	1568.9288	948.7002707	0.725950882	0.006926149	0.019114965	MECP2
ENSG00000233622	49.84144092	22.18548556	1.165449352	0.006934385	0.0191347	CYP2T1P
ENSG00000228307	12.89689852	0.743759607	4.145875885	0.006934773	0.0191347	OR2S1P
ENSG00000197168	31.95790961	11.09862696	1.52549132	0.006937393	0.019139897	NEK5
ENSG00000162746	145.0207592	44.76435628	1.692440376	0.006939671	0.019144149	FCRLB
ENSG00000198160	920.6761082	641.1819992	0.52204721	0.006941988	0.019148508	MIER1
ENSG00000248458	0.616711807	12.83758781	-4.392516904	0.006946291	0.019158342	AL139147.1
ENSG00000163344	801.8842053	409.9120346	0.967337183	0.006947041	0.019158378	PMVK
ENSG00000101417	297.7603892	193.6221586	0.620559123	0.00695284	0.019172334	PXMP4
ENSG00000272839	6.08527455	0	4.938348403	0.006954519	0.019174931	AC011899.3
novel.957	411.1875205	3.005720009	7.100392914	0.006961126	0.019191108	-
ENSG00000233393	14.34895661	1.069617038	3.740737517	0.006963809	0.019196468	AP000688.2
ENSG00000150540	397.1059227	162.4931581	1.288296191	0.006964574	0.019196541	HNMT
ENSG00000090971	581.5508969	1011.871588	-0.798710231	0.006966138	0.019198151	NAT14
ENSG00000274964	66.45140403	20.31190163	1.709072294	0.006967352	0.019198151	AC026356.1
ENSG00000258777	0	13.22267223	-6.308382657	0.006967375	0.019198151	HIF1A-AS1
ENSG00000115607	15.54233439	1.345879667	3.482218783	0.006969888	0.019203039	IL18RAP
ENSG00000104848	0.293240848	7.64188305	-4.550914917	0.006975089	0.019215331	KCNA7
ENSG00000179148	0	7.143576095	-5.412613507	0.006978494	0.019222673	ALOXE3
ENSG00000150527	283.0522896	450.0277918	-0.668417983	0.006979811	0.019224262	MIA2
ENSG00000071189	1027.148421	669.7704174	0.616955551	0.00698705	0.01924216	SNX13

ENSG00000168505	11.39154608	0	5.842836125	0.006995408	0.019263137	GBX2
ENSG00000224114	15.3574775	1.049547942	3.849928169	0.006998506	0.019269624	AL591846.1
ENSG00000242516	49.67377911	185.1085366	-1.89874973	0.00700038	0.019272742	LINC00960
ENSG00000228784	26.26659544	7.672564631	1.785630926	0.007002988	0.019277879	LINC00954
ENSG00000167619	14.54426997	2.149846562	2.756233686	0.007005818	0.019283628	TMEM145
ENSG00000270124	1.49643435	20.74383674	-3.785206028	0.007007615	0.019286012	AC092127.2
ENSG00000246100	91.21099394	15.08996321	2.602872621	0.007008169	0.019286012	LINC00900
ENSG00000185915	11.37768468	0	5.841134639	0.007010007	0.019289028	KLHL34
ENSG00000267857	0.293240848	8.568704665	-4.719434226	0.007012053	0.019292614	AL133499.1
ENSG00000272361	8.216114332	0.336469917	4.409593182	0.007013251	0.019293865	AC005014.2
ENSG00000230965	12.16274247	0.387220594	4.977800245	0.007014046	0.01929401	SNX18P13
ENSG00000130950	6.314294226	0	4.993275207	0.007016281	0.019296155	NUTM2F
ENSG00000107614	336.7776922	151.7680099	1.150681482	0.007016311	0.019296155	TRDMT1
ENSG00000266213	6.009699269	0	4.922128378	0.007020734	0.019306276	AC107892.1
ENSG00000130340	991.7734355	1888.22319	-0.928791495	0.007025872	0.019318359	SNX9
ENSG00000286412	15.62493336	0	6.300247145	0.007036693	0.019346066	AC007682.1
ENSG00000159658	2350.950079	1558.55441	0.5930356	0.00704335	0.019360419	EFCAB14
ENSG00000100218	8.25010539	0.387220594	4.41490346	0.007043404	0.019360419	RSPH14
ENSG00000169122	248.3908331	83.12461089	1.579202719	0.007047681	0.019370126	FAM110B
ENSG00000258457	26.8556557	7.418811243	1.847876357	0.007054247	0.019386123	AL132780.2
ENSG00000125878	15.32466882	1.487519214	3.388794357	0.007059555	0.019398656	TCF15
ENSG00000066468	25.55342325	0.672939833	5.204063439	0.007061979	0.019403266	FGFR2
ENSG00000072134	290.8766224	537.1534524	-0.884942546	0.007063497	0.019405385	EPN2
ENSG00000198668	9355.982119	6322.114187	0.565435365	0.007079077	0.019446129	CALM1
ENSG00000286721	6.289078709	0	4.988036675	0.007095276	0.019488568	AC010623.1
ENSG00000253177	6.28782506	0	4.987777354	0.00709654	0.019489978	AC104211.2
ENSG00000104067	165.0588502	69.60146298	1.242622489	0.00710514	0.019511536	TJP1
ENSG00000150593	1511.014527	964.9145019	0.647261728	0.007110311	0.019523673	PDCD4
ENSG00000106400	1480.832133	2223.971235	-0.58658731	0.007120864	0.019550583	ZNHIT1
ENSG00000178202	913.8580158	1692.496757	-0.88931808	0.00712358	0.019554272	POGLUT3
ENSG00000144524	565.7964528	888.2888756	-0.650699845	0.007123713	0.019554272	COPS7B
ENSG00000269001	162.6591309	89.40170106	0.864602822	0.007128442	0.019565186	AC092070.2
ENSG00000200087	5.642136789	33.26545609	-2.555510038	0.007155172	0.019636477	SNORA73B
ENSG00000065609	20.62968671	1.41669944	3.855382857	0.007163961	0.019658521	SNAP91
ENSG00000177380	88.59091577	245.1127711	-1.467840476	0.007170087	0.019673252	PPFIA3
ENSG00000259124	5.745434885	0	4.856343222	0.007170857	0.019673287	AC008050.1
ENSG00000184232	7435.700191	2860.468051	1.378280533	0.007175445	0.019683797	OAF
ENSG00000162813	463.5224536	733.2252353	-0.662081715	0.007181144	0.019697351	BPNT1
ENSG00000164073	416.0104999	269.7322126	0.624230193	0.00718358	0.019699544	MFSD8
ENSG00000125995	798.4473012	1267.089912	-0.66627765	0.007183941	0.019699544	ROMO1
ENSG00000146776	182.5229647	98.01632329	0.898253769	0.0071845	0.019699544	ATXN7L1
ENSG00000198106	7.550225139	0.356539013	4.288935648	0.007184976	0.019699544	AC025279.1
ENSG00000128283	2288.24384	1468.150497	0.640106586	0.007185964	0.019700172	CDC42EP1
ENSG00000119685	357.2450714	592.8777029	-0.730601389	0.007216954	0.019783042	TTLL5
ENSG00000237938	7.796867169	26.53580097	-1.759639542	0.00721838	0.019784864	AL450998.3
ENSG00000241494	131.8977474	240.1155395	-0.864285401	0.00722907	0.019812075	AL355032.1
ENSG00000281091	5.768143105	26.12277908	-2.182617309	0.007237491	0.019833063	AL117327.1
ENSG00000156603	254.3711724	439.0782661	-0.788203534	0.007242531	0.01984478	MED19
ENSG00000198089	325.1202283	560.7355811	-0.786147355	0.007268903	0.01991494	SFI1
ENSG00000224023	8.38710981	0.336469917	4.441724231	0.00727142	0.019919734	EDRF1-DT
ENSG00000100711	898.7911164	573.0052449	0.648933769	0.007282815	0.019945073	ZFYVE21

ENSG00000230061	6.094121362	0	4.940262464	0.007282973	0.019945073	TRPM2-AS
ENSG00000180044	6.094121362	0	4.940262464	0.007282973	0.019945073	C3orf80
ENSG00000136003	2354.896433	1453.881252	0.695707328	0.007286609	0.019952929	ISCU
ENSG00000213519	6.355878449	0	5.001873348	0.00729027	0.019960849	AC132008.1
ENSG00000261840	36.73823095	15.14328243	1.280409979	0.007310365	0.020013761	AC093249.6
ENSG00000117899	990.7297245	1684.89548	-0.766281534	0.007317496	0.020031173	MESD
novel.372	184.110987	70.75986707	1.382791109	0.007327138	0.020055455	-
ENSG00000264577	21.52660468	65.73473244	-1.608186618	0.007329724	0.020060419	AC010761.2
ENSG00000146535	1481.64514	2242.793549	-0.598033797	0.00733298	0.020067216	GNA12
ENSG00000182870	2.125753916	22.72226126	-3.4157073	0.00734178	0.020089181	GALNT9
ENSG00000249628	1.832513069	14.94947954	-3.027659046	0.007345059	0.020096039	LINC00942
ENSG00000231521	6.300432819	0	4.990379526	0.007350175	0.020107919	AL162385.2
ENSG00000257803	13.00088079	0.356539013	5.073990015	0.007351491	0.020109401	AC010200.1
ENSG00000013293	26.10047201	116.8029261	-2.163143133	0.007359309	0.020128667	SLC7A14
ENSG00000168301	247.1435362	135.7049695	0.86607621	0.007365465	0.020143384	KCTD6
ENSG00000142676	10514.78691	16274.24836	-0.630174493	0.007374718	0.020166567	RPL11
ENSG00000225975	42.50261311	13.12459132	1.69023578	0.00737728	0.020171335	LINC01534
ENSG00000170476	16.64620421	0	6.390392834	0.007378015	0.020171335	MZB1
ENSG00000062582	5.749195831	22.08154406	-1.939355474	0.007403827	0.020239775	MRPS24
ENSG00000136147	1062.598011	603.6710555	0.816251896	0.007421756	0.020286653	PHF11
ENSG00000254987	10.22972334	0.672939833	3.862678572	0.007439264	0.02033237	AP002989.1
ENSG00000264017	7.880035613	0.336469917	4.34997037	0.007443764	0.020342529	RN7SL336P
ENSG00000237765	635.882736	428.1584971	0.570573099	0.007453218	0.020366223	FAM200B
ENSG00000144161	216.6188365	114.6725879	0.915462203	0.007461978	0.020388016	ZC3H8
novel.461	3.955759687	17.3167941	-2.13683887	0.007468254	0.020403018	-
ENSG00000164008	92.19957116	35.74804815	1.366567326	0.00747204	0.020411217	C1orf50
ENSG00000258565	0	5.767143241	-5.108488899	0.007473982	0.020412358	BLZF2P
ENSG00000120555	9.334885741	0.743759607	3.687479029	0.00747403	0.020412358	SEPT7P9
ENSG00000140839	31.25706045	9.853092774	1.673140003	0.007489174	0.020451569	CLEC18B
ENSG00000101544	317.603923	558.8641677	-0.81468217	0.007500729	0.020479383	ADNP2
ENSG00000225507	55.21018178	24.52057749	1.173727496	0.007500936	0.020479383	AC069282.1
ENSG00000148444	336.6913873	207.1962327	0.700577351	0.007507646	0.020495551	COMMD3
ENSG00000271980	7.988348304	0	5.330401043	0.007524312	0.020538889	AC012640.4
ENSG00000277977	13.96273159	2.180528143	2.687635181	0.007533926	0.020562971	AC010332.1
ENSG00000154227	7.151178898	0	5.173558761	0.007557354	0.02062475	CERS3
ENSG00000196205	6837.533327	11000.72654	-0.686058875	0.007576793	0.020675628	EEF1A1P5
ENSG00000133597	493.5415741	267.7303458	0.881334056	0.007598323	0.020732203	ADCK2
ENSG00000175513	9.512505469	0.336469917	4.620263514	0.007599226	0.02073249	TSGA10IP
ENSG00000133265	715.944938	1121.233942	-0.647330386	0.007604438	0.020744531	HSPBP1
ENSG00000154188	126.0259773	789.782502	-2.648064558	0.007610268	0.020756776	ANGPT1
ENSG00000238246	5.775664997	0	4.863109149	0.007611324	0.020756776	AC069549.1
ENSG00000125462	5.775664997	0	4.863109149	0.007611324	0.020756776	C1orf61
ENSG00000173452	0	11.04800468	-6.04893166	0.00761599	0.020767321	TMEM196
ENSG00000136193	7432.657101	4898.495121	0.601600674	0.007622416	0.020782661	SCRN1
ENSG00000166848	2003.306094	1344.798781	0.574885234	0.007631861	0.020806231	TERF2IP
ENSG00000276115	38.67884324	12.25096053	1.648508683	0.007642345	0.020832626	AC026356.2
ENSG00000231875	5.730319829	0	4.85294547	0.007650545	0.02085279	AL359885.1
ENSG00000100330	237.2727423	107.638767	1.142660686	0.007656411	0.020866592	MTMR3
ENSG00000185070	1895.241423	401.9536438	2.237303095	0.007658159	0.020869165	FLRT2
ENSG00000271553	20.16996678	4.545274171	2.134443909	0.00766027	0.020872731	AC018638.7
ENSG00000101441	7.639733098	0	5.266423586	0.00766492	0.020883208	CST4

ENSG00000125691	9182.495924	14553.34505	-0.664402688	0.007670115	0.020895172	RPL23
ENSG00000260231	81.95991738	26.53836951	1.623207812	0.007680415	0.020919219	KDM7A-DT
ENSG00000231760	17.78628799	3.587770973	2.310554985	0.007680552	0.020919219	AL355312.2
ENSG00000130935	540.910841	962.1996764	-0.831249369	0.007686909	0.020934338	NOL11
ENSG00000103257	639.2439074	2202.47905	-1.784802518	0.007696889	0.020959321	SLC7A5
ENSG00000247626	78.70708688	145.3378158	-0.886292207	0.007701271	0.020969055	MARS2
ENSG00000105131	18.21047848	3.12857473	2.52035754	0.007722389	0.021024353	EPHX3
ENSG00000261025	12.40207607	1.161661783	3.479597388	0.007727479	0.021036005	AL445471.2
ENSG00000128710	0	11.02793558	-6.046392943	0.007738725	0.021064412	HOXD10
ENSG00000225269	0	5.390535133	-5.011455058	0.007744295	0.021076042	LINC00705
ENSG00000205181	122.4017528	53.37307074	1.194021825	0.00774462	0.021076042	LINC00654
ENSG00000159267	307.4684649	455.6596353	-0.567926118	0.007762751	0.02112317	HLCS
ENSG00000229127	40.50723936	13.45147623	1.596892728	0.007769625	0.021139661	AC007038.1
ENSG00000181038	310.3891198	502.9529211	-0.696003771	0.007771074	0.021141389	METTL23
ENSG00000180228	331.7464531	491.3977605	-0.567054581	0.007773101	0.02114469	PRKRA
ENSG00000172175	290.0297801	521.4112607	-0.846105302	0.007774293	0.021145718	MALT1
ENSG00000012983	1863.222472	1036.174229	0.846489899	0.007782535	0.021165921	MAP4K5
ENSG00000274893	6.568458149	0	5.05094436	0.007785617	0.021172086	AL136317.2
ENSG00000116237	1818.801933	3037.887294	-0.740195472	0.007806083	0.021225521	ICMT
ENSG00000147687	224.731182	360.9117756	-0.684118902	0.007814731	0.021246812	TATDN1
ENSG00000232434	83.32608592	179.7452841	-1.10898976	0.007820225	0.021259523	AJM1
ENSG00000160818	381.3199681	802.2321548	-1.073420532	0.007837035	0.02130149	GPATCH4
ENSG00000196935	417.5637638	904.4892447	-1.114742399	0.007837302	0.02130149	SRGAP1
novel.482	153.1875254	346.8906803	-1.178721548	0.007868262	0.021382263	-
ENSG00000114315	545.6951899	131.1921404	2.055277616	0.007868666	0.021382263	HES1
ENSG00000066185	19.69688364	6.094156548	1.691357661	0.007872072	0.021389279	ZMYND12
ENSG00000133665	12.92963593	0	6.025729635	0.007873431	0.021390736	DYDC2
ENSG00000183077	258.1278574	483.9333231	-0.906523919	0.007881498	0.021410414	AFMID
ENSG00000281490	451.9815408	188.2836924	1.263667935	0.007884238	0.021415618	CICP14
ENSG00000284675	6.74231693	0	5.085619179	0.007889578	0.021427883	AC116667.2
ENSG00000145331	73.68897033	130.7884339	-0.828919163	0.007898894	0.021450943	TRMT10A
ENSG00000092067	10.3431219	0.713078025	3.850286825	0.007904687	0.021463034	CEBPE
ENSG00000120256	32.7032063	83.16590496	-1.346273466	0.007904999	0.021463034	LRP11
ENSG00000125414	10.65899969	0.774441189	3.854442711	0.007905903	0.021463247	MYH2
ENSG00000164897	615.7911796	898.9391287	-0.545859121	0.007925052	0.021512985	TMUB1
ENSG00000223813	2.096777453	28.80533859	-3.778124427	0.007934393	0.021535132	AC007255.1
ENSG00000177105	1444.903875	992.7213604	0.541590388	0.007934869	0.021535132	RHOG
ENSG00000233690	8.093868963	0.356539013	4.390315547	0.007952005	0.021579385	EBAG9P1
ENSG00000224897	29.24058814	9.171852206	1.6849322	0.007954624	0.021584239	POT1-AS1
ENSG00000134882	862.9982372	1370.087215	-0.667027238	0.007957602	0.021590063	UBAC2
novel.1053	31.8408923	5.830946549	2.431235805	0.007968591	0.021617623	-
ENSG00000174365	294.7806421	159.5486728	0.884708795	0.007975148	0.021633152	SNHG11
ENSG00000154001	627.5415133	911.8490299	-0.539337443	0.00799682	0.021689673	PPP2R5E
ENSG00000248383	12.22717711	0.387220594	4.983317358	0.008001001	0.021698748	PCDHAC1
ENSG00000139174	328.1970592	88.48618108	1.889838083	0.00800624	0.021710689	PRICKLE1
ENSG00000144736	191.447335	325.8205322	-0.767831859	0.008009491	0.02171724	SHQ1
ENSG00000134020	16.26116191	1.365948763	3.538144335	0.008014675	0.021729027	PEBP4
ENSG00000169221	1070.721312	1637.44276	-0.612658989	0.008017874	0.021735433	TBC1D10B
ENSG00000166913	4393.132037	6567.87409	-0.580181897	0.008023876	0.021749434	YWHAB
ENSG00000162366	0	11.21775727	-6.066885966	0.00804598	0.021807075	PDZK1IP1
ENSG00000167525	153.0707219	75.78697512	1.016767526	0.00805018	0.02181618	PROCA1

ENSG00000168993	36.15314509	7.765893646	2.211910196	0.008063724	0.021850606	CPLX1
ENSG00000222044	11.6203523	0.723690511	4.015076717	0.008066887	0.021856898	AL031587.1
ENSG00000137876	1224.229271	1743.823817	-0.510471327	0.008075164	0.021877041	RSL24D1
novel.724	54.49052788	118.7893474	-1.125918041	0.008083738	0.021897827	-
ENSG00000093010	5174.717859	2858.786907	0.856058788	0.008085287	0.021897827	COMT
ENSG00000204967	7.348999548	0	5.210215389	0.008085364	0.021897827	PCDHA4
ENSG00000103145	1392.49601	784.7803621	0.827727046	0.008086973	0.0218999	HCFC1R1
ENSG00000287516	39.48821856	16.85631359	1.227026201	0.008113483	0.0219694	AL359764.2
ENSG00000164587	11335.71986	16360.91142	-0.529384031	0.008115066	0.021971399	RPS14
ENSG00000125629	461.2668887	1044.497316	-1.178983277	0.008117674	0.021976168	INSIG2
ENSG00000079134	793.1318866	477.050307	0.733846448	0.008124394	0.021992069	THOC1
ENSG00000087191	1674.413527	2427.411442	-0.535846162	0.008129995	0.022004938	PSMC5
ENSG00000165724	446.4609058	724.7451549	-0.699336274	0.008133047	0.022010905	ZMYND19
ENSG00000171853	387.2188182	747.6127073	-0.949074546	0.008135483	0.022015205	TRAPPC12
ENSG00000173209	2152.978144	1267.333027	0.764683909	0.008143097	0.022033515	AHSA2P
ENSG00000093167	1799.307703	1181.805458	0.606621577	0.008143963	0.022033562	LRRFIP2
ENSG00000165923	10.04730248	0.743759607	3.789583674	0.008149863	0.02204723	AGBL2
ENSG00000139537	32.52920498	67.74867166	-1.055887589	0.008154607	0.022057766	CCDC65
ENSG00000197586	963.8832605	1566.889097	-0.701181032	0.008157253	0.022060684	ENTPD6
ENSG00000132591	602.8643792	974.6562903	-0.693182314	0.008157384	0.022060684	ERAL1
ENSG00000280587	0	5.319715359	-4.994672855	0.00816076	0.022067518	LINC01348
ENSG00000103429	22.7048674	50.13340965	-1.145017445	0.008170392	0.022091263	BFAR
ENSG00000231178	8.267799015	0	5.380375184	0.00818363	0.022124753	AL357832.1
ENSG00000271993	34.06192422	7.817800198	2.120997211	0.00818819	0.022134778	AC126118.1
ENSG00000279058	21.36313109	5.279577167	2.006234006	0.008195859	0.022153205	AGAP14P
ENSG00000131446	2922.180792	5580.303587	-0.933336869	0.008204728	0.02217487	MGAT1
ENSG00000082515	475.9734227	712.1714066	-0.58132935	0.008206578	0.022177563	MRPL22
ENSG00000137691	48.88753894	136.0104195	-1.478059069	0.008217317	0.022204276	CFAP300
ENSG00000204131	194.761693	53.27388114	1.868604656	0.00822202	0.022212501	NHSL2
ENSG00000122566	13149.1311	20392.07282	-0.633049188	0.008222071	0.022212501	HNRNPA2B1
ENSG00000232000	28.0449878	6.207554658	2.157862747	0.008230547	0.022233088	CLCN3P1
ENSG00000124249	5.771904051	0	4.862288347	0.008239207	0.022251855	KCNK15
ENSG00000105352	5.771904051	0	4.862288347	0.008239207	0.022251855	CEACAM4
ENSG00000241769	37.10543742	13.55310598	1.45051213	0.008240479	0.022252976	LINC00893
ENSG00000222071	35.89980789	5.716264169	2.671101818	0.008244001	0.022260174	MIR1915
ENSG00000284791	6.982690717	0	5.137238124	0.008246045	0.022263379	SMIM41
ENSG00000144674	1255.555271	2007.188291	-0.676675455	0.008247112	0.022263947	GOLGA4
ENSG00000141279	1222.404964	2018.652833	-0.723527463	0.008250981	0.022272076	NPEPPS
ENSG00000181873	132.3289616	217.1385918	-0.715658526	0.008252827	0.022274746	IBA57
novel.425	60.92172506	9.284094441	2.712728198	0.008269266	0.022316797	-
ENSG00000272341	50.94405709	18.80187317	1.439717759	0.008271785	0.022321275	AL137003.2
ENSG00000162086	398.6257094	220.109987	0.856742677	0.008279432	0.022339591	ZNF75A
ENSG00000247095	2.932408476	200.8862179	-6.094465385	0.008293334	0.022374778	MIR210HG
ENSG00000122970	280.4265996	473.9726113	-0.756585435	0.008301822	0.022395353	IFT81
ENSG00000198756	129.4585796	30.96091735	2.061377285	0.008305566	0.022403126	COLGALT2
ENSG00000265100	15.66930962	3.566546002	2.131557448	0.008309276	0.022408671	AC005332.1
ENSG00000116690	11.82820214	78.622861	-2.734066473	0.008309347	0.022408671	PRG4
ENSG00000185433	5.732827126	0	4.853487914	0.00831454	0.022420349	LINC00158
ENSG00000197363	274.0878731	180.3583207	0.604249315	0.008319076	0.022430252	ZNF517
ENSG00000186501	949.0398809	659.8496816	0.524298893	0.008329626	0.022456366	TMEM222
ENSG00000100628	7.645930071	0	5.267539228	0.008335987	0.022471186	ASB2

ENSG00000102870	649.3413867	965.1764873	-0.571775876	0.008337021	0.022471642	ZNF629
ENSG00000109084	165.0017518	375.6432389	-1.187723806	0.008345019	0.022489297	TMEM97
ENSG00000260604	4.252761481	23.53508964	-2.474226074	0.008345303	0.022489297	AL590004.3
ENSG00000171132	242.7877885	127.7718446	0.925890046	0.008355328	0.022513977	PRKCE
ENSG00000184857	112.2598569	207.1562701	-0.88443582	0.008384662	0.022588352	TMEM186
ENSG00000135945	721.2118917	445.172795	0.696392559	0.008384668	0.022588352	REV1
ENSG00000172766	364.0415532	224.8700322	0.694832065	0.008392987	0.022608418	NAA16
ENSG00000162910	565.3811366	821.9942978	-0.539684584	0.008395713	0.022613416	MRPL55
ENSG00000241218	15.10428249	3.210006989	2.230005904	0.008397257	0.022615231	AC063944.2
ENSG00000273456	29.25946414	9.508450517	1.61218795	0.008403016	0.022628396	AC064836.3
ENSG00000122390	1000.889758	665.9648535	0.587653243	0.008405679	0.022633221	NAA60
ENSG00000279457	242.2768397	110.9792531	1.12559077	0.008411222	0.022645798	WASH9P
ENSG00000127663	942.1829986	1725.411378	-0.872639985	0.008413936	0.022650759	KDM4B
ENSG00000226986	3.311325065	17.20583614	-2.378228172	0.008422111	0.022667861	AC092017.1
ENSG00000279017	12.93465052	0	6.026288194	0.008422259	0.022667861	AC098476.1
ENSG00000287454	1.524157165	10.11989879	-2.734714634	0.008422906	0.022667861	AC008149.2
ENSG00000245870	6.862952646	0	5.114106285	0.008427443	0.022677267	LINC00682
ENSG00000109066	443.3862645	690.2594049	-0.639085544	0.008428147	0.022677267	TMEM104
ENSG00000119686	525.2547162	112.8430765	2.218834363	0.008436347	0.022696979	FLVCR2
ENSG00000165115	125.0374168	65.31194998	0.938856889	0.008446378	0.022721613	KIF27
ENSG00000271425	95.64646101	165.8245599	-0.795121428	0.008478983	0.022806963	NBPF10
ENSG00000067601	66.93565529	31.08223101	1.108476858	0.008482159	0.022813145	PMS2P4
ENSG00000249502	17.61654616	3.159256312	2.46450916	0.00849719	0.022851206	AC006160.1
ENSG00000215458	19.83514171	4.616093944	2.097747247	0.008505987	0.022872496	AATBC
ENSG00000196586	1271.654382	875.1763775	0.53895023	0.008508935	0.022878054	MYO6
novel.710	6.012206566	0	4.92258688	0.008529255	0.022930317	-
ENSG00000092203	1005.200587	1357.512264	-0.433509361	0.008531367	0.02293362	TOX4
ENSG00000228314	971.7851179	40.53119776	4.584282706	0.008536871	0.022946043	CYP4F29P
ENSG00000187715	13.96412779	0.774441189	4.23418577	0.008555796	0.022994531	KBTBD12
ENSG00000147003	11.16489151	1.386017859	2.979518094	0.008557951	0.022996132	CLTRN
ENSG00000287511	0.907445357	9.04912588	-3.315011895	0.008558162	0.022996132	AL109984.1
ENSG00000023909	337.6955339	656.0165736	-0.958138915	0.008575836	0.02304124	GCLM
ENSG00000275318	25.3074654	5.901766323	2.084390275	0.008584097	0.023061052	AL136981.2
ENSG00000099783	3719.246738	6120.884838	-0.718787761	0.008589876	0.023074191	HNRNPM
novel.756	12.35540599	0	5.960969473	0.008590799	0.023074283	-
ENSG00000169006	5.787019107	0	4.865696279	0.008609257	0.023121472	NTSR2
ENSG00000160345	32.10648161	66.4664669	-1.048459673	0.008610164	0.023121516	C9orf116
ENSG00000258234	7.989317219	0	5.333648608	0.008615983	0.023134751	AC024257.1
ENSG00000198105	483.9184631	273.1948987	0.825461824	0.00862038	0.023144165	ZNF248
ENSG00000228852	31.08452659	7.998422062	1.964052249	0.008627479	0.023160832	AC092802.2
ENSG00000226352	18.70383381	3.678659843	2.361616077	0.008630039	0.023165128	PSPC1-AS2
ENSG00000154451	247.362866	61.18761325	2.017462897	0.008630863	0.023165128	GBP5
ENSG00000064989	245.024789	62.10147627	1.978023258	0.008637184	0.023179701	CALCRL
ENSG00000230730	5.763057239	0	4.86029903	0.008640378	0.023185878	AC074011.1
ENSG00000215452	5.756788995	0	4.858893229	0.008648554	0.023205419	ZNF663P
ENSG00000158805	683.9656889	394.5743413	0.793170065	0.008656096	0.023223258	ZNF276
novel.632	10.45874302	0.387220594	4.759732503	0.008673768	0.023267926	-
ENSG00000104047	860.7830945	1840.045824	-1.095821207	0.008674536	0.023267926	DTWD1
ENSG00000100348	965.8299832	1534.94595	-0.668548054	0.008677521	0.023272551	TXN2
ENSG00000133800	87.67429613	11.99720714	2.862487698	0.008678052	0.023272551	LYVE1
ENSG00000225783	41.17613405	14.817425	1.476302526	0.008682679	0.023282556	MIAT

ENSG00000248593	104.2302091	52.89928081	0.980618777	0.008692661	0.023306917	DSTNP2
ENSG00000268621	5.71771207	0	4.85006095	0.008699179	0.023321987	IGFL2-AS1
ENSG00000269976	18.72138489	3.362259022	2.501010905	0.0087038	0.023331968	AC012065.3
ENSG00000127124	251.5462933	586.7999598	-1.221709805	0.008710159	0.023346604	HIVEP3
ENSG00000197646	62.47629762	161.3367493	-1.367455793	0.008721481	0.023374542	PDCD1LG2
ENSG00000068976	105.8554414	18.48102443	2.511832371	0.008728476	0.023390875	PYGM
ENSG00000067560	9254.769892	14220.24918	-0.619689933	0.008729917	0.023392324	RHOA
ENSG00000139505	651.8612992	937.1007339	-0.523699563	0.008743542	0.023426417	MTMR6
ENSG00000286685	6.704564926	0	5.078172125	0.008763578	0.023477678	AL158077.2
ENSG00000135373	2.775203133	220.9168483	-6.316617745	0.008766185	0.023482239	EHF
ENSG00000167797	1323.991164	847.5079875	0.643661137	0.008767168	0.023482453	CDK2AP2
ENSG00000231625	10.00955048	1.130980201	3.191229918	0.008775101	0.023501278	SLC47A1P2
ENSG00000287958	6.584826854	0	5.054167423	0.008780774	0.023514046	AC092053.5
ENSG00000156853	265.7454243	175.9737276	0.594693847	0.008784154	0.023520674	ZNF689
ENSG00000011566	1048.603283	654.3117273	0.680166248	0.008786384	0.023524222	MAP4K3
ENSG00000064313	505.7386792	762.5753676	-0.592225029	0.008790613	0.023533118	TAF2
ENSG00000270659	28.13915435	5.158006716	2.430447408	0.008795634	0.023544134	AC079610.2
ENSG00000231613	6.272710004	0	4.984485175	0.008815671	0.023594328	AC099568.1
ENSG00000219201	220.1785123	427.9523308	-0.959245351	0.008816202	0.023594328	AC138392.1
ENSG00000235531	79.09513466	291.4808703	-1.881954966	0.008836979	0.023647497	MSC-AS1
ENSG00000105538	153.1614832	439.1151684	-1.51929938	0.008846754	0.023671217	RASIP1
ENSG00000125304	2282.356124	3428.294794	-0.586976066	0.008848599	0.023673716	TM9SF2
ENSG00000282386	5.452194037	19.31182009	-1.820590819	0.008856986	0.023692734	AL358472.4
ENSG00000240401	16.96444711	4.290236513	1.980372968	0.008857531	0.023692734	AC012358.3
ENSG00000096093	768.2026689	448.6960863	0.77577419	0.008865496	0.023711597	EFHC1
ENSG00000100603	915.4917542	1249.024255	-0.448288628	0.008868557	0.023717342	SNW1
ENSG00000187109	5887.856948	8982.688434	-0.609420395	0.008888844	0.02376915	NAP1L1
ENSG00000168734	824.8955621	521.1139959	0.662882041	0.008891067	0.023772647	PKIG
ENSG00000136457	3.65116473	15.33695692	-2.075705762	0.00889926	0.023792104	CHAD
ENSG00000228393	84.78720464	38.93541751	1.126556183	0.008908808	0.023815182	LINC01004
ENSG00000120314	456.3035933	729.8327958	-0.677286717	0.00891108	0.023818803	WDR55
ENSG00000159763	0.601596751	15.61895175	-4.691623362	0.008914904	0.023826572	PIP
ENSG00000237039	11.24039552	45.4830381	-2.017695534	0.008919142	0.023835449	AC018738.1
ENSG00000198743	1151.540587	514.1003843	1.163045895	0.008926999	0.023853993	SLC5A3
ENSG00000269954	0.616711807	11.92137868	-4.283740016	0.008928726	0.023856153	AC022239.1
ENSG00000275340	6.114251013	0	4.944670534	0.00893004	0.02385721	FGD5P1
ENSG00000287336	11.76022002	0.774441189	3.992317561	0.008932955	0.023862545	AL137157.1
ENSG00000198650	6.107982769	0	4.943325334	0.008939663	0.023878007	TAT
novel.958	1165.564706	192.3476469	2.599583231	0.008941115	0.023879433	-
ENSG00000172731	160.0096771	320.7045559	-1.004137906	0.008949093	0.023898281	LRRC20
novel.336	52.49139319	16.55065365	1.660232809	0.008951036	0.023901015	-
ENSG00000147592	115.9514944	276.8040355	-1.256480662	0.008953009	0.023903646	LACTB2
ENSG00000125775	63.49964883	31.6631261	1.004297278	0.008953862	0.023903646	SDCBP2
ENSG00000215114	483.2848399	173.2303314	1.478903182	0.008956126	0.023907234	UBXN2B
ENSG00000186960	0	6.73513053	-5.328250729	0.008959323	0.023913312	LINC01551
ENSG00000136158	1731.446466	963.66955	0.845691426	0.008970161	0.023939779	SPRY2
ENSG00000267370	24.40886685	7.836713419	1.635534343	0.008985566	0.02397843	AC008752.3
ENSG00000275591	0	5.73646166	-5.101657879	0.008989542	0.023986577	XKR5
novel.87	6.062637601	0	4.933526287	0.009009353	0.024036968	-
ENSG00000254695	6.553343093	0	5.047794071	0.009013523	0.024045625	AC087379.2
ENSG00000152133	429.6020502	288.1501101	0.576259471	0.009020621	0.024062091	GPATCH11

ENSG00000170638	777.2197936	1251.274901	-0.686734248	0.009031396	0.024088357	TRABD
novel.833	7.014103207	0	5.143272555	0.009043264	0.024117537	-
ENSG00000124215	26.11043028	6.827303669	1.936938656	0.009056152	0.024149428	CDH26
ENSG00000182871	5503.589753	2362.646985	1.219899294	0.009064282	0.024168627	COL18A1
ENSG00000198763	48953.40371	31678.5131	0.627906806	0.009068753	0.024178067	MT-ND2
ENSG00000105726	1179.20478	1694.778086	-0.523233439	0.009078961	0.024202801	ATP13A1
ENSG00000179562	387.2458578	565.8742894	-0.547465098	0.009080909	0.02420551	GCC1
ENSG00000250995	16.27724588	3.557089392	2.189035655	0.009082719	0.024207849	AL391280.1
ENSG00000276404	0.894837599	9.099876557	-3.329562819	0.009086185	0.024214603	MIR6835
ENSG00000117643	627.5623634	101.4590811	2.627902177	0.00909802	0.024243658	MAN1C1
ENSG00000124479	119.2599134	16.63884559	2.846854975	0.009099479	0.024245058	NDP
ENSG00000279863	19.91329556	5.727005049	1.796779307	0.00910514	0.024257655	AC069547.1
ENSG00000272338	26.30191142	6.247564456	2.085613874	0.009107546	0.024261576	AC067838.1
ENSG00000225171	13.64656906	0.713078025	4.253385958	0.009109471	0.024264216	DUTP6
ENSG00000264743	15.0034917	1.762625967	3.077367727	0.009115441	0.024277629	DPRXP4
ENSG00000077380	1892.517686	2612.386155	-0.46508021	0.009121816	0.024292118	DYNC1I2
ENSG00000249700	47.35661533	21.97174197	1.110602441	0.009123891	0.024295153	SRD5A3-AS1
ENSG00000115541	561.9418952	907.5563327	-0.691740159	0.009136521	0.024321689	HSPE1
ENSG00000126262	7.743928837	0	5.288153953	0.009137183	0.024321689	FFAR2
ENSG00000146216	7.53761738	0.336469917	4.286754675	0.009137482	0.024321689	TTBK1
ENSG00000197933	124.0768196	48.22696078	1.360629328	0.009138468	0.024321689	ZNF823
ENSG00000265702	25.63226128	5.981914313	2.119572121	0.009138537	0.024321689	AC005821.1
ENSG00000243742	4.625338556	55.50331493	-3.587799145	0.009141816	0.024327925	RPLP0P2
ENSG00000267002	255.6369499	148.1802389	0.788067468	0.009152551	0.024353998	AC060780.1
ENSG00000100796	1347.949756	971.859279	0.471997436	0.009170484	0.024399216	PPP4R3A
ENSG00000115762	1623.39241	2743.431215	-0.757038628	0.009179286	0.024420135	PLEKHB2
ENSG00000134253	210.6488549	128.1909027	0.716351444	0.009183767	0.024429553	TRIM45
ENSG00000155111	825.2582254	485.3447096	0.766318864	0.009189166	0.024441414	CDK19
ENSG00000173559	1165.405945	2225.529684	-0.933274848	0.009215909	0.024510034	NABP1
ENSG00000281026	79.0664158	32.23212444	1.297535513	0.009259561	0.024623609	N4BP2L2-IT2
ENSG00000223776	0	5.310258749	-4.992165344	0.009289082	0.024699586	LGALS8-AS1
ENSG00000033178	782.5305117	1376.010926	-0.814022515	0.009293271	0.024708193	UBA6
ENSG00000272784	17.17863646	1.874739808	3.221375395	0.009298767	0.024720276	AC019193.2
ENSG00000124733	846.5445268	1299.532581	-0.618596209	0.009309086	0.024742928	MEA1
ENSG00000158234	305.8478199	148.8565649	1.037444654	0.009309192	0.024742928	FAIM
ENSG00000245468	19.51299567	3.984448178	2.298571997	0.009329334	0.024793142	LINC02447
ENSG00000120156	182.6353512	391.9343598	-1.102274427	0.009330157	0.024793142	TEK
ENSG00000259298	19.72808267	3.537020296	2.473394553	0.009330947	0.024793142	AC012170.2
novel.1111	8.742135802	0.356539013	4.500461782	0.009348959	0.024837714	-
novel.369	5.466055445	0	4.783771155	0.009349634	0.024837714	-
ENSG00000272568	7.043079671	0	5.148838622	0.009357633	0.024856422	AC005162.3
ENSG00000121406	176.5264426	112.2235843	0.653497606	0.0093605	0.024859649	ZNF549
ENSG00000105245	714.1705475	1035.312963	-0.535587213	0.009360761	0.024859649	NUMBL
ENSG00000125676	962.8989584	1415.040198	-0.555229439	0.009365782	0.024870439	THOC2
ENSG00000258274	5.453447686	0	4.780794603	0.009366821	0.024870656	AC012085.2
ENSG00000260468	5.450940389	0	4.780205744	0.009370256	0.024876651	LINC01290
ENSG00000279812	2.450478525	14.93899545	-2.612305286	0.009370994	0.024876651	AC120057.4
novel.438	236.1802812	674.4129148	-1.513947673	0.009372467	0.024877806	-
ENSG00000259871	7.228007828	0.356539013	4.226512757	0.009373344	0.024877806	AC130456.1
ENSG00000118894	99.50895179	205.33806	-1.046582034	0.009386189	0.024904679	EEF2KMT
novel.490	43.68603296	9.995888197	2.136262051	0.009386258	0.024904679	-

ENSG00000272953	37.41805282	11.3843462	1.712744648	0.009386345	0.024904679	AC092171.4
ENSG00000133874	165.131705	86.35314592	0.938040352	0.009388002	0.024906533	RNF122
ENSG00000147118	225.0753524	121.809871	0.88561495	0.009392872	0.024916294	ZNF182
ENSG00000246308	25.86281934	6.737570675	1.934162493	0.009393599	0.024916294	AC116535.1
ENSG00000131748	1649.171954	1055.073414	0.644558568	0.009394917	0.024917245	STARD3
ENSG00000168994	1391.340526	855.9680113	0.700608845	0.009416454	0.024971818	PXDC1
ENSG00000260917	202.8618465	118.8841706	0.772599466	0.009418098	0.024973628	AL158212.3
ENSG00000255455	250.6642926	112.6634349	1.155460705	0.00942318	0.024984555	AP003486.1
ENSG00000138767	609.2158563	381.5297096	0.675860613	0.009427436	0.024993286	CNOT6L
ENSG00000251003	33.84002699	173.5773541	-2.359472307	0.009445988	0.025039915	ZFPM2-AS1
ENSG00000011590	13.25916167	2.51699806	2.399588473	0.009450183	0.02504848	ZBTB32
novel.309	5.980722805	0	4.915670547	0.00945886	0.025068921	-
ENSG00000221968	4151.908727	1814.120902	1.194592418	0.00946468	0.025081788	FADS3
ENSG00000115350	298.6317631	528.9631796	-0.8253562	0.009470375	0.02509432	POLE4
ENSG00000160055	178.7299944	300.8134633	-0.751457418	0.009478821	0.025112442	TMEM234
ENSG00000114902	1073.943168	1749.189733	-0.703951135	0.009479147	0.025112442	SPCS1
ENSG00000214548	6673.24534	11359.33352	-0.767386152	0.009489993	0.025138613	MEG3
ENSG00000285554	16.83976571	3.250145181	2.377875592	0.00950272	0.025169758	AC242988.2
ENSG00000172006	141.2204248	81.90633784	0.78545371	0.009506334	0.025176765	ZNF554
ENSG00000259520	2.06654734	17.67906531	-3.080908151	0.009517027	0.025202515	AC051619.5
ENSG00000235438	17.59480686	1.00940975	4.081681856	0.009524811	0.025220558	ESRRAP2
ENSG00000253593	1.524157165	12.67575078	-3.054381467	0.009526331	0.025222013	AC110741.1
ENSG00000245060	117.7523102	62.1800832	0.91832453	0.00952741	0.025222299	LINC00847
ENSG00000267809	17.2639562	52.17661778	-1.593796507	0.009546048	0.025269064	NDUFV2P1
ENSG00000164089	12.30038763	0	5.953784591	0.009547546	0.025270455	ETNPPL
ENSG00000155304	968.4743792	1497.895307	-0.629006142	0.009549298	0.025272518	HSPA13
ENSG00000273265	10.29652308	0.713078025	3.844641831	0.009557697	0.02528977	CNNM3-DT
ENSG00000236871	55.42534005	106.4833982	-0.939877876	0.009557763	0.02528977	LINC00106
ENSG00000015133	79.71191774	21.3402246	1.899006884	0.009559733	0.025292407	CCDC88C
ENSG00000157578	22.07805513	48.12391837	-1.123482643	0.009571776	0.025321689	LCA5L
ENSG00000143341	146.1128466	32.18163055	2.181080117	0.009577066	0.025333106	HMCN1
ENSG00000286507	7.632139934	0.387220594	4.302694923	0.009592138	0.02537039	AC004947.3
ENSG00000235245	49.49239844	21.45105417	1.212218466	0.009598221	0.025383895	AL360181.2
ENSG00000286835	14.47113071	3.240688571	2.162336109	0.009605946	0.02540174	AL133477.2
ENSG00000278811	3.944405577	20.85826233	-2.398534901	0.009616115	0.025426043	LINC00624
ENSG00000117481	280.0750776	417.0953508	-0.574595993	0.009623855	0.025443917	NSUN4
ENSG00000287569	28.63773774	5.483864147	2.365731493	0.009641172	0.025487108	AL078587.2
ENSG00000215190	185.629445	85.2359075	1.120481481	0.009653363	0.025516739	LINC00680
ENSG00000137193	286.3705943	648.8371663	-1.17980658	0.009659537	0.025528649	PIM1
ENSG00000127328	151.3744585	72.58937846	1.058609	0.009659834	0.025528649	RAB3IP
ENSG00000226329	43.54294593	12.56363693	1.803373913	0.009662036	0.025530207	AC005682.1
ENSG00000025796	1556.799985	2321.178324	-0.576338678	0.009662389	0.025530207	SEC63
ENSG00000178074	279.2428085	410.8851805	-0.557127249	0.009682766	0.025581415	C2orf69
ENSG00000137944	954.5939924	589.3786034	0.69608098	0.009683739	0.025581415	KYAT3
novel.1078	10.14433233	0.387220594	4.713203061	0.009691006	0.02559801	-
ENSG00000101670	31.7311125	7.408198758	2.092618871	0.009698781	0.025615943	LIPG
ENSG00000137875	0	6.381031662	-5.256282798	0.009701678	0.02562099	BCL2L10
ENSG00000147684	979.2976785	1429.058016	-0.545299116	0.009707439	0.025633599	NDUFB9
ENSG00000111834	21.75304579	2.475703993	3.128184872	0.009709221	0.025635697	RSPH4A
ENSG00000186453	18.51758074	5.02453951	1.881423317	0.009712631	0.025642096	FAM228A
ENSG00000256683	194.716036	109.9133946	0.824299735	0.009722166	0.025664662	ZNF350

ENSG00000277702	17.52488691	108.7642718	-2.635058665	0.009732421	0.025687857	AC239859.6
ENSG00000107020	242.2291725	369.1449907	-0.608213819	0.009732931	0.025687857	PLGRKT
ENSG00000118655	185.4174348	316.7711496	-0.773214345	0.00973476	0.025690077	DCLRE1B
ENSG00000273447	11.74879464	1.41669944	3.042023246	0.009736184	0.025691224	AC004067.1
ENSG00000224479	7.507387268	0.336469917	4.28161068	0.009743307	0.02570741	AC104162.1
ENSG00000255320	17.05646236	41.33572541	-1.281133946	0.009744518	0.025707995	AP000759.1
ENSG00000187742	1227.646192	777.9558494	0.65846624	0.009748026	0.025714638	SECISBP2
ENSG00000258429	51.84494947	114.3232409	-1.143114074	0.009750839	0.025717624	PDF
ENSG00000263293	6.070159493	36.52899223	-2.586843186	0.009751342	0.025717624	AC068234.2
ENSG00000168890	422.7778894	268.9094609	0.651762792	0.009752128	0.025717624	TMEM150A
ENSG00000217825	0	10.98394458	-6.03593705	0.009761533	0.025739814	AC099552.1
ENSG00000251768	8.803849676	0.693008929	3.630021057	0.00976313	0.025741412	RNA5SP217
ENSG00000178694	262.9784985	167.9661058	0.646070186	0.009780455	0.025783477	NSUN3
ENSG00000110218	503.4191076	1290.010557	-1.357364737	0.009781724	0.025783477	PANX1
ENSG00000155903	828.8453827	503.8048942	0.718516739	0.009782061	0.025783477	RASA2
ENSG00000100024	26.23407149	5.787212337	2.182803181	0.009786587	0.025792789	UPB1
ENSG00000120896	3128.830728	1790.872811	0.804884047	0.009789351	0.025797458	SORBS3
ENSG00000135913	68.73110053	137.2103273	-0.99569824	0.009793063	0.025804622	USP37
ENSG00000252147	6.115504662	0	4.944986835	0.009800426	0.025821403	RNY3P16
ENSG00000205476	131.2533837	258.8204065	-0.979725674	0.00980621	0.025834024	CCDC85C
ENSG00000105983	472.4189593	711.4421463	-0.590873876	0.009807887	0.025835822	LMBR1
ENSG00000166181	1467.395166	1975.933871	-0.429346753	0.009813003	0.025846679	API5
ENSG00000131323	633.464852	1079.45849	-0.768788969	0.009821632	0.025866784	TRAF3
ENSG00000143632	7.95937184	0.336469917	4.362991402	0.009832415	0.025892558	ACTA1
ENSG00000072364	1685.315052	2511.169534	-0.575240426	0.009840803	0.025912021	AFF4
ENSG00000175745	6959.605462	2468.761381	1.495131714	0.009848116	0.025928649	NR2F1
ENSG00000235903	14.6374676	2.120320855	2.776086878	0.009851855	0.025935863	CPB2-AS1
ENSG00000104872	809.7121446	1294.237136	-0.676328694	0.009853988	0.025938852	PIH1D1
ENSG00000281207	13.60025498	32.7245708	-1.26859934	0.009868911	0.025975501	SLFNL1-AS1
ENSG00000107897	868.9476198	555.4395649	0.645505853	0.009878574	0.025998301	ACBD5
ENSG00000272970	18.60171809	3.486269618	2.40008318	0.009884233	0.026010561	AC107294.2
ENSG00000149596	18.48226476	102.0752216	-2.46445096	0.009897082	0.026041733	JPH2
ENSG00000251151	0.616711807	13.07743666	-4.423755608	0.009944695	0.026164367	HOXC-AS3
ENSG00000100003	293.4144891	900.9422764	-1.618133153	0.009946229	0.026165752	SEC14L2
ENSG00000178950	1636.943093	2551.142968	-0.640000228	0.009959247	0.026197348	GAK
ENSG00000116652	19.18193155	4.371668772	2.14417846	0.009964047	0.026207319	DLEU2L
ENSG00000237115	5.423217574	0	4.773630415	0.009965265	0.026207871	AL139805.1
ENSG00000166250	493.6407511	2431.759414	-2.300537989	0.009990412	0.026270055	CLMP
ENSG00000198838	81.56413389	32.99266109	1.300235092	0.009990932	0.026270055	RYR3
ENSG00000248174	63.4624819	8.918098818	2.830680387	0.010012171	0.026322156	LINC02268
ENSG00000174529	45.5004964	21.01436717	1.110255836	0.010012773	0.026322156	TMEM81
ENSG00000232352	24.53738047	8.060941101	1.618113055	0.010013857	0.026322341	SEMA3B-AS1
ENSG00000137502	429.888224	192.8027929	1.155522778	0.010023441	0.026344868	RAB30
ENSG00000134955	748.9378084	248.6896256	1.591189584	0.010028085	0.026353129	SLC37A2
ENSG00000231177	28.4512712	7.285344037	1.973183721	0.010028613	0.026353129	LINC00852
ENSG00000105662	478.3232954	284.2065364	0.750165798	0.010037594	0.026374063	CRTC1
ENSG00000267990	5.979469157	0	4.915356426	0.010051808	0.026408739	AC063977.2
ENSG00000185739	11.57396694	1.130980201	3.394994896	0.010057375	0.026420693	SRL
ENSG00000104517	1334.380743	2177.11847	-0.706111491	0.010060031	0.026424998	UBR5
ENSG00000141551	2581.798578	3796.218546	-0.556090133	0.01006377	0.026430918	CSNK1D
ENSG00000149506	9.854852431	80.63966022	-3.033845538	0.010064319	0.026430918	ZP1

ENSG00000139292	10.03476599	1.049547942	3.232325191	0.01007546	0.026457503	LGR5
ENSG00000233247	18.8335298	46.95536851	-1.322172024	0.010082106	0.026472278	AC002524.1
ENSG00000131378	854.894995	1309.781486	-0.6157726	0.010085944	0.026479681	RFTN1
ENSG00000140265	448.0519061	290.8827783	0.623904791	0.010091446	0.026491449	ZSCAN29
ENSG00000218416	29.34582406	2.832243005	3.367921953	0.010094918	0.026497884	AC110619.1
ENSG00000230102	11.99167572	1.365948763	3.095496719	0.010106969	0.026526838	LINC02028
ENSG00000138641	12.05722181	1.854670712	2.728053021	0.010115687	0.026547037	HERC3
ENSG00000259205	20.04904633	1.936102971	3.413814278	0.010136365	0.026598617	PRKXP1
ENSG00000163634	653.4932277	982.3800589	-0.588351479	0.010147323	0.026622179	THOC7
ENSG00000143443	128.6088458	75.41293555	0.771465732	0.010147394	0.026622179	C1orf56
ENSG00000227373	5.439586279	0	4.777509721	0.010158024	0.026647376	AL121983.2
ENSG00000135960	7.375397443	0	5.215171836	0.010163284	0.026658294	EDAR
ENSG00000214955	5.435825332	0	4.776621393	0.010164238	0.026658294	AP000317.1
ENSG00000262410	14.9027009	1.406086955	3.392295512	0.010177331	0.026689941	AC024361.1
ENSG00000177469	10845.64482	18035.35077	-0.733732932	0.010184367	0.026705252	CAVIN1
ENSG00000172940	0.293240848	6.816691183	-4.388705843	0.010185226	0.026705252	SLC22A13
ENSG00000143493	170.0140708	328.4788787	-0.951144818	0.010191809	0.026719816	INTS7
ENSG00000166960	7.029218263	0	5.14621271	0.010204142	0.026748767	CCDC178
ENSG00000173889	1230.406779	780.6195294	0.656591809	0.010204911	0.026748767	PHC3
ENSG00000105877	6.330662931	26.10484616	-2.038650988	0.01020813	0.026754507	DNAH11
ENSG00000235180	0.61420451	9.05858249	-3.893877118	0.010214546	0.026768621	LINC00601
ENSG00000117461	277.9301474	105.3226824	1.397990954	0.010234434	0.026818036	PIK3R3
ENSG00000152463	18.79842729	328.4630948	-4.127750901	0.010255692	0.026868801	OLAH
ENSG00000196562	900.4346028	170.9676003	2.396291995	0.010255875	0.026868801	SULF2
novel.142	4.546002329	17.04053148	-1.909946422	0.010263014	0.026884792	-
ENSG00000174498	7.686260644	0	5.274789827	0.010268073	0.02689262	IGDCC3
ENSG00000245954	7.686260644	0	5.274789827	0.010268073	0.02689262	LINC02273
ENSG00000079150	335.7751222	727.5246954	-1.115604591	0.010269849	0.026894561	FKBP7
ENSG00000271646	108.68857	36.86512382	1.555067577	0.010280256	0.026919102	AC099343.3
ENSG00000163993	0.909952655	15.3123115	-4.078010563	0.01028403	0.026926272	S100P
ENSG00000164406	44.58524442	13.88243104	1.681332512	0.010309364	0.026989883	LEAP2
ENSG00000164506	605.8071739	998.5205969	-0.721118058	0.010310586	0.02699036	STXBP5
ENSG00000116793	314.631753	532.07228	-0.758368609	0.010318484	0.027008316	PHTF1
ENSG00000106069	7.23685464	0.356539013	4.228017801	0.010353154	0.027096332	CHN2
novel.380	16.03024785	0.672939833	4.526657364	0.010355122	0.027098753	-
ENSG00000012211	125.6004348	209.7094592	-0.738343803	0.010357606	0.027102523	PRICKLE3
ENSG00000284505	7.326291328	0.387220594	4.243630399	0.010363069	0.027114088	LYNX1-SLURP2
ENSG00000224367	5.454701335	0	4.781095046	0.010369201	0.027127399	OACYLP
ENSG00000117298	3785.888518	6298.352694	-0.734278905	0.010376021	0.02714251	ECE1
ENSG00000167523	107.7219181	177.8556595	-0.723161534	0.010380126	0.027150514	SPATA33
ENSG00000100558	50.95247663	156.6543307	-1.61940436	0.010383195	0.027155806	PLEK2
ENSG00000184900	1550.182464	2260.719761	-0.544453351	0.010384432	0.027156309	SUMO3
ENSG00000227719	9.654595755	0.672939833	3.780488325	0.010387115	0.027160591	AC006042.1
ENSG00000188846	7479.744721	11379.06928	-0.605342412	0.010398092	0.027186559	RPL14
ENSG00000268529	7.616953607	0	5.262432305	0.01040843	0.027210848	CYP2T3P
ENSG00000248608	5.421963925	0	4.773319105	0.010426196	0.027248256	AC133963.1
ENSG00000285697	5.421963925	0	4.773319105	0.010426196	0.027248256	AC002059.3
ENSG00000067836	315.7551396	196.5413092	0.684923306	0.010426429	0.027248256	ROGDI
ENSG00000270035	6.554596742	0	5.047943845	0.010426934	0.027248256	AL359881.2
ENSG00000224843	15.17351826	3.311508345	2.212902182	0.010444168	0.027290548	LINC00240
ENSG00000196678	334.3740535	200.3567752	0.73793985	0.01044575	0.027291937	ERI2

ENSG00000261584	6.382276343	0	5.007377559	0.010456724	0.027317862	AL513548.1
ENSG00000119541	735.9603055	1062.44745	-0.529483339	0.010460133	0.027324021	VPS4B
ENSG00000134815	372.7822925	584.3396583	-0.648825506	0.010461726	0.027325435	DHX34
ENSG00000281344	9.590303313	32.09878562	-1.738375646	0.010466378	0.027334837	HELLPAR
ENSG00000226137	656.8621657	369.5108923	0.830712244	0.010471712	0.027346021	BAIAP2-DT
ENSG00000236914	38.49169252	11.25216326	1.765901955	0.010487601	0.027384761	LINC01852
ENSG00000167874	44.92995614	15.32737192	1.558031661	0.010502455	0.027418313	TMEM88
ENSG00000228013	15.72851619	2.844011366	2.46181066	0.010503476	0.027418313	IL6R-AS1
ENSG00000267041	60.07736123	27.2831566	1.138695634	0.010503617	0.027418313	ZNF850
ENSG00000266910	23.85261527	5.604150329	2.109726047	0.010508188	0.02742749	AC008507.1
ENSG00000213145	23.8818052	4.230029225	2.487939114	0.010529279	0.027479778	CRIP1
ENSG00000225205	102.3675806	21.80502464	2.236995856	0.01053155	0.027482944	AC078883.1
ENSG00000104442	507.5392653	798.2491411	-0.653652848	0.010537348	0.027495313	ARMC1
ENSG00000083799	1798.287824	907.5870442	0.986832814	0.010547505	0.02751704	CYLD
ENSG00000231652	1.232169966	10.1293554	-3.051260112	0.010547793	0.02751704	AL590428.1
ENSG00000267299	8.343018291	0.356539013	4.434546179	0.010556533	0.027537078	AC011444.3
ENSG00000071537	1973.966	3094.16785	-0.648360416	0.010577088	0.027587925	SEL1L
ENSG00000229056	0	5.02453951	-4.910064739	0.010586776	0.027610423	HECW2-AS1
ENSG00000128881	465.541548	252.6991984	0.882207476	0.010590762	0.027618045	TTBK2
ENSG00000257252	13.46575786	1.069617038	3.647008419	0.010616635	0.027682738	AC124947.1
ENSG00000100985	16.49706274	721.8196354	-5.450656948	0.010629894	0.027714529	MMP9
ENSG00000125735	49.83287884	7.999449543	2.652809519	0.010638931	0.027735305	TNFSF14
ENSG00000204055	18.55407909	3.893559308	2.243810411	0.010678557	0.027835816	AL158151.1
ENSG00000165119	9071.440536	12683.56067	-0.483581209	0.010693487	0.027871939	HNRNPK
ENSG00000169919	1348.859735	968.4366502	0.477945654	0.010696462	0.027876895	GUSB
ENSG00000280107	17.75974756	3.342189926	2.430844564	0.010704339	0.027894627	AL022393.1
ENSG00000136807	2024.503695	1228.993577	0.720178957	0.010717919	0.027927213	CDK9
novel.38	7.713698725	0	5.282747095	0.010722048	0.027933557	-
ENSG00000203326	128.570524	64.38435767	0.995862676	0.010722503	0.027933557	ZNF525
ENSG00000053371	683.1868389	1153.291806	-0.755597025	0.010743584	0.027985668	AKR7A2
ENSG00000101460	872.0397569	486.4393235	0.842718231	0.010747727	0.027993653	MAP1LC3A
ENSG00000196072	976.5681766	578.7198053	0.754278814	0.010763279	0.028031351	BLOC1S2
ENSG00000143801	239.3973413	119.4859524	1.000337623	0.010767021	0.028038284	PSEN2
ENSG00000245532	11063.10148	24575.38551	-1.151446848	0.010769147	0.028041012	NEAT1
ENSG00000106018	15.0943955	0.336469917	5.287184297	0.010776341	0.028056931	VIPR2
ENSG00000145107	0.615458159	11.3204145	-4.208740731	0.010783787	0.028071738	TM4SF19
ENSG00000141519	119.2608972	47.69424783	1.322711759	0.010784189	0.028071738	CCDC40
ENSG00000198841	52.89344493	105.2076148	-0.994308733	0.01084363	0.028223639	KTI12
ENSG00000085377	792.1289759	1297.580083	-0.711810316	0.010864292	0.028274583	PREP
ENSG00000127054	1607.472518	2335.330529	-0.538748098	0.010871996	0.028291799	INTS11
ENSG00000231359	5.687481958	0	4.843060305	0.010886675	0.028325879	AC072052.1
ENSG00000110723	47.49844872	10.92514996	2.114570698	0.010887273	0.028325879	EXPH5
ENSG00000213300	98.46672458	159.7608754	-0.699117368	0.010911216	0.02838533	HNRNPA3P6
ENSG00000232480	0	5.34094033	-4.999364244	0.010921422	0.028407884	TGFB2-AS1
ENSG00000132600	563.7744517	924.6977697	-0.71421995	0.010922072	0.028407884	PRMT7
ENSG00000148411	2305.526242	1351.052556	0.770777033	0.010942021	0.028456922	NACC2
novel.8	11.85341765	1.161661783	3.411296551	0.010955075	0.02848802	-
ENSG00000185860	0.308355904	11.99720714	-5.206964998	0.010962158	0.028503586	CCDC190
ENSG00000225206	29.75052564	10.54854185	1.48450485	0.010975774	0.028536133	MIR137HG
ENSG00000104888	41.28834988	2.200597239	4.235963021	0.010981856	0.028549089	SLC17A7
ENSG00000144589	826.5372941	539.5724298	0.615581893	0.010987713	0.028561459	STK11IP

ENSG00000137959	3141.53916	1425.443706	1.139996063	0.011000243	0.028591168	IFI44L
ENSG00000263753	1029.068959	598.5031035	0.781374213	0.011021877	0.028644532	LINC00667
ENSG00000187676	162.9145053	281.9571151	-0.792339834	0.011026019	0.02865243	B3GLCT
ENSG00000007520	1062.049108	1548.105016	-0.543868039	0.01102764	0.028653776	TSR3
ENSG00000103187	5500.952802	7995.499854	-0.539529147	0.01103596	0.028672528	COTL1
ENSG00000226950	309.7214788	547.5416427	-0.822524625	0.011038712	0.028676809	DANCR
ENSG00000198780	28.07055932	6.776552991	2.046640899	0.011039824	0.028676832	FAM169A
ENSG00000198689	529.4083324	357.8566704	0.564611101	0.011065246	0.028739994	SLC9A6
ENSG00000273448	19.62639423	4.606637334	2.084051935	0.011067871	0.028743939	AC006480.2
ENSG00000059769	195.3337883	342.9073668	-0.811719774	0.011086211	0.028787262	DNAJC25
ENSG00000248668	18.73649995	4.545274171	2.027314298	0.011086769	0.028787262	OXCT1-AS1
ENSG00000207725	0.586481695	8.85557978	-3.889632824	0.011089009	0.0287902	MIR222
ENSG00000198633	15.36693722	0.672939833	4.465699247	0.011094289	0.028801032	ZNF534
ENSG00000259345	2.080408748	13.79740276	-2.71596212	0.011105113	0.02882625	AC013652.1
ENSG00000229921	7.687514293	0	5.275050476	0.011134077	0.028898546	KIF25-AS1
ENSG00000181798	5.469816391	21.74879856	-1.997456622	0.011158221	0.028956545	LINC00471
ENSG00000179914	6.102896903	0	4.942307294	0.011158652	0.028956545	ITLN1
ENSG00000197301	67.90292048	232.442367	-1.774580409	0.011169284	0.028979858	HMGA2-AS1
ENSG00000176018	460.9621237	761.3578524	-0.723642604	0.011169906	0.028979858	LYSMD3
ENSG00000151779	823.2672964	1330.612193	-0.692408898	0.011170982	0.028979858	NBAS
ENSG00000254786	6.090360416	0	4.939589107	0.011187129	0.029018849	AP001636.1
ENSG00000230330	29.19928865	73.44658336	-1.334088607	0.01119948	0.029047988	HMGN2P3
ENSG00000259341	9.367623151	0.774441189	3.669813892	0.011200706	0.029048268	AC015660.1
ENSG00000196110	29.01031481	58.62525838	-1.013825961	0.011204066	0.02905408	ZNF699
ENSG00000123500	26.36752843	572.9182401	-4.441706799	0.011213522	0.029075699	COL10A1
ENSG00000177425	250.27976	1013.852677	-2.018495508	0.011221851	0.029094392	PAWR
ENSG00000104853	1796.365363	2589.107414	-0.527344803	0.011234711	0.029124827	CLPTM1
ENSG00000150594	43.32495219	7.203911778	2.588455812	0.01124392	0.029145794	ADRA2A
ENSG00000127314	1496.210179	2535.052964	-0.760855393	0.011247272	0.029151573	RAP1B
ENSG00000185127	529.8757438	846.9602135	-0.677042025	0.011255451	0.029169862	C6orf120
ENSG00000255819	7.632068663	0	5.265155345	0.011258126	0.029173886	KLRC4-KLRK1
novel.224	6.939852846	0	5.128955832	0.011296346	0.029267088	-
ENSG00000258847	6.939852846	0	5.128955832	0.011296346	0.029267088	AL391261.2
ENSG00000111291	0.616711807	12.40449683	-4.34762957	0.0113038	0.02928348	GPRC5D
ENSG00000100583	7.256984291	23.23747365	-1.683050066	0.011314192	0.029307481	SAMD15
ENSG00000258024	7.110848325	0	5.165674427	0.011320539	0.029320113	OR5BT1P
ENSG00000164120	9.52260593	0.387220594	4.621949893	0.011321326	0.029320113	HPGD
ENSG00000142609	17.82418219	0.387220594	5.527255882	0.011329176	0.029337518	CFAP74
ENSG00000257303	66.49313044	29.08463649	1.197848887	0.011351434	0.029392228	AC073896.2
ENSG00000154274	16.18906285	2.231278821	2.881682793	0.011356201	0.029401641	C4orf19
ENSG00000125398	564.9133042	126.5779602	2.158110447	0.011360402	0.029409586	SOX9
ENSG00000120318	408.4095749	812.0003233	-0.99108309	0.011371816	0.029436202	ARAP3
ENSG00000281333	18.58534939	4.62670643	2.002749618	0.011378178	0.029449734	AC024941.2
ENSG00000144659	483.5765577	709.1705197	-0.552298284	0.01138446	0.029463059	SLC25A38
ENSG00000263745	0.293240848	6.54158443	-4.326626493	0.011390751	0.029476403	AP005230.1
ENSG00000181017	6.894436407	0	5.120091266	0.011401799	0.029502056	OR56B2P
ENSG00000100445	218.9663729	119.5661003	0.870693501	0.011406907	0.029512333	SDR39U1
ENSG00000261349	10.38846707	0.774441189	3.813157748	0.011437465	0.029588446	AL031432.2
ENSG00000154027	1169.767427	614.8268544	0.928257891	0.011439669	0.029591204	AK5
ENSG00000182310	168.6623484	322.6652446	-0.934858407	0.011463804	0.029650681	SPACA6
ENSG00000167136	152.8500509	236.8288522	-0.631045602	0.011475065	0.029676852	ENDOG

novel.295	9.397853263	0.774441189	3.673439027	0.011499503	0.029737094	-
ENSG00000124104	581.039875	403.7759291	0.524952373	0.011501677	0.029737771	SNX21
ENSG00000104388	2623.10583	3710.399829	-0.500282338	0.011502054	0.029737771	RAB2A
ENSG00000108298	14018.02012	20147.63831	-0.523331909	0.011504006	0.02973986	RPL19
ENSG00000157429	56.29406449	26.75082883	1.070811635	0.011514144	0.029763106	ZNF19
ENSG00000173597	13.28054497	93.32128408	-2.813398361	0.011521213	0.029778415	SULT1B1
ENSG00000164253	668.5602282	1102.18114	-0.721573213	0.011523608	0.029781644	WDR41
ENSG00000274105	16.32857456	2.10970837	2.941773069	0.011525611	0.029783857	AC084824.3
novel.917	28.53597802	8.539050564	1.755971584	0.011529765	0.029791627	-
ENSG00000243970	272.7321767	133.4335525	1.032174835	0.011536101	0.029805037	PPIEL
ENSG00000273769	10.93934805	0.387220594	4.825087418	0.011549032	0.029835477	U52112.1
ENSG00000103197	1463.02878	2138.161225	-0.547261225	0.01155082	0.02983713	TSC2
ENSG00000199697	0	4.94310725	-4.889427006	0.011564556	0.029869643	RNU6-446P
ENSG00000171388	101.0023097	260.7857783	-1.367746357	0.011573779	0.029890493	APLN
ENSG00000182150	507.0999886	309.2247317	0.713338514	0.011595489	0.029943583	ERCC6L2
novel.589	11.50347753	2.191140629	2.407079063	0.011600664	0.029953971	-
ENSG00000218175	160.0761921	260.4595014	-0.702941307	0.011609827	0.02997465	AC016739.1
ENSG00000131067	809.7191686	550.2703981	0.557415103	0.011614365	0.029983388	GGT7
novel.57	35.64500323	4.851062506	2.866843239	0.011615703	0.029983863	-
ENSG00000271631	9.103358767	0.713078025	3.666430847	0.011618704	0.029988629	AL139041.1
novel.1071	11.0239836	0.387220594	4.833723139	0.011635943	0.03003014	-
ENSG00000120742	2478.205486	4228.417649	-0.770840194	0.011648683	0.030060036	SERP1
ENSG00000066926	315.3781887	545.2569974	-0.790474573	0.011654617	0.03007236	FECH
ENSG00000272841	104.5006704	56.08335811	0.897966985	0.011676583	0.030126049	AL139393.3
ENSG00000134318	850.2166476	1236.251146	-0.540296889	0.01169121	0.030160791	ROCK2
ENSG00000111737	1118.964685	1640.239479	-0.551924857	0.011693697	0.030164211	RAB35
ENSG00000055957	11.68242218	0	5.879423342	0.011728086	0.030249917	ITIH1
ENSG00000260910	19.46123971	60.10447686	-1.624268145	0.011740497	0.030278922	LINC00565
ENSG00000183570	94.47439526	173.6342693	-0.876593242	0.011765198	0.030339614	PCBP3
ENSG00000266472	1175.942802	1672.876103	-0.508530441	0.011768058	0.030343977	MRPS21
ENSG00000232021	13.47327975	1.4981317	3.198212773	0.011778113	0.03036689	LEF1-AS1
ENSG00000255145	14.1464061	2.261960402	2.67915973	0.011780869	0.030370981	STX17-AS1
ENSG00000256139	15.40504523	3.475657132	2.12611625	0.01180519	0.030430662	AC007637.1
ENSG00000101134	93.27169996	19.94731867	2.219214207	0.011809673	0.030439199	DOK5
ENSG00000105699	202.4891548	124.2676893	0.704864544	0.011841826	0.030519043	LSR
ENSG00000161544	907.3600579	1821.871118	-1.005763886	0.011844331	0.030522473	CYGB
novel.926	12.01585105	0	5.919969953	0.011848731	0.030530784	-
ENSG00000100815	774.6119679	1113.455085	-0.523301292	0.011849958	0.030530917	TRIP11
ENSG00000221930	62.42239037	132.6736106	-1.088250427	0.011861738	0.030558236	FAM45BP
ENSG00000162419	270.0484034	384.6326586	-0.509908999	0.011881252	0.030605417	GMEB1
ENSG00000130812	48.41840273	12.13512227	2.003396588	0.011882408	0.030605417	ANGPTL6
ENSG00000285918	6.936020629	0.356539013	4.166754057	0.011888265	0.030617468	AC092376.3
ENSG00000099984	10.09773352	31.5095898	-1.644199543	0.011897641	0.030638578	GSTT2
ENSG00000268849	5.440839927	0	4.777789391	0.011910972	0.030669867	SIGLEC22P
ENSG00000257398	5.434571684	0	4.776296779	0.011925415	0.030704015	AC126177.3
novel.943	3.992329314	39.34044263	-3.304221441	0.011949575	0.03076317	-
ENSG00000186280	19.03106572	50.44647124	-1.40834043	0.01196205	0.030792234	KDM4D
ENSG00000115355	973.1562745	1442.394829	-0.567914452	0.011967708	0.030803748	CCDC88A
ENSG00000187118	252.9770394	392.5453292	-0.633288051	0.011972172	0.030812185	CMC1
ENSG00000262050	13.61286274	2.782648203	2.267334688	0.011974605	0.030815392	AC005696.1
ENSG00000131386	279.3530308	647.3046318	-1.21261016	0.011975964	0.030815839	GALNT15

ENSG00000139890	26.54855345	7.947671385	1.743549578	0.01198321	0.03083143	REM2
ENSG00000151952	1.525410814	10.21207193	-2.753674996	0.01199348	0.030854799	TMEM132D
ENSG00000105373	6243.924127	4011.47138	0.638350165	0.012006305	0.030884734	NOP53
ENSG00000154736	141.7646656	48.60112874	1.54267174	0.012013983	0.030901426	ADAMTS5
ENSG00000284634	11.79922567	2.51699806	2.230539344	0.012022315	0.030919794	AC092821.3
ENSG00000180998	82.80160318	39.32867427	1.072350663	0.012024075	0.030921259	GPR137C
ENSG00000161010	88.2068424	40.01325407	1.141550954	0.012039978	0.030959092	MRNIP
ENSG00000088888	2381.031531	3489.624243	-0.55142012	0.012046563	0.03097296	MAVS
ENSG00000164591	115.1287844	19.66707484	2.552643864	0.012052058	0.030984021	MYOZ3
ENSG00000135525	18.22057894	4.923038154	1.874164711	0.012053851	0.030985566	MAP7
ENSG00000129194	63.17721806	29.9618633	1.078733782	0.012057527	0.030991947	SOX15
ENSG00000147457	759.2613125	1113.701497	-0.552865044	0.012068844	0.031017969	CHMP7
ENSG00000066027	624.7507244	416.2493192	0.585293953	0.012093915	0.031079329	PPP2R5A
ENSG00000279953	0.908699006	10.38567733	-3.520766318	0.012103435	0.031100716	AC117503.3
ENSG00000140943	2113.907738	3104.250313	-0.554277402	0.012109445	0.031112167	MBTPS1
ENSG00000139637	582.4328691	849.8391451	-0.545195924	0.012110286	0.031112167	C12orf10
ENSG00000141349	1145.365748	1781.945405	-0.637713755	0.012114995	0.031121188	G6PC3
ENSG00000186184	1144.169395	1581.102018	-0.466507451	0.012123579	0.031140159	POLR1D
ENSG00000187682	5.983230103	0	4.916061822	0.012138454	0.031175284	ERAS
ENSG00000157823	424.219092	274.3507811	0.6297689	0.01214439	0.031187446	AP3S2
novel.29	13.80559752	1.803920035	2.93821279	0.012150463	0.031197191	-
ENSG00000230606	68.19251475	15.17524828	2.165580343	0.012150586	0.031197191	AC092683.1
ENSG00000103512	82.81206	266.3708183	-1.686148286	0.012161647	0.031222505	NOMO1
ENSG00000178795	5.129976726	0	4.693202363	0.012165604	0.031229577	GDPD4
ENSG00000286705	15.74363125	3.973835692	1.99041709	0.012170659	0.031239468	AC079140.6
novel.33	15.58746609	2.567748738	2.616845576	0.012174397	0.031243762	-
ENSG00000125744	79.68029179	220.6568147	-1.469244346	0.012174737	0.031243762	RTN2
ENSG00000287385	32.45119367	7.060987961	2.213177923	0.012193826	0.031289657	AL121820.3
ENSG00000189367	18.12104179	3.068367442	2.533083543	0.012200785	0.031304423	KIAA0408
ENSG00000198538	254.8200802	163.2508222	0.641236485	0.012208331	0.031320691	ZNF28
ENSG00000207342	5.49377826	0	4.790434154	0.012215571	0.031336172	RF00019
ENSG00000196503	5.730319829	26.25868643	-2.199270481	0.012230264	0.031369405	ARL9
ENSG00000175741	45.954917	89.06895555	-0.956650997	0.012230941	0.031369405	RWDD4P2
ENSG00000250041	1.214547612	11.88239637	-3.287324291	0.012257826	0.031435255	AC069360.1
ENSG00000259732	13.44151126	1.722487775	2.936711192	0.012262422	0.03144394	AC092757.2
ENSG00000181924	820.8828625	1277.078667	-0.637782243	0.012265001	0.031447448	COA4
ENSG00000002822	10.30655227	28.45183484	-1.461482144	0.012284978	0.03149556	MAD1L1
ENSG00000099625	192.2472947	357.4572023	-0.894000655	0.012290603	0.031506874	CBARP
ENSG00000111432	42.64240921	3.364699167	3.645608516	0.012307648	0.031547455	FZD10
ENSG00000274682	9.99916528	0	5.656926128	0.012309425	0.031548898	AC015540.1
novel.201	22.68975234	7.4506487	1.600071441	0.012336944	0.03161631	-
ENSG00000090487	1132.935322	1734.200227	-0.614080619	0.012339125	0.031618779	SPG21
ENSG00000155099	531.6128371	324.3457148	0.713516273	0.012353933	0.031653604	PIP4P2
ENSG00000212663	6.856684402	0	5.11265472	0.012397591	0.031760912	AC003035.1
ENSG00000139726	958.4970527	1507.302247	-0.653295421	0.012398259	0.031760912	DENR
ENSG00000205930	5.484931447	0	4.788326129	0.012413489	0.031796543	C21orf62-AS1
ENSG00000159251	0.923814062	11.05031643	-3.597938286	0.012414616	0.031796543	ACTC1
ENSG00000266507	0.308355904	6.991324063	-4.420597869	0.012419247	0.031805269	MIR4479
ENSG00000143079	1314.347494	849.6201226	0.629371412	0.012426458	0.0318206	CTTNBP2NL
ENSG00000171858	3114.962962	4799.874908	-0.623802837	0.012446312	0.0318683	RPS21
ENSG00000130940	33.75825474	5.369310161	2.650391234	0.012449207	0.031872571	CASZ1

ENSG00000103196	880.756527	363.2635034	1.277856918	0.012451959	0.031876475	CRISPLD2
ENSG00000102178	718.853555	979.4962477	-0.446337432	0.012457668	0.031887949	UBL4A
ENSG00000181585	7.973233248	0.356539013	4.365463261	0.012467867	0.03191091	TMIE
ENSG00000151665	203.2364605	295.5029006	-0.540598906	0.012469358	0.031911584	PIGF
ENSG00000279296	24.94903446	7.338534859	1.750741824	0.012510252	0.032013085	PRAL
ENSG00000119574	256.3667752	406.3334978	-0.664578797	0.012518521	0.032031092	ZBTB45
novel.247	52.94896148	170.6670177	-1.689222369	0.012523113	0.032039685	-
ENSG00000143543	276.4403094	155.7305568	0.826574425	0.012531667	0.032058413	JTB
ENSG00000119661	441.9038344	287.1781888	0.6220864	0.012537084	0.032069112	DNAL1
ENSG00000033030	671.8074903	466.749448	0.525421982	0.012540201	0.032073928	ZCCHC8
ENSG00000005961	25.00287044	5.481424002	2.197700333	0.012548243	0.032091337	ITGA2B
ENSG00000174740	68.69398927	21.88342164	1.642958257	0.012558629	0.032113607	PABPC5
ENSG00000206535	28.33593481	60.60625144	-1.098964059	0.012559423	0.032113607	LNP1
ENSG00000265996	6.665488001	0.387220594	4.108520432	0.01257088	0.03213974	MIR3671
ENSG00000135925	7.069548837	0	5.154063013	0.012572786	0.032141449	WNT10A
ENSG00000227069	5.817249219	0	4.872715008	0.012579442	0.032155302	CNN2P2
ENSG00000285865	5.174068246	0	4.704237839	0.01259034	0.032179992	AC010285.3
ENSG00000274423	14.99840583	40.58824139	-1.431439096	0.012596341	0.032192163	SEC22B2
ENSG00000168906	2525.705439	3618.555245	-0.518808119	0.012605761	0.032210571	MAT2A
ENSG00000264727	7.057012349	0	5.151656934	0.012606023	0.032210571	AC005725.1
ENSG00000063245	2427.873818	3519.115242	-0.53548063	0.012613405	0.032226264	EPN1
ENSG00000091106	79.00680978	23.46961688	1.757747262	0.012625149	0.032253097	NLRC4
ENSG00000100288	40.3030792	10.63930232	1.929650399	0.012629701	0.032261554	CHKB
novel.359	5.39424111	0	4.76658582	0.012644045	0.032293697	-
ENSG00000235997	6.440229271	0	5.019551647	0.012645893	0.032293697	LINC01936
ENSG00000182158	2580.609303	4229.932117	-0.712844909	0.012646013	0.032293697	CREB3L2
ENSG00000227992	8.385856162	27.97488126	-1.731463311	0.012664093	0.032336688	AC108463.1
ENSG00000181856	28.34220305	6.584034372	2.113370778	0.012682612	0.032380791	SLC2A4
ENSG00000237840	23.77077175	7.836713419	1.597787577	0.012689358	0.032393318	FAM21FP
ENSG00000213020	167.8980335	87.82085283	0.936587101	0.012690011	0.032393318	ZNF611
ENSG00000174876	5.128723077	0	4.69287938	0.012699336	0.03241075	AMY1B
ENSG00000258443	5.128723077	0	4.69287938	0.012699336	0.03241075	AC005225.1
ENSG00000169288	299.8743186	516.8035744	-0.785879013	0.012718388	0.032456188	MRPL1
ENSG00000143622	843.4797461	507.5068812	0.733251204	0.012722569	0.032463668	RIT1
ENSG00000101972	1829.096021	1272.238294	0.523896727	0.012737876	0.032499534	STAG2
ENSG00000136933	407.8203127	682.634008	-0.743659026	0.012744083	0.032512177	RABEPK
ENSG00000284969	0.600343103	7.154316975	-3.567228902	0.012755868	0.032539049	AL049629.2
ENSG00000287550	6.632750591	0	5.063745058	0.012758631	0.032542902	AL450345.2
ENSG00000198734	6.752417392	0	5.087885501	0.012765792	0.03255797	F5
ENSG00000235649	8.58360554	0.387220594	4.472165444	0.012777493	0.032584614	MXRA5Y
ENSG00000221986	9.08949736	1.029478846	3.099909945	0.012786335	0.032603963	MYBPHL
novel.558	24.3669979	96.19893108	-1.979911514	0.012789964	0.032610016	-
ENSG00000273226	37.33474183	16.36759164	1.184625673	0.012805968	0.032647616	AL391834.2
ENSG00000138326	9884.272524	15989.7683	-0.693948517	0.012813998	0.032664884	RPS24
ENSG00000111325	8.11908448	23.21496441	-1.513494475	0.012818099	0.032672132	OGFOD2
ENSG00000226239	5.726558882	0	4.851966982	0.012823097	0.032681665	AL031658.1
ENSG00000270000	9.343732554	0.723690511	3.699521126	0.012830553	0.032697458	AC005479.2
ENSG00000138750	542.9287807	783.3527244	-0.528936526	0.012844294	0.032727266	NUP54
ENSG00000174748	16371.69437	23306.85023	-0.509552803	0.012844768	0.032727266	RPL15
ENSG00000182628	1247.879168	781.11332	0.67563685	0.012846206	0.032727718	SKA2
ENSG00000164070	347.4998562	142.1429161	1.288174392	0.012849949	0.032732639	HSPA4L

ENSG00000097033	1919.685786	2631.795226	-0.455086667	0.012850657	0.032732639	SH3GLB1
ENSG00000134363	236.5551952	1022.245409	-2.111667303	0.012853134	0.032735738	FST
ENSG00000126775	839.808339	458.3895126	0.873989298	0.012855314	0.032738082	ATG14
ENSG00000165078	5.712697475	0	4.848774568	0.012860856	0.032748985	CPA6
novel.476	37.76692493	131.3990775	-1.797754538	0.012868247	0.032764594	-
ENSG00000127081	169.1175527	93.94124303	0.847337598	0.012877904	0.03278597	ZNF484
ENSG00000085998	1618.218273	2263.596463	-0.484171575	0.012881945	0.032793044	POMGNT1
ENSG00000128590	350.7964504	722.9153955	-1.043117027	0.012892941	0.032814899	DNAJB9
ENSG00000280435	155.0690462	56.57203288	1.456845642	0.012893056	0.032814899	AC006058.4
ENSG00000077150	1178.038912	1971.388093	-0.742626248	0.012897185	0.032822193	NFKB2
ENSG00000173585	12.94593336	0	6.027600119	0.012902126	0.032831552	CCR9
ENSG00000279744	8.503015665	26.95139139	-1.662783028	0.012911812	0.032847821	AC132938.5
ENSG00000236751	9.764162094	0.774441189	3.724562143	0.01291222	0.032847821	LINC01186
ENSG00000241269	12.98605047	2.822786395	2.188984955	0.012912313	0.032847821	AC093620.1
ENSG00000244731	309.1048908	10.15430479	4.924702493	0.012935647	0.032903961	C4A
ENSG00000146826	489.9087593	324.6385918	0.593074064	0.012938705	0.032908516	MAP11
ENSG00000243175	9.350000797	1.130980201	3.092710389	0.012951872	0.03293878	RPSAP36
ENSG00000070601	11.06947131	0	5.801634423	0.012977823	0.033001549	FRMPD1
ENSG00000159871	31.00400763	9.579270291	1.688711607	0.012990398	0.033030293	LYPD5
ENSG00000166548	784.2918779	549.870814	0.511902157	0.013001288	0.033054747	TK2
ENSG00000197182	73.14948689	157.1447689	-1.102166656	0.013028068	0.033119592	MIRLET7BHG
ENSG00000285813	37.42897965	16.85631359	1.151334088	0.013036254	0.033137161	AP000813.1
novel.283	21.84283848	5.584081233	1.986667894	0.013044153	0.033153995	-
ENSG00000105372	12742.67995	20288.29308	-0.670984419	0.013055395	0.033179324	RPS19
ENSG00000187775	97.37009192	50.49940527	0.945263703	0.013059524	0.033186569	DNAH17
ENSG00000059378	887.934894	601.5596086	0.561296045	0.013069583	0.033208883	PARP12
ENSG00000111011	1741.081046	1196.222879	0.541400578	0.013088663	0.033254113	RSRC2
ENSG00000256812	7.740167891	0.387220594	4.326570194	0.013123124	0.033338408	CAPNS2
ENSG00000286207	75.94711351	26.91497762	1.494578355	0.013138084	0.033373149	AC012443.2
ENSG00000180357	768.3089565	1227.413123	-0.675607774	0.013159817	0.033420604	ZNF609
ENSG00000110497	444.7644713	673.4227967	-0.597986029	0.013160163	0.033420604	AMBRA1
ENSG00000211899	6.749838823	0	5.087376316	0.013160625	0.033420604	IGHM
ENSG00000116329	13.40655128	2.578361223	2.396144591	0.013167796	0.033435546	OPRD1
ENSG00000185897	6.849091238	0	5.11111885	0.013176734	0.033454971	FFAR3
ENSG00000130165	686.0715816	1014.048031	-0.563934214	0.013185909	0.033474996	ELOF1
ENSG00000119684	496.44032	346.6556434	0.518565166	0.01319493	0.033494625	MLH3
novel.88	5.705104311	0	4.847006361	0.013198624	0.033499961	-
ENSG00000226864	18.71414774	3.220619475	2.535839219	0.013199611	0.033499961	ATE1-AS1
ENSG00000279933	5.698836068	0	4.845564422	0.013216496	0.033537774	AL031595.1
ENSG00000197444	3.66251884	17.10317891	-2.232770498	0.013217092	0.033537774	OGDHL
ENSG00000147234	5.462294498	0	4.782937129	0.013220285	0.033542601	FRMPD3
ENSG00000254528	5.175321895	0	4.704567804	0.013225974	0.033553756	AP000757.1
ENSG00000172476	54.90558682	27.46377846	1.003226729	0.013230789	0.033562695	RAB40A
ENSG00000104213	313.3214689	77.93049437	2.006323644	0.013275117	0.033671765	PDGFRL
ENSG00000121075	5.156445892	0	4.699839838	0.013276377	0.033671765	TBX4
ENSG00000107859	0	4.932494765	-4.886635567	0.013305027	0.033741132	PITX3
ENSG00000137168	351.2307421	740.8053013	-1.077093133	0.013327631	0.033791332	PPIL1
ENSG00000107438	695.7498476	2374.581082	-1.771067335	0.013328407	0.033791332	PDLIM1
ENSG00000072849	697.9209535	971.7808948	-0.47780934	0.013328724	0.033791332	DERL2
ENSG00000115239	223.0743939	374.6751107	-0.74789806	0.01333808	0.033811752	ASB3
ENSG00000265724	0.293240848	6.257021066	-4.259624018	0.013343762	0.033822855	MIR4284

ENSG00000173838	29.10609101	12.24967626	1.250571354	0.013348267	0.033830976	10-Mar
ENSG00000179362	13.44025761	88.51278742	-2.716866332	0.013355993	0.033847255	HMGN2P46
ENSG00000233261	5.11486167	0	4.689372385	0.013387803	0.033917943	FAM238A
ENSG00000279907	5.11486167	0	4.689372385	0.013387803	0.033917943	AC018695.7
ENSG00000226040	5.11486167	0	4.689372385	0.013387803	0.033917943	AC005740.1
ENSG00000232712	14.43212506	3.240688571	2.158751561	0.013391892	0.033924995	KIZ-AS1
ENSG00000225166	0	5.574753017	-5.064112218	0.013397869	0.033936827	AC012462.1
ENSG00000164329	961.8473149	667.5531375	0.526955481	0.013408122	0.033959486	TENT2
ENSG00000138190	214.2135179	75.55539264	1.501036865	0.013452209	0.034067828	EXOC6
ENSG00000204583	15.35622385	1.00940975	3.883371185	0.01346025	0.034084461	LRCOL1
ENSG00000249846	23.64261414	7.440036214	1.660684101	0.013461401	0.034084461	LINC02021
ENSG00000168904	268.6136829	173.4126574	0.63218562	0.013478989	0.034125669	LRRC28
ENSG00000279330	12.66508681	2.578361223	2.317149396	0.01349078	0.034152193	AJ003147.3
ENSG00000161996	497.289899	784.3382122	-0.657151806	0.013497713	0.034166416	WDR90
ENSG00000185838	128.4195873	226.0232309	-0.8157835	0.013514104	0.034204573	GNB1L
ENSG00000159228	1642.689794	1114.595432	0.559268493	0.013524745	0.034228171	CBR1
ENSG00000186862	49.71131766	20.06619219	1.31540281	0.013551315	0.03429176	PDZD7
ENSG00000246695	409.3571371	220.3538986	0.894020168	0.013552511	0.03429176	RASSF8-AS1
ENSG00000068354	272.8246467	426.1964176	-0.642772581	0.013571874	0.034337409	TBC1D25
ENSG00000260922	0	9.343017459	-5.80286322	0.013574903	0.03434173	AC009139.1
ENSG00000128203	50.28332503	103.5790512	-1.042741709	0.013579879	0.034349756	ASPHD2
ENSG00000173250	6.347031636	0.356539013	4.038373195	0.01358072	0.034349756	GPR151
ENSG00000104899	81.24264385	15.95328549	2.343918713	0.013583854	0.034354338	AMH
ENSG00000168883	727.0683719	1052.555914	-0.533967304	0.013585805	0.034355927	USP39
ENSG00000134873	20.79595268	2.91483114	2.841610943	0.013608475	0.034409168	CLDN10
ENSG00000163751	7.459534802	0.356539013	4.27310474	0.013609507	0.034409168	CPA3
ENSG00000224963	6.362146692	0.336469917	4.041320594	0.01361511	0.034419984	U82695.1
ENSG00000214194	682.2139635	930.1876444	-0.447454075	0.013627177	0.034447139	SMIM30
ENSG00000005379	308.9174907	70.48090751	2.133753259	0.013662182	0.034532266	TSPOAP1
ENSG00000141956	281.7814422	184.8164304	0.608005367	0.013691607	0.034603273	PRDM15
ENSG00000060140	3.380632102	23.25368994	-2.785316619	0.013704258	0.034631877	STYK1
ENSG00000271978	24.07439779	6.258305336	1.927150783	0.013718031	0.034661022	AL359643.2
ENSG00000284664	15.53327411	1.885352294	3.0741612	0.013718459	0.034661022	AL161756.3
ENSG00000197601	2342.754458	1523.636622	0.620608234	0.013722948	0.034668991	FAR1
ENSG00000115207	619.7002139	919.99089	-0.56979191	0.013740068	0.034708869	GTF3C2
ENSG00000266456	0.307102255	5.788368213	-4.150608921	0.013747264	0.034723669	AP001178.3
ENSG00000225663	1925.422434	1319.451552	0.545406446	0.01375481	0.034739353	MCRIP1
ENSG00000212694	243.8158699	143.8347226	0.76083186	0.013761586	0.034753086	LINC01089
ENSG00000169718	1201.044486	1845.088886	-0.619337016	0.013776891	0.034788356	DUS1L
ENSG00000233297	15.50207509	2.894762044	2.426083441	0.013814261	0.034879331	RASA4DP
ENSG00000166888	3110.558359	4154.480943	-0.417462305	0.013837191	0.03493383	STAT6
ENSG00000213523	868.7379611	1268.346431	-0.545772725	0.013845235	0.034950743	SRA1
ENSG00000204257	155.5806372	0	9.615097373	0.013862221	0.034990222	HLA-DMA
ENSG00000187608	3152.661762	6135.891071	-0.960706631	0.013865573	0.034995284	ISG15
ENSG00000129467	5.189183302	24.937067	-2.263483627	0.013874735	0.035015007	ADCY4
novel.607	318.2442959	188.730444	0.752428929	0.013887323	0.035043371	-
ENSG00000105393	393.1534417	568.0469835	-0.530648926	0.013890852	0.035048872	BABAM1
ENSG00000165209	135.4191449	79.68220388	0.763449146	0.013900507	0.035069827	STRBP
ENSG00000111877	220.9585986	118.2438858	0.899778095	0.01390558	0.035079218	MCM9
ENSG00000232956	287.1993601	473.6398786	-0.721929231	0.013910917	0.035089275	SNHG15
ENSG00000128463	1262.222111	1699.485968	-0.429149763	0.013920994	0.035111284	EMC4

ENSG00000144306	433.0805386	286.2629256	0.596256313	0.013931431	0.035134198	SCRN3
ENSG00000070770	398.4353954	549.9443651	-0.464696273	0.013934648	0.0351389	CSNK2A2
ENSG00000124205	154.7069082	0	9.606944798	0.013943112	0.035156832	EDN3
ENSG00000182518	272.0835382	161.3125831	0.752499817	0.013972402	0.035224804	FAM104B
ENSG00000214562	48.74613283	19.66964338	1.308123544	0.013972781	0.035224804	NUTM2D
ENSG00000163322	110.7225934	178.8038345	-0.692159584	0.013976803	0.035231523	ABRAXAS1
ENSG00000165416	1034.396543	1457.126949	-0.494448626	0.013992599	0.03526792	SUGT1
ENSG00000076928	2062.788588	3012.618643	-0.546339407	0.014014118	0.035318732	ARHGEF1
ENSG00000082213	298.3590514	427.7706814	-0.520347862	0.014054794	0.035416467	C5orf22
ENSG00000243364	54.42171904	138.1921379	-1.344627905	0.014055625	0.035416467	EFNA4
ENSG00000134545	15.69230257	0.723690511	4.444072878	0.014060956	0.035426464	KLRC1
ENSG00000188735	304.3129056	479.0632187	-0.654543536	0.014069513	0.035444586	TMEM120B
ENSG00000211450	899.1351996	1312.152899	-0.545339704	0.014076437	0.035458591	SELENOH
ENSG00000196968	571.2924651	993.2744076	-0.797837015	0.014080655	0.035464137	FUT11
novel.868	19.44870323	3.180481283	2.606118972	0.014081369	0.035464137	-
ENSG00000117479	186.7235711	301.562266	-0.691052078	0.014099234	0.03550569	SLC19A2
ENSG00000126903	447.941868	741.5539627	-0.727525007	0.014103376	0.035512677	SLC10A3
ENSG00000197006	52.96289451	140.9741913	-1.411808246	0.0141051	0.035513577	METTL9
novel.15	0	4.73882027	-4.822906985	0.014110727	0.035524303	-
ENSG00000126246	34.46118393	9.357354359	1.868357989	0.014116354	0.035535024	IGFLR1
ENSG00000140481	0.615458159	9.297147071	-3.936664132	0.014120354	0.03554165	CCDC33
ENSG00000125675	27.33905276	86.83441904	-1.665923443	0.014155968	0.035627842	GRIA3
ENSG00000173926	160.9873981	59.58666156	1.430617096	0.014175113	0.035672571	3-Mar
ENSG00000047648	176.3992817	75.33090817	1.224617928	0.014189398	0.035705061	ARHGAP6
ENSG00000224389	173.1742714	2.355289417	6.191153718	0.014196144	0.035718577	C4B
ENSG00000144597	220.6804725	330.535255	-0.582917031	0.014207185	0.035742894	EAF1
ENSG00000206199	11.86477176	2.140389951	2.465342054	0.014209246	0.03574462	ANKUB1
novel.53	6.691885896	0	5.075722474	0.014219127	0.035766013	-
novel.256	10.77992014	0	5.763321129	0.01422234	0.035770632	-
ENSG00000196670	441.826906	292.4538316	0.594608748	0.014241654	0.035815739	ZFP62
ENSG00000237950	34.85758068	11.26979221	1.634866177	0.014250333	0.035834099	AL357079.1
ENSG00000112130	282.0874612	432.8295329	-0.617446709	0.014255025	0.035842427	RNF8
ENSG00000132196	214.3440402	114.1587069	0.907603835	0.014257671	0.035845612	HSD17B7
ENSG00000178184	340.0238818	141.8185872	1.26299233	0.014267999	0.035866431	PARD6G
ENSG00000232112	1008.464514	641.4391046	0.652450282	0.014268713	0.035866431	TMA7
ENSG00000070269	407.9863374	274.5333764	0.571482689	0.014279071	0.035888996	TMEM260
ENSG00000105887	2336.819795	3781.107052	-0.694295262	0.014312147	0.035966052	MTPN
ENSG00000273702	31.70395915	9.822411193	1.69494844	0.014312498	0.035966052	AC091271.1
ENSG00000223548	7.112101973	0	5.165735999	0.014319526	0.035980233	AC034228.1
ENSG00000070388	28.42981663	4.790855218	2.552030542	0.014326858	0.035995174	FGF22
ENSG00000121289	629.7675042	986.0619293	-0.646884934	0.014329362	0.035997985	CEP89
ENSG00000168092	1625.452549	2275.156234	-0.485050815	0.014343832	0.036030853	PAFAH1B2
ENSG00000224259	1.81990531	100.844084	-5.792617861	0.014355376	0.036056363	LINC01133
ENSG00000170989	162.3395909	43.39267532	1.89994523	0.014371155	0.036092473	S1PR1
ENSG00000111229	2583.160682	3861.854582	-0.580118465	0.014372531	0.036092473	ARPC3
ENSG00000137821	155.1369133	92.68856398	0.742283116	0.014378497	0.036103965	LRRC49
ENSG00000188596	15.77260771	39.3226381	-1.315739544	0.014431182	0.036231217	CFAP54
ENSG00000272438	2.976499995	26.36031618	-3.143775301	0.014431964	0.036231217	AL645608.6
ENSG00000141096	5.132484024	0	4.693794859	0.014437263	0.036241017	DPEP3
ENSG00000170144	5705.496601	8332.254456	-0.546391121	0.01443987	0.036244059	HNRNPA3
ENSG00000197746	35837.57522	21498.49332	0.737251501	0.014443031	0.036248492	PSAP

ENSG00000272240	29.5401685	9.252256984	1.671310153	0.014460009	0.036286236	AC004908.1
ENSG00000221164	1.847628125	11.47523507	-2.646432808	0.014460863	0.036286236	SNORA11F
ENSG00000267454	70.56300069	29.63240985	1.256235005	0.014467772	0.036300067	ZNF582-AS1
ENSG00000186063	821.9934831	1306.100583	-0.668197855	0.014470542	0.036303509	AIDA
ENSG00000160469	963.4290853	406.5555168	1.245328259	0.014487462	0.036342449	BRSK1
ENSG00000250569	21.36187744	5.04345273	2.084863633	0.01449506	0.036355014	NTAN1P2
ENSG00000258365	17.15696843	3.005720009	2.539325793	0.014495269	0.036355014	AC073655.2
ENSG00000175606	345.6126533	503.3976645	-0.543118881	0.014508468	0.036384605	TMEM70
ENSG00000198673	28.74103583	10.92514996	1.385708702	0.014522505	0.036416291	TAFA2
ENSG00000105193	9457.387476	14177.88448	-0.584136659	0.014554492	0.036492943	RPS16
ENSG00000258181	5.186676005	0	4.707461673	0.014555882	0.036492943	AC008083.2
ENSG00000105649	42.23577015	118.7488584	-1.490279088	0.014561958	0.036504655	RAB3A
ENSG00000040731	24.66220405	0.672939833	5.158319508	0.014565139	0.036509106	CDH10
novel.382	35.41222261	7.764609376	2.199805485	0.014573075	0.036525475	-
ENSG00000143190	525.3049414	300.2137234	0.8079433	0.014583021	0.036544561	POU2F1
ENSG00000227438	1.22840902	14.95893615	-3.61054407	0.014583504	0.036544561	AP001471.1
ENSG00000274602	250.7768053	90.08140057	1.47791248	0.014591921	0.036562126	PI4KAP1
novel.323	9.966712605	1.803920035	2.47146256	0.014601634	0.036582936	-
novel.805	19.72969232	47.94111315	-1.283533277	0.014604618	0.036586885	-
ENSG00000224958	5.176575543	0	4.704917085	0.014619051	0.03661951	PGM5-AS1
ENSG00000152439	63.50960675	116.1429449	-0.870911217	0.014637084	0.036646897	ZNF773
ENSG00000159579	513.9205731	752.4565094	-0.550224172	0.0146379	0.036646897	RSPRY1
ENSG00000244137	5.1703073	0	4.703346443	0.014639131	0.036646897	AL512328.1
ENSG00000232876	5.1703073	0	4.703346443	0.014639131	0.036646897	AL353596.1
ENSG00000081842	5.1703073	0	4.703346443	0.014639131	0.036646897	PCDHA6
ENSG00000021574	363.8449424	215.4164892	0.754767881	0.014639338	0.036646897	SPAST
ENSG00000227634	0	4.953719736	-4.891803142	0.014639858	0.036646897	LINC01714
ENSG00000280212	10.42217339	0.672939833	3.898611019	0.014647716	0.036663035	AC012254.5
ENSG00000007866	387.5578199	740.158651	-0.933196193	0.014656375	0.036681175	TEAD3
ENSG00000279342	30.7483766	9.87444614	1.631392257	0.014662722	0.036693525	AP000866.6
ENSG00000187800	489.4126068	1587.139447	-1.697433231	0.014664186	0.036693653	PEAR1
ENSG00000075826	436.6528816	231.7315667	0.915084537	0.014666521	0.036695964	SEC31B
ENSG00000262097	5.14759908	0	4.697611369	0.014680211	0.03672665	LINC02185
ENSG00000178445	255.5567992	69.63574058	1.874263501	0.014681613	0.03672665	GLDC
ENSG00000093000	850.4405261	1314.653341	-0.628528997	0.014690169	0.036744516	NUP50
ENSG00000284095	5.476155906	0	4.786288117	0.014711355	0.036790423	AC055733.4
ENSG00000274976	5.476155906	0	4.786288117	0.014711355	0.036790423	AC087588.2
ENSG00000230138	6.425185486	0	5.016482162	0.014716236	0.036799089	AC119428.2
ENSG00000214654	27.92044859	64.79639926	-1.217134582	0.014733399	0.03683846	B3GNT10
ENSG00000104904	5999.700476	9515.760669	-0.665453779	0.014740372	0.036852348	OAZ1
ENSG00000255931	5.117368968	0	4.689963862	0.014777213	0.036935314	AP003108.1
ENSG00000220773	5.117368968	0	4.689963862	0.014777213	0.036935314	RPSAP44
ENSG00000211454	16.24826942	3.903015919	2.051289208	0.014777822	0.036935314	AKR7L
ENSG00000183153	2.435363469	12.41266917	-2.356140447	0.014780516	0.036937166	GJD3
ENSG00000145217	91.63579733	42.50812807	1.105484698	0.014781406	0.036937166	SLC26A1
ENSG00000223784	0.307102255	5.717548439	-4.135706722	0.014801557	0.036983963	LINP1
ENSG00000126858	790.3464527	545.5672209	0.534639346	0.014810728	0.037003321	RHOT1
ENSG00000177733	2980.933777	4364.416993	-0.550066862	0.014866466	0.037134306	HNRNPA0
ENSG00000142233	38.26991001	12.10444069	1.667695729	0.014866483	0.037134306	NTN5
ENSG00000254997	6.596180964	0.336469917	4.09530324	0.014867444	0.037134306	KRTAP5-9
ENSG00000280046	14.48499212	35.2696819	-1.279336423	0.0148764	0.037149713	AC104581.4

ENSG00000254503	7.014174478	0.387220594	4.181100039	0.014876472	0.037149713	AC010319.1
ENSG00000014824	1199.966726	1643.263256	-0.453494693	0.014936327	0.037295598	SLC30A9
ENSG00000284740	21.26638597	7.083497202	1.577167081	0.0149539	0.03733589	AL645728.2
ENSG00000135926	4011.235316	2670.880328	0.58684828	0.014962956	0.037354911	TMBIM1
ENSG00000186230	72.12050818	120.9692336	-0.745248644	0.014974288	0.037379609	ZNF749
ENSG00000164038	186.5880059	362.0983865	-0.95671093	0.014981266	0.037393436	SLC9B2
ENSG00000043514	300.980241	463.7968719	-0.623184577	0.014998945	0.037433966	TRIT1
ENSG00000253250	37.64692995	76.07385024	-1.013160984	0.015008428	0.037454035	C8orf88
novel.763	71.47642956	145.4997816	-1.024330958	0.015017526	0.03747314	-
ENSG00000162148	91.39417025	42.5763793	1.103998941	0.015037131	0.037518458	PPP1R32
novel.211	5.511400613	0	4.794757472	0.015048718	0.037543763	-
ENSG00000277695	10.15038711	0	5.677789974	0.015087378	0.037636597	AC097478.3
ENSG00000234769	57.49690199	21.61751471	1.413151523	0.015091055	0.037642155	WASH4P
ENSG00000119457	4.851850935	0	4.612018861	0.015099321	0.037659159	SLC46A2
ENSG00000267737	13.28939178	3.189937893	2.049868808	0.015101638	0.037661321	AC087645.2
ENSG00000079841	16.15034193	2.415496705	2.717100581	0.015105195	0.037666579	RIMS1
ENSG00000156234	6.982619446	0.387220594	4.175279605	0.015115402	0.037688414	CXCL13
ENSG00000233547	4.836735879	0	4.608011091	0.015147033	0.037763657	AL158212.2
ENSG00000138018	1339.802482	797.4881381	0.748502796	0.015174718	0.03782905	SELENOI
ENSG00000165091	11.7966471	1.029478846	3.484157718	0.015179977	0.037838527	TMC1
ENSG00000258011	1.186824798	9.86370526	-3.036435484	0.015191068	0.037862542	HMGA1P3
ENSG00000211699	6.392376805	0.356539013	4.047276768	0.015202538	0.037887496	TRGV3
ENSG00000092607	102.5706565	363.7937075	-1.827109585	0.01522498	0.037939413	TBX15
ENSG00000244151	22.57133919	7.867395001	1.519803288	0.015227315	0.037939413	AC010973.2
ENSG00000108641	220.3149904	325.4725039	-0.56216478	0.015227751	0.037939413	B9D1
ENSG00000232499	12.68020186	29.39055321	-1.211792939	0.015249969	0.037987115	AL391058.1
ENSG00000170549	6.392376805	0.336469917	4.047278593	0.015251284	0.037987115	IRX1
ENSG00000260541	6.392376805	0.336469917	4.047278593	0.015251284	0.037987115	AL031722.1
ENSG00000127533	3.315086011	27.09277415	-3.026682914	0.015268042	0.03802521	F2RL3
ENSG00000075213	156.3497647	447.1905398	-1.51643885	0.015277646	0.038045482	SEMA3A
ENSG00000284735	11.72615769	1.844058227	2.689903575	0.015285645	0.038061753	AL139424.3
ENSG00000176563	47.24830264	18.31302283	1.375210536	0.015306946	0.038111141	CNTD1
ENSG00000173227	55.22342993	178.6374211	-1.694763168	0.015309946	0.038114958	SYT12
ENSG00000196371	210.1073624	139.4618508	0.591421447	0.015320234	0.038136918	FUT4
ENSG00000254777	7.479664453	0.336469917	4.276494532	0.015321813	0.038137193	AC022182.1
ENSG00000173421	18.43093608	67.99335362	-1.885976801	0.015344187	0.038189226	CCDC36
ENSG00000273125	6.316801524	0.336469917	4.032385825	0.015351204	0.038203031	LINC01990
ENSG00000161896	43.8831416	9.281654296	2.249558763	0.0153643	0.038231959	IP6K3
ENSG00000232912	6.891929109	0.336469917	4.158483312	0.015385628	0.038281363	RERE-AS1
ENSG00000152082	838.248649	1280.872909	-0.611734836	0.015390318	0.038289367	MZT2B
ENSG00000099991	1253.762277	1863.383342	-0.571480211	0.015403189	0.038317719	CABIN1
ENSG00000215559	87.91404444	11.09503094	2.990056435	0.015409587	0.038329964	ANKRD20A11P
ENSG00000171056	23.46205984	1.936102971	3.632835777	0.015428385	0.038373049	SOX7
ENSG00000257267	754.0827007	493.5012227	0.611079887	0.01542991	0.038373169	ZNF271P
ENSG00000133067	16.30134995	1.773238453	3.192280159	0.015435388	0.038383117	LGR6
ENSG00000162836	633.0513362	306.6437551	1.04639217	0.015439339	0.038389268	ACP6
ENSG00000128699	1428.641618	1037.23731	0.461694322	0.015496248	0.038526519	ORMDL1
ENSG00000136280	938.970182	1332.875348	-0.50543187	0.015497504	0.038526519	CCM2
ENSG00000267131	7.579201602	0.693008929	3.414225889	0.015504151	0.038539357	AC005746.2
ENSG00000119650	303.5692898	528.4253983	-0.799414258	0.015512667	0.038553876	IFT43
ENSG00000266258	38.01141567	11.26149148	1.760389188	0.01551296	0.038553876	LINC01909

ENSG00000126467	12.79736137	1.345879667	3.203377765	0.015523696	0.038576868	TSKS
ENSG00000078246	985.5170837	596.1982955	0.725233837	0.015546608	0.038630108	TULP3
ENSG00000100949	93.27858181	258.8216907	-1.471892839	0.015551936	0.038639652	RABGGTA
ENSG00000270170	341.8988771	481.6289596	-0.494173465	0.015567964	0.038675777	NCBP2AS2
ENSG00000267364	9.483529005	0.672939833	3.756919659	0.015581992	0.038706925	AC022706.1
ENSG00000270039	29.72377174	10.2734351	1.528134246	0.015587767	0.038717568	AC025165.5
ENSG00000253875	15.53202046	2.853467977	2.44257753	0.015621117	0.038796697	AC013643.2
ENSG00000269067	5.512654262	0	4.795080881	0.015635631	0.038829032	ZNF728
ENSG00000127528	2163.544759	592.8666147	1.867438969	0.015640118	0.038836463	KLF2
ENSG00000125870	1082.180923	1487.626615	-0.459202866	0.01565448	0.03886841	SNRPB2
ENSG00000057252	782.2781542	1130.559352	-0.53148533	0.015660047	0.038878518	SOAT1
novel.1047	7.669891939	0.356539013	4.309502248	0.015670708	0.038901267	-
ENSG00000087087	1852.142812	2869.230873	-0.631458612	0.01567285	0.038902867	SRRT
ENSG00000248124	84.56392718	14.36426492	2.551676895	0.015694728	0.038953451	RRN3P1
ENSG00000213600	2.139615324	12.18702883	-2.510072688	0.015709019	0.038985196	U73169.1
ENSG00000286163	5.099746614	0	4.685460452	0.015729633	0.039032626	AC002377.1
ENSG00000287018	10.45275951	0	5.718926451	0.015746362	0.039070408	AL161717.1
ENSG00000089060	534.2385851	333.7088954	0.678602588	0.015751764	0.039080078	SLC8B1
ENSG00000225137	97.58965187	158.9192916	-0.704546232	0.015754138	0.039082236	DYNC1I2P1
ENSG00000136960	766.6165487	316.2349562	1.277785777	0.015762218	0.039096744	ENPP2
ENSG00000176261	244.29099	387.6041954	-0.666313334	0.01576357	0.039096744	ZBTB8OS
ENSG00000176953	702.5889328	1083.561486	-0.624805057	0.015764501	0.039096744	NFATC2IP
ENSG00000231749	4.83548223	0	4.607674798	0.015766148	0.039097098	ABCA9-AS1
ENSG00000246640	5.686228309	22.22318361	-1.959135464	0.015768685	0.039099657	PICART1
ENSG00000144357	1170.179407	678.3267242	0.786423536	0.015772783	0.039106086	UBR3
ENSG00000090534	11.04927039	0.672939833	3.982307017	0.015817563	0.039213368	THPO
ENSG00000287171	5.457208632	0	4.781721222	0.015840078	0.039265438	AC006062.1
ENSG00000122870	413.4378856	1549.082746	-1.905763588	0.015851339	0.039289606	BICC1
ENSG00000187049	186.3391688	110.2674122	0.756038074	0.015861336	0.039310632	TMEM216
ENSG00000234498	3.077290793	17.54115018	-2.52162778	0.015881124	0.039355921	RPL13AP20
ENSG00000276593	6.635257889	0.387220594	4.102745431	0.015942666	0.039504664	AC022306.3
ENSG00000270820	53.63469594	25.09800495	1.094863402	0.015945665	0.039508327	AC016727.1
ENSG00000104368	5276.23244	2083.562794	1.340352147	0.015948247	0.039510957	PLAT
ENSG00000254162	11.59889773	1.029478846	3.461683033	0.015954006	0.039521456	AC009812.3
ENSG00000118292	291.0619058	135.6569158	1.100943006	0.01596121	0.039535532	C1orf54
ENSG00000163607	113.9748544	215.147547	-0.917700847	0.015970388	0.039554496	GTPBP8
ENSG00000172201	1178.459243	75.44828749	3.964753328	0.015972266	0.039555374	ID4
ENSG00000183814	81.72893239	164.7139528	-1.012033037	0.015977649	0.039564935	LIN9
ENSG00000123575	1156.517536	771.0788091	0.585077331	0.015985717	0.039581142	FAM199X
ENSG00000220517	0.307102255	5.747074145	-4.142163055	0.01599828	0.039608474	ASS1P1
ENSG00000214563	10.14816455	0	5.676223564	0.016005429	0.039622396	GAPDHP15
ENSG00000100034	2208.658493	1574.833194	0.487912822	0.016021543	0.039658509	PPM1F
ENSG00000278546	1.480065645	12.29811519	-3.039758501	0.016033631	0.03968465	AC003101.2
ENSG00000071994	823.106757	1217.348697	-0.564853805	0.0160359	0.039686485	PDCD2
ENSG00000236565	26.08173855	51.05779112	-0.971222684	0.016048866	0.03971479	HNRNPA3P5
ENSG00000185973	103.5274655	158.6343431	-0.615139671	0.016058365	0.039734513	TMLHE
ENSG00000122543	5.396748408	0	4.767049032	0.016072041	0.039764565	OCM
ENSG00000183935	52.28461102	124.2633698	-1.250695817	0.016074418	0.039766658	HTR7P1
ENSG00000101280	10.71061311	0.672939833	3.936900368	0.016076081	0.039766988	ANGPT4
ENSG00000277128	4.866965991	0	4.616041299	0.016084081	0.039782989	AL589743.4
ENSG00000169756	1432.36089	2842.6149	-0.9887398	0.016089107	0.039791631	LIMS1

ENSG00000213064	1179.605891	789.2368745	0.579372077	0.016095092	0.039802645	SFT2D2
ENSG00000066654	1006.697251	701.7079739	0.520502516	0.016098731	0.039807854	THUMPD1
novel.531	35.61527132	71.45112501	-1.004436355	0.016115283	0.039844992	-
ENSG00000139187	39.8658815	11.51306151	1.791795565	0.016117152	0.039845821	KLRG1
ENSG00000266104	0	4.666844622	-4.803708564	0.016138227	0.039892156	MIR4326
ENSG00000172985	578.2649441	977.1516048	-0.756684833	0.016138965	0.039892156	SH3RF3
ENSG00000149243	75.92679788	40.20346094	0.916811362	0.01615769	0.039934641	KLHL35
ENSG00000004975	826.6084239	1127.295477	-0.447786325	0.016161561	0.039940409	DVL2
ENSG00000203667	34.50004739	12.42328166	1.470323824	0.01616757	0.039951458	COX20
ENSG00000197594	267.9597173	170.5698831	0.652586475	0.016175629	0.039967572	ENPP1
ENSG00000100410	448.7405032	666.1281029	-0.570269115	0.016183064	0.039982139	PHF5A
ENSG00000158156	177.8728375	291.047441	-0.709255561	0.016211484	0.040048546	XKR8
novel.902	10.38435011	0	5.710874851	0.016221506	0.040069496	-
ENSG00000120451	1296.40685	1774.154873	-0.452729249	0.016227743	0.040081089	SNX19
ENSG00000181264	239.6355638	376.0638255	-0.650162152	0.016230363	0.040083752	TMEM136
ENSG00000272564	4.822874471	0	4.604306215	0.016243366	0.040112053	AC012511.1
ENSG00000249465	12.4436603	2.934900236	2.100368978	0.016251112	0.040127365	RBMXP4
ENSG00000198945	172.5022301	271.1635401	-0.652472562	0.016276097	0.040185239	L3MBTL3
ENSG00000279539	1.817398012	9.700840742	-2.416457944	0.016281219	0.040194066	AC006486.2
ENSG00000184863	1530.580866	872.9848864	0.810297562	0.016304419	0.040247517	RBM33
ENSG00000172640	7.096986917	0	5.162745489	0.01630672	0.040249373	OR10AD1
ENSG00000089053	2216.862418	3068.76437	-0.469122279	0.016346041	0.040342593	ANAPC5
ENSG00000187730	11.84553976	0.672939833	4.088164136	0.016354633	0.040359967	GABRD
ENSG00000182583	7.667099907	0	5.274177297	0.0163734	0.040402441	VCX
ENSG00000169306	48.62249162	11.58490877	2.079175383	0.0163761	0.040405266	IL1RAPL1
ENSG00000271752	6.262609543	0	4.98193381	0.016380093	0.04041128	AC112187.3
ENSG00000137869	131.0293077	50.04196002	1.392529065	0.016382597	0.04041362	CYP19A1
ENSG00000274471	86.90200389	36.03607914	1.269534908	0.016384882	0.040415421	AC242376.2
ENSG00000287616	10.42091974	0.713078025	3.864395747	0.016390477	0.040425382	AL357522.1
ENSG00000112357	96.5488205	153.8414329	-0.672935824	0.016394374	0.040431157	PEX7
ENSG00000076053	324.4531408	512.5620682	-0.659749547	0.016402103	0.040446377	RBM7
ENSG00000264673	0.307102255	10.53536082	-5.013395118	0.016406012	0.040452179	AC098850.1
ENSG00000186472	3.033199274	12.43273827	-2.039768769	0.016414115	0.040468317	PCLO
ENSG00000166268	6.068905845	0.336469917	3.972918753	0.016459461	0.040576264	MYRFL
ENSG00000230303	9.952851197	1.365948763	2.824368288	0.016474163	0.040605988	AL353743.2
ENSG00000120669	2.420248412	11.58734891	-2.256332268	0.016474644	0.040605988	SOHLH2
ENSG00000121931	131.2697524	262.1173211	-0.997524801	0.016476261	0.040606121	LRIF1
ENSG00000102978	1066.896414	1488.125016	-0.480193424	0.016485463	0.040624948	POLR2C
novel.739	1.835020366	17.05597707	-3.222160706	0.016509013	0.040677504	-
ENSG00000213062	45.58359357	20.84906251	1.128319892	0.016509922	0.040677504	Z99572.1
ENSG00000287609	0.308355904	7.060987961	-4.434324469	0.016530077	0.0407233	AL589787.2
ENSG00000181541	0	9.501434051	-5.832109687	0.016535574	0.040731415	MAB21L2
ENSG00000141456	848.1527657	1224.264659	-0.52958853	0.016536506	0.040731415	PELP1
ENSG00000196151	199.0603145	127.2687046	0.645875383	0.01655145	0.040764359	WDSUB1
ENSG00000065883	1240.028993	832.7813911	0.574609071	0.016561767	0.040785902	CDK13
ENSG00000171928	598.5079487	968.0348103	-0.693466389	0.016564703	0.040789267	TVP23B
ENSG00000154928	17.61898219	58.55020061	-1.730340792	0.016574903	0.040810515	EPHB1
ENSG00000160392	179.0501317	275.2840712	-0.620547571	0.016578452	0.040815385	C19orf47
ENSG00000021762	929.0468337	1411.295005	-0.603088814	0.016589195	0.040837965	OSBPL5
novel.234	21.79366109	7.785962742	1.476173349	0.016595512	0.040849643	-
ENSG00000250132	67.38721294	31.15317918	1.112614399	0.016605582	0.040870559	AC004803.1

ENSG00000157379	87.91485555	29.24403338	1.590964597	0.01660843	0.040873697	DHRS1
ENSG00000168461	10181.84277	4962.461324	1.03692416	0.016622578	0.040904642	RAB31
ENSG00000215283	10.58217076	2.129777466	2.303765669	0.016645079	0.040956133	HMGB3P24
ENSG00000232233	0.308355904	7.216964408	-4.47672414	0.016651264	0.040967471	LINC02043
ENSG00000234771	179.2876852	88.34256811	1.022708685	0.016664126	0.040995232	SLC25A25-AS1
ENSG00000280649	45.65282934	17.58933232	1.382546361	0.016685983	0.041045118	AC245100.8
ENSG00000248049	171.5561785	106.5909357	0.68696192	0.016699242	0.041073842	UBA6-AS1
ENSG00000226102	2.109385211	21.58362229	-3.355155137	0.01670341	0.041080207	SEPT7P3
ENSG00000163519	10.78493474	0	5.764014006	0.016706686	0.041084375	TRAT1
ENSG00000167861	64.22599825	24.60787034	1.389189023	0.016715479	0.041102107	HID1
ENSG00000181467	871.0194807	606.7690083	0.521194466	0.016718193	0.041104889	RAP2B
ENSG00000253293	3.992329314	161.1144263	-5.336050951	0.01672107	0.041108074	HOXA10
ENSG00000242902	1.232169966	9.894386842	-3.014055599	0.016729786	0.041125609	FLNC-AS1
ENSG00000184260	12.00553713	31.97824265	-1.407238968	0.016731798	0.041126664	HIST2H2AC
ENSG00000241155	22.98299319	8.600542122	1.417252319	0.016739573	0.04114055	ARHGAP31-AS1
ENSG00000174521	7.186423605	0.387220594	4.218741999	0.016740614	0.04114055	TTC9B
ENSG00000153956	244.3736599	469.7588105	-0.943473296	0.01674407	0.041145151	CACNA2D1
ENSG00000230387	0.308355904	10.56604241	-5.01743835	0.016756002	0.041170459	AL118508.1
ENSG00000236977	5.659759143	0	4.836313501	0.016757538	0.041170459	ANKRD44-IT1
ENSG00000269982	81.5042153	39.45957294	1.049710811	0.016782089	0.041226878	AC018809.2
ENSG00000273464	11.18000657	1.844058227	2.620669006	0.01679725	0.041260221	AP000238.1
ENSG00000260727	2.406387005	14.05488056	-2.546098117	0.016809104	0.041285434	SLC7A5P1
ENSG00000205885	38.2988152	83.64439961	-1.128845978	0.01681915	0.041306205	C1RL-AS1
ENSG00000156273	1283.787438	765.3641678	0.746545638	0.016825607	0.041318156	BACH1
ENSG00000268006	30.51684962	13.20717946	1.205640475	0.016837203	0.041342724	PTOV1-AS1
ENSG00000243642	6.582319557	0.336469917	4.092510581	0.016873541	0.041428034	RN7SL526P
ENSG00000166347	914.324761	579.6954857	0.6574009	0.016893058	0.041468209	CYB5A
novel.808	9.658356701	1.773238453	2.435741638	0.016893096	0.041468209	-
ENSG00000092847	950.8100678	653.6869567	0.540755056	0.016897062	0.041474026	AGO1
ENSG00000109667	123.3828078	47.42427815	1.375477342	0.016908187	0.041497412	SLC2A9
ENSG00000176716	10.92548664	0	5.784258631	0.016913761	0.041505361	OR10AB1P
ENSG00000285684	12.14798342	0.774441189	4.029013012	0.016914621	0.041505361	AL137781.1
ENSG00000245680	294.839307	190.8911129	0.628552071	0.01694685	0.041580518	ZNF585B
ENSG00000197826	0	4.809640044	-4.842337998	0.01695797	0.041603874	CFAP299
ENSG00000225331	6.401223617	0.387220594	4.049099321	0.016974135	0.041637203	LINC01678
ENSG00000241081	6.38736221	23.69166121	-1.887895331	0.01697476	0.041637203	RPL22P2
ENSG00000230084	68.88601204	28.36706335	1.280081087	0.016984347	0.041656442	AC006059.1
ENSG00000158092	688.0049849	473.7310115	0.538425416	0.016985811	0.041656442	NCK1
ENSG00000175793	1.186824798	9.498993907	-2.98960576	0.016995178	0.04167548	SFN
ENSG00000280088	119.7930726	70.69662452	0.761375782	0.016999791	0.041682859	AC126474.2
ENSG00000124198	730.2692486	1046.030858	-0.518193909	0.017016105	0.041718923	ARFGEF2
ENSG00000241484	5.501300152	0	4.792392393	0.017031628	0.041752883	ARHGAP8
ENSG00000230107	5.184168707	0	4.706903466	0.01703317	0.041752883	AL022316.1
ENSG00000101945	148.5151611	254.5527732	-0.778027085	0.017036175	0.041756307	SUV39H1
ENSG00000107854	1557.916353	1109.189413	0.490163694	0.017038686	0.041758522	TNKS2
ENSG00000108064	464.7182396	678.5478226	-0.546390938	0.017044557	0.041768971	TFAM
ENSG00000265148	12.39330053	1.813376645	2.77956514	0.017047306	0.041771768	TSPOAP1-AS1
ENSG00000233070	9.655778133	0	5.605261424	0.017068144	0.041815023	ZFY-AS1
ENSG00000243811	11.09934542	1.793307549	2.630581163	0.017068177	0.041815023	APOBEC3D
ENSG00000033867	790.6916772	1332.724281	-0.752986228	0.017075908	0.041830018	SLC4A7
ENSG00000108107	9373.752688	14975.06031	-0.675865824	0.017086445	0.041851883	RPL28

ENSG00000254966	10.87917256	2.129777466	2.344086858	0.017096145	0.041871695	AC103974.1
ENSG00000288002	6.370993505	0.356539013	4.043102559	0.017110874	0.041903819	AC091912.3
ENSG00000179954	289.5254423	481.8734192	-0.73422438	0.017113613	0.041906577	SSC5D
ENSG00000117335	4068.302781	2393.927498	0.76506458	0.017125725	0.041932283	CD46
ENSG00000198865	191.8000397	66.81436685	1.518598819	0.017159682	0.042011469	CCDC152
ENSG00000173825	12.54069015	2.140389951	2.544988707	0.017175335	0.042044646	TIGD3
ENSG00000256576	38.73192377	11.53531396	1.75834136	0.017176561	0.042044646	LINC02361
ENSG00000104059	10.03970932	0	5.66116403	0.017178089	0.042044646	FAM189A1
ENSG00000243232	7.361536035	0.356539013	4.250265471	0.017182453	0.042048621	PCDHAC2
ENSG00000111670	1038.025897	749.2024639	0.470302683	0.017182949	0.042048621	GNPTAB
ENSG00000155749	20.4027474	5.828506405	1.817233306	0.017185174	0.042050105	FLACC1
ENSG00000269068	11.12964681	1.029478846	3.400544147	0.017192265	0.042063494	AC009955.4
ENSG00000240207	43.36457075	19.85270539	1.121305479	0.017198615	0.042075067	AC080013.1
ENSG00000251073	5.937884934	0	4.905692219	0.01720332	0.042082614	NUDT19P5
ENSG00000167395	449.6038136	646.3102103	-0.52338819	0.017238949	0.042165802	ZNF646
ENSG00000277481	6.301686468	0.356539013	4.029292814	0.017242015	0.04216933	PKD1L3
ENSG00000260927	20.74641928	4.503851709	2.22593466	0.017248993	0.042182426	AC009107.2
ENSG00000269834	171.8193315	105.0017396	0.712518244	0.01725099	0.042183339	ZNF528-AS1
ENSG00000198929	16.27223129	3.546476906	2.191476016	0.0172564	0.042192595	NOS1AP
ENSG00000112695	1529.177678	2300.593864	-0.589287428	0.017278555	0.04224279	COX7A2
ENSG00000203724	15.27939492	46.57991628	-1.61215599	0.01728924	0.042264936	C1orf53
ENSG00000267248	11.19386798	28.37040258	-1.337757254	0.017327127	0.042353568	AC025048.2
ENSG00000164056	674.3139495	236.0987528	1.513434208	0.017379069	0.042476537	SPRY1
ENSG00000184743	1580.531439	2301.858032	-0.542545101	0.017405183	0.042536361	ATL3
ENSG00000201700	1.214547612	12.09382821	-3.315172942	0.017412943	0.042551324	SNORD113-3
ENSG00000225762	12.09748112	2.241891306	2.461373232	0.017429623	0.042588079	LINC01389
ENSG00000259256	5.085885207	0	4.681848449	0.017443772	0.042618643	LINC01895
ENSG00000148331	604.4942433	974.8001938	-0.689538247	0.017446176	0.042620507	ASB6
ENSG00000109270	775.087088	541.9469361	0.515791251	0.0174842	0.042709383	LAMTOR3
ENSG00000146802	973.0426384	566.9481444	0.779236412	0.017490071	0.042719708	TMEM168
ENSG00000257815	45.74219511	16.74175961	1.454375457	0.017504137	0.042750046	AC025159.1
ENSG00000143819	1363.45132	534.9883486	1.349584944	0.017527049	0.042801978	EPHX1
ENSG00000162139	197.6441853	305.6634512	-0.629577616	0.017557581	0.042866559	NEU3
novel.1035	121.6947356	15.44650222	2.981960173	0.017558303	0.042866559	-
ENSG00000177455	19.2879504	2.475703993	2.953088278	0.017558896	0.042866559	CD19
ENSG00000162616	775.8326527	1520.051809	-0.970530271	0.017560093	0.042866559	DNAJB4
ENSG00000171476	66.55870436	14.20011613	2.222442875	0.017575218	0.042898961	HOPX
ENSG00000167549	279.9115755	127.7005113	1.133087026	0.017576669	0.042898961	CORO6
ENSG00000243243	0.922560414	8.102363562	-3.151998516	0.017594524	0.042936127	AC073130.2
ENSG00000059588	1311.42267	717.661563	0.869564758	0.017595202	0.042936127	TARBP1
ENSG00000102935	200.4808006	36.93838374	2.437958954	0.017597682	0.042938145	ZNF423
ENSG00000149926	1.495180701	9.305319412	-2.628842035	0.0176025	0.042945869	FAM57B
ENSG00000250510	160.2456764	55.61226511	1.527069366	0.017622606	0.042990885	GPR162
ENSG00000254858	201.8305736	361.4001596	-0.840711469	0.017628025	0.042999861	MPV17L2
ENSG00000248866	56.57372866	24.40743617	1.207445908	0.017629596	0.042999861	USP46-AS1
ENSG00000115966	994.1891126	700.0561647	0.506369496	0.017645741	0.043035201	ATF2
ENSG00000021355	992.5767706	648.6524602	0.613836747	0.017667037	0.043083095	SERPINB1
ENSG00000160917	548.5668993	805.1420126	-0.553675412	0.017671836	0.043090753	CPSF4
ENSG00000163666	32.2072724	13.71712638	1.233639358	0.017678941	0.043104033	HESX1
ENSG00000283345	4.807759415	0	4.600225016	0.017687434	0.043120693	AC127502.3
ENSG00000106100	382.3896646	166.9148068	1.195246998	0.017692222	0.043128319	NOD1

ENSG00000276136	13.94134829	2.772035718	2.306447585	0.017699175	0.043141219	AC016957.2
ENSG00000165526	377.0650709	569.5037396	-0.594834709	0.017705685	0.043153039	RPUSD4
ENSG00000118965	534.0856834	362.2591492	0.559686685	0.017724676	0.043193771	WDR35
ENSG00000130844	422.0638356	272.4703091	0.632190935	0.017725722	0.043193771	ZNF331
ENSG00000096654	134.8310535	80.24662589	0.745709235	0.017742533	0.043230682	ZNF184
ENSG00000220494	0.307102255	6.644370055	-4.355721523	0.017764989	0.043281337	YAP1P1
ENSG00000224361	4.839243176	0	4.60866139	0.017767177	0.043282608	AC011239.1
ENSG00000171792	313.144735	512.9234529	-0.71252111	0.01777001	0.043285453	RHNO1
ENSG00000100439	901.6821096	586.2932146	0.620681484	0.017777728	0.043300193	ABHD4
ENSG00000236155	13.73886905	2.058957692	2.708152231	0.017780048	0.043301785	AL355877.1
ENSG00000178585	515.0986661	344.9991891	0.577444933	0.017786216	0.043312745	CTNNBIP1
ENSG00000117013	17.61138903	4.718751175	1.906542474	0.017788264	0.043313674	KCNQ4
ENSG00000287042	12.37686056	2.241891306	2.493853047	0.017791187	0.043314003	AC090945.1
ENSG00000183688	8.297957856	34.27448066	-2.049816306	0.017791734	0.043314003	RFLNB
ENSG00000184206	9.99443542	1.069617038	3.217490736	0.017793583	0.043314445	GOLGA6L4
ENSG00000260773	11.74127275	1.762625967	2.722898758	0.017817684	0.04336905	AC055855.1
ENSG00000170006	133.1012691	209.1482949	-0.652016424	0.017844776	0.043430925	TMEM154
ENSG00000186838	6.442736568	0	5.020255365	0.017874548	0.043499311	SELENOV
ENSG00000286348	9.801700636	0	5.62757028	0.017888767	0.043529836	AC084834.1
ENSG00000196865	890.2078824	581.842635	0.613320542	0.017904874	0.043561128	NHLRC2
ENSG00000187013	41.84048486	19.87277448	1.068251947	0.01790498	0.043561128	C17orf82
ENSG00000226306	6.075174089	0	4.936335753	0.017937234	0.043635513	NPY6R
ENSG00000163053	98.84216461	34.99025562	1.493986529	0.017961749	0.043691059	SLC16A14
ENSG00000115841	110.6616625	46.87393625	1.235922354	0.017985955	0.043745843	RMDN2
ENSG00000129566	1163.080354	757.5405195	0.618661934	0.018005349	0.043788915	TEP1
ENSG00000111364	412.5328379	575.6761176	-0.48106125	0.0180131	0.043803664	DDX55
ENSG00000165502	2075.389605	2945.726526	-0.505311956	0.018019005	0.043813922	RPL36AL
ENSG00000163032	34.40881506	6.64552593	2.363801762	0.018030003	0.043836563	VSNL1
ENSG00000205730	1256.685649	1889.621574	-0.588493953	0.018040632	0.043856575	ITPRIPL2
ENSG00000163191	5199.428188	8009.994333	-0.623445981	0.018042995	0.043856575	S100A11
ENSG00000181418	2.728604316	12.7785364	-2.224312912	0.018043298	0.043856575	DDN
ENSG00000058729	375.0103321	582.5498996	-0.636021426	0.018046439	0.043860107	RIOK2
ENSG00000135723	960.0735234	1424.58014	-0.569440146	0.018054241	0.043874962	FHOD1
ENSG00000164761	161.7285778	373.4224664	-1.206783854	0.018104069	0.04399194	TNFRSF11B
ENSG00000132323	792.8217076	487.4311168	0.701699698	0.018131418	0.044054275	ILKAP
ENSG00000154429	385.2461537	248.0451968	0.636301091	0.018141126	0.044073741	CCSAP
ENSG00000211666	7.382919335	0	5.216840318	0.018145409	0.044080026	IGLV2-14
ENSG00000100095	10.33803603	1.00940975	3.304425449	0.018156014	0.044101663	SEZ6L
ENSG00000112110	796.9407661	1177.650161	-0.563642671	0.018168786	0.044128562	MRPL18
ENSG00000233913	249.683052	359.8655669	-0.527570782	0.018181021	0.044154152	RPL10P9
ENSG00000198937	150.5502525	260.045452	-0.789451766	0.018187862	0.044163685	CCDC167
ENSG00000186480	894.7754663	2030.4848	-1.182099847	0.018188556	0.044163685	INSIG1
ENSG00000178662	60.85589388	18.62022383	1.703345647	0.018190046	0.044163685	CSRNP3
ENSG00000140199	572.3867487	383.2465592	0.578065738	0.018191896	0.044164048	SLC12A6
ENSG00000112812	1.830005771	13.11487792	-2.839283562	0.018223501	0.044236643	PRSS16
ENSG00000104783	50.79136815	98.36554187	-0.951197865	0.01825745	0.044314911	KCNN4
ENSG00000270433	6.335677526	0.356539013	4.03604459	0.018277754	0.04436005	H3F3AP2
ENSG00000157426	203.6113315	294.3984924	-0.531403673	0.018279487	0.04436011	AASDH
ENSG00000112033	1208.149366	2341.855984	-0.954703994	0.018284465	0.044368048	PPARD
ENSG00000198839	213.622705	376.5112878	-0.817039738	0.018286587	0.044369053	ZNF277
ENSG00000276832	6.09662866	0.336469917	3.97866912	0.018290166	0.044373592	AL354718.3

ENSG00000287021	5.200537412	0	4.711088027	0.018296106	0.044383859	AC097462.3
ENSG00000233093	6.455344327	0	5.022927242	0.018306195	0.044404189	LINC00892
ENSG00000261363	7.362789684	0.336469917	4.250544875	0.018309379	0.044407765	AC106745.1
ENSG00000183011	913.074424	1452.274825	-0.669645466	0.018312128	0.044410285	NAA38
ENSG00000159596	276.2097952	433.9607355	-0.651985673	0.01833455	0.044451499	TMEM69
ENSG00000155115	604.6735284	1025.701555	-0.762702901	0.018336003	0.044451499	GTF3C6
ENSG00000267774	0	5.270120557	-4.981604122	0.01833656	0.044451499	AC010776.2
ENSG00000287063	6.420099619	0.336469917	4.052907995	0.018337523	0.044451499	AL356489.3
ENSG00000157111	33.52798142	155.0977552	-2.210825879	0.018337676	0.044451499	TMEM171
ENSG00000113073	9.665949865	1.10029862	3.152723576	0.01834209	0.044456689	SLC4A9
ENSG00000260830	4.882081047	0	4.620120241	0.01834324	0.044456689	AL135744.1
ENSG00000106459	384.5280816	256.0408876	0.586461261	0.018356677	0.044485106	NRF1
ENSG00000105248	470.2952579	322.460585	0.543755271	0.018367646	0.044507538	YJU2
ENSG00000244274	25.61714622	7.644323194	1.730288234	0.01837176	0.044513353	DBNDD2
novel.507	38.86022392	81.83418662	-1.073901266	0.018381625	0.044533104	-
ENSG00000134874	786.6637329	1125.317122	-0.516421262	0.018387264	0.044542612	DZIP1
ENSG00000270344	47.66880345	20.42054784	1.222690702	0.018392983	0.044552312	POC1B-AS1
ENSG00000115041	72.22555812	148.2378777	-1.03722463	0.018402551	0.04456896	KCNIP3
ENSG00000170191	122.7447127	201.3005838	-0.713670765	0.018403287	0.04456896	NANP
ENSG00000205482	13.2515685	3.169868797	2.051539154	0.018422146	0.044610473	SPDYE18
ENSG00000130958	225.1367538	147.1497798	0.613769464	0.018427508	0.0446193	SLC35D2
ENSG00000265015	5.963100452	0	4.911311084	0.018432575	0.044619396	AP005901.3
ENSG00000257848	5.963100452	0	4.911311084	0.018432575	0.044619396	AC074029.1
ENSG00000164199	21.22634013	2.884149558	2.880681277	0.0184327	0.044619396	ADGRV1
ENSG00000185149	0	8.532162493	-5.676957283	0.018437459	0.044626759	NPY2R
ENSG00000131238	3353.259148	2321.274003	0.53051352	0.018444429	0.044639471	PPT1
ENSG00000182446	2007.899996	2703.444452	-0.429051515	0.018451317	0.044651982	NPLOC4
ENSG00000144635	641.8967813	977.7656472	-0.607437714	0.018456676	0.04466079	DYNC1LI1
ENSG00000108852	90.22743131	328.8304653	-1.865616964	0.018465877	0.044671267	MPP2
ENSG00000253616	0	4.351599676	-4.700772235	0.018466164	0.044671267	AC107959.3
ENSG00000237289	0	4.351599676	-4.700772235	0.018466164	0.044671267	CKMT1B
ENSG00000005700	84.02353014	176.9968664	-1.07469149	0.018476653	0.0446885	IBTK
novel.1076	34.99536803	7.735212064	2.17039354	0.018476728	0.0446885	-
ENSG00000230630	124.0347918	392.641098	-1.662899713	0.018481051	0.044694796	DNM3OS
ENSG00000096433	3737.367067	2003.143995	0.899880787	0.01848635	0.044703449	ITPR3
novel.149	5.487438745	0	4.789073713	0.018492346	0.044713788	-
ENSG00000275765	56.39073833	19.85398966	1.498664938	0.018497393	0.044718318	AC091982.3
ENSG00000267623	4.848089989	0	4.611037983	0.018497662	0.044718318	AC005357.2
ENSG00000164438	8.309240695	0	5.387545275	0.018510503	0.044745196	TLX3
ENSG00000117597	353.6871047	602.2245513	-0.768410433	0.018515644	0.044749775	UTP25
ENSG00000175110	439.5968851	629.8962946	-0.519047824	0.018515841	0.044749775	MRPS22
novel.354	28.20874612	10.97705651	1.354345354	0.018560389	0.044853267	-
ENSG00000256433	38.39061699	14.79979604	1.368356777	0.018564588	0.04485924	AC005840.3
ENSG00000254847	6.056298086	0.356539013	3.970339519	0.018577366	0.044881843	AC009806.1
ENSG00000223393	6.314294226	0.336469917	4.031759165	0.018580366	0.044881843	AL118511.1
ENSG00000257885	5.140077187	0	4.695699369	0.018580851	0.044881843	PHBP18
ENSG00000277521	5.140077187	0	4.695699369	0.018580851	0.044881843	MIR8078
ENSG00000277737	9.3261102	0	5.555288258	0.018592368	0.044905485	FP325317.1
ENSG00000260645	37.44660201	16.00031175	1.233290451	0.018609612	0.044942957	AL359715.2
ENSG00000164331	484.1669428	309.3652969	0.647211151	0.018611962	0.044944455	ANKRA2
ENSG00000164713	1780.180742	2730.470769	-0.617042101	0.018616453	0.04495112	BRI3

ENSG00000272009	9.093258306	1.060160428	3.087503774	0.018619512	0.04495433	AL121944.1
ENSG00000198863	324.5397576	486.3045717	-0.582783455	0.018627339	0.044969047	RUNDC1
ENSG00000141338	5693.251819	412.6249838	3.786233779	0.018630977	0.044973652	ABCA8
novel.824	0	97.75094275	-9.192155631	0.018658001	0.045034702	-
ENSG00000135835	274.0046772	142.4414655	0.944812479	0.018660346	0.045036179	KIAA1614
ENSG00000250215	4.806505766	0	4.599860109	0.018688832	0.045100737	CIR1P2
ENSG00000259972	155.3945538	86.21381812	0.852790222	0.018699113	0.045121357	AC009120.2
ENSG00000230928	13.01850315	1.436768536	3.182285069	0.018731524	0.045195367	AL139241.1
ENSG00000151445	362.2595696	547.7256253	-0.596397914	0.018739297	0.045209924	VIPAS39
ENSG00000196091	5.833617924	0	4.876706928	0.01875099	0.045231603	MYBPC1
ENSG00000287140	5.10350756	0	4.686320683	0.018752548	0.045231603	AC066595.2
ENSG00000225163	0	4.300848999	-4.685969663	0.018753506	0.045231603	LINC00618
ENSG00000251361	35.34828617	8.26651235	2.084386925	0.01875772	0.045237568	AC012625.1
ENSG00000228971	5.098492965	0	4.685037652	0.018776235	0.045278018	LINC02607
ENSG00000229939	5.462294498	18.22110648	-1.740460953	0.018786762	0.045295436	AL589880.1
ENSG00000273363	8.97993102	0.723690511	3.642708866	0.018786945	0.045295436	AL353801.3
ENSG00000111684	75.03794379	134.5628629	-0.843553324	0.018806461	0.045338281	LPCAT3
ENSG00000259438	0	4.290236513	-4.682988259	0.018814716	0.045353976	MAPK6-DT
ENSG00000124145	2136.589835	3985.635935	-0.899405008	0.0188231	0.045369975	SDC4
ENSG00000141219	437.2728833	299.1474113	0.547478594	0.01882655	0.045371693	C17orf80
ENSG00000196569	4711.560132	1202.826693	1.969667622	0.018827306	0.045371693	LAMA2
ENSG00000183690	30.25892475	11.8506873	1.352531627	0.018831811	0.045378342	EFHC2
ENSG00000141098	344.4777705	505.06974	-0.551845041	0.018838049	0.045387733	GFOD2
ENSG00000165029	2722.899348	1006.662509	1.435746599	0.018839202	0.045387733	ABCA1
ENSG00000084073	761.921108	1214.06885	-0.672437733	0.018842087	0.045390473	ZMPSTE24
ENSG00000006451	1122.140501	1860.959473	-0.729650615	0.018893306	0.04550964	RALA
ENSG00000143590	14.89288517	35.75840385	-1.261403822	0.018897722	0.045516058	EFNA3
ENSG00000267100	386.1136957	271.5264193	0.507467529	0.018903806	0.045526491	ILF3-DT
ENSG00000237214	3614.967959	5539.202949	-0.615682787	0.018921291	0.04556048	AL080243.2
ENSG00000265218	6.009699269	0.336469917	3.960676693	0.018921426	0.04556048	AC103810.2
ENSG00000205084	167.7806171	248.272297	-0.564903702	0.018938772	0.045598019	TMEM231
ENSG00000285755	9.573934608	1.10029862	3.140430495	0.018947686	0.045615254	AC132153.1
ENSG00000198730	753.2659016	1211.293556	-0.685614163	0.018951899	0.045621169	CTR9
novel.1089	47.05739063	9.893230966	2.253916764	0.018954318	0.045621866	-
ENSG00000210151	11.51232434	2.211209725	2.399793224	0.0189557	0.045621866	MT-TS1
ENSG00000186416	210.1520668	314.9555084	-0.584228869	0.01896243	0.045633835	NKRF
ENSG00000235027	32.01586254	12.03593267	1.419205587	0.018967081	0.045640802	AC068580.3
ENSG00000152931	9.170158507	0.713078025	3.676664998	0.018969946	0.045643469	PART1
ENSG00000155508	820.7296869	1511.047229	-0.880455265	0.018983203	0.045669016	CNOT8
ENSG00000189223	386.6172191	1930.115666	-2.319762598	0.01898408	0.045669016	PAX8-AS1
ENSG00000242337	11.71480358	1.844058227	2.687535107	0.018990129	0.04567934	INHCAP
ENSG00000205560	11.94633055	1.161661783	3.416987056	0.019005055	0.045711009	CPT1B
ENSG00000171522	605.5109091	161.1592692	1.908781545	0.019007761	0.045713286	PTGER4
ENSG00000163521	96.0548957	217.8644313	-1.182371406	0.019011293	0.04571755	GLB1L
ENSG00000073060	801.3308594	1262.980951	-0.656611249	0.019032061	0.045763254	SCARB1
ENSG00000266302	3.685227059	42.99539018	-3.546641309	0.019034615	0.045765162	AC098850.3
ENSG00000180817	1148.755538	2055.775347	-0.839768624	0.019049578	0.045796898	PPA1
ENSG00000245156	8.087600719	26.95254727	-1.730287878	0.019061405	0.045821092	AP001107.1
ENSG00000071859	1781.843134	2683.477789	-0.590602287	0.019069702	0.045836797	FAM50A
ENSG00000188419	422.3375444	659.318531	-0.642247152	0.019072526	0.045839342	CHM
ENSG00000281195	28.3212192	3.53586442	2.997527483	0.019074861	0.045840715	AC007878.1

ENSG00000228817	13.85950477	2.893606169	2.265426596	0.01907751	0.045842841	BACH1-IT2
novel.40	12.02337295	0	5.920952401	0.019108947	0.045914136	-
ENSG00000206140	19.44021242	5.522718069	1.829016653	0.019110956	0.045914716	TMEM191C
ENSG00000198890	682.1918686	444.6427918	0.616957564	0.019114226	0.045914879	PRMT6
ENSG00000180694	685.1180631	432.9821701	0.662085509	0.019114558	0.045914879	TMEM64
ENSG00000261742	22.77822011	1.00940975	4.464052098	0.019116562	0.045915291	LINC00922
ENSG00000227232	110.5603304	40.51515704	1.446674808	0.019118264	0.045915291	WASH7P
ENSG00000268119	12.42603794	2.261960402	2.49119172	0.019144849	0.045970901	AC010615.2
ENSG00000169062	1521.479827	1063.523501	0.516743824	0.019144958	0.045970901	UPF3A
ENSG00000270021	19.76619068	6.05286248	1.701508713	0.019173405	0.046034953	AC026691.1
ENSG00000175054	470.4842036	758.5037202	-0.689199892	0.019195847	0.046084579	ATR
ENSG00000006128	23.40976224	110.9404461	-2.245924437	0.019221359	0.046141562	TAC1
ENSG00000102904	64.1235978	28.03778548	1.186806561	0.01922341	0.046142224	TSNAXIP1
ENSG00000274523	823.583913	1257.051025	-0.610299356	0.019234621	0.046164868	RCC1L
ENSG00000155090	823.3445695	1720.981887	-1.063539935	0.019253157	0.04620463	KLF10
ENSG00000271870	67.10142271	35.33476948	0.923639255	0.019254745	0.04620463	AC024060.1
ENSG00000038382	3401.306101	5591.783752	-0.717167323	0.019261098	0.046215607	TRIO
ENSG00000133519	5.725305234	0	4.851580463	0.01926315	0.046216262	ZDHHC8P1
ENSG00000233337	11.49337707	2.567748738	2.179107639	0.019265677	0.046218058	UBE2FP3
ENSG00000197818	491.0836605	342.002365	0.521874079	0.019267974	0.046219299	SLC9A8
ENSG00000116157	234.7393797	432.2562497	-0.880039989	0.019282799	0.04625059	GPX7
ENSG00000161265	96.71619758	47.62903186	1.026443703	0.01928743	0.046257428	U2AF1L4
ENSG00000285572	4.543495031	0	4.517463198	0.019294281	0.046269587	AL136441.1
ENSG00000183067	6.720933631	0.387220594	4.119284312	0.019297006	0.046271852	IGSF5
ENSG00000184599	10.13931774	0.723690511	3.814249307	0.019306473	0.04629028	TAFA3
ENSG00000225774	9.581527772	0.713078025	3.742706687	0.019330194	0.046342878	SIRPAP1
ENSG00000010282	5.205552007	0	4.712390414	0.0193343	0.046348445	HHATL
ENSG00000182742	40.64907239	242.9425171	-2.579831347	0.019340686	0.046357478	HOXB4
ENSG00000275228	5.643390438	0	4.832341286	0.019341637	0.046357478	AC024257.5
ENSG00000124151	848.7244011	1283.152972	-0.596535379	0.019344221	0.046359395	NCOA3
ENSG00000180953	32.95095944	60.36426641	-0.874068049	0.019356711	0.046380982	ST20
ENSG00000245322	22.94266261	7.735212064	1.557764228	0.019356799	0.046380982	AC097460.1
ENSG00000261366	22.17132403	7.418811243	1.569701319	0.019367882	0.046403259	MANEA-DT
ENSG00000279696	39.62626317	13.66765997	1.528553751	0.01937492	0.046415841	AP001273.1
ENSG00000155893	257.2799037	144.7312478	0.828346952	0.019383398	0.046431869	PXYLP1
ENSG00000001036	1043.264597	1693.787452	-0.699351194	0.019388165	0.046439008	FUCA2
ENSG00000263002	258.9509245	173.4848898	0.578293368	0.019408482	0.046483386	ZNF234
ENSG00000279103	10.24734569	1.518200796	2.799029029	0.019417172	0.046499912	AC138470.1
ENSG00000100739	1.847628125	16.53734423	-3.165849401	0.019429123	0.046523963	BDKRB1
ENSG00000175130	3332.175126	1846.899451	0.851305741	0.019430796	0.046523963	MARCKSL1
ENSG00000248388	0.308355904	5.848575501	-4.164039051	0.019433884	0.046527069	AC093801.1
ENSG00000083844	616.3653821	458.1309729	0.428237081	0.019448467	0.046557692	ZNF264
ENSG00000087274	6564.053455	4370.325424	0.586836542	0.019455323	0.046569813	ADD1
ENSG00000284669	26.3140919	5.004470414	2.394238299	0.019468643	0.046596382	AC092053.3
ENSG00000198478	313.954451	145.2672528	1.110935632	0.01947001	0.046596382	SH3BGRL2
ENSG00000173068	166.2376107	507.5787875	-1.610856399	0.019523878	0.046720999	BNC2
ENSG00000268225	26.1018682	8.834226414	1.574498357	0.019559709	0.046802431	AC010487.1
ENSG00000134343	65.29977979	9.102316702	2.838658431	0.019566557	0.046814506	ANO3
ENSG00000173678	1.817398012	10.09751795	-2.473426077	0.019569418	0.046817039	SPDYE2B
ENSG00000164332	568.625219	839.2457941	-0.561987311	0.019580764	0.046839869	UBLCP1
ENSG00000234219	5.153938595	0	4.699244122	0.019592344	0.046863257	CDCA4P4

ENSG00000182504	621.1246646	345.0357653	0.848087916	0.019599996	0.046877243	CEP97
ENSG00000142409	568.2809502	770.5381937	-0.439024981	0.019614733	0.046908171	ZNF787
ENSG00000197837	29.35495561	9.680771646	1.601425245	0.019643151	0.046971808	HIST4H4
ENSG00000254877	5.182915058	0	4.706645041	0.019648551	0.046980398	AP001636.2
ENSG00000163624	12.17062037	2.149846562	2.498540518	0.019653785	0.046988588	CDS1
ENSG00000278709	58.72455555	21.44146917	1.460478	0.019660745	0.047000904	NKILA
ENSG00000286886	0	5.420060839	-5.018866182	0.019663325	0.047002746	AJ011931.2
ENSG00000108961	54.23127809	27.85215493	0.964564141	0.019673783	0.04702043	RANGRF
novel.505	28.54607848	11.87306815	1.266360293	0.019674343	0.04702043	-
ENSG00000121073	729.1536252	1090.130661	-0.58047487	0.019677354	0.047023302	SLC35B1
ENSG00000238260	21.475276	6.696276607	1.671668557	0.019690219	0.047049717	AL513320.1
ENSG00000129562	2291.200788	3539.481735	-0.627431544	0.019692293	0.047050347	DAD1
ENSG00000183741	1893.359132	2977.058283	-0.65299666	0.019698508	0.047060641	CBX6
ENSG00000161642	534.0354774	254.9650119	1.067297613	0.019701842	0.047060641	ZNF385A
ENSG00000142449	7.348999548	0.387220594	4.248131634	0.019702036	0.047060641	FBN3
ENSG00000253915	5.949239044	0	4.908139174	0.019715463	0.047088385	MAPRE1P1
ENSG00000187627	1.846374476	13.65332307	-2.891758555	0.01972149	0.047098451	RGPD1
ENSG00000236279	7.677485103	0.356539013	4.311013159	0.019725029	0.047102571	CLEC2L
ENSG00000184349	88.48073617	316.7747331	-1.840257451	0.0197397	0.047133274	EFNA5
ENSG00000226816	13.45948961	1.702418679	2.948494048	0.019784977	0.047237042	AC005082.1
ENSG00000198912	315.304765	441.7587238	-0.486656062	0.019815203	0.04730486	C1orf174
ENSG00000268747	9.657103052	1.00940975	3.205875957	0.019824952	0.047323784	AC022432.1
novel.536	65.31837176	116.1369559	-0.830224049	0.019880982	0.047449278	-
ENSG00000161533	877.0751662	642.5976711	0.448740335	0.019881176	0.047449278	ACOX1
ENSG00000253953	25.52939011	108.4832573	-2.088322662	0.019886645	0.04745797	PCDHGB4
novel.508	66.11012473	131.4509369	-0.993691474	0.019899758	0.047484903	-
ENSG00000188549	5126.725999	1779.7237	1.526482495	0.019911517	0.047508598	CCDC9B
ENSG00000196358	60.58744093	18.92228775	1.681086115	0.019916633	0.047516442	NTNG2
ENSG00000279623	5.084631558	0	4.68142831	0.019946707	0.047579453	AL359697.1
ENSG00000153802	5.084631558	0	4.68142831	0.019946707	0.047579453	TMPRSS11D
ENSG00000265666	74.63792863	37.60515901	0.985089958	0.019958755	0.047600795	RARA-AS1
ENSG00000137942	360.643539	687.5940544	-0.931433679	0.019959961	0.047600795	FNBP1L
ENSG00000107175	889.1764799	1202.566131	-0.435725637	0.019961151	0.047600795	CREB3
ENSG00000281903	1.818651661	12.38057493	-2.760930648	0.01999127	0.047668244	LINC02246
ENSG00000286527	4.542241382	0	4.51711123	0.020011724	0.047712637	AL161665.2
ENSG00000119139	10.01331142	31.88414295	-1.673665429	0.020037836	0.04777051	TJP2
ENSG00000174137	29.11368418	54.57230218	-0.90703102	0.020042179	0.04777648	FAM53A
ENSG00000267757	0	5.187404028	-4.952002532	0.020051646	0.047794663	EML2-AS1
ENSG00000106588	123.3098115	70.98478391	0.795465067	0.02009305	0.047885132	PSMA2
ENSG00000182899	7239.785406	11287.62993	-0.64072888	0.020093288	0.047885132	RPL35A
ENSG00000165997	451.4904192	688.2451832	-0.60789931	0.020118421	0.04794063	ARL5B
ENSG00000081377	783.1721817	461.9460412	0.762269818	0.020120947	0.047942253	CDC14B
ENSG00000105856	108.0896944	43.99269356	1.301598996	0.020132521	0.047965431	HBP1
ENSG00000166750	1596.472475	2891.493688	-0.856985351	0.020151636	0.04800657	SLFN5
ENSG00000232645	15.11807263	3.537020296	2.087168045	0.020218487	0.048161411	LINC01431
ENSG00000287910	12.40716194	1.90542139	2.742849471	0.020229896	0.048184169	AC104024.4
ENSG00000043143	1042.26028	655.9148748	0.667632032	0.020233182	0.04818758	JADE2
ENSG00000160305	1337.061954	811.3457353	0.720873976	0.020263673	0.048255774	DIP2A
novel.143	8.515623423	1.130980201	2.954802861	0.020269852	0.048266064	-
novel.89	208.8011277	89.52849013	1.2244016	0.020289081	0.048307424	-
ENSG00000075391	1246.883963	698.7508677	0.835092071	0.020292772	0.048311785	RASAL2

ENSG00000153914	2202.711971	1365.693093	0.689736225	0.020300969	0.048326871	SREK1
ENSG00000132781	479.7315199	284.9096443	0.751547824	0.02032309	0.048375099	MUTYH
ENSG00000228106	72.0638089	32.7789175	1.132290856	0.02035169	0.048438738	AL392172.1
ENSG00000144655	955.7039621	397.1492696	1.267123139	0.020356359	0.048445412	CSRNP1
ENSG00000221923	104.8109638	200.027964	-0.93243189	0.020362514	0.048455621	ZNF880
ENSG00000260630	126.3671148	82.24982423	0.619581815	0.020375165	0.04847848	SNAI3-AS1
ENSG00000267076	1.817398012	9.222731278	-2.347459428	0.020375852	0.04847848	CCDC58P3
ENSG00000255968	5.753028049	0	4.858048495	0.020395737	0.048516905	AC024145.1
ENSG00000257735	5.753028049	0	4.858048495	0.020395737	0.048516905	AC090115.1
ENSG00000100197	0	4.566499142	-4.775550422	0.020397979	0.048517797	CYP2D6
ENSG00000239998	11.64216287	0	5.87464785	0.020402642	0.048524445	LILRA2
ENSG00000150477	179.8563589	108.5281474	0.730643468	0.020418175	0.048556943	KIAA1328
ENSG00000170160	4.606533824	131.7322766	-4.838919662	0.020421903	0.048561363	CCDC144A
ENSG00000258100	6.146988423	0	4.95227632	0.020427146	0.048569385	AC025035.1
ENSG00000285825	31.54152575	13.19772285	1.253525892	0.020459536	0.048641947	CCDC15-DT
ENSG00000280048	12.63994256	2.211209725	2.533625428	0.020480096	0.048686374	AC012409.5
ENSG00000125841	921.1401745	1332.428399	-0.532564061	0.020488285	0.048701384	NRSN2
ENSG00000225411	10.94973324	2.089639274	2.365740674	0.020498572	0.04872138	CR786580.1
ENSG00000196136	16.39530303	2.231278821	2.897038817	0.02050636	0.048735433	SERPINA3
ENSG00000180245	13.12556219	1.854670712	2.845918924	0.020512697	0.048746032	RRH
ENSG00000182963	538.0537824	1065.212538	-0.985026393	0.020542069	0.048811369	GJC1
ENSG00000261783	2.125753916	10.21207193	-2.271954946	0.020560161	0.04884989	AC009054.2
ENSG00000130695	254.5113683	382.2467344	-0.587537008	0.020570457	0.048869882	CEP85
ENSG00000122176	976.854228	266.8576137	1.87173549	0.020588346	0.048907911	FMOD
ENSG00000185220	135.0944203	77.73193958	0.794567433	0.020605452	0.04894407	PGBD2
ENSG00000186583	9.776698582	0	5.622702238	0.020632279	0.049003314	SPATC1
ENSG00000273055	68.83347316	22.72624246	1.597596001	0.020652457	0.049046754	AC005046.1
ENSG00000144791	256.1375699	370.5654958	-0.533151373	0.020695778	0.049145143	LIMD1
ENSG00000140365	920.6368337	1447.006562	-0.652414557	0.020713987	0.049183888	COMMD4
novel.26	0	87.78691355	-9.036890783	0.020750377	0.049261392	-
ENSG00000111961	2534.395934	1224.273992	1.04991807	0.020750421	0.049261392	SASH1
ENSG00000233184	79.33139185	170.8581365	-1.108512157	0.020764943	0.049289101	AC093157.1
ENSG00000203805	21.34718966	94.19473963	-2.14212922	0.020767428	0.049289101	PLPP4
ENSG00000179909	140.2220315	59.76124726	1.228209184	0.020767784	0.049289101	ZNF154
ENSG00000015568	46.30937352	20.87028748	1.151971751	0.020770611	0.049291307	RGPD5
ENSG00000258230	9.074382304	1.386017859	2.680743305	0.020788603	0.049327541	AC063950.1
ENSG00000163697	968.3599829	1385.26608	-0.516728843	0.020789676	0.049327541	APBB2
ENSG00000224786	29.71617857	10.8837275	1.455481499	0.020811769	0.049375453	CETN4P
ENSG00000102710	771.6357964	1101.058983	-0.512683319	0.020817149	0.049383706	SUPT20H
ENSG00000279022	8.531992128	1.060160428	2.994238782	0.020819845	0.049385592	AL359715.4
ENSG00000166206	74.12472874	15.7987217	2.223651603	0.020829078	0.049402983	GABRB3
ENSG00000269925	19.13687111	6.359806691	1.578914864	0.020842988	0.049431462	Z98884.2
ENSG00000168924	869.418626	1340.815488	-0.624962846	0.020862836	0.049473048	LETM1
ENSG00000205578	1.773306493	12.27090123	-2.775700163	0.020864331	0.049473048	POM121B
ENSG00000117505	1101.310157	1578.547078	-0.519538393	0.020877132	0.049498884	DR1
ENSG00000179826	0	8.783347342	-5.712764007	0.020884341	0.049511458	MRGPRX3
ENSG00000117862	161.1626531	294.9744257	-0.872946719	0.020892528	0.049526347	TXNDC12
ENSG00000114378	56.45775154	26.40734245	1.08927228	0.020899877	0.04953925	HYAL1
ENSG00000242242	11.97781432	2.089639274	2.500180668	0.020910577	0.04955791	NECTIN3-AS1
ENSG00000154265	213.9022979	105.7711377	1.013618403	0.020911565	0.04955791	ABCA5
ENSG00000135469	236.5754403	147.7385904	0.680646031	0.020936505	0.04961249	COQ10A

ENSG00000260097	14.89163152	38.9459016	-1.384313281	0.020948345	0.04963602	SPDYE6
ENSG00000115687	169.6342284	268.9755288	-0.665495751	0.020953698	0.049642046	PASK
ENSG00000141252	87.92069617	179.287852	-1.027062772	0.02095471	0.049642046	VPS53
ENSG00000077943	271.3109995	56.37203107	2.265529179	0.020980376	0.049698319	ITGA8
ENSG00000188385	24.37766783	8.489584156	1.512965263	0.02098374	0.049701756	JAKMIP3
ENSG00000253434	5.922769878	0	4.902093582	0.020990374	0.049712935	LINC02237
ENSG00000100429	30.37608426	56.90623823	-0.905925555	0.020995507	0.049719848	HDAC10
ENSG00000159247	5.503878721	0	4.79313034	0.02099712	0.049719848	TUBBP5
ENSG00000287262	29.6787113	12.43260987	1.26497503	0.021003521	0.049730472	AC021148.2
ENSG00000232907	18.65340278	5.777755727	1.695703761	0.021013294	0.049749078	DLGAP4-AS1
ENSG00000266389	7.312429921	0.743759607	3.330657548	0.021043107	0.049815122	AC002091.2
ENSG00000087495	6.042436679	0.356539013	3.967442048	0.021060424	0.049851575	PHACTR3
ENSG00000232713	9.947836602	1.00940975	3.250590544	0.021076126	0.049884197	AC010733.1
ENSG00000253710	95.23201498	161.7074965	-0.762960245	0.021079664	0.049888027	ALG11
ENSG00000088543	434.9014128	260.2234113	0.742179914	0.021088836	0.049905186	C3orf18
ENSG00000123066	1663.068881	1144.263913	0.5395554	0.021102788	0.049933654	MED13L
ENSG00000286610	4.559863736	0	4.522108407	0.021123235	0.049977486	AL356433.1
ENSG00000186272	109.9844195	56.83336349	0.949801716	0.021133225	0.049996568	ZNF17
ENSG00000253372	4.55610279	0	4.521047524	0.021144115	0.050017778	AC016405.1
ENSG00000285904	6.034914786	0.336469917	3.96587566	0.021147225	0.050020579	AC006452.1
novel.486	0.925067711	9.946293394	-3.435639145	0.021164134	0.050056018	-
ENSG00000141391	269.6925235	144.1705502	0.902480317	0.02116931	0.050063704	PRELID3A
ENSG00000273483	17.11029834	3.281982638	2.392101362	0.021195857	0.05011754	AL354760.1
ENSG00000006555	8.924770125	0	5.490790079	0.021196962	0.05011754	TTC22
ENSG00000259430	6.788631014	0	5.098733197	0.021197862	0.05011754	CERS3-AS1
ENSG00000149179	431.2113133	595.7519763	-0.466120207	0.021201483	0.05012154	C11orf49
ENSG00000143382	216.4156171	523.693538	-1.275078387	0.021214613	0.050148017	ADAMTSL4
ENSG00000108688	0.308355904	6.381031662	-4.294895059	0.021218698	0.050153111	CCL7
ENSG00000229727	6.109236418	0.387220594	3.98140953	0.021256941	0.050235804	AC013460.1
ENSG00000115129	577.6234288	1004.82181	-0.799033188	0.021257551	0.050235804	TP53I3
ENSG00000198182	161.7694504	90.6103891	0.83868895	0.021276114	0.050272012	ZNF607
ENSG00000258890	738.2445069	459.5016736	0.684198181	0.021276743	0.050272012	CEP95
ENSG00000288009	13.05661116	2.169915658	2.592725468	0.021295802	0.050312471	AL353135.2
ENSG00000163785	854.3919665	1210.554138	-0.502704701	0.021298054	0.050313216	RYK
ENSG00000198624	282.8325712	119.1955284	1.245155218	0.021313586	0.050345329	CCDC69
ENSG00000138771	1599.710541	729.380963	1.133388621	0.021320026	0.050355962	SHROOM3
ENSG00000180730	75.33719563	465.0631266	-2.626104253	0.021373763	0.050478296	SHISA2
ENSG00000211698	4.514518568	0	4.509217775	0.02137645	0.050480053	TRGV4
ENSG00000110330	1696.442362	2462.218897	-0.537328325	0.021381965	0.050488487	BIRC2
ENSG00000261036	0	4.42241945	-4.721665961	0.021388433	0.050499172	AC113418.1
ENSG00000255866	0	5.696195073	-5.085240775	0.021430818	0.050594645	AC090023.1
ENSG00000261327	8.286603747	31.07393028	-1.910041046	0.0214469	0.050628013	AC134312.5
ENSG00000183831	11.51601402	1.069617038	3.422570689	0.021452907	0.050637591	ANKRD45
ENSG00000264659	5.781933241	0	4.864782827	0.021466132	0.050664205	AC064805.2
ENSG00000169047	1371.716989	2834.87638	-1.047245473	0.021477797	0.050687133	IRS1
ENSG00000254717	0.308355904	5.410604229	-4.054245351	0.021491751	0.050714196	GLYATL1P2
ENSG00000272918	28.64149868	8.650136924	1.729084533	0.021493169	0.050714196	AC005070.3
ENSG00000232630	27.35639038	9.843636164	1.483242869	0.021501586	0.050725975	PRPS1P2
ENSG00000197696	70.18770251	144.5332538	-1.044071056	0.021502065	0.050725975	NMB
ENSG00000254719	4.538480436	18.07933854	-1.992316106	0.021520765	0.050764926	AC080023.2
ENSG00000108389	778.960078	1485.393637	-0.931026078	0.021522484	0.050764926	MTMR4

ENSG00000267882	1.49643435	9.904999327	-2.714093358	0.021536629	0.050792552	AL031666.2
ENSG00000167604	270.4443172	114.2606278	1.244532726	0.021538106	0.050792552	NFKBID
ENSG00000053254	924.5717922	609.0441237	0.602502353	0.02154896	0.050809938	FOXN3
ENSG00000136146	739.1195114	540.4192315	0.451300268	0.02154939	0.050809938	MED4
ENSG00000180509	8.153075539	0.713078025	3.508535843	0.021555369	0.050819422	KCNE1
novel.260	14.06456258	2.527610546	2.481879457	0.02156737	0.050843103	-
ENSG00000128335	1248.596627	787.7257714	0.66430009	0.021591458	0.050895271	APOL2
ENSG00000164346	1305.11394	1924.377122	-0.560400308	0.021595046	0.050899109	NSA2
novel.495	12.40332972	2.782648203	2.134022692	0.021617541	0.050944057	-
ENSG00000235576	4.895942455	0	4.623930178	0.021619184	0.050944057	LINC01871
ENSG00000254893	14.02200944	37.61936752	-1.429185235	0.021619998	0.050944057	AC113404.3
ENSG00000254348	5.732827126	0.336469917	3.891856887	0.021634052	0.050972551	AC120036.3
ENSG00000250608	35.21246413	10.77289792	1.698283302	0.021638521	0.050978456	AC010210.1
ENSG00000230555	39.42295721	14.33931553	1.457583156	0.021640739	0.05097906	AL450326.1
ENSG00000253741	26.27794955	4.394178013	2.559079143	0.021657383	0.051013641	LNCOC1
ENSG00000189164	145.4856515	86.42337058	0.749695593	0.021661044	0.051017641	ZNF527
ENSG00000134987	534.1279087	763.4951383	-0.515569035	0.021665386	0.05102324	WDR36
ENSG00000173915	1319.204473	1851.56522	-0.489184479	0.021699549	0.051099066	ATP5MD
ENSG00000261652	24.60041926	48.08146842	-0.968067942	0.021703402	0.051103507	C15orf65
ENSG00000140829	1149.498248	1677.826166	-0.545515652	0.021710575	0.051115764	DHX38
ENSG00000166166	610.3637463	375.4622319	0.700239838	0.021719706	0.051132628	TRMT61A
novel.459	14.56439962	32.05163097	-1.141292303	0.021733611	0.051160728	-
ENSG00000074317	0	7.824816664	-5.54698304	0.021742334	0.051176624	SNCB
ENSG00000180938	45.24124661	22.79821811	0.984548644	0.021748167	0.051185716	ZNF572
ENSG00000147082	13.83930385	2.762579107	2.301538775	0.021754391	0.051195727	CCNB3
ENSG00000100206	19.27763648	48.0770205	-1.32029354	0.021758866	0.051201622	DMC1
ENSG00000149476	364.9210181	561.4294547	-0.622108585	0.021763745	0.051204938	TKFC
ENSG00000238280	9.810832183	1.080229524	3.183878902	0.021764217	0.051204938	AC067751.1
ENSG00000091262	17.40013425	4.310305609	2.012099898	0.021786518	0.051252764	ABCC6
ENSG00000256162	6.961236146	0.743759607	3.261886856	0.021798942	0.051277348	SMLR1
ENSG00000131503	71.47571755	34.33366047	1.057858221	0.021803655	0.05128379	ANKHD1
ENSG00000150687	3306.453628	1642.578787	1.009229357	0.021809068	0.051291879	PRSS23
novel.43	9.525113227	0.693008929	3.749268408	0.021811298	0.051292482	-
ENSG00000110801	151.6360731	239.1571032	-0.65767923	0.021826401	0.051323352	PSMD9
ENSG00000249816	0.909952655	8.131760874	-3.155112248	0.021831812	0.051331429	LINC00964
ENSG00000171121	44.26456549	18.35688544	1.263871516	0.021839718	0.051345372	KCNMB3
ENSG00000268592	13.11198552	39.77965099	-1.602238773	0.021847164	0.051358231	AL355312.3
ENSG00000137975	0.909952655	7.693789602	-3.076546234	0.021861109	0.051386363	CLCA2
ENSG00000241975	20.39390059	5.492036488	1.903896638	0.021869634	0.051400956	ELOCP19
ENSG00000198060	615.1371434	854.2908672	-0.47413137	0.02187215	0.051400956	5-Mar
ENSG00000174945	33.31372079	8.650136924	1.945514596	0.021873252	0.051400956	AMZ1
novel.370	22.8409029	6.867441861	1.735892444	0.021878777	0.051409289	-
ENSG00000122085	585.1540232	413.3132631	0.501117623	0.021887292	0.051420933	MTERF4
ENSG00000163320	2363.094007	1715.499961	0.461991874	0.021887691	0.051420933	CGGBP1
ENSG00000104695	2871.122197	2049.015499	0.486542974	0.021951949	0.051567233	PPP2CB
ENSG00000167094	14.10761391	2.51699806	2.485919595	0.021962678	0.051587771	TTC16
ENSG00000125538	1030.021676	14299.55512	-3.795223841	0.02197751	0.051617943	IL1B
ENSG00000279214	4.832974932	0	4.606957213	0.021988264	0.051629199	AL109659.3
ENSG00000231748	4.832974932	0	4.606957213	0.021988264	0.051629199	AL596223.2
ENSG00000184617	4.832974932	0	4.606957213	0.021988264	0.051629199	ZNF840P
ENSG00000123106	429.2401427	292.1627185	0.554981751	0.021990376	0.051629491	CCDC91

ENSG00000164488	11.93518991	0.336469917	4.948599162	0.021999532	0.051646322	DACT2
ENSG00000214184	3.678887545	15.34628514	-2.063468109	0.022024009	0.051699113	GCC2-AS1
ENSG00000100246	237.7016192	354.2038122	-0.574743863	0.022064178	0.051788727	DNAL4
ENSG00000163872	1329.16613	2135.736469	-0.684132244	0.022147048	0.051978542	YEATS2
ENSG00000117400	9.011414781	1.436768536	2.65112675	0.022155109	0.051992764	MPL
ENSG00000228495	4.573725143	0	4.5260607	0.022159064	0.051994383	LINC01013
ENSG00000279148	1.521649867	10.38554894	-2.774553186	0.022159802	0.051994383	AC126474.1
ENSG00000130300	497.4576028	41.11894655	3.595677803	0.022162109	0.051995103	PLVAP
ENSG00000260804	263.8529214	142.0717111	0.891403855	0.022205062	0.052091171	LINC01963
ENSG00000196787	10.34186825	29.56544288	-1.520705283	0.0222275	0.0521391	HIST1H2AG
ENSG00000282556	8.179544705	1.130980201	2.897826482	0.022287009	0.052273272	AC068733.3
ENSG00000058063	688.2321258	987.5730533	-0.521040391	0.022288723	0.052273272	ATP11B
ENSG00000272509	10.77085987	1.406086955	2.92322513	0.022293798	0.052280456	AC087752.4
novel.59	9.720070575	1.456837632	2.748400123	0.022298769	0.052287393	-
ENSG00000167182	464.2224483	325.1515739	0.513759284	0.022304723	0.052296634	SP2
ENSG00000179219	0	4.280779903	-4.679947263	0.022376383	0.052459917	LINC00311
ENSG00000286018	39.12037135	15.77377232	1.310655403	0.022403539	0.052518844	AF129075.3
ENSG00000154370	464.2302983	676.9981702	-0.544015455	0.022406479	0.052520997	TRIM11
novel.721	76.25492778	37.9685861	1.008610878	0.022427943	0.052566565	-
ENSG00000147535	508.3200209	761.8589763	-0.584224742	0.022439185	0.052588172	PLPP5
ENSG00000265752	10.84643515	1.702418679	2.633728675	0.02245276	0.052611861	AC010754.1
ENSG00000203362	14.46611612	32.21321122	-1.150435035	0.022453343	0.052611861	POLH-AS1
ENSG00000260193	15.7021183	3.537020296	2.142678748	0.022490581	0.052694362	AL138781.1
ENSG00000245970	36.52404159	16.27670277	1.160191645	0.022500001	0.052711679	AP003352.1
ENSG00000214914	4.513264919	0	4.508838161	0.022510078	0.052726154	RPL23AP3
ENSG00000262454	25.56295424	54.82361542	-1.09675219	0.022510238	0.052726154	MIR193BHG
ENSG00000205221	630.2199221	29.08160122	4.436670842	0.022513022	0.05272792	VIT
ENSG00000170074	11.09830524	0	5.805416795	0.022518357	0.052735662	FAM153A
ENSG00000271967	13.134409	2.527610546	2.38404695	0.022532119	0.052763136	AL583856.2
ENSG00000187240	473.4266851	781.5784453	-0.723012456	0.022552196	0.05280539	DYNC2H1
ENSG00000140983	1793.335471	2607.452074	-0.539861429	0.022557764	0.052813668	RHOT2
ENSG00000259518	0	4.799027558	-4.840100641	0.022589234	0.052877816	LINC01583
ENSG00000231123	0	4.799027558	-4.840100641	0.022589234	0.052877816	SPATA20P1
ENSG00000120800	439.9725824	760.8943276	-0.790569973	0.022599298	0.052896609	UTP20
ENSG00000275278	7.191509471	0.356539013	4.219457327	0.022630042	0.052963799	AC012150.2
ENSG00000259248	22.66328317	8.876804751	1.346331011	0.02263263	0.052965085	USP3-AS1
ENSG00000103490	404.9630088	229.2288243	0.821504419	0.022639226	0.052975747	PYCARD
ENSG00000168646	57.89268548	24.33892814	1.244599681	0.02265306	0.053003346	AXIN2
ENSG00000157152	2.728604316	12.42443753	-2.190254097	0.022678906	0.053059043	SYN2
novel.489	36.72820176	73.09462067	-0.995349293	0.022686096	0.053071084	-
ENSG00000286787	22.00380477	6.725802314	1.703590183	0.022722387	0.053151197	AL110114.1
ENSG00000160050	136.8176517	219.2620763	-0.681204675	0.022732195	0.053169352	CCDC28B
ENSG00000275426	19.7650083	3.129730605	2.64486177	0.022738241	0.053178705	AC253576.2
ENSG00000129250	6452.250196	4576.865538	0.495481408	0.022745931	0.053191903	KIF1C
ENSG00000179476	161.0607946	75.66882512	1.092714578	0.022749279	0.053194943	C14orf28
ENSG00000249915	133.2638885	273.9158919	-1.038742588	0.022761564	0.053215583	PDCD6
ENSG00000133392	5.527769318	0	4.798978119	0.022762202	0.053215583	MYH11
ENSG00000280140	5.99709151	0.336469917	3.957866158	0.022792578	0.053281804	AC098679.4
ENSG00000286389	5.521501074	0	4.797459781	0.022799924	0.053294182	AL731702.1
ENSG00000224728	30.87535183	10.02900992	1.622999525	0.022840319	0.053383801	IMPDH1P8
ENSG00000158201	354.6652258	188.8889077	0.907687556	0.0228426	0.05338433	ABHD3

ENSG00000179855	137.4342648	39.82886061	1.790083128	0.022854324	0.053406926	GIPC3
ENSG00000124102	0.89358395	13.86693826	-3.944094264	0.022864064	0.053424881	PI3
ENSG00000217094	41.09679782	77.4341952	-0.91553891	0.022868864	0.053427673	PPIAP31
ENSG00000213876	4.855611881	0	4.613075679	0.022869372	0.053427673	RPL7AP64
ENSG00000213809	16.30650708	0.713078025	4.509459939	0.022871732	0.053428381	KLRK1
ENSG00000255198	37.77161135	15.11144497	1.321469372	0.022875801	0.053432266	SNHG9
ENSG00000176401	96.40393818	56.00804323	0.78126636	0.022877508	0.053432266	EID2B
ENSG00000167173	331.7415101	467.8586896	-0.495678612	0.022909512	0.053502205	C15orf39
ENSG00000143340	6.131944638	0	4.949018304	0.022916133	0.053512856	FAM163A
ENSG00000225937	4.84683634	0	4.610710054	0.022924547	0.053521396	PCA3
ENSG00000002726	4.84683634	0	4.610710054	0.022924547	0.053521396	AOC1
ENSG00000285863	13.34197411	1.069617038	3.637007538	0.02292597	0.053521396	AC009554.2
ENSG00000250602	15.58614117	2.792104814	2.466104243	0.022945897	0.053563013	AC093535.1
ENSG00000213073	16.76071422	45.17570871	-1.429125719	0.022947919	0.053563013	AL353625.1
ENSG00000243317	753.6182495	1159.705377	-0.622112278	0.022973897	0.05361883	STMP1
ENSG00000255085	5.826096032	0	4.87509149	0.02298255	0.053634209	AF186192.2
ENSG00000247950	52.05376822	26.86525442	0.953668276	0.022987939	0.053641965	SEC24B-AS1
ENSG00000248092	271.7645399	164.633548	0.721430083	0.022993186	0.053648844	NNT-AS1
ENSG00000101901	683.0247785	484.8605428	0.494318829	0.022995017	0.053648844	ALG13
ENSG00000170464	537.8367695	376.3458204	0.515691223	0.023025928	0.053716138	DNAJC18
ENSG00000140527	10.78096033	1.436768536	2.909463393	0.023033176	0.053728224	WDR93
ENSG00000163444	814.3094607	1103.309831	-0.438256394	0.023043706	0.053747959	TMEM183A
novel.196	138.5112663	65.36300463	1.081329666	0.023046899	0.053750582	-
ENSG00000283443	10.87763417	0	5.777963443	0.023049222	0.053751175	AC018688.1
ENSG00000259498	0.616711807	9.648805795	-3.97766256	0.023088186	0.053837208	TPM1-AS
ENSG00000116691	674.3541445	442.349251	0.608217267	0.023113574	0.053890599	MIIP
ENSG00000285480	6.306701063	0.336469917	4.030064466	0.023115232	0.053890599	AC021097.2
ENSG00000198221	0.616711807	8.293597912	-3.763532304	0.023152539	0.053972734	AFDN-DT
ENSG00000170835	21.54576541	7.888619972	1.448731301	0.023157397	0.053979215	CEL
ENSG00000031698	1922.819946	2892.92145	-0.589435475	0.023162638	0.053986588	SARS
ENSG00000164171	713.9293803	1420.797731	-0.992651545	0.023167877	0.053993956	ITGA2
ENSG00000148288	239.4862357	117.7017162	1.023701221	0.023189875	0.054040376	GBGT1
ENSG00000224397	16.80731304	3.72941052	2.191272372	0.023194906	0.054044111	SMIM25
ENSG00000254057	0	4.768345977	-4.831771104	0.023195638	0.054044111	AC084346.1
ENSG00000143740	383.9453734	667.5240444	-0.798098886	0.023212479	0.0540785	SNAP47
ENSG00000271918	61.51193952	25.21499908	1.280681515	0.023231632	0.054118267	AC034236.2
ENSG00000181315	270.848604	167.2813976	0.694413634	0.023248698	0.054153167	ZNF322
novel.230	6.494136517	0	5.034811432	0.02325147	0.054154769	-
ENSG00000223834	17.36578718	2.679862578	2.728197911	0.023274875	0.054204421	AL161935.1
ENSG00000270059	7.869935152	1.049547942	2.882814394	0.023281221	0.05421292	AC121493.1
ENSG00000123728	601.1318305	447.0109926	0.427618229	0.023282698	0.05421292	RAP2C
ENSG00000074855	765.1904535	440.4158665	0.797620447	0.023286626	0.054216096	ANO8
ENSG00000244187	255.2804843	397.2711096	-0.638462077	0.02329	0.054216096	TMEM141
ENSG00000229321	15.14676436	1.762625967	3.094165963	0.023290322	0.054216096	AC008269.1
ENSG00000230124	713.9389496	951.7586467	-0.414880727	0.023312397	0.054262622	ACBD6
ENSG00000177732	662.2505651	1172.542388	-0.823907841	0.023327676	0.054293321	SOX12
ENSG00000238121	12.26374673	1.406086955	3.109070971	0.023335038	0.054305592	LINC00426
ENSG00000181656	25.87549802	2.11916498	3.603051149	0.023347169	0.054327976	GPR88
ENSG00000164741	273.1650283	162.1459001	0.75212065	0.023348839	0.054327976	DLC1
ENSG00000134897	318.1698318	466.5398955	-0.552223005	0.023364145	0.054358722	BIVM
ENSG00000259359	8.047270146	0.743759607	3.469803966	0.023375635	0.054380586	AC012409.1

ENSG00000113360	748.2623466	1070.940399	-0.517505495	0.023386503	0.054400998	DROSHA
ENSG00000255737	21.18454245	7.062272231	1.575781216	0.023406139	0.054441799	AGAP2-AS1
ENSG00000115252	277.0739146	45.82006878	2.594373962	0.023426738	0.054484834	PDE1A
ENSG00000171984	75.64093673	40.73707297	0.891664584	0.023444695	0.054521716	SHLD1
ENSG00000249806	5.629529031	0	4.828869008	0.023455705	0.054541076	AC139720.1
ENSG00000266947	53.59171552	16.62622532	1.680684271	0.023457436	0.054541076	AC022916.1
ENSG00000168116	139.0236399	229.6604211	-0.724219182	0.023460447	0.054541076	KIAA1586
ENSG00000182511	607.1139499	284.921939	1.09049993	0.023461417	0.054541076	FES
ENSG00000154642	225.4803106	133.7632156	0.75320513	0.023487173	0.054596066	C21orf91
ENSG00000173905	1007.654822	1869.109088	-0.891489883	0.023489516	0.054596629	GOLIM4
ENSG00000148120	859.4157279	369.7841921	1.216070677	0.023509	0.054635887	AOPEP
novel.60	22.39873406	6.45069556	1.797159836	0.023510612	0.054635887	-
ENSG00000100918	386.7294149	112.8917382	1.776317282	0.023513341	0.054637341	REC8
ENSG00000196387	247.9656618	161.2505307	0.621705723	0.023518423	0.054644263	ZNF140
ENSG00000008952	3605.933767	2526.033206	0.51345456	0.023551532	0.054716298	SEC62
ENSG00000165632	332.9542621	231.2909925	0.525065756	0.023558745	0.054724907	TAF3
ENSG00000164190	1301.522353	911.2254964	0.514480267	0.023559451	0.054724907	NIPBL
ENSG00000117877	51.60206839	111.6705923	-1.115932842	0.023569059	0.054742332	CD3EAP
ENSG00000127884	1459.424294	2283.938862	-0.646227555	0.023572674	0.054745835	ECHS1
ENSG00000170370	20.01555347	1.365948763	3.84932666	0.023574939	0.0547462	EMX2
ENSG00000235545	10.0700107	1.069617038	3.227770167	0.023585602	0.054764303	AC103923.1
ENSG00000268584	14.43463236	3.903015919	1.880655612	0.02358695	0.054764303	AC073389.1
ENSG00000287619	10.80359728	2.191140629	2.316957495	0.023596324	0.054781171	AL021308.1
ENSG00000226002	19.45790604	4.555886656	2.082450356	0.023670818	0.054949207	GTF2IP14
ENSG00000185504	668.0019835	919.7474579	-0.46139276	0.02369746	0.055006138	FAAP100
ENSG00000237980	4.574978792	0	4.526450167	0.023716922	0.055031681	AL590648.3
ENSG00000224238	4.574978792	0	4.526450167	0.023716922	0.055031681	WARS2-IT1
ENSG00000277288	4.574978792	0	4.526450167	0.023716922	0.055031681	C10orf142
ENSG00000224429	28.66288198	10.92502156	1.399154575	0.023716937	0.055031681	LINC00539
ENSG00000133640	6.089106767	23.55973506	-1.954858984	0.023748492	0.055084194	LRRIQ1
ENSG00000197872	483.4305027	133.1554573	1.85922835	0.023749311	0.055084194	FAM49A
ENSG00000100249	4.569964197	0	4.525028875	0.023750169	0.055084194	C22orf31
ENSG00000228339	4.569964197	0	4.525028875	0.023750169	0.055084194	AMD1P1
ENSG00000102290	4.569964197	0	4.525028875	0.023750169	0.055084194	PCDH11X
ENSG00000232040	0	4.656232136	-4.800827298	0.02380761	0.055212489	ZBED9
ENSG00000138413	829.4441986	1377.152861	-0.731623609	0.023830671	0.055261038	IDH1
ENSG00000090263	394.2707022	609.9211669	-0.629878624	0.023849783	0.055300263	MRPS33
ENSG00000164118	478.7485977	332.8615327	0.525174509	0.023851843	0.055300263	CEP44
ENSG00000285632	31.03653158	7.510727594	2.061919165	0.023855918	0.055303847	AC084024.4
ENSG00000175582	3732.220583	2598.783126	0.522332381	0.023857647	0.055303847	RAB6A
ENSG00000168405	173.7032719	43.04328117	2.010019436	0.023915547	0.055433119	CMAHP
ENSG00000126768	490.9914597	692.872044	-0.497072995	0.023931768	0.055465767	TIMM17B
novel.166	24.02529167	4.770786122	2.311872583	0.023953839	0.05551197	-
ENSG00000150764	1189.416172	677.6797612	0.811358942	0.023975891	0.055558119	DIXDC1
ENSG00000141994	283.9928719	420.8356778	-0.567559954	0.023988228	0.05558175	DUS3L
novel.795	59.39642826	117.5805313	-0.984490666	0.024000386	0.055604961	-
ENSG00000091490	317.2960764	1094.245917	-1.78625694	0.024018054	0.055640933	SEL1L3
ENSG00000185104	630.658608	925.3001809	-0.553333483	0.024025917	0.055654186	FAF1
ENSG00000137473	0.307102255	5.911222933	-4.18828424	0.024032	0.055662713	TTC29
ENSG00000287307	5.18166141	0	4.70640044	0.024033883	0.055662713	AC005772.2
ENSG00000231920	11.15479105	1.345879667	3.006737051	0.024037653	0.055666483	NEBL-AS1

ENSG00000154478	5.520247426	0	4.797187975	0.024044964	0.055678451	GPR26
ENSG00000238165	9.892390973	1.823989131	2.454011665	0.024051661	0.055688994	AC007560.1
ENSG00000101850	3.60707321	13.97331991	-1.949932828	0.024060123	0.055703623	GPR143
ENSG00000253161	11.98812824	58.8431931	-2.296264124	0.024083103	0.055751857	LINC01605
ENSG00000232855	18.44966989	3.088436538	2.55755196	0.0240984	0.055782299	AF165147.1
ENSG00000143751	447.5370524	631.7580974	-0.497151921	0.024116587	0.055819426	SDE2
ENSG00000170921	899.2709103	1860.391146	-1.048629479	0.024148418	0.055888122	TANC2
ENSG00000120820	705.1412935	339.6862806	1.053408531	0.02415621	0.055901176	GLT8D2
ENSG00000268199	0	4.800183434	-4.840319135	0.024190012	0.055974413	AC010335.1
ENSG00000155959	722.7551787	1091.113486	-0.594482284	0.024199959	0.055992442	VBP1
ENSG00000273403	4.50065716	0	4.505178651	0.024216458	0.056025628	AC107294.3
ENSG00000169427	4.499403511	0	4.504819563	0.024225015	0.056040434	KCNK9
ENSG00000255284	99.30618782	148.4388726	-0.579049665	0.024300165	0.056205312	AP006621.3
ENSG00000091704	7.062026944	0.356539013	4.190384852	0.024300615	0.056205312	CPA1
ENSG00000260588	24.35495961	6.624300959	1.865159228	0.02433536	0.056280665	AC027702.1
ENSG00000162692	428.4988021	90.3590415	2.244505642	0.024338192	0.056281056	VCAM1
ENSG00000223855	28.75142103	6.73513053	2.106922793	0.024339862	0.056281056	AC147651.1
ENSG00000105518	528.5749792	287.652527	0.878665982	0.02434873	0.056296553	TMEM205
novel.141	0	4.882899962	-4.872487154	0.024378755	0.056358115	-
ENSG00000182552	303.7051831	453.3727727	-0.57864008	0.024379695	0.056358115	RWDD4
ENSG00000159761	147.6879775	76.70652348	0.947653037	0.024382623	0.05635987	C16orf86
ENSG00000253549	9.022768891	1.029478846	3.095989438	0.024398299	0.056391087	CA3-AS1
ENSG00000074319	948.5154103	1362.130426	-0.522242928	0.0244117	0.056417042	TSG101
ENSG00000288022	1.17296339	11.89185298	-3.31953844	0.02444091	0.056479523	AC117488.1
ENSG00000250012	23.49662036	4.801467703	2.272487631	0.024455002	0.056507062	AC079848.1
ENSG00000197226	210.5452008	338.4491152	-0.684897911	0.024476147	0.05655089	TBC1D9B
ENSG00000063322	1605.757262	1165.348159	0.46228341	0.024498612	0.056597763	MED29
ENSG00000236352	6.550835796	0.356539013	4.085902249	0.024513557	0.056627255	AC005220.1
ENSG00000119912	440.6340546	669.7505707	-0.604463544	0.024530834	0.056661661	IDE
ENSG00000272733	8.784973673	0.672939833	3.651244395	0.024533286	0.056661661	AP000345.2
ENSG00000185532	92.42890305	264.2578738	-1.515485314	0.024534994	0.056661661	PRKG1
ENSG00000253720	5.718965719	0.387220594	3.888762749	0.024543794	0.056676946	AC022973.2
ENSG00000205424	0	7.671408756	-5.519686469	0.024553301	0.056693859	AL592528.1
ENSG00000101096	468.117989	159.8796077	1.550913259	0.024565674	0.056717389	NFATC2
ENSG00000052126	477.6654263	294.6554907	0.697685897	0.024568528	0.056718937	PLEKHA5
ENSG00000272402	17.62657535	5.83911889	1.603400755	0.024624771	0.056841358	AL031775.2
ENSG00000245864	6.832722533	0.356539013	4.146775501	0.024625932	0.056841358	MEF2C-AS2
ENSG00000145757	16.32760565	5.737617535	1.508665069	0.024640381	0.056869658	SPATA9
ENSG00000270558	22.06043277	9.029056784	1.293319914	0.024646069	0.056877734	AC025449.1
ENSG00000124357	2184.112467	1623.785866	0.427553045	0.024655087	0.056893492	NAGK
ENSG00000164342	272.5461924	86.55161916	1.653127695	0.024688151	0.056964729	TLR3
ENSG00000029363	1950.552832	2610.935772	-0.420759492	0.024708974	0.057007712	BCLAF1
ENSG00000170677	1364.54234	761.5561035	0.841709726	0.024722409	0.057033644	SOCS6
novel.99	28.17719109	12.55546459	1.165784051	0.024738202	0.057065012	-
ENSG00000116688	1891.45379	2534.030416	-0.422061202	0.024762569	0.057116149	MFN2
ENSG00000283141	7.149925249	0.356539013	4.211703501	0.024828621	0.057263419	LINC02666
ENSG00000272902	21.03869122	6.899279318	1.613468205	0.024836395	0.057276263	TBC1D8-AS1
novel.378	0.922560414	10.57665489	-3.523387052	0.024838785	0.057276691	-
ENSG00000165633	556.2446077	203.7876238	1.448811671	0.024841881	0.057278746	VSTM4
ENSG00000173237	0	4.821536799	-4.856068191	0.024853527	0.057300514	C11orf86
ENSG00000228107	10.93719676	1.793307549	2.607077395	0.024874126	0.057342917	AP000692.1

ENSG00000099377	138.5161384	78.87455975	0.811475806	0.024890298	0.057375107	HSD3B7
ENSG00000236882	17.7286198	4.575955752	1.941810987	0.024921545	0.057438398	LINC01554
ENSG00000156502	352.4426273	534.4930297	-0.600432306	0.02492491	0.057438398	SUPV3L1
ENSG00000175040	827.6010703	2498.122042	-1.593913824	0.024925122	0.057438398	CHST2
ENSG00000205238	11.7425264	36.23407317	-1.620488289	0.024926598	0.057438398	SPDYE2
ENSG00000105771	607.5286525	818.2793522	-0.429808828	0.0249649	0.057521557	SMG9
ENSG00000279175	0.308355904	5.248895586	-4.016771133	0.024978489	0.057547762	AL033543.1
ENSG00000177272	7.37790474	0.387220594	4.253545968	0.024982954	0.057547844	KCNA3
ENSG00000156171	906.9802043	627.2727737	0.532279219	0.024982954	0.057547844	DRAM2
ENSG00000241634	4.282991593	16.1515363	-1.92033191	0.024987738	0.05755376	AC069499.1
ENSG00000023228	127.5820181	267.1376226	-1.065756408	0.025010607	0.057601328	NDUFS1
ENSG00000243147	724.7664657	997.7648	-0.461089991	0.025014458	0.05760509	MRPL33
ENSG00000245849	247.0468345	130.1433503	0.92574477	0.025016727	0.05760521	RAD51-AS1
ENSG00000135253	13.33473695	49.86707035	-1.900339377	0.025029353	0.057629175	KCP
novel.304	366.3336013	205.721719	0.832756947	0.02505315	0.057678856	-
ENSG00000265810	5.367771944	0	4.759621404	0.02505619	0.057679879	MIR3907
ENSG00000227500	1114.386858	1578.082022	-0.501837635	0.025058412	0.057679879	SCAMP4
ENSG00000246082	42.76777513	19.13847149	1.155912867	0.025060255	0.057679879	NUDT16P1
ENSG00000166329	5.362757349	0	4.75837637	0.025090508	0.057744396	CCDC182
ENSG00000279901	0.601596751	6.063474966	-3.328915348	0.025093592	0.057746378	AC092117.2
ENSG00000142875	886.2984887	520.4749478	0.767665723	0.025106541	0.057771061	PRKACB
ENSG00000131480	11.25307455	29.90648912	-1.411204032	0.025128845	0.057817262	AOC2
ENSG00000115163	127.0774084	335.5319855	-1.401239085	0.025143834	0.057846625	CENPA
ENSG00000284237	19.52330959	4.178122672	2.208951942	0.025178171	0.057920493	AL356275.1
ENSG00000286938	9.984334959	1.742556871	2.495538256	0.025184662	0.057930298	AC092849.3
ENSG00000008196	8.623936114	0	5.441296285	0.025187264	0.057931153	TFAP2B
ENSG00000238000	17.9223235	38.63553694	-1.107872107	0.025190068	0.057932473	AC116347.1
ENSG00000112290	266.7801734	421.6592343	-0.659997308	0.025211578	0.057974717	WASF1
ENSG00000258017	32.99441022	7.867395001	2.068456928	0.025212899	0.057974717	AC011603.2
ENSG00000287427	5.216906117	0	4.715471618	0.025223843	0.057994749	AL390236.1
ENSG00000148965	0	7.529640814	-5.4908637	0.025226251	0.057995152	SAA4
ENSG00000119707	3505.473877	2250.478698	0.639390322	0.025243184	0.058028947	RBM25
ENSG00000279917	0	3.9336975	-4.557743589	0.02524958	0.058038515	AC079331.2
ENSG00000277879	37.4327406	17.51979682	1.094378353	0.025257161	0.058050805	AL391988.1
ENSG00000151876	176.6629763	110.5272018	0.674957849	0.025321696	0.058193983	FBXO4
ENSG00000171135	569.061492	793.253918	-0.479127179	0.025333958	0.058217015	JAGN1
ENSG00000237491	64.72218905	26.13925216	1.308361634	0.025351062	0.058251166	AL669831.5
ENSG00000204572	10.41207293	0.774441189	3.807608949	0.025388529	0.058332099	KRTAP5-10
ENSG00000131051	5389.32669	4061.317295	0.408104218	0.025395387	0.058342696	RBM39
ENSG00000260196	17.70591158	44.73239043	-1.341986798	0.0254035	0.058353456	AC124798.1
ENSG00000279320	20.14628964	4.984401318	2.010599865	0.025404563	0.058353456	AC069528.2
ENSG00000267251	8.192152463	1.467450118	2.499853571	0.025434546	0.058417163	AC139100.1
ENSG00000260339	10.3833812	1.406086955	2.867460773	0.025452111	0.058452339	HEXA-AS1
ENSG00000176542	383.6272601	239.419063	0.680805047	0.025494453	0.058544403	USF3
ENSG00000162377	308.655361	483.6474065	-0.648579214	0.025524984	0.058609335	COA7
ENSG00000286289	8.306804669	0	5.387253618	0.025528959	0.058613281	AL133482.1
ENSG00000225511	165.8759609	66.86370486	1.308280676	0.025551837	0.058660623	LINC00475
ENSG00000151498	677.9505036	480.2230483	0.497644533	0.025566121	0.058688231	ACAD8
novel.772	5.824842383	0	4.874857128	0.025582085	0.058719688	-
ENSG00000142634	1113.677912	1858.452928	-0.738845068	0.025585819	0.05872307	EFHD2
ENSG00000175467	1498.989005	2021.294603	-0.431152133	0.025594088	0.05873686	SART1

ENSG00000095485	390.8061191	556.9517427	-0.511280235	0.025625578	0.058799391	CWF19L1
ENSG00000120690	908.8208796	586.311648	0.631944339	0.025625861	0.058799391	ELF1
ENSG00000280213	20.03518492	49.61986671	-1.306030055	0.02565144	0.058852884	UCKL1-AS1
ENSG00000088256	1463.470616	2202.435854	-0.589766232	0.02566443	0.058877488	GNA11
ENSG00000173674	1439.28416	2282.574929	-0.665369428	0.025668759	0.058882219	EIF1AX
ENSG00000090686	1096.980528	1522.87124	-0.473071321	0.025687875	0.058920868	USP48
ENSG00000106266	1022.992388	1440.853093	-0.493931842	0.025696144	0.058934632	SNX8
ENSG00000229116	7.932902674	0.672939833	3.500135924	0.025709657	0.058960419	AL137026.1
ENSG00000107816	2310.358234	3064.786015	-0.407596727	0.025717956	0.058974247	LZTS2
ENSG00000110906	1404.551175	2011.149018	-0.517946842	0.025725872	0.058987192	KCTD10
ENSG00000177463	1127.435634	736.2732037	0.614896651	0.025731601	0.058995121	NR2C2
ENSG00000080709	5.44968674	0.336469917	3.8181183	0.025751895	0.05903644	KCNN2
ENSG00000155428	24.76006063	8.804829102	1.482644886	0.025765501	0.059062422	TRIM74
ENSG00000213949	1526.440886	1068.275631	0.514700352	0.025801912	0.059140668	ITGA1
novel.175	12.94446625	2.587817834	2.341591613	0.025842022	0.059227379	-
ENSG00000164520	0.308355904	5.430673325	-4.059021613	0.025861859	0.059267616	RAET1E
ENSG00000207614	0.307102255	5.430673325	-4.059021785	0.025867143	0.059274496	MIR193A
ENSG00000286970	9.726410089	1.456837632	2.748828869	0.025875226	0.059287791	AC010491.2
ENSG00000111203	625.5490623	375.7155057	0.7353212	0.0258842	0.059303123	ITFG2
ENSG00000137672	68.09082631	16.36772004	2.052558721	0.02590642	0.059348796	TRPC6
ENSG00000259251	5.518993777	0	4.796926915	0.025912054	0.059351856	AC104590.1
ENSG00000277957	0	4.585412363	-4.780834855	0.025914567	0.059351856	SENP3-EIF4A1
ENSG00000280913	5.119876265	0	4.690419722	0.025914609	0.059351856	CTSLP3
ENSG00000229204	2.152223082	12.01599197	-2.492507982	0.025918032	0.059354462	PTGES3P3
ENSG00000196544	193.0252138	111.4315141	0.791487148	0.025938803	0.059396796	BORCS6
ENSG00000198092	5.111100724	0	4.688145047	0.025978442	0.059482322	TMPRSS11F
novel.988	9.986842256	1.518200796	2.758892733	0.025990288	0.059501984	-
ENSG00000249592	59.06389702	105.2358091	-0.832231598	0.02599161	0.059501984	AC139887.2
ENSG00000138074	353.0481557	583.2827084	-0.724683409	0.026048144	0.059626152	SLC5A6
ENSG00000139133	35.5690285	69.68045509	-0.973657233	0.026080515	0.059694993	ALG10
ENSG00000224470	1030.960694	722.5156011	0.513157159	0.026084929	0.059698211	ATXN1L
ENSG00000287269	0	4.850934111	-4.854388734	0.026087053	0.059698211	Z82215.1
ENSG00000104883	109.1374066	55.70803427	0.969895908	0.026088815	0.059698211	PEX11G
ENSG00000196632	88.04781394	43.55485068	1.02036783	0.026094305	0.059703152	WNK3
ENSG00000278611	40.00797179	19.38161239	1.045195123	0.026095569	0.059703152	ZNF426-DT
ENSG00000186666	79.24571611	44.52681918	0.828723305	0.026102697	0.05971051	BCDIN3D
ENSG00000167487	352.4079086	193.1391344	0.866108717	0.026103382	0.05971051	KLHL26
ENSG00000285588	5.124962131	0	4.691748454	0.026111675	0.05972422	AL160254.1
ENSG00000187855	8.60763868	0	5.438664165	0.026125824	0.059748586	ASCL4
ENSG00000124593	50.32838547	27.06722965	0.890895594	0.026126927	0.059748586	AL365205.1
ENSG00000189143	7.25823794	19.98360405	-1.457050058	0.026132287	0.059755585	CLDN4
ENSG00000024048	1005.853085	716.3663272	0.489802318	0.026134673	0.05975578	UBR2
ENSG00000137776	1960.515402	1401.059503	0.484617958	0.026161594	0.059812071	SLTM
ENSG00000249609	13.50322513	2.395427609	2.467462533	0.026171866	0.059830291	AC080188.1
ENSG00000115970	416.6023949	656.3927275	-0.656278173	0.026185456	0.05985609	THADA
ENSG00000134030	48.50994727	112.5027194	-1.215897188	0.026193341	0.059868847	CTIF
ENSG00000129204	50.75229122	12.99716028	1.955491675	0.026216599	0.059916737	USP6
novel.789	19.30174054	2.802717299	2.772460298	0.026244453	0.059975119	-
novel.616	7.585541117	22.24569285	-1.553302817	0.0262732	0.060035534	-
ENSG00000276368	0	3.953766596	-4.564204704	0.026281877	0.060050079	HIST1H2AJ
ENSG00000185437	20.34751524	4.505135979	2.159215948	0.026308776	0.06010593	SH3BGR

ENSG00000006377	6.116758311	0.356539013	3.983172658	0.026310948	0.06010593	DLX6
ENSG00000246523	20.82575551	7.776506131	1.413047127	0.026321937	0.060125748	AP001528.1
ENSG00000165323	5.853747575	28.8604088	-2.305887021	0.026336335	0.060153347	FAT3
ENSG00000139352	5.336288183	0	4.751720703	0.026347339	0.060173191	ASCL1
ENSG00000188766	115.3361509	258.5027557	-1.163251366	0.026351344	0.060174817	SPRED3
ENSG00000251095	4.525872677	0	4.512405016	0.026353179	0.060174817	AC093866.1
ENSG00000169375	724.1695706	1025.122873	-0.501115725	0.026355	0.060174817	SIN3A
ENSG00000109511	2.708474665	23.25181055	-3.102554845	0.026386886	0.060242328	ANXA10
ENSG00000272086	61.54865134	23.29182035	1.394713482	0.026394317	0.060253998	AC025181.2
ENSG00000171448	237.7980796	149.8707609	0.667522105	0.026411688	0.060288356	ZBTB26
ENSG00000101888	210.4472733	133.8202592	0.651446025	0.026433045	0.060331805	NXT2
ENSG00000066629	362.4589282	756.6481264	-1.061602452	0.026464214	0.060397641	EML1
ENSG00000142459	598.7410137	967.1085026	-0.691533001	0.026482276	0.060433554	EVI5L
ENSG00000005020	500.157486	826.5846838	-0.725183316	0.026485716	0.060436097	SKAP2
ENSG00000094841	338.6202185	235.4603821	0.524914369	0.026497214	0.060457023	UPRT
ENSG00000250938	17.82160397	4.259554931	2.059313816	0.026523707	0.060512156	AC073475.1
ENSG00000159958	31.1628939	11.18951583	1.478658813	0.02653803	0.060539516	TNFRSF13C
ENSG00000138050	261.8138403	179.8375045	0.542568848	0.026557801	0.0605793	THUMPD2
ENSG00000005339	1374.711387	1021.631093	0.428172314	0.026587764	0.060642323	CREBBP
ENSG00000272695	24.30237728	102.0865233	-2.07145622	0.026594681	0.060652774	GAS6-DT
ENSG00000140598	268.0221157	393.7219698	-0.554645541	0.026603321	0.060667153	EFL1
ENSG00000287762	8.314326561	0	5.388530389	0.026618929	0.06069742	AC108050.1
ENSG00000255920	27.11859517	10.99596973	1.29724771	0.026637211	0.060733776	CCND2-AS1
ENSG00000242419	1.802282956	10.77289792	-2.580868255	0.026669449	0.060801945	PCDHGC4
ENSG00000071655	1472.703261	2090.041995	-0.505203742	0.026677748	0.060811934	MBD3
ENSG00000164022	812.1852879	1132.263067	-0.479615387	0.026678512	0.060811934	AIMP1
ENSG00000149050	41.66662608	20.96361649	0.986912509	0.026692485	0.060838446	ZNF214
ENSG00000130193	297.6648265	150.4540617	0.982605693	0.026701136	0.060852826	THEM6
ENSG00000234690	5.483677798	0.387220594	3.825894197	0.026740373	0.06093156	EPCAM-DT
ENSG00000167711	5.483677798	0.387220594	3.825894197	0.026740373	0.06093156	SERPINF2
ENSG00000258489	4.5888402	0	4.530480987	0.026747026	0.060941375	AC027013.1
ENSG00000249931	5.478663203	0.387220594	3.824754596	0.026760378	0.060966449	GOLGA8K
ENSG00000243504	4.585079253	0	4.529409994	0.026768634	0.06097991	RPS23P1
ENSG00000053900	736.2376918	483.6247216	0.605810581	0.026780822	0.061002326	ANAPC4
ENSG00000133275	1674.015121	2378.16309	-0.506446571	0.02678343	0.061002918	CSNK1G2
ENSG00000244301	1.233423615	10.35371148	-3.081531364	0.026789989	0.061012509	AOX3P
ENSG00000143473	3.297463658	19.69112514	-2.578137071	0.026792719	0.061013377	KCNH1
ENSG00000028116	182.4592695	300.687175	-0.72137721	0.026805715	0.061037623	VRK2
ENSG00000081059	592.0251975	215.3278052	1.459889164	0.026813527	0.06105006	TCF7
ENSG00000236308	10.17177041	1.069617038	3.24487349	0.026826374	0.06107396	AL138921.2
ENSG00000036448	11.84206354	2.813329785	2.058732415	0.026833724	0.061085341	MYOM2
ENSG00000287188	27.70514813	10.80229524	1.361432348	0.026840667	0.061095791	AC068989.1
ENSG00000147130	634.4350955	938.3093754	-0.564225401	0.026876358	0.061171674	ZMYM3
ENSG00000173546	2307.467912	4007.37769	-0.796280998	0.026900037	0.061220204	CSPG4
ENSG00000081307	680.5899563	963.2002459	-0.501213916	0.026913923	0.061239842	UBA5
ENSG00000124920	909.717877	348.126373	1.386344011	0.026915062	0.061239842	MYRF
ENSG00000286379	11.2782188	31.16738769	-1.471002253	0.026915737	0.061239842	AL591845.2
ENSG00000283897	14.60591257	2.406040094	2.577825573	0.027031973	0.061498923	AC011416.3
ENSG00000287117	5.408102518	0.387220594	3.80856604	0.027041591	0.061515416	AC105916.1
ENSG00000105146	38.66713313	14.25544313	1.446696814	0.027056841	0.06154472	AURKC
ENSG00000163935	145.9107678	74.18803122	0.972676616	0.027062509	0.061552223	SFMBT1

ENSG00000231500	23.38329308	48.80053543	-1.062537065	0.027079695	0.061585921	RPS18
ENSG00000130856	453.4743411	268.6292983	0.755512759	0.027085842	0.061594509	ZNF236
ENSG00000166337	283.6412508	475.8693802	-0.746491845	0.027101971	0.061622349	TAF10
ENSG00000287814	5.089646153	0	4.682521109	0.02710555	0.061622349	AC011407.1
ENSG00000272745	5.089646153	0	4.682521109	0.02710555	0.061622349	AC004948.1
ENSG00000213700	12.1516731	29.59227166	-1.281267796	0.027107572	0.061622349	RPL17P50
ENSG00000280407	10.55444795	28.35760674	-1.424524471	0.027121916	0.061649562	AC132872.4
ENSG00000158792	277.9494388	156.6227156	0.827728895	0.027126564	0.061653494	SPATA2L
ENSG00000225921	981.3678301	1536.189044	-0.646661016	0.027128392	0.061653494	NOL7
ENSG00000176476	187.0389782	111.457491	0.745337956	0.027139212	0.061672691	SGF29
ENSG00000121210	118.886624	197.6917162	-0.734285322	0.027162082	0.061719262	TMEM131L
ENSG00000247796	54.42240322	94.3238873	-0.79528632	0.027177294	0.061748428	AC008966.1
ENSG00000162843	23.29052272	2.323323566	3.352495127	0.027205248	0.061806537	WDR64
ENSG00000187688	188.1404825	421.5439693	-1.164523544	0.027236063	0.061871134	TRPV2
ENSG00000278768	158.4060137	82.96007692	0.936064062	0.027246249	0.061888861	BACE1-AS
ENSG00000155761	21.11935236	3.404837359	2.611019097	0.027251261	0.061894835	SPAG17
ENSG00000277283	78.37176361	41.46803674	0.915287096	0.027284702	0.061965371	AC004812.2
ENSG00000197124	127.4449862	67.45135963	0.916468515	0.027303868	0.062003479	ZNF682
ENSG00000258545	7.914026672	1.10029862	2.863657595	0.027314334	0.062021823	RHOXF1-AS1
ENSG00000227671	474.138588	289.4598671	0.712749164	0.027324708	0.062039957	AL390728.4
ENSG00000198556	242.988374	158.6608335	0.613600178	0.027353157	0.062099125	ZNF789
ENSG00000267142	5.220667063	0	4.716492081	0.0273724	0.062137382	AC092296.3
ENSG00000236814	35.58163626	63.95905385	-0.847190402	0.027376558	0.06214139	AC046176.1
ENSG00000113811	1240.624859	839.2236238	0.564167292	0.027395161	0.062178185	SELENOK
ENSG00000173064	1541.886865	960.9296211	0.682374722	0.02740669	0.062197908	HECTD4
ENSG00000145945	276.3824991	175.6902389	0.653300761	0.027408639	0.062197908	FAM50B
ENSG00000279221	4.485542104	0	4.50071875	0.027413752	0.062204078	AC068254.2
ENSG00000251008	4.501910809	0	4.505460342	0.027428797	0.062219935	ORAOV1P1
ENSG00000156574	4.501910809	0	4.505460342	0.027428797	0.062219935	NODAL
ENSG00000239732	4.501910809	0	4.505460342	0.027428797	0.062219935	TLR9
ENSG00000286764	5.213145171	0	4.714564984	0.02743032	0.062219935	AC188617.2
ENSG00000130427	4.237646425	0	4.417029469	0.027436021	0.062226008	EPO
ENSG00000274349	115.3235432	56.12966086	1.036516194	0.027437787	0.062226008	ZNF658
ENSG00000203876	27.11734152	11.34305213	1.252024819	0.027450807	0.062250103	ADD3-AS1
ENSG00000182944	4208.920152	6447.463386	-0.615265255	0.027454559	0.062253177	EWSR1
ENSG00000006283	5.224428009	29.72201445	-2.509817768	0.027462073	0.062261082	CACNA1G
ENSG00000235313	3.619680969	12.25913287	-1.75595581	0.027462838	0.062261082	HM13-IT1
ENSG00000287723	4.889674211	0	4.622387169	0.027485882	0.062307887	AP005901.6
ENSG00000146221	12.20579381	1.834601616	2.747360159	0.02749056	0.062313056	TCTE1
ENSG00000125459	262.4048102	426.0848302	-0.699343329	0.027516578	0.06236659	MSTO1
ENSG00000127947	3743.208516	2181.999784	0.778761549	0.027538985	0.062406543	PTPN12
ENSG00000276107	21.22981635	46.31293469	-1.124419603	0.02753901	0.062406543	AC037198.1
ENSG00000244413	4.222531369	0	4.412423732	0.027560131	0.062448095	RPL23AP56
ENSG00000172785	355.498547	204.9439385	0.794507012	0.027562153	0.062448095	CBWD1
ENSG00000237594	0.61420451	7.66439229	-3.657486789	0.027570513	0.062461589	AP000251.1
ENSG00000242960	113.7336543	276.4930848	-1.280953931	0.02757969	0.062473995	FTH1P23
ENSG00000169894	4.220024071	0	4.411662471	0.027580798	0.062473995	MUC3A
ENSG00000184319	276.2259063	420.3908528	-0.605839116	0.027592088	0.062494119	RPL23AP82
ENSG00000250081	1.17296339	10.84487357	-3.192851765	0.027600071	0.062506751	AC025176.1
ENSG00000259705	6.362146692	17.14203283	-1.432024369	0.027621427	0.062549665	AC084757.3
ENSG00000130035	8.880678605	1.060160428	3.052848983	0.027627904	0.062558879	GALNT8

ENSG00000196455	425.8220872	617.2638666	-0.535458636	0.027639375	0.062579399	PIK3R4
ENSG00000028277	370.0521063	1401.593806	-1.921192388	0.027652799	0.062604336	POU2F2
ENSG00000183760	41.3525714	156.8515068	-1.92332895	0.027727723	0.062768491	ACP7
ENSG00000245910	1167.488779	842.3365898	0.470805168	0.027748872	0.062810895	SNHG6
ENSG00000182853	140.1087043	37.80615394	1.886368288	0.027751814	0.062812081	VMO1
ENSG00000269889	1.232169966	10.20030357	-3.063094568	0.02777194	0.062852158	AC078802.1
ENSG00000112667	774.3222593	1109.181781	-0.518476634	0.02780116	0.062912806	DNPH1
ENSG00000106443	630.9233713	401.1175952	0.652687769	0.027831771	0.062976592	PHF14
ENSG00000243678	28.48024766	68.99219806	-1.276550327	0.027857646	0.063025303	NME2
ENSG00000215196	0.894837599	7.317181494	-3.014976936	0.027858149	0.063025303	BASP1-AS1
ENSG00000282728	8.000956063	0	5.333103075	0.02787428	0.063056305	PGR-AS1
ENSG00000134453	2106.853967	1488.906285	0.500864223	0.027882286	0.063068925	RBM17
ENSG00000169398	1431.873919	2175.947319	-0.603587193	0.027898486	0.063100074	PTK2
ENSG00000273899	83.27321921	151.8477384	-0.867789319	0.027922062	0.06314248	NOL12
ENSG00000243368	18.09339025	4.485066883	1.992917357	0.027922095	0.06314248	MCCC1-AS1
ENSG00000266877	0	4.004517274	-4.580414211	0.027928409	0.063149742	AC007923.4
ENSG00000259614	7.388974116	0.356539013	4.259718052	0.027930168	0.063149742	AC087477.6
ENSG00000274376	8.002209711	0	5.333322161	0.027934662	0.063154407	ADAMTS7P1
ENSG00000237125	50.32251632	5.63483191	3.166919447	0.027951807	0.063187671	HAND2-AS1
ENSG00000065923	326.6034532	872.6372179	-1.417830425	0.02796232	0.063205936	SLC9A7
ENSG00000257086	6.691957167	20.26932329	-1.599007065	0.027991725	0.0632669	AP001453.4
ENSG00000035928	816.7553505	1152.306048	-0.496637308	0.028013069	0.063309634	RFC1
novel.554	45.87704789	15.75357483	1.546556462	0.028016084	0.063310939	-
ENSG00000280378	13.55616346	3.586615098	1.918491455	0.028021489	0.063317647	AL353898.3
novel.881	6.767461177	0	5.091310408	0.028033423	0.063339105	-
ENSG00000010270	789.4998663	1034.618892	-0.390184008	0.028041823	0.063349852	STARD3NL
ENSG00000234773	15.07022017	4.697526203	1.687106195	0.028043057	0.063349852	AC012618.3
ENSG00000229017	7.94934265	0.723690511	3.463655641	0.028070592	0.063406543	LINC01277
ENSG00000198799	454.1834947	317.3834498	0.517412994	0.028076319	0.063413965	LRIG2
ENSG00000196123	343.8710162	507.6709016	-0.561475501	0.028093781	0.063447891	KIAA0895L
ENSG00000143376	1131.985383	783.6952179	0.530934232	0.028137448	0.063540986	SNX27
ENSG00000133055	12.06606863	2.129777466	2.495409932	0.02814618	0.063555179	MYBPH
ENSG00000276600	100.6069803	39.11480228	1.363814349	0.028159273	0.06357922	RAB7B
ENSG00000279164	0	7.111738639	-5.408730791	0.028171479	0.063601251	AL118508.2
ENSG00000154814	116.1827366	198.1021352	-0.771408419	0.02819315	0.063644645	OXNAD1
ENSG00000176490	63.497355	125.6112572	-0.985092998	0.028208976	0.063674839	DIRAS1
ENSG00000144320	584.2314193	804.8497301	-0.462239225	0.02821991	0.063693988	LNPK
ENSG00000242628	13.48184183	2.893606169	2.226031312	0.028244336	0.06374082	AC009228.1
ENSG00000228812	7.29097535	0.693008929	3.361723222	0.02824638	0.06374082	LAMA5-AS1
ENSG00000268362	80.92442913	137.3548394	-0.763598766	0.02824802	0.06374082	AC092279.1
ENSG00000272321	9.441944783	1.069617038	3.135064898	0.028272265	0.06378587	AP003355.2
ENSG00000258376	94.83651657	23.19617958	2.02948895	0.028272895	0.06378587	AC004846.1
ENSG00000277247	5.15895319	0.387220594	3.738524702	0.028302492	0.0638471	AC083809.1
ENSG00000279748	7.54897149	1.049547942	2.823686144	0.0283078	0.063851004	AC008764.9
ENSG00000080802	452.4292777	326.6358823	0.469876951	0.028309138	0.063851004	CNOT4
ENSG00000161904	1311.92427	1796.819112	-0.453600674	0.028330636	0.063893946	LEMD2
ENSG00000110436	39.43424005	13.01478923	1.592442388	0.028344001	0.063913403	SLC1A2
ENSG00000273257	8.72193488	1.386017859	2.626125137	0.028344183	0.063913403	AC069200.1
ENSG00000109771	103.5497465	59.50454015	0.802533583	0.028349131	0.063919011	LRP2BP
ENSG00000139921	928.6028268	1468.329272	-0.661287459	0.028392447	0.064011121	TMX1
ENSG00000196456	293.3767928	196.8274137	0.57622383	0.028399941	0.064022462	ZNF775

ENSG00000172469	336.7090096	496.3685481	-0.560441526	0.028424788	0.064072914	MANEA
novel.501	57.40678112	22.4614914	1.359748416	0.028428729	0.064076239	-
ENSG00000227158	0.616711807	13.18454182	-4.426238504	0.028444643	0.064106547	AC073621.1
ENSG00000105327	514.3595372	292.6810133	0.813438766	0.028450284	0.064113699	BBC3
ENSG00000165525	515.5615772	700.626109	-0.442539436	0.028473355	0.064160126	NEMF
ENSG00000108840	1600.610234	2310.58313	-0.529477457	0.028481167	0.064172162	HDAC5
ENSG00000111716	455.993284	816.0545686	-0.839837559	0.028488618	0.064183384	LDHB
ENSG00000127080	87.89325808	148.2911969	-0.753836473	0.0284974	0.064197603	IPPK
ENSG00000136870	443.5226567	283.8885946	0.643102659	0.028504639	0.064208342	ZNF189
ENSG00000086967	4.764921544	0	4.588176037	0.028510672	0.064216366	MYBPC2
ENSG00000148832	44.98450412	22.17474468	1.025016162	0.02851474	0.06421996	PAOX
ENSG00000122965	594.8766445	844.8600433	-0.506115442	0.028520739	0.064225216	RBM19
ENSG00000198815	1228.600976	838.2139778	0.551953	0.028522018	0.064225216	FOXJ3
ENSG00000153015	363.0203377	549.4036082	-0.597903081	0.028536197	0.064251575	CWC27
ENSG00000137968	11.13591505	1.345879667	3.006462761	0.028548757	0.064274285	SLC44A5
ENSG00000228874	4.590093848	0	4.530884593	0.028577333	0.064333046	AC090954.1
ENSG00000225279	8.954715503	1.110911105	3.03795325	0.028584695	0.064344044	AL121987.1
ENSG00000235072	5.531530264	0	4.800045586	0.028592928	0.064357002	AC012074.1
ENSG00000118804	20.80591059	53.83774239	-1.372378084	0.028597487	0.064361686	STBD1
ENSG00000240694	247.4851103	65.06936983	1.925820636	0.028618268	0.064402879	PNMA2
ENSG00000233665	4.583825605	0	4.529093773	0.028630999	0.064425948	AC060234.2
ENSG00000130487	33.66874678	8.191968162	2.048568435	0.028654643	0.064473566	KLHDC7B
ENSG00000272269	11.18251387	25.32454438	-1.175733508	0.028695501	0.064559908	AL138724.1
ENSG00000166295	1951.798279	1451.826066	0.426878241	0.028703584	0.064572501	ANAPC16
ENSG00000238365	0.879722543	8.173183336	-3.193927458	0.028713249	0.064581108	RNU7-57P
ENSG00000267366	4.252761481	0	4.421637332	0.02871367	0.064581108	AP005131.2
ENSG00000147471	841.3675953	529.9111787	0.666358999	0.02871575	0.064581108	PLPBP
ENSG00000239470	9.365115854	23.52178022	-1.325172508	0.028717353	0.064581108	AC011979.1
ENSG00000226578	4.247746886	0	4.420116771	0.028757386	0.064665539	AL132657.1
ENSG00000149930	1494.036007	2023.25673	-0.43733264	0.028765678	0.064678588	TAOK2
novel.815	29.00766497	5.901766323	2.287158262	0.028784264	0.064713633	-
ENSG00000169629	52.58111461	28.57481796	0.878074229	0.028786246	0.064713633	RGPD8
ENSG00000143502	7.200285013	0.743759607	3.309123606	0.02881481	0.064763636	SUSD4
ENSG00000267515	7.683468612	0.336469917	4.315897076	0.028815134	0.064763636	AP001029.3
ENSG00000261192	4.562371034	0	4.522914854	0.028815967	0.064763636	RNF126P1
ENSG00000271882	17.09170707	4.566499142	1.89140758	0.02884647	0.064826584	AP001330.5
ENSG00000240563	74.75111338	15.48232088	2.265939344	0.028853583	0.064836961	L1TD1
ENSG00000144034	304.8208778	438.5384082	-0.525167365	0.028879444	0.06488946	TPRKB
ENSG00000255987	4.553595492	0	4.520396365	0.028892092	0.064912265	TOMM20P2
ENSG00000231185	13.17126336	39.19893148	-1.578103591	0.028905841	0.064937541	SPRY4-AS1
ENSG00000115267	652.1211473	420.0373488	0.634548016	0.028908359	0.064937582	IFIH1
ENSG00000234337	7.900165265	20.13829623	-1.351203969	0.028918963	0.064955786	AC026462.1
ENSG00000279800	8.232483037	21.94106039	-1.416989949	0.028930305	0.064975645	BCLAF1P2
ENSG00000061987	1386.228556	999.2906117	0.472226249	0.028941613	0.064990926	MON2
ENSG00000166734	2672.530659	1901.788662	0.490885327	0.028942112	0.064990926	CASC4
ENSG00000137077	8.005970658	0	5.333990244	0.028976381	0.065062254	CCL21
ENSG00000179941	488.4882841	350.8436078	0.477268406	0.028979627	0.06506392	BBS10
ENSG00000030582	4149.351034	7723.462529	-0.896341142	0.028997254	0.065093467	GRN
ENSG00000282936	21.1883034	44.9023998	-1.086619833	0.028997798	0.065093467	AC004706.3
ENSG00000114054	521.2884055	806.1321108	-0.629016485	0.029018396	0.065134077	PCCB
ENSG00000250073	10.29269086	2.496928964	2.03872819	0.029025025	0.065143329	AP000866.2

ENSG00000064270	5.138823539	19.72753891	-1.940280711	0.029030593	0.065150197	ATP2C2
ENSG00000149582	468.8948845	293.5733946	0.674528992	0.029039969	0.065161137	TMEM25
ENSG00000163913	465.1786166	767.2113466	-0.721950026	0.029041415	0.065161137	IFT122
ENSG00000236028	6.120590528	0.356539013	3.98408624	0.029042992	0.065161137	AL354766.2
ENSG00000188994	1255.087476	721.6027996	0.798822039	0.029051564	0.065171242	ZNF292
ENSG00000197056	229.9621355	342.5439266	-0.574367191	0.029053965	0.065171242	ZMYM1
ENSG00000115353	9.181512616	1.049547942	3.10722104	0.029057134	0.065171242	TACR1
ENSG00000283674	6.67677084	0.387220594	4.110799247	0.029060335	0.065171242	AC068587.4
ENSG00000184557	3163.979347	1134.300218	1.479892462	0.029062123	0.065171242	SOCS3
ENSG00000156030	1032.071033	659.4133198	0.646573486	0.029062546	0.065171242	ELMSAN1
ENSG00000198626	4.207416313	0	4.407778048	0.029112541	0.065270854	RYR2
ENSG00000234715	4.207416313	0	4.407778048	0.029112541	0.065270854	AC005064.1
ENSG00000235590	4.135601978	25.9937598	-2.643157216	0.029116462	0.065270854	GNAS-AS1
ENSG00000273216	11.52582974	0.672939833	4.054595295	0.029123311	0.065270854	AC002059.1
ENSG00000158022	4.206162664	0	4.407395341	0.029123685	0.065270854	TRIM63
ENSG00000237188	4.206162664	0	4.407395341	0.029123685	0.065270854	AC242426.2
ENSG00000037757	347.3099855	488.7421581	-0.492982524	0.029124553	0.065270854	MRI1
ENSG00000234465	161.5968453	95.07423101	0.767425919	0.02913023	0.065277946	PINLYP
ENSG00000274363	9.515012766	1.029478846	3.172740941	0.029167404	0.065355612	AC087742.1
ENSG00000233397	0	3.882946823	-4.541312319	0.029172957	0.065356782	AC008063.2
ENSG00000152611	0	3.882946823	-4.541312319	0.029172957	0.065356782	CAPSL
ENSG00000215009	1.845120827	15.62956423	-3.086232521	0.02921085	0.065436032	ACSM4
ENSG00000137486	573.2666524	227.1752476	1.335869397	0.029228827	0.065470657	ARRB1
ENSG00000107249	354.5044882	822.8901298	-1.214552094	0.029270638	0.065558659	GLIS3
ENSG00000179981	422.3827307	232.5080625	0.861228592	0.029274559	0.06556179	TSHZ1
ENSG00000274512	48.98447004	13.69705728	1.840509884	0.029284108	0.065577522	TBC1D3L
ENSG00000249216	5.221920712	0	4.716862736	0.029309455	0.065622971	AC105389.2
ENSG00000204959	5.221920712	0	4.716862736	0.029309455	0.065622971	ARHGEF34P
ENSG00000023697	463.2651699	814.3051567	-0.814134786	0.029327924	0.065656571	DERA
ENSG00000234969	8.102715775	1.029478846	2.940272235	0.029329516	0.065656571	AL627389.1
ENSG00000278816	6.952389334	0.693008929	3.293387687	0.029337931	0.06566975	AL121890.5
novel.823	28.81786476	58.67220343	-1.024563432	0.029372543	0.065741561	-
ENSG00000078549	72.43229615	0	8.512119436	0.029391559	0.065777047	ADCYAP1R1
ENSG00000179059	0	4.485066883	-4.751727166	0.029393461	0.065777047	ZFP42
ENSG00000250829	7.846044555	0	5.305851757	0.029423321	0.065838197	AC108865.1
ENSG00000281731	13.61920225	3.260757667	2.068072543	0.029440937	0.06587194	METTL14-DT
ENSG00000138443	1704.38102	1173.142456	0.538628892	0.02946231	0.065914085	ABI2
ENSG00000183765	244.1703546	372.8673847	-0.611139828	0.029466604	0.065918016	CHEK2
ENSG00000148541	41.7942417	9.96764676	2.065443762	0.02950252	0.065991264	FAM13C
ENSG00000285971	4.484288455	0	4.500292259	0.029504427	0.065991264	AC023095.1
ENSG00000285621	7.494779509	0.693008929	3.404643897	0.029544436	0.066075062	AL138999.2
ENSG00000236144	148.3666601	232.2440478	-0.647012561	0.029584234	0.066158373	TMEM147-AS1
ENSG00000259494	88.31110941	137.3604903	-0.63763927	0.029601248	0.066185325	MRPL46
ENSG00000167483	25.75561775	8.458902575	1.59844085	0.029601381	0.066185325	FAM129C
novel.836	13.53728746	1.682349583	2.972773481	0.029615793	0.06621185	-
ENSG00000168772	13.60283355	2.904218654	2.234640425	0.029660561	0.066306234	CXXC4
ENSG00000153885	377.6772351	905.6559065	-1.262011444	0.029666203	0.06631314	KCTD15
ENSG00000268061	24.52985857	9.234499639	1.404473938	0.029704792	0.066393685	NAPA-AS1
ENSG00000079805	2642.313979	3636.989639	-0.460871734	0.029709332	0.066398122	DNM2
ENSG00000143570	7.638408178	23.63299498	-1.636146054	0.029725284	0.066428059	SLC39A1
ENSG00000110200	65.0939394	109.8035113	-0.755437776	0.029753241	0.066484817	ANAPC15

ENSG00000257489	5.174068246	0.387220594	3.742167638	0.029771743	0.066520439	AC010203.1
ENSG00000087995	261.721825	409.9010709	-0.648015343	0.029782453	0.066538646	METTL2A
ENSG00000151491	1458.22464	5053.918631	-1.793235993	0.029790481	0.066550859	EPS8
ENSG00000275111	151.126962	95.39885135	0.66559629	0.029800094	0.066566609	ZNF2
ENSG00000215788	580.1209619	320.5186901	0.856750884	0.029820763	0.066607052	TNFRSF25
ENSG00000156886	17.25887033	1.386017859	3.621229421	0.029835368	0.066633946	ITGAD
ENSG00000109272	5.232021173	39.93007082	-2.936550097	0.029856747	0.066675962	PF4V1
ENSG00000273008	25.84261841	11.07740199	1.218594012	0.029864291	0.066687078	AC010864.1
ENSG00000122406	12100.16925	16662.29147	-0.461565111	0.029872268	0.066699157	RPL5
ENSG00000136271	1115.834914	1794.915539	-0.685886278	0.029913296	0.066785026	DDX56
ENSG00000008118	7.396780743	40.97648946	-2.472155911	0.029921603	0.066797832	CAMK1G
ENSG00000273492	5.174068246	0.356539013	3.742171238	0.029928539	0.066807576	AP000229.1
ENSG00000224543	23.55059888	45.27631098	-0.939467119	0.029941985	0.066831849	SNRPGP15
ENSG00000050628	5.67362055	0.387220594	3.878497539	0.029948129	0.06683982	PTGER3
ENSG00000092850	13.49187102	2.261960402	2.6049966	0.02999004	0.066927609	TEKT2
ENSG00000143314	1020.679199	1448.624885	-0.505382382	0.030013168	0.066973472	MRPL24
ENSG00000101940	2455.924451	1659.485789	0.565721225	0.030016773	0.066975763	WDR13
ENSG00000179292	2.40137241	13.70522962	-2.504193845	0.03002169	0.066980981	TMEM151A
ENSG00000283537	0.293240848	5.534614825	-4.094200757	0.030048427	0.067034876	AC073264.3
ENSG00000272282	9.746539741	1.722487775	2.468307498	0.030054081	0.067041735	LINC02084
ENSG00000115641	1447.216463	5888.377833	-2.024622841	0.030058922	0.067046776	FHL2
ENSG00000285796	156.9067873	88.31445507	0.82981295	0.03008449	0.067095999	AL162458.1
ENSG00000176024	96.08931404	61.67702435	0.639077295	0.030086155	0.067095999	ZNF613
ENSG00000115457	4966.772119	1112.251007	2.158722862	0.030098807	0.067118453	IGFBP2
ENSG00000143353	480.9130291	353.9494637	0.442259666	0.030110302	0.067138325	LYPLAL1
ENSG00000243710	18.00388229	2.355289417	2.906597943	0.030118671	0.067145967	CFAP57
ENSG00000118298	35.67107295	7.916861409	2.18364297	0.030118898	0.067145967	CA14
ENSG00000145248	11.46314695	1.548882377	2.935985072	0.030135678	0.06717761	SLC10A4
ENSG00000138801	1351.872263	1767.597026	-0.386772028	0.030176888	0.067263702	PAPSS1
ENSG00000111412	562.2540121	830.7856069	-0.563684926	0.030212782	0.067337933	C12orf49
ENSG00000116132	509.89315	1992.593925	-1.966433002	0.030218756	0.067345471	PRRX1
novel.49	25.23726072	8.579317151	1.553780353	0.030222087	0.067347117	-
novel.496	4.27672335	32.81422258	-2.942251178	0.030231367	0.067362019	-
ENSG00000287245	19.83639536	6.888666832	1.532926745	0.030241137	0.067378011	AP001981.2
ENSG00000211598	4.911057511	0	4.628256145	0.030253796	0.067394657	IGKV4-1
ENSG00000224810	4.911057511	0	4.628256145	0.030253796	0.067394657	AL355482.1
ENSG00000182093	615.6805731	431.8789306	0.511053564	0.030292417	0.067474904	WRB
ENSG00000258572	9.426829727	1.161661783	3.075839181	0.030299875	0.067485732	AL133467.1
ENSG00000002745	50.5901422	15.83513548	1.67578996	0.030319093	0.067522746	WNT16
ENSG00000134775	1201.253563	481.929714	1.318060954	0.030351797	0.067589786	FHOD3
ENSG00000251161	15.49956779	2.742510011	2.475145861	0.030357232	0.067596095	AC020661.1
ENSG00000103657	1468.572646	1048.725139	0.485796805	0.030399089	0.067683496	HERC1
ENSG00000196453	341.853105	473.0816555	-0.468529334	0.030408721	0.067699142	ZNF777
ENSG00000134242	140.1519416	62.59626874	1.165818068	0.030417422	0.067708888	PTPN22
ENSG00000285534	4.826635418	0	4.605157235	0.030418948	0.067708888	AL163541.1
ENSG00000286416	17.83664776	5.952517001	1.569171459	0.030420917	0.067708888	AL442067.3
ENSG00000005483	1375.656114	906.9823206	0.601258534	0.030460891	0.067792053	KMT2E
ENSG00000287346	2.128261214	13.2096196	-2.6401881	0.030478586	0.067825623	AL132712.2
ENSG00000263731	37.00339298	15.92962037	1.211588305	0.030490899	0.067847211	AC145207.5
ENSG00000214223	164.8935538	267.4654663	-0.698437997	0.030526027	0.06791956	HNRNPA1P10
ENSG00000260852	119.1356308	68.15262211	0.805116669	0.030548909	0.067964653	FBXL19-AS1

ENSG00000151176	1288.070683	1915.038968	-0.572091258	0.030583239	0.068035202	PLBD2
ENSG00000287335	0	4.208804254	-4.658177248	0.030601164	0.068069251	AL139351.3
ENSG00000267254	131.746569	75.48503959	0.804499489	0.030654012	0.068180969	ZNF790-AS1
ENSG00000119335	4691.414008	6631.259053	-0.499312611	0.030657639	0.068183198	SET
ENSG00000169228	145.8278129	233.1828477	-0.677048945	0.030663634	0.068190694	RAB24
ENSG00000224057	0.922560414	8.67020602	-3.242336391	0.030666526	0.068191288	EGFR-AS1
ENSG00000267280	103.8716356	202.0090386	-0.95915874	0.030708992	0.068279873	TBX2-AS1
ENSG00000136059	245.9444158	156.8768542	0.647481437	0.030711974	0.068280661	VILL
ENSG00000206530	283.7188355	134.8945341	1.074314738	0.030727951	0.068310335	CFAP44
ENSG00000164037	31.32608275	12.38198759	1.33385445	0.030772324	0.068403126	SLC9B1
ENSG00000137727	215.8200877	54.94484754	1.971869738	0.030776068	0.068405598	ARHGAP20
ENSG00000196517	50.57036891	100.9374474	-0.999098185	0.030812407	0.068480508	SLC6A9
ENSG00000226686	21.45654219	61.94408716	-1.532542282	0.030832166	0.06851373	LINC01535
ENSG00000051382	298.3005568	417.8367515	-0.486431123	0.030832629	0.06851373	PIK3CB
ENSG00000258643	5.141330836	16.30378833	-1.662102083	0.030878496	0.068602072	BCL2L2-PABPN1
ENSG00000232504	9.141182043	1.803920035	2.344124133	0.030880248	0.068602072	ST3GAL5-AS1
ENSG00000197622	2805.564411	1881.892818	0.576242654	0.03088189	0.068602072	CDC42SE1
ENSG00000100911	564.8426187	1095.152619	-0.955466044	0.030882947	0.068602072	PSME2
ENSG00000259867	11.51733894	2.954969332	1.98260443	0.030968747	0.068786784	AC105411.1
ENSG00000224281	45.29633624	16.64513854	1.435068467	0.031014498	0.068882515	SLC25A5-AS1
ENSG00000111321	2291.240374	3426.404801	-0.580569597	0.031022861	0.068895201	AC005840.1
ENSG00000181915	936.3447744	579.5100179	0.692354854	0.031029887	0.068904913	ADO
ENSG00000135074	656.0700971	1196.205679	-0.866427241	0.031040222	0.068921973	ADAM19
ENSG00000065518	1310.018602	1839.689721	-0.489702725	0.031054005	0.068946685	NDUFB4
ENSG00000164967	244.322901	398.3230506	-0.705668135	0.031059468	0.06895292	RPP25L
ENSG00000269653	0.293240848	5.512105584	-4.078126825	0.0310696	0.068969521	AC011479.3
ENSG00000256001	0	4.503851709	-4.746387463	0.031074719	0.06897499	AC079949.1
ENSG00000279108	28.29309694	11.3004738	1.330092759	0.031084091	0.068989901	AC008537.3
ENSG00000103275	1769.940336	2341.80259	-0.40395868	0.031119657	0.069062938	UBE2I
ENSG00000139625	742.2601844	1202.106255	-0.695270536	0.031158312	0.069142818	MAP3K12
ENSG00000152785	32.56286822	2.169915658	3.908833493	0.03119016	0.06920758	BMP3
ENSG00000270673	26.36237164	9.907439472	1.405498531	0.031202237	0.069228464	YTHDF3-AS1
ENSG00000094631	1504.95493	976.6646338	0.6239399	0.031247095	0.06932207	HDAC6
ENSG00000125875	1038.33837	1620.344808	-0.641827923	0.031261444	0.069347983	TBC1D20
ENSG00000277763	8.265220447	0.774441189	3.476689782	0.03127006	0.069361173	AL138995.1
ENSG00000101367	2607.100474	3545.118354	-0.44345686	0.031276247	0.069368975	MAPRE1
ENSG00000178082	34.21210587	61.30524935	-0.8418713	0.031297327	0.069408158	TWF1P1
ENSG00000219487	4.796405305	0	4.596807636	0.031299256	0.069408158	AL603766.1
novel.853	9.411714671	35.10964271	-1.90277217	0.031313938	0.069434788	-
ENSG00000119865	508.5145788	828.7306435	-0.705039018	0.031323281	0.06944958	CNRIP1
ENSG00000235370	12.93311214	2.169915658	2.579775169	0.031336297	0.06947251	DNM1P51
ENSG00000275645	8.535824346	1.029478846	3.014704342	0.031363932	0.069527842	AC068338.3
ENSG00000167081	464.9189073	261.2090744	0.831726931	0.031377578	0.069552159	PBX3
ENSG00000264573	6.35329988	0.356539013	4.039534301	0.031390816	0.069575566	RN7SL15P
ENSG00000142733	549.4354256	850.8466748	-0.631218098	0.031402206	0.069594875	MAP3K6
ENSG00000271709	0.307102255	4.637318915	-3.834033503	0.031430221	0.06965102	AC017033.1
ENSG00000172354	4461.221202	6145.434303	-0.46204701	0.03144105	0.069669077	GNB2
ENSG00000204802	7.212892771	0.672939833	3.364946723	0.03151194	0.069820205	AL590399.1
ENSG00000121716	51.33006865	87.09489775	-0.762296109	0.031520586	0.069833405	PILRB
ENSG00000155330	293.9748699	200.7057842	0.549915982	0.031528578	0.069845156	C16orf87
ENSG00000140465	0.616711807	8.548507175	-3.802302779	0.031549707	0.069886005	CYP1A1

ENSG00000276529	32.77584701	63.45270295	-0.952412073	0.031571725	0.069928813	AP001505.1
ENSG00000213435	7.559071951	1.029478846	2.837413945	0.031576873	0.069934253	ATP6V0CP3
ENSG00000134376	15.06144462	2.659793482	2.531156155	0.031585768	0.069947992	CRB1
ENSG00000117586	135.1984735	48.97213305	1.461853949	0.031596079	0.069964862	TNFSF4
ENSG00000140743	8.25010539	21.94106039	-1.414814468	0.031609089	0.069982157	AC092338.1
ENSG00000150760	1836.289453	1307.703368	0.489969736	0.031609276	0.069982157	DOCK1
ENSG00000267053	40.54828195	12.93078843	1.655898504	0.031614221	0.069987141	AC092296.2
ENSG00000235888	12.9482272	2.558292127	2.351559003	0.031624434	0.070003785	AF064858.1
ENSG00000160957	523.9327904	900.3633582	-0.781030552	0.031713088	0.070194049	RECQL4
ENSG00000076513	962.0543234	712.5064979	0.43342078	0.03173285	0.070229361	ANKRD13A
ENSG00000107263	1719.343854	1273.358641	0.433406842	0.031734448	0.070229361	RAPGEF1
ENSG00000168291	1114.731811	1480.189182	-0.409047469	0.031745406	0.070247627	PDHB
ENSG00000168026	155.5615745	87.55623016	0.831498905	0.03175473	0.070262275	TTC21A
ENSG00000184719	130.6327688	75.44759834	0.788888925	0.03177498	0.070301094	RNLS
ENSG00000287600	0.616711807	8.75163828	-3.838343429	0.031796472	0.070342655	AC122719.3
ENSG00000189014	17.95255361	5.218214004	1.765022615	0.031833116	0.070415887	SHLD2P3
ENSG00000154727	785.9463009	583.5291757	0.429304328	0.031834995	0.070415887	GABPA
ENSG00000251450	10.10016954	0.387220594	4.707688765	0.03188347	0.070517105	RASGRF2-AS1
ENSG00000143178	67.76570225	37.40074364	0.859116962	0.031902516	0.070553224	TBX19
ENSG00000108958	7.810728576	20.51490434	-1.388425748	0.031950859	0.07065412	AC130689.1
ENSG00000167257	188.1587185	278.2443404	-0.56358328	0.031955315	0.070657961	RNF214
ENSG00000162888	3.620934617	11.90259386	-1.712949392	0.03201281	0.070779066	C1orf147
ENSG00000222112	10.5582089	30.22160567	-1.513942212	0.032022402	0.07079425	RN7SKP16
ENSG00000275437	3.344062475	13.1752136	-1.976035266	0.03206384	0.070876842	AL121832.3
ENSG00000168314	4.218770422	0	4.411226579	0.032067945	0.070876842	MOBP
ENSG00000240665	4.218770422	0	4.411226579	0.032067945	0.070876842	LSP1P2
ENSG00000230521	7.557747031	0	5.251749967	0.032080185	0.070897863	AL645929.1
ENSG00000198853	1469.483468	2037.113381	-0.471041189	0.032083283	0.070898679	RUSC2
ENSG00000279943	8.192152463	1.386017859	2.53318803	0.0321052	0.070940349	AC110771.1
ENSG00000149922	33.28710943	15.24478378	1.131937581	0.0321076	0.070940349	TBX6
ENSG00000166224	1045.90844	739.0044849	0.500666314	0.032124159	0.070970898	SGPL1
ENSG00000223969	9.602911072	24.86778828	-1.371373374	0.032172631	0.071071944	AC002456.1
ENSG00000165699	1459.299708	855.7339505	0.770289085	0.03219075	0.071105597	TSC1
ENSG00000170430	557.3467583	299.7413718	0.893962054	0.032193339	0.071105597	MGMT
ENSG00000180914	922.3027848	249.6279025	1.88502407	0.032206045	0.071127614	OXTR
ENSG00000174151	380.8570998	274.236835	0.473594466	0.032214752	0.071140797	CYB561D1
ENSG00000205325	0	4.168666062	-4.645670879	0.032241542	0.071193907	AC005863.1
ENSG00000182154	1333.050389	1797.698115	-0.431406163	0.032255841	0.071215233	MRPL41
ENSG00000273402	15.83027591	3.698728939	2.114938538	0.032260172	0.071215233	AC004908.3
ENSG00000224420	20.3337251	7.908689068	1.362887698	0.032261043	0.071215233	ADM5
ENSG00000211694	5.223174361	0	4.717253457	0.032262165	0.071215233	TRGV10
ENSG00000267123	6.973843905	0.672939833	3.313880062	0.032269835	0.071226114	SCAT1
ENSG00000110274	654.0665196	949.7508249	-0.53800261	0.032288824	0.071261971	CEP164
ENSG00000109576	371.5242673	197.3083956	0.912590147	0.03231076	0.071304328	AADAT
ENSG00000225953	16.78202625	55.66399609	-1.729443536	0.032351802	0.071388838	SATB2-AS1
ENSG00000151164	22.31083575	5.830946549	1.917841637	0.032399289	0.071481616	RAD9B
ENSG00000260271	4.192301256	0	4.403049347	0.032402101	0.071481616	AL132996.1
ENSG00000228734	4.192301256	0	4.403049347	0.032402101	0.071481616	AC096543.2
ENSG00000279989	30.02962034	12.60621527	1.255493747	0.032414587	0.07150309	AC011815.3
ENSG00000212135	0.586481695	6.665595026	-3.48393035	0.032423863	0.071512702	SNORD67
ENSG00000174749	177.4712402	316.3488467	-0.834725191	0.03242445	0.071512702	FAM241A

ENSG00000254744	4.888420562	0	4.622131736	0.032449778	0.071556415	AC084855.1
ENSG00000286510	4.888420562	0	4.622131736	0.032449778	0.071556415	AC018695.8
ENSG00000130332	816.6500162	1106.435465	-0.4381997	0.032496076	0.071652427	LSM7
ENSG00000004766	452.0144322	326.2407462	0.469744835	0.032516969	0.07169241	VPS50
ENSG00000267281	50.78377498	24.15214172	1.078954104	0.032550074	0.071759311	ATF7-NPFF
ENSG00000105669	1508.951157	2228.828206	-0.562606177	0.032558584	0.071771982	COPE
ENSG00000280132	48.60773257	26.33279826	0.887213439	0.032592203	0.071839995	AC026471.6
ENSG00000250318	45.0432125	18.27416891	1.302132731	0.032605523	0.071863259	AC003072.1
ENSG00000258813	9.142435692	1.069617038	3.088569065	0.032614703	0.071877394	AL442663.3
ENSG00000138686	328.0488758	515.4110408	-0.651677951	0.032632605	0.071910748	BBS7
ENSG00000189369	214.610669	310.4692384	-0.533470741	0.032665803	0.071977798	GSPT2
ENSG00000198833	1334.239406	1820.253389	-0.448180833	0.032674851	0.071986121	UBE2J1
ENSG00000180458	0.879722543	8.112976048	-3.185072048	0.032675121	0.071986121	AC022148.1
ENSG00000225649	106.7656792	27.52082209	1.95165883	0.032716066	0.072070214	AC064875.1
ENSG00000283930	24.41492163	4.962020471	2.295193595	0.032719704	0.072072117	AL117339.5
ENSG00000280355	43.16306043	19.42162219	1.159821205	0.032724435	0.072074144	AL132656.4
ENSG00000154874	24.68734865	60.60890119	-1.297794515	0.032726172	0.072074144	CCDC144B
novel.1057	32.74715528	8.909926477	1.864224886	0.032741437	0.072101652	-
ENSG00000271192	0	4.350443801	-4.701125889	0.032744726	0.072102782	AC006058.2
ENSG00000035862	18251.43994	12275.59215	0.572203388	0.032749713	0.072107653	TIMP2
ENSG00000112855	618.9576665	842.2092741	-0.444279189	0.032758045	0.072119886	HARS2
ENSG00000125898	111.072747	177.7445731	-0.678852138	0.032783806	0.072170485	FAM110A
ENSG00000253686	4.204909015	0	4.406939793	0.032787741	0.072173031	LINC01484
ENSG00000204262	5145.483273	8808.159784	-0.77557034	0.032798068	0.072189648	COL5A2
ENSG00000188234	314.2240738	183.7963613	0.775372149	0.032836924	0.072269049	AGAP4
ENSG00000135976	268.6249104	133.4999584	1.009400066	0.032874505	0.072345631	ANKRD36
ENSG00000167005	1609.588167	2163.052867	-0.426411963	0.032890078	0.07237377	NUDT21
ENSG00000223478	20.97314513	47.36569346	-1.172705766	0.032894768	0.072377961	AL441992.1
ENSG00000056487	5.127469429	0.387220594	3.7309312	0.032924547	0.072437349	PHF21B
ENSG00000230207	5.140077187	16.62018916	-1.6909027	0.03294418	0.072474406	RPL4P5
ENSG00000128422	22.86325512	6.268917821	1.855404856	0.03295103	0.072478352	KRT17
ENSG00000226469	3.928036872	0	4.307861036	0.032951553	0.072478352	ADAM1B
ENSG00000271320	4.470427048	0	4.496131751	0.032959002	0.0724886	AC091488.1
ENSG00000135423	7.316190867	0.713078025	3.350950302	0.032976068	0.072519996	GLS2
ENSG00000225721	0.894837599	7.254662455	-3.005551991	0.032986543	0.0725344	AL592166.1
ENSG00000131374	1566.339774	1139.476235	0.459256739	0.032988202	0.0725344	TBC1D5
ENSG00000157554	131.9294446	45.300828	1.544971493	0.032992191	0.072537034	ERG
ENSG00000242759	48.68728226	23.45108884	1.049554531	0.033002937	0.072554519	LINC00882
ENSG00000105656	337.2408011	606.5297764	-0.84633763	0.033008334	0.072560244	ELL
ENSG00000138134	228.0724094	512.8783657	-1.169669512	0.033020507	0.072580862	STAMBPL1
ENSG00000184937	0	6.653826665	-5.317081985	0.033046758	0.072632419	WT1
ENSG00000147316	207.9394548	292.6941472	-0.493040678	0.033053317	0.072640689	MCPH1
ENSG00000131650	29.93796109	13.88127516	1.104956567	0.03307148	0.072670112	KREMEN2
ENSG00000144559	108.4461162	176.5044328	-0.704263988	0.033072299	0.072670112	TAMM41
ENSG00000232287	5.518922506	0	4.797034947	0.033090621	0.072704221	SLC6A1-AS1
ENSG00000267199	62.53053304	20.37454906	1.614885457	0.033110495	0.072741736	AP001029.2
ENSG00000174871	18.2228015	47.34459689	-1.37272144	0.033133194	0.07278055	CNIH2
ENSG00000162236	843.6728629	1167.725431	-0.46892561	0.033133765	0.07278055	STX5
ENSG00000278133	207.7589004	132.4882029	0.649077311	0.033161938	0.072836276	AC135050.6
ENSG00000204946	524.6469301	344.9940864	0.605592958	0.033184396	0.072879441	ZNF783
ENSG00000177994	9.648327511	0.713078025	3.752480778	0.033235151	0.072980781	C2orf73

ENSG00000232759	4.985094408	198.4686663	-5.313216272	0.033236157	0.072980781	AC002480.1
ENSG00000197467	178.3821882	881.0857561	-2.304443227	0.033258674	0.073024053	COL13A1
ENSG00000222009	316.565998	522.1588859	-0.721783652	0.033261778	0.073024696	BTBD19
novel.955	63.37463926	0	8.319395628	0.033271551	0.073039979	-
ENSG00000162643	29.15637951	95.03649861	-1.706372175	0.033305546	0.073108431	WDR63
ENSG00000159459	559.5462223	797.3134924	-0.51071779	0.03331209	0.073116618	UBR1
ENSG00000167863	1395.809096	2115.672593	-0.600060327	0.033320302	0.073128464	ATP5PD
ENSG00000188542	97.54430671	58.19306648	0.745465494	0.033361184	0.073212005	DUSP28
novel.530	35.86037496	102.7990877	-1.516681853	0.033373299	0.073232405	-
ENSG00000279434	21.96974244	8.489584156	1.365276408	0.033405104	0.073296007	AL049776.1
ENSG00000184436	603.1570075	408.0240669	0.563427463	0.033414972	0.073311466	THAP7
ENSG00000086991	21.06265309	50.32567148	-1.258265695	0.033427418	0.07333258	NOX4
ENSG00000172965	440.2699677	613.4063019	-0.478288638	0.033430667	0.073333517	MIR4435-2HG
ENSG00000113387	1817.050249	2695.23572	-0.568850231	0.033438381	0.073344245	SUB1
ENSG00000212807	5.1703073	0.356539013	3.741337529	0.033445498	0.073353663	OR2A42
ENSG00000012504	4.913564808	0	4.629014086	0.033478821	0.073420549	NR1H4
ENSG00000099810	701.6629237	945.927619	-0.430903015	0.033483887	0.073425461	MTAP
ENSG00000214029	147.0310498	84.58846499	0.799234354	0.03350214	0.073459287	ZNF891
ENSG00000117525	1175.589194	3332.109592	-1.503032207	0.033577472	0.073616901	F3
ENSG00000023318	1140.984344	1586.769295	-0.4759916	0.033579689	0.073616901	ERP44
ENSG00000137076	10637.18957	16603.37292	-0.642337744	0.033595875	0.073646171	TLN1
ENSG00000111245	0.879722543	9.690099862	-3.437153442	0.033666828	0.073795482	MYL2
ENSG00000105202	86.61136909	203.2892359	-1.230502872	0.03368105	0.073820429	FBL
ENSG00000153147	1579.66525	2122.548755	-0.426232115	0.033686436	0.073821671	SMARCA5
ENSG00000163104	590.0293371	775.637791	-0.394622075	0.033688967	0.073821671	SMARCAD1
ENSG00000189134	54.6329029	26.48877471	1.03890839	0.033690141	0.073821671	NKAPL
ENSG00000229635	8.974845154	1.436768536	2.645585691	0.033701237	0.073839757	AL137159.1
ENSG00000110711	1421.539884	1044.607413	0.444657503	0.033728521	0.073893304	AIP
ENSG00000203506	10.34946141	1.773238453	2.535992618	0.033810122	0.074065832	RBMS3-AS2
novel.228	69.53893747	32.46110401	1.101020287	0.033845402	0.074136868	-
ENSG00000225790	1.22840902	9.732678199	-2.993969821	0.033848695	0.07413783	AC007953.1
ENSG00000104537	9.551511122	0.336469917	4.626711437	0.033863564	0.074164145	ANXA13
ENSG00000272677	28.06894967	8.959392885	1.647677884	0.033890347	0.074216547	AC124016.1
ENSG00000135862	14195.90453	19379.17749	-0.449030908	0.033899795	0.07423098	LAMC1
ENSG00000023839	30.20222547	13.03485833	1.204081215	0.033912326	0.074252161	ABCC2
ENSG00000105778	690.7203563	1082.489403	-0.647868266	0.033918586	0.074259609	AVL9
ENSG00000104671	545.7071316	366.9183013	0.572345785	0.033929504	0.074272026	DCTN6
ENSG00000132953	482.7777104	657.2624114	-0.445143613	0.033929975	0.074272026	XPO4
ENSG00000132613	859.0679988	429.1189416	1.001823767	0.033975199	0.074364755	MTSS2
ENSG00000232411	7.458281153	0.743759607	3.358374199	0.033997846	0.074408056	AC009495.2
ENSG00000230635	5.041793687	0	4.669728961	0.034007166	0.074422184	CYP4F60P
ENSG00000158859	218.9441076	401.9993043	-0.876181252	0.034010913	0.074424116	ADAMTS4
novel.803	60.13302068	28.19106499	1.091105016	0.034038288	0.074477747	-
ENSG00000238245	11.00106192	0.693008929	3.965286246	0.034069919	0.074538596	MYO5BP2
ENSG00000168884	789.7778652	1131.434828	-0.518386934	0.034071836	0.074538596	TNIP2
ENSG00000287265	17.20392325	1.426156051	3.587919539	0.034080304	0.074550845	AL365272.1
ENSG00000273454	3.957013336	0	4.317316363	0.034101307	0.074590508	AC112503.2
ENSG00000146858	100.8316699	167.4680084	-0.732340484	0.034125778	0.074634157	ZC3HAV1L
ENSG00000183793	15.49949652	4.168666062	1.875482967	0.034127008	0.074634157	NPIPA5
ENSG00000154767	614.0824225	841.7651726	-0.454697433	0.034140689	0.074657792	XPC
ENSG00000232320	206.5246494	398.174253	-0.947153757	0.034155307	0.074683474	AC009299.2

ENSG00000147394	260.5512542	467.8463949	-0.844233472	0.034159741	0.074686883	ZNF185
ENSG00000276127	5.40559522	0.336469917	3.807775935	0.034219393	0.074809054	AL589935.2
ENSG00000162931	22.39748041	6.409401493	1.803212748	0.034221377	0.074809054	TRIM17
ENSG00000287774	0	6.513342993	-5.287697498	0.034237192	0.074837328	AC025614.1
ENSG00000134256	17.14081319	3.892403433	2.13014873	0.034268427	0.074899301	CD101
ENSG00000164404	10.88300477	1.345879667	2.974286956	0.034273587	0.074902553	GDF9
novel.506	104.1698198	53.45565887	0.959392982	0.03427819	0.074902553	-
ENSG00000140406	541.2210193	781.9908983	-0.531251768	0.034283669	0.074902553	TLNRD1
ENSG00000088356	630.1921338	392.8446275	0.681209132	0.03428429	0.074902553	PDRG1
ENSG00000257285	44.45952284	22.69556088	0.965152397	0.034284329	0.074902553	AL132780.1
ENSG00000233491	0.925067711	11.01847897	-3.588189581	0.034317315	0.074968313	AC008163.1
ENSG00000214274	107.453964	65.21977684	0.72339355	0.03432053	0.074969035	ANG
ENSG00000038532	453.9832656	593.1553439	-0.385899082	0.034416856	0.075173127	CLEC16A
ENSG00000280062	0.308355904	4.718751175	-3.85512845	0.034435504	0.075203986	AL031719.1
ENSG00000184274	3.926783224	0	4.307445149	0.034442377	0.075203986	LINC00315
ENSG00000221882	3.926783224	0	4.307445149	0.034442377	0.075203986	AC087498.2
ENSG00000108406	639.1913403	952.6685845	-0.575465315	0.034442563	0.075203986	DHX40
ENSG00000122359	2684.992854	3883.362674	-0.532454036	0.034477241	0.075273378	ANXA11
ENSG00000113070	1295.305978	602.8918027	1.103704786	0.034485076	0.075284157	HBEGF
ENSG00000083642	711.5245704	535.5691493	0.409553406	0.034515192	0.075343572	PDS5B
ENSG00000197563	334.7932568	554.0296848	-0.727315028	0.034563316	0.075442285	PIGN
ENSG00000226981	12.27990198	2.221822211	2.486830074	0.034571969	0.075454833	ABHD17AP6
ENSG00000151692	315.4804469	461.4208239	-0.548417913	0.03457528	0.07545572	RNF144A
ENSG00000226659	9.24190157	0.356539013	4.579133837	0.034594594	0.075491528	AC021028.1
ENSG00000130731	889.6063417	1339.968499	-0.590702178	0.034601849	0.07550102	METTL26
ENSG00000145777	5.128723077	18.21969382	-1.820946129	0.034604931	0.075501403	TSLP
ENSG00000247134	184.6391597	91.23129399	1.019116003	0.034629883	0.075546729	AC090204.1
novel.97	7.38925885	0	5.218308007	0.034631521	0.075546729	-
ENSG00000165118	313.6236872	195.9982874	0.67673289	0.034638128	0.075554797	C9orf64
ENSG00000107077	644.7984615	403.3284413	0.677627169	0.034642403	0.07555778	KDM4C
ENSG00000095906	1018.174571	1399.856222	-0.459366211	0.034652472	0.075573397	NUBP2
ENSG00000122033	704.6159169	517.0826498	0.446730372	0.034679473	0.075621154	MTIF3
ENSG00000102309	488.8559895	703.8973982	-0.525863599	0.034680191	0.075621154	PIN4
ENSG00000160188	11.38123216	1.722487775	2.699749213	0.034691946	0.075640437	RSPH1
ENSG00000117592	3546.487803	5057.615575	-0.512128996	0.034713129	0.075680273	PRDX6
ENSG00000118620	160.6648964	109.9094605	0.54869158	0.034755483	0.075766253	ZNF430
ENSG00000105968	3219.672518	2240.748944	0.522813035	0.034765749	0.075782273	H2AFV
ENSG00000251493	313.2650244	1437.823053	-2.198513094	0.034778105	0.075802848	FOXD1
ENSG00000286150	4.191047608	0	4.402603074	0.034793238	0.07581675	AC097459.1
ENSG00000165188	4.191047608	0	4.402603074	0.034793238	0.07581675	RNF183
ENSG00000264630	4.191047608	0	4.402603074	0.034793238	0.07581675	PRKCA-AS1
ENSG00000123342	290.6746135	593.9696886	-1.030645494	0.034815457	0.075858804	MMP19
ENSG00000169627	14.59713703	55.39659528	-1.922342833	0.034819961	0.075862256	BOLA2B
ENSG00000188566	302.8730436	492.8611478	-0.70250854	0.034825261	0.075867442	NDOR1
ENSG00000143486	951.9921346	1289.211675	-0.437380994	0.034841327	0.075896078	EIF2D
ENSG00000065809	1111.017178	567.0680201	0.970669337	0.034858306	0.075926698	FAM107B
ENSG00000272021	4.836735879	0.336469917	3.646303704	0.034874321	0.075955212	AC008592.5
ENSG00000278903	3.940644631	14.02060296	-1.831512114	0.034902932	0.076011155	CU633906.2
ENSG00000102858	1211.740482	1715.976975	-0.501883601	0.034911601	0.076023661	MGRN1
ENSG00000236094	0	3.597227584	-4.427662993	0.034917847	0.07603089	LINC00545
ENSG00000285571	9.402867858	1.773238453	2.397399013	0.034922619	0.076034908	AL513548.4

ENSG00000121454	15.03246816	39.02656317	-1.374965246	0.034946071	0.076079594	LHX4
ENSG00000079332	2475.215445	3387.432105	-0.452714557	0.034962214	0.076108361	SAR1A
ENSG00000137210	698.0826318	961.3712147	-0.461702132	0.034968685	0.07611607	TMEM14B
ENSG00000205220	291.8503693	183.0435302	0.674214466	0.035002089	0.076182398	PSMB10
ENSG00000246331	4.766175193	0	4.588325398	0.035006011	0.076184552	AC010198.1
ENSG00000223764	35.9569341	417.1974851	-3.536737716	0.035053793	0.07628215	LINC02593
ENSG00000279277	17.79506353	5.216929734	1.789669196	0.035058041	0.076284325	AC012020.1
novel.250	3.955759687	0	4.316921546	0.035063601	0.076284325	-
ENSG00000225079	3.955759687	0	4.316921546	0.035063601	0.076284325	FTH1P22
ENSG00000227401	0	4.534533291	-4.755740589	0.035091928	0.076339562	RPL37P1
ENSG00000119669	827.8842998	1480.553376	-0.83847509	0.035101058	0.076353029	IRF2BPL
ENSG00000168803	93.51834199	168.3309927	-0.849377878	0.035116541	0.076380314	ADAL
novel.1070	5.999598808	0.387220594	3.958033845	0.035155233	0.076458071	-
ENSG00000255220	0	3.577158488	-4.420568779	0.035174879	0.076483913	DDX18P5
ENSG00000225950	0	3.577158488	-4.420568779	0.035174879	0.076483913	NTF4
ENSG00000143995	96.64431197	174.6963563	-0.85259755	0.035175947	0.076483913	MEIS1
ENSG00000227192	3.944405577	0	4.313206358	0.035195496	0.076513612	AL023581.2
ENSG00000004809	3.944405577	0	4.313206358	0.035195496	0.076513612	SLC22A16
ENSG00000011295	649.7399904	841.3132624	-0.372923014	0.035218263	0.076556701	TTC19
ENSG00000177200	1447.718012	1018.94677	0.506609882	0.035224035	0.076562842	CHD9
novel.658	59.08263048	113.7440968	-0.944354755	0.035230717	0.076570478	-
ENSG00000205835	0	3.943154111	-4.560897845	0.035236933	0.076570478	GMNC
ENSG00000275088	6.398645048	0.336469917	4.048960546	0.035239063	0.076570478	AC026469.1
ENSG00000224331	3.940644631	0	4.31198225	0.035239337	0.076570478	AC019181.1
ENSG00000186051	5.102253911	0.356539013	3.72472141	0.035245302	0.07657552	TAL2
ENSG00000173914	498.9601487	365.6172174	0.448714047	0.035247552	0.07657552	RBM4B
ENSG00000176928	12.47772263	31.23448305	-1.323288361	0.035255504	0.076586392	GCNT4
ENSG00000148680	76.80214657	179.5220968	-1.226292144	0.035261124	0.076592196	HTR7
ENSG00000164211	290.5524126	481.6355906	-0.72894935	0.035313193	0.076698886	STARD4
ENSG00000085465	48.68567261	22.3672633	1.1238095	0.035316519	0.076699697	OVGP1
ENSG00000260545	5.391733813	0.336469917	3.80447969	0.035364841	0.076798223	AC026771.1
ENSG00000107779	859.8355713	617.4824221	0.477149397	0.035368246	0.076799198	BMPR1A
ENSG00000239961	7.989673224	0	5.331210344	0.035390048	0.076840118	LILRA4
ENSG00000267413	0.308355904	4.596024848	-3.82331853	0.035408915	0.076874657	LINC01901
ENSG00000179935	11.4430173	2.822786395	2.008579608	0.035455595	0.07696957	LINC00652
ENSG00000100813	3365.459008	4796.189829	-0.51103654	0.035494831	0.077048307	ACIN1
ENSG00000262587	37.13093767	17.13026447	1.112600269	0.03549792	0.077048576	AC133552.2
novel.229	10.95474784	26.31272916	-1.265276111	0.035519504	0.077088984	-
ENSG00000130119	560.5331888	870.4718605	-0.635357119	0.035527571	0.077094441	GNL3L
ENSG00000256422	3.914175465	0	4.303296731	0.03553092	0.077094441	LINC02552
novel.631	3.914175465	0	4.303296731	0.03553092	0.077094441	-
ENSG00000204103	1314.080221	464.4067067	1.500944616	0.035545594	0.077107482	MAFB
ENSG00000235563	3.912921816	0	4.302886743	0.035545834	0.077107482	AL445183.2
ENSG00000183662	3.912921816	0	4.302886743	0.035545834	0.077107482	TAFA1
ENSG00000278876	2.44546393	10.95698741	-2.172659741	0.035558023	0.077127483	AC145207.9
ENSG00000138802	712.8664927	951.8778451	-0.417028318	0.035566966	0.077136222	SEC24B
ENSG00000136011	16.04070432	5.330327845	1.583612104	0.03556799	0.077136222	STAB2
ENSG00000230806	6.958728849	0.713078025	3.279856509	0.035576582	0.077148416	SRGAP2-AS1
ENSG00000163959	73.83174479	37.15760274	0.990492653	0.035696154	0.077401251	SLC51A
ENSG00000283959	8.510608828	27.40827589	-1.686201915	0.035733082	0.077474856	AP002851.1
ENSG00000121753	244.5996028	527.5351404	-1.108387846	0.035827244	0.077672533	ADGRB2

ENSG00000187867	34.93490781	10.28173583	1.75998141	0.035835563	0.077684085	PALM3
ENSG00000166840	6.04118303	18.81963052	-1.636725713	0.035841787	0.077691094	GLYATL1
ENSG00000117523	2262.343481	3145.277898	-0.475318432	0.035864387	0.077733598	PRRC2C
ENSG00000183853	2965.795956	4529.117995	-0.610737694	0.03586752	0.077733903	KIRREL1
ENSG00000159079	279.4852623	199.3222064	0.486741884	0.035879528	0.077753442	CFAP298
ENSG00000103449	143.0583798	288.1431283	-1.009267532	0.035904543	0.077801163	SALL1
ENSG00000117632	3636.720501	5819.768378	-0.678360455	0.035920838	0.07782998	STMN1
ENSG00000152467	18.52760993	5.4318292	1.774138311	0.035933001	0.077849842	ZSCAN1
ENSG00000164048	276.831735	173.8374004	0.672955588	0.035940985	0.077860647	ZNF589
ENSG00000166823	26.54640216	7.695073872	1.77647928	0.035950617	0.077875019	MESP1
ENSG00000105676	420.6597721	697.1375192	-0.729142435	0.036026694	0.078033309	ARMC6
ENSG00000230311	19.37341268	36.62745832	-0.918541071	0.036033304	0.078041119	TOMM20P4
ENSG00000248927	2.34592678	20.19991618	-3.097641463	0.036043301	0.078056265	AC114284.1
ENSG00000175449	29.47712971	13.3192933	1.149029044	0.03607078	0.078109264	RFESD
ENSG00000141198	59.01117215	121.0019698	-1.037464282	0.036107229	0.078181675	TOM1L1
ENSG00000258303	1.495180701	9.643073599	-2.688863777	0.036119367	0.078190447	AC012464.2
ENSG00000229029	6.859191699	0.693008929	3.27779378	0.036121704	0.078190447	CDCA4P1
ENSG00000255639	4.53339457	0	4.514523932	0.036123058	0.078190447	AC005833.1
ENSG00000135482	209.424179	131.6849595	0.671325416	0.036123318	0.078190447	ZC3H10
ENSG00000124702	1323.677391	1930.966491	-0.544857456	0.03615339	0.078247771	KLHDC3
ENSG00000106524	435.0632615	283.788763	0.616233033	0.036155825	0.078247771	ANKMY2
ENSG00000198087	532.1701288	777.9595607	-0.547622007	0.036159393	0.078248975	CD2AP
ENSG00000102189	807.4986631	532.744436	0.599513997	0.03618507	0.078297447	EEA1
ENSG00000244242	26.18718794	8.337332124	1.639337382	0.03618782	0.078297447	IFITM10
ENSG00000231473	4.849343637	0.387220594	3.649511349	0.036214135	0.07834786	RB1-DT
ENSG00000272054	28.39109571	9.934653428	1.510430779	0.036219974	0.078353967	AC007390.2
ENSG00000253395	4.524619029	0	4.511965698	0.036223354	0.078354754	AP003469.2
ENSG00000197694	8631.765285	6043.572824	0.514195815	0.036279902	0.078470541	SPTAN1
ENSG00000114857	3342.010136	1896.782252	0.817204364	0.036296897	0.078500764	NKTR
ENSG00000116752	464.0353963	705.7230696	-0.605235477	0.036304512	0.078508216	BCAS2
ENSG00000148824	98.68933382	172.2841173	-0.803225072	0.036306386	0.078508216	MTG1
ENSG00000169564	206.3287239	292.5339796	-0.504401689	0.03631588	0.078521394	PCBP1
ENSG00000259175	7.634647232	1.060160428	2.834352017	0.036318581	0.078521394	AC108451.1
ENSG00000099992	423.9246413	280.6313861	0.595894795	0.03632205	0.078521394	TBC1D10A
ENSG00000237877	18.10467309	6.899279318	1.399939608	0.036324569	0.078521394	LINC01473
ENSG00000260953	4.853104584	0.356539013	3.650463823	0.036383634	0.07863633	AC009093.4
ENSG00000263675	11.24798869	2.833398881	1.979075214	0.036383793	0.07863633	MIR5581
ENSG00000250942	5.976961859	0.336469917	3.953071064	0.036405481	0.07867666	ENPP7P11
ENSG00000078070	477.7884556	344.7319979	0.470285948	0.036410405	0.078680756	MCCC1
ENSG00000168061	252.5367649	388.0313914	-0.62041585	0.036423511	0.078702269	SAC3D1
ENSG00000131966	980.1474557	1340.716346	-0.452167457	0.036426419	0.078702269	ACTR10
ENSG00000143294	892.4976634	1183.723093	-0.407385475	0.036444471	0.078734724	PRCC
ENSG00000259664	4.496896214	0	4.503826071	0.036543312	0.078941697	LINC02254
ENSG00000151882	135.1049636	58.4704378	1.210073533	0.036581512	0.079017647	CCL28
ENSG00000009413	305.9201599	469.6535851	-0.619136297	0.036598838	0.0790485	REV3L
ENSG00000196357	94.04873766	53.87916485	0.8062533	0.036613542	0.079073686	ZNF565
ENSG00000276048	4.914818457	0	4.629423816	0.036625835	0.079087086	RF02250
ENSG00000230285	4.914818457	0	4.629423816	0.036625835	0.079087086	AC092807.1
ENSG00000250091	6.055044437	0.723690511	3.073158942	0.036636054	0.079102578	DNAH10OS
ENSG00000163660	2564.655624	1505.612471	0.768511852	0.036675117	0.07918034	CCNL1
ENSG00000164167	212.6311223	325.0201617	-0.612249627	0.036749933	0.079335274	LSM6

ENSG00000284693	57.95633718	30.81902102	0.911236334	0.036818255	0.0794741	LINC02606
ENSG00000224743	1.833766717	9.007831812	-2.301844129	0.036820359	0.0794741	TEX26-AS1
ENSG00000124789	725.2914782	1087.137035	-0.583869295	0.036824737	0.079476948	NUP153
ENSG00000181227	21.31270006	7.102410423	1.581476161	0.036841983	0.079507565	DLSTP1
ENSG00000183048	67.26901325	122.2128884	-0.863192002	0.036853208	0.079525183	SLC25A10
ENSG00000136104	614.4468647	380.6311079	0.690061297	0.036881282	0.079579154	RNASEH2B
ENSG00000011243	687.0326929	919.8043603	-0.420706647	0.03688648	0.07958376	AKAP8L
ENSG00000111328	2715.052447	3639.10228	-0.422622438	0.036918524	0.079646281	CDK2AP1
ENSG00000277639	121.6047993	40.26366822	1.594427865	0.036926623	0.079657139	AC007906.2
ENSG00000251148	0	6.33729745	-5.242506111	0.036942323	0.079684392	AL158068.2
ENSG00000186001	1983.51822	1113.238563	0.833398336	0.036960557	0.07970604	LRCH3
ENSG00000086717	8.469024606	1.069617038	2.979109053	0.03696129	0.07970604	PPEF1
ENSG00000012660	3274.866087	2430.959994	0.429825205	0.036961563	0.07970604	ELOVL5
ENSG00000115226	369.9012687	229.38013	0.690048963	0.036989586	0.07975985	FNDC4
novel.32	12.58345675	1.874739808	2.769313583	0.037006491	0.079789679	-
ENSG00000206754	2.431602522	9.661858425	-1.993838754	0.037074531	0.079929748	SNORD101
ENSG00000285693	49.60747792	24.75439017	1.001902348	0.03708698	0.079949952	AP002381.2
ENSG00000204740	15.61045905	2.751966622	2.482510294	0.037090236	0.079950339	MALRD1
ENSG00000236154	4.548509626	0	4.518941002	0.037116097	0.079999446	AL450311.1
ENSG00000239415	7.220485935	23.80621519	-1.715979818	0.037124888	0.080006221	AP001469.3
ENSG00000229659	7.783005762	1.10029862	2.841447427	0.037125399	0.080006221	RPL26P6
ENSG00000254783	14.74298826	4.596024848	1.673530019	0.037149234	0.080049642	AP003084.1
ENSG00000184205	1469.602094	1016.586006	0.532036683	0.03715171	0.080049642	TSPYL2
ENSG00000256029	25.62724668	9.435190599	1.446711455	0.037161675	0.080064472	AL590560.1
ENSG00000090861	2301.974702	3314.320334	-0.525930584	0.037193751	0.080126935	AARS
ENSG00000108774	1241.812467	1732.562327	-0.480453701	0.037206195	0.080147098	RAB5C
ENSG00000278996	4.581318307	0	4.528518273	0.03723042	0.080187425	FP671120.2
ENSG00000258416	4.538480436	0	4.516019831	0.037234175	0.080187425	AF123462.1
ENSG00000251611	4.538480436	0	4.516019831	0.037234175	0.080187425	FAM160A1-DT
ENSG00000080493	33.95335463	97.58019704	-1.524969047	0.03726098	0.080238502	SLC4A4
ENSG00000163788	612.4855805	388.4341388	0.656402102	0.037267329	0.080245522	SNRK
ENSG00000136381	841.4263467	1113.08846	-0.403738055	0.037296302	0.080301254	IREB2
novel.264	5.194269168	0	4.709838697	0.037305286	0.080303923	-
novel.110	5.1916906	0.356539013	3.746626677	0.037306815	0.080303923	-
ENSG00000279839	5.1916906	0.356539013	3.746626677	0.037306815	0.080303923	AL512383.1
ENSG00000107537	383.2630161	560.5890956	-0.548458334	0.037400806	0.080499573	PHYH
ENSG00000007402	44.04593101	15.53880376	1.503819518	0.037418157	0.080530247	CACNA2D2
ENSG00000287156	7.078395649	0	5.15629409	0.037424443	0.080537105	AL138479.2
ENSG00000135775	260.4475727	372.1791277	-0.514959425	0.037438062	0.080559739	COG2
ENSG00000271871	7.617024878	1.10029862	2.807324624	0.037455052	0.080584873	AC005740.4
ENSG00000232190	7.393019796	0	5.21904806	0.037455946	0.080584873	LINC02181
ENSG00000287299	38.71562633	16.0180691	1.276902983	0.037538177	0.080751581	AC012459.1
ENSG00000138172	487.0325635	280.6976635	0.795240061	0.037539648	0.080751581	CALHM2
ENSG00000162891	0	6.398660613	-5.255813495	0.037547511	0.080759401	IL20
ENSG00000266473	9.657103052	2.496928964	1.947682276	0.0375495	0.080759401	AC007448.3
ENSG00000138031	1264.936936	865.0051332	0.54819708	0.037584776	0.080828579	ADCY3
ENSG00000099995	1496.473835	1939.07573	-0.37374126	0.037600283	0.080855235	SF3A1
ENSG00000156875	23.84126116	54.12757123	-1.184933173	0.037620436	0.080891878	MFSD14A
ENSG00000203588	9.279440112	1.793307549	2.372804744	0.037645183	0.08093839	IGBP1-AS1
ENSG00000253730	7.250716047	0.774441189	3.291566601	0.0376676	0.080979887	AC015909.2
ENSG00000173715	161.2543561	88.51673426	0.863176938	0.037706225	0.081056216	C11orf80

ENSG00000163002	138.2723597	238.8664693	-0.789820995	0.0377345	0.081110288	NUP35
ENSG00000076067	2237.741532	1389.248697	0.687808151	0.037770697	0.081181377	RBMS2
novel.965	20.60934359	79.12569775	-1.939084761	0.037776295	0.081186692	-
ENSG00000099957	97.91326503	47.82257796	1.037821944	0.037789901	0.081209215	P2RX6
ENSG00000154099	13.95012384	3.280826763	2.096135621	0.037810719	0.0812463	DNAAF1
ENSG00000110944	15.81544559	35.41735761	-1.165231741	0.037813412	0.0812463	IL23A
ENSG00000173868	33.16980739	12.75962318	1.384536628	0.037818928	0.081251433	PHOSPHO1
ENSG00000231584	72.24741214	28.45928367	1.338497993	0.037835858	0.081277658	FAHD2CP
ENSG00000118520	4.488049402	0	4.501190667	0.037837392	0.081277658	ARG1
novel.37	15.09418203	4.381125383	1.794481495	0.0378557	0.081310264	-
ENSG00000163576	13.0226201	3.913628404	1.728712199	0.037892554	0.081382694	EFHB
ENSG00000170092	9.823083936	30.19336423	-1.615038666	0.037903284	0.08139901	SPDYE5
ENSG00000145293	1209.272022	901.5277641	0.423484082	0.037933138	0.08145639	ENOPH1
ENSG00000120253	635.4756418	865.967354	-0.446737158	0.037949763	0.081485354	NUP43
ENSG00000198417	111.1419122	51.73736224	1.106474351	0.037983492	0.081551038	MT1F
ENSG00000254910	71.36551047	24.08962268	1.570183466	0.038024461	0.081632252	AC136475.2
ENSG00000254656	4.848089989	16.93427823	-1.804277658	0.038032091	0.081641887	RTL1
ENSG00000235454	0.615458159	6.612404203	-3.434567125	0.038056558	0.08168766	HAUS6P3
ENSG00000260581	7.074634703	0	5.155562297	0.038091543	0.081755999	AC011374.1
ENSG00000104879	3.954506038	0	4.316545699	0.03809785	0.081759095	CKM
ENSG00000115380	834.9786879	6357.420137	-2.928657469	0.038099279	0.081759095	EFEMP1
ENSG00000197016	299.8611849	185.8661882	0.691565412	0.038133215	0.081825163	ZNF470
ENSG00000185591	1681.664258	1284.071237	0.38929157	0.038143771	0.081841053	SP1
ENSG00000198040	746.8867339	537.2627065	0.475545796	0.038148528	0.081844502	ZNF84
ENSG00000260618	17.46525306	5.074134312	1.788688612	0.038156313	0.081854444	AC025917.1
ENSG00000182704	492.2255616	1438.050658	-1.54683415	0.038161016	0.081857774	TSKU
ENSG00000066405	7.000313071	20.02387064	-1.519481755	0.03817139	0.081870114	CLDN18
ENSG00000187605	376.9648923	231.8192447	0.702416742	0.038173071	0.081870114	TET3
ENSG00000273972	5.085885207	0.336469917	3.720597236	0.038211341	0.081945426	AC068722.2
ENSG00000224769	12.55175952	0.672939833	4.182027969	0.038228352	0.081975142	MUC20P1
ENSG00000260526	20.27075758	8.112976048	1.311368333	0.038238959	0.081986873	AC109347.1
ENSG00000258077	0	3.546476906	-4.409787109	0.03824329	0.081986873	AC078923.1
ENSG00000199436	0	3.546476906	-4.409787109	0.03824329	0.081986873	SNORD9
ENSG00000146834	1001.112722	1409.372481	-0.493515142	0.038250119	0.081994748	MEPCE
ENSG00000287255	39.66214827	6.15680398	2.677379725	0.038258844	0.082006684	AC007877.1
novel.668	7.562832897	25.71150819	-1.760355944	0.03827124	0.082026488	-
ENSG00000163026	165.3782758	256.7757513	-0.635871952	0.038306167	0.082094574	WDCP
ENSG00000147526	2812.149195	1883.041662	0.578515142	0.038325329	0.082128867	TACC1
ENSG00000120868	447.4138381	694.925744	-0.635167099	0.038331136	0.082134536	APAF1
ENSG00000232679	0.308355904	4.340987191	-3.735526679	0.038351611	0.082171632	LINC01705
ENSG00000272812	38.79446436	15.13511009	1.352674784	0.038384855	0.082236079	AC004908.2
ENSG00000147121	319.5970741	217.1795007	0.557156695	0.038402622	0.082267358	KRBOX4
ENSG00000263338	4.178439849	0	4.398578073	0.03842105	0.082300049	AC027796.5
ENSG00000104957	843.9014866	565.1791787	0.578728299	0.038434155	0.082321334	CCDC130
ENSG00000198840	31176.34452	21221.70511	0.554911534	0.038450219	0.082348953	MT-ND3
ENSG00000101115	9.799406802	29.63369412	-1.598887328	0.038458012	0.082358854	SALL4
ENSG00000083168	970.530031	654.9013767	0.56755451	0.038466294	0.082366079	KAT6A
novel.464	157.2376662	403.7189186	-1.360568578	0.038467726	0.082366079	-
ENSG00000248159	6.080259955	0.743759607	3.063808266	0.038478847	0.082383101	HSPA8P11
ENSG00000229117	9662.219999	15007.8674	-0.635306846	0.038491756	0.082403947	RPL41
ENSG00000235674	4.605208904	0	4.535473664	0.038529681	0.082478342	LDHAP2

ENSG00000067082	6515.271898	3322.318495	0.97164088	0.038544469	0.0825032	KLF6
ENSG00000198483	216.2464016	66.27617849	1.7039607	0.038559259	0.082528058	ANKRD35
ENSG00000153207	866.6956336	1177.538034	-0.442002485	0.038582085	0.082563604	AHCTF1
ENSG00000005243	993.212426	509.4900017	0.962593008	0.038582222	0.082563604	COPZ2
ENSG00000257038	52.2162729	22.37098772	1.215492772	0.038657522	0.082717927	AP002761.3
ENSG00000164815	231.7740482	386.6308267	-0.739082081	0.03870476	0.082812186	ORC5
ENSG00000213261	93.89865477	169.5427632	-0.852989501	0.038727064	0.082853084	EEF1B2P6
ENSG00000126005	521.8379052	953.1897265	-0.868891643	0.03873257	0.08285804	MMP24OS
ENSG00000171988	1396.58669	943.3491374	0.566236743	0.038740587	0.082861079	JMJD1C
ENSG00000119965	204.4655537	289.8639591	-0.502883733	0.038740957	0.082861079	C10orf88
ENSG00000260071	4.45656564	0	4.491860751	0.038743559	0.082861079	AC012178.1
ENSG00000275443	9.915027922	1.456837632	2.776824359	0.038792534	0.082958995	AC012409.2
novel.733	55.88058737	164.5166695	-1.556610519	0.038799146	0.082966305	-
ENSG00000274824	3.899060409	0	4.298256945	0.03882737	0.083019825	MIR7152
ENSG00000105058	1269.677684	1762.357917	-0.473202803	0.038838412	0.083036603	FAM32A
ENSG00000167996	21792.90172	45365.96524	-1.057749233	0.038848224	0.083050745	FTH1
ENSG00000158555	538.9033681	256.8257188	1.06963409	0.038871823	0.083094359	GDPD5
ENSG00000259946	6.910876383	0	5.122998985	0.038886629	0.083119169	BX005019.1
ENSG00000281571	43.3103075	16.51740353	1.396662806	0.03890713	0.083156149	AC241585.2
ENSG00000254288	3.972128392	0	4.322335168	0.038915704	0.083167633	AC087672.2
ENSG00000267274	11.158552	2.822786395	1.971925445	0.03893237	0.083196407	AC008770.3
ENSG00000184154	89.41336957	53.06599814	0.750797559	0.038937273	0.08320004	LRTOMT
ENSG00000177494	6.641526132	28.6628815	-2.105073065	0.038949845	0.083220059	ZBED2
ENSG00000207340	0.307102255	4.270167417	-3.715813129	0.038998101	0.08331631	RNVU1-1
ENSG00000226243	10.18054595	33.86372334	-1.730951459	0.039049787	0.083419875	RPL37AP1
ENSG00000203616	10.46125031	1.161661783	3.219813951	0.03908617	0.083490733	RHOT1P2
ENSG00000160213	1731.097232	2605.346056	-0.589765848	0.0390947	0.083502087	CSTB
ENSG00000049768	16.19052996	4.688069593	1.789234039	0.039102873	0.08351268	FOXP3
ENSG00000137145	639.3567948	469.4237408	0.445696623	0.039110749	0.083522635	DENND4C
ENSG00000272701	12.52550382	2.038888596	2.587481101	0.039135306	0.083568208	MESTIT1
ENSG00000173281	9.170158507	29.62166897	-1.690637073	0.03915925	0.083612465	PPP1R3B
ENSG00000227176	8.73328899	1.4981317	2.576182413	0.039185071	0.083660723	AC092641.1
ENSG00000280135	5.200537412	0.356539013	3.748857784	0.03919485	0.083674725	AL096816.1
ENSG00000220472	36.50419668	66.1433064	-0.86061636	0.039204967	0.083689446	AL139095.2
novel.233	51.44464958	28.4141368	0.857553622	0.039210543	0.083694473	-
ENSG00000136932	198.6469796	120.7440256	0.716982974	0.039222759	0.083713669	TRMO
ENSG00000232311	8.714341717	0.672939833	3.646750217	0.039275774	0.083819934	AL512303.1
ENSG00000228363	15.75086841	2.873537073	2.456446823	0.039282185	0.08382673	AC015971.1
ENSG00000155229	1254.7606	1899.279066	-0.597900898	0.039312712	0.083884984	MMS19
ENSG00000124159	12.05596816	3.220619475	1.903630907	0.039328852	0.083912532	MATN4
ENSG00000254300	0.600343103	8.572300685	-3.834715624	0.03933735	0.083923771	LINC01111
ENSG00000165124	473.711285	1852.770665	-1.967710339	0.039371963	0.08399072	SVEP1
ENSG00000106128	8.625189763	0.356539013	4.479445289	0.039402898	0.08404981	GHRHR
ENSG00000105988	2.138361675	8.947624524	-2.072059936	0.039439195	0.08412033	NHP2P1
ENSG00000116586	748.1494737	1014.803033	-0.439805478	0.039463632	0.084165542	LAMTOR2
ENSG00000258454	3.93054417	0	4.308654032	0.039474743	0.084182328	AC016526.2
ENSG00000117305	629.4619956	963.8967921	-0.61444835	0.039510312	0.084251265	HMGCL
ENSG00000057468	18.15983398	3.993904788	2.191077232	0.03953485	0.084296671	MSH4
ENSG00000174453	3.925529575	0	4.307005895	0.039542942	0.084300088	VWC2L
ENSG00000215533	3.925529575	0	4.307005895	0.039542942	0.084300088	LINC00189
ENSG00000197901	52.37537228	0	8.044374206	0.03956355	0.084337101	SLC22A6

ENSG00000180884	52.36087048	161.9572218	-1.628156804	0.039575734	0.084356152	ZNF792
ENSG00000176532	31.25448188	524.538439	-4.068993217	0.039589314	0.084378177	PRR15
ENSG00000113593	873.1634149	628.3403827	0.474738465	0.03959541	0.084384247	PPWD1
ENSG00000204172	93.33710351	185.7527558	-0.99389558	0.039609316	0.084406958	AGAP9
ENSG00000273551	7.083410244	0	5.157302803	0.039617095	0.084416611	AC120498.8
ENSG00000266265	16.29647789	1.365948763	3.553266975	0.039629993	0.08443717	KLF14
ENSG00000029534	143.9456513	37.00689176	1.957060258	0.039644911	0.084462029	ANK1
ENSG00000230453	3.663772488	12.3818592	-1.757541785	0.039655078	0.08447676	ANKRD18B
ENSG00000264705	3.915429114	0	4.303658521	0.039680985	0.084511163	AC090337.1
ENSG00000271815	3.915429114	0	4.303658521	0.039680985	0.084511163	AC008897.3
ENSG00000248242	3.915429114	0	4.303658521	0.039680985	0.084511163	AC004053.1
ENSG00000270640	5.954253639	0.387220594	3.947979975	0.039689521	0.084515487	AC104695.4
novel.187	5.954253639	0.387220594	3.947979975	0.039689521	0.084515487	-
ENSG00000275585	7.128470678	0	5.168279014	0.039702588	0.084536382	AC241377.2
ENSG00000232545	1.802282956	11.33945611	-2.646942694	0.03971515	0.084548179	AC253536.3
ENSG00000127445	1476.904737	1997.171045	-0.435467379	0.039715847	0.084548179	PIN1
ENSG00000163492	7.072127405	0	5.155085422	0.039717891	0.084548179	CCDC141
ENSG00000256824	5.949239044	0.387220594	3.946878342	0.03973057	0.08456548	AP000721.2
ENSG00000232986	3.911668168	0	4.30241929	0.039732528	0.08456548	AL139081.1
ENSG00000139842	1555.653001	2382.523399	-0.614935445	0.039752451	0.084600953	CUL4A
ENSG00000171408	249.9112286	672.3001769	-1.427940173	0.039759435	0.084608885	PDE7B
ENSG00000223549	6.022307028	0.723690511	3.066449837	0.039827636	0.084742063	MTND5P28
ENSG00000149187	1660.247024	2298.074447	-0.468934207	0.039828542	0.084742063	CELF1
ENSG00000206828	6.615056966	0.693008929	3.224166491	0.039832512	0.084743571	RF00003
ENSG00000261039	1.49643435	8.499040767	-2.501262192	0.039857425	0.084789631	LINC02544
ENSG00000285095	6.09662866	0.743759607	3.06671003	0.039885033	0.084841414	AC025887.2
ENSG00000144671	2.080408748	11.41258764	-2.441890038	0.039914248	0.08489661	SLC22A14
ENSG00000203867	51.2247611	11.07496185	2.213518337	0.039935024	0.084933845	RBM20
ENSG00000198920	331.6733142	441.6890127	-0.413013996	0.039995496	0.085055494	KIAA0753
ENSG00000223839	5.364010998	0.387220594	3.797731933	0.040033457	0.085129255	FAM95B1
ENSG00000147894	356.0342573	220.2444348	0.69433902	0.040042157	0.085140787	C9orf72
ENSG00000166912	246.6001062	139.6819345	0.818821565	0.040060202	0.085172185	MTMR10
ENSG00000258315	21.8125371	9.222731278	1.234924971	0.040097023	0.085243495	C17orf49
novel.993	8.866532464	0.336469917	4.521697915	0.040104391	0.085252183	-
ENSG00000229833	73.3061506	40.21163328	0.86949593	0.040126554	0.085292318	PET100
ENSG00000070610	1542.852717	1026.064746	0.588287242	0.040198326	0.085437885	GBA2
ENSG00000283341	60.559505	31.11188511	0.959703011	0.040233414	0.085505467	AC068205.2
ENSG00000215270	16.0081091	1.733100261	3.191385085	0.040257712	0.085550107	AP000523.1
ENSG00000038274	763.2257323	1200.11039	-0.652992282	0.040266725	0.085562263	MAT2B
ENSG00000187098	232.0714335	80.40773942	1.527306038	0.040274313	0.085571388	MITF
ENSG00000283189	18.16520458	5.73646166	1.66199189	0.040282745	0.085582303	AC104452.1
ENSG00000274383	4.854358232	0.336469917	3.650810237	0.040296921	0.085605422	AC103691.1
ENSG00000270001	12.16302721	2.751966622	2.117185164	0.040327677	0.085663753	AL121894.2
ENSG00000233325	37.96001572	18.56690461	1.038716465	0.040334682	0.08567163	MIPEPP3
ENSG00000243646	40.21023757	11.34536388	1.823482949	0.040340168	0.085676277	IL10RB
ENSG00000225077	0.293240848	4.402350354	-3.752502317	0.040357373	0.085705813	LINC00337
ENSG00000160588	48.69000302	10.29478846	2.233611525	0.040364108	0.085710064	MPZL3
ENSG00000185052	85.31658691	17.43720868	2.289521092	0.040365973	0.085710064	SLC24A3
ENSG00000132846	220.1824311	93.1970167	1.238399439	0.040388391	0.085750657	ZBED3
ENSG00000122367	26.30964677	8.500196642	1.623973532	0.040402147	0.085772853	LDB3
ENSG00000132294	955.9236863	1427.666355	-0.578685025	0.040426087	0.085816664	EFR3A

ENSG00000284951	3.973382041	0	4.322773292	0.04046184	0.085885545	AL355798.1
ENSG00000110074	437.1486726	686.9959162	-0.652233358	0.040478737	0.085914391	FOXRED1
ENSG00000205871	12.10632793	26.49681865	-1.12649902	0.040514022	0.085975707	RPS3AP47
ENSG00000187994	3.969621095	0	4.321543509	0.040514244	0.085975707	RINL
ENSG00000013563	291.7015556	428.8838231	-0.556303778	0.040534032	0.086010673	DNASE1L1
ENSG00000139291	450.7072843	267.6916202	0.750562793	0.040570419	0.086080853	TMEM19
ENSG00000184508	104.664144	153.6208012	-0.554983076	0.040579258	0.086092578	HDDC3
ENSG00000238082	0.308355904	4.566499142	-3.81489196	0.040613865	0.086158964	AC009948.1
ENSG00000173218	384.2111205	622.888544	-0.697556577	0.040635365	0.086197535	VANGL1
ENSG00000171862	18.71984651	4.646775526	2.010511186	0.040676968	0.086278741	PTEN
ENSG00000165521	8.481632365	1.069617038	2.981186896	0.040753754	0.086434554	AL121768.1
ENSG00000268403	33.40301529	13.95325081	1.257976936	0.040799312	0.086524115	AC132192.2
ENSG00000236337	10.58593171	2.200597239	2.279885526	0.040810342	0.086534307	FMR1-IT1
ENSG00000060237	3449.598036	2589.868492	0.413521092	0.040810779	0.086534307	WNK1
ENSG00000261668	3.945659226	0	4.31364403	0.040850915	0.08661234	AC093591.2
ENSG00000116128	443.7587862	294.4649455	0.591968512	0.040858432	0.086621209	BCL9
ENSG00000055917	1922.130124	1442.143294	0.414575566	0.040886724	0.086674118	PUM2
ENSG00000130714	920.3498615	640.6669624	0.522816803	0.040893378	0.086681151	POMT1
ENSG00000237234	43.65570375	12.48220468	1.812045325	0.040899569	0.086687201	Z99289.1
ENSG00000170743	3.939390982	0	4.311583874	0.040939616	0.086765002	SYT9
ENSG00000188856	82.5807614	357.3150957	-2.113109896	0.040963238	0.086807984	RPSAP47
ENSG00000182057	13.64058555	32.93129792	-1.27630154	0.04103934	0.086962164	OGFRP1
ENSG00000149972	5.199283763	0.336469917	3.748602533	0.041066396	0.0870124	CNTN5
ENSG00000214954	30.35935955	11.6276155	1.378504651	0.041087721	0.087043734	LRRC69
ENSG00000170374	7.167547603	0.672939833	3.360523055	0.041087885	0.087043734	AC073611.1
ENSG00000130382	2075.09583	2940.435938	-0.502808696	0.041094649	0.087050964	MLLT1
ENSG00000281912	21.5920795	8.254615595	1.389193862	0.041131488	0.087121897	LINC01144
ENSG00000108433	853.9078581	1119.900238	-0.391081245	0.041136841	0.087126131	GOSR2
ENSG00000130943	18.68614019	5.634960305	1.720757212	0.041166958	0.087182812	PKDREJ
ENSG00000277215	3.33904788	12.28164211	-1.885929438	0.041195475	0.087235306	SPANXA2-OT1
ENSG00000160131	728.1985408	960.6185117	-0.399863802	0.041198461	0.087235306	VMA21
ENSG00000174405	460.949275	309.7672865	0.572853526	0.041245522	0.087327838	LIG4
ENSG00000213533	21.59995739	42.90711703	-0.994491044	0.041252814	0.08733616	STIMATE
ENSG00000249201	7.395527094	0	5.219559906	0.041266308	0.087357609	AC114291.1
ENSG00000213462	295.2717748	144.8328648	1.028963078	0.041276229	0.087371494	ERV3-1
ENSG00000260853	85.90909626	158.91963	-0.885584668	0.041285168	0.087383296	AC109460.2
ENSG00000148688	503.2429546	674.1455669	-0.422031159	0.0413074	0.087423229	RPP30
ENSG00000180581	23.72696496	45.87257045	-0.952922247	0.04132614	0.087455766	SRP9P1
ENSG00000285991	7.487257617	0.774441189	3.335916686	0.041343619	0.087485629	AL355312.6
ENSG00000115423	26.38382621	9.282810171	1.519796853	0.041355897	0.087504484	DNAH6
ENSG00000260552	1.830005771	11.33603567	-2.640710025	0.041374345	0.087530208	AC023043.1
ENSG00000273291	9.255478243	0.774441189	3.633594295	0.041374793	0.087530208	AC092042.3
ENSG00000155530	17.02114639	5.889869568	1.54431743	0.041410267	0.087598124	LRGUK
ENSG00000182979	1240.073	1940.842778	-0.646090726	0.041419891	0.087611349	MTA1
ENSG00000114738	558.9075563	791.9766751	-0.502742706	0.041443688	0.087654547	MAPKAPK3
ENSG00000262209	9.672218109	26.82853667	-1.46739568	0.041457718	0.087670291	PCDHGB3
ENSG00000144681	34.73103273	92.90518008	-1.421525586	0.041457881	0.087670291	STAC
ENSG00000161132	12.70416373	3.709341424	1.79734463	0.041473768	0.08769675	AC007663.1
ENSG00000275759	9.352508095	1.702418679	2.421217066	0.041484518	0.087712343	AC026367.3
ENSG00000174460	5.192944248	0.356539013	3.747036576	0.041493946	0.087725137	ZCCHC12
ENSG00000248522	3.900314058	0	4.298620444	0.041499846	0.087730471	SBF1P1

ENSG00000164100	8.907219042	0	5.488205869	0.041528346	0.087778685	NDST3
ENSG00000143947	7982.567956	12332.84332	-0.627589979	0.041536099	0.087778685	RPS27A
ENSG00000220392	3.89780676	0	4.297790016	0.041536167	0.087778685	FCF1P5
ENSG00000260012	3.89780676	0	4.297790016	0.041536167	0.087778685	AC007494.1
ENSG00000117971	16.9595747	0.356539013	5.45561095	0.041545384	0.087791023	CHRNB4
ENSG00000228192	44.74774909	23.85593839	0.910713127	0.041565367	0.087816126	AL512353.1
ENSG00000231156	6.771293394	0	5.092278667	0.041566462	0.087816126	AC093702.1
ENSG00000155287	951.6107263	645.6613339	0.559693919	0.041567404	0.087816126	SLC25A28
ENSG00000255008	12.58345675	2.547679642	2.310308183	0.041597306	0.087872153	AP000442.1
ENSG00000253857	7.107087378	0	5.164078674	0.041623283	0.087919879	AC022679.1
ENSG00000140043	66.71491296	39.14574065	0.76668352	0.041696788	0.088067983	PTGR2
ENSG00000102471	629.3756082	958.8492198	-0.607703886	0.041714214	0.088097625	NDFIP2
ENSG00000187066	2.403879708	9.243956249	-1.940952086	0.041743006	0.088151266	TMEM262
ENSG00000236753	107.9813101	64.4916384	0.741984816	0.041757283	0.088174251	MKLN1-AS
ENSG00000286084	21.11516414	6.286546772	1.76056485	0.041765483	0.088181587	AL096794.1
ENSG00000273151	174.9088465	88.14786613	0.989746522	0.041767546	0.088181587	AC073957.3
ENSG00000237976	20.16871313	6.594775252	1.59797452	0.041791263	0.088218674	AL391069.2
ENSG00000105135	964.8336428	1293.710916	-0.423350779	0.041791904	0.088218674	ILVBL
ENSG00000187735	1316.336926	1756.207462	-0.415975368	0.041804877	0.088238891	TCEA1
ENSG00000143369	561.7621058	1279.110609	-1.187203775	0.041817609	0.088258594	ECM1
ENSG00000237481	4.543495031	0.387220594	3.55564216	0.041822151	0.08826101	AL117350.1
ENSG00000061337	874.5194518	216.2129385	2.015558568	0.04183794	0.08828716	LZTS1
ENSG00000203497	36.09372505	18.34267693	0.976989003	0.041856335	0.088318805	PDCD4-AS1
novel.1083	15.043751	5.013927024	1.585329682	0.041865659	0.088320676	-
ENSG00000259230	0.908699006	8.908770602	-3.299359577	0.041867371	0.088320676	LINC02323
ENSG00000140274	6.841569346	0	5.108980133	0.04186742	0.088320676	DUOXA2
ENSG00000136450	1080.381219	1535.429873	-0.507322718	0.041932741	0.08845129	SRSF1
ENSG00000231172	0.307102255	4.391737868	-3.749814552	0.041943748	0.088467326	AC007099.1
ENSG00000179918	737.1246199	1102.736654	-0.58139437	0.041950284	0.088473928	SEPHS2
ENSG00000102977	334.6498854	485.2822165	-0.536319363	0.0419553	0.088474697	ACD
ENSG00000125618	66.93121016	188.044533	-1.490764327	0.041957459	0.088474697	PAX8
ENSG00000197417	62.19057866	98.94930946	-0.672211358	0.041977013	0.088508747	SHPK
ENSG00000135821	12138.06629	6582.173289	0.882905165	0.042010791	0.088571955	GLUL
ENSG00000277873	9.465906652	1.722487775	2.427992242	0.042016604	0.088571955	AC131159.2
ENSG00000137200	1067.614345	1449.646235	-0.441098613	0.042017218	0.088571955	CMTR1
ENSG00000100726	874.4260715	1294.485148	-0.565953288	0.042034519	0.088601237	TELO2
ENSG00000153904	925.4933955	1889.19769	-1.02963924	0.042082598	0.088693894	DDAH1
ENSG00000165943	13.88088807	4.596024848	1.585527841	0.042085306	0.088693894	MOAP1
ENSG00000237529	6.951135685	0.774441189	3.230357576	0.042104473	0.088727091	AL137847.2
ENSG00000270300	9.051745355	1.456837632	2.645464277	0.042110232	0.08873203	PHACTR2P1
ENSG00000151338	101.3601987	164.0963871	-0.696393997	0.042118339	0.088741916	MIPOL1
ENSG00000286689	11.9337228	2.120320855	2.486280098	0.042141221	0.088782929	AC090946.1
ENSG00000168096	633.9363418	446.6625919	0.505338988	0.042152846	0.088797887	ANKS3
ENSG00000251359	30.41265388	9.720781443	1.657718241	0.042155157	0.088797887	WWC2-AS2
ENSG00000234840	32.26311748	6.052734086	2.427517971	0.042192364	0.088869056	LINC01239
ENSG00000226669	7.197777715	1.049547942	2.757904209	0.042210831	0.088900746	AL354977.1
ENSG00000166532	943.4112339	603.8302546	0.643237822	0.042227139	0.088927883	RIMKLB
ENSG00000181652	7.459534802	32.20820253	-2.104376864	0.042232945	0.088928652	ATG9B
ENSG00000251637	4.578739738	0	4.527815242	0.04223435	0.088928652	AP003716.1
ENSG00000188895	2781.368948	2046.464699	0.442774086	0.04223921	0.088931678	MSL1
ENSG00000280047	44.19386226	15.942673	1.46239154	0.042244957	0.088936571	AC091825.1

ENSG00000143753	2127.481896	3017.287687	-0.504227543	0.042254767	0.088950015	DEGS1
ENSG00000197961	493.4459282	328.3169476	0.58792448	0.042308918	0.089056792	ZNF121
ENSG00000110697	954.629118	1488.697633	-0.641049082	0.042323337	0.089079926	PITPNM1
ENSG00000166847	1055.725239	1410.370636	-0.417963409	0.042418413	0.089272805	DCTN5
ENSG00000155816	530.6917876	321.5222393	0.723879771	0.04242398	0.08927729	FMN2
ENSG00000157077	358.3010613	529.8023313	-0.563844288	0.042437871	0.089299289	ZFYVE9
ENSG00000114126	1048.745443	748.4742318	0.487074417	0.042442484	0.089301764	TFDP2
ENSG00000187672	18.12605639	5.186248153	1.819977421	0.042470827	0.089354163	ERC2
ENSG00000131730	50.76442861	16.51637605	1.619188969	0.042486524	0.089375178	CKMT2
ENSG00000223573	13.20622334	4.01512976	1.7303908	0.042487696	0.089375178	TINCR
ENSG00000115234	2038.931421	2943.051509	-0.529595795	0.042541995	0.089479335	SNX17
ENSG00000134153	1365.484798	1919.174368	-0.491197167	0.042544375	0.089479335	EMC7
ENSG00000124209	1151.834157	884.9297802	0.380142359	0.042547543	0.089479335	RAB22A
ENSG00000227999	10.15790901	2.191140629	2.22731205	0.042565195	0.08950912	MTND5P1
ENSG00000276966	14.63238173	4.637318915	1.652799049	0.042569348	0.08950912	HIST1H4E
ENSG00000102539	5.072023799	0.356539013	3.716987359	0.042572041	0.08950912	MLNR
ENSG00000283511	11.14469059	1.548882377	2.8915058	0.042586867	0.089533047	AC137936.2
ENSG00000270194	16.31241932	41.17042074	-1.335461989	0.042596723	0.089546521	AC097359.2
ENSG00000164603	189.4768489	288.0125333	-0.603735954	0.042627724	0.089604441	BMT2
ENSG00000248773	4.295599352	0	4.435025786	0.042674398	0.089695294	AC108727.1
ENSG00000214100	4.291838406	0	4.433867494	0.042727882	0.089800445	PLAC9P1
ENSG00000267648	27.40961345	11.65945295	1.224849444	0.04276244	0.089865806	AC060766.5
ENSG00000234129	3.063429386	11.05733289	-1.860611707	0.042795962	0.089928978	AC073529.1
ENSG00000169248	87.02896351	17.6201423	2.304197541	0.042844256	0.09002318	CXCL11
ENSG00000262468	39.7537366	20.59762087	0.94347595	0.042867135	0.090056921	LINC01569
ENSG00000153404	24.92020019	4.148596966	2.574755924	0.042867506	0.090056921	PLEKHG4B
ENSG00000083093	177.1393935	249.1037818	-0.492220226	0.042870968	0.090056921	PALB2
ENSG00000197380	343.5471458	164.1665521	1.066727412	0.042875889	0.090056921	DACT3
ENSG00000257017	6.217264375	0.356539013	4.010687102	0.042877646	0.090056921	HP
ENSG00000177663	1132.722817	721.9404389	0.650298139	0.042901327	0.090099377	IL17RA
ENSG00000274520	4.284245242	0	4.43151012	0.042914089	0.09011161	RF02246
ENSG00000280200	4.284245242	0	4.43151012	0.042914089	0.09011161	AC073862.5
novel.269	4.54474868	0.387220594	3.555980383	0.042942486	0.09016128	-
ENSG00000164902	651.6662987	963.4841329	-0.564478084	0.042944684	0.09016128	PHAX
ENSG00000254978	3.04831433	11.64768459	-1.941972788	0.042990831	0.090250872	ALG1L9P
ENSG00000143320	269.426047	75.38050298	1.836674861	0.043008196	0.090280031	CRABP2
ENSG00000110060	125.7343903	186.9119302	-0.572883422	0.043052609	0.090365958	PUS3
ENSG00000258591	4.558610087	0.356539013	3.559728169	0.043060964	0.090376194	PPIAP4
ENSG00000200502	4.529633624	0.387220594	3.551894542	0.043067761	0.090383158	RF00019
ENSG00000185800	743.3262313	1094.694202	-0.558218383	0.043092092	0.090426916	DMWD
ENSG00000225648	619.7581668	366.6568889	0.756575963	0.04310516	0.090447033	SBDSP1
ENSG00000227063	491.1772688	858.5436486	-0.805940337	0.043121994	0.090475049	RPL41P1
ENSG00000179862	110.8958827	223.9555272	-1.012700941	0.043159465	0.090546356	CITED4
ENSG00000280129	7.282199808	1.467450118	2.329233304	0.043182178	0.09058669	AL132780.5
novel.417	61.30688171	113.1173654	-0.883007059	0.04320674	0.09062834	-
ENSG00000145725	1002.959968	691.8007097	0.535398239	0.043209009	0.09062834	PPIP5K2
ENSG00000243406	32.42841418	12.19661383	1.411340036	0.043233163	0.090666983	MRPS31P5
ENSG00000124535	1042.543135	1378.664033	-0.403052981	0.043234412	0.090666983	WRNIP1
ENSG00000229692	34.60237657	15.13254155	1.203302704	0.043238803	0.090668873	SOS1-IT1
ENSG00000258334	0.307102255	4.361056287	-3.741372872	0.043245143	0.090674849	AC125611.4
ENSG00000140939	337.6694364	548.8488058	-0.700256681	0.043255602	0.090689461	AC074143.1

ENSG00000108423	136.4850492	87.87190747	0.632918861	0.043298527	0.090772132	TUBD1
ENSG00000276542	6.827707938	0	5.106146377	0.043337392	0.090846278	AC097478.2
ENSG00000287037	0	5.919395274	-5.144551325	0.043347934	0.090861046	AC097709.1
ENSG00000237172	295.6995952	627.1111119	-1.084824592	0.043356651	0.090871986	B3GNT9
ENSG00000162877	14.59964432	2.882993683	2.340962867	0.043368519	0.09088953	PM20D1
ENSG00000117448	1284.222183	1751.987802	-0.44800386	0.043377486	0.090899588	AKR1A1
ENSG00000260641	12.65881856	29.04475508	-1.199511029	0.043380316	0.090899588	AC114811.2
ENSG00000270972	4.529633624	0.336469917	3.551891043	0.043460827	0.091060946	AC136475.9
ENSG00000256540	4.240153722	0	4.417799672	0.043474339	0.09107457	AC007406.3
ENSG00000222585	4.240153722	0	4.417799672	0.043474339	0.09107457	RNA5SP494
ENSG00000224468	65.10931171	19.55521779	1.734557845	0.043505534	0.091132571	LAMC1-AS1
ENSG00000232653	6.76502515	0	5.09101147	0.043561036	0.091241477	GOLGA8N
novel.642	25.16293909	67.70416708	-1.425390719	0.043584998	0.091284308	-
ENSG00000136273	249.733483	330.2163329	-0.402797554	0.043601541	0.091307122	HUS1
ENSG00000234286	4.231378181	0	4.415069073	0.04360292	0.091307122	AC006026.3
ENSG00000274943	4.25401513	13.74780796	-1.692109199	0.043619849	0.091331911	AC079684.1
ENSG00000235641	17.06996777	3.392940604	2.352180132	0.043621788	0.091331911	LINC00484
ENSG00000103479	1554.573474	1036.032565	0.585745847	0.043645598	0.091368503	RBL2
ENSG00000174680	30.34814798	5.003186144	2.617692318	0.043646299	0.091368503	GRIK1-AS1
ENSG00000022976	144.0410444	211.9685599	-0.557490167	0.043737502	0.091552049	ZNF839
ENSG00000127074	3.649911081	0	4.200868891	0.043742837	0.09155584	RGS13
ENSG00000287129	8.211028466	1.803920035	2.189668029	0.043761767	0.091588084	AC097500.1
ENSG00000287817	23.01580187	10.03846653	1.196534067	0.043791759	0.091643471	AL096701.4
ENSG00000170854	322.6505727	518.4535726	-0.684902202	0.043830746	0.091716711	RIOX2
ENSG00000103245	594.9642859	415.9442203	0.516294157	0.043833817	0.091716711	CIAO3
ENSG00000120370	204.6762669	308.4326959	-0.590718599	0.04384712	0.091731773	GORAB
ENSG00000260409	6.64535835	0	5.066195186	0.043848077	0.091731773	AC012414.5
ENSG00000224809	3.97463569	0	4.323244096	0.04386346	0.091756566	BEND3P2
ENSG00000095002	462.7835952	694.6626883	-0.586376433	0.043869543	0.091761904	MSH2
ENSG00000169379	397.158707	286.4739722	0.470805447	0.04392648	0.091868333	ARL13B
ENSG00000286339	29.17972882	2.954969332	3.310536541	0.043928661	0.091868333	Z99496.2
ENSG00000258088	0	3.586615098	-4.424074729	0.043936018	0.091868333	AC078820.1
ENSG00000196747	0	3.586615098	-4.424074729	0.043936018	0.091868333	HIST1H2AI
ENSG00000283599	3.636049674	0	4.195974914	0.043941641	0.091868333	BX276092.9
ENSG00000180745	3.636049674	0	4.195974914	0.043941641	0.091868333	CLRN3
ENSG00000286446	3.968367446	0	4.321184347	0.043959805	0.091898913	AL353688.1
ENSG00000213025	2.684512796	13.9238535	-2.372407333	0.043970072	0.09191298	COX20P1
ENSG00000111642	892.7805205	615.4024157	0.536918084	0.04398229	0.091931123	CHD4
ENSG00000170855	459.5421467	765.3092645	-0.736240204	0.043992446	0.091944956	TRIAP1
ENSG00000257773	7.284707106	17.59891732	-1.273811616	0.044051292	0.092060538	ST13P3
ENSG00000112183	19.46653904	53.0489643	-1.444997631	0.044063239	0.092078099	RBM24
ENSG00000157021	29.58551367	54.04202937	-0.865862631	0.044068145	0.092080946	FAM92A1P1
ENSG00000233639	3.960774282	0	4.318664541	0.044077174	0.092085001	PANTR1
ENSG00000228718	3.960774282	0	4.318664541	0.044077174	0.092085001	LINC02521
ENSG00000285079	4.735945081	0	4.579581798	0.044083541	0.09208694	AL513493.1
ENSG00000185885	1899.546499	5427.281284	-1.514602437	0.044085191	0.09208694	IFITM1
ENSG00000265786	4.734691432	0	4.579227577	0.044100713	0.092111957	LINC01906
ENSG00000166819	13.77633633	3.944309986	1.806237243	0.044140051	0.092186709	PLIN1
ENSG00000139117	261.547523	135.3784698	0.94944716	0.044145083	0.092189807	CPNE8
ENSG00000180376	332.6342395	231.5392233	0.522305961	0.044149742	0.092192128	CCDC66
ENSG00000260032	8271.931165	6074.614473	0.445431588	0.044161313	0.092208879	NORAD

ENSG00000230177	72.24386465	43.95422482	0.715615395	0.044182633	0.092217211	AL080317.1
ENSG00000110148	3.620934617	0	4.190626865	0.044185222	0.092217211	CCKBR
ENSG00000255398	3.620934617	0	4.190626865	0.044185222	0.092217211	HCAR3
ENSG00000236958	3.620934617	0	4.190626865	0.044185222	0.092217211	AC051618.1
ENSG00000155719	3.95325239	0	4.316186335	0.044193699	0.092217211	OTOA
ENSG00000269489	3.95325239	0	4.316186335	0.044193699	0.092217211	AL589765.6
ENSG00000277214	3.95325239	0	4.316186335	0.044193699	0.092217211	AC009159.2
ENSG00000275197	3.95325239	0	4.316186335	0.044193699	0.092217211	AC092794.2
ENSG00000172367	3.619680969	0	4.190184895	0.044205504	0.092234436	PDZD3
ENSG00000105854	3398.573981	2064.753165	0.718834681	0.044229026	0.092273402	PON2
ENSG00000268942	4.261608293	14.727692	-1.791283675	0.044231282	0.092273402	CKS1BP3
ENSG00000141646	1519.543018	1095.103384	0.47279705	0.044242253	0.092288878	SMAD4
ENSG00000126947	1055.229979	727.5994151	0.53664625	0.044280196	0.092360611	ARMCX1
ENSG00000233382	32.9421839	16.12085472	1.030378308	0.044313783	0.092423247	NKAPP1
ENSG00000064703	308.377093	428.9961465	-0.476558375	0.044323398	0.092435881	DDX20
ENSG00000162585	858.178072	1225.896104	-0.514191245	0.044327758	0.092437553	FAAP20
ENSG00000272849	40.20794373	21.45105417	0.912632981	0.044366311	0.092510524	AC084018.1
ENSG00000198315	790.1323926	527.2853332	0.583909308	0.044395818	0.092564622	ZKSCAN8
ENSG00000265185	6.678024489	0	5.073001588	0.044460017	0.092691036	SNORD3B-1
ENSG00000264769	3.960774282	13.78679027	-1.800273599	0.04450025	0.092767472	AC145207.8
ENSG00000212664	39.14580033	67.67083542	-0.790813625	0.044543863	0.092850939	AC064799.1
ENSG00000157782	15.7687755	80.6857874	-2.354503991	0.04458979	0.092939216	CABP1
ENSG00000138663	517.465419	822.1865249	-0.668317568	0.044613791	0.092981782	COPS4
ENSG00000129910	215.1502831	84.98321595	1.342194175	0.044659892	0.093067512	CDH15
ENSG00000273311	19.77092054	42.60834516	-1.105144547	0.044662089	0.093067512	DGCR11
ENSG00000196141	1089.234843	1843.965135	-0.759687265	0.044672432	0.0930816	SPATS2L
ENSG00000143365	5.826024761	0.336469917	3.91290089	0.044708119	0.093148488	RORC
ENSG00000168310	711.6814475	476.6137487	0.578923912	0.044721341	0.093168563	IRF2
ENSG00000175664	6.776307989	0	5.093340106	0.044735625	0.093190849	TEX26
ENSG00000072952	297.4016283	102.2179011	1.539492903	0.044762473	0.093239301	MRVI1
ENSG00000275393	2.460578986	11.29101719	-2.203582708	0.044775745	0.09325947	AC018695.6
ENSG00000218305	5.783258161	17.30374147	-1.582560134	0.04478119	0.093263335	CDC14C
ENSG00000112232	4.290584757	0	4.433518018	0.044806989	0.093309585	KHDRBS2
ENSG00000198561	4935.555821	3753.33201	0.395092743	0.044818091	0.093325224	CTNND1
novel.614	101.8957508	35.23005729	1.529269884	0.044843722	0.093371113	-
ENSG00000235308	11.98032161	2.261960402	2.433411871	0.044857619	0.093392564	AL445991.1
ENSG00000225376	8.901777171	0.672939833	3.681042432	0.044878295	0.093428123	TMEM246-AS1
ENSG00000124201	1453.629615	1840.792283	-0.340656446	0.044884836	0.093432065	ZNFX1
ENSG00000142937	16055.54342	21696.65376	-0.434412782	0.044887381	0.093432065	RPS8
ENSG00000241560	11.43291684	2.587817834	2.161907069	0.044905974	0.093463279	ZBTB20-AS1
novel.597	25.24958374	6.297287652	1.99545251	0.044923334	0.09349192	-
ENSG00000278318	89.93269552	168.1205411	-0.9020232	0.044947456	0.09353463	ZNF229
ENSG00000261534	134.0125467	201.3357133	-0.585854785	0.044964483	0.093562569	AL596244.1
ENSG00000163930	1552.572515	2063.173213	-0.41024897	0.044975554	0.093575592	BAP1
ENSG00000164080	710.1863571	404.5845536	0.811967454	0.044977945	0.093575592	RAD54L2
ENSG00000182185	53.67753381	91.45150611	-0.771081392	0.04501741	0.093650198	RAD51B
ENSG00000180917	60.1112101	29.0979459	1.040789086	0.045064055	0.093739729	CMTR2
ENSG00000213225	12.87676887	35.66892765	-1.473719231	0.045084767	0.093775305	NOC2LP1
novel.866	61.80255871	20.32020236	1.603120321	0.045098582	0.09379653	-
ENSG00000087157	559.2446043	816.7447474	-0.546014154	0.045119538	0.0938263	PGS1
ENSG00000153922	995.7257787	694.1692957	0.520322598	0.045120118	0.0938263	CHD1

ENSG00000118689	967.4486629	1272.518166	-0.395430318	0.045139331	0.093856253	FOXO3
ENSG00000161013	1.803536605	7.988965452	-2.138534314	0.045141747	0.093856253	MGAT4B
ENSG00000127083	681.8566932	38.19106278	4.157541993	0.045205759	0.093981822	OMD
novel.199	11.02627744	1.844058227	2.59733174	0.045227179	0.094018829	-
ENSG00000212993	7.194016769	0.774441189	3.278158657	0.045234334	0.094025139	POU5F1B
ENSG00000205106	5.527769318	0.336469917	3.836720657	0.045237452	0.094025139	AC044839.1
ENSG00000214855	8.416086274	1.069617038	2.97117333	0.045241613	0.094026266	APOC1P1
ENSG00000186867	6.444061488	22.2280639	-1.792948119	0.045263531	0.094064295	QRFPR
ENSG00000262406	0	3.963223207	-4.567527059	0.045302551	0.094136612	MMP12
ENSG00000179921	13.6871131	3.11911812	2.114530312	0.045308156	0.094136612	GPBAR1
novel.105	21.07490484	7.071600447	1.592051721	0.0453092	0.094136612	-
ENSG00000255062	31.2027972	13.77977381	1.172569521	0.045334207	0.094181036	AP001318.2
ENSG00000087448	867.5443424	642.982918	0.432251834	0.045343886	0.094193611	KLHL42
ENSG00000013297	1582.030483	3622.121004	-1.195105658	0.045362375	0.094224486	CLDN11
ENSG00000116014	0.307102255	6.755199626	-4.371333859	0.045388892	0.094272029	KISS1R
ENSG00000273132	0	3.506338714	-4.395023611	0.045403926	0.094289066	AL355312.4
ENSG00000250427	7.706390295	0	5.279034216	0.045404354	0.094289066	LINC02148
ENSG00000104447	362.2380868	241.5971982	0.584784872	0.045454571	0.094385806	TRPS1
ENSG00000067798	1258.237152	757.3675436	0.732583978	0.045486414	0.094444381	NAV3
ENSG00000006607	496.7130759	768.8693628	-0.630362674	0.045492895	0.094450289	FARP2
ENSG00000261461	13.60150863	30.84379483	-1.178372694	0.045528127	0.094515882	UBE2MP1
ENSG00000233435	4.810266713	0.387220594	3.63933058	0.045538027	0.094528881	AGGF1P2
ENSG00000124493	0.307102255	4.433031935	-3.76144944	0.045545668	0.094537189	GRM4
ENSG00000240087	41.9418173	95.44973043	-1.184846065	0.04556725	0.09457443	RPSAP12
ENSG00000198342	51.49898375	23.338975	1.137038753	0.045572839	0.094578475	ZNF442
ENSG00000111271	445.8597078	665.1872231	-0.576702284	0.04559894	0.094625085	ACAD10
ENSG00000242852	11.76272732	3.271370153	1.855455585	0.04560797	0.094636264	ZNF709
ENSG00000266501	2.709728314	11.17060261	-2.039355368	0.045616098	0.094645572	AC025198.1
ENSG00000140948	760.7032304	517.3592033	0.556508142	0.045639165	0.094685869	ZCCHC14
ENSG00000255142	12.00428348	28.45183484	-1.23828477	0.045654944	0.094711043	AP006621.2
ENSG00000152049	357.6244688	106.8312982	1.742438013	0.045705427	0.0948082	KCNE4
ENSG00000258045	8.179544705	1.854670712	2.1667565	0.045752236	0.094897721	RF00017
ENSG00000185737	6.564697203	0.723690511	3.191365427	0.04579637	0.094981679	NRG3
ENSG00000267282	0.616711807	6.154363836	-3.332124401	0.045815855	0.095014507	AC011481.2
ENSG00000139697	818.8950534	1133.075986	-0.468193771	0.045836905	0.095050573	SBNO1
ENSG00000136100	1076.4475	780.9220385	0.462742947	0.045841356	0.095052216	VPS36
ENSG00000116783	6.548328498	0	5.045769709	0.045853449	0.095069703	TNNI3K
ENSG00000106992	125.2074433	193.1886824	-0.625090267	0.045874572	0.09510591	AK1
ENSG00000083223	543.4800886	911.0142149	-0.744957242	0.045885555	0.095121089	TUT7
ENSG00000159873	341.1595208	480.0127251	-0.49264595	0.045891967	0.09512679	CCDC117
ENSG00000170775	4.751060137	17.59061659	-1.880874216	0.04591113	0.09515892	GPR37
ENSG00000153560	1313.554731	1710.681121	-0.381087612	0.045939074	0.095209245	UBP1
novel.746	9.109698282	28.28683415	-1.638734591	0.045994417	0.095308878	-
ENSG00000177082	87.5380899	134.5911044	-0.620915096	0.045994485	0.095308878	WDR73
ENSG00000231989	2.723589721	10.05853563	-1.884265956	0.046009592	0.095332579	PPP1R2B
ENSG00000255423	35.99522809	15.96847429	1.178046077	0.046021421	0.095349484	EBLN2
ENSG00000183837	16.16789301	5.482579877	1.568818889	0.046048267	0.095397499	PNMA3
ENSG00000273375	7.463295748	0.774441189	3.329710835	0.046053459	0.095400648	AC055764.2
ENSG00000260823	0.616711807	10.50467924	-4.09793119	0.046061138	0.095408948	AC026461.3
ENSG00000125347	806.2419607	485.8012092	0.731471083	0.0460754	0.095430882	IRF1
ENSG00000285257	13.23645345	4.239485835	1.633954669	0.046105601	0.095485821	AL157893.1

ENSG00000135443	6.461683842	0	5.024750867	0.046115687	0.095499096	KRT85
ENSG00000184434	4.530887272	0.356539013	3.552207856	0.046159048	0.095581272	LRRC19
ENSG00000261114	23.85136162	11.13876515	1.099962922	0.04616521	0.095586414	AC012181.1
ENSG00000240602	1.7871679	10.81303612	-2.592902716	0.046179323	0.095600782	AADACP1
ENSG00000222898	0	3.912472529	-4.550725389	0.046179509	0.095600782	RN7SKP97
ENSG00000187953	165.9268633	259.2934729	-0.644258956	0.046198919	0.095633345	PMS2CL
novel.13	43.94445638	0	7.791171921	0.04622416	0.095677972	-
ENSG00000144834	3.029438327	15.54928785	-2.356484792	0.046243452	0.095710278	TAGLN3
ENSG00000177051	218.5003131	296.4621548	-0.439581605	0.046262356	0.095741776	FBXO46
ENSG00000128274	293.0836052	790.960227	-1.432509547	0.046284664	0.095780315	A4GALT
ENSG00000259828	2.15473038	11.16127439	-2.385876551	0.046293884	0.095791765	AL355596.1
ENSG00000149639	1120.397756	1657.329343	-0.5648331	0.046318273	0.095834598	SOGA1
ENSG00000157150	34.33567581	115.1630265	-1.747522717	0.046337882	0.095865261	TIMP4
ENSG00000265972	11081.18653	5715.497385	0.955194386	0.046346752	0.095865261	TXNIP
ENSG00000152147	201.9000236	317.3705383	-0.653551726	0.046347172	0.095865261	GEMIN6
ENSG00000174028	125.1732666	202.7147147	-0.695483466	0.046347852	0.095865261	FAM3C2
ENSG00000258744	22.29342686	52.36456008	-1.233759753	0.046351942	0.095866088	AL132800.1
ENSG00000104964	6162.493984	4574.125679	0.430066756	0.046364701	0.095884845	TLE5
ENSG00000279086	3.050821627	11.39495869	-1.90722113	0.046388879	0.095927209	AC073130.3
ENSG00000166900	599.86684	307.0370462	0.96706712	0.046411746	0.095961518	STX3
ENSG00000186298	1807.071028	2683.59009	-0.57057107	0.046412857	0.095961518	PPP1CC
ENSG00000236782	9.602911072	2.089639274	2.181488674	0.046455837	0.096042638	AL391650.1
ENSG00000123009	3.591958154	12.39375595	-1.78390612	0.046459485	0.096042638	NME2P1
ENSG00000162688	411.293929	257.9767334	0.671899519	0.046496337	0.096111174	AGL
ENSG00000251455	14.81827881	2.802717299	2.393323013	0.046510525	0.096132852	AC092611.1
ENSG00000114023	595.6930547	1063.948348	-0.836615038	0.046526269	0.096157417	FAM162A
ENSG00000213859	1235.53144	812.9238401	0.604371409	0.046530002	0.096157417	KCTD11
ENSG00000228925	6.908297814	21.34852533	-1.626456305	0.046533513	0.096157417	AC016722.2
ENSG00000141867	1056.907081	1433.196697	-0.439187422	0.046545733	0.096175018	BRD4
ENSG00000280721	5.174068246	18.57507695	-1.840815773	0.04655362	0.096176523	LINC01943
ENSG00000266916	21.94982625	8.122432658	1.425381425	0.046554502	0.096176523	ZNF793-AS1
ENSG00000261839	4.571217846	0.356539013	3.563210907	0.046561011	0.096176523	AL358933.1
ENSG00000269313	29.07711455	13.56359007	1.110003627	0.046561268	0.096176523	MAGIX
ENSG00000237772	4.546002329	0.387220594	3.55632909	0.046565906	0.096178457	LINC02631
ENSG00000103363	2609.323845	4154.520671	-0.671063735	0.046569916	0.096179093	ELOB
ENSG00000226108	4.175932552	0	4.397596126	0.046580493	0.09619329	RAB28P1
ENSG00000140522	8.232411766	0.774441189	3.46474954	0.046595638	0.096216919	RLBP1
ENSG00000082146	377.0519212	600.6615548	-0.672289487	0.046616129	0.096251581	STRADB
ENSG00000175970	630.2692193	859.5122789	-0.447731053	0.046643833	0.096301131	UNC119B
ENSG00000138100	10.33928968	1.426156051	2.852366314	0.046677514	0.09636301	TRIM54
ENSG00000277511	52.67312987	29.31857757	0.843745658	0.046704213	0.096410469	AC116407.2
ENSG00000256826	4.571217846	0.336469917	3.563214026	0.046719678	0.096434732	ATP5MFP4
ENSG00000163932	1013.39026	683.4530441	0.568723563	0.046786818	0.096565643	PRKCD
ENSG00000131100	2034.751602	2691.698841	-0.403709814	0.046926587	0.096846426	ATP6V1E1
novel.719	52.86092133	101.0599169	-0.935559812	0.046931057	0.096847957	-
ENSG00000090432	539.1787189	792.2053289	-0.555553982	0.046963955	0.09690815	MUL1
ENSG00000170088	713.1579104	464.6237457	0.617501924	0.047082517	0.097145083	TMEM192
ENSG00000160703	302.8656486	432.6006477	-0.514496464	0.047093427	0.09714621	NLRX1
ENSG00000234917	8.393449325	1.436768536	2.548537257	0.047096133	0.09714621	AC098484.2
ENSG00000101825	411.0999927	112.7794956	1.865161921	0.04709653	0.09714621	MXRA5
ENSG00000100890	88.45488027	142.0789375	-0.683394425	0.04709802	0.09714621	KIAA0391

ENSG00000188167	16.55662533	34.36318617	-1.05204685	0.047104072	0.09715098	TMPPE
ENSG00000172239	1169.070233	1574.787615	-0.429886318	0.047132687	0.097202281	PAIP1
ENSG00000150907	1085.149021	536.4384387	1.015991154	0.047166971	0.097265265	FOXO1
ENSG00000251675	3.605819561	0	4.185221737	0.047212747	0.097351936	AC010260.1
ENSG00000155324	671.282436	389.9977904	0.782926682	0.047230669	0.097381162	GRAMD2B
ENSG00000156486	34.96653411	10.84243343	1.690048954	0.047254757	0.097423094	KCNS2
ENSG00000115425	155.5011421	252.3227787	-0.699627906	0.047262272	0.097427081	PECR
ENSG00000107521	1296.089022	956.3670007	0.438456541	0.04726419	0.097427081	HPS1
ENSG00000210144	399.4717114	187.8840618	1.089408978	0.047278259	0.09744835	MT-TY
ENSG00000134954	1885.120408	3201.274348	-0.763897785	0.047307717	0.097501331	ETS1
ENSG00000185728	1102.939496	1437.854295	-0.382492512	0.047324504	0.097528193	YTHDF3
ENSG00000286320	6.654133891	1.10029862	2.614531563	0.047343095	0.097558768	AC108053.1
ENSG00000264083	4.79139071	0.387220594	3.634344767	0.047386575	0.097640623	AC005899.1
ENSG00000100023	576.7011684	850.214841	-0.559886318	0.047413225	0.097687788	PPIL2
ENSG00000173153	634.2132258	894.1132596	-0.495457774	0.047431941	0.097718599	ESRRA
ENSG00000183840	4.204909015	13.86822253	-1.7136545	0.047447519	0.097736603	GPR39
ENSG00000164176	3099.797954	800.4489708	1.95316591	0.047451946	0.097736603	EDIL3
ENSG00000173928	60.29635172	35.1105418	0.775554848	0.047454566	0.097736603	SWSAP1
ENSG00000101654	678.0947021	1131.51754	-0.738423375	0.047455727	0.097736603	RNMT
ENSG00000279168	8.534499426	1.456837632	2.559022222	0.047463306	0.097744463	AC105052.4
ENSG00000138600	1390.097272	1011.825134	0.457946691	0.047472134	0.097754895	SPPL2A
ENSG00000217130	34.79991249	58.30415284	-0.745242475	0.04752509	0.097856187	AL139100.1
ENSG00000227473	19.39479598	35.73974742	-0.883681158	0.047530595	0.097859767	TSSK5P
ENSG00000217241	7.002820368	17.84565425	-1.353922492	0.047545326	0.097878754	CBX3P9
ENSG00000139988	9.086990062	1.762625967	2.352727578	0.047547352	0.097878754	RDH12
ENSG00000233355	4.593854795	0	4.53230792	0.047551215	0.097878952	CHRM3-AS2
ENSG00000172350	7.638408178	20.79818343	-1.445305006	0.047559435	0.097888115	ABCG4
ENSG00000023041	851.7810087	631.0593219	0.432584971	0.047579554	0.097921768	ZDHHC6
ENSG00000178343	121.919025	29.76972986	2.032096268	0.047612023	0.097980829	SHISA3
ENSG00000176715	467.5280471	631.7738941	-0.434060557	0.047624089	0.097997898	ACSF3
ENSG00000170846	403.3131739	287.1937287	0.489513887	0.047685197	0.098115871	AC093323.1
ENSG00000148019	394.8465422	617.5453735	-0.645758453	0.047700961	0.098133539	CEP78
ENSG00000168702	28.33238733	3.974991568	2.834785565	0.047704878	0.098133539	LRP1B
ENSG00000244398	1367.437318	1966.939059	-0.524501765	0.047705115	0.098133539	AC116533.1
ENSG00000211778	6.466698437	0	5.025844639	0.047832688	0.098388178	TRAV4
ENSG00000188659	23.95097004	8.35740122	1.506445059	0.047873796	0.098458364	SAXO2
ENSG00000173040	154.9216841	245.0461081	-0.660304277	0.04787439	0.098458364	EVC2
ENSG00000234880	0	5.564140531	-5.059753527	0.047881319	0.098461152	LINC00163
ENSG00000114520	660.2769585	460.059126	0.520541607	0.047883325	0.098461152	SNX4
ENSG00000172292	172.1560509	291.2044443	-0.75921031	0.047890413	0.098467935	CERS6
ENSG00000150281	271.094791	157.1353938	0.788662605	0.047925001	0.098527176	CTF1
ENSG00000267152	13.3573739	4.280779903	1.637556569	0.04792681	0.098527176	AC093227.1
ENSG00000212493	1.50778846	8.74449342	-2.530499742	0.048002962	0.09867592	SNORD19
ENSG00000151500	717.1411631	515.9460658	0.475003893	0.048014095	0.098690997	THYN1
ENSG00000231943	3.663772488	0	4.205824865	0.048127726	0.098908912	PGM5P4-AS1
ENSG00000279994	3.663772488	0	4.205824865	0.048127726	0.098908912	AC017037.4
ENSG00000095066	193.5408938	313.3178264	-0.694138322	0.048146151	0.0989306	HOOK2
ENSG00000152291	3908.897041	5655.585761	-0.532893016	0.048147913	0.0989306	TGOLN2
ENSG00000134597	368.4520179	479.5288835	-0.380029807	0.048149703	0.0989306	RBMX2
ENSG00000163683	1268.563925	855.9756913	0.567514107	0.04816237	0.0989488	SMIM14
ENSG00000072840	806.2152357	1811.79346	-1.16808066	0.048203666	0.099019116	EVC

ENSG00000186714	6.683110355	1.10029862	2.619628014	0.048204218	0.099019116	CCDC73
ENSG00000267466	4.29810665	0	4.435906271	0.048212179	0.099019812	AC021683.1
ENSG00000272866	4.29810665	0	4.435906271	0.048212179	0.099019812	AL135786.2
ENSG00000198917	15.81795288	5.63611618	1.476651228	0.048268287	0.099127212	SPOUT1
ENSG00000135577	10.2234551	2.180528143	2.238050623	0.048314548	0.099214375	NMBR
ENSG00000107164	1389.898838	1802.934818	-0.375215079	0.048320879	0.099219534	FUBP3
ENSG00000126746	280.1115047	391.7719964	-0.48388687	0.048348522	0.099268449	ZNF384
ENSG00000249568	4.289331108	0	4.433187228	0.048354546	0.099272973	AC104793.1
ENSG00000145730	1898.639914	4598.592844	-1.276233909	0.048448193	0.099457372	PAM
ENSG00000142973	41.59795943	0	7.711994204	0.048492024	0.099539485	CYP4B1
ENSG00000155463	1636.166998	2170.024439	-0.407374634	0.048539416	0.099628897	OXA1L
ENSG00000173113	2103.130769	2728.300486	-0.375423906	0.048561012	0.099665349	TRMT112
ENSG00000284642	21.18544009	5.932447905	1.827397764	0.048623404	0.099785517	AL139424.2
ENSG00000225526	15.76222252	3.586615098	2.137128626	0.048663259	0.099859422	MKRN2OS
ENSG00000134247	479.4316048	1365.072762	-1.509680098	0.04867073	0.099866864	PTGFRN
ENSG00000140092	2948.240775	1591.304136	0.889513051	0.048784932	0.100091342	FBLN5
ENSG00000125551	6.3546248	0.743759607	3.125239171	0.048787835	0.100091342	PLGLB2
ENSG00000223345	3.926783224	12.97452264	-1.721794747	0.048831153	0.100160093	HIST2H2BA
ENSG00000144290	6.778815287	0	5.093894226	0.048832912	0.100160093	SLC4A10
ENSG00000228035	6.778815287	0	5.093894226	0.048832912	0.100160093	NGF-AS1
ENSG00000167077	27.94817141	78.54184861	-1.489058417	0.048869002	0.100226204	MEI1
ENSG00000158014	3.622188266	0	4.191040922	0.048879524	0.10023987	SLC30A2
ENSG00000226525	58.25878084	88.43846566	-0.603543747	0.048888555	0.100250477	RPS7P10
novel.770	96.58474939	164.144732	-0.763286364	0.048897137	0.100260161	-
ENSG00000239494	10.85270339	2.904218654	1.910238013	0.048938486	0.100337027	RN7SL333P
ENSG00000249092	3.61842732	0	4.189705088	0.04894835	0.100344674	PPIAP77
ENSG00000236819	7.398034391	0	5.220100371	0.04894994	0.100344674	LINC01563
ENSG00000177197	4.554849141	0.387220594	3.558773134	0.048957533	0.10035232	PCNPP5
ENSG00000236255	80.52935729	43.29981303	0.900313135	0.049004412	0.100440489	AC009404.1
ENSG00000276830	13.80155184	3.862877727	1.826715085	0.049011771	0.100443722	MIR6730
ENSG00000276071	9.669710811	2.476859868	1.955633014	0.049013722	0.100443722	AC074138.1
ENSG00000213763	5.981976454	18.40763611	-1.619247141	0.049025604	0.100460148	ACTBP2
ENSG00000164736	82.88971495	35.89931989	1.202265806	0.049058965	0.100520582	SOX17
ENSG00000178409	51.52502599	89.80929471	-0.802237554	0.049105865	0.100608744	BEND3
ENSG00000131096	1.188078446	7.532080959	-2.651517403	0.049140082	0.10067091	PYY
ENSG00000184305	4.574978792	0.336469917	3.564283739	0.049148354	0.100679917	CCSER1
ENSG00000123572	44.1554977	126.4418429	-1.518594797	0.049182446	0.10074181	NRK
ENSG00000213411	3.66251884	0	4.205398112	0.049200649	0.100771153	RBM22P2
ENSG00000255529	711.44824	524.8391337	0.438429582	0.049218863	0.10080051	POLR2M
ENSG00000121741	1867.879078	1163.991663	0.682576285	0.049271066	0.100899469	ZMYM2
ENSG00000260360	10.25236029	2.51699806	2.028125892	0.049324736	0.101001416	AL353708.1
ENSG00000100403	2637.555282	3457.925897	-0.390738741	0.049344115	0.101033135	ZC3H7B
ENSG00000165509	7.741421539	1.029478846	2.879628396	0.049365052	0.101061481	MAGEC3
ENSG00000280310	5.218159766	0.387220594	3.753506407	0.049370034	0.101061481	AC092437.1
ENSG00000201134	5.218159766	0.387220594	3.753506407	0.049370034	0.101061481	RF00019
ENSG00000266278	0.601596751	7.794135082	-3.68768498	0.049373518	0.101061481	LINC01910
ENSG00000118260	912.3056529	682.0295031	0.419743629	0.049402	0.101111814	CREB1
ENSG00000177917	537.5662647	749.2975099	-0.479266576	0.04950769	0.101320149	ARL6IP6
ENSG00000231793	11.49337707	3.61729668	1.673197294	0.049527204	0.101345751	DOC2GP
ENSG00000138459	911.4710613	634.8304585	0.521303542	0.049528001	0.101345751	SLC35A5
ENSG00000186369	0	5.503933243	-5.044825196	0.049540737	0.101359343	LINC00643

ENSG00000021461	4.50065716	0.387220594	3.543868721	0.049542446	0.101359343	CYP3A43
ENSG00000237522	11.16990611	3.627909165	1.631034321	0.04955628	0.101379662	NONOP2
ENSG00000253771	10.97369511	2.782648203	1.958036871	0.049563986	0.101387444	TPTE2P1
ENSG00000260729	20.75777339	3.097764754	2.768960505	0.049617219	0.101488345	AC009690.1
ENSG00000127481	2527.091359	4128.440854	-0.708046214	0.049629442	0.101505355	UBR4
ENSG00000114125	919.6934996	1277.583932	-0.474191785	0.049662875	0.101555492	RNF7
ENSG00000180347	3.637303322	0	4.196427929	0.049665682	0.101555492	ITPRID1
ENSG00000263846	3.637303322	0	4.196427929	0.049665682	0.101555492	CIAPIN1P
ENSG00000171960	213.0964701	302.9809389	-0.508600592	0.049737065	0.101690626	PPIH
ENSG00000253389	3.632288727	0	4.194648991	0.049739597	0.101690626	AC113133.1
ENSG00000257283	3.368024343	13.59684019	-2.023330422	0.049747294	0.101698358	AC012464.1
ENSG00000257242	6.379769046	1.080229524	2.564175138	0.049782046	0.101761396	LINC01619
ENSG00000239900	58.18159591	104.4876487	-0.845891631	0.049823309	0.101837731	ADSL
ENSG00000169271	0	33.9907692	-7.667895617	0.04984298	0.101869924	HSPB3
ENSG00000244733	39.01610434	16.5882233	1.242064924	0.049864107	0.101905087	AL132656.2
ENSG00000142627	1981.041124	1285.219033	0.624114295	0.049899513	0.101969424	EPHA2
ENSG00000224857	4.442704233	0	4.487405802	0.049928905	0.102021462	LINC01691
ENSG00000279803	8.617383136	1.130980201	2.964999024	0.049944133	0.102044552	AC009090.5
ENSG00000271795	11.66444382	2.822786395	2.03870726	0.04995633	0.102061446	AC011337.1
ENSG00000277050	24.4791428	8.314822883	1.562146408	0.050003959	0.102150625	AL122125.1
ENSG00000163565	2326.922933	3181.837835	-0.451410098	0.050007844	0.102150625	IFI16
ENSG00000165264	726.0472005	1025.91943	-0.498965268	0.050034217	0.102196461	NDUFB6
ENSG00000176920	16.0154888	39.14890431	-1.285187161	0.050068721	0.102257955	FUT2
ENSG00000197822	9.224350487	0.693008929	3.709194807	0.050072195	0.102257955	OCLN
ENSG00000115616	3.975889338	0	4.32375509	0.050096563	0.102291637	SLC9A2
ENSG00000163534	3.975889338	0	4.32375509	0.050096563	0.102291637	FCRL1
ENSG00000177410	423.0916872	590.8042587	-0.481848728	0.050108276	0.10230701	ZFAS1
ENSG00000186377	103.7108821	16.37131606	2.659508863	0.050111967	0.10230701	CYP4X1
ENSG00000160908	453.1033017	329.3452706	0.460286884	0.050123603	0.102322726	ZNF394
ENSG00000281969	14.69757182	3.280826763	2.171516675	0.050140726	0.102349639	AL592158.1
ENSG00000167323	1177.407098	758.9596719	0.633362057	0.050145855	0.102352066	STIM1
ENSG00000272941	14.82991765	5.166179057	1.537678015	0.050163616	0.102372471	AC083862.2
ENSG00000160271	288.5594033	151.3706436	0.932377026	0.05016376	0.102372471	RALGDS
ENSG00000275191	0.61420451	6.258305336	-3.36528205	0.050168036	0.102372471	AC007497.1
ENSG00000285790	8.015999848	0	5.335933311	0.050171613	0.102372471	AC021517.2
ENSG00000111144	866.7734982	1336.01361	-0.624255054	0.050179134	0.102379777	LTA4H
ENSG00000255858	3.60707321	0	4.185626872	0.050213824	0.102442509	AC055720.1
ENSG00000261037	3.967113797	0	4.320849094	0.050253176	0.102498263	AC010266.2
ENSG00000254338	3.604565913	0	4.184732488	0.05026131	0.102498263	MAFA-AS1
ENSG00000169194	3.604565913	0	4.184732488	0.05026131	0.102498263	IL13
ENSG00000164270	3.604565913	0	4.184732488	0.05026131	0.102498263	HTR4
ENSG00000222872	3.604565913	0	4.184732488	0.05026131	0.102498263	RNU4-78P
ENSG00000121903	73.30607933	116.7510539	-0.67008824	0.050264823	0.102498263	ZSCAN20
ENSG00000006576	401.9327163	593.975026	-0.564049291	0.050280075	0.102521318	PHTF2
ENSG00000235065	16.6397225	35.4136332	-1.085753668	0.050308269	0.102570755	RPL24P2
ENSG00000145414	133.8313082	208.28335	-0.638857791	0.05033218	0.102601248	NAF1
ENSG00000278668	5.450940389	0.723690511	2.921339105	0.050333822	0.102601248	AC005899.7
ENSG00000176732	25.31129762	10.90508086	1.206313439	0.050335072	0.102601248	PFN4
ENSG00000277775	0	3.271370153	-4.288653414	0.050341638	0.102606582	HIST1H3F
ENSG00000170122	9.79063126	1.834601616	2.428816448	0.050358479	0.102632855	FOXD4
ENSG00000189283	56.51946541	30.11527121	0.911237215	0.050381839	0.10267241	FHIT

ENSG00000136110	7.381736958	0.356539013	4.254789599	0.050396241	0.102693705	CNMD
ENSG00000227188	5.111100724	0.356539013	3.726619602	0.050422721	0.102739605	MGAT3-AS1
ENSG00000224416	4.164578442	0	4.3939043	0.050453109	0.102793462	IFNA22P
ENSG00000107960	280.7139122	473.1277483	-0.753667237	0.050464708	0.102804044	STN1
ENSG00000104946	1248.692334	746.2023358	0.743207504	0.050466217	0.102804044	TBC1D17
ENSG00000062598	1203.489864	877.5360939	0.455597095	0.050485619	0.102835505	ELMO2
ENSG00000205209	11.09558448	2.435565801	2.170197032	0.050494762	0.102841865	SCGB2B2
ENSG00000222267	4.162071144	0	4.393108626	0.050496658	0.102841865	RNU6-892P
ENSG00000129636	897.6409871	1245.561217	-0.472752395	0.050517846	0.102876952	ITFG1
ENSG00000181031	39.76989149	10.6901814	1.893722463	0.050534531	0.102902864	RPH3AL
ENSG00000113141	1780.201557	2272.067437	-0.352053188	0.050544353	0.102914799	IK
ENSG00000277459	8.311819264	31.46012339	-1.92410021	0.050568893	0.102956698	AP001527.2
ENSG00000072756	321.1292514	432.9429779	-0.431492792	0.050575299	0.10296167	TRNT1
ENSG00000276718	10.82881279	3.240688571	1.744517996	0.050613929	0.103032242	AC005840.5
ENSG00000075856	805.62525	1067.343942	-0.405959722	0.050643347	0.103084048	SART3
ENSG00000254088	5.701343365	0.743759607	2.972711072	0.050667856	0.103125856	SLC2A3P4
ENSG00000198331	91.68679818	162.2938798	-0.825076374	0.05067528	0.103132889	HYLS1
ENSG00000235351	11.98916843	3.200550379	1.902174489	0.050696069	0.103167116	AC114730.3
ENSG00000213204	20.4027474	7.85793839	1.37618745	0.050724287	0.103216455	AL049697.1
ENSG00000158715	50.67212827	108.7946966	-1.102360974	0.050736706	0.103233641	SLC45A3
ENSG00000268201	3.608326859	12.71845751	-1.817756804	0.050868088	0.103492857	AC020915.2
ENSG00000249494	7.622039473	1.386017859	2.43022205	0.050930096	0.103610901	AC008629.1
ENSG00000221983	5828.760968	8268.270777	-0.504401316	0.050965499	0.103674807	UBA52
ENSG00000134470	95.93002832	46.8773567	1.031958109	0.050985573	0.103707521	IL15RA
ENSG00000144821	4.189793959	13.65576321	-1.698422163	0.050994665	0.103717896	MYH15
ENSG00000100490	44.916522	22.99073673	0.960338228	0.051003199	0.103727131	CDKL1
ENSG00000253141	0.308355904	6.367979032	-4.286180634	0.05102758	0.103768594	AC008632.1
ENSG00000079257	196.5462542	511.3851955	-1.379935901	0.051032997	0.103771488	LXN
ENSG00000134014	497.002982	644.1780867	-0.374461432	0.051050788	0.103794004	ELP3
ENSG00000176222	65.33313081	98.12612538	-0.585246617	0.05105206	0.103794004	ZNF404
ENSG00000198034	12168.8182	18823.06483	-0.629313017	0.051064875	0.103811933	RPS4X
ENSG00000136630	114.311973	63.44350312	0.846318014	0.051079515	0.103829045	HLX
ENSG00000267288	22.68946761	7.887464097	1.525911554	0.051081285	0.103829045	AC138150.2
ENSG00000167985	203.4542683	290.7521024	-0.515633492	0.051106805	0.103872793	SDHAF2
ENSG00000256424	3.916682763	0	4.303974265	0.051118199	0.103887823	LINC02414
ENSG00000175931	643.7564481	959.83613	-0.57588357	0.051138126	0.103920191	UBE2O
ENSG00000262480	0	3.53586442	-4.405748032	0.051179496	0.103996127	SAMD11P1
ENSG00000046647	485.5403065	274.5033714	0.823682265	0.051257938	0.104147374	GEMIN8
ENSG00000270179	23.96852112	10.41391877	1.202158861	0.051296182	0.10421693	AP002840.2
ENSG00000133028	578.032006	860.8487276	-0.574928808	0.05131422	0.104245426	SCO1
ENSG00000149196	573.5992444	790.3235601	-0.462568032	0.051325828	0.104260855	HIKESHI
ENSG00000211660	6.154581587	0	4.954473422	0.051345107	0.104291864	IGLV2-23
ENSG00000175220	1488.544603	2145.819971	-0.527687866	0.05136662	0.104327405	ARHGAP1
ENSG00000117242	61.30172457	105.840626	-0.788848709	0.051439716	0.104467699	PINK1-AS
ENSG00000285836	9.538689901	1.447381022	2.727964768	0.051446255	0.104472811	AC092896.2
ENSG00000147432	41.2707988	0.774441189	5.757704059	0.051458287	0.104489079	CHRNB3
ENSG00000233968	4.917325754	0	4.630358757	0.051462565	0.104489599	AL157895.1
ENSG00000260484	3.931797819	0	4.309043852	0.051469961	0.104496449	AC131902.1
ENSG00000185621	146.2007181	225.0904883	-0.621837457	0.051488782	0.104526492	LMLN
ENSG00000179899	80.2216143	44.75819172	0.842193917	0.0514987	0.104538457	PHC1P1
ENSG00000183908	6.153327938	0	4.954192721	0.051525786	0.104577099	LRRC55

ENSG00000227947	6.153327938	0	4.954192721	0.051525786	0.104577099	LINC01738
ENSG00000137831	4255.66037	2045.754183	1.056660388	0.051561535	0.10463014	UACA
ENSG00000090376	660.1954306	1318.20782	-0.997404642	0.05156229	0.10463014	IRAK3
ENSG00000212766	7.033050481	1.029478846	2.734477718	0.051564001	0.10463014	EWSAT1
ENSG00000162972	196.5196439	279.6226655	-0.509282265	0.051647732	0.104791857	MAIP1
ENSG00000276850	0	3.210006989	-4.264979362	0.051715469	0.104912907	AC245041.2
ENSG00000230309	0	3.210006989	-4.264979362	0.051715469	0.104912907	AL121718.1
ENSG00000223638	6.399898697	0	5.011421635	0.051751171	0.104977138	RFPL4A
ENSG00000154723	1161.893909	1590.612455	-0.453194714	0.051765492	0.104992307	ATP5PF
ENSG00000162636	533.2884292	356.8498292	0.579038792	0.051766732	0.104992307	FAM102B
ENSG00000258137	0	3.760092102	-4.485413039	0.051801662	0.105047025	AC079313.2
ENSG00000275882	41.25747938	84.71154247	-1.038597205	0.051801797	0.105047025	IKBKGP1
ENSG00000136444	868.3530899	637.3257999	0.446386426	0.051866291	0.105169602	RSAD1
ENSG00000162670	7.989673224	0.336469917	4.369190716	0.051908459	0.105246891	BRINP3
ENSG00000184368	3.648657432	11.33359552	-1.641864006	0.051916231	0.105249393	MAP7D2
ENSG00000087095	523.6109841	360.9741663	0.536347459	0.051917795	0.105249393	NLK
ENSG00000137868	70.19579352	264.8997722	-1.916586686	0.051935885	0.105277852	STRA6
ENSG00000181090	922.7820335	1240.077786	-0.426288309	0.051968212	0.105335162	EHMT1
ENSG00000091583	0	5.685582588	-5.084771497	0.051991333	0.105373807	APOH
ENSG00000268744	6.362146692	1.029478846	2.58853836	0.052001463	0.105380526	AC008758.4
ENSG00000146453	6.379840317	0	5.007063652	0.052002761	0.105380526	PNLDC1
ENSG00000262179	6.152074289	0	4.953914991	0.052045045	0.105457988	MYMX
ENSG00000172071	432.2285392	321.8425398	0.425717296	0.052050202	0.105460211	EIF2AK3
ENSG00000065320	441.9452845	94.40162951	2.227657504	0.052106453	0.105565951	NTN1
ENSG00000253972	3.896553111	0	4.297196198	0.052127364	0.105600081	MAL2-AS1
ENSG00000181240	9.396670886	1.436768536	2.709428797	0.052151161	0.105640053	SLC25A41
ENSG00000240750	0	3.189937893	-4.257032186	0.052179412	0.10568904	RN7SL559P
ENSG00000141965	72.99389121	40.80917702	0.836416248	0.052214324	0.105751509	FEM1A
ENSG00000159792	700.8386457	963.0672117	-0.458571084	0.052225885	0.105766679	PSKH1
ENSG00000169064	4.586332902	0.336469917	3.567478975	0.052244157	0.105795435	ZBBX
novel.475	428.4202702	156.8806945	1.450396783	0.052279785	0.105855957	-
ENSG00000102897	324.9618681	475.4681057	-0.549753851	0.052282192	0.105855957	LYRM1
ENSG00000051009	875.6341809	584.4550514	0.583473129	0.052303618	0.105891086	FAM160A2
ENSG00000266907	9.118473823	2.51699806	1.857229204	0.052334528	0.105945409	AC006116.1
ENSG00000226121	50.7374609	82.73867457	-0.706873301	0.052351999	0.105972519	AC009487.2
ENSG00000146063	1259.01639	948.7304515	0.408179791	0.052373975	0.106008744	TRIM41
ENSG00000287145	3.988497097	0	4.327987897	0.052381662	0.106016045	AC009413.2
ENSG00000175497	6.760010555	0	5.090049864	0.05239132	0.106027331	DPP10
ENSG00000099999	399.5135949	578.6508996	-0.534034273	0.052442084	0.1061218	RNF215
ENSG00000222057	3.666279786	0	4.206775402	0.052467113	0.106155911	RNU4-62P
ENSG00000234350	3.666279786	0	4.206775402	0.052467113	0.106155911	AC007405.1
ENSG00000184887	1103.377491	788.7118204	0.484201903	0.052531254	0.106277411	BTBD6
novel.150	0.308355904	4.035198856	-3.626925187	0.052559995	0.106327278	-
ENSG00000089225	0.601596751	5.851015645	-3.284727981	0.052567827	0.106334842	TBX5
ENSG00000005436	393.8445899	247.4222498	0.66962485	0.052591373	0.10637419	GCFC2
ENSG00000131584	1225.694666	1636.193094	-0.416764186	0.052695427	0.106576358	ACAP3
ENSG00000236581	61.94000566	18.00749128	1.780714807	0.052735228	0.106648553	STARD13-AS
ENSG00000196811	1.833766717	8.213321528	-2.170822989	0.052758522	0.106687356	CHRNG
ENSG00000197162	243.3276008	179.2310649	0.4402205	0.052788756	0.106740188	ZNF785
ENSG00000271474	7.982008789	0.713078025	3.477326104	0.0527989	0.106752391	AC106881.1
ENSG00000255031	37.80377929	72.06714961	-0.927782403	0.052836725	0.106820555	AP002807.1

ENSG00000285722	7.089678488	0	5.15865646	0.052851025	0.106831912	AC207130.1
ENSG00000223875	7.089678488	0	5.15865646	0.052851025	0.106831912	NBEAP3
ENSG00000125124	2033.481192	1172.232712	0.794897018	0.052861727	0.106831912	BBS2
ENSG00000269376	3.646150135	0	4.199607263	0.052866339	0.106831912	AL356740.3
ENSG00000187862	3.646150135	0	4.199607263	0.052866339	0.106831912	TTC24
ENSG00000115657	41.68808065	68.35323186	-0.713354187	0.052867014	0.106831912	ABCB6
ENSG00000133943	662.6240948	416.3448191	0.671024948	0.052871986	0.106833651	DGLUCY
ENSG00000269894	11.47826201	3.199394504	1.836985041	0.052878837	0.106839184	AC018809.1
ENSG00000254206	50.74741917	22.50201478	1.168222137	0.052903235	0.106880167	NPIPB11
ENSG00000261141	7.050672834	0.713078025	3.297702576	0.052931444	0.106928843	AC092718.5
ENSG00000258484	15.84058983	59.19969776	-1.90433983	0.052949348	0.106953158	SPESP1
ENSG00000128000	19.09779419	8.621767093	1.147628235	0.052951714	0.106953158	ZNF780B
ENSG00000106804	268.5926712	134.1006314	1.000943543	0.052972265	0.106986351	C5
ENSG00000179241	789.0344595	472.9442196	0.737917757	0.052982528	0.10699876	LDLRAD3
ENSG00000228340	162.8055241	38.40711813	2.081526486	0.053014829	0.107051164	MIR646HG
ENSG00000287085	6.878067702	1.080229524	2.675767301	0.053016717	0.107051164	AC012066.2
ENSG00000171236	9.77927715	2.149846562	2.182145267	0.053088871	0.107188526	LRG1
ENSG00000165506	237.3275747	315.2644604	-0.409923685	0.053099563	0.107195556	DNAAF2
ENSG00000153786	1377.508713	1789.42754	-0.377584282	0.053100605	0.107195556	ZDHHC7
ENSG00000164494	224.003935	326.0468149	-0.542182327	0.053164058	0.10730209	PDSS2
ENSG00000235043	23.55937442	44.02864062	-0.903853504	0.053164344	0.10730209	TECRP1
ENSG00000255872	3.946912875	0	4.314110714	0.053165767	0.10730209	AL138752.2
ENSG00000214078	1338.976389	1862.172065	-0.475809692	0.053171115	0.107304546	CPNE1
ENSG00000225535	5.48242415	0.723690511	2.92812094	0.05318846	0.107331213	LINC01393
ENSG00000231341	0.307102255	4.473170127	-3.773616218	0.053203228	0.107352678	VDAC1P6
ENSG00000179397	31.21895244	14.1031911	1.150843342	0.05327972	0.107498672	CATSPERE
ENSG00000173208	18.81340015	6.287831042	1.567916265	0.053307702	0.107546778	ABCD2
ENSG00000214198	20.05685296	8.194408307	1.28684602	0.053331364	0.107573431	TTC41P
ENSG00000286679	3.938137334	0	4.311180507	0.053333334	0.107573431	AC073225.1
ENSG00000254463	3.938137334	0	4.311180507	0.053333334	0.107573431	PPIAP41
ENSG00000114573	826.0598643	1073.078126	-0.377656274	0.053343125	0.107584826	ATP6V1A
ENSG00000130921	339.4040223	444.9449372	-0.39054055	0.053353299	0.107596994	C12orf65
ENSG00000137411	0.307102255	4.485066883	-3.791799587	0.053395545	0.107668517	VARS2
ENSG00000281420	1.828752122	10.28276331	-2.491114592	0.053397053	0.107668517	AP001052.1
ENSG00000259767	6.301686468	0.713078025	3.138430842	0.053402095	0.107670327	AC022558.1
ENSG00000185267	40.83608092	20.48435115	0.989375406	0.053433803	0.107725896	CDNF
ENSG00000198642	859.019414	1151.315517	-0.422742955	0.053547419	0.107946578	KLHL9
ENSG00000179943	358.6301163	272.1402605	0.397912457	0.053566827	0.107977325	FIZ1
ENSG00000156671	1356.576982	895.8667073	0.598971664	0.053571649	0.107978667	SAMD8
ENSG00000196954	981.4708023	1450.744081	-0.563512357	0.05358213	0.107991414	CASP4
ENSG00000287475	6.123097826	0.743759607	3.06947994	0.053602739	0.108024571	AL844892.2
ENSG00000196912	107.9985055	57.13401475	0.921248297	0.053619723	0.108050417	ANKRD36B
ENSG00000162639	151.6878996	84.68325385	0.838880776	0.053715643	0.108235312	HENMT1
ENSG00000208028	21.32649019	5.951232731	1.855106036	0.053766159	0.108320915	MIR616
ENSG00000120341	10.27256121	3.189937893	1.6787047	0.053766465	0.108320915	SEC16B
ENSG00000101421	2106.657138	2690.558636	-0.352900962	0.053795437	0.108370881	CHMP4B
ENSG00000231131	12.94704482	1.782695063	2.856775501	0.053819594	0.108411139	LNCAROD
ENSG00000171861	204.1190464	290.2730938	-0.50890749	0.05382687	0.10841739	MRM3
novel.559	10.97745606	3.260757667	1.755605611	0.053883231	0.108522498	-
ENSG00000280160	1.49643435	8.101079293	-2.423869057	0.05389019	0.108528101	AC135050.7
ENSG00000255650	7.166293954	1.029478846	2.766997869	0.053907131	0.108553605	FAM222A-AS1

ENSG00000159708	6.036168435	1.080229524	2.486115795	0.053911211	0.108553605	LRRC36
ENSG00000275223	1.233423615	8.580473026	-2.809570848	0.053929691	0.108582401	AL121906.2
ENSG00000107669	564.6612967	388.7837897	0.537580698	0.053964676	0.10864442	ATE1
ENSG00000117751	777.980277	1007.231841	-0.372817855	0.053980137	0.108667126	PPP1R8
ENSG00000113249	3.590704505	0	4.179685476	0.053991221	0.108672598	HAVCR1
ENSG00000261456	3.590704505	0	4.179685476	0.053991221	0.108672598	TUBB8
ENSG00000180764	9.974234497	24.62092297	-1.301147207	0.054001889	0.108685652	PIPSL
ENSG00000246203	17.82404	7.429423729	1.254466436	0.054008662	0.108690863	AL353807.3
ENSG00000230082	40.12233926	21.9009222	0.874504061	0.054022448	0.108710187	PRRT3-AS1
ENSG00000100979	2932.356039	1276.008756	1.200438151	0.054030446	0.108717861	PLTP
ENSG00000121905	7.051926483	0.713078025	3.29799399	0.054058333	0.10876555	HPCA
ENSG00000174177	279.455246	428.6474075	-0.617818085	0.054082929	0.108806611	CTU2
ENSG00000104979	1360.195351	1890.806226	-0.47509904	0.05411306	0.108854894	C19orf53
ENSG00000133115	6.159596182	0	4.955629048	0.054115308	0.108854894	STOML3
ENSG00000106868	211.3474808	366.3410832	-0.794285833	0.054154386	0.108925068	SUSD1
ENSG00000287362	11.11069953	1.682349583	2.689158726	0.054206726	0.109021903	AC103563.9
ENSG00000149823	1133.840489	1496.315722	-0.400091381	0.054235628	0.109071588	VPS51
ENSG00000041880	509.8544634	361.7931936	0.494927886	0.054264241	0.109120685	PARP3
ENSG00000147378	4.288077459	0	4.432879494	0.054296399	0.109176901	FATE1
ENSG00000261409	6.393701724	0	5.010088735	0.054328129	0.109232249	AL035425.3
ENSG00000273557	3.88645265	0	4.293742002	0.054338402	0.109244449	AC008781.3
ENSG00000161791	2517.82264	1489.954148	0.757103438	0.054343377	0.109245998	FMNL3
ENSG00000270012	45.93045694	20.82783753	1.14191419	0.05438169	0.10931456	AC232271.1
ENSG00000179799	3.882691704	0	4.2924721	0.054412576	0.109368183	OR7E22P
ENSG00000243659	6.022307028	0.672939833	3.10672399	0.054573045	0.109676505	FO393419.3
ENSG00000070915	6.365907639	0.672939833	3.187663976	0.054574414	0.109676505	SLC12A3
ENSG00000047634	524.4894126	312.7743851	0.745344465	0.054581147	0.109681551	SCML1
ENSG00000160396	14.91684704	4.953719736	1.58159719	0.054586013	0.109682847	HIPK4
ENSG00000248476	19.72933632	6.460152171	1.612270581	0.054624477	0.109751646	BACH1-IT1
ENSG00000225216	18.56235644	2.272572888	3.04974264	0.05469473	0.1098843	AC007362.1
ENSG00000172366	377.364394	506.9710989	-0.42580042	0.054700963	0.109888326	MCRIP2
ENSG00000180182	1410.21167	873.6871076	0.690795254	0.054741314	0.109960884	MED14
ENSG00000182107	62.3347768	16.94031439	1.874202531	0.054782861	0.110035833	TMEM30B
ENSG00000120756	6.125605123	17.4065271	-1.514019128	0.054788536	0.110038726	PLS1
ENSG00000175376	318.6664929	447.079291	-0.488112631	0.054896726	0.110247496	EIF1AD
ENSG00000133639	1961.174944	1234.142886	0.668336852	0.054924824	0.110295398	BTG1
ENSG00000173812	11086.33498	8656.585609	0.356914755	0.05493416	0.110302398	EIF1
ENSG00000141027	1381.671796	1850.901136	-0.421909962	0.054936801	0.110302398	NCOR1
ENSG00000276517	24.49049691	8.458902575	1.528685178	0.055089049	0.110591997	AL133243.3
ENSG00000263934	19.41485436	4.391737868	2.150951823	0.055089551	0.110591997	SNORD3A
ENSG00000169019	287.029247	477.6065351	-0.735296831	0.055127554	0.110652573	COMMD8
ENSG00000129009	7461.87269	691.6398355	3.431363407	0.055128244	0.110652573	ISLR
ENSG00000122008	553.796927	405.5943013	0.449483923	0.055147806	0.110683287	POLK
ENSG00000129946	382.5876512	131.3025251	1.543848386	0.055168144	0.110715553	SHC2
ENSG00000253785	21.34830077	42.39588584	-0.993466385	0.055175999	0.110722764	AC008429.2
ENSG00000134590	2808.043589	3880.821927	-0.466769406	0.055191162	0.110744639	RTL8C
ENSG00000096080	213.8764417	295.8145967	-0.468553217	0.055269396	0.110893056	MRPS18A
ENSG00000229373	1.200686205	6.481377142	-2.425115452	0.055312879	0.11097173	LINC00452
ENSG00000160551	2266.943878	1601.007314	0.501879483	0.055321133	0.11097972	TAOK1
ENSG00000225022	10.56830936	21.87024062	-1.048181114	0.055376649	0.111082513	UBE2D3P1
ENSG00000157734	53.97851001	27.25961987	0.984401134	0.055405348	0.111131503	SNX22

ENSG00000131668	1.789675198	16.66952717	-3.211872709	0.055422102	0.111156525	BARX1
ENSG00000237672	19.54566181	7.000780673	1.494482166	0.055430118	0.111164021	KRR1P1
ENSG00000220267	0.308355904	4.208804254	-3.697686032	0.055447166	0.111189628	ACTBP8
ENSG00000236491	4.233885479	0.336469917	3.454162722	0.05553216	0.111344024	AC234771.4
ENSG00000260086	19.50819454	7.602900733	1.362635139	0.05553273	0.111344024	AC007611.1
ENSG00000261078	5.961846803	0	4.910369308	0.055541402	0.111352818	AC009118.1
ENSG00000101966	964.8851415	1280.483594	-0.408377627	0.055584556	0.111430736	XIAP
ENSG00000204922	172.449363	258.2462839	-0.583556837	0.055665557	0.111581215	UQCC3
ENSG00000226278	141.6747025	32.4061622	2.125469057	0.055668835	0.111581215	PSPHP1
ENSG00000136206	10.79983633	25.04841015	-1.210921494	0.055672502	0.111581215	SPDYE1
ENSG00000204524	188.436417	130.9071791	0.527244788	0.055678385	0.111584396	ZNF805
ENSG00000261215	11.18759973	2.904218654	1.952768747	0.055686351	0.111591753	AL162231.4
ENSG00000137038	661.6597649	975.6656526	-0.560601908	0.055729705	0.111670018	DMAC1
ENSG00000272767	7.306090406	21.16790347	-1.539640989	0.055794787	0.111791808	JMJD1C-AS1
ENSG00000261553	1.84261353	16.72310317	-3.188135383	0.055825422	0.111837349	AL137782.1
ENSG00000007314	3.341555177	0	4.073713166	0.055836774	0.111837349	SCN4A
ENSG00000254239	3.341555177	0	4.073713166	0.055836774	0.111837349	AC002428.2
ENSG00000243709	3.341555177	0	4.073713166	0.055836774	0.111837349	LEFTY1
ENSG00000179242	43.98532824	102.1244437	-1.217513951	0.05583904	0.111837349	CDH4
ENSG00000100239	1327.780445	1748.919258	-0.397334904	0.055854158	0.111850689	PPP6R2
ENSG00000144063	33.18423826	162.5490115	-2.293290175	0.05585431	0.111850689	MALL
ENSG00000197070	502.2320686	378.5059883	0.407545224	0.055869035	0.111867144	ARRDC1
ENSG00000198265	1050.88131	788.1501977	0.415154372	0.055871139	0.111867144	HELZ
ENSG00000276093	8.140539051	1.029478846	2.952735563	0.05588393	0.111884132	AL132822.1
ENSG00000213212	3.268487194	12.20953807	-1.891818479	0.055911981	0.111931667	NCLP1
ENSG00000165630	86.7761676	139.8857891	-0.690133349	0.055945013	0.111989165	PRPF18
ENSG00000104312	337.492173	478.4839934	-0.502936881	0.055959276	0.112009086	RIPK2
ENSG00000275162	0.61420451	6.358522421	-3.375896575	0.055965921	0.112013759	AC005391.1
ENSG00000263990	23.05703009	8.448161695	1.459551381	0.0559723	0.112017896	AC004253.1
ENSG00000287998	6.068905845	0.774441189	3.034189874	0.056039601	0.112135549	AC104232.3
ENSG00000085831	3.987243448	12.90139112	-1.701051354	0.05603972	0.112135549	TTC39A
ENSG00000188612	2074.291589	2848.245891	-0.457520612	0.056056256	0.112159999	SUMO2
ENSG00000187607	155.8656991	209.3853184	-0.425986124	0.056078119	0.112195103	ZNF286A
ENSG00000180185	463.1327806	691.5706564	-0.578470918	0.056092551	0.112210633	FAHD1
ENSG00000117859	1508.129733	2036.196028	-0.433050155	0.056098137	0.112210633	OSBPL9
ENSG00000272666	3.32769377	0	4.068382844	0.056120433	0.112210633	U62317.1
ENSG00000126952	3.32769377	0	4.068382844	0.056120433	0.112210633	NXF5
ENSG00000182798	3.32769377	0	4.068382844	0.056120433	0.112210633	MAGEB17
ENSG00000260613	3.32769377	0	4.068382844	0.056120433	0.112210633	Z98885.2
ENSG00000285000	3.32769377	0	4.068382844	0.056120433	0.112210633	AC008581.2
ENSG00000231741	3.32769377	0	4.068382844	0.056120433	0.112210633	AL353743.3
ENSG00000276768	3.326440121	0	4.067902767	0.056148698	0.112252297	AL121890.2
ENSG00000102760	758.5538693	279.8194346	1.438212797	0.056149911	0.112252297	RGCC
ENSG00000254602	13.65946156	26.44619637	-0.955926468	0.056217641	0.112379051	AP000662.1
ENSG00000102226	1993.71685	1503.647942	0.406987252	0.056227706	0.112390523	USP11
ENSG00000229873	20.15359807	7.896920707	1.352754441	0.056235552	0.11239756	OGFR-AS1
ENSG00000256973	24.92987373	9.201506307	1.433389695	0.056255254	0.112428288	AC053513.2
ENSG00000217165	45.23888186	21.05450536	1.101374247	0.056262756	0.112434634	ANKRD18EP
ENSG00000102781	631.4035215	907.8169819	-0.523572255	0.056282223	0.112464885	KATNAL1
ENSG00000196083	300.5570469	212.6996521	0.497572072	0.056316972	0.112525667	IL1RAP
ENSG00000163131	2291.523327	710.034806	1.69050127	0.056323383	0.112529823	CTSS

ENSG00000171109	674.6411887	966.9623464	-0.519115142	0.056388666	0.112648381	MFN1
ENSG00000100722	706.3477535	911.3201697	-0.367750999	0.056391395	0.112648381	ZC3H14
ENSG00000204619	0	29.41807114	-7.459949027	0.056403274	0.112663447	PPP1R11
ENSG00000173846	835.319435	533.3010715	0.647459624	0.056412877	0.112673966	PLK3
ENSG00000249096	7.226754179	1.386017859	2.355737916	0.05642895	0.112689875	LINC02362
ENSG00000146950	211.7926127	492.9261757	-1.218866468	0.056429517	0.112689875	SHROOM2
ENSG00000131023	756.0295965	552.0046879	0.453561708	0.056441578	0.112705299	LATS1
ENSG00000203288	11.29967337	1.682349583	2.71285898	0.056518943	0.112847461	TDRKH-AS1
ENSG00000204060	7.6799924	0.713078025	3.421553035	0.056521458	0.112847461	FOXO6
ENSG00000176909	49.53656123	78.54022601	-0.662251592	0.056603259	0.113002096	MAMSTR
ENSG00000231530	1.510295757	7.34799147	-2.287799302	0.056631265	0.113049319	AL157932.1
ENSG00000169084	370.8294993	515.6046344	-0.475423206	0.056637461	0.113053002	DHRSX
ENSG00000001084	365.1868647	493.4094695	-0.433518465	0.056653402	0.113076133	GCLC
ENSG00000174276	150.1312623	209.7747223	-0.482038136	0.05668133	0.11311936	ZNHIT2
ENSG00000284695	0	5.167463326	-4.953815222	0.056683767	0.11311936	AC108941.2
ENSG00000189195	134.629757	80.98280828	0.735425632	0.056695108	0.113133303	BTBD8
ENSG00000178860	142.5638689	405.4927534	-1.508202873	0.056706105	0.113146556	MSC
ENSG00000118492	3.951998741	0	4.315845035	0.056754288	0.11322812	ADGB
novel.877	6.213503428	0	4.970339299	0.056755699	0.11322812	-
ENSG00000180438	6.471713032	0	5.027003992	0.056813668	0.113335067	TPRXL
ENSG00000182901	26.59264497	12.70771663	1.071448329	0.056854123	0.113407062	RGS7
ENSG00000259407	8.779887807	1.345879667	2.66379691	0.056864351	0.113418756	AC021739.2
ENSG00000275389	8.13043859	1.456837632	2.490949954	0.056903655	0.113488437	AC068790.9
ENSG00000260923	9.850806751	1.733100261	2.488391197	0.05691636	0.113505063	LINC02193
novel.18	15.05510511	4.768345977	1.670590113	0.056954207	0.113571821	-
ENSG00000108733	222.3322456	136.3752593	0.703132525	0.056964068	0.113582767	PEX12
ENSG00000132274	3462.078978	2020.398722	0.776884166	0.05699501	0.113635741	TRIM22
ENSG00000160180	51.41004526	8.215761673	2.638909307	0.057007275	0.113644749	TFF3
ENSG00000228109	102.796671	56.91659392	0.855546925	0.057008276	0.113644749	MELTF-AS1
ENSG00000136631	913.9344605	675.932838	0.434752404	0.057029338	0.113678013	VPS45
ENSG00000138653	4.262861942	0.356539013	3.462611188	0.057071566	0.113753041	NDST4
ENSG00000144852	11.95768466	1.682349583	2.79828031	0.057075734	0.113753041	NR1I2
ENSG00000274270	9.102105118	1.426156051	2.668539603	0.057171046	0.11392576	AL137060.1
ENSG00000139572	24.69333216	6.429342195	1.957093647	0.057171166	0.11392576	GPR84
ENSG00000166963	1347.802421	1855.416218	-0.461052211	0.057176253	0.11392716	MAP1A
ENSG00000248714	12.86541476	3.179325408	2.006276561	0.057182316	0.113930503	AC091180.2
ENSG00000125434	164.1882592	85.04467315	0.946662365	0.0571868	0.1139307	SLC25A35
ENSG00000184350	6.781322584	0	5.094479633	0.057220945	0.113989985	MRGPRE
ENSG00000138646	177.187416	98.16369503	0.8530305	0.057250866	0.114040846	HERC5
ENSG00000261474	35.97251987	16.20228698	1.153118569	0.05725889	0.114048084	AC026471.4
ENSG00000169217	1476.771264	1966.555061	-0.413338996	0.057275772	0.114072965	CD2BP2
ENSG00000271643	56.88953519	94.06756538	-0.727700306	0.057303995	0.114120428	AC112220.2
ENSG00000234678	63.39599473	38.86934963	0.707528628	0.057331627	0.114166705	ELF3-AS1
ENSG00000228474	1738.416812	2480.211203	-0.512764529	0.057342934	0.114180469	OST4
ENSG00000064651	968.8505707	396.3299505	1.289298031	0.05739099	0.114267401	SLC12A2
ENSG00000286427	6.026067974	0.713078025	3.073226446	0.057405251	0.114287036	AC009486.2
ENSG00000139330	5.847479332	0	4.880598055	0.057425396	0.114315253	KERA
ENSG00000139684	1794.366569	2291.50341	-0.352809248	0.057428224	0.114315253	ESD
ENSG00000162482	40.38953822	17.17027427	1.238660432	0.057448919	0.114347688	AKR7A3
ENSG00000164746	13.28563083	3.72941052	1.853602503	0.057479491	0.114399774	C7orf57
ENSG00000235381	26.93373828	48.47125756	-0.850920819	0.0575237	0.114478993	AL596202.1

ENSG00000278053	112.0396124	190.5123687	-0.766302558	0.057556804	0.1145361	DDX52
ENSG00000205456	4.803998469	0.387220594	3.637473655	0.057572111	0.114555249	TP53TG3D
ENSG00000219665	41.80892983	66.74041778	-0.673571613	0.057575245	0.114555249	ZNF433-AS1
ENSG00000175611	84.27275074	48.15485674	0.803502153	0.057614703	0.114605842	LINC00476
ENSG00000176349	0	5.23699883	-4.966889428	0.057618317	0.114605842	AC104129.1
ENSG00000110244	0	5.23699883	-4.966889428	0.057618317	0.114605842	APOA4
ENSG00000265799	0	5.23699883	-4.966889428	0.057618317	0.114605842	AC090844.3
ENSG00000206562	241.5655059	321.2490244	-0.411772402	0.057628969	0.114618254	METTL6
ENSG00000213799	162.3199323	114.0251926	0.509190057	0.057662982	0.114677122	ZNF845
ENSG00000272702	6.349538934	15.80560977	-1.314804169	0.057678063	0.114693688	AC010913.1
ENSG00000187024	3.342808826	0	4.074203769	0.057680141	0.114693688	PTRH1
ENSG00000176092	9.206656863	1.069617038	3.099125714	0.057685219	0.114695009	CRYBG2
novel.133	11.21908349	24.81459746	-1.145341497	0.057699389	0.114714403	-
ENSG00000177854	183.3382088	268.0922661	-0.54738766	0.057713816	0.114734306	TMEM187
ENSG00000273723	28.78129513	14.02175884	1.037989394	0.057718835	0.114735505	AL139089.1
ENSG00000188039	3.340301529	0	4.073243849	0.057738194	0.114747649	NWD1
ENSG00000272710	3.340301529	0	4.073243849	0.057738194	0.114747649	AC133041.1
ENSG00000261996	3.340301529	0	4.073243849	0.057738194	0.114747649	AC004706.1
ENSG00000271848	5.406848869	0.693008929	2.932925208	0.057751286	0.114764888	AC073389.2
ENSG00000285584	0	5.206317249	-4.958837546	0.057775056	0.114803345	AL158800.1
ENSG00000273381	11.78285697	4.004517274	1.566868301	0.057784301	0.114812933	AL158071.5
ENSG00000185670	109.085109	73.64226564	0.566006313	0.057790045	0.114815567	ZBTB3
ENSG00000165349	5.84873298	0	4.880898304	0.057796086	0.114818789	SLC7A3
ENSG00000141384	75.11240796	122.9227899	-0.709026047	0.057803534	0.114824805	TAF4B
ENSG00000147905	314.2996487	462.7779112	-0.557601637	0.05782184	0.114838255	ZCCHC7
ENSG00000228668	4.484288455	0.336469917	3.539097916	0.057823501	0.114838255	TRGV5P
ENSG00000101883	4.207416313	0.356539013	3.446405844	0.057823565	0.114838255	RHOXF1
ENSG00000287222	60.2358915	36.56737943	0.715909144	0.057857004	0.114895881	AC234917.3
ENSG00000258725	5.088392504	15.61077941	-1.609813888	0.057865447	0.114903866	PRC1-AS1
ENSG00000205622	10.15916265	1.426156051	2.829083914	0.057872967	0.114910016	AP001042.1
ENSG00000211649	4.608969851	0	4.536888443	0.057880137	0.114915469	IGLV7-46
ENSG00000118058	1497.039917	983.2218138	0.606818387	0.057897762	0.114941678	KMT2A
ENSG00000040341	474.6053873	338.8122515	0.486168401	0.057980531	0.115097202	STAU2
ENSG00000277151	12.67644092	4.391737868	1.542335373	0.058024558	0.1151758	AL138820.1
ENSG00000226445	91.37006584	50.68349476	0.849882834	0.058046979	0.115211501	BX322234.1
ENSG00000170296	63.03247793	37.88116485	0.73640303	0.058085831	0.115279809	GABARAP
ENSG00000258162	7.356592712	0	5.212276058	0.058122124	0.115341185	AC069228.1
ENSG00000046604	36.34204766	139.6782913	-1.943299015	0.058125635	0.115341185	DSG2
ENSG00000257671	0	28.38859229	-7.408568479	0.058130998	0.115343016	KRT7-AS
ENSG00000153487	565.9701962	403.2499759	0.489383096	0.058156397	0.115384601	ING1
ENSG00000239388	21.04718202	8.029103644	1.398757865	0.058178248	0.11541914	ASB14
ENSG00000280147	0	5.267680412	-4.974926563	0.058219855	0.115484047	AC084026.3
novel.908	0	5.267680412	-4.974926563	0.058219855	0.115484047	-
ENSG00000123609	543.0549567	279.7769849	0.955913248	0.058335481	0.115704569	NMI
ENSG00000116903	264.4739636	367.6967106	-0.475701807	0.058344521	0.115713664	EXOC8
novel.749	3.312578714	0	4.062538296	0.058386948	0.115753627	-
ENSG00000277893	3.312578714	0	4.062538296	0.058386948	0.115753627	SRD5A2
ENSG00000232070	3.312578714	0	4.062538296	0.058386948	0.115753627	TMEM253
ENSG00000255794	3.312578714	0	4.062538296	0.058386948	0.115753627	RMST
ENSG00000267015	3.312578714	0	4.062538296	0.058386948	0.115753627	AC023090.1
ENSG00000157423	9.884797809	2.211209725	2.178543094	0.058407092	0.115784728	HYDIN

ENSG00000147403	19283.65076	24991.97163	-0.374083931	0.058434172	0.115829574	RPL10
ENSG00000274128	6.162103479	0	4.956233121	0.058455428	0.115862869	AC027807.2
ENSG00000166770	346.1028606	240.3699824	0.527230316	0.05850562	0.115948432	ZNF667-AS1
ENSG00000206149	8.451402252	24.84065554	-1.554281221	0.058507522	0.115948432	HERC2P9
ENSG00000166881	511.7300445	729.4992793	-0.511565887	0.058603424	0.116129628	NEMP1
ENSG00000145214	423.755852	604.739739	-0.513195051	0.058614199	0.116142123	DGKQ
ENSG00000279307	5.782004512	18.55988815	-1.685188076	0.058650995	0.11620617	AC012065.5
ENSG00000091157	400.9487705	299.9722992	0.418933513	0.058669834	0.116234631	WDR7
ENSG00000005893	4455.056069	3372.957297	0.401462853	0.058685295	0.116256397	LAMP2
ENSG00000257258	6.661727055	0.672939833	3.256304471	0.058692396	0.116261599	AC012150.1
ENSG00000112769	8697.451766	11275.71852	-0.374548073	0.058781677	0.116423946	LAMA4
novel.764	24.32730806	11.34305213	1.093312788	0.058783316	0.116423946	-
ENSG00000233885	40.97817121	19.96366335	1.035874612	0.058789701	0.116427718	YEATS2-AS1
ENSG00000171824	1921.271333	1467.288082	0.388993301	0.058837073	0.116512651	EXOSC10
ENSG00000105229	791.7304452	551.7574849	0.521284739	0.058859694	0.116548564	PIAS4
ENSG00000064607	2080.605151	3017.001353	-0.535987541	0.058932199	0.116683239	SUGP2
ENSG00000259921	4.790137061	0.356539013	3.633735493	0.058942509	0.116694759	AC022819.1
ENSG00000099869	72.39468669	11.4882877	2.652694606	0.058981879	0.116763807	IGF2-AS
ENSG00000160539	185.5858086	104.2990173	0.832716767	0.058987794	0.11676662	PLPP7
ENSG00000239552	1.232169966	7.529769209	-2.622666518	0.059001831	0.116773428	HOXB-AS2
ENSG00000135473	823.206282	589.5715884	0.481937297	0.059003739	0.116773428	PAN2
ENSG00000108474	196.3446458	296.2187571	-0.592520241	0.059004717	0.116773428	PIGL
ENSG00000258670	6.771293394	0.336469917	4.130129549	0.059086884	0.116927133	AL049874.3
ENSG00000100380	3647.647906	4876.7662	-0.418928665	0.059096504	0.116937263	ST13
ENSG00000019169	16.21212707	2.557136252	2.669995443	0.059185614	0.117097229	MARCO
ENSG00000214941	456.6987636	345.4491387	0.403039068	0.05918636	0.117097229	ZSWIM7
ENSG00000145495	2543.572918	1977.717709	0.363083779	0.059208907	0.117132918	6-Mar
ENSG00000228983	3.660011542	0	4.204625415	0.059253699	0.117212605	SLC47A1P1
ENSG00000125089	693.0846877	236.7291271	1.549205227	0.059283844	0.117263308	SH3TC1
ENSG00000100227	1525.158441	1984.496822	-0.379956536	0.05939501	0.117474251	POLDIP3
ENSG00000226608	109.5509549	225.3649063	-1.039673094	0.059498254	0.117669494	FTLP3
novel.519	1.801029308	8.814157318	-2.281375872	0.059581108	0.117824384	-
ENSG00000271778	8.371994754	1.753169357	2.242092317	0.05960738	0.117867367	AC080013.3
ENSG00000197106	0.922560414	7.091669543	-2.946156522	0.059633535	0.117910111	SLC6A17
ENSG00000253304	137.0164688	372.3573438	-1.442551752	0.059655965	0.117936844	TMEM200B
ENSG00000119414	1257.159148	984.8677881	0.351926315	0.059656133	0.117936844	PPP6C
ENSG00000074416	4691.367793	6293.550261	-0.423832392	0.059663065	0.117941574	MGLL
ENSG00000145431	669.4175863	478.8059165	0.48392783	0.059687437	0.117972374	PDGFC
ENSG00000122026	5580.178061	8600.321444	-0.624089319	0.059687728	0.117972374	RPL21
ENSG00000177191	56.16164739	29.20980296	0.945006894	0.059696632	0.117980999	B3GNT8
ENSG00000171033	421.7922634	276.0287299	0.612006412	0.05970123	0.11798111	PKIA
ENSG00000105298	458.3864381	642.0843038	-0.486019363	0.059716905	0.118003112	CACTIN
ENSG00000262786	0	5.298361994	-4.982965428	0.059725713	0.11801154	AC005224.1
ENSG00000170852	1116.602975	866.7379767	0.365543941	0.059731287	0.118013579	KBTBD2
ENSG00000156345	193.7861397	117.8258552	0.716329899	0.059774586	0.118090146	CDK20
ENSG00000169446	517.1953289	747.8602874	-0.532527948	0.059797492	0.118126417	MMGT1
ENSG00000153774	637.9429011	961.7124108	-0.592004277	0.059836987	0.118188158	CFDP1
ENSG00000170927	5.851240278	0	4.881510605	0.059837844	0.118188158	PKHD1
ENSG00000287539	7.491018563	1.386017859	2.409903938	0.059852332	0.118207787	AC022966.3
ENSG00000182973	325.290824	454.5700435	-0.483186215	0.059965018	0.11842134	CNOT10
ENSG00000260249	5.842464737	0	4.879431292	0.059974408	0.118430881	AC007608.3

ENSG00000274386	20.05183836	6.279530307	1.663616318	0.060005036	0.118482356	TMEM269
ENSG00000182400	75.58953678	111.5894984	-0.56199058	0.060044817	0.118551895	TRAPPC6B
ENSG00000199005	0	3.342189926	-4.316591824	0.060095034	0.118642028	MIR370
ENSG00000226416	6.664234352	1.130980201	2.597202555	0.060119771	0.118681845	MRPL23-AS1
ENSG00000162735	734.4180122	995.4790585	-0.439078683	0.060179293	0.118790322	PEX19
ENSG00000274929	8.17453011	1.722487775	2.217190431	0.060195466	0.118813219	AL157813.1
ENSG00000188316	11.06660801	2.435565801	2.167834111	0.060203761	0.118820566	ENO4
ENSG00000261220	20.36904108	41.76154314	-1.033220661	0.060277437	0.118949852	AC103706.1
ENSG00000108960	530.7659544	808.832766	-0.60742011	0.060278424	0.118949852	MMD
ENSG00000251819	9.220233536	1.161661783	3.035575002	0.060312328	0.119001297	RNU6-322P
ENSG00000196498	4008.804384	5368.03592	-0.421183746	0.060315301	0.119001297	NCOR2
ENSG00000259032	40.3515724	63.24366407	-0.6487294	0.060320159	0.119001297	ENSAP2
ENSG00000100568	1532.892894	1164.449566	0.396528759	0.060322816	0.119001297	VTI1B
novel.206	5.737912992	0	4.854396232	0.06033277	0.119011897	-
ENSG00000130724	1930.834012	1506.607312	0.35794329	0.060389299	0.119114361	CHMP2A
ENSG00000214553	3.677633896	0	4.210960275	0.060420302	0.119166467	LRRC37A11P
ENSG00000137842	340.6457667	228.8993705	0.57349909	0.060503574	0.119321645	TMEM62
ENSG00000205277	7.656030532	1.436768536	2.411885604	0.060565547	0.1194348	MUC12
ENSG00000167740	406.3297906	300.3481025	0.435243517	0.060640856	0.119574231	CYB5D2
ENSG00000286657	3.356670233	0	4.079573932	0.060698219	0.119660097	AC008453.2
ENSG00000223318	3.356670233	0	4.079573932	0.060698219	0.119660097	RNA5SP111
ENSG00000286984	3.356670233	0	4.079573932	0.060698219	0.119660097	AC090578.3
ENSG00000205002	22.12597887	9.468312325	1.212966739	0.060728618	0.119693413	AARD
ENSG00000239941	3.355416585	0	4.079093285	0.060728939	0.119693413	AC108718.1
ENSG00000234506	3.355416585	0	4.079093285	0.060728939	0.119693413	LINC01506
ENSG00000165055	439.1824391	587.4097296	-0.419594078	0.060757339	0.119740303	METTL2B
ENSG00000104218	302.1630201	211.6263106	0.513502684	0.060773036	0.119762154	CSPP1
ENSG00000184163	24.03650324	48.91868544	-1.028273402	0.060858565	0.119921607	C1QTNF12
ENSG00000164754	2662.56707	3381.938934	-0.345070474	0.060899946	0.119994047	RAD21
ENSG00000271826	8.484139662	19.48311374	-1.198343058	0.060906146	0.119997163	PLS3-AS1
ENSG00000286847	6.613803318	1.049547942	2.636327851	0.060967713	0.120109353	AC007036.4
ENSG00000243943	601.8371252	455.6632317	0.401735381	0.060986528	0.12013731	ZNF512
ENSG00000265787	31.7627388	0	7.322921835	0.061040293	0.120234105	CYP4F35P
ENSG00000113597	469.9616865	333.8570847	0.492494091	0.061072785	0.120288988	TRAPPC13
ENSG00000198580	19.04994172	8.631223704	1.143605215	0.061105317	0.120343938	AC073343.1
ENSG00000212533	0	3.302051734	-4.300859437	0.061150806	0.120424399	RF00072
ENSG00000228021	6.402477266	1.049547942	2.586001006	0.061171845	0.120456323	AL158835.1
ENSG00000239381	3.576843098	0	4.174510327	0.06117629	0.120456323	AC125613.1
ENSG00000187778	885.53137	1152.993771	-0.380942853	0.061251846	0.120595954	MCRS1
ENSG00000273245	6.272710004	1.080229524	2.543560434	0.061337002	0.120754463	AC092653.1
ENSG00000105501	3.328947419	0	4.068858757	0.061384158	0.120838143	SIGLEC5
ENSG00000137409	4009.145197	5445.840442	-0.441801467	0.061408085	0.120876086	MTCH1
ENSG00000114487	5.7517744	0	4.857737366	0.061414901	0.120880344	MORC1
ENSG00000230596	1.493927053	7.621813954	-2.340159843	0.061426135	0.120893297	GPAA1P2
ENSG00000249234	3.325186472	0	4.067408118	0.061478029	0.120983533	AC108063.1
ENSG00000049167	129.9455675	190.5577723	-0.553759729	0.061481297	0.120983533	ERCC8
ENSG00000226237	4.237646425	13.60629681	-1.689201627	0.06153266	0.121075434	GAS1RR
novel.985	31.45535146	0	7.308893389	0.061538949	0.12107864	-
ENSG00000178201	18.33362149	5.869800472	1.653549158	0.061545802	0.121082954	VN1R1
novel.177	4.223785018	0.387220594	3.451160107	0.061591318	0.121163326	-
ENSG00000197905	195.4243361	342.5744582	-0.810532596	0.061596908	0.121165148	TEAD4

ENSG00000177119	2471.278212	1622.450992	0.607056525	0.061614564	0.121182042	ANO6
ENSG00000135185	291.0774923	429.7838034	-0.561733495	0.061614825	0.121182042	TMEM243
ENSG00000111371	1185.319705	2140.729675	-0.852786381	0.061622361	0.121187689	SLC38A1
ENSG00000219085	3.623441915	0	4.19143251	0.061683281	0.121298313	NPM1P37
ENSG00000267108	24.94122784	10.64058659	1.225429823	0.061744377	0.121409268	AP001029.1
ENSG00000254731	3.313832363	0	4.062993015	0.061763224	0.121409574	AP003059.1
ENSG00000275344	3.313832363	0	4.062993015	0.061763224	0.121409574	MIR6503
ENSG00000215704	3.313832363	0	4.062993015	0.061763224	0.121409574	CELA2B
ENSG00000257703	3.313832363	0	4.062993015	0.061763224	0.121409574	AC068643.1
ENSG00000278635	1.788421549	7.765893646	-2.112835966	0.061781256	0.12143583	AC141557.2
ENSG00000135824	3.311325065	0	4.062023003	0.061826386	0.121478696	RGS8
ENSG00000279447	3.311325065	0	4.062023003	0.061826386	0.121478696	AL118508.3
ENSG00000227888	3.311325065	0	4.062023003	0.061826386	0.121478696	FAM66A
ENSG00000253976	3.311325065	0	4.062023003	0.061826386	0.121478696	AC022679.2
ENSG00000253965	3.617173671	0	4.189170291	0.061831793	0.121478696	AC008438.2
ENSG00000260351	3.617173671	0	4.189170291	0.061831793	0.121478696	AC024270.2
ENSG00000228793	13.11080314	4.351599676	1.595758313	0.061836366	0.121478696	AL033523.1
ENSG00000129187	1629.220714	2297.969529	-0.496323665	0.061840469	0.121478696	DCTD
novel.982	7.051641749	0	5.152893779	0.061860491	0.12150884	-
ENSG00000154240	281.3722799	179.7041656	0.646883425	0.061877615	0.121533287	CEP112
ENSG00000139351	12.12903615	3.709341424	1.728348067	0.061948189	0.121662703	SYCP3
ENSG00000232303	5.131230375	0.723690511	2.834798273	0.061964714	0.121685958	DFFBP1
ENSG00000214717	1008.745519	1368.521706	-0.439879871	0.06200123	0.121748466	ZBED1
ENSG00000166833	707.7649114	1132.192654	-0.677724189	0.062039715	0.12181483	NAV2
ENSG00000287059	6.163357128	0	4.956546211	0.062072286	0.121860364	AC090004.2
ENSG00000287289	6.163357128	0	4.956546211	0.062072286	0.121860364	AP000428.2
ENSG00000274114	4.280484296	0.336469917	3.46785942	0.062090839	0.121887578	ALOX15P1
ENSG00000054803	3.676380247	0	4.210537601	0.062110838	0.121908414	CBLN4
ENSG00000226277	3.676380247	0	4.210537601	0.062110838	0.121908414	AC105393.1
ENSG00000181894	264.305974	193.1463137	0.452642917	0.062123878	0.121924797	ZNF329
ENSG00000203434	0	3.790773684	-4.496852127	0.062146262	0.121959516	AL353740.1
ENSG00000285535	3.991004395	0	4.328982275	0.062270229	0.122193566	AC021683.5
ENSG00000197020	15.87966676	4.463713517	1.845782226	0.062294215	0.122231402	ZNF100
ENSG00000119522	283.1169377	414.4322525	-0.549523003	0.062303298	0.122239994	DENND1A
ENSG00000023330	660.3678189	922.5622219	-0.482235835	0.062386011	0.122388625	ALAS1
ENSG00000240498	61.17947921	35.50477886	0.785568603	0.062388474	0.122388625	CDKN2B-AS1
ENSG00000121769	39.76000484	101.087422	-1.344992059	0.062433913	0.122460152	FABP3
ENSG00000085511	1076.570501	732.0090731	0.556911391	0.062434363	0.122460152	MAP3K4
ENSG00000234261	0	5.329043575	-4.991023381	0.062472956	0.122517352	AL138720.1
ENSG00000253270	0	5.329043575	-4.991023381	0.062472956	0.122517352	AC090541.1
ENSG00000120925	525.2763053	400.9629845	0.389110188	0.062509971	0.12258069	RNF170
ENSG00000214413	405.067078	301.9865423	0.423774828	0.062557266	0.122664176	BBIP1
ENSG00000230113	3.977142987	0	4.324330431	0.062578096	0.122686934	AC138207.1
ENSG00000205464	141.3035217	81.2679324	0.795834609	0.062578317	0.122686934	ATP6AP1L
ENSG00000171611	10.84894245	2.557136252	2.095114275	0.062603835	0.122727703	PTCRA
ENSG00000106991	3557.476753	7048.944154	-0.986592943	0.062714758	0.122930197	ENG
ENSG00000253328	5.492524611	0.743759607	2.913890373	0.062716591	0.122930197	SUMO2P19
ENSG00000244588	0.89358395	8.955796865	-3.312864524	0.06274261	0.122971635	RAD21L1
ENSG00000262903	53.5653889	84.59180422	-0.65666847	0.062747198	0.122971635	AC027796.4
ENSG00000178053	273.0981851	187.834561	0.540605695	0.062772337	0.123011622	MLF1
ENSG00000100325	957.4141138	1346.978539	-0.492399372	0.062781281	0.12301987	ASCC2

ENSG00000233585	12.75047782	2.058957692	2.611550574	0.062840581	0.123118321	AC231533.2
ENSG00000125337	6.678024489	0.336469917	4.111159758	0.062841001	0.123118321	KIF25
ENSG00000174233	1445.907056	2048.186159	-0.502302195	0.06287683	0.123173485	ADCY6
ENSG00000050130	801.4356257	1057.400217	-0.400126734	0.062878639	0.123173485	JKAMP
ENSG00000196427	5.66226644	0	4.836015674	0.062889236	0.123183715	NBPF4
ENSG00000105849	347.5440197	631.0963558	-0.861107085	0.062895339	0.123183715	TWISTNB
ENSG00000165886	556.4620215	834.4846963	-0.584240881	0.062898086	0.123183715	UBTD1
ENSG00000151729	997.4157427	546.7500989	0.866815367	0.062960053	0.123295781	SLC25A4
ENSG00000112167	238.6360754	171.560812	0.475602238	0.062997429	0.123359677	SAYSD1
ENSG00000264281	64.11761429	102.9244294	-0.685309385	0.063015245	0.123367172	AC016596.2
ENSG00000266288	5.654744548	0	4.834180312	0.063015501	0.123367172	AP005671.1
ENSG00000236197	5.654744548	0	4.834180312	0.063015501	0.123367172	AC002429.2
ENSG00000179363	7.194016769	1.467450118	2.312411727	0.063046791	0.123419129	TMEM31
ENSG00000129007	1.789675198	8.895717972	-2.306983987	0.063089313	0.123493064	CALML4
ENSG00000164053	225.262661	362.5981536	-0.686794684	0.063112457	0.123529059	ATRIP
ENSG00000165617	138.9287168	433.5045024	-1.641675725	0.06313441	0.12356113	DACT1
ENSG00000083896	222.8610747	352.295822	-0.660249875	0.063138354	0.12356113	YTHDC1
ENSG00000064309	61.07632365	32.11885472	0.929283036	0.063169459	0.123612692	CDON
ENSG00000150361	3.631035079	0	4.194190258	0.063195179	0.123653709	KLHL1
ENSG00000138399	275.6632164	384.0494518	-0.477875972	0.063262444	0.123776003	FASTKD1
ENSG00000277382	1.230916317	6.399944883	-2.392579668	0.063276495	0.123794174	AC005837.3
ENSG00000183186	3.369277992	14.50205166	-2.105391199	0.063291358	0.123813928	C2CD4C
novel.288	11.84331719	3.455588036	1.754148022	0.063303514	0.123828383	-
ENSG00000256006	4.532140921	13.52229601	-1.571506163	0.063321961	0.123855143	AC084117.1
ENSG00000233521	0.308355904	3.627909165	-3.475691776	0.06338777	0.123973867	LINC01638
ENSG00000113734	160.8645123	222.1379251	-0.465641855	0.063392203	0.123973867	BNIP1
novel.478	41.69860839	103.7573141	-1.314864888	0.063456851	0.124090957	-
ENSG00000242616	0	3.159256312	-4.244596704	0.063472187	0.124102265	GNG10
ENSG00000123364	0	3.159256312	-4.244596704	0.063472187	0.124102265	HOXC13
novel.591	5.321173127	0.356539013	3.786485942	0.063543946	0.124233221	-
ENSG00000112146	1107.563054	837.3945913	0.403426859	0.063607706	0.124348519	FBXO9
ENSG00000071243	280.6714467	188.253362	0.57541443	0.063628954	0.124371066	ING3
ENSG00000248663	3.357923882	0	4.08009168	0.063646968	0.124371066	LINC00992
ENSG00000271734	3.357923882	0	4.08009168	0.063646968	0.124371066	Z98200.1
ENSG00000189233	3.357923882	0	4.08009168	0.063646968	0.124371066	NUGGC
ENSG00000259954	3.357923882	0	4.08009168	0.063646968	0.124371066	IL21R-AS1
ENSG00000133101	15.84692935	4.852218381	1.690163889	0.063647962	0.124371066	CCNA1
ENSG00000231770	105.3702925	68.9020327	0.613839896	0.063668826	0.124402479	TMEM44-AS1
ENSG00000100280	1898.852752	2450.097892	-0.367660376	0.063682174	0.124418478	AP1B1
ENSG00000227855	36.25350897	12.5650496	1.521856502	0.063686592	0.124418478	DPY19L2P3
ENSG00000272668	97.09752236	47.26354981	1.037830026	0.06372809	0.124490188	AL590560.2
ENSG00000105223	4245.487635	6021.469153	-0.504174191	0.063739217	0.124502503	PLD3
ENSG00000287655	3.354162936	0	4.078644519	0.063743978	0.124502503	AC023283.1
ENSG00000251023	11.46447187	2.486316478	2.199707437	0.063776538	0.124556735	AC114980.1
ENSG00000224631	400.0706138	570.9104689	-0.513250711	0.063802772	0.124598604	RPS27AP16
ENSG00000229236	12.63353178	1.365948763	3.186777705	0.063817127	0.124617269	TTTY10
ENSG00000273230	90.39771513	58.66642405	0.626756762	0.063846626	0.124665504	AC102953.2
ENSG00000215302	37.23771198	19.4253466	0.932294938	0.063862176	0.124681669	AC127502.1
ENSG00000147036	21.54605015	1.782695063	3.592192095	0.063866574	0.124681669	LANCL3
ENSG00000273812	3.928036872	0.387220594	3.346197	0.063869302	0.124681669	BX640514.2
ENSG00000166004	125.6305227	183.3194074	-0.54485182	0.063876743	0.124686826	CEP295

ENSG00000173702	0.307102255	4.085949533	-3.643243289	0.063883747	0.12469113	MUC13
ENSG00000226049	19.77253019	39.88590424	-1.013564624	0.063898898	0.124711334	TLK2P1
ENSG00000142686	714.6851009	512.2792684	0.47981926	0.063915727	0.124734809	C1orf216
ENSG00000288096	17.6567342	6.072931576	1.536300371	0.063925298	0.124744116	AC109322.2
ENSG00000267278	28.74318712	64.1646251	-1.157430112	0.063960026	0.124802511	MAP3K14-AS1
ENSG00000227963	3.928036872	11.29230146	-1.527883675	0.063973707	0.124819832	RBM15-AS1
ENSG00000226133	3.344062475	0	4.074724623	0.064006155	0.124864387	AL356289.1
ENSG00000238015	3.344062475	0	4.074724623	0.064006155	0.124864387	AC104837.2
ENSG00000159210	743.4540452	960.0008176	-0.368995886	0.064016516	0.124875224	SNF8
ENSG00000240350	0.293240848	4.085949533	-3.643254329	0.064030953	0.124894009	AC017002.3
ENSG00000122643	391.8633188	268.4408552	0.544761682	0.06408572	0.124985579	NT5C3A
ENSG00000173041	199.8590477	137.4999465	0.538311882	0.064087521	0.124985579	ZNF680
ENSG00000231119	6.065144898	16.46092066	-1.446987927	0.064094285	0.124989389	AL031666.1
ENSG00000257923	2005.144903	1481.026339	0.437166447	0.064101298	0.124993683	CUX1
ENSG00000229124	51.12640668	26.56648255	0.947828034	0.064108143	0.124997649	VIM-AS1
ENSG00000170279	3.33904788	0	4.072789161	0.0641367	0.125018137	C7orf33
ENSG00000286957	3.33904788	0	4.072789161	0.0641367	0.125018137	AC079776.5
ENSG00000285838	3.33904788	0	4.072789161	0.0641367	0.125018137	AL646090.2
ENSG00000175787	93.33144852	61.36049513	0.607345875	0.064137899	0.125018137	ZNF169
ENSG00000271614	78.22229362	42.7603404	0.87517643	0.064160467	0.125044663	ATP2B1-AS1
ENSG00000223060	0.293240848	3.597227584	-3.465722689	0.064161133	0.125044663	RF00019
ENSG00000255241	3.589450856	0	4.179056936	0.064215739	0.125140159	AP005436.2
ENSG00000166411	301.1636582	419.6604495	-0.479471234	0.064219766	0.125140159	IDH3A
ENSG00000144744	1049.575503	1321.92297	-0.33292536	0.064240258	0.125170702	UBA3
ENSG00000240505	5.853747575	0	4.882143265	0.064272639	0.125224405	TNFRSF13B
novel.708	818.3334044	1381.801822	-0.75559603	0.064334482	0.125335497	-
ENSG00000265366	10.36325155	3.210006989	1.684677316	0.064359516	0.125374865	GLUD1P2
ENSG00000163132	121.6538063	68.15467708	0.837087535	0.064391145	0.125427076	MSX1
ENSG00000226747	7.767890705	1.00940975	2.90213689	0.064420112	0.125474093	FSIP2-AS2
ENSG00000272417	6.34327069	1.110911105	2.539537428	0.064452496	0.125527759	AC034229.4
ENSG00000133627	101.8725722	147.1024967	-0.530371239	0.064493145	0.125597511	ACTR3B
ENSG00000196367	1060.181766	1486.416509	-0.487366462	0.064504979	0.125611141	TRRAP
ENSG00000106605	555.9881127	796.4542713	-0.51891651	0.064521909	0.125634694	BLVRA
ENSG00000178718	299.1900947	211.4024684	0.501616561	0.064532106	0.125645133	RPP25
ENSG00000260417	0	3.179325408	-4.252667541	0.064537716	0.12564664	AC092127.1
ENSG00000168894	843.2509225	1174.681917	-0.478201948	0.064547275	0.125655834	RNF181
ENSG00000246089	70.29963395	101.3067351	-0.527166705	0.064560339	0.125671849	AC016065.1
ENSG00000178342	12.45619678	38.20154688	-1.619979735	0.064586033	0.125701211	KCNG2
ENSG00000240535	5.537869779	0	4.802110773	0.06458645	0.125701211	AC034238.1
ENSG00000230499	2.098031101	9.822411193	-2.219555574	0.064589938	0.125701211	AC108463.2
ENSG00000100324	857.8134162	656.496832	0.385960362	0.064663425	0.125834803	TAB1
ENSG00000152795	7676.938146	5671.980309	0.436684706	0.064698937	0.125894479	HNRNPDL
ENSG00000259363	81.32529848	47.51002994	0.775407828	0.064704506	0.125895886	AC090825.1
ENSG00000217648	22.09574875	10.3029608	1.09525067	0.064731993	0.125939935	AL136116.3
ENSG00000185839	27.63988678	46.07326141	-0.737526795	0.064760102	0.125985189	AL035411.1
ENSG00000254013	1.21705491	5.757686631	-2.245200287	0.064770534	0.125996049	MAP2K1P1
ENSG00000189366	13.84188242	4.351599676	1.676155162	0.064827791	0.126097988	ALG1L
ENSG00000149609	2.094270155	10.35242721	-2.295029251	0.064944575	0.126315691	C20orf144
ENSG00000232878	7.634647232	1.069617038	2.828665595	0.064958598	0.126333509	DPYD-AS1
ENSG00000238290	0.600343103	4.778958462	-2.979195706	0.064964164	0.126334876	AL034417.2
ENSG00000261447	5.186676005	0.693008929	2.869567349	0.064974906	0.126346309	AL162412.1

ENSG00000176399	7.937988541	1.396630344	2.48727455	0.065007227	0.12639955	DMRTA1
ENSG00000100253	0	3.352802412	-4.320839963	0.06501728	0.12639955	MIOX
ENSG00000262668	0	3.352802412	-4.320839963	0.06501728	0.12639955	AJ003147.2
ENSG00000160808	9.803167748	1.456837632	2.756708139	0.065021746	0.12639955	MYL3
ENSG00000214189	4.528379975	12.84948457	-1.504938001	0.065036324	0.126418429	ZNF788P
ENSG00000142655	357.8283063	504.6058862	-0.495810338	0.065049746	0.12643506	PEX14
ENSG00000180332	63.19827318	13.602444	2.221174866	0.0650621	0.126449613	KCTD4
ENSG00000183458	24.83911212	55.20980853	-1.149697525	0.065085847	0.126486304	AC138932.1
ENSG00000279166	9.218979887	1.518200796	2.637053516	0.065126737	0.126556301	AC009951.1
ENSG00000197948	450.2654589	651.7108915	-0.533021086	0.06515026	0.1265849	FCHSD1
ENSG00000174227	917.2117107	1218.766059	-0.410140638	0.065151198	0.1265849	PIGG
ENSG00000157764	563.1897382	374.7169524	0.588265363	0.065186623	0.126644257	BRAF
ENSG00000235189	3.298717306	0	4.057063864	0.065203503	0.126658109	AL034405.1
ENSG00000239504	3.298717306	0	4.057063864	0.065203503	0.126658109	RN7SL583P
ENSG00000197272	5.541630725	0	4.803055492	0.06522134	0.126683285	IL27
ENSG00000131759	602.9738461	861.6387178	-0.514722355	0.065227583	0.126685411	RARA
ENSG00000182508	3.297463658	0	4.056575521	0.065237062	0.126685411	LHFPL1
ENSG00000225885	3.297463658	0	4.056575521	0.065237062	0.126685411	AC023590.1
ENSG00000250220	17.62908265	6.765940506	1.376421448	0.065278394	0.126756199	AC053527.1
ENSG00000229205	5.53661613	0	4.801803559	0.065310119	0.126808326	LINC00200
ENSG00000230836	0.308355904	6.103484764	-4.223608903	0.065330124	0.126837688	LINC01293
novel.578	14.3291117	4.769501852	1.597574775	0.06534309	0.126847172	-
ENSG00000198586	659.3479439	501.9068876	0.393522738	0.065344773	0.126847172	TLK1
ENSG00000215595	3.856222538	0	4.283209134	0.065365551	0.126878025	C20orf202
ENSG00000253598	9.904998732	2.598430319	1.953309479	0.065411458	0.126957649	SLC10A5
ENSG00000101365	1084.813895	1418.725995	-0.38726489	0.065434452	0.126992792	IDH3B
novel.2	15.26309749	3.557089392	2.096897689	0.065443607	0.127001073	-
ENSG00000169760	147.3355304	75.86460143	0.955229983	0.065449032	0.127002113	NLGN1
ENSG00000196511	25.81113465	10.86378679	1.238155499	0.065457514	0.127009086	TPK1
ENSG00000164970	635.9902383	1021.410133	-0.683170374	0.065480099	0.127043421	FAM219A
ENSG00000237863	5.779497215	0	4.864411982	0.065511784	0.127095405	AL035425.1
ENSG00000240253	5.842393466	0.387220594	3.917033898	0.06557821	0.127214775	FAR2P3
ENSG00000167778	1364.148218	1892.810695	-0.472428096	0.065587209	0.127221506	SPRYD3
novel.192	26.09810726	12.03593267	1.12343274	0.065591474	0.127221506	-
ENSG00000271551	7.272099347	1.823989131	2.007797847	0.065602698	0.127230617	AL355297.4
ENSG00000234380	46.00925117	16.67337998	1.461242637	0.065605965	0.127230617	LINC01426
ENSG00000237352	15.19873378	6.164976321	1.30682244	0.065616131	0.127240835	LINC01358
ENSG00000236390	0	3.139187216	-4.236368356	0.065716713	0.127419578	AC092800.1
ENSG00000128394	188.3756008	133.0726468	0.501643037	0.065718115	0.127419578	APOBEC3F
ENSG00000253948	49.90287006	31.02086785	0.68852401	0.06577984	0.127529737	AC104986.2
ENSG00000213453	0.307102255	5.593537843	-4.09922259	0.065790621	0.127540842	FTH1P3
ENSG00000185515	328.6633216	448.434627	-0.448689893	0.065796654	0.127540842	BRCC3
ENSG00000248757	3.675126598	0	4.210140086	0.065805203	0.127540842	LINC02115
ENSG00000214772	3.675126598	0	4.210140086	0.065805203	0.127540842	AC010168.1
ENSG00000111802	606.2618354	794.9509897	-0.391200362	0.065847196	0.12761271	TDP2
ENSG00000113161	563.0743309	843.1100369	-0.582507532	0.065872531	0.127652288	HMGCR
ENSG00000110492	2089.08093	1298.008837	0.68638877	0.065883124	0.127656923	MDK
ENSG00000188996	3.957013336	0.356539013	3.35519748	0.065884749	0.127656923	HUS1B
ENSG00000243155	17.83790141	8.254615595	1.112109533	0.065893549	0.127664452	AL162431.2
ENSG00000254337	0	24.43122968	-7.191993286	0.065905846	0.127678755	AC083967.1
ENSG00000125449	235.0313238	138.1923603	0.764253692	0.065986053	0.127824608	ARMC7

ENSG00000159461	1982.37029	1497.9052	0.404083983	0.065996258	0.127828954	AMFR
ENSG00000166484	454.0611503	676.1262092	-0.574021363	0.065998137	0.127828954	MAPK7
ENSG00000237437	9.613011533	2.853467977	1.750007527	0.06601322	0.127848637	ASS1P12
ENSG00000285812	0.879722543	8.130604999	-3.187076295	0.066058558	0.127926907	AL390719.3
ENSG00000268670	2.3900183	12.704249	-2.403911782	0.066088408	0.127975176	AC016586.2
ENSG00000259838	23.41957797	48.18870197	-1.03657067	0.066137052	0.128059827	ELOCP2
ENSG00000130997	66.51053933	32.35973105	1.038531107	0.066196287	0.12816497	POLN
ENSG00000277831	7.265831103	1.487519214	2.313971859	0.066214801	0.128187433	AL138960.1
ENSG00000198369	1183.092197	814.6519095	0.538099514	0.066217757	0.128187433	SPRED2
ENSG00000137673	0.894837599	5.451898296	-2.591843411	0.066224278	0.128190505	MMP7
ENSG00000132541	261.6826338	386.42383	-0.562660646	0.066244962	0.128220992	RIDA
ENSG00000215790	284.4366225	197.2473708	0.529031063	0.066250148	0.128221476	SLC35E2A
ENSG00000286417	3.653672027	0	4.202384263	0.066345195	0.128395867	AL133334.1
ENSG00000274210	4.53339457	0.356539013	3.55280587	0.066354944	0.128405169	RF00003
ENSG00000005469	307.4093296	227.5193888	0.433163815	0.066377238	0.128438744	CROT
ENSG00000255277	0.308355904	3.647978261	-3.482515313	0.066404758	0.128482427	ABCC6P2
ENSG00000185834	117.2498946	174.2663946	-0.571886239	0.066486333	0.128630681	RPL12P4
ENSG00000147251	262.8659111	398.8785167	-0.601728379	0.066528184	0.128702067	DOCK11
ENSG00000079387	257.3046211	353.5723417	-0.459258503	0.066537192	0.128709909	SENP1
ENSG00000175106	81.30828904	119.7305875	-0.556349985	0.066566842	0.128749573	TVP23C
ENSG00000270919	3.644896486	0	4.199219402	0.066567607	0.128749573	AC108451.2
ENSG00000102837	28.5730462	0	7.170137094	0.06665576	0.128910474	OLFM4
ENSG00000285955	7.551478787	1.4981317	2.364494742	0.066663866	0.128916554	BX842242.1
ENSG00000126709	6984.384525	12117.01958	-0.794830844	0.066700864	0.128977048	IFI6
ENSG00000158483	43.05342282	72.57162112	-0.753567616	0.066705076	0.128977048	FAM86C1
ENSG00000120254	754.6629138	1123.807564	-0.574807285	0.066722408	0.129000958	MTHFD1L
ENSG00000115540	404.7921284	543.0902798	-0.424213732	0.066740338	0.129026023	MOB4
ENSG00000187726	5.71771207	0.713078025	2.997758212	0.066774604	0.129082663	DNAJB13
ENSG00000101004	233.8995171	326.4274857	-0.480709348	0.066788309	0.129099551	NINL
ENSG00000248208	10.0272441	1.844058227	2.457122509	0.066815103	0.129141735	WDR45P1
ENSG00000142166	1729.379958	1326.987917	0.381860495	0.066860644	0.129212258	IFNAR1
ENSG00000173918	1046.761927	2909.985677	-1.475061794	0.066861537	0.129212258	C1QTNF1
ENSG00000156374	213.5235673	293.0173548	-0.45617569	0.06690333	0.129283408	PCGF6
ENSG00000224536	25.05713369	10.987669	1.184768372	0.066917329	0.129300842	AC096677.1
ENSG00000176055	207.9188544	142.1032446	0.547508925	0.067002197	0.129454837	MBLAC2
novel.16	0.921306765	6.512058724	-2.829274343	0.067006991	0.129454837	-
ENSG00000225871	1.218308559	6.501446238	-2.418526324	0.067020891	0.129472063	AC245100.2
ENSG00000185246	946.893658	646.9277419	0.549803385	0.067037414	0.129494354	PRPF39
ENSG00000273045	8.163176	2.089639274	1.944467638	0.067061048	0.129530375	C2orf15
ENSG00000108773	1619.96829	1219.973126	0.409261381	0.067076621	0.129550825	KAT2A
ENSG00000116209	4252.981106	3221.882918	0.400549907	0.067144396	0.129672084	TMEM59
ENSG00000268926	28.31232895	0	7.156904667	0.067161565	0.129695602	AL354861.3
ENSG00000224712	7.581780171	0.774441189	3.342540547	0.067262941	0.129881714	NPIPA3
ENSG00000139926	3160.28142	1924.51351	0.715702533	0.067313259	0.129969216	FRMD6
ENSG00000239300	14.3187265	4.351599676	1.725956592	0.067323074	0.129978508	AC080162.1
ENSG00000274307	5.185422356	0.693008929	2.869715739	0.067339782	0.130001105	AC023449.2
ENSG00000239246	1.801029308	7.979508841	-2.139553754	0.067372712	0.130055014	AC008026.1
ENSG00000230869	16.96290872	4.769501852	1.841608608	0.067456678	0.130207426	AGAP10P
ENSG00000268798	4.863205045	14.3263913	-1.56120776	0.067468026	0.130219655	AC027307.3
ENSG00000054523	1629.87463	1141.064613	0.514477499	0.067474582	0.130222635	KIF1B
ENSG00000214282	0	4.849778236	-4.856163815	0.067497447	0.130247414	KRT8P14

ENSG00000147571	0	4.849778236	-4.856163815	0.067497447	0.130247414	CRH
ENSG00000100068	61.25895797	108.6894365	-0.828752583	0.067515899	0.130273344	LRP5L
ENSG00000271828	10.24358475	2.455634897	2.047311192	0.067524222	0.130275186	AC008937.3
ENSG00000139714	9.114712877	2.598430319	1.832864808	0.067528621	0.130275186	MORN3
ENSG00000179636	3.940644631	0.356539013	3.350117786	0.067531897	0.130275186	TPPP2
ENSG00000177144	7.298568513	1.753169357	2.038564557	0.067557412	0.130314732	NUDT4B
ENSG00000177628	55.84108317	17.58231585	1.668098719	0.067579993	0.130348612	GBA
ENSG00000140993	40.37059095	20.68735386	0.961844975	0.067586877	0.130352212	TIGD7
ENSG00000188585	5.466055445	0.672939833	2.965482184	0.067601788	0.130371293	CLEC20A
ENSG00000106299	1308.228726	973.3386127	0.426595887	0.067611676	0.130380684	WASL
ENSG00000147117	9.079396899	1.733100261	2.368538695	0.067640819	0.130427202	ZNF157
ENSG00000224905	23.81006213	9.498993907	1.316450991	0.067706352	0.130543876	AP001347.1
ENSG00000279030	0	4.880459818	-4.864809708	0.067774465	0.130665506	AC007336.3
ENSG00000161960	128.7268751	218.337776	-0.761608317	0.06778682	0.130679629	EIF4A1
ENSG00000183617	502.1599258	675.9194005	-0.428827834	0.06785058	0.130780276	MRPL54
ENSG00000177721	61.85213484	25.72738615	1.259701968	0.067851596	0.130780276	ANXA2R
ENSG00000276851	1.846374476	8.437549209	-2.196754264	0.067854129	0.130780276	AC002401.4
ENSG00000232692	0.293240848	5.483864147	-4.079535432	0.067884557	0.130829217	AP001596.1
ENSG00000018610	391.3391914	283.1687912	0.467143337	0.067915597	0.13087933	CXorf56
ENSG00000281706	3.04831433	9.334845119	-1.618935996	0.067940381	0.130912774	AL009179.1
ENSG00000274373	6.144552397	0	4.952329127	0.067944343	0.130912774	AC148476.1
ENSG00000269867	23.27129071	50.86385984	-1.126275837	0.067956786	0.130912774	AC010326.3
ENSG00000234036	0	3.250145181	-4.280919575	0.067958845	0.130912774	TXNP6
ENSG00000231535	27.90623118	0	7.13617668	0.067960253	0.130912774	LINC00278
ENSG00000262075	10.29394451	22.95979836	-1.153363558	0.067963184	0.130912774	DKFZP434A062
ENSG00000260439	12.25873214	1.782695063	2.776788207	0.068007283	0.1309845	LMF1-AS1
ENSG00000266368	3.912921816	0.356539013	3.341451984	0.068010504	0.1309845	AC005410.2
ENSG00000228768	0.308355904	3.872334337	-3.576885794	0.068021256	0.130995497	AC003101.1
ENSG00000172752	0	23.51677154	-7.136320178	0.068037955	0.131017944	COL6A5
ENSG00000275832	187.056572	333.6326815	-0.834999037	0.068095138	0.131118342	ARHGAP23
ENSG00000259772	5.384140649	0	4.763163508	0.068102297	0.131122408	AC012236.1
ENSG00000241544	0	4.819096654	-4.847498303	0.068153598	0.131211459	LINC02029
ENSG00000259291	106.7022566	56.89904619	0.909763538	0.068171142	0.13123551	ZNF710-AS1
ENSG00000152056	73.93736455	31.23016351	1.238118508	0.068218149	0.131316273	AP1S3
ENSG00000164898	13.53220159	4.993857928	1.436315177	0.068292538	0.131449729	FMC1
ENSG00000171126	5.83870379	0	4.878597903	0.068311797	0.131477058	KCNG3
ENSG00000261216	0	2.904218654	-4.117182652	0.068347964	0.131527179	AC007216.2
ENSG00000123407	0	2.904218654	-4.117182652	0.068347964	0.131527179	HOXC12
ENSG00000172943	509.810772	393.7502925	0.373149496	0.068359554	0.131536715	PHF8
ENSG00000269971	3.575589449	0	4.173909015	0.068368107	0.131536715	AL020997.2
ENSG00000185031	3.575589449	0	4.173909015	0.068368107	0.131536715	SLC2A3P2
ENSG00000169727	1872.600091	2444.501217	-0.38448203	0.068383338	0.131556276	GPS1
ENSG00000273061	16.31757646	5.12488499	1.679546104	0.06840102	0.131580549	CDC37L1-DT
ENSG00000172156	5.534108833	0	4.801200931	0.068443294	0.131652123	CCL11
ENSG00000180543	295.886746	557.4219362	-0.914115274	0.06846637	0.131686761	TSPYL5
ENSG00000196458	339.0711073	510.5907754	-0.590068866	0.068482542	0.131708114	ZNF605
ENSG00000232888	5.133737672	14.42904853	-1.488533653	0.068488495	0.131709814	RPS11P5
ENSG00000276900	35.65065857	17.05700455	1.067524189	0.068504842	0.1317315	AC023157.3
ENSG00000135912	626.8727924	394.1286172	0.669569611	0.068557135	0.1318223	TTLL4
ENSG00000134086	641.7453456	927.1035964	-0.530346114	0.068591428	0.13187278	VHL
ENSG00000235448	4.488049402	0.387220594	3.539898506	0.068593539	0.13187278	LURAP1L-AS1

ENSG00000110777	12.33423615	0.336469917	4.996084524	0.068605613	0.131886233	POU2AF1
ENSG00000216775	125.4793722	235.2344974	-0.907384769	0.068667609	0.131992918	AL109918.1
ENSG00000185630	722.5257734	496.0681883	0.542758243	0.06867127	0.131992918	PBX1
ENSG00000102763	326.4268187	464.5750028	-0.509840738	0.068770752	0.132174354	VWA8
ENSG00000115718	7.309922623	1.487519214	2.32094032	0.068828413	0.13227539	PROC
ENSG00000259075	7.857327394	2.140389951	1.872380818	0.068840388	0.132288618	POC1B-GALNT4
ENSG00000144857	267.4340063	81.68498267	1.70995549	0.068865408	0.132326912	BOC
ENSG00000280537	1.54052587	7.876851611	-2.36507514	0.068872599	0.132330941	AC068946.1
ENSG00000273820	109.4904238	62.27071528	0.815072644	0.068902579	0.132378754	USP27X
ENSG00000175894	1.215801261	6.266477676	-2.361458571	0.068919427	0.132396721	TSPEAR
ENSG00000267565	12.31138574	3.852265241	1.663646523	0.068922122	0.132396721	AC011477.3
ENSG00000144224	2735.363994	2198.636881	0.315121656	0.068928264	0.13239873	UBXN4
ENSG00000148943	656.3411584	478.6280856	0.455068653	0.069085003	0.132689987	LIN7C
ENSG00000039319	833.2380699	605.7189333	0.459909399	0.069104242	0.132717128	ZFYVE16
ENSG00000228300	489.6789408	689.4377281	-0.493909539	0.069130272	0.132757307	C19orf24
ENSG00000268049	27.25685324	11.48469168	1.248466536	0.069193349	0.132867065	AC012313.2
ENSG00000228519	4.528379975	12.10803671	-1.419756673	0.069197654	0.132867065	AC097263.1
ENSG00000007168	2361.112495	3019.741542	-0.354908882	0.069236924	0.132932644	PAFAH1B1
ENSG00000185760	8.273995988	24.32715978	-1.561603486	0.069260068	0.132967253	KCNQ5
novel.1003	6.947374739	16.89889193	-1.286687313	0.069272166	0.132980651	-
ENSG00000280083	0.616711807	5.553399651	-3.178790327	0.069279397	0.132984708	AC079777.1
ENSG00000184208	585.2383302	424.8220051	0.461444834	0.069295232	0.133005277	C22orf46
ENSG00000184984	13.9527024	5.289033778	1.388096992	0.069372885	0.133144487	CHRM5
ENSG00000188372	43.66533349	86.16516926	-0.982989747	0.069394248	0.133175651	ZP3
ENSG00000011275	896.618222	1126.234324	-0.328755322	0.069423411	0.13320844	RNF216
ENSG00000150459	2664.684075	2096.759389	0.345744252	0.069426639	0.13320844	SAP18
ENSG00000117281	20.71271296	8.529722348	1.274092541	0.069426715	0.13320844	CD160
ENSG00000232187	183.6632185	359.9779594	-0.970219784	0.069439176	0.133222511	FTH1P7
ENSG00000224051	468.0490821	617.5136991	-0.399643537	0.069471877	0.133274419	CPTP
ENSG00000139644	9138.472343	11516.82838	-0.333720323	0.069476492	0.133274419	TMBIM6
ENSG00000241553	1718.930218	2326.042993	-0.436380449	0.069516595	0.133341503	ARPC4
ENSG00000213640	8.023308277	0.723690511	3.478182314	0.069525531	0.133348798	EEF1DP4
ENSG00000167302	278.5278288	169.895659	0.714246262	0.069583703	0.13345052	TEPSIN
ENSG00000166902	758.0033297	985.9815251	-0.379300334	0.069638009	0.133544812	MRPL16
ENSG00000109265	91.49588617	51.06840361	0.839638738	0.069669764	0.133595848	KIAA1211
ENSG00000048392	1257.575957	902.0670827	0.479626711	0.069690759	0.133626245	RRM2B
ENSG00000184752	716.0252275	965.3187008	-0.431286777	0.069788022	0.133802862	NDUFA12
ENSG00000105677	1160.067395	1510.480944	-0.380954306	0.069809058	0.133833319	TMEM147
ENSG00000233360	0.586481695	5.02453951	-3.073007656	0.069843963	0.133890357	Z83844.2
ENSG00000186951	762.6224873	441.2160146	0.790030019	0.069865669	0.133922086	PPARA
ENSG00000196724	105.1243347	72.57850919	0.535281754	0.06987135	0.133923094	ZNF418
novel.1002	24.27311608	41.21158642	-0.762285401	0.069908677	0.133984755	-
ENSG00000166192	44.09697495	69.179708	-0.650518733	0.069928996	0.134013811	SENP8
ENSG00000225880	52.08378487	26.44362783	0.984710668	0.069980501	0.134102626	LINC00115
ENSG00000231154	16.79442054	6.512058724	1.37304337	0.069992424	0.134115581	MORF4L2-AS1
novel.466	670.266209	485.6800496	0.464519641	0.069998894	0.134118088	-
ENSG00000053328	15.09690279	1.722487775	3.115262366	0.07010169	0.134305141	METTL24
ENSG00000214046	864.4597275	626.8125285	0.463259871	0.070185651	0.134456084	SMIM7
ENSG00000215154	47.79069282	87.80664432	-0.875323053	0.070265665	0.134599442	AC141586.1
ENSG00000150776	506.7182515	643.5120821	-0.345091049	0.070350378	0.134751782	NKAPD1
ENSG00000187266	327.0466232	187.8385766	0.801351267	0.070410946	0.134857854	EPOR

ENSG00000031691	119.8274906	171.7732713	-0.520341242	0.070437441	0.134898655	CENPQ
ENSG00000164082	11.19637527	3.668047357	1.623365239	0.070443505	0.134900324	GRM2
novel.1102	3.359177531	0	4.080654925	0.0705023	0.134993017	-
ENSG00000089169	3.359177531	0	4.080654925	0.0705023	0.134993017	RPH3A
ENSG00000279149	7.657355452	1.41669944	2.423435391	0.07058832	0.135147575	AL356750.1
ENSG00000119599	128.7046933	208.3022292	-0.695898029	0.070593424	0.135147575	DCAF4
ENSG00000074266	352.5042977	463.3144898	-0.394827427	0.070686373	0.135307391	EED
ENSG00000267322	12.42478429	3.740023006	1.75425926	0.070687318	0.135307391	SNHG22
ENSG00000039650	626.474841	852.0109739	-0.443570959	0.070696309	0.135314631	PNKP
ENSG00000161057	1638.584237	2199.053693	-0.424575323	0.070777825	0.135452943	PSMC2
ENSG00000264924	4.45656564	0.387220594	3.530842055	0.070778998	0.135452943	AC090772.1
ENSG00000187944	19.05746361	7.327793979	1.388691001	0.07079647	0.1354764	C2orf66
ENSG00000277687	31.4630159	13.97100816	1.171987512	0.070829699	0.135530005	AL139407.1
ENSG00000253352	2838.053598	2108.381909	0.42880161	0.070837985	0.135535878	TUG1
ENSG00000166068	1759.152521	1248.815704	0.494419997	0.070861215	0.135570341	SPRED1
ENSG00000141428	164.6243458	223.3397251	-0.440486044	0.070894391	0.135623826	C18orf21
ENSG00000176994	11.23921314	22.81713133	-1.025328557	0.070924921	0.135672241	SMCR8
ENSG00000110900	184.0754409	49.88248159	1.881871863	0.070952731	0.135715445	TSPAN11
ENSG00000185222	1580.131422	2141.583765	-0.438672513	0.071009336	0.13581372	TCEAL9
ENSG00000120337	16.19400617	3.822739535	2.071527298	0.07102792	0.135839264	TNFSF18
ENSG00000139797	0	3.149799701	-4.240285351	0.071056085	0.135883125	RNF113B
ENSG00000185585	3137.534939	1530.222017	1.035746715	0.071071793	0.135903162	OLFML2A
ENSG00000234883	31.34893316	61.76174866	-0.977095498	0.071085152	0.135918702	MIR155HG
novel.910	45.101592	7.358603955	2.612127469	0.071117529	0.135969637	-
ENSG00000143603	21.91583519	229.8608768	-3.390715693	0.071122257	0.135969637	KCNN3
ENSG00000171204	950.6643588	743.6806109	0.354128715	0.071178267	0.136066702	TMEM126B
ENSG00000118495	627.5188156	1139.301207	-0.860630132	0.071393829	0.136468736	PLAGL1
ENSG00000227253	10.49022678	2.261960402	2.240795361	0.071424299	0.136516935	AL158834.2
ENSG00000258818	10.57590252	2.587817834	2.047294554	0.071455756	0.136567015	RNASE4
ENSG00000198522	411.7674825	570.2220491	-0.470116679	0.071465738	0.136576046	GPN1
ENSG00000172954	107.257824	155.4340495	-0.53570486	0.071509286	0.136629671	LCLAT1
ENSG00000185761	53.57556028	101.0680421	-0.915602757	0.071512589	0.136629671	ADAMTSL5
ENSG00000203546	21.33945396	7.634866584	1.468971216	0.07151647	0.136629671	AL139353.1
novel.728	21.16964086	4.788415073	2.15078369	0.071517173	0.136629671	-
novel.109	6.466413702	0	5.027958	0.071526745	0.136629671	-
ENSG00000260784	6.466413702	0	5.027958	0.071526745	0.136629671	AC026150.2
ENSG00000279089	27.56151946	10.65937142	1.379591873	0.07153061	0.136629671	AC005839.1
ENSG00000106536	26.20272992	0	7.045179571	0.071559784	0.136675347	POU6F2
ENSG00000125821	345.1357102	470.68178	-0.447444714	0.071583714	0.136711002	DTD1
ENSG00000123104	612.8213595	376.3728116	0.703563096	0.071610801	0.13675268	ITPR2
ENSG00000286912	5.759296292	1.029478846	2.445669951	0.071625157	0.136770044	AC006008.1
ENSG00000183340	319.4963821	422.34086	-0.402968097	0.071682005	0.13686497	JRKL
ENSG00000182376	0.586481695	7.999706332	-3.754038299	0.071686766	0.13686497	AC138028.1
ENSG00000265096	0.925067711	8.099923417	-3.137475413	0.071695529	0.13686497	C1QTNF1-AS1
ENSG00000282390	26.14130113	0	7.041804889	0.071696233	0.13686497	AL392083.1
ENSG00000122035	181.305252	67.36432357	1.426764773	0.071701209	0.13686497	RASL11A
ENSG00000250182	81.43952341	126.5353819	-0.634968358	0.071786368	0.137017456	EEF1A1P13
ENSG00000168802	7.832111876	19.39222487	-1.305928324	0.071797996	0.137029585	CHTF8
ENSG00000230647	0	4.941822981	-4.882104734	0.071830429	0.137081415	AC022816.1
ENSG00000243547	107.3253791	160.9023397	-0.583691731	0.071857841	0.137123656	HNRNPKP4
ENSG00000100373	5.353910537	0	4.755357664	0.071887729	0.137170401	UPK3A

ENSG00000224287	0.615458159	4.901813183	-3.012568228	0.071892897	0.137170401	MSL3P1
ENSG00000162755	41.25282079	22.52195548	0.871246874	0.071939842	0.137249893	KLHDC9
ENSG00000079263	84.4050253	26.49078249	1.675183394	0.071970316	0.137297949	SP140
ENSG00000182952	1128.562395	1486.017269	-0.397076021	0.071995116	0.137335177	HMGN4
ENSG00000254317	5.71771207	0.774441189	2.946786091	0.072015723	0.137364401	AC022973.4
ENSG00000205085	104.9909212	48.31656538	1.119181266	0.072089759	0.137495524	FAM71F2
novel.1049	6.474220329	0	5.027653175	0.072098881	0.137502829	-
ENSG00000258057	22.50991005	10.25221012	1.126106562	0.072127187	0.137545055	BCDIN3D-AS1
ENSG00000177294	9.512220735	1.722487775	2.444227833	0.072135282	0.137545055	FBXO39
ENSG00000273951	4.851850935	0.743759607	2.737673112	0.072136905	0.137545055	AL031667.3
ENSG00000248275	232.0204924	133.3641454	0.799207573	0.072148204	0.137556504	TRIM52-AS1
ENSG00000198346	99.8361837	67.41104586	0.567484918	0.072207348	0.137659165	ZNF813
ENSG00000272650	11.24297409	3.944309986	1.509839638	0.072222053	0.137677097	AC110792.3
ENSG00000287564	6.099135957	1.060160428	2.510977206	0.07228658	0.137789995	AL359546.1
ENSG00000268520	2.094270155	9.75390317	-2.212367999	0.072304288	0.137813638	AC008750.5
ENSG00000135519	23.59748243	8.16257085	1.52615337	0.072359	0.137907804	KCNH3
ENSG00000285527	5.232021173	0	4.720099465	0.072414808	0.138004043	AC136285.3
ENSG00000160221	446.2633142	302.6035604	0.560700336	0.072467863	0.138095024	GATD3A
ENSG00000250506	19.42635101	8.275840566	1.231998675	0.072495743	0.138138019	CDK3
ENSG00000232702	4.873305506	16.85502932	-1.789287822	0.072517061	0.138168506	AL158050.1
novel.673	3.67387295	0	4.209770751	0.072532511	0.138187809	-
ENSG00000131652	423.4314003	609.1153422	-0.524079409	0.072558183	0.138226584	THOC6
ENSG00000258645	4.585079253	13.05364315	-1.511540789	0.072577169	0.138251445	HSPE1P2
ENSG00000173210	2506.238165	6177.523466	-1.301475668	0.072581876	0.138251445	ABLIM3
ENSG00000120159	250.5892437	347.7429834	-0.473502237	0.072594089	0.138264572	CAAP1
ENSG00000157954	1627.31857	2173.057288	-0.417069319	0.072638392	0.138338812	WIPI2
ENSG00000178464	1320.12575	1762.420728	-0.416873308	0.072660377	0.138370538	RPL10P16
ENSG00000224846	0.600343103	5.158006716	-3.104984809	0.072674345	0.138386994	AL133351.1
ENSG00000138279	2229.290476	1550.254408	0.52386432	0.072705289	0.138435773	ANXA7
ENSG00000205853	9.640734348	2.822786395	1.76172943	0.072711736	0.138437903	RFPL3S
ENSG00000156162	769.7520107	543.5157117	0.50159162	0.072722554	0.138444477	DPY19L4
ENSG00000148735	3.28360225	0	4.050999631	0.072725847	0.138444477	PLEKHS1
ENSG00000114200	110.0466605	26.53263731	2.049564245	0.072741126	0.138463419	BCHE
ENSG00000083814	175.2211609	98.93412066	0.822728244	0.072816431	0.138596608	ZNF671
ENSG00000214650	14.38420132	5.044608606	1.515956468	0.072833337	0.138616248	AC073592.1
ENSG00000243056	19.93342521	8.926399553	1.156680791	0.072841576	0.138616248	EIF4EBP3
ENSG00000196782	297.1828965	183.4624127	0.697151837	0.072842756	0.138616248	MAML3
ENSG00000120210	3.970874743	0.336469917	3.359649189	0.072877773	0.138672728	INSL6
ENSG00000108828	4400.809468	6185.597836	-0.491095041	0.072889638	0.138685147	VAT1
ENSG00000272983	47.82697771	17.33198291	1.46989417	0.072915245	0.138723709	AL117339.4
ENSG00000218283	890.2186195	1260.360372	-0.501690914	0.073009861	0.138880863	MORF4L1P1
ENSG00000149452	5.229513876	0	4.719442062	0.073013883	0.138880863	SLC22A8
ENSG00000226767	5.229513876	0	4.719442062	0.073013883	0.138880863	AC114501.1
ENSG00000242588	70.43356161	34.0844834	1.04967389	0.073031364	0.138903944	AC108010.1
ENSG00000262576	39.79922396	13.30739654	1.585621149	0.073037528	0.138905499	PCDHGA4
ENSG00000006015	1294.420406	940.2567244	0.460983292	0.073052385	0.138923584	REX1BD
ENSG00000286882	3.034452922	0	3.934800073	0.07315216	0.139082786	AC116428.1
ENSG00000279137	3.034452922	0	3.934800073	0.07315216	0.139082786	AL356309.3
ENSG00000167880	3.034452922	0	3.934800073	0.07315216	0.139082786	EVPL
ENSG00000112343	671.910318	873.3626969	-0.378239843	0.073162551	0.139092363	TRIM38
ENSG00000185883	55.34106015	92.17746119	-0.73651771	0.073171765	0.139099702	ATP6V0C

ENSG00000166471	532.0113298	776.8163932	-0.545651018	0.073214185	0.139170159	TMEM41B
ENSG00000164061	30.48536586	12.19905398	1.312470147	0.07325124	0.13923041	BSN
ENSG00000287875	10.15694009	1.130980201	3.190727058	0.073264924	0.139246232	AC068193.1
ENSG00000233196	5.435825332	1.080229524	2.334825142	0.073359284	0.139415372	AL096803.3
ENSG00000124098	1928.625528	1415.128553	0.446837795	0.073369868	0.139425288	FAM210B
ENSG00000154645	7.974486897	1.00940975	2.939883068	0.073377443	0.139429484	CHODL
ENSG00000131943	726.367895	555.9361683	0.385821942	0.073437375	0.13953316	C19orf12
ENSG00000122756	16.61604536	2.52645467	2.71779556	0.073480476	0.139604844	CNTFR
ENSG00000186834	1851.845877	1371.470197	0.432989068	0.073505953	0.139643036	HEXIM1
ENSG00000120913	1317.096099	977.6788211	0.429620048	0.073578052	0.139769786	PDLIM2
ENSG00000259932	3.019337866	0	3.928409547	0.07363971	0.139876685	AC051619.7
ENSG00000287799	7.18140901	1.386017859	2.350193303	0.073687794	0.139957788	AC005014.4
novel.817	4.266622888	12.45511911	-1.550976834	0.073724139	0.140016583	-
ENSG00000230747	5.423217574	0.713078025	2.921287331	0.073735785	0.140028465	AC021188.1
ENSG00000249859	170.7714489	266.1406704	-0.63894241	0.07380558	0.140150765	PVT1
ENSG00000188365	9.110951931	2.537067156	1.849817145	0.073887275	0.140295643	AC092171.1
ENSG00000271947	0.586481695	6.03266499	-3.333543803	0.073922102	0.140351514	AC017076.1
ENSG00000181896	197.108775	136.1162404	0.533047381	0.073954533	0.14040283	ZNF101
ENSG00000283118	3.915429114	0.336469917	3.342159252	0.073967317	0.14041684	AC009951.2
ENSG00000187091	588.021343	362.6814996	0.697605122	0.074033037	0.140531331	PLCD1
ENSG00000259129	5.234528471	0	4.72077467	0.074049146	0.14053877	LINC00648
ENSG00000040933	682.2778721	515.2928439	0.405507185	0.07405307	0.14053877	INPP4A
ENSG00000287575	5.7731577	1.029478846	2.449112113	0.074053184	0.14053877	AL390755.3
ENSG00000199906	5.454701335	1.080229524	2.338781415	0.074156107	0.140723819	RNU5B-2P
ENSG00000274400	6.285317763	0.774441189	3.07894704	0.074167952	0.140736017	AC015967.1
novel.408	34.41014033	91.56631688	-1.413335539	0.074198272	0.1407824	-
ENSG00000175746	0.616711807	8.415168363	-3.778961916	0.074203233	0.1407824	C15orf54
ENSG00000010404	230.1259648	156.2173865	0.560527341	0.074212107	0.140788955	IDS
ENSG00000133105	0	2.833398881	-4.086108939	0.074283619	0.140904046	RXFP2
ENSG00000180210	0	2.833398881	-4.086108939	0.074283619	0.140904046	F2
ENSG00000111666	1008.850356	736.5299115	0.454340866	0.074319247	0.140961335	CHPT1
ENSG00000225408	3.37178529	0	4.085621953	0.074331394	0.140974082	AL136980.1
ENSG00000131669	1255.925755	829.1128648	0.599494999	0.074372355	0.141041471	NINJ1
ENSG00000237604	7.065859161	0.356539013	4.191932444	0.074391	0.14104951	AP001056.1
ENSG00000105402	726.2562194	969.2858924	-0.416476394	0.074392188	0.14104951	NAPA
ENSG00000280294	3.369277992	0	4.084649068	0.074407055	0.14104951	AC011008.2
ENSG00000271880	3.369277992	0	4.084649068	0.074407055	0.14104951	AGAP11
ENSG00000268204	3.369277992	0	4.084649068	0.074407055	0.14104951	AC008763.1
ENSG00000267049	2.991615051	10.88488337	-1.854982102	0.074409168	0.14104951	AC002398.1
ENSG00000224661	24.94172604	0	6.974026521	0.07448231	0.141177857	AC010907.1
ENSG00000112041	14.68782736	4.299693123	1.769570934	0.074503874	0.141208429	TULP1
ENSG00000258101	5.209312953	17.73122866	-1.772766016	0.074537082	0.141261064	AC010173.1
ENSG00000165949	541.9107828	284.5959872	0.92840922	0.074549168	0.141273663	IFI27
ENSG00000184226	982.0687309	317.8524188	1.627167471	0.07467083	0.141493899	PCDH9
ENSG00000259033	5.121129914	0	4.690490068	0.074744108	0.141622423	AL356804.1
ENSG00000010704	305.7862757	410.9294754	-0.426891796	0.074757964	0.141627467	HFE
ENSG00000171467	669.4180421	478.7401394	0.484007551	0.074762592	0.141627467	ZNF318
ENSG00000131143	4508.351943	6008.677187	-0.414402893	0.074763123	0.141627467	COX4I1
ENSG00000236935	5.54664532	0	4.804387343	0.074808893	0.141703838	AP003774.4
ENSG00000127366	25.11222332	10.46582532	1.266756591	0.074831951	0.141737181	TAS2R5
ENSG00000254198	14.88153106	28.06589852	-0.915840027	0.0748999	0.141855541	AC113191.1

ENSG00000251002	3.315086011	0	4.063393252	0.074957903	0.141955046	AC244502.1
ENSG00000104427	484.2164647	284.9856011	0.764539894	0.074969342	0.141966361	ZC2HC1A
ENSG00000285900	5.109847075	0	4.687452024	0.074987341	0.141990095	AC079177.1
ENSG00000168135	6.437721973	0.774441189	3.109149185	0.075012946	0.142028229	KCNJ4
ENSG00000261824	323.9112213	246.8862791	0.391898435	0.075067582	0.14211638	LINC00662
ENSG00000233121	5.106014858	0	4.686410666	0.075070444	0.14211638	VN1R20P
ENSG00000232082	0.615458159	4.686913718	-2.941402354	0.0750887	0.142140583	RPS6KA2-IT1
novel.840	66.26217293	28.90902331	1.191648725	0.075167132	0.142278688	-
ENSG00000254491	5.123708482	0	4.691190182	0.075248984	0.142423242	AC022832.1
ENSG00000116095	525.2751226	405.4238256	0.373790299	0.075259964	0.142433649	PLEKHA3
ENSG00000163586	5.095985668	0	4.683703363	0.075288037	0.142466023	FABP1
ENSG00000187527	5.095985668	0	4.683703363	0.075288037	0.142466023	ATP13A5
ENSG00000170776	1642.176122	1229.264865	0.417696439	0.075359868	0.142591562	AKAP13
ENSG00000279253	46.23909757	87.40230868	-0.915696569	0.07542319	0.1427002	AL121753.2
ENSG00000124466	23.40474765	9.313491753	1.342800503	0.075428269	0.1427002	LYPD3
ENSG00000144712	230.1324903	127.1787617	0.854009903	0.075434634	0.142701851	CAND2
novel.976	0.307102255	6.397633132	-4.297833482	0.075520477	0.142853842	-
ENSG00000163885	6.36465399	1.41669944	2.156781908	0.075568012	0.142933351	CFAP100
ENSG00000267506	3.330201067	0	4.06934045	0.075603898	0.142980411	AC021683.2
ENSG00000174885	3.330201067	0	4.06934045	0.075603898	0.142980411	NLRP6
ENSG00000243697	5.505061098	0	4.793926608	0.075632636	0.143024348	AC009108.1
ENSG00000135116	9.507490874	1.783850939	2.409696727	0.075707216	0.143151593	HRK
ENSG00000288101	3.561728042	0	4.16864891	0.075710944	0.143151593	AL359987.1
ENSG00000130338	429.0088021	335.4769968	0.355002085	0.075724857	0.14316748	TULP4
ENSG00000229754	5.235782119	0	4.721120652	0.07585593	0.143404854	CXCR2P1
ENSG00000206073	0	20.53009315	-6.94044402	0.075996842	0.143660794	SERPINB4
ENSG00000035720	24.31373139	0	6.93723782	0.076031323	0.143715518	STAP1
ENSG00000204859	433.4581744	304.2319013	0.510878565	0.076044434	0.143729845	ZBTB48
ENSG00000182087	3184.750557	3945.320264	-0.308925219	0.076076329	0.143779668	TMEM259
ENSG00000278962	15.48660403	4.320918095	1.844996617	0.076109569	0.143832028	AC092645.1
ENSG00000162104	698.1832244	514.2899024	0.440724045	0.076139712	0.143876704	ADCY9
novel.714	32.66781906	85.76501129	-1.392706521	0.076149539	0.143876704	-
ENSG00000163382	823.7359337	1141.295524	-0.470674541	0.076149823	0.143876704	NAXE
ENSG00000145736	11.28928817	39.87015468	-1.815588109	0.076162731	0.143890628	GTF2H2
ENSG00000146674	510.704843	1198.669904	-1.230946054	0.07617562	0.143904513	IGFBP3
ENSG00000267009	2.697120555	9.588598507	-1.8174173	0.076216361	0.143971011	AC007780.1
ENSG00000214439	27.1690262	9.652530209	1.481122771	0.076245487	0.144015557	FAM185BP
ENSG00000145494	1043.825579	1411.222426	-0.435182659	0.07628893	0.144087139	NDUFS6
ENSG00000102606	1594.129864	1087.067906	0.552446614	0.07635236	0.144196458	ARHGEF7
ENSG00000130173	8.426186735	2.51699806	1.746109018	0.076369001	0.144217401	ANGPTL8
ENSG00000159261	1.218308559	10.50467924	-3.107202517	0.076454666	0.144358815	CLDN14
ENSG00000233569	3.04831433	0	3.940680721	0.076454998	0.144358815	AL161630.1
ENSG00000104886	762.2527575	1143.75451	-0.585716144	0.076494003	0.144421967	PLEKHJ1
ENSG00000272583	3.299970955	0	4.057413414	0.076543435	0.144483797	AL592494.2
ENSG00000257210	3.299970955	0	4.057413414	0.076543435	0.144483797	NACAP8
ENSG00000255883	3.299970955	0	4.057413414	0.076543435	0.144483797	FUNDC2P1
ENSG00000167311	12.91319595	2.598430319	2.326095953	0.076573672	0.144530371	ART5
ENSG00000181192	479.7288976	652.375693	-0.443015976	0.076605893	0.144580684	DHTKD1
ENSG00000183873	113.3785295	43.59969359	1.381363636	0.076622935	0.144602344	SCN5A
ENSG00000157184	201.3987191	278.7134728	-0.468803947	0.076643594	0.144630827	CPT2
ENSG00000159197	3.296210009	0	4.055930299	0.07666224	0.144655505	KCNE2

ENSG00000150867	742.1478227	521.3865806	0.508881375	0.076703956	0.14470483	PIP4K2A
ENSG00000086619	411.2038516	248.8422288	0.725766825	0.0767082	0.14470483	ERO1B
ENSG00000261172	24.04359821	0	6.921312622	0.076709995	0.14470483	AC133919.2
ENSG00000272183	8.211028466	1.733100261	2.221450805	0.076710659	0.14470483	AC005041.3
ENSG00000286366	16.04822621	7.072884716	1.173038763	0.076721726	0.1447152	AC005052.2
ENSG00000139800	49.23386031	9.601779531	2.352188232	0.076733094	0.144726136	ZIC5
ENSG00000249456	11.81684803	22.91978856	-0.956894552	0.076749125	0.144745863	AL731577.2
ENSG00000108469	413.9234387	566.2725548	-0.452172118	0.076774106	0.144782467	RECQL5
ENSG00000074201	1185.150414	1515.863118	-0.355131106	0.076798124	0.14481725	CLNS1A
ENSG00000286656	30.86324226	13.47154533	1.19938038	0.076820248	0.144848455	AC008875.2
ENSG00000260558	4.20992361	0.336469917	3.446864493	0.076841727	0.144874684	AC018557.2
ENSG00000109111	2214.926236	2783.086599	-0.329351476	0.076846849	0.144874684	SUPT6H
ENSG00000105607	429.2704879	597.3549611	-0.477184791	0.076850887	0.144874684	GCDH
ENSG00000272654	24.54748093	12.07607086	1.032610342	0.076892814	0.144943205	AL358472.2
ENSG00000100359	23.73079718	47.91359522	-1.012077564	0.076934005	0.14501033	SGSM3
ENSG00000171695	6.873053107	1.467450118	2.24669503	0.077017906	0.145157941	LKAAEAR1
ENSG00000279865	6.929752385	1.447381022	2.266917858	0.077070127	0.145245827	AC006511.3
ENSG00000197555	1713.377038	1336.813095	0.35820365	0.077097783	0.145287409	SIPA1L1
ENSG00000196440	739.6216464	441.9151538	0.743346381	0.077107488	0.145295158	ARMCX4
ENSG00000261879	38.36540147	22.6446818	0.762018925	0.077116305	0.145301235	AC087500.1
ENSG00000214433	6.623903779	1.386017859	2.230916953	0.077141171	0.145337547	GOLGA2P8
ENSG00000287124	6.173172855	0	4.96103928	0.077194363	0.145419655	AL512603.2
ENSG00000185742	23.97500283	4.810924314	2.305542871	0.077195947	0.145419655	C11orf87
ENSG00000163159	634.7915883	805.9042999	-0.344549134	0.07723602	0.145476553	VPS72
ENSG00000180628	1609.097266	1003.916329	0.680337869	0.07723735	0.145476553	PCGF5
ENSG00000162591	568.480674	181.6036105	1.645697374	0.077315816	0.145613789	MEGF6
ENSG00000167920	114.2989661	183.2416655	-0.682182527	0.077411848	0.145784083	TMEM99
ENSG00000092051	13.18129255	3.372871508	1.984411456	0.07743151	0.145810543	JPH4
ENSG00000187566	72.47420925	47.87846571	0.597039235	0.077439498	0.145815017	NHLRC1
ENSG00000175455	1496.579244	1078.8266	0.472327169	0.077463087	0.145848863	CCDC14
ENSG00000205056	3.018084217	0	3.927863671	0.07748043	0.145860375	LINC02397
ENSG00000230399	3.018084217	0	3.927863671	0.07748043	0.145860375	RBBP8P1
ENSG00000214022	1884.585455	1430.30468	0.398160546	0.077498317	0.145883478	REPIN1
ENSG00000288067	4.734691432	0.336469917	3.618172834	0.077535056	0.145942062	AC105180.2
ENSG00000100575	271.2222922	387.2146627	-0.514471221	0.077547387	0.145954698	TIMM9
ENSG00000107341	1400.907236	1927.965682	-0.460535815	0.077557664	0.145963467	UBE2R2
ENSG00000165898	267.4295788	378.3358977	-0.501007072	0.07756447	0.145964852	ISCA2
ENSG00000204380	18.83610837	5.002158663	1.910421009	0.077569636	0.145964852	PKP4-AS1
ENSG00000166681	2669.755122	3915.486942	-0.552486979	0.077578904	0.145971719	BEX3
ENSG00000106013	3.912921816	10.68072479	-1.447956383	0.077604236	0.146008809	ANKRD7
ENSG00000232150	21.60497199	37.86006828	-0.812600364	0.0776333	0.146052915	ST13P4
ENSG00000250075	11.98415383	3.149799701	1.917002181	0.077645203	0.146059436	AC104806.2
ENSG00000236591	9.794107472	31.71960898	-1.689470369	0.077656164	0.146059436	AL357992.1
ENSG00000126254	1515.476108	2041.377786	-0.429839938	0.077656666	0.146059436	RBM42
ENSG00000188916	3.668787083	15.14084228	-2.045090843	0.077659253	0.146059436	INSYN2A
ENSG00000157890	5.10350756	0.723690511	2.827470564	0.077715297	0.146154263	MEGF11
ENSG00000146281	397.5843222	248.7808781	0.675710345	0.07773898	0.14618822	PM20D2
ENSG00000228526	694.7830766	970.4791217	-0.481972013	0.077758842	0.146214987	MIR34AHG
ENSG00000222017	3.373038938	0	4.086158406	0.077798196	0.146257231	AC011997.1
ENSG00000236078	3.373038938	0	4.086158406	0.077798196	0.146257231	LINC01447
ENSG00000286504	3.373038938	0	4.086158406	0.077798196	0.146257231	AC092745.4

ENSG00000172260	1492.31004	617.5669274	1.272614036	0.077835525	0.14631682	NEGR1
ENSG00000222020	28.69569066	14.61557816	0.971037005	0.077856501	0.146345662	HDAC4-AS1
ENSG00000197372	104.8159788	71.53371314	0.550620461	0.077939633	0.146491327	ZNF675
ENSG00000146007	874.8062666	1231.141639	-0.493163223	0.07795501	0.14650963	ZMAT2
ENSG00000204776	0.307102255	3.698728939	-3.500074916	0.077977224	0.146540779	IGKV1OR-3
ENSG00000279799	12.9407053	3.912472529	1.721622107	0.078037326	0.14664312	AC006077.2
ENSG00000066427	649.4640474	403.7727055	0.686192392	0.078069318	0.146692627	ATXN3
ENSG00000146457	1270.072886	1795.679407	-0.499416874	0.078086847	0.146714953	WTAP
ENSG00000261732	2.699627853	10.10928631	-1.898896795	0.078139721	0.146803679	AL031708.1
ENSG00000131398	159.9075336	58.89368699	1.443471775	0.078145848	0.146804574	KCNC3
ENSG00000186635	2697.370306	1727.736033	0.642766453	0.078204459	0.146904059	ARAP1
ENSG00000159409	5.745434885	1.110911105	2.396974055	0.078227783	0.146937249	CELF3
ENSG00000273312	10.11911682	2.466247382	2.024038888	0.078259467	0.14697032	AL121749.1
novel.218	5.841211088	28.61213082	-2.292692032	0.078269922	0.14697032	-
ENSG00000231007	0.308355904	3.506338714	-3.434743534	0.07828228	0.14697032	CDC20P1
ENSG00000272057	6.918398275	1.406086955	2.284353959	0.078282809	0.14697032	AC016575.1
ENSG00000253930	7.608178066	16.90590839	-1.153880486	0.078283396	0.14697032	TNFRSF10A-AS1
ENSG00000267702	5.983230103	0.713078025	3.064340431	0.078287248	0.14697032	AP005131.6
ENSG00000284024	274.5943344	353.6761076	-0.365509815	0.078290463	0.14697032	HSPA14
ENSG00000249685	14.88278471	28.52522316	-0.941740022	0.078290644	0.14697032	AC079921.2
ENSG00000179163	591.7269977	373.9854754	0.662780532	0.07832915	0.147031981	FUCA1
ENSG00000223646	4.194808554	0.356539013	3.442265266	0.078374622	0.147106708	AC002463.1
ENSG00000225727	7.429304689	1.049547942	2.808642487	0.078384875	0.147115325	AL139184.1
ENSG00000226221	112.1544784	178.7902683	-0.673121372	0.078417288	0.147164058	RPL26P19
ENSG00000204178	476.7040747	632.9112829	-0.408428873	0.078422169	0.147164058	MACO1
ENSG00000235408	2.682005499	9.824851338	-1.870085216	0.078441307	0.147189339	SNORA71B
ENSG00000277310	3.049567979	0	3.941227633	0.078458878	0.147211679	AC090246.1
ENSG00000279048	17.45271657	6.634913444	1.383488825	0.078485489	0.147250975	AC080080.1
ENSG00000180432	5.237035768	0	4.721473612	0.078559134	0.147376931	CYP8B1
ENSG00000180257	96.05686136	60.82012342	0.656503184	0.078565107	0.147376931	ZNF816
ENSG00000272068	7.829604579	1.783850939	2.131616585	0.078573543	0.147376931	AL365181.2
ENSG00000138078	1097.877698	813.4294793	0.432314108	0.078577068	0.147376931	PREPL
ENSG00000134996	1007.865382	756.6236816	0.413606148	0.078580987	0.147376931	OSTF1
ENSG00000262833	1.186824798	8.303054522	-2.795723451	0.07861982	0.147439118	AC016245.1
ENSG00000177600	9787.278037	13590.45546	-0.473610215	0.07865105	0.147487039	RPLP2
ENSG00000223523	3.345316124	0	4.075291503	0.078680019	0.147509422	AC092598.1
ENSG00000247193	3.345316124	0	4.075291503	0.078680019	0.147509422	AC104078.1
ENSG00000122859	3.345316124	0	4.075291503	0.078680019	0.147509422	NEUROG3
ENSG00000239827	12.15418039	4.055267952	1.597640026	0.078699002	0.147534365	SUGT1P3
ENSG00000184923	20.74795767	9.04912588	1.199417569	0.078800592	0.147714154	NUTM2A
ENSG00000100523	590.2578582	450.4118956	0.389781087	0.078838711	0.147770613	DDHD1
ENSG00000226054	36.15919987	73.51688956	-1.024230833	0.078842086	0.147770613	MEMO1P1
ENSG00000250462	230.6973318	167.8723916	0.459479639	0.078852883	0.147780188	LRRC37BP1
novel.819	77.3950394	173.2846996	-1.162585482	0.078887067	0.147833589	-
ENSG00000138658	141.246538	206.4255972	-0.546698363	0.078938057	0.147908219	ZGRF1
ENSG00000286036	3.035706571	0	3.935348108	0.078938277	0.147908219	AC017099.2
ENSG00000251301	5.225681658	0.336469917	3.755983345	0.078946197	0.147912393	LINC02384
ENSG00000137040	493.5991714	630.6952869	-0.353719693	0.078973294	0.147952491	RANBP6
ENSG00000225361	11.2518209	22.78760562	-1.022473914	0.079003596	0.147998588	PPP1R26-AS1
ENSG00000102104	3.031945625	0	3.933758202	0.079068935	0.148099632	RS1
ENSG00000273819	3.031945625	0	3.933758202	0.079068935	0.148099632	ENPP7P7

ENSG00000267534	514.9925044	354.5206798	0.539616191	0.079079317	0.148108401	S1PR2
ENSG00000143845	147.225137	232.5965584	-0.660273806	0.079124192	0.148181766	ETNK2
ENSG00000284154	3.029438327	13.78666188	-2.182135745	0.079181152	0.148277751	MIR3605
ENSG00000221643	0	2.92428775	-4.126264069	0.079205739	0.148313103	SNORA77
ENSG00000108349	2532.57939	1934.666127	0.388553105	0.079212747	0.148315535	CASC3
ENSG00000124570	3508.532271	2668.953874	0.394555698	0.079248175	0.148371178	SERPINB6
ENSG00000258559	37.99622934	16.85502932	1.179622317	0.079256849	0.148376726	AC005519.1
ENSG00000144214	43.52532358	22.0944683	0.982191522	0.079334752	0.148511867	LYG1
ENSG00000181215	4.296853001	0.336469917	3.473101805	0.079423393	0.148667088	C4orf50
ENSG00000162814	10.37962025	23.02257418	-1.15550859	0.079484518	0.148770785	SPATA17
ENSG00000287271	29.76410231	12.93194431	1.213203572	0.079521577	0.148829427	AC011455.6
ENSG00000127666	361.1869419	548.0061946	-0.600878504	0.079541702	0.148856369	TICAM1
ENSG00000230383	176.0892446	277.6551105	-0.657023993	0.07954871	0.148858761	AC009245.1
ENSG00000141002	1828.284208	2356.501361	-0.36603624	0.079563115	0.148874995	TCF25
ENSG00000203739	68.34208348	42.10772645	0.700484637	0.079589318	0.14891297	AL645568.1
ENSG00000120664	24.40266988	9.619408483	1.340867428	0.079594874	0.14891297	SPART-AS1
ENSG00000244560	120.7494815	78.09035798	0.631637144	0.079601622	0.148914871	AC004890.2
ENSG00000250327	14.30633221	2.742510011	2.365264179	0.079635898	0.148968267	RPSAP70
ENSG00000174327	23.13922962	43.33421902	-0.905443746	0.07966407	0.149010238	SLC16A13
ENSG00000008282	1440.438063	1801.576249	-0.322856335	0.07967515	0.149020234	SYPL1
ENSG00000169635	118.2518347	207.5534481	-0.810301956	0.079710851	0.149076276	HIC2
ENSG00000266217	4.255268779	0.387220594	3.460563809	0.079763162	0.149163371	CTSLP2
ENSG00000242512	5.054401445	0	4.672368325	0.079859622	0.149325272	LINC01206
ENSG00000211460	1095.461851	1492.965033	-0.446861105	0.079861232	0.149325272	TSN
ENSG00000152782	59.90894433	102.8087667	-0.781452205	0.079906306	0.149391471	PANK1
ENSG00000225849	8.178291056	2.079026788	1.951526101	0.079913136	0.149391471	MKRN7P
ENSG00000279520	15.0601197	30.998487	-1.038989464	0.079913886	0.149391471	AC093525.8
ENSG00000236514	7.317444516	1.456837632	2.336458925	0.079922228	0.149396316	AL135791.1
ENSG00000265874	9.10085147	2.864080462	1.666043359	0.07996217	0.149460225	MIR4489
ENSG00000229536	3.005476459	0	3.922464968	0.079999785	0.149519776	LINC02572
ENSG00000159905	12.64621081	29.56886333	-1.223273831	0.080018384	0.14954378	ZNF221
novel.762	3.00422281	0	3.921931402	0.080044226	0.149570558	-
ENSG00000250250	3.00422281	0	3.921931402	0.080044226	0.149570558	AC010638.1
ENSG00000184675	128.8700335	88.46786297	0.543680878	0.080058381	0.14958625	AMER1
ENSG00000283208	10.4360348	2.506385574	2.058342311	0.080116657	0.149677945	AC001226.2
novel.242	6.992719907	15.96030195	-1.196457087	0.080118979	0.149677945	-
ENSG00000235070	15.23877962	4.53581756	1.738045649	0.080129603	0.14968703	AC062015.1
ENSG00000134463	129.939058	217.6600503	-0.743789537	0.080195004	0.149798432	ECHDC3
ENSG00000154781	416.2549624	316.5437114	0.395662681	0.080283376	0.149952724	CCDC174
ENSG00000087076	178.2568681	89.22270179	1.000647095	0.080301055	0.149974963	HSD17B14
ENSG00000185475	344.7439998	468.2303831	-0.442149737	0.080342643	0.150041849	TMEM179B
ENSG00000150782	592.6541566	166.5559689	1.830600968	0.080364963	0.150072746	IL18
novel.736	37.40967637	86.28789559	-1.205841203	0.080385369	0.150100063	-
ENSG00000204149	36.08251349	18.88214956	0.938252064	0.080440595	0.150190057	AGAP6
ENSG00000166529	109.1296278	68.35811215	0.672021346	0.080445127	0.150190057	ZSCAN21
ENSG00000260368	11.22033714	2.793260689	1.992210711	0.08046249	0.15021168	AC027373.1
ENSG00000143257	18.76289785	4.982089567	1.909714163	0.080486088	0.150244938	NR1I3
ENSG00000141424	1459.90808	1893.398794	-0.375212597	0.080585515	0.150419734	SLC39A6
ENSG00000236938	0	4.523920805	-4.754959862	0.080603569	0.150442624	AC003092.2
ENSG00000105287	486.5743269	615.851851	-0.340034901	0.08064557	0.150503615	PRKD2
ENSG00000100565	3.284855899	0	4.051361461	0.080657434	0.150503615	LRRC74A

ENSG00000174776	3.284855899	0	4.051361461	0.080657434	0.150503615	WDR49
ENSG00000112996	410.4985573	566.924141	-0.46640095	0.080659418	0.150503615	MRPS30
ENSG00000115756	768.022794	1443.816043	-0.910526791	0.080673952	0.150519924	HPCAL1
ENSG00000105464	43.63309428	113.3448511	-1.378001168	0.080693365	0.150545334	GRIN2D
ENSG00000196668	40.77497996	13.7559803	1.57510024	0.080715745	0.150576275	LINC00173
ENSG00000286810	7.842212337	2.089639274	1.88776394	0.080733776	0.150590893	AL513128.3
ENSG00000284266	3.282348601	0	4.0503661	0.08074097	0.150590893	MIR4534
ENSG00000233033	3.282348601	0	4.0503661	0.08074097	0.150590893	CASK-AS1
ENSG00000143951	142.9611361	88.45776407	0.690452355	0.080795888	0.150682505	WDPCP
ENSG00000184227	45.05889702	22.46303246	0.997264456	0.080872448	0.150814461	ACOT1
ENSG00000267421	8.513116126	2.476859868	1.770532931	0.081026242	0.151090417	AC005498.2
ENSG00000004838	26.17207288	12.48464482	1.066637613	0.081045891	0.151116212	ZMYND10
ENSG00000177034	660.1038708	476.5992015	0.469984007	0.081057903	0.151127763	MTX3
ENSG00000116273	503.5116645	685.1893208	-0.444038005	0.081068151	0.151136023	PHF13
ENSG00000140332	1551.625199	987.8501088	0.651673336	0.08110861	0.151200602	TLE3
ENSG00000262772	3.620934617	0.356539013	3.229053384	0.08112331	0.151201833	LINC01977
ENSG00000141644	1255.308016	1589.760794	-0.340580371	0.081126637	0.151201833	MBD1
ENSG00000198121	5082.429742	2991.992812	0.764329316	0.081126729	0.151201833	LPAR1
ENSG00000064601	2221.942942	3278.674703	-0.561195604	0.081217444	0.151360047	CTSA
ENSG00000234791	5.530347887	0	4.8003403	0.08123082	0.151374117	AC108448.1
ENSG00000119042	166.963221	279.3393264	-0.741590524	0.081290127	0.151473772	SATB2
ENSG00000228709	2.756327131	8.95823701	-1.707707481	0.081323235	0.151524597	LINC02575
ENSG00000075407	638.3500265	489.9736934	0.381817996	0.08135956	0.151581408	ZNF37A
ENSG00000117691	1050.100981	802.8079818	0.387174596	0.08138	0.151608617	NENF
ENSG00000143061	78.60059695	28.73781087	1.44982941	0.081425529	0.151682561	IGSF3
ENSG00000236138	3.313832363	11.11998033	-1.751390137	0.081441039	0.151700577	DUX4L26
ENSG00000226440	22.23909269	8.022087178	1.462067829	0.081473638	0.151750418	AL365214.2
ENSG00000226548	0.586481695	5.512105584	-3.206202438	0.08148098	0.151753215	AC016722.1
ENSG00000137936	769.5648464	1177.133802	-0.612874133	0.081519288	0.151813677	BCAR3
ENSG00000272861	10.35189744	2.557136252	2.025323352	0.081528482	0.151819917	AC012360.2
ENSG00000120860	640.2174561	496.6835366	0.366600162	0.081631562	0.152000976	WASHC3
ENSG00000233306	4.923665269	0	4.632442096	0.081653339	0.152030628	TRGV2
ENSG00000255760	7.051926483	1.467450118	2.27763994	0.081692372	0.152086486	LINC02422
ENSG00000280042	1.218308559	6.460152171	-2.41075401	0.081695047	0.152086486	AC022336.3
ENSG00000282458	9.042898543	21.03572053	-1.221685797	0.081703981	0.152086753	WASH5P
ENSG00000213839	41.17584932	62.98148155	-0.612292068	0.081706897	0.152086753	TMX2P1
ENSG00000147041	21.10681588	6.339737595	1.728347203	0.081719808	0.152099888	SYTL5
ENSG00000271265	7.536363731	1.722487775	2.101784431	0.0817543	0.152153185	AL355297.3
ENSG00000197381	1297.251342	2456.031305	-0.920959796	0.081823428	0.152270933	ADARB1
ENSG00000225940	4.924918918	0	4.632795831	0.08195587	0.15250648	C5orf67
ENSG00000183496	106.6101979	168.7602309	-0.663095439	0.081975292	0.152531696	MEX3B
ENSG00000268686	7.927888079	2.191140629	1.865949478	0.08209592	0.152745209	AC010643.1
ENSG00000156398	142.7444398	270.1043385	-0.92058851	0.082108423	0.152757532	SFXN2
novel.666	36.73572365	18.23300323	1.002323161	0.082162244	0.152846717	-
ENSG00000215375	290.361073	196.6178268	0.561417467	0.082222414	0.152939081	MYL5
ENSG00000099219	464.9558767	308.5739156	0.590736836	0.082223667	0.152939081	ERMP1
ENSG00000263843	35.60656705	18.39702363	0.948128499	0.082374746	0.153209125	AC022211.2
ENSG00000261094	45.04475088	27.58663318	0.706964727	0.082382554	0.15321268	AC007066.2
ENSG00000136243	412.339648	296.4175687	0.476459368	0.082409129	0.153251133	NUPL2
ENSG00000213672	616.5338142	918.7924167	-0.575199116	0.082418122	0.153256887	NCKIPSD
ENSG00000114854	18.39060551	4.514592589	2.014261797	0.082522636	0.153440249	TNNC1

ENSG00000136867	3.049567979	9.395052407	-1.625622845	0.082541493	0.153464328	SLC31A2
ENSG00000236527	0	2.965581817	-4.144175446	0.082567533	0.153501759	ARF4P2
ENSG00000104880	452.6370838	601.5035928	-0.410235666	0.082607481	0.153565038	ARHGEF18
novel.317	142.683125	82.79045273	0.786276775	0.082650392	0.153624407	-
ENSG00000178913	1574.07132	2108.935686	-0.422004749	0.082651244	0.153624407	TAF7
ENSG00000109255	0.307102255	3.515795324	-3.438041208	0.082661786	0.153625912	NMU
ENSG00000132670	1976.148135	1456.847813	0.439706113	0.082663879	0.153625912	PTPRA
ENSG00000119699	153.0063151	78.8124259	0.954952325	0.082673073	0.153631196	TGFB3
ENSG00000253347	12.6701014	4.279624027	1.562791468	0.08268067	0.153631196	AC040934.1
ENSG00000287387	6.968758039	1.467450118	2.262941368	0.082684462	0.153631196	AC010883.3
ENSG00000220305	7.215400069	1.069617038	2.749498049	0.082775335	0.153789042	HNRNPH1P1
ENSG00000157895	288.9594463	391.2389111	-0.437650008	0.082790099	0.153805474	C12orf43
ENSG00000183579	259.7032449	181.5050504	0.516289142	0.08280325	0.153818908	ZNRF3
ENSG00000287294	6.445315137	0.713078025	3.168731893	0.082815381	0.153830443	AL035694.1
ENSG00000229498	6.199642021	0.387220594	4.005939797	0.082882003	0.153943188	AC105053.1
ENSG00000186141	488.6687393	646.8944234	-0.40499856	0.082906618	0.153977899	POLR3C
ENSG00000227959	10.60362533	3.179325408	1.728025438	0.08296642	0.154077953	AL451042.2
ENSG00000107223	3505.985667	4501.045074	-0.360435995	0.083008347	0.154131415	EDF1
ENSG00000127125	826.9080596	623.3973226	0.407310164	0.083009387	0.154131415	PPCS
ENSG00000231738	0.307102255	3.240688571	-3.314786231	0.083013005	0.154131415	TSPAN19
ENSG00000199459	0	2.822786395	-4.081378114	0.083172071	0.154404685	RF00019
ENSG00000287161	0	2.822786395	-4.081378114	0.083172071	0.154404685	AC093519.2
ENSG00000138433	734.9793374	528.3160963	0.476692223	0.08319688	0.154439708	CIR1
ENSG00000279365	0	4.554602387	-4.764311057	0.083209384	0.154451884	AP000695.3
ENSG00000143933	11891.63613	9663.390847	0.299346088	0.083219114	0.154452986	CALM2
ENSG00000166359	28.4442475	11.77986753	1.268620496	0.083229431	0.154452986	WDR88
ENSG00000168137	1681.494576	2374.28929	-0.49760696	0.083230588	0.154452986	SETD5
ENSG00000014914	531.3673326	307.4183247	0.789804903	0.083237986	0.154452986	MTMR11
ENSG00000175274	578.5683692	1071.37298	-0.88914212	0.083243485	0.154452986	TP53I11
ENSG00000229444	0.293240848	3.240688571	-3.31478818	0.083245647	0.154452986	AL451062.1
ENSG00000113638	387.7388575	273.6515264	0.503808533	0.083302841	0.154548066	TTC33
ENSG00000172296	174.9791343	62.6505339	1.479885871	0.083318126	0.154565385	SPTLC3
ENSG00000261613	13.88311063	2.272572888	2.63267158	0.083348375	0.154610462	AC093525.6
ENSG00000149792	809.3474569	1148.426933	-0.505061701	0.083482996	0.154849127	MRPL49
ENSG00000171634	1369.564802	996.1473146	0.459280191	0.083494552	0.154859505	BPTF
ENSG00000279456	33.40516658	16.19270197	1.054204414	0.083548869	0.154949187	AL353763.1
ENSG00000170893	2.775203133	184.8044649	-6.058192837	0.083563816	0.154965846	TRH
novel.969	21.58678017	0	6.765846396	0.083599161	0.155020328	-
ENSG00000280287	18.72647076	35.3689999	-0.915468842	0.083623443	0.155054288	AC131212.3
ENSG00000167526	22143.33249	29571.83231	-0.417349589	0.083672855	0.155134836	RPL13
ENSG00000184897	1081.955875	1667.699307	-0.624174628	0.083688759	0.15515325	H1FX
ENSG00000085721	32.86556843	83.41203362	-1.344330737	0.083699836	0.155162714	RRN3
ENSG00000225872	4.793898008	12.8824779	-1.421629715	0.083732788	0.155212726	LINC01529
ENSG00000253829	6.684364003	1.406086955	2.2324414	0.083750303	0.155234117	AC067817.2
ENSG00000095932	8.275320908	1.161661783	2.874643935	0.083786075	0.155289344	SMIM24
ENSG00000235885	0	4.401194479	-4.717445004	0.083928314	0.155541874	LINC01828
novel.140	21.47588891	0	6.758448608	0.083939128	0.155550818	-
ENSG00000171811	18.51131249	33.43623616	-0.850292845	0.083971848	0.155600356	CFAP46
ENSG00000152464	132.922823	188.0392332	-0.500360255	0.083993854	0.155630033	RPP38
ENSG00000211456	1114.750629	801.0787472	0.476294152	0.084100781	0.155817043	SACM1L
ENSG00000102580	1047.275348	1305.580923	-0.3181308	0.084118251	0.155838297	DNAJC3

ENSG00000145592	7601.628964	10255.4685	-0.432016011	0.084131761	0.155852213	RPL37
novel.446	40.74373784	128.6221143	-1.659177193	0.084149541	0.155874036	-
ENSG00000278784	5.686228309	0.774441189	2.936112553	0.084199863	0.15595613	AL136295.7
ENSG00000232439	7.480918102	1.436768536	2.382564025	0.084252824	0.156043102	RPL18AP7
ENSG00000280216	12.01814489	3.331577441	1.866791966	0.084278012	0.156078625	AL022326.2
novel.663	110.0163302	59.24274282	0.891686316	0.084312718	0.15613177	-
ENSG00000079313	581.7662835	837.759675	-0.525805268	0.08435995	0.156208101	REXO1
ENSG00000081853	98.33986406	54.96653924	0.841185819	0.084463635	0.156388947	PCDHGA2
ENSG00000148303	135.5538989	213.4237753	-0.654402033	0.084480361	0.156408769	RPL7A
ENSG00000286496	0.601596751	4.525205075	-2.913505537	0.084530416	0.156490291	AL359764.1
ENSG00000121743	44.33910058	21.43226934	1.046424388	0.084541487	0.156499636	GJA3
ENSG00000225362	0.925067711	6.715189828	-2.872953526	0.084576774	0.156553802	CT62
ENSG00000223396	35.86323827	20.57511163	0.805334492	0.084600133	0.156585884	RPS10P7
ENSG00000237552	4.80274482	0	4.59813111	0.084646374	0.156649152	LINC02567
ENSG00000260412	4.80274482	0	4.59813111	0.084646374	0.156649152	AL353746.1
ENSG00000287252	12.48775182	26.69750961	-1.094250008	0.08470167	0.15674032	AL009179.2
ENSG00000279489	5.408102518	0.774441189	2.86548298	0.08473246	0.15678613	AL355377.2
ENSG00000264148	4.270383835	0.356539013	3.465182637	0.084799928	0.156899795	AC138207.3
ENSG00000126773	1264.101908	1749.71081	-0.468816617	0.084827983	0.15691556	PCNX4
ENSG00000240889	15.73854538	5.379922647	1.548016453	0.084829087	0.15691556	NDUFB2-AS1
ENSG00000270497	1.828752122	8.814157318	-2.268446549	0.084832405	0.15691556	BX322635.1
ENSG00000251417	24.44801505	10.55915433	1.204286444	0.084832607	0.15691556	AC145285.2
ENSG00000099385	1018.349257	713.266675	0.513364927	0.08488023	0.156992471	BCL7C
ENSG00000106723	2224.880381	1800.648773	0.305212573	0.084904796	0.15702673	SPIN1
novel.619	38.66999643	19.5348919	0.993888002	0.085013958	0.157217428	-
ENSG00000273108	6.072666791	1.10029862	2.479493546	0.08509389	0.157346559	AL121929.2
ENSG00000101161	1820.976837	2273.766963	-0.32038522	0.085095897	0.157346559	PRPF6
ENSG00000248375	1.230916317	6.288986917	-2.369703758	0.085133176	0.157404288	AC104066.1
ENSG00000176393	1164.289767	1633.735965	-0.488883226	0.085170333	0.157458144	RNPEP
ENSG00000025423	5.156445892	12.81995886	-1.317585233	0.085176797	0.157458144	HSD17B6
ENSG00000174326	19.06276294	5.126040865	1.900036488	0.085180486	0.157458144	SLC16A11
ENSG00000214146	3.050821627	0	3.941821443	0.085242957	0.157528791	LINC02026
ENSG00000228329	3.050821627	0	3.941821443	0.085242957	0.157528791	LINC01890
ENSG00000285347	3.050821627	0	3.941821443	0.085242957	0.157528791	AL359555.4
ENSG00000246022	3.050821627	0	3.941821443	0.085242957	0.157528791	ALDH1L1-AS2
ENSG00000121350	293.2174792	199.1879685	0.55718511	0.085335253	0.157688138	PYROXD1
ENSG00000183421	60.1182338	18.8606678	1.678609738	0.085359591	0.157721893	RIPK4
ENSG00000272209	3.045807032	0	3.939694005	0.085430382	0.157830246	AL023583.1
ENSG00000254432	3.045807032	0	3.939694005	0.085430382	0.157830246	AC113143.1
ENSG00000224690	0.308355904	3.291439249	-3.333310006	0.085451337	0.157857735	UBE2D3P3
ENSG00000287613	3.351655638	0	4.077841281	0.085501572	0.157928079	AC069549.2
ENSG00000227579	3.351655638	0	4.077841281	0.085501572	0.157928079	AL136114.1
ENSG00000183309	34.17442514	16.80427865	1.03027362	0.085530036	0.157969423	ZNF623
ENSG00000161609	3.68390214	0	4.213553155	0.085575181	0.158041568	CCDC155
ENSG00000247311	0.921306765	6.083544062	-2.73280924	0.085624879	0.15812211	AC010255.1
ENSG00000166106	322.0859722	109.1022484	1.56068492	0.085638402	0.158135843	ADAMTS15
ENSG00000006837	37.95500112	18.95669375	0.995215341	0.085716842	0.158269439	CDKL3
ENSG00000068024	575.0295347	393.7319869	0.54571909	0.085765227	0.158347524	HDAC4
ENSG00000160229	8.976098803	2.129777466	2.070655046	0.085818804	0.158435184	ZNF66
ENSG00000272800	12.37059231	4.983245442	1.307344695	0.085877939	0.158523747	AC021851.1
ENSG00000280401	7.508640916	1.4981317	2.354294569	0.085878979	0.158523747	AC022532.1

ENSG00000236305	1.22840902	8.35740122	-2.776774125	0.085888563	0.158530176	SLC12A9-AS1
ENSG00000119121	4.542241382	0.723690511	2.65792503	0.085905567	0.158539035	TRPM6
ENSG00000261794	4.542241382	0.723690511	2.65792503	0.085905567	0.158539035	GOLGA8H
ENSG00000257663	3.672619301	0	4.20944748	0.085931858	0.158576291	AC025259.1
ENSG00000129990	1.21705491	5.390535133	-2.150561298	0.085987344	0.158667413	SYT5
ENSG00000187922	4.441450584	0.356539013	3.526018651	0.086009881	0.158687018	LCN10
ENSG00000286334	5.146345431	0.713078025	2.844876112	0.086010184	0.158687018	AL161430.1
ENSG00000135596	1883.184354	2407.804605	-0.354406075	0.086047724	0.158745006	MICAL1
ENSG00000241520	7.833365525	1.753169357	2.145731278	0.08607873	0.158790931	AC098820.3
ENSG00000262185	37.04185699	14.69713881	1.326722708	0.086130767	0.158875644	AC005736.1
ENSG00000237593	5.306058071	0	4.742789318	0.086140076	0.158881536	AL445220.1
ENSG00000090932	9.550257474	35.2544931	-1.887183539	0.086149052	0.158886812	DLL3
ENSG00000163900	284.7553915	389.0079702	-0.450917626	0.086155411	0.158887262	TMEM41A
ENSG00000197429	243.2332204	335.7872799	-0.466089992	0.08623617	0.159024908	IPP
ENSG00000280087	7.833365525	1.456837632	2.435764246	0.086283642	0.159097668	AC011481.3
ENSG00000153707	21.73731782	51.72153113	-1.253908364	0.086287873	0.159097668	PTPRD
ENSG00000179604	1461.912727	1107.423777	0.400360441	0.086300523	0.1591097	CDC42EP4
ENSG00000007933	505.5008764	40.66206206	3.635314214	0.0863269	0.159147037	FMO3
ENSG00000168334	8.061131553	25.47422788	-1.652666965	0.086362905	0.159202116	XIRP1
ENSG00000163877	320.5728573	404.9871386	-0.337027791	0.086396961	0.159253597	SNIP1
ENSG00000167332	4.858119179	0	4.613935786	0.086403434	0.159254229	OR51E2
ENSG00000226007	26.69637034	6.836760279	1.964854815	0.086425068	0.159282805	BX005266.2
ENSG00000172061	48.2421354	168.7440021	-1.806431777	0.086524925	0.15945553	LRRC15
ENSG00000112592	284.598087	377.7746733	-0.408117827	0.086548458	0.159487586	TBP
ENSG00000109606	2253.330177	3101.187506	-0.46074219	0.086596708	0.159565182	DHX15
ENSG00000246339	34.32779826	12.18844149	1.487278811	0.086614287	0.159586255	EXTL3-AS1
ENSG00000210112	13.29315273	5.757686631	1.209485234	0.08663981	0.159611237	MT-TM
ENSG00000270049	63.42253517	31.50167425	1.004828498	0.086640132	0.159611237	AC009061.2
ENSG00000273270	203.0391815	115.0046099	0.818269532	0.086672884	0.159660252	AC090114.2
ENSG00000177679	51.74103846	23.42986387	1.139837508	0.086723516	0.159742196	SRRM3
ENSG00000264520	5.395494759	0.713078025	2.914962731	0.086758066	0.159794507	AC005154.4
ENSG00000242457	0.61420451	4.688069593	-2.942016975	0.086835874	0.15992648	RBBP4P2
ENSG00000108551	86.19029846	181.9318016	-1.077086711	0.086843554	0.159929287	RASD1
ENSG00000280383	18.10223706	38.94744266	-1.105250697	0.086943181	0.160101409	Z95331.1
ENSG00000205670	57.45756816	25.73414582	1.160028363	0.086972652	0.160144328	SMIM11A
ENSG00000110011	637.2827805	447.7706777	0.509270371	0.087003193	0.160189211	DNAJC4
ENSG00000131043	638.7960547	869.8073029	-0.44575906	0.08704233	0.160225929	AAR2
ENSG00000276649	14.2094449	5.768299117	1.303699378	0.087046019	0.160225929	AL117335.1
ENSG00000110090	1561.935577	1128.941122	0.468081725	0.087047257	0.160225929	CPT1A
ENSG00000013275	95.14598323	179.8914525	-0.918586549	0.087047804	0.160225929	PSMC4
ENSG00000125845	114.5116164	266.5289056	-1.218547071	0.087071698	0.160258557	BMP2
ENSG00000186104	251.9141131	177.4549539	0.50688616	0.087286465	0.160642463	CYP2R1
ENSG00000085382	439.5245729	297.3946615	0.564223427	0.08730872	0.16067204	HACE1
novel.776	15.0038477	3.068367442	2.27040232	0.087383136	0.160797597	-
ENSG00000123992	817.6540644	1033.794377	-0.338144784	0.087472377	0.160932699	DNPEP
ENSG00000259194	5.030439577	0	4.665730775	0.08747304	0.160932699	AC020891.1
ENSG00000099875	1966.448983	2865.077552	-0.5428606	0.087475139	0.160932699	MKNK2
ENSG00000249084	0	2.842855491	-4.090354321	0.087501288	0.160969409	AC027627.1
ENSG00000205758	602.265006	444.6320852	0.438012035	0.087510633	0.160975202	CRYZL1
ENSG00000275120	14.22072773	6.175588807	1.210967127	0.087517932	0.16097723	AC048382.5
ENSG00000187848	0.307102255	3.220619475	-3.307358633	0.087536639	0.16099884	P2RX2

ENSG00000136315	2.990361403	0	3.915878638	0.087550099	0.16099884	AL355922.1
ENSG00000083307	2.990361403	0	3.915878638	0.087550099	0.16099884	GRHL2
ENSG00000236432	52.47057936	27.29518174	0.936274181	0.087554467	0.16099884	AC097662.1
ENSG00000236739	9.102105118	19.53514869	-1.105892412	0.087582404	0.161038815	CLIC4P1
ENSG00000111077	3398.790064	2279.37053	0.576305147	0.087632462	0.161090855	TNS2
ENSG00000264345	4.918650674	0	4.631077628	0.087633439	0.161090855	LINC01894
ENSG00000211452	9.444452081	2.069570178	2.167912092	0.087640141	0.161090855	DIO1
ENSG00000164808	998.5385754	794.6150334	0.329246244	0.087641461	0.161090855	SPIDR
ENSG00000272482	34.99576748	11.69817848	1.591864651	0.087641709	0.161090855	AC254633.1
ENSG00000286623	4.542241382	0.743759607	2.642221706	0.087696757	0.161180635	AC005041.4
ENSG00000228146	9.180258968	1.874739808	2.313775237	0.087710171	0.161193885	CASP16P
ENSG00000279198	29.38260715	57.5331321	-0.966678496	0.087734712	0.161227583	AC008894.3
ENSG00000266897	0.293240848	3.220619475	-3.307357164	0.087776196	0.161292407	AC005546.1
ENSG00000109920	188.0707645	106.7328321	0.817262604	0.087844883	0.161407206	FNBP4
ENSG00000250011	3.66251884	0.356539013	3.243135025	0.087869116	0.161440315	HMGB1P3
ENSG00000277149	89.33710976	43.56456408	1.036102752	0.087891841	0.16147065	TYW1B
ENSG00000130038	52.83208706	111.8655511	-1.084307916	0.087927621	0.161524961	CRACR2A
ENSG00000162999	74.46589297	37.29119834	0.99379401	0.087953173	0.161552022	DUSP19
ENSG00000275215	15.95391712	3.12857473	2.338420467	0.087954788	0.161552022	RNA5-8SN3
ENSG00000203896	123.6489391	79.59362676	0.635158206	0.087994688	0.161613882	LIME1
ENSG00000287401	5.127469429	0.672939833	2.877441377	0.088082623	0.161763952	AC034139.1
ENSG00000231367	3.665026137	0.336469917	3.243983704	0.088098942	0.161782485	LINC02613
ENSG00000177683	454.9479087	352.5387533	0.367870121	0.088132962	0.16183352	THAP5
ENSG00000105821	406.1859488	549.0251675	-0.435203291	0.088146578	0.161835913	DNAJC2
ENSG00000224940	6.411252807	1.110911105	2.549850649	0.088146723	0.161835913	PRRT4
ENSG00000226318	8.77738051	17.86456747	-1.026794032	0.088166038	0.161859938	RPS3AP38
ENSG00000266129	0.923814062	7.763453501	-3.076666513	0.088217033	0.161926783	SRP68P1
ENSG00000251229	1.201939854	5.339784455	-2.148627699	0.088219112	0.161926783	AL645924.2
ENSG00000279407	15.16975731	27.40369957	-0.856973266	0.088224647	0.161926783	AC007191.1
ENSG00000234925	3.916682763	0.387220594	3.342423649	0.088227379	0.161926783	ATP5PDP4
ENSG00000174799	165.286474	236.5758692	-0.518156703	0.088281054	0.162010633	CEP135
ENSG00000173013	30.02607286	51.69619656	-0.781386323	0.088285537	0.162010633	CCDC96
ENSG00000276449	15.07398111	5.992655192	1.322224365	0.088303869	0.162032829	AC004076.2
ENSG00000259200	0	4.585283968	-4.773715044	0.088325053	0.162060254	AC068722.1
ENSG00000120709	754.8562493	1046.246448	-0.470619496	0.088347129	0.162089312	FAM53C
ENSG00000273173	8.495493772	2.446178286	1.777854822	0.088361312	0.162103887	SNURF
ENSG00000244227	3.269740843	0	4.045187448	0.088405058	0.162149793	LRRC77P
ENSG00000148795	3.269740843	0	4.045187448	0.088405058	0.162149793	CYP17A1
ENSG00000233334	3.269740843	0	4.045187448	0.088405058	0.162149793	FAM53B-AS1
ENSG00000261728	3.268487194	0	4.044685025	0.088450589	0.162210401	AL138690.1
ENSG00000250156	3.268487194	0	4.044685025	0.088450589	0.162210401	LINC02060
ENSG00000286977	37.7912428	17.5304093	1.108606559	0.088610485	0.162492166	Z83745.1
ENSG00000231304	3.916682763	0.356539013	3.342420445	0.088635535	0.162526631	SGO1-AS1
novel.569	20.04188009	0	6.658460466	0.088645265	0.162533001	-
ENSG00000188959	0.293240848	3.189937893	-3.296195333	0.088692203	0.162596113	C9orf152
ENSG00000237560	0.293240848	3.189937893	-3.296195333	0.088692203	0.162596113	LINC01497
novel.637	32.31669655	68.88803381	-1.090936928	0.088699693	0.162598372	-
ENSG00000255624	5.457208632	15.11504099	-1.474688674	0.088833167	0.162831559	AC073585.1
ENSG00000263272	88.09315911	53.15894197	0.730857933	0.088849554	0.162846149	AC004148.2
ENSG00000135966	604.4222708	428.2545699	0.496370541	0.088857693	0.162846149	TGFBRAP1
ENSG00000260448	11.48076931	23.06271238	-1.008814079	0.088859931	0.162846149	LCMT1-AS1

ENSG00000274642	5.157699541	13.79753115	-1.424365339	0.088924232	0.162952495	AC244669.2
ENSG00000110321	9873.507501	13586.26461	-0.460542068	0.089036644	0.163146982	EIF4G2
ENSG00000233527	45.72568386	67.37103607	-0.559709612	0.089161779	0.163364752	ZNF529-AS1
ENSG00000259342	0.308355904	4.911141399	-3.909909733	0.089236535	0.16348353	AC025580.1
novel.426	183.5492935	104.6403203	0.812450401	0.089239191	0.16348353	-
ENSG00000164074	255.8266789	182.8542693	0.48410039	0.089257153	0.163502293	ABHD18
ENSG00000285680	5.223245632	0	4.717887705	0.089262019	0.163502293	AL355481.1
ENSG00000148737	300.8138607	228.6770694	0.395438049	0.089281591	0.163526614	TCF7L2
ENSG00000276234	21.81504439	45.1550445	-1.052919968	0.089373705	0.16368379	TADA2A
ENSG00000183337	589.6164298	858.1063422	-0.54101007	0.089467002	0.163843109	BCOR
ENSG00000117410	1966.198322	2413.199356	-0.295506788	0.089491312	0.163876077	ATP6V0B
ENSG00000142751	622.300475	772.7732441	-0.312319486	0.089500771	0.163881848	GPN2
ENSG00000197361	18.56174353	5.584209627	1.722337384	0.089554693	0.163969027	FBXL22
ENSG00000134900	964.2847584	1256.533441	-0.381903558	0.089581979	0.164007427	TPP2
ENSG00000270661	4.832974932	0.743759607	2.730395042	0.089597767	0.164024774	Z99289.2
ENSG00000044446	351.0161407	465.7914652	-0.407593746	0.089679394	0.164162639	PHKA2
ENSG00000260911	18.35131512	8.060941101	1.198463586	0.089708796	0.164204891	AC135050.3
ENSG00000278384	31.73347726	4.849778236	2.716551493	0.089743629	0.164257079	AL354822.1
ENSG00000260279	23.20914956	59.37397949	-1.353340121	0.089816385	0.164378662	AC137932.1
ENSG00000137338	218.0767356	155.5108583	0.487906386	0.089831277	0.164394338	PGBD1
ENSG00000174695	1739.519003	2300.726068	-0.403438448	0.089880836	0.164473447	TMEM167A
ENSG00000090924	999.5486903	1398.383167	-0.484674023	0.089948771	0.16458617	PLEKHG2
ENSG00000232520	5.193015519	0	4.70981805	0.089989611	0.164649303	AC012507.2
ENSG00000162402	1617.675716	1214.033472	0.41416322	0.090004189	0.164664379	USP24
ENSG00000258302	11.99042207	4.371668772	1.466592141	0.090015413	0.164673316	AC025034.1
ENSG00000176371	71.87393742	111.9907519	-0.638067887	0.090044955	0.164715762	ZSCAN2
ENSG00000228492	46.48796217	21.74879856	1.091509316	0.090060175	0.164732005	RAB11FIP1P1
ENSG00000224215	9.376469963	2.893606169	1.700803762	0.090154787	0.164893455	AL606469.1
ENSG00000221571	3.983482502	0.387220594	3.363987793	0.090192552	0.164939304	RNU6ATAC35P
ENSG00000248840	3.983482502	0.387220594	3.363987793	0.090192552	0.164939304	AL645949.1
ENSG00000100154	470.2024598	320.4305919	0.552783276	0.090219339	0.164976679	TTC28
ENSG00000115459	520.5725716	395.41408	0.396591472	0.090295022	0.165099408	ELMOD3
ENSG00000276846	12.4650436	3.485113743	1.823736595	0.090299534	0.165099408	AC016590.3
ENSG00000178403	3.063429386	0	3.947263143	0.090318228	0.165099408	NEUROG2
ENSG00000225924	3.063429386	0	3.947263143	0.090318228	0.165099408	TAB2-AS1
ENSG00000255733	3.063429386	0	3.947263143	0.090318228	0.165099408	IFNG-AS1
ENSG00000067829	938.1028266	1269.932742	-0.436637726	0.090350442	0.165143035	IDH3G
ENSG00000158427	4.230124532	17.40066651	-2.042627863	0.090364741	0.165143035	TMSB15B
ENSG00000152932	3.062175737	0	3.946730459	0.09036752	0.165143035	RAB3C
ENSG00000254636	3.062175737	0	3.946730459	0.09036752	0.165143035	ARMS2
ENSG00000212994	6.917144626	16.36874752	-1.241897431	0.09038357	0.165150248	RPS26P6
novel.63	10.15185422	31.08120353	-1.617669282	0.09038418	0.165150248	-
ENSG00000261168	14.39207922	3.404837359	2.058255771	0.09066836	0.165657853	AL592424.1
ENSG00000172339	241.7930584	151.2306863	0.675239838	0.090703378	0.165710179	ALG14
ENSG00000278743	15.74997077	6.573421887	1.269826142	0.090787341	0.165851913	AC087239.1
ENSG00000089006	1618.7675	2173.85499	-0.425532414	0.09081123	0.16588389	SNX5
ENSG00000089327	1235.190466	2075.466214	-0.74871852	0.090833056	0.165912093	FXYD5
ENSG00000162384	1137.149379	913.9442392	0.315268695	0.090890601	0.166005532	CZIB
ENSG00000225580	5.47107004	14.45271364	-1.407411429	0.090932498	0.166047366	AL358942.1
ENSG00000278828	3.352909287	10.54854185	-1.661914095	0.090936437	0.166047366	HIST1H3H
ENSG00000114446	505.5150855	697.6627236	-0.46506758	0.090938694	0.166047366	IFT57

ENSG00000156110	644.9660085	801.2788649	-0.312855461	0.09093907	0.166047366	ADK
ENSG00000231995	8.685436524	1.161661783	2.94323676	0.09095476	0.166064344	AL590399.4
ENSG00000273164	4.573725143	0.723690511	2.665986611	0.090963149	0.16606799	DGCR10
ENSG00000267224	4.720830024	0.387220594	3.613960559	0.090987535	0.166097178	AC005498.1
ENSG00000228409	114.4867298	73.57041838	0.636547276	0.090991922	0.166097178	CCT6P1
ENSG00000116675	362.889574	164.9322383	1.136617495	0.091068529	0.166225337	DNAJC6
ENSG00000246560	23.98872205	12.40321256	0.944474584	0.091108848	0.166287248	UBE2D3-AS1
ENSG00000267096	2.388764651	8.397411017	-1.801006842	0.091120736	0.166297263	AC008735.1
ENSG00000137767	451.6248016	793.1618355	-0.812204	0.091138707	0.166318378	SQOR
ENSG00000288049	8.482814742	0.713078025	3.567585369	0.091162317	0.16634978	AC010889.2
ENSG00000114956	607.5292223	797.7394648	-0.392923461	0.091170863	0.16635369	DGUOK
ENSG00000228672	54.06286083	103.2454537	-0.935090777	0.091206205	0.16640649	PROB1
ENSG00000240764	6.27647095	21.12057324	-1.74602223	0.091254781	0.166481252	PCDHGC5
ENSG00000102362	188.3554711	133.0987049	0.501174693	0.091268614	0.166481252	SYTL4
ENSG00000128713	0	4.414247109	-4.727480654	0.091283627	0.166481252	HOXD11
ENSG00000198563	0	4.414247109	-4.727480654	0.091283627	0.166481252	DDX39B
ENSG00000112246	0	4.414247109	-4.727480654	0.091283627	0.166481252	SIM1
ENSG00000105982	37.31049523	20.32007397	0.88188743	0.09128775	0.166481252	RNF32
ENSG00000156381	347.7256844	514.8220677	-0.566590781	0.091292037	0.166481252	ANKRD9
ENSG00000101019	798.7966875	1018.523826	-0.350265204	0.091300241	0.166484529	UQCC1
ENSG00000158050	170.4502711	43.3136832	1.974591816	0.091450375	0.166746591	DUSP2
ENSG00000182541	1001.098921	744.7551769	0.4271863	0.091461937	0.166753329	LIMK2
ENSG00000127252	5.155192243	0.672939833	2.885256528	0.091466906	0.166753329	PLAAT1
ENSG00000254901	198.9421583	295.9101091	-0.572607798	0.091494404	0.166791756	BORCS8
ENSG00000104412	975.0263418	775.007173	0.331570527	0.091505286	0.16679989	EMC2
ENSG00000166908	544.0254836	299.4670354	0.860461879	0.091519827	0.166814693	PIP4K2C
ENSG00000277968	5.469816391	13.92971409	-1.351835695	0.091538397	0.166836835	RF02038
ENSG00000153140	366.7167759	508.2747383	-0.471125545	0.091568887	0.166880699	CETN3
ENSG00000279794	5.43833263	14.07135364	-1.369442156	0.091579999	0.166889243	AC024580.1
ENSG00000227354	80.51452662	42.90223674	0.908358427	0.091592058	0.166899513	RBM26-AS1
ENSG00000157306	18.35632971	7.725755454	1.239690074	0.091609677	0.166919912	ZFHX2-AS1
ENSG00000235770	149.3527426	47.95031297	1.639152517	0.091698053	0.167053928	LINC00607
ENSG00000230773	7.486003968	1.773238453	2.072084776	0.091700621	0.167053928	AC092650.1
ENSG00000279170	214.5203781	134.1460698	0.679489043	0.091702518	0.167053928	TSTD3
ENSG00000203814	16.66250199	4.625550555	1.84521927	0.091754735	0.167137333	HIST2H2BF
ENSG00000136560	951.0267906	726.4713887	0.388695928	0.091812536	0.167230897	TANK
ENSG00000170801	386.3129516	150.3554673	1.361070855	0.091864865	0.167306899	HTRA3
ENSG00000058866	38.63872613	19.70990996	0.964094217	0.091867142	0.167306899	DGKG
ENSG00000102890	107.3125579	67.1904142	0.675450391	0.091903271	0.167348674	ELMO3
ENSG00000213782	66.69421384	33.03035914	1.01287302	0.091907075	0.167348674	DDX47
ENSG00000102096	133.5322969	234.5152423	-0.812175977	0.091909404	0.167348674	PIM2
ENSG00000176912	21.09879544	38.69831277	-0.876071753	0.091930205	0.167374817	TYMSOS
ENSG00000277684	5.544138023	0.336469917	3.841490725	0.091962936	0.167404506	AL392048.1
ENSG00000273343	12.69531692	5.381078522	1.238675824	0.091964793	0.167404506	AC007663.4
ENSG00000286498	4.84683634	0.743759607	2.733512249	0.091971494	0.167404506	AL132982.1
ENSG00000184906	3.021845164	0	3.929419929	0.09197883	0.167404506	AMYH02020865.1
ENSG00000284685	3.021845164	0	3.929419929	0.09197883	0.167404506	AC009093.10
ENSG00000249485	18.76547641	32.44830817	-0.792058223	0.091985171	0.167404506	RBBP4P1
ENSG00000112782	27.93459474	9.061022635	1.616042095	0.092041004	0.167494385	CLIC5
ENSG00000147400	723.7423308	968.0471394	-0.419329043	0.092073986	0.16754267	CETN2
ENSG00000064655	559.4871687	184.437185	1.600328542	0.092095481	0.167570045	EYA2

ENSG00000118513	0.921306765	6.003267678	-2.715635461	0.092116324	0.167596233	MYB
ENSG00000276250	6.986451663	1.4981317	2.249644378	0.09213449	0.167617546	AC127024.6
ENSG00000225449	3.016830569	0	3.927266476	0.092181924	0.1676921	RAB6C-AS1
ENSG00000262155	69.32481939	38.99806494	0.832221385	0.092220117	0.167749832	LINC02175
ENSG00000186432	1478.938675	1912.108892	-0.370749837	0.092261184	0.167803716	KPNA4
ENSG00000223714	6.359639395	1.487519214	2.121851669	0.092262657	0.167803716	LINC02601
ENSG00000163281	427.4879073	565.0042543	-0.402778028	0.09232471	0.167904823	GNPDA2
ENSG00000235172	14.20783524	2.731897526	2.362321928	0.092371928	0.167978937	LINC01366
ENSG00000115977	708.4292299	918.0038476	-0.373684359	0.092391669	0.168003077	AAK1
ENSG00000229666	2.459325337	9.935809303	-2.023301425	0.092426465	0.168054586	MAST4-AS1
ENSG00000091640	1112.076632	1375.120989	-0.306253287	0.092471009	0.168123813	SPAG7
ENSG00000223935	5.379126054	0.713078025	2.911122994	0.092487219	0.168141518	LGALSL-DT
ENSG00000226874	4.554849141	0.693008929	2.684233918	0.092504992	0.168162063	AC005154.1
ENSG00000197483	124.7106118	167.7059782	-0.427156439	0.092514973	0.168168439	ZNF628
ENSG00000143196	4.616563014	0	4.539490029	0.092561455	0.168241162	DPT
ENSG00000108175	1570.326292	2400.926182	-0.612407367	0.092592106	0.168247098	ZMIZ1
ENSG00000231437	3.006730108	0	3.922891618	0.092594129	0.168247098	LINC01750
ENSG00000114204	3.006730108	0	3.922891618	0.092594129	0.168247098	SERPINI2
ENSG00000279771	3.006730108	0	3.922891618	0.092594129	0.168247098	AP000997.3
novel.132	4.615309366	0	4.539116029	0.0925971	0.168247098	-
ENSG00000269235	21.01333316	6.633629175	1.67097113	0.092605688	0.168250935	ZNF350-AS1
ENSG00000226314	32.97832625	15.09368762	1.129354325	0.092644989	0.168310569	ZNF192P1
ENSG00000106034	884.2348488	512.5575475	0.78710781	0.092663561	0.16833254	CPED1
ENSG00000261222	4.285498891	0.336469917	3.469877277	0.09273729	0.168403458	AC064805.1
ENSG00000288039	4.285498891	0.336469917	3.469877277	0.09273729	0.168403458	AC098969.2
ENSG00000231769	3.002969161	0	3.921270777	0.092747973	0.168403458	AL035701.1
ENSG00000265643	3.002969161	0	3.921270777	0.092747973	0.168403458	AC119868.2
ENSG00000224014	3.002969161	0	3.921270777	0.092747973	0.168403458	AL390728.3
ENSG00000286492	3.002969161	0	3.921270777	0.092747973	0.168403458	AC117509.1
ENSG00000287822	3.002969161	0	3.921270777	0.092747973	0.168403458	AC098679.6
ENSG00000227885	9.363862205	2.618499415	1.862441707	0.092816293	0.168515731	AL590652.1
ENSG00000132517	10.1152846	1.41669944	2.828968011	0.092830011	0.16852886	SLC52A1
ENSG00000214776	9.396599615	3.159256312	1.557262468	0.09287759	0.168603456	AC092821.1
ENSG00000173875	684.0845564	522.200762	0.38995814	0.092937513	0.168700449	ZNF791
ENSG00000279970	6.126858772	1.10029862	2.490031116	0.092947886	0.168707491	AC023024.2
ENSG00000170577	66.42524707	14.67360209	2.175772899	0.092955403	0.168709348	SIX2
ENSG00000278600	89.19061849	47.92086848	0.898244522	0.093019794	0.168814422	AC015871.3
ENSG00000273035	5.058162392	0.723690511	2.815618133	0.0930884	0.16892713	AC007684.1
ENSG00000116663	222.6969452	159.6694257	0.481361611	0.093135217	0.168997441	FBXO6
ENSG00000131269	320.677765	427.7693031	-0.415192345	0.093140155	0.168997441	ABCB7
ENSG00000287979	5.369025593	0.336469917	3.797896205	0.093157343	0.169016825	AC253572.2
ENSG00000118922	660.101545	465.1128401	0.504687717	0.093169396	0.16902689	KLF12
ENSG00000244165	159.2425424	99.88648678	0.673120017	0.09321428	0.16909651	P2RY11
ENSG00000245112	13.82418879	28.44147915	-1.040535774	0.093238164	0.169128028	SMARCA5-AS1
ENSG00000081803	68.15306621	121.6210768	-0.836765421	0.093255334	0.169147363	CADPS2
ENSG00000104951	81.340271	273.3429814	-1.748441992	0.093274407	0.169170147	IL4I1
ENSG00000206557	3.064683035	0	3.947835348	0.093322971	0.169223241	TRIM71
ENSG00000145103	3.064683035	0	3.947835348	0.093322971	0.169223241	ILDR1
ENSG00000276075	22.93291816	9.078651586	1.339391124	0.093323221	0.169223241	AC027682.6
ENSG00000254180	4.546002329	0.743759607	2.642361001	0.093408945	0.169366864	AC004083.1
ENSG00000138075	3.060922088	0	3.946233175	0.09347538	0.169475495	ABCG5

ENSG00000163541	983.826814	1262.531807	-0.360030364	0.093495634	0.169500389	SUCLG1
ENSG00000115507	15.68943927	4.157925182	1.934911441	0.093509422	0.169506141	OTX1
ENSG00000271335	27.36050734	44.41342107	-0.700956176	0.093511856	0.169506141	AL117336.2
ENSG00000200253	7.547717841	1.345879667	2.448599578	0.093532139	0.16953108	RNU6-529P
ENSG00000138792	99.93260029	307.3861708	-1.620929682	0.093726566	0.169871636	ENPEP
ENSG00000269054	20.18382819	6.795466212	1.569400031	0.093777443	0.169951991	AC012313.6
ENSG00000075975	749.7763045	985.2211509	-0.393734661	0.093821288	0.170019592	MKRN2
ENSG00000276488	9.560073201	22.72624246	-1.246999984	0.093878667	0.170101605	AC008735.4
ENSG00000261659	20.54512242	10.40561803	0.982064572	0.093879639	0.170101605	Z92544.2
novel.980	18.17523377	43.28261644	-1.254419397	0.093988777	0.170287477	-
ENSG00000068097	317.9180764	412.7774208	-0.376037391	0.094006454	0.170307627	HEATR6
novel.677	92.48886437	27.16890658	1.764123993	0.094019631	0.170319623	-
ENSG00000257270	17.97615947	37.214214	-1.04621123	0.094097908	0.170449539	AL928654.2
ENSG00000255182	46.99156015	28.13787417	0.742918943	0.094120873	0.170479253	AC084125.2
novel.473	523.6597766	866.618257	-0.726859009	0.09416508	0.170547435	-
ENSG00000158828	526.6527885	869.9377658	-0.723746171	0.094183201	0.170568363	PINK1
ENSG00000153071	991.1557761	1874.36048	-0.919271383	0.094257281	0.170690627	DAB2
ENSG00000284773	28.78129513	15.59186618	0.888867558	0.094313601	0.170780713	AC114490.3
ENSG00000184677	844.3060897	587.5538776	0.523368293	0.094339534	0.170815766	ZBTB40
ENSG00000233387	0	4.106018629	-4.615721553	0.094348853	0.170820735	IATPR
ENSG00000237984	30.7751305	15.86812881	0.956418699	0.094414111	0.170926974	PTENP1
ENSG00000142082	482.7772119	373.9654188	0.368283678	0.094447622	0.170975729	SIRT3
ENSG00000255382	3.03696022	0	3.935934123	0.09445759	0.17098186	AP001646.3
ENSG00000197465	5.793287351	16.08901726	-1.477173762	0.094475479	0.171000956	GYPE
ENSG00000106328	3.590704505	0.356539013	3.218570976	0.09448642	0.171000956	FSCN3
ENSG00000094880	584.1703614	742.2592883	-0.345292017	0.094487884	0.171000956	CDC23
ENSG00000124788	673.117754	437.6757967	0.621421372	0.094508344	0.171024689	ATXN1
ENSG00000077684	416.239021	682.6676427	-0.714149083	0.094514164	0.171024689	JADE1
ENSG00000254477	13.17097863	4.239485835	1.629684392	0.09464138	0.171242962	AP000640.1
ENSG00000224721	9.867388919	0.387220594	4.674075952	0.094685255	0.171310419	AC007182.1
ENSG00000233342	3.030691976	0	3.933244719	0.094716624	0.17135524	AL139393.2
ENSG00000167011	0	4.136700211	-4.625894247	0.094853941	0.171576867	NAT16
ENSG00000269124	0	4.136700211	-4.625894247	0.094853941	0.171576867	AC007193.2
ENSG00000135845	480.6619419	377.9802446	0.346712172	0.09485894	0.171576867	PIGC
ENSG00000105499	312.7920462	117.4296791	1.414558891	0.094899251	0.171637831	PLA2G4C
novel.997	4.619070312	0	4.54025673	0.094930155	0.171680648	-
ENSG00000272752	7.896404318	2.496928964	1.655785412	0.094936141	0.171680648	STAG3L5P-PVRIG2P-PILRB
ENSG00000280079	7.517487729	17.90830168	-1.254644231	0.094947245	0.171688779	AC011447.7
ENSG00000258806	2.726097019	0	3.780491529	0.095075287	0.171908346	OR11H7
ENSG00000204623	0.293240848	4.514592589	-3.798140485	0.095145429	0.172022399	ZNRD1ASP
ENSG00000104969	1600.063243	2054.011879	-0.360258018	0.095151607	0.172022399	SGTA
ENSG00000187601	905.0433967	653.8960775	0.469413705	0.095217937	0.172130337	MAGEH1
ENSG00000250362	0.308355904	3.455588036	-3.41617866	0.095233407	0.172146326	AC008592.1
ENSG00000174951	9.682247299	2.853467977	1.759088919	0.095248378	0.17216141	FUT1
ENSG00000237836	5.466055445	1.130980201	2.310803451	0.095300415	0.172243485	PHKA2-AS1
ENSG00000104093	772.1223568	542.5177538	0.509645027	0.095350412	0.17232131	DMXL2
ENSG00000015153	338.4956793	265.2266443	0.352037013	0.09535764	0.17232131	YAF2
ENSG00000165953	0	4.075337048	-4.60552429	0.095368454	0.17232131	SERPINA12
ENSG00000126214	1798.459394	1377.345461	0.384938521	0.095370006	0.17232131	KLC1
ENSG00000258938	3.354162936	9.609951872	-1.525308258	0.09548668	0.172520127	AL162311.3
ENSG00000251093	0	4.339831315	-4.698375931	0.095495541	0.172524139	AC093281.2

ENSG00000105171	485.3815183	644.1429703	-0.408721819	0.095503519	0.172526556	POP4
ENSG00000228242	63.35384104	32.16374481	0.978330264	0.095569403	0.172628799	AC093495.1
ENSG00000102174	9.645748943	3.32212083	1.556964896	0.095573547	0.172628799	PHEX
novel.754	12.70917833	4.953719736	1.351541016	0.095580451	0.172628799	-
ENSG00000263220	5.347642293	0.387220594	3.792475237	0.095586694	0.172628799	AC015727.1
ENSG00000253878	27.99226293	14.27795237	0.967963935	0.095739824	0.172893332	AC087752.3
ENSG00000215277	12.31263939	3.270214277	1.918538167	0.095747886	0.172895873	RNF212B
novel.636	6.617564264	0.743759607	3.176636925	0.095795316	0.172958426	-
ENSG00000162490	11.82068024	24.75683032	-1.070146777	0.095795841	0.172958426	DRAXIN
ENSG00000115592	5.657251845	0.713078025	2.984342242	0.095893082	0.173121963	PRKAG3
ENSG00000183023	369.3773544	175.316503	1.074091103	0.095912783	0.173145499	SLC8A1
ENSG00000205978	434.077349	597.953438	-0.462573023	0.095960096	0.173218873	NYNRIN
ENSG00000237977	7.662370047	1.161661783	2.763702232	0.095973134	0.173219254	EIF4HP2
ENSG00000065621	70.57432697	138.2927058	-0.970527169	0.095973641	0.173219254	GSTO2
ENSG00000264425	0	2.547679642	-3.927636598	0.096052049	1	MIR4653
ENSG00000286468	4.495642565	0	4.502916901	0.096101467	0.173437914	AC010251.1
novel.576	8.942321208	0	5.493954769	0.096134857	0.173474074	-
ENSG00000286330	8.942321208	0	5.493954769	0.096134857	0.173474074	AL353660.1
ENSG00000175575	817.3357673	579.9841118	0.495095627	0.096177451	0.173537791	PAAF1
ENSG00000124067	3220.811899	2631.96497	0.29128811	0.096183526	0.173537791	SLC12A4
ENSG00000135916	3964.263305	2481.258427	0.675982762	0.096212698	0.17357837	ITM2C
ENSG00000171865	603.2691955	801.1123565	-0.409335898	0.096272345	0.173663033	RNASEH1
ENSG00000276691	1.214547612	9.009116082	-2.895588877	0.096272994	0.173663033	AC004466.3
ENSG00000162664	929.5824737	615.244606	0.594964417	0.09628409	0.173670989	ZNF326
ENSG00000147996	446.5227221	269.3907812	0.729068356	0.096413535	0.173892402	CBWD5
ENSG00000133454	0.925067711	7.060987961	-2.937788584	0.096446319	0.173923803	MYO18B
ENSG00000180921	19.89943415	9.151911504	1.112175511	0.09644855	0.173923803	FAM83H
ENSG00000272769	2.989107754	0	3.915210835	0.096466525	0.173923803	AC097532.2
ENSG00000263883	2.989107754	0	3.915210835	0.096466525	0.173923803	EEF1DP7
ENSG00000163249	230.174387	343.1370692	-0.576893658	0.096470466	0.173923803	CCNYL1
ENSG00000104131	1067.110973	1549.024833	-0.53786011	0.096471835	0.173923803	EIF3J
ENSG00000143420	3059.416978	2496.875208	0.293162472	0.096478856	0.173923803	ENSA
ENSG00000285103	35.65441951	19.37087151	0.887167671	0.096484499	0.173923803	AL451123.1
ENSG00000091879	185.7429437	92.03034623	1.011723433	0.096575512	0.174064376	ANGPT2
ENSG00000215012	532.7141164	739.4601663	-0.473197974	0.096575882	0.174064376	RTL10
ENSG00000260219	26.51986172	12.93322858	1.035253799	0.096624805	0.174140473	AC106782.2
ENSG00000154222	1392.332438	1011.675613	0.46069998	0.096678394	0.174224968	CC2D1B
ENSG00000273145	33.48729484	16.72400226	1.0052789	0.096701346	0.174254242	BX537318.1
ENSG00000184564	11.07942923	1.345879667	3.013099484	0.096708382	0.174254835	SLITRK6
ENSG00000259426	11.11195318	26.20318386	-1.239698963	0.09679618	0.17440094	AC027237.4
ENSG00000249406	3.325186472	9.846076309	-1.570961936	0.096812597	0.17440804	AC015909.1
ENSG00000224877	775.2222092	1110.917705	-0.519344705	0.096813547	0.17440804	NDUFAF8
ENSG00000157404	149.5854223	17.54127858	3.090517204	0.096909051	0.174567985	KIT
ENSG00000165060	92.3868919	141.7265553	-0.619008048	0.0969222	0.174579568	FXN
ENSG00000095397	39.75033166	75.09435137	-0.919077859	0.096961198	0.174637705	WHRN
ENSG00000154479	11.655597	3.984448178	1.556184537	0.097054423	0.174793495	CCDC173
ENSG00000168556	164.0081325	233.727025	-0.510608916	0.097098579	0.174852475	ING2
ENSG00000147206	4.48930305	0	4.500965169	0.097100631	0.174852475	NXF3
ENSG00000144648	23.53193634	6.869882006	1.763979621	0.097179964	0.174983203	ACKR2
novel.220	26.05999925	7.859094266	1.729333753	0.097209396	0.17502407	-
novel.635	201.5327618	108.4357175	0.895678482	0.097281922	0.175134946	-

ENSG00000149932	2079.171805	1549.064935	0.424774322	0.09728446	0.175134946	TMEM219
ENSG00000124107	19.91197064	8.022087178	1.300898346	0.097319749	0.175186338	SLPI
ENSG00000232872	4.481781158	0	4.498662638	0.09732948	0.175191716	CTAGE3P
ENSG00000166188	379.932645	545.235362	-0.520494236	0.09738524	0.175279939	ZNF319
ENSG00000101974	462.7369693	668.7516919	-0.531714536	0.097402233	0.17529838	ATP11C
ENSG00000132185	45.26750197	18.86079619	1.268425025	0.097437641	0.175337039	FCRLA
ENSG00000196081	5.676127848	16.20459873	-1.508024741	0.097442985	0.175337039	ZNF724
ENSG00000278206	11.43542414	4.310305609	1.409445414	0.097443959	0.175337039	AL031320.2
ENSG00000150667	5.464801796	15.93064785	-1.542857065	0.097523911	0.175468748	FSIP1
ENSG00000144827	541.752297	735.4598749	-0.440948954	0.09753924	0.175484176	ABHD10
ENSG00000165507	1960.710589	1085.022302	0.853875898	0.097554503	0.175499483	DEPP1
ENSG00000136068	3400.051263	4638.590012	-0.448077599	0.097734083	0.175810373	FLNB
ENSG00000163155	137.4878435	184.4316499	-0.424924679	0.097776612	0.1758747	LYSMD1
ENSG00000229196	5.085885207	0.672939833	2.869071777	0.097830579	0.175959589	AC087071.1
ENSG00000279602	18.85713567	7.500243502	1.329873038	0.097852162	0.175986225	AC109326.1
ENSG00000151090	72.92815913	33.98529379	1.101896589	0.097871038	0.176006186	THRB
ENSG00000073756	298.2274039	772.1771693	-1.372378012	0.097876809	0.176006186	PTGS2
ENSG00000159322	1429.547168	1766.766886	-0.305525696	0.097909815	0.176053354	ADPGK
ENSG00000229766	5.9643541	1.029478846	2.504893711	0.097941484	0.176092262	AL021396.1
ENSG00000253203	0	4.044655466	-4.595281195	0.097945009	0.176092262	GUSBP3
ENSG00000188822	4.931187162	0	4.634672784	0.098035551	0.17624285	CNR2
ENSG00000272444	6.360893044	1.803920035	1.822317681	0.098055812	0.176267076	AL118558.4
ENSG00000148090	422.7896155	269.465137	0.650777617	0.098065947	0.176267966	AUH
ENSG00000167114	436.038918	608.2187951	-0.480578834	0.098073943	0.176267966	SLC27A4
ENSG00000277758	23.1041271	105.5332623	-2.192714486	0.098076661	0.176267966	FO681492.1
ENSG00000077254	1420.531167	1146.203415	0.309690557	0.098115764	0.176326047	USP33
ENSG00000156414	26.72652918	7.34799147	1.856281818	0.098136517	0.176336844	TDRD9
ENSG00000213277	0.601596751	3.995060664	-2.722273668	0.098139982	0.176336844	MARCKSL1P1
novel.1012	3.033199274	16.3004491	-2.427508368	0.098142133	0.176336844	-
ENSG00000122824	19.3296059	52.00656122	-1.427957154	0.098230555	0.176483511	NUDT10
ENSG00000225643	6.631425671	1.130980201	2.583884022	0.098292845	0.176583212	AL606491.1
ENSG00000226380	15.76222252	43.84745799	-1.471289931	0.098399853	0.176756387	AC016831.1
ENSG00000198712	57316.46076	80399.53334	-0.488240222	0.098403044	0.176756387	MT-CO2
ENSG00000286782	11.36110251	3.913628404	1.535529112	0.098409651	0.176756387	AL355377.3
ENSG00000136986	957.654982	1240.916159	-0.373986302	0.098473814	0.176859406	DERL1
ENSG00000125997	3.32769377	0.356539013	3.106645492	0.098497423	0.17688958	BPIFB9P
ENSG00000267637	3.326440121	0.356539013	3.106192495	0.098534752	0.176925377	AC040904.1
ENSG00000231414	2.402626059	8.490740032	-1.820284549	0.098551159	0.176925377	AC016700.2
ENSG00000110925	976.8645977	728.4694496	0.42347098	0.098555154	0.176925377	CSRNP2
ENSG00000225720	2.739958426	0	3.787004327	0.098563738	0.176925377	AL031846.1
ENSG00000261357	2.739958426	0	3.787004327	0.098563738	0.176925377	AC099518.2
ENSG00000204361	2.739958426	0	3.787004327	0.098563738	0.176925377	NXPE2
ENSG00000140443	1067.654165	1411.069458	-0.402309837	0.098565025	0.176925377	IGF1R
ENSG00000132024	835.2311791	607.200815	0.45993601	0.098588497	0.176955285	CC2D1A
ENSG00000276386	15.80416275	55.17035949	-1.805338345	0.098603296	0.176969622	CNTNAP3P2
ENSG00000237004	23.98120015	41.17029235	-0.779028794	0.098632513	0.177009832	ZNRF2P1
ENSG00000204792	30.47583487	4.849778236	2.657495247	0.09866448	0.17705497	LINC01291
ENSG00000251497	0	2.496928964	-3.902462001	0.098677276	1	PITPNM2-AS1
ENSG00000271428	0	2.496928964	-3.902462001	0.098677276	1	AL031281.1
ENSG00000111785	416.2980141	325.0659034	0.356510892	0.098710426	0.177125186	RIC8B
ENSG00000166341	360.1181141	163.5361906	1.13951361	0.098721648	0.17713309	DCHS1

novel.648	2.458071688	11.74790168	-2.25959911	0.098732661	0.177140617	-
ENSG00000169738	595.193147	830.5705113	-0.48104003	0.098749978	0.177145147	DCXR
ENSG00000221886	183.7770443	123.8487596	0.569121288	0.098751667	0.177145147	ZBED8
ENSG00000274414	2.72484337	10.02083758	-1.885852105	0.098755641	0.177145147	AL121772.1
ENSG00000226360	57.22059931	92.94938102	-0.701275728	0.098792066	0.177198252	RPL10AP6
ENSG00000197150	702.0956769	954.6891459	-0.443460966	0.09883134	0.177256458	ABCB8
ENSG00000279513	3.326440121	0.336469917	3.106191632	0.098892139	0.177344267	AL157902.2
ENSG00000054267	874.083053	647.9901537	0.432074126	0.098893951	0.177344267	ARID4B
ENSG00000171864	3.375546236	0	4.087399299	0.098973134	0.177474015	PRND
ENSG00000113594	654.8350631	398.1340801	0.717271081	0.099029397	0.177553672	LIFR
ENSG00000273154	11.54122953	4.371668772	1.408539316	0.099031225	0.177553672	AL121845.3
ENSG00000197915	24.95544525	10.33235811	1.280464228	0.099078629	0.177626406	HRNR
ENSG00000244021	0.308355904	3.251301057	-3.318665321	0.099110259	0.17767085	AC093591.1
ENSG00000126934	2990.906834	3813.448878	-0.350439468	0.099168511	0.17776301	MAP2K2
ENSG00000166797	491.0328145	676.9554291	-0.463557665	0.099178557	0.177768755	CIAO2A
ENSG00000178440	57.17189264	29.80729952	0.940876805	0.099229097	0.177847072	LINC00843
ENSG00000286154	4.474187994	0	4.496312767	0.099267034	0.177883875	AC009974.2
ENSG00000272984	2.72484337	0	3.779906979	0.099278142	0.177883875	AC007349.4
ENSG00000272746	2.72484337	0	3.779906979	0.099278142	0.177883875	AP005131.7
ENSG00000239877	2.72484337	0	3.779906979	0.099278142	0.177883875	IGSF11-AS1
ENSG00000116874	145.2427274	219.4561831	-0.596609561	0.099283864	0.177883875	WARS2
ENSG00000133103	539.5371067	776.3582585	-0.524732039	0.099370537	0.178026888	COG6
ENSG00000187147	1395.691183	1102.927139	0.339778725	0.099404195	0.178074909	RNF220
novel.1097	4.46791975	0	4.494387283	0.099461319	0.17815326	-
ENSG00000260868	5.812234624	18.52432628	-1.671336669	0.099461646	0.17815326	LINC01960
novel.268	3.067190332	10.98522885	-1.842509716	0.099473537	0.178159271	-
ENSG00000131375	722.5972766	585.128248	0.304661383	0.099478717	0.178159271	CAPN7
ENSG00000215859	11.83697768	4.320918095	1.455157218	0.099620111	0.178393945	PDZK1P1
ENSG00000180776	851.5179668	1137.976223	-0.418431845	0.099628689	0.178393945	ZDHHC20
ENSG00000081913	311.9596631	205.5986887	0.602342699	0.099630351	0.178393945	PHLPP1
ENSG00000156521	327.627248	483.5521509	-0.562293706	0.099695557	0.178487447	TYSND1
ENSG00000206262	4.551088195	0	4.51980099	0.099699102	0.178487447	FOXL2NB
ENSG00000242265	996.2873986	1751.847144	-0.814388311	0.09970318	0.178487447	PEG10
ENSG00000106327	27.42605343	12.23089143	1.15516192	0.099710973	0.178489099	TFR2
ENSG00000259086	3.973382041	10.71140637	-1.437266403	0.099800569	0.178637174	AL136298.3
ENSG00000244265	0.907445357	5.004470414	-2.463516775	0.099823747	0.178666353	SIAH2-AS1
novel.611	2.712235611	0	3.773959522	0.099880472	0.178743253	-
ENSG00000273272	2.712235611	0	3.773959522	0.099880472	0.178743253	U62317.4
ENSG00000138835	644.3728574	1217.192645	-0.917445978	0.09990079	0.178767299	RGS3
ENSG00000250238	2.710981963	0	3.77337074	0.099940553	0.178813821	AC253576.1
ENSG00000287322	2.710981963	0	3.77337074	0.099940553	0.178813821	AC068189.2
ENSG00000143337	1498.324437	1147.706684	0.384441447	0.100048353	0.178994371	TOR1AIP1
ENSG00000175416	1932.163563	1474.191753	0.390173544	0.100059823	0.179002565	CLTB
ENSG00000078053	96.53656875	59.54023042	0.695335255	0.100100925	0.179052169	AMPH
ENSG00000171595	5.090899802	0.356539013	3.720857468	0.100107424	0.179052169	DNAI2
ENSG00000257433	19.72453518	8.612310483	1.195260884	0.100108225	0.179052169	AC004241.1
ENSG00000174165	434.953168	595.7122236	-0.453524626	0.100123602	0.179061772	ZDHHC24
ENSG00000250320	123.6793399	34.78622543	1.827880961	0.100132101	0.179061772	AC113383.1
ENSG00000009830	548.2768934	715.1309994	-0.383173826	0.100134271	0.179061772	POMT2
ENSG00000168575	1292.078777	773.5355099	0.739896036	0.100323135	0.179387155	SLC20A2
ENSG00000128383	4.532140921	0.723690511	2.654788258	0.10035124	0.179425062	APOBEC3A

ENSG00000057593	3.065936683	0	3.948456615	0.100361547	0.179431142	F7
ENSG00000137822	212.7308455	290.9887744	-0.452262131	0.100382772	0.17945674	TUBGCP4
ENSG00000006606	20.33386729	3.9336975	2.368207694	0.100411579	0.179495888	CCL26
ENSG00000280143	113.0003514	35.12847472	1.682996845	0.100427803	0.179503926	AP000892.3
ENSG00000265749	30.33575368	14.64638814	1.040236054	0.100429893	0.179503926	AC135178.3
ENSG00000272144	22.71371421	9.367966845	1.265725758	0.100443246	0.179515442	AC025171.4
ENSG00000185917	456.7295788	322.0105073	0.504047877	0.100486537	0.179580459	SETD4
ENSG00000276102	0	2.862924587	-4.099334776	0.100506317	0.179591102	MIR6747
ENSG00000238405	0	2.862924587	-4.099334776	0.100506317	0.179591102	RNA5SP311
ENSG00000156411	966.9632842	1325.152769	-0.45472004	0.100551941	0.17966027	ATP5MPL
ENSG00000140519	1.188078446	8.894433702	-2.893279209	0.100605187	0.179743046	RHCG
ENSG00000138617	160.7796912	115.1748292	0.482261868	0.100666322	0.179839904	PARP16
ENSG00000122068	1696.418457	1287.255631	0.398003733	0.100687663	0.17986566	FYTTD1
ENSG00000108654	15980.32416	12303.15564	0.377250313	0.100704101	0.179880946	DDX5
ENSG00000113318	265.064021	364.1822811	-0.458058945	0.100710067	0.179880946	MSH3
ENSG00000237133	7.262070157	1.487519214	2.308999384	0.100759449	0.179956778	AC020594.1
ENSG00000138083	5.240796714	0	4.722621289	0.100818611	0.180050065	SIX3
ENSG00000238278	61.94838176	96.15116849	-0.633007576	0.10085162	0.180087214	ALG1L6P
novel.222	1.522903516	6.003267678	-1.987864787	0.100853276	0.180087214	-
ENSG00000100053	3.052075276	0	3.942486765	0.10096253	0.180269912	CRYBB3
ENSG00000286923	5.72906618	1.436768536	1.997054311	0.100974273	0.18027849	AC004522.4
ENSG00000204138	1076.082262	731.9429834	0.555903849	0.101032023	0.18036301	PHACTR4
ENSG00000237651	47.53555998	29.49449471	0.684671074	0.101035497	0.18036301	C2orf74
novel.867	10.97244146	2.659793482	2.069757748	0.101051848	0.180379804	-
ENSG00000225655	10.34186825	2.731897526	1.894350762	0.101136273	0.180518103	BX255923.1
novel.1080	8.868070847	23.04734799	-1.380312625	0.101177754	0.18056714	-
ENSG00000234494	54.68702361	30.37886639	0.843445544	0.101181945	0.18056714	SP2-AS1
ENSG00000118579	796.0073504	1048.440812	-0.39736732	0.101184596	0.18056714	MED28
ENSG00000131697	388.5134588	540.3947832	-0.475462872	0.10125121	0.180673606	NPHP4
ENSG00000163029	599.825454	817.0283895	-0.446088578	0.101260668	0.180678073	SMC6
ENSG00000173917	234.0679177	538.7282776	-1.202799799	0.101335151	0.180798556	HOXB2
ENSG00000091317	2486.651329	1724.776435	0.527630257	0.101344847	0.180803438	CMTM6
ENSG00000239959	5.074531097	0.743759607	2.799991898	0.101367178	0.180830861	ENPP7P2
ENSG00000176641	76.66260702	120.5404149	-0.651156266	0.101411534	0.180895238	RNF152
ENSG00000132792	720.5515988	918.9138713	-0.350762961	0.101421243	0.180895238	CTNNBL1
ENSG00000169891	122.8164836	57.96138997	1.084783424	0.101424153	0.180895238	REPS2
ENSG00000267419	26.64167981	14.22720169	0.900904412	0.10147856	0.180979851	AC011477.1
ENSG00000188060	32.56355205	59.48074664	-0.867114002	0.101571469	0.181133113	RAB42
ENSG00000141380	1745.250793	2121.548382	-0.281772971	0.101588716	0.181151436	AC091021.1
ENSG00000237813	1.232169966	5.83911889	-2.251581253	0.101628188	0.181209385	AC002066.1
ENSG00000166454	879.4866398	689.9141547	0.349901223	0.101662553	0.181258221	ATMIN
ENSG00000128805	890.0908896	1543.674593	-0.794228752	0.101754544	0.181409785	ARHGAP22
ENSG00000095321	711.548745	1020.283627	-0.519731059	0.101790575	0.181461571	CRAT
ENSG00000255248	2543.714709	2088.630556	0.284378016	0.10184284	0.181542287	MIR100HG
ENSG00000175772	12.24870295	30.38361828	-1.316294515	0.101886853	0.181585696	LINC01106
novel.364	178.3552378	111.6448725	0.676650708	0.101887417	0.181585696	-
ENSG00000091542	1470.875534	2029.785271	-0.46452572	0.10188816	0.181585696	ALKBH5
ENSG00000072182	5.177829192	1.080229524	2.262124276	0.101907115	0.18160702	ASIC4
ENSG00000260751	12.12144298	23.03305827	-0.925551033	0.101980111	0.18172464	AC008870.2
ENSG00000286859	12.39831513	4.12852787	1.565646781	0.102043109	0.18182443	AL158214.2
ENSG00000181523	612.631448	791.5810594	-0.369333396	0.102112939	0.181936379	SGSH

ENSG00000172840	226.9105584	311.303279	-0.455725466	0.102222374	0.182118872	PDP2
ENSG00000225916	0	4.198063374	-4.646290587	0.102305578	0.18222962	AC007879.2
ENSG00000226012	0	4.198063374	-4.646290587	0.102305578	0.18222962	AP001434.1
novel.371	0	4.198063374	-4.646290587	0.102305578	0.18222962	-
ENSG00000231628	27.51610302	9.701868223	1.514652457	0.102373615	0.182338308	AL133406.2
ENSG00000267493	17.99761405	33.53786591	-0.893930717	0.102385656	0.182347254	CIRBP-AS1
ENSG00000140104	191.1262726	282.9360532	-0.565204186	0.102405458	0.182370018	CLBA1
ENSG00000213621	2.142122621	7.530925084	-1.820356476	0.102465828	0.182465023	RPSAP54
ENSG00000129932	433.4876052	598.1008229	-0.464926537	0.102481779	0.18248092	DOHH
ENSG00000170540	1966.817653	2975.435062	-0.597354144	0.102492973	0.182488346	ARL6IP1
ENSG00000276603	2.124500267	7.042203135	-1.736864282	0.102501047	0.182490215	AL109614.1
ENSG00000177169	816.3342955	1175.53306	-0.525824931	0.102597652	0.182649691	ULK1
ENSG00000137575	2241.030329	3345.071067	-0.577920153	0.102649432	0.182728184	SDCBP
ENSG00000224971	11.20138987	21.80759318	-0.957232011	0.102655809	0.182728184	SUMO2P3
ENSG00000268894	9.118473823	36.23245057	-1.992614161	0.102678652	0.182747827	PLCE1-AS1
ENSG00000226530	13.18763207	3.832196145	1.768398002	0.102680912	0.182747827	AL158055.1
ENSG00000166262	102.7290883	59.79599159	0.784462179	0.102707849	0.182783247	FAM227B
ENSG00000170581	2891.016434	3642.648122	-0.333344016	0.102735575	0.182808147	STAT2
ENSG00000249978	11.24422774	3.873490212	1.526530389	0.102735913	0.182808147	TRGV7
ENSG00000270614	7.777991167	1.069617038	2.858732889	0.10274792	0.182816992	AC011451.2
ENSG00000159214	133.0295257	84.57075483	0.653463607	0.102786647	0.182873374	CCDC24
ENSG00000223754	8.503984579	0	5.422527366	0.102798119	0.18288126	AC008073.1
ENSG00000207870	14.17885878	43.11816368	-1.600855833	0.102818027	0.182904153	MIR221
ENSG00000082805	916.5701085	1184.099302	-0.369578028	0.102834202	0.182920403	ERC1
ENSG00000241015	143.349584	84.16714237	0.768394928	0.102859594	0.182953045	TPM3P9
ENSG00000110871	478.2253537	671.5999763	-0.490017383	0.102872917	0.182964215	COQ5
ENSG00000138138	1070.736894	795.3524657	0.428567445	0.102904494	0.183006647	ATAD1
ENSG00000228696	13.8166669	4.503851709	1.63754234	0.102910862	0.183006647	ARL17B
ENSG00000272447	84.10781005	53.94206907	0.637516103	0.103009495	0.183169509	AL135925.1
ENSG00000136052	139.6395962	86.04620171	0.700279012	0.103189634	0.183477271	SLC41A2
ENSG00000135899	573.8236052	397.6490829	0.528354171	0.103212127	0.183504706	SP110
ENSG00000170293	29.38826249	88.08646861	-1.585522103	0.103231789	0.183527105	CMTM8
ENSG00000266124	1.81990531	8.700887602	-2.25686189	0.103277439	0.183595698	MIR5587
ENSG00000066248	16.47094958	5.219369879	1.648143076	0.103285005	0.183596586	NGEF
ENSG00000243433	0.909952655	4.657388011	-2.357597547	0.103400336	0.183789021	AC010973.1
ENSG00000274340	3.617173671	0.387220594	3.227614437	0.103415049	0.183802599	AC032011.1
ENSG00000124299	1711.125546	1354.36103	0.337274239	0.103434822	0.183825167	PEPD
ENSG00000113389	218.3079893	654.8362585	-1.585034714	0.103459795	0.183856972	NPR3
ENSG00000121101	11.30816418	2.598430319	2.137956982	0.103468165	0.18385927	TEX14
ENSG00000253570	838.0683798	646.3741895	0.375159968	0.103558683	0.184007532	RNF5P1
ENSG00000130584	325.0659762	137.1269813	1.244142556	0.103576971	0.184027442	ZBTB46
ENSG00000124116	4.564878331	16.52801602	-1.855252061	0.103676425	0.184191547	WFDC3
ENSG00000143222	1145.969965	867.437069	0.401599994	0.103686174	0.184196272	UFC1
ENSG00000163827	18.42208927	6.124838129	1.588491885	0.103702744	0.184213113	LRRC2
ENSG00000229222	6.991466258	1.783850939	1.966236968	0.103776265	0.184323143	KRT18P4
ENSG00000206538	5868.833283	3167.116136	0.889861317	0.103778875	0.184323143	VGLL3
ENSG00000109736	1382.296175	1887.731449	-0.449488597	0.103828549	0.184377132	MFSD10
ENSG00000139624	758.9329847	954.685071	-0.330829926	0.103832739	0.184377132	CERS5
ENSG00000285905	3.254625787	0	4.038789738	0.103837658	0.184377132	AL033527.6
ENSG00000255548	3.254625787	0	4.038789738	0.103837658	0.184377132	AP003043.1
ENSG00000100473	21.44790919	56.3908159	-1.397271549	0.103861199	0.184406329	COCH

ENSG00000203965	167.5577232	120.5740974	0.472998325	0.103924156	0.184505501	EFCAB7
ENSG00000203684	8.080007555	1.702418679	2.218510546	0.104021898	0.184666411	IBA57-DT
ENSG00000171180	4.719576376	0	4.573874909	0.104079006	0.184742548	OR2M4
novel.947	4.719576376	0	4.573874909	0.104079006	0.184742548	-
ENSG00000114656	18.65716372	35.37974078	-0.922435717	0.104099946	0.184767093	KIAA1257
ENSG00000253865	3.576843098	0.356539013	3.213542799	0.104109957	0.184772238	AC131025.1
ENSG00000139746	1424.45409	1100.13479	0.372549704	0.104142928	0.184789824	RBM26
ENSG00000174628	82.014651	55.39877831	0.564100605	0.104144119	0.184789824	IQCK
ENSG00000189042	133.2440001	91.16000749	0.54510758	0.104154746	0.184789824	ZNF567
ENSG00000274918	6.927245087	1.365948763	2.311625775	0.104154881	0.184789824	AC116447.1
ENSG00000140297	1.230916317	6.358522421	-2.37027129	0.104159252	0.184789824	GCNT3
ENSG00000252061	11.41654814	3.403553089	1.772150319	0.104177155	0.184789824	RNU6-415P
ENSG00000248975	0	4.077777192	-4.613226026	0.104177434	0.184789824	AL133372.2
ENSG00000215456	0	4.077777192	-4.613226026	0.104177434	0.184789824	BCRP4
ENSG00000234281	13.53728746	4.972632957	1.440640635	0.104183878	0.184789824	LANCL1-AS1
ENSG00000196634	0	2.567748738	-3.937873429	0.10427351	1	LUADT1
ENSG00000242948	0	2.567748738	-3.937873429	0.10427351	1	EPS15P1
ENSG00000286791	0	2.567748738	-3.937873429	0.10427351	1	AC079949.6
ENSG00000259113	5.055655094	0.723690511	2.814378393	0.10427669	0.184941817	AL118556.1
ENSG00000278924	2.976499995	0	3.909578555	0.10433844	0.185026074	AC078883.3
ENSG00000223727	2.976499995	0	3.909578555	0.10433844	0.185026074	AC034195.1
ENSG00000223274	2.975246347	0	3.909029613	0.104395802	0.185079101	RNA5SP498
ENSG00000255250	2.975246347	0	3.909029613	0.104395802	0.185079101	AP003059.2
ENSG00000276868	2.975246347	0	3.909029613	0.104395802	0.185079101	AL353626.2
ENSG00000225335	3.313832363	9.029056784	-1.440829665	0.104396837	0.185079101	AC016027.1
ENSG00000243449	792.8195108	590.6879066	0.424473503	0.104436804	0.185137323	C4orf48
ENSG00000255843	3.356670233	0.356539013	3.117281128	0.104456712	0.185147349	AP000593.3
ENSG00000176381	3.356670233	0.356539013	3.117281128	0.104456712	0.185147349	PRR18
ENSG00000247081	29.93957074	56.89691001	-0.928262728	0.104494153	0.185197183	BAALC-AS1
ENSG00000156113	235.1856503	506.0877635	-1.105462399	0.104500043	0.185197183	KCNMA1
ENSG00000134982	661.9654156	515.9410908	0.359457244	0.104507042	0.185197183	APC
ENSG00000124370	235.7020138	314.97839	-0.417976115	0.10451334	0.185197183	MCEE
ENSG00000165650	730.4353571	561.4315781	0.3797443	0.10452295	0.18520158	PDZD8
ENSG00000233073	3.680141193	0.336469917	3.249461609	0.104540632	0.185220279	AC005009.2
ENSG00000242085	12.38570737	5.4318292	1.195675439	0.104664995	0.185427974	RPS20P33
ENSG00000138604	710.3389391	450.7813589	0.655840501	0.104799266	0.185653194	GLCE
ENSG00000197321	1034.483279	2220.699142	-1.102077817	0.104834526	0.185702994	SVIL
ENSG00000252933	0.293240848	3.352802412	-3.356865277	0.104842938	0.185705235	RNU6-1223P
ENSG00000253921	10.51161008	2.812173909	1.892530515	0.104886115	0.185769047	AC091982.1
ENSG00000180336	21.59215077	8.376186046	1.372174466	0.104897659	0.185776828	MEIOC
ENSG00000272667	101.5789753	70.61763241	0.523038292	0.104964645	0.185882793	AC012306.2
ENSG00000144460	5.054401445	0.356539013	3.711107657	0.105053906	0.186024003	NYAP2
ENSG00000105717	61.15154293	26.54500079	1.210308882	0.105058704	0.186024003	PBX4
ENSG00000163964	389.0492092	556.9376282	-0.518101339	0.10507367	0.186037823	PIGX
ENSG00000086848	146.4759806	219.608692	-0.584861278	0.105109407	0.186080108	ALG9
ENSG00000160194	778.5217692	991.020962	-0.348416879	0.105111877	0.186080108	NDUFV3
ENSG00000128271	3.368024343	11.52470148	-1.773141299	0.105133559	0.186105811	ADORA2A
ENSG00000129151	4.308207111	0	4.439734073	0.105147275	0.186110812	BBOX1
ENSG00000242600	4.236392776	14.18719189	-1.748886642	0.10515071	0.186110812	MBL1P
ENSG00000117222	322.9173169	406.6848053	-0.332336865	0.105185771	0.186158971	RBBP5
ENSG00000219102	1.22840902	6.817847059	-2.480124811	0.10519225	0.186158971	HNRNPA3P12

ENSG00000148730	2229.280257	1656.082889	0.428654263	0.105240194	0.186229196	EIF4EBP2
ENSG00000232882	9.611757884	20.22802922	-1.06862517	0.105246268	0.186229196	PHKA1P1
ENSG00000188342	534.1713717	759.8392455	-0.508318359	0.105254714	0.186231458	GTF2F2
ENSG00000226363	3.978396636	17.594341	-2.150423457	0.105308891	0.186314627	HAGLROS
ENSG00000264772	5.129976726	1.060160428	2.263647361	0.105341903	0.186360342	AC016876.2
ENSG00000214900	34.76334286	69.60522175	-0.998851351	0.105363463	0.186385793	LINC01588
ENSG00000181392	12.04461406	4.666844622	1.369029033	0.105475062	0.186570506	SYNE4
ENSG00000262136	1.511549406	6.602947593	-2.121223177	0.1055468	0.186684689	AC092115.3
ENSG00000100124	389.7530794	534.6704536	-0.456455225	0.105612493	0.186788167	ANKRD54
ENSG00000197254	4.306953462	0	4.439337235	0.105658315	0.18681834	AP000445.1
ENSG00000143105	4.306953462	0	4.439337235	0.105658315	0.18681834	KCNA10
ENSG00000276412	4.306953462	0	4.439337235	0.105658315	0.18681834	AL591926.2
ENSG00000211695	4.306953462	0	4.439337235	0.105658315	0.18681834	TRGV9
ENSG00000164327	877.9941673	592.9427777	0.566813893	0.105734731	0.18694073	RICTOR
ENSG00000158691	169.6142413	123.5382194	0.458187985	0.105764	0.18696327	ZSCAN12
ENSG00000158161	304.0262615	391.8444859	-0.365411424	0.105768845	0.18696327	EYA3
ENSG00000040531	633.2890282	508.1033162	0.31801751	0.105769067	0.18696327	CTNS
ENSG00000279858	3.325186472	0.336469917	3.105717264	0.105851229	0.187095773	AC068880.4
novel.651	22.87837017	9.904999327	1.216495913	0.105859357	0.187097412	-
ENSG00000261971	75.97519233	43.51137326	0.806863325	0.1059245	0.187199811	MMP25-AS1
ENSG00000269019	20.54888337	10.63943072	0.943689683	0.106000999	0.187322266	HOMER3-AS1
ENSG00000242951	2.73619748	10.28289171	-1.913846894	0.106074284	0.187428037	AC007182.2
ENSG00000180304	1859.727704	2438.052876	-0.390612432	0.10607528	0.187428037	OAZ2
ENSG00000287031	3.631035079	0.336469917	3.232430798	0.106118143	0.187491021	AC093307.1
ENSG00000094963	2209.746608	242.4599378	3.187924096	0.106172547	0.187574388	FMO2
ENSG00000224881	0	2.598430319	-3.953034584	0.106196239	1	AC068279.1
ENSG00000234695	0	2.598430319	-3.953034584	0.106196239	1	AC002076.1
ENSG00000157538	875.0555024	1184.271355	-0.436337343	0.106230561	0.187660387	VPS26C
novel.742	224.3521949	148.6280992	0.593575839	0.106235671	0.187660387	-
ENSG00000142530	14.02200944	5.697479343	1.293863483	0.106249526	0.187667181	AC020909.1
ENSG00000213856	3.020591515	9.222602883	-1.601438456	0.106253964	0.187667181	VDAC1P2
ENSG00000168152	84.45606925	59.86018008	0.496120057	0.106295778	0.187728273	THAP9
ENSG00000268707	7.41795058	1.069617038	2.791194304	0.106385186	0.187864709	AL158151.4
ENSG00000269427	44.60655645	27.13804942	0.715914136	0.106387493	0.187864709	AC024075.3
ENSG00000115145	831.2160967	669.0959365	0.313036159	0.106442713	0.187949444	STAM2
ENSG00000120805	1397.685913	1977.348281	-0.50052098	0.106468553	0.187971248	ARL1
ENSG00000114544	638.4655325	784.0403866	-0.296285128	0.106469531	0.187971248	SLC41A3
ENSG00000226085	16.33645246	41.63359819	-1.352212658	0.106482344	0.187973801	UQCRFS1P1
ENSG00000268034	0	2.527610546	-3.917693859	0.106483002	1	AC243960.2
ENSG00000176340	1205.697927	1664.546956	-0.465338764	0.106485447	0.187973801	COX8A
ENSG00000176894	38.17392034	75.47622496	-0.98433983	0.106601256	0.188165448	PXMP2
ENSG00000185219	761.0227548	580.0203497	0.391928186	0.106650133	0.188238934	ZNF445
ENSG00000264057	5.702597014	1.41669944	2.001339761	0.106693393	0.188302496	AC103810.1
ENSG00000251369	133.1532385	178.4372781	-0.421098598	0.106825675	0.188522251	ZNF550
ENSG00000275560	19.03231937	8.061069495	1.229591489	0.10683242	0.188522251	AC008115.3
ENSG00000065325	0.615458159	3.893559308	-2.678611572	0.106842243	0.188526781	GLP2R
ENSG00000104228	405.2768931	285.4275064	0.504812335	0.106872444	0.188567265	TRIM35
ENSG00000137819	15.63177108	34.59931061	-1.149734545	0.106915109	0.18861623	PAQR5
ENSG00000259503	2.697120555	0	3.766734739	0.106943755	0.18861623	AC048383.1
ENSG00000275025	2.697120555	0	3.766734739	0.106943755	0.18861623	AC002401.3
ENSG00000270402	2.697120555	0	3.766734739	0.106943755	0.18861623	AC011458.1

ENSG00000251892	2.697120555	0	3.766734739	0.106943755	0.18861623	RNU7-84P
ENSG00000244378	2.697120555	0	3.766734739	0.106943755	0.18861623	RPS2P45
ENSG00000258839	98.27370506	65.29791705	0.589049092	0.106984151	0.18867467	MC1R
ENSG00000181544	53.75630057	92.58145883	-0.784737321	0.107042871	0.188755584	FANCB
ENSG00000173145	404.4218452	588.4068699	-0.541467031	0.107044562	0.188755584	NOC3L
ENSG00000127311	147.3286923	109.0372424	0.433348764	0.10720084	0.189018325	HELB
ENSG00000280109	8.660221007	2.51699806	1.786513552	0.107223392	0.189045259	PLAC4
ENSG00000188124	0	2.578361223	-3.943026472	0.107295366	1	OR2AG2
ENSG00000230131	4.443957882	0	4.486914655	0.1074241	0.189373425	LINC02641
ENSG00000279375	4.443957882	0	4.486914655	0.1074241	0.189373425	AC244517.7
ENSG00000272247	4.206162664	0.693008929	2.57200786	0.107466314	0.18942777	AC080013.5
ENSG00000158793	569.4357489	428.3480749	0.410161691	0.10746951	0.18942777	NIT1
ENSG00000204463	0.925067711	5.696323468	-2.635067696	0.107775058	0.189953449	BAG6
ENSG00000273254	3.354162936	0.387220594	3.116412323	0.107979878	0.190288625	AF129075.2
ENSG00000185303	3.354162936	0.387220594	3.116412323	0.107979878	0.190288625	SFTPA2
ENSG00000280486	13.51583289	5.788368213	1.230060322	0.108060301	0.190417437	AC005329.3
ENSG00000262700	11.81781694	2.853467977	2.050192195	0.108077224	0.190422947	AC133552.3
ENSG00000064012	275.5039585	389.7197984	-0.500459324	0.108078087	0.190422947	CASP8
ENSG00000224003	3.854968889	0.356539013	3.321850834	0.108115676	0.190473955	YES1P1
ENSG00000127452	427.5264854	573.6678637	-0.423933503	0.1081217	0.190473955	FBXL12
ENSG00000136048	736.7298488	590.3257166	0.319279746	0.108144798	0.190501729	DRAM1
ENSG00000279656	45.66522364	25.94660514	0.821356226	0.108251139	0.190676124	AL132780.4
ENSG00000166246	22.03396361	11.81170499	0.899034431	0.108282407	0.190707106	C16orf71
novel.1020	9.8306771	31.77981626	-1.688330344	0.108288821	0.190707106	-
ENSG00000141738	18.49494379	9.193205571	1.00008947	0.108295277	0.190707106	GRB7
ENSG00000228477	2.694613258	10.64760306	-1.974596531	0.108298089	0.190707106	AL663070.1
ENSG00000227165	3.344062475	0.387220594	3.112675255	0.108324015	0.190739834	WDR11-AS1
ENSG00000144488	13.4720261	31.503986	-1.231099316	0.108344635	0.190763213	ESPNL
ENSG00000255992	4.834228581	1.080229524	2.165343225	0.108421972	0.190886444	AC131009.1
ENSG00000134326	353.6807499	205.0487448	0.785571037	0.1084372	0.190900318	CMPK2
ENSG00000168959	4.226292315	0	4.413303839	0.108444652	0.190900502	GRM5
ENSG00000221890	196.5658154	108.6113306	0.857799827	0.10856306	0.191091525	NPTXR
ENSG00000230314	4.622831258	0	4.541477524	0.108567876	0.191091525	ELOVL2-AS1
ENSG00000105258	244.9411946	177.2295363	0.466992989	0.108594019	0.191124591	POLR2I
ENSG00000236867	0.293240848	3.250145181	-3.318830932	0.108624596	0.191165456	Z98885.1
ENSG00000159556	0.925067711	5.920551149	-2.683760573	0.108792737	0.191448394	ISL2
ENSG00000132471	2394.868028	1763.813711	0.441447944	0.108818948	0.191481549	WBP2
ENSG00000253210	64.96836037	40.15959833	0.698219396	0.10884414	0.191506755	AC040970.1
ENSG00000115020	1000.522794	759.8984587	0.396791447	0.108848014	0.191506755	PIKFYVE
ENSG00000182463	60.12710809	24.31269449	1.309058236	0.108857581	0.191510618	TSHZ2
ENSG00000184619	24.7765006	11.24856724	1.143098832	0.108873725	0.191523599	KRBA2
ENSG00000166482	1325.587364	595.6219802	1.153881039	0.108879702	0.191523599	MFAP4
ENSG00000227344	2.435363469	7.95828387	-1.709374435	0.109001463	0.191720393	HAUS6P1
ENSG00000259687	9.88354416	3.220619475	1.618530511	0.109006337	0.191720393	LINC01220
ENSG00000164751	780.7514348	627.1299439	0.316097453	0.109056485	0.191791631	PEX2
ENSG00000221843	26.11572961	41.38403594	-0.664520902	0.109061605	0.191791631	C2orf16
ENSG00000223546	22.24152871	40.22121828	-0.852652855	0.109103733	0.191840691	LINC00630
ENSG00000244752	4.806505766	0.713078025	2.747985152	0.109104271	0.191840691	CRYBB2
ENSG00000259479	4.806505766	12.02544858	-1.323564816	0.109112092	0.191841461	SORD2P
ENSG00000163510	530.7486725	426.8820718	0.314458166	0.109149654	0.191894515	CWC22
ENSG00000272994	64.7264482	43.55523587	0.569686238	0.109282899	0.192115772	AC012360.3

ENSG00000183666	8.161922351	2.833398881	1.517671978	0.109290481	0.192116103	GUSBP1
ENSG00000255823	4.559863736	13.32746564	-1.54306618	0.109324786	0.192152341	MTRNR2L8
ENSG00000279348	61.50470236	33.80377284	0.859229452	0.109325888	0.192152341	AC012513.3
ENSG00000128218	3.675126598	0.356539013	3.247812429	0.109356303	0.192192797	VPREB3
ENSG00000260163	0.586481695	4.33153058	-2.859428897	0.10939837	0.192253723	AC012508.1
novel.67	4.582571956	0.693008929	2.69418127	0.109479186	0.192382734	-
ENSG00000272707	5.804641461	1.436768536	2.012343688	0.109554619	0.192500147	AC046143.2
ENSG00000112137	162.587174	73.33437549	1.151023501	0.10956082	0.192500147	PHACTR1
ENSG00000239636	19.47162491	8.000733813	1.287789772	0.109573636	0.192509646	AC004865.2
ENSG00000214199	0	2.537067156	-3.922775266	0.109588349	1	EEF1A1P12
ENSG00000183576	1613.342477	1287.936731	0.325158174	0.109609536	0.192559697	SETD3
ENSG00000175183	285.0090854	409.4523463	-0.522399089	0.109631078	0.192584518	CSRP2
ENSG00000128346	99.86806691	64.84560888	0.62463983	0.109643189	0.192592773	C22orf23
ENSG00000285830	4.80274482	0.387220594	3.636622672	0.10968856	0.192659443	AL109628.2
ENSG00000158296	289.7899324	154.4368748	0.908425753	0.109710402	0.19268478	SLC13A3
ENSG00000111199	78.10437902	123.9448328	-0.668358631	0.109737345	0.192709439	TRPV4
ENSG00000224738	18.97304152	8.733880934	1.126866622	0.109739277	0.192709439	AC099850.1
novel.117	4.18854031	0	4.400976029	0.109787211	0.192780585	-
ENSG00000176903	1838.903645	1449.283138	0.343349448	0.109811276	0.19280981	PNMA1
novel.72	7.30232946	1.702418679	2.066996315	0.109859017	0.192880599	-
ENSG00000163364	22.60672609	78.73518478	-1.800777016	0.109876701	0.192895021	LINC01116
ENSG00000272255	5.22449928	0.336469917	3.756034443	0.10988208	0.192895021	AC113361.1
ENSG00000145721	3.644896486	0.387220594	3.237275705	0.109897061	0.192908287	LIX1
ENSG00000112851	2302.630876	1803.759487	0.352133449	0.109906646	0.192912078	ERBIN
ENSG00000213066	273.1079727	378.6277686	-0.470654655	0.109940073	0.192957715	FGFR1OP
ENSG00000235204	27.69218437	11.30162967	1.299065655	0.109962403	0.192983869	AL162724.2
ENSG00000124253	4.57881101	0	4.528221667	0.109970985	0.192985893	PCK1
ENSG00000251703	0	2.466247382	-3.886947261	0.109977962	1	RNU6-998P
ENSG00000206775	0	2.466247382	-3.886947261	0.109977962	1	SNORD37
ENSG00000168124	0	2.466247382	-3.886947261	0.109977962	1	OR1F1
ENSG00000226562	0.923814062	4.697526203	-2.356791737	0.110001505	0.193026415	CYP4F26P
ENSG00000163162	878.3845802	1124.675613	-0.35652488	0.110011974	0.193027595	RNF149
ENSG00000260038	9.263071407	2.934900236	1.67380627	0.110022146	0.193027595	AC009090.1
ENSG00000260060	8.72193488	2.91483114	1.592849047	0.110024466	0.193027595	AC009088.1
ENSG00000175203	2158.037597	2671.937025	-0.308140984	0.110092638	0.193134154	DCTN2
ENSG00000224903	10.8615502	4.33153058	1.331119338	0.110163184	0.193244864	AC005534.1
ENSG00000125046	13.67708391	2.852312101	2.258170845	0.11018105	0.193263156	SSUH2
ENSG00000188997	328.5371724	258.5057223	0.345670565	0.110222179	0.193322247	KCTD21
ENSG00000230185	2.739958426	7.988965452	-1.543358884	0.110231433	0.193325426	C9orf147
ENSG00000198952	1549.143888	1923.365632	-0.312144401	0.110284166	0.193399679	SMG5
ENSG00000236824	0.909952655	7.030434774	-2.953188382	0.110288659	0.193399679	BCYRN1
ENSG00000227492	9.937736141	2.793260689	1.818117074	0.11037232	0.193533323	AL138921.1
ENSG00000141905	2192.963689	2892.31401	-0.399249462	0.110395136	0.193560267	NFIC
ENSG00000124126	2874.223181	1622.426961	0.825169853	0.11040719	0.193568338	PREX1
ENSG00000164307	1037.27271	1378.943809	-0.410762903	0.110433443	0.193601301	ERAP1
ENSG00000179165	3.298717306	0.356539013	3.095873386	0.110447652	0.193613146	PXT1
ENSG00000010318	102.1600864	69.43102124	0.558469867	0.1104618	0.193624883	PHF7
ENSG00000226976	51.59580014	78.03618686	-0.597015271	0.110479485	0.193642818	COX6A1P2
ENSG00000272636	4.932440811	0	4.635103449	0.110497346	0.193647996	DOC2B
ENSG00000211795	4.932440811	0	4.635103449	0.110497346	0.193647996	TRAV8-6
ENSG00000174744	687.1880317	907.5518844	-0.401501358	0.11060843	0.193829598	BRMS1

ENSG00000143367	44.5127459	88.08847639	-0.987218814	0.110621486	0.193839403	TUFT1
ENSG00000275476	14.18136608	1.426156051	3.311708854	0.110637458	0.193854315	AC009318.4
novel.800	11.94160069	26.4946353	-1.153286921	0.110657652	0.193876625	-
ENSG00000166747	1280.226622	1661.537133	-0.37611659	0.110743676	0.194014259	AP1G1
ENSG00000134824	4474.12409	2325.886406	0.943743804	0.110776382	0.194058471	FADS2
ENSG00000273024	17.15438986	7.418811243	1.203037924	0.110935605	0.194324296	INTS4P2
ENSG00000177822	16.94564238	34.47902443	-1.027835715	0.110953732	0.194342946	TENM3-AS1
ENSG00000179044	47.68914657	26.10831379	0.8751767	0.111005712	0.194420884	EXOC3L1
ENSG00000127527	588.202594	737.7100797	-0.326700546	0.111027337	0.194445651	EPS15L1
ENSG00000125505	88.7438898	166.4093082	-0.907341631	0.111053923	0.194479104	MBOAT7
ENSG00000261609	28.83555839	51.07208084	-0.826187846	0.111110065	0.194564306	GAN
ENSG00000100577	237.9641314	318.7879613	-0.422563085	0.111138764	0.194601445	GSTZ1
ENSG00000162601	1298.718893	821.5939983	0.660769798	0.111192411	0.194682258	MYSM1
ENSG00000210174	38.68231946	71.07580117	-0.877248564	0.11127611	0.194815674	MT-TR
ENSG00000112837	1076.364804	398.4527587	1.433505385	0.111462289	0.195128478	TBX18
ENSG00000186825	2.755073482	0	3.794240047	0.111527656	0.195220713	C2orf27B
ENSG00000271966	11.44427095	4.637318915	1.301239198	0.111530004	0.195220713	AC021321.1
ENSG00000151005	7.699837317	0.672939833	3.482387915	0.111576992	0.195289803	TKTL2
ENSG00000213244	0.61420451	6.632473299	-3.438028765	0.111595634	0.195309274	HIST2H3DP1
ENSG00000259182	4.252761481	0.743759607	2.544942263	0.111613082	0.195326652	AC019254.1
ENSG00000183808	251.9093398	357.3932702	-0.504593437	0.111783237	0.195611253	RBM12B
ENSG00000260267	65.6326399	92.29317105	-0.493297681	0.111818349	0.195659517	AC026471.1
ENSG00000259436	23.78860756	8.45646243	1.497760067	0.111828881	0.195664769	AC010247.2
ENSG00000280333	0.586481695	4.800183434	-3.006197248	0.11192338	0.195816925	AC090617.9
ENSG00000226084	264.9180268	402.8286192	-0.604835623	0.111948777	0.195848171	AC113935.1
ENSG00000135541	561.1344028	403.5861415	0.475603433	0.11198508	0.195898489	AHI1
ENSG00000104687	573.2627381	829.2250974	-0.532984242	0.112104957	0.196094991	GSR
ENSG00000281920	5.759296292	1.386017859	2.027794874	0.112119041	0.196106423	AC007389.5
ENSG00000142494	26.10946137	48.41177378	-0.888221796	0.112189147	0.196215836	SLC47A1
ENSG00000114423	372.4475658	528.8585827	-0.50632716	0.11221859	0.196254118	CBLB
ENSG00000219481	228.5671327	172.4177941	0.406700195	0.112243993	0.196285332	NBPF1
ENSG00000085415	507.967119	724.0778657	-0.511709233	0.112290018	0.196352602	SEH1L
ENSG00000227676	0.307102255	3.149799701	-3.280052791	0.112306211	0.1963677	LINC01068
ENSG00000110400	162.1094326	102.3356188	0.661254551	0.112320742	0.196379892	NECTIN1
ENSG00000244009	1.215801261	4.94310725	-2.032052029	0.112415214	0.196531841	B3GAT3P1
ENSG00000273073	4.819113525	0.672939833	2.791178574	0.112428837	0.196542432	AC073869.3
ENSG00000102359	1345.012636	2372.90338	-0.819053896	0.112469891	0.196600972	SRPX2
ENSG00000152954	8.017253496	0	5.336386608	0.112480342	0.196606013	NRSN1
ENSG00000261270	35.1837724	19.391069	0.859142674	0.112488698	0.196607391	AC012181.2
novel.918	561.4900474	96.95773828	2.534122074	0.112507567	0.196627142	-
ENSG00000115652	1355.15672	1734.943101	-0.356284559	0.112517735	0.196631686	UXS1
ENSG00000227189	0	2.486316478	-3.897186899	0.112528605	1	AC092535.1
ENSG00000253508	0	2.486316478	-3.897186899	0.112528605	1	AC004080.2
ENSG00000267575	89.00318303	129.065561	-0.534893774	0.112601367	0.196764603	AC006504.5
ENSG00000184659	10.23606285	2.629111901	1.983045694	0.112621986	0.196787397	FOXD4L4
ENSG00000172717	4.775022005	0.356539013	3.628811718	0.112689896	0.196892817	FAM71D
ENSG00000253768	0	3.749479616	-4.484182065	0.112724399	0.196939856	AC008663.1
ENSG00000266922	3.575589449	0.356539013	3.21290582	0.112768334	0.197003367	AC008543.3
ENSG00000204237	417.6128815	532.1689355	-0.349992043	0.112820631	0.197081478	OXLD1
ENSG00000250056	10.03093378	3.647978261	1.467905172	0.112912708	0.197229063	LINC01018
ENSG00000167107	346.0015557	546.414991	-0.658863199	0.112939822	0.197262342	ACSF2

ENSG00000237943	19.55228606	8.438833479	1.202254129	0.112946946	0.197262342	PRKCQ-AS1
ENSG00000240288	38.60946494	20.21613247	0.940758561	0.113010995	0.197347046	GHRLOS
ENSG00000101546	249.1065285	327.6219309	-0.39569281	0.113015549	0.197347046	RBFA
ENSG00000278389	9.013922079	1.782695063	2.334725685	0.113023598	0.197347046	AC099518.6
novel.52	6.94612109	2.180528143	1.681065288	0.113025828	0.197347046	-
ENSG00000249693	0	3.718798035	-4.473010295	0.113041705	0.197361504	THEGL
ENSG00000259511	12.37309961	5.299646263	1.214710574	0.113177022	0.197576424	UBE2Q2L
ENSG00000120217	73.86867042	40.44287742	0.873499367	0.113180013	0.197576424	CD274
ENSG00000258790	1.833766717	6.430626464	-1.817619888	0.113195561	0.19759029	AL121594.1
ENSG00000223198	0.293240848	2.853467977	-3.133821471	0.113315804	0.197786893	RNU2-22P
ENSG00000230612	5.117368968	1.029478846	2.279819865	0.113334941	0.197794678	AC004039.1
ENSG00000285437	14.27588863	38.10881298	-1.411578334	0.11333549	0.197794678	POLR2J3
ENSG00000117697	657.5314438	466.5833854	0.494234169	0.113367653	0.19783752	NSL1
ENSG00000146109	457.3383258	573.4799211	-0.32658832	0.113454315	0.197975455	ABT1
ENSG00000271503	2.756327131	0	3.794852471	0.11350042	0.198029307	CCL5
ENSG00000279791	2.756327131	0	3.794852471	0.11350042	0.198029307	AC018892.3
ENSG00000213713	95.26411165	60.80809827	0.649041253	0.113510904	0.1980343	PIGCP1
ENSG00000126950	29.85278355	6.245252705	2.260094756	0.113553655	0.198095582	TMEM35A
ENSG00000263823	15.15213496	5.13793762	1.541538028	0.113583595	0.198134509	AC009831.1
ENSG00000255146	2.753819833	0	3.793672017	0.113633186	0.198194402	AP004247.2
ENSG00000271850	2.753819833	0	3.793672017	0.113633186	0.198194402	LINC02343
ENSG00000272795	2.71348926	0	3.774482936	0.113734378	0.198344266	AC126283.1
ENSG00000080293	2.71348926	0	3.774482936	0.113734378	0.198344266	SCTR
ENSG00000273720	5.306058071	0.356539013	3.781626068	0.113753072	0.198363553	AC091812.2
ENSG00000165879	89.99569122	57.67006693	0.642463927	0.113848216	0.198516142	FRAT1
ENSG00000280149	19.80652125	9.436346474	1.073329052	0.113866283	0.198526685	AC004877.2
ENSG00000179178	7.649762288	2.191140629	1.812631338	0.113869545	0.198526685	TMEM125
ENSG00000237629	2.709728314	0	3.77269418	0.113936892	0.198564153	UQCRHP2
ENSG00000272239	2.709728314	0	3.77269418	0.113936892	0.198564153	AC011373.1
ENSG00000257335	2.709728314	0	3.77269418	0.113936892	0.198564153	MGAM
ENSG00000229019	2.709728314	0	3.77269418	0.113936892	0.198564153	AL161457.1
ENSG00000273082	2.709728314	0	3.77269418	0.113936892	0.198564153	Z73429.1
ENSG00000158764	2.709728314	0	3.77269418	0.113936892	0.198564153	ITLN2
ENSG00000155729	452.4887687	357.1830415	0.341648034	0.113990197	0.198643726	KCTD18
ENSG00000146963	1006.834299	761.7848516	0.402392386	0.114019878	0.198682123	LUC7L2
ENSG00000198836	1112.119327	1371.530764	-0.302568434	0.114081226	0.198775692	OPA1
ENSG00000118432	307.7663209	1514.3525	-2.298834892	0.114092803	0.198782532	CNR1
ENSG00000126259	4.303192516	0	4.438188366	0.114178897	0.198919192	KIRREL2
ENSG00000133858	1040.54121	1339.595122	-0.364253051	0.114214777	0.19896836	ZFC3H1
ENSG00000207864	5.127469429	1.049547942	2.269017043	0.114260138	0.199034036	MIR27B
ENSG00000160404	278.4821116	207.6796077	0.42232761	0.114291147	0.199074704	TOR2A
ENSG00000179950	221.1189242	338.9364505	-0.616736491	0.114300057	0.199076877	PUF60
ENSG00000143207	512.3291183	631.8367512	-0.30247059	0.114418513	0.199269835	COP1
ENSG00000004864	428.6986231	332.7531432	0.36609534	0.114427442	0.199272026	SLC25A13
ENSG00000108091	749.5422108	942.5311164	-0.330632542	0.114544506	0.199462522	CCDC6
ENSG00000260062	0	3.780161198	-4.495343611	0.114556897	0.199470728	GOLGA2P11
ENSG00000131626	1245.195196	997.2607945	0.320390225	0.114579599	0.199496887	PPFIA1
ENSG00000279853	4.208669961	0.693008929	2.573501312	0.114615388	0.199545828	AC004453.2
ENSG00000224831	57.60299211	85.85527133	-0.576634839	0.114636063	0.19954728	TMEM183B
ENSG00000272088	5.390480164	0.713078025	2.914004458	0.11463876	0.19954728	AL512413.1
ENSG00000279329	26.13836656	13.8895759	0.907757461	0.114645259	0.19954728	AC020910.5

ENSG00000230373	36.38041292	18.44404989	0.988718009	0.114646944	0.19954728	GOLGA6L5P
ENSG00000254094	1.186824798	5.381078522	-2.167413408	0.114686889	0.199603435	AC078852.2
ENSG00000148985	262.0259927	330.6825928	-0.335564868	0.114709981	0.199630251	PGAP2
ENSG00000258317	8.178291056	2.822786395	1.522614725	0.114729605	0.19965103	AC034102.7
ENSG00000167333	278.2121488	190.2335843	0.546882959	0.11476587	0.199700761	TRIM68
novel.543	41.17334202	68.26568222	-0.729177591	0.114785574	0.199721671	-
ENSG00000243902	0.61420451	4.361056287	-2.835634532	0.114831041	0.199766009	FP325335.1
novel.549	102.8489689	195.1085556	-0.923975131	0.114833202	0.199766009	-
ENSG00000259916	4.16583209	0	4.393471005	0.114834123	0.199766009	AL845331.2
ENSG00000129484	492.0812268	657.6205734	-0.417857955	0.114875093	0.199823902	PARP2
ENSG00000066422	561.514814	441.0781592	0.348109832	0.114930811	0.199907439	ZBTB11
ENSG00000275714	0	2.446178286	-3.876472282	0.114950528	1	HIST1H3A
ENSG00000230189	0	2.446178286	-3.876472282	0.114950528	1	AC008267.2
ENSG00000286736	4.428842826	0	4.482145299	0.114966763	0.199956584	AC005753.2
ENSG00000271555	2.728604316	0	3.781689852	0.114984919	0.199961392	AC113139.1
ENSG00000141668	2.728604316	0	3.781689852	0.114984919	0.199961392	CBLN2
ENSG00000267776	5.411863464	1.060160428	2.342555782	0.115028196	0.200023261	AC006116.10
ENSG00000183665	147.0759243	196.9481322	-0.422430821	0.11505649	0.200059074	TRMT12
ENSG00000178188	1631.744522	1316.448773	0.309968618	0.115077266	0.200081808	SH2B1
ENSG00000147231	17.93590017	36.65582816	-1.028128114	0.115088337	0.200087666	RADX
ENSG00000252206	1.774560141	7.65365141	-2.093978977	0.115141719	0.200167079	RNU7-40P
ENSG00000065989	287.4624978	494.367251	-0.78194894	0.115241783	0.200327631	PDE4A
ENSG00000114124	2.723589721	0	3.779310269	0.115256107	0.200330166	GRK7
ENSG00000283156	6.640272484	1.742556871	1.910112432	0.115258663	0.200330166	AC068620.3
ENSG00000166136	321.9623751	230.2193208	0.484795646	0.115282686	0.200346682	NDUFB8
ENSG00000112308	3446.51611	2658.231807	0.374582019	0.115283588	0.200346682	C6orf62
ENSG00000231811	4.271637483	0	4.428015579	0.115311324	0.200381481	AL159166.1
ENSG00000206503	449.952315	2392.716925	-2.410818692	0.115321607	0.200385946	HLA-A
ENSG00000112186	148.5898384	99.2367797	0.584246819	0.115387155	0.200486435	CAP2
ENSG00000119979	424.7380185	557.0391642	-0.391124374	0.115452276	0.200586171	FAM45A
ENSG00000240303	6.080259955	1.406086955	2.096323445	0.115520468	0.200691226	ACAD11
ENSG00000174032	418.7760139	272.3931148	0.620141644	0.11561217	0.200837071	SLC25A30
ENSG00000265190	1.843867178	7.632426439	-2.054454447	0.115619879	0.200837071	ANXA8
ENSG00000278146	4.558610087	0.713078025	2.669303744	0.11565992	0.200893193	AC012409.4
ENSG00000146729	1097.19463	900.0154111	0.286023192	0.115717417	0.200979625	NIPSNAP2
ENSG00000153339	757.1889832	612.149864	0.306737407	0.115731604	0.200990829	TRAPPC8
ENSG00000137492	982.5041957	775.3430475	0.341381492	0.115767175	0.201039167	THAP12
ENSG00000132478	795.5023444	625.516312	0.346883418	0.11582262	0.201122008	UNK
ENSG00000270020	7.156193493	1.803920035	1.99455471	0.115835888	0.201131606	AC009108.3
ENSG00000186976	32.99727352	17.20108424	0.94071172	0.115891417	0.201214575	EFCAB6
ENSG00000232633	7.930395377	1.548882377	2.394862816	0.115904696	0.201224184	AC079465.1
ENSG00000166086	2435.47722	1924.912271	0.339456961	0.115946775	0.201283787	JAM3
ENSG00000267546	11.24422774	4.330374705	1.378399431	0.116019238	0.201396126	AC015802.4
ENSG00000078061	848.996615	1074.592616	-0.340163514	0.116038736	0.201416516	ARAF
ENSG00000260367	5.410609815	13.11744646	-1.277586996	0.116079635	0.201474046	AC109460.1
ENSG00000100678	9.191613078	35.05092963	-1.930502229	0.116098414	0.201493179	SLC8A3
ENSG00000145216	302.3035282	391.0539228	-0.37152773	0.11618682	0.201619919	FIP1L1
ENSG00000105732	350.3162605	453.2165867	-0.371590313	0.116186961	0.201619919	ZNF574
ENSG00000236114	12.7683849	2.691759334	2.2224396	0.116202694	0.201633754	AL450326.2
ENSG00000141449	7.610685363	16.0818724	-1.083180525	0.116233051	0.20166539	GREB1L
ENSG00000181817	621.9953659	466.9354297	0.41308888	0.11623645	0.20166539	LSM10

ENSG00000130783	16.21295344	6.980711577	1.228318894	0.116264288	0.201700219	CCDC62
ENSG00000165240	289.4278708	223.5416531	0.372985576	0.116277982	0.201710506	ATP7A
ENSG00000057935	539.1704419	689.3869987	-0.354460478	0.116374626	0.20186468	MTA3
ENSG00000262049	46.04127692	28.39175595	0.699226551	0.11643244	0.20195148	AC139530.2
ENSG00000124216	107.1165169	223.1328008	-1.059778782	0.116461437	0.201987315	SNAI1
ENSG00000159200	4520.467324	2775.281691	0.703936177	0.116468649	0.201987315	RCAN1
novel.219	2.698374204	0	3.767228459	0.11663801	0.202159583	-
ENSG00000242829	2.698374204	0	3.767228459	0.11663801	0.202159583	RPS26P21
ENSG00000272218	2.698374204	0	3.767228459	0.11663801	0.202159583	AC108865.2
ENSG00000235974	2.698374204	0	3.767228459	0.11663801	0.202159583	VN2R19P
ENSG00000287379	2.698374204	0	3.767228459	0.11663801	0.202159583	AC006144.2
ENSG00000229677	2.698374204	0	3.767228459	0.11663801	0.202159583	AC018644.1
ENSG00000286506	2.698374204	0	3.767228459	0.11663801	0.202159583	FP325317.2
ENSG00000231544	2.698374204	0	3.767228459	0.11663801	0.202159583	RSL24D1P11
ENSG00000260685	2.698374204	0	3.767228459	0.11663801	0.202159583	AC027104.1
ENSG00000137269	134.4814419	69.18600095	0.956929817	0.116652062	0.20217045	LRRC1
ENSG00000171940	862.2973201	1459.759101	-0.759280126	0.116700706	0.202241264	ZNF217
ENSG00000155858	145.8675027	193.2245351	-0.405779723	0.116769824	0.202300094	LSM11
ENSG00000270424	2.695866906	0	3.766029196	0.116776516	0.202300094	AC145285.5
ENSG00000167858	2.695866906	0	3.766029196	0.116776516	0.202300094	TEKT1
ENSG00000250677	2.695866906	0	3.766029196	0.116776516	0.202300094	AC114781.3
ENSG00000252950	2.695866906	0	3.766029196	0.116776516	0.202300094	RNA5SP490
ENSG00000228794	376.2102513	257.9380082	0.544564438	0.116781371	0.202300094	LINC01128
ENSG00000057757	1116.079573	823.9513814	0.437537694	0.116801674	0.202321773	PITHD1
ENSG00000270104	0.293240848	2.873537073	-3.142148403	0.11681689	0.202334641	AL670729.2
ENSG00000059758	986.140732	696.1843786	0.502747557	0.116850852	0.202379974	CDK17
ENSG00000130876	16.55830625	2.975038428	2.485275227	0.116999685	0.202624237	SLC7A10
ENSG00000239665	444.7804443	242.352037	0.876594843	0.117061671	0.202718075	AL157392.3
ENSG00000178401	14.00814803	35.7991028	-1.358144273	0.117143599	0.202846431	DNAJC22
ENSG00000183161	251.6027791	192.89158	0.382350137	0.117172839	0.20288354	FANCF
ENSG00000285677	7.108341027	1.467450118	2.292299508	0.117198497	0.202914445	AL355388.3
ENSG00000118307	8.460177794	1.682349583	2.297852242	0.11723269	0.20296012	CASC1
ENSG00000137760	229.7518493	176.9301221	0.376268418	0.117251441	0.202979057	ALKBH8
ENSG00000151718	688.5083267	942.3017853	-0.45283413	0.117291645	0.203035127	WWC2
ENSG00000174099	774.1662475	1202.739107	-0.635824762	0.117336228	0.203095079	MSRB3
ENSG00000261715	0.307102255	4.106018629	-3.6523132	0.117346741	0.203095079	LINC01477
ENSG00000256894	17.8404087	8.794088222	1.031477801	0.11734973	0.203095079	AC022509.3
ENSG00000181904	1272.110927	1550.661356	-0.285621764	0.117380112	0.20313413	C5orf24
ENSG00000206228	5.464801796	13.54596112	-1.312327034	0.117419295	0.203188405	HNRNPA1P4
ENSG00000142235	12.23860249	33.87878375	-1.467927416	0.117427672	0.203189368	LMTK3
ENSG00000268731	4.706968617	0	4.570034287	0.117469168	0.203234098	AC010615.3
ENSG00000278847	4.706968617	0	4.570034287	0.117469168	0.203234098	AC006157.1
ENSG00000271550	8.211028466	2.884149558	1.511311651	0.117495055	0.203265351	BNIP3P11
ENSG00000283657	4.705714968	0	4.569665244	0.117509763	0.203277258	AC074101.1
ENSG00000259456	34.76585016	21.3602937	0.699281252	0.117551454	0.203335841	ADNP-AS1
ENSG00000253194	7.250716047	2.211209725	1.731529265	0.117583705	0.203378086	AL137009.1
ENSG00000273687	4.793898008	1.080229524	2.155489063	0.117612535	0.20340453	AC004223.4
ENSG00000197858	2039.031416	2756.891279	-0.435271693	0.117614651	0.20340453	GPAA1
ENSG00000196756	348.3854646	569.2064873	-0.708528222	0.117633176	0.20341977	SNHG17
ENSG00000196937	1721.003404	2151.543307	-0.322094942	0.117639122	0.20341977	FAM3C
ENSG00000164252	438.1427466	349.836895	0.324156356	0.117658883	0.2034404	AGGF1

ENSG00000161091	1354.052853	1894.81311	-0.484582503	0.117672577	0.203444437	MFSD12
ENSG00000266975	0.615458159	5.144954086	-3.070359541	0.117676879	0.203444437	FARSA-AS1
ENSG00000244063	4.600194309	0.723690511	2.672794065	0.117713091	0.203493501	AC104653.2
ENSG00000119321	949.2115887	1158.082391	-0.286804508	0.117762698	0.203565712	FKBP15
ENSG00000202111	2.158491326	14.06677732	-2.711458198	0.117780701	0.203583287	VTRNA1-2
ENSG00000174013	321.6637206	419.3386076	-0.382809847	0.117815533	0.203629947	FBXO45
ENSG00000230148	29.45742698	68.75320112	-1.223408163	0.117959229	0.203864747	HOXB-AS1
ENSG00000169925	734.5243751	587.6566289	0.321728886	0.118155558	0.204190472	BRD3
ENSG00000174206	130.0838256	93.18815521	0.478794321	0.118184071	0.204226163	C12orf66
ENSG00000213801	28.43490249	14.0335272	1.021594317	0.118292043	0.204399146	ZNF321P
ENSG00000274214	5.193015519	0.356539013	3.747831979	0.118407025	0.20458422	AP005212.2
ENSG00000273091	9.231587646	1.548882377	2.610198996	0.118428615	0.204607916	AP000255.1
ENSG00000152284	460.5498015	341.6415064	0.430543302	0.118469547	0.204665025	TCF7L1
ENSG00000123908	781.6525284	1084.417792	-0.472223605	0.118503791	0.204710571	AGO2
ENSG00000151067	16.47492399	34.65006129	-1.074945194	0.118516025	0.204718093	CACNA1C
ENSG00000213995	1289.192546	1043.915268	0.304284317	0.118547684	0.204759166	NAXD
ENSG00000197978	8.394702974	20.04869163	-1.25643829	0.118560838	0.204760619	GOLGA6L9
ENSG00000006042	1934.127694	1348.07299	0.520682346	0.118564288	0.204760619	TMEM98
ENSG00000157483	1696.773037	2612.881834	-0.622767777	0.118604179	0.204815898	MYO1E
ENSG00000277728	0.61420451	4.433031935	-2.855646493	0.11862488	0.204836845	AC097641.2
ENSG00000177106	632.6107054	419.9648252	0.590966978	0.118632078	0.204836845	EPS8L2
ENSG00000239462	0.293240848	4.075337048	-3.642437562	0.118649586	0.204853462	AC091212.1
ENSG00000270362	42.85506053	25.61039202	0.738745563	0.118814738	0.205124971	HMGN3-AS1
ENSG00000185972	5.487438745	14.50218005	-1.402556605	0.118918319	0.205284074	CCIN
ENSG00000165006	926.9313669	1152.004755	-0.313460658	0.118930744	0.205284074	UBAP1
ENSG00000135002	691.347437	512.2063465	0.432133727	0.118936603	0.205284074	RFK
ENSG00000248896	6.845330292	1.406086955	2.272920001	0.1189385	0.205284074	AC105001.1
ENSG00000005102	37.37557095	9.822282798	1.934589588	0.118953877	0.205292553	MEOX1
novel.675	68.57772706	124.9166729	-0.866634952	0.118959216	0.205292553	-
ENSG00000152669	11.90377742	4.586568238	1.365004418	0.11898603	0.205325189	CCNO
ENSG00000287180	5.419456628	1.10029862	2.316305456	0.119010647	0.205354029	AC106818.2
ENSG00000243679	230.4615891	142.6528162	0.692665217	0.11904874	0.205406117	AC018638.5
ENSG00000233056	6.228618484	1.049547942	2.555461476	0.119080586	0.205447421	ERVH48-1
ENSG00000269439	109.3410685	63.46087529	0.785488834	0.119133103	0.20552438	AC010618.4
ENSG00000234882	29.58551367	16.21533961	0.860880707	0.119169514	0.205573543	EIF3EP1
ENSG00000074966	7.960625489	1.813376645	2.137500374	0.119258196	0.205712865	TXK
ENSG00000204758	8.360640644	22.05330262	-1.396988313	0.119270664	0.205720714	AC008429.1
ENSG00000269533	4.559863736	0.713078025	2.669742552	0.119291917	0.205743713	AC003002.3
ENSG00000272632	5.842464737	0.693008929	3.050872795	0.119344483	0.205820711	AC097504.2
ENSG00000248712	24.18500431	11.88368064	1.026932205	0.11942945	0.20595194	CCDC153
ENSG00000267141	1.54052587	7.051531351	-2.197325423	0.11943643	0.20595194	AC012615.4
ENSG00000125952	1378.380313	1048.646978	0.394436056	0.119501487	0.206044393	MAX
ENSG00000147912	167.3918848	125.4958513	0.414050364	0.119505906	0.206044393	FBXO10
ENSG00000234336	4.238900074	0.743759607	2.540141051	0.119524204	0.206062266	JAZF1-AS1
ENSG00000021776	674.0087486	865.4238401	-0.360413583	0.119587515	0.206157736	AQR
ENSG00000274628	5.119876265	0.672939833	2.882161463	0.119610439	0.206169771	AL669942.1
ENSG00000169902	516.4117375	807.9799965	-0.645601538	0.119616107	0.206169771	TPST1
ENSG00000100601	152.3350279	213.6401214	-0.487461785	0.119618303	0.206169771	ALKBH1
ENSG00000224046	15.29193141	5.178075812	1.545963313	0.119633353	0.206182033	AC005076.1
ENSG00000175548	240.3039321	180.6349685	0.410893198	0.119649486	0.206196159	ALG10B
ENSG00000256448	3.28360225	0.387220594	3.090042879	0.119694698	0.206260394	AP000763.3

ENSG00000227706	0	3.741307276	-4.489116392	0.119707083	0.206268054	AL713998.1
ENSG00000156469	280.8369568	364.2674034	-0.375928803	0.119730213	0.206294229	MTERF3
ENSG00000140795	13.89983534	4.495679368	1.614024557	0.119832818	0.206457324	MYLK3
ENSG00000176182	172.4302023	256.2012179	-0.570283637	0.119853512	0.206479286	MYPOP
ENSG00000170542	1048.535769	749.5386174	0.484666847	0.119873337	0.206499745	SERPINB9
ENSG00000203804	27.76142013	10.77045778	1.367156168	0.119888969	0.206512981	ADAMTSL4-AS1
ENSG00000264061	4.146956088	0	4.387189651	0.11994913	0.206602914	FGF7P1
ENSG00000104219	893.0693837	562.1712062	0.667339329	0.120055485	0.206772393	ZDHHC2
ENSG00000287104	81.68136431	46.88308889	0.802418954	0.120123329	0.206843866	AC097382.3
ENSG00000184708	576.2050068	427.2533672	0.432037532	0.120130554	0.206843866	EIF4ENIF1
novel.1025	4.001104856	0	4.332999873	0.12014364	0.206843866	-
ENSG00000275569	4.001104856	0	4.332999873	0.12014364	0.206843866	AL355073.2
ENSG00000117215	4.001104856	0	4.332999873	0.12014364	0.206843866	PLA2G2D
novel.655	2.125753916	7.469433526	-1.804598428	0.120144751	0.206843866	-
ENSG00000274833	3.979721556	0.336469917	3.363178122	0.120210768	0.206943808	AC110285.5
ENSG00000230212	8.019547331	2.160459047	1.897591324	0.120227209	0.20694976	AP000688.1
ENSG00000103066	573.0723406	748.9678596	-0.386465835	0.120230156	0.20694976	PLA2G15
ENSG00000239096	3.28360225	0.356539013	3.090030403	0.120268435	0.207001936	RF01210
ENSG00000166263	257.5688576	181.7781022	0.501704496	0.120283096	0.207013456	STXBP4
ENSG00000188386	2.75758078	0	3.795520218	0.120335103	0.207075527	PPP3R2
ENSG00000287926	2.75758078	0	3.795520218	0.120335103	0.207075527	AC113208.4
ENSG00000170456	296.4264595	392.0308397	-0.403560098	0.120347272	0.207082751	DENND5B
ENSG00000110002	275.282718	156.9900424	0.808776307	0.120398403	0.207157015	VWA5A
ENSG00000182264	2.752566185	0	3.793147077	0.120613632	0.207513593	IZUMO1
ENSG00000149043	4.884588345	0.713078025	2.768737171	0.120627539	0.207523778	SYT8
ENSG00000205090	79.3560379	114.1062396	-0.521693857	0.120659058	0.207564257	TMEM240
ENSG00000109472	1508.45806	788.2457576	0.936361045	0.120725165	0.207664229	CPE
ENSG00000249476	15.71089384	6.981995847	1.156823448	0.120758174	0.207682172	AC008467.1
ENSG00000211638	4.314475354	0	4.441846243	0.120761488	0.207682172	IGLV8-61
ENSG00000000005	4.314475354	0	4.441846243	0.120761488	0.207682172	TNMD
ENSG00000074582	608.2280779	896.4243053	-0.559771775	0.120767571	0.207682172	BCS1L
ENSG00000275805	2.961384939	0	3.902618597	0.120809678	0.207740833	AC090206.1
ENSG00000162620	10.69682297	21.63527205	-1.018500856	0.12084935	0.207795299	LRRIQ3
ENSG00000156500	71.90284261	99.19587081	-0.463329506	0.120907894	0.207882205	FAM122C
ENSG00000156973	355.843986	486.354856	-0.450359443	0.120920311	0.207889797	PDE6D
ENSG00000203644	78.61367578	52.36083566	0.590172796	0.120979063	0.207977043	AC083799.1
ENSG00000132768	319.4237136	403.9488923	-0.339143544	0.1210002	0.207999616	DPH2
ENSG00000164758	228.9141383	178.4471671	0.358497889	0.121043123	0.208059635	MED30
ENSG00000274678	2.743719372	0	3.788920956	0.12110904	0.208159167	AC106886.3
ENSG00000100271	132.9481807	173.863039	-0.388203548	0.12112047	0.208165041	TTLL1
ENSG00000274124	5.362757349	0.713078025	2.907293548	0.121159118	0.20821769	AC074029.3
ENSG00000100350	286.4472535	431.1404021	-0.5905057	0.121181696	0.208242718	FOXRED2
ENSG00000273189	5.132484024	1.110911105	2.232517213	0.12121842	0.208292048	AC010619.2
ENSG00000165471	4.002358504	0	4.333438182	0.121250392	0.208333207	MBL2
ENSG00000238005	4.294345703	11.58619304	-1.435662267	0.121289094	0.208385924	AL391832.2
ENSG00000122707	554.2979736	727.943252	-0.393540805	0.121403738	0.208569101	RECK
novel.676	5.535291211	0.387220594	3.839557702	0.121447533	0.208598195	-
novel.878	0.308355904	4.167381792	-3.672193101	0.121458971	0.208598195	-
ENSG00000286141	0.307102255	4.044655466	-3.632532276	0.121460206	0.208598195	AC097480.2
ENSG00000273198	2.737451129	0	3.785942628	0.121460817	0.208598195	AL096803.4
ENSG00000243480	2.737451129	0	3.785942628	0.121460817	0.208598195	AMY2A

ENSG00000183542	4.624084907	0	4.541933401	0.121547101	0.208732582	KLRC4
ENSG00000149798	0.601596751	3.607840069	-2.57712157	0.121593083	0.208797745	CDC42EP2
ENSG00000153531	256.966162	593.6377347	-1.207941155	0.121659711	0.208898351	ADPRHL1
ENSG00000124334	3.37178529	0.336469917	3.123169033	0.121696382	0.208947509	IL9R
ENSG00000272430	14.16026751	5.554683921	1.335346898	0.121790276	0.209088877	LINC02637
ENSG00000286285	3.369277992	0.336469917	3.12223669	0.121794814	0.209088877	AL138778.1
ENSG00000161664	15.68943927	28.23834804	-0.847784281	0.121959502	0.209357768	ASB16
ENSG00000226803	22.98181081	10.28173583	1.15604036	0.121983375	0.209384915	ZNF451-AS1
ENSG00000172867	4.610223499	0	4.537755982	0.122017527	0.209429699	KRT2
ENSG00000201264	4.538480436	0.723690511	2.655867629	0.122030495	0.209438121	SNORD73B
ENSG00000185924	25.26003986	5.931163635	2.095994927	0.122055741	0.209467614	RTN4RL1
ENSG00000135341	874.7797139	1067.671443	-0.287394916	0.122082032	0.209498896	MAP3K7
novel.940	4.607787473	0	4.537029778	0.12210002	0.209515925	-
ENSG00000257534	3.033199274	0.356539013	2.972347989	0.122203541	0.209679711	AC023794.5
ENSG00000258788	0.894837599	4.82025253	-2.41148988	0.122244245	0.209735701	CKS1BP1
ENSG00000159882	98.72192869	68.53244106	0.529734436	0.122380861	0.20995623	ZNF230
ENSG00000197863	121.3915514	88.67086568	0.454795046	0.12239725	0.209956877	ZNF790
ENSG00000275070	3.330201067	0.387220594	3.107550994	0.1223974	0.209956877	RF00017
ENSG00000196353	1.233423615	6.53212782	-2.412548254	0.122431911	0.210002209	CPNE4
ENSG00000124721	4.795151656	0.713078025	2.745092776	0.1224599	0.210022488	DNAH8
ENSG00000228487	4.795151656	0.713078025	2.745092776	0.1224599	0.210022488	AL450263.1
ENSG00000234076	6.353371151	1.41669944	2.156759048	0.122480932	0.210044693	TPRG1-AS1
ENSG00000149201	28.41255028	16.81501953	0.755663373	0.122492077	0.21004994	CCDC81
ENSG00000274750	27.71044746	15.18342062	0.869579923	0.122516507	0.210077967	HIST1H3E
ENSG00000263916	5.101000263	1.10029862	2.230635937	0.122535682	0.210091072	AC100778.2
ENSG00000280387	4.519533163	0.356539013	3.548581639	0.122540322	0.210091072	AL109806.1
ENSG00000130413	48.05824743	89.99308024	-0.905710563	0.122556549	0.210091383	STK33
ENSG00000276417	4.79139071	0.713078025	2.744098995	0.122556676	0.210091383	AC092111.2
ENSG00000085224	1483.083281	1175.67115	0.335299804	0.122580934	0.210119104	ATRX
ENSG00000122644	317.825021	184.4646089	0.783595827	0.122620642	0.210160095	ARL4A
ENSG00000189120	11.84833179	3.423622185	1.814572145	0.122621026	0.210160095	SP6
ENSG00000213937	3.323932824	0.387220594	3.105205096	0.12264494	0.210187216	CLDN9
ENSG00000063601	469.5955908	590.7472019	-0.330735748	0.122700649	0.210268819	MTMR1
ENSG00000130177	1353.754611	1642.296275	-0.278813432	0.12276829	0.210370857	CDC16
ENSG00000233178	9.595389179	2.272572888	2.103837374	0.122787636	0.210390131	AL161457.2
ENSG00000231519	4.540987734	0.713078025	2.664627252	0.12298411	0.210712884	AC007285.2
ENSG00000272656	4.503164458	0.743759607	2.627093711	0.123059793	0.21082865	AC024933.1
ENSG00000222012	7.99210925	1.854670712	2.122870962	0.123170424	0.211004272	AC005481.1
ENSG00000278214	3.019337866	0.336469917	2.96681521	0.123211301	0.211060382	LINC02139
ENSG00000154359	53.67613762	28.19718237	0.924671066	0.123245104	0.211104367	LONRF1
ENSG00000271976	93.01585546	58.44250034	0.666708464	0.12328118	0.211152241	AC012467.2
ENSG00000240344	483.3862871	391.9392748	0.302663856	0.123336208	0.211232565	PPIL3
ENSG00000286572	3.330201067	0.336469917	3.107550972	0.123350218	0.211242634	AL592146.2
ENSG00000171790	8.282558066	26.39801423	-1.670577489	0.123365502	0.211254884	SLFNL1
ENSG00000136051	1642.931652	1314.874252	0.321527246	0.123400084	0.211300177	WASHC4
ENSG00000170214	13.37750355	35.27610325	-1.4024546	0.123423254	0.211325923	ADRA1B
ENSG00000285373	4.52336538	0.356539013	3.549727903	0.123472049	0.211395539	LINC02478
ENSG00000276216	3.917936411	0	4.304127056	0.123565094	0.211540901	AC245014.3
ENSG00000120458	402.7862704	305.2376338	0.400267488	0.123579366	0.211551395	MSANTD2
ENSG00000214967	17.7068805	8.060941101	1.146368453	0.123597606	0.211568678	NPIPA7
ENSG00000139193	23.84154589	10.26166673	1.210211347	0.123663001	0.211666674	CD27

novel.449	4.003612153	0	4.333885956	0.123680361	0.211682441	-
ENSG00000177459	3.944405577	0.693008929	2.476948938	0.123718732	0.211734166	ERICH5
ENSG00000271862	24.88348837	11.1364534	1.159524972	0.123761636	0.211793641	AC104118.1
ENSG00000137960	28.38636585	13.68772906	1.043875105	0.123770188	0.211794326	GIPC2
ENSG00000272758	23.15531359	11.23209417	1.03633621	0.123794014	0.211814441	AC083798.2
ENSG00000168077	1229.93273	2825.707205	-1.200067124	0.123798248	0.211814441	SCARA3
ENSG00000135272	1094.201603	724.5577256	0.594301689	0.123874843	0.211931535	MDFIC
ENSG00000080503	1549.059045	1052.893411	0.556837392	0.12396875	0.212073496	SMARCA2
ENSG00000251682	10.94944851	2.169915658	2.33929416	0.123974145	0.212073496	AC122718.2
ENSG00000144713	10149.27912	12989.64187	-0.355987715	0.123983415	0.21207539	RPL32
ENSG00000149634	28.38608111	13.00160821	1.136628771	0.124015572	0.212116431	SPATA25
ENSG00000105379	632.6612308	940.2866103	-0.571573126	0.124025757	0.212119886	ETFB
ENSG00000237512	10.29519816	3.108505634	1.708471273	0.124079924	0.212198558	UNC5B-AS1
ENSG00000151304	182.2810803	240.6498407	-0.401496977	0.124119612	0.21225246	SRFBP1
ENSG00000265539	0.293240848	2.92428775	-3.163224908	0.124145203	0.212282252	MIR3164
ENSG00000104722	43.52639159	100.3112298	-1.204895731	0.124201764	0.212364991	NEFM
ENSG00000266256	24.50367414	67.45280665	-1.460262408	0.124294967	0.212510367	LINC00683
ENSG00000100099	801.5447811	625.7524368	0.357576034	0.124323407	0.212545005	HPS4
ENSG00000162298	963.522907	1237.533305	-0.360819872	0.12436064	0.212589903	SYVN1
ENSG00000277020	9.918860139	2.731897526	1.837283453	0.124366034	0.212589903	AL590096.1
ENSG00000150977	398.7809888	590.2140226	-0.56563552	0.124406909	0.212639318	RILPL2
ENSG00000170291	607.7852806	793.2333479	-0.384608803	0.124411311	0.212639318	ELP5
ENSG00000008438	2.726097019	8.090466807	-1.562461102	0.124457538	0.212702545	PGLYRP1
ENSG00000265975	12.22216251	3.036401591	2.028818487	0.124464678	0.212702545	AC002091.1
ENSG00000279413	14.88034868	28.20766646	-0.924869101	0.124486868	0.212726475	AC112497.1
ENSG00000101166	742.2030705	958.2610046	-0.368950115	0.124516745	0.212744219	PRELID3B
ENSG00000198883	3.895299463	0	4.296181848	0.124521817	0.212744219	PNMA5
ENSG00000229484	3.895299463	0	4.296181848	0.124521817	0.212744219	AC243836.1
ENSG00000163832	503.6517012	689.0852515	-0.452615992	0.124532528	0.212748529	ELP6
ENSG00000236540	110.5171087	70.43701054	0.648947642	0.124566612	0.212792764	AC006547.1
ENSG00000182000	2.683259148	0	3.759848089	0.124575618	0.212794157	PPIAP73
ENSG00000262655	96.71841944	39.93349126	1.273449704	0.124608495	0.212822912	SPON1
ENSG00000262313	0.293240848	2.91483114	-3.158958674	0.124608835	0.212822912	AC127496.3
ENSG00000254762	3.914175465	0.693008929	2.467874766	0.124626142	0.21283848	AP001107.7
ENSG00000230710	2.682005499	0	3.759242725	0.124648972	1	LINC00332
ENSG00000223652	3.912921816	0.693008929	2.467501382	0.124663724	0.212888669	AC106786.1
ENSG00000171161	691.4585149	526.0555961	0.394994805	0.124694609	0.212927417	ZNF672
ENSG00000103707	203.6305635	273.1653386	-0.424708129	0.124729702	0.212973343	MTFMT
ENSG00000234062	30.85926786	17.90701741	0.784616564	0.124780093	0.213045384	AL390879.1
ENSG00000273306	21.26234029	9.171852206	1.222355838	0.124801888	0.213048996	AC018690.1
ENSG00000266903	14.70684591	4.250098321	1.785274517	0.124803962	0.213048996	AC243964.2
ENSG00000007062	7.685078266	0.774441189	3.348106072	0.12480681	0.213048996	PROM1
ENSG00000260328	0.922560414	4.984401318	-2.445176228	0.124829721	0.213074108	AC104024.2
ENSG00000259648	4.48930305	0.336469917	3.539560883	0.124895396	0.213172204	AL132640.2
ENSG00000203734	14.38420132	4.167381792	1.806771487	0.124991517	0.21332225	ECT2L
ENSG00000103034	298.5740675	525.7646801	-0.815882304	0.125170832	0.21360695	NDRG4
novel.66	9.328546226	2.92428775	1.684228486	0.125174774	0.21360695	-
ENSG00000228839	7.473396209	1.844058227	2.036002018	0.12518458	0.213609653	PIK3IP1-AS1
ENSG00000182534	5380.946855	4049.18563	0.410234628	0.125225827	0.213666002	MXRA7
ENSG00000218426	461.1889914	643.6413588	-0.48084132	0.125257565	0.213702195	AL590867.2
ENSG00000264538	260.3118932	188.0542464	0.470102128	0.125263559	0.213702195	SUZ12P1

ENSG00000177045	128.4478792	200.7980388	-0.644140491	0.125271715	0.213702195	SIX5
ENSG00000224870	431.2016405	321.4566038	0.424688353	0.12532215	0.213774196	MRPL20-AS1
ENSG00000145982	211.3735234	278.828412	-0.40037501	0.125366091	0.213835111	FARS2
ENSG00000149483	388.9566668	542.399805	-0.480194153	0.125385989	0.213855011	TMEM138
ENSG00000106077	225.410691	314.78704	-0.482368053	0.125402556	0.213869227	ABHD11
ENSG00000280069	6.007191971	16.31311655	-1.433493121	0.125420642	0.213886033	AC127024.8
ENSG00000184702	12.76713126	23.25497421	-0.862937165	0.125441567	0.213907677	SEPT5
ENSG00000209042	1.802282956	6.318512624	-1.811632626	0.125496037	0.213986516	SNORD12C
ENSG00000286116	19.71074505	8.794216616	1.155772961	0.125545415	0.214056662	AL157394.2
ENSG00000260454	0.600343103	3.566546002	-2.565738714	0.12562218	0.214173493	AL355607.2
ENSG00000259895	0	2.506385574	-3.907393187	0.125656084	1	AC106820.2
ENSG00000222389	0	2.506385574	-3.907393187	0.125656084	1	RNU2-28P
ENSG00000264044	0	2.506385574	-3.907393187	0.125656084	1	AC005726.2
ENSG00000213642	0.307102255	4.61596555	-3.819515581	0.125656363	0.214217715	AC016769.1
ENSG00000197057	7.400541689	0	5.220888966	0.125691905	0.214264246	DTHD1
ENSG00000066117	713.28916	938.0500438	-0.395142254	0.125730607	0.214316159	SMARCD1
novel.847	29.75246347	8.445849944	1.821212103	0.12575285	0.214340011	-
novel.640	32.11944537	62.25342433	-0.957017227	0.125821209	0.214442458	-
ENSG00000267122	0.600343103	3.557089392	-2.562390393	0.125983332	0.214704687	AC004490.1
ENSG00000283297	11.74378004	4.983245442	1.233265152	0.126091682	0.214875246	TEX52
ENSG00000254999	3085.558946	4184.028114	-0.43929312	0.126100017	0.214875354	BRK1
ENSG00000118418	1238.031479	914.3767136	0.436854542	0.126113612	0.214884428	HMGN3
ENSG00000261744	0.601596751	3.557089392	-2.561139935	0.126162681	0.214953939	AC116552.1
ENSG00000122786	5779.979851	8917.375745	-0.625589356	0.126231424	0.215056451	CALD1
ENSG00000123349	4118.788919	3266.978818	0.33429034	0.126239403	0.215056451	PFDN5
ENSG00000233061	3.881438055	0	4.2912844	0.126298885	0.215143675	TTLL7-IT1
ENSG00000107796	883.8739801	467.2906154	0.919068741	0.126374256	0.215257953	ACTA2
ENSG00000152620	644.5932737	489.889483	0.395942344	0.126466115	0.215400297	NADK2
ENSG00000288055	2.432856171	0	3.61565042	0.126496855	1	AC107373.3
ENSG00000233828	3.345316124	0.387220594	3.11326134	0.126512004	0.215464332	LINC01949
ENSG00000026950	654.8595225	450.4503175	0.539997893	0.126523448	0.215469697	BTN3A1
ENSG00000120784	279.5553244	218.4876351	0.355312362	0.126696848	0.215750857	ZFP30
ENSG00000130684	126.2391432	77.11047394	0.713330316	0.126708161	0.215755979	ZNF337
ENSG00000229688	3.04831433	0.336469917	2.978424224	0.126740692	0.21579723	CRPPA-AS1
ENSG00000223849	6.268949058	1.080229524	2.54072695	0.126800556	0.215872228	AL354893.1
ENSG00000099797	1000.010078	1314.160674	-0.393999761	0.126801358	0.215872228	TECR
ENSG00000199805	0	3.505182839	-4.392864999	0.126845184	0.215932691	RNU1-134P
ENSG00000225470	86.94666488	60.71592513	0.520637231	0.126867297	0.215956185	JPX
ENSG00000188779	33.91195604	13.9295857	1.289391314	0.126882313	0.215967595	SKOR1
ENSG00000286735	0.586481695	3.953766596	-2.728888731	0.127062304	0.216259791	AC126182.3
ENSG00000256720	3.345316124	0.356539013	3.113264788	0.127080254	0.216262008	BTG1P1
ENSG00000114786	3.345316124	0.356539013	3.113264788	0.127080254	0.216262008	ABHD14A-ACY1
ENSG00000279133	21.74831592	12.08681174	0.843083045	0.127093701	0.216270725	AC018628.1
ENSG00000249115	325.5147303	471.452334	-0.534397411	0.127122893	0.216292706	HAUS5
ENSG00000177182	3.944405577	0.723690511	2.453441691	0.127123268	0.216292706	CLVS1
ENSG00000189184	458.4094252	1260.540285	-1.459469728	0.127215732	0.216435854	PCDH18
ENSG00000228290	12.8880517	3.892403433	1.719641327	0.127263702	0.21650329	TBX18-AS1
ENSG00000138400	27.0205964	13.95325081	0.952021659	0.12733763	0.216614874	MDH1B
ENSG00000205863	2.418994764	0	3.608347268	0.127371083	1	C1QTNF9B
ENSG00000286110	2.418994764	0	3.608347268	0.127371083	1	AC090983.2
ENSG00000237883	35.14566439	19.02738513	0.886720104	0.127375506	0.216665119	DGUOK-AS1

ENSG00000236206	2.417741115	0	3.607689138	0.127453802	1	AL356441.1
ENSG00000186842	2.417741115	0	3.607689138	0.127453802	1	LINC00846
ENSG00000183785	3.020591515	0.356539013	2.967294393	0.127493867	0.216842089	TUBA8
ENSG00000104419	11223.05529	6526.000246	0.782230564	0.127496238	0.216842089	NDRG1
ENSG00000253227	0.293240848	2.985650913	-3.188630933	0.127521087	0.216870156	AC090192.2
ENSG00000285492	3.018084217	0.356539013	2.966293877	0.127601685	0.216993021	AL356417.3
ENSG00000141698	742.0703975	971.8851749	-0.389135863	0.127641637	0.217046756	NT5C3B
ENSG00000267655	18.24300243	5.564140531	1.704724231	0.127677807	0.217094051	AC125437.1
ENSG00000233064	4.133094681	0	4.382508018	0.127713191	0.217140005	AL136307.1
ENSG00000246067	212.8027032	143.4511793	0.569061753	0.127772118	0.217225978	RAB30-DT
ENSG00000283491	3.28360225	8.998375202	-1.445874923	0.127818558	0.217290712	AC017104.5
ENSG00000211669	4.004865802	0	4.334346221	0.127874028	0.217370787	IGLV3-10
ENSG00000132932	28.54378465	62.06052053	-1.121313482	0.127901426	0.217403137	ATP8A2
ENSG00000178750	6.939781575	1.447381022	2.267129948	0.128080479	0.217691957	STX19
ENSG00000095787	2640.444864	2140.985214	0.30254261	0.128088101	0.217691957	WAC
ENSG00000283538	9.144942989	20.19503589	-1.14442839	0.128096965	0.217692782	AC005972.3
ENSG00000084463	2227.155328	1635.809808	0.445214062	0.128129273	0.217724309	WBP11
ENSG00000135749	539.0122551	372.0241659	0.535663024	0.128132277	0.217724309	PCNX2
ENSG00000261131	7.531349136	2.527610546	1.580893312	0.128235872	0.217881801	AC012186.2
ENSG00000213222	0.615458159	3.557089392	-2.546845716	0.128241735	0.217881801	AC093724.1
ENSG00000230397	5.701343365	12.89077864	-1.173467505	0.128273344	0.217921255	SPTLC1P1
ENSG00000258983	4.777529303	0.713078025	2.740425204	0.128293248	0.217934665	AL162171.3
ENSG00000185513	133.7197765	79.81181828	0.745771895	0.12830562	0.217934665	L3MBTL1
ENSG00000134152	799.8159937	619.0308724	0.36981203	0.128306402	0.217934665	KATNBL1
novel.412	10.90313443	24.63770001	-1.18134919	0.128321724	0.217940442	-
ENSG00000259881	3.047060681	0.387220594	2.977942477	0.128334969	0.217940442	AC092384.2
ENSG00000174469	3.047060681	0.387220594	2.977942477	0.128334969	0.217940442	CNTNAP2
ENSG00000175115	1270.814519	1540.848063	-0.278033111	0.128503785	0.218212865	PACS1
ENSG00000174136	387.592595	578.0409792	-0.576282161	0.128548274	0.218274146	RGMB
ENSG00000137699	6.773800692	0.672939833	3.294353728	0.128621296	0.218383864	TRIM29
ENSG00000284431	0.308355904	2.822786395	-3.120907442	0.128644763	0.218409435	AL022238.4
ENSG00000105289	9.826844882	2.486316478	1.979751335	0.128680019	0.218455017	TJP3
ENSG00000285667	12.10758158	2.691759334	2.146027431	0.128696371	0.218468502	AC012291.2
ENSG00000286771	4.499403511	0.713078025	2.653145333	0.128728819	0.218509306	AC097658.3
ENSG00000165671	1321.602127	1634.667499	-0.30675779	0.128781222	0.218583976	NSD1
ENSG00000254694	6.552089444	1.406086955	2.209712084	0.128847374	0.218681971	AP001893.1
ENSG00000005075	565.0358116	700.6774676	-0.31079668	0.128866889	0.218700807	POLR2J
ENSG00000112977	2471.742242	3010.291947	-0.284325242	0.128875729	0.218701522	DAP
ENSG00000081019	418.633797	301.7445917	0.473241478	0.128944484	0.218803909	RSBN1
ENSG00000126756	464.8190579	617.1881928	-0.409274152	0.128965855	0.21882588	UXT
ENSG00000230536	0.293240848	2.822786395	-3.120896309	0.129005004	0.218863721	AL360268.1
ENSG00000243339	0.293240848	2.822786395	-3.120896309	0.129005004	0.218863721	RN7SL738P
ENSG00000236797	4.412474121	0	4.476889545	0.129042968	0.218913833	SPA17P1
ENSG00000112874	406.1506885	280.5639052	0.533227158	0.129134949	0.219046743	NUDT12
ENSG00000136925	253.5764574	177.5619775	0.513840462	0.129138176	0.219046743	TSTD2
ENSG00000154620	13.82161022	2.395427609	2.514504082	0.129170016	0.219086446	TMSB4Y
ENSG00000243094	3.872591243	0	4.288131591	0.129226134	0.21916732	AC079203.1
ENSG00000217930	2.137108026	7.265274941	-1.766914117	0.129241552	0.219179163	PAM16
ENSG00000180739	16.10360057	6.378719912	1.329971742	0.129307935	0.2192733	S1PR5
ENSG00000277692	4.235139127	0.672939833	2.602008167	0.129313941	0.2192733	AL121583.1
ENSG00000260081	34.99640822	17.80680032	0.966326853	0.129408758	0.219404929	AF274858.1

novel.373	6.564697203	1.396630344	2.219126885	0.129409569	0.219404929	-
ENSG00000133318	2379.812634	1852.651147	0.361073509	0.129416902	0.219404929	RTN3
ENSG00000274810	3.047060681	0.336469917	2.977954216	0.129430366	0.219413438	NPHP3-ACAD11
ENSG00000197993	4.875812803	0.774441189	2.707309761	0.129487347	0.219495711	KEL
ENSG00000110700	6285.866628	8066.94264	-0.359923906	0.129527863	0.219550065	RPS13
ENSG00000125970	2740.655024	3352.657263	-0.29084629	0.129649667	0.219742188	RALY
ENSG00000167889	14.05070117	32.62751737	-1.210734893	0.129682502	0.219783502	MGAT5B
ENSG00000215417	36.54800346	56.43185318	-0.628587534	0.129750942	0.219885149	MIR17HG
ENSG00000234456	1471.054853	1025.586041	0.520169943	0.129819125	0.219986348	MAGI2-AS3
ENSG00000250365	20.72245742	9.835463823	1.067975187	0.129887432	0.220087744	AL139353.2
ENSG00000047056	455.0916514	561.4142059	-0.302909713	0.129978844	0.220228273	WDR37
ENSG00000286292	8.033408738	2.149846562	1.903335206	0.130053657	0.220340662	AC099343.4
ENSG00000183291	2156.078359	2849.244363	-0.402246001	0.130162129	0.220510059	SELENOF
ENSG00000197296	208.1057204	265.1545874	-0.349389289	0.130197866	0.220556221	FITM2
ENSG00000225920	12.27614103	4.340987191	1.505096689	0.130277457	0.220676661	RIMKLBP2
ENSG00000181826	393.477154	274.5876762	0.519646281	0.130322775	0.220739033	RELL1
ENSG00000231768	7.351435574	1.793307549	2.032676433	0.130458575	0.220954643	LINC01354
ENSG00000247151	2.127007565	7.000780673	-1.713293778	0.130512228	0.221031105	CSTF3-DT
ENSG00000140543	57.67047603	34.70068357	0.73583499	0.130535752	0.221056536	DET1
ENSG00000286443	5.7731577	1.406086955	2.02169038	0.130550954	0.22106787	AC091178.2
ENSG00000235554	7.624546771	2.058957692	1.861045334	0.130593822	0.221126049	AC005822.1
novel.64	8.487900608	16.23412444	-0.934224493	0.130604301	0.221129381	-
ENSG00000234377	4.880827398	1.049547942	2.195473458	0.130639248	0.221174137	RNF219-AS1
ENSG00000167978	8034.055126	10917.0969	-0.44236649	0.130668581	0.221209383	SRRM2
ENSG00000266149	5.163967785	1.029478846	2.293569101	0.130713591	0.221264444	AP001094.3
ENSG00000167283	676.0447926	491.7308574	0.459218518	0.130724519	0.221264444	ATP5MG
ENSG00000139323	383.9139053	294.7111688	0.382128057	0.130726655	0.221264444	POC1B
ENSG00000279019	21.99718052	8.244003109	1.417278212	0.1307794	0.221339299	AC009090.4
ENSG00000233110	14.26105831	6.288986917	1.166996131	0.13079864	0.221357443	AC093797.1
ENSG00000234233	0	3.424906455	-4.361432787	0.130818609	0.221376817	KCNH1-IT1
ENSG00000209482	6.271456355	15.21525808	-1.271377799	0.130868065	0.221446086	SNORD83A
ENSG00000068137	519.5658294	711.1976056	-0.453245731	0.130877761	0.221448069	PLEKHH3
ENSG00000276434	3.00422281	0.356539013	2.960675522	0.13088947	0.221453459	AL136221.1
novel.914	17.82808568	4.401194479	2.022483911	0.130900166	0.221457134	-
ENSG00000118200	1351.462122	1039.539254	0.378697073	0.130924471	0.221483832	CAMSAP2
novel.1105	1.466204238	8.193124037	-2.464997228	0.13093392	0.221485395	-
ENSG00000273124	2.431602522	0	3.615012665	0.130936457	1	AL365434.2
ENSG00000260704	2.431602522	0	3.615012665	0.130936457	1	LINC00543
ENSG00000205693	2.431602522	0	3.615012665	0.130936457	1	MANSC4
ENSG00000265566	2.431602522	0	3.615012665	0.130936457	1	RN7SL605P
ENSG00000224216	2.431602522	0	3.615012665	0.130936457	1	AC234781.1
ENSG00000239650	2.431602522	0	3.615012665	0.130936457	1	GUSBP4
ENSG00000135747	0	2.42610919	-3.865460494	0.130951844	1	ZNF670-ZNF695
ENSG00000267523	21.62488818	39.23534525	-0.856915712	0.130962268	0.221518925	AC008735.2
ENSG00000173039	1448.305067	1891.370506	-0.384877852	0.131196266	0.221900279	RELA
ENSG00000165782	739.848925	559.8334864	0.402662555	0.13122539	0.22193509	PIP4P1
ENSG00000118412	464.4188022	368.7332059	0.332751592	0.131252168	0.22196593	CASP8AP2
ENSG00000261183	6.751163743	1.00940975	2.70483142	0.131347278	0.22208977	SPINT1-AS1
ENSG00000232186	29.15992699	9.465743786	1.633490194	0.131348387	0.22208977	AL137013.1
ENSG00000122958	1113.507291	1400.852836	-0.331398203	0.131351041	0.22208977	VPS26A
ENSG00000233476	1105.596483	1466.972958	-0.408071492	0.131449323	0.222241483	EEF1A1P6

ENSG00000287249	4.621577609	0.356539013	3.578777961	0.131479322	0.222277738	AC005400.1
ENSG00000100600	731.6252565	1013.162024	-0.46984352	0.131527635	0.222344947	LGMN
ENSG00000206145	0.307102255	2.893606169	-3.15069796	0.131653641	0.222543478	P2RX6P
ENSG00000265437	0.923814062	4.351599676	-2.244943186	0.131669252	0.222555387	AP005212.1
ENSG00000232807	48.09550124	32.34667842	0.573203206	0.131731148	0.222631752	AL137186.2
ENSG00000133773	457.7316458	577.3785835	-0.33529043	0.13173157	0.222631752	CCDC59
ENSG00000075035	4.621577609	0.336469917	3.578781093	0.131786331	0.222709813	WSCD2
ENSG00000128254	8.791241917	2.772035718	1.644779694	0.131850958	0.222804536	C22orf24
ENSG00000258947	15.96004317	31.18989693	-0.969330091	0.13189043	0.222856742	TUBB3
ENSG00000276805	71.34978251	39.87187131	0.837497718	0.132019866	0.223060944	AL133216.2
ENSG00000227008	24.28948479	41.21860288	-0.760353285	0.132057474	0.223109977	AL009174.1
ENSG00000118276	135.2445306	200.0311277	-0.56461783	0.132094313	0.223157706	B4GALT6
ENSG00000143183	1133.752389	1523.829762	-0.426782557	0.132157932	0.223250665	TMCO1
ENSG00000112514	191.2869976	454.4272289	-1.248156296	0.132324123	0.223516874	CUTA
ENSG00000010361	361.3117662	284.6210997	0.344365856	0.132336449	0.223523162	FUZ
ENSG00000272699	4.515772216	0.774441189	2.601751962	0.132373842	0.223571786	AC007620.3
ENSG00000265735	2.743719372	9.527363738	-1.802087009	0.132446591	0.223680114	RN7SL5P
novel.734	48.42552553	97.07709134	-1.002076991	0.132476612	0.223716272	-
ENSG00000196154	977.0074513	577.2802208	0.759433689	0.132501241	0.223743322	S100A4
ENSG00000169371	237.4684113	295.7047475	-0.316700329	0.132540578	0.223781307	SNUPN
ENSG00000259994	46.27405755	26.56789521	0.797907568	0.132540963	0.223781307	AL353796.1
ENSG00000138286	539.4971481	419.6164929	0.363036094	0.132726762	0.224080448	FAM149B1
ENSG00000273248	4.554849141	1.080229524	2.077901873	0.132756209	0.224115599	AC010997.5
ENSG00000099812	0.308355904	3.861721852	-3.571649337	0.132808309	0.224188985	MISP
novel.217	2.403879708	0	3.600317746	0.132820852	1	-
ENSG00000224430	2.403879708	0	3.600317746	0.132820852	1	MKRN5P
ENSG00000258569	2.403879708	0	3.600317746	0.132820852	1	AC007376.2
ENSG00000259262	2.403879708	0	3.600317746	0.132820852	1	NDUFA3P4
ENSG00000236202	2.403879708	0	3.600317746	0.132820852	1	GRM7-AS1
novel.975	27.67137054	55.7427786	-1.007892522	0.13283329	0.224216588	-
ENSG00000163960	871.5946959	1204.94688	-0.466959334	0.132865333	0.224256104	UBXN7
ENSG00000266420	0	3.751919761	-4.492549804	0.13302752	0.224515266	RN7SL118P
ENSG00000117983	9.083229116	3.302051734	1.47317478	0.133149112	0.224702663	MUC5B
ENSG00000261377	8.802596027	18.55988815	-1.078351891	0.133155853	0.224702663	PDCD6IPP2
ENSG00000226029	46.23630554	25.88293023	0.842057484	0.133286457	0.224908452	LINC01772
ENSG00000132429	418.6145219	262.6773296	0.673221303	0.133305361	0.224925743	POPDC3
ENSG00000186073	160.7986815	226.686153	-0.495979648	0.133336006	0.224962841	C15orf41
ENSG00000077616	9.88354416	3.577158488	1.466888533	0.133367291	0.224998327	NAALAD2
ENSG00000183166	4.308207111	0.387220594	3.477394624	0.13337436	0.224998327	CALN1
ENSG00000245017	5.789526405	1.396630344	2.031810615	0.133519836	0.225206512	LINC02453
ENSG00000034713	1769.694459	1306.58857	0.437782336	0.133521455	0.225206512	GABARAPL2
ENSG00000232671	4.262861942	10.10928631	-1.246846597	0.133523772	0.225206512	ZNF687-AS1
ENSG00000269887	1.203193503	5.54522731	-2.20826867	0.133552521	0.225232188	AL391001.1
ENSG00000247595	35.48690025	17.96966484	0.977720735	0.133556333	0.225232188	SPTY2D1OS
ENSG00000156427	6.026067974	1.753169357	1.766004896	0.133586803	0.225268951	FGF18
ENSG00000280693	5.759296292	1.456837632	1.992192677	0.133670846	0.225380225	SH3PXD2A-AS1
ENSG00000226432	6.079006306	1.753169357	1.776388436	0.133677321	0.225380225	AC010342.1
ENSG00000262228	2.095523804	6.512058724	-1.625919429	0.133678814	0.225380225	AC087392.3
ENSG00000178796	5.785765458	1.069617038	2.42926277	0.133788862	0.225551127	RIIAD1
ENSG00000166889	709.4422119	933.9255528	-0.396271889	0.133807378	0.225561035	PATL1
ENSG00000152229	206.1235948	271.3121149	-0.395978564	0.133812102	0.225561035	PSTPIP2

ENSG00000227527	8.793749215	3.311508345	1.423868551	0.133835756	0.22558627	AC096540.1
ENSG00000231205	99.82447358	47.53536451	1.067353414	0.133862078	0.225616001	ZNF826P
ENSG00000144645	151.2044313	280.5022977	-0.89236396	0.133879567	0.22562101	OSBPL10
ENSG00000197582	183.9048455	138.7948187	0.405570371	0.133882418	0.22562101	GPX1P1
ENSG00000125877	888.4573398	1108.007931	-0.318729404	0.133899712	0.225635518	ITPA
ENSG00000261101	11.84582449	5.370466037	1.138684013	0.133953778	0.225711986	AC234775.3
ENSG00000270589	13.20622334	5.379922647	1.296647715	0.133969656	0.225724101	AL158163.1
ENSG00000279382	49.80555548	25.29411959	0.973992067	0.134015153	0.225786116	AC018665.1
ENSG00000179922	106.0405556	150.5509399	-0.505428908	0.134041984	0.225810587	ZNF784
ENSG00000168813	605.1575539	470.4994412	0.362969765	0.134047972	0.225810587	ZNF507
ENSG00000166128	1307.151325	970.555614	0.429789157	0.134055752	0.225810587	RAB8B
ENSG00000203993	430.4394766	534.2361127	-0.311788774	0.134084673	0.22584466	ARRDC1-AS1
ENSG00000175073	550.8750738	428.5852952	0.362543755	0.134122512	0.22589375	VCPIP1
ENSG00000100191	7.180155362	1.834601616	1.983825082	0.134193161	0.225998089	SLC5A4
ENSG00000210082	91692.773	60787.68161	0.593030306	0.134233386	0.22605118	MT-RNR2
ENSG00000280163	7.752775649	1.426156051	2.439917265	0.134260211	0.2260817	AC040160.2
ENSG00000253633	0.908699006	4.463713517	-2.286865363	0.134270747	0.226084788	AP002852.1
ENSG00000286696	5.906401173	21.02022776	-1.825903496	0.134337328	0.226182238	AC090826.3
ENSG00000224680	2.697120555	8.653732944	-1.685253882	0.134356234	0.226199411	PLA2G12AP1
ENSG00000106258	88.78537492	62.76915063	0.498899886	0.134376255	0.22621254	CYP3A5
ENSG00000168569	272.6676548	361.1516717	-0.406133457	0.134381446	0.22621254	TMEM223
ENSG00000278112	3.591958154	9.264025345	-1.362990361	0.134400609	0.226230142	AC145423.3
ENSG00000272455	42.60089661	74.43181442	-0.805491685	0.134429633	0.226264337	AL391244.2
ENSG00000158796	514.0430482	405.1125618	0.343619254	0.134514294	0.226392166	DEDD
ENSG00000228714	2.442956632	9.243956249	-1.924780263	0.134542407	0.226424814	AL691420.1
ENSG00000074054	937.9799127	1215.09153	-0.373268671	0.134551535	0.226425509	CLASP1
ENSG00000181619	54.4314635	79.84622428	-0.554311231	0.134613669	0.226515397	GPR135
ENSG00000232415	13.04274975	5.595977988	1.206949385	0.134657922	0.226575188	ELN-AS1
ENSG00000286349	4.722083673	0.336469917	3.613494082	0.134703742	0.226637606	AC108471.3
ENSG00000169777	4.301867596	0	4.437861202	0.134772409	0.226723773	TAS2R1
ENSG00000196092	4.301867596	0	4.437861202	0.134772409	0.226723773	PAX5
ENSG00000166922	5.892539766	24.02800273	-2.021041847	0.134806992	0.226756698	SCG5
ENSG00000105085	105.9048761	140.6870591	-0.408492115	0.134809436	0.226756698	MED26
ENSG00000248121	15.26546225	28.95373782	-0.926750492	0.134846422	0.226804227	SMURF2P1
ENSG00000107929	1223.738525	1520.327717	-0.313054496	0.13486165	0.226815157	LARP4B
ENSG00000269352	71.93028069	44.11020127	0.702546569	0.135114317	0.227225392	PTOV1-AS2
ENSG00000271133	17.32831991	6.167416466	1.474541303	0.135146203	0.227264305	AC004130.1
ENSG00000113356	181.7411696	258.0107545	-0.504908399	0.135208252	0.227353931	POLR3G
ENSG00000232732	1.230916317	5.868644597	-2.258185175	0.135222064	0.227362441	AC097717.1
ENSG00000132879	589.8484987	398.9698726	0.564843987	0.135243278	0.227383395	FBXO44
ENSG00000197766	3.374292587	0.336469917	3.124332485	0.135269164	0.227412201	CFD
ENSG00000236213	5.541630725	0.743759607	2.91736529	0.13532996	0.227499689	AC006369.1
ENSG00000155657	276.4238142	179.9352342	0.620513717	0.135396195	0.227590711	TTN
ENSG00000121022	903.2984972	1108.390609	-0.295387472	0.135402369	0.227590711	COPS5
ENSG00000142621	15.89094959	5.12488499	1.637768552	0.135410385	0.227590711	FHAD1
ENSG00000110243	0	3.362259022	-4.327074701	0.135434509	0.227616533	APOA5
ENSG00000125354	453.1028422	279.5038732	0.696611114	0.135504789	0.227719917	SEPT6
ENSG00000175536	26.59633464	15.04293695	0.82181118	0.135518758	0.227728662	LIPT2
ENSG00000177548	437.2326514	540.3324054	-0.305548151	0.135528593	0.22773046	RABEP2
ENSG00000122692	1048.594462	1277.502884	-0.284877087	0.135602205	0.227839416	SMU1
novel.534	8.59091397	22.37787579	-1.375411	0.135702591	0.227993341	-

ENSG00000278224	4.906042916	0.672939833	2.819472867	0.135766772	0.228086422	PRICKLE4
ENSG00000132604	441.0932047	544.2871995	-0.303368481	0.135833026	0.228180559	TERF2
novel.592	11.89367696	4.758889366	1.330000405	0.135840372	0.228180559	-
ENSG00000122203	2009.537652	2615.192526	-0.379910588	0.135858936	0.228196988	KIAA1191
ENSG00000269226	5.703850663	13.21663607	-1.210054592	0.135871739	0.228203739	TMSB15B
ENSG00000084731	1467.572455	955.4499359	0.61940166	0.135882199	0.228206555	KIF3C
ENSG00000117713	1305.783693	1679.308475	-0.362797863	0.135922455	0.228259408	ARID1A
ENSG00000251562	8168.582315	5254.383567	0.636577399	0.135980612	0.228342313	MALAT1
ENSG00000243989	6.972590256	14.44081689	-1.049005616	0.136035009	0.228418896	ACY1
ENSG00000213593	1288.332767	1668.036436	-0.372833148	0.1360543	0.228436524	TMX2
ENSG00000127585	21.02225124	45.4744334	-1.111526207	0.136130605	0.22854987	FBXL16
ENSG00000287937	0.308355904	3.983292303	-3.61252748	0.1361506	0.22856867	AC079753.2
novel.720	1.186824798	9.649833275	-3.012170456	0.136203656	0.228642967	-
ENSG00000148187	419.4190404	533.2095876	-0.346576921	0.136236095	0.228682645	MRRF
ENSG00000286780	2.758834428	0	3.796275347	0.136258346	0.22870312	AL139379.1
ENSG00000197181	51.0646928	22.35451464	1.185154737	0.136265898	0.22870312	PIWIL2
ENSG00000152102	2461.833895	3010.234105	-0.290156823	0.136279004	0.228710341	FAM168B
ENSG00000161558	129.07294	181.101999	-0.488467463	0.136306396	0.228732601	TMEM143
ENSG00000169180	2325.423162	3097.669575	-0.413647281	0.136309875	0.228732601	XPO6
ENSG00000123607	379.4945931	478.7253358	-0.334964279	0.136354911	0.228793396	TTC21B
ENSG00000279357	4.486795753	0.672939833	2.691149236	0.13637162	0.228806656	AC007224.2
novel.890	0	3.392940604	-4.339442878	0.136422982	0.228835218	-
ENSG00000287229	0	3.392940604	-4.339442878	0.136422982	0.228835218	AC009731.1
ENSG00000125798	0	3.392940604	-4.339442878	0.136422982	0.228835218	FOXA2
ENSG00000145850	6.564697203	1.161661783	2.541817266	0.136423875	0.228835218	TIMD4
ENSG00000284747	6.065144898	15.59443472	-1.369235994	0.136452552	0.228868544	AL034417.4
ENSG00000183770	7.965711355	2.089639274	1.912688314	0.136478207	0.228896797	FOXL2
ENSG00000148143	391.3424701	261.0903636	0.584741977	0.136560605	0.22902021	ZNF462
ENSG00000160867	64.32023572	35.44315891	0.862390048	0.136611885	0.22909142	FGFR4
ENSG00000119953	539.9636342	666.0895405	-0.30274234	0.136674347	0.229181374	SMNDC1
novel.798	54.82627878	163.239216	-1.574642902	0.136788444	0.229357893	-
ENSG00000278416	5.14759908	12.28164211	-1.260680025	0.136852665	0.229450768	PMS2P2
ENSG00000136754	1057.318676	874.7094888	0.273406223	0.136900824	0.2295167	ABI1
ENSG00000109103	435.2736461	678.782137	-0.640948343	0.136969351	0.229616771	UNC119
ENSG00000254595	32.25838762	13.30636906	1.278775211	0.137270497	0.230106767	AC084337.1
ENSG00000251102	7.584216197	16.48916209	-1.121548561	0.137315976	0.230156054	CTBP2P4
ENSG00000111801	635.543422	456.5554717	0.477311544	0.137317616	0.230156054	BTN3A3
ENSG00000247679	84.90526179	60.37829934	0.494472932	0.137364972	0.230208315	AC139795.2
ENSG00000267092	0.307102255	2.782648203	-3.103366128	0.137366518	0.230208315	AC027307.1
ENSG00000226386	9.494811844	2.231278821	2.102969806	0.137386488	0.230226932	PARD3-AS1
ENSG00000124177	1238.704464	893.9884523	0.470781294	0.137547291	0.230479088	CHD6
ENSG00000245149	50.55962771	31.50013319	0.686439524	0.137567886	0.230479088	RNF139-AS1
ENSG00000056998	213.9351348	124.1195	0.78644284	0.137575068	0.230479088	GYG2
ENSG00000134287	2413.43723	3046.028281	-0.335835473	0.137575566	0.230479088	ARF3
ENSG00000238164	3.692748952	0	4.217268639	0.137599058	0.230479088	TNFRSF14-AS1
ENSG00000262681	3.692748952	0	4.217268639	0.137599058	0.230479088	AC005722.2
ENSG00000215785	3.692748952	0	4.217268639	0.137599058	0.230479088	CFL1P6
ENSG00000188818	46.24446817	24.46062699	0.912200107	0.137610599	0.230483559	ZDHHC11
ENSG00000155066	41.75201709	19.75911958	1.087525624	0.137626671	0.23049562	PROM2
ENSG00000273123	3.899060409	0.743759607	2.422118611	0.137707486	0.230616102	AC020634.1
ENSG00000112685	601.7972768	804.4277187	-0.418594884	0.137813664	0.23077904	EXOC2

ENSG00000279778	6.938599197	0.774441189	3.202623115	0.13782627	0.230785275	AL445437.1
ENSG00000264548	3.576843098	9.731522323	-1.435378418	0.137904683	0.230888969	AC132872.2
ENSG00000188559	239.9452881	318.8350219	-0.40927715	0.137905971	0.230888969	RALGAPA2
novel.715	14.38441478	3.92424089	1.871459737	0.13797793	0.230982356	-
ENSG00000166313	1194.370121	894.2860818	0.417820719	0.13797953	0.230982356	APBB1
ENSG00000081087	1040.919284	795.1261358	0.388206049	0.138045388	0.231077715	OSTM1
ENSG00000003989	957.5056389	1742.017763	-0.863518284	0.138110492	0.231171801	SLC7A2
ENSG00000267342	24.64924064	10.75282883	1.18753095	0.1381316	0.231192237	AC087289.3
ENSG00000141179	271.5426864	340.2109368	-0.324974463	0.138140661	0.231192509	PCTP
ENSG00000123136	1371.935773	1844.899476	-0.427382008	0.138198924	0.23127512	DDX39A
ENSG00000231258	3.694002601	0	4.217738546	0.13823517	0.231303963	ZSWIM5P2
ENSG00000270792	3.694002601	0	4.217738546	0.13823517	0.231303963	AL050403.2
ENSG00000280193	13.40877385	4.411806964	1.614595165	0.138242867	0.231303963	AC132219.2
ENSG00000144455	795.4056257	614.7031606	0.371643547	0.138350231	0.23146663	SUMF1
ENSG00000226952	0.293240848	3.749479616	-3.520470783	0.138357906	0.23146663	AC093019.1
ENSG00000248079	13.60777687	5.399991743	1.334508673	0.138378428	0.231486056	DPH6-DT
ENSG00000166444	910.7120176	615.5154842	0.565697805	0.138536896	0.231736229	ST5
ENSG00000166272	1559.377924	1243.248425	0.326813571	0.138562342	0.231754816	WBP1L
ENSG00000225506	1.200686205	5.554683921	-2.212116182	0.138565849	0.231754816	CYP4A22-AS1
ENSG00000180422	9.94031471	2.180528143	2.193923311	0.138581039	0.231765303	LINC00304
ENSG00000287315	6.07524536	1.00940975	2.551140256	0.138598155	0.231779008	AL670729.3
ENSG00000245275	53.27096568	29.92082602	0.827774486	0.138620609	0.231801638	SAP30L-AS1
ENSG00000261512	59.12303233	88.64822805	-0.581679113	0.138729836	0.231969357	AC092368.3
ENSG00000196209	91.33241293	29.14099096	1.651071924	0.138775138	0.232030174	SIRPB2
ENSG00000070476	719.3076508	503.8888138	0.513777133	0.138813844	0.232079954	ZXDC
ENSG00000124228	724.4129662	957.13285	-0.401996827	0.138840418	0.232109445	DDX27
ENSG00000266598	4.998955816	0.356539013	3.695688128	0.138896332	0.232187981	AC037487.2
ENSG00000168454	7.823336335	2.200597239	1.842305442	0.138956119	0.23227298	TXNDC2
ENSG00000130287	4.907296564	0.713078025	2.775339225	0.139024189	0.232361179	NCAN
ENSG00000139112	1197.454289	1670.420946	-0.480429149	0.13902677	0.232361179	GABARAPL1
ENSG00000241685	937.0846608	749.4804095	0.322216857	0.139053866	0.232391516	ARPC1A
ENSG00000050165	5136.76467	3048.056141	0.753047475	0.139071421	0.232405905	DKK3
ENSG00000267174	4.217516774	0.743759607	2.531789341	0.13911458	0.232460116	AC011472.2
ENSG00000247324	8.1845593	2.884149558	1.507368973	0.139121756	0.232460116	AC010547.1
ENSG00000277595	6.010952918	1.518200796	2.02245239	0.139141055	0.232472593	AC007546.1
ENSG00000271811	3.66251884	10.11057058	-1.47561352	0.139153984	0.232472593	Z97200.1
ENSG00000145908	321.7735594	209.6915391	0.617483813	0.139156066	0.232472593	ZNF300
ENSG00000134769	574.5447276	329.2196593	0.804017811	0.13919362	0.232520378	DTNA
ENSG00000268573	9.321024334	17.86572334	-0.936894864	0.139301554	0.232685719	AC011815.1
ENSG00000130775	498.843186	284.4869305	0.810852061	0.13932769	0.232714415	THEMIS2
ENSG00000224097	11.29333385	3.740023006	1.611377145	0.139460057	0.232910404	AC021148.1
ENSG00000125703	383.1378643	279.8393971	0.453547937	0.13946296	0.232910404	ATG4C
ENSG00000149527	8.933545666	1.365948763	2.687189619	0.139482638	0.232928296	PLCH2
ENSG00000265683	7.473396209	2.149846562	1.798557141	0.13955006	0.233025909	SYPL1P2
ENSG00000135828	322.3376299	206.0183543	0.644630805	0.139583316	0.233066461	RNASEL
ENSG00000273489	3.268487194	0.356539013	3.083836345	0.139670264	0.233196655	AC008264.2
ENSG00000170185	489.932935	396.8120287	0.304055773	0.139851113	0.233483598	USP38
ENSG00000073417	1446.493599	1018.291999	0.506365385	0.139888172	0.233530463	PDE8A
ENSG00000181350	54.85851729	27.05764464	1.019715115	0.139922552	0.233572849	LRRC75A
ENSG00000197223	286.8403444	383.4257687	-0.418682282	0.139933435	0.233576009	C1D
ENSG00000173614	198.3812756	282.6318528	-0.511764881	0.139945704	0.233581481	NMNAT1

ENSG00000171202	337.8940826	458.2825955	-0.440396565	0.139968888	0.233605169	TMEM126A
ENSG00000267692	3.269740843	0.336469917	3.084309516	0.140005734	0.233651656	TAF9P3
ENSG00000147573	2.158491326	16.84197669	-2.96612067	0.140026537	0.233671364	TRIM55
ENSG00000196152	141.5042056	100.8061164	0.489489313	0.140147364	0.233857974	ZNF79
ENSG00000116266	972.3907773	799.7342677	0.281903808	0.140157831	0.233860421	STXBP3
ENSG00000196204	288.3608988	395.0944683	-0.454279815	0.140177963	0.233878992	RNF216P1
novel.81	4.622831258	0.336469917	3.579231429	0.140193784	0.233890367	-
ENSG00000228065	2.76893489	11.67134971	-2.084959556	0.140263505	0.233991661	LINC01515
ENSG00000060566	2.447971227	0	3.623732946	0.140272942	1	CREB3L3
ENSG00000244692	2.447971227	0	3.623732946	0.140272942	1	RN7SL724P
ENSG00000198961	3259.331653	2460.225176	0.405871653	0.140274206	0.233994488	PJA2
ENSG00000251396	14.55304551	25.42861428	-0.81045455	0.140333202	0.234077871	LINC01301
ENSG00000171488	757.2094964	596.9024177	0.34364589	0.140352198	0.234094527	LRRC8C
ENSG00000238217	2.446717578	0	3.623072572	0.140361289	1	LINC01877
ENSG00000231165	2.446717578	0	3.623072572	0.140361289	1	TRBV26OR9-2
ENSG00000279940	2.446717578	0	3.623072572	0.140361289	1	AC073862.3
ENSG00000176601	2.446717578	0	3.623072572	0.140361289	1	MAP3K19
ENSG00000285752	21.58581125	10.76215704	1.005963246	0.140450007	0.234242626	AL031281.2
ENSG00000165417	758.7721172	947.4710943	-0.320354532	0.140516702	0.234338817	GTF2A1
ENSG00000215717	1132.161005	872.5512474	0.375847951	0.14053582	0.234351565	TMEM167B
ENSG00000271780	41.99922858	24.76615854	0.761294222	0.140542386	0.234351565	AL118558.3
ENSG00000104290	193.0106407	120.7629732	0.678600701	0.140615433	0.234458321	FZD3
ENSG00000261594	21.14317169	7.856654121	1.438413372	0.140661999	0.234520837	TPBGL
ENSG00000170946	391.9392219	316.7767534	0.307219887	0.140673277	0.234520837	DNAJC24
ENSG00000270108	32.83261756	18.66023363	0.816160435	0.140683417	0.234520837	AL049840.5
ENSG00000152409	574.6677708	379.8367819	0.598006534	0.140689033	0.234520837	JMY
ENSG00000269176	14.26865147	6.756483895	1.072665834	0.140708184	0.234537713	AP001160.3
ENSG00000224261	7.28094616	2.527610546	1.530177906	0.140732806	0.234563704	RPSAP18
ENSG00000198298	54.27279105	74.5867162	-0.459472882	0.140777409	0.234622995	ZNF485
ENSG00000176842	18.73169882	44.1586402	-1.240236297	0.140817116	0.234674118	IRX5
ENSG00000267530	27.05103998	9.040953539	1.573862321	0.140845218	0.234705894	LINC01836
ENSG00000206888	2.990361403	0.336469917	2.954874487	0.140892223	0.234769166	RNU6-48P
ENSG00000268364	16.84227301	6.960770875	1.263726525	0.141011142	0.234952254	SMC5-AS1
ENSG00000111653	667.1956693	472.4350652	0.498559167	0.141025604	0.234961281	ING4
ENSG00000278987	4.767428842	14.35822875	-1.585781737	0.141128854	0.235118227	AL031009.1
ENSG00000287828	4.585079253	11.2308099	-1.294195227	0.14114561	0.235131065	AL354743.2
ENSG00000155368	1922.817196	1522.686231	0.336660524	0.141351822	0.235445768	DBI
novel.497	2.06654734	12.92004755	-2.63474016	0.141352645	0.235445768	-
ENSG00000110680	0	3.300895859	-4.302205811	0.141383597	0.235482084	CALCA
ENSG00000162779	5.9643541	1.00940975	2.526756023	0.141392576	0.235482084	AXDND1
ENSG00000161243	128.6904046	87.01650075	0.567582525	0.141575435	0.235765563	AC010605.1
ENSG00000272419	75.54236815	141.6204648	-0.905772402	0.141580937	0.235765563	LINC01145
ENSG00000122386	314.9958118	225.4606411	0.483484103	0.141596487	0.235776347	ZNF205
ENSG00000127603	3428.59242	4357.651319	-0.345949568	0.141660597	0.235867981	MACF1
ENSG00000228369	0	2.211209725	-3.721155613	0.141698568	1	TXNDC12-AS1
ENSG00000226790	0	2.211209725	-3.721155613	0.141698568	1	HNRNPA3P1
ENSG00000274949	0	2.211209725	-3.721155613	0.141698568	1	AL109804.1
ENSG00000248568	3.609580508	0	4.186014824	0.141705	0.235926792	KRT8P48
ENSG00000168297	594.7008204	441.7406153	0.429347967	0.141727761	0.235949568	PXK
ENSG00000078237	247.6412929	149.6325471	0.725420624	0.141795896	0.236047874	TIGAR
novel.179	2.668144092	0	3.75221187	0.14195985	1	-

ENSG00000234718	2.668144092	0	3.75221187	0.14195985	1	AC007161.1
ENSG00000142528	138.8444101	178.014928	-0.358348004	0.141967095	0.236317728	ZNF473
ENSG00000071575	1832.715966	1260.613731	0.539639029	0.141977304	0.236319581	TRIB2
ENSG00000256525	246.3876683	313.2372929	-0.346029043	0.142000794	0.236341838	POLG2
ENSG00000206712	1.538018572	6.123682254	-2.001398521	0.142010279	0.236341838	RNU6-26P
ENSG00000230006	5.826024761	1.10029862	2.415310597	0.142017966	0.236341838	ANKRD36BP2
ENSG00000136279	2392.23054	3051.734618	-0.351350273	0.142070859	0.236414718	DBNL
ENSG00000197744	28.12557767	41.80437826	-0.572886866	0.142114669	0.236472474	PTMAP2
ENSG00000235078	11.5413008	4.617249819	1.315901673	0.14214289	0.236504285	AC231981.1
ENSG00000162105	42.38211958	17.87402408	1.244002252	0.142169556	0.2365203	SHANK2
ENSG00000260276	30.16064125	45.98365681	-0.609073653	0.142170722	0.2365203	AC022167.2
ENSG00000275005	2.420248412	0	3.608993142	0.142251441	1	AL354950.1
ENSG00000249639	2.420248412	0	3.608993142	0.142251441	1	AC022092.1
ENSG00000181778	2.420248412	0	3.608993142	0.142251441	1	TMEM252
ENSG00000143167	2.420248412	0	3.608993142	0.142251441	1	GPA33
ENSG00000239218	1.54052587	6.735258924	-2.139191637	0.142380382	0.236853932	RPS20P22
ENSG00000223138	3.839853833	0	4.276369157	0.142401851	0.236874478	RNA5SP450
novel.169	7.260745237	1.813376645	2.006526152	0.142478836	0.236987364	-
ENSG00000186732	2.416487466	0	3.606994444	0.14252256	1	MPPED1
ENSG00000248884	2.416487466	0	3.606994444	0.14252256	1	AC010280.2
ENSG00000276814	9.9717272	3.995060664	1.327236527	0.142543822	0.237080278	AC004801.6
ENSG00000230280	2.72484337	7.540381694	-1.468549201	0.14258586	0.237135017	HNRNPA1P59
ENSG00000249348	53.78932271	37.34053635	0.527326679	0.142604852	0.237151421	UGDH-AS1
ENSG00000114742	873.9580875	709.6583766	0.300506125	0.142645787	0.237204313	WDR48
ENSG00000224687	15.14955639	28.30099547	-0.904912832	0.142669123	0.237227935	RASAL2-AS1
ENSG00000100242	2190.058193	1686.020668	0.377369988	0.142694263	0.237241431	SUN2
ENSG00000067191	160.0171003	222.9484761	-0.477222305	0.142695502	0.237241431	CACNB1
ENSG00000279897	3.900314058	0.743759607	2.4218337	0.142767552	0.23734603	BIRC6-AS2
ENSG00000287095	4.484288455	0.713078025	2.648965952	0.14278713	0.237363391	AF228727.1
ENSG00000280023	0	3.596071708	-4.427600034	0.142798933	0.237367823	AD000813.1
ENSG00000267510	24.84699002	11.92625897	1.051470083	0.142820861	0.237389085	AC011451.1
ENSG00000234779	8.933545666	33.33285542	-1.903390581	0.14284578	0.237415315	BNC2-AS1
ENSG00000077721	1324.818787	1613.229618	-0.284322573	0.142870211	0.237440732	UBE2A
ENSG00000109756	1071.958268	769.4655158	0.478661884	0.142891184	0.237460397	RAPGEF2
ENSG00000253200	56.90067583	38.80811486	0.549128518	0.142912568	0.237473307	AC037459.2
ENSG00000132824	2945.141356	3716.888993	-0.335765122	0.142917233	0.237473307	SERINC3
ENSG00000227972	3.062175737	0.356539013	2.984265892	0.142982353	0.237566317	THAP12P3
ENSG00000149218	5183.190133	3050.198489	0.764860527	0.143005799	0.23759008	ENDOD1
ENSG00000261407	0	3.383483993	-4.335665345	0.143084215	0.23770516	AC013565.3
ENSG00000262312	7.584216197	2.241891306	1.780736032	0.143256021	0.237975364	AC004494.1
ENSG00000226031	2.405133356	0	3.600907272	0.143348218	1	FGF13-AS1
ENSG00000213918	348.780963	261.6484111	0.415114367	0.143359108	0.238131384	DNASE1
ENSG00000165731	24.44801505	11.4739508	1.101433041	0.14342451	0.238224792	RET
ENSG00000215795	8.359386996	2.058957692	2.001408455	0.143454112	0.238238458	AL390728.2
ENSG00000198857	6.986451663	16.38406472	-1.22807314	0.1434565	0.238238458	HSD3BP5
ENSG00000131725	350.6416536	459.3985368	-0.389587515	0.143460247	0.238238458	WDR44
ENSG00000169105	725.0314139	907.5337759	-0.32419754	0.143485121	0.238258713	CHST14
ENSG00000214273	3.588197208	0	4.17787958	0.143501151	0.238258713	AGGF1P1
ENSG00000138161	3.588197208	0	4.17787958	0.143501151	0.238258713	CUZD1
ENSG00000151572	15.1609105	29.10342131	-0.93629287	0.143509126	0.238258713	ANO4
ENSG00000285081	2.402626059	0	3.599568875	0.143531038	1	AC004593.2

ENSG00000228923	2.402626059	0	3.599568875	0.143531038	1	AP000355.1
ENSG00000272696	22.88374077	8.671233501	1.41173119	0.143537298	0.238290258	AL359091.4
ENSG00000237595	1.50778846	5.747074145	-1.927998437	0.143558086	0.238309542	LINC01275
ENSG00000027644	20.64222319	3.761376372	2.446138045	0.143571309	0.238316264	INSRR
ENSG00000277351	3.62978143	0	4.193669719	0.143625234	0.238390546	AC013553.3
ENSG00000198589	598.4515769	758.2967238	-0.341417348	0.143658063	0.238429802	LRBA
ENSG00000225822	5.439586279	1.069617038	2.341338639	0.143765153	0.238592299	UBXN7-AS1
ENSG00000188529	1725.259104	2114.913853	-0.293822389	0.143787627	0.238601276	SRSF10
ENSG00000057294	397.7362265	141.4765732	1.490563567	0.14378893	0.238601276	PKP2
ENSG00000172971	1.521649867	7.000909067	-2.208375926	0.143812246	0.238624727	UNC93B3
ENSG00000089505	35.50222876	22.91978856	0.629642575	0.144113679	0.239109617	CMTM1
ENSG00000111906	784.3239872	1126.281307	-0.522324698	0.144149159	0.239153212	HDDC2
ENSG00000267424	0	2.180528143	-3.703638643	0.144161391	1	AC020934.1
ENSG00000257727	209.7043139	146.9926475	0.513509612	0.144185763	0.239198666	CNPY2
ENSG00000239830	3.021845164	0.387220594	2.967745388	0.144301724	0.239375755	RPS4XP22
ENSG00000168067	461.8072426	628.4651515	-0.444109981	0.144335511	0.239416518	MAP4K2
ENSG00000110318	232.4545539	147.8158659	0.654846741	0.144369027	0.239456824	CEP126
ENSG00000254506	3.696509898	0	4.218712312	0.144448472	0.239573301	AP003080.1
ENSG00000182257	22.40277974	8.685570401	1.358424662	0.144527502	0.239689075	PRR34
novel.212	3.016830569	0.387220594	2.965698876	0.14455113	0.239712958	-
ENSG00000174977	223.103442	166.3070833	0.423263785	0.144585365	0.239754427	AC026271.1
ENSG00000258701	30.6554637	18.8845897	0.694870946	0.144689992	0.23991261	LINC00638
ENSG00000118271	3.688988006	0	4.215914936	0.144820926	0.240068428	TTR
ENSG00000250891	3.688988006	0	4.215914936	0.144820926	0.240068428	LINC02208
ENSG00000286805	3.688988006	0	4.215914936	0.144820926	0.240068428	AL138716.1
ENSG00000201724	3.688988006	0	4.215914936	0.144820926	0.240068428	RF00019
ENSG00000175197	661.5181324	436.4246118	0.600706636	0.144899646	0.240171989	DDIT3
ENSG00000261175	16.61486299	3.873490212	2.095268204	0.144901887	0.240171989	LINC02188
ENSG00000226824	29.70643412	46.13102855	-0.632301069	0.144960455	0.240253737	AC006001.2
ENSG00000261052	5.14759908	12.05600176	-1.222762693	0.145002258	0.240307691	SULT1A3
ENSG00000116288	3751.944185	4752.849584	-0.341195266	0.14509859	0.240452	PARK7
ENSG00000267192	3.649911081	0.693008929	2.364567487	0.145172405	0.24055898	AC006116.4
ENSG00000260144	3.016830569	0.356539013	2.965695397	0.145243308	0.240661122	AC100827.2
ENSG00000249986	0.909952655	4.433031935	-2.278206746	0.145342802	0.240810621	YWHAQP6
ENSG00000204815	44.74391687	30.30778983	0.564820227	0.145419041	0.240921573	TTC25
ENSG00000270112	1.200686205	5.349241065	-2.152211385	0.145457049	0.240969176	AC090241.2
ENSG00000167118	989.3991883	1261.779409	-0.351008067	0.145471041	0.24097699	URM1
ENSG00000255154	5.072023799	1.110911105	2.215748769	0.145585747	0.241151628	HTD2
ENSG00000144026	548.8277031	413.9585909	0.407622619	0.145633006	0.241214531	ZNF514
ENSG00000147813	42.44745221	80.6893831	-0.92771109	0.145733556	0.241365686	NAPRT
ENSG00000145723	139.9965597	103.5026276	0.43482463	0.145915343	0.241651359	GIN1
ENSG00000272205	5.198030114	1.10029862	2.252162584	0.145996947	0.241771093	AL451050.2
ENSG00000137860	1.201939854	5.012771149	-2.05652718	0.146121467	0.241961876	SLC28A2
ENSG00000248126	5.18166141	1.029478846	2.300018358	0.146131986	0.241963873	AC091849.1
ENSG00000103044	38.07939779	21.14526584	0.854525142	0.146188746	0.242042431	HAS3
ENSG00000253394	3.579350395	0	4.174479825	0.146206438	0.242056296	LINC00534
ENSG00000207973	0.293240848	4.057708096	-3.646341414	0.14622604	0.242073324	MIR589
ENSG00000171794	2.449224876	0	3.624454056	0.146297163	1	UTF1
ENSG00000242113	2.449224876	0	3.624454056	0.146297163	1	RN7SL124P
ENSG00000007350	2.449224876	0	3.624454056	0.146297163	1	TKTL1
ENSG00000268027	2.449224876	0	3.624454056	0.146297163	1	AC243960.1

ENSG00000260302	2.449224876	0	3.624454056	0.146297163	1	AP005482.1
ENSG00000226849	8.687943822	2.435565801	1.820673298	0.146297913	0.242147648	AL109811.1
ENSG00000117500	1247.882343	1577.707292	-0.338541914	0.146298337	0.242147648	TMED5
ENSG00000277991	7.934227594	2.241891306	1.843267109	0.146299891	0.242147648	FP236241.1
ENSG00000077713	618.3545307	482.0506803	0.35871842	0.146312292	0.242147648	SLC25A43
ENSG00000258659	3.619680969	0.693008929	2.354854385	0.146317537	0.242147648	TRIM34
ENSG00000184524	65.84732746	129.7127811	-0.977820769	0.146337967	0.242166033	CEND1
ENSG00000163635	21.34167652	9.312464272	1.196337251	0.146441046	0.242301866	ATXN7
ENSG00000153815	614.0022439	837.8764464	-0.448499679	0.146442798	0.242301866	CMIP
ENSG00000188396	6.554596742	19.05228733	-1.535643749	0.146449702	0.242301866	TCTEX1D4
ENSG00000147873	3.5743358	0	4.172563871	0.14646668	0.242301866	IFNA5
ENSG00000183632	3.5743358	0	4.172563871	0.14646668	0.242301866	TP53TG3
ENSG00000149091	960.7063175	1163.014419	-0.27581139	0.146537817	0.24239898	DGKZ
ENSG00000106113	4.855611881	1.029478846	2.204856357	0.146544043	0.24239898	CRHR2
ENSG00000259377	2.44546393	0	3.622464245	0.14657063	1	AC026770.1
ENSG00000198670	2.44546393	0	3.622464245	0.14657063	1	LPA
ENSG00000231789	12.99991188	6.379875787	1.019813625	0.146660914	0.242576852	PIK3CD-AS2
ENSG00000121068	1695.431688	2303.763938	-0.442293989	0.146677892	0.242589491	TBX2
ENSG00000226913	6.712086818	2.211209725	1.617414248	0.14678491	0.242751035	BSN-DT
ENSG00000286650	3.064683035	0.356539013	2.985347209	0.146820339	0.242794172	AL139234.1
ENSG00000249258	7.41795058	1.722487775	2.087898865	0.1468566	0.242838679	AC079193.2
ENSG00000156232	344.4981849	274.8555094	0.32617436	0.146898012	0.242891697	WHAMM
ENSG00000165568	24.98148714	42.40893847	-0.764929388	0.146964673	0.242972361	AKR1E2
ENSG00000198918	4603.514784	6115.156565	-0.409663603	0.146970244	0.242972361	RPL39
ENSG00000230943	9.785616665	3.607840069	1.440383657	0.146974852	0.242972361	LINC02541
ENSG00000232606	5.688735607	1.069617038	2.407528987	0.147003348	0.242989941	LINC01412
ENSG00000100417	696.1489167	918.3843008	-0.399618153	0.147008897	0.242989941	PMM1
ENSG00000118939	27.60338842	45.7709407	-0.72914818	0.147013544	0.242989941	UCHL3
ENSG00000279425	23.00158446	9.344173335	1.311820604	0.147175612	0.243242339	AC092279.2
ENSG00000236449	7.195270418	2.241891306	1.708670243	0.147199557	0.243266439	AC010894.3
ENSG00000260456	21.30155941	43.31453511	-1.026712822	0.147224407	0.243292032	C16orf95
ENSG00000107890	236.8862604	310.8528158	-0.392105002	0.147269499	0.243351068	ANKRD26
ENSG00000128581	264.4746756	358.0325536	-0.437659786	0.147280097	0.243353104	IFT22
ENSG00000278071	5.230767524	0.743759607	2.834039264	0.14729701	0.243365571	AL161669.3
ENSG00000271860	2.435363469	0	3.617071615	0.147311292	1	AL589740.1
ENSG00000254401	2.435363469	0	3.617071615	0.147311292	1	AC111188.1
ENSG00000276302	2.435363469	0	3.617071615	0.147311292	1	AL021997.3
ENSG00000149646	5.453447686	1.396630344	1.946475077	0.147316151	0.243381719	CNBD2
ENSG00000248027	3.060922088	0.336469917	2.983823073	0.14745431	0.243587127	AP001351.1
ENSG00000047230	323.6023672	422.3455651	-0.383560763	0.147459233	0.243587127	CTPS2
ENSG00000286347	0	2.140389951	-3.680034809	0.147509575	1	AC114802.1
ENSG00000279903	0	2.140389951	-3.680034809	0.147509575	1	AP006248.3
ENSG00000171451	0	2.140389951	-3.680034809	0.147509575	1	DSEL
ENSG00000254244	0.293240848	2.547679642	-2.964650038	0.147583632	0.243777122	PAICSP4
novel.825	92.62307746	42.9168776	1.106039127	0.14763495	0.243846385	-
ENSG00000255587	2.430348874	0	3.614406644	0.147679995	1	RAB44
ENSG00000167807	2.430348874	0	3.614406644	0.147679995	1	AC011511.1
ENSG00000229648	2.430348874	0	3.614406644	0.147679995	1	RPSAP22
ENSG00000262096	2.430348874	0	3.614406644	0.147679995	1	PCDHB19P
ENSG00000228918	2.430348874	0	3.614406644	0.147679995	1	LINC01344
ENSG00000244044	2.430348874	0	3.614406644	0.147679995	1	RN7SL735P

ENSG00000177697	7210.294515	9350.847726	-0.375008413	0.147700669	0.243939424	CD151
ENSG00000271849	10.58843901	2.355289417	2.14450711	0.147832924	0.244142334	AC012603.1
ENSG00000256229	122.9856563	171.6131037	-0.482222953	0.147915135	0.244262577	ZNF486
ENSG00000107821	226.3246306	660.3182224	-1.545004963	0.147968029	0.244334394	KAZALD1
novel.393	21.62391926	46.40968415	-1.101228009	0.148032036	0.244424551	-
ENSG00000244509	949.2482709	1256.269602	-0.404393826	0.148099547	0.244520483	APOBEC3C
ENSG00000142546	860.3802119	1096.030151	-0.349246977	0.148142421	0.244570246	NOSIP
ENSG00000267064	11.2518209	22.33876508	-0.986303796	0.148148514	0.244570246	UXT-AS1
ENSG00000053438	138.0064408	43.66405766	1.65901637	0.148271171	0.244757182	NNAT
ENSG00000280828	11.9312155	3.912472529	1.60645264	0.148337953	0.244851865	AC090114.3
ENSG00000152580	26.86038556	12.19776971	1.140298225	0.148361058	0.244874446	IGSF10
ENSG00000238913	0	2.129777466	-3.674069902	0.148393852	1	RNU7-196P
ENSG00000274879	0	2.129777466	-3.674069902	0.148393852	1	AC009804.1
ENSG00000213030	0	2.129777466	-3.674069902	0.148393852	1	CGB8
ENSG00000224985	1.846374476	6.613560079	-1.843751602	0.148473767	0.245044908	AL590714.1
ENSG00000013561	1046.443948	835.4135639	0.324602066	0.148502449	0.245076678	RNF14
ENSG00000187079	4660.191113	3269.121142	0.51143731	0.148534523	0.245114039	TEAD1
ENSG00000170379	1.773306493	7.408198758	-2.054981539	0.148595896	0.245199744	TCAF2
ENSG00000149927	28.9006772	14.32053071	1.005806765	0.148624752	0.245219206	DOC2A
ENSG00000164743	33.42439859	50.8453318	-0.608582736	0.148626567	0.245219206	C8orf48
ENSG00000148690	645.8574405	521.7547127	0.307861522	0.148653333	0.245247793	FRA10AC1
ENSG00000250384	0	3.12857473	-4.229776038	0.148676193	0.245269933	UBE2CP3
novel.594	10.34939014	4.199347643	1.285715079	0.148725211	0.245335219	-
ENSG00000274276	49.36152007	117.4076619	-1.250911187	0.148761342	0.245365818	CBSL
ENSG00000162695	573.6596483	743.7146314	-0.374794446	0.148762648	0.245365818	SLC30A7
ENSG00000232909	5.760549941	12.3217803	-1.104026724	0.148786127	0.245388965	AL157823.2
ENSG00000238083	29.33246085	15.00981522	0.972678126	0.14880166	0.245399005	LRRC37A2
ENSG00000128607	954.4220681	783.7625826	0.283989757	0.148863539	0.245485472	KLHDC10
novel.1050	4.007373099	0	4.335340279	0.148873091	0.245485641	-
ENSG00000270629	261.7841087	347.6799036	-0.409807106	0.149064958	0.245777695	NBPF14
ENSG00000101220	803.846842	1055.597547	-0.393376513	0.149073312	0.245777695	C20orf27
ENSG00000285650	7.81699682	2.537067156	1.630221891	0.149078584	0.245777695	AL157827.2
ENSG00000255434	6.958728849	2.51699806	1.468487543	0.149155408	0.245888747	AP001922.6
ENSG00000226853	11.26818961	3.781445468	1.55522896	0.149184308	0.245920786	AC010894.2
ENSG00000148450	379.4621679	511.0363374	-0.429372745	0.149221218	0.245966023	MSRB2
ENSG00000171843	182.0948269	269.827327	-0.566604544	0.149235582	0.245974095	MLLT3
novel.1031	42.25339251	25.91361181	0.710082989	0.149282134	0.246016426	-
ENSG00000112658	1060.150751	1365.09742	-0.36477165	0.149286768	0.246016426	SRF
ENSG00000161217	1240.092771	1550.409203	-0.322377003	0.149289672	0.246016426	PCYT1A
ENSG00000186468	5666.159207	7452.707897	-0.395375511	0.149302915	0.246022644	RPS23
ENSG00000273733	7.209131825	2.547679642	1.51208701	0.149345913	0.24607789	AC011472.4
ENSG00000272620	23.34819056	7.213368388	1.694422272	0.14936804	0.246084044	AFAP1-AS1
ENSG00000254122	103.4517477	138.7854433	-0.423430367	0.149368592	0.246084044	PCDHGB7
ENSG00000287781	0	3.270214277	-4.289627242	0.149388558	0.24608573	AC018714.2
ENSG00000225751	0	3.270214277	-4.289627242	0.149388558	0.24608573	AC087501.1
ENSG00000078403	859.5150947	643.8309102	0.41714266	0.149410342	0.246100099	MLLT10
ENSG00000179832	766.2481079	1142.266213	-0.575749927	0.14941998	0.246100099	MROH1
ENSG00000070759	141.3339221	77.26002904	0.873249983	0.149425698	0.246100099	TESK2
ENSG00000185236	1585.5843	2077.735764	-0.389988349	0.149458321	0.246138225	RAB11B
ENSG00000246792	8.74331818	2.904218654	1.59685538	0.149492134	0.246149333	AC106038.1
ENSG00000224190	3.993582963	0	4.33056934	0.149499131	0.246149333	LINC02667

ENSG00000204338	3.993511692	0	4.330534982	0.149502963	0.246149333	CYP21A1P
ENSG00000285476	3.993511692	0	4.330534982	0.149502963	0.246149333	AC139491.7
ENSG00000280128	0.308355904	3.465044647	-3.417371206	0.149618076	0.246323251	AL662795.2
novel.1069	11.38004978	2.0188195	2.474430034	0.149691236	0.246428082	-
ENSG00000156531	591.722596	755.4084778	-0.352010917	0.149718213	0.246450546	PHF6
ENSG00000106392	656.7177103	829.992638	-0.338013404	0.149723853	0.246450546	C1GALT1
ENSG00000280670	50.82342172	27.3622771	0.893439828	0.149766213	0.246494987	CCDC163
ENSG00000173261	13.84689701	6.562809401	1.086955452	0.149769827	0.246494987	PLAC8L1
ENSG00000166183	4.27672335	0.672939833	2.618706283	0.1498676	0.246640281	ASPG
ENSG00000170310	529.7711646	416.2607367	0.348645705	0.149893192	0.246666773	STX8
ENSG00000135477	0	3.108505634	-4.221080734	0.149911841	0.246681838	KRT87P
ENSG00000127364	24.96386479	14.08427787	0.830095142	0.149950923	0.246730521	TAS2R4
ENSG00000286340	15.70204703	7.74582455	1.010375219	0.149979221	0.246761455	AL356776.2
ENSG00000164879	16.94814967	7.408198758	1.186867129	0.149995592	0.246772763	CA3
ENSG00000136861	1026.121523	1310.2162	-0.352652672	0.150051697	0.246849436	CDK5RAP2
ENSG00000250274	6.451298646	0	5.024101335	0.150093611	0.246901782	AC034199.1
ENSG00000163618	1.522903516	5.93000776	-1.957121581	0.150102523	0.246901782	CADPS
ENSG00000251556	0	2.10970837	-3.66210823	0.150128317	1	AC091887.1
ENSG00000253102	0	2.10970837	-3.66210823	0.150128317	1	AC004707.1
ENSG00000273012	0	2.10970837	-3.66210823	0.150128317	1	AL353600.1
ENSG00000234390	29.72029552	18.55873227	0.67619598	0.150210327	0.247060807	USP27X-AS1
ENSG00000258861	0	3.148643826	-4.238383214	0.15021822	0.247060807	MIR381HG
novel.698	90.96385371	129.793875	-0.511422572	0.150248593	0.247079533	-
ENSG00000170100	218.6033265	172.0945052	0.344622122	0.150248626	0.247079533	ZNF778
ENSG00000170409	0.293240848	2.862924587	-3.137814111	0.15027852	0.247112371	AC091390.1
ENSG00000255725	1.803536605	5.676254372	-1.653623627	0.150287617	0.247112371	TDGP1
ENSG00000260325	7.488511265	2.527610546	1.573558066	0.150432327	0.247326545	HSPB9
ENSG00000103024	1112.019105	872.6152738	0.349999287	0.150436911	0.247326545	NME3
ENSG00000237986	4.706968617	0.356539013	3.608925181	0.150450236	0.247332801	CELF2-AS2
ENSG00000285230	35.67071695	52.87103937	-0.566655368	0.150461244	0.247335247	RALY-AS1
novel.725	225.8723056	354.4118583	-0.649181512	0.150511835	0.247402758	-
ENSG00000101558	3626.208979	2936.171686	0.304581047	0.150560756	0.247467515	VAPA
ENSG00000064835	2.726097019	0.387220594	2.818663169	0.150666093	0.247624984	POU1F1
ENSG00000176783	42.36894236	73.72043988	-0.799395926	0.150683503	0.247634171	RUFY1
ENSG00000270832	0.293240848	2.496928964	-2.941196341	0.150690745	0.247634171	AC092120.2
ENSG00000251189	2.3900183	0	3.592708051	0.150693873	1	AC010425.1
ENSG00000088002	7.136063842	1.793307549	1.994599047	0.150725134	0.247675018	SULT2B1
ENSG00000089280	6137.830941	7940.998217	-0.371616941	0.150788798	0.247763962	FUS
ENSG00000230661	2.388764651	0	3.592032746	0.15078918	1	YY1P1
ENSG00000236732	2.388764651	0	3.592032746	0.15078918	1	AC094019.1
ENSG00000230992	2.388764651	0	3.592032746	0.15078918	1	FAM201B
ENSG00000287508	2.388764651	0	3.592032746	0.15078918	1	AC009560.3
ENSG00000219361	2.388764651	0	3.592032746	0.15078918	1	RPSAP72
ENSG00000261465	2.388764651	0	3.592032746	0.15078918	1	AC099518.4
ENSG00000170153	123.1526339	58.98351402	1.059512242	0.150911367	0.247949677	RNF150
ENSG00000138623	3111.126275	4713.173156	-0.599194908	0.150954627	0.24800507	SEMA7A
ENSG00000270147	8.850448493	2.772035718	1.655055633	0.150988578	0.248045162	AC068620.2
ENSG00000122966	1499.918011	956.7383299	0.648696808	0.151026043	0.248091024	CIT
ENSG00000128609	1155.604475	935.8324468	0.304266849	0.151126535	0.248240407	NDUFA5
ENSG00000182544	566.2910455	722.1116295	-0.35099343	0.151158427	0.248277096	MFSD5
ENSG00000278985	17.0409913	7.174386071	1.249811961	0.151174809	0.248288306	AC092718.7

ENSG00000250337	7.66863829	22.40598883	-1.545964296	0.1513114	0.248496933	PURPL
ENSG00000271092	19.28397599	10.4965069	0.883053093	0.15134663	0.248539081	TMEM56-RWDD3
novel.1075	89.13628396	124.6538824	-0.482765608	0.151362786	0.248549901	-
ENSG00000090266	1173.69047	1578.794213	-0.427921129	0.151414607	0.248619283	NDUFB2
ENSG00000233009	3.560474393	0	4.167206274	0.151424943	0.248620541	NALCN-AS1
ENSG00000272334	2.726097019	0.356539013	2.818663835	0.151476695	0.248685338	AC011816.2
ENSG00000224837	68.34147058	109.2290375	-0.678850133	0.151483552	0.248685338	GCSHP5
ENSG00000287738	4.150717034	0.356539013	3.427334391	0.151521582	0.248732053	AL359764.3
ENSG00000078699	578.0308949	429.2479609	0.430087306	0.15154178	0.248742963	CBFA2T2
ENSG00000270953	17.71844807	3.92192914	2.173089956	0.151547375	0.248742963	AC007938.3
ENSG00000230805	3.697763547	0	4.219224898	0.151593884	0.248803581	AL132709.1
ENSG00000169583	23.20971903	63.75918043	-1.460103942	0.151624738	0.248838502	CLIC3
ENSG00000130348	417.8895408	310.6457163	0.427025846	0.151724621	0.248986697	QRSL1
ENSG00000099290	1690.223539	1205.221545	0.4879679	0.151769084	0.249043932	WASHC2A
ENSG00000137573	195.7286022	1018.601959	-2.37972614	0.151779688	0.249045603	SULF1
ENSG00000286881	6.673081165	1.029478846	2.673216463	0.151860172	0.249161927	AL358780.1
ENSG00000151466	255.6192563	339.0148346	-0.40769872	0.151901433	0.249213888	SCLT1
ENSG00000261513	0.308355904	3.444975551	-3.409754143	0.151938762	0.249259392	AC009097.4
ENSG00000225611	3.649911081	0.743759607	2.323542466	0.151965749	0.249287924	LINC02158
ENSG00000189127	3.687734357	0	4.215487457	0.152102927	0.249481449	ANKRD34B
ENSG00000267130	3.687734357	0	4.215487457	0.152102927	0.249481449	AC008738.2
ENSG00000126583	0.601596751	4.401194479	-2.863876382	0.152241288	0.249688282	PRKCG
ENSG00000212421	0	3.415449845	-4.357082703	0.152248249	0.249688282	RF00568
ENSG00000261329	8.913131281	2.884149558	1.633634905	0.152280963	0.24972617	AC016597.1
ENSG00000230306	8.822725678	3.016332495	1.57344536	0.152306928	0.249752985	BANF1P2
ENSG00000186312	88.17202497	133.7333047	-0.600527571	0.15234135	0.249793664	CA5BP1
ENSG00000111224	188.300595	147.6549748	0.351639163	0.152365779	0.249817954	PARP11
ENSG00000173894	93.57977148	132.2293124	-0.497876731	0.152403823	0.249864564	CBX2
ENSG00000184058	48.97579325	117.1442166	-1.257741007	0.152521287	0.250041365	TBX1
ENSG00000259542	5.687481958	1.396630344	2.010948687	0.152553393	0.250069502	AC087477.5
ENSG00000255310	13.51834019	6.032793384	1.160064552	0.1525577	0.250069502	AF131215.5
ENSG00000261054	4.790137061	0.774441189	2.68029123	0.152649612	0.250204377	AC036108.2
ENSG00000138311	31.59786903	14.84566643	1.098213159	0.15271553	0.250296631	ZNF365
ENSG00000171067	2242.837588	1627.103107	0.46305922	0.152728314	0.250301794	C11orf24
ENSG00000197122	673.0877693	917.1856729	-0.446033153	0.152769978	0.250354039	SRC
ENSG00000286943	8.339257344	2.200597239	1.934027386	0.152779465	0.250354039	AP001992.2
ENSG00000085185	263.5000039	366.1636125	-0.473876066	0.152910154	0.250552391	BCORL1
ENSG00000166016	231.0735272	309.9046412	-0.422396238	0.152949321	0.250600764	ABTB2
ENSG00000167733	122.9046392	172.6802338	-0.488915051	0.153001977	0.25067123	HSD11B1L
ENSG00000005381	6.469205734	0.713078025	3.175794786	0.153097777	0.250812368	MPO
ENSG00000185022	823.6637251	1306.983681	-0.665884715	0.153212208	0.250984009	MAFF
ENSG00000124678	2.128261214	7.245205845	-1.767423298	0.153363681	0.251208093	TCP11
ENSG00000143971	469.1226662	347.8510688	0.431530471	0.153368337	0.251208093	ETAA1
ENSG00000259556	8.67784336	17.78300681	-1.02783911	0.153445177	0.251318108	AC090971.3
ENSG00000060971	972.2757847	784.2226776	0.309873977	0.153491863	0.251378725	ACAA1
ENSG00000255026	8.567236835	2.11916498	2.006000334	0.153591418	0.251525915	AC136475.3
ENSG00000124659	351.2368827	448.2249121	-0.351974392	0.153618177	0.25155388	TBCC
novel.564	3.657504245	0	4.204142865	0.153654677	0.251597792	-
ENSG00000048540	20.06319247	8.663189555	1.199304617	0.153733151	0.25170679	LMO3
ENSG00000150401	358.8711034	282.7639888	0.344228316	0.15374062	0.25170679	DCUN1D2
ENSG00000270195	20.51363866	11.52598575	0.83455635	0.153840847	0.251855012	AC016773.1

ENSG00000080371	1343.291435	1092.595856	0.297956954	0.153897688	0.251926412	RAB21
ENSG00000112511	43.53918499	21.52290143	1.02352071	0.153903853	0.251926412	PHF1
ENSG00000164258	746.153017	922.7434986	-0.306572828	0.153965334	0.252011173	NDUFS4
ENSG00000238390	7.18140901	2.241891306	1.705617743	0.154012806	0.252072994	RF01241
ENSG00000143158	581.6706896	811.1187679	-0.480119508	0.154047875	0.25211451	MPC2
novel.562	0.908699006	3.943154111	-2.118583016	0.154096528	0.25217825	-
ENSG00000243667	116.4293787	150.4246988	-0.36935955	0.154134712	0.25222485	WDR92
ENSG00000070831	4243.054102	5198.957696	-0.293169838	0.1541897	0.252298942	CDC42
ENSG00000103485	741.140084	502.33783	0.561459423	0.154203568	0.252305743	QPRT
ENSG00000168827	522.3691124	738.0643192	-0.499122304	0.154254151	0.252370944	GFM1
ENSG00000111540	2802.805342	2365.695782	0.244686479	0.154262844	0.252370944	AC034102.1
ENSG00000198911	3302.636477	2451.273574	0.430151958	0.154291881	0.252402553	SREBF2
ENSG00000116151	85.59608176	122.8082831	-0.518816031	0.154380443	0.252531529	MORN1
ENSG00000111361	865.2420635	1119.614882	-0.372104993	0.154429566	0.252595981	EIF2B1
ENSG00000095303	711.9389726	950.6255519	-0.417032761	0.154486335	0.252672928	PTGS1
ENSG00000227347	1.789675198	6.471920532	-1.846121763	0.154519158	0.252710704	HNRNPKP2
ENSG00000178146	7.911519374	16.21174359	-1.03620894	0.154561684	0.252764343	AL672207.1
ENSG00000134046	1617.075559	1253.031259	0.367915777	0.154605003	0.252819271	MBD2
ENSG00000114383	545.7136838	704.0168325	-0.367501604	0.154645389	0.252869397	TUSC2
ENSG00000279637	4.208669961	0.713078025	2.556537559	0.154659375	0.252876352	AC018445.3
ENSG00000133216	411.7086316	644.4505999	-0.646076725	0.154791908	0.253077123	EPHB2
ENSG00000273274	6.722116008	1.069617038	2.646634073	0.154804056	0.253081058	ZBTB8B
ENSG00000225972	10.22721604	3.300895859	1.641527112	0.154829581	0.253106862	MTND1P23
ENSG00000262919	359.1671362	473.3783381	-0.398866727	0.154880605	0.253174342	CCNQ
ENSG00000174226	2.741212075	0.387220594	2.825346279	0.154892478	0.253177821	SNX31
ENSG00000260034	5.408102518	12.74669894	-1.230339466	0.154910816	0.253191867	LCMT1-AS2
ENSG00000256806	33.52859432	53.6039297	-0.673415978	0.15500823	0.253335147	C17orf100
ENSG00000103126	803.7298841	639.7432229	0.329238191	0.155036087	0.253364736	AXIN1
ENSG00000111788	87.08109073	123.1140715	-0.497467032	0.155097399	0.253448992	AC009533.1
ENSG00000249767	4.162071144	0.743759607	2.513787352	0.155196958	0.253595734	ENPP7P10
ENSG00000198691	28.6330788	7.970180625	1.838745707	0.155364002	0.253852722	ABCA4
ENSG00000262202	3.933051467	0.356539013	3.347656028	0.155376967	0.253857942	AC007952.4
ENSG00000153046	322.0538484	406.4414076	-0.335706872	0.155388162	0.253860268	CDYL
ENSG00000260018	25.06716288	14.49285183	0.784263185	0.155461419	0.253963979	AC040169.1
ENSG00000244490	21.31298479	35.73743567	-0.748448741	0.155502649	0.254015361	RWDD4P1
ENSG00000122376	399.2996034	514.1708193	-0.365433827	0.155528078	0.254040928	SHLD2
ENSG00000231967	3.902821355	0.336469917	3.337354458	0.155593565	0.254131918	AL135786.1
ENSG00000252448	4.207416313	0.774441189	2.498621902	0.155621792	0.254162042	RF00092
ENSG00000232608	4.513264919	1.060160428	2.080394128	0.15566864	0.254222574	TIMM9P2
ENSG00000283761	5.102253911	1.467450118	1.816534956	0.155678553	0.254222784	AC118553.2
ENSG00000101109	924.660374	749.5812894	0.302811671	0.155711096	0.254248802	STK4
ENSG00000251787	3.065936683	0.356539013	2.985998574	0.155723754	0.254248802	RNU7-47P
ENSG00000279203	11.99418302	4.809640044	1.334139977	0.155723844	0.254248802	AC005785.2
ENSG00000075651	321.8340587	523.8940624	-0.703003946	0.15574821	0.254272606	PLD1
novel.436	4.810266713	1.110911105	2.138296524	0.155759869	0.254275662	-
ENSG00000141371	3.052075276	0.387220594	2.980258701	0.155803763	0.254331338	C17orf64
ENSG00000137878	3.687734357	20.57241469	-2.480030473	0.15582332	0.254347281	GCOM1
ENSG00000271584	0.616711807	6.412997513	-3.390799594	0.155867718	0.254403768	LINC02550
ENSG00000139200	37.41142822	97.1947618	-1.376583986	0.155907882	0.254453337	PIANP
ENSG00000138303	452.8179666	560.1516507	-0.307358412	0.155926277	0.254467374	ASCC1
ENSG00000058600	57.40377527	31.72590193	0.852315914	0.155973218	0.254527993	POLR3E

ENSG00000161813	925.8943124	1229.684286	-0.409548508	0.156058133	0.254645047	LARP4
ENSG00000111305	1.788421549	6.268917821	-1.808471784	0.156068198	0.254645047	GSG1
ENSG00000213380	204.4721776	268.3814999	-0.393024734	0.156074351	0.254645047	COG8
ENSG00000243404	0.89358395	3.964379082	-2.136254185	0.156135436	0.254728714	RPL35AP32
ENSG00000188886	3.065936683	0.336469917	2.986005189	0.156164274	0.254759764	ASTL
ENSG00000275325	3.997343909	0.336469917	3.369419296	0.156218462	0.254831428	PDCD6IPP1
ENSG00000231704	2.741212075	0.336469917	2.825359168	0.15622782	0.254831428	AC004895.1
ENSG00000278627	5.11486167	1.00940975	2.300393919	0.156486086	0.255226614	AC005962.1
ENSG00000227857	2.727350667	0.356539013	2.819218916	0.156489741	0.255226614	AL358075.2
ENSG00000268536	0.307102255	2.567748738	-2.974396881	0.156502083	0.255230722	AC005523.1
ENSG00000255129	7.319951813	1.885352294	1.981508565	0.156564228	0.255316044	AP000880.1
ENSG00000259429	0.908699006	4.116631115	-2.167873055	0.156591941	0.25534521	UBE2Q2P2
ENSG00000251593	8.860477683	20.20783173	-1.188173356	0.156623931	0.255358048	MSNP1
ENSG00000006007	845.709848	1131.619803	-0.420179175	0.1566317	0.255358048	GDE1
ENSG00000196757	227.8475653	159.6640787	0.5132108	0.156634874	0.255358048	ZNF700
ENSG00000141510	629.4498148	955.3757716	-0.602300902	0.156639129	0.255358048	TP53
ENSG00000104856	413.7686266	587.8407062	-0.50605191	0.156744039	0.255513043	RELB
ENSG00000135108	640.3536056	788.7161611	-0.300288838	0.156763867	0.255529332	FBXO21
ENSG00000151365	3.044553384	0.356539013	2.977167087	0.156884827	0.255690899	THRSP
ENSG00000266304	0.293240848	2.567748738	-2.974410742	0.15689251	0.255690899	LIVAR
ENSG00000245008	0.293240848	2.567748738	-2.974410742	0.15689251	0.255690899	AP001122.1
ENSG00000106355	472.1048768	630.1583616	-0.417099479	0.156953141	0.255773665	LSM5
ENSG00000185483	151.5471777	51.45387321	1.556814564	0.157014368	0.255846128	ROR1
ENSG00000047644	1077.641473	1326.607957	-0.300020781	0.157018122	0.255846128	WWC3
ENSG00000108953	7.577947953	14.96022042	-0.976287427	0.157027149	0.255846128	YWHAE
ENSG00000223576	0.601596751	3.32212083	-2.451669703	0.157118922	0.255979602	AL355001.1
ENSG00000262585	2.72484337	0.336469917	2.818116695	0.157171447	0.256049119	LINC01979
ENSG00000259031	3.60707321	0.693008929	2.351774002	0.157187388	0.256059034	AL845552.2
ENSG00000271002	0.308355904	3.424906455	-3.401972668	0.157270318	0.256178065	AC129492.6
ENSG00000178776	0.307102255	3.424906455	-3.401971437	0.157301784	0.256213257	C5orf46
ENSG00000011677	11.06194942	1.345879667	3.017879819	0.157312639	0.256214874	GABRA3
ENSG00000240048	2.123246619	6.65498254	-1.656022944	0.157346624	0.256254162	DDX50P2
ENSG00000174844	12.0697583	5.381078522	1.166149012	0.15736856	0.256270794	DNAH12
ENSG00000286089	3.887706299	0.356539013	3.332170504	0.157386428	0.256270794	AC095057.4
ENSG00000287011	3.887706299	0.356539013	3.332170504	0.157386428	0.256270794	AC003077.1
ENSG00000272366	3.825992426	0	4.271245158	0.157449155	0.256356865	AL158211.3
ENSG00000230202	1456.62124	1835.078588	-0.333257359	0.157560506	0.25652209	AL450405.1
ENSG00000089472	624.8040619	289.2123508	1.110750271	0.157613448	0.256592205	HEPH
ENSG00000267934	6.859191699	2.10970837	1.691958532	0.15762995	0.25660299	AC010300.1
ENSG00000213189	39.22653275	59.47886725	-0.603141373	0.157719273	0.256732312	BTF3L4P2
ENSG00000100354	795.5586316	590.1372475	0.431195304	0.157759351	0.256781463	TNRC6B
ENSG00000262265	3.296210009	8.580473026	-1.376157098	0.157770004	0.256782714	AC002558.3
novel.1059	15.15457099	4.83099341	1.636892718	0.157885313	0.256954291	-
ENSG00000153237	29.5455391	8.788356026	1.739601759	0.157933353	0.257016376	CCDC148
ENSG00000188869	0.615458159	3.189937893	-2.391053826	0.157949706	0.257026889	TMC3
ENSG00000260495	12.96341352	5.676254372	1.186612986	0.158001337	0.257094804	AC009148.1
ENSG00000279845	4.816606227	0.693008929	2.774701464	0.158046499	0.257148692	AC097372.2
ENSG00000142039	598.0355928	438.9141641	0.445575318	0.15805425	0.257148692	CCDC97
ENSG00000174669	67.76355096	95.17231192	-0.488990585	0.158104528	0.257214385	SLC29A2
ENSG00000103769	2082.077528	2663.130303	-0.355154654	0.158127384	0.257229699	RAB11A
ENSG00000231607	93.55043901	64.61449312	0.531865622	0.158137118	0.257229699	DLEU2

ENSG00000248858	14.42578554	5.93000776	1.296308923	0.158143643	0.257229699	AC104211.1
ENSG00000179611	129.573959	176.2808002	-0.444649226	0.15819813	0.257302218	DGKZP1
ENSG00000170959	7.206624528	1.426156051	2.333910905	0.158253126	0.257375554	DCDC1
ENSG00000145358	30.0134651	18.20103738	0.718793408	0.158343408	0.25749286	DDIT4L
ENSG00000189376	58.75582585	39.13743992	0.583548326	0.15835145	0.25749286	C8orf76
ENSG00000136319	267.1954736	343.3523542	-0.362597332	0.158354987	0.25749286	TTC5
ENSG00000132952	588.4192907	482.4697255	0.286757744	0.158422172	0.257585984	USPL1
ENSG00000270533	5.201791061	1.436768536	1.853744719	0.158531327	0.257747335	CR382285.1
ENSG00000275066	70.84557161	43.07211772	0.721441283	0.158596741	0.257829059	SYNRG
ENSG00000231426	3.385646697	0	4.091951097	0.158634191	0.257829059	FILNC1
ENSG00000176134	3.385646697	0	4.091951097	0.158634191	0.257829059	AL445665.1
novel.455	3.385646697	0	4.091951097	0.158634191	0.257829059	-
ENSG00000203711	3.385646697	0	4.091951097	0.158634191	0.257829059	C6orf99
ENSG00000229994	14.21822044	23.86770675	-0.744283056	0.158641135	0.257829059	RPL5P4
ENSG00000110888	1151.571551	798.7445884	0.527708962	0.158661592	0.257846177	CAPRIN2
ENSG00000272170	0.586481695	3.678659843	-2.620807514	0.158694008	0.257871837	AL355385.1
ENSG00000260433	3.384393048	0	4.091444513	0.158707158	0.257871837	LINC01917
novel.973	3.384393048	0	4.091444513	0.158707158	0.257871837	-
ENSG00000156928	600.4493317	748.4939282	-0.317901172	0.158748455	0.257922807	MALSU1
ENSG00000242375	12.76204539	5.451898296	1.232185154	0.15879073	0.2579664	AL590705.3
ENSG00000226393	8.087600719	1.682349583	2.237800363	0.158795144	0.2579664	IFNA20P
ENSG00000210140	3319.00623	2414.064674	0.459351506	0.158913246	0.258142118	MT-TC
ENSG00000272047	985.6508088	760.9719545	0.372812839	0.158978448	0.258231889	GTF2H5
ENSG00000153048	895.6107127	1170.785733	-0.386696725	0.159006237	0.258260881	CARHSP1
ENSG00000036257	973.6641035	1181.361078	-0.279134072	0.159282756	0.258693835	CUL3
ENSG00000279594	7.014174478	1.813376645	1.955285846	0.159366291	0.258813327	AL049780.3
ENSG00000224939	2.431602522	7.04335901	-1.541915061	0.159416104	0.258856813	LINC00184
ENSG00000238181	3.549120283	0	4.162757255	0.159422958	0.258856813	AHCYP2
ENSG00000274759	3.549120283	0	4.162757255	0.159422958	0.258856813	RNA5SP527
ENSG00000137364	548.4851698	434.5604626	0.335218472	0.159435036	0.258860247	TPMT
ENSG00000176289	4.148209737	0.693008929	2.559331201	0.159456237	0.258875271	IDSP1
ENSG00000253485	16.34906022	8.611154608	0.924729734	0.159464217	0.258875271	PCDHGA5
ENSG00000279278	54.34216935	34.42467773	0.654966549	0.159482032	0.258888015	AC245060.6
ENSG00000068308	1544.701024	1915.796004	-0.310475529	0.159511542	0.258919741	OTUD5
ENSG00000258881	8.812625217	2.0188195	2.098439528	0.159563726	0.258988265	AC007040.2
ENSG00000253661	13.05911845	36.14271758	-1.471446936	0.159586691	0.259009359	ZFHX4-AS1
ENSG00000259985	17.39379473	8.020802909	1.121765541	0.159612549	0.25902434	AC017100.1
ENSG00000258424	20.94639123	10.10813043	1.054998608	0.159615861	0.25902434	AL512791.1
ENSG00000109065	655.1141731	811.4212861	-0.308691842	0.159639848	0.259041648	NAT9
ENSG00000105072	147.6234716	111.5272361	0.4033577	0.159646468	0.259041648	C19orf44
ENSG00000236039	54.11319312	22.8440885	1.24796082	0.159699086	0.259110844	AC019117.1
ENSG00000043355	384.8802805	63.72849885	2.593878381	0.159722773	0.259114892	ZIC2
ENSG00000248873	2.755073482	8.387954407	-1.607450264	0.159729287	0.259114892	SERBP1P6
ENSG00000166866	5.157699541	1.069617038	2.263934788	0.1597315	0.259114892	MYO1A
ENSG00000255303	3.928036872	0.713078025	2.45588445	0.159782593	0.259181591	OR5BA1P
ENSG00000232259	5.478663203	1.406086955	1.946578739	0.15984759	0.259270835	AL158166.2
ENSG00000154237	790.4884008	590.5065355	0.421237088	0.159936691	0.259399162	LRRK1
ENSG00000285736	2.976499995	0.387220594	2.948750302	0.159967354	0.259432699	AC131025.3
ENSG00000151917	143.1838875	106.0434192	0.4317487	0.160073347	0.259588392	BEND6
ENSG00000271664	12.04336041	5.166179057	1.235743836	0.160160072	0.259712821	AC004890.3
ENSG00000232926	2.666890443	8.621767093	-1.683453909	0.160239014	0.259824616	AC000078.1

ENSG00000228816	2.124500267	6.185045417	-1.538673192	0.160251685	0.259828945	AK3P5
ENSG00000241131	3.386900346	0	4.092469241	0.160320248	0.259923892	LINC02032
ENSG00000226526	0	2.149846562	-3.685908329	0.160334007	1	AL049569.1
ENSG00000242325	0	2.149846562	-3.685908329	0.160334007	1	RPS12P31
ENSG00000173548	941.2177106	1373.551379	-0.545131303	0.160394771	0.260028488	SNX33
ENSG00000258844	3.3831394	0	4.090948672	0.160540291	0.260199457	AL162511.1
ENSG00000283945	3.3831394	0	4.090948672	0.160540291	0.260199457	LINC00032
ENSG00000235832	3.3831394	0	4.090948672	0.160540291	0.260199457	CNN2P3
ENSG00000211789	3.3831394	0	4.090948672	0.160540291	0.260199457	TRAV12-2
ENSG00000223685	0.921306765	4.972632957	-2.442060084	0.16060687	0.260291129	LINC00571
ENSG00000255538	2.975246347	0.356539013	2.948211724	0.160742834	0.260484101	OR10V2P
ENSG00000235376	1.232169966	5.176791542	-2.073828573	0.160745991	0.260484101	RPEL1
ENSG00000138162	321.6524377	415.7145513	-0.37050413	0.160792323	0.26054293	TACC2
ENSG00000157500	1057.009378	833.8782349	0.341883988	0.160843064	0.260608895	APPL1
ENSG00000121900	163.5319285	286.3678946	-0.808846412	0.160894611	0.26067616	TMEM54
ENSG00000085662	4241.837529	7552.53069	-0.832256321	0.160928272	0.260714438	AKR1B1
ENSG00000134644	1315.640039	1574.912417	-0.259433119	0.161010569	0.260816867	PUM1
ENSG00000199476	5.132484024	1.00940975	2.305608448	0.161018259	0.260816867	RF00019
ENSG00000260698	12.51414971	4.993857928	1.32123092	0.161021614	0.260816867	AL591848.3
ENSG00000172809	4820.12458	6046.316435	-0.327000853	0.161213643	0.26110695	RPL38
ENSG00000255561	40.83357362	25.16412001	0.694490696	0.161220804	0.26110695	FDXACB1
ENSG00000197614	145.2471276	829.3420137	-2.513561742	0.161308104	0.261232054	MFAP5
ENSG00000230673	6.873053107	2.211209725	1.65498079	0.16132992	0.2612511	PABPC1P3
ENSG00000106993	378.0497287	303.8753623	0.314843477	0.161386086	0.261325765	CDC37L1
ENSG00000253958	3.60707321	0.723690511	2.326628614	0.161442519	0.261400852	CLDN23
ENSG00000125520	1302.499234	1713.091999	-0.395436571	0.161472104	0.261432463	SLC2A4RG
ENSG00000173626	3.962027931	0.693008929	2.486839026	0.161570368	0.261575257	TRAPPC3L
ENSG00000262692	27.47863575	17.01802224	0.696181989	0.161582453	0.261578522	AC116914.2
ENSG00000112699	252.7543147	432.104023	-0.774064452	0.161607438	0.261588952	GMDS
ENSG00000224855	4.133094681	0.356539013	3.421489319	0.161609033	0.261588952	OPA1-AS1
ENSG00000084734	1.84261353	9.037357519	-2.298238216	0.161621276	0.261592473	GCKR
ENSG00000114735	522.1112303	644.9717454	-0.304852761	0.16163911	0.261605041	HEMK1
novel.596	74.21717092	140.7955769	-0.924178276	0.161691558	0.261673625	-
ENSG00000184575	2422.715923	3016.584983	-0.316258338	0.161766252	0.261771851	XPOT
ENSG00000179010	6838.135572	8378.577592	-0.293134309	0.161772404	0.261771851	MRFAP1
novel.804	70.24802054	47.59634249	0.558817907	0.1617918	0.261783977	-
ENSG00000266651	7.508640916	16.61086094	-1.146135597	0.16180005	0.261783977	AC093484.4
ENSG00000069667	271.7474863	468.9960184	-0.787155126	0.161832443	0.261820082	RORA
ENSG00000279530	5.058162392	1.110911105	2.210760312	0.161856326	0.261842416	AC092881.2
ENSG00000275719	7.892572101	2.598430319	1.620588061	0.161908143	0.261906204	AC008622.2
ENSG00000125352	267.291107	352.7811238	-0.399982295	0.161915917	0.261906204	RNF113A
ENSG00000181004	159.033538	121.2125029	0.391831797	0.162033674	0.262080363	BBS12
ENSG00000075234	475.7427656	596.8125691	-0.326932131	0.16218428	0.262307631	TTC38
ENSG00000089775	359.7115355	461.3678087	-0.358579257	0.162247032	0.262392788	ZBTB25
ENSG00000275413	10.80359728	4.596024848	1.226933774	0.162389543	0.262606916	AC002553.2
ENSG00000204011	1.493927053	5.64557279	-1.914969761	0.162413789	0.262627529	COL5A1-AS1
ENSG00000124097	3.88645265	0.723690511	2.434580627	0.162431335	0.262627529	HMGB1P1
ENSG00000267365	5.146345431	14.0773898	-1.458489615	0.162432615	0.262627529	KCNJ2-AS1
ENSG00000132359	420.0663258	228.9681228	0.876280412	0.162470289	0.262672097	RAP1GAP2
ENSG00000228886	1.214547612	5.023383634	-2.050216393	0.162550634	0.262785641	AL138963.1
ENSG00000227964	0.586481695	3.873490212	-2.705896865	0.162604787	0.262849147	LINC01429

ENSG00000185808	410.1445993	293.5190479	0.481760737	0.162610151	0.262849147	PIGP
ENSG00000111846	2.450478525	0	3.625269913	0.162618699	1	GCNT2
ENSG00000198049	3.31633966	0	4.063619493	0.162750788	0.263050907	AVPR1B
ENSG00000260781	2.744973021	9.733962468	-1.833394068	0.162755218	0.263050907	ARHGAP23P1
ENSG00000213694	1150.410348	1775.147522	-0.625908247	0.162844202	0.263178354	S1PR3
ENSG00000166821	155.9347214	114.1512109	0.451884676	0.162865221	0.263195951	PEX11A
novel.1054	11.5651914	4.158053576	1.45961195	0.162906093	0.263245628	-
ENSG00000260657	0.61420451	5.24773971	-3.104816643	0.163005303	0.263374336	AC107871.2
novel.1063	61.90031583	114.1831428	-0.882532722	0.163006017	0.263374336	-
ENSG00000249306	4.924918918	0.743759607	2.746777491	0.163077378	0.263456869	LINC01411
ENSG00000233684	4.924918918	0.743759607	2.746777491	0.163077378	0.263456869	LINC01865
ENSG00000183145	2.444210281	0	3.621918783	0.163108611	1	RIPPLY3
ENSG00000260840	2.444210281	0	3.621918783	0.163108611	1	LINC01964
ENSG00000274115	2.444210281	0	3.621918783	0.163108611	1	MIR6081
ENSG00000272501	7.708897593	0.693008929	3.459025952	0.163158447	0.263571449	AL662844.4
ENSG00000256280	2.742465724	0.356539013	2.825979783	0.163271377	0.26373748	AP006289.1
ENSG00000286550	10.74313705	4.045811341	1.423052031	0.163320984	0.263801211	AL591002.1
ENSG00000261588	0.293240848	2.537067156	-2.96029953	0.163336167	0.263809335	AC093249.4
ENSG00000173542	960.7958109	751.3915839	0.354720524	0.163447258	0.263972352	MOB1B
ENSG00000265907	2.738704777	0.356539013	2.824314123	0.163497516	0.26403557	AP000919.2
ENSG00000253910	24.01511994	13.89073177	0.78532126	0.163506728	0.26403557	PCDHGB2
ENSG00000224163	2.464339932	9.180152941	-1.900435261	0.16354501	0.264080977	AC025594.1
ENSG00000242485	1242.372851	1607.860124	-0.3722431	0.163578096	0.264105963	MRPL20
ENSG00000273374	5.434571684	1.396630344	1.943360935	0.163580815	0.264105963	AC069222.1
ENSG00000196704	1113.742836	1352.705065	-0.280284211	0.163639826	0.26418482	AMZ2
ENSG00000271380	13.36622071	4.891200698	1.439216022	0.163652717	0.264189216	AL451085.2
ENSG00000255399	0.601596751	4.117915384	-2.778790817	0.163677913	0.264213473	TBX5-AS1
ENSG00000250130	0.586481695	3.566546002	-2.58328458	0.163707223	0.264244369	AC090519.1
ENSG00000134853	723.3109231	1919.746683	-1.408256294	0.16371865	0.264246397	PDGFRA
ENSG00000143578	128.1450799	165.3214199	-0.367700398	0.163747396	0.264276376	CREB3L4
ENSG00000260988	4.41372777	0.336469917	3.516212859	0.163841401	0.264411667	AC090260.1
ENSG00000102738	325.7779824	407.1498281	-0.321425196	0.16392492	0.264530021	MRPS31
ENSG00000126945	1428.526978	1710.425956	-0.259861328	0.163960442	0.264570912	HNRNPH2
ENSG00000213777	5.421963925	1.456837632	1.906074885	0.163973909	0.26457621	AC011487.1
novel.557	1.835020366	6.959486606	-1.924115899	0.164072339	0.264718589	-
ENSG00000178425	377.3965029	573.2017322	-0.60311128	0.164104106	0.264738008	NT5DC1
ENSG00000235904	41.95506579	21.49735692	0.957224687	0.164137142	0.264738008	RBMS3-AS3
ENSG00000246084	3.388153995	0	4.093001696	0.164140087	0.264738008	LINC02325
ENSG00000224177	3.388153995	0	4.093001696	0.164140087	0.264738008	LINC00570
novel.61	3.322679175	0	4.066238665	0.164145513	0.264738008	-
ENSG00000267084	3.322679175	0	4.066238665	0.164145513	0.264738008	AC015802.2
ENSG00000107938	289.0614194	356.0007155	-0.300751514	0.164227527	0.264853841	EDRF1
ENSG00000087903	122.7616947	83.16492466	0.560882756	0.164245704	0.264866714	RFX2
ENSG00000103742	70.74438067	203.7329262	-1.525984412	0.164269206	0.264888173	IGDCC4
novel.793	8.607282675	27.26971878	-1.65770788	0.164283324	0.264894498	-
ENSG00000189326	0	3.005720009	-4.16469794	0.164324018	0.26492723	SPANXN4
ENSG00000253477	0	3.005720009	-4.16469794	0.164324018	0.26492723	AC012213.1
ENSG00000166664	3.381885751	0	4.090464471	0.164510922	0.265208994	CHRFAM7A
ENSG00000174963	157.7802246	63.7718007	1.305789737	0.164519201	0.265208994	ZIC4
ENSG00000286753	12.48266595	4.872287477	1.343176571	0.164605295	0.265322822	AC091978.1
ENSG00000132313	613.7685816	778.7152086	-0.343789839	0.164610237	0.265322822	MRPL35

ENSG00000287562	61.32644189	34.32291959	0.839476733	0.164627872	0.265334785	AL109615.4
ENSG00000162994	221.9376727	151.9753793	0.547297625	0.164661037	0.265371776	CLHC1
ENSG00000258412	0	2.975038428	-4.150816542	0.164732334	0.265470213	AL355102.1
ENSG00000228828	5.497539206	1.487519214	1.906580072	0.1647836	0.265536358	TLK2P2
ENSG00000223313	6.640272484	1.548882377	2.13947214	0.164847696	0.265623169	RNU6-516P
ENSG00000228253	690.738221	1047.030129	-0.600106108	0.164910536	0.265707947	MT-ATP8
ENSG00000086061	2680.2844	3614.662799	-0.431555292	0.164928587	0.265720552	DNAJA1
ENSG00000254352	16.58399213	5.522718069	1.595309657	0.165021566	0.265853866	AC100854.1
ENSG00000279977	0.616711807	3.832196145	-2.653601533	0.165073282	0.265920693	AC008764.10
ENSG00000273419	6.581065908	2.180528143	1.604291741	0.165108523	0.265960973	AC004877.1
ENSG00000182223	2.697120555	0.387220594	2.805685208	0.165212519	0.266111993	ZAR1
ENSG00000249159	4.204909015	0.774441189	2.49584611	0.165268699	0.266185982	AC091965.1
ENSG00000226604	0.921306765	4.697526203	-2.357060728	0.165367061	0.266327895	PAPPA-AS2
ENSG00000254389	6.667995298	2.466247382	1.421540328	0.16555979	0.266621763	RHPN1-AS1
ENSG00000183054	10.5481797	4.208804254	1.314562858	0.165579522	0.266637013	RGPD6
ENSG00000233806	0.615458159	3.790773684	-2.622151847	0.165624037	0.266692166	LINC01237
ENSG00000135537	68.909005	97.49260023	-0.503226244	0.165688136	0.266778846	AFG1L
ENSG00000066933	808.7408073	606.9110542	0.414439736	0.165752716	0.26686629	MYO9A
ENSG00000166169	416.8888262	509.4432409	-0.288812813	0.165784162	0.266893769	POLL
ENSG00000170899	575.8638415	432.9390095	0.410863773	0.165790329	0.266893769	GSTA4
ENSG00000116748	3.699017196	0	4.219778262	0.165890689	0.267038785	AMPD1
ENSG00000141933	320.5382967	252.5829065	0.343324684	0.165927497	0.267081488	TPGS1
ENSG00000270462	4.763667895	1.029478846	2.182907204	0.165941569	0.267087591	AC005034.2
ENSG00000165807	18.12229544	9.457571445	0.945052933	0.165999803	0.267114624	PPP1R36
ENSG00000154889	393.9643982	276.6618352	0.509023958	0.166001631	0.267114624	MPPE1
ENSG00000286524	5.682467363	0.672939833	3.04377996	0.166028897	0.267114624	AC092567.2
ENSG00000237781	20.03393127	8.844967294	1.176369058	0.166031287	0.267114624	AL356356.1
ENSG00000184185	17.53853451	58.7000125	-1.742755988	0.166034886	0.267114624	KCNJ12
ENSG00000233090	3.970874743	0.672939833	2.511201948	0.166039299	0.267114624	AC015922.1
ENSG00000186166	2.697120555	0.356539013	2.805672608	0.166040614	0.267114624	CCDC84
ENSG00000235613	2.697120555	0.356539013	2.805672608	0.166040614	0.267114624	NSRP1P1
ENSG00000178691	669.7696907	824.8724449	-0.300510871	0.16626982	0.267466795	SUZ12
ENSG00000105755	250.5749115	339.6775819	-0.438433065	0.166306722	0.267498043	ETHE1
ENSG00000155008	309.2118264	384.5763041	-0.314647693	0.166313206	0.267498043	APOOL
ENSG00000106633	40.15410776	60.13284669	-0.583169923	0.166320133	0.267498043	GCK
ENSG00000250899	118.4204933	69.62880533	0.763519305	0.166348023	0.267526338	AC125807.2
ENSG00000237575	8.489154257	2.894762044	1.556672144	0.166401933	0.267584108	PYY2
ENSG00000269934	16.15027066	27.78976428	-0.787090221	0.166404543	0.267584108	AL353593.1
ENSG00000274721	2.139615324	6.653698271	-1.634046561	0.166518755	0.267751192	RF02039
ENSG00000225127	3.686480708	0	4.215089094	0.166555689	0.267777435	LINC00237
ENSG00000275465	3.686480708	0	4.215089094	0.166555689	0.267777435	AL449403.2
ENSG00000175093	8.630204358	1.00940975	3.070382874	0.166576343	0.26779407	SPSB4
ENSG00000185900	33.58153266	49.64823654	-0.565240365	0.166621519	0.267825696	POMK
novel.923	3.685155788	0	4.214581683	0.166626941	0.267825696	-
ENSG00000228423	3.685155788	0	4.214581683	0.166626941	0.267825696	AL357552.2
ENSG00000169020	607.5318996	423.5853333	0.520655945	0.166647884	0.267842788	ATP5ME
ENSG00000287826	3.281094953	0	4.049007545	0.166697642	0.267906187	AC090994.1
ENSG00000187678	1640.23023	2723.720128	-0.731736791	0.166753669	0.267979655	SPRY4
ENSG00000240045	2.125753916	0	3.421194371	0.166763828	1	STRIT1
ENSG00000205669	2.125753916	0	3.421194371	0.166763828	1	ACOT6
ENSG00000223675	2.125753916	0	3.421194371	0.166763828	1	AC093117.1

ENSG00000225715	2.125753916	0	3.421194371	0.166763828	1	AL512504.1
ENSG00000252367	2.125753916	0	3.421194371	0.166763828	1	RF00019
ENSG00000286112	8.854209439	3.546476906	1.310605354	0.166776316	0.267999473	AL441992.2
ENSG00000104320	1054.326749	841.7051534	0.324694698	0.166828149	0.268066185	NBN
ENSG00000129451	2.124500267	0	3.420446079	0.166879726	1	KLK10
ENSG00000167759	2.124500267	0	3.420446079	0.166879726	1	KLK13
ENSG00000206869	2.124500267	0	3.420446079	0.166879726	1	SNORA70F
ENSG00000286932	2.124500267	0	3.420446079	0.166879726	1	AC013716.1
ENSG00000256937	2.124500267	0	3.420446079	0.166879726	1	KRT17P8
ENSG00000214546	2.124500267	0	3.420446079	0.166879726	1	AC087491.1
ENSG00000181109	2.124500267	0	3.420446079	0.166879726	1	OR52P1P
ENSG00000286625	2.124500267	0	3.420446079	0.166879726	1	AC113370.1
ENSG00000287087	2.124500267	0	3.420446079	0.166879726	1	AC025175.2
ENSG00000162409	153.4248228	96.12344063	0.672942104	0.166886332	0.268129743	PRKAA2
ENSG00000232043	4.836735879	10.52603261	-1.117293776	0.166888344	0.268129743	AL133230.1
ENSG00000271424	3.899060409	0.672939833	2.487859173	0.166920094	0.268147589	AL157791.2
ENSG00000237972	3.899060409	0.672939833	2.487859173	0.166920094	0.268147589	TUBG1P
ENSG00000161149	10.28391532	3.567701877	1.525326664	0.166932302	0.26815062	TUBA3FP
ENSG00000155393	441.3938253	565.8793924	-0.358750331	0.166964246	0.268169007	HEATR3
ENSG00000198837	1412.619208	1086.033034	0.379573099	0.166964392	0.268169007	DENND4B
ENSG00000273983	0.308355904	2.476859868	-2.931357034	0.167025551	0.268250655	HIST1H3G
ENSG00000233461	26.6858426	51.43846197	-0.943520232	0.167075845	0.268314842	AL445524.1
ENSG00000162521	1760.802402	2076.58518	-0.237994119	0.167092441	0.26832491	RBBP4
ENSG00000248477	3.85371524	0.387220594	3.320360635	0.167184762	0.268444747	AC139495.1
ENSG00000228315	142.0713566	86.61532844	0.71566047	0.167189774	0.268444747	GUSBP11
ENSG00000272597	6.095375011	1.783850939	1.768125101	0.167198064	0.268444747	AC063944.3
ENSG00000279392	6.009699269	12.19892558	-1.017602046	0.167223573	0.268469112	AL158801.5
ENSG00000249304	0.293240848	3.404837359	-3.393932016	0.167242333	0.26848264	AC104779.1
ENSG00000160999	52.76758115	87.48374094	-0.72700532	0.167287879	0.268539165	SH2B2
ENSG00000265912	5.375365108	1.080229524	2.31903459	0.167313588	0.268563841	AC037487.1
ENSG00000148386	3.671365652	0	4.209405242	0.167363665	0.268627626	LCN9
ENSG00000251720	4.469173399	0.713078025	2.64470672	0.16746362	0.268764201	RNU7-123P
ENSG00000109625	10.46780329	2.079026788	2.318779461	0.167469445	0.268764201	CPZ
ENSG00000145919	819.3852023	1053.779684	-0.362783825	0.16750443	0.268803742	BOD1
ENSG00000221926	156.1511608	251.7120886	-0.688394376	0.167561286	0.268878375	TRIM16
ENSG00000250471	2.15473038	6.857985251	-1.677315315	0.167605715	0.268933058	GMPSP1
ENSG00000248905	10.57339522	23.68947785	-1.161352488	0.167695739	0.269051181	FMN1
ENSG00000233058	43.84671417	89.12590515	-1.022293406	0.167700043	0.269051181	LINC00884
ENSG00000275454	17.83414046	10.01839744	0.830981249	0.167729821	0.26907755	AC105020.6
ENSG00000169045	909.3859672	708.8002042	0.359276805	0.167749854	0.26907755	HNRNPH1
ENSG00000224790	17.73084236	8.46005845	1.061972518	0.167753816	0.26907755	AP000704.1
ENSG00000287032	4.797658954	0.743759607	2.712293674	0.167757906	0.26907755	AC018647.3
ENSG00000221819	2.962638588	9.416277378	-1.658322616	0.167886446	0.269267101	GAS8-AS1
ENSG00000092345	0	3.036401591	-4.178584139	0.167991	0.269401529	DAZL
ENSG00000199038	0	3.036401591	-4.178584139	0.167991	0.269401529	MIR210
ENSG00000283752	3.857476187	0.336469917	3.321647136	0.168084299	0.269534512	AC241585.3
ENSG00000074276	2.128261214	8.406996022	-1.987047366	0.168095221	0.269535391	CDHR2
ENSG00000287264	9.117220175	3.944309986	1.207373366	0.168117602	0.269554642	AP003778.1
ENSG00000286373	9.969219902	4.382281258	1.196699001	0.168136246	0.269567899	AC022387.2
ENSG00000184414	2.11063886	0	3.412101969	0.168172891	1	IRS3P
ENSG00000224016	2.11063886	0	3.412101969	0.168172891	1	AC092681.1

ENSG00000186439	2.11063886	0	3.412101969	0.168172891	1	TRDN
ENSG00000254226	2.11063886	0	3.412101969	0.168172891	1	LINC01933
ENSG00000238194	2.11063886	0	3.412101969	0.168172891	1	AL121908.1
ENSG00000235026	2.11063886	0	3.412101969	0.168172891	1	DPP10-AS1
ENSG00000076321	524.6595935	393.0495904	0.415858857	0.168278285	0.269778978	KLHL20
ENSG00000216809	9.333632093	3.944309986	1.243941395	0.168378476	0.269922946	AL589993.1
ENSG00000207574	0	3.188782018	-4.255399052	0.168410883	0.26995824	MIR661
ENSG00000147889	1973.512819	1077.411919	0.873331377	0.168506714	0.270089757	CDKN2A
ENSG00000259343	5.190436951	13.23542089	-1.349055132	0.16851372	0.270089757	TMC3-AS1
novel.1032	22.00903283	39.54930593	-0.845955371	0.168569753	0.270162899	-
ENSG00000110931	1040.097289	1253.987863	-0.269847284	0.168687693	0.270335243	CAMKK2
ENSG00000280780	2.374903244	0	3.58423893	0.168692724	1	JAKMIP2-AS1
ENSG00000287631	2.374903244	0	3.58423893	0.168692724	1	AC005071.1
ENSG00000177076	29.33253212	17.34272379	0.760775661	0.168725337	0.270378892	ACER2
ENSG00000164031	971.9785694	772.7959155	0.330716129	0.168738575	0.270383428	DNAJB14
ENSG00000054148	2457.623129	1908.649371	0.364762442	0.168774046	0.270423589	PHPT1
ENSG00000138463	212.0511658	272.8031014	-0.364099247	0.16879134	0.270434621	SLC49A4
ENSG00000271817	5.406848869	1.406086955	1.930660067	0.168815382	0.270456462	RF00012
ENSG00000224888	18.14751096	9.353758339	0.957085839	0.168841054	0.270480911	AC138028.2
ENSG00000171016	210.2342663	258.1348464	-0.295919555	0.168864819	0.270502303	PYGO1
ENSG00000026036	54.17143078	89.12556682	-0.716823166	0.16896331	0.270643388	RTEL1-TNFRSF6B
ENSG00000270116	2.094270155	6.777708867	-1.689701797	0.168999429	0.270684555	AP001429.1
ENSG00000271579	0.61420451	5.298361994	-3.110597696	0.1690328	0.270708395	AC078880.3
ENSG00000151576	344.7673213	432.8261593	-0.3285073	0.169035152	0.270708395	QTRT2
ENSG00000151332	417.8138794	283.6728432	0.558038684	0.169106261	0.270805583	MBIP
ENSG00000232202	4.006119451	0.387220594	3.372493454	0.169117775	0.270807329	AC098824.1
ENSG00000268499	1.200686205	5.216929734	-2.106750769	0.169141684	0.270828923	AC011479.2
ENSG00000287200	11.66472855	4.320918095	1.431935691	0.169228005	0.27095044	AC022506.2
ENSG00000287707	1.846374476	6.309056013	-1.786605029	0.169263904	0.270991218	AC115284.4
ENSG00000255503	7.151178898	2.211209725	1.710931061	0.16934549	0.271105131	AP001767.3
novel.243	5.802134163	12.1481749	-1.07154546	0.169359152	0.271106395	-
ENSG00000258083	5.864816951	0	4.886642466	0.169367149	0.271106395	OR9A4
ENSG00000279232	5.088392504	1.436768536	1.827621369	0.169703711	0.271616101	AC008522.1
ENSG00000170631	169.684446	127.7883177	0.409942712	0.169712373	0.271616101	ZNF16
ENSG00000106610	201.2539793	251.0164764	-0.319536387	0.169716939	0.271616101	STAG3L4
ENSG00000151702	665.1205433	447.5713519	0.57125841	0.169748206	0.271649409	FLI1
ENSG00000109680	147.2557666	201.4785556	-0.453586471	0.169910798	0.271885056	TBC1D19
ENSG00000273925	2.755073482	0.387220594	2.831718339	0.169916387	0.271885056	AC084757.4
ENSG00000276523	11.97781432	5.023383634	1.255409796	0.169953013	0.271897913	AC025287.3
ENSG00000185156	4.851850935	1.00940975	2.222622332	0.169954401	0.271897913	MFSD6L
ENSG00000165392	320.7136943	414.1980199	-0.369482437	0.169955818	0.271897913	WRN
novel.510	4.006119451	0.336469917	3.372502999	0.17011018	0.272111902	-
ENSG00000224858	59.04705795	95.64353332	-0.696583467	0.170110524	0.272111902	RPL29P11
ENSG00000100926	3.369277992	8.75395003	-1.378578914	0.17021818	0.272267349	TM9SF1
novel.1024	5.85876217	0	4.883791691	0.17034878	0.272459472	-
ENSG00000173077	5.195522817	1.10029862	2.250493006	0.170416611	0.272551182	1-Dec
ENSG00000163755	556.3313287	823.4605536	-0.565410243	0.17043283	0.272560345	HPS3
ENSG00000134058	30.30036643	52.58202808	-0.797451023	0.170491442	0.272637296	CDK7
ENSG00000214803	3.692748952	0.356539013	3.254857312	0.170503896	0.272640432	AC090921.1
ENSG00000150779	621.3869589	901.5695971	-0.537314119	0.170664633	0.272880659	TIMM8B
ENSG00000186162	201.114325	255.2055499	-0.342921983	0.170686091	0.272898173	CIDECP1

ENSG00000258010	0	2.996263399	-4.160399515	0.17073205	0.272923647	AC016705.1
ENSG00000156239	346.561968	256.0108951	0.435749013	0.170732381	0.272923647	N6AMT1
ENSG00000146830	2223.346858	1606.510139	0.46895636	0.170733537	0.272923647	GIGYF1
ENSG00000152977	1320.091742	325.9140493	2.017940308	0.170758546	0.272946831	ZIC1
ENSG00000164627	22.65722839	10.83297682	1.064911597	0.17078075	0.272965529	KIF6
ENSG00000162869	466.9231441	373.5501195	0.322465557	0.17085603	0.273059585	PPP1R21
ENSG00000260996	4.562371034	1.029478846	2.115245574	0.170860616	0.273059585	BX255925.1
ENSG00000175906	289.6227409	478.7959338	-0.725005721	0.170910257	0.273122117	ARL4D
ENSG00000204666	6.084020901	1.844058227	1.737439898	0.170933673	0.273142736	AC010624.1
ENSG00000272655	51.46477923	69.4724437	-0.43353518	0.170955818	0.273161322	POLR2J4
ENSG00000107745	1115.662191	884.5431204	0.334646608	0.171057054	0.273306274	MICU1
ENSG00000080603	69.6335313	100.3137984	-0.525146668	0.171151424	0.273440238	SRCAP
ENSG00000277304	0.908699006	3.61729668	-1.990728199	0.17117569	0.273462191	AC142086.6
ENSG00000163463	75.8642298	117.2597638	-0.626925641	0.171191514	0.273470654	KRTCAP2
ENSG00000204091	3.389407643	0	4.093555618	0.171240534	0.273499936	TDRG1
ENSG00000229214	3.389407643	0	4.093555618	0.171240534	0.273499936	LINC00242
ENSG00000264290	14.98830537	6.562809401	1.199452425	0.171241425	0.273499936	AC104564.3
ENSG00000230894	2.755073482	0.336469917	2.831738336	0.17125973	0.273512358	AL133284.1
ENSG00000271357	3.270994492	0	4.044759658	0.171296482	0.273554237	AC048344.3
novel.458	62.99486847	92.5290387	-0.554782147	0.171308221	0.27355617	-
novel.275	16.86644834	6.64552593	1.333968171	0.171359056	0.273613587	-
ENSG00000251143	17.65652073	7.274731551	1.284600963	0.171365239	0.273613587	AP002490.1
ENSG00000132549	757.5085507	589.3775625	0.362238304	0.171382945	0.273625041	VPS13B
novel.1039	2.44546393	9.918180352	-2.02825361	0.171440806	0.273700602	-
ENSG00000251139	3.267233545	0	4.043189598	0.171533819	0.273781801	AC084871.1
ENSG00000259219	3.267233545	0	4.043189598	0.171533819	0.273781801	AC084855.2
ENSG00000232433	3.267233545	0	4.043189598	0.171533819	0.273781801	GXYLT1P3
ENSG00000287433	3.267233545	0	4.043189598	0.171533819	0.273781801	AC107222.2
ENSG00000014257	39.81928269	21.85705959	0.872285111	0.17168153	0.274000727	ACPP
ENSG00000104472	463.461424	569.3420182	-0.296358117	0.171759124	0.274107727	CHRAC1
ENSG00000229240	3.380632102	0	4.089996195	0.171769747	0.274107843	LINC00710
ENSG00000133997	263.8481202	339.8473001	-0.36501803	0.171817802	0.27416769	MED6
ENSG00000103037	273.1384879	192.9616762	0.500407977	0.171880651	0.2742458	SETD6
ENSG00000215271	217.8455646	299.4233231	-0.457773643	0.171889992	0.2742458	HOMEZ
ENSG00000137628	728.2110624	947.8246665	-0.380137549	0.17189842	0.2742458	DDX60
ENSG00000178381	396.3188881	289.0194345	0.454996767	0.171938266	0.274292527	ZFAND2A
ENSG00000283498	72.69083463	96.79561009	-0.412585467	0.171990166	0.27434802	MIR1244-2
novel.180	11.71988945	5.084746798	1.212007357	0.171994171	0.27434802	-
ENSG00000151366	485.2412511	373.5070404	0.377276832	0.172177762	0.274624006	NDUFC2
ENSG00000255537	6.625157428	2.058957692	1.660226689	0.172362372	0.274901584	AP000708.1
ENSG00000142188	55.59203294	113.8326736	-1.035115	0.172403339	0.274950043	TMEM50B
ENSG00000229474	44.53259082	24.37290178	0.875374196	0.172428803	0.274973774	PATL2
ENSG00000145882	484.6601282	368.8263725	0.393947781	0.172442219	0.27497829	PCYOX1L
ENSG00000104231	414.8182614	526.0502147	-0.342975417	0.172472332	0.27500943	ZFAND1
ENSG00000224885	2.753819833	0.387220594	2.831196352	0.172575864	0.275157626	EIPR1-IT1
ENSG00000228960	9.037812676	2.169915658	2.061145646	0.172683477	0.275312311	OR2A9P
ENSG00000148483	5.731573477	11.6274871	-1.014068916	0.172754806	0.275409131	TMEM236
ENSG00000262966	2.709728314	0.387220594	2.811284738	0.172787523	0.275444388	AC005695.1
ENSG00000187790	118.5548478	157.3776013	-0.410064108	0.172872112	0.275562326	FANCM
ENSG00000185390	3.361684828	0	4.082255806	0.172922754	0.275609231	FGF7P2
ENSG00000234965	3.361684828	0	4.082255806	0.172922754	0.275609231	SHISA8

ENSG00000250280	1.188078446	5.04345273	-2.07450682	0.173142156	0.275941993	AC026124.1
ENSG00000254718	12.77723172	6.103613158	1.064485906	0.173286967	0.276155842	AL157756.1
ENSG00000223476	2.71348926	0.356539013	2.812970257	0.173379647	0.276286593	VN1R42P
ENSG00000272420	9.944004385	3.180481283	1.639189689	0.173410618	0.276318998	AL513477.2
ENSG00000107281	874.2990651	1159.450819	-0.407542006	0.173431256	0.276321403	NPDC1
ENSG00000231105	9.206372128	1.936102971	2.27847216	0.173433398	0.276321403	AL031728.1
ENSG00000119636	157.5308351	120.5156411	0.38814566	0.173509072	0.276425019	BBOF1
ENSG00000231028	16.86122028	8.142501754	1.045452295	0.173520867	0.276426859	LINC00271
ENSG00000109854	505.247389	647.6306526	-0.358619923	0.173533571	0.276430148	HTATIP2
ENSG00000149090	1236.243616	2283.924092	-0.88560668	0.173558535	0.276442929	PAMR1
ENSG00000224376	5.160206839	12.71704484	-1.293805952	0.173562875	0.276442929	AC017104.1
ENSG00000279031	3.35040199	0	4.077644176	0.173613915	0.276490323	AC004232.2
ENSG00000287950	3.35040199	0	4.077644176	0.173613915	0.276490323	AC009432.3
novel.399	4.249000535	1.060160428	1.991077681	0.173696994	0.276605676	-
ENSG00000133131	490.3071927	595.0072361	-0.27967661	0.173719683	0.276624852	MORC4
ENSG00000251548	0.89358395	3.923085015	-2.125245144	0.173767317	0.276683745	AC106760.2
ENSG00000287005	2.726097019	6.950029995	-1.343421497	0.174075106	0.277156841	AC090018.2
novel.1077	27.02038294	53.73953309	-0.988430237	0.174158799	0.277273103	-
ENSG00000267317	108.8001455	72.90179808	0.579995931	0.174355321	0.27756897	AC027307.2
ENSG00000232727	13.64058555	23.23734525	-0.770090481	0.174439608	0.277686138	YWHAEP1
ENSG00000237017	3.058414791	0.356539013	2.983109537	0.174451952	0.277688774	AC245052.4
ENSG00000078725	1.541779519	10.63125838	-2.792759417	0.174481756	0.277719201	BRINP1
ENSG00000132300	1147.560128	1390.90313	-0.277435665	0.174555701	0.277819876	PTCD3
novel.532	8.855463088	18.29321052	-1.053673766	0.175000613	0.27851093	-
ENSG00000273112	3.532751578	0	4.156262698	0.175063445	0.278559735	AL590385.2
ENSG00000252271	3.532751578	0	4.156262698	0.175063445	0.278559735	RNU6-1110P
ENSG00000258121	3.532751578	0	4.156262698	0.175063445	0.278559735	AC089987.2
ENSG00000205089	3.93054417	0.713078025	2.456910794	0.175110499	0.278617544	CCNI2
ENSG00000165355	258.90045	326.2205487	-0.334222943	0.175126612	0.278626118	FBXO33
ENSG00000214435	3.617173671	0.693008929	2.356840116	0.175189154	0.278708555	AS3MT
ENSG00000259527	3.609580508	0.336469917	3.224491261	0.1752309	0.278747987	LINC00052
ENSG00000259209	8.726949475	3.159256312	1.455394888	0.175235398	0.278747987	AC004943.1
ENSG00000006625	403.3478491	529.1571109	-0.392191277	0.175274548	0.278777153	GGCT
ENSG00000196199	1253.994714	1058.076132	0.24509743	0.175285172	0.278777153	MPHOSPH8
ENSG00000108384	220.8860998	309.5432815	-0.487846419	0.175293487	0.278777153	RAD51C
ENSG00000214784	3.074783496	9.548460315	-1.636513725	0.175296653	0.278777153	AC010468.1
ENSG00000261420	5.073277448	1.41669944	1.835048959	0.175313253	0.278786487	AL022069.1
ENSG00000254119	3.615920022	0.356539013	3.226834931	0.175340102	0.278803259	AC025524.2
ENSG00000265443	1.480065645	5.940620245	-1.987090367	0.175345262	0.278803259	AC127024.3
ENSG00000257594	4.264115591	10.85317431	-1.355749633	0.175440871	0.278938209	GALNT4
ENSG00000282951	8.115323534	2.91483114	1.487283728	0.175511607	0.279033598	AC008537.4
ENSG00000272831	2.728604316	0.336469917	2.819809328	0.175556055	0.279060694	AC027644.3
ENSG00000272788	0	3.078979928	-4.20753104	0.175560873	0.279060694	AP000864.1
ENSG00000254131	0	3.078979928	-4.20753104	0.175560873	0.279060694	AC093367.1
ENSG00000286223	19.52177121	8.620482824	1.19342457	0.1756207	0.279138713	AC064836.4
ENSG00000130734	190.8426618	242.184001	-0.3429691	0.175728873	0.279286016	ATG4D
ENSG00000272870	102.9467539	58.97666031	0.806645937	0.175742763	0.279286016	AC097534.2
ENSG00000132475	11743.11155	9458.137007	0.312177829	0.175745625	0.279286016	H3F3B
novel.857	67.65204714	30.8592876	1.131172157	0.175799281	0.279354197	-
ENSG00000231851	13.29064543	5.574624622	1.268297153	0.175823365	0.27937538	AC104655.1
ENSG00000137094	656.3945828	1133.497096	-0.787920633	0.175927334	0.279519431	DNAJB5

ENSG00000166261	300.4685516	224.3574356	0.42157065	0.17593554	0.279519431	ZNF202
ENSG00000135823	912.4948402	768.8416828	0.247258118	0.176028604	0.279650186	STX6
ENSG00000153391	123.299711	179.367533	-0.541981214	0.176146918	0.279809876	INO80C
ENSG00000286952	6.908297814	1.813376645	1.935430029	0.176157886	0.279809876	AC093928.1
ENSG00000228335	7.404089172	1.702418679	2.09910505	0.176170581	0.279809876	AC073063.1
ENSG00000260903	3.940644631	0.774441189	2.401813147	0.176172201	0.279809876	XKR7
ENSG00000152558	3419.930835	4193.750108	-0.294242752	0.17625815	0.279929274	TMEM123
ENSG00000236423	4.499403511	10.24146924	-1.176236568	0.176293847	0.279968854	LINC01134
ENSG00000224945	102.0274559	186.6054059	-0.869729483	0.176317206	0.279988835	AL353150.1
ENSG00000128294	403.1976508	626.5883098	-0.635890369	0.176360189	0.280030391	TPST2
ENSG00000237803	0.894837599	5.206317249	-2.528291731	0.176364932	0.280030391	LINC00211
ENSG00000242175	3.594465451	0.356539013	3.218911431	0.176462842	0.28016873	RN7SL127P
ENSG00000230295	12.66884776	5.677410247	1.153733928	0.176475687	0.280172002	GTF2IP23
ENSG00000273805	3.342808826	0.693008929	2.238758178	0.176523127	0.280230195	AC083805.3
ENSG00000249222	0	2.913675265	-4.122806145	0.176554317	0.280246629	ATP5MGL
ENSG00000259318	18.92490432	5.083590922	1.899592567	0.176555053	0.280246629	AL356801.1
ENSG00000287319	2.139615324	0	3.429591747	0.176623392	1	AC079822.1
ENSG00000249167	2.139615324	0	3.429591747	0.176623392	1	SEMA6A-AS2
ENSG00000146521	2.139615324	0	3.429591747	0.176623392	1	LINC01558
ENSG00000272395	2.139615324	0	3.429591747	0.176623392	1	IFNL4
ENSG00000233936	2.139615324	0	3.429591747	0.176623392	1	AL390778.2
ENSG00000156860	1619.917474	2029.306694	-0.324894435	0.176640948	0.280365843	FBRS
ENSG00000213965	179.0187901	239.2287405	-0.419521349	0.176766765	0.280548401	NUDT19
ENSG00000099246	1213.005957	1471.050194	-0.278377237	0.176802453	0.280587902	RAB18
ENSG00000178057	1680.471807	1372.186102	0.292384673	0.17681846	0.280596164	NDUFAF3
ENSG00000086589	855.2608064	714.0478158	0.260195054	0.176838305	0.280610517	RBM22
ENSG00000039139	27.92811303	47.49556465	-0.762874654	0.176873694	0.280649532	DNAH5
ENSG00000250687	9.616772479	3.362259022	1.535030108	0.176985224	0.280802163	AC146944.2
ENSG00000115539	332.9429083	438.2203848	-0.396976967	0.176991503	0.280802163	PDCL3
novel.375	50.08486364	28.60896717	0.808269804	0.177077062	0.280912819	-
ENSG00000188312	64.66634398	93.00290984	-0.526525043	0.177082874	0.280912819	CENPP
ENSG00000072736	183.7236071	254.9542364	-0.473616359	0.177094297	0.280913787	NFATC3
ENSG00000108947	48.86128324	110.2865354	-1.174494053	0.177116838	0.280932391	EFNB3
ENSG00000186300	166.5873815	119.5001952	0.481472844	0.177154691	0.280962207	ZNF555
ENSG00000276462	2.695866906	0.356539013	2.804947037	0.177161494	0.280962207	BX255923.2
ENSG00000278905	10.94944851	2.139234076	2.354285808	0.177168078	0.280962207	AC106818.1
ENSG00000213676	6.67809576	18.28730275	-1.454228647	0.177224752	0.28102286	ATF6B
ENSG00000225855	114.4402022	79.32656396	0.530104528	0.177227957	0.28102286	RUSC1-AS1
ENSG00000119048	1188.928482	991.9546588	0.261298386	0.177245679	0.281033809	UBE2B
ENSG00000198658	30.20125656	17.50918433	0.783648228	0.17727556	0.281064034	ABHD17AP1
ENSG00000214940	4.179693498	0.672939833	2.59320067	0.177390233	0.281228682	NPIPA8
ENSG00000121680	374.3226758	463.1562831	-0.306773881	0.177403589	0.281232696	PEX16
ENSG00000162302	1484.690962	1167.199503	0.346825258	0.177578839	0.281493338	RPS6KA4
ENSG00000278845	4.561117385	10.53908524	-1.216096575	0.177643582	0.281578292	MRPL45
novel.1101	7.827097281	3.260757667	1.270931521	0.177654107	0.281578292	-
ENSG00000068697	4644.449074	6140.508108	-0.402859551	0.177699169	0.281619451	LAPTM4A
ENSG00000144840	355.2918517	439.4583758	-0.307289361	0.177701754	0.281619451	RABL3
ENSG00000280300	11.40394038	5.370466037	1.087434847	0.177799875	0.281757765	AC117503.4
ENSG00000104901	10.71695262	3.392940604	1.676259173	0.177868376	0.281849127	DKKL1
ENSG00000160584	882.8712775	653.3854411	0.434694544	0.177908164	0.281894981	SIK3
ENSG00000254577	7.814489523	2.893606169	1.438989512	0.177969254	0.281974582	AC087276.1

ENSG00000159915	14.82608543	7.234593359	1.03865167	0.178063738	0.282092111	ZNF233
ENSG00000275106	5.174068246	1.161661783	2.199448083	0.178085092	0.282092111	AC025594.2
ENSG00000239828	5.174068246	1.161661783	2.199448083	0.178085092	0.282092111	AC063944.1
ENSG00000099960	3.945659226	0.713078025	2.461796205	0.178086863	0.282092111	SLC7A4
ENSG00000081154	2913.488403	2454.843378	0.247152857	0.17813802	0.282155941	PCNP
ENSG00000175691	84.40180599	58.60968439	0.52820071	0.178200494	0.282213204	ZNF77
ENSG00000173376	28.29574643	74.25084141	-1.391958581	0.178206263	0.282213204	NDNF
ENSG00000147586	160.3336901	206.0162997	-0.362572038	0.178206759	0.282213204	MRPS28
ENSG00000228057	1.832513069	5.677410247	-1.639706136	0.178309628	0.282358899	SEC63P1
ENSG00000235688	14.71311415	33.69512663	-1.199146556	0.178412791	0.282505042	SNTG2-AS1
ENSG00000160075	2029.469549	2431.431953	-0.260783471	0.178426614	0.282509711	SSU72
ENSG00000144468	447.6499528	537.9235397	-0.265436148	0.178520009	0.282640363	RHBDD1
ENSG00000223343	6.056298086	1.702418679	1.799466993	0.178549058	0.282649407	AC137630.1
ENSG00000102057	99.28236814	145.5434686	-0.550670039	0.178554864	0.282649407	KCND1
ENSG00000200737	0.308355904	3.148643826	-3.278030901	0.178558359	0.282649407	RF00019
ENSG00000226564	13.66705472	27.48243489	-1.003503995	0.178781586	0.282985524	FTH1P20
ENSG00000251322	5.839886168	19.35054562	-1.727132169	0.17879546	0.282990243	SHANK3
ENSG00000139220	12.3214862	33.14550824	-1.423844042	0.178811336	0.282998132	PPFIA2
ENSG00000197768	6.933513331	2.42610919	1.494922239	0.178983892	0.283253974	STPG3
ENSG00000275468	0.293240848	3.148643826	-3.27802118	0.178996218	0.283256228	AC004678.1
ENSG00000259028	2.111892509	0	3.412816963	0.179309872	1	AL135746.1
ENSG00000280222	2.111892509	0	3.412816963	0.179309872	1	AL365209.1
ENSG00000237614	2.111892509	0	3.412816963	0.179309872	1	AC073257.2
ENSG00000285165	2.111892509	0	3.412816963	0.179309872	1	AC124014.1
ENSG00000236542	2.111892509	0	3.412816963	0.179309872	1	MED28P7
ENSG00000270554	2.111892509	0	3.412816963	0.179309872	1	AC069431.2
ENSG00000183128	2.111892509	0	3.412816963	0.179309872	1	CALHM3
ENSG00000231102	2.111892509	0	3.412816963	0.179309872	1	AL035588.1
ENSG00000236905	2.109385211	0	3.411300221	0.179555314	1	AC104333.3
ENSG00000288061	2.109385211	0	3.411300221	0.179555314	1	AP000926.2
novel.582	2.109385211	0	3.411300221	0.179555314	1	-
ENSG00000234210	2.109385211	0	3.411300221	0.179555314	1	AC006372.3
ENSG00000186150	2.109385211	0	3.411300221	0.179555314	1	UBL4B
ENSG00000253838	2.109385211	0	3.411300221	0.179555314	1	AC007991.2
ENSG00000237062	2.109385211	0	3.411300221	0.179555314	1	AL512649.2
ENSG00000175820	3.255879435	0	4.038359851	0.179580919	0.284155721	CCDC168
ENSG00000119403	826.3005116	1183.793261	-0.518853325	0.179586503	0.284155721	PHF19
ENSG00000089723	0.922560414	4.534533291	-2.295817818	0.17972673	0.284360281	OTUB2
ENSG00000223963	3.253372138	0	4.0373067	0.179743274	0.284369138	THAP12P8
ENSG00000226781	13.56117806	25.37516667	-0.897691213	0.179769714	0.284393652	TBCAP1
ENSG00000163737	8.180798353	28.46416396	-1.801464807	0.179787287	0.284404134	PF4
ENSG00000117153	651.789457	779.6404761	-0.258665536	0.179811289	0.284424785	KLHL12
ENSG00000179008	15.34995561	36.42774767	-1.246898757	0.179930976	0.284596778	C14orf39
ENSG00000236264	15.11932628	27.4518817	-0.856254542	0.180115258	0.284870915	RPL26P30
ENSG00000246662	8.557207645	3.200550379	1.412211676	0.180135984	0.284886352	LINC00535
ENSG00000142871	10616.57228	18670.84133	-0.814469077	0.180170938	0.284924288	CCN1
ENSG00000277368	3.579350395	0.356539013	3.213286452	0.180189057	0.284935599	AL138966.2
ENSG00000254363	30.31749059	14.07250951	1.110022315	0.180233713	0.284988869	AC011379.2
ENSG00000224975	27.59049593	47.43561415	-0.778805032	0.18031145	0.285094437	INE1
ENSG00000137154	16286.53526	20440.21107	-0.327737842	0.180361188	0.285155725	RPS6
ENSG00000257763	2.961384939	0.356539013	2.94190911	0.180395616	0.285192802	OR5BK1P

ENSG00000236810	19.04109491	30.82745015	-0.698072118	0.180446154	0.285255341	ELOA-AS1
ENSG00000285996	10.31665273	4.381125383	1.243447682	0.180458786	0.285257953	AC006116.11
ENSG00000272087	13.91996499	7.042203135	0.973088506	0.180531047	0.285354817	AC080013.4
ENSG00000128534	853.1089545	700.5225658	0.284102486	0.180572454	0.285402904	LSM8
ENSG00000232573	1402.177058	1736.816548	-0.308740983	0.180594039	0.285404453	RPL3P4
ENSG00000237424	134.5707201	67.46004555	0.994398508	0.180595404	0.285404453	FOXD2-AS1
ENSG00000184182	212.4787894	161.810248	0.392409071	0.180679834	0.285520514	UBE2F
ENSG00000254349	2.140868972	0	3.430378531	0.180697065	1	MIR2052HG
novel.27	2.140868972	0	3.430378531	0.180697065	1	-
ENSG00000233008	2.140868972	0	3.430378531	0.180697065	1	LINC01725
ENSG00000274508	2.140868972	0	3.430378531	0.180697065	1	AC007336.2
ENSG00000279275	2.140868972	0	3.430378531	0.180697065	1	AP002505.2
ENSG00000250988	20.27326487	31.25712068	-0.627788123	0.180716254	0.285547463	SNHG21
ENSG00000228653	9.937736141	4.330374705	1.202413946	0.18072816	0.285547463	HNRNPCP7
ENSG00000172725	2023.716507	2481.261231	-0.294061049	0.180729859	0.285547463	CORO1B
ENSG00000171566	991.0349498	1205.247197	-0.282490252	0.18076174	0.285580466	PLRG1
ENSG00000250959	61.67659548	40.9095225	0.591000733	0.180801145	0.285581557	GLUD1P3
ENSG00000204022	3.885199001	0.672939833	2.485475027	0.180801651	0.285581557	LIPJ
ENSG00000181007	158.0022655	117.9517452	0.421144622	0.180806197	0.285581557	ZFP82
ENSG00000262519	3.01557692	9.153067379	-1.603722538	0.18081723	0.285581557	TXNP4
ENSG00000267080	101.2998806	140.2038594	-0.470882725	0.1808177	0.285581557	ASB16-AS1
ENSG00000125266	656.6069446	368.3857636	0.833258408	0.180828382	0.285581557	EFNB2
ENSG00000259169	2.961384939	0.336469917	2.941898634	0.180865454	0.285622742	GNRHR2P1
ENSG00000226855	2.75758078	0.387220594	2.833006979	0.180889326	0.285625718	RPSAP17
ENSG00000235917	2.75758078	0.387220594	2.833006979	0.180889326	0.285625718	MTCO2P11
ENSG00000287259	2.138361675	0	3.428872685	0.180940158	1	AL359732.1
novel.80	2.138361675	0	3.428872685	0.180940158	1	-
ENSG00000238755	2.138361675	0	3.428872685	0.180940158	1	LINC02006
ENSG00000252172	2.138361675	0	3.428872685	0.180940158	1	RNU6-720P
ENSG00000204283	2.138361675	0	3.428872685	0.180940158	1	LINC01973
ENSG00000235538	2.138361675	0	3.428872685	0.180940158	1	AL078602.1
ENSG00000207325	2.138361675	0	3.428872685	0.180940158	1	RNY1P4
ENSG00000130702	1932.558397	1194.874575	0.693458111	0.18104397	0.28585253	LAMA5
ENSG00000255100	1.84261353	8.205020793	-2.160163884	0.181068161	0.285873352	AP003119.2
ENSG00000272078	8.290435964	2.171071533	1.935329862	0.181145943	0.285978777	AL139423.1
ENSG00000251188	15.38491558	8.15311424	0.910690905	0.18117065	0.286000404	AC079140.3
ENSG00000186891	9.239394272	1.844058227	2.332835859	0.181207119	0.286040596	TNFRSF18
ENSG00000008405	341.4775068	239.7388158	0.509338585	0.181366174	0.286263772	CRY1
ENSG00000160838	6.318055173	14.58502497	-1.211341857	0.181370538	0.286263772	LRRC71
ENSG00000124134	20.81099646	9.091704216	1.186076774	0.181390749	0.286278281	KCNS1
ENSG00000049656	479.60477	644.2483717	-0.426219573	0.181410818	0.286283718	CLPTM1L
ENSG00000142396	109.4850528	71.05945649	0.621154173	0.181416233	0.286283718	ERVK3-1
ENSG00000160951	3.914175465	10.03029419	-1.363721192	0.181484035	0.286358233	PTGER1
ENSG00000140740	106.7738571	172.0135869	-0.6873899	0.181485496	0.286358233	UQCRC2
ENSG00000161618	477.045451	687.2209146	-0.526844644	0.181685567	0.286656507	ALDH16A1
ENSG00000277400	15.49942525	4.096562019	1.928383196	0.18178257	0.28679214	AC145212.1
ENSG00000239884	8.452655901	3.546476906	1.246527479	0.181838371	0.286862755	RN7SL608P
ENSG00000234009	87.45262762	117.2492797	-0.423065169	0.181905984	0.286951996	RPL5P34
ENSG00000169758	20.94388393	10.53780097	0.997349338	0.181951463	0.287006311	TMEM266
ENSG00000163497	5.550406267	0	4.805774196	0.181980719	0.287035032	FEV
ENSG00000271761	2.127007565	0	3.421991857	0.182050784	1	AL021368.1

ENSG00000233760	2.127007565	0	3.421991857	0.182050784	1	AC004947.1
ENSG00000132704	2.127007565	0	3.421991857	0.182050784	1	FCRL2
ENSG00000249026	2.127007565	0	3.421991857	0.182050784	1	CTNNA1P1
ENSG00000179873	2.127007565	0	3.421991857	0.182050784	1	NLRP11
ENSG00000147459	1458.373502	925.3109517	0.656530345	0.182151077	0.287286295	DOCK5
ENSG00000255201	3.675126598	14.3462036	-1.963562566	0.182240592	0.287406475	AC087623.1
ENSG00000228434	2.666890443	8.640680314	-1.687290476	0.182249401	0.287406475	AC004951.1
ENSG00000278311	32.93716931	52.59272178	-0.676482011	0.182371725	0.287581925	GGNBP2
ENSG00000241889	2.449224876	6.716345703	-1.466055247	0.182403158	0.287614035	AC079944.2
ENSG00000248514	2.123246619	0	3.419721584	0.182419826	1	AC008443.4
ENSG00000256039	2.123246619	0	3.419721584	0.182419826	1	LINC02446
ENSG00000231665	3.649911081	0.672939833	2.389294017	0.182443193	0.287648142	OGFOD1P1
ENSG00000164796	3.082305388	10.3029608	-1.748031518	0.182446931	0.287648142	CSMD3
ENSG00000227733	5.951746342	1.753169357	1.75321503	0.182491337	0.287700693	AC239809.3
ENSG00000188868	39.27814616	24.246755	0.693007233	0.182553314	0.287764285	ZNF563
ENSG00000230069	2.752566185	0.336469917	2.830762385	0.182553826	0.287764285	LRRC37A15P
ENSG00000269373	2.743719372	0.356539013	2.826715147	0.182623983	0.287839948	AC008739.2
ENSG00000215910	2.743719372	0.356539013	2.826715147	0.182623983	0.287839948	C1orf167
ENSG00000129460	524.6185947	413.0862572	0.344721506	0.182684172	0.287907977	NGDN
ENSG00000284471	5.921516229	1.00940975	2.522266864	0.182689308	0.287907977	CR769775.2
ENSG00000256393	31.21923717	47.54417915	-0.606219157	0.182871919	0.288178282	RPL41P5
ENSG00000234264	0	2.751966622	-4.045835036	0.182895461	0.288197899	DEPDC1-AS1
ENSG00000104432	28.2148009	14.53299003	0.954813389	0.182921315	0.288221158	IL7
ENSG00000232134	2.737451129	0.356539013	2.823878069	0.183047882	0.288384224	RPS15AP12
ENSG00000249937	2.737451129	0.356539013	2.823878069	0.183047882	0.288384224	LINC02223
novel.1	1.846374476	9.078523192	-2.298080932	0.183058105	0.288384224	-
ENSG00000223431	5.640883141	0.713078025	2.980902794	0.18311615	0.288432807	MTND6P21
ENSG00000204514	181.9185321	229.922732	-0.338383536	0.18311672	0.288432807	ZNF814
ENSG00000235363	13.41163715	25.74373083	-0.943085147	0.183126191	0.288432807	SNRPGP10
ENSG00000178537	285.593601	227.5288454	0.326773079	0.183133351	0.288432807	SLC25A20
ENSG00000274987	8.719427583	3.352802412	1.399933023	0.183262589	0.28861886	AC092794.1
ENSG00000275074	142.7026693	204.1414787	-0.517597497	0.183284371	0.288621341	NUDT18
ENSG00000175175	8.523145316	3.169868797	1.416001576	0.183286382	0.288621341	PPM1E
ENSG00000279622	3.077290793	0	3.954147645	0.183360483	0.288685535	AC025280.2
novel.453	3.077290793	0	3.954147645	0.183360483	0.288685535	-
ENSG00000226965	3.077290793	0	3.954147645	0.183360483	0.288685535	AC073114.1
ENSG00000103274	220.5270997	171.6646251	0.36074194	0.183384933	0.288706535	NUBP1
ENSG00000276997	2.387511003	7.733927794	-1.684585793	0.183426698	0.288754789	AL513314.2
ENSG00000263887	3.313832363	0.693008929	2.229155612	0.183471866	0.288804277	AC053481.2
ENSG00000112419	611.5205772	1188.911163	-0.959326829	0.183480581	0.288804277	PHACTR2
ENSG00000146373	458.7961539	593.4085018	-0.371539528	0.183491482	0.288804277	RNF217
ENSG00000101574	192.7275003	241.0908944	-0.323201003	0.183560818	0.288895906	METTL4
ENSG00000278834	16.00538834	8.205020793	0.96211638	0.183627151	0.288982798	AC073508.3
ENSG00000164932	641.5076719	1321.205924	-1.0423915	0.183661057	0.289018651	CTHRC1
ENSG00000263072	174.2012584	229.2294191	-0.395059566	0.183683263	0.289036088	ZNF213-AS1
ENSG00000167306	17.48316015	7.561606666	1.208525191	0.183729649	0.28909157	MYO5B
ENSG00000154548	18.8385444	9.87675789	0.923756932	0.183755523	0.289114774	SRSF12
ENSG00000205531	321.75498	427.6227493	-0.410290861	0.183789383	0.289150537	NAP1L4
ENSG00000233232	3.619680969	0.672939833	2.378983753	0.183871462	0.289262152	NPIPB7
ENSG00000267044	0.586481695	3.515795324	-2.566986096	0.183917891	0.289317675	AC005757.1
novel.1109	33.37493647	17.48654669	0.941854652	0.183938995	0.289333355	-

ENSG00000256238	3.352909287	8.315978758	-1.311558307	0.183950834	0.28933446	SUPT16HP1
ENSG00000146066	514.9320716	661.1078364	-0.360353369	0.184001683	0.289396921	HIGD2A
ENSG00000000460	138.7158965	182.2853994	-0.394883169	0.184150485	0.289613424	C1orf112
ENSG00000145246	424.2078366	604.0005679	-0.51028885	0.184211397	0.289691683	ATP10D
ENSG00000133083	201.2908608	83.23425023	1.272697013	0.184319121	0.289791301	DCLK1
ENSG00000169696	559.4836535	668.117945	-0.255825873	0.184327076	0.289791301	ASPSCR1
ENSG00000272817	3.078544442	0	3.95471052	0.184341666	0.289791301	AL359198.1
ENSG00000211787	3.078544442	0	3.95471052	0.184341666	0.289791301	TRAV8-3
ENSG00000167910	3.078544442	0	3.95471052	0.184341666	0.289791301	CYP7A1
ENSG00000143355	3.078544442	0	3.95471052	0.184341666	0.289791301	LHX9
ENSG00000214753	1623.392101	1344.863085	0.271676465	0.184358437	0.289800131	HNRNPUL2
ENSG00000260920	34.18946892	48.86408195	-0.512411711	0.184411516	0.28986603	AL031985.3
ENSG00000117620	488.5213222	367.6999683	0.409298696	0.184438364	0.289890692	SLC35A3
ENSG00000261762	1.233423615	5.909938664	-2.264471521	0.184474918	0.289921239	AC027228.2
ENSG00000138376	178.4167386	266.0393443	-0.576098428	0.184480117	0.289921239	BARD1
ENSG00000183036	3.076037145	0	3.953598079	0.184519068	0.289947375	PCP4
ENSG00000233942	3.076037145	0	3.953598079	0.184519068	0.289947375	AC004012.1
ENSG00000267650	4.134348329	0.723690511	2.524234058	0.184634811	0.290097548	AC008742.1
ENSG00000143575	1054.853766	1340.753462	-0.346258997	0.184636967	0.290097548	HAX1
ENSG00000006025	357.0202559	278.4087931	0.359328753	0.18474335	0.290247142	AC003665.1
ENSG00000166887	1697.730935	2074.383611	-0.288903096	0.184847236	0.290392795	VPS39
ENSG00000234998	0.921306765	4.555886656	-2.318252412	0.184869002	0.290409428	AC244453.3
ENSG00000167565	647.0792684	506.2572974	0.353996629	0.185027581	0.290640967	SERTAD3
ENSG00000230162	2.096777453	0	3.403566739	0.185057124	1	CT45A11P
ENSG00000279135	2.096777453	0	3.403566739	0.185057124	1	AL512652.2
ENSG00000236894	2.096777453	0	3.403566739	0.185057124	1	AL160287.1
ENSG00000229356	2.096777453	0	3.403566739	0.185057124	1	LRRC3-DT
ENSG00000204389	2.096777453	0	3.403566739	0.185057124	1	HSPA1A
ENSG00000275846	2.096777453	0	3.403566739	0.185057124	1	AL513548.3
ENSG00000100721	2.096777453	0	3.403566739	0.185057124	1	TCL1A
ENSG00000166349	10.02466553	21.24548292	-1.078417472	0.185071573	0.290692493	RAG1
novel.862	8.795002863	3.944309986	1.156068325	0.185095489	0.290712483	-
ENSG00000104361	488.2982534	311.730779	0.64699911	0.185153632	0.290786224	NIPAL2
ENSG00000224666	2.095523804	0	3.402802199	0.185183147	1	Z84484.1
ENSG00000284987	2.095523804	0	3.402802199	0.185183147	1	U52112.2
ENSG00000287449	2.095523804	0	3.402802199	0.185183147	1	AC108019.2
ENSG00000136213	500.0250682	648.949387	-0.376065468	0.18519229	0.290820754	CHST12
ENSG00000285518	5.69632877	0.774441189	2.917354874	0.185198011	0.290820754	AC004900.1
ENSG00000243926	19.68176858	9.711324833	1.032893658	0.185210478	0.290820754	TIPARP-AS1
novel.460	29.55152261	47.75419833	-0.688782604	0.185226333	0.290820754	-
ENSG00000250271	0.308355904	3.384768263	-3.385488754	0.185231586	0.290820754	AC068647.2
ENSG00000160161	15.2428253	29.68418801	-0.958247974	0.185265173	0.29085591	CILP2
ENSG00000236723	6.65538754	2.537067156	1.39760139	0.185437211	0.291107909	AL606760.2
ENSG00000212978	194.9210225	245.2549842	-0.332107371	0.185448097	0.291107909	AC016747.1
ENSG00000204540	5.969368695	1.487519214	2.025584358	0.185554324	0.291257063	PSORS1C1
ENSG00000263624	1.788421549	5.817893919	-1.690115167	0.185597117	0.291306634	AC055811.1
ENSG00000251692	0	2.793260689	-4.065558726	0.185646975	0.291367286	PTX4
ENSG00000136111	631.1095642	973.7165088	-0.625910531	0.185802153	0.291552087	TBC1D4
ENSG00000261033	3.390661292	0	4.094151094	0.185809609	0.291552087	AC005730.2
ENSG00000278196	3.390661292	0	4.094151094	0.185809609	0.291552087	IGLV2-8
ENSG00000239389	3.390661292	0	4.094151094	0.185809609	0.291552087	PCDHA13

ENSG00000180525	4.206162664	9.945137519	-1.230893293	0.185934338	0.291725081	PRR26
novel.188	10.6703538	4.717595299	1.181185424	0.185942317	0.291725081	-
ENSG00000076201	1042.259841	1295.683454	-0.313922237	0.186074907	0.291889877	PTPN23
ENSG00000277386	4.267876537	1.029478846	2.017636063	0.186079163	0.291889877	AL138999.1
ENSG00000256745	73.62858137	100.128215	-0.4422212	0.18608106	0.291889877	AP002784.2
ENSG00000286024	8.821472029	3.882946823	1.172171741	0.18612682	0.291944029	AC026803.3
ENSG00000101132	386.7263897	489.8762679	-0.341117991	0.186359907	0.292291985	PFDN4
ENSG00000230896	46.53860666	31.26888904	0.570676276	0.186397707	0.292333625	AL604028.1
ENSG00000235480	3.379378453	0	4.089557472	0.186514263	0.292481112	AC013270.1
ENSG00000211788	3.379378453	0	4.089557472	0.186514263	0.292481112	TRAV13-1
ENSG00000204650	7.929141728	2.375358513	1.71128972	0.186530731	0.292489283	LINC02210
ENSG00000257543	11.19762892	5.259508071	1.077731068	0.186723595	0.292774033	AC063948.1
ENSG00000248254	4.822874471	1.069617038	2.168358251	0.186806249	0.292884501	AC107398.2
ENSG00000279801	6.875560404	16.31208907	-1.241433607	0.186816595	0.292884501	AC111170.3
ENSG00000260971	7.76287611	1.518200796	2.377271815	0.186865224	0.292943064	AC119674.1
ENSG00000226053	2.682005499	0.356539013	2.798439144	0.186878397	0.292946038	LINC01776
ENSG00000205488	3.993511692	0.356539013	3.368358117	0.186918402	0.29299107	CALML3-AS1
ENSG00000257831	7.569101141	2.884149558	1.394736892	0.187103535	0.293263571	AL136418.1
ENSG00000230658	13.097013	4.606637334	1.502534282	0.187258895	0.293489374	KLHL7-DT
ENSG00000258976	0.616711807	3.587770973	-2.552102267	0.187288082	0.293517413	AC013451.2
ENSG00000243284	2.683259148	0.336469917	2.799003302	0.187345683	0.293589975	VSIG8
ENSG00000130377	5.789526405	1.783850939	1.693849024	0.187357337	0.293590529	ACSBG2
ENSG00000226455	3.364263397	0	4.083369349	0.187465642	0.293742527	AL121787.1
ENSG00000002016	233.8373766	177.1151107	0.401165342	0.18748128	0.293749314	RAD52
ENSG00000131381	785.7466137	645.941051	0.282707544	0.187590392	0.293891342	RBSN
ENSG00000284308	13.38635036	6.593490983	1.030553795	0.18759455	0.293891342	C2orf81
ENSG00000213638	44.9190293	67.50591594	-0.590738955	0.187659756	0.293920876	ADAT3
ENSG00000143815	692.1812449	889.3924656	-0.362039426	0.187669979	0.293920876	LBR
ENSG00000081320	262.4311484	438.5905244	-0.741243511	0.18769254	0.293920876	STK17B
ENSG00000147408	850.5704793	676.3085814	0.330801481	0.187718467	0.293920876	CSGALNACT1
ENSG00000211786	3.079798091	0	3.955289135	0.187726531	0.293920876	TRAV8-2
ENSG00000211799	3.079798091	0	3.955289135	0.187726531	0.293920876	TRAV19
ENSG00000260035	3.079798091	0	3.955289135	0.187726531	0.293920876	AC051619.8
ENSG00000211997	3.079798091	0	3.955289135	0.187726531	0.293920876	MIR708
ENSG00000255258	3.079798091	0	3.955289135	0.187726531	0.293920876	AP001979.2
ENSG00000266573	3.079798091	0	3.955289135	0.187726531	0.293920876	AC018697.1
ENSG00000164292	2549.551011	3945.940967	-0.630200317	0.187750052	0.293939989	RHOBTB3
ENSG00000196581	85.3686283	39.43377164	1.113116863	0.187834281	0.294054137	AJAP1
ENSG00000272734	79.30972382	113.2453575	-0.511588773	0.187998741	0.294293866	ADIRF-AS1
ENSG00000251310	3.074783496	0	3.953061971	0.188083903	0.294394486	AC107391.1
ENSG00000263400	14.81097038	7.419967118	0.990373618	0.188085681	0.294394486	TMEM220-AS1
ENSG00000104660	977.2670441	754.6129526	0.372650666	0.188120426	0.294424534	LEPROTL1
ENSG00000138639	952.3975913	788.7066233	0.272429643	0.188127543	0.294424534	ARHGAP24
ENSG00000009844	800.6382456	996.2082713	-0.315615264	0.188166859	0.294468326	VTA1
ENSG00000103599	18.63320186	30.31853071	-0.70154065	0.188219409	0.294532823	IQCH
ENSG00000122121	15.99431896	5.656185276	1.49391649	0.188246618	0.29455766	XPNPEP2
ENSG00000231503	3.32769377	7.510855988	-1.175397917	0.188306607	0.294633782	PTMAP4
ENSG00000104369	14.23842136	6.625456834	1.091234753	0.188402123	0.29476548	JPH1
ENSG00000201820	6.99021261	2.833398881	1.292826622	0.188491848	0.294888102	RF00019
ENSG00000168246	578.4374206	448.2844302	0.367987619	0.18850955	0.294898039	UBTD2
ENSG00000160685	784.5492608	630.0647501	0.3166759	0.188642781	0.295088692	ZBTB7B

ENSG00000137709	4.262861942	1.10029862	1.967438129	0.188657125	0.295090892	POU2F3
ENSG00000205746	134.3038219	93.07231695	0.529326399	0.188666903	0.295090892	AC126755.1
ENSG00000124459	217.5577656	170.5516118	0.350550859	0.188700048	0.295124967	ZNF45
ENSG00000234618	10.3594906	19.33445773	-0.907558697	0.188749667	0.295184801	RPSAP9
ENSG00000107593	3.001715513	0	3.920174413	0.188822102	0.295280307	PKD2L1
ENSG00000005889	735.1936116	555.6500508	0.404098826	0.188877535	0.295323803	ZFX
ENSG00000236842	3.01557692	0	3.926461087	0.188881621	0.295323803	AC010997.2
ENSG00000156304	552.2030355	672.4186184	-0.283794373	0.188884017	0.295323803	SCAF4
ENSG00000224072	8.758504507	3.566546002	1.293057135	0.188966421	0.295432479	AL139811.1
ENSG00000129682	77.14477826	30.32400611	1.349565099	0.188976266	0.295432479	FGF13
ENSG00000231345	9.920113788	24.02034397	-1.277656701	0.189030621	0.295499673	BEND3P1
ENSG00000124224	349.1139371	230.0112151	0.603008362	0.189222757	0.29578223	PPP4R1L
ENSG00000188848	3.687734357	0.336469917	3.253213077	0.189269054	0.295809143	BEND4
ENSG00000273335	6.613803318	2.241891306	1.58563413	0.189280663	0.295809143	AP005432.2
ENSG00000269952	10.68922981	4.596024848	1.208055114	0.189294289	0.295809143	AL117336.1
ENSG00000224295	8.172022812	3.291439249	1.322732913	0.189294405	0.295809143	OLFM5P
ENSG00000196975	1450.731722	1190.287537	0.285264693	0.189296902	0.295809143	ANXA4
ENSG00000227788	4.274216052	0.774441189	2.511523296	0.189390434	0.295937504	MTCO3P43
ENSG00000287665	0.616711807	3.637365776	-2.569361122	0.189407136	0.295945802	AC092428.1
ENSG00000130254	1525.31062	1257.258031	0.278909964	0.189454472	0.296001962	SAFB2
ENSG00000196628	1775.419299	1003.055399	0.82362913	0.189482405	0.296027803	TCF4
ENSG00000248636	5.186676005	17.35192361	-1.740012794	0.189545089	0.296107928	AC002070.1
ENSG00000272807	19.94352567	11.79048002	0.75646872	0.189619048	0.296205658	AC007038.2
ENSG00000185495	162.3628849	111.4729366	0.542119623	0.189780085	0.29643551	AC138393.1
ENSG00000162004	61.64944214	84.65958873	-0.460195452	0.18978901	0.29643551	CCDC78
ENSG00000258824	18.07318933	10.42568713	0.796203909	0.189979064	0.296714522	AL122035.1
ENSG00000229990	0.600343103	2.904218654	-2.263625824	0.19022755	0.297084754	AC022400.2
ENSG00000273628	6.213503428	1.10029862	2.509262054	0.190312883	0.297200157	AL354798.1
ENSG00000226334	3.943151929	9.923912548	-1.3269864	0.190509491	0.297480229	AL359182.1
ENSG00000286034	0	2.731897526	-4.035831563	0.190534372	0.297480229	AC100822.1
ENSG00000231756	0	2.731897526	-4.035831563	0.190534372	0.297480229	AL449983.1
ENSG00000232642	4.486795753	1.130980201	2.023319193	0.190553097	0.297480229	AC008073.2
novel.333	2.9928687	0	3.916136008	0.19056152	0.297480229	-
ENSG00000135625	2.9928687	0	3.916136008	0.19056152	0.297480229	EGR4
ENSG00000269720	6.69947906	2.567748738	1.395599198	0.190572377	0.297480229	CCDC194
ENSG00000140320	560.3621937	463.9590129	0.272734659	0.190588895	0.29748814	BAHD1
ENSG00000142444	301.6334077	385.1445793	-0.352471633	0.190669593	0.297596221	TIMM29
ENSG00000287918	7.929141728	2.406040094	1.699480726	0.190723113	0.297647482	AF274853.1
ENSG00000102970	3.038213869	0	3.936673302	0.190725349	0.297647482	CCL17
ENSG00000101384	857.3880076	402.4913433	1.090666116	0.190821341	0.297779401	JAG1
ENSG00000261242	3.916682763	0.672939833	2.49750561	0.190841372	0.297792773	AL136038.3
ENSG00000178234	1092.407581	840.0992149	0.378961168	0.190896866	0.297861478	GALNT11
ENSG00000235724	4.207416313	1.10029862	1.951899908	0.190970977	0.297958358	AC009299.3
ENSG00000186448	16.81455019	8.846123169	0.922116789	0.190981892	0.297958358	ZNF197
ENSG00000268030	23.44096127	37.56129641	-0.676817253	0.191005932	0.297977971	AC005253.1
ENSG00000236947	3.328947419	0.743759607	2.190779891	0.191036126	0.29800718	AL139412.1
ENSG00000162618	250.1330834	494.5189114	-0.983733676	0.191100062	0.29808902	ADGRL4
ENSG00000130810	130.9840773	185.0988107	-0.497454608	0.191219925	0.298243897	PPAN
ENSG00000196998	698.183181	567.8978952	0.298434341	0.19122231	0.298243897	WDR45
ENSG00000182986	74.91089764	54.98904848	0.446952128	0.191298616	0.298345	ZNF320
ENSG00000271833	11.14852281	4.698682079	1.251065696	0.191327514	0.29836178	AL445222.1

ENSG00000176095	992.9156268	1188.617714	-0.259704554	0.191345681	0.29836178	IP6K1
ENSG00000196932	6.442736568	1.803920035	1.837290746	0.191349923	0.29836178	TMEM26
ENSG00000261367	3.029438327	0	3.932726389	0.191366795	0.29836178	AC012645.2
ENSG00000261292	3.029438327	0	3.932726389	0.191366795	0.29836178	AC110491.1
ENSG00000196663	706.3389498	868.8786578	-0.298475962	0.191389716	0.298379611	TECPR2
ENSG00000214973	1.541779519	6.430626464	-2.068721847	0.191424024	0.298415191	CHCHD3P3
ENSG00000177855	5.787019107	11.44455349	-0.988078038	0.191472647	0.298468146	CACYBPP2
ENSG00000130055	10.40490704	3.597227584	1.531643104	0.191480969	0.298468146	GDPD2
ENSG00000275549	5.420710276	1.793307549	1.596457708	0.191497471	0.298475962	STPG3-AS1
ENSG00000178096	337.6206154	264.8092217	0.349517902	0.191514532	0.298484647	BOLA1
ENSG00000261207	10.19949323	19.42175058	-0.92574656	0.191540844	0.298507748	AL031717.1
ENSG00000129654	5.654744548	0.774441189	2.906641706	0.191631971	0.298631852	FOXJ1
ENSG00000172992	480.1967367	613.0312136	-0.352826714	0.191686892	0.298679697	DCAKD
ENSG00000235192	2.432856171	0.356539013	2.653500555	0.191697161	0.298679697	AC009495.3
ENSG00000211696	2.432856171	0.356539013	2.653500555	0.191697161	0.298679697	TRGV8
ENSG00000113805	12.89431995	4.545274171	1.493696412	0.191771756	0.298770446	CNTN3
ENSG00000066322	1887.479368	2336.035134	-0.307745408	0.191778405	0.298770446	ELOVL1
ENSG00000205309	83.70296593	58.03747522	0.527228578	0.192183779	0.299367116	NT5M
ENSG00000184584	988.7332932	1481.397394	-0.583449409	0.192184448	0.299367116	TMEM173
ENSG00000250764	14.10106093	6.664310756	1.096247286	0.192316442	0.299554765	AC025178.1
ENSG00000228238	8.217367981	2.0188195	1.998590202	0.192330031	0.299557973	AL096803.1
ENSG00000173276	353.7221081	482.4701454	-0.447115273	0.192393522	0.299627462	ZBTB21
ENSG00000166439	228.0929819	158.4748518	0.526370128	0.19241939	0.299627462	RNF169
novel.848	5.278335256	0	4.734689641	0.192429906	0.299627462	-
ENSG00000018607	5.278335256	0	4.734689641	0.192429906	0.299627462	ZNF806
ENSG00000060749	994.2559249	809.8165837	0.29598303	0.192432309	0.299627462	QSER1
ENSG00000232656	32.70661125	17.8728682	0.871723766	0.192455306	0.299645311	IDI2-AS1
ENSG00000254860	144.2975453	89.42446709	0.690155147	0.19248106	0.29966034	TMEM9B-AS1
ENSG00000113595	406.6069478	330.291729	0.300201853	0.192488026	0.29966034	TRIM23
ENSG00000238160	1.230916317	4.320918095	-1.82101205	0.192589882	0.299800942	AC116366.2
ENSG00000269967	4.572471495	1.436768536	1.668358639	0.192755864	0.300041346	AL136115.2
ENSG00000256262	3.983482502	0.672939833	2.520119303	0.192801021	0.300093657	USP30-AS1
ENSG00000172379	1543.647964	1005.835335	0.618114758	0.192893898	0.300220234	ARNT2
ENSG00000112697	4310.412791	5455.978293	-0.339998319	0.192939372	0.300259425	TMEM30A
ENSG00000124731	23.93417406	11.3004738	1.08801705	0.192942192	0.300259425	TREM1
ENSG00000287644	0	2.882993683	-4.108555686	0.192970725	0.300285842	AC080112.4
ENSG00000278108	2.376156893	7.409354633	-1.635975337	0.193033146	0.300364985	MIR6757
ENSG00000198791	1220.521934	1502.009104	-0.299551062	0.193139562	0.300512575	CNOT7
ENSG00000213994	3.385646697	0.387220594	3.129477468	0.193152486	0.300514686	AL157395.1
ENSG00000215097	7.492272212	2.822786395	1.400751277	0.19320631	0.300580428	AL731733.1
ENSG00000130700	3.384393048	0.387220594	3.128986723	0.193226802	0.300594308	GATA5
ENSG00000258704	13.8569262	23.76389365	-0.775616884	0.193300291	0.300690627	SRP54-AS1
ENSG00000246366	2.3900183	6.521515334	-1.438075921	0.193336121	0.300728357	LACTB2-AS1
ENSG00000234518	159.4988576	106.5060358	0.583086664	0.193394489	0.300801139	PTGES3P1
ENSG00000227053	2.12199297	7.327793979	-1.784679186	0.193432951	0.300842951	AC105446.1
ENSG00000184785	319.9134783	216.518376	0.561994921	0.193488603	0.300911493	SMIM10
ENSG00000205361	2.417741115	0.336469917	2.646043071	0.193509967	0.300926704	MT1DP
ENSG00000133606	1613.649134	1325.97784	0.28343757	0.193700707	0.301192848	MKRN1
ENSG00000213197	4.164578442	0.713078025	2.543269853	0.193704296	0.301192848	AC012066.1
ENSG00000170234	452.8119556	345.3173753	0.391818648	0.19378809	0.301294476	PWWP2A
ENSG00000175505	254.7704637	420.7979798	-0.723684758	0.193792848	0.301294476	CLCF1

ENSG00000183597	320.8378058	256.1684942	0.324157482	0.193956468	0.301530816	TANGO2
ENSG00000272181	5.478663203	1.854670712	1.583276708	0.193977492	0.301545457	AC012557.2
ENSG00000201499	9.090751008	2.882993683	1.657661322	0.193990387	0.301546972	RNU6-312P
ENSG00000242445	1.509042109	4.809640044	-1.660323749	0.19400168	0.301546972	RPL7AP11
ENSG00000198482	108.8342078	71.93034317	0.598934226	0.194017455	0.301553452	ZNF808
ENSG00000167380	404.0933314	328.3399236	0.300304882	0.194178645	0.30178593	ZNF226
ENSG00000282855	5.106014858	0.672939833	2.88981333	0.194271148	0.301911634	AC093591.3
ENSG00000130349	225.5577528	296.723965	-0.396504616	0.194333405	0.301990323	C6orf203
ENSG00000257790	0.894837599	4.444928691	-2.313329913	0.19441164	0.302093829	EIF4A1P4
ENSG00000213149	2.668144092	7.480174406	-1.47929743	0.194424019	0.302094996	CNN2P9
ENSG00000128272	6544.100415	5260.482041	0.314972131	0.194511833	0.302213368	ATF4
ENSG00000254929	0.89358395	3.597227584	-1.997094255	0.194601124	0.302322162	AL591684.2
ENSG00000236924	3.08105174	0	3.95589051	0.194628401	0.302322162	AL162411.1
ENSG00000215475	3.08105174	0	3.95589051	0.194628401	0.302322162	SIAH3
ENSG00000200488	3.08105174	0	3.95589051	0.194628401	0.302322162	RN7SKP203
ENSG00000143258	517.0851652	428.2303916	0.272188022	0.194653895	0.302343686	USP21
novel.153	3.637303322	0.672939833	2.38699273	0.194678856	0.30236438	-
ENSG00000126653	892.1707886	745.2586678	0.259666875	0.194721897	0.302413152	NSRP1
ENSG00000187950	3.605819561	11.70087542	-1.701134647	0.194808147	0.302529018	OVCH1
ENSG00000229122	6.33191658	13.22596428	-1.054915962	0.194871264	0.302608948	AGBL5-IT1
ENSG00000286626	3.632288727	0.672939833	2.385275235	0.194936701	0.302692471	AC090469.1
ENSG00000240891	35.67608755	74.22820377	-1.055854218	0.194960909	0.302711968	PLCXD2
ENSG00000178125	7.65728418	1.682349583	2.158922029	0.195011968	0.302773152	PPP1R42
ENSG00000118680	5434.69245	6955.17863	-0.355934544	0.19508709	0.302871688	MYL12B
ENSG00000109072	17.45808717	6.717501579	1.372998231	0.195168011	0.302912785	VTN
ENSG00000253706	3.073529847	0	3.95254312	0.195171857	0.302912785	AC011632.1
novel.740	3.073529847	0	3.95254312	0.195171857	0.302912785	-
ENSG00000274624	3.073529847	0	3.95254312	0.195171857	0.302912785	AC007671.1
ENSG00000170128	3.073529847	0	3.95254312	0.195171857	0.302912785	GPR25
ENSG00000254521	2.977753644	0	3.909206869	0.195217279	0.302947086	SIGLEC12
ENSG00000228141	2.977753644	0	3.909206869	0.195217279	0.302947086	AC105339.1
ENSG00000287647	5.924023527	1.386017859	2.0801727	0.195359829	0.303144921	AC093575.1
ENSG00000276651	3.386900346	0.387220594	3.129992947	0.195368098	0.303144921	AC007950.2
ENSG00000158220	24.17518859	11.94517219	1.00970838	0.195407206	0.303187496	ESYT3
ENSG00000173482	1046.369286	1262.462455	-0.270859669	0.195426569	0.303189283	PTPRM
ENSG00000278716	0	1.823989131	-3.445121459	0.195437005	1	AC133540.1
ENSG00000177202	0	1.823989131	-3.445121459	0.195437005	1	SPACA4
ENSG00000236065	0	1.823989131	-3.445121459	0.195437005	1	AL020995.1
ENSG00000223305	0	1.823989131	-3.445121459	0.195437005	1	RN7SKP30
ENSG00000212384	0	1.823989131	-3.445121459	0.195437005	1	SNORD113-2
ENSG00000254303	0	1.823989131	-3.445121459	0.195437005	1	AC037486.1
ENSG00000233055	3.827246074	0.356539013	3.310733277	0.195445737	0.303189283	AL353621.1
ENSG00000136425	72.66088925	49.55696249	0.555047549	0.19548414	0.303189283	CIB2
ENSG00000129214	25.50786462	13.1895505	0.941292238	0.195491766	0.303189283	SHBG
ENSG00000259937	2.973992698	0	3.907486289	0.195501715	0.303189283	AC023158.1
ENSG00000162947	2.973992698	0	3.907486289	0.195501715	0.303189283	LINC01931
ENSG00000237990	2.973992698	0	3.907486289	0.195501715	0.303189283	CNTN4-AS1
ENSG00000235426	2.973992698	0	3.907486289	0.195501715	0.303189283	AL133481.1
ENSG00000273209	3.3831394	0.387220594	3.128519034	0.195593048	0.30331282	AC069148.1
ENSG00000280268	3.239510731	0	4.031308181	0.195612147	0.303314729	AC025125.1
ENSG00000154529	17.48706364	44.30528843	-1.344142083	0.195629225	0.303314729	CNTNAP3B

ENSG00000127980	340.1998648	270.4273577	0.331134881	0.195629303	0.303314729	PEX1
novel.983	5.220738334	0	4.717537462	0.195667788	0.303355497	-
ENSG00000154493	11.36278343	46.35397196	-2.028790465	0.195678949	0.303355497	C10orf90
ENSG00000280239	24.36978993	40.34612796	-0.723602	0.19577343	0.30348386	AC011498.7
ENSG00000231830	2.43410982	0.387220594	2.654144497	0.195831165	0.303555248	AC245140.1
ENSG00000183114	10.47908613	37.27275184	-1.830696022	0.195896622	0.303631517	FAM43B
ENSG00000252473	8.018293682	2.506385574	1.679025265	0.195903742	0.303631517	RF00272
novel.433	58.3001516	34.73239263	0.750893186	0.195992093	0.303750332	-
novel.165	2.431602522	0.387220594	2.652912636	0.196025007	0.303781429	-
ENSG00000224959	1.511549406	5.084746798	-1.746165258	0.196035543	0.303781429	AC017002.1
ENSG00000110911	955.9151291	1341.1552	-0.488264578	0.196100457	0.303863898	SLC11A2
ENSG00000227811	2.449224876	6.461308046	-1.405596421	0.196123991	0.303868818	INKA2-AS1
ENSG00000257438	3.65116473	0.713078025	2.349288947	0.196127024	0.303868818	AC011595.1
ENSG00000109689	625.3407797	421.4993835	0.568583403	0.196147282	0.303882083	STIM2
ENSG00000213089	7.646001342	1.782695063	2.095975904	0.196168641	0.303885515	PDCL3P5
ENSG00000198468	32.20107543	18.98737533	0.754954868	0.19617289	0.303885515	FLVCR1-DT
ENSG00000227782	15.38240829	8.254615595	0.899015391	0.196252154	0.30396315	AC002553.1
novel.802	3.575589449	0.743759607	2.294650596	0.196254568	0.30396315	-
ENSG00000268601	4.25401513	1.049547942	1.99986035	0.196258106	0.30396315	AC115522.1
ENSG00000272084	16.55662533	8.702043477	0.933606027	0.196278906	0.303977244	AL137127.1
ENSG00000049883	277.7714471	338.7795621	-0.287089197	0.196507933	0.304313799	PTCD2
ENSG00000268858	73.10123498	95.57271354	-0.387873126	0.196638837	0.304453892	AL118506.1
ENSG00000225689	3.053328925	0	3.943485821	0.19664527	0.304453892	AL008633.1
ENSG00000206633	3.053328925	0	3.943485821	0.19664527	0.304453892	SNORA80B
ENSG00000277863	3.053328925	0	3.943485821	0.19664527	0.304453892	AL162574.1
ENSG00000258604	2.142122621	0	3.431266842	0.196729009	1	AL161668.4
ENSG00000096746	2728.711569	3338.102195	-0.290914976	0.196794503	0.304666784	HNRNPH3
ENSG00000157335	2.43410982	0.356539013	2.654151611	0.196807377	0.304668561	CLEC18C
ENSG00000243477	166.8414742	226.5569585	-0.440009184	0.196868245	0.304734341	NAA80
ENSG00000006062	491.7234793	308.6590851	0.671147552	0.196886537	0.304734341	MAP3K14
ENSG00000272764	11.82318754	6.124838129	0.950768119	0.196887978	0.304734341	AL596094.1
ENSG00000010017	821.0431164	697.5363541	0.235101886	0.196896785	0.304734341	RANBP9
ENSG00000108061	792.4567352	969.3606459	-0.291025853	0.196916651	0.304746932	SHOC2
ENSG00000188599	78.01945873	51.45690847	0.598878739	0.197033544	0.304909674	NPIPP1
ENSG00000139725	23.7546165	44.54774018	-0.905518114	0.197074989	0.304955645	RHOF
ENSG00000232821	3.083559037	11.93327544	-1.956055674	0.197161146	0.305070795	AC003986.2
ENSG00000255036	2.137108026	0	3.428225064	0.197240366	1	STRA6LP
ENSG00000229409	2.137108026	0	3.428225064	0.197240366	1	AL117328.1
ENSG00000282886	2.137108026	0	3.428225064	0.197240366	1	PTMAP12
ENSG00000275672	2.137108026	0	3.428225064	0.197240366	1	AC025580.3
ENSG00000135406	2.137108026	0	3.428225064	0.197240366	1	PRPH
ENSG00000257218	310.2015425	421.3683904	-0.44254112	0.197297691	0.305263894	GATC
ENSG00000100906	2087.556585	3050.381267	-0.547067546	0.197311325	0.30526681	NFKBIA
ENSG00000256968	0.908699006	3.537020296	-1.965605193	0.197330878	0.305278882	SNRPEP2
ENSG00000172346	14.21438822	26.34353914	-0.891338561	0.197378124	0.305320311	CSDC2
ENSG00000164338	261.9916457	363.2059243	-0.471965791	0.197381161	0.305320311	UTP15
ENSG00000187144	4.514518568	1.467450118	1.639692374	0.197403895	0.305337298	SPATA21
ENSG00000018869	62.79586509	42.9779368	0.543926722	0.197504172	0.305474217	ZNF582
ENSG00000247572	126.2902146	164.696277	-0.381795911	0.197517658	0.30547689	CKMT2-AS1
ENSG00000286913	2.096777453	5.717548439	-1.442202919	0.197563656	0.305529841	AC092943.2
ENSG00000189266	2171.988032	1793.006658	0.27648381	0.197626064	0.305608164	PNRC2

ENSG00000287859	3.88645265	0.672939833	2.488373906	0.197677536	0.305669566	AC008507.4
ENSG00000107362	194.0320972	242.3068557	-0.320276664	0.197767343	0.305784679	ABHD17B
ENSG00000144339	5.230767524	22.80438267	-2.12811604	0.197775518	0.305784679	TMEFF2
novel.415	28.06261015	45.34627885	-0.695073774	0.197846147	0.305875676	-
ENSG00000237413	0	1.803920035	-3.431144777	0.197890253	1	AC096531.2
ENSG00000228016	0	1.803920035	-3.431144777	0.197890253	1	RAPGEF4-AS1
ENSG00000230183	0	1.803920035	-3.431144777	0.197890253	1	CNOT6LP1
ENSG00000154839	0	1.803920035	-3.431144777	0.197890253	1	SKA1
ENSG00000107282	33.35007695	18.58466196	0.847100745	0.197966922	0.306044186	APBA1
ENSG00000241278	11.05149296	4.381125383	1.343840278	0.19803656	0.306121796	ENPP7P4
ENSG00000235397	0.879722543	4.280779903	-2.265873773	0.198040689	0.306121796	EPN2-AS1
ENSG00000112584	40.07853213	17.71334292	1.178544728	0.198061773	0.306136173	FAM120B
ENSG00000099256	202.5730074	164.4404686	0.301468628	0.198080399	0.306146749	PRTFDC1
ENSG00000273682	4.148209737	0.672939833	2.586072278	0.198262438	0.306409875	AC109583.2
ENSG00000234902	1.481319294	5.553399651	-1.889262005	0.198302007	0.306452798	AC007879.3
ENSG00000176054	14.17914351	7.46010531	0.92225423	0.198333787	0.30648368	RPL23P2
ENSG00000115274	15.14579545	8.35611695	0.86420495	0.198384258	0.306543441	INO80B
ENSG00000226124	21.96751987	11.98531038	0.865871393	0.198403573	0.306555053	FTCDNL1
ENSG00000224424	20.60056805	12.57553369	0.712441182	0.198424599	0.30656931	PRKAR2A-AS1
ENSG00000122484	319.3390777	394.3756922	-0.304756342	0.198444205	0.306570128	RPAP2
ENSG00000233926	3.605819561	0.713078025	2.333803241	0.198448728	0.306570128	AL591368.1
ENSG00000258870	0.586481695	3.32212083	-2.473976694	0.198648689	0.306860788	EIF4EBP1P1
ENSG00000135457	768.9594025	637.1620832	0.270820537	0.19870241	0.306925524	TFCP2
ENSG00000164654	587.3952991	454.9694177	0.368117446	0.198735976	0.306959122	MIOS
ENSG00000266998	0.909952655	3.993904788	-2.132578343	0.198823673	0.30707632	AC111182.1
ENSG00000169330	40.40576438	61.77017779	-0.61114437	0.198850625	0.30709969	MINAR1
ENSG00000253746	3.699017196	12.57424942	-1.766723297	0.199114722	0.307489277	AC091182.2
ENSG00000279598	0.308355904	2.944356846	-3.173785093	0.199188641	0.307585145	AC009948.4
ENSG00000246334	8.218621629	16.80196689	-1.029837614	0.199403111	0.307898029	PRR7-AS1
ENSG00000205111	5.732827126	1.702418679	1.721111576	0.199577614	0.308149165	CDKL4
ENSG00000084628	4.614055717	13.7559803	-1.575947364	0.199828274	0.30851785	NKAIN1
ENSG00000199550	2.403879708	0.336469917	2.639116661	0.199858533	0.308546233	RF00019
ENSG00000260509	4.285498891	0.713078025	2.580706249	0.199890925	0.308577904	AL590787.1
ENSG00000135535	4401.773935	5463.561175	-0.311772747	0.200125021	0.308905009	CD164
novel.694	0.308355904	3.036401591	-3.214034459	0.200126597	0.308905009	-
ENSG00000182551	594.1810783	458.8839452	0.372206768	0.200153893	0.308928788	ADI1
ENSG00000233901	104.3970163	66.01613215	0.657882878	0.200178793	0.308948866	LINC01503
ENSG00000260388	6.404984563	2.058957692	1.610096381	0.200251148	0.309042178	LINC00562
ENSG00000128159	1471.952855	1157.138536	0.347321132	0.20027389	0.309049103	TUBGCP6
ENSG00000163961	287.8834415	353.8298195	-0.29814061	0.200279425	0.309049103	RNF168
ENSG00000100387	1336.093689	1609.211967	-0.26840828	0.200303547	0.309067969	RBX1
ENSG00000283031	8.991213859	3.251301057	1.472529698	0.200359292	0.309135624	AC009242.1
ENSG00000234367	1.539272221	5.430673325	-1.825623101	0.200375054	0.309141585	PFN1P3
ENSG00000288045	16.62335379	8.35740122	0.980005534	0.200426813	0.309203078	AL352979.4
ENSG00000272175	0	2.618499415	-3.965921447	0.200456	1	AL050343.2
ENSG00000243574	0	2.618499415	-3.965921447	0.200456	1	AC006148.2
ENSG00000264006	0	2.618499415	-3.965921447	0.200456	1	AKR1C8P
ENSG00000287872	0	2.618499415	-3.965921447	0.200456	1	AC026956.2
ENSG00000131737	0	2.618499415	-3.965921447	0.200456	1	KRT34
ENSG00000134758	430.7138696	350.4554069	0.297005851	0.200553683	0.309380432	RNF138
ENSG00000244018	0.293240848	3.036401591	-3.214053486	0.200578534	0.309400397	RPL35P6

ENSG00000106591	790.631502	1007.04773	-0.349355381	0.200651556	0.309494661	MRPL32
ENSG00000135972	219.0062762	281.7991309	-0.364542948	0.200667893	0.309501486	MRPS9
ENSG00000143393	1906.267371	1585.472065	0.265969642	0.200702947	0.30952468	PI4KB
ENSG00000163682	5268.584493	6976.895476	-0.405117212	0.200706758	0.30952468	RPL9
ENSG00000051523	3881.833145	5293.788371	-0.447535907	0.20073474	0.309549458	CYBA
ENSG00000153292	1.843867178	8.986478447	-2.285791125	0.200910619	0.30980229	ADGRF1
ENSG00000229036	59.13564008	38.75864845	0.605513825	0.201008819	0.309935319	VDAC1P8
ENSG00000283162	0.925067711	6.275934287	-2.766734559	0.201103318	0.310046112	AL390726.5
ENSG00000225778	8.153075539	3.546476906	1.194386731	0.201104541	0.310046112	PROSER2-AS1
ENSG00000114648	368.118233	447.824125	-0.283244165	0.201166201	0.310122037	KLHL18
ENSG00000226471	15.4503904	25.60897935	-0.7239191	0.201188834	0.310122037	Z93930.2
ENSG00000155096	2971.142771	2418.952359	0.296686243	0.201192838	0.310122037	AZIN1
ENSG00000237422	6.553343093	1.813376645	1.860480112	0.201209357	0.310122037	AL158071.4
ENSG00000218823	6.449076083	1.722487775	1.881681601	0.20121347	0.310122037	PAPOLB
ENSG00000162997	31.56659873	20.36149643	0.629943382	0.201228918	0.310127448	PRORSD1P
ENSG00000110237	2747.263717	2144.547748	0.357225054	0.201252359	0.310129096	ARHGEF17
ENSG00000258427	5.774411348	11.81286086	-1.034896448	0.201253861	0.310129096	RBM8B
ENSG00000235105	17.28164982	26.4049023	-0.614372955	0.201278229	0.310148251	AL356968.2
ENSG00000225968	16.35908941	34.49138791	-1.072283007	0.201398929	0.310315832	ELFN1
ENSG00000162522	128.8332775	197.2180547	-0.614573642	0.201422469	0.3103337	KIAA1522
novel.96	3.896553111	0.713078025	2.446202798	0.201477693	0.310400376	-
ENSG00000089289	672.3295492	862.0875586	-0.358468968	0.201527862	0.310459257	IGBP1
ENSG00000160570	392.9151045	545.2750678	-0.472335084	0.201586405	0.310531032	DEDD2
ENSG00000121236	45.57761007	74.48765499	-0.710637958	0.201608194	0.310546184	TRIM6
ENSG00000103381	610.8774441	757.7665926	-0.310786159	0.201652853	0.31059656	CPPED1
ENSG00000157654	0	1.773238453	-3.409972071	0.201695747	1	PALM2-AKAP2
ENSG00000206603	0	1.773238453	-3.409972071	0.201695747	1	SNORA22B
ENSG00000258445	0	1.773238453	-3.409972071	0.201695747	1	AL132777.1
ENSG00000188263	0	1.773238453	-3.409972071	0.201695747	1	IL17REL
ENSG00000198826	0	1.773238453	-3.409972071	0.201695747	1	ARHGAP11A
ENSG00000249942	0	1.773238453	-3.409972071	0.201695747	1	AC239584.1
ENSG00000249464	12.81373007	5.54522731	1.192872953	0.201760708	0.310744262	LINC01091
ENSG00000225684	15.04152844	6.481377142	1.213736204	0.201818483	0.31081482	FAM225B
ENSG00000179277	124.2574014	81.80098368	0.603592758	0.201844624	0.310836653	MEIS3P1
ENSG00000284862	18.01147545	9.234499639	0.961678588	0.201894928	0.310895693	CCDC39
ENSG00000149577	835.397299	1094.31804	-0.38944924	0.201959926	0.31097735	SIDT2
ENSG00000255093	0	1.854670712	-3.466212031	0.201979709	1	AP002008.2
ENSG00000207200	0	1.854670712	-3.466212031	0.201979709	1	RNU6-45P
ENSG00000261780	0	1.854670712	-3.466212031	0.201979709	1	LINC02582
ENSG00000186940	3.676380247	8.846123169	-1.275954816	0.201990251	0.311005613	CHCHD2P9
ENSG00000262904	0.601596751	3.2194636	-2.416794091	0.202022984	0.311037579	TMPOP2
ENSG00000277072	150.1347385	196.7734993	-0.39054363	0.202045337	0.311053562	STAG3L2
ENSG00000027075	245.9833071	344.0663657	-0.484161411	0.202071779	0.311075837	PRKCH
ENSG00000273802	10.11034127	20.46171352	-1.017181927	0.202085182	0.311078038	HIST1H2BG
ENSG00000249264	5.439586279	10.71140637	-0.978479039	0.202236664	0.311292777	EEF1A1P9
ENSG00000286670	3.649911081	0.774441189	2.289694578	0.202355906	0.311457868	AL450467.1
ENSG00000205356	863.2084833	703.0259508	0.296460162	0.2024399	0.311568689	TECPR1
ENSG00000134569	754.9500283	489.5652697	0.625343588	0.202458478	0.311578825	LRP4
ENSG00000065029	734.5400322	585.6135026	0.327404237	0.202545532	0.311694335	ZNF76
ENSG00000181666	617.7883219	501.0742469	0.302431043	0.202576185	0.311723044	ZNF875
ENSG00000207205	5.121129914	0.713078025	2.840300209	0.202645758	0.311811634	RNVU1-15

ENSG00000285925	0	2.649180997	-3.981725172	0.202652848	1	AC007391.2
ENSG00000226819	0	2.649180997	-3.981725172	0.202652848	1	MEIS1-AS3
ENSG00000279694	0	2.649180997	-3.981725172	0.202652848	1	AC011270.2
ENSG00000082458	440.8865105	324.2528182	0.443698476	0.202805788	0.312039393	DLG3
ENSG00000230424	17.99030562	9.539132099	0.907195997	0.202845758	0.312082408	EMC1-AS1
ENSG00000274717	0.601596751	4.057708096	-2.758693916	0.203046422	0.312372637	AL049757.1
ENSG00000226005	2.081662396	0	3.39404229	0.203048944	1	LINC02660
ENSG00000254932	2.081662396	0	3.39404229	0.203048944	1	AP001007.1
ENSG00000234943	2.081662396	0	3.39404229	0.203048944	1	AC092839.2
ENSG00000260306	2.081662396	0	3.39404229	0.203048944	1	AC092375.2
ENSG00000271064	1.541779519	7.478890137	-2.280248781	0.203088134	0.312418309	AC027644.2
ENSG00000114626	1166.259175	845.1980296	0.464923028	0.203183963	0.31254722	ABTB1
ENSG00000286095	0	2.587817834	-3.95008256	0.203189853	1	SPEGNB
ENSG00000255965	0	2.587817834	-3.95008256	0.203189853	1	AC073916.1
ENSG00000236559	0	2.587817834	-3.95008256	0.203189853	1	AL117381.1
ENSG00000267346	0	2.587817834	-3.95008256	0.203189853	1	EIF5AP3
ENSG00000103121	507.8963167	386.8006608	0.392839141	0.203224263	0.312590704	CMC2
ENSG00000246982	22.02518807	13.53419276	0.699868996	0.203281635	0.31266044	Z84485.1
ENSG00000273297	10.95098689	18.5375073	-0.763798375	0.203348202	0.312730309	AC009275.1
ENSG00000147465	3.871337594	10.84474518	-1.479458828	0.203351135	0.312730309	STAR
ENSG00000287403	0.308355904	2.211209725	-2.757092247	0.203398828	1	AC016245.2
ENSG00000248559	2.157237677	7.784806866	-1.86005525	0.2034165	0.312812317	AC109454.2
ENSG00000160868	4.849343637	1.161661783	2.105688645	0.203441122	0.312822105	CYP3A4
ENSG00000277067	8.826486624	3.862877727	1.178334356	0.203468081	0.312822105	CU634019.1
ENSG00000267522	2.962638588	0	3.902202942	0.203471027	0.312822105	LINC01864
ENSG00000277591	2.962638588	0	3.902202942	0.203471027	0.312822105	RN7SL575P
ENSG00000250742	242.730647	378.0018891	-0.639099867	0.203517939	0.312875715	LINC02381
ENSG00000285943	14.46360882	7.968896356	0.866254565	0.203535447	0.312884115	AC112128.1
ENSG00000271869	12.59703342	5.239438975	1.256826316	0.203597687	0.312961278	AC026979.2
novel.647	69.42721984	104.5027563	-0.590835529	0.20362166	0.312979609	-
ENSG00000087245	7585.478853	16588.90372	-1.128909293	0.203642472	0.312993082	MMP2
ENSG00000224414	2.96013129	0	3.901048486	0.203664658	0.312999277	AC010886.1
novel.440	101.9549574	156.644441	-0.619284427	0.203670597	0.312999277	-
ENSG00000213963	62.0016761	43.27756058	0.522598256	0.203689989	0.313010564	AC019080.1
ENSG00000252965	7.79561352	3.220619475	1.276432349	0.203878085	0.313281082	RF00019
ENSG00000163352	0.293240848	2.211209725	-2.757114249	0.203937361	1	LENEP
ENSG00000151651	134.9631855	81.95742685	0.722184337	0.204094949	0.31359207	ADAM8
ENSG00000100744	268.0787002	373.6207422	-0.479725008	0.204104611	0.31359207	GSKIP
ENSG00000044115	8232.394202	6916.410503	0.251263361	0.204150493	0.313644016	CTNNA1
novel.931	37.36601213	55.84248178	-0.576620668	0.204168003	0.31365237	-
ENSG00000187559	3.346569772	0.336469917	3.114073603	0.204205166	0.313672999	FOXD4L3
ENSG00000134248	1298.213535	1519.758732	-0.227304517	0.204205577	0.313672999	LAMTOR5
ENSG00000198171	941.1287888	1185.995644	-0.333869808	0.204376682	0.313917268	DDRGK1
ENSG00000131591	289.1871132	387.0487976	-0.419856095	0.204473768	0.314025298	C1orf159
ENSG00000135540	374.0925168	229.2468509	0.705628656	0.204479929	0.314025298	NHSL1
ENSG00000138764	1124.676442	792.0520459	0.506200513	0.204483276	0.314025298	CCNG2
ENSG00000173762	88.43775611	52.75905392	0.743788813	0.204560275	0.314124978	CD7
ENSG00000287757	8.608892329	2.149846562	1.999122101	0.204579934	0.314136599	AC244636.3
ENSG00000245937	469.1771975	359.6656463	0.382796343	0.204606098	0.314158208	LINC01184
novel.55	6.539481686	2.10970837	1.624681149	0.204632474	0.314180138	-
ENSG00000258458	7.5603256	3.159256312	1.245400921	0.204712756	0.314284826	AL160314.2

ENSG00000122687	4.290584757	10.02785405	-1.230351731	0.20476395	0.314344847	MRM2
ENSG00000245711	8.976098803	3.32212083	1.448781765	0.204802754	0.31438584	NADK2-AS1
ENSG00000273308	2.402626059	6.614844348	-1.465504817	0.204869138	0.314460334	AC024560.3
ENSG00000160799	715.9897137	587.4410534	0.285579849	0.204875489	0.314460334	CCDC12
ENSG00000102125	731.0506601	576.5868048	0.342866348	0.204940957	0.314542238	TAZ
ENSG00000142252	33.39040753	49.17218204	-0.557333167	0.205027448	0.314656396	GEMIN7
ENSG00000172661	1076.762921	892.8182243	0.270093994	0.205086071	0.314686326	WASHC2C
ENSG00000101193	898.5930666	1055.561622	-0.232221096	0.205095023	0.314686326	GID8
ENSG00000278200	0.616711807	3.189937893	-2.386406164	0.205095399	0.314686326	LINC01971
ENSG00000104044	0.616711807	3.189937893	-2.386406164	0.205095399	0.314686326	OCA2
ENSG00000183475	313.3837129	252.411005	0.313283192	0.205129372	0.314719866	ASB7
ENSG00000244257	6.492882868	1.467450118	2.158335383	0.205191825	0.314797095	PKD1P1
ENSG00000233122	13.9363337	7.847325905	0.824787786	0.205278363	0.314889382	CTAGE7P
ENSG00000109133	1029.880842	1246.142943	-0.275230941	0.205279987	0.314889382	TMEM33
ENSG00000166199	230.1173314	287.0111331	-0.319410323	0.205288339	0.314889382	ALKBH3
ENSG00000010626	188.4455485	146.9317167	0.357342755	0.2053589	0.314979018	LRRC23
ENSG00000103343	166.9100971	129.3904381	0.366262245	0.205543401	0.315243394	ZNF174
ENSG00000164402	1020.766978	1498.061335	-0.553454433	0.205626154	0.315351697	SEPT8
ENSG00000163399	4370.722096	6121.954936	-0.486144327	0.205659151	0.315383686	ATP1A1
ENSG00000198843	2144.277041	1754.951341	0.28891514	0.205810683	0.315597436	SELENOT
ENSG00000138685	2013.210516	2687.942033	-0.41687773	0.20584033	0.315624269	FGF2
ENSG00000286561	0	2.558292127	-3.934382644	0.205895787	1	AC011092.3
ENSG00000264201	0	2.558292127	-3.934382644	0.205895787	1	MIR4701
ENSG00000160472	0	2.558292127	-3.934382644	0.205895787	1	TMEM190
ENSG00000227477	2.697120555	6.796622087	-1.329159491	0.205922107	0.315731027	STK4-AS1
ENSG00000141579	2.72484337	7.142548615	-1.392004797	0.205972254	0.315789279	ZNF750
ENSG00000196576	6654.901225	8335.97649	-0.324920226	0.206067173	0.315916164	PLXNB2
ENSG00000164291	377.7793817	526.9475703	-0.480672217	0.206101729	0.315950497	ARSK
ENSG00000133030	6154.62113	7878.212486	-0.356162428	0.20612913	0.315961488	MPRIP
ENSG00000064886	13.41532682	35.87612149	-1.417409132	0.206134841	0.315961488	CHI3L2
ENSG00000115808	714.251937	578.6838794	0.303808107	0.206152473	0.315961488	STRN
ENSG00000169814	306.2105378	373.5809767	-0.286615471	0.206157544	0.315961488	BTD
ENSG00000260648	0	2.609042805	-3.960949047	0.206204633	1	AC020658.3
ENSG00000169599	472.8773981	568.9353836	-0.266875899	0.206258486	0.316086207	NFU1
ENSG00000259376	7.553986085	2.292641984	1.748172981	0.206263252	0.316086207	AC090907.1
ENSG00000124786	618.4581142	511.7216401	0.273147675	0.206276873	0.316088437	SLC35B3
ENSG00000148110	1435.121236	1756.200457	-0.291478139	0.206456608	0.316345196	MFSD14B
ENSG00000160785	428.9252773	520.3518835	-0.279107316	0.206602776	0.316550494	SLC25A44
ENSG00000019144	4227.924263	3177.718278	0.41191343	0.206701083	0.316682442	PHLDB1
ENSG00000188626	3.671365652	0.356539013	3.247316849	0.206837693	0.316873053	GOLGA8M
ENSG00000227440	1.801029308	5.932447905	-1.721887027	0.206853398	0.316878428	ATP5MC1P4
ENSG00000270647	0.907445357	3.546476906	-1.969707194	0.206906127	0.316940515	TAF15
ENSG00000286518	0.293240848	2.844011366	-3.128530859	0.206973644	0.317025247	AL354743.1
ENSG00000079999	1362.605043	1633.419017	-0.26165492	0.206987431	0.317027675	KEAP1
ENSG00000285294	11.68492947	3.149799701	1.883150698	0.207025459	0.317067228	LINC00842
ENSG00000146842	304.457005	382.525356	-0.330159311	0.20750486	0.317782715	TMEM209
ENSG00000224383	9.780530799	3.465044647	1.480937378	0.2075209	0.317785411	PRR29
ENSG00000122432	23.37451754	14.02407059	0.73742301	0.207531083	0.317785411	SPATA1
ENSG00000263982	3.270994492	0.387220594	3.083733398	0.20763699	0.317928844	AC009716.1
ENSG00000146540	2232.208995	1688.024384	0.40295178	0.207665971	0.317952818	C7orf50
ENSG00000228532	142.7383138	191.9535039	-0.426461	0.207677123	0.317952818	AC005000.1

ENSG00000088451	257.677811	310.3245168	-0.267739138	0.207873173	0.318234218	TGDS
ENSG00000224999	2.446717578	0.336469917	2.660570409	0.208027821	0.318452204	VTA1P1
ENSG00000188554	2487.772787	3136.436238	-0.334203344	0.208104671	0.31855037	NBR1
ENSG00000156587	1551.385912	2032.138287	-0.389455487	0.208116469	0.31855037	UBE2L6
ENSG00000269053	7.380483309	1.518200796	2.301404277	0.208136839	0.318562781	AC010319.3
ENSG00000223722	9.185344834	1.722487775	2.401084038	0.208452111	0.319026526	AC023157.1
ENSG00000179029	256.6070346	327.2191707	-0.350008886	0.208476522	0.319045092	TMEM107
ENSG00000243414	0.616711807	4.493239223	-2.869313132	0.208666104	0.319316413	TICAM2
ENSG00000265917	17.17709808	9.141299019	0.902114977	0.208758147	0.319433443	MIR3685
ENSG00000108509	796.65948	1008.749678	-0.340185269	0.208769673	0.319433443	CAMTA2
ENSG00000236896	0.308355904	2.913675265	-3.160348973	0.208779466	0.319433443	AL354726.1
ENSG00000101160	4624.591083	3503.687089	0.400494126	0.208923246	0.319634605	CTSZ
ENSG00000002549	1662.41978	2267.877607	-0.448128993	0.208953524	0.319662104	LAP3
ENSG00000166105	4.933694459	0	4.635815965	0.208968285	0.319665862	GLB1L3
ENSG00000111676	2614.11713	3147.458504	-0.26785283	0.209011566	0.319712628	ATN1
ENSG00000089234	412.0343255	485.6737907	-0.237095057	0.209023467	0.319712628	BRAP
ENSG00000078668	1719.451896	2092.260045	-0.2832722	0.209038942	0.319717475	VDAC3
ENSG00000232004	28.50958013	43.6977745	-0.613436276	0.209059957	0.319730795	CAP1P2
ENSG00000168958	1228.436589	1470.518954	-0.259694735	0.209101494	0.319775496	MFF
ENSG00000260634	3.561728042	0.743759607	2.288544901	0.209225741	0.319946673	AC012508.2
ENSG00000276141	92.76670613	129.2066869	-0.476177232	0.209245047	0.319957364	WHAMMP3
ENSG00000232775	3.082305388	0	3.956536926	0.209308838	0.320036072	BMS1P22
ENSG00000199545	5.377872405	1.4981317	1.870622134	0.209362614	0.320099457	RNA5SP195
ENSG00000123870	43.74731956	30.18390762	0.534942714	0.209502292	0.320280634	ZNF137P
ENSG00000226210	101.607212	39.10714352	1.376066626	0.209505769	0.320280634	WASH8P
ENSG00000199477	9.652017186	3.72941052	1.388971953	0.209522303	0.320287064	SNORA31
ENSG00000228317	0	1.793307549	-3.424100018	0.209543959	1	AL158070.1
ENSG00000259155	0	1.793307549	-3.424100018	0.209543959	1	AL591767.3
ENSG00000171872	0.308355904	2.772035718	-3.096051589	0.209588455	0.320369338	KLF17
ENSG00000260197	32.45599446	7.85922266	2.041108529	0.209659746	0.320459458	AC010889.1
ENSG00000070413	1466.909562	1721.674652	-0.230899963	0.209741605	0.320565718	DGCR2
ENSG00000106236	207.2847222	105.4004374	0.97513328	0.209760868	0.3205763	NPTX2
ENSG00000230257	1.521649867	7.591003978	-2.31516309	0.209825497	0.320656211	NFE4
ENSG00000215630	9.163534258	2.842855491	1.687310989	0.209876906	0.32071591	GUSBP9
ENSG00000276282	3.943151929	1.10029862	1.856207863	0.209897468	0.320728466	AC022960.2
ENSG00000133835	1441.2253	1710.471231	-0.247224417	0.209928455	0.32075695	HSD17B4
ENSG00000183648	840.1121911	705.6014051	0.251895902	0.209953098	0.320775739	NDUFB1
ENSG00000223804	148.484575	110.2000601	0.430264339	0.210045914	0.320898677	AC244669.1
ENSG00000223959	389.9481953	262.4994174	0.571153769	0.210094747	0.320954409	AFG3L1P
ENSG00000108256	1806.353302	2109.440961	-0.223774206	0.210275797	0.321212105	NUFIP2
ENSG00000280515	5.450940389	1.345879667	1.983091423	0.21030541	0.321214014	SALRNA2
ENSG00000269304	0.308355904	2.792104814	-3.105294128	0.210325208	0.321214014	FKBP1AP1
ENSG00000077942	1840.18665	4390.14186	-1.254447181	0.210325705	0.321214014	FBLN1
ENSG00000186766	3.068443981	0	3.950326602	0.210338864	0.321214014	FOXI2
ENSG00000188120	3.068443981	0	3.950326602	0.210338864	0.321214014	DAZ1
ENSG00000268081	2.416487466	0.336469917	2.645372585	0.210555151	0.321506517	AC123912.1
ENSG00000158482	2.416487466	0.336469917	2.645372585	0.210555151	0.321506517	SNX29P1
ENSG00000219653	0	2.50754145	-3.907452209	0.210630655	1	GCNT1P4
ENSG00000149308	258.3378589	319.5510537	-0.306934399	0.210662097	0.321650913	NPAT
ENSG00000172977	296.5772262	240.8242168	0.300463416	0.210775783	0.321805583	KAT5
ENSG00000273305	9.933975195	4.596024848	1.10522352	0.210802237	0.321827061	AC009237.15

ENSG00000276968	2.405133356	0.356539013	2.639630071	0.210854378	0.321887749	AL158196.1
ENSG00000261442	0	2.557136252	-3.934119785	0.210869201	1	AC023830.1
ENSG00000271779	0	2.557136252	-3.934119785	0.210869201	1	AC009994.1
ENSG00000287352	0	2.557136252	-3.934119785	0.210869201	1	AC011029.1
ENSG00000057149	0	2.557136252	-3.934119785	0.210869201	1	SERPINB3
ENSG00000234043	11.84206354	6.206270388	0.940844991	0.210907356	0.321949448	NUDT9P1
ENSG00000285577	0.308355904	2.129777466	-2.713328881	0.21090869	1	AC019127.1
ENSG00000278472	0.308355904	3.048298346	-3.234874827	0.210926114	0.321949448	AC009268.2
ENSG00000279765	13.31077508	7.286499912	0.87785609	0.21093197	0.321949448	AC013394.1
ENSG00000230583	5.790780053	1.762625967	1.703766038	0.210945317	0.321950906	GTF2IRD1P1
ENSG00000103061	103.6946288	140.9063249	-0.44390473	0.21096108	0.321956052	SLC7A6OS
ENSG00000104804	10.23097699	5.095359283	1.015668436	0.210992012	0.321984345	TULP2
ENSG00000226688	44.53001225	31.03045286	0.518434334	0.21101756	0.322004419	ENTPD1-AS1
ENSG00000006432	19.82288996	34.54753245	-0.805839461	0.211102049	0.322114427	MAP3K9
ENSG00000161249	23.26885434	44.74513909	-0.944383205	0.211119102	0.322121528	DMKN
ENSG00000178150	13.4745334	5.879257082	1.203242253	0.21114149	0.322123057	ZNF114
ENSG00000272277	24.65550888	14.90014152	0.723533826	0.211176565	0.322123057	AL031963.3
ENSG00000286048	1.17296339	5.146109961	-2.114131913	0.211178241	0.322123057	AC008966.3
ENSG00000258998	3.057161142	0	3.945264829	0.211182096	0.322123057	LINC02302
ENSG00000258342	3.057161142	0	3.945264829	0.211182096	0.322123057	AL133304.3
ENSG00000174106	561.9499869	451.8391604	0.314296283	0.211337449	0.322325149	LEMD3
ENSG00000101442	214.9920654	161.372662	0.41303481	0.211339398	0.322325149	ACTR5
ENSG00000261408	29.27841141	17.83491337	0.72077299	0.211377516	0.32236436	TEN1-CDK3
ENSG00000269210	16.93826268	4.881744087	1.787802808	0.211441375	0.322410739	AC019171.1
ENSG00000124614	24.62814207	37.16590347	-0.596187311	0.211442785	0.322410739	RPS10
ENSG00000283378	2.403879708	7.999706332	-1.736961567	0.211445156	0.322410739	CNTNAP3C
ENSG00000261143	4.927426216	0.672939833	2.837354118	0.211462376	0.322418075	ADAMTS7P3
ENSG00000228280	2.405133356	0.336469917	2.639623974	0.211517556	0.322447892	AL731568.1
ENSG00000269392	2.405133356	0.336469917	2.639623974	0.211517556	0.322447892	AC008655.2
ENSG00000162408	463.727128	591.803826	-0.352157921	0.211519165	0.322447892	NOL9
ENSG00000120699	555.6134987	712.1786923	-0.358559251	0.211583468	0.322526994	EXOSC8
ENSG00000255893	9.701194572	4.616093944	1.064667174	0.211627408	0.322575048	AP000786.1
ENSG00000254459	4.735945081	1.060160428	2.154575767	0.211644217	0.322581743	AP002812.2
ENSG00000273524	3.35040199	0.387220594	3.115641061	0.211675215	0.322610064	RF00017
ENSG00000126267	1752.765445	2114.662974	-0.270769954	0.211708207	0.322641419	COX6B1
ENSG00000132330	34.71896661	59.16619085	-0.769346756	0.211824221	0.322793562	SCLY
ENSG00000054793	619.7426208	856.9752586	-0.467829708	0.211832887	0.322793562	ATP9A
ENSG00000151327	1291.931579	1053.706578	0.293732343	0.212254294	0.323416739	FAM177A1
ENSG00000177432	287.9168467	184.8102314	0.638473388	0.212330418	0.323513758	NAP1L5
ENSG00000253175	3.591958154	0.713078025	2.329086924	0.212501902	0.32375605	AC023632.1
ENSG00000182329	5.200537412	1.456837632	1.841668293	0.21257616	0.323850194	KIAA2012
ENSG00000164535	505.6919657	641.7977542	-0.343360489	0.212603077	0.323872211	DAGLB
ENSG00000236679	6.627664725	2.069570178	1.660133823	0.212627341	0.32387792	RPL23AP24
ENSG00000169612	131.3763133	171.0332361	-0.381853908	0.212631757	0.32387792	RAMAC
ENSG00000247092	108.8069832	139.660534	-0.361200082	0.21265517	0.323894593	SNHG10
ENSG00000024862	319.2455797	240.1212377	0.411025906	0.212671802	0.323900938	CCDC28A
ENSG00000039600	6.757431987	2.089639274	1.675930088	0.212727208	0.32396633	SOX30
ENSG00000176273	104.7399328	163.5751854	-0.644637254	0.212760421	0.323997919	SLC35G1
ENSG00000139219	3.284855899	0.693008929	2.220968116	0.212914869	0.324214114	COL2A1
ENSG00000279255	7.128470678	1.456837632	2.297543671	0.213023999	0.32436128	Z97653.2
ENSG00000003137	118.0841573	60.05663305	0.977374191	0.213244945	0.324678676	CYP26B1

ENSG00000134265	577.7439939	706.7918216	-0.291025231	0.213344255	0.324810846	NAPG
ENSG00000113430	0.308355904	2.751966622	-3.086621499	0.213437101	0.324933161	IRX4
ENSG00000151292	731.0254286	624.4309454	0.227525954	0.213480554	0.324963323	CSNK1G3
ENSG00000279144	0.307102255	2.751966622	-3.086619893	0.213481929	0.324963323	AC099667.1
ENSG00000287728	3.672619301	0.693008929	2.37967844	0.21354126	0.324965716	AL360015.1
ENSG00000274443	2.770188538	0	3.802404773	0.21354604	0.324965716	C8orf89
ENSG00000216863	2.770188538	0	3.802404773	0.21354604	0.324965716	LY86-AS1
ENSG00000234471	2.770188538	0	3.802404773	0.21354604	0.324965716	AC147651.3
ENSG00000224137	2.770188538	0	3.802404773	0.21354604	0.324965716	LINC01857
novel.310	0	2.679862578	-3.997671586	0.213581524	1	-
ENSG00000232456	0	2.679862578	-3.997671586	0.213581524	1	AL355994.2
ENSG00000224629	1.847628125	5.748230021	-1.64549252	0.213635488	0.325082793	AC004975.1
ENSG00000244471	2.76893489	0	3.801788318	0.213654199	0.325092225	AL137058.1
ENSG00000105993	2264.422776	2794.330599	-0.303280538	0.213697936	0.325134359	DNAJB6
ENSG00000284649	4.282991593	1.10029862	1.972028261	0.21372131	0.325134359	AC009093.8
ENSG00000213079	700.7866495	569.0987145	0.30036104	0.213722482	0.325134359	SCAF8
ENSG00000236397	46.40328317	27.58650479	0.751748045	0.213731947	0.325134359	DDX11L2
ENSG00000241549	8.090108017	3.280826763	1.312323859	0.213774715	0.325180378	GUSBP2
ENSG00000201151	4.851850935	1.4981317	1.722637842	0.213788271	0.32518196	RF00275
ENSG00000131844	596.2778714	716.473026	-0.265062589	0.213885509	0.325310819	MCCC2
novel.1072	17.6199511	8.448290089	1.055957681	0.214089694	0.325602314	-
ENSG00000157212	193.6825134	246.3971248	-0.346326172	0.214130201	0.325644858	PAXIP1
ENSG00000252680	3.534005227	0.387220594	3.19585884	0.21423562	0.325786107	RNA5SP449
ENSG00000164169	158.8901228	195.5278367	-0.300042714	0.21428973	0.325849319	PRMT9
ENSG00000116205	357.1784142	290.4357356	0.298240612	0.214407534	0.326009373	TCEANC2
ENSG00000038295	11.60043611	4.097846288	1.483242825	0.214456848	0.326065272	TLL1
ENSG00000261684	2.449224876	0.356539013	2.661912566	0.214556112	0.326197106	AC018362.1
ENSG00000168658	6.929752385	2.415496705	1.500299322	0.214578602	0.32621221	VWA3B
ENSG00000095539	109.3023042	157.9690148	-0.530158634	0.214667982	0.326328994	SEMA4G
ENSG00000227382	0	1.753169357	-3.395474556	0.214772337	1	EIF4A2P2
ENSG00000259514	0	1.753169357	-3.395474556	0.214772337	1	AC027243.1
ENSG00000228251	7.609431715	2.862924587	1.407021985	0.21477536	0.326473124	AC012442.1
ENSG00000225131	7.868681504	13.88011929	-0.821897753	0.214801909	0.326494377	PSME2P2
ENSG00000180209	6.121844177	1.834601616	1.748556189	0.21487368	0.326558365	MYLPF
novel.547	191.006677	113.5786294	0.748380089	0.214876699	0.326558365	-
ENSG00000276408	3.983482502	1.080229524	1.881978538	0.214881714	0.326558365	AC025287.2
ENSG00000186564	247.4649383	103.0740314	1.262663721	0.214954413	0.32664974	FOXD2
ENSG00000137221	848.6311916	691.0033616	0.296770606	0.215059651	0.326790548	TJAP1
ENSG00000285280	8.428694033	3.668047357	1.213387338	0.215229954	0.327022791	AL390957.1
ENSG00000033800	661.6698365	516.7991948	0.356317276	0.215237663	0.327022791	PIAS1
ENSG00000125149	277.6242431	343.981817	-0.308911936	0.215257556	0.327033891	C16orf70
ENSG00000222345	5.215652468	11.6274871	-1.158686818	0.215366292	0.327179957	SNORD19C
ENSG00000168000	34.99382965	53.52848643	-0.611839374	0.215400673	0.327213055	BSCL2
ENSG00000137880	7.278438862	2.782648203	1.369510029	0.215425375	0.327230727	GCHFR
ENSG00000204790	3.034452922	0.723690511	2.07464752	0.215437496	0.327230727	AL163540.1
ENSG00000213339	1051.384041	847.1258225	0.311505126	0.215480968	0.327262831	QTRT1
ENSG00000287601	1.510295757	4.799027558	-1.658427959	0.215483824	0.327262831	BX323046.2
ENSG00000149346	176.5018678	137.3395221	0.360147045	0.215509601	0.327282847	SLX4IP
ENSG00000155329	259.185813	199.2424436	0.377936055	0.215572897	0.327359229	ZCCHC10
ENSG00000126249	67.22603284	92.12329006	-0.45682367	0.21559339	0.327359229	PDCD2L
ENSG00000228763	2.06654734	7.193299292	-1.78969544	0.215597697	0.327359229	LIMS1-AS1

ENSG00000145423	42.54684647	223.2367767	-2.391701608	0.215642491	0.327408109	SFRP2
ENSG00000166143	3.534005227	0.356539013	3.195838912	0.215663484	0.327420848	PPP1R14D
ENSG00000186868	2.435363469	0.356539013	2.654908313	0.215740849	0.327519166	MAPT
ENSG00000116809	595.2488655	771.7778548	-0.374227826	0.215827779	0.327631991	ZBTB17
ENSG00000269656	2.449224876	6.573421887	-1.42488569	0.215985175	0.327851766	NOP53-AS1
ENSG00000197603	536.8593763	423.949821	0.340176692	0.21601087	0.327871614	CPLANE1
ENSG00000286990	14.59810594	5.725849174	1.350704312	0.216091793	0.327975281	AC016026.2
ENSG00000273138	2.430348874	0.356539013	2.652387686	0.216169726	0.3280744	AC005021.1
ENSG00000235488	0.586481695	4.178122672	-2.820445834	0.216218689	0.328088361	JARID2-AS1
ENSG00000115526	426.9607748	313.4785201	0.444830146	0.216226849	0.328088361	CHST10
ENSG00000270513	2.771442187	0	3.803038972	0.216229437	0.328088361	AC113368.1
ENSG00000217455	2.771442187	0	3.803038972	0.216229437	0.328088361	AC073316.1
ENSG00000230262	14.17036797	7.399898022	0.92989353	0.216264394	0.328122239	LINC02603
ENSG00000280183	0.616711807	3.311508345	-2.43114734	0.216307412	0.328162847	AC067852.4
ENSG00000272979	15.69320022	7.102282028	1.157471467	0.21631642	0.328162847	AC093388.1
ENSG00000147166	17.45522387	9.496553762	0.886528703	0.216336344	0.32817391	ITGB1BP2
ENSG00000286585	5.727812531	2.129777466	1.421075612	0.216546133	0.32839079	AC104472.4
ENSG00000267147	2.767681241	0	3.801188088	0.216555152	0.32839079	LINC01842
ENSG00000226375	2.767681241	0	3.801188088	0.216555152	0.32839079	AL022310.1
ENSG00000116785	2.767681241	0	3.801188088	0.216555152	0.32839079	CFHR3
ENSG00000258732	2.767681241	0	3.801188088	0.216555152	0.32839079	AC025884.1
ENSG00000105370	2.767681241	0	3.801188088	0.216555152	0.32839079	LIM2
ENSG00000252759	4.809013064	1.406086955	1.761859124	0.216568627	0.328392056	RF00019
ENSG00000162066	298.421236	367.7034362	-0.301177779	0.216780265	0.328688984	AMDHD2
ENSG00000229534	2.430348874	0.336469917	2.652390355	0.216813625	0.328688984	HNRNPA1P53
ENSG00000251453	2.430348874	0.336469917	2.652390355	0.216813625	0.328688984	HAUS1P1
ENSG00000166326	2196.887804	1851.187781	0.246836599	0.216815049	0.328688984	TRIM44
ENSG00000239653	23.74172401	12.77969228	0.900041309	0.216849097	0.328721418	PSMD6-AS2
ENSG00000184612	3.018084217	0.693008929	2.0942122	0.216972298	0.32888899	RPL7P26
ENSG00000213023	6.377261748	11.89198137	-0.899117184	0.21702112	0.328931418	SYT3
ENSG00000255052	15.59491672	6.114225644	1.35231955	0.21702561	0.328931418	FAM66D
ENSG00000236296	5.763057239	11.05733289	-0.943308661	0.217120377	0.329055854	GUSBP5
ENSG00000205832	7.586794766	3.260757667	1.223034625	0.21714218	0.329069703	C16orf96
ENSG00000277496	19.31818051	5.757558237	1.757761876	0.217189713	0.329122539	AL357033.4
ENSG00000160953	1263.89259	1058.016405	0.256706893	0.217330266	0.329316321	PWWP3A
ENSG00000272693	25.02056407	15.63444452	0.675314501	0.217367582	0.329353657	AC073107.1
ENSG00000240143	3.368024343	0.743759607	2.202188788	0.217428229	0.329426336	AL023653.1
ENSG00000172340	1131.757748	1422.251761	-0.329593741	0.217449441	0.329439263	SUCLG2
ENSG00000109775	378.1235796	489.5780097	-0.372950071	0.217495135	0.329489277	UFSP2
ENSG00000287686	13.06169702	6.430626464	1.019527949	0.217526287	0.329517256	AC062029.1
ENSG00000171724	785.0596117	421.6631433	0.897072737	0.217603073	0.329614356	VAT1L
ENSG00000214711	3.885199001	9.170696331	-1.227858849	0.217678158	0.329708868	CAPN14
ENSG00000223177	3.576843098	0.672939833	2.368871233	0.217715614	0.329739178	RNA5SP39
ENSG00000282542	0	2.435565801	-3.868708862	0.217724299	1	AC008993.1
ENSG00000254115	0	2.435565801	-3.868708862	0.217724299	1	AC090572.3
ENSG00000267677	0	2.435565801	-3.868708862	0.217724299	1	AC016229.2
ENSG00000177752	5.199283763	1.456837632	1.841266089	0.217732474	0.329739178	YIPF7
ENSG00000265630	5.476155906	1.854670712	1.581079005	0.217736243	0.329739178	BX547991.2
ENSG00000266835	1.526664462	5.961845217	-1.962732727	0.217793986	0.3298074	GAPLINC
ENSG00000234789	1.524157165	4.320918095	-1.506680561	0.217852987	0.329854301	AL590369.1
ENSG00000268322	3.255879435	0.387220594	3.077471746	0.217858184	0.329854301	BNIP3P25

ENSG00000139540	5.980722805	2.211209725	1.454735204	0.217864289	0.329854301	SLC39A5
ENSG00000006740	75.13819295	48.88659119	0.619544329	0.217881746	0.329854301	ARHGAP44
ENSG00000122741	644.0723971	784.2548877	-0.283768987	0.217891913	0.329854301	DCAF10
ENSG00000157800	562.1216232	712.8980505	-0.343232708	0.217901134	0.329854301	SLC37A3
ENSG00000227467	1.817398012	0	3.195523219	0.217972253	1	LINC01537
ENSG00000214203	1.817398012	0	3.195523219	0.217972253	1	RPS4XP1
ENSG00000267781	1.817398012	0	3.195523219	0.217972253	1	SLC25A36P1
ENSG00000280432	1.817398012	0	3.195523219	0.217972253	1	AP000962.2
ENSG00000204965	1.817398012	0	3.195523219	0.217972253	1	PCDHA5
ENSG00000123843	1.817398012	0	3.195523219	0.217972253	1	C4BPB
ENSG00000261837	1.817398012	0	3.195523219	0.217972253	1	AC046158.2
ENSG00000265252	1.817398012	0	3.195523219	0.217972253	1	MIR3132
ENSG00000288076	1.817398012	0	3.195523219	0.217972253	1	AC018398.1
ENSG00000279400	34.55069189	59.24218206	-0.775153978	0.218022176	0.330016625	AC008957.3
ENSG00000185252	215.9648712	162.001996	0.413550558	0.218033769	0.330016625	ZNF74
ENSG00000267216	5.715204773	11.31108628	-0.976431279	0.218130723	0.33014414	AC020915.1
novel.1061	33.84336101	52.46760249	-0.635207146	0.218266006	0.330329651	-
novel.653	645.093185	502.7318227	0.359994816	0.21832388	0.330397992	-
ENSG00000283654	0.894837599	4.474454397	-2.321583634	0.218353538	0.330410338	LMLN2
ENSG00000259374	2.714742909	0	3.774825594	0.218382908	0.330410338	NDUFB4P11
ENSG00000286196	2.714742909	0	3.774825594	0.218382908	0.330410338	AL162586.2
ENSG00000269699	2.714742909	0	3.774825594	0.218382908	0.330410338	ZIM2
ENSG00000132763	132.7660446	184.3460265	-0.475141618	0.218468344	0.330520354	MMACHC
ENSG00000131871	1248.952292	1526.380186	-0.289577404	0.218592531	0.330688981	SELENOS
novel.477	106.3077267	54.67068705	0.957368725	0.218657566	0.330768105	-
ENSG00000213569	3.885199001	8.611154608	-1.141961357	0.218684378	0.330789405	AL390039.1
ENSG00000131174	1047.781595	1279.775457	-0.288557714	0.218754792	0.330876651	COX7B
ENSG00000212123	43.00807765	24.72743301	0.793053157	0.218813847	0.330946708	PRR22
ENSG00000147174	92.78905835	59.49953147	0.644999588	0.218828578	0.330949723	GCNA
ENSG00000201944	3.253372138	0.356539013	3.076439736	0.218864489	0.330984767	RF00139
ENSG00000138944	24.30857425	63.46040856	-1.386424563	0.219023662	0.331206202	SHISAL1
ENSG00000258130	9.430590673	3.067083173	1.643763987	0.219055104	0.331224162	AC106782.1
ENSG00000105219	13.65319331	7.510855988	0.859717898	0.219061036	0.331224162	CNTD2
ENSG00000132823	589.4374417	494.081429	0.254915582	0.219165491	0.331362815	OSER1
ENSG00000187010	23.9511835	8.24271884	1.5449718	0.21933398	0.331598262	RHD
ENSG00000241697	2.755073482	7.428139459	-1.42765827	0.219392959	0.331668129	TMEFF1
ENSG00000244040	3.253372138	0.336469917	3.076432111	0.219418197	0.331686983	IL12A-AS1
ENSG00000111335	842.7625075	1304.505934	-0.630334505	0.219434792	0.33169277	OAS2
ENSG00000164187	370.6494005	296.3366028	0.322311398	0.219506876	0.331782428	LMBRD2
ENSG00000171101	4.909803862	1.00940975	2.245821651	0.219647008	0.33195957	SIGLEC17P
ENSG00000132204	0	2.415496705	-3.857594473	0.219724527	1	LINC00470
ENSG00000216867	0	2.415496705	-3.857594473	0.219724527	1	RPL22P12
ENSG00000123965	0	2.415496705	-3.857594473	0.219724527	1	PMS2P5
ENSG00000246451	94.01815155	134.2990922	-0.513052738	0.219727774	0.33195957	AL049840.1
ENSG00000273100	2.699627853	0	3.767222934	0.219741976	0.33195957	AL596442.2
novel.1008	2.699627853	0	3.767222934	0.219741976	0.33195957	-
ENSG00000232354	2.699627853	0	3.767222934	0.219741976	0.33195957	VIPR1-AS1
ENSG00000279619	2.699627853	0	3.767222934	0.219741976	0.33195957	AC020907.5
ENSG00000279377	2.699627853	0	3.767222934	0.219741976	0.33195957	AC003973.2
ENSG00000065243	1393.441251	1120.581674	0.314523405	0.219754387	0.33195957	PKN2
ENSG00000104805	4492.738802	5831.169506	-0.376144727	0.219759571	0.33195957	NUCB1

ENSG00000249738	2.946269883	0	3.894485751	0.219764621	0.33195957	AC008691.1
ENSG00000202318	2.946269883	0	3.894485751	0.219764621	0.33195957	RF00019
ENSG00000200091	2.388764651	0.356539013	2.631212831	0.219793208	0.331964151	RN7SKP163
ENSG00000280116	2.388764651	0.356539013	2.631212831	0.219793208	0.331964151	AC231657.2
novel.827	3.915429114	8.620611218	-1.136000821	0.219832207	0.332003753	-
ENSG00000285851	12.37943912	4.880459818	1.358157623	0.219857425	0.332022539	AL359762.3
ENSG00000151773	44.26406694	71.34602763	-0.688124936	0.219962328	0.332161561	CCDC122
ENSG00000214653	5.774411348	10.83297682	-0.907252917	0.219975052	0.332161561	HNRNPA3P3
ENSG00000197140	9.657103052	4.616093944	1.059552753	0.220004466	0.33218667	ADAM32
ENSG00000119782	105.2336876	73.51611887	0.515401824	0.220062141	0.332243013	FKBP1B
ENSG00000272944	0.601596751	3.424906455	-2.514282374	0.220067357	0.332243013	AC079834.2
ENSG00000065060	452.3928072	324.7415867	0.477509336	0.220186495	0.332397366	UHRF1BP1
ENSG00000285769	2.694613258	0	3.764698926	0.220195184	0.332397366	AP003100.2
ENSG00000207291	4.763667895	1.00940975	2.205406461	0.22030254	0.332540105	RNU6-30P
ENSG00000147133	822.8100831	701.8892721	0.229296581	0.220452181	0.332718194	TAF1
ENSG00000166503	1159.49268	1430.576297	-0.303330674	0.220453896	0.332718194	HDGFL3
ENSG00000168916	365.7002788	268.3143445	0.447594828	0.22045894	0.332718194	ZNF608
ENSG00000267426	0.89358395	4.177994278	-2.205537229	0.220484256	0.332737074	AC087289.4
ENSG00000229750	0	2.456790772	-3.879998859	0.2205057	1	AC096649.1
ENSG00000203877	2.722336072	0	3.778638913	0.220546527	0.332780391	RIPPLY2
ENSG00000228622	2.722336072	0	3.778638913	0.220546527	0.332780391	Z82198.1
ENSG00000183134	3.359177531	8.306522148	-1.308113433	0.22055446	0.332780391	PTGDR2
ENSG00000100664	3884.084646	3162.256121	0.296505352	0.220564194	0.332780391	EIF5
ENSG00000199015	0	2.455634897	-3.879685679	0.22059441	1	MIR377
ENSG00000198975	0	2.455634897	-3.879685679	0.22059441	1	MIRLET7A2
ENSG00000249345	0.61420451	3.280826763	-2.422999416	0.220643453	0.332880643	LINC02405
ENSG00000278231	3.926783224	1.130980201	1.831097374	0.220685055	0.332924074	AL133342.1
ENSG00000068383	556.2575494	442.1843315	0.33089913	0.220823151	0.333106209	INPP5A
ENSG00000104164	1421.59942	1202.325709	0.241898509	0.220840883	0.333106209	BLOC1S6
ENSG00000172530	294.8277673	363.5037034	-0.302074639	0.220855926	0.333106209	BANP
ENSG00000123080	825.1555836	587.9197021	0.488610837	0.220857071	0.333106209	CDKN2C
ENSG00000239112	3.546612985	10.73019119	-1.586374435	0.220918141	0.333178976	SNORD123
ENSG00000187713	672.762986	815.7973213	-0.27833345	0.221040301	0.333343863	TMEM203
ENSG00000167397	293.2797191	409.4224829	-0.480765349	0.221080202	0.333384685	VKORC1
ENSG00000197550	3.077290793	0.356539013	2.991634082	0.221241089	0.333607935	AL359955.1
ENSG00000124226	1367.184138	1149.943871	0.249720924	0.221286988	0.333657782	RNF114
ENSG00000141971	655.1205832	798.7658956	-0.285853081	0.221390866	0.333795038	MVB12A
ENSG00000138796	979.5587844	751.3735486	0.382570194	0.221465022	0.333887468	HADH
ENSG00000011465	31288.5467	13876.60302	1.172974581	0.221489017	0.333904268	DCN
ENSG00000214309	52.2428846	72.90796264	-0.482354411	0.221509323	0.333915505	MBLAC1
ENSG00000128989	2961.162846	2484.012516	0.253531955	0.221594547	0.334024595	ARPP19
ENSG00000267742	0.909952655	3.698728939	-2.016517866	0.221738529	0.334216923	SINHCAFP2
ENSG00000261105	15.94994271	29.2916204	-0.879983087	0.221747867	0.334216923	LMO7-AS1
ENSG00000272431	4.274216052	1.060160428	2.000539357	0.221778321	0.334243435	AC104117.5
ENSG00000228862	3.077290793	0.336469917	2.99164063	0.221860702	0.33434475	AC068389.1
ENSG00000088970	328.8069507	238.0357424	0.467057454	0.221871284	0.33434475	KIZ
ENSG00000232344	6.20089567	1.773238453	1.804071379	0.221918204	0.33439606	AC087163.2
ENSG00000285730	1.495180701	4.63616304	-1.627597756	0.221949345	0.334423589	Z94721.3
ENSG00000159335	4690.263417	3934.459036	0.253456985	0.22205521	0.334563698	PTMS
ENSG00000257576	16.05958032	7.593572517	1.066789546	0.222090362	0.334597257	HSPD1P4
ENSG00000228624	17.3622397	27.98446626	-0.6906716	0.222207743	0.334754689	HDAC2-AS2

ENSG00000276087	3.341555177	0.713078025	2.221721822	0.222497781	0.335167739	AC008073.3
ENSG00000164919	1958.997721	1609.925337	0.283137678	0.222515572	0.335167739	COX6C
ENSG00000261167	51.79444716	34.69965609	0.576875791	0.222520624	0.335167739	AC107027.3
ENSG00000006016	697.4476552	2403.375165	-1.784935074	0.22262053	0.335298783	CRLF1
ENSG00000253854	27.7768912	16.63910238	0.74217806	0.222677981	0.335323359	AC010834.3
ENSG00000114779	824.9715785	1049.928716	-0.347865847	0.222708411	0.335323359	ABHD14B
ENSG00000212348	2.772695836	0	3.803697765	0.222714286	0.335323359	RNU6-1300P
ENSG00000183625	2.772695836	0	3.803697765	0.222714286	0.335323359	CCR3
ENSG00000249509	2.772695836	0	3.803697765	0.222714286	0.335323359	AC023886.1
ENSG00000259123	2.772695836	0	3.803697765	0.222714286	0.335323359	AC103996.3
ENSG00000255045	0.586481695	3.139187216	-2.406038914	0.222749124	0.335352224	AP000866.5
ENSG00000125611	289.8794556	373.6007546	-0.366653431	0.222759273	0.335352224	CHCHD5
ENSG00000142544	181.2342654	233.7908752	-0.368686515	0.222778919	0.335362367	CTU1
ENSG00000273973	3.940644631	1.130980201	1.834853211	0.222891591	0.335493101	AC025162.2
ENSG00000230149	3.940644631	1.130980201	1.834853211	0.222891591	0.335493101	AL021707.3
ENSG00000025770	814.4731511	1084.056527	-0.412461112	0.222908869	0.335499671	NCAPH2
ENSG00000258603	3.973382041	1.060160428	1.893700606	0.22296879	0.335570418	AC005225.2
ENSG00000239480	1.509042109	4.402350354	-1.53442503	0.223338698	0.336107664	AC073517.1
ENSG00000285897	5.661012792	1.803920035	1.656380144	0.223379536	0.336149651	AC004687.2
ENSG00000287205	3.675126598	0.672939833	2.405209036	0.223498208	0.336308754	AL451054.4
ENSG00000226245	18.13622812	8.487144012	1.103624293	0.223616984	0.336467994	ZNF32-AS1
ENSG00000130544	147.6203945	111.5709703	0.402805251	0.223683001	0.336533259	ZNF557
ENSG00000260083	19.58724603	11.86245566	0.725641206	0.223686265	0.336533259	MIR762HG
ENSG00000176046	900.9994322	499.8808202	0.849663263	0.223703264	0.336539346	NUPR1
ENSG00000128915	833.8783449	687.070306	0.2795325	0.223844087	0.336731702	ICE2
ENSG00000285793	22.11559367	11.14706589	0.990364811	0.223919103	0.336825047	AC125232.2
ENSG00000148156	2.684512796	0	3.759565485	0.22396309	0.336832709	ACTL7B
ENSG00000280247	2.684512796	0	3.759565485	0.22396309	0.336832709	AC005578.1
ENSG00000261765	2.684512796	0	3.759565485	0.22396309	0.336832709	AC009127.1
ENSG00000286781	7.51623408	3.169868797	1.23681442	0.224028665	0.336911828	AC099332.1
ENSG00000237301	0.601596751	2.782648203	-2.214477155	0.224117498	0.337025914	AL121992.1
ENSG00000113231	117.8702248	89.93788163	0.38879194	0.224181403	0.337086383	PDE8B
ENSG00000168214	1464.713074	1914.392994	-0.386133339	0.224183658	0.337086383	RBPJ
ENSG00000006530	133.2619506	178.4755369	-0.421100967	0.224277214	0.33720754	AGK
ENSG00000270171	2.68075185	0	3.757661835	0.224306736	1	AL359881.3
ENSG00000213033	2.68075185	0	3.757661835	0.224306736	1	AURKAP1
ENSG00000254664	2.68075185	0	3.757661835	0.224306736	1	AC103681.2
ENSG00000249736	2.68075185	0	3.757661835	0.224306736	1	LINC02242
ENSG00000266173	183.3380387	133.5261108	0.457309049	0.22436169	0.337315032	STRADA
ENSG00000252835	0.308355904	2.149846562	-2.724347074	0.224550738	1	SCARNA21
ENSG00000286104	3.60707321	1.080229524	1.744375401	0.224616149	0.337678057	AC016629.3
ENSG00000198758	4.625338556	0	4.542688005	0.224685361	0.337762562	EPS8L3
ENSG00000117601	5.802134163	2.191140629	1.413681101	0.224714053	0.337785863	SERPINC1
ENSG00000167566	893.3098898	1111.32134	-0.314849379	0.224726864	0.337785863	NCKAP5L
ENSG00000137413	430.1176156	518.4639158	-0.269093305	0.224812662	0.337889694	TAF8
ENSG00000138760	6535.030515	5639.933904	0.212519922	0.224821952	0.337889694	SCARB2
ENSG00000164209	1118.917089	961.285625	0.218861611	0.2248903	0.337972865	SLC25A46
ENSG00000118482	1321.451876	1023.918492	0.368310349	0.224922903	0.338002311	PHF3
ENSG00000275491	0.293240848	2.149846562	-2.724345014	0.225110287	1	LINC01730
ENSG00000207744	0.293240848	2.149846562	-2.724345014	0.225110287	1	MIR10B
ENSG00000257103	149.0550009	108.8306437	0.4531735	0.225118428	0.338276569	LSM14A

ENSG00000001461	904.6386955	716.5760427	0.3366119	0.225185384	0.338357612	NIPAL3
ENSG00000230551	323.8802952	218.6415909	0.567663281	0.225217141	0.338385759	AC021078.1
ENSG00000266824	3.007983756	0.387220594	2.961539497	0.225343159	0.338519519	AC129492.5
ENSG00000249771	3.007983756	0.387220594	2.961539497	0.225343159	0.338519519	AC108210.1
ENSG00000237232	3.04831433	6.899279318	-1.177864583	0.225345255	0.338519519	ZNF295-AS1
ENSG00000102390	374.9418521	489.020103	-0.383806026	0.225512603	0.338746342	PBDC1
ENSG00000214176	350.4221802	263.861419	0.41012603	0.225522323	0.338746342	PLEKHM1P1
ENSG00000213901	3.047060681	8.164882601	-1.426593041	0.22560023	0.338843772	SLC23A3
ENSG00000257512	3.676380247	0.713078025	2.358762034	0.225736577	0.339028961	AC124947.2
ENSG00000275763	9.795645855	22.04842234	-1.170089875	0.225755138	0.339030984	C18orf65
ENSG00000259107	5.85876217	0.693008929	3.06334455	0.225764022	0.339030984	LINC00911
ENSG00000276029	3.001715513	0.387220594	2.958804477	0.225806696	0.339069877	MIR4477A
ENSG00000272630	19.46410302	11.71966024	0.725524251	0.225816023	0.339069877	AL731563.3
ENSG00000241129	2.73619748	0	3.785577657	0.225934534	0.339208618	RPL22P19
ENSG00000287468	2.73619748	0	3.785577657	0.225934534	0.339208618	AC007317.2
ENSG00000274816	27.34106187	11.00645382	1.317432305	0.225954614	0.339219163	MIR6772
ENSG00000063587	394.813179	491.1707415	-0.314790987	0.226117352	0.339443862	ZNF275
ENSG00000237887	3.340301529	0.713078025	2.221339181	0.226294543	0.339690232	RPL23AP32
ENSG00000113971	338.5010648	255.3959324	0.406657567	0.22635264	0.33975781	NPHP3
ENSG00000159128	175.3463133	218.929412	-0.320789842	0.226387728	0.339790847	IFNGR2
ENSG00000204954	145.9281051	182.8020588	-0.324950257	0.226476317	0.339904176	C12orf73
ENSG00000135837	1084.170559	835.1993491	0.376718922	0.226530284	0.339965534	CEP350
ENSG00000220920	0	2.395427609	-3.846282688	0.226699308	1	AL023807.1
ENSG00000287673	0	2.395427609	-3.846282688	0.226699308	1	AC051635.1
ENSG00000276704	3.005476459	0.723690511	2.063398727	0.226743627	0.340263272	AL442067.2
ENSG00000215492	43.3419338	59.98894257	-0.471848329	0.226754871	0.340263272	HNRNPA1P7
ENSG00000226287	29.98824958	15.61450382	0.933816756	0.22679315	0.340301058	TMEM191A
ENSG00000269980	3.900314058	1.060160428	1.871395294	0.22700197	0.340594721	AC068768.1
ENSG00000135297	520.3174388	424.0047759	0.294782129	0.227158171	0.340809403	MTO1
ENSG00000258727	15.20625567	8.264072205	0.878942621	0.227189554	0.340830288	AL135999.1
ENSG00000214872	14.80219483	7.458821041	1.004145315	0.227198328	0.340830288	SMTNL1
ENSG00000273069	1.832513069	0	3.206129555	0.227343963	1	AC211433.2
novel.138	1.832513069	0	3.206129555	0.227343963	1	-
ENSG00000175800	1.832513069	0	3.206129555	0.227343963	1	OR52B3P
ENSG00000254561	1.832513069	0	3.206129555	0.227343963	1	AP003393.1
ENSG00000280312	1.832513069	0	3.206129555	0.227343963	1	Z83836.1
ENSG00000240499	1.832513069	0	3.206129555	0.227343963	1	AC004594.1
ENSG00000267314	1.832513069	0	3.206129555	0.227343963	1	AC104532.1
ENSG00000267690	1.832513069	0	3.206129555	0.227343963	1	LDLRAD4-AS1
ENSG00000239268	1.832513069	0	3.206129555	0.227343963	1	AC092691.1
ENSG00000182348	1.832513069	0	3.206129555	0.227343963	1	ZNF804B
ENSG00000274134	1.832513069	0	3.206129555	0.227343963	1	MIR6774
ENSG00000268658	5.725305234	1.854670712	1.645344067	0.227349778	0.341037793	LINC00664
ENSG00000235776	29.57784923	44.73799423	-0.595137432	0.227428873	0.341136745	AC000089.1
novel.775	5.214398819	1.447381022	1.851791674	0.227476506	0.341188495	-
ENSG00000149534	1.83125942	0	3.205257266	0.227509223	1	MS4A2
ENSG00000198643	1.83125942	0	3.205257266	0.227509223	1	FAM3D
ENSG00000236528	1.83125942	0	3.205257266	0.227509223	1	AL033528.2
ENSG00000236434	1.83125942	0	3.205257266	0.227509223	1	AL162430.2
ENSG00000109158	1.83125942	0	3.205257266	0.227509223	1	GABRA4
ENSG00000212413	1.83125942	0	3.205257266	0.227509223	1	RNU11-3P

ENSG00000267723	1.83125942	0	3.205257266	0.227509223	1	AC011509.2
ENSG00000225043	0	2.52645467	-3.917944744	0.227592703	1	RPL18AP2
ENSG00000229314	0	2.52645467	-3.917944744	0.227592703	1	ORM1
ENSG00000164659	63.53865448	45.8005948	0.470634473	0.227606404	0.34136362	KIAA1324L
ENSG00000269148	3.620934617	8.111691778	-1.153195902	0.227741873	0.34154708	AC092301.1
ENSG00000263847	6.231125782	14.61211054	-1.22543072	0.227766215	0.341561974	AP005899.1
ENSG00000164951	1069.98012	1691.104543	-0.660226039	0.227778098	0.341561974	PDP1
ENSG00000100580	450.6401285	559.8064349	-0.312694333	0.227893566	0.3417154	TMED8
ENSG00000136819	1066.514662	883.5871583	0.271162225	0.22801005	0.341870331	C9orf78
ENSG00000167491	1555.152519	1830.675399	-0.235270378	0.228037451	0.341885148	GATAD2A
ENSG00000066135	380.1203648	456.497833	-0.263805222	0.22804625	0.341885148	KDM4A
ENSG00000286786	9.118473823	4.391737868	1.063717628	0.228076848	0.341911291	AC116158.3
novel.241	5.134991321	1.447381022	1.832422397	0.228113422	0.341934226	-
ENSG00000162959	101.6929437	73.02824882	0.477836492	0.228127597	0.341934226	MEMO1
ENSG00000183530	564.0456414	727.5876333	-0.367055335	0.22813163	0.341934226	PRR14L
ENSG00000161999	354.427999	447.1333806	-0.335625546	0.228166795	0.341967205	JMJD8
ENSG00000102804	3091.105659	2405.525619	0.361866917	0.228205694	0.342005777	TSC22D1
ENSG00000218890	1.229662669	4.280779903	-1.809658642	0.228489135	0.342397127	NUFIP1P1
ENSG00000185664	9.356269041	17.14318871	-0.873644537	0.228493183	0.342397127	PMEL
ENSG00000023171	701.105359	961.5015138	-0.455480236	0.228566253	0.342470346	GRAMD1B
ENSG00000228485	0.586481695	3.199394504	-2.430002635	0.228568407	0.342470346	GRK5-IT1
ENSG00000232124	3.344062475	0.672939833	2.266605648	0.228794736	0.342789691	AP001057.1
ENSG00000279199	9.372709017	4.290236513	1.125650617	0.228813901	0.342798638	AC068669.1
ENSG00000151023	3.646150135	8.457618305	-1.208777236	0.228851022	0.34283448	ENKUR
ENSG00000149735	5.542884374	1.41669944	1.959165365	0.228888105	0.342870264	GPHA2
ENSG00000182372	3.031945625	0.743759607	2.054726607	0.229031631	0.343060056	CLN8
ENSG00000143919	99.66246746	77.52521246	0.360919582	0.229041212	0.343060056	CAMKMT
ENSG00000227678	3.902821355	0.743759607	2.413885811	0.229153367	0.343208256	AL355581.1
ENSG00000226490	1.818651661	0	3.196404306	0.229186833	1	AC138647.1
ENSG00000225873	1.818651661	0	3.196404306	0.229186833	1	C3orf86
ENSG00000231024	1.818651661	0	3.196404306	0.229186833	1	AC092431.1
ENSG00000259935	1.818651661	0	3.196404306	0.229186833	1	AC009754.1
ENSG00000241622	1.818651661	0	3.196404306	0.229186833	1	RARRES2P1
ENSG00000286294	1.818651661	0	3.196404306	0.229186833	1	AC107398.5
ENSG00000259192	1.818651661	0	3.196404306	0.229186833	1	AC109631.1
ENSG00000273816	1.818651661	0	3.196404306	0.229186833	1	AC005695.3
ENSG00000279954	2.987854105	0.356539013	2.952692872	0.229247314	0.343309381	BX324167.2
ENSG00000270077	2.987854105	0.356539013	2.952692872	0.229247314	0.343309381	AP003117.1
ENSG00000114999	1050.723908	1305.630625	-0.313499034	0.229272161	0.343326803	TTL
ENSG00000231327	19.18089136	9.261713595	1.047002456	0.229421376	0.343530446	LINC01816
ENSG00000227741	16.84227301	9.282938566	0.858462562	0.22947239	0.343587031	AL121987.2
ENSG00000248099	1.816144364	0	3.194649542	0.229521624	1	INSL3
ENSG00000288023	1.816144364	0	3.194649542	0.229521624	1	AL157936.1
ENSG00000204706	1.816144364	0	3.194649542	0.229521624	1	MAMDC2-AS1
ENSG00000242696	1.816144364	0	3.194649542	0.229521624	1	RN7SL40P
ENSG00000164076	1.816144364	0	3.194649542	0.229521624	1	CAMKV
ENSG00000258696	1.816144364	0	3.194649542	0.229521624	1	AL359233.1
ENSG00000213424	1.816144364	0	3.194649542	0.229521624	1	KRT222
ENSG00000229992	1.816144364	0	3.194649542	0.229521624	1	HMGB3P9
ENSG00000254396	1.816144364	0	3.194649542	0.229521624	1	AL355432.1
ENSG00000260695	1.816144364	0	3.194649542	0.229521624	1	AC022164.1

ENSG00000278330	7.561579249	3.199394504	1.235242483	0.229527186	0.343649273	AC018529.2
ENSG00000130779	1465.449582	1217.703505	0.266933783	0.229585011	0.343696321	CLIP1
ENSG00000262663	7.81699682	2.475703993	1.652894195	0.229585068	0.343696321	AC087222.1
ENSG00000166170	706.0564783	817.6860808	-0.211720111	0.229648041	0.343764782	BAG5
ENSG00000162669	9.279440112	4.250098321	1.122656724	0.229657262	0.343764782	HFM1
ENSG00000141127	504.4701097	423.1568992	0.253034381	0.229725686	0.343847393	PRPSAP2
ENSG00000237070	1.538018572	6.809674718	-2.157574778	0.229791513	0.343912565	AC005550.2
ENSG00000196418	98.48810788	71.7783951	0.4531568	0.229795702	0.343912565	ZNF124
ENSG00000090975	386.721106	301.524906	0.359293659	0.22983036	0.343944622	PITPNM2
ENSG00000264112	428.1478721	598.7191807	-0.483384301	0.229867853	0.343980917	AC015813.1
ENSG00000166002	131.5194716	89.59323937	0.552239384	0.229883982	0.343982308	SMCO4
ENSG00000278967	2.987854105	0.336469917	2.952687168	0.229895262	0.343982308	AL138847.2
ENSG00000103319	14.19049762	22.17487308	-0.644151869	0.23004751	0.344190288	EEF2K
ENSG00000267662	0.879722543	4.534533291	-2.343291281	0.230126573	0.344288754	AC025809.1
ENSG00000279140	25.65873044	16.40888571	0.643267202	0.230180767	0.344350003	AL590326.1
ENSG00000223695	4.266622888	1.436768536	1.568511659	0.230264522	0.344455467	FO393418.1
ENSG00000150433	437.1151363	371.4247556	0.234735191	0.230367757	0.344590056	TMEM218
ENSG00000260808	12.06349006	5.705780078	1.077691238	0.230384969	0.344595964	AP003096.1
ENSG00000205704	0.879722543	4.168666062	-2.234006504	0.230443856	0.344654403	LINC00634
ENSG00000258875	9.835976429	22.51938694	-1.194910782	0.230450571	0.344654403	AL135818.1
ENSG00000104953	17.14833508	32.3076961	-0.915722513	0.230612654	0.344872226	TLE6
ENSG00000238517	3.038213869	0.336469917	2.974738607	0.230622765	0.344872226	MIR1270
ENSG00000161526	1826.087729	1461.300833	0.321529299	0.230711256	0.344984699	SAP30BP
ENSG00000178636	6.244987189	2.089639274	1.567379428	0.230733479	0.344998073	AC092656.1
ENSG00000233203	0.307102255	2.089639274	-2.690319091	0.230761188	1	DHCR24-DT
ENSG00000226571	8.102715775	2.945512721	1.473008186	0.230797971	0.345074644	AL592429.2
ENSG00000049540	529.1589334	2083.086405	-1.976993712	0.230856186	0.345141821	ELN
ENSG00000145388	544.3209481	451.2873718	0.270234545	0.230936252	0.345223499	METTL14
ENSG00000168386	416.3005764	570.3162044	-0.453993054	0.230942444	0.345223499	FILIP1L
novel.987	112.5686115	69.33298751	0.699336391	0.230950681	0.345223499	-
ENSG00000250131	16.20410663	8.571016416	0.918736904	0.231039352	0.345336175	AC078881.1
ENSG00000134717	801.9096343	978.112764	-0.286865506	0.231078167	0.345374324	BTF3L4
ENSG00000186532	286.0417968	346.4954286	-0.276171252	0.231106963	0.345397493	SMYD4
ENSG00000146576	663.2743158	551.7310885	0.265641205	0.231217885	0.345543392	C7orf26
novel.276	1.803536605	0	3.185741065	0.231222112	1	-
ENSG00000181023	1.803536605	0	3.185741065	0.231222112	1	OR56B1
ENSG00000283360	1.803536605	0	3.185741065	0.231222112	1	AL592295.3
ENSG00000283075	1.803536605	0	3.185741065	0.231222112	1	AL157931.2
ENSG00000286802	1.803536605	0	3.185741065	0.231222112	1	AC104459.1
ENSG00000223642	1.803536605	0	3.185741065	0.231222112	1	AC008277.1
ENSG00000235920	1.803536605	0	3.185741065	0.231222112	1	AC073109.1
ENSG00000261202	1.803536605	0	3.185741065	0.231222112	1	Z83847.1
ENSG00000276740	1.510295757	4.250098321	-1.496186208	0.231297199	0.34564204	AL445649.1
ENSG00000237979	0.293240848	2.089639274	-2.690297069	0.231298899	1	AC007389.4
ENSG00000166455	1.802282956	0	3.184858351	0.231391709	1	C16orf46
ENSG00000213703	1.802282956	0	3.184858351	0.231391709	1	AL138847.1
ENSG00000259188	1.802282956	0	3.184858351	0.231391709	1	AC025040.1
ENSG00000274370	1.802282956	0	3.184858351	0.231391709	1	AC130371.2
ENSG00000253168	1.802282956	0	3.184858351	0.231391709	1	AC124069.1
ENSG00000223558	1.802282956	0	3.184858351	0.231391709	1	TRIM60P17
ENSG00000237640	1.802282956	0	3.184858351	0.231391709	1	AC004522.2

ENSG00000226342	1.802282956	0	3.184858351	0.231391709	1	NMD3P1
ENSG00000253309	7.754029298	2.406040094	1.672812671	0.231417334	0.345801677	SERPINE3
novel.50	9.080721818	2.495773089	1.860365838	0.231447465	0.34582681	-
ENSG00000159239	3.268487194	0.723690511	2.185791906	0.231574245	0.345996346	AC005041.1
ENSG00000114784	841.3951324	690.6472553	0.285098639	0.231630835	0.346060996	EIF1B
ENSG00000180189	5.408102518	1.702418679	1.638876527	0.231644436	0.346061416	HMGB1P14
ENSG00000109323	743.2294268	598.0256582	0.314034838	0.231695773	0.346118208	MANBA
ENSG00000223922	1.832513069	5.196860638	-1.498391455	0.231719671	0.346134006	ASS1P2
ENSG00000286633	0.600343103	3.404837359	-2.508292413	0.231752375	0.346146829	AL160400.1
ENSG00000203663	3.298717306	0.672939833	2.249598756	0.231754901	0.346146829	OR2L2
ENSG00000157765	2.66939774	0	3.751827051	0.232017982	1	SLC34A2
ENSG00000263167	2.66939774	0	3.751827051	0.232017982	1	AC115099.1
ENSG00000233791	2.66939774	0	3.751827051	0.232017982	1	LINC01136
ENSG00000207308	2.66939774	0	3.751827051	0.232017982	1	RNU6-878P
ENSG00000104221	112.7671445	145.8971008	-0.372026418	0.232039266	0.34655163	BRF2
ENSG00000110921	390.3153858	301.7712233	0.370521107	0.232081116	0.346585779	MVK
novel.152	14.46640085	3.718798035	1.966058982	0.232088811	0.346585779	-
ENSG00000131941	90.97355472	66.55992431	0.449468961	0.232109536	0.346596807	RHPN2
ENSG00000107104	706.006688	886.815483	-0.329348687	0.232134952	0.346614839	KANK1
ENSG00000202502	2.666890443	0	3.750549336	0.232250878	1	RNA5SP151
ENSG00000226758	2.666890443	0	3.750549336	0.232250878	1	DISC1-IT1
ENSG00000286891	2.666890443	0	3.750549336	0.232250878	1	AC080132.1
ENSG00000251187	2.666890443	0	3.750549336	0.232250878	1	AC010261.2
ENSG00000286948	2.666890443	0	3.750549336	0.232250878	1	AP000867.5
ENSG00000056586	964.0398102	1136.257892	-0.237194933	0.232253158	0.34677141	RC3H2
ENSG00000159674	1934.209101	1219.975484	0.664864674	0.232276295	0.346786026	SPON2
ENSG00000149016	217.5036446	264.9785419	-0.284644124	0.232396829	0.346946045	TUT1
ENSG00000266189	0	2.475703993	-3.890551144	0.232543306	1	MIR3186
ENSG00000079459	77.31972067	106.063955	-0.453414118	0.232643293	0.347294036	FDFT1
ENSG00000286129	8.403478515	15.99098353	-0.927544034	0.232797681	0.347504542	AC245060.7
ENSG00000224854	3.960774282	1.060160428	1.890270493	0.23284212	0.347550909	CDKN2A-DT
ENSG00000267666	3.623441915	8.510809128	-1.233776006	0.232917263	0.347643098	AC004156.2
ENSG00000106049	613.8946443	804.370675	-0.389767211	0.232978193	0.347714065	HIBADH
ENSG00000254433	7.210385474	2.985650913	1.295261983	0.233028307	0.347768881	AP001001.1
ENSG00000249863	10.62626228	17.27421577	-0.701176917	0.233297038	0.348149934	AC021106.1
ENSG00000138741	9.786870314	4.361056287	1.169600652	0.23333587	0.348178093	TRPC3
ENSG00000215237	9.506237225	2.355289417	1.992574339	0.23334271	0.348178093	AL592293.1
ENSG00000233858	2.155984028	6.510902848	-1.600321853	0.233451725	0.348320753	LINC02599
ENSG00000119760	1155.456761	953.771944	0.276903504	0.233542347	0.34842016	SUPT7L
ENSG00000204052	14.6613582	25.79435311	-0.814919646	0.233545171	0.34842016	LRRC73
ENSG00000254397	1.49643435	4.637318915	-1.626929074	0.233640658	0.348542601	AC132192.1
ENSG00000139211	223.0975016	133.6027568	0.738315416	0.233687135	0.34859192	AMIGO2
ENSG00000243716	325.9619728	200.0632691	0.704530244	0.233828717	0.348783092	NPIPB5
ENSG00000130748	453.4375414	548.7403477	-0.275388383	0.233900568	0.348870237	TMEM160
ENSG00000258515	21.72943992	11.43638115	0.915892249	0.233979953	0.348952945	AL355075.2
ENSG00000278002	2.450478525	0.336469917	2.662798713	0.233982882	0.348952945	AL627171.1
ENSG00000204520	48.23697897	110.7507531	-1.199693891	0.234013993	0.348979311	MICA
ENSG00000230267	28.26229736	12.36996244	1.200359539	0.234039668	0.348997567	HERC2P4
ENSG00000187624	7.016681776	2.782648203	1.315157606	0.234091481	0.349039415	C17orf97
ENSG00000279035	3.96328158	0.723690511	2.457652882	0.2340946	0.349039415	AC022211.4
ENSG00000165487	741.4133544	870.3035896	-0.231395561	0.234224806	0.349213513	MICU2

ENSG00000158113	12.13279709	6.767096381	0.837285459	0.234321329	0.349337375	LRRC43
ENSG00000186265	18.6851	5.04704875	1.877218438	0.234393996	0.34942566	BTLA
ENSG00000102931	113.5008024	85.11056546	0.41415094	0.234515825	0.349587219	ARL2BP
ENSG00000244232	0.293240848	2.618499415	-3.001720026	0.234571774	0.34965056	RN7SL698P
ENSG00000228701	6.034914786	2.079026788	1.518428668	0.234618919	0.34970077	TNKS2-AS1
ENSG00000162775	184.9383407	234.5043727	-0.341767032	0.23469119	0.349788424	RBM15
ENSG00000270427	3.342808826	7.14370449	-1.099748226	0.234714303	0.349802805	NRBF2P5
ENSG00000285509	1.525410814	4.351599676	-1.513372757	0.23476507	0.349858396	AP000646.1
ENSG00000107882	860.1214919	654.8852426	0.393781201	0.234793161	0.34988019	SUFU
ENSG00000176681	22.17856119	8.721984179	1.357079271	0.234819807	0.349899828	LRRC37A
ENSG00000158615	1101.229828	895.2308488	0.298955835	0.234893903	0.3499735	PPP1R15B
ENSG00000253559	11.39258627	5.391691008	1.0823307	0.234896189	0.3499735	OSGEPL1-AS1
ENSG00000271200	6.605027776	1.548882377	2.12308831	0.234946654	0.350028615	AC099791.2
ENSG00000227698	0.89358395	3.932541625	-2.130181442	0.23496406	0.350034475	AP001619.1
ENSG00000241313	9.99443542	5.055221091	0.986941215	0.235011805	0.350085528	WWTR1-AS1
ENSG00000229298	2.081662396	5.900482053	-1.486918578	0.235081977	0.350166936	TUBB8P1
ENSG00000100360	492.2694455	605.2596093	-0.297569619	0.235093409	0.350166936	IFT27
ENSG00000180423	43.24246793	58.61109705	-0.439478067	0.235177236	0.350271714	HARBI1
ENSG00000270638	3.89780676	1.10029862	1.841258428	0.235261904	0.350364167	AL023806.1
ENSG00000280351	14.70265769	24.06968198	-0.70798633	0.235266281	0.350364167	AC127496.7
novel.262	3.073529847	0.387220594	2.990149266	0.235283196	0.350369274	-
ENSG00000164107	48.62227781	20.10645877	1.274268173	0.235328199	0.350416204	HAND2
ENSG00000269556	337.2338209	275.3956712	0.291370355	0.235396159	0.350497312	TMEM185A
ENSG00000100151	520.756404	386.1012774	0.431361288	0.235450519	0.350558162	PICK1
ENSG00000251550	0.616711807	4.106018629	-2.73923695	0.235466786	0.350562293	AC091133.4
ENSG00000230176	1.22840902	5.105971769	-2.058828997	0.235500793	0.35057265	LINC01433
ENSG00000260063	8.781141456	15.19647325	-0.796401844	0.235509112	0.35057265	AL512408.1
ENSG00000248256	11.7400191	6.430626464	0.867683195	0.235514223	0.35057265	OCIAD1-AS1
ENSG00000167524	91.8825819	62.25115976	0.558990256	0.235578647	0.350632777	RSKR
ENSG00000196793	101.9542735	72.35330121	0.492035825	0.235581608	0.350632777	ZNF239
ENSG00000228594	126.3778556	184.2074222	-0.544550066	0.235641077	0.350701199	FNDC10
ENSG00000270084	22.31083575	14.1031911	0.666193985	0.23566966	0.350705514	GAS5-AS1
ENSG00000275540	7.239361937	2.0188195	1.816637412	0.235670973	0.350705514	AC011477.7
ENSG00000265739	0.586481695	2.904218654	-2.283457051	0.235694962	0.350721123	AC104984.3
ENSG00000004399	3593.1603	5919.536057	-0.720271057	0.235806029	0.350866298	PLXND1
ENSG00000188428	258.0070799	197.2896108	0.385760978	0.235823414	0.350872073	BLOC1S5
ENSG00000111605	1304.488753	1509.741747	-0.210925744	0.235891371	0.350953085	CPSF6
novel.679	173.5034144	258.3839082	-0.574813446	0.235955221	0.351027977	-
ENSG00000112619	51.50657692	77.18301035	-0.586034752	0.235972932	0.351034225	PRPH2
ENSG00000102531	2074.115982	1801.732821	0.203013295	0.23600854	0.351067095	FNDC3A
ENSG00000266786	4.691853561	0.356539013	3.603625625	0.236161617	0.35125887	LGALS9DP
ENSG00000164241	65.84013374	47.71444532	0.462681495	0.236164503	0.35125887	C5orf63
ENSG00000205592	3.073529847	0.356539013	2.990157228	0.236189791	0.351276373	MUC19
ENSG00000284473	3.560474393	9.416277378	-1.39455451	0.236213373	0.351291336	MIR4720
ENSG00000133678	219.6646432	270.9572925	-0.303167582	0.236227096	0.351291637	TMEM254
ENSG00000153006	503.2837838	607.600947	-0.271404243	0.236289386	0.351351323	SREK1IP1
ENSG00000255557	1.186824798	4.411806964	-1.878281156	0.236294279	0.351351323	AP001266.2
ENSG00000251152	4.398612713	0	4.471745007	0.236532304	0.351685121	AC025539.1
ENSG00000253931	4.691853561	0.336469917	3.603622472	0.236566794	0.351716273	AC105118.1
ENSG00000273162	4.590093848	10.21322781	-1.162265107	0.236659751	0.351834343	AL133215.2
ENSG00000102078	165.6182936	202.4433323	-0.288985409	0.2366848	0.351842186	SLC25A14

ENSG00000239608	0.308355904	2.587817834	-2.986569042	0.236702885	0.351842186	RUVBL1-AS1
ENSG00000272308	10.69048345	4.923038154	1.107682145	0.236711409	0.351842186	AC104113.1
ENSG00000169220	175.4453081	121.5631813	0.528592778	0.236719195	0.351842186	RGS14
ENSG00000171735	730.6203005	590.8939789	0.306747702	0.236741246	0.35184927	CAMTA1
ENSG00000228061	0.307102255	2.587817834	-2.986570176	0.236751047	0.35184927	AC131571.1
ENSG00000252585	3.240764379	0.387220594	3.071017376	0.236799758	0.351901534	RF00019
ENSG00000182118	109.0011141	141.939143	-0.382603529	0.236847145	0.351939378	FAM89A
ENSG00000243389	9.356269041	4.279624027	1.125642616	0.236863618	0.351939378	AC012442.2
ENSG00000230650	3.970874743	8.784760006	-1.15555105	0.236865862	0.351939378	AC140479.2
ENSG00000072401	417.4364723	514.1291744	-0.300643327	0.236903876	0.351975731	UBE2D1
ENSG00000162194	49.88134422	70.55742512	-0.501200723	0.236946459	0.352018867	LBHD1
ENSG00000286878	1.218308559	6.552068521	-2.422834571	0.237044225	0.352132785	AC100805.1
ENSG00000278959	20.4027474	10.07732046	1.029242865	0.237050245	0.352132785	AC006378.2
ENSG00000245067	3.634796025	1.049547942	1.772536265	0.237116536	0.352211121	IGFBP7-AS1
ENSG00000106344	571.0578506	768.7155013	-0.428881413	0.237223937	0.352320814	RBM28
ENSG00000213683	0.586481695	2.884149558	-2.275080997	0.237239047	0.352320814	AC002056.1
ENSG00000267769	11.38882532	19.88223109	-0.803292381	0.237255007	0.352320814	AC011498.6
ENSG00000281160	2.773949485	0	3.804404919	0.237274075	0.352320814	AC018358.1
ENSG00000211779	2.773949485	0	3.804404919	0.237274075	0.352320814	TRAV5
ENSG00000184697	0.293240848	2.587817834	-2.98658273	0.237285308	0.352320814	CLDN6
ENSG00000234650	0.293240848	2.587817834	-2.98658273	0.237285308	0.352320814	PCCA-AS1
ENSG00000232850	6.392376805	12.37009083	-0.950881069	0.237434473	0.352522146	AL590708.1
ENSG00000223612	18.2520627	28.11780507	-0.623719514	0.237477907	0.352566485	AC241585.1
ENSG00000151233	694.4592622	516.6454142	0.426209847	0.237513491	0.352599166	GXYLT1
ENSG00000258711	3.043299735	0.387220594	2.976993692	0.237556741	0.352643223	AL358334.2
novel.741	190.6199104	257.5315236	-0.432781931	0.237591143	0.352674141	-
ENSG00000204519	80.28418238	106.0290823	-0.402452737	0.237665146	0.352763834	ZNF551
ENSG00000172375	381.6698088	317.5612935	0.265837176	0.237833417	0.35299343	C2CD2L
ENSG00000277895	0.615458159	3.281982638	-2.421016981	0.238017577	0.353219515	AC135279.3
ENSG00000062650	937.8694884	1109.054032	-0.241907761	0.238027858	0.353219515	WAPL
ENSG00000103248	416.0905634	330.5794688	0.331475997	0.238043951	0.353219515	MTHFSD
novel.329	2.765173943	0	3.800061252	0.238053721	0.353219515	-
ENSG00000140478	2.765173943	0	3.800061252	0.238053721	0.353219515	GOLGA6D
ENSG00000207750	0.601596751	3.423622185	-2.494963018	0.238104311	0.353274405	MIR553
ENSG00000274769	3.240764379	0.336469917	3.070998046	0.238160249	0.353331805	AC016747.4
ENSG00000109083	423.9959133	510.5573629	-0.267546403	0.238170878	0.353331805	IFT20
ENSG00000204256	205.9327992	48.36774842	2.089405526	0.238183797	0.353331805	BRD2
ENSG00000283839	20.85089976	9.119817259	1.205575227	0.238409876	0.353646988	AC096667.1
ENSG00000105865	3.035706571	6.828459544	-1.17036348	0.238462059	0.353695126	DUS4L
ENSG00000226167	14.72912685	6.237080364	1.229802681	0.238469556	0.353695126	AP4B1-AS1
ENSG00000205078	17.31195121	30.1211318	-0.795975107	0.238507208	0.353713085	SYCE1L
ENSG00000273768	2.760088077	0	3.797521198	0.238508892	0.353713085	RF00003
ENSG00000136877	1135.166244	1410.603259	-0.31326092	0.238603761	0.35383358	FPGS
ENSG00000109016	418.514885	342.843178	0.287138909	0.238651851	0.353884695	DHRS7B
ENSG00000189334	4.583825605	1.069617038	2.093394425	0.238720427	0.35396618	S100A14
ENSG00000121057	510.9701143	396.3976413	0.365990655	0.238739933	0.353974902	AKAP1
ENSG00000273724	7.217907366	1.682349583	2.077856362	0.23881269	0.354062572	AC106782.5
ENSG00000272100	4.203655366	9.528391219	-1.171973954	0.238892024	0.354159982	AL513218.1
ENSG00000070404	4910.439402	6401.34406	-0.382576488	0.239201475	0.354598512	FSTL3
ENSG00000226937	9.643241645	3.904171794	1.301164706	0.23925033	0.354650701	CEP164P1
ENSG00000132017	564.7240939	669.2505946	-0.245017281	0.239303144	0.354707068	DCAF15

ENSG00000118961	342.6660709	428.5818404	-0.322898597	0.239315662	0.354707068	LDAH
ENSG00000140612	2217.549129	2650.15075	-0.257205087	0.23934752	0.35473405	SEC11A
ENSG00000287333	2.750058887	0	3.792533678	0.239407411	0.354782337	AL161447.2
ENSG00000109991	2.750058887	0	3.792533678	0.239407411	0.354782337	P2RX3
ENSG00000096872	275.8098509	325.8175785	-0.24024313	0.239538703	0.354956655	IFT74
ENSG00000201240	0	2.406040094	-3.85177981	0.239640579	1	SNORD114-9
ENSG00000231167	5.419456628	10.73147546	-0.984141922	0.239656132	0.355110412	YBX1P2
ENSG00000185522	7.946479348	1.548882377	2.384470649	0.239823584	0.355338269	LMNTD2
ENSG00000237351	1.17296339	5.04345273	-2.090046979	0.239864694	0.355363072	AL513366.1
ENSG00000275494	24.42906777	14.7774152	0.718573302	0.239876722	0.355363072	AC133552.5
ENSG00000153823	313.2686015	639.2157373	-1.028903524	0.239881358	0.355363072	PID1
ENSG00000178809	16.99224119	9.141299019	0.885780944	0.239989731	0.355503347	TRIM73
ENSG00000118600	181.2618457	248.8851236	-0.45862617	0.24006339	0.355592186	RXYLT1
ENSG00000207959	1.17296339	4.758889366	-2.001942901	0.240082046	0.355599547	MIR656
ENSG00000120910	430.4744365	364.1851536	0.241512998	0.240148372	0.355677509	PPP3CC
ENSG00000279636	9.538689901	3.882946823	1.292357528	0.240166806	0.355684534	AL132989.2
ENSG00000227799	18.82719029	28.85925293	-0.615849159	0.240340154	0.355920973	AC012358.2
ENSG00000217488	2.374903244	0.356539013	2.623668667	0.240387702	0.355971096	AL356057.1
ENSG00000267750	3.94189828	10.1894343	-1.362164624	0.24042242	0.356002217	RUNDC3A-AS1
ENSG00000253408	2.124500267	0.387220594	2.458440088	0.240521049	1	AC083973.1
ENSG00000271185	2.124500267	0.387220594	2.458440088	0.240521049	1	AC007029.1
novel.324	35.68876658	22.62461271	0.656558347	0.240539625	0.356139562	-
ENSG00000133983	85.45134164	62.59798537	0.44734701	0.24054259	0.356139562	COX16
ENSG00000273084	13.7451373	6.307900138	1.113531762	0.240596077	0.356198454	AC092171.5
ENSG00000178761	394.8090902	334.8510832	0.238220836	0.24065627	0.356250061	FAM219B
ENSG00000119283	12.80495453	4.394178013	1.526074162	0.240658359	0.356250061	TRIM67
ENSG00000261723	3.85371524	0.723690511	2.421076008	0.240736269	0.35634509	AC008870.4
ENSG00000168517	69.17298465	50.47201574	0.458254741	0.240855769	0.356501666	HEXIM2
ENSG00000110851	685.1659325	818.7662632	-0.256852569	0.240918799	0.356574645	PRDM4
ENSG00000221539	9.91760649	3.749479616	1.419976473	0.240951528	0.356602771	SNORD99
ENSG00000149657	799.3102434	940.8676803	-0.234937706	0.240994838	0.356646553	LSM14B
novel.398	33.62855875	17.94355958	0.909136108	0.241099607	0.356772515	-
ENSG00000132321	17.03500779	7.206351922	1.228924848	0.241107418	0.356772515	IQCA1
ENSG00000273139	5.74042029	2.140389951	1.420515496	0.241141669	0.356802877	AC007663.3
ENSG00000272646	3.678887545	1.080229524	1.766820186	0.241177988	0.356815974	AC079766.1
ENSG00000251535	3.678887545	1.080229524	1.766820186	0.241177988	0.356815974	AC079140.4
ENSG00000224875	3.297463658	0.713078025	2.205549845	0.24121692	0.356853251	AC083949.1
ENSG00000114353	8530.094575	10432.90267	-0.290478971	0.241250921	0.35688323	GNAI2
ENSG00000165966	3.992258043	0.713078025	2.478461567	0.241316701	0.356960215	PDZRN4
ENSG00000121058	274.4854801	333.3060065	-0.280129982	0.241544058	0.357252217	COIL
ENSG00000139218	2098.630998	1797.361524	0.223595516	0.241548497	0.357252217	SCAF11
ENSG00000267432	1.22840902	6.889951102	-2.495096979	0.241558599	0.357252217	DNAH17-AS1
ENSG00000271590	9.838198992	4.310305609	1.19376823	0.241569106	0.357252217	AC108463.3
ENSG00000258476	1.833766717	0	3.207108638	0.241735126	1	LINC02207
ENSG00000119614	1.833766717	0	3.207108638	0.241735126	1	VSX2
ENSG00000254924	1.833766717	0	3.207108638	0.241735126	1	AP000867.2
ENSG00000254880	1.833766717	0	3.207108638	0.241735126	1	PKNOX2-AS1
ENSG00000230054	1.833766717	0	3.207108638	0.241735126	1	TEX53
ENSG00000163348	847.8386519	694.584934	0.287734626	0.241790897	0.357549487	PYGO2
ENSG00000249947	9.65961035	4.973788832	0.952847573	0.24179764	0.357549487	XBP1P1
ENSG00000279667	6.417592322	1.682349583	1.902836718	0.241840655	0.357572242	AL603839.4

ENSG00000023445	768.7907175	528.4787387	0.541214068	0.241851854	0.357572242	BIRC3
ENSG00000113441	1146.040841	969.2292548	0.241793764	0.241854317	0.357572242	LNPEP
ENSG00000067445	500.6961586	620.1724995	-0.308347347	0.241962982	0.357712543	TRO
ENSG00000258561	6.023560676	2.486316478	1.271213213	0.241991377	0.357720758	AL359232.1
ENSG00000254855	9.636973401	4.055267952	1.260794485	0.241996076	0.357720758	AP001107.8
ENSG00000163995	33.47977294	50.91396822	-0.608289073	0.242142036	0.357916154	ABLIM2
ENSG00000251441	3.649911081	1.060160428	1.771700255	0.242199566	0.357980825	RTEL1P1
ENSG00000161973	1.830005771	0	3.204467564	0.242245414	1	CCDC42
ENSG00000271156	1.830005771	0	3.204467564	0.242245414	1	AC090578.2
ENSG00000228804	1.830005771	0	3.204467564	0.242245414	1	AC072022.1
ENSG00000224478	1.830005771	0	3.204467564	0.242245414	1	AL356417.1
ENSG00000239472	1.830005771	0	3.204467564	0.242245414	1	RN7SL221P
ENSG00000182095	2300.024602	1913.814346	0.265093843	0.242290225	0.35809445	TNRC18
ENSG00000256898	3.631035079	0.774441189	2.276453545	0.242452402	0.358313758	AP002770.2
ENSG00000255478	2.124500267	0.336469917	2.458447071	0.242493187	1	AP000944.1
ENSG00000169203	23.49013866	14.52237754	0.689664463	0.242514254	0.358384781	NPIPB12
ENSG00000115289	330.3083711	266.9169566	0.30793136	0.242570494	0.358447503	PCGF1
ENSG00000257718	4.601447958	1.00940975	2.152519103	0.24258752	0.358452275	CPNE8-AS1
ENSG00000132570	146.5606874	107.6268359	0.443294481	0.242612168	0.358453655	PCBD2
ENSG00000270923	7.446927043	2.558292127	1.552288306	0.24262957	0.358453655	TAS2R6P
ENSG00000156689	12.56465202	24.88528884	-0.985799186	0.242629844	0.358453655	GLYATL2
ENSG00000241157	0.293240848	2.71182843	-3.066605857	0.242751512	0.358613012	AC104763.1
ENSG00000128656	839.0353419	583.6257627	0.523549357	0.24284404	0.358729304	CHN1
ENSG00000279738	23.18178275	35.10938592	-0.600138386	0.242900007	0.358791579	AL022311.1
ENSG00000286276	2.11063886	0.356539013	2.450671086	0.243156614	1	AL390066.2
ENSG00000219392	1.17296339	4.697526203	-1.985594617	0.243163325	0.359126914	ZNF602P
ENSG00000269836	2.052685933	8.110535903	-1.970452775	0.243167946	0.359126914	AC020913.3
ENSG00000172738	12.067251	5.932447905	1.012943776	0.243168495	0.359126914	TMEM217
ENSG00000093134	3.944405577	12.6248717	-1.673290389	0.243315848	0.359324109	VNN3
ENSG00000092820	1326.849446	1898.099188	-0.516715544	0.243359308	0.359345592	EZR
ENSG00000144355	302.1840912	171.3855243	0.819176569	0.243368471	0.359345592	DLX1
ENSG00000251131	6.64403343	1.782695063	1.894642647	0.243378701	0.359345592	AC025171.3
ENSG00000204152	94.98627059	125.9586436	-0.407746204	0.24338572	0.359345592	TIMM23B
ENSG00000143514	670.1733955	797.6051194	-0.25096449	0.243400175	0.359346513	TP53BP2
ENSG00000221944	121.981794	97.04962027	0.329801316	0.243576228	0.359585999	TIGD1
ENSG00000120675	883.1476898	728.631077	0.277475524	0.243636366	0.359645788	DNAJC15
ENSG00000259381	6.09662866	2.231278821	1.467454537	0.243644414	0.359645788	AC090695.1
ENSG00000280388	7.635900881	13.73719547	-0.848634101	0.243782749	0.359829542	AC006330.1
ENSG00000077782	1758.549916	2817.929001	-0.680257812	0.243844256	0.359899882	FGFR1
ENSG00000232677	275.394792	205.1677939	0.423444743	0.243918323	0.359975029	LINC00665
ENSG00000259605	22.70737469	38.63780151	-0.763600579	0.243923689	0.359975029	AC074212.1
novel.915	2718.310717	4437.742907	-0.707105512	0.24394843	0.359975029	-
ENSG00000157933	2057.749969	2533.814164	-0.300336666	0.243950592	0.359975029	SKI
ENSG00000243289	14.91656231	7.103566298	1.069100351	0.243991705	0.359998439	AGAP13P
ENSG00000251370	2.96013129	0.387220594	2.940249619	0.243994169	0.359998439	SEMA5A-AS1
ENSG00000279110	5.688735607	1.813376645	1.656157408	0.244044386	0.360052083	AL022323.4
ENSG00000267939	6.679349408	13.26982689	-0.997886625	0.244065182	0.360062319	AC008946.1
ENSG00000188388	0.307102255	2.50754145	-2.945717359	0.244125807	0.360131308	GOLGA6L3
ENSG00000259244	3.615920022	0.693008929	2.361978876	0.244232563	0.360268337	AC048382.2
ENSG00000105290	264.7446669	206.2319698	0.359810673	0.244308064	0.360359249	APLP1
ENSG00000255302	6178.93119	5084.297762	0.281293909	0.244437238	0.360529315	EID1

ENSG00000101405	3.063429386	0.743759607	2.065396325	0.24451324	0.360620941	OXT
ENSG00000145715	734.3737506	868.5056582	-0.242375094	0.244584561	0.360705653	RASA1
ENSG00000110628	24.4417468	44.65270916	-0.866493001	0.245042859	0.361361024	SLC22A18
ENSG00000206560	1377.482505	1725.249776	-0.324665806	0.245205132	0.361579802	ANKRD28
ENSG00000173451	136.1546258	97.65272063	0.479992209	0.245337884	0.361755027	THAP2
ENSG00000277039	2.962638588	0.336469917	2.941343519	0.245390597	0.361812219	RF00017
ENSG00000231731	47.08078338	28.9644787	0.698679298	0.245528154	0.361994494	AC010976.1
ENSG00000143552	9.355015392	4.575955752	1.021918005	0.245641648	0.362141273	NUP210L
ENSG00000240440	0.616711807	2.884149558	-2.23627589	0.24570911	0.36220323	AC007879.4
ENSG00000242352	3.883945353	1.10029862	1.836382179	0.245711556	0.36220323	FAM91A3P
ENSG00000260285	50.89194548	33.66876458	0.60129593	0.245735224	0.362217568	AL133367.1
ENSG00000229512	14.17914351	7.806031838	0.856496478	0.245768466	0.362246015	AC068580.1
ENSG00000274897	11.25056725	19.17860968	-0.773916882	0.245784882	0.36224966	PANO1
ENSG00000282995	9.537436252	3.495726228	1.440316191	0.24598384	0.362522329	FRG1EP
ENSG00000272345	20.98317432	8.928839698	1.222969716	0.246206956	0.362830568	AL031775.1
ENSG00000258738	41.97623563	28.54387959	0.562942785	0.246262669	0.362892088	AL121603.2
ENSG00000152705	7.503626321	2.731897526	1.43633164	0.246420159	0.36310357	CATSPER3
ENSG00000188981	13.44151126	6.888666832	0.971375926	0.246437175	0.363108049	MSANTD1
ENSG00000279500	9.699940923	16.39583308	-0.752882656	0.24653216	0.363227405	AC108704.2
ENSG00000143217	7.572862087	1.874739808	2.028928481	0.246624957	0.363342329	NECTIN4
ENSG00000272153	24.46807343	14.33114319	0.763542059	0.2466441	0.363342329	AL365330.1
ENSG00000223842	0.601596751	3.108505634	-2.373606754	0.24666543	0.363342329	AL360093.1
ENSG00000171757	59.61179999	82.1766927	-0.464048722	0.246666102	0.363342329	LRRC34
ENSG00000224848	29.95167996	17.4760626	0.781055099	0.246745478	0.363438646	AL589843.1
ENSG00000162927	161.5900354	129.3280475	0.319523005	0.246882759	0.363620235	PUS10
ENSG00000148337	1666.375093	1998.425869	-0.262041805	0.24691919	0.363653278	CIZ1
ENSG00000087299	114.6095446	142.7747718	-0.317942577	0.246940025	0.363663349	L2HGDH
ENSG00000163681	801.6156811	970.3675086	-0.27555462	0.247004684	0.363737953	SLMAP
ENSG00000237523	32.15788156	46.25632342	-0.522729896	0.247067089	0.36380923	LINC00857
ENSG00000285692	5.337541832	1.487519214	1.863617785	0.247207977	0.363996058	AC023509.5
ENSG00000280916	44.63749822	18.63083631	1.25796334	0.247243272	0.364027397	FOXCUT
ENSG00000254154	12.30762479	6.410557368	0.939617069	0.247263799	0.364031139	CRYZL2P-SEC16B
ENSG00000264635	1.203193503	3.882946823	-1.691572875	0.247274519	0.364031139	AP001020.2
ENSG00000157617	1111.82487	1493.869192	-0.426189655	0.247287847	0.364031139	C2CD2
ENSG00000267219	6.355878449	2.221822211	1.532284822	0.247306917	0.364038584	AC010504.1
ENSG00000165219	1452.182762	1125.600448	0.367654876	0.247340431	0.36406729	GAPVD1
ENSG00000142178	43.05801014	105.4020128	-1.291042495	0.247372572	0.364088159	SIK1
ENSG00000152104	1350.469926	1682.301981	-0.316885475	0.247382636	0.364088159	PTPN14
ENSG00000080200	392.2810704	300.7099754	0.384021214	0.247474758	0.364194225	CRYBG3
ENSG00000176659	65.09401068	32.18697756	1.019561099	0.247486036	0.364194225	C20orf197
ENSG00000186283	643.2418078	845.7544108	-0.395262864	0.247496757	0.364194225	TOR3A
ENSG00000228323	6.652880242	2.954969332	1.188561089	0.247524837	0.364214917	AC008440.1
ENSG00000183943	538.5776492	408.3730971	0.399675947	0.247550983	0.36423276	PRKX
ENSG00000196420	7.547717841	3.647978261	1.059874439	0.247667023	0.364371167	S100A5
ENSG00000254419	13.55867076	7.878007486	0.783750288	0.2476731	0.364371167	AL139349.1
ENSG00000174473	7.944328055	2.42610919	1.697283529	0.247702946	0.364389318	GALNTL6
ENSG00000216588	0.616711807	2.934900236	-2.256986066	0.247727513	0.364389318	IGSF23
ENSG00000259709	0.616711807	2.934900236	-2.256986066	0.247727513	0.364389318	AC023906.4
ENSG00000177673	0	2.261960402	-3.753804897	0.247751947	1	TEX44
ENSG00000231971	0	2.261960402	-3.753804897	0.247751947	1	CT69
ENSG00000248530	0	2.261960402	-3.753804897	0.247751947	1	BCL2L12P1

ENSG00000236457	0	2.261960402	-3.753804897	0.247751947	1	AC090617.1
ENSG00000170231	0	2.261960402	-3.753804897	0.247751947	1	FABP6
ENSG00000257477	0	2.261960402	-3.753804897	0.247751947	1	LINC01154
novel.814	164.6254858	101.3929664	0.698659552	0.24788282	0.364597121	-
ENSG00000225484	172.3977496	213.8706421	-0.312086339	0.247946111	0.364669567	NUTM2B-AS1
ENSG00000273338	3.925529575	8.314822883	-1.079325471	0.24798129	0.364700662	AC103591.3
ENSG00000075142	1488.737641	1734.546449	-0.220460712	0.248009656	0.364721734	SRI
ENSG00000233191	1.788421549	0	3.174828358	0.248015524	1	AC006372.2
ENSG00000203908	1.788421549	0	3.174828358	0.248015524	1	KHDC3L
ENSG00000114455	1.788421549	0	3.174828358	0.248015524	1	HHLA2
ENSG00000276668	1.788421549	0	3.174828358	0.248015524	1	AC104237.3
ENSG00000167210	1.788421549	0	3.174828358	0.248015524	1	LOXHD1
ENSG00000287490	1.788421549	0	3.174828358	0.248015524	1	AC015818.9
ENSG00000232528	1.788421549	0	3.174828358	0.248015524	1	AL109809.1
ENSG00000276180	95.30669227	66.83246253	0.512557743	0.248031209	0.364732785	HIST1H4I
ENSG00000227852	0	2.231278821	-3.735438932	0.248207694	1	RPS29P17
ENSG00000184115	0	2.231278821	-3.735438932	0.248207694	1	AC112229.1
ENSG00000253335	0	2.231278821	-3.735438932	0.248207694	1	AC009884.1
ENSG00000119913	0	2.231278821	-3.735438932	0.248207694	1	TECTB
ENSG00000260701	0	2.231278821	-3.735438932	0.248207694	1	AC025284.1
ENSG00000232300	2.654282684	0	3.743946087	0.248269091	1	FAM215B
novel.401	2.654282684	0	3.743946087	0.248269091	1	-
ENSG00000258928	2.654282684	0	3.743946087	0.248269091	1	AL358333.2
ENSG00000260776	2.653029035	0	3.743301458	0.248388271	1	AC104758.3
ENSG00000279042	2.653029035	0	3.743301458	0.248388271	1	AP005242.4
ENSG00000200753	2.653029035	0	3.743301458	0.248388271	1	RF00275
ENSG00000112739	1588.043217	1354.954729	0.228996476	0.248434705	0.365305454	PRPF4B
ENSG00000232600	3.36043118	8.102363562	-1.277829124	0.248472013	0.365339636	TONSL-AS1
ENSG00000110046	564.555792	704.5207776	-0.319013539	0.248505545	0.365368262	ATG2A
ENSG00000286874	4.202401718	1.080229524	1.963375152	0.248569085	0.365441002	AC072054.1
ENSG00000079101	5.484931447	1.762625967	1.626122839	0.24861608	0.365489412	CLUL1
ENSG00000231861	4.747299191	0.774441189	2.652718043	0.248953004	0.365964014	OR5K2
ENSG00000007545	383.6497553	315.5934814	0.282271432	0.249014421	0.366033588	CRAMP1
ENSG00000219932	0.89358395	3.210006989	-1.83616557	0.249111153	0.366155061	RPL12P8
ENSG00000162607	735.2535017	921.1723762	-0.325556301	0.249177044	0.366231192	USP1
ENSG00000081026	219.2028432	170.4172456	0.362290878	0.249268251	0.36634452	MAGI3
ENSG00000274515	6.596180964	2.435565801	1.423077655	0.249371468	0.366475485	AC105020.5
ENSG00000129559	48.43477144	26.02102094	0.898230773	0.249407718	0.366502887	NEDD8
ENSG00000248127	2.461832635	0	3.632113548	0.249409231	1	AC026774.1
ENSG00000254965	2.461832635	0	3.632113548	0.249409231	1	C1DP5
ENSG00000259724	2.461832635	0	3.632113548	0.249409231	1	LINC01581
ENSG00000211640	2.461832635	0	3.632113548	0.249409231	1	IGLV6-57
ENSG00000268401	6.248748136	1.803920035	1.797286171	0.249418327	0.366502887	AC007785.3
ENSG00000178015	19.11792384	11.69013454	0.703081382	0.249663031	0.366841715	GPR150
ENSG00000125895	10.01205777	22.67151058	-1.174668786	0.249756178	0.366957828	TMEM74B
ENSG00000230155	6.93476698	2.985650913	1.238607955	0.249795161	0.3669785	FO393401.1
ENSG00000285665	15.15464226	9.019600173	0.751204847	0.249798498	0.3669785	AL031274.1
ENSG00000130818	312.5041326	373.1265323	-0.255492616	0.24988522	0.367084561	ZNF426
ENSG00000261136	14.92060799	8.500196642	0.80396123	0.24989895	0.367084561	AC023908.3
ENSG00000127922	1215.633812	1457.139407	-0.261489896	0.249923319	0.367099602	SEM1
ENSG00000187288	3.328947419	8.286581446	-1.323964541	0.250061805	0.367276496	CIDEC

ENSG00000129219	749.3819871	575.6529539	0.381049156	0.250072022	0.367276496	PLD2
novel.924	62.42600878	156.4953058	-1.325672329	0.250193028	0.367415357	-
ENSG00000114019	2880.582024	3765.236259	-0.386429749	0.250194854	0.367415357	AMOTL2
ENSG00000184497	160.7272941	110.5121757	0.542399688	0.250263235	0.367495004	TMEM255B
ENSG00000213983	311.9963464	256.8319302	0.280361946	0.250364855	0.367623449	AP1G2
ENSG00000285331	36.71684765	23.12651568	0.660036069	0.250453339	0.367732503	AC090517.5
ENSG00000267065	5.055655094	1.161661783	2.159264106	0.250467433	0.367732503	LINC02080
ENSG00000124343	1.511549406	5.158006716	-1.778662025	0.25058276	0.367881036	XG
ENSG00000213160	88.38661342	120.6287009	-0.450715896	0.250665802	0.367982156	KLHL23
novel.864	60.60821202	119.3231219	-0.978027998	0.250860755	0.368247543	-
ENSG00000129315	586.3609498	704.8041032	-0.265291832	0.250927862	0.368325242	CCNT1
ENSG00000155269	0.307102255	2.435565801	-2.909043506	0.250957689	0.368348212	GPR78
ENSG00000180440	2.463086283	0	3.632815968	0.250984009	1	SERTM1
ENSG00000230725	2.463086283	0	3.632815968	0.250984009	1	AL035252.2
ENSG00000242534	2.463086283	0	3.632815968	0.250984009	1	IGKV2D-28
ENSG00000236469	2.463086283	0	3.632815968	0.250984009	1	AC007881.2
ENSG00000189152	2.463086283	0	3.632815968	0.250984009	1	GRAPL
ENSG00000198598	1061.288642	682.8986616	0.63643086	0.251090596	0.368522471	MMP17
ENSG00000256258	2.460578986	0	3.631431984	0.251250342	1	AC117500.3
ENSG00000241186	2.460578986	0	3.631431984	0.251250342	1	TDGF1
ENSG00000211801	2.460578986	0	3.631431984	0.251250342	1	TRAV21
ENSG00000163081	2.460578986	0	3.631431984	0.251250342	1	CCDC140
ENSG00000249459	29.4900222	41.8822145	-0.505830617	0.25129014	0.368794505	ZNF286B
ENSG00000214338	1.774560141	5.73646166	-1.683162103	0.251427026	0.36896979	SOGA3
ENSG00000287165	3.636049674	1.10029862	1.738976029	0.251457449	0.36896979	AL078621.4
ENSG00000105655	693.5152448	539.2977551	0.362551136	0.251464248	0.36896979	ISYNA1
ENSG00000274598	0.586481695	3.983292303	-2.748223695	0.251466382	0.36896979	AC087893.1
ENSG00000196372	310.5911287	391.0367134	-0.332137036	0.25197128	0.369689734	ASB13
ENSG00000224609	11.5274394	6.175588807	0.906105962	0.252166725	0.369954178	AC093425.1
ENSG00000229320	114.2484638	87.21556911	0.387246097	0.252179997	0.369954178	KRT8P12
ENSG00000168397	1180.962501	1390.634715	-0.235621292	0.25225931	0.370047402	ATG4B
novel.811	10.01456507	16.41834232	-0.716179041	0.252272029	0.370047402	-
ENSG00000231829	3.082305388	0.387220594	2.994110046	0.252307068	0.370077906	AL157834.1
ENSG00000085449	932.7614099	1102.48597	-0.241356616	0.252343675	0.370110705	WDFY1
ENSG00000253180	5.39424111	2.129777466	1.336432224	0.252358737	0.370111902	AC104986.1
ENSG00000188672	11.65434336	20.69783795	-0.822314873	0.252378087	0.370119388	RHCE
ENSG00000140463	465.699821	362.5631525	0.360301446	0.252409524	0.370144597	BBS4
ENSG00000274183	0	2.221822211	-3.729559012	0.252410256	1	H2AFB1
ENSG00000223586	0	2.221822211	-3.729559012	0.252410256	1	LINC01312
ENSG00000163736	4.903535618	1.069617038	2.191127679	0.252506151	0.370265397	PPBP
ENSG00000189144	37.43900884	26.36232396	0.509128182	0.252536275	0.370288672	ZNF573
ENSG00000117507	2.15473038	8.512093397	-1.991097734	0.252569911	0.370317091	FMO6P
ENSG00000126777	3110.167178	3633.202284	-0.224255785	0.252752681	0.370564156	KTN1
ENSG00000153767	194.1534449	238.1716154	-0.29576521	0.252791306	0.370599871	GTF2E1
ENSG00000272333	986.3124266	1239.316162	-0.32918392	0.252839961	0.370650286	KMT2B
ENSG00000287731	0.308355904	2.415496705	-2.898469743	0.252885288	0.370695817	AL035603.1
ENSG00000241973	1354.178118	1693.76657	-0.322613647	0.25290543	0.370704428	PI4KA
ENSG00000107872	457.5088791	363.5518855	0.331705562	0.252959096	0.370762172	FBXL15
ENSG00000287986	2.139615324	0.336469917	2.4670536	0.25300228	1	AL135841.1
ENSG00000273367	4.398612713	0.336469917	3.51058082	0.253047794	0.370871255	AL355472.3
ENSG00000224040	1.233423615	4.657388011	-1.925573993	0.253140407	0.370986062	HMGN1P4

ENSG00000165629	2495.758074	2894.167123	-0.213728796	0.25317117	0.371010218	ATP5F1C
ENSG00000218631	3.636049674	1.110911105	1.732883495	0.253290848	0.371164666	AL117344.1
ENSG00000127993	303.2737276	253.8998556	0.256997888	0.253308346	0.371169372	RBM48
ENSG00000229951	5.292196663	15.25539627	-1.520933036	0.253369024	0.371237345	AC104695.2
ENSG00000158301	151.63281	215.240478	-0.505342396	0.253493422	0.371398669	GPRASP2
ENSG00000153822	0.293240848	2.415496705	-2.898447774	0.253534262	0.371437559	KCNJ16
ENSG00000281649	1205.277973	960.089018	0.328438186	0.253617957	0.371539224	EBLN3P
ENSG00000148408	6.094121362	2.200597239	1.478887074	0.253673251	0.371578727	CACNA1B
ENSG00000198774	56.58028129	19.8564298	1.507423305	0.253673526	0.371578727	RASSF9
ENSG00000138798	3.3831394	8.775303396	-1.384434831	0.253700132	0.371590893	EGF
ENSG00000104518	65.11338523	112.1572253	-0.786274837	0.253710436	0.371590893	GSDMD
ENSG00000163714	2395.821765	1905.536275	0.330342289	0.25373982	0.371612979	U2SURP
ENSG00000204604	182.1162815	138.2782405	0.39549856	0.253809953	0.37169474	ZNF468
ENSG00000139428	443.7935884	340.5522998	0.381269088	0.253885854	0.371774902	MMAB
ENSG00000253305	26.12332277	37.42196861	-0.518831319	0.25389331	0.371774902	PCDHGB6
ENSG00000236409	6.893182758	2.853467977	1.271525732	0.2539166	0.371788051	NRADDP
ENSG00000172062	2.388764651	6.73513053	-1.48107822	0.253971936	0.371848118	SMN1
ENSG00000274849	0.909952655	4.117915384	-2.186570331	0.254004346	0.371874615	AC023043.4
ENSG00000249965	2.111892509	0.387220594	2.45131566	0.25405632	1	CDC42P4
ENSG00000157343	15.48814241	26.72489914	-0.791246128	0.254252037	0.372185339	ARMC12
ENSG00000203772	16.40694188	25.31277602	-0.624660316	0.254256922	0.372185339	SPRN
ENSG00000171148	1771.648097	1525.592586	0.215801498	0.254262255	0.372185339	TADA3
ENSG00000111678	631.0090307	495.5772149	0.348508514	0.254273883	0.372185339	C12orf57
ENSG00000213185	33.82817469	49.00371372	-0.537702224	0.25434637	0.372259938	FAM24B
ENSG00000174225	3.88645265	1.049547942	1.874629832	0.254353505	0.372259938	ARL13A
ENSG00000074356	1371.59867	1095.950315	0.323840939	0.254416028	0.37231315	NCBP3
novel.126	10.06875705	4.698682079	1.101460601	0.254418524	0.37231315	-
ENSG00000249936	56.60234912	74.06260796	-0.38978235	0.254446046	0.372332454	RAC1P2
ENSG00000129355	214.1857669	264.1357211	-0.3021438	0.254494645	0.372353257	CDKN2D
ENSG00000286945	4.105371866	0	4.372244375	0.254503258	0.372353257	AL513478.4
ENSG00000169836	4.105371866	0	4.372244375	0.254503258	0.372353257	TACR3
ENSG00000141337	262.9932576	365.3012705	-0.473248072	0.254625249	0.372491476	ARSG
novel.705	4.882081047	9.743290684	-0.998554374	0.254626405	0.372491476	-
ENSG00000119392	453.2502481	551.4366358	-0.283338485	0.25478698	0.372705395	GLE1
ENSG00000236833	6.912130031	1.682349583	2.015620713	0.254803607	0.372708733	AC024560.1
ENSG00000273289	5.156445892	1.702418679	1.568290385	0.254893066	0.372818598	AL121672.3
ENSG00000180263	358.547315	206.7825214	0.793793808	0.254909332	0.372821401	FGD6
ENSG00000141577	414.3519325	526.8054047	-0.346220884	0.255054901	0.372999542	CEP131
ENSG00000201916	0	2.200597239	-3.716960175	0.255060623	1	RF00019
ENSG00000279372	0	2.200597239	-3.716960175	0.255060623	1	AC137723.1
ENSG00000248416	0	2.200597239	-3.716960175	0.255060623	1	AC107464.2
ENSG00000254814	0	2.200597239	-3.716960175	0.255060623	1	AP003031.1
ENSG00000254710	0	2.200597239	-3.716960175	0.255060623	1	AP001970.1
ENSG00000236456	0	2.200597239	-3.716960175	0.255060623	1	AL035458.1
ENSG00000268970	33.39005152	20.96104795	0.679491164	0.255063231	0.372999542	AC022150.2
ENSG00000239523	32.52411912	20.61653409	0.655669189	0.255074203	0.372999542	MYLK-AS1
ENSG00000247675	3.605819561	7.775350256	-1.110077189	0.255174866	0.373125742	LRP4-AS1
ENSG00000230637	2.991615051	0.743759607	2.036784026	0.255264751	0.373236169	AL022324.1
ENSG00000282890	2.76893489	0.336469917	2.83923161	0.255280114	0.373237627	AC009975.1
ENSG00000217950	0.308355904	2.455634897	-2.919428046	0.255389686	0.373376818	NOC2LP2
ENSG00000258580	2.406387005	0	3.601126285	0.255406239	1	AL136298.1

ENSG00000280409	2.406387005	0	3.601126285	0.255406239	1	LINC01101
ENSG00000287972	2.406387005	0	3.601126285	0.255406239	1	AC073648.7
ENSG00000138622	2.406387005	0	3.601126285	0.255406239	1	HCN4
ENSG00000275576	0	2.171071533	-3.698528734	0.255421145	1	AL049539.1
ENSG00000279034	0	2.171071533	-3.698528734	0.255421145	1	AC011266.1
ENSG00000267439	0	2.171071533	-3.698528734	0.255421145	1	AD000671.3
novel.839	0	2.171071533	-3.698528734	0.255421145	1	-
ENSG00000159650	2.421502061	0	3.609633088	0.255468772	1	UROC1
ENSG00000260510	2.421502061	0	3.609633088	0.255468772	1	AC004381.1
ENSG00000258273	3.297463658	0.774441189	2.142204527	0.255575477	0.373627418	AC024257.2
ENSG00000260608	7.486003968	2.557136252	1.557942939	0.255614406	0.373663302	AC024270.4
ENSG00000230626	7.255730642	12.76907979	-0.808872025	0.255636081	0.373673962	AC011005.1
ENSG00000228318	1.817398012	4.586568238	-1.342417103	0.255816338	0.373916415	AP001610.1
ENSG00000233351	2.140868972	0.387220594	2.467796429	0.255864887	1	AL356124.2
ENSG00000204969	4.601447958	1.069617038	2.098795698	0.255945954	0.374084824	PCDHA2
ENSG00000229379	2.40137241	0	3.598301184	0.255958875	1	AC006041.1
ENSG00000202399	2.40137241	0	3.598301184	0.255958875	1	RF00019
ENSG00000172139	2.40137241	0	3.598301184	0.255958875	1	SLC9C1
ENSG00000151239	1731.34536	2042.78171	-0.238780814	0.256018786	0.374170224	TWF1
ENSG00000137804	3.928036872	8.060941101	-1.028583412	0.256114759	0.374289434	NUSAP1
ENSG00000269534	1.49643435	4.941951375	-1.722064628	0.256136373	0.374299967	AC011466.3
ENSG00000287688	2.138361675	0.387220594	2.466384259	0.256136479	1	AC011317.1
ENSG00000149654	2.138361675	0.387220594	2.466384259	0.256136479	1	CDH22
novel.9	2.415233817	0	3.606118451	0.256154481	1	-
novel.301	2.415233817	0	3.606118451	0.256154481	1	-
ENSG00000162897	2.415233817	0	3.606118451	0.256154481	1	FCAMR
ENSG00000219607	47.86153824	31.98641499	0.587214514	0.256199944	0.374371808	PPP1R3G
ENSG00000185499	56.48630072	93.83675359	-0.733885264	0.25629585	0.374490889	MUC1
ENSG00000107874	2182.884139	1721.398916	0.342648244	0.256393838	0.374612997	CUEDC2
ENSG00000261790	17.62879792	9.293551051	0.924318749	0.256499138	0.374745774	AC005606.2
ENSG00000127580	235.243405	286.4779538	-0.284052993	0.256564925	0.374820811	WDR24
ENSG00000006652	1059.306959	854.1199247	0.310503756	0.2566012	0.374821484	IFRD1
ENSG00000147439	301.2525689	385.0283086	-0.35314729	0.256606959	0.374821484	BIN3
ENSG00000235174	884.5974106	1108.589293	-0.325686733	0.256617944	0.374821484	RPL39P3
ENSG00000269821	235.1469735	157.6111912	0.577080527	0.256623092	0.374821484	KCNQ1OT1
ENSG00000279484	0.601596751	3.168712922	-2.397346609	0.256648849	0.374838031	KLHL30-AS1
novel.271	3.076037145	11.10821197	-1.858427103	0.256673679	0.374853224	-
ENSG00000136002	158.6785402	111.1029598	0.514484018	0.256759092	0.374956886	ARHGEF4
ENSG00000277311	2.464339932	0	3.633545673	0.256765916	1	RF02247
ENSG00000174450	2.464339932	0	3.633545673	0.256765916	1	GOLGA6L2
ENSG00000268869	2.464339932	0	3.633545673	0.256765916	1	ESPNP
ENSG00000260719	2.464339932	0	3.633545673	0.256765916	1	AC009133.3
ENSG00000256577	2.464339932	0	3.633545673	0.256765916	1	AC007406.4
ENSG00000188393	2.464339932	0	3.633545673	0.256765916	1	CLEC2A
ENSG00000197442	646.4212856	552.1554461	0.22703523	0.256862932	0.375087445	MAP3K5
ENSG00000265490	1.466204238	5.482579877	-1.886988116	0.256901895	0.375111945	AP001178.2
ENSG00000236393	2.766427592	8.213193133	-1.568822552	0.256938365	0.375111945	AC091806.1
ENSG00000257354	277.7272012	184.1309046	0.593741783	0.256940125	0.375111945	AC048341.1
ENSG00000196821	1525.41236	1903.395604	-0.319297086	0.256950255	0.375111945	ILRUN
ENSG00000077092	531.6614602	441.527946	0.267796557	0.256951899	0.375111945	RARB
ENSG00000176871	1466.406743	1161.255544	0.336334875	0.256993943	0.375142712	WSB2

ENSG00000244738	3.931797819	1.10029862	1.850417482	0.257001852	0.375142712	AC026316.3
ENSG00000255326	6.022307028	2.537067156	1.25428772	0.257025256	0.375155797	AP001922.5
ENSG00000280744	0	2.292641984	-3.772271054	0.257044293	1	LINC01173
ENSG00000132693	0	2.292641984	-3.772271054	0.257044293	1	CRP
ENSG00000227432	0	2.292641984	-3.772271054	0.257044293	1	AC053503.1
ENSG00000243572	0	2.292641984	-3.772271054	0.257044293	1	LINC02017
ENSG00000286988	0	2.292641984	-3.772271054	0.257044293	1	AC024933.2
ENSG00000276176	0	2.292641984	-3.772271054	0.257044293	1	MIR6090
ENSG00000268889	0	2.292641984	-3.772271054	0.257044293	1	AC008750.7
ENSG00000197111	3440.013119	3000.459368	0.197222206	0.257200158	0.375389996	PCBP2
ENSG00000158089	12.41217653	23.98670866	-0.944679898	0.257223421	0.375402859	GALNT14
ENSG00000236662	2.138361675	0.356539013	2.466397364	0.257293316	1	AL133215.1
ENSG00000198502	2.459325337	0	3.630772964	0.257300647	1	HLA-DRB5
ENSG00000187621	2.459325337	0	3.630772964	0.257300647	1	TCL6
ENSG00000285876	2.459325337	0	3.630772964	0.257300647	1	AC093912.1
ENSG00000125084	2.459325337	0	3.630772964	0.257300647	1	WNT1
ENSG00000250921	2.459325337	0	3.630772964	0.257300647	1	LINC02063
ENSG00000241657	2.459325337	0	3.630772964	0.257300647	1	TRBV11-2
ENSG00000238363	1.480065645	6.22749536	-2.062880784	0.257316151	0.375517099	SNORA13
ENSG00000134480	452.4604333	533.9020829	-0.239068292	0.25741972	0.375640373	CCNH
ENSG00000181690	367.0876327	256.9708728	0.514837632	0.257429539	0.375640373	PLAG1
ENSG00000224677	1.7871679	5.064677702	-1.492128356	0.257505627	0.375730298	PDIA3P2
ENSG00000241907	0.586481695	3.749479616	-2.65577447	0.257565937	0.375764074	RPS20P4
ENSG00000255921	0.586481695	3.749479616	-2.65577447	0.257565937	0.375764074	AC026310.2
ENSG00000207751	1.541779519	5.696323468	-1.893732967	0.257572164	0.375764074	AP000553.1
ENSG00000261240	1.215801261	3.566546002	-1.557913466	0.25758764	0.375765551	AC009065.6
ENSG00000269794	1.214547612	4.004517274	-1.71903754	0.257623986	0.375797473	AC010642.1
ENSG00000233087	4.585079253	1.406086955	1.690330517	0.257656349	0.37582358	RAB6D
ENSG00000164542	83.28894717	56.5459748	0.561807023	0.257694028	0.375857437	KIAA0895
ENSG00000214018	2.140868972	0.336469917	2.467821036	0.257784557	1	RRM2P3
ENSG00000260394	3.594465451	0.743759607	2.295240076	0.257922297	0.376169261	Z92544.1
ENSG00000113722	5.841211088	1.00940975	2.506821023	0.25800028	0.376261873	CDX1
ENSG00000138411	79.86415228	118.8745384	-0.571975231	0.258028858	0.376268458	HECW2
ENSG00000286331	1.789675198	5.064677702	-1.490870282	0.25803376	0.376268458	AC011388.1
ENSG00000168955	1.218308559	4.22887335	-1.803029089	0.258097751	0.37633309	TM4SF20
ENSG00000171262	724.3090956	910.2746502	-0.329962384	0.258107052	0.37633309	FAM98B
ENSG00000179168	17.70938779	27.89113725	-0.650518458	0.258224727	0.376483537	GGN
ENSG00000246863	0.894837599	3.230076085	-1.842564414	0.25829128	0.376559438	AC012377.1
ENSG00000186399	3.588197208	0.743759607	2.293009001	0.258351667	0.376626341	GOLGA8R
ENSG00000110315	1518.342068	1107.947046	0.454379969	0.258510036	0.376836069	RNF141
ENSG00000196743	1968.85289	1538.114411	0.356405077	0.258524871	0.376836551	GM2A
ENSG00000178723	2.391271949	0	3.592562984	0.258805753	1	GLULP4
ENSG00000279430	2.391271949	0	3.592562984	0.258805753	1	AL590560.3
ENSG00000285750	2.391271949	0	3.592562984	0.258805753	1	AL157385.2
ENSG00000231401	2.391271949	0	3.592562984	0.258805753	1	AC023481.1
ENSG00000262119	2.391271949	0	3.592562984	0.258805753	1	AL079343.1
ENSG00000182854	2.391271949	0	3.592562984	0.258805753	1	OR4F15
ENSG00000249489	2.391271949	0	3.592562984	0.258805753	1	GAPDHP70
ENSG00000166228	482.8750518	629.1443283	-0.382184769	0.25880581	0.377224027	PCBD1
ENSG00000162630	338.513599	82.43387935	2.037468043	0.258819734	0.377224027	B3GALT2
ENSG00000286216	12.39072196	5.971430221	1.044653285	0.258867868	0.377273018	AC104335.1

ENSG00000251259	23.68287344	14.70518276	0.695827225	0.258945629	0.377362455	AC004069.1
ENSG00000157315	14.45100106	8.225089889	0.813120611	0.258958285	0.377362455	TMED6
ENSG00000244050	2.123246619	0.356539013	2.45779084	0.258961217	1	DEFB109F
ENSG00000151475	2.123246619	0.356539013	2.45779084	0.258961217	1	SLC25A31
ENSG00000138398	1254.408433	1089.258627	0.20359385	0.258991249	0.377380134	PPIG
ENSG00000215644	7.092185785	1.049547942	2.747053739	0.258999468	0.377380134	GCGR
ENSG00000285130	16.01165658	9.620564358	0.732012067	0.25920051	0.377651887	AL358113.1
ENSG00000228600	2.387511003	0	3.590431755	0.259224995	1	POLR2CP1
ENSG00000286715	2.387511003	0	3.590431755	0.259224995	1	AL589863.2
ENSG00000226235	2.387511003	0	3.590431755	0.259224995	1	LEMD1-AS1
ENSG00000288026	2.387511003	0	3.590431755	0.259224995	1	AC096996.3
ENSG00000265737	2.387511003	0	3.590431755	0.259224995	1	AP006565.1
ENSG00000275392	2.387511003	0	3.590431755	0.259224995	1	AL445493.3
ENSG00000204779	2.387511003	0	3.590431755	0.259224995	1	FOXD4L5
ENSG00000255974	3.254625787	0.743759607	2.157104197	0.259288864	0.377754617	CYP2A6
ENSG00000125351	351.2297175	285.6733786	0.298077847	0.259300097	0.377754617	UPF3B
ENSG00000188828	2.127007565	0.336469917	2.459923107	0.259319866	1	GLRA4
ENSG00000152443	245.7819115	198.1958497	0.309675299	0.259420037	0.377908157	ZNF776
ENSG00000197535	1230.226881	956.0699067	0.363969297	0.259496877	0.377998899	MYO5A
ENSG00000185607	4.469173399	1.00940975	2.115472923	0.259615964	0.378151167	ACTBP7
ENSG00000116584	1658.617369	1986.315495	-0.260032564	0.259647568	0.378175999	ARHGEF2
ENSG00000160447	136.6877141	184.0466127	-0.429022143	0.259685943	0.378210689	PKN3
ENSG00000101307	45.77256706	16.33202977	1.488875485	0.259718758	0.378237278	SIRPB1
novel.195	2.436617117	0	3.618110733	0.259747659	1	-
ENSG00000279952	2.436617117	0	3.618110733	0.259747659	1	AC068787.1
ENSG00000216360	2.436617117	0	3.618110733	0.259747659	1	AL136968.1
ENSG00000253618	2.708474665	0.356539013	2.810213216	0.259937371	0.378534434	GRPEL2-AS1
ENSG00000167281	7.507387268	3.280826763	1.203899545	0.259965586	0.378554304	RBFOX3
ENSG00000265625	2.694613258	0.387220594	2.803498889	0.260066884	0.378680587	AC104564.5
ENSG00000226964	282.8974494	341.5637514	-0.272656224	0.26010516	0.378715095	RHEBP2
ENSG00000174564	26.32573074	15.1941615	0.788525133	0.260129453	0.378718021	IL20RB
ENSG00000227321	3.299970955	0.713078025	2.206594659	0.260136323	0.378718021	MTND4P15
ENSG00000205726	1547.673479	1233.952703	0.3265501	0.260198847	0.378787821	ITSN1
ENSG00000185798	136.3813239	171.3291827	-0.32909423	0.260216202	0.378789969	WDR53
ENSG00000163491	13.546063	21.59641813	-0.674795866	0.260229481	0.378789969	NEK10
ENSG00000207741	3.857476187	10.15888111	-1.38968614	0.260417259	0.379042061	MIR590
ENSG00000182950	10.8426742	5.748230021	0.91775328	0.260446417	0.379063266	ODF3L1
ENSG00000149313	700.814898	880.8114447	-0.330147784	0.260492223	0.379108697	AASDHPPT
ENSG00000105427	12.99231871	6.307900138	1.033457233	0.260526226	0.379136945	CNFN
ENSG00000267784	2.429095225	0	3.613907945	0.260564704	1	AC010680.1
ENSG00000277504	2.429095225	0	3.613907945	0.260564704	1	AC010536.3
ENSG00000233235	2.429095225	0	3.613907945	0.260564704	1	BX119321.1
ENSG00000276203	2.429095225	0	3.613907945	0.260564704	1	ANKRD20A3
ENSG00000239322	2.729857965	0.387220594	2.820519991	0.260576917	0.379189474	ATP6V1B1-AS1
ENSG00000273784	5.379126054	2.160459047	1.321882873	0.260639414	0.379259177	AL137058.2
ENSG00000253414	11.65204952	4.545274171	1.348388719	0.260668627	0.379280444	AC124067.2
ENSG00000135506	3713.08614	4424.496342	-0.252860535	0.260688466	0.379288068	OS9
ENSG00000231645	2.699627853	0.356539013	2.805914264	0.26073966	0.37934131	KRT17P6
ENSG00000134056	229.0242306	321.7138245	-0.490575648	0.260831328	0.379445348	MRPS36
ENSG00000165887	7.031725561	17.8632832	-1.345381651	0.26084038	0.379445348	ANKRD2
ENSG00000156222	2.095523804	0.387220594	2.441924935	0.26086839	1	SLC28A1

ENSG00000188624	2.095523804	0.387220594	2.441924935	0.26086839	1	IGFL3
ENSG00000242953	3.373038938	0.713078025	2.234442297	0.26101318	0.379675463	AC139453.1
ENSG00000277051	3.018084217	0.672939833	2.120100461	0.261183043	0.379890926	RF00017
ENSG00000286376	2.694613258	0.356539013	2.803490255	0.261194134	0.379890926	AC006041.2
ENSG00000257509	12.85126861	6.23695197	1.053506632	0.261220682	0.379890926	AC073487.1
ENSG00000264266	2.710981963	0.693008929	1.940647227	0.261224983	0.379890926	MIR4322
ENSG00000235558	7.0002418	2.038888596	1.757672493	0.261236656	0.379890926	AC146949.1
ENSG00000153060	2.722336072	0.387220594	2.816908329	0.261249035	0.379890926	TEKT5
ENSG00000278420	2.415233817	6.836760279	-1.499785063	0.261295514	0.379937248	MIR6819
ENSG00000242950	14.00277743	4.665688746	1.586131049	0.261420491	0.380087109	ERVW-1
ENSG00000279196	10.27381486	4.494523493	1.177818472	0.261427837	0.380087109	AC135048.4
ENSG00000168509	2.699627853	0.336469917	2.805909586	0.261482371	0.380145123	HJV
ENSG00000261707	6.733541389	1.682349583	1.975864497	0.261594155	0.380275291	AC092134.1
ENSG00000250403	0.615458159	2.527610546	-2.047605262	0.261603528	0.380275291	AC097460.2
ENSG00000176723	37.52622297	22.88923537	0.707784797	0.261615817	0.380275291	ZNF843
ENSG00000132773	217.4215879	293.9298524	-0.435542607	0.261671033	0.380334272	TOE1
ENSG00000287634	4.008626748	0	4.336195274	0.261724787	0.380391121	AC114277.1
ENSG00000163536	56.83505847	91.71296511	-0.692296199	0.261854308	0.380558079	SERPINI1
ENSG00000286864	1.526664462	4.62670643	-1.606233465	0.261871175	0.380561305	AC012076.1
ENSG00000178996	1237.598814	1024.373801	0.273054297	0.261916124	0.380605337	SNX18
ENSG00000242622	46.23282933	33.76106611	0.454226244	0.261958383	0.380645455	AC092910.3
ENSG00000282826	232.7812883	325.2281728	-0.482660707	0.261976953	0.380651151	FRG1CP
ENSG00000171189	18.03035146	10.67956891	0.753766715	0.262054996	0.380743253	GRIK1
ENSG00000285747	2.095523804	0.356539013	2.441907658	0.26206323	1	AL133485.2
ENSG00000140400	2216.999801	1662.706699	0.415178125	0.262080445	0.380758936	MAN2C1
ENSG00000231927	0.921306765	4.159209451	-2.186428591	0.26213215	0.380800833	AC093734.1
ENSG00000273451	2.052685933	7.357319686	-1.828886515	0.262138597	0.380800833	AL031666.3
ENSG00000198542	198.606123	409.8090043	-1.045181998	0.262235935	0.380920933	ITGBL1
ENSG00000249602	5.484931447	2.089639274	1.372928604	0.262381611	0.381111234	AL589765.4
ENSG00000137198	52.86991033	85.47216033	-0.695448778	0.262607797	0.381418447	GMPR
ENSG00000102241	1414.779803	1195.157466	0.243094037	0.262629902	0.381429229	HTATSF1
novel.548	3.992329314	0	4.33045405	0.262754098	0.381566946	-
ENSG00000260653	3.992329314	0	4.33045405	0.262754098	0.381566946	AC237221.1
ENSG00000257453	1.49643435	4.63616304	-1.627467976	0.262844929	0.381660237	AC011611.3
ENSG00000197779	117.4657371	82.21477593	0.514028496	0.262847721	0.381660237	ZNF81
ENSG00000214359	2.095523804	0.336469917	2.441896291	0.262854973	1	RPL18P10
ENSG00000225185	2.095523804	0.336469917	2.441896291	0.262854973	1	PPIAP8
ENSG00000104343	591.6400245	506.3194181	0.225058213	0.262915062	0.381736684	UBE2W
ENSG00000257222	7.88379656	3.862877727	1.015785813	0.262995664	0.381832375	AC079907.1
ENSG00000265194	20.92500793	10.84127755	0.951875498	0.263020764	0.381847479	AL359922.2
ENSG00000167632	420.0443418	345.7981945	0.279887907	0.263089697	0.381926213	TRAPPC9
ENSG00000172890	1257.082678	1551.408468	-0.303433032	0.263113908	0.381940018	NADSYN1
ENSG00000231966	3.910414519	1.130980201	1.821229454	0.263133618	0.381944687	AL359853.2
ENSG00000161671	3143.862062	2692.236657	0.223642576	0.263146526	0.381944687	EMC10
ENSG00000227097	3072.820316	1871.715125	0.715298021	0.263215062	0.382022821	RPS28P7
ENSG00000259091	2.157237677	8.592369781	-2.001946353	0.263254007	0.382058003	LINC00517
ENSG00000122547	71.56328733	111.4543617	-0.638513602	0.263269591	0.382059277	EEPD1
ENSG00000105643	908.648247	713.7471299	0.347832668	0.263347064	0.382150362	ARRDC2
ENSG00000100938	39.06277443	54.52412004	-0.484105929	0.263479897	0.382321765	GMPR2
ENSG00000143674	36.86799821	59.97879681	-0.699956723	0.263534429	0.382358771	MAP3K21
novel.834	4.83548223	9.008987688	-0.895014555	0.263534833	0.382358771	-

ENSG00000212283	4.281737945	1.069617038	1.994841875	0.263608828	0.382444771	SNORD89
ENSG00000134548	25.56198533	36.64765581	-0.523742343	0.263640183	0.382468904	SPX
novel.469	27.55421103	53.77595968	-0.967258295	0.263669693	0.382490357	-
ENSG00000277662	3.352909287	8.079854321	-1.26648577	0.263746639	0.382580617	AL354696.1
ENSG00000269191	4.514518568	9.670030766	-1.087594802	0.26381238	0.382654613	AC005387.2
ENSG00000221540	1.215801261	3.822739535	-1.664460126	0.26390141	0.382762381	MIR1180
ENSG00000227518	0.61420451	2.985650913	-2.281290023	0.264130137	0.383072742	AL928970.1
ENSG00000127870	649.5056279	776.7886775	-0.258591823	0.264156136	0.383089063	RNF6
ENSG00000165675	144.2030784	184.2495682	-0.354080401	0.264217366	0.383156473	ENOX2
ENSG00000213236	1.816144364	4.718751175	-1.373239577	0.264265799	0.38320532	YWHAZP2
ENSG00000256340	4.202401718	0.713078025	2.555625215	0.264297164	0.383229413	ABCC6P1
ENSG00000264943	2.947523532	9.882618481	-1.737218353	0.264365357	0.3833069	SH3GL1P2
ENSG00000239739	1.789675198	6.052734086	-1.743775744	0.264414804	0.383354281	AC026316.2
ENSG00000188176	5.224428009	1.161661783	2.200032309	0.264427546	0.383354281	SMTNL2
ENSG00000273542	0.601596751	3.068367442	-2.356968349	0.264558475	0.383522694	HIST1H4K
ENSG00000108465	353.9435068	271.9069145	0.381263058	0.264593948	0.383552718	CDK5RAP3
ENSG00000156009	2.684512796	0.387220594	2.798540628	0.264680821	0.383657242	MAGEA8
ENSG00000226699	5.116115319	1.874739808	1.474765451	0.264730198	0.383707408	AL360181.1
ENSG00000090989	955.2835153	804.5906772	0.247852481	0.264757427	0.383715021	EXOC1
novel.717	169.8587599	282.0005238	-0.732179001	0.264764989	0.383715021	-
ENSG00000279369	92.39294633	69.48305619	0.410099123	0.264808549	0.383756744	AC046185.3
ENSG00000172935	177.4599845	341.4184818	-0.944349497	0.2648683	0.383821925	MRGPRF
ENSG00000271383	1.218308559	3.903015919	-1.686525726	0.264917817	0.383861181	NBPF19
ENSG00000103723	44.39007326	23.61421015	0.906148593	0.264924939	0.383861181	AP3B2
ENSG00000261019	8.043509199	2.965581817	1.454761175	0.265028098	0.383985357	AC010132.4
ENSG00000235609	67.018539	39.77926581	0.756488804	0.265040199	0.383985357	AF127577.4
ENSG00000223658	3.939390982	11.28412911	-1.523794733	0.265072452	0.384010672	C1GALT1C1L
ENSG00000236474	0.879722543	3.658590747	-2.035040904	0.26514478	0.384094035	GCNT1P1
ENSG00000264107	7.930395377	14.30734968	-0.848004067	0.265320412	0.38430837	AC138207.2
ENSG00000270587	9.926382031	4.892356573	1.009388203	0.265322321	0.38430837	AC046185.2
ENSG00000140632	1696.182329	1955.736557	-0.205521406	0.265469124	0.384498612	GLYR1
ENSG00000261118	15.66805597	24.88413297	-0.660952504	0.265483261	0.384498612	AC092123.1
ENSG00000188042	309.123554	171.171618	0.852872764	0.265556742	0.384583596	ARL4C
ENSG00000165695	21.17311707	11.85312745	0.827913802	0.265680057	0.384712808	AK8
ENSG00000039537	9.250677111	1.069617038	3.109715809	0.265689279	0.384712808	C6
ENSG00000260021	0	3.484985349	-4.378979226	0.265690386	0.384712808	AC098934.4
ENSG00000267327	2.766427592	0.387220594	2.838140671	0.265715664	0.384727969	AC009271.1
ENSG00000272931	4.237646425	1.365948763	1.601670466	0.265756818	0.384745395	AC099568.2
ENSG00000122783	348.9670297	283.1402586	0.300548239	0.265771153	0.384745395	CYREN
ENSG00000188175	2.684512796	0.356539013	2.798529469	0.265801744	0.384745395	HEPACAM2
ENSG00000258647	2.684512796	0.356539013	2.798529469	0.265801744	0.384745395	LINC00930
ENSG00000224406	2.684512796	0.356539013	2.798529469	0.265801744	0.384745395	LINC02052
ENSG00000254449	2.416487466	5.656185276	-1.2310239	0.265839166	0.384778126	SF3A3P2
ENSG00000153064	43.01867631	16.94391041	1.338932659	0.265865272	0.384794475	BANK1
ENSG00000109184	792.4176023	940.4334203	-0.246747022	0.265945379	0.384888975	DCUN1D4
ENSG00000241556	3.66251884	7.622969829	-1.05755221	0.266024532	0.384982085	AC018475.1
ENSG00000235619	0.293240848	2.52645467	-2.955985603	0.266090679	0.385056362	RPL36AP33
novel.681	16.93547064	26.68920888	-0.65131617	0.266136048	0.38509661	-
ENSG00000266145	2.68075185	0.356539013	2.796700404	0.266148136	0.38509661	RHOT1P1
ENSG00000279483	32.95124418	48.39799764	-0.557819925	0.266186465	0.38513062	AC090498.1
ENSG00000261188	35.91896863	49.7000147	-0.469151211	0.266292806	0.385263024	Z95115.1

ENSG00000233860	4.7800366	1.518200796	1.687422422	0.266389234	0.385381072	AC107072.2
ENSG00000282876	2.376156893	0	3.5839117	0.266402865	1	Z98752.4
ENSG00000256377	3.881438055	9.619408483	-1.306369019	0.266510848	0.385535542	AC009509.1
ENSG00000113621	1922.051258	1632.306436	0.235588138	0.266544962	0.385548872	TXNDC15
ENSG00000084072	744.808489	897.5341164	-0.269143532	0.266560329	0.385548872	PPIE
ENSG00000133704	1207.275725	1013.177071	0.252778688	0.266564581	0.385548872	IPO8
ENSG00000278238	4.240153722	9.039669269	-1.090228035	0.266645859	0.38564496	AL359513.1
novel.270	2.373649595	0	3.582480707	0.266685954	1	-
ENSG00000249022	2.373649595	0	3.582480707	0.266685954	1	AC093599.1
ENSG00000204025	2.373649595	0	3.582480707	0.266685954	1	TRPC5OS
ENSG00000278213	2.373649595	0	3.582480707	0.266685954	1	CNTNAP3P5
ENSG00000239805	2.373649595	0	3.582480707	0.266685954	1	AC007547.2
ENSG00000271716	2.373649595	0	3.582480707	0.266685954	1	AC022001.2
ENSG00000224094	7.623293122	13.73835135	-0.850237917	0.266741732	0.385762145	RPS24P8
ENSG00000260345	2.766427592	0.356539013	2.838150322	0.266762098	0.385770125	AC009052.1
ENSG00000260954	0.616711807	2.864080462	-2.227037792	0.266805508	0.385790718	AL133297.1
ENSG00000115221	0.909952655	3.169868797	-1.807560849	0.266818411	0.385790718	ITGB6
ENSG00000287543	5.369025593	13.97229243	-1.37967876	0.266820885	0.385790718	AC013486.1
ENSG00000139985	6.627664725	12.35130601	-0.901075801	0.266873063	0.385844689	ADAM21
ENSG00000248491	2.772695836	0.336469917	2.841144942	0.266890217	0.385848017	AC093772.1
ENSG00000268912	41.52933692	27.64097988	0.584939288	0.266906151	0.385849583	AC012313.5
ENSG00000263155	6.134451935	2.180528143	1.495684943	0.266951676	0.385874238	MYZAP
ENSG00000116885	112.8863841	146.4540739	-0.374889495	0.26695291	0.385874238	OSCP1
ENSG00000219747	5.42697852	2.140389951	1.340051887	0.266971461	0.385876838	AL133260.1
ENSG00000266110	0	2.079026788	-3.640964509	0.266978641	1	MIR4423
ENSG00000239280	0	2.079026788	-3.640964509	0.266978641	1	AC108693.1
ENSG00000177770	0	2.079026788	-3.640964509	0.266978641	1	CDKN2AIPNLP1
ENSG00000234426	0	2.079026788	-3.640964509	0.266978641	1	AL139042.1
ENSG00000235962	0	2.079026788	-3.640964509	0.266978641	1	RPL7AP53
ENSG00000269559	1.481319294	4.881744087	-1.716829843	0.266984414	0.385876838	AC093677.2
novel.790	64.39122368	35.27597486	0.864860054	0.2670061	0.385886715	-
ENSG00000169126	31.37769616	44.20211762	-0.495015718	0.267055422	0.385936529	ARMC4
ENSG00000236364	6.335677526	12.66873431	-0.996438266	0.267100534	0.385978264	AL358115.1
ENSG00000253330	10.24358475	5.259508071	0.951435724	0.267114014	0.385978264	AC024451.2
ENSG00000279138	13.54230205	5.5357707	1.28139793	0.26715204	0.386011743	AP002847.1
ENSG00000140497	1623.564802	1362.961606	0.252266971	0.267201784	0.386062148	SCAMP2
ENSG00000179082	28.06859366	13.09750576	1.093827898	0.267252174	0.386113481	C9orf106
ENSG00000104980	748.3163129	977.7078801	-0.385819412	0.267277592	0.386128732	TIMM44
ENSG00000286136	8.728274395	16.42895481	-0.911673136	0.267308722	0.386147331	AC107071.1
ENSG00000180385	30.69766083	50.76017513	-0.723119138	0.267320192	0.386147331	EMC3-AS1
ENSG00000138613	571.5626577	475.2499358	0.266798923	0.267359329	0.386182395	APH1B
ENSG00000244414	2.742465724	0.693008929	1.95475423	0.267410389	0.386215533	CFHR1
ENSG00000176853	982.2087037	1140.486077	-0.215540652	0.267412002	0.386215533	FAM91A1
ENSG00000186395	577.2879199	710.2339034	-0.299440869	0.267438186	0.386231879	KRT10
ENSG00000220848	45.71049753	60.48583686	-0.40474996	0.267612529	0.386462182	RPS18P9
ENSG00000233010	0	2.099095884	-3.653784662	0.267625386	1	RPEP4
ENSG00000240106	0	2.099095884	-3.653784662	0.267625386	1	RN7SL146P
ENSG00000179674	0	2.099095884	-3.653784662	0.267625386	1	ARL14
ENSG00000286909	5.745434885	11.16011852	-0.965312691	0.267693322	0.38655737	AC024560.4
ENSG00000175206	4.853104584	1.742556871	1.458796438	0.267811478	0.386706498	NPPA
novel.296	3.687734357	0.723690511	2.352437921	0.267934441	0.38686255	-

ENSG00000257221	0.879722543	3.61729668	-2.021234126	0.267987855	0.38691817	AC007569.1
ENSG00000232811	3.620934617	7.205067653	-0.989128509	0.268086236	0.38701798	AL360270.1
ENSG00000101230	7.043079671	14.49285183	-1.046843038	0.268089658	0.38701798	ISM1
ENSG00000163545	392.007647	508.2388724	-0.374158516	0.268101675	0.38701798	NUAK2
ENSG00000101577	1200.01874	1010.637599	0.248026515	0.268219048	0.387165903	LPIN2
ENSG00000273102	0.307102255	1.823989131	-2.481742549	0.268308382	1	AP000569.1
ENSG00000272182	4.515772216	1.773238453	1.342061627	0.268318796	0.387288369	AC135507.1
ENSG00000109586	375.2232678	458.1756874	-0.288205595	0.268352391	0.387315342	GALNT7
ENSG00000238123	15.90112133	25.80278224	-0.701489974	0.268443239	0.387424942	MID1IP1-AS1
ENSG00000166595	1061.914753	1245.49194	-0.229890582	0.268659846	0.387701267	CIAO2B
ENSG00000202337	6.301686468	2.853467977	1.141935772	0.268664548	0.387701267	RNU6-8
ENSG00000166886	1657.182028	1320.606561	0.327413235	0.268707544	0.387741777	NAB2
ENSG00000267605	10.84142055	5.461354906	0.996561726	0.26881586	0.387862344	AC016590.1
novel.654	127.0155242	184.5245597	-0.539216213	0.268820955	0.387862344	-
ENSG00000287456	0.293240848	1.823989131	-2.481758281	0.268945568	1	AL157369.1
ENSG00000230005	0.293240848	1.823989131	-2.481758281	0.268945568	1	SNAP47-AS1
ENSG00000071205	462.840864	607.8843329	-0.393724196	0.268987232	0.388080702	ARHGAP10
ENSG00000278129	218.3233776	282.8286781	-0.372584875	0.269023794	0.388111899	ZNF8
ENSG00000117054	893.6328888	694.0190302	0.364334577	0.269185682	0.388323887	ACADM
ENSG00000241978	4.864458693	9.029056784	-0.89199704	0.269261807	0.388412138	AKAP2
ENSG00000270031	6.66674165	2.985650913	1.18074262	0.269448848	0.388660367	AL020997.3
ENSG00000113790	102.8025832	140.8851471	-0.456515192	0.269549923	0.388784576	EHHADH
ENSG00000132522	60.02044849	42.26023527	0.504067492	0.269586241	0.388815373	GPS2
ENSG00000227210	8.679097009	3.465044647	1.312090579	0.269699226	0.388956736	AC079145.1
ENSG00000103269	71.02938831	107.2287804	-0.591956657	0.269740716	0.388994979	RHBDL1
ENSG00000253537	19.76988035	30.64425974	-0.632541106	0.26983192	0.389104908	PCDHGA7
ENSG00000236472	0.600343103	4.278468152	-2.830622689	0.27000432	0.389331904	AC002401.1
ENSG00000269937	6.057551735	12.16593225	-1.00712654	0.270119442	0.389476288	AC093525.7
ENSG00000196296	24.4870207	34.87184882	-0.505897976	0.270151939	0.389483762	ATP2A1
ENSG00000112212	4.105371866	0.387220594	3.41113109	0.270154608	0.389483762	TSPO2
novel.226	6.734723767	2.42610919	1.455307315	0.270185891	0.389507248	-
ENSG00000233654	25.02468102	13.96283582	0.833404358	0.270253763	0.389583478	AC108047.1
ENSG00000092054	3.991004395	1.049547942	1.909023501	0.27030931	0.389641933	MYH7
ENSG00000127540	541.685968	437.5361437	0.308216669	0.270335606	0.389653496	UQCR11
ENSG00000261423	17.68800449	9.438786619	0.899297867	0.270365336	0.389653496	TMEM202-AS1
ENSG00000153832	80.61105828	105.9500901	-0.395457774	0.2703717	0.389653496	FBXO36
ENSG00000115998	185.7781599	152.5721524	0.284509025	0.270377323	0.389653496	C2orf42
ENSG00000270175	18.4080144	11.31352643	0.696627315	0.270479742	0.389779477	AC023509.3
ENSG00000234028	34.89561742	24.89731399	0.482534543	0.270559669	0.389873031	EIF2AK3-DT
ENSG00000187720	538.4378604	1698.454724	-1.657407897	0.27068687	0.390034694	THSD4
ENSG00000147099	188.6050477	228.9246582	-0.278351897	0.270768338	0.39012905	HDAC8
novel.111	10.69528459	3.475657132	1.614468617	0.270782385	0.39012905	-
ENSG00000271109	2.465593581	0	3.634327669	0.270876225	1	AC008555.4
ENSG00000240405	2.465593581	0	3.634327669	0.270876225	1	SAMMSON
ENSG00000211710	2.465593581	0	3.634327669	0.270876225	1	TRBV4-1
ENSG00000207590	0.308355904	1.803920035	-2.468791847	0.270913737	1	MIR215
ENSG00000227470	0.308355904	1.803920035	-2.468791847	0.270913737	1	AC073415.1
ENSG00000274552	0.308355904	1.803920035	-2.468791847	0.270913737	1	MIR6889
ENSG00000244286	0.307102255	1.803920035	-2.468792352	0.270972098	1	ITGB5-AS1
ENSG00000235618	15.47184497	6.35749494	1.278591448	0.271004154	0.390426912	FAM21EP
ENSG00000025293	1002.174725	1162.593516	-0.214180351	0.271062681	0.390489577	PHF20

ENSG00000154832	962.4523173	796.1939085	0.273833389	0.271402404	0.390957299	CXXC1
ENSG00000251503	0	2.169915658	-3.698237329	0.271499059	1	CENPS-CORT
ENSG00000243498	0	2.169915658	-3.698237329	0.271499059	1	UBA52P5
ENSG00000254606	0	2.169915658	-3.698237329	0.271499059	1	AC013714.1
ENSG00000178607	235.714479	303.944033	-0.366078692	0.271669168	0.391319879	ERN1
ENSG00000272537	2.458071688	0	3.630152374	0.271685117	1	AC005014.3
ENSG00000254926	2.458071688	0	3.630152374	0.271685117	1	AP000445.2
ENSG00000223392	2.458071688	0	3.630152374	0.271685117	1	CLDN10-AS1
ENSG00000113262	15.4315144	9.589882776	0.680839171	0.27184186	0.391546921	GRM6
ENSG00000129518	616.9288705	524.6585934	0.233958155	0.271910309	0.391623801	EAPP
ENSG00000169231	649.9947119	521.7036709	0.317070194	0.2720823	0.391849791	THBS3
ENSG00000181555	1274.146034	1087.295473	0.228862963	0.272142485	0.391914745	SETD2
ENSG00000152942	52.20491843	98.99055635	-0.923542237	0.272310397	0.39213482	RAD17
ENSG00000226286	2.451732174	0	3.626604399	0.272371737	1	AL008733.1
ENSG00000173930	2.451732174	0	3.626604399	0.272371737	1	SLCO4C1
ENSG00000258390	2.451732174	0	3.626604399	0.272371737	1	LINC02318
ENSG00000286217	2.451732174	0	3.626604399	0.272371737	1	AC064829.1
ENSG00000171475	921.1908631	761.197522	0.275453682	0.272422566	0.392254538	WIPF2
ENSG00000196436	114.5221167	48.04179695	1.255059118	0.272428096	0.392254538	NPIPB15
ENSG00000248774	6.684364003	3.210006989	1.052833488	0.272438826	0.392254538	AC097534.1
ENSG00000262180	5.141330836	1.874739808	1.479818563	0.272488277	0.392303996	OCLM
ENSG00000168610	2804.545067	2314.667493	0.27682045	0.272567147	0.392395802	STAT3
ENSG00000135940	2140.931341	2493.13703	-0.219724386	0.272584233	0.392398657	COX5B
ENSG00000233705	9.517520064	3.812127049	1.305039393	0.272652967	0.392475856	SLC26A4-AS1
ENSG00000101493	1215.604925	917.0114123	0.406966028	0.272696905	0.392517356	ZNF516
ENSG00000274641	0	2.058957692	-3.627920592	0.272831934	1	HIST1H2BO
ENSG00000162723	0	2.058957692	-3.627920592	0.272831934	1	SLAMF9
ENSG00000184307	11.32356397	21.97431051	-0.962473942	0.272939258	0.392844432	ZDHHC23
ENSG00000157326	27.82517094	12.64049287	1.134497598	0.273121574	0.393085066	DHRS4
ENSG00000172775	1425.552131	1234.517931	0.207380906	0.273187192	0.393157727	FAM192A
ENSG00000121542	239.1660713	281.6568834	-0.235765331	0.273329275	0.393340418	SEC22A
ENSG00000258521	14.60437418	7.082341327	1.041933204	0.273364192	0.393368877	AL157871.2
ENSG00000213071	13.69972086	25.14302343	-0.880947202	0.273441166	0.393457849	LPAL2
ENSG00000204072	4.83548223	1.783850939	1.436098603	0.273596379	0.393659386	ARMCX7P
ENSG00000243302	236.3684606	160.5785712	0.55860881	0.273661221	0.393725682	AC018638.4
ENSG00000114904	318.4111748	378.3395277	-0.24925806	0.273672765	0.393725682	NEK4
ENSG00000198420	31.52487231	52.35723964	-0.72894908	0.273718011	0.393768972	TCAF1
ENSG00000197702	2287.406978	2756.277784	-0.269122884	0.27384079	0.39392379	PARVA
ENSG00000255237	6.639018835	3.210006989	1.04534345	0.273882417	0.393961857	AC138230.1
ENSG00000162641	6.681856706	3.220619475	1.050156586	0.273947489	0.394005173	AKNAD1
ENSG00000074211	3.680141193	8.082294466	-1.145944269	0.273947644	0.394005173	PPP2R2C
ENSG00000085276	94.90313033	64.34653123	0.559016062	0.273958025	0.394005173	MECOM
ENSG00000276757	1.773306493	5.461354906	-1.609789	0.27415112	0.394261057	RN7SL192P
ENSG00000198744	9.172737075	3.291439249	1.48370984	0.274239462	0.394366273	MTCO3P12
ENSG00000255200	26.74512045	16.63324179	0.680072112	0.274290971	0.394418515	PGAM1P8
ENSG00000163632	6.915890978	3.169868797	1.116825901	0.274476048	0.394662803	C3orf49
ENSG00000161542	1003.582605	840.5362999	0.255644547	0.274499856	0.394675194	PRPSAP1
ENSG00000236875	3.812131018	0	4.265366706	0.274576022	0.394762859	DDX11L5
ENSG00000270105	2.66939774	0.387220594	2.791036655	0.274608595	0.39478219	AC136475.8
ENSG00000183506	293.8257719	382.6778523	-0.380436823	0.274619858	0.39478219	PI4KAP2
ENSG00000230732	4.149463386	9.945137519	-1.249852623	0.274640514	0.39479004	AC016949.1

ENSG00000207561	33.22274572	48.63959748	-0.546007534	0.274896585	0.395118919	MIR635
ENSG00000261342	17.53964596	8.216917548	1.082147107	0.274900006	0.395118919	AC006538.2
ENSG00000234118	0.615458159	2.446178286	-2.011322271	0.274914926	0.395118919	RPL13AP6
ENSG00000101363	948.3068638	1100.550767	-0.21467259	0.274956001	0.395156094	MANBAL
ENSG00000256452	0.308355904	1.773238453	-2.449431306	0.274976374	1	AP003170.3
ENSG00000073598	0.307102255	1.773238453	-2.449430186	0.275036307	1	FNDC8
ENSG00000157322	7.243122884	3.597227584	1.012103336	0.275051912	0.395272069	CLEC18A
ENSG00000116016	3899.227343	3096.119206	0.332722557	0.275098578	0.395317265	EPAS1
ENSG00000140044	1307.290453	1075.121579	0.282342046	0.27523846	0.395496401	JDP2
ENSG00000267470	41.74693122	28.74945084	0.540055734	0.275321716	0.395594154	ZNF571-AS1
ENSG00000268140	0.308355904	1.854670712	-2.501397342	0.275427641	1	PCGF7P
ENSG00000167419	0.308355904	1.854670712	-2.501397342	0.275427641	1	LPO
ENSG00000229376	5.414370761	1.386017859	1.951107644	0.275446465	0.395751513	CICP3
ENSG00000119946	0.307102255	1.854670712	-2.501399956	0.275482881	1	CNNM1
ENSG00000287458	0.89358395	3.801514564	-2.088053348	0.275525486	0.395805897	AL033519.4
ENSG00000196712	1726.535437	1407.585894	0.294825417	0.27552604	0.395805897	NF1
ENSG00000112139	131.0045904	91.09491991	0.523151967	0.275539766	0.395805897	MDGA1
ENSG00000225808	8.093868963	3.9336975	1.041714199	0.275551287	0.395805897	DNAJC19P5
ENSG00000248734	7.935481243	15.20477398	-0.943538926	0.275560489	0.395805897	AC008906.1
ENSG00000171496	3.296210009	0.774441189	2.138251565	0.275629465	0.395883085	OR1L8
ENSG00000263466	1.481319294	4.555886656	-1.616026125	0.275701302	0.395957018	AC006441.1
ENSG00000207467	0.293240848	1.773238453	-2.449417776	0.275701989	1	RF00019
ENSG00000200356	2.66939774	0.356539013	2.791022261	0.275711421	0.395957018	RNU6-833P
ENSG00000231550	2.142122621	0.336469917	2.468757145	0.275772279	1	PTCHD3P2
ENSG00000164530	41.77418332	16.09505343	1.371593728	0.275854215	0.396140192	PI16
novel.357	5.66226644	1.069617038	2.402115923	0.275873488	0.396145974	-
ENSG00000215867	2.666890443	0.356539013	2.789792352	0.275948245	0.396231422	KRT18P57
ENSG00000259424	1.21705491	3.587770973	-1.562644627	0.275988189	0.396266878	IRAIN
ENSG00000223984	0.586481695	3.485113743	-2.559798119	0.276034433	0.396311373	HNRNPRP1
ENSG00000162378	771.1213704	642.5165637	0.26336002	0.276058046	0.396323374	ZYG11B
ENSG00000189136	47.27950167	29.62192576	0.676846311	0.276098593	0.396359682	UBE2Q2P1
ENSG00000228906	10.58600298	5.003314538	1.079455604	0.276126045	0.39637719	AL353804.1
ENSG00000053501	355.7361444	297.4034889	0.258200061	0.276170812	0.396419551	USE1
ENSG00000251667	0	1.467450118	-3.130003763	0.276221108	1	BRCC3P1
ENSG00000254314	0	1.467450118	-3.130003763	0.276221108	1	AC021915.2
ENSG00000241362	5.072023799	1.722487775	1.538653498	0.276397916	0.39672362	RPL36AP43
ENSG00000126883	904.2411873	1034.636555	-0.194321233	0.276490698	0.39683487	NUP214
ENSG00000125965	16.9595747	108.6761614	-2.680079357	0.27658513	0.396943647	GDF5
ENSG00000267416	3.977142987	9.069194976	-1.190729851	0.276597043	0.396943647	AC025048.4
ENSG00000223806	0.908699006	2.954969332	-1.690980377	0.276643149	0.396987885	LINC00114
ENSG00000171310	517.452355	357.3177804	0.534778224	0.276783798	0.397167782	CHST11
ENSG00000247287	12.7520162	5.454338441	1.209545354	0.276865048	0.397262429	AL359220.1
ENSG00000114395	240.4723491	303.6068524	-0.33696686	0.276949663	0.397361895	CYB561D2
ENSG00000237931	0.907445357	3.159256312	-1.805923992	0.276987406	0.397383314	CLIC4P3
ENSG00000169884	14.34540913	6.63478505	1.119460124	0.276995182	0.397383314	WNT10B
ENSG00000178229	79.27601749	61.84934548	0.358052658	0.277051106	0.397441598	ZNF543
ENSG00000269903	9.941497088	4.872287477	1.015907189	0.277086312	0.397470156	AC025165.4
ENSG00000049130	531.6132087	921.9757383	-0.794514674	0.277109196	0.397481037	KITLG
ENSG00000164096	2379.780166	3132.083728	-0.396229509	0.277146388	0.397512438	C4orf3
ENSG00000274026	22.86869699	13.47167372	0.758183242	0.277264941	0.397658681	FAM27E3
ENSG00000152242	559.3686731	483.2467352	0.211085279	0.27727896	0.397658681	C18orf25

ENSG00000107643	590.0449228	503.4456711	0.228906904	0.277361143	0.397754587	MAPK8
ENSG00000228463	23.38300834	14.06189703	0.73671703	0.277444713	0.397847717	AP006222.1
ENSG00000204842	808.5544113	975.4999711	-0.270488031	0.27745671	0.397847717	ATXN2
ENSG00000006047	5.794541	2.241891306	1.389579485	0.277645565	0.398096547	YBX2
ENSG00000115204	1064.708164	1266.820806	-0.25095998	0.27771401	0.398172711	MPV17
ENSG00000176125	27.61592491	40.1552788	-0.54482072	0.277834978	0.398324167	UFSP1
ENSG00000274020	62.86545686	89.44312352	-0.509648957	0.277991903	0.398527156	LINC01138
ENSG00000116251	3239.654913	2698.435608	0.263731141	0.278061867	0.398605461	RPL22
ENSG00000253608	0.616711807	3.657434872	-2.574333609	0.278226797	0.39881037	AC022915.2
ENSG00000116096	150.5285845	187.0253283	-0.314050699	0.278235509	0.39881037	SPR
ENSG00000181781	7.698868403	1.548882377	2.333678773	0.2782557	0.398817308	ODF3L2
ENSG00000232838	26.02754657	35.72785066	-0.454971818	0.278295277	0.398826259	PET117
ENSG00000259432	0.293240848	2.406040094	-2.89238531	0.278300092	0.398826259	AC023908.1
ENSG00000265136	1.541779519	5.390535133	-1.812696939	0.2783185	0.398826259	AC124283.4
ENSG00000244056	4.515772216	1.753169357	1.35137891	0.278323348	0.398826259	RN7SL417P
ENSG00000247315	657.1473416	557.9568669	0.23575997	0.27846213	0.399003121	ZCCHC3
ENSG00000250848	1.81990531	4.963176346	-1.451759369	0.278506058	0.399044057	AC021087.2
ENSG00000016402	15.70734636	5.747074145	1.449425781	0.278550282	0.399085413	IL20RA
ENSG00000286905	9.102105118	4.993857928	0.861634038	0.27865853	0.399218487	AC108488.2
ENSG00000159625	6.983944366	2.375358513	1.53254776	0.278684427	0.399233574	DRC7
ENSG00000080618	3.034452922	0.774441189	2.020856884	0.27870133	0.399235776	CPB2
ENSG00000177725	0	3.208851114	-4.263619085	0.278767362	0.399308348	AC105206.1
ENSG00000218027	3.033199274	0.774441189	2.020359194	0.278801787	0.399335643	AL512329.1
ENSG00000278570	5.951746342	12.00537948	-1.010230669	0.278985679	0.399577008	NR2E3
ENSG00000285090	17.39351	9.998328342	0.799009516	0.279112775	0.399737004	AC002074.1
ENSG00000276578	3.925529575	1.436768536	1.451204467	0.279185106	0.399818556	AC004696.1
ENSG00000240280	5.423217574	2.058957692	1.372950036	0.279226874	0.399856331	TCAM1P
ENSG00000286575	4.747299191	1.130980201	2.093981266	0.279287007	0.3999204	AL356608.2
ENSG00000185674	3.895299463	0.672939833	2.501628043	0.279305014	0.399924143	LYG2
ENSG00000226887	1.536764924	4.881744087	-1.680199068	0.279328263	0.39993539	ERVMER34-1
novel.979	15.72364379	3.841652756	2.028056126	0.279431558	0.400061239	-
ENSG00000144749	328.0215083	541.0394091	-0.721601921	0.279503722	0.400117156	LRIG1
ENSG00000274528	3.08105174	8.030259519	-1.384827435	0.279510881	0.400117156	AC090970.2
ENSG00000257660	2.961384939	0.693008929	2.07570196	0.279516816	0.400117156	AC117498.2
ENSG00000255409	3.281094953	0.693008929	2.223908709	0.279545993	0.400136877	RSF1-IT1
ENSG00000230724	11.92745455	5.727005049	1.059931445	0.279617885	0.400200329	LINC01001
ENSG00000166578	32.30143895	48.34210988	-0.577916059	0.279631425	0.400200329	IQCD
ENSG00000168228	250.6594209	188.8162429	0.4086877	0.279636533	0.400200329	ZCCHC4
ENSG00000172819	960.7951419	755.3528344	0.347491999	0.279707787	0.400280254	RARG
ENSG00000213625	2306.720262	2922.551993	-0.341498062	0.279734932	0.400297052	LEPROT
ENSG00000182810	186.266429	229.7435228	-0.303224804	0.279752117	0.400299596	DDX28
ENSG00000261570	3.019337866	0.774441189	2.01480758	0.279921609	0.400520064	AC073878.1
ENSG00000197580	176.942071	97.47805339	0.858759145	0.279992335	0.400599199	BCO2
ENSG00000146409	256.3307316	198.9695202	0.364237473	0.280072363	0.400691633	SLC18B1
ENSG00000262703	2.463086283	6.421169854	-1.390403718	0.280111132	0.400725032	AC009121.1
ENSG00000133313	2096.792881	1632.616487	0.360792321	0.280145977	0.400752815	CNDP2
ENSG00000148175	2035.081534	2777.117955	-0.448608058	0.280325204	0.400987124	STOM
ENSG00000148814	249.9507929	199.4998746	0.326123871	0.280402276	0.401075288	LRRC27
ENSG00000134809	387.2209271	547.560136	-0.500386771	0.280460108	0.401126338	TIMM10
ENSG00000234338	13.48184183	7.561606666	0.838246293	0.280468845	0.401126338	AC073349.2
ENSG00000167780	11.70248056	3.331577441	1.818040179	0.280523251	0.401182066	SOAT2

ENSG00000090565	140.4135565	177.7962229	-0.339828252	0.280816204	0.401578919	RAB11FIP3
ENSG00000273432	5.788272756	2.567748738	1.183921805	0.280836703	0.40158613	AC004951.4
ENSG00000272829	6.625157428	3.210006989	1.043092477	0.280893729	0.401645568	AC002470.1
ENSG00000231701	7.970797221	1.783850939	2.157653635	0.280928638	0.401673378	BMS1P13
ENSG00000277782	25.72420526	15.02184037	0.76860685	0.280962121	0.401699145	AC068870.2
ENSG00000158865	2.081662396	0.387220594	2.43354797	0.280983077	1	SLC5A11
ENSG00000285420	3.969621095	1.436768536	1.463864303	0.281029345	0.401773148	AC011342.1
ENSG00000153130	1432.056565	1697.131402	-0.245075936	0.281203682	0.401995979	SCOC
ENSG00000106617	535.8584751	420.759849	0.348274295	0.281216155	0.401995979	PRKAG2
ENSG00000226478	3.063429386	6.909891803	-1.174893464	0.281295713	0.402087584	UPF3AP1
ENSG00000240571	0.923814062	3.159256312	-1.791013821	0.28134821	0.402140498	AC087071.2
ENSG00000114127	899.1197319	724.3366834	0.31198326	0.281431035	0.402227603	XRN1
ENSG00000270574	4.841750474	1.773238453	1.442153354	0.281440114	0.402227603	AC010680.2
ENSG00000203780	37.85943838	55.44978578	-0.553091291	0.281473837	0.402253673	FANK1
ENSG00000285560	0.293240848	2.375358513	-2.876037519	0.281520007	1	AC103739.3
ENSG00000214796	8.263966798	15.17640415	-0.883940382	0.281586384	0.402392379	AC098934.1
ENSG00000261730	4.314475354	0.713078025	2.59155891	0.281687972	0.402515411	AL034346.1
ENSG00000276178	0	1.436768536	-3.103943883	0.28186515	1	RF00019
ENSG00000185130	0	1.436768536	-3.103943883	0.28186515	1	HIST1H2BL
ENSG00000231074	0	1.436768536	-3.103943883	0.28186515	1	HCG18
ENSG00000269898	0	1.436768536	-3.103943883	0.28186515	1	AC018695.2
ENSG00000267909	0	1.436768536	-3.103943883	0.28186515	1	CCDC177
ENSG00000214424	0	1.436768536	-3.103943883	0.28186515	1	FAM149B1P1
ENSG00000225830	452.4121536	637.4362558	-0.495021208	0.281889763	0.402770139	ERCC6
ENSG00000278356	3.340301529	1.029478846	1.665155513	0.28189724	0.402770139	AC005911.1
ENSG00000131944	48.15728638	68.15408196	-0.504263771	0.281993064	0.402884894	FAAP24
ENSG00000140391	4600.937027	3931.849946	0.226639903	0.282054494	0.402950501	TSPAN3
ENSG00000176753	3.341555177	1.049547942	1.650928008	0.28208525	0.402972281	C15orf56
ENSG00000233613	8.856432003	3.260757667	1.447705655	0.282110192	0.402985754	DCUN1D2-AS
ENSG00000285018	1.188078446	4.819096654	-2.008102435	0.282144712	0.402990111	AC245140.3
ENSG00000266126	2.081662396	0.356539013	2.43352419	0.282156469	1	AC005730.3
ENSG00000224413	3.368024343	7.409354633	-1.147139216	0.282157895	0.402990111	AP001476.1
ENSG00000152778	819.5433046	659.9934699	0.311853468	0.282159775	0.402990111	IFIT5
ENSG00000133247	366.8163012	272.6915359	0.428752599	0.282206399	0.403034545	KMT5C
ENSG00000104824	1.200686205	3.577158488	-1.570757174	0.282233272	0.403050768	HNRNPL
ENSG00000246859	70.0322928	97.79564445	-0.480246081	0.282385517	0.403246022	STARD4-AS1
ENSG00000249001	2.361041837	0	3.575103849	0.282477668	1	AC093895.1
ENSG00000246774	2.361041837	0	3.575103849	0.282477668	1	ZCCHC23
ENSG00000227269	2.361041837	0	3.575103849	0.282477668	1	AL162427.1
ENSG00000139613	1754.245871	1483.618699	0.241843494	0.282496543	0.403382395	SMARCC2
ENSG00000241962	9.343732554	4.179278547	1.149323351	0.282594248	0.403476608	AC079447.1
ENSG00000213785	2.359788188	0	3.574381482	0.282621222	1	AKR1B1P3
ENSG00000287069	2.359788188	0	3.574381482	0.282621222	1	AC061958.1
ENSG00000219507	169.9081229	268.4039498	-0.658866098	0.282629693	0.403476608	FTH1P8
ENSG00000285873	3.700270845	0	4.220691767	0.2826557	0.403476608	AL445253.1
ENSG00000251158	3.700270845	0	4.220691767	0.2826557	0.403476608	AC131392.2
ENSG00000154611	3.700270845	0	4.220691767	0.2826557	0.403476608	PSMA8
ENSG00000225760	3.700270845	0	4.220691767	0.2826557	0.403476608	LINC00431
ENSG00000287729	4.590093848	1.4981317	1.636814781	0.282684447	0.403495475	AF241728.2
ENSG00000272910	7.212892771	3.577158488	1.0122404	0.282744748	0.403559374	AC090425.2
ENSG00000105642	30.6822176	16.43712715	0.902501996	0.28280642	0.403625225	KCNN1

ENSG00000172164	481.2445924	596.0774826	-0.30845753	0.283098167	0.404014004	SNTB1
ENSG00000255769	3.839853833	9.752747295	-1.336667508	0.283109925	0.404014004	GOLGA2P10
ENSG00000171462	33.064358	43.92341485	-0.409302792	0.283181681	0.404094209	DLK2
ENSG00000287291	3.282348601	0.774441189	2.131846636	0.283266726	0.404193366	AC068286.2
ENSG00000111300	628.0657993	807.5090228	-0.362519821	0.283295985	0.404212916	NAA25
ENSG00000244681	3.685227059	0	4.214955791	0.283320943	0.404226328	MTHFD2P1
ENSG00000260672	2.695866906	0.693008929	1.935793518	0.283408659	0.404314484	AC100827.4
ENSG00000183535	1.229662669	4.83099341	-1.985855124	0.283440884	0.404314484	COL18A1-AS1
ENSG00000196507	1006.186373	866.4054411	0.216052036	0.283443645	0.404314484	TCEAL3
ENSG00000140548	400.5032673	510.166709	-0.348600775	0.28346188	0.404314484	ZNF710
ENSG00000251669	26.08542823	40.61575931	-0.642920977	0.283470982	0.404314484	FAM86EP
ENSG00000287242	3.001715513	7.194455167	-1.257129491	0.283476104	0.404314484	AC073593.2
ENSG00000258102	64.91918298	85.69557047	-0.398764335	0.283579767	0.404440134	MAP1LC3B2
ENSG00000275236	0.308355904	1.793307549	-2.462459968	0.283603199	1	AC009120.5
ENSG00000287029	0.308355904	1.793307549	-2.462459968	0.283603199	1	AL162713.2
ENSG00000250551	0.307102255	1.793307549	-2.462459848	0.283661306	1	MIR583HG
ENSG00000108187	176.8250541	116.1464594	0.604669407	0.283697675	0.404550353	PBLD
ENSG00000255874	56.42655216	31.49080497	0.840453089	0.283703721	0.404550353	LINC00346
ENSG00000259982	2.773949485	0.356539013	2.84191585	0.283718977	0.404550353	CDC37P1
ENSG00000234492	6.136959233	2.241891306	1.469163432	0.283719333	0.404550353	RPL34-AS1
ENSG00000163312	223.2659467	185.8587863	0.264215396	0.283773705	0.404605676	HELQ
ENSG00000189079	411.4460919	328.2465002	0.326722535	0.283792921	0.404610869	ARID2
ENSG00000276945	4.48930305	1.10029862	2.039883859	0.283832529	0.404645135	AC008498.1
ENSG00000168992	4.175932552	1.00940975	2.017659876	0.284144473	0.40506763	OR7E102P
ENSG00000151553	584.7883822	729.9782534	-0.32040147	0.284187317	0.405106478	FAM160B1
ENSG00000136521	1050.317832	1341.198437	-0.35283865	0.284221923	0.40513358	NDUFB5
ENSG00000189089	0	2.069570178	-3.634360578	0.284292142	1	RIMKLBP1
ENSG00000178772	2.765173943	0.356539013	2.837700625	0.284529307	0.40554948	CPN2
ENSG00000017797	2323.477644	2025.579316	0.197953817	0.284659523	0.405712823	RALBP1
ENSG00000148660	580.9816377	742.3501515	-0.353170571	0.284718855	0.405770903	CAMK2G
ENSG00000241322	3.060922088	0.672939833	2.14179122	0.284731509	0.405770903	CDRT1
ENSG00000276533	4.542241382	1.854670712	1.314243885	0.284846759	0.405912881	AC018926.2
ENSG00000164616	0	3.097764754	-4.208659622	0.285151436	0.406280201	FBXL21P
ENSG00000258760	0	3.097764754	-4.208659622	0.285151436	0.406280201	AL355076.2
novel.933	0	3.097764754	-4.208659622	0.285151436	0.406280201	-
ENSG00000269696	33.00765872	22.32584084	0.570339853	0.285174589	0.406290908	AC005498.3
ENSG00000182040	2.765173943	0.336469917	2.837705896	0.285194041	0.406296342	USH1G
ENSG00000125650	59.50606587	76.48370846	-0.361501704	0.285328756	0.406465973	PSPN
novel.849	10.92201043	6.135450615	0.834559461	0.285396176	0.406539726	-
ENSG00000227329	9.271918219	3.689272328	1.340936477	0.285414278	0.406543222	AL139396.1
ENSG00000275132	0.307102255	2.231278821	-2.771306815	0.285442001	1	RN7SL663P
ENSG00000236035	0.307102255	2.231278821	-2.771306815	0.285442001	1	AL513343.1
ENSG00000110169	51.67714547	33.81271588	0.617288278	0.285479755	0.406614194	HPX
ENSG00000163513	7072.004212	9151.938552	-0.371996246	0.285523521	0.406654237	TGFBR2
ENSG00000282034	14.82232449	8.824769804	0.75931113	0.285603364	0.406745655	AC106886.5
ENSG00000227620	4.306953462	1.029478846	2.040185911	0.28562833	0.406755161	ALG1L8P
ENSG00000055147	362.2560221	457.1182368	-0.336248376	0.28564135	0.406755161	FAM114A2
ENSG00000254485	0	1.41669944	-3.086337318	0.285691199	1	AC026191.1
ENSG00000264230	0	1.41669944	-3.086337318	0.285691199	1	ANXA8L1
ENSG00000198074	0	1.41669944	-3.086337318	0.285691199	1	AKR1B10
ENSG00000287080	0	1.41669944	-3.086337318	0.285691199	1	HIST1H3C

ENSG00000235077	0	1.41669944	-3.086337318	0.285691199	1	AC073842.1
ENSG00000225705	0	1.41669944	-3.086337318	0.285691199	1	AC004899.1
ENSG00000232486	0	1.41669944	-3.086337318	0.285691199	1	AL592437.2
ENSG00000237505	11.16489151	5.787212337	0.951730306	0.285696455	0.406811334	PKN2-AS1
ENSG00000105443	1478.136084	1223.522347	0.272847825	0.285786494	0.406917242	CYTH2
ENSG00000134864	78.04037131	107.9004016	-0.469010453	0.285808271	0.406925948	GGACT
ENSG00000100483	168.0233557	140.0556701	0.263164653	0.285896611	0.407029419	VCPKMT
ENSG00000286675	2.750058887	0.356539013	2.830389131	0.285936907	0.407064482	AC068700.2
ENSG00000278949	8.603521728	17.2825165	-1.001230119	0.28605682	0.407212878	AC127070.4
ENSG00000205981	537.9224883	455.7976448	0.238577547	0.28608709	0.407233656	DNAJC19
novel.603	235.510889	188.4677479	0.321194921	0.286356813	0.407595265	-
ENSG00000047579	220.2600708	164.3076906	0.421535717	0.286388947	0.407618672	DTNBP1
ENSG00000110367	2878.201347	2535.34427	0.18293567	0.28654045	0.407811966	DDX6
ENSG00000254458	3.053328925	7.939370649	-1.380372966	0.286623352	0.407907609	AP001107.3
ENSG00000260644	0.293240848	2.261960402	-2.788885922	0.286648431	1	HERC2P5
ENSG00000172728	93.0776406	119.9815624	-0.368235786	0.286667305	0.407947815	FUT10
ENSG00000182013	364.9713195	180.7973791	1.012737529	0.286705758	0.407980189	PNMA8A
ENSG00000260517	13.07165494	6.84852864	0.933532007	0.28674398	0.408012232	AC009093.2
ENSG00000160062	153.5513982	121.9394854	0.333712187	0.286761652	0.408015031	ZBTB8A
ENSG00000126456	1210.686672	1434.43049	-0.244517552	0.286907874	0.408200727	IRF3
ENSG00000167981	82.45381435	64.32761801	0.355003625	0.28713808	0.408505885	ZNF597
ENSG00000228606	17.21241406	10.3230299	0.73467654	0.287569842	0.409097742	AL139011.1
ENSG00000183624	636.7773873	799.2183446	-0.328134211	0.287606792	0.409127905	HMCES
ENSG00000137757	7.848551852	2.975038428	1.413088119	0.2876382	0.409150182	CASP5
ENSG00000272360	3.299970955	1.080229524	1.61622811	0.287687476	0.409197871	AC116036.2
ENSG00000114638	0.925067711	7.402338167	-3.007052199	0.287759447	0.40927551	UPK1B
ENSG00000267858	52.25005049	39.28506845	0.410198947	0.287773566	0.40927551	MZF1-AS1
ENSG00000187231	740.3013644	892.2691192	-0.269604544	0.288038999	0.409630589	SESTD1
ENSG00000244479	1.541779519	5.522718069	-1.843685739	0.288055078	0.409631034	OR2A1-AS1
ENSG00000090060	2774.898228	3253.331688	-0.229418364	0.288141979	0.409732186	PAPOLA
ENSG00000251676	0	3.02822925	-4.182829964	0.288186245	0.40973484	SNHG27
ENSG00000163161	1275.255318	1070.784294	0.252183557	0.288187257	0.40973484	ERCC3
ENSG00000263050	28.71589159	18.23415911	0.648456132	0.288191157	0.40973484	AC090617.5
ENSG00000101843	774.4028488	910.0516259	-0.233111826	0.288268521	0.409822404	PSMD10
ENSG00000170632	247.0973643	307.576193	-0.316520374	0.288313459	0.409863863	ARMC10
ENSG00000186806	131.4271712	94.1320922	0.481999992	0.288340983	0.409880564	VSIG10L
ENSG00000258773	0	2.038888596	-3.614488039	0.288378421	1	AC087636.1
ENSG00000274354	0	2.038888596	-3.614488039	0.288378421	1	AC105105.3
ENSG00000143494	44.95964461	28.89109039	0.638128561	0.288590981	0.410213496	VASH2
ENSG00000257913	8.465192389	16.05936316	-0.920333974	0.288668209	0.41029872	DDN-AS1
ENSG00000286482	11.57779916	5.207601518	1.140287062	0.288682522	0.41029872	AL117344.2
ENSG00000171169	165.3204651	200.1872797	-0.276425479	0.288723349	0.410319723	NAIF1
ENSG00000224251	0.908699006	2.822786395	-1.642488049	0.288728885	0.410319723	AL391427.1
ENSG00000228008	3.331454716	0.743759607	2.18347425	0.288806548	0.410407642	AC105935.1
ENSG00000160318	30.47422521	18.58581783	0.71615109	0.288895348	0.410511378	CLDND2
ENSG00000124257	15.67181692	9.445803084	0.736796615	0.289133633	0.410827504	NEURL2
ENSG00000260211	0.308355904	1.753169357	-2.43591338	0.289246702	1	AC139426.1
ENSG00000242853	0.307102255	1.753169357	-2.435911393	0.289306789	1	RN7SL749P
ENSG00000182636	1216.924734	687.5941397	0.823379043	0.289318149	0.4110672	NDN
ENSG00000186446	2.127007565	4.963176346	-1.229049617	0.289639961	0.411501932	ZNF501
ENSG00000143153	387.8678248	518.2989963	-0.418371986	0.289672467	0.41152561	ATP1B1

ENSG00000228808	0.293240848	1.753169357	-2.435889383	0.289974128	1	HMGB3P4
ENSG00000226650	0.293240848	1.753169357	-2.435889383	0.289974128	1	KIF4B
ENSG00000011052	0.293240848	1.753169357	-2.435889383	0.289974128	1	NME1-NME2
ENSG00000228049	13.85316526	23.68947785	-0.770192904	0.289990971	0.41193823	POLR2J2
ENSG00000272562	8.172022812	3.995060664	1.038824488	0.290006151	0.41193823	AL512343.2
ENSG00000287377	2.111892509	5.104815894	-1.266395177	0.290010475	0.41193823	AC090948.4
ENSG00000165832	308.7775347	392.3389526	-0.346289247	0.290055703	0.411979949	TRUB1
novel.151	10.51286373	5.207473124	1.029759085	0.290210912	0.412177866	-
ENSG00000196972	70.97318723	49.67074578	0.512043959	0.290276956	0.412249131	SMIM10L2B
ENSG00000281100	4.267876537	1.487519214	1.539681578	0.290334473	0.412306655	AC105749.1
ENSG00000226312	25.46843169	15.16219565	0.75117707	0.290364156	0.412306655	CFLAR-AS1
ENSG00000280211	32.49779249	21.94106039	0.568679244	0.290365069	0.412306655	AC106886.4
ENSG00000131408	1519.793547	1870.836163	-0.299611212	0.290510215	0.412476992	NR1H2
ENSG00000050327	4.222531369	9.336129388	-1.151333531	0.290516781	0.412476992	ARHGEF5
ENSG00000204852	547.6685292	647.424345	-0.241479247	0.29066778	0.41266883	TCTN1
ENSG00000112659	654.3042436	806.0524635	-0.300539866	0.290711459	0.412708291	CUL9
ENSG00000127530	2.991615051	0.672939833	2.112973796	0.29073803	0.412723459	AC005255.1
ENSG00000278153	2.709728314	0.743759607	1.892645941	0.290849475	0.412859106	AL121782.1
ENSG00000254152	3.518890171	0	4.149963297	0.29093358	0.412944261	PPIAP84
ENSG00000010818	571.5472579	723.8375461	-0.340390139	0.290941253	0.412944261	HIVEP2
ENSG00000159176	8437.834991	5903.213996	0.515336404	0.291034071	0.413053436	CSRP1
ENSG00000157796	682.3319375	541.102771	0.334925515	0.291098222	0.413121915	WDR19
ENSG00000286193	7.52758819	13.8399811	-0.879449897	0.291231602	0.41328863	AC025449.2
ENSG00000162517	1088.8554	1258.032309	-0.208527526	0.291299046	0.413361763	PEF1
ENSG00000268107	0.293240848	2.221822211	-2.765653546	0.291324622	1	AC003005.1
ENSG00000228261	2.094270155	5.951361125	-1.507237028	0.291331188	0.413384795	ITPRIP-AS1
ENSG00000141664	390.1728248	289.075425	0.433516013	0.291445352	0.413511406	ZCCHC2
ENSG00000215817	97.02537985	74.57340679	0.382842913	0.291452249	0.413511406	ZC3H11B
ENSG00000260924	26.33339518	37.50712528	-0.513663689	0.291501852	0.413559199	LINC01311
ENSG00000229413	56.64844974	35.02397246	0.698174959	0.291518092	0.413559657	AC018638.1
ENSG00000182600	54.67850498	34.93321199	0.649770156	0.291537212	0.4135642	SNORC
ENSG00000106028	564.6744451	742.8004782	-0.395428392	0.291572013	0.41358525	SSBP1
ENSG00000286262	3.005476459	0.774441189	2.007381388	0.291583889	0.41358525	BX322557.2
ENSG00000167779	7391.863528	4489.606198	0.719314768	0.291606476	0.413594708	IGFBP6
ENSG00000132563	460.7402974	341.6992611	0.431858621	0.291761248	0.413791636	REEP2
ENSG00000168159	143.8554603	241.127072	-0.745340845	0.291807835	0.413835118	RNF187
ENSG00000164776	32.30276387	23.2551026	0.47348766	0.291891048	0.413930534	PHKG1
ENSG00000007952	13.89224218	8.601697997	0.691820793	0.292111867	0.414221068	NOX1
ENSG00000259710	1.524157165	0	2.940566483	0.292327546	1	NUTF2P6
ENSG00000167414	1.524157165	0	2.940566483	0.292327546	1	AC093503.1
ENSG00000286542	1.524157165	0	2.940566483	0.292327546	1	AC124242.3
ENSG00000249955	1.524157165	0	2.940566483	0.292327546	1	AC084866.1
ENSG00000205444	1.524157165	0	2.940566483	0.292327546	1	AL034376.1
ENSG00000237586	1.524157165	0	2.940566483	0.292327546	1	AC090192.1
ENSG00000256674	1.524157165	0	2.940566483	0.292327546	1	AC020549.1
ENSG00000110195	3.699017196	0.743759607	2.329935387	0.292333228	0.414497306	FOLR1
ENSG00000270720	2.461832635	0.387220594	2.669444414	0.29233858	0.414497306	AC104785.1
ENSG00000188095	4.926172567	11.89429312	-1.275041439	0.292433121	0.414608727	MESP2
ENSG00000155313	951.4375914	840.1278883	0.179500116	0.29247804	0.414648603	USP25
ENSG00000196549	2470.417003	4774.721472	-0.950656761	0.292505399	0.414648603	MME
ENSG00000122299	1112.366396	1318.533685	-0.245553288	0.292513132	0.414648603	ZC3H7A

ENSG00000279161	1.186824798	5.390406738	-2.169383849	0.292525086	0.414648603	AC093503.4
ENSG00000270426	5.713951124	2.476859868	1.19986192	0.292552738	0.414665176	AC099343.2
ENSG00000260266	0.615458159	2.772035718	-2.188578823	0.292748555	0.414920092	PPIAP46
ENSG00000144233	703.3061785	836.7495758	-0.250383876	0.292768849	0.414926221	AMMECR1L
ENSG00000200152	1.835020366	5.289033778	-1.535398947	0.293074094	0.415326706	RNU6-216P
ENSG00000232871	13.9149504	8.010190423	0.803276128	0.2930834	0.415326706	SEC1P
ENSG00000272909	7.580455251	3.068367442	1.282512201	0.293130788	0.415371203	AL122035.2
ENSG00000279491	6.272710004	14.49858403	-1.201808967	0.293180776	0.415419379	AP003733.4
ENSG00000163116	3.341555177	1.10029862	1.616116676	0.293257405	0.415505298	STPG2
ENSG00000008256	2008.359011	1440.744496	0.479017179	0.293356193	0.4156226	CYTH3
ENSG00000223653	5.486185096	12.44463502	-1.188769143	0.29344928	0.415731814	AL078459.1
ENSG00000237350	18.53903531	26.81090772	-0.530633919	0.293568182	0.415877587	CDC42P6
ENSG00000092020	363.3451054	427.1972355	-0.23360829	0.293739942	0.416098218	PPP2R3C
ENSG00000058673	9.235348592	4.402350354	1.081608	0.293788666	0.41614455	ZC3H11A
ENSG00000160183	2.15473038	0	3.439802386	0.293821416	1	TMPRSS3
ENSG00000143556	2.15473038	0	3.439802386	0.293821416	1	S100A7
ENSG00000277984	2.15473038	0	3.439802386	0.293821416	1	AP001264.1
ENSG00000286792	2.15473038	0	3.439802386	0.293821416	1	AC005703.7
ENSG00000276772	2.15473038	0	3.439802386	0.293821416	1	AC025271.4
ENSG00000279281	2.153476731	0	3.439014157	0.29398728	1	AC015883.1
ENSG00000285698	2.153476731	0	3.439014157	0.29398728	1	AL049825.1
ENSG00000230817	2.153476731	0	3.439014157	0.29398728	1	LINC01362
ENSG00000263482	2.153476731	0	3.439014157	0.29398728	1	ANTXRLP1
ENSG00000256553	2.153476731	0	3.439014157	0.29398728	1	TRAV1-2
ENSG00000180329	402.3534744	468.3979523	-0.219744202	0.294171893	0.416664665	CCDC43
ENSG00000279805	5.098492965	2.160459047	1.24359522	0.294205861	0.416690061	Z95114.3
ENSG00000107295	1.218308559	3.852265241	-1.670380746	0.29436432	0.416891763	SH3GL2
ENSG00000226519	2.461832635	0.336469917	2.669467478	0.29440905	0.416932384	LINC00390
ENSG00000161649	2.463086283	0.387220594	2.670133426	0.294452748	0.416971541	CD300LG
ENSG00000198354	3.994836612	0.672939833	2.53803548	0.294486164	0.416996131	DCAF12L2
ENSG00000205155	264.0107675	319.8670349	-0.277719059	0.29471101	0.417289544	PSENEN
ENSG00000236710	11.59639043	4.832149285	1.25376424	0.294744941	0.417289544	AC108448.2
ENSG00000157216	929.7436636	738.8928381	0.331366823	0.294755877	0.417289544	SSBP3
ENSG00000247708	63.45018671	48.72385507	0.378425566	0.294757619	0.417289544	STX18-AS1
ENSG00000226580	1.510295757	0	2.928958021	0.29488247	1	RPL39P40
ENSG00000053108	1.510295757	0	2.928958021	0.29488247	1	FSTL4
ENSG00000262098	1.510295757	0	2.928958021	0.29488247	1	AC127496.2
ENSG00000228470	1.510295757	0	2.928958021	0.29488247	1	AL929236.1
ENSG00000225731	1.510295757	0	2.928958021	0.29488247	1	AP001627.1
novel.209	1.510295757	0	2.928958021	0.29488247	1	-
ENSG00000203489	1.510295757	0	2.928958021	0.29488247	1	HMGB1P39
ENSG00000287870	1.510295757	0	2.928958021	0.29488247	1	AC093433.2
ENSG00000288070	1.509042109	0	2.927910596	0.295114448	1	AC016902.1
ENSG00000237154	1.509042109	0	2.927910596	0.295114448	1	MCFD2P1
ENSG00000258199	1.509042109	0	2.927910596	0.295114448	1	AC073896.4
ENSG00000270248	1.509042109	0	2.927910596	0.295114448	1	AC011468.4
ENSG00000243915	1.509042109	0	2.927910596	0.295114448	1	THAP12P2
ENSG00000251201	1.509042109	0	2.927910596	0.295114448	1	TMED7-TICAM2
ENSG00000213279	1.509042109	0	2.927910596	0.295114448	1	Z97192.2
ENSG00000232837	1.509042109	0	2.927910596	0.295114448	1	LINC01700
ENSG00000225636	2.654282684	0.356539013	2.783280913	0.295119343	0.417778872	HNRNPA3P15

ENSG00000157510	749.555946	551.6957581	0.442675539	0.295211761	0.417886933	AFAP1L1
ENSG00000176087	1198.006241	1386.911692	-0.21138976	0.295236897	0.417899745	SLC35A4
ENSG00000248610	0.616711807	3.424906455	-2.487918411	0.295601565	0.418393128	HSPA8P4
ENSG00000138378	39.47213459	65.3068129	-0.725472324	0.295660649	0.418453959	STAT4
ENSG00000259397	0.879722543	3.515795324	-1.987100257	0.29572865	0.418527403	AC021231.2
ENSG00000203325	1.203193503	3.566546002	-1.566067294	0.295836909	0.41865781	AL445248.1
ENSG00000168538	672.3845672	804.8561043	-0.259613465	0.295901648	0.418726618	TRAPPC11
ENSG00000178217	2.460578986	0.356539013	2.668818393	0.295957181	0.418782394	SH2D4B
ENSG00000221947	0.586481695	2.51699806	-2.080483568	0.296015605	0.418842253	XKR9
ENSG00000241030	4.845582691	1.823989131	1.418563757	0.29617812	0.419049379	RPL29P24
ENSG00000179152	601.8559577	510.1189718	0.238000129	0.29641291	0.419358735	TCAIM
ENSG00000235703	123.4625438	80.00634468	0.62781601	0.296479805	0.419430537	LINC00894
novel.952	165.5052799	307.9526731	-0.895835567	0.296591369	0.419549045	-
ENSG00000178803	8.487900608	15.6804433	-0.882854717	0.29659587	0.419549045	ADORA2A-AS1
ENSG00000185115	11.50473118	6.696276607	0.771137014	0.29669793	0.419670564	NSMCE3
ENSG00000266983	5.691242904	11.49414829	-1.010630559	0.296833528	0.419839506	AC011444.1
ENSG00000235508	6.037422084	10.33364238	-0.776812431	0.296894219	0.419902487	RPS2P7
ENSG00000100104	194.9217067	238.1454289	-0.289139596	0.296936214	0.419928748	SRRD
ENSG00000196437	155.7899812	124.8608663	0.317717145	0.296945112	0.419928748	ZNF569
ENSG00000159685	202.3887912	250.9935008	-0.31095803	0.297034828	0.420032759	CHCHD6
ENSG00000172671	90.26224909	114.3224234	-0.340731539	0.297214418	0.420263839	ZFAND4
ENSG00000163820	762.8117299	873.312169	-0.195380479	0.29725224	0.420287336	FYCO1
ENSG00000235244	1.847628125	5.196860638	-1.491714596	0.297263388	0.420287336	DANT2
ENSG00000179097	7.513726783	3.311508345	1.194595822	0.297433541	0.420451141	HTR1F
ENSG00000288060	3.391914941	0	4.095108439	0.29746619	0.420451141	AL355338.2
ENSG00000168671	3.391914941	0	4.095108439	0.29746619	0.420451141	UGT3A2
ENSG00000143297	3.391914941	0	4.095108439	0.29746619	0.420451141	FCRL5
ENSG00000231419	3.391914941	0	4.095108439	0.29746619	0.420451141	LINC00689
ENSG00000273096	2.752566185	6.553352791	-1.251906114	0.297476343	0.420451141	AL021707.8
ENSG00000249850	3.311325065	1.060160428	1.63468684	0.297641652	0.420659513	KRT18P31
ENSG00000137764	293.8941375	357.0406315	-0.280187254	0.297656152	0.420659513	MAP2K5
ENSG00000132510	558.9913102	768.4278993	-0.458723241	0.297730012	0.420741009	KDM6B
ENSG00000286458	4.764921544	1.793307549	1.413033465	0.297761259	0.42076228	AC083870.1
ENSG00000106692	539.2185391	677.9969199	-0.330259343	0.297937683	0.420983276	FKTN
ENSG00000227632	19.48401921	9.396336676	1.046134804	0.297950059	0.420983276	AC018804.1
ENSG00000234777	3.378124804	0	4.089384958	0.298153466	0.421224861	LINC02529
ENSG00000260518	3.378124804	0	4.089384958	0.298153466	0.421224861	BMS1P8
ENSG00000266289	5.118622616	2.129777466	1.259706081	0.298243128	0.421328622	AC012213.3
ENSG00000140876	32.8542856	23.1854387	0.499335913	0.298337036	0.421438371	AC092724.1
ENSG00000280055	0.601596751	2.793260689	-2.216915012	0.298381543	0.421478328	TMEM75
ENSG00000111249	2.155984028	0	3.440621883	0.298475221	1	CUX2
ENSG00000236209	2.155984028	0	3.440621883	0.298475221	1	AC104135.1
ENSG00000253223	2.155984028	0	3.440621883	0.298475221	1	AC110998.1
novel.1088	2.155984028	0	3.440621883	0.298475221	1	-
ENSG00000179304	14.62485984	23.04123062	-0.653016738	0.298537968	0.421676359	FAM156B
ENSG00000213514	6.553343093	2.904218654	1.183833992	0.298564358	0.421690709	AL731556.1
ENSG00000288025	4.821620823	1.762625967	1.442153516	0.298733033	0.421906009	AC093849.4
ENSG00000065600	186.0096434	234.3043241	-0.332549821	0.298794958	0.421970527	TMEM206
ENSG00000267119	2.40137241	0.387220594	2.637012386	0.298864259	0.422015351	RPL10P15
ENSG00000175213	210.6270874	262.3723244	-0.31698135	0.298864615	0.422015351	ZNF408
ENSG00000147274	2936.893706	3384.087766	-0.204570129	0.298882727	0.422015351	RBMX

ENSG00000256061	13.97283206	7.24764599	0.932978493	0.29889167	0.422015351	DNAAF4
ENSG00000185002	2.152223082	0	3.43825349	0.298920285	1	RFX6
ENSG00000188730	2.152223082	0	3.43825349	0.298920285	1	VWC2
ENSG00000211943	2.152223082	0	3.43825349	0.298920285	1	IGHV3-15
ENSG00000273618	3.253372138	0.693008929	2.213977688	0.298924704	0.422039058	AL135999.2
ENSG00000183444	5.700089716	2.527610546	1.179695811	0.298982498	0.422097716	OR7E38P
ENSG00000152219	544.707058	460.5076969	0.242177066	0.299035321	0.422149353	ARL14EP
ENSG00000204839	9.799406802	4.586568238	1.086519206	0.29907238	0.422160628	MROH6
ENSG00000166024	274.8543675	337.9871883	-0.297721959	0.299075806	0.422160628	R3HCC1L
ENSG00000253522	217.1852171	431.2522085	-0.989376772	0.299151737	0.422244867	MIR3142HG
novel.617	2.111892509	5.074134312	-1.259908016	0.299276428	0.422397918	-
ENSG00000249740	44.53857433	65.20325658	-0.547291809	0.299375204	0.422499076	OSMR-AS1
ENSG00000140006	198.0277171	239.8701812	-0.277063945	0.299380624	0.422499076	WDR89
ENSG00000186318	1050.399593	901.3618172	0.220468804	0.299420202	0.422523256	BACE1
ENSG00000141959	4304.548528	5039.297733	-0.227297913	0.299430283	0.422523256	PFKL
ENSG00000243518	2.113146158	0	3.413357823	0.299570378	1	AC108729.2
ENSG00000252097	2.113146158	0	3.413357823	0.299570378	1	RNU6-346P
ENSG00000274885	2.113146158	0	3.413357823	0.299570378	1	AC087241.3
ENSG00000243746	2.113146158	0	3.413357823	0.299570378	1	EEF1A1P10
ENSG00000253398	2.113146158	0	3.413357823	0.299570378	1	AC021733.1
ENSG00000285820	2.113146158	0	3.413357823	0.299570378	1	AL157829.1
ENSG00000236466	2.113146158	0	3.413357823	0.299570378	1	AL035670.1
ENSG00000168828	2.113146158	0	3.413357823	0.299570378	1	OR13J1
ENSG00000230665	2.113146158	0	3.413357823	0.299570378	1	THAP12P1
ENSG00000228826	2.113146158	0	3.413357823	0.299570378	1	AL592494.1
ENSG00000230147	2.113146158	0	3.413357823	0.299570378	1	AL158071.2
ENSG00000242571	0.586481695	3.332733316	-2.48739544	0.300082169	0.42338835	RPL21P11
ENSG00000187783	3.270994492	0.743759607	2.159517633	0.300093471	0.42338835	TMEM72
ENSG00000114021	750.5189079	894.6644315	-0.253755417	0.300093757	0.42338835	NIT2
ENSG00000182768	780.1704065	615.1466067	0.342419097	0.300108535	0.42338835	NGRN
ENSG00000259986	2.40137241	0.356539013	2.637004496	0.300155629	0.423413941	AC103876.1
ENSG00000107443	209.0910345	251.7779122	-0.268760252	0.300159269	0.423413941	CCNJ
ENSG00000166407	2.421502061	0.356539013	2.647864639	0.300198958	0.423446937	LMO1
ENSG00000287338	2.108131562	0	3.410147782	0.300241533	1	AL929091.1
ENSG00000260469	2.108131562	0	3.410147782	0.300241533	1	INSYN1-AS1
ENSG00000227201	2.108131562	0	3.410147782	0.300241533	1	CNN2P1
ENSG00000134007	2.108131562	0	3.410147782	0.300241533	1	ADAM20
ENSG00000270876	2.108131562	0	3.410147782	0.300241533	1	ZNF30-AS1
ENSG00000268738	2.108131562	0	3.410147782	0.300241533	1	HSFX2
ENSG00000274973	2.108131562	0	3.410147782	0.300241533	1	BSNDP2
ENSG00000264534	2.108131562	0	3.410147782	0.300241533	1	MIR378B
ENSG00000271964	6.912130031	2.822786395	1.285537033	0.300436504	0.423759002	AC090948.1
ENSG00000186094	2.406387005	0.336469917	2.639703361	0.30045481	0.423761816	AGBL4
ENSG00000173517	1337.905014	1722.325641	-0.364536681	0.300643192	0.424004493	PEAK1
ENSG00000143924	853.5275009	1093.591035	-0.357852092	0.300760222	0.424146519	EML4
ENSG00000149488	5.102253911	2.089639274	1.272700926	0.300810721	0.424194711	TMC2
ENSG00000270641	0.907445357	3.688116453	-2.017941419	0.300918708	0.424323959	TSIX
ENSG00000162191	1904.880087	2237.113198	-0.231805989	0.300943689	0.424336154	UBXN1
ENSG00000071282	310.2939128	183.5431811	0.756575043	0.300986482	0.424373463	LMCD1
ENSG00000119660	2.421502061	0.336469917	2.64786449	0.301036263	0.42440902	DPPA5P4
ENSG00000241627	24.25800103	35.17203336	-0.54105718	0.301044372	0.42440902	UBQLN4P1

ENSG00000161547	2494.661747	2054.528529	0.279937161	0.301079975	0.424436181	SRSF2
novel.920	8.296704208	2.323323566	1.855784264	0.301117476	0.424466017	-
ENSG00000126231	5.160206839	9.305319412	-0.852726802	0.30116012	0.424485577	PROZ
ENSG00000008300	121.2825267	192.8735058	-0.668214637	0.301164029	0.424485577	CELSR3
ENSG00000148357	11.82945579	6.267761946	0.903108497	0.301248774	0.42458199	HMCN2
ENSG00000224746	5.529022967	1.762625967	1.638660483	0.30128106	0.424598926	AC015987.1
ENSG00000215481	4.860697747	1.345879667	1.823028324	0.301293476	0.424598926	BCRP3
ENSG00000271888	6.919651924	12.55418032	-0.849440824	0.301353738	0.424653612	AL136162.1
ENSG00000279412	6.494136517	15.14097068	-1.215972268	0.301364971	0.424653612	AC020763.3
ENSG00000157625	574.835832	457.8993308	0.328771445	0.301419015	0.424706731	TAB3
ENSG00000149300	2.098031101	0	3.403634745	0.301531632	1	C11orf52
ENSG00000157119	2.098031101	0	3.403634745	0.301531632	1	KLHL40
ENSG00000250900	2.098031101	0	3.403634745	0.301531632	1	AC008443.6
ENSG00000214510	2.098031101	0	3.403634745	0.301531632	1	SPINK13
ENSG00000238963	2.098031101	0	3.403634745	0.301531632	1	RF00096
ENSG00000152969	2.098031101	0	3.403634745	0.301531632	1	JAKMIP1
ENSG00000226302	2.098031101	0	3.403634745	0.301531632	1	AC122683.1
ENSG00000234545	291.5692105	232.9312301	0.323739495	0.301543376	0.424858919	FAM133B
ENSG00000130414	1803.282392	2128.379235	-0.239110464	0.301575423	0.42488103	NDUFA10
ENSG00000212541	9.308416575	4.319762219	1.10974246	0.301750724	0.425104954	RNU6-510P
ENSG00000153879	840.8917194	718.4689507	0.226590807	0.301773391	0.425113836	CEBPG
ENSG00000266913	1.817398012	0.387220594	2.233687524	0.301781365	1	LINC01841
ENSG00000272582	7.163786657	2.91483114	1.306464774	0.301839231	0.425175281	AL031587.3
ENSG00000278918	16.49484018	25.86298953	-0.646200785	0.30185724	0.425175281	AC080112.3
ENSG00000231686	41.62998518	58.65927919	-0.492566578	0.301866103	0.425175281	Z97180.1
ENSG00000141434	3.685227059	0.356539013	3.25289226	0.302039932	0.425361189	MEP1B
ENSG00000262188	2.094270155	0	3.401212699	0.302046286	1	LINC01978
ENSG00000248529	2.094270155	0	3.401212699	0.302046286	1	AC026746.1
ENSG00000197616	2.094270155	0	3.401212699	0.302046286	1	MYH6
ENSG00000286733	2.094270155	0	3.401212699	0.302046286	1	AC090164.5
ENSG00000273890	2.094270155	0	3.401212699	0.302046286	1	AC034102.9
ENSG00000261603	2.094270155	0	3.401212699	0.302046286	1	PRSS46P
ENSG00000229268	2.094270155	0	3.401212699	0.302046286	1	PES1P2
ENSG00000205041	14.40816319	22.31651262	-0.627724972	0.302053912	0.425361189	AC118344.1
ENSG00000042317	189.2427868	155.6970027	0.282680952	0.302059854	0.425361189	AL049834.1
ENSG00000113300	807.5784269	690.4784826	0.226093529	0.302064533	0.425361189	CNOT6
ENSG00000221916	15.69452514	9.132998283	0.773534555	0.302079954	0.425361189	C19orf73
ENSG00000106025	52.376199	36.59549247	0.518646051	0.302120883	0.425395765	TSPAN12
ENSG00000234425	2.128261214	0	3.423027477	0.302129196	1	AL138930.1
ENSG00000198488	2.128261214	0	3.423027477	0.302129196	1	B3GNT6
ENSG00000277568	2.128261214	0	3.423027477	0.302129196	1	RF02271
ENSG00000285581	2.128261214	0	3.423027477	0.302129196	1	AC091196.1
ENSG00000242488	2.128261214	0	3.423027477	0.302129196	1	AF107885.1
ENSG00000287066	2.128261214	0	3.423027477	0.302129196	1	AP000962.3
ENSG00000247121	33.38148944	23.31877751	0.517033992	0.302141313	0.425401477	AC009126.1
ENSG00000275198	8.441301791	3.525251935	1.252919215	0.302234889	0.425507399	AL512791.2
ENSG00000213013	2.391271949	0.387220594	2.631502279	0.302255435	0.425507399	RPS15AP36
ENSG00000224401	1.215801261	3.240688571	-1.41570355	0.302265677	0.425507399	RPL7P57
ENSG00000162341	296.2497534	222.2148623	0.415003141	0.302323638	0.425565934	TPCN2
ENSG00000278737	1.525410814	0	2.941668656	0.302484027	1	AC013553.4
ENSG00000262497	1.525410814	0	2.941668656	0.302484027	1	FAM187B2P

ENSG00000252768	1.525410814	0	2.941668656	0.302484027	1	RNU6-856P
ENSG00000261615	1.525410814	0	2.941668656	0.302484027	1	LINC01858
ENSG00000266750	1.525410814	0	2.941668656	0.302484027	1	MIR4645
ENSG00000271723	1.525410814	0	2.941668656	0.302484027	1	MROH7-TTC4
ENSG00000214992	1.525410814	0	2.941668656	0.302484027	1	AKAP17BP
ENSG00000204252	1.525410814	0	2.941668656	0.302484027	1	HLA-DOA
ENSG00000173801	984.6879866	700.9820913	0.489932775	0.302502423	0.425794531	JUP
ENSG00000267284	3.685227059	0.336469917	3.252894234	0.302597589	0.425871069	AC022031.2
ENSG00000231638	0.616711807	2.547679642	-2.05423299	0.302617577	0.425871069	LUARIS
ENSG00000219186	4.554849141	1.854670712	1.316047549	0.302620937	0.425871069	FTH1P19
ENSG00000230701	2.464339932	6.339737595	-1.37370172	0.302628711	0.425871069	FBXW4P1
ENSG00000170684	39.60634698	55.2614583	-0.476633762	0.302668407	0.425871069	ZNF296
ENSG00000259005	2.387511003	0.387220594	2.629464986	0.302671675	0.425871069	AC005479.1
ENSG00000244459	16.82618904	26.74124382	-0.669540864	0.302677465	0.425871069	AC147067.1
ENSG00000227076	4.294345703	8.75395003	-1.028295176	0.302687932	0.425871069	AL158166.1
ENSG00000118849	306.3622297	621.9644575	-1.021768731	0.302729567	0.425906584	RARRES1
ENSG00000251988	5.027932279	1.518200796	1.755456905	0.302749134	0.42591105	RNU4ATAC18P
ENSG00000109180	1914.682413	2240.649147	-0.226959524	0.302797724	0.425956341	OCIAD1
ENSG00000103569	1.522903516	0	2.93957469	0.302946194	1	AQP9
ENSG00000260011	1.522903516	0	2.93957469	0.302946194	1	AC132938.1
ENSG00000272449	1.522903516	0	2.93957469	0.302946194	1	AL139246.5
ENSG00000141433	1.522903516	0	2.93957469	0.302946194	1	ADCYAP1
ENSG00000229436	1.522903516	0	2.93957469	0.302946194	1	AC073850.1
ENSG00000257588	1.522903516	0	2.93957469	0.302946194	1	AC025154.2
ENSG00000280060	1.522903516	0	2.93957469	0.302946194	1	AL512506.2
ENSG00000274776	7.220485935	2.792104814	1.359999853	0.302954875	0.426135293	AC090241.3
ENSG00000163702	991.4857669	848.39655	0.224884185	0.302972348	0.426135293	IL17RC
ENSG00000135974	431.0104437	517.2861193	-0.262749172	0.30297414	0.426135293	C2orf49
ENSG00000287896	2.12199297	0	3.419033332	0.302975766	1	AC073548.2
ENSG00000266846	2.12199297	0	3.419033332	0.302975766	1	AP001021.3
ENSG00000226995	2.12199297	0	3.419033332	0.302975766	1	LINC00658
ENSG00000268433	2.12199297	0	3.419033332	0.302975766	1	MTDHP3
ENSG00000160445	984.2695397	1242.581788	-0.335934661	0.302996579	0.426143783	ZER1
ENSG00000101868	352.1146685	443.0575768	-0.331890576	0.30306658	0.426219163	POLA1
ENSG00000177699	1.817398012	0.356539013	2.233688513	0.303198472	1	AC011944.1
ENSG00000244153	1.817398012	0.356539013	2.233688513	0.303198472	1	WWP1P1
ENSG00000170608	0.61420451	3.362259022	-2.453838898	0.303211206	0.426399477	FOXA3
ENSG00000167992	56.94963941	41.38275167	0.463385536	0.303394603	0.426634292	VWCE
ENSG00000230979	13.30450684	20.56681089	-0.626225422	0.303429783	0.426646045	AC079250.1
ENSG00000181577	12.41085161	5.899326178	1.08120628	0.303449535	0.426646045	C6orf223
ENSG00000276791	15.85201521	9.66070255	0.711269048	0.303452226	0.426646045	AC092117.1
ENSG00000107968	251.4006712	181.8928318	0.466046121	0.303482193	0.426665089	MAP3K8
ENSG00000254703	20.83112611	11.26277575	0.880919813	0.30352559	0.426675709	SENCR
ENSG00000285807	2.391271949	0.356539013	2.631490764	0.303544451	0.426675709	AL353768.2
ENSG00000210191	23.37451754	35.1648885	-0.593590108	0.303557728	0.426675709	MT-TL2
novel.977	14088.74822	5248.885724	1.424455619	0.303564348	0.426675709	-
ENSG00000286535	10.54058654	6.023336774	0.802149572	0.30357186	0.426675709	AC139019.1
ENSG00000200344	0.293240848	2.120320855	-2.706117982	0.303604737	1	RF00019
ENSG00000276712	0.89358395	2.934900236	-1.698073732	0.303707395	0.426832128	MIR7111
ENSG00000231742	7.661116398	14.0335272	-0.876352884	0.303716006	0.426832128	LINC01273
ENSG00000255521	8.092615314	3.270214277	1.312767306	0.303771558	0.426887108	AL356215.1

ENSG00000259467	0.308355904	2.079026788	-2.681853783	0.303796599	1	NDUFAF4P1
ENSG00000273415	0.308355904	2.079026788	-2.681853783	0.303796599	1	AP000904.1
ENSG00000279532	0.308355904	2.079026788	-2.681853783	0.303796599	1	AC002094.5
ENSG00000226945	0.308355904	2.079026788	-2.681853783	0.303796599	1	AC098935.1
ENSG00000268750	0.586481695	2.567748738	-2.105360808	0.303818481	0.426929955	AC010522.1
ENSG00000170236	1.817398012	0.336469917	2.233689164	0.304138826	1	USP50
ENSG00000270228	5.683721012	1.90542139	1.603737479	0.304149851	0.427372487	AC079880.1
ENSG00000285531	7.766637057	14.39135048	-0.890505739	0.304179576	0.42739114	Z83840.1
ENSG00000186019	6.67809576	12.25096053	-0.882628902	0.304288694	0.427518079	AC021092.1
ENSG00000251323	8.325395937	3.638521651	1.203100794	0.304302831	0.427518079	AP003086.1
ENSG00000257696	2.459325337	0.336469917	2.668236804	0.304419389	0.427616484	AC010203.2
ENSG00000249006	2.459325337	0.336469917	2.668236804	0.304419389	0.427616484	AL136360.1
ENSG00000119402	1575.188986	1372.081762	0.199064046	0.304422251	0.427616484	FBXW2
novel.362	0.293240848	2.079026788	-2.681829389	0.304569953	1	-
ENSG00000248100	0.293240848	2.079026788	-2.681829389	0.304569953	1	AC087257.1
ENSG00000164574	1696.485835	1164.242375	0.542952077	0.304600947	0.427844364	GALNT10
ENSG00000132405	319.4219179	393.444795	-0.300599408	0.304748807	0.428018735	TBC1D14
ENSG00000125730	16203.24373	8764.765649	0.886503667	0.304758038	0.428018735	C3
ENSG00000145349	1210.458437	984.5323921	0.297786488	0.304775448	0.428020049	CAMK2D
ENSG00000170482	14.1111614	8.142501754	0.789386975	0.304851441	0.428103632	SLC23A1
ENSG00000204086	0.61420451	2.51699806	-2.043393392	0.304983994	0.428266628	RPA4
ENSG00000168438	406.175848	466.5796954	-0.200441328	0.30510781	0.428417339	CDC40
novel.748	145.7589209	86.82539479	0.748430316	0.305335161	0.428713404	-
ENSG00000124160	832.4219282	691.9611816	0.266474884	0.305459308	0.428864538	NCOA5
ENSG00000276633	0.307102255	2.099095884	-2.693926751	0.305516566	1	AJ011931.1
ENSG00000002586	6054.672174	7271.303474	-0.264165424	0.305559838	0.4289825	CD99
ENSG00000259459	0.923814062	3.688116453	-2.00031354	0.305603806	0.429006517	LINC02568
ENSG00000124140	8.066146148	17.9328187	-1.146410592	0.30560997	0.429006517	SLC12A5
ENSG00000261338	46.30464366	34.17999577	0.442421715	0.305694864	0.429089484	AC021016.2
ENSG00000279571	2.429095225	0.387220594	2.651982768	0.305702935	0.429089484	AL162426.1
ENSG00000196172	54.48329072	36.62771511	0.568688649	0.305718619	0.429089484	ZNF681
ENSG00000263603	3.56298169	8.497756497	-1.242429552	0.305816311	0.429203413	AC127024.2
ENSG00000063127	34.66025823	24.49819664	0.500948409	0.305888145	0.42928104	SLC6A16
ENSG00000102974	905.4667177	757.0737249	0.2579145	0.305913447	0.429293359	CTCF
ENSG00000287211	3.225649323	0	4.024537666	0.306015286	0.429320328	AL121904.2
ENSG00000249553	3.225649323	0	4.024537666	0.306015286	0.429320328	PPP2R2B-IT1
ENSG00000182722	3.225649323	0	4.024537666	0.306015286	0.429320328	SEPHS1P1
ENSG00000282870	3.225649323	0	4.024537666	0.306015286	0.429320328	FRG1DP
ENSG00000236358	3.225649323	0	4.024537666	0.306015286	0.429320328	AL355472.2
ENSG00000235902	2.436617117	0.356539013	2.656024142	0.306105948	0.429424332	AC108472.1
ENSG00000113558	3106.856472	3672.828511	-0.241502698	0.306143231	0.429447389	SKP1
ENSG00000263264	7.178901713	3.159256312	1.176210126	0.306155442	0.429447389	AC119396.1
ENSG00000204173	12.95582036	7.785962742	0.728954781	0.306397374	0.429763546	LRRC37A5P
ENSG00000164323	707.6970677	868.5895303	-0.295902359	0.306447408	0.429810522	CFAP97
ENSG00000206885	4.178439849	1.447381022	1.538002027	0.306521327	0.429888213	SNORA75
ENSG00000188152	28.63515917	19.60828021	0.544927377	0.306535893	0.429888213	NUTM2G
ENSG00000181381	668.4993686	790.8721719	-0.242688441	0.306604976	0.429961888	DDX60L
ENSG00000213420	87.99014574	110.9329032	-0.332171636	0.306675348	0.430017125	GPC2
ENSG00000233469	7.777991167	3.016332495	1.386143437	0.306677468	0.430017125	ST6GALNAC4P1
ENSG00000255202	1.201939854	3.801514564	-1.666931867	0.306721037	0.430055007	AL049629.1
ENSG00000100836	2437.41794	2067.035552	0.237904001	0.306750644	0.430073308	PABPN1

ENSG00000128683	3.933051467	0.774441189	2.379031455	0.306807816	0.430130254	GAD1
ENSG00000279339	8.646359599	15.45968325	-0.833320172	0.306875573	0.430202032	AC100788.2
ENSG00000277150	10.03727329	5.768299117	0.800279465	0.306902691	0.430216834	F8A3
ENSG00000286981	3.620934617	1.41669944	1.345491634	0.307088628	0.430454256	AL731553.1
ENSG00000253716	59.1687335	74.23950541	-0.326827286	0.30721258	0.430604772	MINCR
ENSG00000241679	6.459176544	1.762625967	1.86555061	0.307285409	0.430683616	AC018450.1
ENSG00000270585	2.683259148	0.743759607	1.879782821	0.307350143	0.43075111	AL445435.1
ENSG00000008323	10.17427771	17.0594447	-0.744411491	0.307395651	0.430765587	PLEKHG6
ENSG00000198874	313.1273977	386.3780064	-0.304074955	0.307421927	0.430765587	TYW1
ENSG00000137166	695.7890023	810.1460975	-0.219749502	0.307441175	0.430765587	FOXP4
ENSG00000151846	9.3688768	15.27662124	-0.700352406	0.307448241	0.430765587	PABPC3
ENSG00000117020	65.64413656	98.43512423	-0.585887602	0.307463946	0.430765587	AKT3
ENSG00000229852	78.26115709	56.63511268	0.463128101	0.307474659	0.430765587	AC019205.1
ENSG00000261239	2.682005499	0.743759607	1.879238173	0.307476533	0.430765587	ANKRD26P1
ENSG00000227331	2.710981963	0.672939833	1.967050262	0.307578941	0.430885823	AC005042.1
ENSG00000225265	15.18361872	27.36001253	-0.852610034	0.307630463	0.430934764	TAF1A-AS1
ENSG00000287574	18.44361511	8.55094732	1.106217151	0.307720666	0.431037881	AC004938.2
novel.889	0	1.874739808	-3.483162647	0.307740319	1	-
ENSG00000231292	0	1.874739808	-3.483162647	0.307740319	1	IGKV1OR2-108
ENSG00000170965	0	1.874739808	-3.483162647	0.307740319	1	PLAC1
ENSG00000249112	0	1.874739808	-3.483162647	0.307740319	1	AC112196.1
ENSG00000285642	0	1.874739808	-3.483162647	0.307740319	1	AL139330.1
ENSG00000253286	0	1.874739808	-3.483162647	0.307740319	1	AC135166.1
ENSG00000183242	0	1.874739808	-3.483162647	0.307740319	1	WT1-AS
ENSG00000281186	0	1.874739808	-3.483162647	0.307740319	1	LINC00706
ENSG00000257918	0	1.874739808	-3.483162647	0.307740319	1	AC079385.3
ENSG00000101278	0	1.874739808	-3.483162647	0.307740319	1	RPS10P5
ENSG00000188992	5.479916852	2.200597239	1.325690195	0.307909396	0.431278992	LIPI
ENSG00000235121	4.288077459	10.09995809	-1.243728441	0.307929108	0.43128335	AL645504.1
ENSG00000213432	0.586481695	2.578361223	-2.110596613	0.308025369	0.431394918	RPL17P34
ENSG00000276831	1.495180701	0	2.916111202	0.308145524	1	AC020922.4
ENSG00000225195	1.495180701	0	2.916111202	0.308145524	1	AC078962.1
ENSG00000240567	1.495180701	0	2.916111202	0.308145524	1	LINC02067
ENSG00000234065	1.495180701	0	2.916111202	0.308145524	1	MTND4P26
ENSG00000265542	1.495180701	0	2.916111202	0.308145524	1	AC015845.2
ENSG00000270457	1.495180701	0	2.916111202	0.308145524	1	AC093424.1
ENSG00000248228	1.495180701	0	2.916111202	0.308145524	1	SLIT2-IT1
ENSG00000252695	1.495180701	0	2.916111202	0.308145524	1	MIR2276
ENSG00000244104	1.495180701	0	2.916111202	0.308145524	1	RN7SL659P
ENSG00000272988	1.495180701	0	2.916111202	0.308145524	1	AC022392.1
ENSG00000273791	1.495180701	0	2.916111202	0.308145524	1	AC007204.1
ENSG00000149260	988.1585591	788.4296715	0.32606502	0.308161685	0.431558934	CAPN5
ENSG00000198885	57.9765381	44.82901149	0.371369673	0.308175701	0.431558934	ITPRIPL1
ENSG00000103160	613.4277741	506.4784298	0.276770132	0.308199611	0.431564175	HSDL1
ENSG00000234936	10.56454841	6.003267678	0.808035459	0.308220566	0.431564175	AC010883.1
ENSG00000276903	0.909952655	2.934900236	-1.683461286	0.308229276	0.431564175	HIST1H2AL
ENSG00000241449	2.082916045	0	3.393817034	0.308249557	1	AC092666.1
ENSG00000206965	2.082916045	0	3.393817034	0.308249557	1	RNU6-5P
ENSG00000271742	2.082916045	0	3.393817034	0.308249557	1	AL121992.2
ENSG00000286142	2.082916045	0	3.393817034	0.308249557	1	AL122018.2
ENSG00000163630	2.082916045	0	3.393817034	0.308249557	1	SYNPR

ENSG00000265246	2.082916045	0	3.393817034	0.308249557	1	AC129926.1
ENSG00000145425	11358.47388	13892.00614	-0.29049797	0.308258673	0.431582077	RPS3A
ENSG00000241439	0	2.71054416	-4.015511187	0.308380198	0.431705691	AC092903.1
ENSG00000272028	0	2.71054416	-4.015511187	0.308380198	0.431705691	RNU6-87P
ENSG00000235128	0.923814062	2.904218654	-1.659467953	0.308401045	0.431711614	AC013474.1
ENSG00000196976	318.9767276	426.1325456	-0.418409913	0.308567527	0.43192139	LAGE3
ENSG00000213757	2.753819833	6.05286248	-1.14394937	0.308604016	0.431949194	AC020898.1
ENSG00000236024	0	2.691759334	-4.01329691	0.30865367	0.431989265	PRRX2-AS1
ENSG00000182324	37.04755577	25.01173958	0.566592487	0.308665899	0.431989265	KCNJ14
ENSG00000254409	4.850597286	1.548882377	1.681613507	0.308745429	0.432077295	AC087521.3
ENSG00000279378	2.080408748	0	3.392188838	0.308769686	1	AC009159.4
ENSG00000232097	2.080408748	0	3.392188838	0.308769686	1	AC079781.3
ENSG00000270986	2.080408748	0	3.392188838	0.308769686	1	HMGB1P51
ENSG00000269043	2.080408748	0	3.392188838	0.308769686	1	AC008554.2
ENSG00000244932	30.89709113	42.21038368	-0.448866533	0.308961919	0.432356976	AL449212.1
ENSG00000234134	2.739958426	5.797824823	-1.081031752	0.30903712	0.43243892	AL158835.2
ENSG00000131931	148.5586397	179.9260004	-0.276125685	0.309175878	0.432609785	THAP1
novel.430	8.441301791	15.96950177	-0.912591295	0.309242765	0.432680073	-
ENSG00000241985	5.337541832	1.742556871	1.604688898	0.309275455	0.432702509	WWTR1-IT1
ENSG00000259571	1.17296339	5.503933243	-2.224734037	0.309442717	0.432913211	BLID
ENSG00000183598	0.601596751	2.882993683	-2.256982553	0.309604984	0.433116901	HIST2H3D
ENSG00000163848	666.4220525	570.2208672	0.225253575	0.309805123	0.433356955	ZNF148
ENSG00000150712	642.0528321	546.0323246	0.233135738	0.309809942	0.433356955	MTMR12
ENSG00000262052	4.133094681	0.774441189	2.450570804	0.309862689	0.433407404	AC090618.1
ENSG00000119314	989.945522	1226.704693	-0.309577827	0.309884777	0.433414966	PTBP3
ENSG00000105618	6.379769046	11.39495869	-0.840322447	0.310057057	0.433632578	PRPF31
ENSG00000286895	6.311786929	1.782695063	1.821601279	0.310101592	0.433671518	AC092745.5
ENSG00000158023	81.27164814	63.81638682	0.347325102	0.310286852	0.433907245	WDR66
ENSG00000162623	438.1431464	515.0014394	-0.233311758	0.310399431	0.434041313	TYW3
ENSG00000214140	70.36092055	98.19296395	-0.478490655	0.310488656	0.434142715	PRCD
ENSG00000143793	626.9382795	524.3374063	0.257576618	0.310530763	0.434178224	C1orf35
ENSG00000272226	4.897196103	1.426156051	1.774457548	0.310605306	0.434259079	AL136985.2
ENSG00000287586	0.308355904	2.058957692	-2.669431855	0.310609074	1	Z98886.1
novel.550	7.838451391	3.270214277	1.266087775	0.310667622	0.434300061	-
ENSG00000064763	248.5870875	331.8270104	-0.417472943	0.310668051	0.434300061	FAR2
ENSG00000259516	2.709728314	5.819049794	-1.094964214	0.310722392	0.434349473	ANP32AP1
ENSG00000112787	866.0556159	723.4115316	0.259918024	0.310736833	0.434349473	FBRSL1
ENSG00000249492	17.18970584	11.19897244	0.622362956	0.310802113	0.434417349	AC114956.3
ENSG00000232412	3.016830569	0.774441189	2.008644604	0.310912457	0.434548203	AL121601.1
ENSG00000228623	23.7670108	15.77492819	0.59232768	0.31098569	0.434609653	ZNF883
ENSG00000263648	2.726097019	0.713078025	1.928948336	0.310990113	0.434609653	AC005244.1
ENSG00000109163	5.129976726	2.058957692	1.292552795	0.311025186	0.434609653	GNRHR
ENSG00000100461	1144.462806	981.7559432	0.221038586	0.31103427	0.434609653	RBM23
ENSG00000163605	1283.833537	1132.465111	0.181093768	0.311040064	0.434609653	PPP4R2
ENSG00000266970	8.44255544	4.453101031	0.941175277	0.311105764	0.434678078	AC061992.1
ENSG00000263968	1.511549406	4.473170127	-1.555961835	0.31117947	0.434757679	RN7SL381P
ENSG00000149658	913.0671472	1038.347356	-0.185476417	0.311351946	0.43495536	YTHDF1
ENSG00000172572	80.82170015	309.0691186	-1.935249404	0.311362614	0.43495536	PDE3A
ENSG00000253967	0.293240848	2.058957692	-2.669401795	0.31138206	1	AC022730.4
ENSG00000254990	0.879722543	3.271370153	-1.875130485	0.311382684	0.43495536	AP001781.1
ENSG00000219023	5.478663203	2.446178286	1.145723572	0.311401994	0.43495536	AL033519.2

ENSG00000141258	1293.556344	1050.055013	0.301201405	0.311404667	0.43495536	SGSM2
ENSG00000163608	826.9432215	715.6088858	0.208609035	0.311453657	0.43498585	NEPRO
ENSG00000132406	481.6544223	416.1689144	0.210331175	0.311459981	0.43498585	TMEM128
ENSG00000267919	2.376156893	0.387220594	2.623162049	0.311609396	0.435171131	AC009955.2
ENSG00000279727	2.157237677	0	3.441497181	0.311687257	1	LINC02033
ENSG00000231739	2.157237677	0	3.441497181	0.311687257	1	GAPDHP59
ENSG00000254041	2.157237677	0	3.441497181	0.311687257	1	AC021546.1
ENSG00000242324	2.157237677	0	3.441497181	0.311687257	1	AL049634.1
ENSG00000287430	2.157237677	0	3.441497181	0.311687257	1	AL136090.2
ENSG00000281772	1.832513069	0.387220594	2.243553595	0.311757828	1	AC019226.2
ENSG00000249437	1.832513069	0.387220594	2.243553595	0.311757828	1	NAIP
ENSG00000176428	65.68725916	91.32094577	-0.474357861	0.311787542	0.435396513	VPS37D
ENSG00000267315	2.373649595	0.387220594	2.621791814	0.31189265	0.435519883	AC060766.3
ENSG00000139372	503.0022527	610.2446991	-0.279306962	0.311953811	0.435581876	TDG
ENSG00000272905	6.956221551	3.200550379	1.116011819	0.312063074	0.435711024	AC018648.1
ENSG00000249207	34.15415294	25.45685571	0.424072943	0.312119481	0.435766362	AC079921.1
ENSG00000272049	4.178439849	1.406086955	1.562086589	0.312172972	0.435817623	AC091965.4
ENSG00000131508	1763.381082	2002.767207	-0.183568268	0.312319796	0.435984667	UBE2D2
ENSG00000233602	0.586481695	2.537067156	-2.091496774	0.312342966	0.435984667	ERI3-IT1
ENSG00000251523	0.586481695	2.537067156	-2.091496774	0.312342966	0.435984667	AC108159.1
ENSG00000250412	3.938137334	1.436768536	1.454783422	0.312412472	0.436058259	KLHL2P1
ENSG00000279862	0	1.844058227	-3.461175302	0.312417787	1	AC092964.1
ENSG00000241026	0	1.844058227	-3.461175302	0.312417787	1	AC105910.1
ENSG00000226115	0	1.844058227	-3.461175302	0.312417787	1	AP001476.2
ENSG00000188013	17.21388117	8.011474693	1.095442952	0.312510443	0.436159639	MEIS3P2
ENSG00000184221	2.150969433	0	3.437535322	0.312512378	1	OLIG1
ENSG00000256379	2.150969433	0	3.437535322	0.312512378	1	TRAV8-5
ENSG00000175779	2.150969433	0	3.437535322	0.312512378	1	C15orf53
ENSG00000230098	2.150969433	0	3.437535322	0.312512378	1	TCERG1L-AS1
ENSG00000211784	2.150969433	0	3.437535322	0.312512378	1	TRAV10
ENSG00000260691	2.418994764	0.693008929	1.775640793	0.312525918	0.436159639	ANKRD20A1
ENSG00000253406	1.17296339	5.53217468	-2.226046788	0.312535645	0.436159639	AC012613.2
novel.524	27.31390852	37.82681815	-0.466864947	0.312552256	0.436159639	-
ENSG00000258245	0.308355904	2.169915658	-2.736117466	0.312591633	1	RPL10P13
ENSG00000176692	335.5891094	677.0035675	-1.012679248	0.312738756	0.436396456	FOXC2
ENSG00000145191	905.9691646	1072.667532	-0.243420677	0.312763052	0.43640692	EIF2B5
ENSG00000168566	336.1564206	408.089608	-0.27940105	0.313003758	0.43671933	SNRNP48
ENSG00000232814	8.108984019	14.25544313	-0.806787519	0.313024492	0.436724806	COL4A2-AS1
ENSG00000147164	1170.895082	1363.245656	-0.219586626	0.31308489	0.436785617	SNX12
ENSG00000248576	1.832513069	0.356539013	2.243569644	0.313136683	1	AC023794.2
ENSG00000283828	7.226754179	3.537020296	1.024936831	0.313161398	0.436840654	AL137002.2
ENSG00000206991	2.373649595	0.356539013	2.621774565	0.313162647	0.436840654	RNU6-610P
ENSG00000286616	9.236602241	3.384768263	1.432663085	0.313174782	0.436840654	AL008730.1
ENSG00000276550	982.204358	815.7055548	0.268198716	0.313204538	0.436858705	HERC2P2
ENSG00000130167	1.83125942	0.356539013	2.242758982	0.313316424	1	TSPAN16
ENSG00000128886	6.362146692	2.862924587	1.149712976	0.313335757	0.437018268	ELL3
ENSG00000259959	62.95794284	81.32184673	-0.367671928	0.313358081	0.437025943	AC107068.1
ENSG00000188269	2.14337627	0	3.432697734	0.313519104	1	OR7A5
ENSG00000174403	2.14337627	0	3.432697734	0.313519104	1	MIR1-1HG-AS1
ENSG00000213075	2.14337627	0	3.432697734	0.313519104	1	RPL31P11
ENSG00000112195	2.14337627	0	3.432697734	0.313519104	1	TREML2

ENSG00000258689	2.14337627	0	3.432697734	0.313519104	1	LINC01269
ENSG00000213438	2.14337627	0	3.432697734	0.313519104	1	YBX2P1
ENSG00000120436	2.14337627	0	3.432697734	0.313519104	1	GPR31
ENSG00000172803	57.78387459	42.35086735	0.451300357	0.313640121	0.437395812	SNX32
ENSG00000225026	2.376156893	0.336469917	2.62313507	0.313716091	0.437455125	AC091492.1
ENSG00000140992	705.3375517	606.846703	0.217063824	0.313729024	0.437455125	PDPK1
ENSG00000237054	3.308817768	7.693789602	-1.210743752	0.313733164	0.437455125	PRMT5-AS1
ENSG00000065615	294.8257582	246.1210377	0.26076883	0.313761223	0.43747077	CYB5R4
ENSG00000284240	0	1.90542139	-3.505146498	0.313787084	1	AC099062.1
ENSG00000242593	0	1.90542139	-3.505146498	0.313787084	1	AC006148.1
ENSG00000177257	0	1.90542139	-3.505146498	0.313787084	1	DEFB4B
ENSG00000250896	6.378515397	10.81290772	-0.761323802	0.313790155	0.437487632	RNPS1P1
ENSG00000070193	1.850135422	8.492024301	-2.205137699	0.313880692	0.437590377	FGF10
ENSG00000206634	2.373649595	0.336469917	2.621763228	0.314002379	0.43771525	SNORA22
ENSG00000278408	0	1.834601616	-3.45402407	0.314016111	1	AC108861.1
ENSG00000262358	0	1.834601616	-3.45402407	0.314016111	1	AC116914.1
ENSG00000265380	0	1.834601616	-3.45402407	0.314016111	1	AC079070.1
ENSG00000286502	6.568458149	12.35130601	-0.910497566	0.31401801	0.43771525	AL645768.1
ENSG00000051108	1860.074547	2442.614379	-0.393096053	0.314020805	0.43771525	HERPUD1
ENSG00000185049	653.7187586	552.8958327	0.241828441	0.314078114	0.437771647	NELFA
ENSG00000259706	4.841750474	9.802342097	-1.014933979	0.31411165	0.437794903	HSP90B2P
ENSG00000257139	3.28360225	1.029478846	1.647644093	0.314170486	0.437853417	AC084032.1
ENSG00000161594	1.83125942	0.336469917	2.24276871	0.314231931	1	KLHL10
ENSG00000223953	1.83125942	0.336469917	2.24276871	0.314231931	1	C1QTNF5
ENSG00000214832	12.30253892	6.676207511	0.87544434	0.314255317	0.437938902	UPF3AP2
ENSG00000103460	3.083559037	0	3.957541854	0.314282392	0.437938902	TOX3
ENSG00000235615	3.083559037	0	3.957541854	0.314282392	0.437938902	AJ239322.1
ENSG00000182873	18.09840484	25.59965114	-0.497116421	0.314452278	0.438152131	PRKCZ-AS1
ENSG00000287745	2.135854377	0	3.42791497	0.314518667	1	AL359380.1
ENSG00000285045	2.135854377	0	3.42791497	0.314518667	1	AC006548.3
ENSG00000242790	2.135854377	0	3.42791497	0.314518667	1	AC104831.1
ENSG00000243103	2.135854377	0	3.42791497	0.314518667	1	RN7SL452P
ENSG00000268093	6.917144626	3.597227584	0.946829828	0.314531555	0.438239091	AC022154.1
ENSG00000224316	3.315086011	1.029478846	1.659333122	0.314598035	0.438308213	GTF2IP5
ENSG00000222011	91.92996365	70.31770466	0.385833848	0.314752649	0.438480552	FAM185A
ENSG00000232725	21.80598412	11.74790168	0.898643075	0.314755487	0.438480552	U52111.1
ENSG00000275212	3.518890171	0.336469917	3.188905204	0.314784982	0.438498129	AC005186.1
ENSG00000089486	69.65165185	53.64548056	0.376740936	0.314990096	0.438694674	CDIP1
ENSG00000281769	3.07102255	0	3.951833342	0.314993616	0.438694674	LINC01230
ENSG00000226516	3.07102255	0	3.951833342	0.314993616	0.438694674	FAM138B
ENSG00000079689	3.07102255	0	3.951833342	0.314993616	0.438694674	SCGN
ENSG00000114405	143.8623845	180.7533753	-0.330556407	0.315021522	0.438710021	C3orf14
ENSG00000272529	4.855611881	1.702418679	1.484799747	0.315086369	0.438746551	AC090948.3
ENSG00000250090	4.855611881	1.702418679	1.484799747	0.315086369	0.438746551	AL390198.2
ENSG00000227258	7.077142	2.099095884	1.743403387	0.315098414	0.438746551	SMIM2-AS1
ENSG00000138207	11.71752434	63.55592093	-2.440050927	0.31512128	0.438754875	RBP4
ENSG00000163915	2.459325337	6.04340587	-1.305441478	0.315169582	0.438798612	IGF2BP2-AS1
ENSG00000265321	2.391271949	5.768299117	-1.263516334	0.315208768	0.438829654	MIR4263
ENSG00000196754	33.954181	47.160379	-0.470389725	0.315373417	0.439035351	S100A2
ENSG00000139197	965.0366373	782.7669365	0.302373348	0.315430754	0.439091643	PEX5
ENSG00000170584	583.3348573	697.44145	-0.257918816	0.315462954	0.439112939	NUDCD2

ENSG00000253139	1.816144364	0.356539013	2.232882428	0.315507958	1	AC013644.1
ENSG00000259628	1.816144364	0.356539013	2.232882428	0.315507958	1	AC007000.3
ENSG00000082641	5884.512054	7139.51916	-0.278908736	0.31562218	0.439311039	NFE2L1
ENSG00000118096	452.0893661	379.7311494	0.251587214	0.315697079	0.439391751	IFT46
ENSG00000286672	3.870083945	7.907533193	-1.023504998	0.315726351	0.43940422	AL096712.2
ENSG00000258745	4.1771862	1.487519214	1.512431616	0.315753359	0.43940422	AL358334.3
ENSG00000084110	5.364010998	1.90542139	1.522458495	0.315756776	0.43940422	HAL
novel.1090	111.1482507	66.76339342	0.734470217	0.315913908	0.439586679	-
ENSG00000274501	0.616711807	2.598430319	-2.077735138	0.315923773	0.439586679	AL121845.4
ENSG00000166173	427.3885521	624.8969488	-0.548136137	0.31593865	0.439586679	LARP6
ENSG00000254391	1.803536605	0.387220594	2.224611176	0.31594395	1	AC011363.1
ENSG00000069812	2.14337627	5.797824823	-1.438142945	0.315983563	0.439625627	HES2
ENSG00000230154	6.985198015	12.41254078	-0.825852071	0.316051677	0.439696847	AC018463.1
ENSG00000233154	1.818651661	0.336469917	2.234511874	0.316069804	1	LINC01762
ENSG00000259130	1.802282956	0.387220594	2.223795167	0.316125769	1	AL133371.3
ENSG00000246375	12.2021754	4.291392388	1.506632363	0.316214445	0.439883584	PPM1K-DT
ENSG00000176845	134.4147566	86.96587847	0.629576238	0.316219764	0.439883584	METRNL
ENSG00000010803	636.0865992	535.1639146	0.249383093	0.316291305	0.439952907	SCMH1
ENSG00000172426	42.53750181	30.37732533	0.491985819	0.316303466	0.439952907	RSPH9
ENSG00000255408	8.571069053	3.505182839	1.27967085	0.316332933	0.439970339	PCDHA3
ENSG00000234632	1.816144364	0.336469917	2.232882142	0.316434636	1	NECAP1P2
ENSG00000186130	227.1322697	266.2087117	-0.228891693	0.316729911	0.440498893	ZBTB6
ENSG00000242094	3.376799885	0.723690511	2.225186817	0.316766523	0.44052623	FOXP1-IT1
ENSG00000188883	3.37178529	1.049547942	1.664224565	0.316823235	0.440581516	KLRG2
ENSG00000127928	0	1.885352294	-3.490803403	0.317006229	1	GNGT1
ENSG00000187959	5.095985668	1.406086955	1.849851444	0.31708204	0.44089962	CPSF4L
ENSG00000274341	8.163176	3.749479616	1.140384363	0.317085924	0.44089962	AC005899.6
ENSG00000261229	0.61420451	2.578361223	-2.07163883	0.317259629	0.441117543	AC021483.2
novel.799	3.619680969	1.467450118	1.319680403	0.317459031	0.441300974	-
ENSG00000279259	19.5898246	12.9521418	0.599170498	0.317472594	0.441300974	AC087741.3
ENSG00000278997	27.76206087	18.57764549	0.57637094	0.317483005	0.441300974	AL662907.1
ENSG00000213085	9.547465442	18.13723408	-0.921164598	0.31748394	0.441300974	CFAP45
ENSG00000120733	1463.730583	1250.851895	0.226911118	0.317488894	0.441300974	KDM3B
ENSG00000185730	207.5707095	175.9830559	0.23881844	0.317493469	0.441300974	ZNF696
ENSG00000175985	4.204909015	1.518200796	1.502430119	0.317530095	0.441328273	PLEKHD1
ENSG00000269444	0.925067711	3.475657132	-1.924471206	0.317555597	0.441340107	AC011491.2
ENSG00000279930	5.68497466	2.446178286	1.205304838	0.317585654	0.44135827	AL032819.2
ENSG00000126698	1371.759189	1577.364624	-0.201595005	0.317649313	0.44141767	DNAJC8
ENSG00000180596	16.23086053	27.06581699	-0.737144337	0.317662375	0.44141767	HIST1H2BC
ENSG00000241680	3.619680969	1.069617038	1.754695432	0.317721263	0.441475886	RPL31P49
ENSG00000127337	236.275247	289.4389589	-0.293752512	0.31780265	0.441552115	YEATS4
ENSG00000100285	64.22216603	133.4969359	-1.056468668	0.317810113	0.441552115	NEFH
ENSG00000068078	254.2103049	144.9212919	0.80972822	0.318130687	0.441970532	FGFR3
ENSG00000287620	8.531992128	4.637318915	0.874429376	0.318145294	0.441970532	AC092053.4
ENSG00000228801	89.70341894	67.91307723	0.399044758	0.318178604	0.441993173	AC064807.1
ENSG00000233862	1.803536605	0.336469917	2.224588742	0.318291301	1	AC016907.2
ENSG00000187589	1.803536605	0.336469917	2.224588742	0.318291301	1	TERF1P2
ENSG00000231794	1.803536605	0.336469917	2.224588742	0.318291301	1	AC009542.1
ENSG00000183396	1.535511275	5.422372589	-1.823696194	0.318299891	0.442138017	TMEM89
ENSG00000257175	7.601909822	3.331577441	1.201720419	0.318503798	0.442397604	CR383656.1
ENSG00000260369	4.240153722	1.396630344	1.587986569	0.318861513	0.442868951	AC120024.1

ENSG00000278899	7.23685464	3.088436538	1.209773683	0.318877236	0.442868951	AL358852.1
ENSG00000075275	20.89373763	12.92390036	0.683833641	0.318922596	0.442903706	CELSR1
ENSG00000189045	15.25543306	8.735165204	0.793108478	0.318936355	0.442903706	ANKDD1B
ENSG00000260487	2.465593581	0.387220594	2.671741037	0.319041345	0.443025825	AC116348.1
ENSG00000104852	6100.989553	7572.031075	-0.311607442	0.319151093	0.443154538	SNRNP70
ENSG00000233893	3.368024343	1.029478846	1.679535147	0.319376059	0.443432229	EZR-AS1
ENSG00000135452	623.8610154	536.4874606	0.217434705	0.319385216	0.443432229	TSPAN31
novel.879	3.895299463	9.081091731	-1.222730495	0.319455602	0.443506252	-
ENSG00000119608	14.64595841	7.35616381	1.001651235	0.319536877	0.443595383	PROX2
ENSG00000277232	9.362608556	5.299646263	0.813134753	0.319592319	0.44363406	GTSE1-DT
ENSG00000269481	10.91574218	6.277090162	0.811434444	0.319598888	0.44363406	AC010319.4
ENSG00000112200	777.8537728	667.553557	0.22094766	0.319676925	0.443703254	ZNF451
ENSG00000229081	5.795865919	2.058957692	1.472457175	0.319682892	0.443703254	LINC01165
ENSG00000174483	13.8141596	22.74387141	-0.716321388	0.319752946	0.443776779	BBS1
ENSG00000207554	10.81745868	17.06992879	-0.651531422	0.319799239	0.443794576	MIR647
ENSG00000232748	5.420710276	2.446178286	1.133736075	0.319799933	0.443794576	AC135050.1
ENSG00000133243	1924.015234	2292.414059	-0.252603403	0.319824199	0.443804545	BTBD2
ENSG00000272070	10.30780592	6.094156548	0.757103655	0.319990005	0.444010913	AC005618.1
ENSG00000205571	16.15381814	6.32784084	1.359243559	0.32004157	0.444042111	SMN2
novel.945	6.066398547	10.07860473	-0.732149579	0.320046671	0.444042111	-
ENSG00000103978	1127.310895	984.0795582	0.196235328	0.320070203	0.444051046	TMEM87A
ENSG00000162076	568.1175472	666.0215932	-0.22945849	0.320143238	0.444128656	FLYWCH2
ENSG00000153495	6.27647095	1.936102971	1.724147416	0.32019151	0.444171905	TEX29
ENSG00000267277	7.308668974	3.678659843	1.001770695	0.320241473	0.444217497	AC024575.1
ENSG00000025156	238.0656342	280.403224	-0.236826127	0.320289354	0.444260194	HSF2
ENSG00000220583	0.600343103	2.211209725	-1.865667805	0.320364798	0.444341117	RPL35P2
ENSG00000087338	336.3953544	390.6387516	-0.216067941	0.320405605	0.444373994	GMCL1
ENSG00000156983	486.7788584	573.5726553	-0.236447398	0.320429999	0.444384104	BRPF1
ENSG00000135587	141.1242492	174.4908791	-0.306954919	0.320447349	0.444384445	SMPD2
ENSG00000237135	3.632288727	1.41669944	1.349366918	0.32049116	0.444421478	DDX10P1
ENSG00000164077	358.3262772	288.9887529	0.309647645	0.320590301	0.444535231	MON1A
ENSG00000170906	0.601596751	2.618499415	-2.109205354	0.320613968	0.444544322	NDUFA3
ENSG00000286365	4.175932552	1.161661783	1.883050348	0.320834168	0.444825899	AP000553.9
ENSG00000136114	127.1854795	80.22827343	0.662399825	0.320927069	0.44493096	THSD1
ENSG00000129988	0.586481695	3.088436538	-2.388369766	0.321024397	0.445042146	LBP
ENSG00000120685	449.8575937	540.2713459	-0.264614245	0.321192389	0.445251278	PROSER1
ENSG00000136040	459.6844212	848.2613368	-0.883769345	0.321327169	0.445360573	PLXNC1
ENSG00000273391	8.484139662	13.41133804	-0.657092499	0.32133803	0.445360573	AC083880.1
ENSG00000231181	6.627664725	2.844011366	1.217736342	0.321338793	0.445360573	AL954705.1
ENSG00000121005	273.5465068	84.41726538	1.69573644	0.321339798	0.445360573	CRISPLD1
ENSG00000279039	1.229662669	3.52640781	-1.533419159	0.321357896	0.445361898	AC011447.6
ENSG00000275092	1.17296339	3.9336975	-1.732048418	0.32146257	0.4454832	AL031710.2
ENSG00000137100	1029.662747	1226.87187	-0.252937624	0.321543047	0.445542419	DCTN3
ENSG00000207088	0.894837599	2.985650913	-1.718541774	0.321551447	0.445542419	SNORA7B
ENSG00000203279	3.068443981	7.827256809	-1.35631605	0.321556749	0.445542419	AL590705.1
ENSG00000225339	14.99213759	8.815441588	0.761237522	0.321647549	0.445644463	AL354740.1
ENSG00000272663	10.46375761	5.379922647	0.961643629	0.321665575	0.445645672	AC093635.1
ENSG00000231369	2.451732174	0.356539013	2.664276524	0.321734414	0.445717278	Z97353.1
ENSG00000171298	2543.58357	2113.018926	0.26769774	0.32184282	0.445843684	GAA
ENSG00000271789	2.654282684	6.857985251	-1.359650137	0.322036245	0.446087849	AL080317.2
ENSG00000249786	4.592601146	1.773238453	1.363409648	0.322114082	0.446171881	EAF1-AS1

ENSG00000213608	0	1.783850939	-3.416628296	0.322118002	1	SLC25A14P1
ENSG00000286268	0	1.783850939	-3.416628296	0.322118002	1	AF196969.1
ENSG00000139974	225.0876473	263.7391723	-0.228736925	0.32217199	0.446228301	SLC38A6
ENSG00000136943	15.4503904	22.67651926	-0.552722428	0.322210674	0.446249473	CTSV
ENSG00000235481	7.23685464	3.586615098	1.013077326	0.322221628	0.446249473	UBE2R2-AS1
ENSG00000127507	102.3072626	129.8827089	-0.343460858	0.322604032	0.446754015	ADGRE2
ENSG00000130559	810.0319978	693.8314134	0.223549753	0.322620331	0.446754015	CAMSAP1
ENSG00000250986	19.71339454	6.453135705	1.603569183	0.322669095	0.446797727	LINC02600
ENSG00000272516	2.442956632	0.356539013	2.659545911	0.32271366	0.446835621	AL158211.4
ENSG00000286145	0	2.495773089	-3.901177557	0.322713926	1	AC068282.1
ENSG00000278275	0	2.495773089	-3.901177557	0.322713926	1	AC103705.1
ENSG00000125962	153.0767054	188.308351	-0.299279469	0.322929971	0.447111301	ARMCX5
ENSG00000198400	9.834651509	3.852265241	1.343400338	0.323006433	0.447193335	NTRK1
ENSG00000271904	2.932408476	0	3.887159215	0.323126074	0.447287471	AC091826.2
ENSG00000188620	2.932408476	0	3.887159215	0.323126074	0.447287471	HMX3
ENSG00000245205	2.932408476	0	3.887159215	0.323126074	0.447287471	EEF1A1P4
ENSG00000280353	3.299970955	1.049547942	1.638233078	0.323195239	0.447359377	AC011466.4
ENSG00000201457	2.421502061	6.469608781	-1.41774785	0.323273518	0.447443891	SNORA55
ENSG00000196950	696.9862412	575.6994444	0.275303893	0.323306214	0.447465307	SLC39A10
ENSG00000116138	371.7842284	442.2853062	-0.250193308	0.323473869	0.447663661	DNAJC16
ENSG00000224511	2.442956632	0.336469917	2.659549711	0.323483991	0.447663661	LINC00365
ENSG00000198000	708.2770826	821.8131277	-0.214751187	0.32360039	0.447800891	NOL8
ENSG00000132972	2.067800989	0	3.38382635	0.323804324	1	RNF17
ENSG00000283886	2.067800989	0	3.38382635	0.323804324	1	BX664615.2
ENSG00000278413	2.067800989	0	3.38382635	0.323804324	1	RF02271
ENSG00000223390	2.067800989	0	3.38382635	0.323804324	1	AL445685.1
ENSG00000282121	2.067800989	0	3.38382635	0.323804324	1	AL592430.2
ENSG00000129535	2.067800989	0	3.38382635	0.323804324	1	NRL
ENSG00000163602	7.270845699	3.953766596	0.879384329	0.323828016	0.448070523	RYBP
ENSG00000170579	13.34233011	6.196942172	1.094830078	0.32382973	0.448070523	DLGAP1
ENSG00000239291	0.601596751	2.587817834	-2.094817336	0.323905547	0.448151562	AC002558.1
novel.232	2.06654734	0	3.383004577	0.323979071	1	-
ENSG00000249851	2.06654734	0	3.383004577	0.323979071	1	RPS27AP18
ENSG00000254330	2.06654734	0	3.383004577	0.323979071	1	AC104232.2
ENSG00000223650	2.06654734	0	3.383004577	0.323979071	1	UHRF2P1
ENSG00000280232	2.06654734	0	3.383004577	0.323979071	1	AL589666.2
ENSG00000229206	2.06654734	0	3.383004577	0.323979071	1	AL162408.1
novel.320	2.06654734	0	3.383004577	0.323979071	1	-
ENSG00000188107	12.98103588	21.40847583	-0.715533661	0.324012665	0.448275896	EYS
novel.1100	9.058013599	16.0676639	-0.822860836	0.324041355	0.448291716	-
ENSG00000256128	3.374292587	7.295956522	-1.11365649	0.324321044	0.448654759	LINC00944
ENSG00000235437	380.7860406	332.7640125	0.194719829	0.324374279	0.448704512	LINC01278
ENSG00000233246	0.879722543	3.230076085	-1.860505717	0.324658613	0.449073919	AL513327.2
ENSG00000272195	7.794359872	3.953766596	0.982338651	0.325089306	0.449645723	AL356512.1
ENSG00000032742	348.5909924	265.9527406	0.389718755	0.325246193	0.449838773	IFT88
ENSG00000113732	2001.212917	2462.006223	-0.299067315	0.325336775	0.449940102	ATP6V0E1
ENSG00000243004	0.308355904	2.069570178	-2.675105801	0.325419453	1	AC005062.1
ENSG00000227398	41.99378671	56.8179179	-0.43778028	0.325627411	0.45031808	KIF9-AS1
ENSG00000110852	466.3166872	313.4805969	0.573325263	0.325683033	0.450361786	CLEC2B
ENSG00000107731	605.2797093	365.1404987	0.72871703	0.325693684	0.450361786	UNC5B
ENSG00000160633	2029.811386	2307.091085	-0.184763658	0.325795955	0.45045886	SAFB

ENSG00000251602	32.59245724	46.11827989	-0.504128195	0.325798562	0.45045886	AL928654.1
ENSG00000159753	62.74955101	83.77277692	-0.418171467	0.325851527	0.450473417	CARMIL2
ENSG00000286989	12.15292675	7.094109687	0.770573454	0.325859639	0.450473417	AL691432.4
ENSG00000261669	5.759296292	2.802717299	1.023576218	0.325878986	0.450473417	AC008731.1
ENSG00000137834	289.6838141	446.6140024	-0.625056711	0.325882793	0.450473417	SMAD6
ENSG00000158062	411.3411838	337.4974517	0.285034353	0.325895784	0.450473417	UBXN11
ENSG00000211455	784.6749115	905.0608029	-0.206258517	0.325918392	0.450480701	STK38L
ENSG00000014919	411.8483575	484.0642815	-0.233577542	0.32596058	0.450515046	COX15
ENSG00000233170	26.13557453	16.15500393	0.695451995	0.32609694	0.450679535	AC138356.2
ENSG00000033122	62.28037171	38.73973523	0.682599039	0.326224358	0.450794737	LRRC7
ENSG00000178631	8.466517309	4.677457107	0.857754328	0.326228082	0.450794737	ACTG1P1
ENSG00000103091	798.3163831	651.3441255	0.293976967	0.326232348	0.450794737	WDR59
ENSG00000277425	5.763057239	2.435565801	1.226014248	0.326256676	0.450804377	AL121890.4
ENSG00000260493	5.601806216	1.456837632	1.949995396	0.326311372	0.450855975	AC011773.4
ENSG00000205913	29.14606558	41.79620592	-0.520895533	0.326571208	0.451173992	SRRM2-AS1
ENSG00000285979	42.64560104	58.62641425	-0.457261317	0.326576271	0.451173992	AC009090.6
ENSG00000232901	3.052075276	6.45069556	-1.083811182	0.326641984	0.451240782	CYCSP10
ENSG00000162396	74.22265623	90.61899381	-0.288686127	0.326760856	0.451380998	PARS2
ENSG00000261170	10.94471865	17.80076416	-0.700177928	0.326916382	0.451571829	AC009053.3
ENSG00000174485	418.5373363	517.2437033	-0.30517448	0.326942232	0.451583526	DENND4A
ENSG00000237356	11.26059644	5.837963015	0.952462103	0.327123572	0.45180998	AL365295.1
ENSG00000107290	1625.178726	1440.884031	0.173592665	0.327166537	0.451845301	SETX
novel.787	4.792644359	2.10970837	1.174393068	0.327194146	0.451859412	-
ENSG00000259425	3.378124804	0.356539013	3.127316964	0.327361811	0.45206693	AC011767.1
ENSG00000251000	7.371636497	3.139187216	1.217405576	0.327399502	0.452094948	AC008592.3
ENSG00000163263	3.895299463	1.049547942	1.880495167	0.327623152	0.452374336	C1orf189
ENSG00000214827	22.03493252	36.45410971	-0.725425719	0.327636653	0.452374336	MTCP1
ENSG00000184634	844.432409	698.6270549	0.273819698	0.327935537	0.45276295	MED12
ENSG00000277203	157.7237833	122.2261038	0.36921315	0.328111111	0.452981283	F8A1
ENSG00000251413	0	1.813376645	-3.438919601	0.328135513	1	AC068898.2
ENSG00000248235	0	1.813376645	-3.438919601	0.328135513	1	AC037459.1
ENSG00000283176	0	1.813376645	-3.438919601	0.328135513	1	AC103706.2
ENSG00000257568	0	1.813376645	-3.438919601	0.328135513	1	AC079035.1
ENSG00000235813	0	1.813376645	-3.438919601	0.328135513	1	AL672032.1
ENSG00000262172	0	1.813376645	-3.438919601	0.328135513	1	AC116025.2
ENSG00000200403	2.723589721	0.672939833	1.975891764	0.328285595	0.453186118	RNU6-1099P
ENSG00000179846	3.929290521	7.509700113	-0.935234005	0.328294366	0.453186118	NKPD1
ENSG00000235602	11.2593428	6.767096381	0.728901093	0.32839383	0.453299335	POU5F1P3
ENSG00000287261	8.915638578	3.873490212	1.199140632	0.328418081	0.453308727	AL139243.1
ENSG00000131069	649.2649419	738.1748326	-0.184985181	0.328464837	0.453349178	ACSS2
ENSG00000002079	2.68075185	6.460152171	-1.263001982	0.328595146	0.45350494	MYH16
ENSG00000284657	4.778782952	1.844058227	1.390236063	0.328726819	0.453662568	AL031432.5
ENSG00000244607	3.038213869	0.743759607	2.049432532	0.328746773	0.453666007	CCDC13
ENSG00000267011	0.615458159	2.180528143	-1.832142481	0.328853285	0.453788889	AC011498.1
ENSG00000162885	19.74989324	33.28899281	-0.756363132	0.328975377	0.453933255	B3GALNT2
ENSG00000248634	0	1.742556871	-3.385828322	0.328978991	1	AC008588.1
ENSG00000230188	0	1.742556871	-3.385828322	0.328978991	1	AL158156.1
ENSG00000199151	0	1.742556871	-3.385828322	0.328978991	1	MIR337
ENSG00000251303	0	1.742556871	-3.385828322	0.328978991	1	CAB39P1
ENSG00000201710	0	1.742556871	-3.385828322	0.328978991	1	RF00181
ENSG00000002746	11.39029243	24.97887464	-1.136147149	0.329020045	0.453970781	HECW1

ENSG00000280739	56.77835919	43.52802191	0.382129234	0.329059449	0.454001038	EIF1B-AS1
ENSG00000185527	8.846687547	4.12852787	1.081644006	0.329090621	0.454019935	AC139530.1
ENSG00000267336	0.600343103	2.140389951	-1.829871344	0.329305839	0.45429273	EIF4A2P1
ENSG00000169914	310.9311978	372.8224134	-0.261668928	0.329421434	0.45442807	OTUD3
ENSG00000254862	5.172814597	2.496928964	1.045073576	0.329474928	0.454477734	AC100771.2
ENSG00000197114	149.0122061	180.4679943	-0.275207516	0.329576612	0.454593861	ZGPAT
ENSG00000272914	8.504269313	4.73882027	0.851267493	0.329611658	0.454618065	AL359532.1
ENSG00000198682	1581.56076	1902.430458	-0.266650977	0.329660244	0.454660941	PAPSS2
ENSG00000279692	20.16488091	11.53672663	0.799277927	0.329709959	0.454705369	AC110285.6
ENSG00000138029	2252.470691	1972.403273	0.191606846	0.329748966	0.454735026	HADHB
ENSG00000138823	1.833766717	0.336469917	2.24457952	0.329752685	1	MTTP
ENSG00000225489	2.697120555	0.713078025	1.91610949	0.329787902	0.454764582	AL354707.1
ENSG00000162493	1080.458266	617.657647	0.806996873	0.329806169	0.454765634	PDPN
ENSG00000116661	227.8322799	130.4040733	0.806157979	0.329902642	0.454874518	FBXO2
ENSG00000278703	0.293240848	2.038888596	-2.656290075	0.330212831	1	AC100847.1
ENSG00000260051	9.230333997	19.32944905	-1.060293311	0.330250831	0.455328414	AL031600.1
novel.465	10.93433345	4.53581756	1.264468306	0.330266886	0.455328414	-
ENSG00000267052	0.879722543	3.576002612	-2.010412434	0.330358118	0.455430026	AC005050.1
ENSG00000170909	10.92451773	5.308974479	1.058591761	0.330413711	0.455477714	OSCAR
ENSG00000253582	8.218621629	4.116631115	1.014818633	0.330427773	0.455477714	AC090579.1
ENSG00000284977	24.90111073	12.13769081	1.031011598	0.330494328	0.455545288	AL160272.1
ENSG00000169682	11.10310637	18.87269295	-0.760905698	0.330678635	0.455775152	SPNS1
ENSG00000148153	422.0019635	354.5277899	0.250541182	0.330709026	0.45579286	INIP
ENSG00000167080	0.601596751	2.129777466	-1.823456816	0.330961917	0.456117206	B4GALNT2
ENSG00000259562	24.72230862	17.05114396	0.533034938	0.331020488	0.456173729	AC090607.2
ENSG00000104808	4.807759415	2.211209725	1.139444944	0.331060308	0.456204405	DHDH
ENSG00000005882	352.5009641	287.4971585	0.295014901	0.331085722	0.456215229	PDK2
ENSG00000229251	0.921306765	3.189937893	-1.801776686	0.331232158	0.456392803	HNRNPA1P8
ENSG00000230042	7.57167971	3.903015919	0.949767354	0.331417592	0.456624089	AK3P3
ENSG00000276505	3.021845164	6.502602113	-1.102723346	0.331569839	0.456809629	AP000892.2
ENSG00000273472	18.01928208	11.24026651	0.682769496	0.33159174	0.456815579	AC096733.2
ENSG00000214367	365.9022594	307.1489158	0.2528971	0.331609867	0.456816329	HAUS3
ENSG00000285569	2.127007565	5.13665335	-1.267416327	0.331692902	0.456884909	AC099344.3
ENSG00000230537	11.68582712	6.522671209	0.847633222	0.331694821	0.456884909	AL158071.3
ENSG00000228444	8.732035341	4.566499142	0.926674979	0.331731523	0.456911239	AL137244.1
ENSG00000264853	2.405133356	0.693008929	1.770557684	0.331765328	0.456919064	AC011933.2
ENSG00000108001	408.9690685	206.1314613	0.987971297	0.331772378	0.456919064	EBF3
ENSG00000173486	18.21674672	11.01591043	0.732694275	0.331926431	0.457088024	FKBP2
ENSG00000115825	1030.63336	1272.083076	-0.303502075	0.331930247	0.457088024	PRKD3
ENSG00000189129	549.2081241	284.6518878	0.947748098	0.331962075	0.457107625	PLAC9
ENSG00000083812	175.3342472	210.6629468	-0.26509673	0.332027294	0.457146362	ZNF324
ENSG00000245317	2.402626059	0.693008929	1.769372176	0.33205852	0.457146362	AC008393.1
ENSG00000273559	2.402626059	0.693008929	1.769372176	0.33205852	0.457146362	CWC25
ENSG00000259453	2.361041837	0.356539013	2.614540868	0.332113375	0.457146362	AC087286.4
ENSG00000268615	2.361041837	0.356539013	2.614540868	0.332113375	0.457146362	AL353803.5
ENSG00000278797	2.361041837	0.356539013	2.614540868	0.332113375	0.457146362	AC011551.1
ENSG00000183638	2.361041837	0.356539013	2.614540868	0.332113375	0.457146362	RP1L1
ENSG00000182010	21.26004646	35.30794071	-0.736271836	0.332173016	0.457204235	RTKN2
ENSG00000170289	3.310071416	1.10029862	1.603204448	0.332247769	0.4572757	CNGB3
ENSG00000183273	2.359788188	0.356539013	2.613845744	0.332260139	0.4572757	CCDC60
ENSG00000226472	3.604565913	1.069617038	1.74956742	0.332296297	0.457301239	AC008013.1

ENSG00000225706	1.229662669	4.434316205	-1.86348764	0.332348515	0.457348875	PTPRD-AS1
ENSG00000251689	0	1.722487775	-3.370385607	0.332420817	1	AC079140.5
ENSG00000257456	0	1.722487775	-3.370385607	0.332420817	1	AC009320.1
ENSG00000233370	0	1.722487775	-3.370385607	0.332420817	1	AC140479.3
ENSG00000229172	0	1.722487775	-3.370385607	0.332420817	1	AC073065.1
ENSG00000273760	0	1.722487775	-3.370385607	0.332420817	1	AC245041.1
ENSG00000283240	0	1.722487775	-3.370385607	0.332420817	1	AC007529.2
ENSG00000201579	5.058162392	2.10970837	1.254780992	0.332456989	0.45747372	RNU6-343P
ENSG00000263276	5.073277448	2.191140629	1.224726779	0.332474454	0.45747372	AC020978.7
ENSG00000071051	877.0847678	737.8073303	0.249815323	0.332652632	0.457694648	NCK2
ENSG00000261159	11.41278719	6.369263301	0.837162506	0.332694969	0.457728659	AC112484.3
ENSG00000042781	6.012206566	2.934900236	1.049095252	0.332733368	0.457757248	USH2A
ENSG00000259138	3.330201067	1.10029862	1.610334998	0.332802388	0.457827959	AL049780.2
ENSG00000068394	499.0433731	592.8526851	-0.248647033	0.332820422	0.457828319	GPKOW
ENSG00000166343	84.74651806	65.53271004	0.373349651	0.332837893	0.457828319	MSS51
ENSG00000283138	0	2.355289417	-3.821149817	0.333005386	1	AC006207.1
ENSG00000108452	0	2.355289417	-3.821149817	0.333005386	1	ZNF29P
ENSG00000135736	369.9064964	474.2136504	-0.358158066	0.333050278	0.458090333	CCDC102A
ENSG00000204388	8.628950709	2.406040094	1.831374386	0.333063638	0.458090333	HSPA1B
ENSG00000184384	577.5019942	679.210955	-0.234062312	0.333125513	0.45815118	MAML2
ENSG00000020129	854.7553095	1108.32809	-0.374956708	0.333175178	0.458195231	NCDN
ENSG00000240857	323.5400401	373.5155508	-0.207678873	0.333209156	0.458217704	RDH14
ENSG00000280157	13.55240252	7.256974206	0.905818996	0.33325088	0.458250826	AL359510.2
ENSG00000177485	406.1533533	492.3934628	-0.27722086	0.333516916	0.458592379	ZBTB33
ENSG00000237372	2.775203133	0	3.805461913	0.333595359	0.458606725	AL606807.1
ENSG00000214039	2.775203133	0	3.805461913	0.333595359	0.458606725	LINC02418
ENSG00000287373	2.775203133	0	3.805461913	0.333595359	0.458606725	AL122014.1
ENSG00000141858	652.8752779	755.148911	-0.209730118	0.333597956	0.458606725	SAMD1
ENSG00000157693	214.560052	157.4815428	0.445120331	0.33373832	0.458775412	TMEM268
ENSG00000264937	3.297463658	6.613560079	-0.991322178	0.33391026	0.458987486	AC100830.2
ENSG00000261582	2.067800989	5.533330555	-1.405100977	0.333943791	0.459009291	AL121753.1
ENSG00000232860	21.23712478	12.9816675	0.713305804	0.333983784	0.459039978	SMG7-AS1
ENSG00000149600	448.6455812	379.9094255	0.239607352	0.334045139	0.459100018	COMMD7
ENSG00000083123	413.4467375	339.7504219	0.282738454	0.334079456	0.459122895	BCKDHB
ENSG00000171295	172.1924071	144.7133149	0.249632783	0.334154644	0.459201936	ZNF440
ENSG00000107404	2198.185526	2581.849062	-0.23202009	0.334191946	0.459228907	DVL1
ENSG00000251126	2.763920295	0	3.799771558	0.33433272	0.459398053	AC017007.5
ENSG00000249779	1.788421549	0.387220594	2.214348457	0.334347126	1	AC106800.2
ENSG00000137814	373.9246697	439.0534791	-0.231722516	0.334463853	0.459553935	HAUS2
ENSG00000243304	6.302940116	3.210006989	0.972409626	0.334499583	0.459578724	AC008494.1
ENSG00000185112	466.1651775	792.7612964	-0.766111878	0.334581446	0.45966689	FAM43A
ENSG00000267475	16.38054398	9.733962468	0.739114592	0.334669615	0.459763709	AC008736.2
ENSG00000148672	1819.490229	2126.032562	-0.224794058	0.334697102	0.459777158	GLUD1
ENSG00000112541	56.94526555	89.97382869	-0.661973924	0.335000865	0.460170111	PDE10A
ENSG00000263766	13.08684127	8.305366273	0.657453439	0.335042575	0.460203073	AC025682.1
ENSG00000167987	428.7063584	492.5574488	-0.199828612	0.335152717	0.460330024	VPS37C
ENSG00000271749	0.879722543	4.106018629	-2.206967491	0.335173847	0.460334711	AC005828.6
ENSG00000065526	1141.904271	1399.867237	-0.293637474	0.335449891	0.460689482	SPEN
ENSG00000144057	0.921306765	4.158053576	-2.183160048	0.335521176	0.460763025	ST6GAL2
ENSG00000102271	40.92461962	20.87272762	0.969188545	0.335608741	0.460858917	KLHL4
ENSG00000223442	10.18563182	5.198144908	0.95880446	0.335702155	0.460944814	TH2LCRR

ENSG00000054392	5.175321895	10.44588462	-1.020919689	0.335706777	0.460944814	HHAT
ENSG00000232398	1.788421549	0.356539013	2.214322186	0.335730587	1	TMPRSS11CP
ENSG00000150783	1.788421549	0.356539013	2.214322186	0.335730587	1	TEX12
ENSG00000167612	2.449224876	5.492036488	-1.164473196	0.335780551	0.461021746	ANKRD33
ENSG00000202343	7.024203668	3.546476906	0.977066017	0.335806924	0.461033591	RF00410
ENSG00000234185	0.894837599	2.842855491	-1.662228368	0.336024713	0.461286344	AC068533.3
ENSG00000284874	13.2565831	7.988965452	0.736792503	0.336026533	0.461286344	AC000093.1
ENSG00000254274	0.293240848	2.852312101	-3.132255861	0.336289672	0.461623182	TDGF1P5
ENSG00000251079	1.541779519	5.298490388	-1.788966517	0.336323315	0.461644972	BMS1P2
ENSG00000237082	6.062637601	2.934900236	1.05836598	0.336404752	0.461712047	COX5BP6
ENSG00000287808	23.43469303	14.70659542	0.666143075	0.336407723	0.461712047	AL355999.1
ENSG00000187555	1842.643495	2077.248907	-0.172801416	0.336429073	0.461716959	USP7
ENSG00000227193	0	1.762625967	-3.401008363	0.336433782	1	AC244453.1
ENSG00000237001	0	1.762625967	-3.401008363	0.336433782	1	WASF3-AS1
ENSG00000227227	0	1.762625967	-3.401008363	0.336433782	1	AC017101.1
ENSG00000223039	0	1.762625967	-3.401008363	0.336433782	1	RN7SKP268
ENSG00000162923	1975.230722	2348.274609	-0.249442452	0.336553259	0.461862995	WDR26
ENSG00000105523	0.922560414	2.822786395	-1.62823606	0.336706407	0.46204876	FAM83E
ENSG00000230847	0	2.323323566	-3.792455617	0.336747267	1	OCLNP1
ENSG00000287622	0	2.323323566	-3.792455617	0.336747267	1	AC068787.5
ENSG00000243016	0	2.323323566	-3.792455617	0.336747267	1	AC112482.1
ENSG00000144369	103.7904325	168.6439259	-0.700978863	0.336793042	0.462143236	FAM171B
ENSG00000250658	2.753819833	0.713078025	1.942003789	0.336958068	0.462345264	AC097652.1
ENSG00000281469	4.810266713	1.813376645	1.413245729	0.336988292	0.462362316	AC019226.1
ENSG00000280291	4.150717034	1.130980201	1.899747816	0.337134646	0.462538693	AC016251.2
ENSG00000173992	824.374713	712.2215153	0.211147635	0.337265261	0.462693461	CCS
ENSG00000137507	555.3648281	891.4877472	-0.682968892	0.337307941	0.462705999	LRRC32
ENSG00000169714	5820.474174	5143.347264	0.178386473	0.33731002	0.462705999	CNBP
ENSG00000231443	9.080721818	4.485066883	1.001677816	0.337407685	0.462815536	AC124944.1
ENSG00000218052	12.18197448	19.6885566	-0.69481656	0.337507238	0.462910355	ADAMTS7P4
ENSG00000224658	0.586481695	3.005720009	-2.336663617	0.337512446	0.462910355	AL513164.1
ENSG00000138061	4736.914703	2541.532881	0.898224188	0.33756557	0.462958777	CYP1B1
ENSG00000101104	498.908836	637.8067812	-0.354447931	0.337696608	0.463114044	PABPC1L
ENSG00000243759	4.911057511	9.723221588	-0.988978137	0.338186876	0.463761915	ST13P15
ENSG00000102984	47.25360197	35.98160405	0.3972436	0.338368359	0.463986296	ZNF821
ENSG00000273002	16.6183392	23.92906991	-0.521979456	0.338463779	0.464092645	AL355388.2
ENSG00000235919	24.23278551	32.78233794	-0.435005338	0.338550334	0.464161461	ASH1L-AS1
ENSG00000257896	2.699627853	6.074087452	-1.167174569	0.338558273	0.464161461	AC093012.1
ENSG00000091073	115.5765522	154.3115456	-0.418265227	0.338567562	0.464161461	DTX2
ENSG00000126106	159.9317802	131.6370341	0.280967146	0.338656621	0.464259058	TMEM53
ENSG00000116745	3.686480708	0.672939833	2.424420158	0.338814837	0.464451447	RPE65
ENSG00000132002	2291.450077	1858.65034	0.30194192	0.338843786	0.464466624	DNAJB1
novel.546	349.9139243	236.0068496	0.567421632	0.338895994	0.464498872	-
novel.1107	4.269130186	8.194408307	-0.94562788	0.338918928	0.464498872	-
ENSG00000186160	3.685155788	0.672939833	2.423918575	0.338920947	0.464498872	CYP4Z1
ENSG00000224549	3.225649323	0.387220594	3.063496021	0.338984257	0.464548215	AC017067.1
ENSG00000287966	4.5888402	1.426156051	1.680641365	0.33899271	0.464548215	AL590062.1
ENSG00000144061	154.1660735	127.9879	0.268770546	0.339028337	0.464572533	NPHP1
ENSG00000155256	868.166737	733.3419945	0.243929239	0.33915813	0.464725877	ZFYVE27
ENSG00000119725	27.41058237	19.26004194	0.507872581	0.339305058	0.464883665	ZNF410
ENSG00000188032	0.600343103	2.679862578	-2.141602321	0.339309069	0.464883665	C19orf67

ENSG00000008324	230.4487515	276.9217063	-0.26575012	0.33935565	0.464922967	SS18L2
ENSG00000136888	1273.804779	1540.297781	-0.27417036	0.339426471	0.464995473	ATP6V1G1
ENSG00000274297	4.576232441	1.426156051	1.677051518	0.339469003	0.465029219	AC009269.5
ENSG00000146587	425.3075623	368.5354218	0.207173806	0.339546135	0.465110357	RBAK
ENSG00000084733	2737.784728	3048.170157	-0.154935721	0.339568863	0.465114875	RAB10
ENSG00000189403	7116.945851	8302.442016	-0.222316032	0.33959896	0.465114875	HMGB1
novel.942	27.63555636	104.8743429	-1.923534504	0.339620839	0.465114875	-
ENSG00000280392	1.203193503	3.555933516	-1.562908654	0.339621042	0.465114875	AC007496.3
ENSG00000260290	2.43410982	5.02453951	-1.048244696	0.339803207	0.465339823	AC092115.1
ENSG00000173588	295.6338245	238.5978995	0.30926018	0.340020967	0.46561349	CEP83
ENSG00000267179	2.991615051	1.080229524	1.475652318	0.340116265	0.465703322	AC008770.2
ENSG00000167671	2222.068606	2614.514312	-0.234550085	0.340122418	0.465703322	UBXN6
novel.395	17.68202098	25.83346382	-0.549148303	0.340176157	0.465752357	-
ENSG00000225169	3.576843098	1.00940975	1.794446256	0.340225125	0.465794855	BRI3P1
ENSG00000143774	4317.519909	5005.745693	-0.213419603	0.340283719	0.465850527	GUK1
ENSG00000187097	337.6197608	411.269203	-0.285258912	0.340310912	0.465863207	ENTPD5
ENSG00000272109	2.989107754	1.080229524	1.474721879	0.340374597	0.465918208	AC008906.2
ENSG00000280385	8.093868963	4.340987191	0.905389617	0.340386956	0.465918208	AP000648.3
ENSG00000235100	6.051283491	2.475703993	1.282342717	0.340521143	0.466077327	AL157400.3
ENSG00000217555	12.97595001	8.203864917	0.659349675	0.340628522	0.466199739	CKLF
ENSG00000130699	47.47252119	61.51770867	-0.373152926	0.340671616	0.466234159	TAF4
ENSG00000073711	223.751211	296.8762382	-0.40741677	0.340794028	0.466377121	PPP2R3A
ENSG00000068781	2.153476731	0.387220594	2.476270874	0.340904455	1	STON1-GTF2A1L
ENSG00000251537	2.153476731	0.387220594	2.476270874	0.340904455	1	AC005324.3
ENSG00000151413	204.2681604	250.4393529	-0.294742521	0.340925988	0.466533135	NUBPL
ENSG00000225518	4.598940661	9.111773312	-0.996195083	0.340962146	0.466558041	LINC01703
ENSG00000213563	590.022757	492.2937249	0.261362787	0.340994729	0.466578052	C8orf82
novel.689	0.615458159	3.484985349	-2.499845692	0.341032362	0.466604972	-
ENSG00000243071	8.193406112	4.697526203	0.805501174	0.341073186	0.466636252	AC107032.1
ENSG00000101247	213.2193997	255.5300415	-0.261847873	0.341127747	0.466686325	NDUFAF5
ENSG00000270823	3.072276198	0.693008929	2.127261832	0.341222399	0.466791236	AC007938.2
ENSG00000255306	21.8840667	13.93074157	0.660032064	0.341444787	0.467068437	AC004923.4
ENSG00000223113	0	1.733100261	-3.378132116	0.341451561	1	RNA5SP247
ENSG00000229228	0	1.733100261	-3.378132116	0.341451561	1	LINC00582
ENSG00000251320	0	1.733100261	-3.378132116	0.341451561	1	AC011352.3
ENSG00000287974	0	1.733100261	-3.378132116	0.341451561	1	Z84486.1
ENSG00000127329	308.426341	192.7411726	0.677459466	0.341460987	0.467068437	PTPRB
ENSG00000228686	4.202401718	1.436768536	1.55016161	0.341497862	0.46708248	AL590723.1
ENSG00000172113	353.8356057	415.3803119	-0.231894684	0.341507208	0.46708248	NME6
ENSG00000185722	1233.685963	1411.824938	-0.194616105	0.341538949	0.467101302	ANKFY1
ENSG00000286205	0.879722543	3.159256312	-1.833723931	0.341694796	0.467289846	AC139887.5
ENSG00000140379	53.19288275	103.4906368	-0.959363639	0.341745804	0.467335004	BCL2A1
ENSG00000277855	3.269740843	1.029478846	1.644580066	0.341809022	0.467396852	AC097634.3
ENSG00000154813	683.8913509	806.7179458	-0.238695168	0.341874989	0.467462454	DPH3
ENSG00000185650	7560.71537	6026.433049	0.32726949	0.341954504	0.467523563	ZFP36L1
ENSG00000081177	624.0921158	550.1984565	0.182033475	0.34195567	0.467523563	EXD2
ENSG00000272256	7.80564271	1.936102971	2.029302254	0.342100082	0.467696391	AC044849.1
ENSG00000233762	347.3829543	439.0386546	-0.338282363	0.342176371	0.467776074	AC007969.1
ENSG00000278467	10.59603217	6.440083075	0.716450313	0.342211813	0.467799911	AC138393.3
ENSG00000141622	36.753346	63.86516407	-0.79546948	0.342255661	0.467835235	RNF165
ENSG00000171055	1640.037058	2165.519272	-0.401115822	0.342289495	0.467852325	FEZ2

ENSG00000166426	14.45748277	6.370419176	1.178181872	0.342304178	0.467852325	CRABP1
ENSG00000260209	2.153476731	0.356539013	2.476287319	0.342331634	1	AP000842.3
ENSG00000271732	2.153476731	0.356539013	2.476287319	0.342331634	1	AL137798.1
ENSG00000267197	1.510295757	3.566546002	-1.241056324	0.342344721	0.467883124	AC011461.1
ENSG00000230715	155.2534758	110.8007203	0.487836431	0.342380561	0.467885413	AC018638.2
ENSG00000249487	4.816606227	1.406086955	1.768130265	0.342382413	0.467885413	LINC01586
ENSG00000197847	10.26880026	6.094156548	0.752899599	0.342407382	0.467894925	SLC22A20P
ENSG00000228221	8.992538779	3.404837359	1.38738494	0.342553385	0.468069817	LINC00578
ENSG00000275155	1.215801261	3.139187216	-1.381718298	0.342629005	0.468148524	AC027348.1
ENSG00000124613	81.80526277	62.31093468	0.394843595	0.342676779	0.468171798	ZNF391
ENSG00000236792	1.525410814	3.627909165	-1.250342324	0.342682078	0.468171798	AL513175.2
ENSG00000068724	776.8038801	895.1246896	-0.204661791	0.342732624	0.468216233	TTC7A
ENSG00000234906	2.639167628	0	3.73530758	0.342760157	1	APOC2
ENSG00000224239	2.639167628	0	3.73530758	0.342760157	1	AC090044.1
ENSG00000211697	2.639167628	0	3.73530758	0.342760157	1	TRGV5
ENSG00000287736	2.639167628	0	3.73530758	0.342760157	1	AF305872.2
ENSG00000263924	2.639167628	0	3.73530758	0.342760157	1	AC022960.1
ENSG00000232382	2.639167628	0	3.73530758	0.342760157	1	OR5K1
ENSG00000275516	2.639167628	0	3.73530758	0.342760157	1	AC100791.3
ENSG00000228764	2.639167628	0	3.73530758	0.342760157	1	ZNF885P
ENSG00000228509	3.020591515	1.049547942	1.507784565	0.3427693	0.468241715	AC006460.1
ENSG00000257446	5.501371423	2.10970837	1.372032131	0.342789069	0.468244101	ZNF878
ENSG00000264370	6.065144898	2.475703993	1.285317435	0.342859982	0.468316344	MIR3125
ENSG00000239920	5.689989255	2.169915658	1.395073071	0.342883553	0.468323919	AC104389.4
ENSG00000287009	1.524157165	3.546476906	-1.227748907	0.34290577	0.468329644	AC021037.1
ENSG00000259540	4.803998469	1.702418679	1.473537734	0.342934094	0.468343708	AC027020.1
ENSG00000255337	14.28125923	8.416324238	0.769063806	0.342976218	0.468376616	AP001830.1
ENSG00000128602	436.8643457	609.6421945	-0.480879321	0.343028183	0.468422959	SMO
ENSG00000259414	3.579350395	0.774441189	2.243670681	0.343071295	0.468446497	AC242376.1
ENSG00000148204	3.299970955	1.130980201	1.576327907	0.343099511	0.468446497	CRB2
ENSG00000182885	3.299970955	1.130980201	1.576327907	0.343099511	0.468446497	ADGRG3
ENSG00000072506	963.71953	1174.795353	-0.286013445	0.343136011	0.468471713	HSD17B10
ENSG00000287849	2.153476731	0.336469917	2.476298126	0.343275838	1	AC079148.2
ENSG00000233163	2.153476731	0.336469917	2.476298126	0.343275838	1	RPS12P17
ENSG00000114098	643.0352558	760.2484739	-0.241751279	0.343366302	0.468761488	ARMC8
ENSG00000279879	4.251507832	1.722487775	1.27804678	0.343399718	0.468777264	AC091152.4
ENSG00000186665	194.9761834	154.237177	0.33684715	0.343413943	0.468777264	C17orf58
ENSG00000176978	2691.032112	2158.962771	0.317956485	0.343536632	0.468920103	DPP7
ENSG00000099337	463.9809199	307.6373119	0.592181184	0.343589641	0.468967821	KCNK6
ENSG00000128191	712.2105502	892.0647044	-0.324681484	0.343642192	0.469014908	DGCR8
ENSG00000242252	6.023560676	2.649180997	1.210175036	0.343710255	0.469077898	BGLAP
ENSG00000231395	4.175932552	1.456837632	1.528870724	0.343724454	0.469077898	ARL4AP4
ENSG00000159904	3.883945353	1.396630344	1.463693055	0.343786643	0.469138126	ZNF890P
ENSG00000100814	620.8386884	750.1094739	-0.272518176	0.343943383	0.469322792	CCNB1IP1
ENSG00000187908	1.493927053	4.096562019	-1.439528661	0.343958096	0.469322792	DMBT1
ENSG00000259868	5.102253911	2.099095884	1.269785297	0.343977465	0.469324574	AL163952.1
ENSG00000048052	717.3869805	510.4319612	0.491434057	0.344006937	0.469340137	HDAC9
ENSG00000271816	5.980722805	11.39136267	-0.920970786	0.344087589	0.469425523	AC022400.5
ENSG00000083828	136.4033482	107.4171079	0.346172992	0.344161306	0.469501438	ZNF586
ENSG00000286729	2.406387005	5.778911602	-1.259759009	0.344242418	0.469579012	AC137695.3
ENSG00000278642	1.539272221	3.944309986	-1.366100443	0.344261697	0.469579012	AC015813.4

ENSG00000119333	995.1615193	1199.050152	-0.269113125	0.344272392	0.469579012	WDR34
ENSG00000183726	2325.595819	2617.271684	-0.170504785	0.344302625	0.469595596	TMEM50A
ENSG00000259762	2.435363469	0.723690511	1.755570455	0.344351457	0.469637544	AC021739.5
ENSG00000223551	5.155192243	2.120320855	1.274168962	0.344466875	0.469770294	TMSB4XP4
ENSG00000054282	94.00812201	63.93847117	0.558681602	0.344528459	0.469829618	SDCCAG8
ENSG00000279894	10.2612071	6.206270388	0.735568484	0.344593432	0.469893558	Z73965.1
ENSG00000200913	2.96013129	7.063428106	-1.250247072	0.344690987	0.470001918	SNORD46
ENSG00000103168	1023.808705	843.050695	0.280186885	0.344763372	0.470065741	TAF1C
ENSG00000262874	3.385646697	0.713078025	2.241378029	0.344782739	0.470065741	C19orf84
ENSG00000226310	5.15895319	2.486316478	1.045433325	0.344806781	0.470065741	AL022157.1
ENSG00000236519	2.743719372	5.706935953	-1.062015409	0.344810165	0.470065741	LINC01424
ENSG00000123552	310.1075338	384.9076366	-0.311729319	0.344878233	0.470119989	USP45
ENSG00000125447	714.0721656	813.5723222	-0.188235143	0.344886147	0.470119989	GGA3
ENSG00000165516	692.014438	592.2593666	0.22487531	0.345048385	0.470316463	KLHDC2
ENSG00000116544	9.93898979	4.414247109	1.154566573	0.345079842	0.470319349	DLGAP3
ENSG00000165084	1.848881773	5.216929734	-1.496196579	0.345109951	0.470319349	C8orf34
ENSG00000228554	2.405133356	0.743759607	1.720596998	0.345115294	0.470319349	AC004837.2
ENSG00000008086	154.7800211	192.1050193	-0.311333309	0.345139214	0.470319349	CDKL5
ENSG00000112245	6.948628387	11.39264694	-0.715405208	0.345141015	0.470319349	PTP4A1
ENSG00000285108	2.127007565	4.881744087	-1.209069299	0.345159314	0.470319617	AC103718.1
ENSG00000259563	2.402626059	0.743759607	1.719415854	0.345415627	0.470644189	AC025430.1
ENSG00000243960	6.986451663	3.2194636	1.11498748	0.345501368	0.470736328	AL390195.1
ENSG00000250746	2.094270155	5.176791542	-1.293244782	0.345572249	0.470787387	AC105250.1
ENSG00000278733	3.031945625	1.049547942	1.512376792	0.345595281	0.470787387	AC022079.2
novel.1005	3.685227059	14.41270384	-1.970674328	0.34560911	0.470787387	-
ENSG00000120008	1186.519974	1351.05099	-0.187265526	0.345611325	0.470787387	WDR11
ENSG00000141503	1912.673242	2390.618238	-0.321653047	0.345641944	0.470804411	MINK1
ENSG00000266783	2.682005499	5.676254372	-1.077908124	0.345705405	0.470856101	AP005136.2
ENSG00000256789	0.616711807	2.506385574	-2.033791117	0.345716138	0.470856101	AP000753.2
ENSG00000148719	1283.394402	1105.866513	0.214523056	0.345756686	0.470886641	DNAJB12
ENSG00000261056	6.594927315	2.812173909	1.22217974	0.345775944	0.470888185	AC079416.1
novel.528	7.726306483	14.43850514	-0.896568613	0.345961441	0.471116106	-
ENSG00000102103	646.951068	754.689214	-0.222408962	0.34599711	0.471139985	PQBP1
ENSG00000197019	699.4795002	550.2620286	0.345702198	0.346167307	0.471347036	SERTAD1
ENSG00000047932	1235.569959	1433.29605	-0.214015244	0.346263446	0.471453233	GOPC
ENSG00000252614	2.113146158	0.387220594	2.451758461	0.346265324	1	RNU6-807P
ENSG00000278763	17.10682213	8.255899865	1.041360476	0.346355351	0.471553654	FAM27B
ENSG00000250635	3.052075276	1.080229524	1.498320793	0.346399727	0.471567062	CXXC5-AS1
ENSG00000167930	2490.632641	2080.943151	0.259420515	0.346401501	0.471567062	FAM234A
ENSG00000164548	1588.717	1372.838189	0.210665993	0.346601953	0.471815223	TRA2A
ENSG00000244045	223.1182011	187.4125961	0.250450705	0.346633937	0.47183404	TMEM199
ENSG00000172171	108.8978161	129.5699041	-0.251187533	0.346698307	0.471896936	TEFM
novel.444	8.850519764	20.77683007	-1.230004509	0.346718882	0.471900218	-
ENSG00000204592	0	1.702418679	-3.354504949	0.346727694	1	HLA-E
ENSG00000285545	0	1.702418679	-3.354504949	0.346727694	1	AC124798.2
ENSG00000288080	0	1.702418679	-3.354504949	0.346727694	1	AC005104.3
ENSG00000275987	0	1.702418679	-3.354504949	0.346727694	1	RF00003
ENSG00000229156	0	1.702418679	-3.354504949	0.346727694	1	CR769776.2
ENSG00000242550	0	1.702418679	-3.354504949	0.346727694	1	SERPINB10
ENSG00000177879	1085.686507	1315.031128	-0.276382528	0.346775378	0.471952389	AP3S1
ENSG00000239040	2.668144092	6.205114513	-1.20586765	0.346829953	0.472001938	RF00019

ENSG00000162929	235.222635	293.7396795	-0.320915664	0.346965638	0.472161861	KIAA1841
ENSG00000258752	2.752566185	0.713078025	1.941687895	0.34701098	0.472198831	AL357093.2
ENSG00000089127	1070.90713	767.227109	0.480842608	0.347066207	0.472249248	AC004551.1
ENSG00000268836	4.83548223	2.079026788	1.198161383	0.347262548	0.472491661	Z69706.1
ENSG00000240877	4.514518568	2.160459047	1.067627467	0.347353346	0.472590452	RN7SL521P
ENSG00000144031	39.49803429	27.23227752	0.53994372	0.347409877	0.472642615	ANKRD53
ENSG00000141748	1.846374476	0	3.216940164	0.347413624	1	ARL5C
ENSG00000163421	1.846374476	0	3.216940164	0.347413624	1	PROK2
ENSG00000285883	1.846374476	0	3.216940164	0.347413624	1	AC123784.1
ENSG00000182035	1.846374476	0	3.216940164	0.347413624	1	ADIG
ENSG00000280037	1.846374476	0	3.216940164	0.347413624	1	AC092653.2
ENSG00000279928	1.846374476	0	3.216940164	0.347413624	1	FO538757.1
ENSG00000253116	1.846374476	0	3.216940164	0.347413624	1	AC027698.1
ENSG00000280485	1.846374476	0	3.216940164	0.347413624	1	AL358154.1
ENSG00000265033	1.846374476	0	3.216940164	0.347413624	1	RN7SL262P
ENSG00000196408	1.846374476	0	3.216940164	0.347413624	1	NOXO1
ENSG00000154305	1067.020769	1205.569527	-0.176162684	0.347457982	0.472683309	MIA3
ENSG00000117151	171.6674967	204.0193475	-0.249198282	0.347618556	0.472876994	CTBS
ENSG00000117262	145.8899262	190.1132169	-0.383131596	0.34764437	0.472887349	GPR89A
ENSG00000261693	2.113146158	0.356539013	2.451755311	0.34772756	1	AC134682.1
ENSG00000260926	2.113146158	0.356539013	2.451755311	0.34772756	1	AC016134.1
ENSG00000258955	5.688735607	13.38767293	-1.229379344	0.348127728	0.473520051	LINC00519
ENSG00000248196	2.155984028	0.356539013	2.477867472	0.348130964	1	AC092658.1
ENSG00000196655	2.155984028	0.356539013	2.477867472	0.348130964	1	TRAPPC4
ENSG00000259831	2.155984028	0.356539013	2.477867472	0.348130964	1	LINC00567
ENSG00000210154	15.08157427	10.01839744	0.589946431	0.348209909	0.473607038	MT-TD
novel.876	0.616711807	4.374108917	-2.835546619	0.348250359	0.47363726	-
ENSG00000164512	2.098031101	0.387220594	2.442488558	0.348307533	1	ANKRD55
ENSG00000286854	2.098031101	0.387220594	2.442488558	0.348307533	1	AC079382.2
ENSG00000151414	2081.522521	1539.856683	0.434672865	0.34844261	0.473873924	NEK7
ENSG00000262678	2.961384939	6.836760279	-1.199016595	0.348495226	0.473920674	AC004771.5
ENSG00000224976	2.152223082	0.356539013	2.47560051	0.34862938	1	PARP4P2
ENSG00000172831	573.5566197	679.8979878	-0.245771824	0.348742857	0.474232606	CES2
ENSG00000228677	2.094270155	0.387220594	2.440185587	0.348816947	1	TTC3-AS1
ENSG00000184492	5.792033702	2.802717299	1.031517153	0.348908608	0.474412641	FOXD4L1
ENSG00000182149	2372.492956	2111.605767	0.16814456	0.348911772	0.474412641	IST1
novel.1117	13.067894	7.968896356	0.71655563	0.349005809	0.474515669	-
ENSG00000235726	2.155984028	0.336469917	2.477878437	0.349053367	1	AC010148.1
ENSG00000279162	6.918398275	3.587770973	0.949405404	0.349119181	0.474644973	AC141586.5
ENSG00000287431	57.15483976	73.87034613	-0.372745802	0.349158134	0.474673093	AC027601.6
ENSG00000255933	6.372247153	2.659793482	1.283035783	0.349508278	0.475124245	AC117500.2
ENSG00000263326	7.475903507	3.300895859	1.189093799	0.349550006	0.475156108	AC133552.4
ENSG00000163064	62.1817315	95.8774744	-0.625500973	0.349606497	0.475199605	EN1
ENSG00000128731	420.0985772	356.8718589	0.235447106	0.349633862	0.475199605	HERC2
ENSG00000204642	6.46293749	2.466247382	1.380599214	0.349636875	0.475199605	HLA-F
novel.396	7.929141728	4.230029225	0.895647027	0.349671018	0.47522115	-
ENSG00000161055	3.96328158	1.10029862	1.85698294	0.349731083	0.475258752	SCGB3A1
ENSG00000141446	348.5169399	288.7276663	0.272336752	0.349735271	0.475258752	ESCO1
ENSG00000128791	1568.656576	1993.928335	-0.346210301	0.349776709	0.475290202	TWSG1
ENSG00000236878	2.098031101	0.356539013	2.442477507	0.349783232	1	MTATP6P26
ENSG00000270040	2.098031101	0.356539013	2.442477507	0.349783232	1	AL356055.1

ENSG00000173862	2.098031101	0.356539013	2.442477507	0.349783232	1	AC008080.1
ENSG00000246582	10.67160745	16.1019415	-0.597317424	0.349807807	0.475307601	AC100861.1
ENSG00000187546	13.79306104	34.04837359	-1.306220075	0.35007047	0.475639624	AGMO
ENSG00000163380	7.946764082	3.505182839	1.170267734	0.350103617	0.475659786	LMOD3
ENSG00000234147	5.539123428	1.742556871	1.655589996	0.350136254	0.475663064	AL035446.1
ENSG00000153574	320.2889183	390.8823249	-0.287952136	0.350157875	0.475663064	RPIA
ENSG00000197275	18.46979954	26.80157951	-0.538311657	0.350160955	0.475663064	RAD54B
ENSG00000263961	1.847628125	0	3.217876757	0.350224024	1	RHEX
ENSG00000269736	1.847628125	0	3.217876757	0.350224024	1	AC010319.5
ENSG00000094755	1.847628125	0	3.217876757	0.350224024	1	GABRP
ENSG00000211653	1.847628125	0	3.217876757	0.350224024	1	IGLV1-40
novel.897	1.847628125	0	3.217876757	0.350224024	1	-
ENSG00000265215	1.847628125	0	3.217876757	0.350224024	1	MIR4269
ENSG00000287097	1.847628125	0	3.217876757	0.350224024	1	AL590550.1
ENSG00000258551	1.847628125	0	3.217876757	0.350224024	1	AC104035.1
ENSG00000226004	1.847628125	0	3.217876757	0.350224024	1	AL591468.1
ENSG00000156269	1.847628125	0	3.217876757	0.350224024	1	NAA11
ENSG00000286697	1.847628125	0	3.217876757	0.350224024	1	AC092623.1
ENSG00000227508	1.847628125	0	3.217876757	0.350224024	1	LINC01624
ENSG00000266283	1.847628125	0	3.217876757	0.350224024	1	AC091588.3
ENSG00000177688	8.156907756	4.340987191	0.914202311	0.350260563	0.475773498	SUMO4
ENSG00000272103	7.211639123	3.300895859	1.137212477	0.350283107	0.475779246	AC026741.1
ENSG00000279693	2.094270155	0.356539013	2.440172452	0.350296031	1	AC099521.2
ENSG00000260160	35.29061798	26.39300555	0.422858824	0.350367869	0.475869497	AC011468.1
ENSG00000197782	5.787019107	2.934900236	0.990547209	0.350409192	0.475900744	ZNF780A
ENSG00000174255	2.128261214	0.387220594	2.460998645	0.350426727	1	ZNF80
novel.638	83.21465337	56.87517146	0.55105298	0.3505036	0.476004079	-
ENSG00000286132	10.98504922	17.24237831	-0.652786873	0.350631222	0.476152508	AC022415.2
ENSG00000236941	1.845120827	0	3.21604234	0.350636247	1	AC106872.1
ENSG00000270415	1.845120827	0	3.21604234	0.350636247	1	DPPA3P3
ENSG00000283648	1.845120827	0	3.21604234	0.350636247	1	AC006974.2
ENSG00000230461	1.845120827	0	3.21604234	0.350636247	1	PROX1-AS1
ENSG00000229101	1.845120827	0	3.21604234	0.350636247	1	ELOCP20
ENSG00000286739	1.845120827	0	3.21604234	0.350636247	1	AC007387.2
ENSG00000216813	1.845120827	0	3.21604234	0.350636247	1	AL358178.1
ENSG00000224944	1.845120827	0	3.21604234	0.350636247	1	CASC6
ENSG00000228115	1.845120827	0	3.21604234	0.350636247	1	AL161725.2
ENSG00000270403	1.845120827	0	3.21604234	0.350636247	1	AP001554.1
ENSG00000203900	1.845120827	0	3.21604234	0.350636247	1	AL121827.1
ENSG00000266969	12.66132586	7.287784182	0.784614944	0.350665832	0.476166312	AP002449.1
ENSG00000181741	2.430348874	0.743759607	1.731900034	0.350678042	0.476166312	FDX1P1
ENSG00000182347	2.098031101	0.336469917	2.442470243	0.350759664	1	PDSS1P1
ENSG00000249014	3.634796025	6.961926751	-0.947623757	0.350845739	0.476369121	HMGN2P4
ENSG00000181222	40.27997153	58.0031164	-0.52810944	0.350886663	0.47639979	POLR2A
ENSG00000103423	76.50644151	118.4590424	-0.630521359	0.3509274	0.476425018	DNAJA3
ENSG00000264964	5.443347225	10.19071857	-0.900925065	0.350953283	0.476425018	AP001033.1
ENSG00000227212	5.353910537	11.94157617	-1.152674931	0.350960256	0.476425018	PFN1P6
ENSG00000102316	4199.851302	4993.638979	-0.249754219	0.350990351	0.476440977	MAGED2
ENSG00000157601	1967.582068	2626.756327	-0.416973928	0.351068397	0.476522022	MX1
ENSG00000287637	0.293240848	1.874739808	-2.518141931	0.351075941	1	AP001437.2
ENSG00000259577	4.311968057	9.537847829	-1.146404228	0.351193185	0.4766665	CERNA1

ENSG00000287663	0.615458159	2.455634897	-2.01077451	0.351213193	0.476668754	AL122020.2
ENSG00000006468	1047.584753	741.5401956	0.498569803	0.35123282	0.476670492	ETV1
ENSG00000254272	2.094270155	0.336469917	2.440163819	0.351274712	1	AC087854.2
ENSG00000119514	27.78595147	44.79041436	-0.692150269	0.351436578	0.476922107	GALNT12
ENSG00000115464	1014.063829	1204.223317	-0.247836296	0.351463304	0.476933464	USP34
ENSG00000065559	473.8416137	546.3909185	-0.206091908	0.351501606	0.476960528	MAP2K4
ENSG00000286608	6.525620278	2.853467977	1.194568037	0.35154378	0.476992843	AC010201.3
ENSG00000261740	1.201939854	3.485113743	-1.540038325	0.351618626	0.477050871	BOLA2-SMG1P6
ENSG00000237357	11.91645645	6.461308046	0.88154924	0.35162327	0.477050871	BX088651.4
ENSG00000216895	21.30170195	30.40227472	-0.515303283	0.351679413	0.477102126	AC009403.1
ENSG00000251226	0.308355904	2.691759334	-3.053339927	0.35175948	0.477160918	AP001999.1
ENSG00000262319	0.308355904	2.691759334	-3.053339927	0.35175948	0.477160918	AC007952.6
ENSG00000233661	2.128261214	0.356539013	2.461002854	0.351843768	1	SPIN4-AS1
ENSG00000108826	790.971655	923.6711034	-0.224112917	0.351987786	0.477445686	MRPL27
ENSG00000148498	557.7082496	733.074837	-0.394444993	0.352028276	0.47747568	PARD3
ENSG00000143199	5.746688534	2.92428775	0.985439839	0.352203705	0.477688685	ADCY10
novel.406	3.699017196	1.080229524	1.774604889	0.35222298	0.47768989	-
ENSG00000196361	2.714742909	0.693008929	1.949273642	0.352273723	0.477733771	ELAVL3
ENSG00000125734	1075.621512	904.1005434	0.25097583	0.352344381	0.477804654	GPR108
ENSG00000145283	3.388153995	0.713078025	2.242534307	0.352379589	0.477827458	SLC10A6
ENSG00000216639	0.586481695	3.048298346	-2.371771931	0.35266056	0.47816262	AL133406.1
ENSG00000232118	7.750268352	2.832243005	1.449281759	0.35268812	0.47816262	BACH1-AS1
ENSG00000279145	10.82755915	6.512058724	0.739396714	0.352688649	0.47816262	AC011912.1
ENSG00000271819	2.12199297	0.356539013	2.457186131	0.352688951	1	RNU6-94P
ENSG00000159110	50.62377761	37.37365808	0.436548649	0.352700375	0.47816262	IFNAR2
ENSG00000122863	1725.911788	1264.529811	0.448574519	0.352770922	0.478233306	CHST3
ENSG00000260278	2.128261214	0.336469917	2.46100562	0.35278134	1	AC098818.2
ENSG00000254233	2.128261214	0.336469917	2.46100562	0.35278134	1	LINC02365
ENSG00000239265	2.128261214	0.336469917	2.46100562	0.35278134	1	CLRN1-AS1
ENSG00000229018	3.375546236	7.225136749	-1.101263962	0.353013357	0.478516719	PMS2P7
ENSG00000287737	37.93111053	59.40620245	-0.644092268	0.353016818	0.478516719	AC132872.5
ENSG00000105705	625.531068	714.7025188	-0.192010814	0.353035418	0.478516966	AC004475.1
ENSG00000169495	3.575589449	1.00940975	1.795335378	0.353070145	0.47853907	HTRA4
ENSG00000181026	1057.926456	1269.994399	-0.263766581	0.353143733	0.478613841	AEN
ENSG00000278616	10.91323489	17.99431026	-0.717142441	0.353187133	0.478647692	BEND3P3
ENSG00000100225	2095.103649	1779.953774	0.235248074	0.353257248	0.478717742	FBXO7
ENSG00000171303	5.797048297	2.171071533	1.41940244	0.353458911	0.478947637	KCNK3
ENSG00000072042	1301.753809	1473.966892	-0.179112022	0.353463762	0.478947637	RDH11
ENSG00000274447	1.804790254	5.503933243	-1.612749224	0.353512402	0.478988563	AC005790.1
ENSG00000227057	2.12199297	0.336469917	2.457186884	0.353630011	1	WDR46
novel.1094	2.946269883	0.693008929	2.072903498	0.353681349	0.479192486	-
ENSG00000228589	162.1842524	118.2566004	0.454355403	0.353726106	0.479228135	SPCS2P4
ENSG00000273474	9.843284858	3.362259022	1.55924714	0.353748291	0.479231971	AL157392.4
novel.633	19.02981207	28.65599343	-0.585458598	0.353765828	0.479231971	-
ENSG00000279319	2.699627853	0.693008929	1.942261618	0.353943909	0.479448211	AC105074.1
ENSG00000269815	6.640272484	3.647978261	0.874809519	0.354109699	0.479647782	AC010463.3
ENSG00000260955	1.816144364	4.22887335	-1.225548007	0.354208435	0.479750536	AC100821.2
ENSG00000111707	894.3380833	772.6232098	0.211351864	0.354238149	0.479750536	SUDS3
ENSG00000198795	132.20133	232.6213322	-0.815476584	0.354240956	0.479750536	ZNF521
ENSG00000257818	1.804790254	0	3.186141464	0.354358364	1	C1GALT1P1
ENSG00000267535	1.804790254	0	3.186141464	0.354358364	1	LINC00868

ENSG00000224280	1.804790254	0	3.186141464	0.354358364	1	AC005014.1
ENSG00000240015	1.804790254	0	3.186141464	0.354358364	1	AC084024.1
ENSG00000226661	1.804790254	0	3.186141464	0.354358364	1	AC004973.1
ENSG00000202412	1.804790254	0	3.186141464	0.354358364	1	RNY3P13
ENSG00000156920	1.804790254	0	3.186141464	0.354358364	1	ADGRG4
ENSG00000287105	1.804790254	0	3.186141464	0.354358364	1	AC090577.1
ENSG00000254361	1.804790254	0	3.186141464	0.354358364	1	AC046195.2
ENSG00000132010	7.838451391	3.718798035	1.090031383	0.354372973	0.479893502	ZNF20
ENSG00000164744	3.887706299	8.510809128	-1.128796711	0.354383462	0.479893502	SUN3
ENSG00000186854	118.7869725	71.56264373	0.729223655	0.354463177	0.479976432	TRABD2A
ENSG00000104756	1350.898079	1106.045255	0.28831234	0.354542394	0.48005868	KCTD9
ENSG00000140941	1953.26917	2211.846772	-0.179306226	0.35457856	0.480082628	MAP1LC3B
ENSG00000286186	5.671113253	2.598430319	1.145896744	0.354628057	0.480124625	AC018445.5
ENSG00000182196	36.77820552	25.53242738	0.524286555	0.354681833	0.480172409	ARL6IP4
ENSG00000230876	2.682005499	0.713078025	1.909207013	0.354742189	0.480229096	LINC00486
ENSG00000137731	1.81990531	0	3.197406419	0.35482722	1	FXYD2
ENSG00000260422	1.81990531	0	3.197406419	0.35482722	1	Z97205.2
ENSG00000286133	1.81990531	0	3.197406419	0.35482722	1	AC093817.2
ENSG00000272855	1.81990531	0	3.197406419	0.35482722	1	AC104458.1
ENSG00000231621	1.81990531	0	3.197406419	0.35482722	1	AC013264.1
ENSG00000279317	1.81990531	0	3.197406419	0.35482722	1	AC006994.2
ENSG00000182574	1.81990531	0	3.197406419	0.35482722	1	Z97985.1
ENSG00000287274	1.81990531	0	3.197406419	0.35482722	1	AL627232.1
ENSG00000222069	1.81990531	0	3.197406419	0.35482722	1	RN7SKP285
ENSG00000237633	1.81990531	0	3.197406419	0.35482722	1	AC104623.1
ENSG00000249426	1.81990531	0	3.197406419	0.35482722	1	AC093206.1
ENSG00000227537	1.81990531	0	3.197406419	0.35482722	1	AL139281.1
ENSG00000259780	1.81990531	0	3.197406419	0.35482722	1	AC009065.1
ENSG00000271171	1.81990531	0	3.197406419	0.35482722	1	AC008555.5
ENSG00000237737	1.466204238	4.686913718	-1.664812966	0.35484143	0.480338415	DCTN1-AS1
ENSG00000169252	174.2712343	115.7994116	0.591266055	0.354914258	0.48041197	ADRB2
ENSG00000215853	0.308355904	1.844058227	-2.497383606	0.354923216	1	RPTN
ENSG00000268333	0.307102255	1.844058227	-2.497385255	0.354993042	1	AL132655.1
ENSG00000269155	1.801029308	0	3.183334298	0.354995879	1	AL009178.2
ENSG00000265110	1.801029308	0	3.183334298	0.354995879	1	MIR4731
ENSG00000285666	1.801029308	0	3.183334298	0.354995879	1	AC006972.1
ENSG00000254675	1.801029308	0	3.183334298	0.354995879	1	AP003032.1
ENSG00000213293	1.801029308	0	3.183334298	0.354995879	1	AC012618.1
ENSG00000260372	1.801029308	0	3.183334298	0.354995879	1	AQP4-AS1
ENSG00000258284	1.801029308	0	3.183334298	0.354995879	1	POLR2KP1
ENSG00000263154	1.801029308	0	3.183334298	0.354995879	1	AC110285.3
ENSG00000239212	1.801029308	0	3.183334298	0.354995879	1	RPL6P7
ENSG00000248537	1.801029308	0	3.183334298	0.354995879	1	AC091906.1
ENSG00000096088	1.801029308	0	3.183334298	0.354995879	1	PGC
ENSG00000233785	1.801029308	0	3.183334298	0.354995879	1	AC131011.1
ENSG00000087086	44184.72297	56874.12844	-0.364221252	0.355035155	0.480547534	FTL
ENSG00000085982	1178.65051	1016.660704	0.213222332	0.355051401	0.480547534	USP40
ENSG00000196993	7.848551852	3.893559308	1.006217713	0.355102763	0.480592013	NPIPB9
ENSG00000270765	5.454701335	2.415496705	1.154623137	0.355207951	0.480709333	GAS2L2
ENSG00000128645	0.616711807	2.42610919	-1.99385601	0.355294095	0.480800869	HOXD1
ENSG00000283128	7.283453457	3.872334337	0.900112034	0.355333624	0.480809818	AC009403.2

ENSG00000185245	27.94663303	18.01694789	0.630282988	0.35533772	0.480809818	GP1BA
ENSG00000267316	5.155192243	2.51699806	1.034793715	0.355369229	0.480827411	AC090409.2
ENSG00000233799	4.807759415	9.030341053	-0.915244953	0.355410513	0.480858227	AC139887.1
ENSG00000151353	499.7832005	590.5063856	-0.240823659	0.355493026	0.480944819	TMEM18
ENSG00000205927	1.814890715	0	3.193686295	0.35566846	1	OLIG2
ENSG00000279707	1.814890715	0	3.193686295	0.35566846	1	AC120349.3
ENSG00000277590	1.814890715	0	3.193686295	0.35566846	1	MIR7845
ENSG00000207119	1.814890715	0	3.193686295	0.35566846	1	RF00012
ENSG00000235880	1.814890715	0	3.193686295	0.35566846	1	AL158832.1
ENSG00000177212	1.814890715	0	3.193686295	0.35566846	1	OR2T33
ENSG00000199508	1.814890715	0	3.193686295	0.35566846	1	RNA5SP110
ENSG00000253817	1.814890715	0	3.193686295	0.35566846	1	AC120036.2
ENSG00000286225	1.814890715	0	3.193686295	0.35566846	1	AC079163.2
ENSG00000237631	1.814890715	0	3.193686295	0.35566846	1	AL161454.1
ENSG00000259353	2.722336072	0.693008929	1.953113525	0.355693356	0.481189341	AC090515.4
novel.355	18.04045192	10.69848213	0.752701154	0.355710807	0.481189341	-
ENSG00000188958	3.368024343	6.960642481	-1.046221852	0.355753198	0.481221214	UTS2B
ENSG00000270106	0.293240848	1.844058227	-2.497403523	0.355768136	1	TSNAX-DISC1
ENSG00000142949	2250.161935	3421.467395	-0.60452101	0.355771413	0.481221214	PTPRF
ENSG00000069345	1245.544166	1391.274148	-0.159657378	0.355816919	0.481257711	DNAJA2
ENSG00000259933	9.849553102	16.18221788	-0.71164449	0.356011612	0.481495975	AC009065.2
ENSG00000234608	286.6181346	333.0176847	-0.216537829	0.356081436	0.481564194	MAPKAPK5-AS1
ENSG00000197705	2.46684723	0	3.635440994	0.356084462	1	KLHL14
ENSG00000287414	2.46684723	0	3.635440994	0.356084462	1	AC093787.2
novel.1074	2.46684723	0	3.635440994	0.356084462	1	-
ENSG00000253164	2.46684723	0	3.635440994	0.356084462	1	AC023403.1
ENSG00000236846	2.46684723	0	3.635440994	0.356084462	1	AL359979.2
novel.447	2.46684723	0	3.635440994	0.356084462	1	-
ENSG00000236485	2.46684723	0	3.635440994	0.356084462	1	LINC01945
ENSG00000226240	2.46684723	0	3.635440994	0.356084462	1	LINC00381
ENSG00000256591	12.05972911	17.68034958	-0.548379933	0.356099122	0.481564194	AP003108.2
ENSG00000164308	1083.26651	718.3041943	0.592694947	0.356335688	0.481859028	ERAP2
ENSG00000237331	0.308355904	1.834601616	-2.490442588	0.35645904	1	XIAP-AS1
ENSG00000232626	0.308355904	1.834601616	-2.490442588	0.35645904	1	AC099336.1
ENSG00000180861	2.082916045	0.387220594	2.43309026	0.356593974	1	LINC01559
ENSG00000286179	2.45681804	0	3.629773202	0.356850439	1	AL139351.2
ENSG00000259692	2.45681804	0	3.629773202	0.356850439	1	AC104041.1
novel.164	2.45681804	0	3.629773202	0.356850439	1	-
ENSG00000253563	2.45681804	0	3.629773202	0.356850439	1	NKX2-1-AS1
ENSG00000197177	2.45681804	0	3.629773202	0.356850439	1	ADGRA1
ENSG00000229924	2.45681804	0	3.629773202	0.356850439	1	FAM90A26
ENSG00000225451	2.45681804	0	3.629773202	0.356850439	1	AC092634.2
ENSG00000244219	11.47074012	6.225183609	0.889031988	0.356895722	0.482571099	TMEM225B
ENSG00000163293	44.1010919	62.71796759	-0.506201081	0.356899414	0.482571099	NIPAL1
ENSG00000239908	2.080408748	0.387220594	2.431541355	0.356938811	1	RN7SL75P
ENSG00000166793	58.99702601	43.06497286	0.458204815	0.356946305	0.482609385	YPEL4
ENSG00000236463	0.879722543	3.82158366	-2.111773509	0.356970666	0.482617207	LINC00427
ENSG00000088808	166.7468373	137.6994816	0.275121439	0.357138702	0.48279901	PPP1R13B
ENSG00000274227	7.518741378	3.892403433	0.944240541	0.357142303	0.48279901	AC073575.2
ENSG00000164621	0.293240848	1.834601616	-2.490460381	0.357309182	1	SMAD5-AS1
ENSG00000170604	778.7487516	677.8135671	0.200168162	0.357383139	0.483099445	IRF2BP1

ENSG00000142599	1937.755348	2341.5406	-0.272937704	0.357485715	0.483212963	RERE
ENSG00000188026	577.691696	507.000753	0.188484455	0.357573068	0.483303803	RILPL1
ENSG00000173809	11.04808801	4.097846288	1.419260308	0.357590124	0.483303803	AC008736.1
ENSG00000268070	1.188078446	3.455588036	-1.540548834	0.357882623	0.483650695	AC006539.2
ENSG00000116299	16.79846622	26.83914916	-0.674090551	0.357884015	0.483650695	KIAA1324
ENSG00000180340	676.1850828	1087.498827	-0.685590148	0.357961533	0.483730292	FZD2
ENSG00000236992	11.5488227	16.92597749	-0.55196407	0.357986542	0.483738927	RPL12P12
ENSG00000233680	2.082916045	0.356539013	2.433072055	0.358049543	1	HNRNPA1P27
ENSG00000226499	2.082916045	0.356539013	2.433072055	0.358049543	1	AL136380.1
ENSG00000156206	2.082916045	0.356539013	2.433072055	0.358049543	1	CFAP161
ENSG00000175727	713.5577273	581.3792826	0.296026807	0.358132526	0.483911024	MLXIP
ENSG00000119878	324.5551421	413.1246788	-0.348293615	0.358212383	0.483993755	CRIPT
ENSG00000239247	1.509042109	3.689272328	-1.278274524	0.358305289	0.484094109	RN7SL589P
ENSG00000251348	3.608326859	9.149599754	-1.343394785	0.358335804	0.484094849	HSPD1P11
novel.639	58.95586906	42.41197373	0.480319757	0.358343102	0.484094849	-
ENSG00000200579	2.080408748	0.356539013	2.43152179	0.35839656	1	RF00019
ENSG00000271215	2.080408748	0.356539013	2.43152179	0.35839656	1	RPL21P136
ENSG00000239367	4.775022005	1.161661783	2.066107131	0.358418176	0.484171093	RN7SL477P
ENSG00000239335	1.539272221	3.862877727	-1.342087252	0.358622863	0.484415148	LLPH-DT
ENSG00000221823	591.9193602	688.0666291	-0.217309796	0.358636133	0.484415148	PPP3R1
ENSG00000248554	5.491270962	2.42610919	1.159538609	0.35867137	0.484437558	AC114956.2
ENSG00000228322	0.586481695	3.047014077	-2.356617184	0.358786223	0.484567493	AL162419.1
ENSG00000162642	520.3358875	439.1562779	0.24428655	0.358873113	0.48465965	C1orf52
ENSG00000172771	2.698374204	5.401147618	-0.994848579	0.358974581	0.484771484	EFCAB12
ENSG00000227038	0.308355904	1.90542139	-2.539291886	0.359065928	1	GTF2IP7
ENSG00000270863	0.308355904	1.90542139	-2.539291886	0.359065928	1	DDX55P1
ENSG00000287848	0.308355904	1.90542139	-2.539291886	0.359065928	1	AL355303.2
ENSG00000288054	0.586481695	2.792104814	-2.241150805	0.359072119	0.484878001	AL360178.2
ENSG00000235748	0.307102255	1.90542139	-2.539295064	0.359130227	1	SEPT14P12
ENSG00000178449	433.0309186	520.1468261	-0.264686131	0.359166773	0.484980611	COX14
ENSG00000237513	1.215801261	3.444975551	-1.515338447	0.35924954	0.485067161	AC007384.1
ENSG00000215912	2.080408748	0.336469917	2.431508925	0.359361183	1	TTC34
ENSG00000235897	5.716458421	9.539132099	-0.741682967	0.359586802	0.485497308	TM4SF19-AS1
ENSG00000165912	917.3465652	740.9763003	0.308415143	0.359622157	0.485519811	PACSIN3
ENSG00000083838	118.9483809	95.04154165	0.323235627	0.35965495	0.485538855	ZNF446
ENSG00000118094	18.97304152	29.40257836	-0.634974522	0.359820141	0.485736625	TREH
ENSG00000272563	0.293240848	1.90542139	-2.539330262	0.359843907	1	AC016745.2
ENSG00000196417	175.6016596	150.281775	0.22558456	0.359864438	0.485771183	ZNF765
ENSG00000198099	1.789675198	0	3.174776192	0.359956802	1	ADH4
ENSG00000112164	1.789675198	0	3.174776192	0.359956802	1	GLP1R
ENSG00000223656	1.789675198	0	3.174776192	0.359956802	1	HMGB3P10
ENSG00000187762	1.789675198	0	3.174776192	0.359956802	1	HSPE1P11
novel.246	1.789675198	0	3.174776192	0.359956802	1	-
ENSG00000275040	1.789675198	0	3.174776192	0.359956802	1	AC009159.1
ENSG00000126251	1.789675198	0	3.174776192	0.359956802	1	GPR42
ENSG00000233430	1.789675198	0	3.174776192	0.359956802	1	AC239798.1
ENSG00000248115	1.789675198	0	3.174776192	0.359956802	1	AC023154.1
ENSG00000263232	1.789675198	0	3.174776192	0.359956802	1	ATP5F1AP3
ENSG00000215274	1.789675198	0	3.174776192	0.359956802	1	GAGE10
ENSG00000259274	1.789675198	0	3.174776192	0.359956802	1	AC107241.1
ENSG00000233708	1.7871679	0	3.172889763	0.360385848	1	AL512353.2

ENSG00000234504	1.7871679	0	3.172889763	0.360385848	1	AL353801.2
ENSG00000235806	1.7871679	0	3.172889763	0.360385848	1	AF241728.1
ENSG00000229621	1.7871679	0	3.172889763	0.360385848	1	LINC01822
ENSG00000167195	1.7871679	0	3.172889763	0.360385848	1	GOLGA6C
ENSG00000201544	1.7871679	0	3.172889763	0.360385848	1	SNORA16B
ENSG00000252321	1.7871679	0	3.172889763	0.360385848	1	RN7SKP83
ENSG00000271763	1.7871679	0	3.172889763	0.360385848	1	AC091544.5
ENSG00000218872	1.7871679	0	3.172889763	0.360385848	1	AL445189.1
ENSG00000229093	1.7871679	0	3.172889763	0.360385848	1	OR51AB1P
ENSG00000200711	1.7871679	0	3.172889763	0.360385848	1	RNA5SP28
ENSG00000280103	1.7871679	0	3.172889763	0.360385848	1	AC007792.1
ENSG00000111331	1508.612954	1800.85508	-0.255537488	0.360401196	0.486470463	OAS3
ENSG00000236991	3.902821355	8.457618305	-1.108795543	0.360439349	0.48648131	EDRF1-AS1
ENSG00000121361	76.41570773	34.12864997	1.160672337	0.360446681	0.48648131	KCNJ8
ENSG00000199719	0.907445357	2.862924587	-1.658303714	0.360492349	0.486505523	RN7SKP74
ENSG00000227598	2.391271949	5.370466037	-1.162478459	0.360502072	0.486505523	Z94721.1
ENSG00000092439	920.6867513	798.4134055	0.20568994	0.36073928	0.486800355	TRPM7
ENSG00000215014	20.10595907	12.63818112	0.661845221	0.360951379	0.487061275	AL645728.1
ENSG00000259592	4.596433363	1.345879667	1.745141379	0.360995034	0.487093816	PRELID1P4
ENSG00000138336	79.58792088	58.85346726	0.432437885	0.361012991	0.487093816	TET1
ENSG00000205352	889.4249006	1071.254454	-0.268573444	0.361054687	0.487124776	PRR13
ENSG00000146247	1088.827978	941.4957978	0.209899068	0.361095885	0.487155063	PHIP
ENSG00000022267	2900.390206	1824.543701	0.668639472	0.361130353	0.487167016	FHL1
ENSG00000204588	8.223636224	13.35241502	-0.707229891	0.361142247	0.487167016	LINC01123
ENSG00000152495	33.7178529	51.57359863	-0.614121637	0.36118734	0.487202548	CAMK4
ENSG00000270808	2.374903244	5.379922647	-1.171759485	0.361236405	0.487243435	AC022400.4
ENSG00000274584	5.133737672	2.171071533	1.246578935	0.361404087	0.487430387	AP001282.2
ENSG00000204531	4.220024071	1.426156051	1.561820423	0.361412531	0.487430387	POU5F1
ENSG00000141026	288.2616739	251.786808	0.195430668	0.361528999	0.487562155	MED9
ENSG00000183891	172.4859761	138.6668269	0.316694272	0.361563134	0.487567822	TTC32
ENSG00000225311	3.839853833	1.060160428	1.853692887	0.361570734	0.487567822	AL024474.2
ENSG00000060339	1183.151551	1422.133961	-0.265604943	0.361730804	0.48773752	CCAR1
ENSG00000174007	97.03898436	72.34456811	0.424083421	0.361734124	0.48773752	CEP19
ENSG00000163472	130.1102948	107.2211688	0.280178089	0.361772757	0.487744415	TMEM79
ENSG00000122778	127.334194	162.8319741	-0.353794905	0.361776784	0.487744415	KIAA1549
ENSG00000172987	1.848881773	0	3.218874006	0.361844822	1	HPSE2
ENSG00000285777	1.848881773	0	3.218874006	0.361844822	1	AL139142.2
ENSG00000237412	1.848881773	0	3.218874006	0.361844822	1	PRSS56
ENSG00000166828	1.848881773	0	3.218874006	0.361844822	1	SCNN1G
ENSG00000211804	1.848881773	0	3.218874006	0.361844822	1	TRDV1
ENSG00000206249	1.848881773	0	3.218874006	0.361844822	1	AL359555.1
ENSG00000256288	1.848881773	0	3.218874006	0.361844822	1	LINC02617
ENSG00000144035	1.848881773	0	3.218874006	0.361844822	1	NAT8
ENSG00000235659	1.848881773	0	3.218874006	0.361844822	1	AL391987.4
ENSG00000286707	19.53333878	9.661986819	1.007761557	0.361944067	0.487936788	AC010896.1
ENSG00000112941	751.4215959	893.2385545	-0.249055368	0.361957034	0.487936788	TENT4A
ENSG00000138744	353.7790487	438.5184332	-0.310510258	0.361977792	0.487939453	NAAA
ENSG00000255508	62.02613616	86.57865787	-0.480852435	0.362018003	0.487957054	AP002990.1
ENSG00000234996	4.591347497	1.702418679	1.404257793	0.362028412	0.487957054	AC098934.2
ENSG00000140905	149.0379911	110.606883	0.427989034	0.36206676	0.487983426	GCSH
ENSG00000091009	425.5635494	369.4545506	0.203368168	0.362099417	0.488002125	RBM27

ENSG00000247735	8.160668702	12.5353955	-0.61921636	0.362118909	0.488003079	AC120114.1
ENSG00000280219	11.28574069	21.87280916	-0.958783449	0.362226626	0.488122924	AC093908.1
ENSG00000228169	0.307102255	1.467450118	-2.165789288	0.362389063	1	PPIAP19
ENSG00000084234	12790.59152	10622.84841	0.267943793	0.362516302	0.488487944	APLP2
ENSG00000136152	574.0271402	660.8626746	-0.202755025	0.362590881	0.488563099	COG3
ENSG00000200033	0	2.0188195	-3.59940977	0.362631337	1	RNU6-403P
ENSG00000238107	0	2.0188195	-3.59940977	0.362631337	1	AC245100.6
ENSG00000145839	1.843867178	0	3.215192042	0.362664051	1	IL9
ENSG00000267079	1.843867178	0	3.215192042	0.362664051	1	AP001269.2
ENSG00000268531	1.843867178	0	3.215192042	0.362664051	1	AC060814.3
ENSG00000234142	1.843867178	0	3.215192042	0.362664051	1	LINC01675
ENSG00000277631	1.843867178	0	3.215192042	0.362664051	1	PGM5P3-AS1
ENSG00000279370	1.843867178	0	3.215192042	0.362664051	1	AC004777.1
ENSG00000189269	7.234347342	3.475657132	1.044616703	0.362678556	0.488645522	DRICH1
ENSG00000120662	199.937245	168.9504849	0.242807467	0.362689668	0.488645522	MTRF1
ENSG00000103174	377.9153894	315.1442214	0.261783044	0.36272606	0.488669213	NAGPA
novel.669	22.72248975	34.32659682	-0.59448668	0.362750541	0.488676854	-
ENSG00000124701	3.297463658	1.00940975	1.674996714	0.362786906	0.488700503	APOBEC2
ENSG00000114120	2022.557771	1556.746728	0.377806736	0.362806966	0.488702187	SLC25A36
ENSG00000272899	49.27813781	32.25322101	0.615179677	0.36290834	0.488813395	ATP6V1FNB
ENSG00000105479	3.677633896	7.285344037	-0.987176208	0.362932419	0.488820487	CCDC114
ENSG00000188760	102.7006101	85.98398664	0.255658376	0.362955392	0.488826087	TMEM198
ENSG00000197355	718.5299707	540.597949	0.410959945	0.362992937	0.488851311	UAP1L1
ENSG00000264304	3.549120283	8.653732944	-1.284985298	0.363142795	0.48902778	AC024267.3
ENSG00000230832	0.293240848	1.467450118	-2.165815711	0.36316292	1	AC241584.1
ENSG00000273196	0.293240848	1.467450118	-2.165815711	0.36316292	1	AC062037.2
ENSG00000230501	0.293240848	1.467450118	-2.165815711	0.36316292	1	ANKRD30BP3
ENSG00000235328	1.203193503	3.342189926	-1.460917016	0.363370954	0.48930967	AC006946.1
ENSG00000274653	4.796405305	9.121101528	-0.919671539	0.363523461	0.489489663	AC106782.6
ENSG00000170473	417.880765	478.8803794	-0.196332865	0.363578228	0.489538035	PYM1
ENSG00000177910	2.43410982	0.672939833	1.811380434	0.363702804	0.489680393	SPATA31C2
ENSG00000260577	2.150969433	0.387220594	2.474992218	0.363822151	1	AC126773.2
ENSG00000254866	2.729857965	0.723690511	1.920249618	0.363869777	0.489879815	DEFB109D
ENSG00000280057	6.728455523	2.742510011	1.27674495	0.363967547	0.489986053	AL022069.2
ENSG00000280120	59.42791202	77.52179201	-0.385813726	0.364022855	0.49003512	AC073857.1
ENSG00000264229	1.835020366	0	3.208643884	0.364079967	1	RNU4ATAC
ENSG00000225884	1.835020366	0	3.208643884	0.364079967	1	AC098872.1
ENSG00000233085	1.835020366	0	3.208643884	0.364079967	1	BX322234.2
ENSG00000161905	1.835020366	0	3.208643884	0.364079967	1	ALOX15
ENSG00000285907	1.835020366	0	3.208643884	0.364079967	1	AL161910.1
ENSG00000286162	41.90818224	32.14239144	0.386084769	0.364082939	0.490090608	AL162253.2
ENSG00000243415	2.460578986	5.971301827	-1.276795878	0.364110965	0.490102941	AC107021.1
ENSG00000279059	71.80205181	55.73422075	0.363364371	0.36418529	0.49017759	AC007485.2
ENSG00000232530	2.157237677	0.356539013	2.478815401	0.364308023	1	LIF-AS1
ENSG00000112763	783.3658695	910.5396459	-0.216917914	0.364382255	0.490393592	BTN2A1
ENSG00000140367	930.5801933	1084.973913	-0.221650333	0.364383522	0.490393592	UBE2Q2
ENSG00000212588	4.807759415	2.058957692	1.201464679	0.364459061	0.490454697	SNORA26
ENSG00000213614	986.0320025	852.9210188	0.209242529	0.364466681	0.490454697	HEXA
ENSG00000100441	957.0749865	1122.132192	-0.229347496	0.364490957	0.490461962	KHNYN
ENSG00000224805	3.914175465	1.803920035	1.123321925	0.364543699	0.490495815	LINC00853
ENSG00000226900	2.75758078	0.774441189	1.873119671	0.364553874	0.490495815	AL451069.1

ENSG00000215914	6.871799458	2.945512721	1.234001211	0.364608073	0.490543335	MMP23A
ENSG00000220157	3.328947419	6.309056013	-0.930445745	0.364668456	0.49059917	HNRNPA1P12
ENSG00000227001	7.846044555	15.88588616	-1.016527591	0.364867449	0.490841465	NBPF2P
ENSG00000253767	2.080408748	5.023383634	-1.264440351	0.364931854	0.490902687	PCDHGA8
ENSG00000183439	80.8664762	60.26533359	0.421604429	0.364965668	0.490922756	TRIM61
ENSG00000247934	25.39718682	15.9083954	0.669743746	0.365053998	0.491016149	AC022364.1
ENSG00000143437	1121.900641	978.8167453	0.197161055	0.365073942	0.491017554	ARNT
ENSG00000134108	1480.52238	1665.165138	-0.169486443	0.365101326	0.491028966	ARL8B
ENSG00000260930	1.828752122	0	3.204009373	0.365121284	1	LINC01416
ENSG00000280870	1.828752122	0	3.204009373	0.365121284	1	AC233296.1
ENSG00000288086	1.828752122	0	3.204009373	0.365121284	1	AL157414.4
ENSG00000229815	1.828752122	0	3.204009373	0.365121284	1	CCNB1IP1P1
ENSG00000278744	1.828752122	0	3.204009373	0.365121284	1	AL449363.1
ENSG00000237410	2.460578986	7.314869743	-1.57365676	0.365126817	0.49103783	AP001092.1
ENSG00000227825	19.55826957	27.74962609	-0.505234913	0.36530685	0.491254517	SLC9A7P1
ENSG00000101473	368.455565	433.4571163	-0.233844807	0.365419768	0.491379917	ACOT8
novel.434	92.34234527	62.70380626	0.56052785	0.36545423	0.491379917	-
ENSG00000273828	21.59709409	14.18577923	0.612524564	0.365456839	0.491379917	AL133227.1
ENSG00000181433	2.34592678	0	3.565570261	0.365600305	1	SAGE1
ENSG00000167355	2.34592678	0	3.565570261	0.365600305	1	OR51B5
ENSG00000167941	2.34592678	0	3.565570261	0.365600305	1	SOST
ENSG00000180016	2.34592678	0	3.565570261	0.365600305	1	OR1E1
ENSG00000279573	2.34592678	0	3.565570261	0.365600305	1	AC134407.2
ENSG00000240240	2.34592678	0	3.565570261	0.365600305	1	BX664727.3
ENSG00000278492	6.301686468	3.200550379	0.975434779	0.365841505	0.49187167	AC006213.5
ENSG00000270149	5.000209465	1.386017859	1.841462581	0.365896452	0.491912561	AL591806.3
ENSG00000111596	1105.665351	958.5671868	0.206244747	0.365909786	0.491912561	AC092881.1
ENSG00000177156	3223.675825	3715.340678	-0.204754963	0.365945682	0.491935363	TALDO1
ENSG00000246985	35.72114798	47.62817995	-0.417012358	0.366005817	0.491984764	SOCS2-AS1
ENSG00000279227	17.55726832	25.59006613	-0.53885497	0.366020304	0.491984764	AC009303.4
ENSG00000157445	33.09953143	60.51338914	-0.86884292	0.366055209	0.49199946	CACNA2D3
ENSG00000136918	1.200686205	3.841652756	-1.679689426	0.36606911	0.49199946	WDR38
ENSG00000100652	2.14337627	0.356539013	2.470338117	0.366181126	1	SLC10A1
ENSG00000239482	2.14337627	0.356539013	2.470338117	0.366181126	1	AC112487.1
ENSG00000149357	1632.875044	1885.520814	-0.207547621	0.366200088	0.492150035	LAMTOR1
ENSG00000085719	1987.619266	1747.567633	0.185657033	0.366426962	0.492429468	CPNE3
ENSG00000267800	3.604565913	1.365948763	1.372665176	0.36648227	0.492477231	AC012254.4
ENSG00000167515	629.6030892	707.2332608	-0.167697411	0.366500414	0.492477231	TRAPPC2L
ENSG00000080815	1116.836396	1252.995231	-0.166106775	0.366602973	0.492589565	PSEN1
ENSG00000133488	10.14530125	4.554602387	1.171903976	0.366701067	0.492674447	SEC14L4
ENSG00000125458	542.0198401	448.7033727	0.272326604	0.366704071	0.492674447	NT5C
ENSG00000232176	3.62978143	1.060160428	1.768987261	0.366729779	0.49268351	AL161909.1
ENSG00000138448	3563.197281	4065.598031	-0.190279307	0.366841369	0.492807943	ITGAV
ENSG00000134744	721.3590247	615.4597841	0.229258274	0.366902871	0.492845616	TUT4
ENSG00000100941	2319.717245	2702.430277	-0.220257425	0.366907352	0.492845616	PNN
ENSG00000135317	929.2535602	1055.343897	-0.18334734	0.366961446	0.492892794	SNX14
ENSG00000234206	2.14337627	0.336469917	2.470344475	0.367063609	1	AL121957.1
ENSG00000169075	2.135854377	0.356539013	2.46574146	0.367200662	1	Z99496.1
novel.556	2.135854377	0.356539013	2.46574146	0.367200662	1	-
ENSG00000112365	231.1371077	184.4722545	0.32463004	0.36731422	0.493310098	ZBTB24
ENSG00000234369	1.804790254	4.361056287	-1.266562981	0.367315915	0.493310098	TATDN1P1

ENSG00000188693	5.524008372	2.069570178	1.39902882	0.367329092	0.493310098	CYP51A1-AS1
ENSG00000235527	2.3900183	5.166179057	-1.097298552	0.367349644	0.493312198	HIPK1-AS1
ENSG00000110375	1.539272221	4.025742245	-1.389389789	0.367378961	0.493326069	UPK2
ENSG00000233614	4.844329042	1.753169357	1.45656969	0.367451474	0.493397941	DDX11L10
ENSG00000270996	2.758834428	0.713078025	1.945081375	0.367540142	0.493491495	AC005034.4
ENSG00000229585	2.714742909	0.743759607	1.888418057	0.367595414	0.493540202	RPL21P44
ENSG00000226419	9.847045804	5.166179057	0.943099573	0.367668092	0.493612272	SLC16A1-AS1
ENSG00000272768	15.95656696	8.988918592	0.826652353	0.367790501	0.493751098	AC004854.2
ENSG00000198814	226.1428817	293.8445889	-0.3767633	0.367872119	0.493835152	GK
ENSG00000142089	20133.84773	17280.07438	0.220499769	0.367967615	0.493937824	IFITM3
ENSG00000095739	198.2613091	286.0704885	-0.529124876	0.368074378	0.494055611	BAMBI
ENSG00000251972	2.932408476	0.387220594	2.926116105	0.368097607	0.494061266	RNU6-123P
ENSG00000230585	0.307102255	1.436768536	-2.142126874	0.368231394	1	PHBP12
ENSG00000213996	6.435214676	13.55169332	-1.071218598	0.368273919	0.494272378	TM6SF2
ENSG00000206384	8.79876381	17.08640186	-0.953985848	0.368365261	0.494369433	COL6A6
ENSG00000266733	8.371994754	3.761376372	1.13986531	0.368430871	0.494431945	TBC1D29P
ENSG00000164081	783.7025329	884.0186613	-0.173713902	0.368518232	0.494500321	TEX264
ENSG00000166153	9.540228284	18.7806482	-0.979078608	0.368529939	0.494500321	DEPDC4
ENSG00000279762	6.038675732	3.230076085	0.903224176	0.368538921	0.494500321	AC005899.8
ENSG00000143033	460.4958508	400.3482314	0.202594328	0.368634856	0.494603501	MTF2
ENSG00000271670	4.459072938	1.069617038	2.058067133	0.368766428	0.494754483	AC010998.2
ENSG00000266967	28.74451204	41.4302103	-0.527454481	0.36882647	0.494801839	AARSD1
ENSG00000196981	226.9065837	176.0986842	0.364250709	0.368839814	0.494801839	WDR5B
ENSG00000164180	386.6993667	316.6482477	0.288542123	0.368881527	0.494832246	TMEM161B
ENSG00000286509	11.73876545	5.820334064	0.999336302	0.368944756	0.494891513	AC026408.1
ENSG00000165792	633.561514	536.4554947	0.240106621	0.36899218	0.494929573	METTL17
ENSG00000215482	0.293240848	1.436768536	-2.142127337	0.369027002	1	CALM2P3
ENSG00000232194	0.293240848	1.436768536	-2.142127337	0.369027002	1	Z99943.1
ENSG00000259922	0.293240848	1.436768536	-2.142127337	0.369027002	1	AC023825.1
ENSG00000258581	3.605819561	1.406086955	1.347644213	0.369075234	0.495015417	AL157871.3
ENSG00000029559	6.173172855	1.702418679	1.845845428	0.369146904	0.495085986	IBSP
ENSG00000273416	3.931797819	1.456837632	1.439408821	0.369234354	0.495177708	AL732292.2
ENSG00000228283	11.18000657	16.28487511	-0.53855678	0.369259694	0.495186131	KATNBL1P6
ENSG00000176435	293.9687713	151.5706707	0.955032903	0.369330939	0.495249109	CLEC14A
ENSG00000155366	6613.905174	5781.734339	0.19398933	0.36934478	0.495249109	RHOC
ENSG00000248240	3.016830569	1.060160428	1.50062931	0.369383526	0.495275501	AC114956.1
ENSG00000205559	39.40727268	54.57731086	-0.471586644	0.369568714	0.495485746	CHKB-DT
ENSG00000161914	124.7817854	100.7399201	0.3097995	0.369578472	0.495485746	ZNF653
ENSG00000140153	410.5454568	484.9804434	-0.24046932	0.369645489	0.495544778	WDR20
ENSG00000245213	35.80836211	23.51959687	0.603693262	0.369661328	0.495544778	AC105285.1
ENSG00000125901	715.137956	854.4222585	-0.256933845	0.369679723	0.495544778	MRPS26
ENSG00000152137	191.520103	280.9255813	-0.553176749	0.369869711	0.49576164	HSPB8
ENSG00000267673	2.694613258	0.743759607	1.879094749	0.369879667	0.49576164	FDX2
ENSG00000185561	4.222531369	1.702418679	1.282894333	0.370026467	0.495932816	TLCD2
ENSG00000180964	1270.588277	1462.614396	-0.203068023	0.37016073	0.496087173	TCEAL8
ENSG00000256671	1.218308559	3.210006989	-1.403051302	0.370193702	0.49610577	LIMS4
ENSG00000177238	2.729857965	0.743759607	1.895103968	0.370235911	0.496136745	TRIM72
ENSG00000268357	18.55135833	10.98638473	0.762911415	0.370330312	0.496237652	VN1R81P
ENSG00000174943	500.1672457	420.6156285	0.25072165	0.370388036	0.496272818	KCTD13
ENSG00000173821	4135.822369	3598.542375	0.20075888	0.370394758	0.496272818	AC124319.1
ENSG00000229955	3.006730108	1.060160428	1.496644672	0.37047491	0.496354612	Z98749.1

ENSG00000188517	26.17235762	17.91403388	0.551574025	0.370519878	0.496389262	COL25A1
ENSG00000263033	3.32769377	1.467450118	1.197096807	0.370584144	0.49644976	AC007220.1
ENSG00000283980	0.925067711	3.149799701	-1.780572905	0.370610989	0.496460123	GNG14
ENSG00000105929	2.744973021	6.725673919	-1.288399299	0.370703748	0.496558779	ATP6V0A4
ENSG00000135801	400.7249511	461.7889686	-0.204704273	0.370815281	0.49668257	TAF5L
ENSG00000105939	1052.905334	1262.348055	-0.261893072	0.37086712	0.496726396	ZC3HAV1
ENSG00000243422	20.35385475	11.37501798	0.830336786	0.370905619	0.496752353	RPL23AP49
ENSG00000269919	1.759445085	5.542787165	-1.642830107	0.370953891	0.496791394	AL022067.1
ENSG00000218510	46.0816075	33.83676617	0.441841765	0.371120695	0.496989164	LINC00339
ENSG00000114331	953.1883597	1077.791599	-0.17726613	0.371214703	0.497074342	ACAP2
ENSG00000215808	111.3193452	188.5638082	-0.760468233	0.371222565	0.497074342	LINC01139
ENSG00000207697	5.406848869	1.936102971	1.510096334	0.371259727	0.497098483	MIR573
ENSG00000234171	146.7071794	171.8438343	-0.228015819	0.371353508	0.497198427	RNASEH1-AS1
ENSG00000224613	1.54052587	5.434269345	-1.828856226	0.371494814	0.497361989	AC095032.1
ENSG00000196653	5.437078981	2.873537073	0.921078487	0.371664131	0.497563033	ZNF502
ENSG00000126368	209.8297069	284.491554	-0.438245533	0.371800672	0.497720181	NR1D1
ENSG00000269956	7.240615586	3.943154111	0.876690488	0.372016291	0.497983166	MKNK1-AS1
ENSG00000266821	0.293240848	1.742556871	-2.426547634	0.372065662	1	AC018521.7
ENSG00000199890	0.293240848	1.742556871	-2.426547634	0.372065662	1	RF00019
ENSG00000160973	1.801029308	4.505135979	-1.327564119	0.372082384	0.498045977	FOXH1
ENSG00000250751	0.308355904	1.41669944	-2.12603664	0.37213981	1	AC015795.1
ENSG00000249077	0.307102255	1.41669944	-2.12603516	0.372212709	1	AL158068.1
ENSG00000287903	0.307102255	1.41669944	-2.12603516	0.372212709	1	AL359852.2
ENSG00000267681	4.573725143	1.90542139	1.291006127	0.372303152	0.49831581	AC135721.1
ENSG00000136371	22.81959087	15.52104641	0.557022506	0.372329265	0.49832509	MTHFS
ENSG00000279373	0	1.936102971	-3.528501137	0.37244816	1	AC027228.3
ENSG00000287856	0	1.936102971	-3.528501137	0.37244816	1	AL445524.2
ENSG00000250302	0	1.936102971	-3.528501137	0.37244816	1	LINC01618
ENSG00000253968	0	1.936102971	-3.528501137	0.37244816	1	AC016573.1
ENSG00000197919	0	1.936102971	-3.528501137	0.37244816	1	IFNA1
ENSG00000136834	0	1.936102971	-3.528501137	0.37244816	1	OR1J1
ENSG00000271412	0	1.936102971	-3.528501137	0.37244816	1	PRR13P3
ENSG00000287560	0	1.936102971	-3.528501137	0.37244816	1	AL731533.3
ENSG00000278496	0	1.936102971	-3.528501137	0.37244816	1	AL158062.1
ENSG00000223361	8.460177794	4.177994278	1.038549903	0.372458769	0.498472738	FTH1P10
ENSG00000175595	202.8930021	236.4135185	-0.220701798	0.372498911	0.498500783	ERCC4
ENSG00000151092	448.7583393	524.2848575	-0.224879957	0.372634446	0.498656479	NGLY1
ENSG00000253112	0.879722543	3.749479616	-2.075455779	0.372716507	0.498740604	AC102945.1
ENSG00000268218	32.39818407	45.00120423	-0.470289538	0.372737804	0.498743415	AC137932.3
ENSG00000261527	0.293240848	1.41669944	-2.126018743	0.373022818	1	AC026464.5
ENSG00000134899	70.89363754	91.81959073	-0.372348594	0.3730431	0.499126212	ERCC5
ENSG00000265478	7.550225139	4.33153058	0.805894184	0.373155604	0.49925103	AC107982.3
ENSG00000164610	219.5516722	255.9709669	-0.22159044	0.373211776	0.499300471	RP9
ENSG00000207352	0.308355904	1.813376645	-2.476525846	0.373244482	1	RNU6-540P
ENSG00000239617	0.308355904	1.813376645	-2.476525846	0.373244482	1	AC073610.1
ENSG00000123144	3327.797041	3748.34199	-0.171670522	0.373367394	0.499469534	TRIR
ENSG00000118363	1561.447764	1391.138078	0.166487124	0.373407896	0.499469534	SPCS2
ENSG00000127334	435.1733256	534.8784862	-0.297834459	0.373420544	0.499469534	DYRK2
ENSG00000272556	87.20624285	60.1861659	0.534984851	0.373429745	0.499469534	GTF2IP13
novel.413	69.49856346	113.7238997	-0.7099544	0.373434267	0.499469534	-
ENSG00000261360	16.8123989	10.02900992	0.743390141	0.373513997	0.499550456	AC010491.1

ENSG00000259804	5.705104311	2.833398881	1.005066367	0.373736933	0.499822888	AC027682.1
ENSG00000273199	5.380379703	2.598430319	1.070897063	0.373763244	0.499832347	AP000692.2
ENSG00000133398	822.071696	688.0168372	0.256376582	0.373839066	0.499882846	MED10
ENSG00000224531	495.9442005	571.7070338	-0.205538425	0.373839487	0.499882846	SMIM13
ENSG00000286106	1.524157165	4.167381792	-1.442268042	0.373959152	0.500017124	AC253572.1
ENSG00000005810	1092.179844	897.4989603	0.283116963	0.374018161	0.500065632	MYCBP2
ENSG00000113721	3148.036669	4167.369069	-0.404747299	0.374033926	0.500065632	PDGFRB
ENSG00000199107	0.293240848	1.813376645	-2.476531959	0.37406988	1	MIR409
ENSG00000134072	112.3236508	133.9051591	-0.255069377	0.374132936	0.500172266	CAMK1
ENSG00000220867	0.586481695	2.191140629	-1.877905975	0.374195161	0.500229714	HSPE1P26
ENSG00000138081	414.9122576	488.202544	-0.23427666	0.374230796	0.500237707	FBXO11
ENSG00000274444	5.163967785	2.038888596	1.316334369	0.374239649	0.500237707	AC078909.2
ENSG00000199940	1.774560141	0	3.163220146	0.374403809	1	RN7SKP75
ENSG00000235772	1.774560141	0	3.163220146	0.374403809	1	AP001625.2
ENSG00000235118	1.774560141	0	3.163220146	0.374403809	1	FAM237A
ENSG00000275836	1.774560141	0	3.163220146	0.374403809	1	MIR6068
ENSG00000224933	1.774560141	0	3.163220146	0.374403809	1	LINC01034
ENSG00000273365	1.774560141	0	3.163220146	0.374403809	1	AL451074.5
ENSG00000249131	1.774560141	0	3.163220146	0.374403809	1	PSD2-AS1
ENSG00000251767	1.774560141	0	3.163220146	0.374403809	1	RNU7-8P
ENSG00000253148	1.774560141	0	3.163220146	0.374403809	1	RGS21
ENSG00000237286	1.774560141	0	3.163220146	0.374403809	1	AC004906.1
ENSG00000227914	1.774560141	0	3.163220146	0.374403809	1	AL136979.1
ENSG00000263821	1.774560141	0	3.163220146	0.374403809	1	AP005121.2
ENSG00000234040	0.307102255	1.722487775	-2.412072215	0.374604736	1	RPL10P12
ENSG00000256056	0.307102255	1.722487775	-2.412072215	0.374604736	1	AC087257.2
ENSG00000274294	1.773306493	0	3.162266528	0.374624876	1	AC015660.3
novel.28	1.773306493	0	3.162266528	0.374624876	1	-
ENSG00000224204	1.773306493	0	3.162266528	0.374624876	1	PHEX-AS1
ENSG00000226330	1.773306493	0	3.162266528	0.374624876	1	AL606489.1
ENSG00000282051	1.773306493	0	3.162266528	0.374624876	1	AC010507.1
ENSG00000237307	1.773306493	0	3.162266528	0.374624876	1	SRRM1P3
ENSG00000286457	1.773306493	0	3.162266528	0.374624876	1	AC009560.2
ENSG00000285548	1.773306493	0	3.162266528	0.374624876	1	AC068725.1
ENSG00000213744	1.773306493	0	3.162266528	0.374624876	1	RPS10P14
ENSG00000254787	1.773306493	0	3.162266528	0.374624876	1	AP001825.1
ENSG00000263904	1.773306493	0	3.162266528	0.374624876	1	AC015563.1
ENSG00000271389	1.773306493	0	3.162266528	0.374624876	1	OSBPL9P1
ENSG00000262151	1.773306493	0	3.162266528	0.374624876	1	AC133065.1
ENSG00000279474	1.773306493	0	3.162266528	0.374624876	1	AC125437.2
ENSG00000279130	2.446717578	0.672939833	1.819657595	0.374718839	0.500852462	AC091925.1
ENSG00000144426	186.7588158	241.3026983	-0.369503679	0.374781727	0.500910749	NBEAL1
novel.1098	4.859444098	1.548882377	1.676507368	0.374849258	0.500975235	-
ENSG00000136235	4062.039162	2739.979003	0.568106123	0.375032841	0.501194807	GPNMB
ENSG00000257058	9.407953725	5.553399651	0.774494595	0.375067394	0.501215202	AP001363.2
ENSG00000260475	6.909551463	2.793260689	1.298796273	0.375188557	0.501351329	AL353719.1
ENSG00000243607	2.067800989	0.387220594	2.423402521	0.375271969	1	RPL35AP26
ENSG00000157110	1251.905832	1577.015239	-0.332922176	0.375349076	0.50154003	RBPMS
ENSG00000074755	1179.350532	1029.014553	0.196868464	0.375406366	0.501590785	ZZEF1
ENSG00000255348	0.293240848	1.722487775	-2.412039541	0.375447415	1	AP001775.2
ENSG00000266965	0.293240848	1.722487775	-2.412039541	0.375447415	1	AC090220.1

ENSG00000272779	6.412577727	11.31352643	-0.825102981	0.375520235	0.501717126	AC245060.4
ENSG00000258896	6.427621512	2.618499415	1.308343035	0.375552651	0.501734634	SCOCP1
ENSG00000157778	549.4781487	633.907362	-0.206288089	0.375888276	0.502142089	PSMG3
novel.934	1122.748714	4539.047871	-2.015365312	0.375896289	0.502142089	-
ENSG00000280927	44.93385962	57.18365673	-0.346187347	0.375925455	0.502155231	CTBP1-AS
ENSG00000104450	48.20904234	68.59218162	-0.511347818	0.37597925	0.502184423	SPAG1
ENSG00000277157	0.61420451	2.160459047	-1.822096604	0.375997902	0.502184423	HIST1H4D
ENSG00000255495	15.81419194	9.326672778	0.74923178	0.376005296	0.502184423	AC145124.1
ENSG00000152433	53.42949559	41.22348317	0.373327563	0.37603212	0.502194434	ZNF547
ENSG00000204936	21.5697982	6.553481185	1.714732476	0.376082194	0.502235492	CD177
ENSG00000260615	3.951998741	1.487519214	1.426247148	0.376115519	0.502254179	RPL23AP97
ENSG00000102554	228.0045673	431.4029351	-0.920267542	0.376220697	0.50236881	KLF5
ENSG00000110675	13.21410123	29.35473455	-1.150620222	0.376446611	0.50264464	ELMOD1
ENSG00000244411	2.067800989	0.356539013	2.423379689	0.376663025	1	KRTAP5-7
ENSG00000242882	2.067800989	0.356539013	2.423379689	0.376663025	1	RPL5P11
ENSG00000285799	2.067800989	0.356539013	2.423379689	0.376663025	1	AL645929.3
ENSG00000264739	2.067800989	0.356539013	2.423379689	0.376663025	1	AC093484.2
ENSG00000085274	301.4527112	262.3697559	0.200167339	0.376788003	0.503074624	MYNN
ENSG00000142513	5.369025593	2.160459047	1.317183358	0.376809118	0.503076963	ACP4
ENSG00000233144	2.06654734	0.356539013	2.422593172	0.376840335	1	AL512656.1
ENSG00000230068	1.21705491	2.904218654	-1.253179383	0.376842952	0.503093921	CDC42-IT1
ENSG00000143498	82.25982592	100.4877077	-0.290689032	0.376860548	0.503093921	TAF1A
ENSG00000228779	0.894837599	2.51699806	-1.48216375	0.376936017	0.503168816	Z69666.1
ENSG00000278875	6.891929109	13.81618759	-0.996378098	0.377142596	0.503404775	AC025809.2
ENSG00000288007	5.506386018	2.598430319	1.09749076	0.377153629	0.503404775	AC092803.5
ENSG00000121390	706.7313722	821.0769793	-0.216509738	0.377176518	0.503404775	PSPC1
ENSG00000230613	5.381633352	2.169915658	1.314961258	0.377190339	0.503404775	HM13-AS1
ENSG00000280327	6.099135957	9.96764676	-0.714520447	0.37720966	0.503404775	Z97633.1
ENSG00000286811	9.387752802	5.4318292	0.793453037	0.377256359	0.503441237	AL353138.1
ENSG00000285658	1.200686205	3.159256312	-1.398157147	0.377315494	0.503494291	AC127035.1
ENSG00000136940	414.0617524	480.053972	-0.213228052	0.377400036	0.503581241	PDCL
ENSG00000006715	1016.239072	888.6388045	0.193616566	0.377634817	0.503868641	VPS41
ENSG00000270987	2.06654734	0.336469917	2.422577749	0.37776149	1	AL133338.2
ENSG00000227154	0.293240848	2.495773089	-2.93967276	0.377771881	0.504025638	AL022721.1
ENSG00000129292	1112.456561	972.9939832	0.193123193	0.377825898	0.504063739	PHF20L1
ENSG00000253327	8.217367981	3.741307276	1.11626243	0.377839241	0.504063739	RAD21-AS1
ENSG00000268628	3.991004395	1.396630344	1.499713995	0.377859528	0.504064921	AL121761.1
ENSG00000269386	85.30180073	70.27499793	0.282481106	0.377953124	0.504163891	RAB11B-AS1
ENSG00000124333	903.8992513	769.0279213	0.232881263	0.378036039	0.504248606	VAMP7
ENSG00000234810	29.52756074	16.95922761	0.794649837	0.378204066	0.504446832	AL603840.1
ENSG00000106615	2096.415306	1823.675847	0.20095838	0.378288019	0.504532907	RHEB
ENSG00000092068	101.8492504	63.25928524	0.685824039	0.378442152	0.504712569	SLC7A8
ENSG00000240204	2.416487466	0.672939833	1.804376434	0.378543286	0.504821534	SMKR1
ENSG00000163684	384.9438526	328.2996227	0.229127825	0.378594248	0.504849994	RPP14
novel.551	4.306953462	1.386017859	1.618598664	0.378603491	0.504849994	-
ENSG00000248489	21.82013026	14.26978003	0.603024354	0.378639836	0.504872547	LINC02062
ENSG00000081181	31.40925119	18.74855396	0.751338432	0.37880093	0.505061427	ARG2
ENSG00000214376	6.112997364	3.210006989	0.922831427	0.379197171	0.505563797	VSTM5
ENSG00000063761	157.2157127	126.3744908	0.313487974	0.379256844	0.50561741	ADCK1
ENSG00000198736	465.4477371	399.2314725	0.220685556	0.37928747	0.505632293	MSRB1
ENSG00000153162	523.1996606	960.8304859	-0.876995731	0.379451355	0.505805897	BMP6

ENSG00000266392	3.016830569	6.287831042	-1.065171526	0.379456631	0.505805897	MIR4740
ENSG00000215529	6.155835235	2.099095884	1.542124402	0.379582207	0.505947328	EFCAB8
ENSG00000112299	73.2218707	43.77406968	0.745601235	0.379651334	0.505989219	VNN1
ENSG00000127184	3009.035076	3595.465099	-0.256887458	0.379652586	0.505989219	COX7C
ENSG00000224086	132.3912884	108.2901436	0.289012211	0.379776456	0.506128346	AC245452.1
ENSG00000221869	3180.664428	2532.880034	0.328481824	0.379843554	0.506191803	CEBPD
ENSG00000229981	2.12199297	5.207473124	-1.288047904	0.379925473	0.506275002	LINC01435
ENSG00000123700	87.68193238	131.3360445	-0.584093861	0.380157036	0.506557593	KCNJ2
ENSG00000257704	408.2862724	499.8976535	-0.291869585	0.380230222	0.50662913	INAFM1
ENSG00000288012	6.044943976	2.50754145	1.269443709	0.380310726	0.506710409	AP003307.1
ENSG00000270332	1.50778846	3.627909165	-1.26109052	0.380470113	0.506874048	SMC2-AS1
ENSG00000218739	710.1159074	816.6022681	-0.201725455	0.380478933	0.506874048	CEBPZOS
ENSG00000228847	3.619680969	6.602947593	-0.857489946	0.380492074	0.506874048	ATP5MC2P4
ENSG00000277678	2.66939774	0.723690511	1.891723195	0.380614874	0.50701164	RF00003
ENSG00000138085	1689.633201	1496.682115	0.174883919	0.38103658	0.507511432	ATRAID
ENSG00000225447	3.617173671	1.386017859	1.364996944	0.381041957	0.507511432	RPS15AP10
ENSG00000147324	39.41640423	52.13228845	-0.407323008	0.38104867	0.507511432	MFHAS1
ENSG00000163975	115.5378313	157.3602635	-0.445733874	0.381244483	0.507698281	MELTF
ENSG00000234825	0.586481695	2.140389951	-1.850305304	0.381247583	0.507698281	XRCC6P2
ENSG00000225037	0.586481695	2.140389951	-1.850305304	0.381247583	0.507698281	EIF1AX-AS1
ENSG00000140262	1313.624603	1107.417214	0.246191944	0.381408617	0.507886693	TCF12
ENSG00000248202	5.123708482	1.722487775	1.558207295	0.381434215	0.507894749	LINC02234
ENSG00000183607	0.307102255	1.762625967	-2.440711575	0.381537652	1	GKN2
ENSG00000286996	11.19637527	5.880541352	0.917182393	0.381587065	0.508072236	AC099332.2
ENSG00000137720	161.4822922	130.3300556	0.308535686	0.381622442	0.508093301	C11orf1
ENSG00000159496	7.053180132	3.566546002	0.977834822	0.381654022	0.508109308	RGL4
ENSG00000170262	3.077290793	0.713078025	2.10359402	0.381831247	0.508319206	MRAP
ENSG00000143028	73.8408329	45.05251566	0.709811765	0.381967497	0.508474537	SYPL2
ENSG00000073803	461.3727813	407.8935406	0.178160958	0.381991525	0.50848047	MAP3K13
ENSG00000165806	366.5621923	427.2367314	-0.221384011	0.382064737	0.508549835	CASP7
ENSG00000285868	2.11063886	0.693008929	1.580849514	0.382082783	0.508549835	AC008676.3
ENSG00000108523	1365.028937	1575.645346	-0.207204242	0.382179561	0.508652588	RNF167
ENSG00000264961	0.293240848	1.762625967	-2.440698248	0.382320395	1	MIR4730
ENSG00000185013	0.293240848	1.762625967	-2.440698248	0.382320395	1	NT5C1B
ENSG00000237550	90.35236996	68.88658711	0.39238991	0.382406081	0.508927998	AC243919.1
ENSG00000137802	597.8236824	499.1362989	0.260601802	0.382452187	0.508963288	MAPKBP1
novel.529	0.921306765	3.881790948	-2.08150361	0.382537414	0.509050633	-
ENSG00000273679	0.616711807	2.211209725	-1.845733537	0.382610023	0.509121178	AC100757.1
ENSG00000273369	10.93085724	6.808390448	0.681691025	0.38264268	0.509138557	AC096586.2
ENSG00000263818	3.914175465	1.345879667	1.510767122	0.382663429	0.50914009	RDM1P5
novel.751	2.158491326	0	3.442673231	0.382722782	1	-
ENSG00000166863	2.158491326	0	3.442673231	0.382722782	1	TAC3
ENSG00000179930	2.158491326	0	3.442673231	0.382722782	1	ZNF648
ENSG00000250620	2.158491326	0	3.442673231	0.382722782	1	LINC02515
novel.1092	2.158491326	0	3.442673231	0.382722782	1	-
ENSG00000269707	2.158491326	0	3.442673231	0.382722782	1	AC018730.1
ENSG00000257060	2.158491326	0	3.442673231	0.382722782	1	AC091078.1
ENSG00000236056	2.158491326	0	3.442673231	0.382722782	1	GAPDHP14
ENSG00000201595	2.158491326	0	3.442673231	0.382722782	1	RNA5SP132
ENSG00000229106	2.158491326	0	3.442673231	0.382722782	1	BTBD6P1
ENSG00000201003	2.158491326	0	3.442673231	0.382722782	1	RF00418

ENSG00000251604	2.158491326	0	3.442673231	0.382722782	1	LINC01385
ENSG00000285292	2.158491326	0	3.442673231	0.382722782	1	ABCF2
ENSG00000228527	2.158491326	0	3.442673231	0.382722782	1	AC016396.1
ENSG00000258826	2.158491326	0	3.442673231	0.382722782	1	LINC01147
ENSG00000261469	2.158491326	0	3.442673231	0.382722782	1	AC020978.4
ENSG00000261226	2.158491326	0	3.442673231	0.382722782	1	AC092384.3
ENSG00000276700	2.158491326	0	3.442673231	0.382722782	1	RNA5-8SN4
ENSG00000206418	771.9387379	685.9811338	0.170688618	0.382741124	0.509217387	RAB12
ENSG00000116035	17.53330645	25.78245636	-0.550243932	0.382901784	0.509405052	VAX2
ENSG00000197969	676.129119	537.7538211	0.33039632	0.382922358	0.509406338	VPS13A
ENSG00000271889	4.85686553	9.683083396	-0.996113322	0.383066312	0.509561639	AC016747.2
ENSG00000215835	18.16638696	26.00950937	-0.522772935	0.383078324	0.509561639	AL596087.1
ENSG00000239948	1.789675198	4.330374705	-1.267091527	0.383107846	0.50957482	RN7SL368P
ENSG00000229598	3.882691704	7.225136749	-0.888215656	0.383164074	0.509623518	PRDX3P1
ENSG00000175874	2.125753916	4.250098321	-1.006788868	0.383235427	0.509692328	CREG2
ENSG00000278383	8.857970386	4.097846288	1.09519997	0.383281277	0.509720281	AL031673.1
ENSG00000100811	2280.65438	2035.647086	0.16407121	0.383306702	0.509720281	YY1
ENSG00000232719	0.89358395	2.567748738	-1.506103027	0.383315301	0.509720281	AC007272.1
ENSG00000090905	1329.959779	1529.065134	-0.201192697	0.383381238	0.509780874	TNRC6A
ENSG00000258920	54.08277702	39.7385325	0.43988864	0.383400111	0.509780874	FOXN3-AS1
ENSG00000267811	6.959982497	3.995060664	0.808054082	0.383430433	0.509795102	AP001160.2
ENSG00000136327	2.149715785	0	3.437034176	0.383520162	1	NKX2-8
ENSG00000229611	2.149715785	0	3.437034176	0.383520162	1	AL354707.2
ENSG00000286628	2.149715785	0	3.437034176	0.383520162	1	AL096828.4
ENSG00000283204	2.149715785	0	3.437034176	0.383520162	1	MIR4434
ENSG00000273361	2.149715785	0	3.437034176	0.383520162	1	AC021016.3
ENSG00000266602	2.149715785	0	3.437034176	0.383520162	1	AP005262.2
ENSG00000172548	6.068905845	3.32212083	0.884731582	0.383649797	0.510054583	NIPAL4
ENSG00000244515	1.83125942	4.444928691	-1.294246772	0.38366486	0.510054583	KRT18P34
ENSG00000174446	344.8871585	284.1050823	0.278638974	0.383810431	0.510222002	SNAPC5
novel.1044	18.06058157	10.94624653	0.728025398	0.383836184	0.51023013	-
ENSG00000078900	4.850597286	1.782695063	1.440324917	0.383948904	0.510353857	TP73
ENSG00000203722	10.6753684	5.860343861	0.871267971	0.384039739	0.510448481	RAET1G
ENSG00000166266	654.5200982	728.0353783	-0.15367505	0.38420629	0.510643729	CUL5
ENSG00000253676	0.909952655	2.496928964	-1.459614206	0.384228366	0.510646948	TAGLN2P1
ENSG00000198759	1.230916317	4.228744955	-1.776611967	0.384336908	0.510744201	EGFL6
ENSG00000204104	482.9838797	414.832397	0.219557813	0.384343684	0.510744201	TRAF3IP1
ENSG00000236498	7.457027504	3.515795324	1.079419943	0.384360518	0.510744201	AC107081.2
ENSG00000125485	267.0946116	323.7764722	-0.278597071	0.38454601	0.510964552	DDX31
ENSG00000116350	1900.019564	2170.943191	-0.192175826	0.384568589	0.510968421	SRSF4
ENSG00000234694	5.459787201	10.46826546	-0.944687362	0.384915699	0.511392104	AL139289.2
ENSG00000088367	677.7132188	569.4063822	0.251140648	0.384926831	0.511392104	EPB41L1
ENSG00000230530	15.29067776	9.66070255	0.659053894	0.384954922	0.511393383	LIMD1-AS1
ENSG00000177738	120.6317804	140.0734275	-0.215668513	0.38496716	0.511393383	AC025171.1
ENSG00000120215	15.35719277	9.822411193	0.650762064	0.385065305	0.511497607	MLANA
ENSG00000127954	347.7793612	132.6173974	1.390574127	0.385138143	0.511568205	STEAP4
ENSG00000285672	6.305447414	3.32212083	0.941062073	0.385188263	0.511608622	AL160396.2
ENSG00000176410	176.2081997	208.6620602	-0.2451356	0.385280629	0.511705144	DNAJC30
ENSG00000174792	1.493927053	4.493239223	-1.577575997	0.385494744	0.511963346	ODAPH
ENSG00000224324	0.586481695	2.10970837	-1.833647536	0.385565115	0.512028524	THAP5P1
ENSG00000121053	5.348895942	2.120320855	1.332250861	0.385604815	0.512028524	EPX

ENSG00000168385	10396.40974	11679.18195	-0.167850648	0.385611923	0.512028524	SEPT2
ENSG00000111913	479.4707103	248.0625471	0.950407756	0.385622652	0.512028524	RIPOR2
ENSG00000267610	0.600343103	2.261960402	-1.897540777	0.385827801	0.51223971	AC007787.1
ENSG00000226742	326.4261783	269.8961862	0.275257548	0.385836976	0.51223971	HSBP1L1
ENSG00000256538	3.62978143	1.130980201	1.704115424	0.385858698	0.51223971	AC046130.2
ENSG00000113658	1449.981581	1262.970639	0.199251832	0.385860566	0.51223971	SMAD5
ENSG00000196531	5774.054489	6635.883238	-0.20070675	0.386139495	0.512575415	NACA
ENSG00000272645	168.3989108	134.6794719	0.322366785	0.386152904	0.512575415	GTF2IP20
ENSG00000235034	21.93087898	33.54488238	-0.612084413	0.386304923	0.512751006	C19orf81
ENSG00000143387	1192.345688	778.3836968	0.615348208	0.386356159	0.512791128	CTSK
ENSG00000241458	5.128723077	2.506385574	1.032107084	0.386374625	0.512791128	RPL7P19
ENSG00000042286	488.3912535	409.5084565	0.253578109	0.386416634	0.512820687	AIFM2
ENSG00000260022	1.215801261	2.873537073	-1.243325668	0.386544128	0.512939397	AL031716.1
ENSG00000240731	46.0791002	58.50471541	-0.341639911	0.386556558	0.512939397	AL139287.1
novel.394	10.68045426	6.339737595	0.742549888	0.386565313	0.512939397	-
novel.129	1.21705491	0	2.616235889	0.386632843	1	-
novel.308	1.21705491	0	2.616235889	0.386632843	1	-
ENSG00000260918	1.21705491	0	2.616235889	0.386632843	1	AC107398.3
ENSG00000272922	1.21705491	0	2.616235889	0.386632843	1	AC107294.1
novel.696	1.21705491	0	2.616235889	0.386632843	1	-
ENSG00000287645	1.21705491	0	2.616235889	0.386632843	1	AC092939.1
ENSG00000250415	1.21705491	0	2.616235889	0.386632843	1	AC022113.1
ENSG00000249203	1.21705491	0	2.616235889	0.386632843	1	LINC02224
ENSG00000179460	1.21705491	0	2.616235889	0.386632843	1	EEF1A1P27
ENSG00000255418	1.21705491	0	2.616235889	0.386632843	1	AC090092.1
ENSG00000271623	1.21705491	0	2.616235889	0.386632843	1	AC138894.3
ENSG00000235029	1.21705491	0	2.616235889	0.386632843	1	MNX1-AS2
ENSG00000225725	1.21705491	0	2.616235889	0.386632843	1	FAM66E
ENSG00000220694	1.21705491	0	2.616235889	0.386632843	1	AL096711.1
ENSG00000280234	1.21705491	0	2.616235889	0.386632843	1	AC124303.1
ENSG00000287470	1.21705491	0	2.616235889	0.386632843	1	AP001037.1
ENSG00000227850	1.21705491	0	2.616235889	0.386632843	1	SEPT2P1
ENSG00000240710	1.21705491	0	2.616235889	0.386632843	1	AL512306.3
ENSG00000221445	1.21705491	0	2.616235889	0.386632843	1	MIR1301
ENSG00000230649	1.21705491	0	2.616235889	0.386632843	1	AC024084.1
ENSG00000228024	1.21705491	0	2.616235889	0.386632843	1	CYP1D1P
ENSG00000225899	1.21705491	0	2.616235889	0.386632843	1	FRG2B
ENSG00000180389	1.21705491	0	2.616235889	0.386632843	1	ATP5F1EP2
ENSG00000231249	4.850597286	8.508368983	-0.801857673	0.386830085	0.513264513	ITPR1-DT
ENSG00000137449	823.4992499	661.8885352	0.314721921	0.386892324	0.51332088	CPEB2
ENSG00000242142	1.215801261	0	2.614939712	0.386953316	1	SERBP1P3
novel.258	1.215801261	0	2.614939712	0.386953316	1	-
ENSG00000287709	1.215801261	0	2.614939712	0.386953316	1	AC008780.2
ENSG00000236674	1.215801261	0	2.614939712	0.386953316	1	AL049636.1
ENSG00000276406	1.215801261	0	2.614939712	0.386953316	1	AC015726.2
ENSG00000266928	1.215801261	0	2.614939712	0.386953316	1	AC020905.1
ENSG00000263787	1.215801261	0	2.614939712	0.386953316	1	SKAP1-AS1
ENSG00000204872	1.215801261	0	2.614939712	0.386953316	1	NAT8B
ENSG00000287899	1.215801261	0	2.614939712	0.386953316	1	AC055714.1
ENSG00000118733	1.215801261	0	2.614939712	0.386953316	1	OLFM3
ENSG00000254213	1.215801261	0	2.614939712	0.386953316	1	PRXL2AP2

ENSG00000281406	1.215801261	0	2.614939712	0.386953316	1	BLACAT1
ENSG00000235371	1.215801261	0	2.614939712	0.386953316	1	AL122018.1
ENSG00000287389	1.215801261	0	2.614939712	0.386953316	1	AC007106.2
ENSG00000287079	1.215801261	0	2.614939712	0.386953316	1	AL109806.2
ENSG00000171603	5163.364522	4414.553687	0.226091811	0.386978154	0.513408539	CLSTN1
ENSG00000177335	9.117220175	14.27564062	-0.649999127	0.387149893	0.51361016	C8orf31
ENSG00000154710	146.3834237	124.8725063	0.228026878	0.387207697	0.513644153	RABGEF1
ENSG00000275160	8.91975553	17.36394876	-0.962731501	0.387219624	0.513644153	AL354718.1
ENSG00000214432	4.839243176	2.038888596	1.221271224	0.387234826	0.513644153	VPS33B-DT
ENSG00000272662	0.293240848	1.733100261	-2.418899301	0.387345758	1	AC073352.1
ENSG00000225498	0.293240848	1.733100261	-2.418899301	0.387345758	1	AC002064.1
ENSG00000284651	0.293240848	1.733100261	-2.418899301	0.387345758	1	AP000553.6
ENSG00000260123	3.680141193	1.365948763	1.400854367	0.387387737	0.513820747	AC013565.1
ENSG00000251595	41.74693122	30.59594921	0.447493584	0.387566726	0.514024974	ABCA11P
ENSG00000107518	7.696361105	2.772035718	1.46249261	0.387597451	0.514024974	ATRNL1
ENSG00000260361	3.688988006	1.130980201	1.724589113	0.387601818	0.514024974	AC106028.3
ENSG00000218418	47.9521573	38.01232031	0.33418942	0.387626894	0.514024974	AL591135.1
ENSG00000169862	14.28125923	8.130604999	0.820205241	0.387652651	0.514024974	CTNND2
ENSG00000049618	1151.104747	1003.345833	0.198419977	0.387660419	0.514024974	ARID1B
ENSG00000237686	9.509713437	22.94546146	-1.267060613	0.38770469	0.514057441	AL109615.3
ENSG00000269904	1.524157165	0.387220594	1.978072759	0.387771098	1	MAP2K4P1
ENSG00000261687	1.524157165	0.387220594	1.978072759	0.387771098	1	AC068724.1
ENSG00000224459	1.524157165	0.387220594	1.978072759	0.387771098	1	SLC25A34-AS1
ENSG00000054611	582.0988263	688.7325056	-0.242560842	0.387836716	0.514206252	TBC1D22A
ENSG00000287091	14.06108637	22.52208387	-0.68472205	0.387916406	0.514285664	AC009961.4
ENSG00000121067	926.7603989	811.8794074	0.190676253	0.388070384	0.51446355	SPOP
ENSG00000120071	34.53153115	22.0897164	0.649501157	0.388192912	0.514599727	KANSL1
ENSG00000278979	2.124500267	0.723690511	1.559577087	0.388333462	0.514759779	AC007598.3
ENSG00000279971	0	1.782695063	-3.415972101	0.38836564	1	AC020910.6
ENSG00000278259	0	1.782695063	-3.415972101	0.38836564	1	MYO19
ENSG00000096395	0	1.782695063	-3.415972101	0.38836564	1	MLN
ENSG00000270159	0	1.782695063	-3.415972101	0.38836564	1	AC018695.3
ENSG00000234247	0	1.782695063	-3.415972101	0.38836564	1	AC096537.2
ENSG00000251273	0	1.782695063	-3.415972101	0.38836564	1	LINC02228
ENSG00000285603	0	1.782695063	-3.415972101	0.38836564	1	AL035693.1
ENSG00000214344	0	1.782695063	-3.415972101	0.38836564	1	OR4F13P
ENSG00000104827	0	1.782695063	-3.415972101	0.38836564	1	CGB3
ENSG00000235347	0	1.782695063	-3.415972101	0.38836564	1	Z84478.1
ENSG00000279355	12.50634308	7.25581833	0.792126708	0.3885511	0.515021995	AGPAT4-IT1
ENSG00000211673	2.775203133	0.336469917	2.843304313	0.38861524	0.515080734	IGLV3-1
ENSG00000237280	1.201939854	3.281982638	-1.441002039	0.388730465	0.515207173	AL136982.3
ENSG00000170469	27.5255193	39.15506887	-0.510105964	0.388773493	0.515235554	SPATA24
ENSG00000178896	279.1529723	348.3116909	-0.319952339	0.388791541	0.515235554	EXOSC4
ENSG00000227256	5.448433091	2.833398881	0.935885001	0.38884287	0.515277293	MIS18A-AS1
ENSG00000223949	0	1.487519214	-3.150001507	0.388970208	1	ROR1-AS1
ENSG00000144331	0	1.487519214	-3.150001507	0.388970208	1	ZNF385B
[truncated: 1,227,846 more chars]
