# Supplementary material for: Synergistic Effect of Erastin Combined with Nutlin-3 on Vestibular Schwannoma Cells as p53 Modulates Erastin-Induced Ferroptosis Response
Source: J Oncol. 2022 Mar 21;2022:7507857. doi: 10.1155/2022/7507857 (PMC8961447; doi:10.1155/2022/7507857)
Supplement: Supplementary 3 — Supplementary Table 2: top deregulated gene sets from GSEA analysis. [file 7507857.f3.pdf]

Supplementary Table 2. Top deregulated gene sets from GSEA analysis

	GSEA Set Name	Number of Genes in Set	Gene Count	ES (VS vs. Ctrl)	NES (VS vs. Ctrl)	Nominal P Value	FDR Q Value
No.1	BIOSYNTHESIS_OF_AMINO_ACIDS(HSA01230)	258	49	-0.5846092	-1.5621203	0	0.050999973
No.2	THIAMINE_METABOLISM(HSA00730)	28	9	-0.72221303	-1.5126305	0	0.0697793
No.3	CYSTEINE_AND_METHIONINE_METABOLISM(HSA00270)	117	26	-0.5321748	-1.491069	0	0.063519545
No.4	GLYCOLYSIS_/_GLUCONEOGENESIS(HSA00010)	215	68	-0.5107251	-1.4887606	0	0.060389634
No.5	BASE_EXCISION_REPAIR(HSA03410)	99	22	-0.6410489	-1.4730167	0	0.067292206
No.6	OOCYTE_MEIOSIS(HSA04114)	266	75	-0.62000644	-1.4513403	0	0.08394495
No.7	CENTRAL_CARBON_METABOLISM_IN_CANCER(HSA05230)	144	36	-0.52707756	-1.4492519	0	0.076623864
No.8	2-OXOCARBOXYLIC_ACID_METABOLISM(HSA01210)	37	14	-0.63670623	-1.4374844	0	0.10112876
No.9	GALACTOSE_METABOLISM(HSA00052)	50	16	-0.65431637	-1.4310215	0	0.0999859
No.10	CELL_CYCLE(HSA04110)	275	91	-0.6482852	-1.4166309	0	0.11969377
No.11	GLUTATHIONE_METABOLISM(HSA00480)	118	36	-0.61331385	-1.4088451	0	0.117778234
No.12	GLUCAGON_SIGNALING_PATHWAY(HSA04922)	187	38	-0.4297487	-1.4000245	0	0.12035187
No.13	ONE_CARBON_POOL_BY_FOLATE(HSA00670)	49	14	-0.6032422	-1.3995095	0	0.11572843
No.14	P53_SIGNALING_PATHWAY(HSA04115)	163	36	-0.5109601	-1.385536	0	0.12957214
No.15	UBIQUINONE_AND_OTHER_TERPENOID-QUINONE_BIOSYNTHESIS(HSA00130)	20	8	-0.67280245	-1.3805438	0	0.13386089
No.16	CELLULAR_SENESCENCE(HSA04218)	316	85	-0.47804257	-1.3728352	0	0.13377704
No.17	NON-HOMOLOGOUS_END-JOINING(HSA03450)	25	9	-0.75347996	-1.3700392	0	0.13370176

No.1 8	AMINO_SUGAR_AND_NUC LEOTIDE_SUGAR_METAB OLISM(HSA00520)	70	32	-0.59526 32	-1.3674 239	0	0.1337 2059
No.1 9	ARGININE_AND_PROLINE _METABOLISM(HSA00330)	114		-0.39263 725	-1.2872 913	0	0.1745 1757
No.2 0	NUCLEOTIDE_EXCISION_ REPAIR(HSA03420)	86		-0.66549 66	-1.2869 13	0	0.1718 3232
